# Negative σ-Holes on Fluorine in Molecules Revisited: Halogen Bonding or Counterintuitive, Anti-Electrostatic Interactions?

**DOI:** 10.3390/ijms27146519

**Published:** 2026-07-22

**Authors:** Pradeep R. Varadwaj, Helder M. Marques, Ireneusz Grabowski, Mohd. Mudassir Husain

**Affiliations:** 1 Institute of Physics, Faculty of Physics, Astronomy, and Informatics, Nicolaus Copernicus University in Toruń, 87-100 Toruń, Poland; 2 Molecular Sciences Institute, School of Chemistry, University of the Witwatersrand, Johannesburg 2050, South Africa; 3 Institute of Advanced Studies, Nicolaus Copernicus University in Toruń, ul. Wileńska 4, 87-100 Toruń, Poland; 4 Department of Applied Sciences and Humanities, Faculty of Engineering and Technology, Jamia Millia Islamia, New Delhi 110025, India

**Keywords:** negative–negative interactions, intermolecular interactions, environmental effects, anti-electrostatic interactions, halogen-centric interactions, chemical physics and physical chemistry, computational chemistry

## Abstract

Halogen bonding develops when an electrophilic region associated with a covalently bonded halogen interacts attractively with a nucleophilic site on another same or different molecular entity. Here, we show that in many crystals in the Cambridge Structural Database, the intermolecular distances and directional features of F···F close contacts involve σ-hole-like regions on covalently bound fluorine. However, such interactions cannot readily be classified as classical halogen bonds, as the interacting fluorine atoms may lack a positive site (e.g., a positive σ-hole). In this context, we investigated directional F···F interactions involving HF, CH_4−*n*_F*_n_* (*n* = 1–4), and C_6_H_6−*n*_F*_n_* (*n* = 1–6) interacting with negative sites in the same or different partner interacting species using computational methods, which exhibit complex geometries reminiscent of σ-hole interactions. However, such interactions between sites of like polarity are more appropriately described as σ-hole-centered anti-electrostatic interaction motifs, driven in part by dispersion, rather than being recognized as conventional σ-hole-centered halogen bonds. This interpretation is supported by molecular electrostatic surface potential and symmetry-adapted perturbation theory analyses.

## 1. Introduction

Noncovalent interactions play a central role in determining the structure, stability, and function of molecular assemblies in chemistry [1,2,3], biology [4,5,6], medicinal [7,8] and materials science [9,10,11,12]. Among these, halogen bonding has emerged as a powerful and highly directional interaction [2,9,13], widely exploited in crystal engineering [3,14,15,16], supramolecular chemistry [17,18], catalysis [13,19] and molecular recognition [20,21]. It is commonly described as an attractive interaction between an electrophilic region associated with a covalently bonded halogen and a nucleophilic site such as a lone pair, π-system, or anionic center [2,22], analogous to the definitions proposed for chalcogen bond [23], tetrel bond [24], and pnictogen bond [25,26].

The concept of the σ-hole has provided a unifying electrostatic framework for understanding halogen bonding and related directional interactions [27]. This description has been successfully extended to a wide range of directional noncovalent interactions [28], involving aerogens [29,30,31], chalcogens [32,33,34,35,36,37], and pnictogens [38,39,40,41]. However, what happens when the terminal region on atom X (X = F, Cl, Br, I) in R–X is insufficiently polarized by the remainder of the substituent R? In such cases, does this region develop a positive electrostatic potential, or does it instead exhibit reduced electron depletion with negative electrostatic character, and can it still engage attractively with a nearby Lewis base?

Fluorine, however, has traditionally been regarded as an exception within the halogen bonding paradigm [42,43]. Due to its high electronegativity and low polarizability, covalently bonded fluorine is generally considered incapable of sustaining a significant positive σ-hole and therefore has often been assumed to be a poor participant in conventional halogen bonding interactions [27,44,45]. As a result, close F···F contacts [46,47] observed in crystal structures have often been interpreted as packing effects, or forced consequence of primary interactions rather than directional, [48] attractive interactions [43,49].

Despite this view, systematic analyses of crystallographic databases have revealed recurring short and directional F···F contacts, suggesting a more nuanced interaction landscape than previously assumed [9,50,51,52,53,54]. In particular, increasing attention has been given to classical and non-counterintuitive interaction regimes [55], including halogen bonding [56,57,58,59,60]. Related theoretical and experimental studies have also highlighted the role of polarizable environments and condensed-phase effects in modulating interaction potentials that differ significantly from gas-phase electrostatic expectations [2,42,54].

In this context, the present work revisits the nature of directional F···F interactions formed by a range of fluorinated molecular systems, including HF, CH_4−*n*_F*_n_*, and C_6_H_6−*n*_F*_n_* derivatives. By combining structural data from the Cambridge Structural Database (CSD) [16] with electronic structure calculations predominantly in the solution phase, we examine whether these interactions can be reconciled within existing σ-hole-based frameworks or whether they represent a distinct class of noncovalent attraction. This question is motivated by increasing interest in non-classical and counter-intuitive interaction regimes, including so-called anti-electrostatic interactions, in which attractive forces may arise between like-charged or nominally repulsive sites under specific structural or environmental constraints. Our results suggest that many of these F···F interactions are better interpreted in terms of negative σ-hole centered anti-electrostatic stabilization rather than classical halogen bonding, prompting a reassessment of how directional interactions between like-polarity sites are classified. Additionally, we examine how solvation influences close-contact interaction motifs between localized negative sites in interacting neutral molecular systems, and whether solvent screening can produce patterns analogous to those observed in anion–anion assemblies reported elsewhere [30,61,62,63,64].

## 2. Materials and Methods

The CSD [16] was used to analyze fluorine-containing crystal structures, with additional case-by-case inspection performed using Mercury [65]. Selected binary complexes (e.g., HF and C_6_F_6_) were extracted from crystals and subjected to energy minimization to evaluate their intrinsic interaction preferences. However, it was observed that analogous supramolecular arrangements are not always preserved across different crystals. In most cases, crystal packing is strongly influenced by additional functional groups, which compete with or modulate F···F interaction motifs. For example, the C_6_H_3_F_3_ dimer examined in this study exhibits F···F contacts similar to those identified in several crystal structures; however, in the latter, the remaining hydrogen atoms are often partially or fully replaced by other substituents, leading to altered packing arrangements while still retaining related local F···F interaction motifs (see below).

All quantum chemical calculations were performed using Gaussian 16 [66], including geometry optimizations, frequency calculations, and wavefunction generation for post-processing in AIMAll [67] and Multiwfn [68]. The majority of the reported binary complexes exist as high-energy conformations, i.e., saddle points on the potential energy surface.

The self-consistent reaction field (SCRF) model implemented in Gaussian 16 was employed for all cases using the solvation model based on solute electron density (SMD) [69] with water as the solvent. Gas-phase calculations were performed only for selected cases where intuitive expectations did not match the results obtained. The M06-2X functional was used [70,71]. The def2-TZVPPD basis set was used, obtained from the EMSL Basis Set Library [72].

The molecular electrostatic surface potential (MESP) [13,73,74,75], the quantum theory of atoms in molecules (QTAIM) [76], the independent gradient model based on Hirshfeld partition (IGMH) [77], and the interaction region indicator (IRI) [78] were applied. While MESP was useful for understanding the chemical reactivity of the monomers and visually confirming dimer formation, QTAIM, IRI, and IGMH were used to examine the topological nature of bonding interactions between interacting molecules.

Symmetry-adapted perturbation theory (SAPT) analysis [79,80], including dispersion corrections evaluated at the coupled-cluster doubles (CCD) level, was performed using the same basis set described above to obtain a physically meaningful decomposition of the intermolecular interaction energies governing the observed F···F and related contacts. Unlike total interaction energy approaches, SAPT partitions the interaction energy *E*_SAPT_ (*E_SAPT_* = *E_elst_* + *E_exch_* + *E_ind_* + *E_disp_*) into well-defined components—electrostatics, exchange repulsion, induction (polarization), and dispersion—allowing direct assessment of the reasonable origins of stabilization or destabilization between molecular fragments. This decomposition is particularly important in the present work, where conventional electrostatic intuition (e.g., σ-hole-based attraction) may not fully capture the nature of interactions between fluorinated systems. SAPT therefore provides a somewhat rigorous framework to distinguish whether the observed contacts are dominated by electrostatic attraction, dispersion-driven stabilization, or a balance of competing repulsive and attractive contributions, thereby clarifying whether these interactions can be classified within classical halogen bonding or alternative regimes such as anti-electrostatic interactions. Unless otherwise stated, and due to computational memory limitations, high-level SAPT calculations at the SAPT2+(3) level were performed only for the dimers of C_6_H_6−*n*_F*_n_* using the aug-cc-pVDZ basis set. The PSI4 package was used [81].

The binding energy, $E_{b}$, for each dimer in solution was calculated using the supermolecular procedure defined by Equation (1), where

(1) $$E_{b}=E_{\mathrm{dimer}}-\left(E_{\mathrm{monomer}\;A}+E_{\mathrm{monomer}\;B}\right),$$

with $E_{\mathrm{dimer}}$ representing the total electronic energy of the optimized dimer and $E_{\mathrm{monomer}\;A}$ and $E_{\mathrm{monomer}\;B}$ corresponding to total electronic energies of the individual monomers computed at the same level of theory.

### The Concept of σ-Hole and Its Positive and Negative Character

The σ-hole is a surface phenomenon defined at the outer molecular boundary and is typically evaluated on the 0.001 a.u. electron density isosurface [28]. The absence of a visible σ-hole on a covalently bonded atom X is not solely an artifact of the chosen isodensity value but primarily reflects the intrinsic dominance of negative electrostatic character of around the atom in the system, which is governed by the underlying electron density distribution. The magnitude and sign of the maximum and minimum of the electrostatic potential are, however, dependent on the chosen isodensity envelope [57,82]. For example, in CF_4_, the fluorine atom exhibits a near-neutral region on the conventional 0.001 a.u. isodensity envelope, along the C–F bond extension. Upon contraction of the isodensity envelope (e.g., to approximately 0.0014 a.u.), the maximum of the electrostatic potential becomes positive owing to the increasing influence of the nuclear contribution at shorter distances [57]. Thus, in some fluorinated systems, a positive maximum of the electrostatic potential along the R–F bond extension may be absent on the 0.001 a.u. envelope yet appear on a slightly more contracted envelope, reflecting the distance dependence of the electrostatic potential rather than the emergence of a fundamentally new electrostatic feature, where R denotes the remainder of the molecular entity.

Following the systematic electrostatic interpretation of molecular surfaces by Politzer and co-workers [28,45], the σ-hole was recognized as an anisotropy-based feature arises from the non-uniform distribution of electron density around a bonded atom, leading to directionally dependent electrostatic interactions that can drive phenomena such as halogen bonding [22,83]. In classical σ-hole systems [27], this feature is typically associated with a local most maximum of positive potential, $V_{S,max}$, that contrasts with surrounding regions of negative potential, providing a directional site for molecular recognition and assembly.

**Scheme 1:**
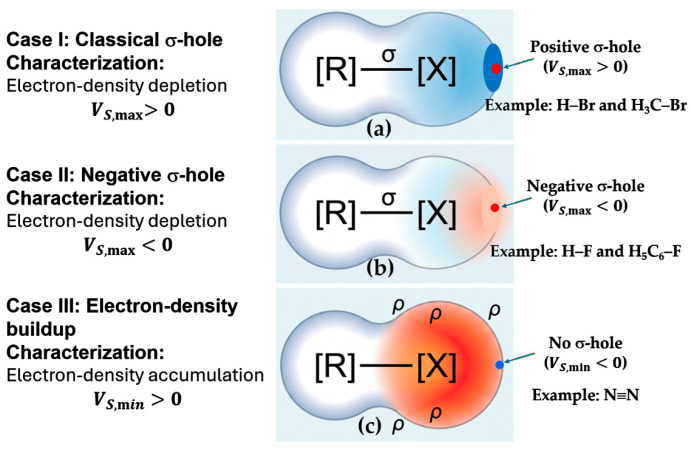
Visual representation of the three possible chemical instances along the extension of an R–X bond: (**a**) a classical σ-hole characterized by electron-density depletion and $V_{S,max}$ > 0; (**b**) a negative σ-hole characterized by electron-density depletion and $V_{S,max}$ < 0; and (**c**) an electron-density buildup characterized by electron accumulation and $V_{S,min}$ < 0.

Clearly, the defining characteristic of a σ-hole on atom X in R–X is a depletion of electron density along the extension of a covalent bond [44,84,85,86,87,88]. The magnitude of $V_{S,max}$ provides a measure of its electrostatic strength, and its sign distinguishes between positive and negative manifestations of the same underlying electron-density anisotropy. This is concordant with Politzer and co-workers [28,89], and others [2,44,56], who have emphasized that the electrostatic potential along a bond extension may exhibit three distinct situations, schematically illustrated in Scheme 1. The first corresponds to a classical σ-hole, characterized by a depletion of electron density characterized by $V_{S,max}>0$ (Scheme 1a). The second corresponds to a negative σ-hole, in which the electron-density depletion remains present but $V_{S,max}<0$ (Scheme 1b). The third situation involves a buildup of electron density along the bond extension rather than a depletion and is associated with a negative $V_{S,min}$($V_{S,min}$ < 0) (Scheme 1c). In this latter case, the defining feature of a σ-hole is absent despite the negative potential. These distinctions demonstrate that the sign of $V_{S,max}$ alone is sufficient to distinguish positive and negative σ-holes, whereas the underlying electron-density distribution must be considered to determine whether the bond extension is characterized by electron depletion or electron accumulation.

The σ-hole framework extended to include the concept of its negative character was an attempt to describe chemical instances in which the expected positive potential along a bond extension is absent and is instead replaced by a locally enhanced negative surface electrostatic potential. In this case, the relevant descriptor, $V_{S,max}<0$, indicates that even the local most positive point on the surface of atom X along the extension remains negative. This behavior can be illustrated in diatomic hydrogen fluoride (HF), where the maximum of potential on fluorine along the H–F bond extension is negative and surrounded by an even more negative potential minimum, $V_{S,min}$ < 0 (see discussion below). This effect is particularly relevant for highly electronegative substituents, where strong electron withdrawal suppresses the formation of a positive electrostatic cap. In such cases, the anisotropy of the electrostatic potential does not generate a bonding-favorable positive site but instead produces a directionally structured negative region, reflecting a fundamentally different interaction landscape. The recognition of negative σ-holes thus broadens the original σ-hole concept and emphasizes that electrostatic anisotropy can manifest not only as attraction-driving positive regions but also as organized negative potentials that influence molecular interaction patterns.

## 3. Results

### 3.1. Statistical Analysis of Y–F···X Contacts in the CSD

We performed a survey of the CSD to analyze the distribution of C–F···X contacts with C–F···X angles constrained between 160° and 180°, where X represents F, Cl, Br, I, O, Se, Te, Ar, Kr, Xe, and elements of groups 3A–5A. Only structures with an R-factor < 5%, no reported disorder or errors, and determined from single-crystal data with available three-dimensional (3D) coordinates were considered. This search yielded 20,901 hits, from which 56,726 close contacts were extracted. When the angular range was further restricted to 175–180°, the number of hits decreased to 1761, comprising 1940 contacts.

When the carbon atom in C–F···X was replaced by a generic atom Y (Y–F···X contacts), and the F···X distance range was constrained between 1.7 and 3.5 Å, the number of hits increased to 27,179, from which a total of 69,468 contacts were extracted. This search did not include close contacts between aromatic ring centroids and X–F interactions.

The distributions of *r*(F···X) distances and ∠C–F···X angles obtained from the first search criterion were analyzed using histograms (see Table S1 for raw data). Gaussian functions constructed from the corresponding sample means and standard deviations are included as visual guides for comparison. The *x*-axis denotes either *r*(F···X) (Å) or ∠C–F···X (°), with frequency on the left *y*-axis and normalized probability density on the right *y*-axis.

**Figure 1:**
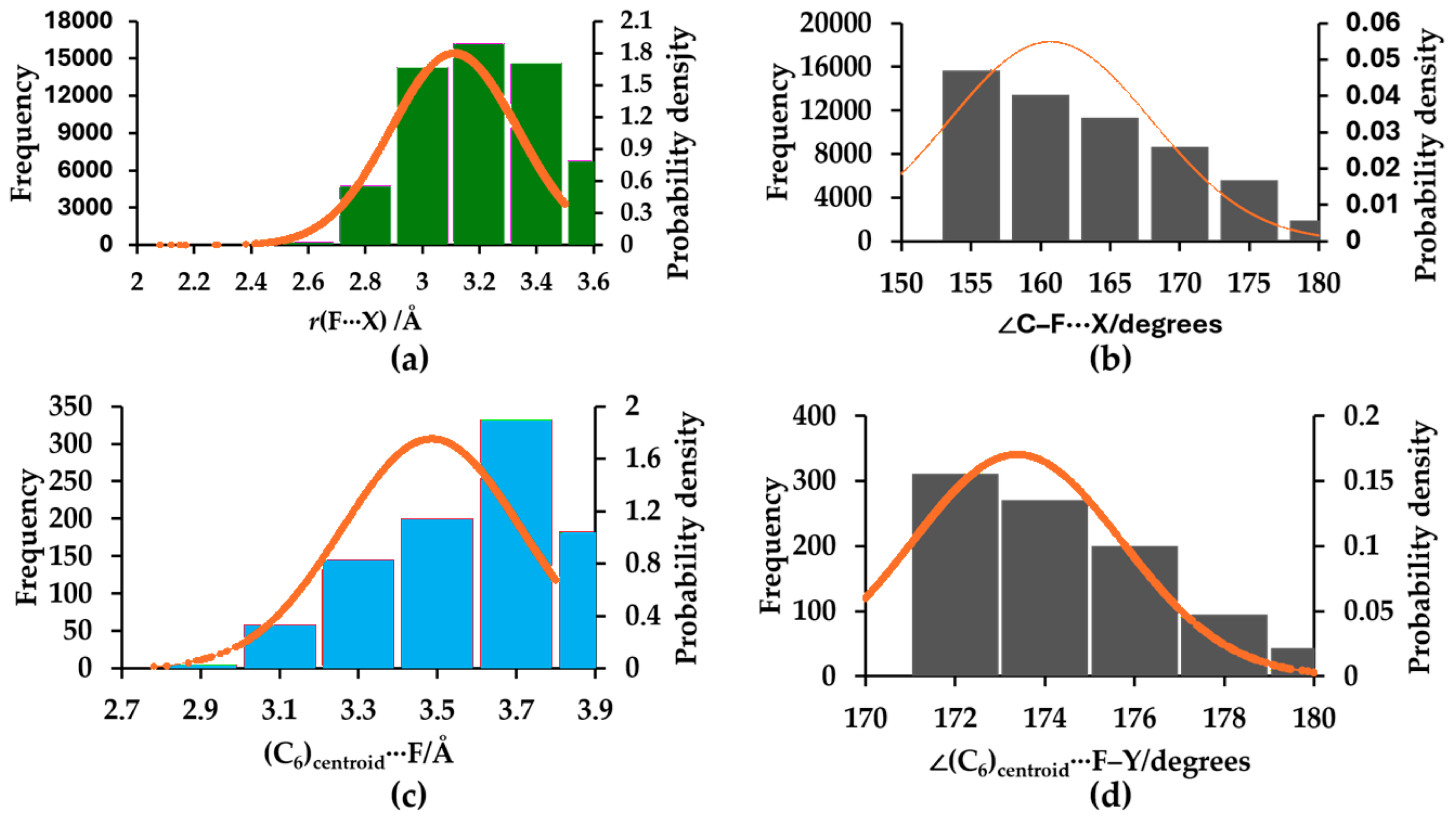
Histograms of (**a**) *r*(F···X) distances and (**b**) ∠C–F···X angles for C–F···X contacts in crystals identified from the Cambridge Structural Database (CSD). Corresponding plots are also shown for (**c**) C_6_(centroid)···F distances and (**d**) ∠C_6_(centroid)···F–Y angles, describing interactions between the centroid of aromatic C_6_ rings and Y–F fragments. The distributions are shown with overlaid Gaussian curves constructed from the corresponding sample means and standard deviations. The left *y*-axis represents frequency, while the right *y*-axis shows the normalized probability density. The angular distributions indicate a preference for near-linear geometries with a gradual decrease toward 180°.

Figure 1a shows that the F···X distance distribution has a maximum around 3.1 Å. For the broader search (Y–F···X contacts), the corresponding distribution shows a maximum around 3.1–3.2 Å. Similarly, the angular distribution in Figure 1b exhibits a maximum at ~160–163° for both cases, showing an enrichment in the ~160–163° range, with a gradual decline toward 180° and relatively few perfectly linear arrangements.

A similar search was performed in which both the centroid of an aromatic C_6_ ring and the fluorine atom of a Y–F fragment were considered as the interacting fragments. When the angle between them was constrained to 175–180° and the separation distance was set between 2.5 and 3.8 Å, 430 hits were identified, corresponding to 472 contacts. When the angular range was expanded to 171–180°, the number of hits increased to 880, yielding 923 close contacts. The analysis was limited to benzene-type (C_6_) aromatic rings; systems containing heteroatoms within the ring (e.g., N) or non-six-membered and non-aromatic rings were excluded. The histograms indicate a maximum at a centroid···F distance of ~3.4–3.5 Å (Figure 1c) and a C_6_(centroid)···F–Y angle of ~173–174° (Figure 1d) (see Table S2 for raw data). The distribution indicates a preference for quasi-linear geometries, with a gradual decline in frequency toward 180°, where fully linear arrangements are relatively uncommon. While these may represent σ-hole–centered interactions of F along the Y–F bond extension, it is not straightforward to conclude whether each interaction formed via Y–F bond extension can be attributed to halogen bonding, negative σ-hole–centered anti-electrostatic interactions, π-hole interactions, or something similar. A definitive conclusion on whether the interaction is intuitive or counterintuitive can be reached only through case-by-case analysis of each interacting building block responsible for crystal packing, using multilevel computational approaches.

**Figure 2:**
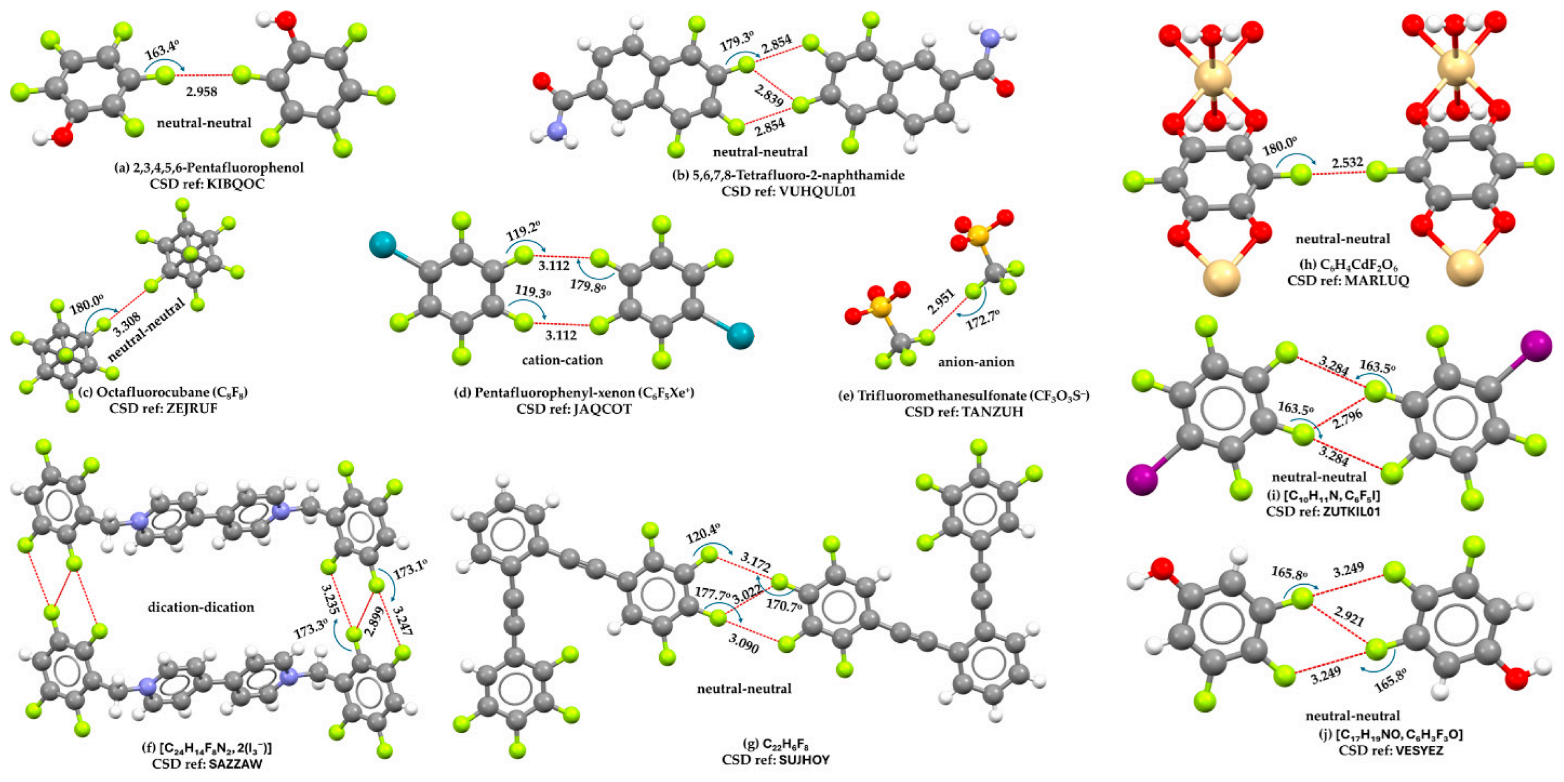
(**a**–**j**) Illustration of selected crystal systems with C–F···F close contacts that are either linear or quasi-linear, occurring between (**a**–**c**,**g**–**j**) neutral or (**d**–**f**) charged building blocks. For clarity, the molecular entities such as AsF_6_^–^ in (**d**), FNH_3_^+^ in (**e**), 2(I_3_^−^) in (**f**), C_10_H_11_N in (**i**), and C_17_H_19_NO in (**j**) were omitted. Selected bond distances and bond angles are given in Å and °, respectively. The CSD reference is shown in uppercase letters for each case. Atoms are shown as balls: O—red; N—blue; C—gray; S—yellow; Xe—cyan; F—green; H—white.

Figure 2a–j shows representative crystal systems featuring C–F···F close contacts, occurring either between neutral molecular building blocks (Figure 2a–c,g–j) or between cations (Figure 2d,f) or anions (Figure 2e). The shortest F···F distance observed in the crystal of C_6_H_4_CdF_2_O_6_ (Figure 2h) and the longest in the crystal of the rare molecule octafluorocubane (C_8_F_8_) [1] (Figure 2c). The interaction is linear (∠C–F···F = 180.0°). The interaction shown in Figure 2h is not only linear but also occurs between two identical fragments; thus, it remains unclear whether the F···F interaction is of lump–hole, lump–lump, or hole–hole type, or whether it arises between sites of identical or dissimilar polarities [60,90,91,92].

Sixteen other crystal structures exhibit relatively short F···F contacts with r(F···F) < 2.5 Å and C–F···F angles ranging between 160° and 180°. For example, the crystals C_27_H_38_BaF_12_O_11_ (CSD ref: HEXYAK) [93] and C_38_H_32_F_6_HgN_2_O_8_P_2_S_2_ (CSD ref: RAQZUG) [94], feature the F···F geometries between interacting –CF_3_ groups are 2.382 Å (∠C–F···F = 160.4°) and 2.283 Å (∠C–F···F = 166.7°), respectively. A short C–F···O close contact was observed in the crystal of C_28_H_30_F_12_N_14_O_12_Rh_2_ (CSD ref: WUNXIM) [95], with an F···O distance of 2.081 Å and a C–F···O angle of 165.1°. Similarly, the shortest F···F close contact was found between PF_6_^−^ anions in the crystal of [C_108_H_108_Au_10_N_4_P_8_S_4_]^2+^·2(PF_6_^−^) (CSD ref: MAYCUK) [96], with an F···F distance of 1.843 Å and a P–F···F angle of 153.0°. Both contacts are significantly shorter than the sum of the van der Waals radii of the respective interacting atoms (*r*_vdW_ (O) = 1.55 Å, *r*_vdW_ (F) = 1.46 Å [97]).

Clearly, geometric parameters derived from CSD analyses do not provide direct information about the electrostatic nature of the interacting sites. In particular, the sign and magnitude of the electrostatic potential at fluorine—whether it behaves as a positive (σ-hole) or negative site—cannot be inferred solely from intermolecular distances and angles. To establish the electrostatic character of fluorine in these systems, complementary electronic structure calculations are required, such as MESP analysis, evaluation of $V_{S,\max}$ along the C–F bond extension, or energy decomposition approaches (e.g., SAPT). Such analyses are essential for correlating observed structural motifs with the underlying physical origin of the interactions, especially in cases where directional contacts arise despite predominantly negative electrostatic character, as observed in fluorinated aromatic systems.

### 3.2. Negative σ-Holes and Their Intermolecular Bonding Capability

#### 3.2.1. The Directional Bonding Propensity of Negative σ-Hole on F in HF

**Figure 3:**
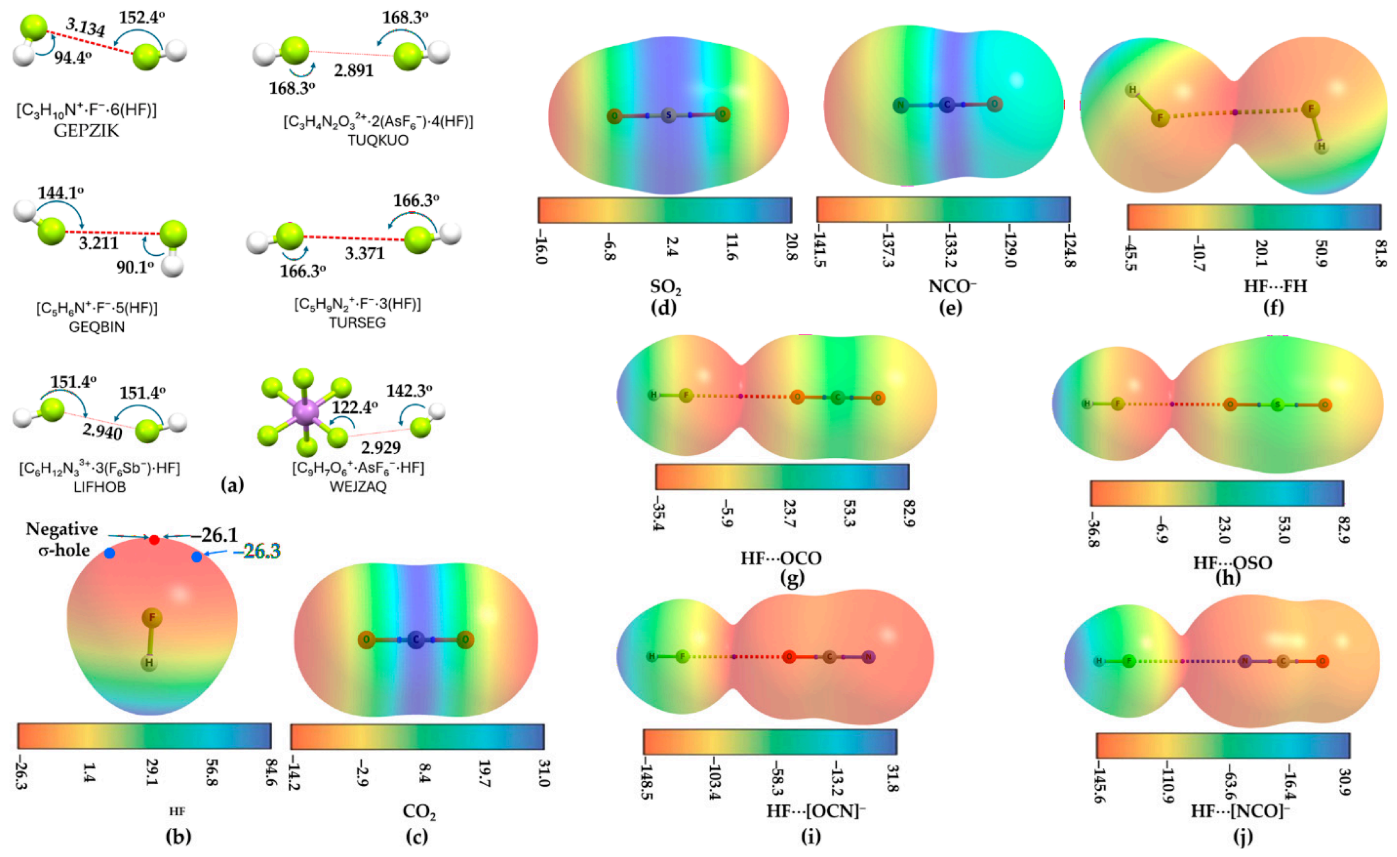
(**a**) Illustration of the spatial arrangement of the HF molecule in selected crystal structures retrieved from the CSD (organic or inorganic moieties omitted for clarity). (**b**–**j**) QTAIM topology superimposed on molecular electrostatic potential maps for (**b**) HF; (**c**) CO_2_; (**d**) SO_2_; (**e**) [NCO]^−^; (**f**) HF···FH; (**g**) HF···CO_2_; (**h**) HF···SO_2_; (**i**) HF···[OCN]^−^; and (**j**) HF···[NCO]^−^, with potential values given in kcal mol^−1^. Bond paths in (**b**–**j**) are represented as solid and dotted lines in atomic colors, and bond critical points are shown as small blue spheres along the bond paths. Selected bond distances and bond angles in (**a**) are reported in Å and degrees (°), respectively. The tiny red and blue circles represent the maximum and minimum of potential (values in kcal mol^−1^), respectively.

Depending on the nature of the building blocks involved, the intermolecular geometry of the HF···FH dimeric arrangement in these crystals varies; in some cases, quasi-linear interactions are observed, while in others, attraction occurs between the lateral sides (Figure 3a). Accordingly, *r*(F···F) values range from 2.6 to 3.4 Å, and the H–F···F angle varies between 140 and 168.3°. This interaction is weak, not a conventional σ-hole-driven halogen bond, and is likely, at least in part, governed by packing forces in the crystalline phase.

Note that the HF molecule is fundamentally known to have a negative F end and a positive H end, and its dimer is non-existent in the gas phase especially when the negative ends are in proximity as a result of coulombic repulsion. This argument is supported by the MESP plot, Figure 3b, showing that the fluorine atom exhibits a negative potential along the extension of the H–F bond, reflecting a negative σ-hole (*V_S,max_* = −26.1 kcal mol^−1^) and the dominance of localized lone-pair electron density around the lateral portions of F (*V_S,min_* = −26.3 kcal mol^−1^). Of course, this feature is in contrast to the heavier hydrogen halides HX (X = Cl, Br, I), [83,98] which each display a positive σ-hole on the halogen atom along the H–X bond extension, analogous to the σ-hole observed in X_2_ diatomic molecules along the outer extension of the X–X bond axis [82,99]. This feature is consistent with their ability to engage in halogen bonding interactions.

The O-ends in CO_2_, SO_2_, and NCO^−^ are characterized by minima in the electrostatic potential, $V_{S,min}$, with values of approximately −14.2, −16.0, and −131.2 kcal mol^−1^, respectively. The N-end of the latter species is increasing more electron-density rich, exhibiting a $V_{S,min}$ value of −141.7 kcal mol^−1^. Figure 3c–e presents the superimposed molecular graphs and MESP maps for these species.

The minimization of the HF···FH geometry (Figure 3f) extracted from the crystal structure [C_3_H_4_N_2_O_3_^2+^·2(AsF_6_^−^)·4(HF)] (CSD ref: TUQKUO) resulted in a configuration analogous to that observed in [C_5_H_6_N^+^·F^−^·5(HF)] (CSD ref: GEQBIN). However, the intermolecular distance is longer, with *r* = 3.417 Å, and the H–F···H angles are inequivalent at 100.9° and 130.0°. Interestingly, this distance is significantly greater than twice the van der Waals radius of fluorine (2 × *r*_vdW_(F) = 2.92 Å; *r*_vdW_(F) = 1.46 Å [97]), indicating a very weak, long-range contact rather than a conventional close-contact interaction. The deviation from the van der Waals contact distance is too large to be attributed to typical crystallographic uncertainties (±0.2 Å), and instead reflects a genuine weak intermolecular association influenced by the crystal environment. The overlap between the MESP surfaces of the monomers in the dimer supports the presence of a measurable intermolecular interaction between the interacting atomic basins.

Three additional σ-hole-centered binary complexes of HF were investigated, in which the negative σ-hole on F in HF engages attractively with the Lewis bases as on the oxygen site in CO_2_, SO_2_, and NCO^−^, as well as with the nitrogen site in NCO^−^ (see Figure 3g–j for molecular graphs and MESP plots). The F···O distances in the first three complexes were 3.186, 3.402, and 3.232 Å, respectively. In the HF···[NCO]^−^ molecule–anion complex, the F···N separation is 3.603 Å, which is appreciably longer than the sum of the van der Waals radii, 3.21 Å, of nitrogen (1.66 Å) and oxygen (1.55 Å). The longer bond distance is consistent with increased repulsion between the N and F atoms and can be attributed to the greater negative character of the N atom relative to the O atom in OCN^−^ (see above).

While the results obtained using the M06-2X functional are consistent with the available crystal geometry of the HF dimer shown in Figure 3a, it is of interest to examine how the results change upon adopting alternative configurations, when the theoretical method and solvent are varied. Such configurations led to hydrogen-bond formation via reorientation of the monomers, as is expected for the FH···FH dimer in the gas phase. We did not extend this analysis to all dimeric systems, but considered only two representative cases, such as HF···FH and HF···OCO. Our calculations for the latter suggest that the intermolecular distance between the monomers increases compared to that obtained in aqueous solution when solvents such as tetrahydrofuran (THF) and dimethyl sulfoxide (DMSO) are used, as well as when the MP2 method is adopted. This indicates that the choice of solvent plays an important role in modulating the intermolecular separation and stabilizing the equilibrium geometry of the dimer.

Nevertheless, and in all four cases above, QTAIM analysis identifies both bond critical points and bond paths. The electron charge densities at the corresponding bond critical points, ρ_b_, are approximately 0.0026, 0.0019, 0.0027, and 0.0018 a.u., respectively, while the corresponding Laplacian values, ∇^2^ρ_b_, are about 0.0152, 0.0090, 0.0145, and 0.0078 a.u., respectively. For comparison, the corresponding values of ρ_b_ and ∇^2^ρ_b_ for the (HF)_2_ dimer are 0.0019 and 0.0084 a.u., respectively. The relatively low values of ρ_b_, together with the positive values of ∇^2^ρ_b_, are characteristic of weak closed-shell interactions.

The SAPT energy decomposition for the HF-containing dimers shown in Table 1 reveals a clear distinction between neutral and anionic interaction regimes, along with a notable divergence between intrinsic gas-phase interactions and apparent solution-phase binding energies (*E_b_*). For the neutral systems (HF···FH, HF···OCO, and HF···OSO), electrostatic contributions are modest (≈ 0.24–0.48 kcal/mol) and are largely offset by exchange repulsion and relatively strong dispersion (up to −0.36 kcal/mol), resulting in small and slightly positive total SAPT2+3(CCD) interaction energies *E_SAPT_* (≈0.14–0.26 kcal/mol). This indicates that the proximity of the monomers in the dimers are repulsive in the gas phase.

The anionic complexes (HF···OCN^−^ and HF···NCO^−^) exhibit a dramatic increase in electrostatic energy (≈5.4 kcal/mol), accompanied by significantly enhanced induction (≈−0.58 kcal/mol), reflecting strong charge–dipole and polarization interactions. Although exchange repulsion also increases, it is comparatively minor relative to the dominant attractive terms, yielding large positive SAPT totals (~4.7–4.8 kcal/mol). However, the corresponding *E_b_* values (−0.27 to −0.46 kcal/mol for neutrals and −0.13 to −0.34 kcal/mol for anions) do not track these SAPT trends and instead suggest non-negligible stabilization across all systems, with even weaker binding for the highly charged complexes as a result of increasing Coulombic repulsion between negative sites of interacting monomers within the charge-assisted complexes, which counteract the localized attractive interactions.

#### 3.2.2. The Directional Bonding Propensity of Negative σ-Hole on F in H_3_CF

**Figure 4:**
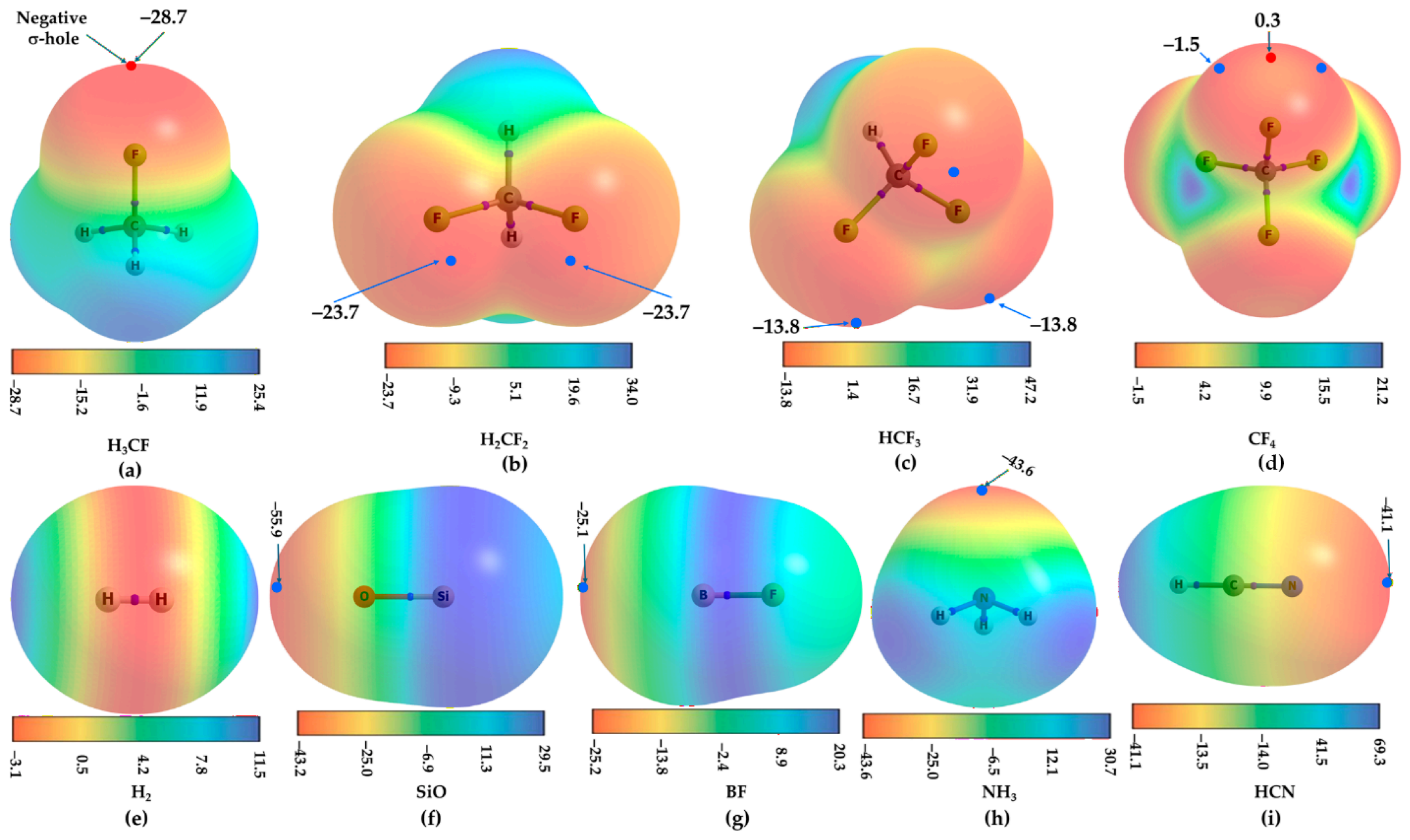
(**a**–**i**) QTAIM-based molecular graphs superimposed on the corresponding MESP surfaces for the nine neutral monomeric entities, with the electrostatic potential mapped onto the 0.001 a.u. electron-density isosurface. Selected local maxima (small red circles) and minima (small blue circles) of the potential are indicated. Bond paths are shown in atomic colors, while bond critical points are represented by small blue spheres along the bond paths, and potential values are given in kcal mol^−1^.

We examined fluorinated derivatives of CH_4_, namely CH_3_F, CH_2_F_2_, CHF_3_, and CF_4_, to assess how progressive fluorination influences the electrostatic potential distribution at fluorine. In CH_3_F, the region along the extension of the C–F bond exhibits a distinctly negative σ-hole (*V_S,max_* = −28.7 kcal mol^−1^), indicating a predominantly electron-rich character at that site. With increasing fluorination in CH_2_F_2_ and CHF_3_, this negative region is progressively attenuated and effectively neutralized, reflecting the cumulative electron-withdrawing influence of additional fluorine substituents. In CF_4_, the σ-hole along the C–F bond extension becomes weakly positive (*V_S,max_* = 0.3 kcal mol^−1^), consistent with a reversal in the local electrostatic character due to the symmetric and strongly electron-withdrawing environment created by complete fluorination. The positive character of the σ-hole on F in CF_4_ was resolved at the standard 0.001 a.u. electron density isosurface, in contrast to what was discussed elsewhere [27,57]. Figure 4a–d shows the MESP plots for the series CH_4−*n*_F*_n_* (*n* = 1–4), illustrating regions dominated by negative and positive electrostatic potentials.

Assuming the arbitrary nature of the isodensity envelope, values up to 0.0020 a.u. were used to map the potential for CH_2_F_2_ and CHF_3_; however, no maximum of potential was observed on the surface of F in these two molecules. For all four systems, increasing the isodensity envelope to 0.0020 a.u. does not appreciably alter the sign of the electrostatic potential. For instance, *V_S,max_* and *V_S,min_* values on F in CF_4_ were found to be 1.9 and −1.0 kcal mol^−1^, respectively, suggesting that the electrophilic character of the σ-hole is preserved.

The aforementioned behavior, namely the absence of a σ-hole on F in CH_2_F_2_ and CHF_3_ in the solution phase, contrasts with that observed in the gas phase. For instance, the *V_S,max_* on each fluorine is −13.9 and −6.1 kcal mol^−1^ for CH_2_F_2_ and CHF_3_, respectively, when the 0.0013 a.u. isodensity surface is used to map the potential, indicating that both systems exhibit a negative σ-hole. The apparent discrepancy may be attributed to dielectric screening and molecular polarization in solution, which smooth the anisotropic electrostatic potential on the molecular surface, thereby eliminating the directional extremum required to define a σ-hole on F.

Apart from the CH_4−*n*_F*_n_* (*n* = 1–4) systems discussed above, we also considered H_2_, SiO, BF, NH_3_, and HCN as interacting partner molecules. This choice is motivated by the presence of electron-rich regions that can act as Lewis basic sites, as reflected in the negative minima of the electrostatic potential. In particular, the central region of H_2_ and the terminal atoms O in Si=O, B in B≡F, N in NH_3_, and N in HCN exhibit such nucleophilic character. The electrophilic and nucleophilic features of these molecules are illustrated in Figure 4e–i.

**Figure 5:**
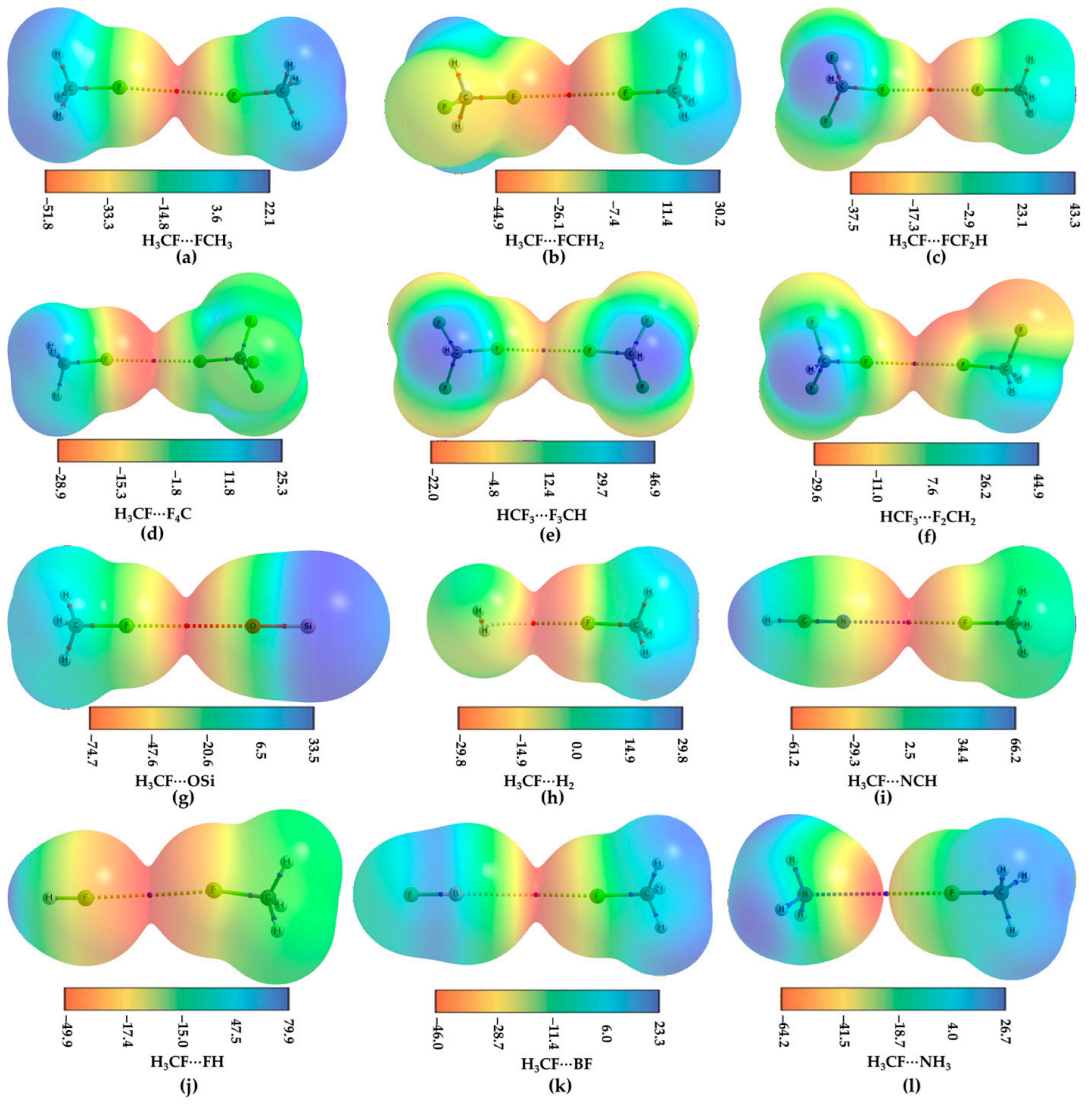
(**a**–**l**) QTAIM-based molecular graphs superimposed on the corresponding MESP surfaces for twelve 1:1 dimers, illustrating singular F···B (B = F, O, N, and B) close contacts between the monomers, where the negative σ-holes along the C–F bond extensions interact attractively with negative sites on the same or a different molecular entity. The electrostatic potential is mapped onto the 0.001 a.u. electron-density isosurface and values are in kcal mol^−1^. Bond paths (solid and dotted lines) are shown in atomic colors, while bond critical points are represented by small blue spheres along the bond paths.

Figure 5a–l presents the QTAIM-based molecular graphs superimposed on the corresponding MESP surfaces for the dimers formed between CH_3_F and CH_2_F_2_, CHF_3_, CF_4_, HCN, SiO, H_2_, HF, BF, and NH_3_. In these complexes, the negative σ-hole located on F along the extension of the C–F bond in CH_3_F (as well as in CH_2_F_2_ and CHF_3_) engages in interactions with either similarly polarized regions on other fluorinated species or with Lewis basic sites present on the partner molecules, including F, O, B, and N atoms.

The dielectric environment does not alter the intrinsic MESP characteristics of the isolated monomers; in particular, the σ-hole on fluorine remains negative in the absence of interaction. However, upon complex formation, the electron density distribution of each monomer undergoes polarization and relaxation in response to the electric field of the partner molecule. Consequently, the electrostatic potential in the dimer cannot be interpreted as a simple superposition of monomeric MESP features. The apparent interaction between regions of similar (negative) potential arises from a combination of anisotropic polarization, charge redistribution, and contributions from other electrostatic regions of the molecules, rather than from a reversal of the sign of σ-hole on F. In this sense, the σ-hole concept is strictly valid only for isolated monomers, and its direct extension to interacting geometries requires caution.

Nevertheless, the optimized intermolecular geometries adopt linear or quasi-linear arrangements, consistent with directional electrostatic alignment. The F···B intermolecular separations fall in the range of 3.003–4.146 Å, while the corresponding bond angles span 167–180°, reflecting a pronounced tendency toward near-linear interaction geometries.

QTAIM molecular graphs further confirm the presence of well-defined bond paths and corresponding bond critical points between the interacting regions, indicating the existence of specific intermolecular interaction channels. The electron density $\left(\rho_{b}\right)$ at the bond critical points is very small, while the corresponding Laplacian values $\left(\nabla^{2}\rho_{b}\right)$ are small and positive, consistent with weak closed-shell intermolecular interactions. Complementary MESP analysis reveals partial overlap of the interacting electrostatic basins in all cases except the CH_3_F···NH_3_ complex (Figure 5l), thereby supporting the presence of directionally governed interactions mediated by the fluorine-centered electrostatic σ-hole feature along the C–F bond extension.

Table 1 also lists *E_b_* of all the binary complexes. The largest binding energy is observed for the CHF_3_···CH_2_F_2_ complex (−0.54 kcal mol^−1^), while the weakest interactions (−0.20 kcal mol^−1^) are found for both the H_3_CF···BF and H_3_CF···NH_3_ complexes. This trend is broadly consistent with the corresponding intermolecular separations, with shorter distances in the more strongly bound system and comparatively longer separations in the weakly interacting cases.

In most cases, formation of these dimers is not favorable in the gas phase, as geometry optimization often leads to partial or complete dissociation of the interacting monomers due to dominant Coulombic repulsion. As discussed below, this behavior is consistent with the SAPT energy decomposition results, where the total interaction energy was found to be positive for several complexes.

Since both interacting regions correspond to Lewis basic sites associated with negative σ-hole regions in CH_4−*n*_F*_n_* (see Figure 5a–f), the resulting intermolecular interactions cannot be classified as conventional halogen bonds. Instead, these contacts are more appropriately described as fluorine-centered, σ-hole driven anti-electrostatic interactions given they lack classical electrostatic complementarity. Table 2 lists the detailed physical properties of all the 12 binary complexes investigated.

The SAPT energy decomposition for the dimers of H_3_CF with CH_4−*n*_F*_n_* (*n* = 1–4) reveals a systematic evolution of intermolecular interactions with increasing fluorination. As can be seen from the data in Table 3, the electrostatic contribution is highest for H_3_CF···FCH_3_ (1.09 kcal mol^−1^), decreases slightly for H_3_CF···FCFH_2_ (0.74 kcal mol^−1^), and then drops sharply to 0.01 kcal mol^−1^ for H_3_CF···FCF_3_, indicating an overall reduction in Coulombic interactions as hydrogen atoms are progressively replaced by fluorine, consistent with the increasing electrophilic character of interacting σ-hole on F in CH_4−*n*_F*_n_* across the series. The exchange term remains consistently positive across the series (≈0.11–0.26 kcal mol^−1^) and becomes relatively more important in the highly fluorinated systems, reflecting enhanced Pauli repulsion. Induction contributions are small and weakly attractive (≈−0.03 to −0.07 kcal mol^−1^), suggesting limited polarization effects throughout. Dispersion interactions increase steadily in magnitude from −0.32 to −0.48 kcal mol^−1^, indicating that they play an increasingly dominant stabilizing role as fluorination increases. Despite this, the *E_SAPT_* decreases from a clearly repulsive value (0.81 kcal mol^−1^) for H_3_CF···FCH_3_ (Figure 5a) to a slightly attractive value (−0.27 kcal mol^−1^) for H_3_CF···FCF_3_ (Figure 5d), highlighting a gradual shift toward dispersion-dominated stabilization. On the other hand, the binding energies *E_b_* become progressively more negative from −0.34 to −0.52 kcal mol^−1^. This apparent discrepancy arises because SAPT calculations were performed in the gas phase on solvated geometries, whereas *E_b_* reflects environment-mediated stabilization. We did not observe any clear dependence of *E_b_* or *E_SAPT_* on the intermolecular distance across the entire series of complexes listed in Table 1 and Table 2.

The H_3_CF···NCH (Figure 5i), H_3_CF···OSi (Figure 5g) and H_3_CF···FH (Figure 5j) heterodimers are dominated by electrostatic repulsion, with positive SAPT interaction energies (*E_SAPT_* = +1.37, +1.53 and +0.76 kcal mol^−1^, respectively) despite stabilizing dispersion and induction contributions, indicating that their negative binding energies likely arise from solvent-mediated stabilization rather than intrinsically favorable gas-phase interactions. However, the H_3_CF···H_2_ complex, Figure 5h, exhibits an almost negligible electrostatic contribution (+0.02 kcal mol^−1^) and is stabilized mainly by dispersion, resulting in a weakly attractive interaction (*E_SAPT_* = −0.06 kcal mol^−1^) characteristic of a dispersion-bound complex. The small positive electrostatic term may originate from interaction between the negative σ-hole on fluorine in H_3_CF and the lateral positive region on H in H_2_, although the H···F contact (*r*(H···F) = 3.003 Å and ∠C–F···H = 173.0°) itself remains anti-electrostatic in character.

The H_3_CF···BF (Figure 5k) and H_3_CF···NH_3_ (Figure 5l) complexes exhibit comparable supermolecular binding energies in solution of approximately −0.20 kcal mol^−1^, yet the SAPT analysis reveals substantial differences in the physical origin and nature of the corresponding intermolecular interactions. For H_3_CF···NH_3_, the interaction is dominated by a significant electrostatic repulsion component, $E_{elst}=+1.11$ kcal mol^−1^, while the stabilizing induction and dispersion contributions are relatively small, amounting to only −0.06 and −0.16 kcal mol^−1^, respectively. Consequently, the total SAPT interaction energy remains strongly positive ($E_{SAPT}=+0.93$ kcal mol^−1^), indicating that the complex is intrinsically unbound in the gas phase. Furthermore, the very long intermolecular separation, r(F⋯N)=4.146 Å, together with the absence of significant overlap between the negative electrostatic regions on F and N in the MESP map, suggests that the observed QTAIM bond path likely reflects a weak density-topological feature (ρ_b_ = 0.0009 a.u. and ∇^2^ρ_b_ = 0.0030 a.u. at bcp) rather than a physically meaningful directional intermolecular interaction. In this case, the small negative binding energy obtained in solution may largely originate from dielectric stabilization and methodological limitations associated with continuum solvation treatment of diffuse long-range contacts.

On the other hand, the H_3_CF···BF complex (Figure 5l) displays a comparatively smaller electrostatic repulsion term, $E_{elst}=+0.77$ kcal mol^−1^, together with somewhat larger induction and dispersion contributions of −0.09 and −0.29 kcal mol^−1^, respectively. Although the total SAPT interaction energy remains positive ($E_{SAPT}=+0.60$ kcal mol^−1^), indicating that the isolated dimer is likewise not intrinsically stabilized in the gas phase, the interaction is supported by additional physical descriptors. In particular, partial overlap between the complementary electrostatic regions on F and B is evident from the MESP surface, and a corresponding bond path is identified within the QTAIM framework at r(F⋯B)=3.927Å, with the ρ_b_ and ∇^2^ρ_b_ values of 0.0017 and 0.0054 a.u. at the bcp, respectively. These observations suggest that, unlike the H_3_CF···NH_3_ complex, the H_3_CF···BF assembly may represent a weak but physically meaningful intermolecular interaction whose stabilization in solution arises from a combination of dispersion, polarization, and solvent-mediated attenuation of electrostatic repulsion.

We employed the IGMH method to analyze the binary complexes. This approach is based on comparing the actual electron density gradient with a promolecular reference density constructed via Hirshfeld partitioning, thereby enabling visualization of interaction-induced deviations in the electron density gradient in real space. The method is particularly useful for identifying weak noncovalent interactions through localized gradient features [100].

**Figure 6:**
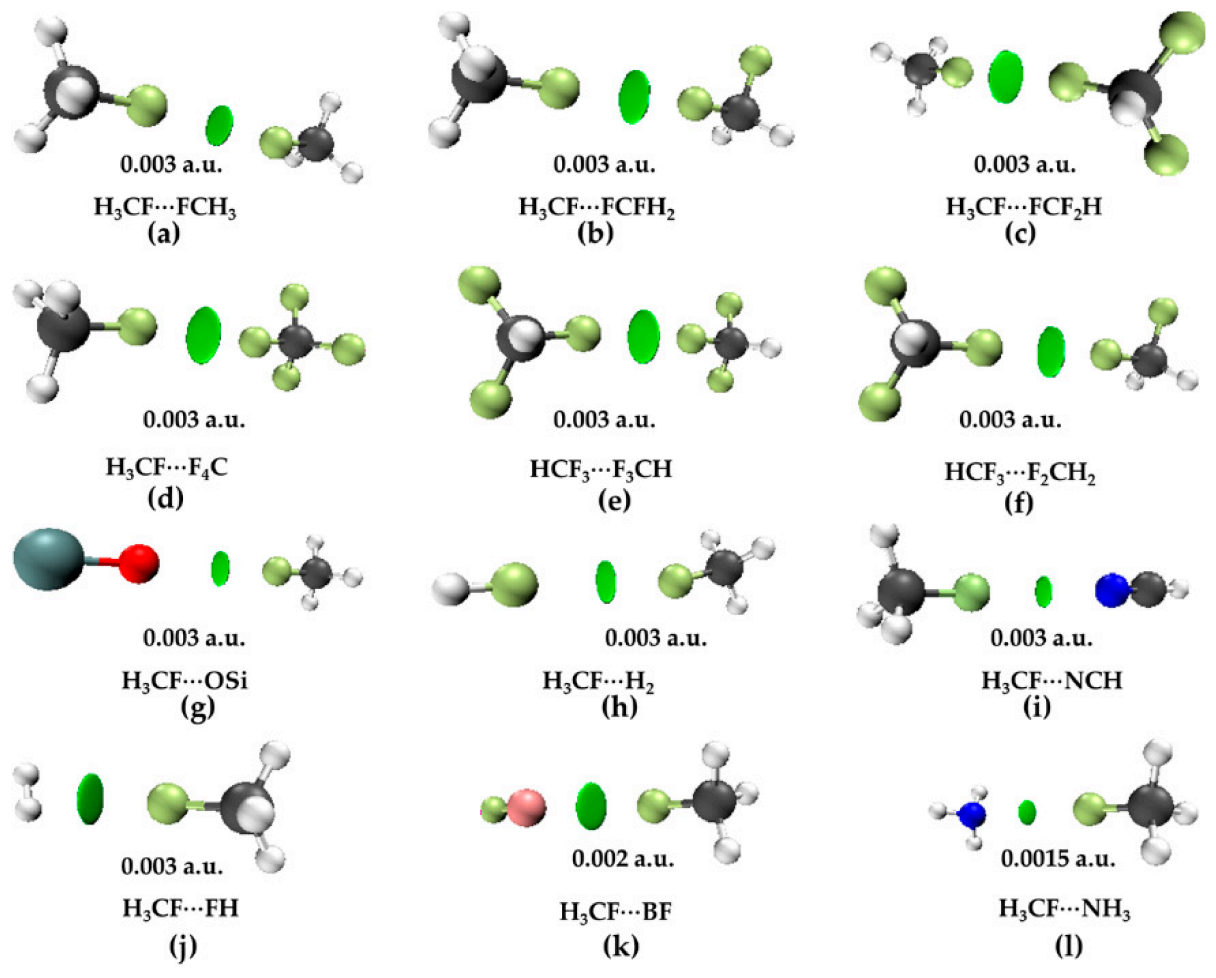
(**a**–**l**) IGMH-based isosurface plots (circular volumes in green) illustrating localized intermolecular interactions between atomic fragments of the interacting monomers in the twelve 1:1 complexes examined. The greenish isosurfaces represent weak and diffuse noncovalent interaction regions. The isovalue used to generate each isosurface is indicated for the corresponding complex.

As shown in Figure 6a–l, a semi-circular green isosurface appears in the intermolecular region between the monomers for each binary complex examined. These greenish isosurfaces correspond to weak attractive interaction regions between the interacting fragments. In most cases, the isosurface is faint at an isovalue of 0.004 a.u. and disappears when the isovalue is increased to 0.005 a.u., whereas it becomes more continuous and spatially pronounced at an isovalue of 0.003 a.u. The extent and intensity of the isosurface provide a qualitative indication of the spatial distribution and relative strength of the intermolecular interaction. For the H_3_CF···BF and H_3_CF···NH_3_ complexes, very low isovalues were required to visualize the intermolecular interaction regions, consistent with the extremely weak and diffuse nature of these interactions.

#### 3.2.3. Negative σ-Holes on Fluorine in C_6_H_6−*n*_F*_n_* and Their Directional Bonding Propensity

**Figure 7:**
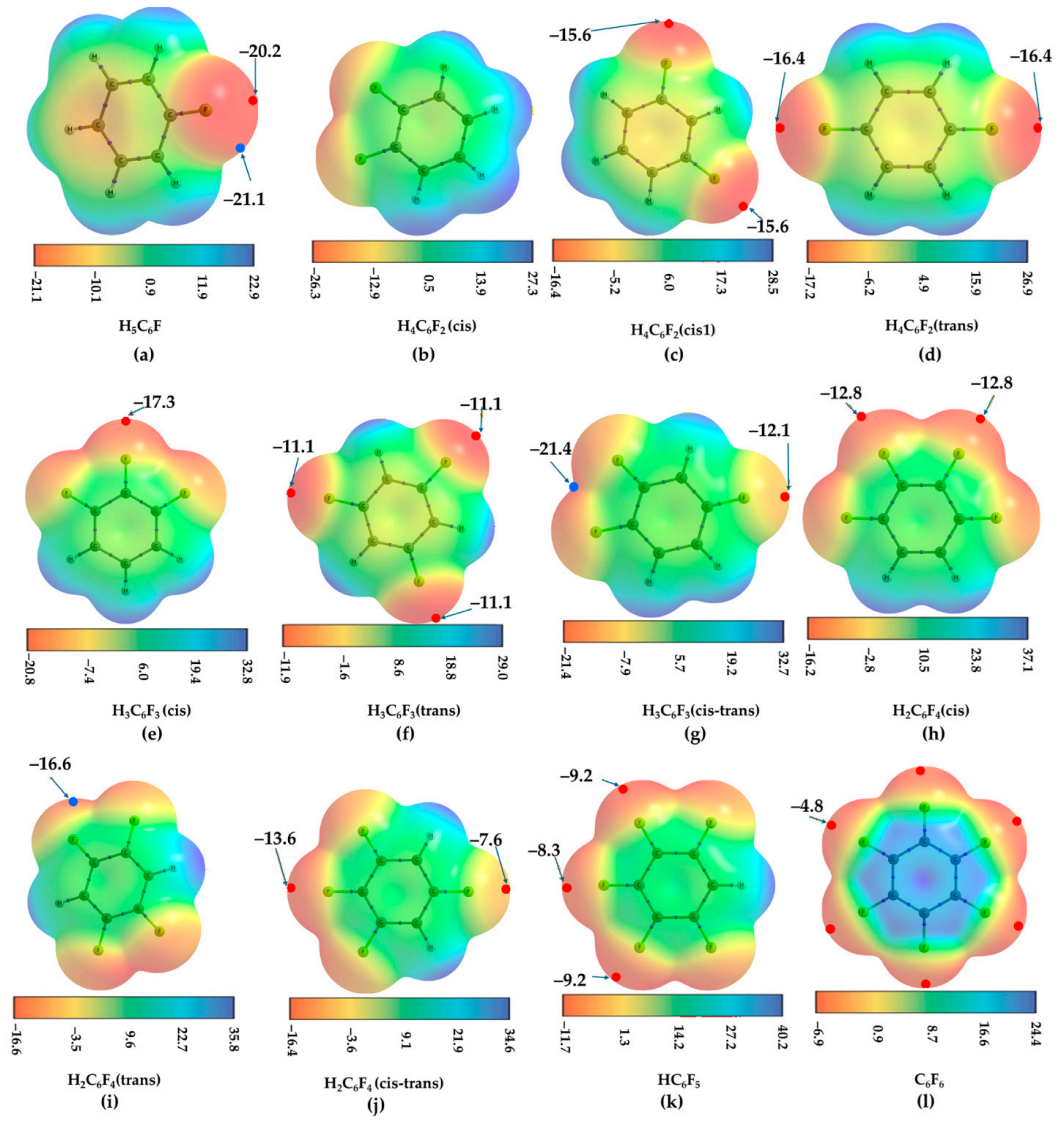
(**a**–**l**) Illustration of the 0.001 a.u. isodensity surface mapped electrostatic potential for the twelve-membered set of fluorinated benzene derivatives, C_6_H_6−*n*_F*_n_* (*n* = 1–6), obtained at the M06-2X/SMD level of theory using water as the solvent. The QTAIM-based molecular graph is superimposed in each case. Maxima of the electrostatic potential (red circles) and selected minima (blue circles) are indicated, with values given in kcal mol^−1^. “cis” and “trans” denote fluorine atoms adjacent to each other and opposite each other on the ring, respectively, whereas “cis–trans” refers to adjacent and trans-arranged fluorine substituents on the ring. Bond paths (solid and dotted lines) are shown in atomic colors, while bond critical points are represented by small blue spheres along the bond paths.

Figure 7a–l illustrates the QTAIM molecular graph superimposed with the MESP plots for 12 fluorinated benzene derivatives, C_6_H_6−*n*_F*_n_* (*n* = 1–6). The MESP plots reveal that fluorine atoms are characterized by negative electrostatic potential. This effect is most pronounced in the monofluorinated species (C_6_H_5_F) and least pronounced in perfluorobenzene (C_6_F_6_), as reflected in the negative $V_{S,\max}$ values along the C–F bond extensions (cf. Table 4).

As observed for certain members of the CH_4−*n*_F*_n_* series, a similar trend is found in several fluoro-arene derivatives that do not exhibit a well-defined σ-hole on fluorine in the solution phase. In particular, with the exception of C_6_H_5_F (Figure 7a), C_6_H_4_F_2_ (Figure 7c,d), C_6_H_3_F_3_ (Figure 7f) and C_6_F_6_ (Figure 7l), the remaining fluorinated species do not exhibit the expected σ-holes on fluorine atoms along the outermost extension of the C–F bonds. For instance, in C_6_H_4_F_2_ (Figure 7b), C_6_H_3_F_3_ (Figure 7g), C_6_H_2_F_4_ (Figure 7i), and C_6_HF_5_ (Figure 7k), where σ-holes would be anticipated on each fluorine atom, no such features are observed even when the 0.002 a.u. isodensity envelope was used to map the potential.

This result contrasts with that obtained in the gas phase. As summarized in Table 4, the σ-hole remains negative on the surface of each fluorine atom across all 12 monomers investigated. The data show a clear and systematic evolution of both the σ-hole at the C–F bond extension and the π-hole at the C_6_ centroid as fluorination increases in C_6_H_6−*n*_F*_n_* (*n* = 1–6). In fact, the σ-hole potential at the C–F region becomes progressively less negative with increasing fluorination, indicating a reduction in local electron depletion at individual C–F bonds as the aromatic system becomes more heavily substituted and is electronically redistributed. This demonstrates that the negative nature of the σ-hole is an inherent feature of fluorine in C_6_H_6−*n*_F*_n_*, consistent with findings reported elsewhere.

The π-hole at the C_6_ ring centroid becomes progressively more positive from mono- to perfluorination, reflecting the cumulative electron-withdrawing effect of fluorine substituents. This trend is consistent across both isodensity surfaces (0.001 and 0.0013 a.u.) and is maintained in both gas and solution phases. Variations in substitution pattern (cis, trans, and mixed (cis–trans)) introduce multiple symmetry-inequivalent sites, leading to inequivalent σ-holes on fluorine atoms (e.g., *V_S,max_* values −5.9, −4.9, −5.9, −4.4, and −4.5 kcal mol^−1^ in C_6_HF_5_; Figure 7k).

Solvation modifies the magnitude of electrostatic extrema without altering their overall trends. In C_6_H_5_F, both the C–F σ-hole and the π-centroid become more negative in solution, indicating a uniform enhancement of electrostatic potential. In contrast, C_6_F_6_ exhibits only minor changes in the σ-hole, while the π-centroid, already strongly positive in the gas phase, is further enhanced in solution, consistent with a near-saturated σ-hole and a dominant π-delocalized response.

Among the two isodensity contours examined, the 0.0013 a.u. isodensity envelope consistently captures σ-holes on fluorine atoms across all 12 monomers and is therefore preferred over the 0.001 a.u. surface, which fails to identify maxima in several cases (e.g., C_6_H_2_F_4_; see Figure 7i and Table 4).

**Figure 8:**
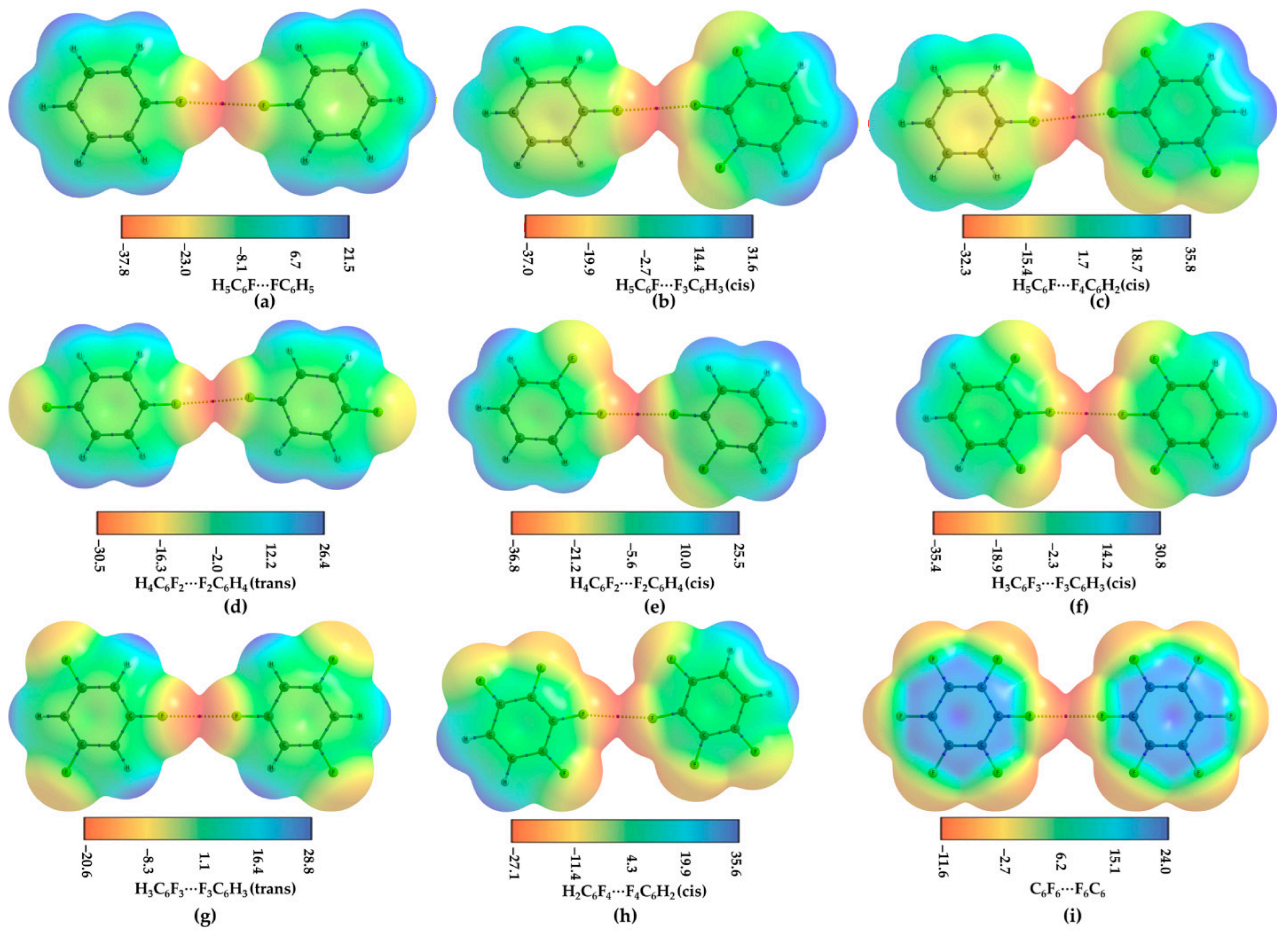
(**a**–**i**) Electrostatic potential mapped onto the 0.001 a.u. electron-density isosurface for nine dimers containing fluorinated benzene derivatives, C_6_H_6−*n*_F*_n_* (*n* = 1–6), with the corresponding QTAIM molecular graphs superimposed, illustrating overlap of the MESP regions associated with the interacting atomic basins. Potential values are given in kcal mol^−1^. Bond paths (solid and dotted lines) are shown in atomic colors, while bond critical points are represented by small blue spheres along the bond paths.

**Figure 9:**
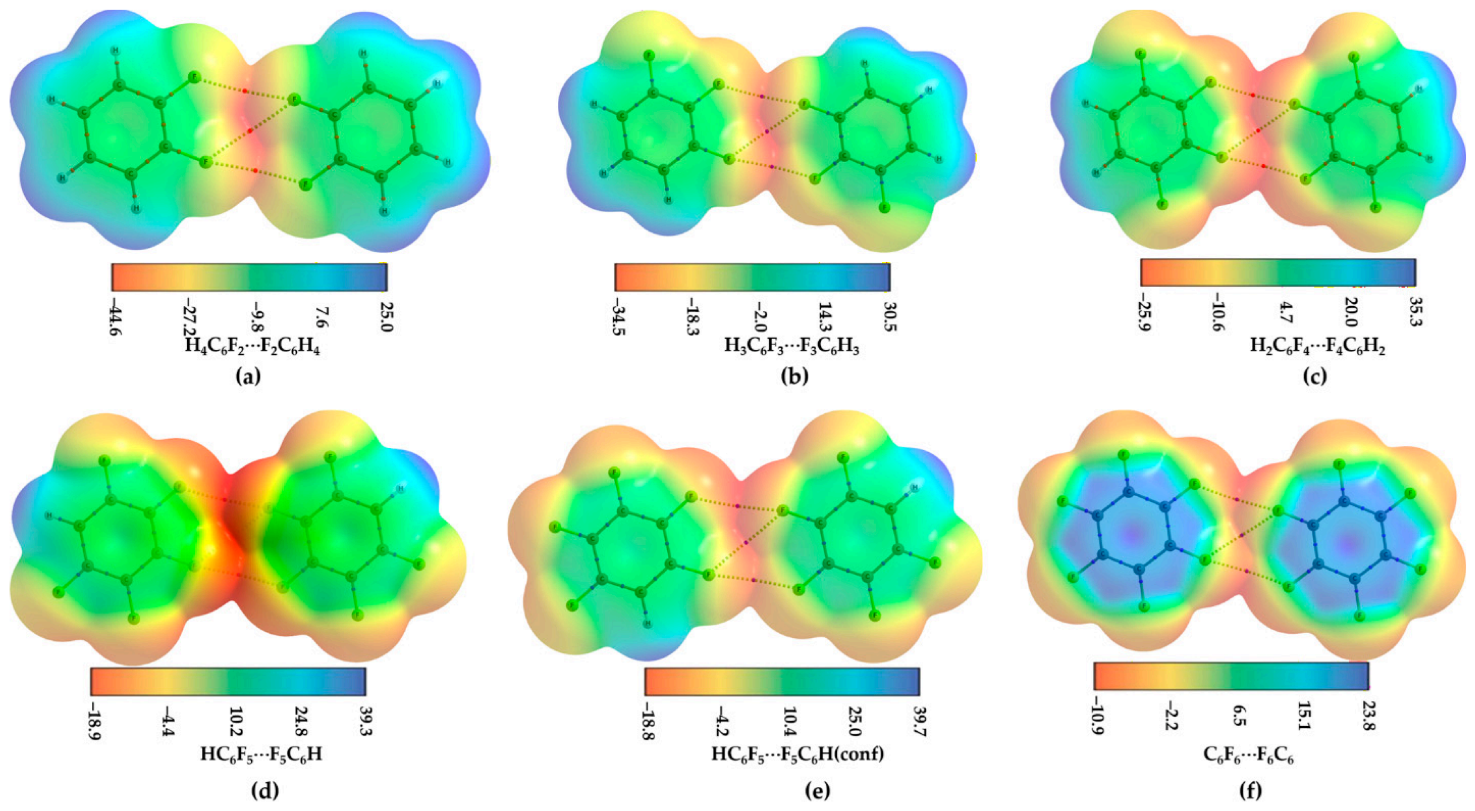
(**a**–**f**) Electrostatic potential mapped onto the 0.001 a.u. electron-density isosurface for six dimers containing fluorinated benzene derivatives, C_6_H_6−*n*_F*_n_* (*n* = 1–6), with the corresponding QTAIM molecular graphs superimposed, illustrating multiple F···F close contacts and overlap of the MESP regions associated with the interacting atomic basins. Potential values are given in kcal mol^−1^. Bond paths (solid and dotted lines) are shown in atomic colors, while bond critical points are represented by small blue spheres along the bond paths.

Two sets of dimers were examined for C_6_H_6−*n*_F*_n_* (*n* = 1–6), illustrated in Figure 8 and Figure 9. The intermolecular close contacts observed in these dimers resemble those found between molecular building blocks in related crystal structures, despite the monomers not being compositionally identical (see Figure 2 for representative examples).

Each dimer in Figure 8a–i exhibits a single F···F contact, analogous to those shown in Figure 2a,c. The F···F close contacts in each display C–F···F and F···C–F angles ranging from approximately 164.3° in H_2_C_6_F_4_···F_4_C_6_H_2_ (cis) to 177.6° in H_3_C_6_F_3_···F_3_C_6_H_3_ (cis), Table 5. The deviation from linearity suggests that the intermolecular interactions occur between anisotropic regions of unequal charge density on the opposing monomers. The intermolecular distances decrease with increasing fluorine substitution in the dimers, with *r*(F···F) values spanning 3.115 to 2.986 Å (see Table 5). This trend is somehow consistent with the increasing positive character of the interacting fluorine atoms in the corresponding monomers, suggesting a concomitant strengthening of the intermolecular interactions.

Each dimer in Figure 9 displays three F···F contacts, similar to those in Figure 2b. In each, two contacts are directional (negative σ-hole centered) and are assigned as Type II interactions based on the quasi-linear nature of ∠C–F···F (see Table 6), while the third arises as a geometric consequence of the former pair and follows Type I topology of bonding. Although this latter contact emerges from a cooperative arrangement, its classification as a secondary interaction may not be ambiguous, particularly because the F···F distance in some cases is longer than that associated with σ-hole-centered interactions.

The F···F distances vary between 3.070 and 3.381 Å for all six dimers (Table 6) and are longer than twice the van der Waals radius of fluorine (2.92 Å), similar to that observed for the other set of complexes listed in Table 5. This corresponds to an increase of approximately 5.1% at the lower limit and 15.8% at the upper limit relative to the summed van der Waals contact distance. The shorter F···F contacts (~3.070 Å) fall within the commonly accepted variability range around van der Waals contact distances, whereas the longer contacts (~3.381 Å) extend beyond this regime. This behavior highlights that noncovalent interactions are not confined to strict van der Waals contact distances but can persist at larger separations where long-range dispersion and electrostatic potential features still contribute to stabilization. In this context, the reported offset of approximately 0.2 Å between *V_S,max_* positions on the 0.001 a.u. electron density surface and van der Waals radii provide a useful geometric reference for interpreting extended intermolecular contacts [86], although it should not be interpreted as a statistical uncertainty in vdW radii. This interpretation is consistent with previous analyses by Dance [101] and Alvarez [97], as well as Politzer et al. [28], who have shown that a significant fraction of noncovalent interactions occur at distances exceeding the sums of van der Waals radii, reflecting the gradual decay of intermolecular forces rather than a sharp spatial cutoff.

The ρ_b_ and ∇^2^ρ_b_ at the F···F bond critical points are very small and positive, consistent with weak, closed-shell interactions. However, no clear linear correlation is observed between these QTAIM descriptors and the F···F bond distances, indicating that the interaction strength is not solely determined by the separation distance but is also influenced by local anisotropy, polarization effects, and the surrounding electrostatic environment.

The results of the SAPT decomposition analysis in Table 7 demonstrate that the F···F interactions in the fluorinated benzene dimers arise from a delicate balance between repulsive electrostatic/exchange contributions and stabilizing dispersion forces. Across the series, both the electrostatic and exchange terms remain positive, indicating that direct F···F contacts are intrinsically unfavorable from Coulombic and Pauli-repulsion viewpoints. Nevertheless, the electrostatic contribution decreases steadily with increasing fluorination, from 0.62 kcal mol^−1^ for H_5_C_6_F···FC_6_H_5_ to only 0.03 kcal mol^−1^ for the fully fluorinated C_6_F_6_···F_6_C_6_ dimer, reflecting reduced charge anisotropy in highly fluorinated systems.

The dispersion term is consistently the dominant attractive component and becomes progressively more stabilizing upon fluorination, increasing in magnitude from −0.58 to −0.72 kcal mol^−1^. The induction term remains relatively small (−0.02 to −0.07 kcal mol^−1^), showing that polarization effects contribute only marginally to the overall stabilization. As a consequence, weakly fluorinated systems such as H_5_C_6_F···FC_6_H_5_ exhibit positive $E_{SAPT2+(3)}$ ($E_{SAPT2+(3)}$ = 0.18 kcal mol^−1^), indicating net repulsion despite negative binding energies. Intermediate systems, including H_5_C_6_F···F_3_C_6_H_3_ (cis) and H_3_C_6_F_3_···F_3_C_6_H_3_ (cis), lie close to the borderline between attraction and repulsion with *E_SAPT_* values near zero, whereas more highly fluorinated dimers such as H_3_C_6_F_3_···F_3_C_6_H_3_ (trans), H_2_C_6_F_4_···F_4_C_6_H_2_ (cis), and C_6_F_6_···F_6_C_6_ exhibit clearly negative *E*_SAPT_ values, signifying genuine intermolecular stabilization. The *E_b_* values follow the same overall trend and become increasingly negative with increasing fluorination, reaching −0.73 kcal mol^−1^ for C_6_F_6_···F_6_C_6_. Clearly, the data confirm that stabilization in these F···F bonded dimers is governed primarily by dispersion, while electrostatic and exchange effects largely oppose complex formation.

The SAPT decomposition across the fluorinated benzene dimers containing multiple F···F interactions (Figure 9) shows a clear transition toward dispersion-dominated binding as fluorination increases, analogous to the singly F···F bonded dimers discussed previously. While the magnitude of $E_{SAPT2+(3)}$ is greater than that of $E_{SAPT2+}$ for each complex (Table 8), electrostatics is generally weak and decreases from ~0.90 kcal mol^−1^ in H_4_C_6_F_2_···F_2_C_6_H_4_ to near-zero or slightly negative in C_6_F_6_···F_6_C_6_, while exchange repulsion gradually increases from ~0.48 to ~0.78 kcal mol^−1^, reflecting enhanced Pauli repulsion in more electron-rich and symmetric systems. Induction remains consistently small and stabilizing (−0.06 to −0.13 kcal mol^−1^), indicating limited polarization effects across the series. The dispersion component is the dominant attractive term in all cases (≈−1.19 to −1.48 kcal mol^−1^), nearly twice as large as that observed for the dimers in Figure 8, and becomes slightly more stabilizing with increased fluorination, thereby governing the overall interaction profile. Consequently, the total binding energies cluster within a narrow range (≈−0.99 to −1.25 kcal mol^−1^), with the most stable complexes being HC_6_F_5_···F_5_C_6_H and C_6_F_6_···F_6_C_6_, highlighting that fluorination primarily tunes stability through dispersion enhancement rather than electrostatics. Despite negative binding energies in both solution and gas phase, the electrostatic contribution remains repulsive, indicating an overall anti-electrostatic character of the F···F interactions.

We also investigated moderately and weakly polar DMSO (ε = 46.7) and THF (ε = 7.6), as well as non-polar n-hexane (ε = 1.9), as solvents using the M06-2X/SMD method. Geometry optimization of the C_6_H_5_F···C_6_F_6_ complex in these solvents resulted in only slightly perturbed structures, with σ-hole centered F···F distances of 3.110, 3.126, and 3.129 Å, respectively, corresponding to ∠C–F···F angles of 167.1°/177.5°, 167.5°/168.1°, and 168.1°/168.2°, respectively. These results indicate that the dimer geometry obtained in water (ε ≈ 78.4; Table 5) is not an artifact of the implicit solvation model, although the intermolecular angles exhibit modest solvent dependence, while the key intermolecular distance remains essentially unchanged. The binding energies of the dimer are approximately −0.52, −0.33, and −0.21 kcal mol^−1^ in DMSO, THF, and n-hexane, respectively, showing a systematic decrease in stabilization with decreasing solvent polarity. Nevertheless, even in non-polar media, the interaction remains weakly attractive and structurally persistent.

Furthermore, we have also evaluated the stability of the fluorinated benzene complexes in the gas phase using the MP2/cc-pVTZ level of theory, consistent with that reported elsewhere [102]. The binding energies show a clear trend of increasing stabilization with greater fluorination of the benzene dimers. The uncorrected ΔE(MP2) values become progressively more negative across the series, ranging from −0.67 kcal mol^−1^ for H_4_C_6_F_2_···F_2_C_6_H_4_ to −1.29 kcal mol^−1^ for the fully fluorinated C_6_F_6_···F_6_C_6_ complex, indicating that fluorination systematically enhances overall binding strength. A consistent correction is observed upon inclusion of basis set superposition error (BSSE), where ΔE(MP2(BSSE)) refines the interaction energies while preserving the same ordering and overall trend. Even after BSSE correction, the interaction energies remain increasingly stabilizing, varying from weak binding in lightly fluorinated systems (+0.22 mol^−1^) to the most stable interaction in the fully fluorinated pair (−0.42 mol^−1^), thereby confirming that the M06-2X level results discussed above for the arene systems are not computational artefacts.

**Figure 10:**
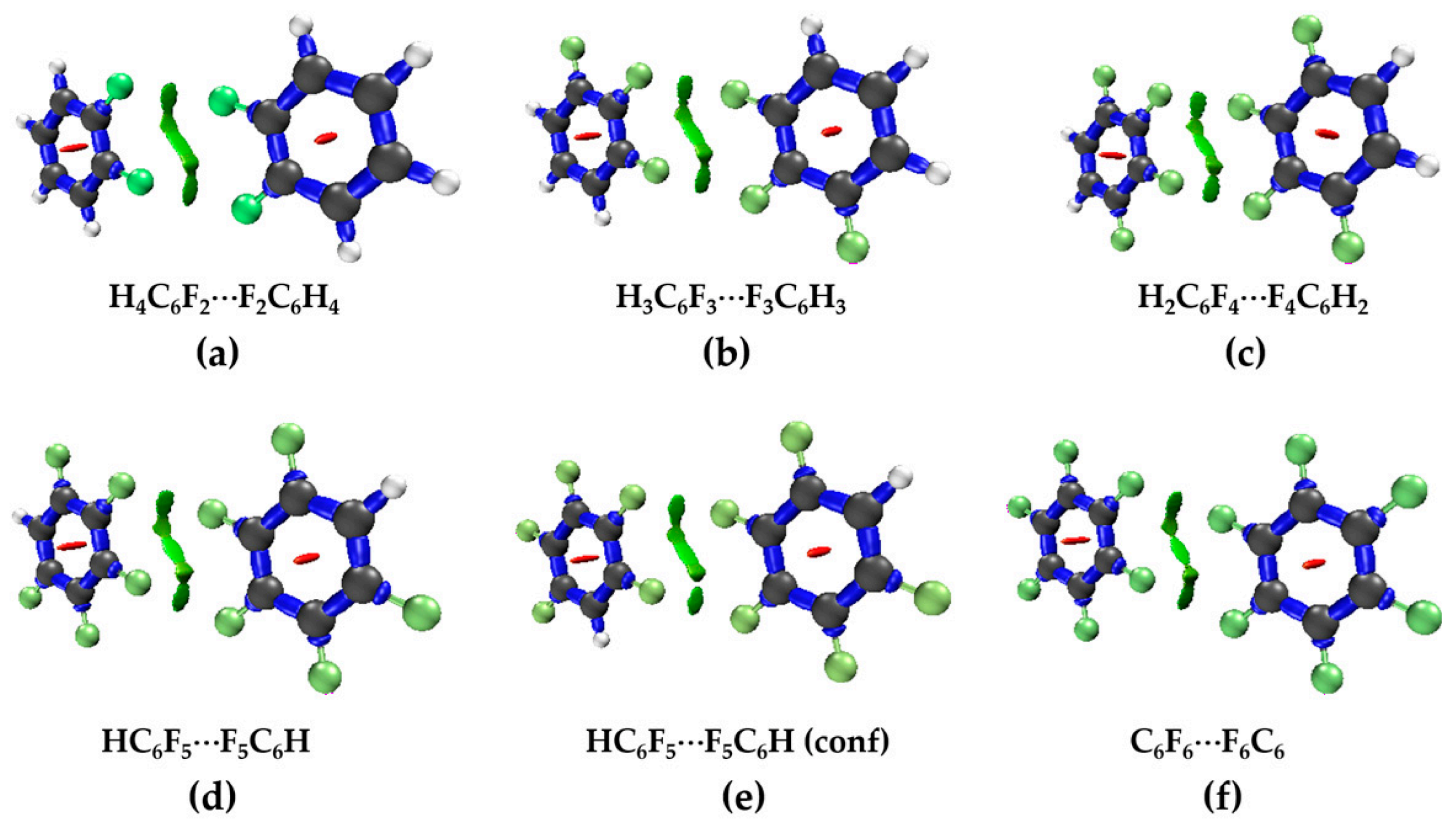
(**a**–**f**) IRI isosurface plots of homomolecular dimers of C_6_H_6−*n*_F*_n_* (*n* = 2–6). The green and brown isosurface regions appearing between the molecules indicate weak an extremely weak, noncovalent interactions. The green/brown isosurfaces within the monomer framework correspond to attractive interactions (e.g., covalent bonding regions), whereas the red spikes denote regions of strong steric repulsion within the molecular framework.

Nonetheless, we have employed the IRI approach to the dimers listed in Figure 10, which is a real-space scalar field based on a reduced density gradient-like function that provides a continuous representation of interaction regions, capturing both covalent bonding domains and noncovalent interaction regions within a unified visualization framework. Figure 10a–f for the homomolecular dimers of C_6_H_6−*n*_F*_n_* (*n* = 2–6), in which, the covalent bonds (e.g., C=C and C–F) are indicative of blue isosurface regions, whereas the F···F close contacts correspond to green isosurfaces located between the interacting atomic basins. In addition, brownish isosurfaces between the monomers suggest possible attractive interactions between fluorine atoms of one monomer and the carbon atoms of the arene moiety in the other. These results are consistent with those revealed using the MESP and QTAIM models.

Short contacts involving F···F interactions and triangular F_3_ motifs are key structural features of the high-pressure perfluorobenzene (C_6_F_6_) phase II formed above 0.89 GPa [54]. Fluorine atoms are strongly electronegative and weakly polarizable [43,60]; nevertheless, their proximity in molecular crystal structures has been interpreted in terms of electron-pair···σ-hole-type interactions. In C_6_F_6_ phase II, the molecules are arranged into unprecedented planar pseudohexagonal sheets containing multiple F···F contacts, which are significantly more frequent than in the ambient-pressure low-temperature phase I, where F···F contacts organize molecules into linear trimers.

The short F···F contacts observed in phase II are geometrically compatible with halogen-bond-like angular preferences and resemble the characteristic X_3_ motif found in heavier halobenzenes (C_6_X_6_; X = Cl, Br, I). However, in isostructural C_6_X_6_; crystals, molecules are strongly inclined with respect to one another and form three-dimensional networks dominated by conventional X···X halogen bonding interactions.

Despite the absence of a conventional σ-hole on fluorine, theoretical and experimental studies of fully fluorinated aromatic systems have shown that directional F···F contacts can arise. In such systems, attractive dispersion forces can overcome electrostatic repulsion between fluorine atoms, while anisotropic charge density distributions and electrostatic potential gradients contribute to directionality. The coexistence of these competing effects can lead to organized motifs such as “windmill”-type arrangements, containing multiple C–F···F interactions between neighboring molecules.

The formation of F···F-bonded sheets in the C_6_F_6_ phase II has therefore been rationalized not only in terms of weak electron-pair···σ-hole-type interactions analogous to halogen bonding concepts, but also through enhanced electron-density redistribution, polarization effects, and resonance-driven stabilization of fluorinated molecular frameworks under highly electronegative and high-pressure environments.

**Figure 11:**
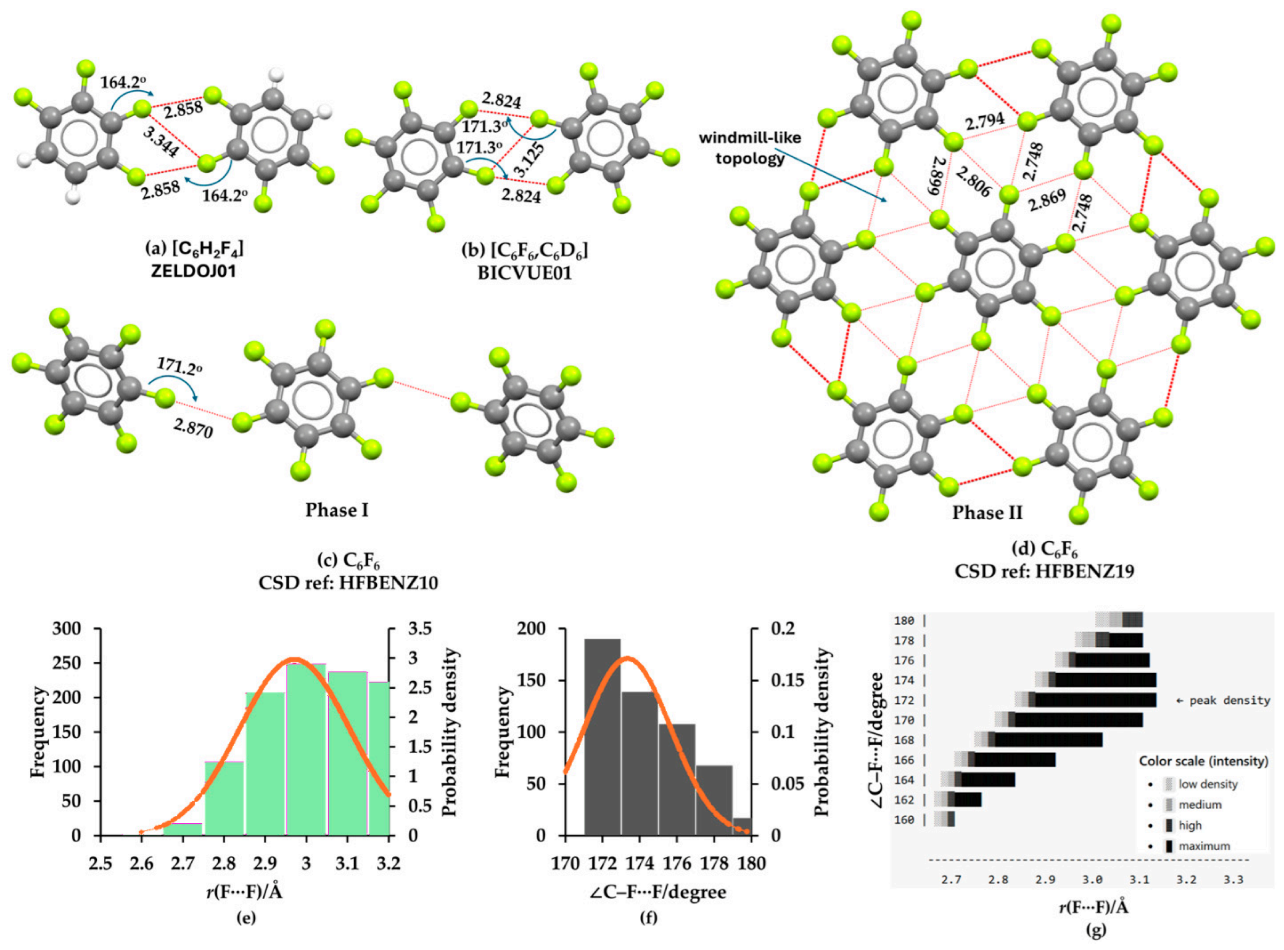
Selected crystal structures of (**a**) 1,2,3,4-tetrafluorobenzene (C_6_H_2_F_4_) and (**b**–**d**) perfluorobenzene (C_6_F_6_) illustrating the directional nature of the negative σ-hole and its role in the formation of F···F close contacts between interacting building blocks, along with their variability. (**e**,**f**) Histograms representing the distributions of F···F distances and C–F···F angles observed in crystals featuring 1044 close contacts with intermolecular geometries similar to those shown in (**b**). The distributions are shown with overlaid Gaussian curves constructed from the corresponding sample means and standard deviations. The left *y*-axis represents frequency, while the right *y*-axis shows the normalized probability density. The angular distributions indicate a preference for near-linear geometries with a gradual decrease toward 180°. The D_6_-benzene molecule is omitted in (**a**) for clarity. Selected intermolecular distances and angles are given in Å and degrees, respectively. (**g**) Schematic representation of the C–F···F geometric distribution as a function of F···F distance (2.7–3.3 Å) and C–F···F angle (160–180°), derived from 931 hits comprising 2210 close contacts. Density is qualitatively encoded using a grayscale ASCII shading scheme, where darker regions correspond to higher relative population. The most populated region is centered at approximately 172–173° and 2.9–3.0 Å.

We note that approximately 464 crystal structures exhibit similar F···F close contacts between interacting monomers, as illustrated by the dimers in Figure 11. These were identified from a CSD survey in which pairs of F···F distances between fluoro-substituted fragments on two aromatic units were constrained to 2.5–3.2 Å, with at least one contact having a C–F···F angle in the range 170–180°. This resulted in a total of 1044 close contacts, with F···F distances ranging from 2.599 to 3.200 Å (standard deviation = 0.124 Å) and C–F···F angles varying between 170° and 180° (standard deviation = 2.3°). The corresponding histograms Figure 11e,f show the distributions of F···F distances and C–F···F angles, with maxima at ~2.9–3.0 Å and ~173°, respectively. Three representative examples are shown in Figure 11a,b,d, whereas Figure 11c was obtained from a different search.

Note that the variable nature of the constraints can lead to different results. For example, when the distance between a pair of directional F···F contacts was constrained to 1.8–3.3 Å, with at least one contact exhibiting a C–F···F angle in the range of 165–180°, the resulting number of hits was 1112, corresponding to 1337 × 2 = 2674 close contacts. However, when the angular constraint was relaxed to 160–180° for both the C–F···F angles while maintaining the same distance range, a total of 931 hits was obtained, corresponding to 2210 close contacts.

The C–F···F geometric distribution, derived from 931 crystals (2210 interaction hits), exhibits a well-defined preference in both distance and angular space. As shown in the schematic density map, Figure 11g, the majority of contacts cluster around an F···F distance of ~2.9–3.0 Å, and a C–F···F angle near 172–173°. This indicates a strong directional preference for near-linear geometries, consistent with a stereoelectronic alignment of the interacting C–F bonds. The relatively narrow angular spread suggests that these contacts are not randomly distributed but instead reflect a constrained interaction regime. The broader distribution along the distance coordinate implies a higher tolerance in intermolecular separation compared to angular distortion.

Short F···F contacts and their windmill-like triangular F_3_ motifs (∠C–F···F)_mean_ = 60.0°) are prominent features of the high-pressure phase II (space group *C*_2_/*c*, *Z* = 4) of C_6_F_6_ (Figure 11c), formed above 0.89 GPa [54]. Similar motifs could also be traceable in gas-phase calculations [50,51,58]. These are interpreted as arising from electron-pair···σ-hole interactions characteristic of Type II halogen bonding [48,54].

The interacting molecules in phase II adopt an unusual arrangement into planar pseudohexagonal sheets. This is in contrast to the ambient-pressure, low-temperature phase I (monoclinic space group *P*2_1_/*n*, *Z* = 6), where F···F contacts link molecules into linear trimer-like geometries (Figure 11b). While the geometry of these contacts in the phase II geometry of the system may satisfy angular criteria often associated with halogen bonding and resembles the X_3_ motifs observed in heavier halobenzenes (C_6_X_6_, X = Cl, Br, I), their physical origin is fundamentally different. Given that fluorine in C_6_F_6_ does not exhibit a positive electrostatic potential along the extension of the C–F bond, it would be misleading to recognize the F···F contacts between molecular building blocks in crystalline C_6_F_6_ as conventional halogen bonds [22,83].

We add that each C_6_F_6_ molecule in phase II occupies a pseudohexagonal environment, coordinated by six neighboring molecules within nearly planar sheets. Each molecule participates in 18 short intermolecular F···F contacts within a sheet (Figure 11d), in which each fluorine donates a negative σ-hole to, and accepts two negative σ-holes from, neighboring molecules in order to engage in directional F···F close contacts. Although such an electronegative environment may promote mesomeric contributions leading to localized electronic redistributions, there is no evidence for an instantaneous F^δ−^···F^δ+^ attraction that would stabilize these contacts via classical halogen bonding. Instead, as discussed above, the close packing of molecules within the sheets is more plausibly governed by dispersion and inductive interactions. Consequently, qualitative arguments invoking mesomeric resonance structures that assign a positive character to a fluorine atom in F···F contacts are not supported [54], regardless of whether the contacts occur in phase I or are associated with the windmill-like topology in phase II of C_6_F_6_.

## 4. Discussion

The current work is based primarily on the experimentally observed crystal structures and the CSD analysis, while the DFT-M06-2X calculations provide complementary theoretical support. Although the nature of stationary points for isolated dimers may depend on the computational methodology, the geometrical characteristics of the intermolecular contacts obtained with the adopted functional remain consistent with the crystallographic evidence.

The MESP results that emerged from gas- and solution-phase calculations did not differ markedly in revealing the reactivity of atomic surfaces in isolated molecular entities. A few discrepancies were observed in which the fluorine surface lacked the presence of a negative σ-hole in the solvated geometry, yet these could be identified in the gas-phase calculations. We attribute this difference to dielectric screening and molecular polarization in solution, effects that are absent in the gas phase.

The 0.001 a.u. electron density isosurface is widely used as a proxy for the molecular van der Waals surface and generally encompasses electrophilic and nucleophilic regions for most of the systems examined [28,86,103]. However, in certain cases, this surface does not fully capture the location or magnitude of electrostatic potential extrema, indicating that its reliability as a universal descriptor is limited. This reflects the fact that the 0.001 a.u. isodensity envelope is a geometric approximation rather than a strict boundary of electrostatic behavior. In some systems, analysis on slightly higher-density isosurfaces is required to more accurately probe localized variations in the electrostatic potential distribution. This observation may also be consistent with previous reports that MESP extrema evaluated on the 0.001 a.u. surface may lie approximately 0.2 Å beyond typically known van der Waals radii [28,97].

A rigorous assessment of close contacts within a pair of fluorine atoms on interacting molecules examined shows that electrostatics alone cannot account for the observed stabilization. The MESP analysis confirms that the interacting regions on both the interacting basins in complexes are intrinsically negative, consistent with a purely repulsive electrostatic picture. Energy decomposition analysis performed on the solvated geometries in the gas phase indicates that net interaction arises from a balance between repulsive electrostatics and exchange contributions and stabilizing induction and dispersion terms. In most cases, dispersion is found to be the dominant attractive component, often exceeding individual electrostatic, exchange and induction contributions (*E_disp_* ≫ E_eles_). Clearly, the negative binding energy observed in solution is environment-driven in several cases and mediated by the solvent. Therefore, the observed F···B (B = Lewis base) close contacts are best described as arising from a cooperative interplay of noncovalent forces, rather than from a simple attractive interaction between like-charged regions.

The MESP plot-based representation of molecular dimers provides a useful visual criterion for identifying intermolecular interactions, often yielding interpretations consistent with those obtained from QTAIM, IGMH, or IRI isosurface analyses. In cases where no discernible overlap is observed between the electrostatic surfaces associated with interacting atomic basins, even in the presence of a QTAIM bond path and bond critical point, the corresponding long-range contact is unlikely to represent a physically significant directional interaction, as illustrated for the H_3_CF···NH_3_ dimer. While QTAIM bond paths and bond critical points provide topological information on intermolecular contacts, their presence alone does not necessarily imply energetic stabilization, as such features may also arise in weakly interacting or repulsive regions of the potential energy surface. Accordingly, conclusions regarding the existence and nature of intermolecular interactions are based on a combined interpretation of QTAIM topology, MESP analysis, SAPT energy decomposition, and crystallographic evidence.

Second-order NBO analysis describes (electrophilic) σ-hole interactions in terms of donor–acceptor orbital mixing, typically involving electron donation from a lone-pair orbital, *n*, or a bonding orbital σ into a σ* or RY* antibonding orbital (*n* → σ* or RY* or σ → σ* or RY*). Although this is often referred to as “charge transfer,” it is more accurately interpreted as stabilization arising from orbital delocalization and mixing. For example, the F···F interaction in the H_5_C_6_F···FC_6_H_5_ complex is characterized by the interaction *n*(3)F → RY*(5) F with an *E*^(2)^ stabilization energy of 0.06 kcal mol^−1^. In the HF···FH dimer, the corresponding interactions are *n*(3)F → RY*(1)F and σ(H–F) → RY*(1)F, with *E*^(2)^ values of 0.06 and 0.09 kcal mol^−1^, respectively. Similarly, for H_3_CF···FCH_3_, the delocalization involves σ(C–F) → RY*(6)F in both forward and backward directions, with an associated *E*^(2)^ value of 0.09 kcal mol^−1^. Similar donor–acceptor delocalization can also occur in systems that are not intuitively attractive based solely on charge considerations, such as anion–anion assemblies (e.g., (IO_4_^−^)_2_ [63] and (IO_3_^−^)_2_ dimers [64]. In such cases, the observed stabilization arises in combination with environmental effects, including polarization and dispersion contributions [104,105]. Therefore, second-order NBO “charge-transfer” terms should not be interpreted as definitive evidence for the primary physical origin of intermolecular stabilization. Rather, they represent energetic stabilization arising from orbital mixing within a single-determinant electronic structure description, rather than a uniquely separable physical mechanism of attraction.

Histograms used to illustrate the high abundance of F-centered noncovalent interactions in crystals of CSD do not readily reveal what drives these interactions—whether they originate from positive or negative sites on F in Y–F systems. This is because statistical analyses of crystal structures alone do not provide clear insight into the electrophilic or nucleophilic character of fluorine. It remains unclear whether crystal packing effects, the surrounding environment, or an inherent tendency of covalently bound fluorine to interact with another fluorine atom in a neighboring molecule play the dominant role. However, this may not be the case for crystals containing F-substituted arene moieties similar to those explored in this work, where a majority of observed F···F interactions are suggested by computational models to be influenced by negative σ-hole regions on covalently bound fluorine, with or without solvation.

The present findings open new perspectives on how directional intermolecular interactions involving negative σ-hole centered on covalently bound fluorine are classified and interpreted. The results suggest that motifs traditionally attributed to σ-hole chemistry may, in certain cases, arise from a broader interplay of polarization, dispersion, and environmental effects rather than a pre-existing electrostatic driving force. This highlights the need for a more general framework capable of describing interactions between regions of like or unfavorable electrostatic character under realistic condensed-phase conditions. Future work should focus on systematically mapping these interaction regimes across chemical space and on developing predictive models that incorporate solvent response and many-body effects. Such efforts will be essential for refining bonding classifications and for understanding how weak, cooperative forces govern molecular assembly in both crystals and solution.

## 5. Conclusions

SAPT analysis has enabled us to show that the electrostatic component of the interaction energy is positive (repulsive) and, in some cases, even the total SAPT interaction energy remains weakly positive for isolated gas-phase dimers, but this does not preclude the formation of directional F···F or related contacts in condensed-phase environments. In such cases, stabilization arises from a balance of dispersion, induction, polarization, and environment-dependent effects such as solvation, crystal packing, and counterion screening, which are not fully captured in the isolated dimer model. While the repulsive frozen-electrostatic contribution of SAPT may indicate that classical Coulombic attraction is not the dominant stabilizing factor in these systems, it should be regarded as a mechanistic descriptor rather than an independent criterion for defining bonding categories.

Polarization- and interaction-induced electrostatic anisotropy may develop upon complex formation, but such effects reflect electronic relaxation within the interacting system and do not alter the SAPT observation that the frozen-electrostatic term is repulsive in the isolated fragments. The distinction between pre-existing electrostatic features and interaction-induced redistribution is therefore relevant for interpreting the origin of stabilization.

In this context, systems in which stabilization is observed despite repulsive frozen-electrostatic contributions are described within a decomposition-based framework as exhibiting “anti-electrostatic” behavior. This term is used strictly as a descriptive label and is not intended to redefine halogen bonding or introduce a new interaction class; rather, it highlights interaction regimes that may appear counterintuitive in conventional electrostatic terms. These regimes are characterized by a balance in which dispersion and polarization effects can outweigh classical σ-hole electrostatics, underscoring the need for a mechanistic description that accounts for multiple weak, cooperative interactions in both gas-phase and condensed-phase environments.

## Figures and Tables

**Table 1:** Decomposed SAPT-based energies for the HF···X dimers (X = FH, CO_2_, SO_2_, OCN^−^, NCO^−^). Electrostatic, exchange, induction, and dispersion components, along with the total interaction energy *E_SAPT_* at the SAPT2+3(CCD) level, are in kcal mol^−1^. The corresponding solution-mediated binding energies (*E_b_*) are also included for comparison ^a^.

Dimer	*E_elst_*	*E_exch_*	*E_ind_*	*E_disp_*	*E_SAPT_*	*E_b_*
HF···FH	0.24	0.12	−0.04	−0.18	0.14	−0.27
HF···CO_2_	0.47	0.20	−0.05	−0.36	0.25	−0.46
HF···SO_2_	0.48	0.15	−0.06	−0.30	0.26	−0.26
HF···OCN^−^	5.44	0.29	−0.58	−0.42	4.73	−0.34
HF···NCO^−^	5.49	0.23	−0.59	−0.32	4.82	−0.13

^a^ All SAPT energies are evaluated in the gas phase at the geometries optimized in solution.

**Table 2:** Computed solution phase binding energies (*E_b_*), intermolecular bond distances (*r*(F···B)) and angles (∠C–F···B and ∠F···B–Y), and electron density (ρ_b_) along with its Laplacian (∇^2^ρ_b_) at the corresponding bond critical points for the 12 dimers examined, where B = F, O, N, and B; Y = H, F, O, Si, and C.

Complex ^a^	*E_b_*/(kcal mol^−1^)	*r*(F···B)/Å	∠C–F···B/°	∠F···B–Y/°	ρ_b_/a.u.	∇^2^ρ_b_/a.u.
(a) H_3_CF···FCH_3_	−0.34	3.285	174.2	169.2	0.0019	0.0099
(b) H_3_CF···FCFH_2_	−0.40	3.167	178.4	176.3	0.0023	0.0133
(c) H_3_CF···FCF_2_H	−0.46	3.114	179.6	174.4	0.0025	0.0152
(d) H_3_CF···F_4_C	−0.52	3.047	173.5	173.01	0.0029	0.018
(e) HCF_3_···F_3_CH	−0.51	3.092	176.9	175.6	0.0026	0.0158
(f) HCF_3_···F_2_CH_2_	−0.54	3.015	171.5	174.3	0.003	0.0194
(g) H_3_CF···OSi	−0.25	3.479	180.0	180	0.0019	0.0085
(h) H_3_CF···H_2_	−0.31	3.003	173.0	---	0.0026	0.0119
(i) H_3_CF···NCH	−0.21	3.587	179.1	179.6	0.0017	0.0076
(j) H_3_CF···FH	−0.34	3.274	167.7	176.2	0.0019	0.0102
(k) H_3_CF···BF	−0.20	3.927	176.5	177.1	0.0017	0.0054
(l) H_3_CF···NH_3_	−0.20	4.146	180.0	---	0.0009	0.003

^a^ See Figure 5 for complex geometries.

**Table 3:** SAPT-based decomposition energies for the dimers of H_3_CF with CH_4−*n*_F*_n_* (*n* = 1–4), H_2_, SiO, HF, BF, HCN, and NH_3_, including electrostatics, exchange, induction, and dispersion components along with the total interaction energy *E_SAPT_* computed at the SAPT2+3(CCD) level. Binding energies *E_b_* are included for comparison. All energies are in kcal mol^−1^ ^a,b^.

Dimer System ^c^	*E_elst_*	*E_exch_*	*E_ind_*	*E_disp_*	*E_SAPT_*	*E_b_*
(a) H_3_CF···FCH_3_	1.09	0.11	−0.07	−0.32	0.81	−0.34
(b) H_3_CF···FCFH_2_	0.74	0.16	−0.07	−0.39	0.45	−0.40
(c) H_3_CF···FCF_2_H	0.38	0.19	−0.06	−0.42	0.10	−0.46
(d) H_3_CF···FCF_3_	0.01	0.24	−0.06	−0.46	−0.27	−0.52
(e) H_3_CF···FCF_2_H	0.22	0.26	−0.03	−0.48	−0.03	−0.54
(f) H_3_CF···FCFH_2_	0.25	0.20	−0.04	−0.43	−0.02	−0.51
(g) H_3_CF···OSi	1.89	0.14	−0.14	−0.36	1.53	−0.25
(h) H_3_CF···H_2_	0.02	0.27	−0.05	−0.30	−0.06	−0.31
(i) H_3_CF···NCH	1.37	0.12	−0.09	−0.30	1.10	−0.21
(j) H_3_CF···FH	0.97	0.10	−0.05	−0.26	0.76	−0.34
(k) H_3_CF···BF	0.77	0.20	−0.09	−0.29	0.60	−0.20
(l) H_3_CF···NH_3_	1.11	0.04	−0.06	−0.16	0.93	−0.20

^a^ Calculations were performed in the gas phase using geometries optimized in a solvated environment. ^b^ Binding energies obtained using super-molecular framework in which the energies of induvial optimized species were used. ^c^ See Figure 5 for geometries of the binary complexes.

**Table 4:** Comparison of gas- and solution-phase maxima of electrostatic potential associated with the π-hole at the C_6_ ring centroid and the σ-hole along the C–F bond extensions in C_6_H_6−*n*_F*_n_* (*n* = 1–6). Values are given in kcal mol^−1^. Two isodensity envelopes (0.001 and 0.0013 a.u.) are used for each system ^a–c^.

System	$V_{S,\max}$(C–F)	$V_{S,\max}$(C_6_)_centroid_	$V_{S,\max}$(C–F)	$V_{S,\max}$(C_6_)_centroid_
	0.001	0.001	0.0013	0.0013
C_6_H_5_F	−15.5 [−20.2]	-- [--]	−15.7 [−20.6]	−8.9 [−13.2]
C_6_H_4_F_2_ (cis)	−13.2 (two) [--]	-- [--]	−13.3 (two) [--]	−2.8 [−5.2]
C_6_H_4_F_2_ (cis-1)	−12.1 (two) [−15.6] (two)	−3.5 [−5.7]	−12.2 (two) [−16.0]	−2.7 [−3.8]
C_6_H_4_F_2_ (trans)	−12.9 (two) [−16.4] (two)	−3.5 [−5.6]	−13.1 [−16.7]	−2.6 [−4.8]
C_6_H_3_F_3_ (cis)	−10.0, −11.6, −10.0 [−17.3] (one)	2.0 [1.7]	−10.0, −11.5, −10.0 [−17.3]	3.0 [2.7]
C_6_H_3_F_3_ (trans)	−8.7 (three) [−11.1] (three)	2.4 [2.2]	−8.7 (three) [−11.2]	3.5 [3.3]
C_6_H_3_F_3_ (cis–trans)	−9.8 (one) [−12.1] (one)	2.0 [2.2]	−9.8, −9.8, −10.8 [−12.3] (one)	3.3 [3.1]
C_6_H_2_F_4_ (cis)	−8.4 (two) [−12.8] (two)	7.6 [9.3]	−8.2, −8.2, −7.7, −7.7 [−12.7] (two)	8.9 [10.8]
C_6_H_2_F_4_ (trans)	-- [--]	7.8 [9.6]	−7.5, −7.5, −7.5, −7.5 [--]	9.1 [11.0]
C_6_H_2_F_4_ (cis–trans)	−9.2, −6.6 (two) [−13.6, −7.6] (two)	7.8 [9.5]	−9.0, −6.6, −6.6, −6.6 [−13.6, −7.7]	9.1 [11.0]
C_6_HF_5_	−6.2, −5.2, −6.2 [−9.2, −8.3, −9.2]	13.1 [16.9]	−5.9, −4.9, −5.9, −4.4, −4.5 [−9.1, −8.1, −9.1]	14.6 [18.6]
C_6_F_6_	−3.2 (six) [−4.8 (six)]	18.3 [24.4]	−2.8 (six) [−4.6]	20.0 [26.4]

^a^ “--” denotes the absence of a maximum of potential at the specified isodensity surface. ^b^ Text in parentheses indicate the total number of σ-holes on F present in the molecule. Values in brackets correspond to the solution phase (water solvent). ^c^ The fluorine atom displaying the maximum of potential in the solution-phase is highlighted in Figure 7.

**Table 5:** Computed solution phase intermolecular bond distances (*r*(F···F)) and angles (∠C–F···F and ∠F···F–C), and electron density (ρ_b_) along with its Laplacian (∇^2^ρ_b_) at the corresponding bond critical points for the nine 1:1 complexes examined.

Complex ^a^	*r*(F···F)/Å	∠C–F···F/°	∠F···F–C/°	ρ_b_/a.u.	∇^2^ρ_b_/a.u.
(a) H_5_C_6_F···FC_6_H_5_	3.115	174.0	171.0	0.0025	0.0151
(b) H_5_C_6_F···F_3_C_6_H_3_ (cis)	3.079	176.6	167.2	0.0026	0.0165
(c) H_5_C_6_F···F_4_C_6_H_2_(cis)	3.070	170.1	169.2	0.0027	0.0168
(d) H_4_C_6_F_2_···F_2_C_6_H_4_ (trans)	3.106	176.3	168.4	0.0025	0.0155
(e) H_4_C_6_F_2_···F_2_C_6_H_4_ (cis)	3.074	176.2	176.2	0.0026	0.0167
(f) H_3_C_6_F_3_···F_3_C_6_H_3_ (cis)	3.055	177.6	175.2	0.0027	0.0173
(g) H_3_C_6_F_3_···F_3_C_6_H_3_ (trans)	3.085	167.7	173.7	0.0026	0.0162
(h) H_2_C_6_F_4_···F_4_C_6_H_2_ (cis)	3.016	164.3	164.3	0.003	0.0196
(i) C_6_F_6_···F_6_C_6_	2.986	171.3	171.2	0.0031	0.0208
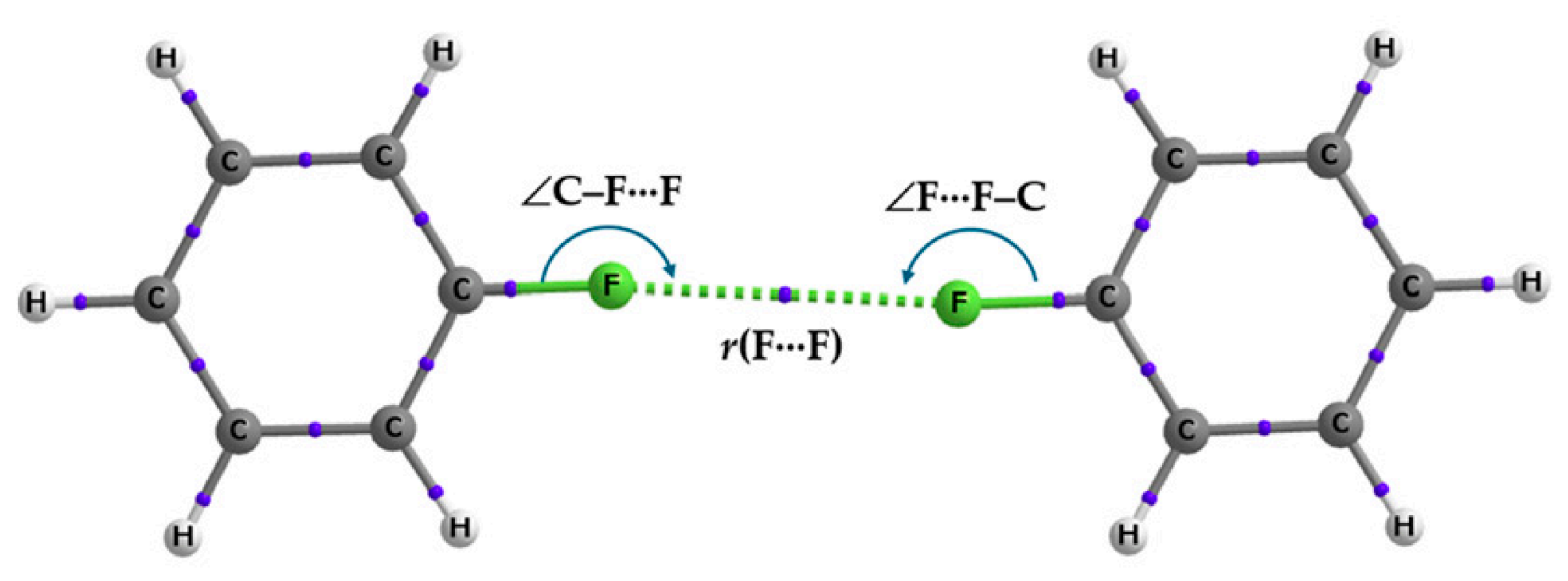					

^a^ See Figure 8 for geometries.

**Table 6:** Computed solution phase intermolecular bond distances (*r*(F···F)) and angles ∠C–F···F, and electron density (ρ_b_) along with its Laplacian (∇^2^ρ_b_) at the corresponding bond critical points for the nine 1:1 complexes examined that possess multiple F···F interactions ^a,b^.

Complex	*r*(F···F)_1_	*r*(F···F)_2_	*r*(F···F)_3_	(∠C–F···F)_1_	(∠C–F···F)_2_	ρ_b_	ρ_b_	ρ_b_	∇^2^ρ_b_	∇^2^ρ_b_	∇^2^ρ_b_
(a) H_4_C_6_F_2_···F_2_C_6_H_4_	3.190	3.381	3.190	170.0	170.1	0.0024	0.0019	0.0024	0.013	0.0090	0.013
(b) H_3_C_6_F_3_···F_3_C_6_H_3_	3.135	3.319	3.135	172.3	172.3	0.0026	0.0022	0.0026	0.015	0.0104	0.015
(c) H_2_C_6_F_4_···F_4_C_6_H_2_	3.093	3.218	3.093	173.1	173.1	0.0029	0.0026	0.0029	0.0168	0.0132	0.0168
(d) HC_6_F_5_···F_5_C_6_H	3.092	3.181	3.059	173.1	174.2	0.0029	0.0028	0.0031	0.0168	0.0145	0.0184
(d) HC_6_F_5_···F_5_C_6_H (conf)	3.121	3.305	3.070	169.9	173.0	0.003	0.0022	0.0027	0.0178	0.0107	0.0154
(d) C_6_F_6_···F_6_C_6_	3.105	3.124	3.105	175.3	175.3	0.0028	0.0031	0.0028	0.0162	0.0167	0.0162
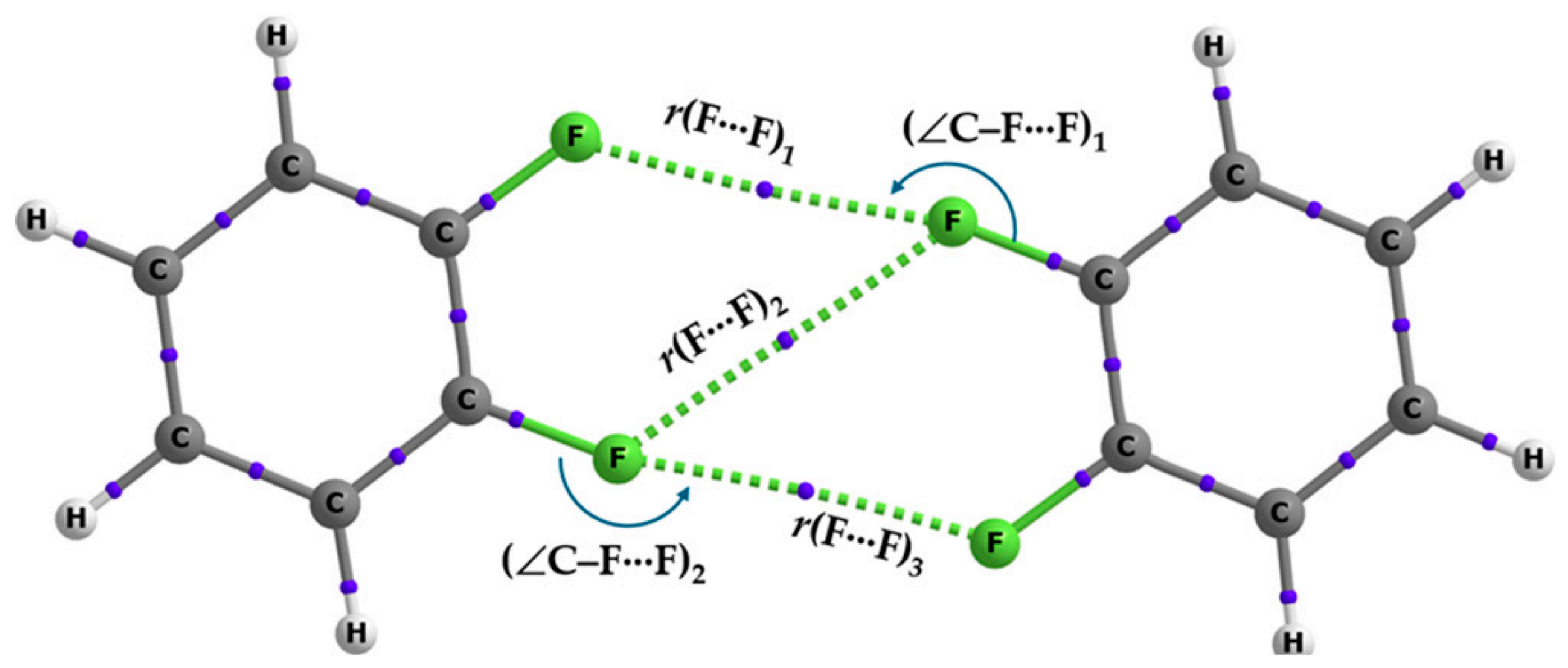											

^a^ The three sets of ρ_b_ and ∇^2^ρ_b_ values correspond to the three types of F···F distances denoted as *r*(F···F)_1–3_. ^b^ See Figure 9 for geometries.

**Table 7:** Symmetry-adapted perturbation theory (SAPT) based energy decomposition analysis for the F···F bonded dimers of some partially and fully fluorinated benzene derivatives. Electrostatic ($E_{\mathrm{elst}}$), exchange ($E_{\mathrm{exch}}$), induction ($E_{\mathrm{ind}}$), dispersion ($E_{\mathrm{disp}}$), and total SAPT2+(3) interaction energies ($E_{\mathrm{SAPT}2+(3)}$). The corresponding solution-phase binding energies ($E_{b}$) obtained from supermolecular calculations are also included. Positive values denote repulsive contributions, whereas negative values indicate stabilizing interactions.

Complex ^a^	*E_elst_*	*E_exch_*	*E_ind_*	*E_disp_*	*E_SAPT_*_2+(3)_	*E_b_*
(a) H_5_C_6_F···FC_6_H_5_	0.62	0.21	−0.07	−0.58	0.18	−0.42
(b) H_5_C_6_F···F_3_C_6_H_3_ (cis)	0.47	0.23	−0.06	−0.63	0.01	−0.49
(c) H_5_C_6_F···F_4_C_6_H_2_ (cis)	0.32	0.23	−0.06	−0.63	−0.13	−0.48
(d) H_4_C_6_F_2_···F_2_C_6_H_4_ (trans)	0.46	0.22	−0.06	−0.58	0.04	−0.45
(e) H_4_C_6_F_2_···F_2_C_6_H_4_ (cis)	0.40	0.22	−0.07	−0.62	−0.07	−0.51
(f) H_3_C_6_F_3_···F_3_C_6_H_3_ (cis)	0.50	0.22	−0.06	−0.65	0.01	−0.49
(g) H_3_C_6_F_3_···F_3_C_6_H_3_ (trans)	0.24	0.22	−0.04	−0.60	−0.17	−0.71
(h) H_2_C_6_F_4_···F_4_C_6_H_2_ (cis)	0.26	0.28	−0.05	−0.72	−0.23	−0.52
(i) C_6_F_6_···F_6_C_6_	0.03	0.28	−0.02	−0.72	−0.43	−0.73

^a^ See Figure 8 for geometries.

**Table 8:** Symmetry-adapted perturbation theory (SAPT) based energy decomposition analysis for the multiple F···F bonded complexes of some partially and fully fluorinated benzene derivatives. Electrostatic ($E_{elst}$), exchange ($E_{exch}$), induction ($E_{ind}$), dispersion ($E_{disp}$), and total SAPT2 and SAPT2+(3) interaction energies ($E_{SAPT2+}$ and $E_{SAPT2+(3)}$) are in kcal mol^−1^. The corresponding solution-phase binding energies ($E_{b}$) obtained from supermolecular calculations are also included. Positive values denote repulsive contributions, whereas negative values indicate stabilizing interactions.

Complex ^a^	*E_elst_*	*E_exch_*	*E_ind_*	*E_disp_*	*E_SAPT_*_2+_	*E _SAPT_*_2+(3)_	*E_b_*
(a) H_4_C_6_F_2_···F_2_C_6_H_4_	0.90	0.48	−0.13	−1.19	−0.11	0.06	−0.99
(b) H_3_C_6_F_3_···F_3_C_6_H_3_	0.45	0.59	−0.11	−1.29	−0.49	−0.37	−1.07
(c) H_2_C_6_F_4_···F_4_C_6_H_2_	0.27	0.72	−0.08	−1.43	−0.65	−0.52	−1.09
(d) HC_6_F_5_···F_5_C_6_H	0.07	0.78	−0.06	−1.48	−0.78	−0.69	−1.25
(d) HC_6_F_5_···F_5_C_6_H (conf)	0.08	0.65	−0.06	−1.36	−0.77	−0.69	−1.23
(d) C_6_F_6_···F_6_C_6_	−0.04	0.77	−0.04	−1.47	−0.85	−0.78	−1.23

^a^ See Figure 9 for geometries.

## Data Availability

The original contributions presented in this study are included in the article/Supplementary Materials. Further inquiries can be directed to the corresponding author.

## Supplementary Materials

The following supporting information can be downloaded at: https://www.mdpi.com/article/10.3390/ijms27146519/s1.
