# Supplementary material for: Negative σ-Holes on Fluorine in Molecules Revisited: Halogen Bonding or Counterintuitive, Anti-Electrostatic Interactions?
Source: Int J Mol Sci. 2026 Jul 22;27(14):6519. doi: 10.3390/ijms27146519 (PMC13410908; doi:10.3390/ijms27146519)
Supplement: Supplementary file 1 [file ijms-27-06519-s001.zip › Table S1 Formatted.pdf]

Table S1. Statistical distributions of C–F···X contacts extracted from the Cambridge Structural Database (CSD) including CSD ref, F···X distance, ∠C–F···X angle, and r(F···X

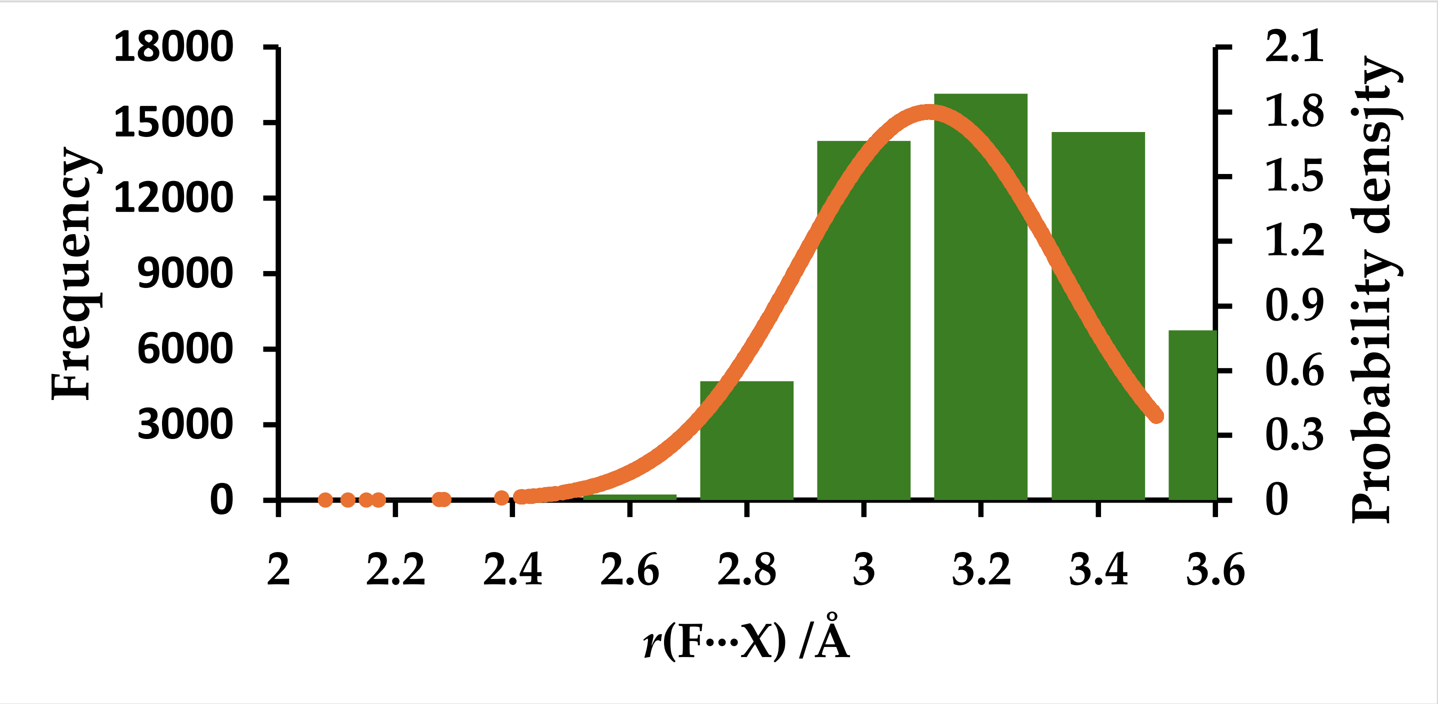

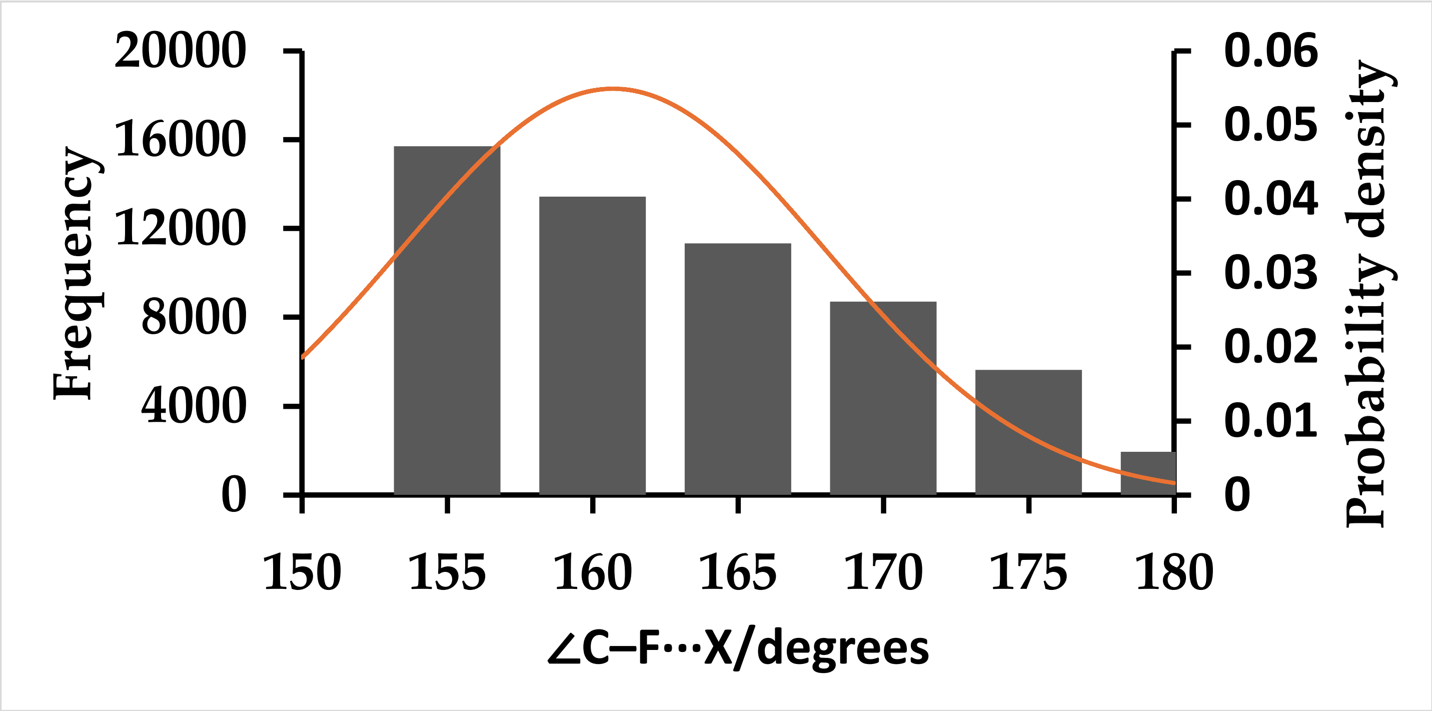

Refcode	∠C–F···X	r(F···X)	Refcode	∠C–F···X	r(F···X)	Refcode	∠C–F···X	r(F···X)	Refcode	∠C–F···X	r(F···X)	Refcode	∠C–F···X	r(F···X)
ABAQOM	155.323	3.408	ABAQOM	174.074	2.996	ABAKIY	157.593	2.883	ABANUQ	176.811	3.339	ABAPEC	176.1	3.299
ABATEC	170.947	3.477	ABATIG	150.219	2.993	ABAQUS	154.273	3.239	ABASUR	155.791	3.063	ABATEC	153.023	2.898
ABATOM	154.431	3.133	ABATOM	155.718	3.22	ABATIG	169.223	3.345	ABATIG	170.811	3.113	ABATOM	150.532	3.424
ABAZIP	160.978	3.434	ABAZUY	170.454	3.27	ABATOM	172.174	3.471	ABATOM	160.9	2.951	ABAZAH	155.164	3.4
ABENEB	160.399	2.844	ABENEB01	154.38	3.038	ABDARU	163.635	3.395	ABDARU	151.079	3.497	ABELUO	155.284	2.972
ABEVAE	157.346	3.494	ABEVAE	172.805	3.299	ABENEB01	150.924	3.297	ABESEI	164.84	3.49	ABETOQ	150.548	3.435
ABEZOA	155.152	3.37	ABEZOA	167.358	3.404	ABEYUF	156.467	2.92	ABEYUF	159.355	3.024	ABEYUF	153.108	3.399
ABEZOA	157.768	2.907	ABEZOA	155.786	2.735	ABEZOA	151.466	3.355	ABEZOA	155.399	3.472	ABEZOA	159.484	2.82
ABEZOA	178.02	2.82	ABEZOA	156.208	2.757	ABEZOA	155.576	2.79	ABEZOA	159.094	2.937	ABEZOA	168.252	3.355
ABEZOA	151.736	2.735	ABEZOA	159.33	2.777	ABEZOA	161.811	3.368	ABEZOA	170.728	2.907	ABEZOA	164.044	2.945
ABEZOA	151.366	3.306	ABEZOA	153.67	2.79	ABEZOA	156.16	2.724	ABEZOA	162.407	3.077	ABEZOA	161.221	2.877
ABEZOA	161.942	2.606	ABEZOA	155.684	2.757	ABEZOA	160.834	2.785	ABEZOA	167.265	2.999	ABEZOA	158.527	3.024
ABEZOA	170.8	2.545	ABEZOA	150.199	2.972	ABEZOA	150.016	2.949	ABEZOA	157.521	3.308	ABEZOA	151.284	3.242
ABIHOL	170.416	3.178	ABIHOL	156.142	3.481	ABEZOA	151.679	2.977	ABEZOA	159.264	2.718	ABIHOL	159.356	2.829
ABODEB	152.794	3.203	ABODEB	170.336	2.999	ABILUU	171.253	2.812	ABIRIP	155.854	3.442	ABIRIP	156.317	3.481
ABONUC	154.492	3.308	ABONUC	160.656	2.694	ABODED	172.789	3.432	ABODON	161.065	3.311	ABOLOV	162.882	3.274
ABORUH	161.678	3.485	ABOTOC	156.833	3.054	ABONUC	167.862	3.283	ABONUC	150.577	2.688	ABONUC	150.199	3.074
ABOTUK	174.242	3.14	ABOVAS	164.142	2.973	ABOTOC	161.12	2.935	ABOTOC	150.824	3.032	ABOTOC	157.84	2.82
ABUDUY	152.991	2.973	ABUDUY	168.657	2.907	ABUDIM	152.835	3.079	ABUDOS	152.778	3.358	ABUDUY	173.998	2.973
ABUDUY	162.959	2.907	ABUJIS	153.225	3.375	ABUDUY	154.026	3.058	ABUDUY	168.737	2.999	ABUDUY	152.443	2.999
ABUQAR	156.038	3.301	ABUQAR	166.962	3.009	ABUKEO	162.87	2.974	ABUKEO	155.839	2.974	ABUKOZ	160.361	3.363
ABUREW	166.979	3.256	ABUREW	162.403	3.488	ABUQAR	156.899	3.35	ABUREW	167.039	3.256	ABUREW	159.959	3.499
ABUTEX01	161.497	3.398	ABUTEX01	156.82	3.288	ABUREW	165.755	3.059	ABUREW	157.314	3.342	ABUTEX01	150.549	3.288
ABUZEF	166.716	3.026	ABUZEF	170.737	3.371	ABUTEX01	159.012	3.492	ABUTEX01	165.159	3.103	ABUTEX01	167.217	3.492
ABUZEF	168.492	3.092	ABUZEF	161.21	2.851	ABUZEF	178.106	3.353	ABUZEF	164.713	3.038	ABUZEF	152.841	3.066
ABUZEF	164.412	3.238	ABUZEF	170.526	3.148	ABUZEF	175.246	2.903	ABUZEF	171.941	3.061	ABUZEF	154.039	3.495
ABUZIJ	150.737	3.422	ABUZIJ	154.431	3.026	ABUZEF	172.152	3.353	ABUZIJ	161.185	3.118	ABUZIJ	159.1	2.817
ABUZIJ	156.677	3.026	ABUZOP	151.709	3.335	ABUZIJ	151.773	2.707	ABUZIJ	173.711	3.06	ABUZIJ	151.133	3.351
ABUZOP	173.855	3.075	ABUZOP	152.708	2.699	ABUZOP	151.463	3.407	ABUZOP	154.453	3.004	ABUZOP	157.366	3.004
ABUZUV	170.408	3.379	ABUZUV	177.692	3.373	ABUZOP	160.725	3.097	ABUZOP	158.037	2.833	ABUZUV	166.768	3.024
ABUZUV	161.576	2.847	ABUZUV	174.961	2.906	ABUZUV	164.861	3.054	ABUZUV	153.098	3.061	ABUZUV	168.846	3.109
ABUZUV	170.382	3.159	ABUZUV	171.791	3.373	ABUZUV	172.2	3.063	ABUZUV	153.979	3.497	ABUZUV	164.491	3.235

ACAJIX	168.88	3.268	ACAJOG	157.423	3.365	ACABUD	165.805	3.015	ACACEM	157.922	3.419	ACAGUJ	167.724	2.876
ACAVAB	156.986	3.138	ACAXUA	158.951	2.83	ACATIL	169.206	2.763	ACATIL	153.299	2.665	ACATIL	156.165	3.299
ACEDAN	172.877	2.863	ACEDAN	161.079	2.825	ACEDAN	155.96	2.825	ACEDAN	156.365	2.909	ACEDAN	151.211	3.175
ACEDAN0	158.251	3.235	ACEDAN0	150.341	3.029	ACEDAN0	163.328	3.215	ACEDAN0	170.748	2.966	ACEDAN0	153.142	3.195
ACEDER	151.278	3.053	ACEDER	151.111	2.85	ACEDAN0	170.348	2.712	ACEDAN0	153.734	2.83	ACEDER	154.654	3.147
ACEDIV	159.207	3.076	ACEDIV	166.665	2.938	ACEDER	151.077	3.369	ACEDIV	159.551	3.049	ACEDIV	153.758	3.132
ACEDOB	160.136	3.405	ACEDOB	167.47	3.199	ACEDIV	156.076	3.176	ACEDIV	161.423	3.069	ACEDIV	173.827	3.234
ACEDOB	151.593	2.903	ACEDOB	156.363	2.893	ACEDOB	152.451	2.884	ACEDOB	152.23	2.884	ACEDOB	167.153	3.009
ACEDOB	150.474	2.92	ACEDOB	166.091	3.027	ACEDOB	174.648	2.969	ACEDOB	150.192	2.951	ACEDOB	151.101	3.049
ACEDOB	165.287	2.906	ACEDOB	151.153	3.414	ACEDOB	160.553	3.007	ACEDOB	154.473	2.931	ACEDOB	162.857	3.206
ACEDUH	153.599	3.109	ACEDUH	162.785	3.092	ACEDUH	161.158	3.092	ACEDUH	160.406	2.927	ACEDUH	150.683	2.931
ACEFAP	170.265	3.417	ACEFET	173.166	3.285	ACEDUH	170.299	3.25	ACEFAP	159.778	2.873	ACEFAP	165.637	3.332
ACEJIC	156.426	3.294	ACELOL	154.046	3.353	ACEFET	157.062	3.268	ACEFET	160.655	2.893	ACEFET	165.469	3.265
ACERIJ	165.249	3.199	ACERIJ	165.766	2.842	ACEPEH	167.309	3.304	ACERIJ	173.494	3.061	ACERIJ	167.219	3.148
ACEXIS	159.946	2.936	ACEXIS	153.81	3.138	ACEVEN	157.712	2.88	ACEXIS	166.49	3.335	ACEXIS	168.226	3.384
ACINAF	150.804	3.403	ACISIP	164.544	3.259	ACEXIS	161.411	2.815	ACEXIS	166.967	3.115	ACEXIS	171.861	2.652
ACOHAF	150.833	3.236	ACOHEF	166.106	3.2	ACISIP	152.47	3.343	ACISIP	156.586	3.332	ACISIP	171.452	3.234
ACOKAE	157.032	3.323	ACOMAH	151.625	2.789	ACOJOT	153.753	3.077	ACOKAE	153.595	3.404	ACOKAE	163.603	3.309
ACOWEV	155.846	3.111	ACOWEV	168.798	3.217	ACOWEV	152.664	3.467	ACOWEV	158.267	3.006	ACOWEV	169.4	3.045
ACOWEV	161.134	2.831	ACOWEV	166.423	3.079	ACOWEV	165.53	3.365	ACOWEV	154.708	3.032	ACOWEV	155.126	3.128
ACUKIU	152.534	3.066	ACUKIU	165.327	3.322	ACUKIU	164.796	2.909	ACUKIU	171.505	3.102	ACUKIU	150.174	3.1
ACURAR	160.84	3.121	ACUWAW	167.431	3.046	ACUKIU	164.52	3.125	ACUNAP	169.709	3.202	ACURAR	152.337	3.442
ACUYOP	170.401	2.813	ACUYUV	154.234	3.386	ACUWAW	158.4	3.425	ACUWAW	154.649	3.106	ACUWAW	150.502	3.306
ADABIR	159.142	3.215	ADAFOD0	150.022	2.995	ACUYUV	175.252	3.297	ACUYUV	154.556	2.822	ACUZEH	154.966	3.081
ADAHEU	156.182	2.703	ADAHEU	169.762	3.386	ADAFOD0	150.523	3.392	ADAHEU	168.748	3.186	ADAHEU	174.234	2.91
ADAJAS	154.049	3.054	ADAJAS	161.01	3.16	ADAHEU	152.922	2.703	ADAJAS	163.675	3.26	ADAJAS	170.955	3.409
ADAJAS	160.458	3.228	ADAJAS	153.664	3.111	ADAJAS	163.957	3.26	ADAJAS	151.52	3.111	ADAJAS	168.687	3.409
ADAMUQ	164.857	2.883	ADAMUQ	169.396	3.35	ADAJAS	153.175	3.166	ADAJAS	168.088	3.227	ADAJAS	162.539	3.16
ADAQEB	154.275	3.351	ADAQEB	162.355	3.118	ADAQAZ	167.517	2.905	ADAQAZ	155.682	2.976	ADAQEB	153.147	3.242
ADARAY	151.887	3.225	ADARAY	150.351	3.497	ADAQEB	159.18	2.913	ADAQII	153.419	3.495	ADARAY	163.079	2.904
ADARII	164.431	3.393	ADARII	151.577	3.433	ADAREE	155.804	3.002	ADAREE	156.762	3.323	ADAREE	157.652	3.325
ADECOC	154.166	3.236	ADECOC	154.028	3.203	ADARII	158.334	3.107	ADECOC	154.138	3.32	ADECOC	171.78	3.374
ADECOC	173.742	3.024	ADEFEX	153.958	3.048	ADECOC	159.927	2.84	ADECOC	163.824	3.485	ADECOC	160.385	3.374
ADELUT	168.562	2.724	ADELUT	152.108	3.355	ADEFEX	150.933	3.363	ADEHOI	156.071	3.081	ADELII	152.029	2.859
ADEQEI	172.396	3.456	ADEQEI	165.738	2.974	ADEQEI	150.841	3.466	ADEQEI	170.552	2.801	ADEQEI	152.515	3.052
ADEYIV	171.379	3.118	ADFGLP	163.234	3.495	ADEQEI	164.269	2.77	ADEQEI	152.509	2.918	ADESOU	156.398	2.938
ADIHUS	150.714	2.996	ADIJOO	165.673	3.041	ADFGLP	179.136	3.182	ADICEX	167.713	3.338	ADIGEA	159.662	2.727
ADIXEQ	161.533	3.174	ADIXEQ	164.52	2.968	ADIQEJ	153.542	2.876	ADIWOZ	155.875	2.934	ADIWOZ	158.688	3.352
ADIXEQ	156.129	2.873	ADIXEQ	169.036	3.378	ADIXEQ	161.662	3.378	ADIXEQ	157.594	3.105	ADIXEQ	155.278	3.344
ADIXEQ	171.032	2.953	ADIXEQ	151.82	3.071	ADIXEQ	168.17	3.184	ADIXEQ	151.496	3.21	ADIXEQ	151.896	3.167
ADIXEQ	153.624	3.468	ADIXEQ	168.451	3.069	ADIXEQ	152.655	3.083	ADIXEQ	171.962	3.247	ADIXEQ	153.171	3.471
ADOHAF	152.891	3.498	ADOHAF	151.063	3.438	ADIXEQ	175.88	2.953	ADOHAF	158.385	2.962	ADOHAF	166.849	3.456
ADONAM	168.955	2.976	ADONEQ	157.255	3.379	ADOKAH	158.406	3.06	ADOLIQ	153.274	3.351	ADOLIQ	151.954	3.281
ADONOA	150.979	3.237	ADONOA	176.147	3.246	ADONEQ	164.615	3.17	ADONEQ	155.583	3.137	ADONIU	178.054	3.182
ADOSAQ	160.212	3.221	ADUJEQ	150.22	2.849	ADOPOY	155.741	3.499	ADOSAQ	156.134	2.968	ADOSAQ	169.88	3.26
ADUJIT	159.348	3.447	ADUJIU	158.944	2.912	ADUJEQ	157.105	3.249	ADUJEQ	151.995	3.466	ADUJEQ	154.487	2.849
ADUPAS	175.184	3.192	ADUPAS	156.78	2.978	ADUJUF	155.441	3.152	ADUMAP	166.748	3.425	ADUPAS	150.593	2.913
ADUPIA	161.789	2.88	ADUPIA	175.224	3.371	ADUPIA	164.73	2.867	ADUPIA	153.447	3.224	ADUPIA	157.402	3.381
ADUPIA	151.886	2.646	ADUPIA	152.101	3.361	ADUPIA	160.191	3.357	ADUPIA	151.756	2.788	ADUPIA	158.352	3.25
ADUPIA	160.363	3.104	ADUPIA	162.992	3.186	ADUPIA	151.804	3.453	ADUPIA	159.054	3.443	ADUPIA	161.818	2.796
ADUPIA	158.267	3.031	ADUPIA	163	3.078	ADUPIA	150.917	3.443	ADUPIA	152.965	3.329	ADUPIA	160.86	3.361
ADUPIA	162.692	2.822	ADUPIA	159.83	3.326	ADUPIA	171.469	2.677	ADUPIA	163.384	2.794	ADUPIA	157.681	3.388
ADUPIA	168.098	3.224	ADUPIA	164.833	2.895	ADUPIA	158.733	3.353	ADUPIA	162.996	2.888	ADUPIA	176.129	3.357
ADUPIA	151.864	3.25	ADUPIA	152.962	3.094	ADUPIA	157.2	3.365	ADUPIA	154.357	2.992	ADUPIA	151.244	2.801
ADUPIA	159.277	2.715	ADUPIA	160.674	3.381	ADUPIA	151.911	3.137	ADUPIA	173.509	2.938	ADUPIA	150.291	2.985
ADUPIA	157.882	3.14	ADUPIA	164.11	3.395	ADUPIA	170.925	3.03	ADUPIA	160.128	3.371	ADUPIA	163.066	2.791
ADUPOG	168.065	3.243	ADUPOG	156.38	2.769	ADUPIA	170.251	3.198	ADUPIA	157	3.293	ADUPIA	157.332	3.177
ADUPOG	172.367	2.76	ADUPOG	153.094	2.769	ADUPOG	157.431	3.28	ADUPOG	173.536	3.406	ADUPOG	152.323	2.859
ADUQAT	150.938	3.006	ADUQAT	166.266	3.206	ADUPOG	160.362	2.876	ADUPOG	154.144	2.876	ADUQAT	154.055	3.066
ADUXOP	152.765	3.283	AFABEQ	168.993	3.047	ADURAS	171.46	3.343	ADUTEC	151.93	2.76	ADUXOP	151.768	3.115
AFADUK	152.365	3.09	AFAFEV	159.724	2.954	AFABEQ	159.214	3.492	AFABEQ	162.262	2.896	AFACAP	175.643	2.68
AFAMUS	155.154	3.287	AFAMUS	152.676	3.343	AFAFEV	156.44	3.145	AFAFEV	151.116	2.952	AFAGEW	168.809	2.914
AFANAZ	176.121	3.2	AFANUT	156.29	2.994	AFAMUS	156.194	3.377	AFAMUS	153.916	3.486	AFANAZ	166.698	3.108
AFAQOR	155.198	3.094	AFARAE	167.682	2.872	AFANUT	172.559	3.078	AFANUT	154.309	3.144	AFAQOR	156.054	3.329
AFAZUF	171.18	3.209	AFBACO	151.31	3.071	AFASEF	164.458	3.106	AFAWUB	154.332	3.087	AFAZOZ	170.39	3.397
AFEZEU	157.785	2.928	AFEZEU	157.144	3.46	AFEPAG	156.467	3.177	AFEPAG	176.112	3.287	AFEYOC	164.533	3.497
AFIQOV	150.518	3.302	AFIQOV	150.702	2.934	AFEZIY	165.817	3.099	AFEZIY	151.03	3.443	AFIQOV	159.589	3.32
AFIXAP	151.663	2.93	AFIXAP	172.99	3.044	AFIQOV	164.026	2.936	AFIQOV	152.167	3.47	AFIQOV	165.575	3.169
AFMZRH	156.848	3.127	AFOBAZ	168.458	3.414	AFIXAP	158.407	3.166	AFMZRH	169.871	2.893	AFMZRH	151.384	3.385
AFOSAR	168.113	3.077	AFOSAR	166.464	3.01	AFOFEI	165.247	3.364	AFOGAF	157.232	3.358	AFOJEO	159.798	3.148
AFOSAR	162.834	3.142	AFOSAR	152.39	3.047	AFOSAR	158.616	3.367	AFOSAR	166.162	2.788	AFOSAR	166.698	2.99
AFOSAR	163.177	3.058	AFOSAR	172.119	2.817	AFOSAR	162.358	3.388	AFOSAR	163.028	3.293	AFOSAR	151.435	3.414
AFOSIZ	168.422	2.884	AFOSIZ	159.649	3.355	AFOSAR	167.47	2.92	AFOSEV	163.448	2.687	AFOSEV	155.686	3.264
AFOTAS	172.921	3.233	AFOTAS	156.554	2.685	AFOSOF	158.642	3.355	AFOSUL	152.212	3.329	AFOSUL	178.486	2.94
AFOTEW	163.416	2.974	AFOTEW	157.826	3.41	AFOTAS	154.875	3.022	AFOTAS	150.013	3.273	AFOTAS	154.567	2.685
AFOTEW	171.167	3.051	AFOTEW	160.225	2.996	AFOTEW	152.132	2.996	AFOTEW	160.296	2.974	AFOTEW	174.539	3.41
AFOTEW	157.532	2.865	AFOTIA	162.281	3.324	AFOTEW	152.64	3.098	AFOTEW	169.291	2.898	AFOTEW	171.89	2.898
AFOVAU	167.148	2.899	AFOVAU	165.68	3.357	AFOTIA	151.143	3.324	AFOTOG	164.207	3.348	AFOTOG	152.999	3.348
AFOVEY	160.273	3.095	AFOVEY	153.033	2.641	AFOVAU	163.117	2.84	AFOVAU	171	2.84	AFOVEY	154.994	2.981

AFOVIC	150.176	3.319	AFOZEC	151.213	3.052	AFOVIC	163.897	2.746	AFOVIC	160.228	3.319	AFOVIC	161.131	3.475
AFOZOM	171.547	3.057	AFUBIM	164.047	3.035	AFOZIG	165.827	2.755	AFOZIG	153.254	3.137	AFOZIG	156.646	2.755
AFUBIM	169.042	2.818	AFUBIM	159.531	2.989	AFUBIM	152.475	3.005	AFUBIM	155.61	3.362	AFUBIM	172.589	3.459
AFUPUN	156.759	3.39	AFUPUN	165.679	2.715	AFUBIO	164.399	3.308	AFUBIO	163.96	3.094	AFUBIO	155.876	3.378
AGATAD	153.817	2.943	AGATAD	169.503	2.976	AFUPUN	153.506	3.137	AGACAQ	170.92	3.462	AGAPOP	167.073	3.494
AGATAD	152.643	3.18	AGATAD	157.009	3.177	AGATAD	159.544	3.378	AGATAD	170.331	2.719	AGATAD	164.391	3.355
AGATAE	159.22	3.221	AGAVOU	173.805	2.726	AGATAE	155.632	3.043	AGATAE	156.672	2.822	AGATAE	150.919	3.043
AGESOY	165.204	3.413	AGEVUD	166.573	3.274	AGELEH	170.894	3.303	AGEPIN	158.142	3.258	AGESOY	153.105	3.376
AGIDEC	156.38	3.229	AGIDEC	153.907	2.952	AGEVUD	153.266	3.283	AGEVUD	157.01	3.35	AGIDEC	163.099	3.206
AGIKUW	158.337	3.043	AGIMEL	159.344	3.295	AGIDEC	164.29	3.314	AGIDEC	172.524	3.17	AGIJEJ	161.216	3.404
AGIMEL	160.073	2.688	AGIMEL	165.702	3.343	AGIMEL	156.236	3.184	AGIMEL	159.493	3.014	AGIMEL	171.1	3.223
AGINIO	167.545	3.075	AGINOU	152.856	3.445	AGIMEL	173.466	2.916	AGIMEL	163.327	2.799	AGIMEL	152.313	3.27
AGIPAK	161.497	2.789	AGIPAK	176.992	3.091	AGINUA	152.32	3.213	AGIPAK	162.932	3.339	AGIPAK	157.485	3.384
AGIPAK	154.436	2.775	AGIPAK	177.302	3.389	AGIPAK	170.652	2.932	AGIPAK	152.131	2.863	AGIPAK	160.136	3.406
AGIPAK	158.179	2.79	AGIPAK	159.385	2.921	AGIPAK	168.354	3.437	AGIPAK	172.961	3.325	AGIPAK	151.487	2.854
AGIPEO	167.779	3.338	AGIPEO	160.495	2.844	AGIPAK	156.256	3.251	AGIPAK	163.087	3.244	AGIPAK	159.033	3.387
AGIPEO	175.009	3.102	AGIPEO	167.611	3.155	AGIPEO	155.289	3.142	AGIPEO	171.952	3.373	AGIPEO	167.478	3.077
AGIPEO	156.956	2.804	AGIPEO	160.515	3.137	AGIPEO	155.838	3.241	AGIPEO	161.611	3.098	AGIPEO	153.882	3.375
AGIQAL	170.548	3.158	AGIQAL	173.937	2.844	AGIPEO	172.67	3.244	AGIPEO	154.479	3.381	AGIPEO	162.413	3.239
AGIQAL	157.306	2.972	AGIQAL	173.516	3.406	AGIQAL	159.987	2.913	AGIQAL	159.065	2.972	AGIQAL	150.236	3.076
AGIQAL	153.816	3.079	AGIQAL	157.578	2.744	AGIQAL	166.333	2.896	AGIQAL	162.969	3.16	AGIQAL	152.143	2.911
AGIQAL	152.883	3.181	AGIQAL	160.109	3.416	AGIQAL	160.434	2.991	AGIQAL	165.733	2.948	AGIQAL	168.78	2.86
AGIRUG	156.078	2.916	AGISUF	164.699	3.376	AGIQAL	163.276	3.144	AGIQAL	168.636	2.864	AGIQOY	151.891	2.779
AGITOC	151.49	2.871	AGOBEH	163.084	3.416	AGITOC	150.439	2.874	AGITOC	171.801	2.895	AGITOC	153.332	2.895
AGOBIL	168.127	2.952	AGOBIL	152.877	3.183	AGOBEH	158.286	3.422	AGOBEH	165.861	3.366	AGOBEH	154.331	2.928
AGOBUT	156.66	3.342	AGOBUT	161.655	3.061	AGOBIL	170.698	3.053	AGOBIL	153.963	3.114	AGOBIL	155.962	3.496
AGOBUT	165.072	2.946	AGOBUT	168.507	3.462	AGOBUT	150.256	3.308	AGOBUT	155.212	3.267	AGOBUT	156.543	3.325
AGOCAA	154.424	2.922	AGOCAA	163.112	3.349	AGOBUX	150.689	3.227	AGOCAA	151.51	3.485	AGOCAA	164.776	3.049
AGOCEE	166.57	2.918	AGOCEE	168.829	3.315	AGOCAA	168.556	3.001	AGOCAA	168.36	3.025	AGOCAA	165.853	3.383
AGOCEE	152.356	3.39	AGOCEE	158.486	3.305	AGOCEE	162.7	3.019	AGOCEE	165.218	3.292	AGOCEE	155.766	3.369
AGOCEI	159.64	3.082	AGOCEI	158.486	3.143	AGOCEE	155.086	3.301	AGOCEE	168.213	3.369	AGOCEI	154.037	3.025
AGOCEI	173.404	3.008	AGOCEI	173.57	3.008	AGOCEI	159.67	3.076	AGOCEI	158.499	3.137	AGOCEI	154.012	3.032
AGOCIK	155.859	3.39	AGOCIK	159.575	3.226	AGOCEI	158.934	3.106	AGOCEI	159.014	3.114	AGOCIK	162.848	3.08
AGOYAW	169.484	3.097	AGTFAC0	152.447	2.954	AGOCOQ	164.532	3.342	AGOMAK	155.444	3.237	AGOWEB	154.281	3.494
AGUFIS	159.399	3.131	AGUFIS	152.828	3.3	AGTFAC0	151.463	3.035	AGTFAC0	153.331	2.986	AGUFIS	157.449	2.869
AGUKIZ	157.45	3.41	AGUQEB	153.139	3.376	AGUHOD	160.318	3.185	AGUHOD	161.031	2.895	AGUKIZ	152.729	3.228
AGUREC0	164.633	2.923	AGUREC0	160.319	2.925	AGUQEB	154.982	3.489	AGUQIF	151.336	3.432	AGUREC	160.684	2.959
AGUXUW	156.559	2.81	AGUXUW	155.138	2.864	AGUREC0	161.91	2.933	AGUXUW	162.761	2.956	AGUXUW	153.185	3.075
AHAFAT	157.494	3.302	AHAFIB	150.864	3.387	AGUXUW	166.441	2.81	AHADUL0	160.709	2.955	AHAFAT	170.004	3.076
AHAGIA	164.904	3.293	AHAGOG	172.585	3.083	AHAFIB	155.902	3.197	AHAFIB01	156.094	3.182	AHAFIB01	150.897	3.368
AHATUZ	152.095	3.357	AHAXOW	154.28	3.432	AHAGOG	162.442	2.971	AHAGOG	158.045	3.106	AHAMAX	161.835	3.153
AHECOH	153.274	3.406	AHECOI	152.288	3.411	AHAXOW	155.61	3.19	AHEBOH	172.392	3.489	AHECEY	155.202	3.045
AHEVES	175.595	3.029	AHEVES	172.39	2.804	AHECUO	163.336	2.966	AHEDUN	151.504	2.825	AHEDUN	150.496	2.83
AHEYES	154.803	3.232	AHEYUI	150.997	2.888	AHEVES	171.553	3.029	AHEVES	172.081	3.174	AHEYES	159.66	3.027
AHIDUQ	155.588	3.227	AHIKAH	155.896	3.236	AHEYUI	156.123	2.736	AHIDOM	164.621	3.257	AHIDUQ	150.632	3.393
AHIKAH	153.773	3.077	AHIKAH	165.313	3.152	AHIKAH	164.257	3.231	AHIKAH	171.556	3.024	AHIKAH	151.217	2.753
AHINOV	163.492	3.068	AHINOV	159.835	3.114	AHIKAH	155.579	3.305	AHIKAH	171.584	3.338	AHIMUD	152.144	3.368
AHIPEN	154.362	3.397	AHIVOE02	167.848	3.376	AHINOV	155.481	3.119	AHIPEN	162.425	3.257	AHIPEN	150.721	3.397
AHIWOE	155.843	3.068	AHIWOE	160.059	3.343	AHIWOE	157.68	2.808	AHIWOE	157.982	3.155	AHIWOE	174.292	3.483
AHIXAS	157.795	2.909	AHIXAS	160.809	2.92	AHIWUL	172.008	2.835	AHIWUL	162.784	3.006	AHIWUL	171.985	2.778
AHOCIL	167.249	3.06	AHOMAN	171.873	2.635	AHIXAS	160.378	3.197	AHIXAS	164.245	2.753	AHOCIL	159.301	2.702
AHUJUK	156.482	2.893	AHULOE	167.16	3.367	AHOWAW	155.641	2.789	AHUJUK	154.686	3.067	AHUJUK	152.714	2.893
AHURUQ	158.571	3.208	AHUXIM	152.969	3.191	AHULOG	169.303	3.495	AHUQUR	165.041	3.286	AHURIG	165.964	3.39
AJAHUQ	152.711	2.835	AJAHUQ	150.853	2.865	AHUYUB	158.244	3.353	AHUZOW	157.858	3.323	AHUZOW	163.216	3.278
AJAHUQ	165.088	2.973	AJAHUQ	151.93	3.333	AJAHUQ	164.619	3.243	AJAHUQ	154.774	3.076	AJAHUQ	150.92	2.895
AJAHUQ0	150.774	2.865	AJAHUQ0	164.552	3.243	AJAHUQ	160.331	2.814	AJAHUQ	158.063	2.915	AJAHUQ0	152.747	2.832
AJAHUQ0	151.983	3.332	AJAHUQ0	160.298	2.811	AJAHUQ0	155.091	3.072	AJAHUQ0	151.118	2.891	AJAHUQ0	165.191	2.973
AJAKEE	152.658	2.854	AJAKEE	153.951	3.289	AJAHUQ0	158.218	2.909	AJAJOM	150.892	3.268	AJAJOM	163.149	2.96
AJANAD	151.226	3.141	AJAPIM	160.937	3.417	AJAMIK	157.739	3.272	AJAMIK	165.878	2.96	AJAMIK	154.031	2.994
AJAQIL	169.582	3.102	AJAQIL	151.349	3.038	AJAPIM	162.111	3.222	AJAQIL	158.565	2.877	AJAQIL	153.933	2.78
AJAQOR	155.53	2.717	AJAQOR	162.888	3.38	AJAQIL	152.136	2.825	AJAQOR	155.792	2.717	AJAQOR	160.346	3.272
AJAZAM	161.732	3.327	AJEFAX	164.996	2.764	AJAQOR	168.217	3.398	AJAWEP	151.265	3.2	AJAWEP	164.186	3.2
AJEJOP	151.837	3.049	AJEMUB	169.076	3.2	AJEHAA	158.781	3.274	AJEHIJ	166.224	3.185	AJEJEG	164.39	2.983
AJENEJ	159.491	3.013	AJENIN	166.818	2.875	AJENAF	172.321	3.003	AJENEJ	167.124	2.892	AJENEJ	163.494	2.993
AJERAK	156.982	3.215	AJEVAQ	150.86	2.914	AJENOT	157.231	3.016	AJERAK	164.593	3.324	AJERAK	151.864	3.323
AJICOL	160.2	3.446	AJICOL	156.082	3.163	AJEVEU	151.328	3.203	AJICIF	161.879	3.443	AJICIF	160.159	2.901
AJIGEI	165.112	3.143	AJIGEI	164.635	2.838	AJICOL	170.948	3.07	AJIDII	156.658	3.223	AJIFIK	158.658	2.952
AJIGIM	151.238	2.96	AJIGUV	164.931	3.064	AJIGEI	176.749	3.229	AJIGEI	157.402	2.912	AJIGIM	157.73	3.187
AJIJOV	157.203	3.175	AJIJOV	153.378	3.182	AJIJOV	164.109	3.093	AJIJOV	151.486	3.423	AJIJOV	161.211	3.286
AJIJOV	165.817	3.084	AJIJOV	158.853	2.705	AJIJOV	152.365	2.985	AJIJOV	157.8	2.93	AJIJOV	160.348	3.177
AJIJOV	162.378	3.078	AJIJOV	158.556	3.157	AJIJOV	155.574	2.825	AJIJOV	159	3.071	AJIJOV	166.684	2.921
AJIJUB	164.218	3.003	AJIJUB	153.919	3.281	AJIJOV	159.692	2.705	AJIJUB	170.813	3.109	AJIJUB	165.889	2.969
AJIRUI	171.687	2.866	AJIRUI	164.843	2.65	AJIJUB	155.774	3.154	AJIMOV	155.976	2.874	AJIMOV	153.53	2.874
AJISUI02	154.316	2.885	AJIVIB	153.66	3.194	AJIRUI	171.37	3.387	AJISIY	165.664	2.428	AJISIY01	165.636	2.434
AJIVOH	151.982	3.008	AJIVOH	158.558	3.241	AJIVOH	168.668	3.29	AJIVOH	152.749	3.046	AJIVOH	162.176	3.397
AJIVOH	157.083	3.411	AJIVOH	166.335	2.922	AJIVOH	155.978	3.093	AJIVOH	164.38	2.889	AJIVOH	154.179	3.418
AJOGUB	160.512	3.219	AJOGUB	152.278	2.915	AJIXID	150.519	3.467	AJOFEN	155.452	3.293	AJOGUB	156.316	3.389
AJUDER	160.546	3.006	AJUFAQ	156.631	2.934	AJOGUE0	169.57	3.381	AJOSEZ	168.208	3.168	AJUDER	151.929	3.106
AJUMOI	170.89	3.456	AJUMOL	169.406	3.005	AJUGUL	165.538	3.116	AJUGUL	151.761	2.71	AJUMIC	158.164	3.005

AJUMOL	158.474	2.783	AJUMUR	167.29	2.699	AJUMOL	157.443	3.465	AJUMOL	166.285	2.783	AJUMOL	152.993	3.201
AJUZIP	158.189	3.267	AJUZIP	155.242	2.662	AJUNAY	155.612	3.477	AJUNAY	166.436	2.682	AJUZIP	170.259	2.903
AKAQOS	155.28	3.357	AKAQOS	165.554	3.219	AJUZIP	173.209	3.267	AJUZIP	158.928	2.889	AKAMAA	155.581	3.12
AKATUC	151.247	3.166	AKATUC	161.278	3.257	AKASIR	152.866	2.946	AKASOX	167.824	2.991	AKATIQ	156.709	3.204
AKECAU	158.927	2.992	AKEFAY	160.846	3.16	AKAVOB	153.169	3.31	AKECAU	174.967	3.421	AKECAU	150.633	2.665
AKEREP	154.131	3.415	AKEROZ	163.89	3.266	AKEFAY	169.977	3.16	AKEREP	174.745	3.305	AKEREP	156.336	3.355
AKIDOQ	156.474	2.96	AKIDOQ	168.403	3.031	AKEROZ	157.624	3.253	AKIDEG	153.764	3.463	AKIDEG	161.141	2.878
AKIMAL	154.208	2.993	AKIMAL	164.993	2.861	AKIHUA	160.154	2.936	AKIHUA	155.473	2.854	AKIMAL	163.073	2.879
AKIWIA	166.445	2.733	AKIWUO	155.998	3.032	AKIMOZ	171.169	2.883	AKIMOZ	155.931	3.012	AKIQUK	177.441	3.351
AKOHIT	156.905	3.12	AKOHIT	151.678	2.777	AKIXAV	154.459	2.975	AKOGOA	171.596	3.255	AKOHIT	169.215	3.353
AKOWAY	161.762	3.094	AKOWAY	164.436	3.389	AKOHIT	163.197	3.232	AKOTIG	162.197	2.975	AKOTOM	157.371	3.457
AKOWII	156.677	2.772	AKOWII	153.719	3.01	AKOWAY	155.888	3.06	AKOWAY	158.782	3.269	AKOWAY	158.341	3.291
AKOXUT0	165.27	3.484	AKOYAA	163.815	3.213	AKOWII	156.955	3.175	AKOWII	174.594	3.182	AKOWII	153.347	3.261
AKUGIW	156.504	3.416	AKUMUR	152.13	3.477	AKUBOA	150.785	3.04	AKUBOA	165.161	3.213	AKUDIV	167.709	3.01
AKUSOS	173.778	2.906	AKUSOS	162.682	3.274	AKUNOK	166.656	2.803	AKUSOS	164.796	3.224	AKUSOS	151.062	3.465
AKUTAD	156.529	2.803	AKUTAD	155.035	2.919	AKUSOS	165.846	3.274	AKUSUW	152.553	3.143	AKUTAD	160.587	2.919
AKUVEL	152.696	2.913	AKUVIP	167.778	3.066	AKUTUZ	166.095	3.051	AKUTUZ	160.248	3.132	AKUVEI	157.405	3.388
AKUXEN	173.634	3.102	AKUXEN	169.668	3.393	AKUVIP	162.551	3.022	AKUXEN	162.92	3.379	AKUXEN	170.779	3.344
AKUYIS	171.301	3.228	AKUYIS	169.064	3.387	AKUYIS	176.116	3.144	AKUYIS	165.224	2.949	AKUYIS	155.218	2.958
ALAMEF	162.107	3.374	ALAMEF	158.876	3.213	AKUYIS	163.63	3.034	AKUYIS	161.814	3.071	ALAMEF	168.124	2.959
ALAMEF	168.683	2.966	ALAMOP	158.5	3.104	ALAMEF	156.943	3.157	ALAMEF	153.367	3.483	ALAMEF	175.664	3.145
ALAYIW	168.718	3.321	ALEPAJ	150.551	3.266	ALATEN	158.688	3.264	ALATEN	157.718	3.232	ALAYIW	155.431	2.95
ALESUI	172.543	3.023	ALESUI	152.965	3.3	ALEPOA	162.083	3.409	ALEPOA	165.624	3.332	ALEPUF	151.091	3.047
ALEZEZ	162.418	2.843	ALEZEZ	153.871	3.016	ALEWUJ	150.1	3.466	ALEXAQ	158.259	3.305	ALEXOI	161.662	3.376
ALIGUZ	152.068	3.387	ALINIS	152.612	2.9	ALEZID	153.573	3.087	ALEZID	163.817	2.877	ALEZID	160.95	2.953
ALINIS	154.305	2.944	ALITOH	160.216	3.371	ALINIS	156.775	2.763	ALINIS	164.182	3.332	ALINIS	178.044	3.109
ALOBUB	152.757	3.031	ALOBUB	150.423	3.031	ALIYOK	152.437	3.452	ALIZAX	165.648	2.709	ALIZAX	169.844	3.13
ALOKES	161.913	3.324	ALOMOD	173.105	3.033	ALOHIW	152.455	3.42	ALOJER	160.269	3.414	ALOKES	156.131	2.905
ALUVEJ	150.707	2.951	ALUVEJ	170.775	3.333	ALUVEJ	157.02	3.094	ALUVEJ	152.697	2.892	ALUVEJ	169.957	2.943
ALUYEN	155.394	3.183	ALUYEN	157.766	3.5	ALUVEJ	154.716	2.968	ALUVEJ	150.29	3.241	ALUYEN	151.633	2.95
ALUYEN	156.333	3.241	ALUYEN	166.146	3.393	ALUYEN	155.601	3.201	ALUYEN	153.351	2.979	ALUYEN	167.067	2.895
ALUYEN	152.559	3.127	ALUYEN	162.367	2.797	ALUYEN	163.479	3.241	ALUYEN	164.418	3.393	ALUYEN	152.046	3.337
ALUYEN	165.353	2.92	ALUYIR	167.322	2.761	ALUYEN	152.64	2.918	ALUYEN	154.217	3.047	ALUYEN	156.115	3.26
ALUYIR	162.249	3.068	ALUYIR	158.808	3.075	ALUYIR	159.563	2.761	ALUYIR	155.066	3.304	ALUYIR	154.059	3.328
ALUYOX	156.973	3.171	ALUYOX	155.565	3.069	ALUYIR	152.345	3.078	ALUYIR	162.249	2.914	ALUYIR	162.366	3.075
AMABOG	153.362	3.471	AMADEZ	150.058	3.38	ALUYOX	164.26	3.131	ALUYOX	165.263	3.141	ALUYOX	162.324	2.93
AMAPIQ	152.628	2.708	AMAPIQ	151.847	2.708	AMAJUT	150.139	3.005	AMAJUT	150.221	3.487	AMAKAA	150.004	3.052
AMAYIZ	159.793	2.831	AMAYOF	165.498	3.221	AMARUB	166.327	3.145	AMATUD	156.478	3.338	AMATUD0	155.415	3.159
AMDFAC	172.025	3.169	AMEBUS	153.849	3.143	AMAYOF	157.822	3.308	AMAZAS	153.188	3.053	AMDFAC	164.555	3.037
AMEGOR	171.473	2.889	AMEGOR	164.933	2.946	AMECIE	155.761	3.468	AMEGOR	155.729	3.319	AMEGOR	169.058	2.817
AMEKAG0	165.591	2.828	AMEKAG0	171.696	2.832	AMEGOR	158.58	3.075	AMEKAG0	167.232	2.948	AMEKAG0	163.054	3.01
AMEKAG0	168.518	2.932	AMENIT	153.216	3.269	AMEKAG0	166.974	2.831	AMEKAG0	171.256	2.841	AMEKAG0	170.291	2.882
AMEPAM	172.83	2.888	AMEPAM	163.894	3.086	AMENIT	178.956	3.144	AMENOZ	152.737	3.465	AMEPAM	159.782	3.197
AMEPAM	167.798	3.035	AMEPAM	167.919	3.168	AMEPAM	155.156	3.058	AMEPAM	158.371	3.227	AMEPAM	171.663	2.976
AMEPAM	163.683	3.308	AMEPAM	173.683	3.362	AMEPAM	153.068	3.384	AMEPAM	176.822	2.786	AMEPAM	156.34	2.86
AMEPAM	156.47	2.802	AMEPAM	156.341	3.33	AMEPAM	157.858	3.298	AMEPAM	151.186	2.855	AMEPAM	154.694	3.025
AMEZIC	165.775	3.441	AMIBAA	166.017	3.417	AMEPAM	157.528	3.206	AMERAM	163.763	2.971	AMEZIC	154.304	2.977
AMIBEE	173.744	2.985	AMIBEE	152.602	3.226	AMIBAA	173.247	3.004	AMIBEE	167.75	3.453	AMIBEE	162.893	2.791
AMIBOO	167.366	3.025	AMIBOO	170.929	3.138	AMIBII	173.467	3.091	AMIBII	152.719	3.166	AMIBOO	169.4	3.021
AMIGOU	155.515	3.389	AMIXAX	171.313	3.064	AMIBOO	150.295	3.168	AMICAD	165.393	3.212	AMIGIO	151.268	3.326
AMOCEL	160.667	2.961	AMOCEL	156.789	3.033	AMIXEB	157.848	2.788	AMIXEB	176.239	3.476	AMIXEB	161.999	2.788
AMOCUA	157.461	3.369	AMOCUA	159.021	3.195	AMOCOV	174.077	3.251	AMOCUA	153.447	2.921	AMOCUA	159.915	2.861
AMTFAC0	169.196	3.157	AMTFAC0	154.788	3.299	AMOPOJ	168.692	3.013	AMORIE	152.872	3.042	AMTFAC0	164.19	3.045
AMUPOQ0	150.207	2.92	AMUQUV	159.33	3.408	AMTFAC0	163.921	3.045	AMTFAC0	168.956	3.171	AMTFAC0	154.997	3.298
ANABUO	175.42	3.369	ANAMOS	161.702	2.945	AMUQUV	171.359	3.047	AMUQUV	168.311	3.084	AMUREI	150.132	2.9
ANAZOH	160.194	3.344	ANAZOH	163.585	3.35	ANAMOS	163.011	2.965	ANAZOH	171.325	2.985	ANAZOH	157.934	2.931
ANEQOD	151.537	2.825	ANEQOD	169.033	2.889	ANAZOH	166.634	3.333	ANAZOH	150.583	3.032	ANEQOD	178.758	3.094
ANINOE	159.258	3.095	ANINOE	164.733	3.143	ANEXAT	154.094	3.437	ANEXEZ	158.35	2.969	ANINOE	150.382	3.185
ANIPAS	160.629	2.868	ANIYAA	162.115	3.162	ANINOE	172.327	3.329	ANIPAS	150.399	3.13	ANIPAS	164.842	2.796
ANIYAA	167.657	3.017	ANIYAA	175.824	2.934	ANIYAA	154.192	2.957	ANIYAA	161.549	3.328	ANIYAA	152.356	3.481
ANIYAA	165.023	2.896	ANIYAA	156.911	3.328	ANIYAA	160.429	3.372	ANIYAA	153.981	2.796	ANIYAA	151.386	3.162
ANIYEE	167.735	3.051	ANIYEE	166.005	3.496	ANIYAA	163.846	3.189	ANIYEE	156.874	2.897	ANIYEE	160.894	3.444
ANIYEE	163.543	2.889	ANIZEF	165.879	3.107	ANIYEE	164.304	2.897	ANIYEE	169.006	3.458	ANIYEE	165.429	3.392
ANIZEF	160.93	2.665	ANIZEF	166.559	3.016	ANIZEF	163.643	2.946	ANIZEF	170.455	3.001	ANIZEF	169.616	2.991
ANIZEF	170.698	2.923	ANIZEF	166.802	3.071	ANIZEF	156.544	3.343	ANIZEF	150.569	3.5	ANIZEF	157.734	3.141
ANOCEO	160.579	2.777	ANOCEO	173.923	2.886	ANIZEF	171.231	2.935	ANIZEF	157.317	2.946	ANOCEO	158.403	3.405
ANOCEO	150.697	3.399	ANOPOK	170.519	3.157	ANOCEO	151.384	2.723	ANOCEO	163.305	2.734	ANOCEO	164.067	3.07
ANUFOH	179.689	3.13	ANUFOH	158.843	3.08	ANUFOH	163.677	2.923	ANUFOH	169.023	2.901	ANUFOH	172.744	3.13
ANUHUP	166.706	2.985	ANUPAD	168.948	3.446	ANUFOH	158.845	2.901	ANUFOH	172.292	3.08	ANUHUP	157.746	3.019
ANURAE	154.286	2.991	APASUG	157.175	3.3	ANUPAD	160.261	3.047	ANUPIJ	168.367	2.85	ANUPIJ	170.522	3.481
APATOB0	163.392	3.26	APAXEX	150.609	3.351	APATER	152.203	2.8	APATER	152.941	2.8	APATOB	153.09	3.486
APEBIH	153.473	3.322	APEBON	150.018	2.855	APAXEX	154.453	3.439	APEBIH	157.074	3.412	APEBIH	167.591	3.341
APECII	155.482	2.997	APECOO	159.373	3.485	APECAA	161.877	3.449	APECAA	159.209	3.088	APECII	164.862	2.963
APEJUC	164.353	3.475	APEJUC	150.065	3.43	APECUU	171.252	2.94	APECUU	155.813	2.897	APEDEF	173.784	3.391
APENEQ	161.966	2.976	APENEQ	153.981	2.839	APEJUC	153.978	3.158	APEMOZ	166.148	3.343	APEMOZ	161.8	3.195
APEQEU	151.312	3.088	APEQEU	164.042	2.696	APENEQ	164.961	2.848	APENEQ	157.588	3.34	APEQEU	170.472	3.242
APIHAJ	168.305	3.346	APIHAJ	164.863	2.789	APEQEU	175.08	2.696	APEQOC	156.218	3.124	APETOG	163.999	3.349
APINUK	160.012	3.384	APIPUM	164.155	3.25	APIHAJ	164.412	3.33	APILES	156.784	2.884	APILES	152.451	3.102
APIQAT	161.345	3.447	APIQAT	164.619	3.302	APIPUM	174.894	3.491	APIPUM	167.552	3.056	APIPUM	151.21	3.276

APIQEX	163.649	2.582	APIQEX	161.656	3.144	APIQAT	167.103	3.443	APIQAT	160.48	2.561	APIQEX	160.712	3.098
APIREY	166.088	3.083	APIREY	153.649	3.302	APIQEX	168.921	3.229	APIRAU	158.954	2.952	APIRAU	157.349	3.051
APIWED	153.848	3.333	APIWED	155.767	3.482	APITOJ	152.888	3.25	APITOJ	164.245	3.319	APIWED	155.135	3.175
APIWEE	168.898	3.154	APIWEE	170.235	3.296	APIWED	154.462	3.072	APIWEE	151.621	3.091	APIWEE	156.849	2.859
APIWIH	151.044	3.19	APIXAB	163.659	2.972	APIWEE	152.406	3.344	APIWEE	159.953	3.344	APIWEE	158.099	2.99
APIZIK	160.217	3.426	APIZIK	177.644	3.064	APIZEG	157.655	3.164	APIZIK	173.77	3.09	APIZIK	160.334	3.155
APOSEG	165.349	2.891	APOSEG	165.375	3.12	APIZIK	155.871	3.255	APIZIK	168.043	2.615	APOSEG	151.634	2.799
APOSEG	163.379	3.221	APOSEG	169.653	2.952	APOSEG	159.723	2.799	APOSEG	162.431	3.229	APOSEG	154.197	2.997
APUGIC	159.752	3.144	APUKII	158.093	3.255	APOSEG	167.46	3.028	APUBOF	165.085	2.935	APUGIC	152.644	3.144
APUTUC	151.509	3.131	APUTUC	150.25	3.105	APUKII	162.76	2.935	APUMEF	167.492	2.978	APUTUB	159.282	3.034
APUVEN	155.714	3.499	APUVEN	159.788	3.065	APUTUC	158.737	3.326	APUTUC	155.211	3.044	APUTUC	152.725	3.105
AQABEB	160.301	3.018	AQABOK	175.181	3.409	APUVEN	150.7	2.786	APUVEN	154.082	2.65	APUVEN	160.054	3.438
AQAKEK	170.89	2.834	AQAKEK	158.712	2.91	AQABOK	152.803	3.409	AQABOK	171.627	3.136	AQABUQ	170.424	3.223
AQATUI	152.157	3.078	AQATUI	164.541	3.161	AQALEL	156.883	2.8	AQALEL	164.563	2.494	AQATAO	155.126	3.07
AQAZEX	169.077	3.203	AQAZEY	158.717	3.299	AQAYOG	160.953	2.803	AQAYUM	155.897	2.937	AQAYUM	170.112	3.282
AQEPIV	167.227	3.466	AQEPOB	155.09	3.47	AQAZIC	150.127	2.679	AQAZIC	160.552	2.679	AQEPIV	160.182	3.369
AQEQEV	154.538	2.62	AQETAR	168.434	3.426	AQEPOB	169.572	3.384	AQEPUH	171.038	3.378	AQEPUH	173.27	3.336
AQEZUS	161.206	3.086	AQFSZN	164.416	3.187	AQETAR	151.679	2.986	AQETAR	165.443	2.986	AQETIC	159.242	3.432
AQITOJ	170.993	2.913	AQITOJ	153.609	2.779	AQFSZN	173.784	3.355	AQILUH	157.963	3.247	AQILUH	158.036	3.455
AQIXUT	156.103	2.904	AQIXUT	169.93	3.15	AQITOJ	157.893	3.48	AQITOJ	164.987	2.913	AQITUP	164.6	3.095
AQIXUT	160.364	3.193	AQIXUT	161.293	3.451	AQIXUT	166.382	2.767	AQIXUT	153.31	3.217	AQIXUT	156.324	3.104
AQOMEZ	168.596	3.224	AQONEZ	171.98	3.472	AQIXUT	159.999	2.874	AQIXUT	155.675	3.391	AQOJOI	153.977	3.495
AQOXOV	172.244	3.059	AQOXOV	165.357	3.131	AQOSIL	156.724	3.182	AQOSIL	154.476	3.052	AQOXOV	169.696	2.937
AQUJEE	157.773	3.453	AQUJEE	158.628	2.858	AQOXOV	153.732	3.082	AQOYOW	163.65	3.294	AQOZUB0	172.208	3.413
AQUTEN0	166.783	3.279	AQUTEN0	158.681	3.372	AQUJIF	155.49	3.153	AQUJIF	150.79	3.269	AQUTEN0	165.348	3.288
AQUTEN0	155.459	3.104	AQUTEN0	158.351	2.898	AQUTEN0	174.893	2.746	AQUTEN0	155.029	3.395	AQUTEN0	159.213	2.898
AQUTOX0	161.944	3.144	AQUTOX0	162.453	2.67	AQUTEN0	150.479	2.768	AQUTEN0	153.774	3.244	AQUTOX0	152.13	2.689
ARAQIU	170.076	3.437	ARAVIY	152.448	3.16	AQUVAL0	167.669	2.859	ARAFAD	150.985	3.245	ARAFAD	161.225	3.231
ARAVIY	151.898	2.854	ARAVIY	167.09	3.443	ARAVIY	150.336	3.016	ARAVIY	159.203	2.912	ARAVIY	163.867	3.281
ARAVIY	165.218	3.344	ARAYOI	177.789	3.171	ARAVIY	154.016	3.293	ARAVIY	164.572	3.3	ARAVIY	154.504	3.171
AREPUI	173.565	3.324	ARIBOV	161.306	3.2	AREFIP	155.353	3.068	AREFIP	156.911	3.469	AREPUI	164.28	3.243
ARINIZ	152.837	2.986	ARINIZ	169.368	2.971	ARIBOV	152.041	3.175	ARIBOV	167.499	3.357	ARIBOV	163.916	3.11
AROBAN	177.85	3.062	AROBAN	166.211	3.024	ARINIZ	165.694	3.428	ARIZUZ	168.461	3.446	ARIZUZ	163.716	3.274
ARODIW	164.733	3.204	AROJEW	168.988	3.171	AROBUF	172.954	3.446	AROBUF	167.733	3.052	AROBUF	170.52	3.049
AROJEW	155.431	3.01	AROJEW	168.674	3.346	AROJEW	161.285	3.158	AROJEW	150.826	3.494	AROJEW	157.976	3.494
AROKUO	152.198	2.897	AROKUO	167.092	3.28	AROJIA	158.139	3.09	AROJIA	154.554	3.243	AROJIA	158.251	2.773
AROMOJ	153.605	3.17	AROMOJ	155.994	3.331	AROKUO	152.969	3.324	AROMOJ	168.682	3.219	AROMOJ	158.856	3.312
AROMOJ	160.875	3.419	AROMOJ	151.794	3.414	AROMOJ	150.469	3.266	AROMOJ	156.991	3.273	AROMOJ	154.566	2.732
AROWOV	161.381	3.337	AROYIS	151.828	3.196	AROMOJ	165.737	3.419	AROMOJ	150.301	3.438	AROPEF	152.4	3.452
ARUHUT	161.084	3.156	ARUJOO	167.3	3.491	ARUGUR	177.903	3.496	ARUGUR	159.22	3.062	ARUGUR	155.12	3.293
ARUJOO	162.99	2.97	ARUKAB	158.339	3.257	ARUJOO	172.398	3.309	ARUJOO	166.177	3.491	ARUJOO	159.704	3.087
ARUKEF	161.358	3.044	ARUKEF	175.186	3.132	ARUKAB	157.119	3.3	ARUKEF	177.324	2.899	ARUKEF	168.169	3.135
ARUREM	159.521	3.163	ARUREM	152.767	3.295	ARUKEF	166.213	2.942	ARUKEF	158.34	3.412	ARUKEF	170.836	2.91
ARURIQ	159.795	2.935	ARURIQ	167.102	3.129	ARUREM	156.453	2.998	ARUREM	164.406	3.191	ARUREM	153.316	2.998
ARUTOY	175.867	2.924	ARUTOY	159.665	3.422	ARUROV	151.089	2.641	ARUROV	160.674	2.924	ARUTEO	158.887	3.432
ARUXES	158.871	3.181	ARUXES	161.782	3.171	ARUXES	161.408	3.259	ARUXES	175.679	2.837	ARUXES	173.225	2.943
ASABED	157.409	2.999	ASABED	170.986	3.039	ARUXES	177.469	2.854	ASABED	154.944	2.903	ASABED	154.527	3.084
ASABED	159.038	3.349	ASABED	151.097	3.106	ASABED	173.281	2.878	ASABED	172.615	2.894	ASABED	160.35	3.153
ASABIH	153.072	3.306	ASABIH	153.085	3.298	ASABED	158.357	3.289	ASABED	163.803	3.077	ASABIH	156.418	2.839
ASABIH	177.998	3.28	ASABUT	151.113	2.804	ASABIH	155.183	3.124	ASABIH	160.618	3.065	ASABIH	151.111	3.107
ASABUT	156.677	3.462	ASABUT	161.297	3.059	ASABUT	152.569	3.345	ASABUT	152.348	3.255	ASABUT	157.792	2.88
ASAGAD	150.247	3.404	ASAJIN	158.356	3.464	ASABUT	153.551	3.191	ASABUT	178.437	3.334	ASACUU	162.375	3.437
ASAJIN	164.897	3.476	ASAJIN	158.76	3.429	ASAJIN	165.489	3.476	ASAJIN	154.903	3.066	ASAJIN	164.228	3.073
ASAKEK	163.495	3.249	ASAKEK	162.344	3.038	ASAJOT	157.161	3.009	ASAJUZ	150.96	2.809	ASAJUZ	158.065	2.892
ASAKEK	170.605	3.418	ASAKEK	153.377	3.35	ASAKEK	154.821	3.275	ASAKEK	159.046	3.169	ASAKEK	160.846	2.958
ASASEU	170.494	3.33	ASAVUN	163.328	3.108	ASAKEK	151.118	3.286	ASAKOU	154.384	3.119	ASAPIV	167.368	2.966
ASAWAS	157.26	2.796	ASAWID	174.833	2.812	ASAWAS	161.858	3.125	ASAWAS	171.678	2.796	ASAWAS	166.94	3.499
ASELAM	150.277	3.384	ASEPOE	158.679	3.256	ASEGUA	163.043	3.118	ASELAM	166.011	3.176	ASELAM	162.647	3.244
ASEVEA	155.343	3.322	ASIBAG	169.489	3.147	ASEPOE	153.953	3.14	ASEPOE	152.159	3.393	ASEQAR	154.44	2.962
ASIKUJ	163.705	3.199	ASIKUJ	151.5	2.914	ASIJET	175.73	3.207	ASIJET	154.198	2.989	ASIJET	166.969	3.42
ASIKUJ	166.621	2.681	ASILEU	164.436	3.125	ASIKUJ	161.631	3.169	ASIKUJ	167.858	3.051	ASIKUJ	158.79	3.101
ASILEU	155.718	2.893	ASINOH	167.925	3.493	ASILEU	153.947	2.838	ASILEU	161.901	2.961	ASILEU	155.483	2.968
ASOFEV	155.534	3.256	ASOFEV	153.815	3.443	ASINUO	168.033	3.348	ASINUO	160.399	2.83	ASIWIJ	158.791	3.223
ASONUT	166.09	3.087	ASONUT	154.095	2.962	ASOGAQ	152.452	3.17	ASOGIY	165.586	3.207	ASONUT	165.565	2.945
ASUBUN	164.711	3.126	ASUCAU	167.316	3.293	ASOROP	161.15	2.807	ASOROP	161.164	2.807	ASUBOH	155.696	2.884
ASULOP	168.892	3.19	ASUNOR	152.911	3.405	ASUCEY	169.936	3.317	ASULOP	156.757	3.34	ASULOP	158.593	3.279
ASUNOR	165.906	3.122	ASUPIQ	159.684	3.309	ASUNOR	153.267	3.254	ASUNOR	167.418	3.342	ASUNOR	153.357	3.126
ATAGAG	178.349	2.711	ATAGAG	174.147	2.713	ASUSEP	161.092	3.113	ASUSIT	152.271	3.315	ASUSIT	163.577	2.799
ATAGAG	160.071	3.498	ATAGAG	153.026	2.837	ATAGAG	174.674	2.711	ATAGAG	173.359	2.713	ATAGAG	161.717	2.947
ATAGEK	160.653	2.668	ATAGIO	166.716	2.712	ATAGAG	168.96	3.423	ATAGAG	171.708	3.351	ATAGEK	153.268	3.261
ATAGOU	163.611	2.888	ATAGUA	157.347	2.598	ATAGIO	156.065	3.138	ATAGIO	166.677	2.712	ATAGOU	165.334	2.877
ATAHIP	160.435	3.051	ATAHUB	154.262	3.199	ATAGUA	163.332	2.949	ATAGUA	155.995	2.598	ATAHIP	160.195	3.078
ATAJIR	157.334	2.985	ATAJIR	169.964	2.883	ATAJAJ	172.588	3.225	ATAJEN	151.316	2.988	ATAJIR	171.925	2.881
ATAKEO	155.018	3.234	ATAKEO	170.212	3.031	ATAJIR	154.995	2.985	ATAJIR01	168.085	2.944	ATAJIR01	167.261	2.936
ATAKIR	154.056	2.766	ATANIT	156.02	3.304	ATAKEO	167.819	3.031	ATAKEO	159.092	3.216	ATAKEO	152.543	3.031
ATAWEZ	152.761	3.487	ATEGUD	156.923	3.326	ATANIT	163.483	3.114	ATAVOI	161.511	3.237	ATAVUN	166.311	3.476
ATEZOR	153.538	3.063	ATEZOR	152.982	3.383	ATEGUD	165.115	3.274	ATEGUD	158.233	3.302	ATELET	169.92	3.131
ATEZUX	150.426	3.102	ATEZUX	159.426	3.102	ATEZOR	161.928	3.063	ATEZOR	154.721	3.447	ATEZUX	162.894	3.465
ATIKUL	171.366	2.635	ATIKUL	151.985	3.236	ATIKUL	158.762	3.242	ATIKUL	152.371	2.946	ATIKUL	163.011	2.833

ATISAZ	157.014	2.945	ATISAZ	161.217	3.38	ATIKUL	150.885	2.972	ATIKUL	156.439	3.149	ATISAZ	170.095	3.477
ATIVOP	150.214	3.049	ATIVOP	153.905	3.049	ATISAZ	155.567	3.178	ATISAZ	170.443	2.72	ATISAZ	151.548	3.238
ATIVOQ	150.36	3.231	ATIVUV	167.721	2.974	ATIVOP	161.661	3.463	ATIVOP	159.214	2.933	ATIVOP	167.562	3.483
ATIVUV	153.993	2.809	ATIVUV	153.658	2.745	ATIVUV	155.831	2.745	ATIVUV	172.377	2.717	ATIVUV	170.412	2.717
ATIWAC	160.469	3.495	ATIWAC	159.817	2.771	ATIVUV	154.399	3.476	ATIWAC	158.658	3.215	ATIWAC	157.013	3.162
ATIWEG	152.932	2.88	ATIWEG	156.563	3.084	ATIWAC	150.908	3.215	ATIWEG	152.813	2.88	ATIWEG	161.161	3.313
ATOFAR	166.519	2.834	ATOGAS	163.508	2.814	ATIWEG	151.363	3.118	ATIWEG	159.415	2.869	ATIWEG	151.88	3.329
ATONUV	165.9	3.186	ATONUV	158.332	2.917	ATOGEW	173.107	3.226	ATONUV	170.587	2.602	ATONUV	150.187	2.796
ATONUV	151.789	3.151	ATONUV	150.334	2.97	ATONUV	165	3.092	ATONUV	165.523	2.715	ATONUV	152.452	2.791
ATONUV	152.561	2.778	ATONUV	159.963	3.192	ATONUV	165.229	2.917	ATONUV	159.576	3.456	ATONUV	168.623	2.741
ATONUV	166.53	2.736	ATONUV	154.869	2.915	ATONUV	155.268	2.837	ATONUV	164.311	3.423	ATONUV	163.134	3.082
ATOQIK	164.529	2.805	ATOQIK	160.438	3.042	ATONUV	174.579	2.932	ATOQIK	170.212	2.842	ATOQIK	157.834	2.848
ATOQIK	150.836	2.956	ATOQOQ	167.48	3.258	ATOQIK	153.362	3.111	ATOQIK	171.936	3.415	ATOQIK	152.199	2.948
ATOQOS	159.074	3.139	ATOQUW	157.62	2.844	ATOQOS	171.784	3.031	ATOQOS	164.615	3.031	ATOQOS	153.429	2.684
ATOQUW	157.207	2.747	ATOQUW	158.638	3.45	ATOQUW	152.311	2.747	ATOQUW	151.336	3.039	ATOQUW	172.331	3.033
ATORAD	162.797	2.989	ATORAD	160.982	3.258	ATOQUW	153.363	3.033	ATORAD	159.332	2.974	ATORAD	156.913	3.418
ATORAD	151.413	3.141	ATORAD	153.345	3.422	ATORAD	167.821	2.914	ATORAD	162.916	3.487	ATORAD	156.94	3.441
ATOREH	154.098	2.82	ATOREH	177.28	3.495	ATORAD	166.45	2.84	ATORAD	161.905	3.018	ATORAD	157.795	3.258
ATOREH	178.078	2.96	ATOREH	160.47	2.938	ATOREH	169.178	3.115	ATOREH	165.029	3.309	ATOREH	153.713	2.82
ATOREH	160.983	2.784	ATOREH	164.593	3.311	ATOREH	159.289	2.948	ATOREH	165.747	3.174	ATOREH	153.837	3.089
ATORIL	156.46	3.311	ATORIL	158.368	2.906	ATOREH	153.795	3.106	ATOREH	160.659	2.684	ATOREH	160.653	2.957
ATORUX	161.166	2.809	ATOSAE	160.649	3.436	ATORIL	166.081	2.906	ATOROR	164.861	3.395	ATORUX	159.966	2.809
ATOSAE	169.799	2.838	ATOSAE	157.582	3.164	ATOSAE	168.995	2.782	ATOSAE	167.566	3.098	ATOSAE	151.215	3.493
ATOZOY0	153.646	2.744	ATOZOY0	154.34	2.744	ATOSAE	165.14	3.142	ATOSAE	171.371	3.356	ATOSEI	164.407	3.271
ATUDOJ	166.039	3.325	ATUJEF	153.083	3.029	ATUDOJ	153.571	2.993	ATUDOJ	156.381	2.993	ATUDOJ	169.847	3.325
ATUWAQ	160.474	2.963	ATUXAR	161.161	3.034	ATUVIX	162.317	2.932	ATUVIX	157.953	2.771	ATUWAQ	158.479	2.779
AVACUX	153.801	2.88	AVAGUB	159.88	2.998	ATUXAR	157.232	3.105	ATUXAR	175.113	2.653	ATUXAR	155.086	3.048
AVANET	175.943	3.031	AVANET	153.944	3.202	AVAJAL	152.444	3.396	AVAKOA	175.784	3.096	AVAKOA	152.018	3.334
AVEFAI	158.452	3.258	AVEGAJ	176.257	2.771	AVATUM	173.423	2.889	AVATUM	154.172	2.778	AVECUZ	159.459	3.273
AVEQIB	155.523	3.2	AVEQIB	151.772	3.413	AVEGAJ	159.383	2.771	AVEQEX	163.42	2.837	AVEQIB	157.738	3.474
AVEQOH	174.202	3.135	AVEQOH	155.122	3.022	AVEQIB	157.974	2.828	AVEQIB	170.843	3.167	AVEQOH	154.889	2.769
AVEVAZ	173.254	2.69	AVEVED	162.056	2.753	AVEQOH	172.147	3.088	AVEQOH	151.937	2.808	AVEVAZ	172.793	2.69
AVEWEF	174.122	3.094	AVEWEG	161.389	3.075	AVEVIH	162.886	3.472	AVEWAA	157.677	3.429	AVEWEF	155.211	3.308
AVIFUH	178.343	3.039	AVIFUH	167.297	2.768	AVEXEG	174.641	3.15	AVIFAO	161.685	3.485	AVIFUH	161.122	3.201
AVIRUT	157.548	3.014	AVIRUT	169.524	3.326	AVILUM	153.092	3.293	AVILUM	153.55	3.256	AVIPEC	157.877	3.108
AVIRUT	169.583	3.213	AVIRUT	168.603	3.079	AVIRUT	151.962	3.079	AVIRUT	152.692	2.951	AVIRUT	178.767	3.134
AVUHII	172.436	3.161	AVUHII	171.754	3.161	AVIZIQ	150.132	3.122	AVIZIQ	166.157	3.438	AVIZIQ	155.444	3.204
AVULIN	161.164	3.128	AVULIN	150.767	2.868	AVUJIM	160.494	2.839	AVUJIM	163.512	2.839	AVUJIM	165.734	3.499
AVUSUH	162.975	2.921	AVUZEZ	154.206	2.931	AVULIN	163.629	3.135	AVULIO	162.718	3.445	AVULUZ	154.811	3.037
AVUZID	165.212	3.349	AWABEI	150.275	2.966	AVUZEZ	161.019	2.898	AVUZID	152.278	2.781	AVUZID	161.47	3.352
AWAPEW	150.073	2.914	AWAPEW	170.265	3.483	AWABUX	154.108	3.102	AWABUX	153.329	3.148	AWAPAS	159.887	3.071
AWEGIS	167.439	3.179	AWEQEB	150.859	2.646	AWAVAY	164.284	2.669	AWEFUF	157.673	2.923	AWEFUF	173.028	3.225
AWIGIZ	150.254	3.034	AWIVEK	156.939	2.794	AWEWIJ	154.002	3.361	AWIFIW	165.05	3.428	AWIFIW	153.583	3.38
AWIVEK	162.442	3.452	AWIVEK	156.95	3.257	AWIVEK	171.066	2.713	AWIVEK	173.811	3.453	AWIVEK	172.161	3.357
AWIVEK	152.279	3.195	AWIVIL	169.309	2.964	AWIVEK	160.926	3.488	AWIVEK	167.28	2.719	AWIVEK	151.356	3.187
AWUCEC	156.621	2.924	AWUCEC	158.669	3.079	AWIYAJ	155.046	3.37	AWODOH	161.908	3.143	AWUCEC	150.828	2.687
AWUCUS	173.901	3.436	AWUCUS	161.098	2.914	AWUCEC	171.935	3.488	AWUCUS	168.756	3.441	AWUCUS	150.138	2.917
AWUCUS	156.962	2.878	AWUCUS	169.359	3.03	AWUCUS	151.705	2.755	AWUCUS	169.045	3.422	AWUCUS	158.895	3.479
AWUCUT	163.283	3.422	AWUCUT	158.937	2.932	AWUCUS	163.334	3.226	AWUCUS	168.452	3.42	AWUCUT	173.61	2.418
AWUDII	160.535	3.05	AWUDII	163.894	3.217	AWUCUT	165.12	3.354	AWUCUT	156.004	3.337	AWUDII	172.081	3.219
AWUDII	165.4	3.123	AWUFAC	155.492	3.436	AWUDII	155.048	2.893	AWUDII	160.199	2.693	AWUDII	150.876	2.87
AWUFAC	158.997	3.033	AWUFAC	164.993	2.827	AWUFAC	170.905	3.447	AWUFAC	178.848	2.946	AWUFAC	161.724	3.322
AWUKUA	160.682	2.789	AWUKUA	150.737	2.814	AWUFAC	152.66	2.954	AWUKAE	169.715	3.326	AWUKUA	158.534	2.784
AWUKUB	167.239	3.295	AWUWUN	165.849	3.156	AWUKUA	168.557	2.784	AWUKUB	163.778	2.769	AWUKUB	160.079	2.801
AXAKER	156.957	3.119	AXEGOB	155.734	2.83	AWUXAU	150.956	3.344	AWUXUM	168.952	3.422	AXAGUB	153.078	3.491
AXEGUH	151.827	2.855	AXEGUH	167.892	2.855	AXEGOB	152.419	3.143	AXEGOB	157.137	3.307	AXEGUH	152.557	3.481
AXEHAO	150.261	3.238	AXEHAO	175.42	2.911	AXEGUH	156.534	3.362	AXEHAO	151.867	3.333	AXEHAO	161.896	2.987
AXEHAO	156.542	3.244	AXEHAO	154.824	3.334	AXEHAO	162.365	2.868	AXEHAO	151.998	3.477	AXEHAO	162.829	3.036
AXEHAO0	153.042	3.255	AXEHAO0	163.303	2.959	AXEHAO	152.262	3.271	AXEHAO	163.38	2.907	AXEHAO	152.308	3.366
AXEHAO0	156.405	3.219	AXEHAO0	158.737	3.202	AXEHAO0	172.863	3.051	AXEHAO0	161.912	2.806	AXEHAO0	164.87	3.028
AXEHIW	157.28	2.998	AXEHIW	158.272	3.173	AXEHAO0	156.07	3.42	AXEHAO0	158.006	3.059	AXEHAO0	151.264	3.357
AXEHIW	164.144	2.967	AXEHIW	151.263	2.967	AXEHIW	154.879	2.932	AXEHIW	154.101	3.239	AXEHIW	153.268	2.853
AXEROM	166.561	2.645	AXESEC	167.532	3.193	AXEHIW	160.266	2.777	AXELIA	171.78	3.043	AXELIA	177.369	3.245
AXICIV	156.564	3.367	AXICIV	152.951	2.865	AXEWUX	169.75	3.399	AXEWUX	150.753	3.168	AXICIV	153.924	3.258
AXIFAQ	160.049	2.732	AXIFAQ	176.53	2.899	AXICOC	161.803	2.821	AXICOC	163.262	2.821	AXIFAP	153.037	3.398
AXIHAQ	152.083	3.262	AXIJOI	163.07	3.297	AXIFAQ	169.263	3.088	AXIHAQ	158.215	3.004	AXIHAQ	163.348	3.114
AXIQON	157.268	3.081	AXIQON	153.193	3.448	AXIQAC	156.379	3.374	AXIQIJ	158.03	3.112	AXIQON	154.882	3.482
AXIZEP	151.571	3.279	AXIZOY02	172.698	3.102	AXIXEK	160.147	2.995	AXIXIO	152.907	2.959	AXIYOX	157.804	3.265
AXOJUV02	157.549	3.208	AXOKAC	162.75	3.015	AXIZOY02	167.894	3.293	AXOJAB	157.544	3.036	AXOJUV	157.502	3.173
AXOPIO	166.922	3.2	AXOPIO	172.616	3.269	AXONEJ	152.209	3.001	AXONEJ	154.835	3.259	AXOPIO	157.829	3.397
AXOZAP	161.137	2.812	AXOZOF	155.135	3.266	AXOXES	171.416	3.376	AXOXIW	166.231	3.264	AXOXIW	154.814	3.31
AXUBEA	169.724	2.977	AXUBEA	162.233	3.127	AXOZOF	153.716	2.796	AXOZOF	150.034	3.276	AXOZOF	155.262	3.183
AXUBEA	150.044	3.149	AXUBEA	163.498	3.285	AXUBEA	172.705	3.445	AXUBEA	158.044	2.984	AXUBEA	169.668	3.283
AXUBIE	156.979	2.794	AXUDAY	151.518	2.901	AXUBIE	161.631	3.158	AXUBIE	152.933	2.794	AXUBIE	159.271	3.404
AXUDON	161.889	2.789	AXURAN	162.39	3.329	AXUDEC	158.974	2.838	AXUDEC	159.418	2.84	AXUDON	150.888	3.362
AXURUI	155.834	2.87	AXURUI	163.896	2.588	AXURAO	159.165	3.141	AXURAO	159.171	3.449	AXURAO	161.897	3.423
AXUWEX	167.248	2.793	AXUXEZ	156.96	2.999	AXUTUI	163.337	2.813	AXUTUI	165.25	2.813	AXUVAQ	167.817	3.155
AXUYID	152.696	3.013	AXUYID	151.517	3.47	AXUXEZ	157.168	2.881	AXUYID	173.96	3.47	AXUYID	160.338	2.818
AYACAG	151.173	3.126	AYACAG	158.688	3.429	AYACAG	161.891	3.459	AYACAG	156.135	3.394	AYACAG	162.532	3.061

AYACIO	150.716	3.357	AYADAI	163.472	2.852	AYACAG	154.475	2.866	AYACIO	167.948	3.086	AYACIO	176.968	3.391
AYAMIZ	163.194	3.115	AYAPEX	169.604	3.481	AYAMAR	166.883	2.843	AYAMAR	153.606	3.031	AYAMIZ	150.192	3.328
AYAPEX	155.578	3.49	AYARIC	161.459	3.005	AYAPEX	152.878	3.416	AYAPEX	150.867	3.167	AYAPEX	154.079	2.979
AYAROI	150.358	2.895	AYAROI	150.238	3.438	AYARIC	166.985	2.873	AYAROI	158.775	3.21	AYAROI	161.099	2.901
AYAVUS	159.491	3.155	AYAVUS	167.259	3.336	AYAVUS	151.399	3.277	AYAVUS	155.927	3.345	AYAVUS	165.976	2.954
AYAZEG	151.429	3.074	AYAZEG	164.125	3.067	AYAVUS	174.714	3.155	AYAZEG	156.215	3.293	AYAZEG	164.501	3.076
AYEHAP	166.218	3.231	AYEHAP	160.352	3.405	AYEHAP	161.652	3.041	AYEHAP	154.109	3.041	AYEHAP	164.095	3.206
AYEJUL	160.116	3.201	AYEJUL	154.617	2.961	AYEJUL	157.655	3.173	AYEJUL	156.184	3.205	AYEJUL	170.482	3.028
AYEKAQ	163.54	3.217	AYEKAQ	165.318	3.147	AYEJUL	163.084	2.897	AYEJUL	164.631	3.185	AYEJUL	165.24	3.193
AYISEG	175.816	3.363	AYISEG	163.907	3.05	AYEKAQ	153.962	3.07	AYIFUM	162.441	3.242	AYISEG	168.383	3.172
AYIVUC	164.224	3.057	AYIWEN	170.816	3.118	AYIVEK	171.33	3.094	AYIVEK	165.124	3.094	AYIVEK	166.211	2.875
AYOLAC	151.311	2.928	AYOPEK	159.097	3.254	AYOHUR	157.1	2.882	AYOHUR	156.448	2.898	AYOLAC	158.498	3.129
AYOPEL	173.555	3.361	AYOPEL	164.204	3.333	AYOPEL	160.032	3.18	AYOPEL	159.677	3.215	AYOPEL	157.995	3.01
AYOXUH	153.137	3.423	AYUGUW	175.997	2.822	AYOPEL	169.725	3.176	AYOPEL	167.262	3.082	AYOPEL	151.374	2.938
AYURET	173.84	3.022	AYURIX01	153.346	2.787	AYUGUW	154.434	3.159	AYUGUW	167.188	2.977	AYUGUW	159.778	2.871
AYURIX01	154.108	3.097	AYUZAW	154.372	3.009	AYURIX01	153.75	2.897	AYURIX01	163.192	3.015	AYURIX01	155.078	2.787
AZEFOD	161.615	3.138	AZEFOD	174.632	3.282	AZACOU	175.466	3.275	AZAHAL	169.994	3.461	AZEFOD	152.36	3.138
AZEFOD	159.765	3.111	AZEFOD	153.801	3.108	AZEFOD	156.538	3.165	AZEFOD	160.446	2.942	AZEFOD	165.625	3.027
AZEHAO	153.854	3.315	AZEHAP	168.848	3.364	AZEHAO	154.744	3.077	AZEHAO	160.925	3.046	AZEHAO	156.355	2.897
AZEKIA	161.211	3.24	AZEKIB	167.39	2.926	AZEHAP	169.048	3.421	AZEHOC	159.086	2.873	AZEHOC	171.084	3.224
AZEWOU	160.133	3.335	AZEWOU	159.702	3.093	AZELIB	153.062	3.11	AZELIB	150.876	3.216	AZEWOU	160.488	3.396
AZEZAH	153.685	2.875	AZEZAH	173.341	3.362	AZEWOU	164.344	3.024	AZEWOU	151.964	3.396	AZEZAH	171.433	3.31
AZFAPD15	154.99	2.916	AZFAPD16	157.562	2.952	AZEZAH	159.345	2.942	AZFAPD10	160.884	3.057	AZFAPD14	154.267	2.912
AZFAPD20	159.709	3.093	AZFAPD25	160.21	3.05	AZFAPD17	159.372	2.992	AZFAPD18	160.901	3.069	AZFAPD19	159.888	3.084
AZIKIF	171.075	3.066	AZISUX	159.885	2.827	AZICET	154.714	2.925	AZIDOC	169.736	3.306	AZIDOC01	173.034	3.308
AZISUX	153.894	2.926	AZISUX	155.065	2.814	AZISUX	154.405	2.907	AZISUX	168.274	3.035	AZISUX	164.173	2.829
AZIVEK	154.234	3.258	AZIVEK	153.263	2.776	AZIVEK	156.51	2.776	AZIVEK	160.62	3.082	AZIVEK	159.429	2.753
AZIVEK	154.877	2.861	AZIVEK	172.073	2.861	AZIVEK	155.046	3.238	AZIVEK	153.773	2.753	AZIVEK	164.098	3.005
AZODOI	151.545	2.916	AZODOI	150.83	3.146	AZIVEK	160.21	3.01	AZIWOY	151.233	3.479	AZODOI	152.793	2.916
AZOSEP	172.381	2.86	AZOSEP	157.754	2.945	AZOSEP	153.69	3.207	AZOSEP	163.174	3.305	AZOSEP	156.857	2.945
AZOSEP02	167.301	3.349	AZOTEQ	169.468	3.056	AZOSEP02	154.033	3.221	AZOSEP02	164.098	3.248	AZOSEP02	154.176	3.248
AZOVIW	155.399	3.177	AZOWAQ	172.898	3.486	AZOTEQ	159.116	3.309	AZOVIW	159.28	3.142	AZOVIW	161.985	3.177
AZOYAP	166.137	3.165	AZOYAP	164.168	3.116	AZOXEU	162.672	3.446	AZOXEU	174.108	3.334	AZOYAP	173.336	2.946
AZUCAC	152.77	3.277	AZUCAC	176.367	3.036	AZOZEV	158.332	3.385	AZUCAC	155.263	2.982	AZUCAC	156.317	3.297
AZUCAC	154.549	2.938	AZUCAC	160.837	3.424	AZUCAC	150.779	2.979	AZUCAC	151.693	3.465	AZUCAC	167.371	3.491
AZUCUW	165.14	2.921	AZUCUW	157.275	3.053	AZUCUW	156.19	3.053	AZUCUW	154.127	3.237	AZUCUW	150.709	2.803
AZUPAN	154.701	3.33	AZUPAN	151.031	2.832	AZUDIL	152.601	3.239	AZUKOY	154.118	3.169	AZUPAN	156.635	2.715
AZUYAY	173.873	2.936	BABLUO	150.579	3.267	AZUPAN	150.146	3.395	AZUYAY	161.016	2.941	AZUYAY	160.698	2.859
BABQOO	157.526	3.33	BABYIR	161.597	2.916	BABLUO	153.334	3.345	BABPUS	154.773	3.373	BABPUS	164.876	3.373
BACFIV	154.316	2.739	BACGEV	171.499	3.021	BABYIR	160.654	2.963	BABZAF	174.38	3.14	BACFIV	155.95	2.867
BACGEV0	162.083	3.5	BACGEV0	171.318	3.025	BACGEV	162.588	2.891	BACGEV	159.611	3.109	BACGEV	157.454	2.891
BACMIG	162.999	3.216	BACMIG	151.819	3.273	BACGEV0	162.623	2.895	BACGEV0	159.736	3.1	BACGEV0	157.489	2.895
BACWIO	151.315	3.392	BACWOW	153.597	3.103	BACMIG	153.48	3.273	BACVIN	161.885	3.192	BACWAG	150.603	3.397
BACYAF	163.352	3.2	BACYAF	161.007	2.998	BACXUY	159.177	3.456	BACYAF	162.525	2.961	BACYAF	158.338	2.898
BACYAF	161.923	2.867	BACYAF	150.461	2.995	BACYAF	163.081	2.934	BACYAF	168.518	2.995	BACYAF	156.322	2.906
BADCUI	153.118	3.425	BADDOB	174.62	2.815	BADCUI	152.349	3.439	BADCUI	173.878	2.652	BADCUI	150.422	3.264
BADHIC	163.597	3.405	BADJAT	166.565	3.104	BADHIC	162.674	2.938	BADHIC	166.578	2.958	BADHIC	172.425	3.4
BADKOK	169.539	3.332	BADMIH	166.799	3.059	BADKOH	154.551	3.229	BADKOH	163.892	3.293	BADKOH	165.605	3.415
BADMIH	152.548	3.496	BADMIH	159.824	3.007	BADMIH	160.419	3.48	BADMIH	170.277	3.367	BADMIH	157.5	3.423
BADSUY	152.416	3.433	BADSUY	158.336	3.316	BADMIH	155.851	3.367	BADNEB	171.486	3.081	BADSOS	155.792	2.94
BADTEJ	155.198	3.419	BADZUE	167.079	3.079	BADTEJ	173.063	2.935	BADTEJ	165.48	3.423	BADTEJ	150.313	3.221
BAFBAO	152.51	3.343	BAFBAO	151.251	3.084	BAFBAO	161.91	3.343	BAFBAO	155.49	3.187	BAFBAO	165.669	3
BAFPOP	176.986	3.264	BAFRIM	163.267	3.431	BAFKEA	163.122	3.152	BAFNUV	163.986	3.34	BAFNUV	158.929	3.325
BAFWUB	150.589	2.971	BAFWUB	161.458	3.332	BAFWUB	150.348	2.949	BAFWUB	156.881	2.971	BAFWUB	157.739	2.845
BAFYAJ01	168.993	3.108	BAFYAJ01	164.912	3.34	BAFYAJ01	172.504	2.846	BAFYAJ01	159.817	2.799	BAFYAJ01	167.862	3.442
BAGCAN	153.027	3.182	BAGCAN	175.289	2.856	BAFYAJ01	150.589	3.16	BAGCAN	165.069	3.254	BAGCAN	151.014	3.087
BAGPOR	160.578	3.485	BAGRIO	163.163	3.155	BAGCAS	151.583	2.663	BAGCAS	173.262	3.077	BAGCUH	155.64	3.176
BAGXOB	158.95	3.316	BAGXOB	162.427	3.057	BAGTOX	166.098	3.451	BAGXOB	151.536	3.057	BAGXOB	165.546	3.018
BAHJII	174.519	3.367	BAHJII	155.831	3.061	BAGXOB	161.145	3.143	BAGZUH	157.899	3.036	BAHHEZ	164.34	3.266
BAHJII	177.63	3.138	BAHJII	173.082	3.042	BAHJII	152.317	3.352	BAHJII	155.861	3.187	BAHJII	151.379	3.367
BAHRUA	163.092	3.12	BAHRUA	153.209	3.485	BAHJII	161.345	3.242	BAHJII	152.772	3.339	BAHKOL	163.264	3.062
BAJFUR	158.045	3.142	BAJFUR	157.301	3.172	BAHZAN	173.502	2.982	BAHZAN	162.749	3.229	BAJDIB	154.811	2.798
BAJFUR	173.729	3.47	BAJFUR	151.52	3.013	BAJFUR	150.83	3.142	BAJFUR	151.022	2.911	BAJFUR	160.103	3.104
BAJFUR	155.79	3.148	BAJHUQ	155.915	3.372	BAJFUR	158.909	3.347	BAJFUR	166.669	3.29	BAJFUR	167.168	3.266
BAJMEE10	167.573	3.406	BAJMEE10	160.393	3.477	BAJHUQ	166.751	3.078	BAJMEE10	176.708	3.477	BAJMEE10	177.576	3.067
BAJRUB	157.138	3.092	BAKGOM	152.616	3.067	BAJRUB	155.706	2.961	BAJRUB	164.801	3.481	BAJRUB	165.956	2.989
BAKHIF01	154.933	3.39	BAKLAE	150.594	3.396	BAKGOM	159.128	3.239	BAKGOM	156.392	3.231	BAKGOM	163.096	3.027
BAKPAE02	166.579	2.963	BAKPAE04	166.549	2.942	BAKLIM	156.008	3.471	BAKPAE	167.193	3.015	BAKPAE01	166.605	2.972
BALGOO	171.214	3.187	BALGOO	164.317	2.93	BAKPAE05	179.844	2.981	BAKPAE05	179.548	3.298	BAKZOD	174.28	3.343
BALSUF	170.029	2.877	BALTAM	172.082	2.778	BALLIK	152.781	3.327	BALSAM	156.242	3.493	BALSOZ	171.483	2.819
BAMDOI10	153.386	2.907	BAMDOI10	151.303	3.046	BALZIB	151.027	2.963	BAMBIC	150.415	3.192	BAMBIC	153.391	3.029
BAMGUV	162.944	3.281	BAMJIM	153.639	2.84	BAMGAY0	160.992	3.498	BAMGEF	155.624	3.32	BAMGUV	155.404	3.164
BAMKAD	150.512	3.287	BAMKAD	160.935	3.173	BAMJIM	151.414	3.022	BAMKAD	153.816	3.218	BAMKAD	173.09	3.408
BAMMAF	154.899	3.486	BAMMAF	151.181	3.191	BAMKAD	163.079	3.462	BAMMAF	160.06	3.072	BAMMAF	159.755	3.056
BAMMIN	164.635	3.375	BAMMIN	167.06	2.979	BAMMAF	167.294	3.245	BAMMAF	157.814	3.384	BAMMIN	150.398	3.142
BAMMIN	168.62	2.97	BAMMIN	159.111	3.292	BAMMIN	154.277	3.343	BAMMIN	161.312	3.187	BAMMIN	158.766	3.413
BAMNUC	167.402	2.869	BAMSOB	168.436	3.424	BAMMOR	155.988	3.133	BAMMOR	156.369	3.133	BAMNUC	171.279	3.126
BAMWOE	167.921	2.971	BAMWOE	153.114	2.927	BAMTOA	156.783	3.286	BAMTOA	163.068	3.031	BAMVIY	163.127	3.04
BANCIC	152.936	3.486	BANCIC	153.595	3.086	BAMWOE	157.147	3.081	BAMWOE	162.051	3.189	BANCIC	155.959	2.977

BANCIC	156.43	3.437	BANCIC	154.862	3.112	BANCIC	162.41	2.822	BANCIC	163.29	3.34	BANCIC	168.829	2.822
BANCIC10	162.41	2.822	BANCIC10	163.29	3.34	BANCIC10	155.959	2.977	BANCIC10	152.936	3.486	BANCIC10	153.595	3.086
BANGOM0	154.546	3.293	BANXIB	160.644	3.346	BANCIC10	168.829	2.822	BANCIC10	156.455	3.435	BANCIC10	154.862	3.112
BAPBEA	154.535	2.907	BAPBEA	172.288	3.31	BANYOH	158.73	3.016	BANYOH	164.857	3.198	BANYOH	152.685	3.113
BAPCIH	166.964	3.283	BAPFUV	161.08	3.445	BAPCAW	158.002	3.206	BAPCIH	153.909	3.283	BAPCIH	159.879	3.029
BAPMEN	152.165	3.335	BAPMEN	154.072	3.351	BAPGIK	163.473	3.453	BAPHIK	150.987	3.401	BAPJEK	170.757	3.159
BAPNOW	164.505	2.989	BAPNOW	162.713	3.147	BAPMEN	172.538	2.934	BAPNOW	159.477	2.941	BAPNOW	159.91	3.31
BAPNUC	166.478	3.429	BAPVEX	161.926	3.229	BAPNUC	150.875	3.439	BAPNUC	154.897	3.108	BAPNUC	151.925	2.981
BAPWEV	150.757	3.136	BAPWEV	155.46	3.376	BAPVEX	174.456	2.979	BAPWEV	163.508	3.11	BAPWEV	150.55	3.398
BAPZEZ	156.106	3.193	BAPZUP	150.55	2.921	BAPWEV	167.169	2.785	BAPYUP	156.275	2.839	BAPYUP	159.973	3.069
BAQGOR	155.213	2.91	BAQGOR	157.035	3.402	BAQGAE	162.05	3.138	BAQGOR	163.636	2.91	BAQGOR	158.551	2.936
BAQGOR	174.252	2.995	BAQGOR	153.473	3.151	BAQGOR	154.19	3.214	BAQGOR	157.503	3.068	BAQGOR	164.268	2.962
BAQGUX	165.381	2.932	BAQGUX	161.16	2.933	BAQGUX	158.998	2.963	BAQGUX	153.129	3.067	BAQGUX	160.447	3.087
BAQGUX	159.921	3.315	BAQGUX	154.566	3.105	BAQGUX	162.062	3.37	BAQGUX	162.092	3.438	BAQGUX	153.433	3.404
BAQKOV	154.278	3.097	BAQKOV	150.047	2.962	BAQGUX	160.446	2.926	BAQGUX	154.323	3.122	BAQKOV	150.116	3.32
BAQSER	160.969	3.075	BAQVEV	153.122	3.103	BAQLUE	155.041	3.252	BAQSER	155.186	3.205	BAQSER	154.083	3.361
BAQYAT	151.641	3.357	BAQYEX	157.811	2.932	BAQVEV	150.002	3.468	BAQYAT	156.921	3.102	BAQYAT	176.55	3.444
BAQYEX	151.65	3.127	BAQYEX	158.117	3.206	BAQYEX	165.869	3.314	BAQYEX	151.684	3.377	BAQYEX	151.358	2.947
BAQZEY	150.151	2.915	BAQZEY	160.599	2.905	BAQYEX	163.851	2.726	BAQZEY	153.244	2.883	BAQZEY	152.202	3.199
BAQZIC	154.267	3.239	BAQZIC	152.275	2.811	BAQZEY	167.522	2.954	BAQZEY	155.721	3.147	BAQZEY	155.778	2.903
BARGAD	155.378	3.44	BARHAG	153.997	3.337	BAQZIC	160.115	3.105	BAQZIC	154.919	2.992	BAQZIC	153.078	3.109
BARHEK	166.201	3.212	BARHEK	177.881	3.209	BARHAG	171.391	3.263	BARHAG	151.168	3.146	BARHAG	167.21	3.208
BARKOY	153.052	2.973	BARKOY	162.781	2.91	BARHIO	150.955	2.92	BARHIO	161.933	3.156	BARKEO	157.456	3.111
BARLIP	175.538	2.998	BARLUE	156.795	2.893	BARLIP	155.647	3.303	BARLIP	160.52	3.204	BARLIP	152.47	3.078
BARVEW	169.708	3.416	BARVEW	156.524	3.35	BARLUE	169.371	3.259	BARVEW	175.312	3.108	BARVEW	161.912	2.982
BASBOM	158.8	3.166	BASBOM	159.431	2.979	BARYEB	160.848	2.991	BASBOM	164.208	3.121	BASBOM	152.364	3.166
BASPES	163.607	3.287	BASPUI	157.151	3.298	BASBOM	157.892	3.121	BASGEI	169.751	3.094	BASGEI	171.363	3.006
BATFAD	166.976	2.935	BATFAD	171.53	2.86	BASRIZ	162.814	3.194	BASXEZ	153.511	2.701	BATCUV	164.408	2.912
BATLIR10	155.838	3.271	BATLIU	155.878	2.863	BATFAD	171.124	2.878	BATFAD	161.17	3.001	BATLIR	155.838	3.271
BATLIU	153.876	3.176	BATLIU	154.319	2.938	BATLIU	155.418	3.174	BATLIU	156.82	3.347	BATLIU	160.553	2.836
BATNAP	161.659	2.974	BATNAP	157.65	2.911	BATLIU	158.815	2.863	BATNAP	164.685	2.778	BATNAP	164.811	2.996
BAVJIS	173.738	3.105	BAVJIS	169.48	3.009	BATNAP	162.139	2.953	BATNAP	167.297	2.979	BAVJIS	151.016	3.198
BAVJIS	153.764	3.211	BAVJIS	159.162	3.1	BAVJIS	161.883	2.926	BAVJIS	170.529	3.177	BAVJIS	166.841	3.177
BAVJIS	153.429	3.006	BAVJIS	162.746	3.113	BAVJIS	168.312	2.737	BAVJIS	150.004	3.086	BAVJIS	159.032	3.077
BAWPUK	154.348	3.031	BAWPUK	152.843	3.002	BAVJIS	159.418	3.202	BAVJIS	151.083	3.166	BAVJIS	157.6	2.65
BAWYIJ	164.18	3.432	BAXFUE	163.992	3.396	BAWPUK	160.739	3.042	BAWPUK	165.45	2.957	BAWQUL	159.475	3.485
BAXJET	165.757	3.498	BAXJET	165.134	3.192	BAXHOA	166.74	3.143	BAXHUG	156.879	3.489	BAXJET	158.103	3.106
BAXLUH01	160.194	2.812	BAXLUH01	153.834	2.812	BAXKOC	157.571	3.488	BAXKUI	154.861	3.011	BAXKUI	170.007	3.35
BAXPOI	150.348	3.459	BAYCUC	166.276	3.248	BAXLUH01	160.226	3.359	BAXLUH01	162.397	3.386	BAXNOE	164.037	3.107
BAYKOE	172.942	3.124	BAYKOE	162.013	2.838	BAYCUC	164.522	3.334	BAYHER	153.595	2.981	BAYKOE	167.562	2.891
BAYKOE	169.174	3.014	BAYKOE	162.711	3.328	BAYKOE	168.379	2.905	BAYKOE	160.001	3.124	BAYKOE	150.63	3.132
BAYKOE	166.86	2.887	BAYKOE	158.02	3	BAYKOE	152.089	3.281	BAYKOE	153.586	2.966	BAYKOE	160.813	3.435
BAYMOG	172.804	2.989	BAYMUM	154.816	3.148	BAYKOE	157.293	2.846	BAYLAR	171.639	3.116	BAYMOG	154.027	3.285
BAYSAZ	153.439	3.475	BAYSEZ	167.157	3.121	BAYSAX	152.416	3.441	BAYSAZ	152.05	3.298	BAYSAZ	166.221	3.052
BAYZUY	159.032	3.125	BAYZUY	154.202	2.985	BAYSEZ	156.368	2.993	BAYXAD	169.953	2.788	BAYZUY	156.418	2.932
BAZGOZ	163.169	3.372	BAZGOZ	166.465	2.933	BAZGAL	166.165	2.99	BAZGAL	158.032	3.045	BAZGOZ	155.081	3.161
BAZKUL	159.604	3.231	BAZKUL	151.586	3.122	BAZGOZ	154.64	3.045	BAZKUL	160.094	3.432	BAZKUL	155.226	3.032
BAZMEU	159.354	3.333	BAZPEX	163.613	3.389	BAZKUL	155.368	2.938	BAZLAS	154.362	3.112	BAZLOG	154.114	3.217
BAZPOH	160.212	3.469	BAZREC	155.537	3.174	BAZPEX	154.25	3.063	BAZPEX	153.749	3.26	BAZPEX	159.976	3.15
BAZRIE	152.47	2.755	BAZRIE	152.704	3.412	BAZREC	156.793	3.068	BAZREC	158.763	2.892	BAZRIE	169.406	2.822
BAZRIE	168.319	3.06	BAZRIE	150.301	3.462	BAZRIE	158.699	2.755	BAZRIE	163.646	3.339	BAZRIE	167.971	2.888
BAZRIE	154.011	3.077	BAZRIE	169.209	3.374	BAZRIE	152.935	3.498	BAZRIE	167.169	2.912	BAZRIE	153.438	3.144
BAZROK	158.477	2.88	BAZROK	153.09	3.378	BAZRIE	156.275	2.947	BAZRIE	166.952	2.947	BAZROK	170.786	3.094
BAZSEA	160.716	2.756	BAZSEA	156.398	2.756	BAZROK	170.376	2.733	BAZSEA	151.084	2.999	BAZSEA	151.167	3.015
BAZVON	160.624	3.223	BAZVON	158.686	3.116	BAZSEA	157.068	3.028	BAZSEA	155.108	3.141	BAZVON	172.767	3.326
BAZVON	164.154	3.281	BAZYEG	155.228	3.002	BAZVON	169.843	3.138	BAZVON	152.193	3.118	BAZVON	150.781	3.145
BEBGOI	151.804	3.155	BEBHIA	164.471	2.942	BAZYEG	157.374	2.74	BEBBES	154.751	3.444	BEBBES	162.932	3.486
BEBXEN	164.062	3.195	BEBXIR	164.811	2.96	BEBWAJ	157.501	2.759	BEBXAJ	154.14	3.327	BEBXEN	158.872	3.105
BEBXUD	156.75	3.191	BEBXUD	155.075	3.249	BEBXIR	163.697	2.96	BEBXIR	164.175	3.2	BEBXUD	151.423	3.193
BEBYUG	153.111	3.456	BEBYUG	158.688	3.11	BEBXUD	155.617	3.479	BEBYEO	155.074	2.839	BEBYUG	155.526	3.401
BEBYUG	174.805	3.407	BEBYUG	168.874	2.872	BEBYUG	162.521	2.738	BEBYUG	161.761	3.152	BEBYUG	151.735	3.162
BEBYUG	161.291	3.389	BEBYUG	171.586	2.702	BEBYUG	152.387	2.94	BEBYUG	157.023	3.353	BEBYUG	163.519	3.489
BEBZEN	157.179	3.435	BEBZIT	159.243	3.087	BEBZEN	151.14	3.417	BEBZEN	153.343	2.927	BEBZEN	160.075	3.018
BECBEU	153.722	3.077	BECCET	158.503	3.405	BECBEU	158.876	2.941	BECBEU	164.264	2.786	BECBEU	154.68	3.202
BECCIX	159.498	3.175	BECCIX	157.024	3.124	BECCET	155.039	3.091	BECCET	169.616	3.315	BECCET	156.307	2.829
BECDAQ	159.306	2.893	BECFAS	150.661	3.31	BECCIX	163.934	2.876	BECCIX	155.108	3.284	BECCIX	153.643	3.295
BECFEW	152.205	3.04	BECFIA	156.316	2.97	BECFAS	151.229	3.019	BECFAS	161.378	3.197	BECFAS	158.61	2.861
BECFUM	161.494	2.96	BECFUM	174.228	3.039	BECFOG	160.13	3.429	BECFOG	156.766	3.429	BECFUM	166.082	2.798
BECFUM	172.064	2.909	BECFUM	154.4	3.301	BECFUM	152.514	2.972	BECFUM	167.044	2.979	BECFUM	160.976	3.103
BECGAT	168.989	3.34	BECGAT	159.374	2.66	BECFUM	160.953	2.884	BECFUM	159.963	2.954	BECGAT	175.839	2.849
BECGAT	158.221	3.297	BECGAT	157.473	3.101	BECGAT	157.09	2.646	BECGAT	164.551	2.849	BECGAT	170.313	3.34
BECGAT0	155.904	3.197	BECGAT0	163.847	3.192	BECGAT0	151.672	3.3	BECGAT0	164.632	3.102	BECGAT0	155.932	3.455
BEDGOK	154.799	3.317	BEDGUN	151.433	2.918	BECQOS	151.152	2.867	BECYUE	155.644	3.099	BECYUE	158.9	3.306
BEDKAA	165.134	3.162	BEDNUV	156.286	2.938	BEDGUN	161.623	3.451	BEDHAU	160.61	3.46	BEDHAU	150.861	2.935
BEFGOL	166.853	3.002	BEFGOL	177.595	3.002	BEDTAF	167.545	2.868	BEDTAF	165.507	2.959	BEFFEZ	157.392	3.134
BEGNOT	171.299	2.8	BEGNOT	173.001	2.833	BEFGUS	151.477	2.865	BEFMEH	160.642	3.235	BEFSOW	154.332	3.326
BEGNOT	156.637	2.902	BEGNOT	154.069	2.87	BEGNOT	177.853	2.87	BEGNOT	173.072	2.884	BEGNOT	161.495	2.818
BEHQOY	164.441	2.837	BEHQOY	166.953	2.708	BEGNOT	152.43	2.884	BEGXAP	163.668	3.331	BEGXAP	153.837	3.331
BEJDOM	160.618	3.035	BEJSAL	166.536	2.88	BEHRAL	168.999	3.32	BEHSUC	168.087	3.338	BEHWAM	157.758	3.261

BEJSAL	152.145	3.308	BEJSAL	166.029	2.988	BEJSAL	151.005	3.141	BEJSAL	159.099	3.144	BEJSAL	159.991	3.322
BEJSAL	153.888	3.391	BEJSAL	152.729	3.143	BEJSAL	156.037	3.337	BEJSAL	163.546	3.464	BEJSAL	158.071	3.348
BEKBAX	159.363	2.986	BEKBAX	160.256	2.821	BEJSAL	150.37	3.224	BEJSAL	152.678	3.489	BEJXOG	166.456	2.977
BEKBEB	163.42	2.979	BEKBEC	163.834	2.964	BEKBAX	163.968	2.981	BEKBEB	158.868	3.004	BEKBEB	161.706	2.804
BEKKEL	153.259	3.004	BEKSIW	162.794	3.083	BEKBEC	167.333	3.244	BEKBUS	169.571	3.165	BEKDUR	157.873	3.45
BEKTET	158.555	3.226	BEKTET	175.013	3.417	BEKSIW	158.311	3.178	BEKTET	163.266	2.989	BEKTET	154.434	3.123
BEKZOI	164.887	3.245	BEKZOI	163.509	2.855	BEKZIE	153.309	2.899	BEKZIE	160.063	2.951	BEKZIE	153.317	2.951
BEMLAI	166.437	2.959	BEMLOW	159.747	2.892	BEKZOI	164.957	2.976	BEKZOI	169.098	2.986	BELBON	165.038	3.258
BEMSAO0	153.72	3.067	BEMSAO0	168.282	2.985	BEMLOW	152.489	3.033	BEMLUC	150.528	3.021	BEMSAO0	150.14	3.135
BEMSAO0	152.681	2.926	BEMSAO0	150.133	2.895	BEMSAO0	165.935	3.413	BEMSAO0	156.873	3.277	BEMSAO0	156.998	3.226
BEMWIB	154.468	3.092	BEMWIB	168.644	2.589	BEMSAO0	152.711	3.293	BEMSAO1	175.088	3.278	BEMSAO1	159.672	3.483
BENJIO	153.637	3.457	BENJIO	155.111	3.468	BEMWIB	169.43	2.589	BEMWIB	151.451	3.324	BENJIO	159.659	3.491
BENMOZ	151.457	3.308	BENQUG	161.863	3.028	BENKAK	158.409	3.179	BENLUE	151.105	3.497	BENMOA	150.182	3.27
BEPLER	174.278	3.486	BEPLIV	158.942	3.004	BENSUK	152.626	2.965	BENWAV	157.369	3.016	BENWAV	167.291	3.324
BEPQUL	160.088	2.901	BEPQUL	154.123	2.92	BEPLIV	156.861	2.918	BEPMEP	151.096	3.044	BEPQOF	168.511	2.845
BEPWUS	151.186	3.213	BEPXED	166.229	3.078	BEPQUL	158.004	3.431	BEPTEY	173.171	2.958	BEPWUS	153.764	3.087
BEQDUY	166.16	3.047	BEQFAH	155.054	3.131	BEQBIJ	153.014	3.391	BEQBIK	164.834	3.223	BEQDUY	165.956	3.087
BEQRUL	168.756	3.218	BERBIN	169.115	2.86	BEQLIT	173.417	3.038	BEQQUN	157.831	2.853	BEQRUL	159.66	3.217
BERDOU	162.012	2.827	BERDOU	151.116	3.084	BERDAH	153.226	2.823	BERDAH	166.598	3.238	BERDOU	167.094	2.911
BERDOU	163.968	2.968	BERDOU	167.286	2.956	BERDOU	156.257	2.636	BERDOU	152.799	2.796	BERDOU	153.176	2.901
BERDOU	161.025	3.069	BERDOU	159.075	3.153	BERDOU	169.569	3.245	BERDOU	159.04	3.089	BERDOU	162.051	3.182
BERDOU	150.622	3.252	BERDOU	158.501	3.089	BERDOU	150.905	3.333	BERDOU	155.05	3.19	BERDOU	155.454	3.069
BERDOU	172.499	3.162	BERDOU	156.564	3.064	BERDOU	158.329	3.423	BERDOU	155.093	2.984	BERDOU	155.669	3.454
BERGEN	156.678	2.848	BERGEN	171.836	3.469	BERDOU	172.904	3.018	BERGEN	171.128	3.386	BERGEN	152.285	3.397
BESGAI	157.759	3.176	BESGAI	161.906	2.974	BERGEN	169.734	2.848	BERXAY	153.456	3.47	BESGAI	155.483	3.378
BESGEM	151.631	2.74	BESGEM	156.272	3.342	BESGAI	156.442	3.193	BESGEM	151.986	2.89	BESGEM	155.353	2.79
BESSOJ01	161.531	2.734	BESSOJ01	152.158	2.929	BESGEM	154.597	2.933	BESSOJ01	167.906	3.026	BESSOJ01	151.808	2.815
BESZAB	155.54	3.016	BESZAB	164.459	3.016	BESYAB	165.821	3.241	BESZAB	158.802	2.848	BESZAB	152.766	3.023
BESZEF	150.061	3.151	BETJOZ	150.614	3.027	BESZAB	166.97	3.093	BESZAB	157.611	3.141	BESZAB	161.696	3.431
BEVCIP	151.788	3.419	BEVCIP	158.425	3.003	BETJOZ	151.44	2.967	BETJOZ	152.476	2.967	BETRAW	150.158	3.021
BEVVIK	162.142	2.924	BEVVOQ	165.608	3.475	BEVGUH	161.411	2.675	BEVVIK	158.538	3.246	BEVVIK	172.216	2.924
BEVWAE	154.119	2.968	BEWCIQ	156.996	3.267	BEVVOQ	157.939	3.113	BEVVUT	171.17	3.023	BEVVUT	154.651	3.155
BEWDOY	164.858	3.171	BEWHEQ	158.497	3.264	BEWCIQ	150.993	2.916	BEWDIS	166.216	3.154	BEWDOY	153.21	3.327
BEWHOC	153.775	3.272	BEWHOC	151.971	2.927	BEWHIW	172.719	2.793	BEWHIW	156.9	2.825	BEWHIW	158.37	2.953
BEWJEV	162.855	3.403	BEWRIF	162.269	2.747	BEWJAR	155.81	3.461	BEWJAR	163.197	3.406	BEWJEV	178.477	2.996
BEWYOR	175.264	2.976	BEWYOR	155.084	3.058	BEWROL	167.76	3.351	BEWTIH	154.421	2.892	BEWWUW	164.303	3.035
BEXCOZ	150.884	3.057	BEXDEQ	161.266	3.45	BEWZEJ	150.741	2.945	BEWZEJ	150.419	2.894	BEWZEJ	150.633	2.944
BEXHEU	165.9	3.031	BEXHIX	158.211	3.181	BEXGEU	150.899	3.323	BEXGEU	154.191	3.378	BEXHET	157.026	3.457
BEXHOD	162.282	3.029	BEXHOD	173.283	3.307	BEXHIX	169.579	2.98	BEXHIX	155.441	2.954	BEXHIX	161.271	2.926
BEXHUJ	165.879	2.879	BEXHUJ	156.612	3.335	BEXHOD	153.874	3.273	BEXHOD	159.692	2.687	BEXHUJ	154.729	2.985
BEXHUJ	154.8	3.301	BEXHUJ	164.934	3.07	BEXHUJ	160.214	2.824	BEXHUJ	156.294	3.007	BEXHUJ	160.178	2.824
BEXKEY	157.993	2.949	BEXPUP	165.988	3.157	BEXHUJ	165.98	2.875	BEXKEY	162.881	3.329	BEXKEY	168.993	3.253
BEXQIG	158.853	2.836	BEXQIG	159.775	3.159	BEXPUP	153.791	3.484	BEXPUP	159.544	2.939	BEXQIG	156.405	2.836
BEXQOM	152.976	3.168	BEXQUS	150.321	3.009	BEXQIG	173.255	3.319	BEXQIG	152.375	3.364	BEXQOM	153.918	3.082
BEXQUS	157.031	3.413	BEXQUS	163.916	3.157	BEXQUS	158.752	2.862	BEXQUS	172.051	3.472	BEXQUS	156.801	3.218
BEXRAZ	153.756	3.199	BEXRAZ	164.553	2.823	BEXQUS	159.888	3.399	BEXQUS	150.257	3.479	BEXRAZ	164.174	2.812
BEXRAZ	156.096	3.341	BEXWUA	151.45	3.013	BEXRAZ	167.377	2.998	BEXRAZ	155.521	2.974	BEXRAZ	161.479	3.122
BEXYUA	158.185	3.141	BEXYUA	172.779	3.177	BEXYUA	153.319	3.104	BEXYUA	162.52	3.459	BEXYUA	160.498	3.014
BEXZAH	159.862	3.304	BEYBEO	154.581	3.248	BEXYUA	168.911	2.963	BEXYUA	160.516	3.233	BEXZAH	168.473	3.043
BEYBUE	175.198	3.259	BEYDIS	160.064	3.197	BEYBEO	153.762	3.248	BEYBOW	157.457	3.039	BEYBOW	161.094	2.907
BEYDUH	151.549	2.878	BEYDUH	158.861	3.295	BEYDIS	171.517	2.741	BEYDIS	154.211	3.048	BEYDIS	156.327	3.147
BEYDUH	169.235	3.298	BEYDUH	171.989	3.192	BEYDUH	155.484	3.112	BEYDUH	168.137	3.014	BEYDUH	173.637	3.014
BEYFIU	171.686	3.183	BEYGAQ	160.835	3.364	BEYDUH	166.353	3.462	BEYFIU	159.505	3.416	BEYFIU	156.473	2.832
BEYQUV	163.002	2.734	BEYQUV	171.457	3.074	BEYHOF	151.403	2.92	BEYJAT	161.825	2.81	BEYQUV	155.116	3.074
BEZJOG	170.142	2.987	BEZJOG	162.784	2.955	BEYQUV	171.212	3.431	BEYQUV	158.819	3.079	BEYQUV	165.103	2.927
BEZPEE	167.019	3.38	BEZRUU	151.296	3.185	BEZJOG	163.169	3.421	BEZJUM	153.064	3.105	BEZKAT	150.717	2.873
BEZSIJ	154.718	3.255	BEZXOW	172.642	3.498	BEZSEH	165.252	3.472	BEZSIJ	153.501	3.241	BEZSIJ	163.824	3.163
BFACRH	170.386	3.293	BIBKUS	163.774	2.644	BEZXOW	165.583	3.325	BEZXOW	151.174	3.009	BEZXOW	155.446	3.029
BIBLON	169.951	3.46	BIBLON	173.208	3.356	BIBKUS	155.372	3.145	BIBKUS01	166.161	3.397	BIBKUS01	155.985	2.84
BIBPEH	154.599	3.168	BIBPEH	171.086	2.924	BIBLON	159.471	3.148	BIBMIL	157.033	3.26	BIBMIL	166.653	3.329
BICSUD	163.712	2.937	BICSUD	173.676	3.101	BIBRAF	167.881	3.397	BICKUV	150.591	2.865	BICSUD	170.882	3.101
BICTUD	161.376	3.029	BICVUE01	171.336	2.824	BICTIR	167.794	2.778	BICTOX	158.548	2.947	BICTUD	153.762	3.084
BIDDOI	160.378	2.762	BIDFEA	156.706	2.746	BICYIZ	170.318	2.977	BIDDOI	152.016	3.084	BIDDOI	150.936	2.933
BIDFEA	152.516	3.04	BIDFEA	153.534	3.106	BIDFEA	157.961	3.397	BIDFEA	153.009	3.353	BIDFEA	154.096	3.046
BIDFEA	150.516	3.025	BIDFIH	172.922	3.306	BIDFEA	153.786	3.444	BIDFEA	174.364	3.462	BIDFEA	158.913	3.428
BIDFON	165.441	2.898	BIDFUT	169.68	3.11	BIDFIH	177.62	3.064	BIDFIH	167.274	3.009	BIDFON	154.071	3.033
BIDGAA	176.514	3.163	BIDGAA	173.915	3.341	BIDFUT	153.924	3.157	BIDFUT	161.702	3.137	BIDGAA	164.662	3.005
BIDJEH	153.491	2.743	BIDMIO	170.65	3.215	BIDHAY	156.287	2.964	BIDJAD	168.092	3.451	BIDJEH	150.708	2.743
BIDMIO	151.727	2.837	BIDMIO	176.701	3.211	BIDMIO	165.778	3.115	BIDMIO	151.387	3.327	BIDMIO	154.648	2.835
BIDMOU	159.476	3.068	BIDMUA	169.101	3.176	BIDMOU	162.812	3.492	BIDMOU	151.253	3.15	BIDMOU	155.745	3.3
BIDSUD	163.449	3.441	BIDTOB	156.21	3.227	BIDMUA	175.364	2.924	BIDMUA	170.057	3.019	BIDRIS	151.488	3.153
BIDTOB	154.891	3.038	BIDTUH	155.141	3.037	BIDTOB	151.528	3.158	BIDTOB	152.241	2.922	BIDTOB	150.818	2.906
BIDTUH	151.653	3.155	BIDTUH	150.884	2.973	BIDTUH	153.113	3.095	BIDTUH	150.602	2.97	BIDTUH	154.913	2.802
BIDVAP	151.246	2.952	BIDVAP	154.522	2.828	BIDTUH	153.636	2.802	BIDVAP	154.835	3.029	BIDVAP	152.009	3.02
BIDVAP	150.774	3.145	BIDVAP	154.118	2.83	BIDVAP	150.981	3.137	BIDVAP	154.21	2.828	BIDVAP	150.774	2.915
BIDVOB	156.199	3.106	BIDVUH	157.748	3.236	BIDVAP	150.997	2.915	BIDVOB	173.474	2.845	BIDVOB	161.777	3.138
BIDZAT	160.662	3.025	BIFSOC	156.736	2.913	BIDVUH	157.065	3.269	BIDXIX	150.231	2.748	BIDXIX	151.207	3.111
BIFZEW	165.705	3.083	BIFZEW	166.631	3.343	BIFSOC	156.78	2.881	BIFZEW	168.608	3.129	BIFZEW	166.322	3.31
BIGXOF	160.468	3.048	BIGXOF	172.091	3.166	BIGXOF	167.83	3.13	BIGXOF	161.823	3.079	BIGXOF	154.162	2.987

BIGXUL	157.852	2.893	BIGXUL	176.792	3.093	BIGXUL	157.854	3.024	BIGXUL	151.267	3.198	BIGXUL	162.721	2.791
BIGZEX	178	3.105	BIGZEX	161.01	3.28	BIGXUL	159.531	3.349	BIGXUL	170.912	3.242	BIGZEX	151.385	3.12
BIGZEX	154.343	3.135	BIGZEX	154.973	3.16	BIGZEX	154.705	3.106	BIGZEX	155.346	3.05	BIGZEX	153.636	3.07
BIGZEX	159.025	2.885	BIGZEX	176.292	3.289	BIGZEX	160.02	3.13	BIGZEX	159.156	3.304	BIGZEX	157.764	3.464
BIGZEX	173.535	3.176	BIGZEX	161.006	3.322	BIGZEX	164.161	3.149	BIGZEX	166.273	2.921	BIGZEX	155.88	3.46
BIHCIG	158.233	3.427	BIHCIH	158.023	3.217	BIGZEX	157.245	3.147	BIHCAY	154.39	3.239	BIHCAY	156.864	3.296
BIHFIK	170.746	2.842	BIHFIK	153.815	3.156	BIHCOM	173.67	2.745	BIHCOM	157.994	2.745	BIHCOM	171.879	3.269
BIHFIK	161.957	3.131	BIHFUW	170.927	3.22	BIHFIK	162.57	3.198	BIHFIK	151.384	2.892	BIHFIK	164.105	3.472
BIHGOR	156.16	3.38	BIHJEJ	157.042	3.344	BIHFUW	163.148	2.995	BIHFUW	150.97	3.219	BIHFUW	156.957	3.39
BIJBOL	170.065	2.921	BIJDAZ	157.94	2.791	BIHJOV	153.688	2.687	BIHMUD	157.292	3.41	BIHMUD	162.296	3.123
BIJDAZ	151.937	3.036	BIJDEE	152.649	3.353	BIJDAZ	161.257	3.218	BIJDAZ	165.6	2.964	BIJDAZ	155.825	3.354
BIJHUA	164.025	2.927	BIJHUA	159.626	3.151	BIJGUX	151.565	3.253	BIJGUX	153.244	3.245	BIJGUX	161.881	3.023
BIJJOW	151.641	2.922	BIJJOW	159.306	2.977	BIJJEM	168.759	3.164	BIJJEM	161.46	2.772	BIJJEM	173.907	3.457
BIJJUC	158.965	3.004	BIJJUC	161.413	3.228	BIJJOW	164.794	2.84	BIJJUC	167.333	2.887	BIJJUC	153.333	3.379
BIJVOI	177.726	3.337	BIJVOI	169.858	2.957	BIJTEU	154.789	2.935	BIJVEY	156.061	3.018	BIJVEY	154.23	3.436
BIKCOO01	170.898	3.256	BIKSUK	163.905	3.203	BIKCOO01	177.179	2.883	BIKCOO01	169.143	3.247	BIKCOO01	163.422	3.05
BIKTAR	160.5	3.434	BIKTAR	158.334	2.791	BIKSUK	167.932	3.015	BIKTAR	151.193	3.467	BIKTAR	154.261	3.348
BIKWEY	177.384	3.157	BIKWEY	161.68	2.944	BIKTOE	150.558	3.402	BIKWEY	170.453	3.177	BIKWEY	163.512	3.422
BIKWEY	150.123	3.225	BIKWEY	162.379	2.698	BIKWEY	171.509	2.998	BIKWEY	150.054	2.822	BIKWEY	158.088	2.834
BILRET	170.508	3.43	BILRET	154.916	3.135	BIKWEY	161.563	3.382	BILRAP	151.282	3.492	BILRET	166.755	3.135
BIMFIN	150.999	3.309	BIMKUG	166.285	3.439	BIMCEF11	165.318	3.392	BIMCEF11	160.738	2.787	BIMCEF11	158.315	3.246
BINCAC	160.023	3.183	BINCAC	162.752	2.948	BIMKUG	163.827	3.148	BINCAC	163.151	3.166	BINCAC	166.556	2.987
BIPKUK	166.26	3.254	BIPPIZ	161.928	3.428	BINGIO	170.467	2.842	BINGUA	175.201	2.794	BIPKEQ	163.878	2.915
BIPZIL	173.702	3.271	BIPZOR	154.911	3.294	BIPRIF	151.763	3.061	BIPZIL	151.12	3.392	BIPZIL	156.869	3.464
BIQGEQ	171.507	3.066	BIQKUJ	150.631	2.988	BIPZOR	170.864	2.964	BIQGEQ	161.794	3.066	BIQGEQ	150.462	3.334
BIRCOX	165.208	2.864	BIRCOX	157.318	2.864	BIQKUJ	162.598	3.106	BIQXUW	159.792	3.335	BIQXUW	157.038	2.942
BIRLIZ	172.074	2.642	BIRLIZ	164.479	3.237	BIRCOX01	164.847	2.784	BIRCOX01	157.198	2.784	BIRLIZ	167.835	3.02
BISJIA	159.84	2.992	BISJIA	151.494	3.477	BIRLIZ	172.935	2.642	BIRVUV	159.593	2.933	BISHIW	154.164	2.893
BISMOJ	161.039	2.635	BISXEK	150.373	3.13	BISJIA	164.003	3.049	BISKAR	160.464	3.146	BISMOJ	159.962	2.635
BITYAI	156.706	3.124	BITYAI	155.34	2.989	BITCEM13	150.418	2.797	BITCEM13	158.231	3.155	BITSEF	152.365	2.88
BIVYAK	152.939	2.845	BIVYAK	150.104	2.959	BIVBUF	161.535	3.322	BIVCEP	154.191	2.904	BIVQAC	165.639	3.278
BIVYAK	175.134	3.015	BIVYAK	177.88	3.216	BIVYAK	154.134	3.491	BIVYAK	175.447	3.093	BIVYAK	163.915	2.885
BIXCEQ	150.843	3.353	BIXHAT	163.196	2.801	BIVYAK	155.305	3.438	BIWGEX	157.511	3.143	BIXCEQ	155.571	3.429
BIXHEX	164.76	3.119	BIXHEX	162.653	2.982	BIXHAT	163.582	2.948	BIXHAT	169.173	2.801	BIXHEX	167.161	2.982
BIXZIU	154.496	3.27	BIXZIU	167.097	3.167	BIXLAZ	151.938	3.234	BIXZIU	156.295	3.146	BIXZIU	165.908	2.886
BIYCUJ	163.217	3.497	BIYCUJ	154.238	3.268	BIXZIU	152.424	3.09	BIYBUK	157.244	2.998	BIYBUK	173.607	3.143
BIYDAQ	156.544	3.415	BIYDEU	158.226	3.054	BIYDAQ	159.541	3.177	BIYDAQ	154.112	3.236	BIYDAQ	150.591	3.415
BIYGAT	170.592	3.049	BIYGAT	174.366	3.489	BIYDIY	164.445	3.006	BIYDIY	156.984	3.133	BIYFEW	152.795	2.679
BIYHIE	164.434	2.979	BIYHIE	152.674	3.013	BIYGIB	171.113	3.217	BIYHIE	161.964	2.911	BIYHIE	154.153	3.002
BIYNOP	152.712	3.05	BIYNOP	153.922	3.029	BIYHIE	151.736	3.28	BIYHIE	159.185	2.982	BIYHIE	167.066	2.949
BIYPEI	160.984	3.389	BIYYIT	160.12	2.659	BIYPAA	174.674	3.23	BIYPAA	154.878	2.849	BIYPAE	161.839	3.209
BIZKIG	155.841	3.274	BIZKIG01	158.037	3.218	BIYYIT	165.289	3.093	BIYYIT	150.93	2.81	BIZKEA	158.854	3.014
BIZLUV	158.573	2.818	BIZLUV	166.456	3.347	BIZLAB	156.38	2.704	BIZLUV	159.162	2.911	BIZLUV	155.289	2.777
BIZWAM	168.301	3.405	BIZXAN	156.133	3.445	BIZLUV	155.049	3.289	BIZROS	155.794	3.344	BIZWAM	162.274	3.376
BOBKAF	162.016	3.082	BOBMIS	152.219	3.165	BOBGEG	175.006	3.378	BOBKAF	158.68	3.354	BOBKAF	155.369	3.166
BOCGOR	164.895	3.126	BOCGOR	167.46	2.858	BOBMIS	158.375	3.239	BOBMIS	155.82	3.487	BOBVAT	155.752	3.398
BOCKUA	155.753	3.162	BOCKUA	150.048	3.162	BOCHIL	169.567	2.904	BOCHOR	164.157	2.903	BOCHOR	152.863	3.395
BODFEG	171.944	2.884	BODFEG	163.313	2.964	BOCRIV	165.316	3.074	BOCRIW	154.424	3.29	BOCRIW	153.413	3.342
BODFNB	161.072	2.965	BODFNB	161.046	3.419	BODFNB	151.587	3.4	BODFNB	175.154	2.934	BODFNB	165.912	3.213
BODFOQ	161.693	3.075	BODFUW	172.07	2.875	BODFNB	150.807	3.213	BODFOQ	162.945	3.237	BODFOQ	170.44	3.237
BODJOW	175.683	3.239	BODJOW	152.897	3.135	BODFUW	163.35	2.965	BODGEH	152.594	2.83	BODJOW	157.441	3.168
BODLEO	156.029	3.004	BODLEO	163.065	3	BODJUA	160.931	2.729	BODLEN	164.14	3.451	BODLEO	171.496	2.811
BODLIS	155.705	3.21	BODLIS	165.25	3.451	BODLEO	169.017	3.22	BODLIS	174.375	3.117	BODLIS	161.489	3.371
BODNOX	164.368	2.933	BODNOX	162.752	2.676	BODLIS	150.358	3.085	BODLIS	151.719	3.341	BODMOA	164.843	3.349
BODNOX	159.407	2.676	BODSET	160.303	3.292	BODNOX	174.778	3.093	BODNOX	170.32	3.453	BODNOX	180	3.223
BODTIZ	165.175	3.454	BODWUP	160.619	2.879	BODSET	159.254	3.391	BODSOD	150.211	3.069	BODTIY	161.617	3.044
BODWUP	157.077	2.918	BODWUP	151.269	2.709	BODWUP	163.348	2.761	BODWUP	158.791	3.431	BODWUP	153.213	3.455
BODWUP	155.564	3.32	BODYEY	165.98	3.159	BODWUP	161.952	3.071	BODWUP	157.331	2.826	BODWUP	156.564	2.918
BOFJIR	169.847	3.311	BOFJIR	163.694	2.795	BODYEY1	165.98	3.159	BOFCOS	155.928	3.118	BOFCOS	155.948	2.898
BOFJIR	176.697	3.11	BOFJIR	166.996	3.387	BOFJIR	157.275	3.213	BOFJIR	162.538	3.42	BOFJIR	171.423	2.994
BOFPIY	171.79	3.326	BOFWOI	155.667	3.106	BOFJIR	164.435	2.798	BOFJIR	168.699	3.119	BOFLIU	163.279	3.2
BOFZOP	155.346	3.158	BOFZOP	160.213	3.173	BOFZOP	159.984	3.048	BOFZOP	151.876	3.441	BOFZOP	163.633	3.077
BOGBOS	160.752	3.487	BOGBOS	170.501	3.306	BOFZOP	151.249	3.47	BOGBOS	167.104	2.887	BOGBOS	156.405	3.257
BOGCEJ	165.347	2.723	BOGCEJ	155.547	3.158	BOGBOS	163.803	2.62	BOGCEJ	161.537	3.361	BOGCEJ	164.178	3.465
BOGCEJ	151.142	3.061	BOGCEJ	171.196	3.178	BOGCEJ	162.498	3.109	BOGCEJ	153.878	2.981	BOGCEJ	159.632	3.158
BOGCOT	156.951	3.156	BOGHAI	151.981	3.227	BOGCOT	152.662	3.24	BOGCOT	151.973	3.1	BOGCOT	155.214	3.401
BOGJAM	160.836	3.004	BOGJAM	158.982	3.261	BOGHUC	165.114	2.886	BOGHUC	171.009	3.181	BOGJAM	172.432	3.302
BOGKUF	151.827	2.805	BOGSID	162.896	3.417	BOGJAM	158.994	2.91	BOGJAM	164.518	3.446	BOGJAM	174.446	3.465
BOGYUV	165.896	3.221	BOGYUV	154.97	3.221	BOGSID	159.534	3.498	BOGVIC	160.477	3.215	BOGYEF	152.348	3.039
BOHMUH	157.216	3.038	BOHMUH	151.366	2.98	BOHDOR	150.371	3.471	BOHHIR	155.775	3.494	BOHMUH	164.201	3.354
BOHWIE	162.149	3.33	BOHWIE	160.142	3.355	BOHTEA	152.09	3.001	BOHTEA	159.435	3.099	BOHWED	173.51	2.805
BOJWII	154.712	3.25	BOJWII	167.662	2.813	BOHWIE	163.182	3.33	BOJDIR	162.047	3.098	BOJWAC	167.139	3.275
BOJWII	158.695	2.739	BOJWII	171.829	3.083	BOJWII	159.542	2.987	BOJWII	172.967	2.999	BOJWII	153.474	3.05
BOJWOO	161.054	2.8	BOJWOO	158.161	3.221	BOJWII	168.375	2.757	BOJWII	169.343	3.498	BOJWII	151.345	3.263
BOJWOO	169.715	3.13	BOKCEK	158.594	3.495	BOJWOO	152.023	3.406	BOJWOO	160.389	2.805	BOJWOO	169.148	2.849
BOKHOY	150.925	3.17	BOKHOY	161.252	2.783	BOKDOV	159.959	3.32	BOKFAI10	168.492	3.421	BOKHOY	160.895	3.314
BOKJUK	160.762	3.287	BOKPUN	170.948	3.372	BOKJUK	153.54	2.795	BOKJUK	156.817	2.975	BOKJUK	167.839	2.934
BOKQUR	156.125	2.995	BOKQUR	167.233	2.994	BOKPUN	156.924	3.314	BOKQOJ	168.163	3.059	BOKQOJ	167.661	3.041
BOKREC	153.298	3.336	BOKTUS	152.293	2.979	BOKQUR	173.004	2.955	BOKRAY	150.896	2.798	BOKREC	156.147	3.339

BOLKEV	169.215	2.941	BOLKOF	154.781	2.864	BOKTUS	162.122	3.324	BOKWOO	151.116	3.408	BOKWUW	160.224	3.331
BOLNAR	152.029	2.912	BOLNAR	165.958	2.728	BOLKOF	156.895	2.834	BOLMAR	176.721	3.077	BOLNAR	160.628	3.168
BOLQAV	165.341	2.871	BOLYAG	153.208	2.879	BOLNUO	163.692	2.793	BOLQAV	155.717	3.176	BOLQAV	171.665	3.224
BOLZIP	160.024	3.298	BOLZIP	159.764	3.492	BOLYAG	153.699	2.737	BOLZEL	166.609	3.355	BOLZIP	168.467	3.038
BOMHOE	152.52	3.198	BOMHUK	163.32	3.223	BOLZIP	166.59	2.993	BOMGIT	150.242	3.197	BOMGIT	158.498	3.443
BOMJAS	153.423	3.431	BOMKIX	154.513	3.317	BOMJAS	165.836	3.431	BOMJAS	163.133	3.248	BOMJAS	161.732	3.026
BOMLUO	154.622	2.955	BOMLUO	155.107	3	BOMKIX	159.34	2.9	BOMKIX	161.377	2.9	BOMLUO	152.6	2.935
BOMPOM	168.513	2.798	BOMPOM	169.17	3.38	BOMLUO	153.862	2.923	BOMNEZ	152.246	2.882	BOMPOM	155.077	2.847
BOMVEG	157.357	3.443	BOMVEG	175.389	2.959	BOMQON	172.977	3.096	BOMQON	156.838	2.774	BOMVEG	172.06	3.104
BOMVEG	175.121	2.988	BOMVOQ	165.807	2.765	BOMVEG	153.884	2.819	BOMVEG	158.037	2.801	BOMVEG	169.486	2.919
BOMWAD	150.478	3.05	BOMWAD	151.973	2.978	BOMVUW	171.744	3.022	BOMVUW	156.682	3.315	BOMVUW	172.415	2.989
BOMWAD	151.811	3.32	BOMWEH	152.85	2.928	BOMWAD	150.563	3.128	BOMWAD	154.213	2.883	BOMWAD	152.447	2.972
BOMWOR	165.57	2.996	BOMWUX	153.684	3.408	BOMWEH	151.903	3.271	BOMWEH	158.531	3.273	BOMWEH	155.218	3.028
BOMYAF	170.938	3.047	BONHES	165.396	3.235	BOMWUX	168.819	3.426	BOMYAF	150.525	2.986	BOMYAF	156.884	2.986
BOPHUM	159.991	3.386	BOPRUT1	152.132	3.387	BONHES	160.527	3.213	BONPEC	153.004	3.429	BONWAE	163.198	3.074
BOPRUT1	160.445	3.387	BOPRUT1	158.107	2.985	BOPRUT1	152.887	3.24	BOPRUT1	164.64	2.985	BOPRUT1	177.15	3.379
BOQFOF	157.372	3.063	BOQFOF	163.617	3.38	BOPRUT1	178.107	2.871	BOPRUT1	167.909	3.461	BOPVEL	171.032	3.384
BOQHUM	162.371	2.749	BOQHUM	161.591	3.356	BOQHEW	154.294	3.448	BOQHOG	161.693	3.284	BOQHOG	155.099	3.284
BOQTUA	154.241	3.311	BOQTUA	153.141	2.488	BOQPAB	151.191	3.151	BOQRIL	160.299	3.125	BOQRIL	171.141	2.752
BOQXAH	174.5	2.736	BOQXAH	169.799	2.972	BOQVAI	156.64	3.318	BOQXAH	172.035	2.972	BOQXAH	158.823	2.736
BOQZAK	161.583	3.059	BOQZAK	159.067	3.19	BOQXAH	163.314	3.356	BOQYOZ	150.716	2.809	BOQZAK	153.073	3.273
BOQZUG	165.667	2.719	BOQZUG	170.211	2.804	BOQZAK	151.184	2.665	BOQZAK	166.721	3.19	BOQZAK	152.922	2.982
BORBOD	162.14	3.23	BORBOD	166.648	3.432	BORBAP	172.628	3.062	BORBIX	169.328	3.113	BORBOD	161.672	3.089
BORBOD	166.476	2.787	BORBOD	167.359	2.817	BORBOD	153.17	3.152	BORBOD	163.287	3.226	BORBOD	163.604	3.107
BORCAQ	154.786	3.399	BORCAQ	161.758	2.874	BORBOD	153.331	3.071	BORBUJ	164.563	3.025	BORBUJ	150.384	3.448
BORGEY	150.715	3.215	BORGEY	168.506	2.832	BORFAR	166.818	3.274	BORFUN	151.694	3.494	BORFUN	156.894	2.769
BORGEY	160.142	3.248	BORGEY	156.297	2.891	BORGEY	150.146	3.479	BORGEY	160.041	2.793	BORGEY	154.95	3.07
BORNAZ	160.605	3.003	BORNAZ	163.577	3.132	BORGEY	163.594	3.383	BORGEY	151.135	2.924	BORNAZ	156.68	2.829
BORQUW	154.655	2.984	BORQUW	153.943	3.466	BORNAZ	170.884	2.996	BORNAZ	178.004	3.121	BORQUW	176.216	3.403
BORTUB	169.278	3.117	BORXAL	155.124	2.845	BORSIM	166.635	3.465	BORSIM	161.217	3.175	BORSUY	163.459	3.328
BOSFIA	154.06	3.439	BOSFIA01	163.623	2.879	BOSFIA	152.54	2.854	BOSFIA	164.535	2.817	BOSFIA	151.678	2.926
BOSLON	162.745	3.328	BOSLON	172.367	3.466	BOSFIA01	153.238	2.96	BOSFIA01	153.026	3.459	BOSLON	161.679	3.447
BOSVIR	166.044	2.875	BOSVIR	150.488	3.403	BOSVIR	153.861	3.37	BOSVIR	169.02	3.457	BOSVIR	153.274	2.973
BOSVIR	174.89	3.182	BOSVIR	165.215	3.303	BOSVIR	169.89	2.903	BOSVIR	159.65	3.419	BOSVIR	160.298	3.182
BOTCOE	150.056	2.833	BOTCOE	162.71	2.74	BOSVIR	159.467	2.907	BOSVIR	158.566	3.111	BOSVIR	166.772	2.973
BOTDUM	160.057	3.216	BOTFAV	169.44	3.49	BOTCOE	167.162	2.941	BOTCOE	156.056	3.187	BOTCOE	161.5	3.224
BOTQEH	155.996	3.065	BOTQEH	152.39	3.147	BOTFAV	163.589	2.818	BOTKOL	162.173	3.095	BOTLIG	167.378	2.994
BOTXUD0	166.187	3.494	BOTZUH	155.612	3.205	BOTQEH	175.037	2.978	BOTQIL	175.143	3.05	BOTXUD0	151.586	3.409
BOTZUI	156.852	3.214	BOTZUI	154.425	3.442	BOTZUI	158.072	3.378	BOTZUI	174.023	2.783	BOTZUI	164.818	3.26
BOVCEV0	162.395	2.834	BOVCEV0	152.367	2.83	BOTZUI	160.281	2.882	BOTZUI	173.893	2.934	BOVCEV0	162.949	3.159
BOVCEV0	152.157	3.019	BOVCEV0	157.39	2.713	BOVCEV0	173.109	2.787	BOVCEV0	155.963	3.209	BOVCEV0	165.902	3.036
BOVCEV0	151.65	3.138	BOVCOH	160.69	3.152	BOVCEV0	160.286	2.713	BOVCEV0	152.569	3.014	BOVCEV0	168.712	2.936
BOVCOH	163.346	3.347	BOVCOH	150.278	3.035	BOVCOH	159.542	2.655	BOVCOH	162.034	3.29	BOVCOH	152.147	2.856
BOVCUN	171.683	2.912	BOVCUN	175.376	3.433	BOVCOH	158.381	3.487	BOVCOH	164.406	3.302	BOVCOH	155.536	3.005
BOVFAV	162.73	2.919	BOVFAV	164.263	3.127	BOVCUN	152.492	2.753	BOVDUM0	162.644	3.164	BOVFAV	167.156	3.184
BOVFAV	163.5	2.809	BOVFAV	161.721	3.199	BOVFAV	151.513	3.407	BOVFAV	161.43	3.3	BOVFAV	166.452	2.809
BOVWOA	171.5	2.951	BOVWUG	166.321	3.21	BOVFAV	169.47	3.294	BOVWEP	152.212	3.233	BOVWEP	168.319	2.935
BOWCAT	157.213	3.381	BOWKUV	152.078	3.372	BOVXOB	152.485	3.2	BOVYAO	151.782	3.283	BOVZAP	162.866	3.396
BOXGEE	171.169	3.177	BOXKAC	158.072	2.961	BOWKUV	165.262	3.372	BOXGEE	156.326	2.894	BOXGEE	170.76	3.177
BOXPIQ	150.973	3.474	BOXPIQ	155.464	3.402	BOXKAC	172.415	3.418	BOXPIQ	162.579	3.17	BOXPIQ	179.653	3.369
BOXRIT	160.483	2.812	BOXRIT	150.891	2.985	BOXRIQ	156.718	2.827	BOXRIQ	166.345	3.179	BOXRIQ	154.747	3.412
BOXROZ	160.757	3.314	BOXROZ	150.538	3.377	BOXROZ	161.275	3.017	BOXROZ	150.816	3.215	BOXROZ	177.103	3.031
BOXROZ	170.575	3.38	BOXTEP	152.217	3.288	BOXROZ	156.029	3.157	BOXROZ	157.828	3.083	BOXROZ	157.742	2.955
BOYZUM	168.081	2.977	BOYZUM	156.869	3.104	BOXTEP	152.918	2.958	BOXZOG	151.098	3.303	BOYQAJ	154.182	3.119
BOZWOF	167.794	2.963	BOZWOF	169.142	3.274	BOZVES	158.621	3.188	BOZVES	154.278	2.858	BOZVIW	174.911	3.433
BPPFBP0	155.71	3.089	BTFARH0	174.563	2.872	BPPFBP	157.362	3.102	BPPFBP	152.337	3.27	BPPFBP0	152.599	3.2
BUBFIR	165.746	3.205	BUBFIR	173.549	3.197	BTFARH0	155.814	2.905	BTFPBU	163.644	3.133	BTFPBU	151.511	3.133
BUBGIS	150.794	3.377	BUBGUE	150.382	3.369	BUBFIR	155.078	3.463	BUBFIR	163.4	2.844	BUBFIR	150.284	3.178
BUBKAN	165.412	3.397	BUBKAN	156.248	3.044	BUBKAN	154.633	2.966	BUBKAN	157.622	3.384	BUBKAN	156.01	2.753
BUBKAN	160.522	3.227	BUCCIP	158.428	3.431	BUBKAN	172.048	3.226	BUBKAN	159.735	3.204	BUBKAN	154.489	3.044
BUCJAM	158.642	2.871	BUCJER	167.779	3.195	BUCGUD	170.604	2.934	BUCHAK	154.057	3.019	BUCHEO	156.177	2.968
BUCKIV	167.85	2.873	BUCKOB	151.321	3.042	BUCJOA	157.152	3.327	BUCKAN	167.145	3.334	BUCKIV	152.552	3.071
BUCMOD	159.352	3.073	BUCMUJ	164.417	3.177	BUCLES	154.888	3.046	BUCLUI	169.075	3.245	BUCMET	173.799	2.968
BUCWEE	152.581	3.308	BUCWEE	154.182	3.17	BUCNIY	162.797	2.708	BUCWAA	164.973	3.365	BUCWAA	162.304	2.721
BUCWEE	153.116	2.92	BUCXIJ	168.638	3.257	BUCWEE	156.627	3.446	BUCWEE	152.515	2.886	BUCWEE	161.613	3.025
BUDBAH	166.762	3.485	BUDBAH	151.453	2.997	BUCXIJ	170.132	3.103	BUCXIJ	165.224	3.286	BUCXIJ	160.251	3.437
BUDREA	152.595	3.47	BUDRIF	166.163	3.171	BUDDUD	166.838	2.912	BUDNIZ	164.837	2.717	BUDNIZ	154.349	3.418
BUDSAX	156.922	3.002	BUFCEO	158.206	3.336	BUDRIF	158.482	3.298	BUDRIF	160.682	3.353	BUDRIF	155.876	3.494
BUFSEC	175.33	2.834	BUFSII	172.758	3.303	BUFSAY	159.933	3.224	BUFSAY	168.8	2.948	BUFSEC	163.767	3.229
BUFSII	157.977	3.2	BUFSII	158.18	3.22	BUFSII	176.534	3.189	BUFSII	156.384	3.055	BUFSII	167.103	3.189
BUGDUG	171.834	3.207	BUGFAO	169.487	3.188	BUFSII	159.765	3.113	BUFSII	165.41	3.453	BUFSII	171.438	3.32
BUGHIW	150.004	3.407	BUGHIW	159.071	2.991	BUGHES	168.079	3.139	BUGHES	159.597	2.993	BUGHIW	168.233	3.144
BUGQOJ	173.836	2.915	BUGROL	155.476	2.891	BUGQIE	159.481	2.943	BUGQOJ	163.788	3.471	BUGQOJ	165.578	3.35
BUHPOK	151.256	3.45	BUHTIL	168.397	2.711	BUGROL	157.681	2.891	BUGTEE	151.371	2.911	BUHBOZ	164.337	3.016
BUHVOR	166.646	3.181	BUHXAE	166.759	3.145	BUHVEH	175.201	3.249	BUHVIL	157.099	3.063	BUHVIL	164.827	3.374
BUHYOW	150.732	3.131	BUHZEL	152.004	3.213	BUHYOW	155.782	3.411	BUHYOW	155.548	3.4	BUHYOW	152.839	3.076
BUJMIG	155.685	3.492	BUKBIU	158.848	2.982	BUJLIC	153.703	3.315	BUJLIC	153.747	3.374	BUJMEC	160.798	3.283
BUKNAZ	161.854	3.182	BUKNED	152.212	3.432	BUKFIY	161.891	3.396	BUKNAZ	162.41	3.14	BUKNAZ	150.632	3.184
BUKQEF	174.368	3.163	BUKQEF	159.422	2.959	BUKNED	154.139	3.195	BUKNIH	168.539	2.959	BUKNUT	160.048	3.322

BUKQOP	165.518	3.281	BUKQOP	161.731	3.068	BUKQEF	157.376	3.18	BUKQEF	155.268	3.163	BUKQEF	164.375	2.76
BUKQOP	154.31	3.076	BUKQOP	169.052	2.837	BUKQOP	156.074	3.411	BUKQOP	160.021	3.068	BUKQOP	153.567	3.001
BUKSAE	152.557	3.294	BUKSAE	153.862	3.091	BUKRUV	173.074	2.747	BUKSAE	161.133	2.717	BUKSAE	150.305	2.879
BULJOK	155.005	3.386	BULNAA	171.731	3.439	BUKSAE	163.525	3.091	BUKTEH	151.882	3.053	BUKTIL	161.645	3.028
BULVAH	154.359	2.976	BULVEM	154.02	3.058	BULNAA	155.532	3.202	BULNAA	158.053	2.998	BULNAA	156.564	3.075
BULWAH	171.97	3.378	BULZIS	169.833	3.233	BULVIQ	152.711	3.037	BULVOU	166.556	2.787	BULVOU	151.817	3.358
BUNDUN0	163.252	3.188	BUNDUN0	166.464	3.364	BUNBUK	158.772	3.257	BUNBUK	153.119	3.404	BUNDUN0	160.011	3.123
BUNGEY	152.438	2.604	BUNHOJ	156.46	3.183	BUNDUN0	177.09	3.136	BUNDUN0	164.254	3.406	BUNFAV	156.359	2.96
BUNQOT	163.246	3.117	BUNVUC	167.555	2.773	BUNJAX	153.687	2.796	BUNLAA	167.104	3.303	BUNQAF	153.034	3.467
BUNVUC	153.533	2.998	BUNVUC	161.397	3.294	BUNVUC	150.046	3.294	BUNVUC	165.09	2.966	BUNVUC	165.208	2.872
BUNWUD	163.426	2.891	BUNWUD	163.594	2.944	BUNVUC	171.708	2.792	BUNWEN	159.336	3.167	BUNWEN	174.793	2.981
BUNWUD	151.583	2.973	BUNWUD	159.881	3.474	BUNWUD	152.905	2.956	BUNWUD	163.525	3.458	BUNWUD	158.884	3.468
BUNWUD(	151.691	2.957	BUNWUD(	163.163	2.882	BUNWUD(	163.59	2.921	BUNWUD(	158.76	3.453	BUNWUD(	159.571	3.462
BUNXAK	170.845	2.838	BUNXAK	167.423	3.45	BUNWUD(	163.363	3.424	BUNWUD(	152.946	2.947	BUNXAK	159.969	3.206
BUNXAK	159.503	3.055	BUNXEO	158.613	3.424	BUNXAK	152.805	3.386	BUNXAK	159.794	3.325	BUNXAK	170.321	3.388
BUNXEO	157.799	3.142	BUNXEO	163.084	2.996	BUNXEO	154.127	3.144	BUNXEO	164.788	3.178	BUNXEO	150.442	3.178
BUQDEA	154.72	3.296	BUQGUQ(	156.912	3.445	BUPBIB	156.488	3.497	BUPHON	159.515	3.143	BUQDEA	159.769	2.871
BUQLUX	150.905	2.961	BUQLUX	172.588	3.133	BUQGUQ(	160.497	3.239	BUQLOR	166.222	3.317	BUQLOR	160.283	3.473
BUQMAE	150.619	2.659	BUQMAE	155.014	3.348	BUQMAE	156.954	3.137	BUQMAE	152.994	2.659	BUQMAE	161.807	3.109
BUQTUD	159.306	2.931	BUQZIY	151.75	3.163	BUQTIR	166.246	3.357	BUQTIR	155.452	2.867	BUQTUD	158.326	2.993
BURFON	154.415	3.482	BURFON	154.743	3.052	BUQZIY	157.158	3.119	BUQZIY	150.065	3.021	BURFED	168.017	3.208
BURHON	167.818	3.255	BURHON	150.916	3.23	BURFON	153.602	3.429	BURFON	169.2	2.937	BURFUT	166.204	2.924
BURHON	159.637	3.138	BURHON	160.256	3.441	BURHON	174.129	3.292	BURHON	164.731	3.225	BURHON	175.761	3.353
BURRUF	157.235	3.422	BURRUF	155.74	3.384	BURNIO	175.343	3.02	BURRUD	157.175	3.465	BURRUD	168.482	3.426
BURRUF	167.3	2.944	BURRUF	150.615	2.921	BURRUF	150.419	3.121	BURRUF	152.845	3.036	BURRUF	164.761	2.992
BURVAM	150.459	2.884	BURVAM	158.574	2.872	BURRUF	159.331	3.18	BURSIU	164.794	2.827	BURSIU	161.544	2.851
BURVEQ	158.586	3.049	BURVEQ	163.771	2.748	BURVAM	160.54	2.872	BURVAM	171.913	3.479	BURVEQ	166.739	3.035
BURWAO	161.214	3.044	BURWAP	157.516	2.919	BURVEQ	162.534	3.339	BURVEQ	164.292	2.65	BURVEQ	157.897	3.401
BUSVOA	156.05	3.152	BUSYEW	166.91	3.136	BUSRIT	152.354	3.009	BUSSIR	175.573	3.241	BUSVOA	164.386	3.221
BUTBUO	159.261	3.437	BUTCAV	170.258	3.472	BUSYEW	160.319	3.463	BUTBUO	165.34	3.202	BUTBUO	156.298	3.314
BUTCAV	173.161	3.489	BUTCAV	173.048	3.21	BUTCAV	152.327	3.419	BUTCAV	154.803	3.255	BUTCAV	156.986	3.419
BUTKAE	160.151	3.286	BUTKAE	150.738	2.975	BUTCAV	161.389	3.234	BUTFUR	167.398	3.02	BUTKAE	161.263	3.376
BUTVAP	167.054	2.793	BUVFEH	155.617	3.121	BUTKAE	153.397	3.332	BUTSUF	153.577	3.12	BUTVAP	153.836	3.124
BUVQOB	160.504	3.46	BUVQOB	153.874	3.245	BUVLUC	162.338	2.856	BUVQOB	151.476	3.059	BUVQOB	151.977	2.935
BUVVIX	153.787	3.048	BUVVIX01	159.74	3.252	BUVTIX	171.257	3.087	BUVTIX	156.435	3.384	BUVVIX	159.773	3.248
BUVVOD	156.218	3.012	BUVVOD0	154.245	3.31	BUVVIX01	153.845	3.058	BUVVOD	153.994	3.295	BUVVOD	152.566	3.016
BUVVOD0	156.989	3.158	BUVVOD0	153.997	2.932	BUVVOD0	151.966	3.032	BUVVOD0	156.817	3.015	BUVVOD0	153.845	2.931
BUVVUJ	160.388	2.916	BUVVUJ	156.167	3.13	BUVVOD0	157.159	3.154	BUVVOD0	153.302	2.91	BUVVOD0	157.563	3.137
BUVWEU(	154.217	3.033	BUVWIY01	158.499	3.251	BUVWAQ	159.058	2.964	BUVWAQ	157.64	3.07	BUVWEU(	159.085	3.243
BUVWOE(	157.609	3.239	BUVWOE(	155.925	3.019	BUVWIY01	154.628	3.04	BUVWOE(	157.42	3.254	BUVWOE(	155.692	3.032
BUVWOE(	153.359	2.972	BUVWOE(	152.931	2.946	BUVWOE(	157.107	3.255	BUVWOE(	154.997	3.031	BUVWOE(	158.5	3.175
BUVWUK(	155.522	3.011	BUVWUK(	157.57	3.154	BUVWOE(	159.208	3.14	BUVWUK(	155.335	3.27	BUVWUK(	150.75	3.046
BUVXAR1	156.041	3.007	BUVXAR1	153.513	2.928	BUVWUK(	153.624	2.934	BUVXAR1	155.42	3.278	BUVXAR1	151.739	3.041
BUVXEV0	157.621	3.015	BUVXEV0	153.592	3.007	BUVXAR1	157.612	3.145	BUVXEV0	153.957	3.337	BUVXEV0	154.227	3.003
BUVXEV0	156.467	3.157	BUVXIZ01	152.343	3.387	BUVXEV0	157.75	3.022	BUVXEV0	152.749	3.337	BUVXEV0	154.38	2.919
BUVXOF0	155.766	2.979	BUVXOF0	158.275	3.032	BUVXIZ01	156.737	2.984	BUVXIZ01	157.254	3.037	BUVXOF0	152.568	3.356
BUWDIH	163.941	2.722	BUWDOQ	156.98	3.225	BUVYEW(	161.627	2.918	BUVYEW(	156.62	3.107	BUWDAZ	151.177	3.229
BUWGIN	164.713	3.205	BUWGIN	170.871	2.567	BUWDOQ	161.81	3.05	BUWFOR	152.123	3.401	BUWGIN	168.016	2.778
BUWGIN0	168.136	2.962	BUWGIN0	158.134	2.888	BUWGIN	176.828	2.567	BUWGIN	163.194	3.066	BUWGIN	155.912	2.759
BUWGUZ	161.248	3.15	BUWGUZ	159.042	3.292	BUWGIN0	165.72	3.114	BUWGUZ	166.191	2.946	BUWGUZ	167.118	3.198
BUWHEK	156.905	2.854	BUWHUA	167.817	2.854	BUWGUZ	150.209	3.141	BUWHEK	161.593	2.919	BUWHEK	165.378	3.096
BUWJEM	171.063	2.938	BUWLIR	154.457	2.702	BUWHUA	164.493	2.879	BUWJAI	159.079	3.075	BUWJAI	159.856	3.079
BUWLOX	158.766	3.484	BUWREU	159.693	3.231	BUWLIR	155.292	2.763	BUWLOX	154.013	3.027	BUWLOX	158.591	2.919
BUXRUL	152.489	3.093	BUXTEV	168.648	2.887	BUWSAR	175.791	3.279	BUWXIC	156.901	3.234	BUXBUT	176.91	3.109
BUXTUM	164.15	3.262	BUXVAU	161.234	2.967	BUXTUM	153.549	3.357	BUXTUM	152.539	3.218	BUXTUM	166.772	3.177
BUXVAU	154.607	2.967	BUXVIA	156.692	2.948	BUXVAU	163.391	3.376	BUXVAU	161.06	3.38	BUXVAU	153.863	2.952
BUXXAV	161.523	3.111	BUXXAV	157.124	3.425	BUXVIA	153.648	2.968	BUXVIA	151.382	3.334	BUXVIB	157.295	3.116
BUXXEA	151.128	2.888	BUXXEA	169.548	3.108	BUXXAW	152.167	2.932	BUXXAW	170.355	3.147	BUXXAW	153.515	2.932
BUYHAG	163.497	2.932	BUYHUZ0	153.962	3.015	BUXXEA	152.558	2.887	BUYHAG	154.964	3.459	BUYHAG	173.187	2.932
BUYJAH	151.189	2.944	BUYJAH	161.266	2.771	BUYHUZ0	151.712	3.109	BUYHUZ0	161.205	3.023	BUYHUZ0	160.717	2.947
BUYJAH02	152.803	3.042	BUYJAH02	155.544	2.909	BUYJAH	153.649	2.992	BUYJAH	151.04	2.984	BUYJAH	158.728	2.771
BUYJAH03	150.51	2.996	BUYJAH03	163.307	2.798	BUYJAH02	166.708	3.206	BUYJAH02	154.463	3.018	BUYJAH02	154.081	3.053
BUYJAH03	160.733	2.798	BUYJEL	171.796	3.038	BUYJAH03	150.611	3.311	BUYJAH03	151.593	3.044	BUYJAH03	153.155	2.991
BUZBOP	159.683	3.271	BUZBOP	152.033	3.271	BUYJEL	152.737	2.636	BUYJEL	171.509	3.142	BUYJEL	151.535	2.636
BUZCOS	152.133	2.995	BUZDAF	178.706	2.93	BUZCAC	150.731	2.891	BUZCAC	158.113	3.049	BUZCOS	171.832	2.78
BUZDAF	157.241	3.343	BUZDAF	166.278	3.119	BUZDAF	170.816	2.866	BUZDAF	162.25	2.93	BUZDAF	151.864	2.838
BUZDAF01	160.04	2.837	BUZDAF01	160.466	2.67	BUZDAF	153.149	3.174	BUZDAF	170.268	2.69	BUZDAF01	171.028	3.092
BUZDAF01	160.94	2.816	BUZDAF01	169.1	3.18	BUZDAF01	172.385	3.179	BUZDAF01	166.644	2.866	BUZDAF01	161.722	3.494
BUZDEJ	164.489	2.947	BUZDEJ	165.55	2.888	BUZDEJ	160.701	3.01	BUZDEJ	163.374	2.988	BUZDEJ	156.279	3.331
BUZDOT	162.949	2.854	BUZDOT	166.741	3.2	BUZDEJ	167.805	2.858	BUZDEJ	164.875	3.147	BUZDOT	169.627	2.703
BUZFAH	175.41	2.788	BUZFAH	158.274	3.203	BUZDOT	161.391	3.048	BUZDOT	152.86	2.992	BUZFAH	155.084	2.717
BUZFAH	156.379	3.116	BUZFAH	166.141	2.788	BUZFAH	151.103	2.568	BUZFAH	178.035	3.052	BUZFAH	153.721	2.568
BUZFAH	167.719	2.823	BUZFAH	161.029	2.823	BUZFAH	159.451	2.902	BUZFAH	152.868	2.717	BUZFAH	165.105	3.052
BUZFEL	150.462	3.429	BUZFEL	165.868	2.946	BUZFEL	158.776	2.655	BUZFEL	166.121	3.429	BUZFEL	155.448	2.818
BUZFEL	168.558	2.595	BUZFEL	151.284	2.88	BUZFEL	161.152	2.655	BUZFEL	150.593	3.443	BUZFEL	161.184	3.234
CABHOF	172.298	3.322	CABHOF	150.143	3.367	BUZFEL	170.162	2.812	BUZFEL	162.884	3.136	CABBOX	166.07	3.105
CABKEZ	151.997	3.195	CABKID	154.757	3.124	CABJUK	159.477	3.264	CABKEZ	174.591	3.127	CABKEZ	170.965	3.234
CABSUX	151.706	3.405	CABSUX	162.908	2.923	CABLEB	164.927	3.348	CABLEB	166.325	3.287	CABSOR	156.1	3.011
CACKOJ0	167.322	2.87	CACKOJ0	168.625	2.863	CACKOJ	170.658	3.055	CACKOJ	161.072	3.427	CACKOJ	166.639	3.082

CACNON	155.555	3.04	CACNON	151.461	3.21	CACNED	158.736	3.175	CACNED	169.024	3.02	CACNON	163.724	3.035
CACNON	152.17	3.154	CACNON	156.828	2.803	CACNON	156.249	2.783	CACNON	152.913	2.857	CACNON	174.716	3.31
CACNUT	168.737	3.438	CACNUT	156.085	3.414	CACNON	150.089	2.783	CACNUT	169.239	2.655	CACNUT	151.291	3.45
CACNUT	164.565	2.952	CACPIJ	166.54	2.92	CACNUT	152.753	2.88	CACNUT	153.618	3.228	CACNUT	153.485	3.019
CACPIJ	159.452	3.251	CACPIJ	153.696	3.061	CACPIJ	155.334	3.178	CACPIJ	152.684	3.293	CACPIJ	154.235	3.131
CACQAC	165.423	2.929	CACRAD	156.725	2.689	CACPIJ	173.933	2.626	CACPIJ	169.206	3.071	CACQAC	171.882	3.228
CACRAD	170.466	3.041	CACRAD	158.478	2.689	CACRAD	156.38	3.072	CACRAD	167.272	2.965	CACRAD	158.156	3.089
CACVEH	155.688	2.869	CACVOR	166.355	2.876	CACRAD	170.123	2.965	CACVEH	162.272	3.071	CACVEH	176.375	3.257
CACWAE	150.994	3.002	CACXOT	155.321	3.378	CACVOR	154.584	3.12	CACVOR	153.041	3.12	CACVOR	164.215	3.402
CADFOF	164.573	2.988	CADFUL	158.095	3.036	CACXOT	171.745	2.93	CACXOT	155.369	2.917	CADFOF	151.095	3.34
CADJID	165.648	2.718	CADJID	162.098	2.97	CADFUL	154.71	3.449	CADFUL	170.645	2.957	CADFUN	152.35	2.941
CADKEA	157.583	3.354	CADKEA	162.43	2.756	CADKAY	171.981	2.952	CADKEA	158.297	3.101	CADKEA	158.343	3.148
CADKEA	151.065	3.249	CADNEF	161.525	3.436	CADKEA	157.805	3	CADKEA	157.013	3.101	CADKEA	170.284	3.032
CADSOR	163.418	3.496	CADYIS	150.323	3.331	CADNEF	159.26	3.309	CADRUX	167.136	2.939	CADSOR	177.431	3.401
CADZUC	159.887	3.282	CADZUC	164.204	3.262	CADYIS	151.806	3.109	CADYIS	156.425	3.2	CADYIS	166.307	3.205
CAFFOJ	175.227	2.945	CAFFOJ	174.704	2.658	CADZUC	152.871	3.434	CAFFOJ	157.801	3.05	CAFFOJ	158.357	2.658
CAFGEW	168.186	2.996	CAFGEW	158.948	3.481	CAFFOJ	153.023	3	CAFFOJ	170.573	3.057	CAFFOJ	158.634	2.945
CAFGOG	169.056	3.448	CAFJUQ	156.273	2.888	CAFGEW	152.486	3.467	CAFGOG	169.689	2.989	CAFGOG	153.36	3.488
CAFLAZ	159.097	2.845	CAFLAZ	168.557	3.151	CAFJUQ	158.674	2.865	CAFKUS	154.574	3.113	CAFKUS	171.484	2.7
CAFMAB	177.812	3.243	CAFMAB	150.904	2.772	CAFLAZ	164.19	3.151	CAFMAB	155.474	2.859	CAFMAB	154.766	2.843
CAFMIG	152.636	3.403	CAFQIK	153.755	3.317	CAFMAB	161.959	2.824	CAFMAB	161.342	2.772	CAFMIG	152.653	3.318
CAFVAG	156.821	2.874	CAGHOM	169.222	2.87	CAFVAG	152.333	3.014	CAFVAG	161.204	2.88	CAFVAG	159.712	2.874
CAGQOR	176.789	3.163	CAGQOR	169.298	2.571	CAGHOM	155.372	2.967	CAGQOR	155.176	3.214	CAGQOR	175.73	3.403
CAGRAI	155.161	3.314	CAGVEP	172.829	3.214	CAGQOR	158.44	3.403	CAGQOR	153.232	3.359	CAGQOR	160.849	3.145
CAGWUG	171.798	2.831	CAGXER	150.97	3.208	CAGVOZ	168.754	2.872	CAGWAM	151.302	2.944	CAGWUG	171.633	2.831
CAHFIA	166.303	2.882	CAHFIA	164.532	3.404	CAGXER	173.733	3.133	CAGYOY	163.569	3.435	CAGYOY	160.369	3.496
CAJCEZ	161.67	3.305	CAJLIM	160.202	3.062	CAHFIA	173.827	2.997	CAHKOP	156.679	3.452	CAHKOP	169.635	3.073
CAKKIM	150.095	2.958	CAKKIM	170.632	2.78	CAJZIA	165.602	3.433	CAJZOG	165.889	3.437	CAKKEI	154.604	3.403
CALCEB	172.115	3.32	CALCEB	170.866	3.239	CAKKIM	161.949	3.389	CAKTAJ	156.274	2.887	CAKTAJ	154.216	2.917
CALCOL	176.667	3.235	CALCOL	155.412	3.356	CALCOL	162.271	3.369	CALCOL	151.355	3.114	CALCOL	167.96	3.39
CALGOO	158.194	3.381	CALMIO	173.779	3.163	CALCOL	151.446	3.459	CALCOL	161.221	3.167	CALCOL	167.337	3.263
CALPUC	157.456	3.193	CALPUC	155.404	3.318	CALPUC	153.753	3.277	CALPUC	161.385	3.262	CALPUC	153.279	3.368
CALQEN	155.894	3.081	CALTES	153.536	2.758	CALPUC	160.579	2.948	CALPUC	160.345	3.262	CALPUC	155.821	3.394
CALTES	157.627	3.087	CALTES	158.643	3.064	CALTES	173.108	2.962	CALTES	174.29	3.425	CALTES	163.431	3.187
CALTOC	163.479	2.653	CALTOC	162.791	3.496	CALTES	167.387	2.821	CALTES	159.27	3.315	CALTES	164.594	2.788
CALTOC	160.171	2.667	CALTOC	164.004	2.721	CALTOC	165.38	2.789	CALTOC	176.022	3.225	CALTOC	154.192	3.103
CALTOC	174.843	3.458	CALTOC	172.75	2.836	CALTOC	170.441	3.134	CALTOC	176.295	3.325	CALTOC	167.176	3.5
CALXEV	175.594	3.059	CALXEV	163.52	3.14	CALTOC	167.744	2.864	CALTOC	167.827	3.149	CALWIY	153.475	3.267
CAMFOM	152.331	3.253	CAMFOM	172.437	3.205	CALXEV	154.485	3.385	CAMBUQ	156.02	2.889	CAMFAZ	152.499	3.455
CAMHIJ	158.739	3.343	CAMHIJ	160.607	3.412	CAMFOM	152.075	3.412	CAMFOM	172.267	3.367	CAMFOM	156.768	3.395
CAMHOP	151.481	3.482	CAMHOP	175.901	2.996	CAMHIJ	155.22	3.123	CAMHOP	164.062	3.04	CAMHOP	153.614	2.847
CAMNAG	157.584	3.349	CAMNAG	166.105	3.032	CAMHOP	157.851	2.888	CAMHOP	161.314	2.898	CAMMAF	176.409	3.324
CANGUT	150.999	3.006	CANHAA	156.972	3.383	CAMSAO	154.942	3.467	CANGUT	152.952	3.199	CANGUT	154.132	3.159
CANQIT	159.237	3.36	CANQIT	170.86	2.794	CANLEJ	150.804	3.126	CANMUC	159.374	3.15	CANMUC	153.925	3.234
CANQOZ	162.668	3.446	CANVOF	159.222	2.831	CANQIT	166.309	3.175	CANQIT	154.463	2.666	CANQOZ	163.046	3.366
CANWIA	158.683	2.851	CANWIA	157.41	2.806	CANVUL	172.917	3.127	CANVUM	155.63	3.018	CANWEW	162.572	3.361
CANZIE	167.636	3.364	CANZIE	151.954	3.376	CANWIA	162.313	3.01	CANWIA	150.63	2.777	CANWIA	151.01	2.777
CAPJIN	163.787	3.167	CAPMUB	164.677	3.125	CAPDOQ	158.578	3.145	CAPDUW	166.014	3.314	CAPFAE	150.489	3.309
CAQBEE	151.543	3.333	CAQCON	165.686	3.163	CAPMUB	155.901	3.298	CAPRUJ	155.838	3.228	CAPSER	150.419	3.387
CAQCON	158.952	3.332	CAQCON	150.312	2.928	CAQCON	155.628	3.083	CAQCON	156.083	3.128	CAQCON	150.042	3.012
CAQKUC	152.25	3.199	CAQLAJ	177.436	3.081	CAQCON	151.338	3.27	CAQCON	153.618	2.968	CAQKOW	155.636	3.382
CAQMOZ	157.287	2.853	CAQMOZ	155.217	2.885	CAQLAJ	158.685	3.278	CAQMAI	157.674	3.128	CAQMAI	168.724	2.748
CAQSAR0	160.524	2.891	CAQSAR0	160.318	3.006	CAQMOZ	158.392	3.381	CAQMOZ	164.211	3.247	CAQSAR	150.675	3.493
CAQTUL	153.982	2.737	CAQTUL	152.214	3.213	CAQSUM0	166.489	3.337	CAQTAQ	150.672	3.231	CAQTUL	150.396	3.411
CARHIP	154.945	3.056	CARHIP	172.409	3.296	CAQTUL	155.032	3.451	CAQTUL	152.409	3.09	CAQZIH	158.853	3.421
CARHOV	173.197	3.226	CARHOV	155.592	3.429	CARHIP	163.16	2.854	CARHIP	158.26	3.465	CARHIP	177.015	2.96
CARHUB	154.416	3.455	CARHUB	152.975	2.891	CARHOV	160.731	2.99	CARHUB	157.917	2.773	CARHUB	160.323	3.312
CARJAJ	156.243	3.002	CARJAJ	162.657	2.804	CARHUB	153.841	2.997	CARHUB	153.673	3.491	CARHUB	150.238	3.477
CARWOI	177.728	2.992	CARWOI	154.956	3.289	CARJAJ	166.513	3.291	CARJAJ	153.308	3.179	CARWOI	155.688	3.331
CARWOI	158.214	3.158	CARWOI	171.613	3.411	CARWOI	167.047	3.179	CARWOI	158.318	2.579	CARWOI	175.164	2.65
CASMUI	152.264	3.265	CASPAO	170.703	2.741	CARWOI	164.222	3.328	CARWOI	162.853	2.673	CARWOI	159.533	3.181
CASVAV	173.759	3.005	CASVAV	154.32	3.284	CASPAO	153.997	3.259	CASPAO	157.497	3.248	CASPAO	159.852	3.075
CATPIX	157.492	3.319	CATPIX	157.946	3.013	CASVAV	151.047	2.93	CATNIV	156.946	3.016	CATPIX	157.662	3.438
CATPUI	151.381	3.457	CATWOL	165.647	3.07	CATPIX	151.735	3.435	CATPUI	159.571	2.904	CATPUI	153.337	3.429
CATXAY0	166.6	3.354	CATXAY0	150.408	3.443	CATWOL	156.452	3.07	CATXAY0	168.554	3.455	CATXAY0	151.414	3.455
CATXEC	157.438	3.279	CATXIG	173.457	3.062	CATXAY0	150.724	3.354	CATXEC	162.647	3.279	CATXEC	153.26	3.457
CAVVEE	152.278	2.982	CAVVEE	168.022	3.184	CATYUV	154.225	3.208	CAVHUG	156.816	2.664	CAVHUG	157.372	2.664
CAVVEE	160.221	3.165	CAVVEE	163.451	2.976	CAVVEE	162.742	2.98	CAVVEE	164.788	2.851	CAVVEE	150.638	2.955
CAWFAI	165.023	3.447	CAWKAO	150.898	3.48	CAVVEE	163.132	3.488	CAVYEG	158.07	3.058	CAVYUX	158.487	2.993
CAWPEY	154.662	2.974	CAWPEY	152.136	3.052	CAWPEY	152.548	3.052	CAWPEY	152.548	3.05	CAWPEY	154.835	2.968
CAWPEY	153.794	2.968	CAWPEY	153.855	2.974	CAWPEY	152.394	3.05	CAWPEY	154.068	2.895	CAWPEY	153.905	2.89
CAWQEX	161.692	3.123	CAWQIB	152.782	3.235	CAWPEY	152.089	2.895	CAWPEY	152.368	2.89	CAWQEX	152.341	3.239
CAWQOH	157.594	3.201	CAWQOH	162.748	3.367	CAWQIB	161.731	3.119	CAWQOH	158.363	3.463	CAWQOH	170.646	2.862
CAWQUN	155.031	3.183	CAWQUN	150.318	2.968	CAWQOH	166.484	3.067	CAWQOH	151.598	3.333	CAWQOH	163.012	3.328
CAWTAV	154.348	3.35	CAWWUU	167.971	2.508	CAWQUN	159.064	3.022	CAWQUN	168.6	3.117	CAWTAV	161.094	3.453
CAWZUY	157.43	2.905	CAWZUY	152.986	2.905	CAWWUU	152.473	3.21	CAWZUY	151.867	3.488	CAWZUY	160.372	3.242
CAXGIU	172.167	2.889	CAXGIU	162.084	2.812	CAWZUY	151.73	3.285	CAXFIR	158.237	3.247	CAXFIR	155.717	3.017
CAXGIU	169.054	3.122	CAXGIU	159.316	3.238	CAXGIU	157.099	3.459	CAXGIU	162.506	2.802	CAXGIU	169.778	3.013
CAXHAN	154.728	3.143	CAXHER	175.997	2.796	CAXGOA	153.516	3.298	CAXGUG	172.894	3.464	CAXHAN	154.593	3.174

CAXNUL	159.491	3.443	CAXNUL01	160.664	3.395	CAXMIY	161.593	3.226	CAXMIY	160.76	3.279	CAXNUL	160.884	3.409
CAXNUL03	161.165	3.43	CAXNUL03	159.345	3.442	CAXNUL01	159.758	3.417	CAXNUL02	160.919	3.41	CAXNUL02	159.419	3.428
CAXNUL05	158.837	3.485	CAXNUL06	160.473	3.382	CAXNUL04	161.415	3.451	CAXNUL04	159.125	3.456	CAXNUL05	161.858	3.488
CAXPID	153.02	3.198	CAXXEE	157.28	3.481	CAXNUL06	159.854	3.41	CAXPAT	162.114	3.22	CAXPID	151.218	2.941
CAXYUW	151.409	3.255	CAXYUW	165.584	3.103	CAXXEE	159.031	3.4	CAXXEE	160.212	3.275	CAXXEE	164.098	3.4
CAYBAJ	175.274	2.766	CAYJER01	162.135	3.329	CAXZAD	158.616	2.86	CAXZAD	159.588	2.837	CAXZAD	165.137	3.25
CAYRIH	174.882	3.383	CAYRIH	157.734	3.135	CAYJEU	162.486	3.026	CAYJEU	153.232	2.963	CAYJEU	156.904	2.873
CAZDUF	163.542	3.154	CAZFIV	168.782	3.376	CAYVOP	164.278	2.973	CAZDUF	158.95	3.495	CAZDUF	150.997	2.786
CAZKOH	168.269	2.973	CAZKOH	160.612	3.079	CAZFIV	156.185	3.4	CAZFOB	164.186	3.045	CAZKEW	161.739	3.38
CAZWUW	154.121	2.982	CEBLIG	169.162	3.405	CAZWOQ	154.254	3.453	CAZWOQ	177.28	3.474	CAZWOT	159.308	3.363
CEBVIR	164.738	2.943	CEBVIR	159.324	3.146	CEBLIG	163.886	3.298	CEBPOQ	168.369	2.981	CEBVIR	164.754	3.107
CECBAO	156.771	2.842	CECBAO	167.905	3.256	CEBVIR	150.095	3.245	CEBVIR	155.973	3.082	CEBZUG	153.235	2.981
CECCES	169.055	2.973	CECCES1	169.055	2.973	CECBAO	155.624	3.049	CECCAS	162.234	3.346	CECCAS	155.668	2.96
CECQOR	174.55	3.02	CECQOR	154.87	3.016	CECQOR	156.784	3.004	CECQOR	171.203	2.762	CECQOR	156.167	3.009
CECQUX	160.115	3.063	CECQUX	160.128	3.479	CECQOR	152.186	2.726	CECQUX	150.101	3.109	CECQUX	151.571	2.943
CECRUA	160.646	3.446	CECRUA	164.969	3.479	CECQUX	163.066	2.681	CECQUX	168.016	2.883	CECQUX	176.171	3.212
CECSAG	170.93	3.04	CEDCIY	162.496	3.201	CECRUA	175.107	3.14	CECSAG	172.308	3.242	CECSAG	177.757	3.156
CEFFEB	169.044	2.456	CEFFIE	153.029	2.842	CEDCOE	158.264	2.787	CEDQIN	150.252	2.92	CEFFEB	161.179	2.456
CEFTUE	171.235	3.181	CEFTUE	155.602	2.922	CEFJAB	152.199	3.486	CEFJEF	151.985	2.999	CEFSAK	157.181	3.017
CEFYAR	161.057	2.885	CEFYAR	155.02	3.028	CEFTUE	157.235	3.063	CEFXUK	157.983	2.839	CEFXUK	150.092	3.146
CEFYAR	157.486	2.922	CEFYAR	155.457	2.885	CEFYAR	165.445	3.053	CEFYAR	164.313	2.761	CEFYAR	157.6	2.778
CEFZET	151.996	3.32	CEGFAV	160.072	2.778	CEFYAR	155.64	3.273	CEFYAR	162.797	3.216	CEFYEV	166.502	2.928
CEGFUQ	159.618	3.174	CEGFUQ	174.343	2.845	CEGFAV	159.804	3.323	CEGFAV	150.794	3.362	CEGFUQ	156.381	3.166
CEGGAX	155.379	3.346	CEGGAX	168.656	3.109	CEGFUQ	151.382	2.845	CEGGAX	163.015	3.095	CEGGAX	166.478	2.794
CEGGEB	154.186	3.405	CEGGEB	163.833	3.467	CEGGAX	153.395	3.496	CEGGAX	155.735	2.794	CEGGAX	168.134	2.863
CEGGEB	152.722	3.185	CEGLUW	171.294	3.414	CEGGEB	159.834	3.411	CEGGEB	158.393	3.187	CEGGEB	161.243	3.217
CEGMEH	157.9	2.956	CEGMEH	165.056	3.013	CEGLUW	157.234	3.371	CEGLUW	164.892	3.277	CEGMEH	156.907	2.817
CEGMEH	156.627	3.286	CEGMEH	159.561	3.253	CEGMEH	170.136	3.056	CEGMEH	154.946	3.234	CEGMEH	153.241	3.236
CEGMOR	163.779	2.786	CEGMUX	155.654	3.325	CEGMIL	163.16	2.884	CEGMIL	166.413	3.294	CEGMIL	153.685	3.211
CEGMUX	153.434	3.393	CEGQUD	152.367	2.989	CEGMUX	151.341	3.272	CEGMUX	153.77	2.978	CEGMUX	164.192	3.01
CEGSEN	169.29	3.2	CEGSOW	161.526	3.205	CEGSEN	160.156	3.435	CEGSEN	171.396	2.929	CEGSEN	152.498	2.998
CEGSOX	153.662	3.394	CEGVIW	158.717	2.877	CEGSOW	157.13	2.859	CEGSOW	171.956	3.3	CEGSOX	153.808	3.333
CEHQEO	158.275	2.778	CEHQOZ	154.833	3.151	CEGVIW	153.303	3.332	CEHGIG	178.17	3.125	CEHPEK	163.804	3.108
CEJBEZ	170.162	3.359	CEJBEZ	150.215	2.839	CEHQUF	153.766	2.896	CEJBEC	160.431	3.236	CEJBEZ	163.056	2.839
CEJBEZ	155.021	3.227	CEJBEZ	150.406	3.087	CEJBEZ	152.214	3.497	CEJBEZ	159.204	3.143	CEJBEZ	154.95	3.262
CEJFAB	153.501	3.236	CEJFAB	154.675	3.339	CEJDIH	160.595	3.234	CEJDON	159.829	3.154	CEJFAB	170.855	2.909
CEJKIL	157.164	3.131	CEJKIL	153.284	3.284	CEJFAB	150.579	3.43	CEJFEF	172.181	3.431	CEJFII	153.762	3.229
CEJNOV	160.098	3.235	CEJNOV	165.08	2.865	CEJNOV	168.898	2.963	CEJNOV	159.053	3.101	CEJNOV	158.485	3.423
CEKCON	151.71	2.993	CEKDOO	171.516	3.394	CEJNOV	165.323	2.963	CEJNUB	173.315	3.329	CEJNUB	177.753	3.351
CEKDOO	170.262	3.012	CEKDOO	153.221	3.423	CEKDOO	165.189	3.453	CEKDOO	169.438	3.168	CEKDOO	155.565	2.711
CEKFUX	169.847	3.141	CEKJIL01	165.03	3.29	CEKDOO	161.21	3.065	CEKDUT	152.664	2.707	CEKDUT	175.137	3.388
CEKQIW01	159.767	3.024	CEKVEW	156.151	3.381	CEKLUD	164.632	2.984	CEKQIW	159.635	3.025	CEKQIW0	159.728	3.028
CEKVEW	151.847	2.838	CEKXIC	160.896	2.927	CEKVEW	151.024	3.124	CEKVEW	154.149	2.969	CEKVEW	151.223	2.821
CEKZUQ	163.78	3.018	CEKZUQ	173.899	2.779	CEKXOI	157.334	3.146	CEKYUM	154.612	2.876	CEKYUM	153	2.876
CELHIN	153.701	2.795	CELHOR	154.211	3.3	CEKZUQ	165.182	2.987	CEKZUQ	168.952	2.929	CELFII	150.65	2.811
CELMEM	151.022	3.033	CELMEM	163.224	2.827	CELHOR	156.388	3.323	CELLOY	159.24	3.081	CELMEM	168.313	2.827
CELMEM0	163.014	2.88	CELMEM0	167.835	2.936	CELMEM	168.216	2.856	CELMEM0	168.279	2.909	CELMEM0	151.374	3.064
CEMBAB	162.739	3.234	CEMBON	172.089	3.115	CELPOZ	174.263	2.841	CELPOZ	157.795	3.316	CELVOI	171.073	2.703
CEMGEJ	155.315	3.132	CEMGEJ	155.513	2.872	CEMBON	153.761	3.44	CEMBUT	154.964	3.275	CEMBUT	150.545	3.452
CEMGEJ	161.345	2.825	CEMGEJ	155.16	3.229	CEMGEJ	164.618	2.825	CEMGEJ	158.723	3.062	CEMGEJ	158.604	3.132
CEMGIN	159.205	3.096	CEMGIN	151.097	3.317	CEMGIN	153.147	3.113	CEMGIN	163.436	2.852	CEMGIN	159.155	3.04
CEMGIN	158.842	3.206	CEMGIN	157.669	3.113	CEMGIN	161.009	2.989	CEMGIN	163.709	2.882	CEMGIN	160.379	2.864
CEMGOT	175.533	3.067	CEMLAL	171.907	3.45	CEMGIN	158.252	2.852	CEMGIN	151.893	3.341	CEMGOT	159.015	3.319
CENMOY	156.592	3.224	CENMOY	154.036	3.224	CEMWUQ	169.783	3.371	CENKEO	160.008	3.241	CENMOY	158.211	2.982
CENTEV	157.623	3.405	CENZEE	151.856	2.95	CENPUK	177.08	3.028	CENPUK	157.969	2.971	CENTEV	170.212	2.911
CENZEE	162.309	3.023	CENZEE	161.437	3.076	CENZEE	156.183	2.963	CENZEE	160.066	2.964	CENZEE	159.656	2.978
CEQCUY	156.824	2.942	CEQCUY	170.152	2.839	CEPRUL	151.566	3.341	CEPRUL	173.334	2.894	CEPXAZ	152.869	3.427
CEQDAH	165.53	3.21	CEQDEJ	151.136	3.102	CEQCUY	159.195	3.374	CEQCUY	163.882	2.965	CEQCUY	174.064	3.111
CEQDEJ	150.746	3.2	CEQDEJ	163.285	3.171	CEQDEJ	169.921	2.673	CEQDEJ	178.118	3.351	CEQDEJ	179.074	3.215
CEQDEJ	158.292	3.141	CEQDEL	163.257	3.476	CEQDEJ	172.281	3.011	CEQDEJ	176.053	2.723	CEQDEJ	161.589	2.813
CEQKER	158.301	3.198	CEQKIT	164.959	3.006	CEQKAN	152.103	2.811	CEQKAN	177.687	3.489	CEQKAN	167.516	2.828
CEQKOB	167.651	2.927	CEQKOC	157.939	3.273	CEQKIT	154.548	3.1	CEQKIT	152.669	3.498	CEQKOB	155.438	2.976
CEQKUH	151.062	3.432	CEQLAN	173.029	2.973	CEQKUH	150.744	3.353	CEQKUH	166.079	3.363	CEQKUH	173.011	3.439
CEQLIW	164.362	2.845	CEQLIW	174.519	3.194	CEQLAO	172.107	3.494	CEQLES	164.649	3.252	CEQLIW	164.176	2.866
CEQLIW	169.786	3.194	CEQLIW	167.335	3.202	CEQLIW	170.805	3.202	CEQLIW	155.368	3.299	CEQLIW	159.71	3.343
CEQNEV	165.214	2.865	CEQNEV	155.438	3.369	CEQNEV	154.678	3.224	CEQNEV	153.786	3.466	CEQNEV	152.362	2.734
CEQTAX	176.808	2.811	CEQVOM	153.692	3.436	CEQNEV	156.662	3.47	CEQQOI	163.132	3.3	CEQREY	150.836	2.969
CERJOA	158.856	3.142	CERJOA	152.919	2.923	CEQZUW	157.857	3.049	CEQZUW0	154.718	3.189	CERJOA	153.011	3.466
CERJOA	162.33	2.79	CERJOA	167.78	3.027	CERJOA	153.975	3.403	CERJOA	154.248	2.768	CERJOA	170.255	3.056
CERLIY	157.649	3.355	CERSAU	154.247	3.074	CERKOD	162.695	3.363	CERKOD	158.748	3.18	CERLIY	151.46	3.279
CESBEL	158.995	2.914	CESBEL	153.44	3.373	CERSAU	162.287	3.223	CERTOK	153.548	3.208	CESBEL	160.536	2.835
CESBEL	167.307	3.491	CESBEL	162.981	3.293	CESBEL	163.283	2.937	CESBEL	174.076	2.549	CESBEL	172.692	3.188
CESBIP	155.091	3.19	CESBIP	154.453	3.162	CESBEL	157.072	2.952	CESBEL	162.868	3.272	CESBEL	152.626	3.057
CESGAM	165.626	2.985	CESGAM	162.745	3.06	CESFIT	155.852	3.093	CESFOZ	152.114	2.716	CESFUF	156.802	2.762
CESMEU	160.099	2.817	CESMEU	171.485	3.132	CESGIU	162.979	2.64	CESJIT	157.029	3.035	CESMEU	162.788	3.261
CESMEU	166.052	3.233	CESPIB	156.024	3.01	CESMEU	162.666	3.135	CESMEU	157.972	3.283	CESMEU	151.807	2.953
CETFIT	164.402	2.909	CETFIT	157.036	2.855	CESVUV	172.531	3.36	CETCIO	172.362	3.05	CETCOU	166.968	3.361
CETFIT	153.511	2.613	CETFIT	167.628	3.12	CETFIT	167.522	2.911	CETFIT	156.719	3.071	CETFIT	151.601	2.874
CETFIT	178.426	2.924	CETVOQ	166.369	3.488	CETFIT	159.87	3.293	CETFIT	155.973	3.154	CETFIT	164.711	3.12

CEVNEA	168.53	3.26	CEVPIE	151.047	3.135	CETXEE	161.926	3.129	CETXEE	163.205	3.174	CEVJUK	166.014	3.301
CEVRED	169.413	3.361	CEVRED	150.965	3.401	CEVRAZ	156.742	3.057	CEVRAZ	172.267	3.193	CEVRED	155.969	3.464
CEVXEJ	164.971	3.12	CEVXEJ	161.261	3.069	CEVRIH	157.573	3.3	CEVRON	170.982	2.987	CEVRON	159.609	2.959
CEVXEJ	177.604	2.945	CEVXEJ	161.279	2.867	CEVXEJ	170.791	3.128	CEVXEJ	155.972	3.035	CEVXEJ	153.318	3.064
CEVXEJ	158.039	3.274	CEVZIO	162.222	3.154	CEVXEJ	159.001	2.848	CEVXEJ	163.68	2.863	CEVXEJ	155.808	2.848
CEWBEP	159.695	2.689	CEWBEP	162.671	2.689	CEVZIO	168.122	3.202	CEWBEP	159.049	2.801	CEWBEP	150.159	2.857
CEWCUF	150.802	3.386	CEWDAM	155.616	2.964	CEWCUF	155.972	3.321	CEWCUF	151.148	3.289	CEWCUF	150.142	3.308
CEWHAP	174.562	3.278	CEWHAP	156.981	3.051	CEWFAL	155.483	3.149	CEWFOZ	158.807	2.95	CEWHAP	154.86	3.227
CEWNAX	153.189	3.162	CEWNAX	151.217	3.204	CEWHAP	151.442	2.965	CEWMIE	153.445	3.328	CEWMUQ	165.23	3.018
CEWTEG	150.946	3.1	CEWTEG	166.337	3.259	CEWNAX	164.673	3.215	CEWTEG	156.747	2.845	CEWTEG	154.16	3.271
CEXHES	162.806	3.382	CEXHES	165.922	3.33	CEWWIO	157.809	3.46	CEXFAQ	154.782	2.912	CEXFAQ	160.525	3.143
CEXNUS	151.609	2.661	CEXQED	155.281	3.379	CEXHES	157.57	3.36	CEXHES	150.845	3.09	CEXNIG	160.503	2.922
CEXTAD	161.64	2.938	CEXTAD	155.776	3.119	CEXQED	176.004	2.987	CEXTAD	152.84	2.872	CEXTAD	152.193	3.162
CEYCAL	171.01	3.371	CEYCAL	169.306	3.021	CEXTEG	156.296	2.95	CEXVUZ	154.451	3.451	CEXVUZ	154.996	3.133
CEYDES	151.106	2.778	CEYDES	161.593	3.073	CEYCAL	157.01	3.106	CEYDES	173.218	2.819	CEYDES	156.551	3.141
CEYFAO	169.656	3.215	CEYGAO	153.519	3.37	CEYDES	175.281	3.044	CEYDIU	159.982	3.315	CEYDUG	162.598	2.576
CEYYOY	155.776	2.693	CEYYOY	169.707	3.017	CEYGEW	157.51	3.261	CEYGEW	161.205	3.063	CEYGEW	165.539	3.293
CEZHUM	158.09	3.289	CEZPIH	164.624	3.166	CEZHUM	163.998	2.699	CEZHUM	162.376	3.272	CEZHUM	173.773	3.408
CEZSOS	164.533	2.966	CEZXAK	166.577	2.855	CEZPIK	161.662	3.33	CEZPIK	156.247	3.115	CEZSIM	165.171	2.939
CFBXMO	153.886	3.272	CFBYRH	154.438	3.061	CFBXMO	160.559	3.365	CFBXMO	154.97	3.046	CFBXMO	164.158	2.934
CFNPYR	162.282	3.065	CFNPYR	163.466	3.118	CFBYRH	151.649	3.12	CFBYRH	151.811	3.061	CFBYRH	155.306	3.12
CFPOFE1	158.316	3.071	CFPOFE1	160.733	3.003	CFNPYR	156.786	3.246	CFPOFE1	158.845	3.276	CFPOFE1	166.228	2.973
CHPFMO	156.581	3.479	CIBDOG	159.169	2.793	CFPOFE1	159.032	2.973	CFPOTL	164.585	3.145	CHPFBF	160.112	3.465
CIBNEK	173.553	2.92	CIBNEK	167.17	3.389	CIBDUM	168.533	3.256	CIBKUT	161.023	3.291	CIBNEK	158.905	2.811
CIBNOU	168.817	2.849	CIBNOU	159.744	3.322	CIBNIO	161.013	3.027	CIBNIO	153.65	3.436	CIBNOU	165.205	3.074
CIBWOB	161.18	2.771	CIBZIZ	161.511	3.356	CIBNOU	155.721	3.074	CIBQAJ	156.83	3.027	CIBWOB	150.246	3.497
CIBZIZ	160.085	3.257	CIBZIZ	167.901	3.236	CIBZIZ	164.392	3.296	CIBZIZ	171.025	2.848	CIBZIZ	160.718	3.039
CIBZIZ	169.41	3.037	CIBZIZ	150.534	3.237	CIBZIZ	154.32	2.941	CIBZIZ	152.041	2.807	CIBZIZ	167.601	3.086
CIBZIZ	168.962	2.994	CIBZIZ	157.844	2.807	CIBZIZ	165.53	2.908	CIBZIZ	154.864	3.236	CIBZIZ	160.718	3.29
CIBZOF	164.864	3.196	CIBZOF	174.397	3.082	CIBZOF	163.566	3.141	CIBZOF	163.239	3.373	CIBZOF	166.148	2.773
CICDUP	159.931	3.239	CICDUP	163.154	3.295	CIBZOF	177.311	2.672	CICDID	150.955	3.214	CICDUP	156.032	3.231
CIDDEB	177.746	3.351	CIDGAX	159.483	2.772	CICZOE	175.637	3.446	CICZOE	153.508	3.427	CICZOE	165.039	2.793
CIDSER	156.84	3.089	CIFBIF	172.956	3.096	CIDSER	171.313	2.671	CIDSER	152.971	2.858	CIDSER	158.67	2.671
CIFGON	155.345	3.006	CIFGUX	157.15	2.86	CIFGEH	157.307	3.383	CIFGEH	161.96	3.152	CIFGEH	157.977	3.215
CIFHUY	157.989	2.898	CIFJIL	169.862	2.888	CIFHUY	173.255	2.961	CIFHUY	171.752	3.015	CIFHUY	161.3	2.792
CIFJIO	150.308	3.027	CIFJIO	162.973	3.38	CIFJIL	150.316	3.238	CIFJIO	153.501	3.42	CIFJIO	160.557	3.242
CIFVAP	157.089	2.851	CIFVAP	158.643	3.121	CIFNIO	169.509	3.484	CIFNOU	157.552	3.323	CIFNOU	166.308	3.038
CIFWEW	154.514	2.973	CIFWEW	152.701	2.885	CIFVAP	163.905	3.349	CIFWEW	156.866	3.176	CIFWEW	160.429	2.833
CIFWEW	166.254	2.974	CIFWEW	160.124	2.809	CIFWEW	153.529	3.45	CIFWEW	154.794	3.209	CIFWEW	175.375	2.96
CIFWIA	170.565	2.782	CIFWIA	154.538	3.149	CIFWEW	164.937	2.807	CIFWEW	155.975	2.842	CIFWEW	155.287	3.09
CIFWIA	155.443	3.327	CIFWIA	172.366	2.782	CIFWIA	167.841	3.283	CIFWIA	154.171	2.944	CIFWIA	150.289	3.055
CIFWIA	158.73	3.095	CIFXEX	169.532	2.839	CIFWIA	161.633	2.823	CIFWIA	170.333	2.891	CIFWIA	159.148	2.944
CIGDIH	174.641	3.462	CIHHIM	173.095	2.987	CIFXEX	155.199	2.999	CIFXEX	157.165	3.093	CIGDEC	153.266	3.443
CIHLAH	159.867	3.383	CIHLAH	164.87	3.14	CIHHIM	163.688	2.7	CIHHIM	158.024	3.295	CIHLAH	169.164	2.953
CIHQAO	165.816	3.084	CIHQAO	162.489	3.139	CIHLAH	176.526	3.212	CIHMAK	166.443	3.21	CIHMAK	166.854	3.257
CIHQAO	152.64	3.255	CIHQUI	152.745	3.029	CIHQAO	151.315	3.207	CIHQAO	163.928	3.125	CIHQAO	175.223	3.139
CIHQUI	160.421	2.977	CIHQUI	168.213	2.82	CIHQUI	163.848	2.844	CIHQUI	171.798	3.154	CIHQUI	152.814	2.69
CIHQUI	158.97	3.494	CIHRAP	153.826	3.317	CIHQUI	168.256	2.967	CIHQUI	150.17	3.11	CIHQUI	159.121	2.836
CIHRET	153.172	3.412	CIHRET	166.974	3.333	CIHRAP	158.626	3.248	CIHRAP	165.244	3.257	CIHRET	162.257	3.195
CIHRET	155.426	3.421	CIHROD	167.899	3.203	CIHRET	172.599	3.006	CIHRET	151.474	3.087	CIHRET	154.995	3.006
CIHROD	174.422	3.34	CIHROD	162.747	2.927	CIHROD	159.452	3.242	CIHROD	163.396	3.329	CIHROD	171.651	3.222
CIHROD	168.937	3.232	CIHSUI	154.437	3.401	CIHROD	169.432	2.847	CIHROD	173.089	2.876	CIHROD	150.776	3.222
CIHTUM	176.156	3.226	CIHTUM	173.253	3.045	CIHSUI	174.103	3.119	CIHSUI	150.335	3.423	CIHSUI	154.536	3.116
CIHWOI	169.298	3.038	CIHWOI	171.128	3.371	CIHTUM	152.522	3.068	CIHVEX	160.764	3.157	CIHWOI	166.049	2.882
CIHWOI	154.88	2.853	CIHWOI	152.092	3.495	CIHWOI	168.122	3.418	CIHWOI	156.099	3.151	CIHWOI	157.322	3.406
CIHWOI	152.63	2.978	CIHWOI	153.017	2.968	CIHWOI	161.935	3.079	CIHWOI	158.766	3.343	CIHWOI	166.956	2.736
CIHWOI	164.405	3.331	CIJBIK	152.121	3.389	CIHWOI	152.884	2.736	CIHWOI	158.947	2.906	CIHWOI	168.844	3.311
CIJBIK	157.903	3.463	CIJBIK	162.444	2.682	CIJBIK	157.12	3.023	CIJBIK	160.873	3.392	CIJBIK	166.27	2.792
CIJBIK	172.169	3.448	CIJBIK	157.409	3.467	CIJBIK	161.522	2.887	CIJBIK	162.356	3.13	CIJBIK	155.125	3.084
CIJBOQ	154.236	3.021	CIJBOQ	162.374	3.064	CIJBOQ	152.915	3.11	CIJBOQ	162.754	2.915	CIJBOQ	154.209	2.803
CIJDIL	157.503	3.017	CIJDIL	165.279	3.316	CIJBOQ	151.266	3.333	CIJBOQ	154.407	2.991	CIJDIL	151.801	3.219
CIJHOW	161.3	3.116	CIJHOW	158.509	3.249	CIJDIL	155.859	3.493	CIJDIL	167.176	3.332	CIJDIL	167.361	3.187
CIJHOW	163.025	3.214	CIJJAK	156.666	3.064	CIJHOW	171.739	2.719	CIJHOW	157.926	3.287	CIJHOW	163.714	3.007
CIJJEO	163.114	3.158	CIJJEO	155.337	3.158	CIJJAK	155.917	3.011	CIJJAK	153.842	3.051	CIJJAK	150.488	2.88
CIJNAO	151.891	3.338	CIJSOG	162.014	3.163	CIJJIS	159.768	2.902	CIJJIS	173.014	3.427	CIJJIS	165.867	3.427
CIJYUT	151.449	3.49	CIJYUT	154.603	2.916	CIJYAZ	151.022	2.913	CIJYAZ	171.838	2.887	CIJYUT	161.538	3.099
CIKNIT	173.039	2.84	CIKNIT	170.046	2.916	CIJYUT	153.21	3.35	CIKNIT	162.044	2.979	CIKNIT	172.445	3.165
CIKSUM01	158.505	3.492	CIKSUM01	166.628	3.297	CIKNIT	152.211	3.316	CIKSUM	158.159	3.479	CIKSUM	166.322	3.266
CILLIW	155.515	3.15	CILLOC	167.343	3.176	CIKYIH	171.896	2.88	CIKZIJ	152.011	3.207	CIKZIJ	155.527	3.048
CILLOC	165.541	3.021	CILLOC	151.74	3.065	CILLOC	163.931	3.059	CILLOC	154.782	2.746	CILLOC	157.286	3.113
CILNIX	155.078	3.318	CILNIX	162.458	3.29	CILNIX	175.86	3.427	CILNIX	155.438	2.809	CILNIX	152.37	3.341
CILNIX	159.277	3.299	CILTID	159.351	3.425	CILNIX	160.908	2.774	CILNIX	161.568	2.838	CILNIX	154.476	3.299
CIMXAY	152.442	3.292	CIMXAY	152.224	2.895	CILZIJ	153.192	3.499	CIMGEL	157.577	3.396	CIMXAY	154.538	3.306
CIMXAY	155.672	3.117	CIMXAY	155.794	3.144	CIMXAY	169.189	3.475	CIMXAY	168.33	3.499	CIMXAY	165.083	3.05
CIMXEC	157.692	3.475	CIMXEC	166.063	2.896	CIMXAY	150.699	2.848	CIMXAY	157.029	3.374	CIMXAY	165.583	3.144
CIMXED	152.35	3.152	CIMXED	161.963	2.901	CIMXEC	153.753	3.143	CIMXEC	158.917	3.071	CIMXED	162.881	3.266
CIMXUT	151.932	2.783	CIMYOO	157.4	2.734	CIMXED	151.441	3.467	CIMXED	152.55	2.858	CIMXED	161.932	3.188
CINMUH	158.909	3.103	CINMUH	150.244	3.02	CIMZUU	163.209	3.274	CINBOR	150.174	3.37	CINBOR	161.032	2.891
CINVEC	160.53	3.327	CINVIG	162.719	2.939	CINMUH	151.941	3.031	CINQIB	161.911	3.01	CINVEC	170.996	3.168

CINWOO	164.091	3.195	CINWUU	167.44	3.199	CINWII	169.205	3.263	CINWII	158.979	3.42	CINWII	173.25	3.247
CINXEF	156.584	3.136	CIPBAH	158.463	2.963	CINWUU	172.4	3.126	CINXAB	155.726	3.169	CINXAB	156.952	3.373
CIPZOR	158.112	3.392	CIQFUH	150.683	2.99	CIPBAH	157.552	2.963	CIPYEJ	152.597	2.824	CIPYOR	159.726	3.422
CIRJUK	162.14	3.138	CIRNOH	151.277	2.754	CIQFUH	153.686	3.257	CIQGAO	156.766	3.263	CIRHOC	161.562	3.25
CIRTII	153.517	3.152	CIRWIJ	159.378	3.034	CIRNOH	150.415	2.727	CIRNOH	162.175	3.321	CIRPUR	150.164	2.883
CIRZOW	158.028	3.203	CIRZOW	159.421	3.328	CIRWOP	175.755	3.47	CIRWOP	157.163	3.412	CIRWOP	151.87	3.126
CIRZUC	159.929	3.165	CIRZUC	164.137	3.355	CIRZOW	176.389	3.446	CIRZUC	155.228	3.167	CIRZUC	150.28	3.276
CIRZUC	164.892	3.316	CIRZUC	158.591	3.336	CIRZUC	160.126	3.193	CIRZUC	168.187	2.942	CIRZUC	159.063	2.848
CISBAL	150.679	2.976	CISBAL	150.035	2.864	CISBAL	155.461	3.003	CISBAL	156.68	3.024	CISBAL	175.563	2.934
CISDEP	164.084	3.345	CISDIT	155.446	3.206	CISBAL	168.719	2.97	CISDAL	161.717	3.162	CISDAL	170.601	3.355
CISGAN	150.934	2.947	CISGAN	150.797	3.46	CISGAN	152.239	3.404	CISGAN	151.638	3.388	CISGAN	175.353	3.192
CISVIN	172.923	3.111	CISVOT	174.616	3.265	CISNAX01	170.752	2.851	CISTUV	156.259	2.909	CISTUV	153.398	2.909
CISWAG	166.567	2.92	CISWAG	163.582	2.817	CISVOT	165.816	2.961	CISVOT	153.74	3.206	CISVOT	154.15	3.015
CITBEN	159.282	2.931	CITMEB	156.307	2.815	CISYIQ	161.198	3.244	CISYIQ	158.45	2.935	CITBEN	158.8	3.199
CITMEB	159.601	3.493	CITMEB	167.725	3.374	CITMEB	170.98	3.43	CITMEB	152.764	3.4	CITMEB	154.431	3.284
CIVPUU	158.819	3.36	CIVQAB	167.559	3.399	CITQIF	165.881	2.919	CIVBOB	159.159	3.292	CIVLUQ	158.437	2.983
CIWSEI	162.62	2.952	CIWSEI	151.139	3.487	CIVWIQ	157.496	3.127	CIWSEI	152.611	2.958	CIWSEI	155.498	2.925
CIXBIU01	161.713	3.439	CIXBIU01	176.306	3.091	CIWSEI	157.751	2.953	CIWSEK	157.106	3.329	CIWVAJ	157.367	2.795
CIXGOI	155.292	2.967	CIXJUR	160.946	2.738	CIXBIU01	161.291	3.252	CIXGIC	162.776	3.263	CIXGOI	154.397	2.985
CIXMAA	153.125	2.883	CIXMAA	171.407	3.292	CIXLUT	175.561	3.005	CIXLUT	150.421	2.914	CIXLUT	153.162	2.914
CIYHUQ	158.436	3.479	CIYHUQ	162.257	2.891	CIXQOP	153.49	2.806	CIXQOP01	153.294	2.806	CIXSAF	166.767	3.036
CIYNED01	155.502	3.392	CIYNED01	162.003	3.454	CIYJAY	173.938	2.902	CIYJIG	173.847	2.809	CIYJUS	159.726	3.487
CIYNUX	161.875	2.98	CIYNUX	169.166	3.167	CIYNUX	166.302	2.735	CIYNUX	164.665	2.695	CIYNUX	167.565	3.319
CIYPIN	163.155	3.468	CIYQAG	170.003	3.263	CIYNUX	172.406	3.319	CIYNUX	154.824	2.695	CIYPIN	169.314	3.253
CIYVAJ	155.082	3.45	CIYVIR	158.708	3.326	CIYQEK	162.828	3.433	CIYQEK	162.973	2.968	CIYQEK	151.905	2.794
CIZBET	150.276	2.708	CIZHIE	164.272	3.406	CIZBET	169.57	2.708	CIZBET	161.018	2.742	CIZBET	168.513	2.742
CIZJUS	161.86	3.334	CIZLUV	153.581	3.148	CIZHIE01	166.253	3.136	CIZJIF	163.704	3.041	CIZJIF	160.762	3.064
COBRAP	151.603	3.394	COCFEI	160.282	2.927	COBCOM	151.912	3.331	COBCOM	157.905	3.251	COBCOM	160.01	3.1
COCFEI05	162.117	2.952	COCFEI06	162.602	2.954	COCFEI01	161.06	2.931	COCFEI02	160.999	2.935	COCFEI04	161.823	2.946
COCFIM	168.061	3.078	COCFIM	151.962	2.975	COCFEI07	162.983	2.957	COCFEI08	163.328	2.964	COCFEI11	161.327	2.943
COCFUY	168.376	3.214	COCFUY	172.582	2.757	COCFUY	151.684	2.757	COCFUY	175.099	3.391	COCFUY	165.581	2.83
COCRIX	157.987	3.448	COCYEB	168.834	3.022	COCQER	156.791	3.234	COCQER	151.089	3.132	COCQER	157.987	2.731
COCYEB	171.655	2.795	COCZAY	152.912	2.943	COCYEB	158.41	3.325	COCYEB	170.338	2.902	COCYEB	162.718	2.795
COCZEC	151.481	3.087	COCZEC	169.17	3.398	COCZAY	156.397	3.05	COCZAY	150.974	3.407	COCZAY	156.733	3.148
COCZOM	153.348	3.11	COCZOM	157.56	3.455	COCZEC	169.277	3.333	COCZEC	150.825	3.126	COCZOM	153.411	3.073
COCZOM	151.896	3.001	COCZOM	170.234	3.265	COCZOM	151.842	2.82	COCZOM	150.29	2.82	COCZOM	151.766	2.98
COCZOM	153.499	2.946	COCZUT	157.666	3.457	COCZOM	175.556	3.125	COCZOM	152.546	2.957	COCZOM	151.25	3.118
CODCAD	166.304	2.916	CODCUX	169.534	3.254	CODBEG	175.525	3.17	CODCAD	161.304	3.139	CODCAD	156.4	2.88
CODCUX	166.895	3.383	CODCUX	150.782	3.213	CODCUX	173.599	3.103	CODCUX	163.325	3.239	CODCUX	172.207	3.254
CODCUX	159.196	3.114	CODCUX	150.788	3.011	CODCUX	170.954	3.488	CODCUX	168.828	3.458	CODCUX	165.851	3.049
CODCUX	155.576	3.156	CODFOQ	171.267	3.169	CODCUX	160.576	3.435	CODCUX	153.958	3.116	CODCUX	153.52	3.143
CODMOZ	175.192	3.176	CODPIW	154.155	3.226	CODFOQ	176.078	3.361	CODFOQ	158.637	2.901	CODMOZ	155.495	3.171
COFMPT	157.179	3.482	COFMPT	156.677	3.448	CODVUN	166.634	3.389	COFBNI10	168.559	3.226	COFCIM	151.607	3.086
COFMPT	152.949	2.828	COFMUH	159.983	3.194	COFMPT	161.455	3.105	COFMPT	158.954	2.943	COFMPT	150.778	3.211
COGBUX	159.842	3.47	COGCUA	162.166	3.38	COFMUH	158.29	2.854	COFXAY	167.673	3.147	COFXOM	157.831	3.06
COGHOV	172.097	3.218	COGHOV	154.35	2.727	COGCUA	151.7	3.33	COGCUA	150.836	3.336	COGDOV	176.774	3.167
COGNAR	162.539	3.405	COHFIO01	153.482	3.239	COGHOZ	167.937	2.867	COGHUF	156.631	3.024	COGMAP	163.101	2.922
COHFIO01	152.278	2.607	COHFIO01	162.173	2.925	COHFIO01	154.517	2.65	COHFIO01	166.357	3.337	COHFIO01	150.552	3.242
COHFIO01	176.744	2.944	COHFIO01	166.18	2.607	COHFIO01	166.626	3.053	COHFIO01	171.64	3.349	COHFIO01	158.663	2.878
COHFIP	160.39	2.719	COHFIP	165.782	3.393	COHFIO01	170.68	3.135	COHFIO01	172.81	3.357	COHFIO01	162.139	2.65
COHFUB	168.772	3.436	COHFUB	159.696	2.997	COHFIP	168.904	3.393	COHFUB	166.495	3.436	COHFUB	162.929	3.331
COHGAI	175.137	3.415	COHGAI	150.26	2.962	COHFUB	173.082	3.412	COHGAI	168.104	3.383	COHGAI	153.227	2.899
COHGEM	165.851	3.101	COHGEM	175.708	2.831	COHGEM	157.33	3.236	COHGEM	154.588	3.411	COHGEM	151.484	3.423
COHJOZ	166.288	2.872	COHJOZ	165.368	3.04	COHGEM	162.215	2.946	COHGEM	165.711	3.315	COHJOZ	161.233	2.809
COHJOZ	161.204	3.158	COHPAR	158.646	3.035	COHJOZ	157.994	2.809	COHJOZ	176.795	2.834	COHJOZ	151.765	3.129
COJBEJ	157.169	3.247	COJBEK	170.455	3.049	COHPEW	154.987	3.075	COHPEW	152.097	3.126	COHYII	150.36	3.007
COJCAH	172.532	2.808	COJGOY	155.901	2.977	COJBEK	153.591	2.861	COJBIN	167.891	3.222	COJBIN	164.909	3.289
COJGOY	164.435	3.058	COJKIX	163.913	3.293	COJGOY	165.197	3.227	COJGOY	158.81	3.067	COJGOY	168.68	3.479
COJRAV	153.149	3.428	COJRAV	165.488	2.848	COJKIX01	163.808	3.182	COJPOJ	163.742	3.224	COJRAV	159.829	3.048
COKDEN	159.365	3.028	COKDEN	177.431	3.148	COJROJ	151.834	3.441	COKDAJ	152.45	2.739	COKDAJ	168.566	2.724
COKRAY	150.432	2.83	COKRAY	167.206	3.214	COKDOX	153.559	2.921	COKGAN	158.345	2.971	COKMOH	162.219	3.158
COLGOA	161.786	2.673	COLGOA	165.093	3.165	COLBOW	166.37	3.053	COLDIT	150.155	2.856	COLGOA	151.037	3.215
COLMEX	152.664	3.434	COLNAU	173.585	3.168	COLGOA	152.983	3.205	COLHIW	151.393	3.15	COLKOF0	152.194	3.23
COLNUM	165.398	3.08	COLNUM	155.994	3.165	COLNAU	167.629	3.291	COLNAU	165.327	2.876	COLNAU	162.976	2.876
COMKIZ	166.488	2.733	COMQAZ	157.937	2.977	COLNUM	150.258	3.361	COLZEM	162.412	2.799	COMKIZ	154.299	3.052
COMXEL	152.716	2.967	COMXEL	153.982	3.386	COMVIL	150.124	2.85	COMVIL	165.615	3.42	COMVIL	168.766	3.003
COMXOV	173.868	2.946	COMXOV	164.995	2.755	COMXEL	161.422	3.196	COMXEL	152.512	2.885	COMXOV	171.286	3.379
CONNOJ	155.233	3.325	CONPUU	161.118	3.397	COMXOV	172.573	2.87	COMYEL	150.508	3.409	CONLEY	171.009	3.025
CONYIO	155.503	2.85	CONYIO	150.545	2.96	CONQAZ	151.051	2.943	CONQUT	153.649	2.893	CONRAA	174.151	3.323
CONZIS	163.644	3.03	CONZOY	167.28	2.738	CONZIS	165.083	3.274	CONZIS	153.116	3.02	CONZIS	156.611	3.02
COPFOE	173.324	2.974	COPGEX	161.369	3.016	CONZOY	154.377	3.305	CONZOY	167.574	3.282	CONZOY	169.147	2.859
COPROS	156.748	3.088	COPROS	158.352	3.209	COPGEX	152.93	2.828	COPNUS	174.802	3.001	COPQUW	167.652	2.92
COPROS	159.558	3.248	COPROS	151.225	2.861	COPROS	154.045	3.216	COPROS	164.57	3.248	COPROS	153.543	3.39
COPRUY	161.955	3.199	COPSAF	176.018	3.421	COPROS	162.359	3.029	COPRUY	150.023	2.976	COPRUY	178.067	3.159
COPSAF	160.977	3.129	COPSAF	155.32	3.311	COPSAF	150.725	3.293	COPSAF	161.007	3.222	COPSAF	168.57	2.865
COPSAF	155.824	3.303	COPSAF	153.182	2.855	COPSAF	150.329	3.298	COPSAF	155.415	3.156	COPSAF	165.93	2.667
COPXOX	160.556	3.493	COPXUD	152.547	2.935	COPTIN	156.282	3.369	COPXOX	161.698	3.393	COPXOX	157.352	2.944
COPXUD	162.739	2.82	COPXUD	153.274	2.858	COPXUD	154.387	3.473	COPXUD	155.929	3.046	COPXUD	155.502	2.769
COPXUD	153.287	3.259	COPYAK	162.183	2.808	COPXUD	154.724	3.418	COPXUD	151.632	3.446	COPXUD	153.887	3.31

COPYAK	158.495	3.008	COPYAK	159.486	3.03	COPYAK	153.816	3.03	COPYAK	172.391	3.08	COPYAK	170.347	2.914
COQBAN	169.722	3.415	COQBER	164.044	3.002	COPYAK	157.986	3.422	COPYAK	169.278	3.217	COPZAJ	152.347	2.978
COQGOF	157.762	2.7	COQQIJ	158.714	3.288	COQGOF	161.227	3.004	COQGOF	159.023	3.336	COQGOF	158.065	2.799
CORFEV	152.798	3.367	CORFEV	162.849	3.367	COQQIJ	162.245	3.159	COQWOU	161.735	3.342	CORCIX	174.396	3.42
CORFOF	155.843	2.732	CORNAZ	162.595	3.094	CORFEV	170.569	2.77	CORFEV	156.878	3.173	CORFIZ	160.885	2.972
CORVUD	150.002	2.891	CORWIS	155.011	2.784	CORQOP	160.001	3.323	CORVUD	151.615	3.054	CORVUD	163.701	3.011
CORZEQ	150.234	2.729	COSDUM	166.124	2.997	CORXIT	173.42	2.997	CORZEQ	159.218	3.427	CORZEQ	169.309	3.111
COSDUM	158.582	3.297	COSDUM	159.895	3.387	COSDUM	175.949	2.763	COSDUM	152.084	2.993	COSDUM	155.615	3.287
COSHEB	161.737	2.659	COSHEB	156.266	2.934	COSDUM	157.209	2.961	COSHEB	158.529	2.843	COSHEB	157.844	3.247
COSHEB	163.965	3.247	COSHEB	157.308	3.216	COSHEB	162.043	2.901	COSHEB	168.536	2.901	COSHEB	158.845	3.126
COSHEB	156.09	2.956	COSHEB	166.238	3.123	COSHEB	155.368	2.889	COSHEB	151.75	3.105	COSHEB	159.682	3.5
COSHEB	151.761	3.441	COSLIG	161.446	3.061	COSHEB	151.914	2.848	COSHEB	155.724	2.956	COSHEB	153.805	3.247
COSREI	164.429	3.038	COSREI	166.306	3.228	COSQAE	169.478	3.136	COSQAE	150.173	2.955	COSQAE	173.608	3.123
COTDIB	164.489	3.491	COTDIB	165.271	2.837	COTDEX	150.774	3.386	COTDEX	156.302	3.434	COTDIB	174.581	2.718
COTMOQ(	166.037	3.216	COTREL	170.132	2.972	COTDIB	175.761	3.473	COTJOM	152.529	3.095	COTMOQ	165.387	3.21
COTSEM	167.651	2.605	COTWER	171.691	2.767	COTREL	155.228	3.411	COTRIP	164.615	2.77	COTRIP	171.129	2.938
COTWOB	156.401	3.151	COTXUI	159.188	3.041	COTWER	154.987	3.257	COTWER	155.421	3.242	COTWOB	152.947	2.953
COVZAR	165.773	2.745	COVZAR	165.351	3.441	COVJEF	173.918	2.919	COVYIY	162.31	3.04	COVYIY	157.223	3.186
COWGOL	159.038	2.886	COWJEE	165.688	3.291	COWBEX	160.886	3.321	COWCUN	152.645	3.049	COWCUN	172.35	2.75
COWLOQ	157.484	3.187	COWQAG	153.521	2.934	COWJII	167.863	3.26	COWJUU	154.958	3.335	COWKII	153.16	3.417
COXCEX1	164.978	3.137	COXDUQ	169.451	2.966	COWQAG	153.544	3.155	COWQEK	152.01	3.024	COWQEK	150.518	3.175
COXFAY	150.838	3.095	COXFAY	167.432	3.104	COXDUQ	153.468	3.453	COXFAY	154.807	2.942	COXFAY	155.905	3.383
COXRAJ	166.89	3.017	COXYIX	151.645	3.247	COXFAY	167.262	3.222	COXGED	158.072	3.293	COXRAJ	168.991	3.258
COYSER	161.315	3.393	COYSER	166.678	2.942	COYPOX	158.369	3.244	COYPOX	172.384	3.408	COYROB	161.294	3.199
COYSER	167.112	3.26	COYXIA	165.586	3.312	COYSER	171.573	3.079	COYSER	171.885	3.28	COYSER	164.476	3.132
COZFAZ	163.776	3.314	COZFAZ	152.773	3.468	COZBUR	155.091	2.524	COZBUS	173.454	2.758	COZFAZ	166.643	3.288
COZLEI10	170.896	2.883	COZLOT	170.891	3.231	COZFIG	168.278	3.225	COZLEI10	150.603	3.478	COZLEI10	156.254	3.027
COZQEQ	153.269	2.847	COZQEQ	156.301	3.238	COZQEQ	162.941	3.097	COZQEQ	151.382	2.683	COZQEQ	156.69	3.492
COZQEQ	158.37	3.35	COZQEQ	165.376	2.954	COZQEQ	150.623	3.02	COZQEQ	151.424	3.055	COZQEQ	163.573	2.793
COZQEQ	154.672	2.952	COZQEQ	157.247	3.352	COZQEQ	156.086	2.927	COZQEQ	163.038	3.388	COZQEQ	164.307	3.169
COZQOY	157.995	3.492	COZQOY	158.471	3.124	COZQEQ	163.409	3.181	COZQOY	155.446	3.422	COZQOY	156.18	3.274
COZRAL	156.061	3.263	COZRAL	154.892	3.123	COZQOY	167.692	3.12	COZQOY	151.687	2.92	COZRAL	155.757	3.405
COZREP	157.402	3.266	COZREP	157.467	3.491	COZRAL	166.247	3.094	COZRAL	150.267	2.9	COZREP	155.111	3.439
COZRER	157.923	3.173	COZRER	157.701	3.217	COZREP	156.978	3.134	COZREP	167.573	3.126	COZREP	151.126	2.916
COZRIV	167.014	3.262	COZRIV	156.184	3.407	COZRER	154.544	3.173	COZRER	160.505	3.39	COZRER	162.632	3.427
COZXAT	166.106	3.117	COZYOH	152.159	3.229	COZRIV	150.152	3.401	COZSOB	162.843	3.491	COZSOC	157.474	3.36
CPAZCO	156.583	2.95	CPAZCO1	156.583	2.95	COZYOI	150.39	3.344	COZYOI	175.318	2.572	COZYUN	153.791	3.313
CPFDEL1(	155.662	3.162	CPFDEL1(	163.349	3.113	CPEFRH	151.837	3.195	CPFDEL1(	166.884	3.269	CPFDEL1(	152.577	2.888
CPFPPA0'	162.82	3.227	CPFPPA0'	158.285	2.82	CPFDEL1(	162.341	3.284	CPFMPI1(	171.765	3.082	CPFMPI1(	161.18	3.082
CSFMFB	161.623	3.111	CSMRHC1	165.221	3.305	CPFPPA0'	173.802	3.208	CPFVLV	162.074	3.284	CPFVLV	162.582	3.291
CUCCOS	162.57	2.954	CUCCOS	170.22	2.954	CSMRHC1	157.184	2.912	CUCCOS	155.162	3.22	CUCCOS	159.988	3.114
CUCJUI	154.7	2.703	CUCJUI	172.561	2.888	CUCFIQ	161.689	2.902	CUCGUC	160.374	3.386	CUCJUI	151.056	3.016
CUCKOB	160.053	2.673	CUCKOB	160.179	3.191	CUCJUI	161.727	3.396	CUCJUI	172.776	3.172	CUCJUI	153.007	3.001
CUCPOF	178.698	3.052	CUCPOF	158.895	2.758	CUCKOB0	162.472	2.918	CUCKOB0	154.672	2.687	CUCKOE	157.083	3.407
CUCZEF	151.47	3.34	CUDFUG	159.337	2.767	CUCPOF	156.619	2.942	CUCPOF	161.116	2.736	CUCQAU	164.514	3.263
CUDMOE	158.66	3.041	CUDMOE	161.734	3.374	CUDHAL	161.893	2.857	CUDHAL	150.163	3.468	CUDJUH	155.238	2.705
CUDMOE(	161.734	3.374	CUDNEX	157.979	3.309	CUDMOE	154.901	3.311	CUDMOE(	158.66	3.041	CUDMOE(	154.901	3.311
CUDNIZ	156.727	3.236	CUDROJ	150.253	3.304	CUDNEX0	158.016	3.31	CUDNIZ	159.527	3.052	CUDNIZ	161.871	3.301
CUDSAY	153.153	3.422	CUDSAY	150.594	3.334	CUDSAY	160.925	3.485	CUDSAY	153.135	3.416	CUDSAY	171.222	3.362
CUDSAY	158.443	3.395	CUDSAY	171.651	3.052	CUDSAY	150.738	3.206	CUDSAY	150.438	3.331	CUDSAY	175.022	2.889
CUDXAB	152.887	3.217	CUDZUW(	150.036	3.234	CUDSAY	156.532	2.986	CUDTUT	163.744	3.079	CUDTUT	154.471	2.865
CUFBUA0	160.622	2.86	CUFBUA0	162.454	3.457	CUENFP	157.12	2.918	CUENFP0	157.71	2.918	CUFBUA0	170.121	2.705
CUFGIW	159.376	3.263	CUFJOG	158.007	3.163	CUFCOZ	155.042	3.123	CUFDIU	150.411	3.261	CUFGIW	164.681	3.263
CUFKIA	173.23	3.489	CUFKOE0	159.192	2.917	CUFJUM	167.864	3.315	CUFJUM	176.594	3.32	CUFKAT	177.697	3.425
CUFKOE0	170.621	2.782	CUFKOH	156.591	2.761	CUFKOE0	155.018	3.156	CUFKOE0	155.119	3.276	CUFKOE0	151.224	3.374
CUFTII	158.57	3.457	CUFTII	156.124	2.96	CUFRUS	175.652	2.759	CUFRUS	159.561	2.722	CUFRUS	161.483	2.759
CUFTOO	168.348	3.238	CUFTOO	150.719	3.263	CUFTII	151.32	3.194	CUFTII	154.644	3.176	CUFTII	152.402	3.088
CUGCAM	167.558	3.467	CUGCIU	160.783	2.841	CUFTOO	171.089	2.964	CUFTOO	169.619	2.964	CUFVAE	162.096	2.934
CUGJIB	158.844	3.259	CUGJOH	164.703	2.416	CUGCIU	153.299	3.453	CUGDAM	165.903	3.449	CUGHOF	152.039	3.041
CUGPEZ	166.547	3.114	CUGXEJ	153.821	3.195	CUGLOI	153.54	3.32	CUGMEW	163.104	3.07	CUGMEW	159.569	3.154
CUGZUA	150.941	2.863	CUGZUA	155.732	3.23	CUGXEJ	165.202	3.195	CUGXEJ	162.421	3.007	CUGXOT	153.552	3.292
CUHCEQ0	150.691	2.952	CUHCEQ0	165.252	2.884	CUHBAJ	161.199	3.492	CUHCEQ	164.447	2.979	CUHCEQ	152.922	3.019
CUHPED	161.105	3.01	CUHYEN	151.514	2.702	CUHJOF	155.371	3.303	CUHPED	150.892	3.26	CUHPED	159.462	3.004
CUJSOQ	163.65	2.744	CUJSOQ	166.486	2.955	CUHYEN	165.355	3.428	CUJJUP	152.986	3.051	CUJSAC	172.661	3.373
CUJXEL	150.092	2.782	CUKCUJ	162.105	2.738	CUJSOQ	153.736	2.955	CUJSUW	167.231	2.846	CUJSUW	159.697	2.846
CUKFIB	169.217	3.001	CUKGUK	158.202	2.772	CUKCUJ	162.218	2.708	CUKFIB	161.286	2.963	CUKFIB	151.874	3.385
CUKNUV	152.41	3.053	CUKQIJ	175.35	2.864	CUKNUV	155.224	2.745	CUKNUV	166.741	3.053	CUKNUV	152.751	2.835
CUKXIP	155.835	3.373	CUMFAV	159.299	3.264	CUKQIJ	159.989	3.474	CUKWEO	159.533	3.071	CUKWEO	172.931	2.952
CUMHEB	154.023	3.257	CUMHEB	157.183	3.04	CUMFAV	173.884	3.151	CUMFAV	161.618	2.871	CUMHEB	157.241	3.396
CUMTAI	160.981	3.076	CUMYOB	156.349	3.201	CUMTAI	166.714	3.076	CUMTAI	152.587	3.372	CUMTAI	162.432	3.491
CUNLON	153.857	3.423	CUNLON	150.482	3.423	CUMYOB	160.518	3.201	CUNCIZ	158.993	3.435	CUNKUT	167.471	3.464
CUNVAM	150.84	2.942	CUNVEQ0	150.318	2.924	CUNLON	167.982	2.793	CUNPEH	163.423	3.05	CUNPEH	172.525	3.452
CUPFEY	168.954	2.977	CUPFUP	152.475	3.197	CUNVEQ0	150.87	2.95	CUNWUF	165.369	3.26	CUPCEY	154.899	2.979
CUPJIH	158.43	3.165	CUPJUT	158.703	3.098	CUPFUP	150.232	3.158	CUPGEA	151.259	3.162	CUPGUS	160.021	3.391
CUPZUL	166.102	3.277	CUPZUL	160.698	3.141	CUPKEE	156.847	2.818	CUPKII	170.673	2.952	CUPMAB	153.263	3.302
CUQKIL	162.642	2.755	CUQLAC	164.377	2.729	CUQCUM	175.205	2.744	CUQHOO	151.273	2.952	CUQKEH	158.2	2.815
CUQLEG	160.253	2.788	CUQLEG	158.48	3.446	CUQLAC	151.836	3.01	CUQLAC	162.093	2.833	CUQLAC	155.798	2.729
CUQLEG	159.093	3.46	CUQLEG	153.966	2.977	CUQLEG	154.07	3.153	CUQLEG	164.39	2.814	CUQLEG	152.222	2.863
CUQLEG	155.746	2.977	CUQLEG	162.016	3.446	CUQLEG	171.937	3.447	CUQLEG	172.25	2.788	CUQLEG	155.666	2.868

CUQNIO	150.955	2.846	CUQNIO	160.965	2.902	CUQLEG	157.354	2.935	CUQLEG	155.699	2.868	CUQLEG	154.165	3.2
CUQNIO	153.705	3.462	CUQNIO	167.57	3.462	CUQNIO	154.663	3.249	CUQNIO	165.071	3.249	CUQNIO	152.733	3.34
CUQNIO	166.012	3.302	CUQREL	153.241	2.813	CUQNIO	159.503	3.012	CUQNIO	152.052	2.74	CUQNIO	155.17	3.047
CUQREL0	169.583	2.661	CUQREL0	151.58	2.969	CUQREL	169.853	2.656	CUQREL	152.189	2.97	CUQREL	164.932	2.656
CUQYAP	161.153	2.925	CUQYAP	159.369	3.498	CUQREL0	165.554	2.661	CUQREL0	153.102	2.803	CUQXER	150.749	3.341
CUQZIY	158.274	2.952	CUQZIY	155.255	2.691	CUQYAR	154.081	3.004	CUQZAQ	159.922	3.147	CUQZAQ	152.714	2.954
CURCEA	155.702	2.801	CURFUU	150.015	2.781	CUQZIY	172.158	2.96	CUQZIY	166.737	3.344	CURCEA	153.848	2.747
CURGEF	156.744	3.357	CURGIJ	158.149	3.412	CURFUU	152.099	2.781	CURFUU	150.617	2.762	CURGEF	158.329	3.423
CURJUW	153.677	3.38	CURJUW	171.217	3.173	CURGIJ	156.571	3.344	CURJIL	163.587	3.276	CURJUW	166.263	3.103
CURJUX	165.492	2.975	CURYAT	156.806	2.912	CURJUW	152.017	2.852	CURJUW	153.091	3.169	CURJUX	152.356	3.348
CUSNUZ	160.641	2.929	CUSNUZ	166.907	3.16	CUSFEC	164.47	3.442	CUSNUZ	151.585	3.196	CUSNUZ	169.313	3.184
CUSNUZ	160.911	3.486	CUSNUZ	152.402	3.277	CUSNUZ	169.105	3.048	CUSNUZ	171.399	3.329	CUSNUZ	163.566	3.354
CUSPAK	151.282	2.773	CUSPAK	161.557	2.773	CUSNUZ	174.825	3.259	CUSNUZ	155.892	3.094	CUSNUZ	150.938	3.299
CUSPEO	153.434	3.318	CUSTUH	169.693	3.088	CUSPAK	171.988	2.788	CUSPAK	151.958	3.393	CUSPAK	158.47	2.788
CUSTUH	151.116	3.114	CUSTUH	166.664	3.131	CUSTUH	166.147	2.837	CUSTUH	175.094	3.034	CUSTUH	158.052	3.227
CUSWAQ	152.256	2.744	CUSWOE	155.111	2.948	CUSTUH	165.939	2.914	CUSTUH	159.684	3.053	CUSTUH	162.769	3.053
CUTDAW	159.302	3.421	CUTDAW	154.914	3.284	CUTDAW	165.64	2.99	CUTDAW	154.278	2.828	CUTDAW	158.853	2.923
CUTFUW	150.43	3.176	CUTGAZ	158.011	3.47	CUTDAW	162.04	2.821	CUTFAC	158.525	3.2	CUTFUW	155.136	3.157
CUTGUX	156.475	2.819	CUTGUX	166.859	2.899	CUTGEH	158.968	3.346	CUTGIL	167.084	2.853	CUTGOR	161.781	3.324
CUTHEI	158.437	3.164	CUTHUY	161.942	3.008	CUTHAE	156.717	2.912	CUTHAE	155.06	2.78	CUTHEI	156.274	3.319
CUTLAI	155.064	3.06	CUTLAI	152.906	2.844	CUTJAD	160.481	3.326	CUTJIL	167.655	2.831	CUTJIL	153.298	3.288
CUTLEM	176.336	3.406	CUTLOW	169.955	3.064	CUTLAI	158.183	3.398	CUTLEM	175.533	3.19	CUTLEM	169.685	2.872
CUTMEN	160.798	2.76	CUTMEN	157.225	2.976	CUTLOW	151.273	2.777	CUTLOW	160.382	3.422	CUTLOW	154.337	2.842
CUVBOM	150.478	3.026	CUVBOM	159.106	3.284	CUTNEO	170.574	3.363	CUTVIX01	158.848	3.493	CUVBOM	152.548	3.295
CUVBOM	165.065	3.145	CUVBOM	154.45	3.339	CUVBOM	151.168	3.255	CUVBOM	159.452	3.197	CUVBOM	160.989	3.143
CUVBOM	156.711	3.102	CUVBOM	172.266	3.318	CUVBOM	157.14	3.249	CUVBOM	166.969	3.338	CUVBOM	158.44	3.02
CUVBOM	166.37	3.314	CUVBOM	151.586	3.066	CUVBOM	171.978	3.276	CUVBOM	164.404	3.03	CUVBOM	154.701	3.351
CUVDEE	150.347	3.118	CUVDEE	157.934	2.989	CUVBOM	152.576	3.244	CUVCED	158.943	3.242	CUVDEE	156.441	3.116
CUVDOM	161.602	3.242	CUVDOM	164.481	3.499	CUVDEE	153.721	3.348	CUVDOM	164.105	3.185	CUVDOM	162.141	3.193
CUVMAL	168.457	3.239	CUVNOY	164.857	2.852	CUVJOS	162.614	3.139	CUVJOS	171.528	3.107	CUVJOS	168.029	2.91
CUVNOY	156.271	2.957	CUVQIW	176.578	3.317	CUVNOY	151.442	3.115	CUVNOY	163.3	3.094	CUVNOY	151.974	3.235
CUVSIV	154.083	3.064	CUVSIV	160.867	3.396	CUVSAP	167.295	2.932	CUVSAP	166.934	3.234	CUVSAP	165.739	2.951
CUVSIV02	168.027	3.456	CUVSOB	162.983	2.92	CUVSIV	160.514	3.064	CUVSIV01	156.177	3.008	CUVSIV01	161.576	3.39
CUVWEX	156.731	3.085	CUVWEX	151.228	3.499	CUVSOB	154.993	2.94	CUVSOB	158.276	3.302	CUVSUJ	172.309	2.891
CUVZAY	175.283	3.479	CUVZAY	157.314	3.097	CUVWEX	152.686	3.273	CUVWEX	152.714	3.273	CUVWEX	164.184	3.351
CUWDEF	166.511	3.274	CUWFIL	170.09	3.278	CUVZAY	157.288	3.306	CUVZAY	175.422	3.361	CUVZAY	153.883	3.237
CUWFOT	150.981	3.184	CUWKIS	155.191	3.467	CUWFOP	162.778	3.227	CUWFOT	150.455	3.127	CUWFOT	154.223	3.089
CUWYOL	153.576	3.461	CUWYOL	153.709	3.125	CUWKIS	154.716	3.409	CUWPOA	163.735	3.154	CUWWUN	155.458	3.127
CUXDAD	154.131	3.386	CUXDAD	152.231	3.371	CUXCIL	156.049	3.045	CUXDAD	164.08	3.069	CUXDAD	165.468	3.14
CUXDAD	153.038	3.218	CUXDAD	156.509	3.223	CUXDAD	152.876	3.219	CUXDAD	159.563	3.209	CUXDAD	170.176	3.352
CUXPAP	151.87	2.994	CUXWAT	160.419	3.104	CUXNAO	171.213	2.753	CUXNAO	151.828	2.943	CUXPAP	153.506	2.81
CUXZIG	156.896	3.143	CUYJEL	162.877	3.048	CUXWAT1	160.419	3.104	CUXWEX1	164.751	2.889	CUXWEX1	157.973	3.487
CUYLUD	152.809	2.959	CUYQUI	160.556	3.231	CUYJEL	151.104	3.478	CUYJEL	156.552	3.163	CUYLUD	178.084	3.366
CUYTEY	156.9	3.217	CUYTEY	162.766	3.273	CUYTEY	158.116	3.423	CUYTEY	166.998	3.423	CUYTEY	160.926	2.893
CUZBUU	151.562	3.477	CUZFAI	156.646	3.308	CUYTEY	154.048	3.356	CUZBUU	161.783	3.331	CUZBUU	155.194	2.973
DABFOF	169.669	3.027	DABFUL	164.037	3.173	CUZFAI	156.76	3.306	CUZZII	162.444	2.689	DABFIZ	158.594	3.288
DABGEW	160.792	3.028	DABJAV	177.321	3.261	DABGEW	163.731	3.028	DABGEW	167.721	3.34	DABGEW	162.308	3.452
DABLOK	173.043	2.842	DABLUQ	154.747	3.082	DABJAV	150.622	3.38	DABJOH	152.392	3.051	DABJOJ	157.628	3.452
DABMIG01	153.212	2.783	DABRAC	151.843	3.174	DABMIG	167.985	2.871	DABMIG	153.212	2.783	DABMIG01	167.985	2.871
DABVEM	153.066	3.426	DABVEM0	150.464	3.47	DABRAC	166.045	3.287	DABRAC0	151.843	3.174	DABRAC0	166.045	3.287
DABVEM0	150.215	3.456	DABWUD	164.073	2.908	DABVEM0	151.202	3.429	DABVEM0	153.447	3.404	DABVEM0	153.629	3.408
DACFIZ	160.02	2.896	DACFIZ	166.438	3.009	DABWUD0	164.761	2.91	DABXIQ	170.186	2.904	DACCUG	158.765	3.352
DACGEV	173.215	2.991	DACGEV	168.82	3.285	DACFIZ02	152.724	2.919	DACFIZ02	166.665	3.199	DACGEV	172.999	3.027
DACMEE	158.222	3.001	DACVOW	162.518	2.839	DACGEV	169.196	3.473	DACLUQ	172.061	2.973	DACMEE	172.853	3.001
DADGOE	165.95	3.446	DADHOF	155.35	3.274	DACVUC	157.964	3.043	DACVUC	159.884	3.142	DACYEN	165	3.391
DADSIL	170.489	3.276	DAFCAP	159.553	3.081	DADHOF	155.949	3.379	DADMEA	158.356	2.946	DADPAE	170.41	3.39
DAFCAP	151.771	3.024	DAFCAP	159.691	2.963	DAFCAP	162.436	2.979	DAFCAP	156.415	2.968	DAFCAP	166.692	3.035
DAFCAP	166.608	2.793	DAFCAP	156.825	2.864	DAFCAP	156.678	2.911	DAFCAP	167.478	2.776	DAFCAP	161.65	2.883
DAFCAP	150.343	3.337	DAFCAP	161.981	3.257	DAFCAP	150.469	3.383	DAFCAP	161.035	3.185	DAFCAP	158.094	2.798
DAFCIX	158.047	3.42	DAFCIX	160.548	2.778	DAFCIX	162.775	3.335	DAFCIX	157.68	2.996	DAFCIX	155.811	3.172
DAFDAP	157.141	3.435	DAFDIY	152.117	3.093	DAFCUJ	164.181	3.253	DAFCUJ	165.602	3.236	DAFCUJ	153.639	3.065
DAFGUP	168.095	3.238	DAFGUP	163.91	3.263	DAFDIY	162.285	3.29	DAFFIZ	151.335	3.212	DAFFIZ01	151.326	3.225
DAFMUV	159.211	3.111	DAFMUV	158.863	3.449	DAFJEC	160.539	3.412	DAFKOP	151.419	3.172	DAFMUV	161.407	3.139
DAFNAD	153.176	3.116	DAFPOQ	153.92	3.316	DAFMUV	171.15	3.037	DAFNAD	169.258	3.275	DAFNAD	170.597	3.098
DAFVUF	155.453	2.999	DAGFEZ	169.084	3.496	DAFQIO	155.738	2.93	DAFVUF	153.492	3.102	DAFVUF	157.878	3.137
DAHDOF	160.922	2.811	DAHDOJ	153.13	3.214	DAGFEZ	162.022	2.691	DAGMOO	151.169	3.023	DAGQEL	176.247	3.322
DAHFEZ	161.296	2.857	DAHFEZ	170.057	3.104	DAHFEZ	154.473	2.975	DAHFEZ	169.7	3.365	DAHFEZ	155.816	3.476
DAHFEZ	174.134	2.857	DAHFEZ	168.326	3.396	DAHFEZ	168.391	3.052	DAHFEZ	161.021	3.058	DAHFEZ	160.559	3.078
DAHFEZ	172.582	2.743	DAHGUR	169.598	3.249	DAHFEZ	158.646	2.977	DAHFEZ	178.332	3.155	DAHFEZ	151.784	3.421
DAHGUR	158.6	3.452	DAHHAY	175.896	3.182	DAHGUR	153.919	3.373	DAHGUR	173.269	3.349	DAHGUR	164.65	3.342
DAHLAA	154.936	3.191	DAHLAA	153.31	2.985	DAHKUT	172.238	3.001	DAHLAA	169.553	3.192	DAHLAA	168.463	2.886
DAHLAA	158.998	3.197	DAHLAA	160.722	2.894	DAHLAA	152.782	2.985	DAHLAA	164.617	2.858	DAHLAA	166.091	3.321
DAHXES	157.16	3.39	DAHYAM	170.122	3.125	DAHLAA	159.647	3.142	DAHRAI01	160.795	2.825	DAHSEL	151.172	2.902
DAJHEF	175.713	2.99	DAJHIJ	156.72	3.425	DAHYAM	159.465	3.125	DAJHAX	171.641	3.266	DAJHAX	150.39	3.178
DAJROY	166.298	3.024	DAJROY	175.352	3.074	DAJQOX	170.095	3.205	DAJRAK	153.604	3.451	DAJROY	152.513	3.093
DAKBUQ	164.305	3.212	DAKBUQ	155.11	2.906	DAJSEP	155.389	3.032	DAJTUD	153.449	3.141	DAJTUD	161.969	3.344
DAKGIG	165.667	3.485	DAKGIG	153.491	3.205	DAKBUQ	165.553	2.715	DAKCIF	167.347	2.844	DAKGEC	150.114	3.027
DAKRAL	164.021	3.427	DAKRAL01	164.181	3.425	DAKGIH	159.338	3.136	DAKHED	156.295	2.932	DAKHED	175.269	2.811
DALBOL	170.159	3.38	DALGUV	166.835	2.991	DAKREP	159.935	2.705	DAKREP	160.127	2.705	DAKYIB	157.336	2.842

DALJAC	159.967	3.401	DALJAC	157.742	3.218	DALGUV	161.543	2.991	DALHAB	151.971	3.377	DALHAB	160.827	3.306
DALNUA	164.625	3.405	DALPOZ	171.037	2.892	DALNIR	153.861	2.759	DALNUA	156.828	3.232	DALNUA	150.55	2.888
DALQEQ	158.02	2.967	DALQEQ	154.286	3.219	DALPOZ	166.373	3.25	DALQEQ	159.676	3.231	DALQEQ	158.406	3.259
DALVIX	164.19	3.114	DALZID	151.328	3.426	DALROY	177.479	2.99	DALVIW	159.957	3.021	DALVIW	158.265	3.021
DAMWOD	155.259	3.01	DAMWOD	155.528	3.187	DALZID	169.157	2.935	DAMREO	172.698	2.914	DAMWOD	163.783	2.878
DAMWOD	159.221	3.449	DAMWOD	163.499	3.162	DAMWOD	150.327	3.042	DAMWOD	169.922	3.314	DAMWOD	151.81	3.271
DAMWOD	153.685	3.229	DAMWOD	160.501	3.39	DAMWOD	159.967	2.878	DAMWOD	172.448	2.993	DAMWOD	153.851	2.97
DAMXEY	170.482	3.204	DAMXEY	162.903	3.133	DAMWOD	152.273	3.148	DAMWOD	167.786	3.485	DAMXEY	150.76	3.299
DAMZOI	164.494	3.179	DANBAV	155.168	3.095	DAMXEY	160.762	3.482	DAMXEY	152.218	2.711	DAMZEY	155.814	3.428
DANSIX	166.163	3.403	DANSIX	152.027	3.086	DANSIX	155.309	3.017	DANSIX	160.596	2.93	DANSIX	151.34	3.197
DANSIX	165.315	3.49	DANSIX	154.519	3.169	DANSIX	154.616	3.1	DANSIX	170.227	2.944	DANSIX	156.19	3.207
DANSIX	151.142	3.063	DANSIX	155.257	3.383	DANSIX	175.022	2.832	DANSIX	152.887	3.419	DANSIX	160.752	3.38
DANWIC	157.271	3.297	DANWOI	160.813	2.922	DANWIC	169.782	3.17	DANWIC	152.728	3.492	DANWIC	158.439	3.345
DANWUO	151.193	3.186	DANWUO	154.575	3.25	DANWOI	164.407	3.223	DANWOI	158.923	2.858	DANWUO	160.728	3.461
DANWUO	154.26	3.064	DANWUO	164.134	3.124	DANWUO	151.503	3.41	DANWUO	153.459	2.935	DANWUO	161.749	3.266
DANXAV	163.184	3.352	DANXAV	165.86	3.345	DANXAV	157.608	3.37	DANXAV	157.749	3.019	DANXAV	170.601	2.802
DANXEZ	162.384	3.213	DANXEZ	166.748	3.277	DANXEZ	153.164	3.227	DANXEZ	153.581	3.425	DANXEZ	157.149	2.889
DANXID	172.713	3.147	DANXID	160.461	2.865	DANXEZ	151.747	2.804	DANXEZ	160.99	3.29	DANXID	162.517	3.318
DAPDAC	162.363	3.448	DAPDAC	166.733	3.19	DANXID	153.053	3.36	DANXID	153.246	3.035	DANXID	157.705	3.05
DAPHEL	161.393	3.438	DAPHEL	150.484	3.448	DAPDAC	169.996	3.388	DAPHEL	161.957	3.461	DAPHEL	156.497	3.456
DAPHIP	161.083	3.214	DAPHIP	153.42	2.858	DAPHEL	152.7	3.137	DAPHEL	150.163	2.956	DAPHIP	156.232	3.384
DAPKEO	154.762	2.753	DAPKEO	175.555	2.954	DAPHIP	162.798	3.171	DAPJIR	169.141	2.945	DAPJOX	166.265	2.865
DAPKOY	166.226	3.237	DAPKUE	155.772	2.839	DAPKEO	164.113	2.925	DAPKIS	162.45	3.443	DAPKOY	154.758	3.139
DAQXUP	174.689	3.091	DAQXUP	154.238	3.265	DAPKUE	176.894	3.046	DAPTOF	155.096	2.786	DAQXUP	164.584	3.398
DARFIO	150.785	3.029	DARGEL	167.422	3.472	DAQZAA	156.526	3.347	DARBIL	156.582	2.74	DARFIO	164.291	3.102
DARSAR	159.672	2.721	DARXUQ	160.96	3.035	DARGEL0	168.578	3.427	DARLAL	168.517	2.781	DARSAR	162.786	2.721
DASBEG	152.176	3.138	DASBIL	155.03	3.494	DASBAE	154.884	3.246	DASBAE	152.862	3.21	DASBEG	156.74	3.292
DASCUW	151.215	3.178	DASCUW	150.57	3.057	DASBUW	150.112	2.928	DASCUW	161.794	3.353	DASCUW	163.484	3.022
DASCUW	169.705	3.195	DASCUW	171.456	2.704	DASCUW	160.952	3.03	DASCUW	163.748	3.257	DASCUW	158.697	3.151
DASCUW	167.812	3.063	DASFAI	152.421	2.866	DASCUW	172.528	2.933	DASCUW	151.743	3.383	DASCUW	169.019	2.89
DASFIQ	154.464	2.866	DASFIQ	160.261	2.891	DASFAI	168.835	3.137	DASFIQ	163.503	2.92	DASFIQ	164.586	2.873
DASKER	167.288	3.036	DASKER	150.667	3.143	DASFOS	162.083	3.014	DASFOS	163.064	2.877	DASKER	152.905	3.344
DASRUO	150.365	2.905	DATCEI	163.691	3.031	DASKER	160.361	3.091	DASKER	174.646	3.198	DASKER	155.16	3.091
DATHOV	154.209	3.362	DATKAL	159.354	3.305	DATCEI	165.035	3.031	DATCEI	161.072	3.158	DATCOS	177.961	3.096
DATQUO	165.334	3.128	DATQUO	155.588	2.99	DATKAL	160.772	3.425	DATKAL	173.302	3.247	DATLIV01	152.303	2.921
DATSOH	151.466	3.304	DATSOH	152.099	3.38	DATQUO	166.334	3.422	DATQUO	168.646	3.251	DATQUO	167.917	3.397
DATTAV	169.611	3.489	DATTAV	159.77	3.497	DATTAV	164.575	3.291	DATTAV	155.274	3.261	DATTAV	156.083	3.284
DATTAV	150.112	3.161	DATTAV	151.15	2.979	DATTAV	177.567	2.833	DATTAV	153.315	3.42	DATTAV	150.696	3.485
DATTAV	155.441	3.066	DATTAV	169.83	3.402	DATTAV	150.894	3.33	DATTAV	155.897	3.431	DATTAV	150.191	2.967
DATTAV	160.306	3.366	DATTAV	165.2	3.249	DATTAV	156.177	3.472	DATTAV	169.079	3.201	DATTAV	166.347	3.252
DAVCAF0	167.063	3.203	DAVJAP	159.542	2.835	DATTAV	162.894	3.335	DATTAV	163.327	3.474	DAVCAF	168.583	3.272
DAVLES	155.844	2.924	DAVSID	153.277	3.081	DAVJAP	162.777	2.835	DAVLAO	174.516	3.281	DAVLAO	167.178	3.436
DAVSID	167.005	3.005	DAVSID	160.236	3.026	DAVSID	165.109	2.887	DAVSID	177.82	3.005	DAVSID	166.792	3.306
DAVSID	158.293	2.949	DAVSOJ	157.399	3.06	DAVSID	155.659	3.149	DAVSID	164.325	3.204	DAVSID	155.97	3.204
DAVYIL	156.066	2.909	DAVYIL	161.591	3.079	DAVYIL	160.837	3.196	DAVYIL	156.726	3.383	DAVYIL	158.539	3.19
DAVYIL	155.542	2.734	DAVYIL	164.197	3.002	DAVYIL	161.944	3.23	DAVYIL	154.524	3.047	DAVYIL	151.163	3.141
DAVYIL	162.75	2.886	DAVYIL	163.134	3.45	DAVYIL	159.159	2.734	DAVYIL	159.448	3.113	DAVYIL	159.821	3.342
DAVYIL	157.504	2.945	DAVYIL	157.985	3.154	DAVYIL	163.389	3.293	DAVYIL	154.88	2.932	DAVYIL	172.781	3.121
DAWFIR	157.433	3.435	DAWFIR	169.913	3.069	DAWCIN	170.854	3.212	DAWCIN1	171.649	3.227	DAWFIR	150.265	2.965
DAWKIW	173.077	3.355	DAWKIW	162.731	3.058	DAWFIR	172.386	3.427	DAWFIR	150.552	2.905	DAWFIR	167.432	3.058
DAWXII	164.908	3.289	DAWYAF	164.974	3.143	DAWKIW	159.68	3.33	DAWWUX	160.608	3.213	DAWWUX	168.809	2.972
DAXQAY	161.735	3.186	DAXQAY	162.109	3.433	DAXGIT	162.959	3.273	DAXGIT	160.382	2.679	DAXGIT	159.356	2.777
DAXQEC	164.716	3.376	DAXQEC	154.804	3.075	DAXQAY	169.756	3.498	DAXQEC	155.563	3.211	DAXQEC	153.931	3.325
DAXQIG	167.465	3.113	DAXQIG	166.943	3.052	DAXQEC	155.055	3.075	DAXQEC	161.389	3.269	DAXQIG	168	2.818
DAXQIG	152.029	3.135	DAXQOJ	150.946	3.045	DAXQIG	175.425	3.322	DAXQIG	159.127	2.915	DAXQIG	164.124	2.818
DAXQOJ	160.894	3.398	DAXQOJ	161.845	2.674	DAXQOJ	158.352	3.175	DAXQOJ	152.419	2.805	DAXQOJ	159.033	2.846
DAXQOJ	163.538	3.413	DAXQOJ	150.56	2.742	DAXQOJ	152.54	2.925	DAXQOJ	150.218	3.486	DAXQOJ	166.947	3.396
DAXREZ	156.897	3.323	DAXRID	160.84	3.175	DAXQOJ	161.851	2.805	DAXQOJ	161.648	3.31	DAXQOJ	159.196	2.932
DAXROJ	159.409	3.406	DAXROJ	172.1	3.306	DAXRID	160.886	3.412	DAXRID	152.793	3.103	DAXRID	155.168	3.175
DAXTUS	171.795	3.057	DAYFOZ	172.024	3.202	DAXRUP	153.283	3.186	DAXRUP	158.924	3.186	DAXTUS	174.62	3.453
DAYXAG	157.421	3.342	DAYXEH	170.93	3.49	DAYWOT	164.349	3.288	DAYWOT	167.621	3.494	DAYXAG	160.111	3.152
DAYXIO	162.223	2.834	DAYYAD	157.881	2.715	DAYXEK	150.342	3.03	DAYXIO	159.566	2.883	DAYXIO	154.935	3.493
DAZVUY	178.602	2.908	DBOFBP1	170.909	3.358	DAYYAD	160.927	3.262	DAZKOF	154.019	3.089	DAZVUY	167.763	3.215
DEBJEC	157.002	3.226	DEBJED	168.383	2.97	DBOFBP1	158.878	3.061	DBOFBP1	169.505	3.465	DEBJEC	165.537	3.226
DEBYIT	158.109	3.055	DEBYIT	162.515	3.16	DEBJEZ	164.38	3.297	DEBJEZ	162.248	3.297	DEBPIN	159.383	3.468
DEBYIT	154.884	3.06	DEBYIT	156.92	2.829	DEBYIT	159.357	3.407	DEBYIT	171.611	3.248	DEBYIT	173.278	3.02
DEBYIT	162.009	3.267	DEBYIT	152.269	3.253	DEBYIT	172.042	2.658	DEBYIT	161.616	3.317	DEBYIT	165.547	3.008
DECCAS	167.737	3.04	DECCAS	165.014	3.024	DEBYIT	154.58	3.266	DEBYIT	168.241	2.845	DECCAS	173.106	3.243
DECFDP0	160.758	2.851	DECFDP0	155.223	3.247	DECFAV	166.217	3.241	DECFDP0	151.914	2.706	DECFDP0	154.191	2.706
DECMII	152.527	3.263	DECMII	158.43	3.263	DECFDP0	157.329	3.121	DECFDP0	160.443	2.799	DECFDP0	168.048	3.307
DECQEK0	157.565	2.829	DECQEK0	173.743	2.829	DECNIJ	170.831	2.873	DECNIJ	168.856	3.172	DECPIL	150.027	2.844
DEDCIA	152.723	3.156	DEDKOP	166.658	3.068	DECQIO0	150.548	2.946	DECQIO0	150.469	2.942	DECQIP	153.376	3.132
DEDKOP	170.577	3.068	DEDKOP	159.988	3.192	DEDKOP	162.175	2.911	DEDKOP	158.264	3.415	DEDKOP	168.357	3.09
DEDKOP	163.725	3.09	DEDKOP	155.289	2.78	DEDKOP	153.911	3.219	DEDKOP	161.469	3.498	DEDKOP	150.555	2.925
DEDKOP	151.939	2.918	DEDKOP	169.584	2.811	DEDKOP	158.199	2.928	DEDKOP	152.604	3.141	DEDKOP	153.491	3.271
DEDKOP	158.344	3.158	DEDKOP	163.299	3.216	DEDKOP	173.14	3.322	DEDKOP	152.856	3.137	DEDKOP	161.385	3.113
DEDKOP	151.074	2.962	DEDKOP	165.683	2.908	DEDKOP	170.02	3.188	DEDKOP	154.791	3.119	DEDKOP	156.027	3.174
DEDLIH	151.705	2.972	DEDLIH	157.261	2.83	DEDLIH	173.98	2.9	DEDLIH	159.982	3.456	DEDLIH	150.795	2.989
DEDLIH	158.799	2.86	DEDLIH	155.38	3.446	DEDLIH	151.114	3.379	DEDLIH	162.319	3.318	DEDLIH	153.067	3.382

DEDLIH	157.662	3.426	DEDLIH	161.106	2.988	DEDLIH	156.736	2.886	DEDLIH	167.329	2.958	DEDLIH	155.095	2.969
DEDLON	173.323	3.155	DEDLON	167.334	2.792	DEDLIH	154.395	3.39	DEDLIH	153.463	2.83	DEDLIH	177.716	2.924
DEDLON	156.782	3.078	DEDLON	172.617	3.371	DEDLON	175.403	2.961	DEDLON	152.483	3.019	DEDLON	169.855	3.309
DEDMAE	156.141	2.79	DEDMAE	156.535	3.052	DEDLON	153.408	3.498	DEDLON	158.099	2.792	DEDMAE	172.029	3.451
DEDMEI	174.758	2.907	DEDMEI	151.015	3.454	DEDMAE	174.023	2.79	DEDMAE	156.97	3.159	DEDMAE	162.68	3.052
DEFBUL	171.015	3.333	DEFBUL	158.533	3.023	DEDMEI	150.703	3.031	DEFBUL	154.636	3.115	DEFBUL	171.564	3.41
DEFGIH	155.899	2.992	DEFHIJ	166.747	2.996	DEFBUL	154.698	3.319	DEFBUL	160.786	3.479	DEFBUL	159.164	2.968
DEFHUV	155.64	3.128	DEFJEH	157.379	3.334	DEFHIJ	158.493	3.179	DEFHIJ	161.651	2.991	DEFHIJ	150.416	2.854
DEFLEH	169.451	2.701	DEFMAD	161.517	3.469	DEFJEH	167.881	3.334	DEFKIJ	163.3	3.084	DEFLEH	170.398	3.005
DEGHUU	164.796	3.007	DEGKUW	157.867	3	DEFNAG	159.604	3.272	DEFNAG	153.581	3.306	DEFZUL	158.911	3.157
DEGMEL	158.11	3.43	DEGMEL	175.976	3.354	DEGMEL	152.743	2.88	DEGMEL	163.005	2.899	DEGMEL	162.515	3.141
DEGVER	166.899	3.468	DEGVER	173.456	3.348	DEGMEL	168.796	2.899	DEGROZ	163.199	3.205	DEGROZ	164.463	3.452
DEGVIV	156.197	2.875	DEGVIV	159.248	2.771	DEGVER	156.553	3.18	DEGVIV	156.805	2.838	DEGVIV	151.949	2.978
DEGVIV	156.254	3.318	DEGVIV	175.559	3.061	DEGVIV	160.829	2.867	DEGVIV	154.276	2.998	DEGVIV	163.33	2.703
DEHLIM	157.934	3.108	DEHQUD	153.144	3.32	DEGWAN	157.57	3.131	DEGWAN	152.073	3.131	DEGWER	160.054	3.255
DEHWIW	150.27	3.354	DEJDAX	161.892	2.984	DEHQUD	156.912	2.843	DEHWIW	152.285	3.273	DEHWIW	164.498	3.156
DEJGOR	169.427	3.254	DEJGOR	171.124	3.254	DEJDAX	154.081	3.01	DEJGOR	165.385	3.198	DEJGOR	165.904	3.198
DEJGOR	173.495	3.142	DEJHEH	160.236	3.085	DEJGOR	159.416	3.186	DEJGOR	164.629	3.186	DEJGOR	162.423	2.839
DEJHEH	162.974	3.07	DEJHEH	169.409	3.462	DEJHEH	163.34	3.416	DEJHEH	166.284	3.264	DEJHEH	173.655	2.931
DEJLAH	150.448	3.24	DEJXEY	168.735	3.361	DEJHEH	164.918	3.309	DEJLAH	157.217	3.24	DEJLAH	166.118	3.404
DEKQIW	171.546	3.364	DEKXID	160.466	3.068	DEJXEY	167.897	2.986	DEKHAF	176.474	3.438	DEKHAF	158.076	3.26
DEKYEB	166.129	2.72	DEKYEB	175.037	3.076	DEKYEB	172.877	2.924	DEKYEB	161.37	3.074	DEKYEB	161.556	3.044
DEKYEB	159.066	2.953	DEKYEB	152.412	2.72	DEKYEB	155.984	3.025	DEKYEB	169.585	2.885	DEKYEB	156.942	3.074
DELLIT	172.297	3.011	DELLIT	167.688	2.836	DEKYEB	152.689	3.283	DELBEE	158.601	3.062	DELBIG	155.832	3.11
DEMCIH	158.836	3.382	DEMDUU	162.638	3.366	DELPIX	170.457	2.869	DELPIX	154.217	3.493	DEMCIH	164.773	3.251
DEMRAR	154.314	3.354	DEMRAR	158.422	3.33	DEMDUU	159.727	3.489	DEMNIT	152.72	3.158	DEMNIT	167.031	3.331
DEMYAY	150.505	3.136	DEMYAY	153.46	3.007	DEMRAR	161.609	3.355	DEMSEW	150.088	3.35	DEMYAY	154.565	3.45
DENFOV	175.518	3.079	DENFOV	157.28	3.35	DEMYAY	164.027	2.76	DEMYAY	157.898	3.361	DENFEI	158.606	3.419
DENJEP	172.609	3.43	DENJEP	178.368	2.835	DENFOV	153.495	3.465	DENHAG	157.102	3.488	DENJEP	152.956	3.025
DEPHAK	163.611	3.337	DEPHOZ	150.149	2.783	DENJEP	172.687	3.189	DENMIT	153.548	3.355	DENXEZ	150.681	2.957
DEPMUJ	151.413	3.059	DEPPOG	153.275	3.389	DEPMUJ	153.95	3.435	DEPMUJ	162.567	3.175	DEPMUJ	159.659	3.171
DEQDIP	170.771	2.94	DEQDIP	150.09	3.347	DEPPOH	150.298	3.184	DEQDIP	158.459	2.732	DEQDIP	167.19	2.665
DEQLOE	163.78	3.156	DEQPOF	156.166	3.3	DEQDIP	150.101	2.634	DEQLOE	150.393	3.156	DEQLOE	162.133	3.094
DEQPUL	161.094	3.42	DEQPUL	157.38	3.421	DEQPOF	164.488	3.308	DEQPOF	150.02	3.472	DEQPOF	153.285	3.004
DEQSOH	160.547	3.18	DEQXAY	168.081	3.338	DEQPUL	162.524	3.243	DEQPUL	160.249	3.42	DEQPUL	166.033	3.406
DEQXEC	162.27	3.386	DEQXIG	152.148	2.98	DEQXAY	161.153	3.293	DEQXAY	170.794	3.041	DEQXEC	153.85	3.028
DERGAL	158.687	2.952	DERGIT	163.913	3.432	DEQXIG	160.185	3.183	DEQXIG	162.428	3.463	DERFEP	175.573	3.232
DERXEH	160.334	2.919	DERXEH	167.137	3.16	DERGIT	171.227	2.975	DERGIT	165.769	3.474	DERGUG	175.505	3
DESSIG	161.275	2.753	DESZIN	167.234	3.051	DERXEH	166.773	3.182	DERXEH	166.652	3.161	DESFOZ	158.856	2.878
DETBIQ	169.39	2.864	DETBIQ	169.969	3.18	DESZIN	165.949	2.824	DESZIN	155.051	3.035	DESZIN	171.972	2.852
DETBOW	167.986	2.687	DETBOW	153.161	3.39	DETBIQ	150.981	3.264	DETBIQ	174.484	3.482	DETBIQ	170.176	3.328
DEVCOZ	166.322	2.921	DEVCOZ	161.145	3.205	DETBOW	156.09	3.078	DETBUC	161.504	3.09	DEVCOZ	152.809	3.365
DEVFOZ	171.043	3.219	DEVFOZ	166.218	3.033	DEVCOZ	158.046	3.464	DEVCUF	167.741	2.797	DEVDOA	151.654	3.462
DEWDOC	163.005	2.829	DEWHUM	154.513	3.209	DEVMEX	168.546	2.773	DEVMEX	151.926	3.048	DEVPAY	166.286	2.872
DEXCIU	150.156	3.383	DEXCIU	156.625	2.924	DEWHUM	152.162	2.721	DEWHUM	153.19	3.266	DEWPAW	158.823	3.064
DEXFAQ	152.211	2.952	DEXHOG	164.868	3.429	DEXCIU	162.502	3.265	DEXCIU	158.734	2.821	DEXDUI	173.948	3.267
DEXRIL	157.871	2.781	DEXRIL	158.152	2.781	DEXHOG	150.324	3.143	DEXPEF	156.573	3.098	DEXPUV	153.188	3.229
DEYFUM	152.247	3.108	DEYFUM	152.613	3.276	DEXRIL	170.932	3.032	DEXSOQ	166.172	3.463	DEYBER	157.162	2.903
DEYPIJ	156.642	3.2	DEYPIJ	157.788	3.159	DEYPIJ	166.394	2.99	DEYPIJ	155.507	3.072	DEYPIJ	155.838	2.651
DEYROS	161.178	2.791	DEYROS	174.104	3.141	DEYPIJ	150.3	2.877	DEYQAC	156.465	3.143	DEYROS	168.159	3.473
DEYSUZ	150.046	2.87	DEYSUZ	155.81	3.028	DEYSEJ	161.737	2.64	DEYSOT	169.752	3.367	DEYSOT	167.479	2.462
DEYTIO	156.491	2.575	DEYTUA	155.098	2.709	DEYTAG	161.155	2.606	DEYTAG	151.345	2.878	DEYTIO	161.215	3.378
DEYVIQ	155.746	3.454	DEYVIQ	154.849	2.588	DEYVAI	154.413	2.606	DEYVAI	156.588	2.606	DEYVAI	150.777	2.733
DEYWEN	152.323	2.802	DEZJUP	155.688	3.014	DEYWEN	152.166	2.858	DEYWEN	154.477	2.831	DEYWEN	152.813	3.087
DEZJUP	167.251	3.44	DEZJUP	160.322	2.877	DEZJUP	151.368	3.201	DEZJUP	150.374	2.706	DEZJUP	162.761	2.661
DEZJUP	150.632	3.236	DEZJUP	171.588	3.314	DEZJUP	164.073	2.981	DEZJUP	159.767	2.706	DEZJUP	164.225	3.412
DFCBDI	152.98	3.351	DFCHFE	161.465	2.858	DEZPIK	152.544	2.909	DEZYEP	153.054	3.2	DFCBDI	153.451	2.799
DFTNBD	177.191	3.267	DIBBOI	155.354	3.469	DFCHFE	162.299	3.235	DFNAPQ	163.117	3.4	DFPPTP	153.969	2.735
DIBBUL02	167.955	3.36	DIBBUL03	154.015	3.096	DIBBOI	177.019	3.054	DIBBOI	154.347	3.228	DIBBUL01	167.893	3.358
DIBBUO	153.985	3.202	DIBBUO	153.581	3.494	DIBBUL03	157.68	3.496	DIBBUL03	152.273	3.174	DIBBUO	174.836	3.08
DIBDEX	155.48	3.322	DIBDEY	155.324	3.071	DIBCAV	154.284	3.15	DIBCAV	154.547	3.491	DIBCAV	174.997	3.079
DIBDUO	155.273	2.943	DIBDUO	159.806	3.463	DIBDEY	158.307	3.429	DIBDOI	158.949	2.812	DIBDOI	165.494	3.273
DIBKUU	168.488	2.954	DIBKUU	158.792	3.377	DIBFED	151.345	3.307	DIBFED	173.87	3.286	DIBFED	152.583	3.372
DIBSUD	158.617	3.36	DICFOL	150.006	3.276	DIBQEL	157.479	3.447	DIBQEL	167.39	3.23	DIBQEL	152.333	3.19
DICFUR	153.22	3.361	DICHOQ	172.132	2.699	DICFOL	171.771	3.276	DICFOL	158.086	3.329	DICFOL	153.458	3.126
DICHOQ	156.221	2.978	DICHOQ	153.789	3.466	DICHOQ	156.436	2.699	DICHOQ	157.641	2.881	DICHOQ	159.895	2.919
DICHOQ	154.478	2.886	DICHOQ	169.414	2.938	DICHOQ	150.645	2.902	DICHOQ	151.194	2.992	DICHOQ	155.774	2.846
DICJAE	167.797	3.197	DICJAE	158.516	3.184	DICHOQ	150.183	3.01	DICJAE	154.135	2.682	DICJAE	161.055	3.17
DICJAE	151.565	3.197	DICJAE	160.759	3.255	DICJAE	154.753	3.218	DICJAE	159.301	2.938	DICJAE	162.118	3.477
DICPOY	167.625	3.292	DICPOY	158.433	3.23	DICKUY	167.398	3.127	DICPOY	168.256	3.36	DICPOY	159.171	3.281
DICPOY	156.357	3.161	DICPOY	172.883	3.165	DICPOY	174.935	3.06	DICPOY	158.565	2.805	DICPOY	151.347	2.955
DIDJAD	154.329	3.227	DIDJAD	156.533	2.976	DICQUE	159.197	2.939	DICREO	152.941	3.383	DIDJAD	152.051	3.365
DIDJAD	161.429	3.114	DIDJAD	164.254	2.912	DIDJAD	154.057	3.49	DIDJAD	153.124	3.13	DIDJAD	161.97	2.813
DIDJAD	158.726	2.812	DIDJAD	159.328	3.041	DIDJAD	163.224	2.811	DIDJAD	160.123	3.113	DIDJAD	150.958	2.906
DIDMEI	162.641	2.904	DIDMEI	159.839	2.841	DIDJAD	161.762	3.113	DIDJAD	170.06	2.922	DIDMEI	154.239	3
DIDMEI	163.836	3.152	DIDQAK	153.26	2.947	DIDMEI	161.979	3.137	DIDMEI	154.25	3.338	DIDMEI	161.475	2.841
DIDQAK	167.059	3.352	DIDQAK	162.583	2.825	DIDQAK	153.343	3.052	DIDQAK	165.505	3.052	DIDQAK	158.65	3.312
DIDQAK	150.634	3.016	DIDQAK	158.914	3.155	DIDQAK	162.093	2.645	DIDQAK	169.024	3.014	DIDQAK	152.537	2.867
DIDQAK	168.427	2.517	DIDQAK	153.185	3.098	DIDQAK	167.111	3.31	DIDQAK	158.247	3.136	DIDQAK	150.347	3.466

DIDQAK	168.817	2.728	DIDQAK	160.887	3.193	DIDQAK	163.555	2.975	DIDQAK	168.918	3.204	DIDQAK	173.524	2.676
DIDQAK	164.796	2.844	DIDQAK	162.893	3.029	DIDQAK	159.132	3.413	DIDQAK	164.201	2.728	DIDQAK	159.175	2.988
DIDQAK	153.841	3.193	DIDTUF	150.796	3.267	DIDQAK	162.969	2.825	DIDQAK	150.344	3.333	DIDQAK	150.186	3.185
DIDWUM	150.682	3.225	DIDYAS	150.408	3.379	DIDVEV	163.665	3.316	DIDWAR	166.51	2.898	DIDWUM	157.102	3.364
DIDZAS	153.805	3.432	DIDZIA	155.364	3.353	DIDYAS	169.132	2.928	DIDZAS	165.4	2.941	DIDZAS	155.026	2.984
DIFBAY	150.902	3.186	DIFBUQ	151.795	2.945	DIDZIA	178.555	3.105	DIDZIA	158.379	3.044	DIFBAY	168.784	3.493
DIFHIM	174.346	3.046	DIFHIM	166.385	2.926	DIFBUQ	168.335	3.128	DIFBUQ	167.519	3.168	DIFHIM	179.31	2.896
DIFLIP	169.786	3.301	DIFLIP	150.634	2.892	DIFHIM	174.609	2.926	DIFLIP	155.645	2.9	DIFLIP	176.93	3.321
DIFLIP	165.04	3.057	DIFLIP	150.299	3.293	DIFLIP	150.125	3.479	DIFLIP	162.782	2.819	DIFLIP	155.842	2.925
DIFLIP	158.245	2.886	DIFLIP	161.051	3.127	DIFLIP	150.472	2.9	DIFLIP	173.849	3.172	DIFLIP	168.659	3.387
DIFLIP	155.147	2.97	DIFLIP	167.792	3.117	DIFLIP	153.403	2.855	DIFLIP	167.045	3.029	DIFLIP	150.988	2.943
DIGMIT	171.127	3.438	DIGMIT	163.073	2.726	DIFMOV	157.239	3.106	DIGMEP	152.526	3.355	DIGMEP	160.081	3.355
DIGPAL	161.86	2.872	DIGVIZ	159.246	3.231	DIGMIT	166.295	2.998	DIGMIT	169.863	3.438	DIGPAL	173.221	2.848
DIHNUH	160.441	3.005	DIHTUM	152.552	3.21	DIGXAU	156.386	3.077	DIHCUW	156.7	2.941	DIHNEO	154.285	3.366
DIJDUY	156.806	2.78	DIJDUY	150.373	2.83	DIHVAU	163.526	3.038	DIHVOG	153.513	3.299	DIHWIB	174.488	3.216
DIJFAH	151.881	3.134	DIJFAH	153.486	3.399	DIJDUY	158.698	3.439	DIJDUZ	159.981	2.954	DIJFAH	178.993	3.085
DIJVUP	161.705	3.323	DIJWOK	157.66	3.198	DIJHEK	152.748	2.917	DIJHEK	161.685	3.157	DIJVUP	152.863	3.262
DILJOA	150.384	3.483	DILMIX	152.272	3.101	DIJWOK	167.134	3.295	DIJWOK	152.227	3.211	DILDIL	159.478	3.187
DILYIJ	163.176	3.07	DILYUV	161.156	2.969	DILPIA	160.246	2.66	DILQOH	153.766	3.174	DILVAX	172.527	3.212
DILZOQ	154.705	3.104	DILZOQ	150.451	2.655	DILZOQ	165.867	3.494	DILZOQ	176.134	3.389	DILZOQ	169.137	2.826
DILZOQ	160.531	2.842	DILZOQ	151.239	3.04	DILZOQ	151.13	2.655	DILZOQ	154.459	3.267	DILZOQ	161.234	2.655
DIMTEA	153.593	3.13	DINJUH	157.329	3.391	DIMLUG	164.21	3	DIMLUG	160.862	3.485	DIMLUG	151.662	3.216
DINVED	156.775	3.236	DINVED	167.963	3.45	DINKES	165.839	3.005	DINNEW	155.871	3.087	DINVED	161.993	3.032
DINVED	157.929	3.45	DINVED	176.273	2.811	DINVED	171.11	2.785	DINVED	172.302	3.303	DINVED	162.011	3.288
DINVED	168.311	3.288	DINVED	158.558	3.235	DINVED	152.039	3.146	DINVED	150.122	2.782	DINVED	171.4	2.794
DIPDEP	154.196	2.838	DIPDEP	161.572	3.39	DINVED	160.625	2.954	DINVED	174.181	3.396	DIPDEP	167.046	3.402
DIPDUF	162.253	2.905	DIPDUF	156.055	3.317	DIPDIT	167.417	3.059	DIPDIT	169.485	2.935	DIPDIT	167.827	3.272
DIPDUF	171.327	3.372	DIPDUF	154.879	3.263	DIPDUF	167.599	2.865	DIPDUF	155.558	2.868	DIPDUF	155.331	2.935
DIPJIX	165.312	3.36	DIPKEW	174.215	3.137	DIPDUF	151.5	3.052	DIPDUF	156.884	2.937	DIPDUF	161.124	3.18
DIPKIA	167.614	3.091	DIPKOG	173.729	3.135	DIPKEW	150.373	3.035	DIPKEW	167.375	3.081	DIPKIA	174.184	3.124
DIPREB	167.468	3.403	DIPREB	172.325	2.886	DIPKOG	167.229	3.084	DIPLIB	160.019	3.442	DIPREB	167.981	3.443
DIQDIU	150.378	3.081	DIQDIU	162.254	2.78	DIPTEE	156.481	2.993	DIPXAE	161.936	3.121	DIQBAI	161.64	3.103
DIRWOS	155.872	3.072	DIRWOS	171.88	3.373	DIQDIU	173.2	3.481	DIQDIU	157.472	2.78	DIRFIW	172.812	3.005
DIRWOS	150.514	3.307	DIRWOS	153.688	3.313	DIRWOS	161.784	3.437	DIRWOS	160.325	2.998	DIRWOS	154.026	3.102
DIRWOS	153.101	3.338	DIRWOS	154.492	3.145	DIRWOS	162.922	3.206	DIRWOS	153.556	3.481	DIRWOS	158.965	3.002
DISFES	158.152	3.159	DISFES	153.662	3.373	DIRWOS	158.832	3.114	DIRWOS	163.987	3.114	DISFES	150.015	2.954
DISFES	151.986	2.937	DISFES	155.799	3.328	DISFES	151.536	2.833	DISFES	159.198	2.8	DISFES	164.796	3.32
DISFOC	156.291	3.5	DISFOC	150.85	3.358	DISFES	169.949	3.259	DISFOC	160.458	3.038	DISFOC	154.403	3.358
DISPEE	152.664	3.201	DISPEE	151.814	3.215	DISFOC	155.952	3.1	DISFOC	151.172	2.891	DISFOC	156.699	3.08
DISQIG	173.869	3.008	DISQIG	159.844	3.093	DISPEE01	154.36	3.249	DISPEE01	153.331	3.296	DISQAB	161.322	3.456
DISSIJ	166.627	3.333	DISSIJ	154.958	2.981	DISQIG	154.546	2.895	DISRUU	153.138	3.196	DISRUU	154.115	3.275
DISTOS	168.294	3.404	DISTUY	163.536	3.005	DISTIM	150.801	3.023	DISTIM	162.531	3.185	DISTIM	169.494	3.004
DITLAV	150.335	2.786	DITLAV	151.195	3.485	DITBOB	158.35	3.122	DITBOB01	157.49	3.065	DITCES01	152.253	3.128
DITQEC	159.044	3.25	DITQEC	153.512	3.371	DITLEZ	151.476	3.18	DITQEC	168.591	3.074	DITQEC	151.689	3.014
DITXUB	169.617	3.155	DIVDAO	152.191	3.146	DITQED	152.853	2.993	DITXUB	166.925	3.185	DITXUB	153.989	2.882
DIVLOL	169.541	3.458	DIVLOL	176.236	3.423	DIVGUN	167.784	3.107	DIVLOL	161.276	3.039	DIVLOL	151.377	3.311
DIVMIG	151.049	3.487	DIVMIG	161.118	2.972	DIVMEC	158.912	2.853	DIVMIG	160.005	3.359	DIVMIG	173.089	3.149
DIWFEX	153.344	2.984	DIXMUW	150.736	3.331	DIVRIM	167.236	3.318	DIVROS	150.27	3.063	DIVROS01	152.55	3.096
DIXZUH	150.127	2.756	DIXZUH	160.225	3.303	DIXSAG	152.657	3.258	DIXXIR	153.563	3.369	DIXXIR	152.866	3.472
DIYPUX	153.684	3.143	DIYYAM	170.24	3.422	DIYBAQ	170.971	3.251	DIYBAQ	157.727	2.986	DIYMEH	164.256	2.877
DIYYAM	161.52	3.452	DIYYAM	157.055	2.851	DIYYAM	165.879	2.879	DIYYAM	151.164	3.006	DIYYAM	157.533	2.955
DIYYAM	168.433	3.022	DIYYAM	168.825	3.087	DIYYAM	169.46	2.879	DIYYAM	160.484	2.849	DIYYAM	168.449	3.39
DIZXAP	150.325	3.285	DOBDAC	160.985	3.476	DIZLED	171.228	3.165	DIZWES	154.635	3.463	DIZXAP	152.527	2.722
DOBDEG	154.894	2.756	DOBDEG	164.172	2.97	DOBDAC	178.413	2.915	DOBDAC	162.51	3.393	DOBDAC	158.349	3.393
DOBDEG	150.865	3.491	DOBDEG	156.074	2.874	DOBDEG	153.338	2.874	DOBDEG	170.196	3.125	DOBDEG	171.436	3.093
DOBDOQ	150.32	3.478	DOBFIM	175.124	3.208	DOBDOQ	160.964	2.701	DOBDOQ	152.455	2.87	DOBDOQ	156.218	2.834
DOBPAP	159.091	3.003	DOBTOF	158.074	2.834	DOBFIM	171.947	3.022	DOBPAP	157.322	3.399	DOBPAP	153.701	2.931
DOCBON	155.336	3.117	DOCBON	159.007	3.279	DOBTOF	151.678	2.756	DOBTOF	151.049	2.834	DOCBON	164.268	3.296
DOCBON	167.659	3.006	DOCBON	153.968	3.322	DOCBON	150.792	3.3	DOCBON	156.257	3.018	DOCBON	169.082	3.279
DOCCEF0	150.954	2.893	DOCCEF0	153.284	3.191	DOCBON	150.132	3.173	DOCCEF	151.35	2.959	DOCCEF	151.055	3.191
DOCCUV	163.465	2.922	DOCFUA	159.904	3.09	DOCCOP	151.247	3.214	DOCCOP	150.965	3.027	DOCCUV	163.126	2.952
DOCKEO	174.108	2.997	DOCKEO	164.137	2.884	DOCKEO	157.373	2.989	DOCKEO	175.684	2.968	DOCKEO	173.485	3.323
DOCREW	163.123	2.798	DOCREW	175.857	2.798	DOCKEO	154.44	3.314	DOCKEO	155.051	3.392	DOCKEO	154.897	3.391
DODBOR	172.617	3.462	DODCEH	162.951	2.643	DOCREW	161.057	2.804	DOCREW	160.48	3.442	DOCREW	154.013	3.193
DODMUF	158.528	3.151	DODMUF	177.122	3.151	DODCEH	163.667	2.643	DODCEH	171.077	3.047	DODCEH	153.135	3.26
DODRET	168.969	3.273	DODRET	160.6	3.236	DODNIX	150.355	3.478	DODQAQ	157.49	3.083	DODQEU	172.688	3.347
DODSAS	156.228	3.379	DODVUP	150.804	3.134	DODRET	168.371	3.461	DODSAS	150.314	2.888	DODSAS	151.4	2.932
DODXOL	153.455	3.206	DODXOL	163.549	3.206	DODXOL	164.943	3.154	DODXOL	168.833	3.21	DODXOL	161.651	3.136
DODZAX	161.216	3.208	DOFPAR	154.074	2.737	DODXOL	165.117	3.213	DODYUQ	162.316	3.031	DODYUQ	171.068	3.449
DOFXAX	150.378	3.042	DOFXAX	155.049	2.949	DOFXAX	164.731	3.212	DOFXAX	153.227	3.223	DOFXAX	156.991	2.904
DOGGIR	153.911	3.04	DOGGIR	169.508	3.275	DOFXON	157.883	3.151	DOFXOO	156.975	3.369	DOFXUT	154.497	2.703
DOGLET	171.761	3.16	DOGLET	161.251	3.233	DOGJEP	152.263	3.084	DOGJIT	162.431	3.131	DOGKUF	174.693	2.881
DOGVON	150.115	2.818	DOGWAA	158.846	3.223	DOGLET	153.475	3.434	DOGSEZ0	155.997	3.327	DOGVON	155.119	3.289
DOHKIU	168.635	2.952	DOHKUJ	165.349	3.364	DOGXAB	155.289	3.355	DOHHIV	159.613	3.352	DOHKIU	163.986	3.331
DOHVED0	152.766	3.068	DOHVED0	154.795	3.067	DOHKUJ	168.343	3.381	DOHNOD	178.721	3.261	DOHVED	154.876	3.067
DOJQUR	151.984	3.426	DOJQUR	152.624	3.342	DOJDEN	151.073	2.918	DOJLEX	169.6	2.874	DOJQUR	151.189	3.019
DOJXEG	153.978	3.025	DOKBEM	155.5	3.259	DOJSAX	165.061	3.43	DOJSAX	161.617	3.257	DOJSAX	161.224	3.29
DOKCIQ	153.43	2.731	DOKCIQ	172.381	2.88	DOKBEM	169.287	3.235	DOKCAI	167.092	2.646	DOKCIQ	155.25	2.917
DOKCOW	171.383	3.099	DOKCOW	150.976	3.248	DOKCIQ	155.211	2.752	DOKCIQ	162.355	3.076	DOKCIQ	153.638	2.752

DOKCOW	158.374	3.261	DOKCOW	155.791	2.842	DOKCOW	159.07	2.8	DOKCOW	165.49	3.378	DOKCOW	162.326	3.461
DOKNOI	158.617	3.226	DOKPOJ	160.888	3.196	DOKHUJ	161.838	3.425	DOKJIY	158.193	3.48	DOKNOI	158.91	2.855
DOKWIM	163.152	3.175	DOKWIM	162.409	3.166	DOKVEF	158.966	3.286	DOKVEF	160.85	3.208	DOKWAE	165.67	3.452
DOKWUY	154.573	2.819	DOKWUY	160.599	2.839	DOKWIM	151.599	3.455	DOKWIM	163.005	2.961	DOKWOS	153.204	3.499
DOLDIT	151.495	3.311	DOLDIT	155.334	3.179	DOKWUY	152.316	2.819	DOKXAF	157.972	3.471	DOKXAF	153.156	3.367
DOLDIT01	153.232	3.118	DOLDIT01	155.312	3.178	DOLDIT	154.944	3.188	DOLDIT	153.255	3.117	DOLDIT01	151.451	3.311
DOLDIT02	153.343	3.128	DOLDIT02	155.483	3.176	DOLDIT01	154.956	3.188	DOLDIT02	150.144	3.36	DOLDIT02	151.488	3.315
DOLKIB	150.996	3.488	DOLLEY	161.896	2.986	DOLDIT02	154.997	3.188	DOLHUL0	160.201	3.19	DOLKIB	162.147	3.488
DOLSIJ	154.791	2.946	DOLSIJ	159.564	2.748	DOLPON	162.135	3.057	DOLSAB	152.681	3.398	DOLSAB	157.329	2.714
DOLVAE	164.098	2.837	DOLVAE	167.768	2.821	DOLSOP	170.234	3.18	DOLVAE	156.497	2.69	DOLVAE	166.935	3.039
DOMCOA	153.551	2.753	DOMDAO	150.088	3.4	DOLVAE	160.266	3.313	DOLVAE	154.528	2.837	DOMBOW	172.82	2.757
DOMFOE	151.869	3.11	DOMFOE	171.817	3.107	DOMDAO	170.992	2.991	DOMDIS1	150.473	3.321	DOMFIY	171.923	3
DOMJOG	179.599	2.882	DOMJOG	153.52	3.29	DOMHOG	171.933	3.447	DOMJIA	159.25	3.062	DOMJIA	160.11	3.335
DOMMIF	151.642	3.097	DOMMIF	166.018	2.951	DOMJOG	150.685	3.126	DOMJOG	167.757	3.321	DOMJOG	170.556	3.094
DOMVAE	173.256	3.134	DOMVAE	156.318	3.322	DOMSOQ	155.293	3.164	DOMTAB	164.194	3.466	DOMTAD	158.646	3.442
DOMWUY	154.535	2.851	DONCAK	165.83	3.012	DOMWUY	160.637	3.015	DOMWUY	150.906	3.12	DOMWUY	159.664	3.24
DONPUU	155.916	3.124	DONYUB	151.325	3.01	DONLID	168.498	3.278	DONMAX	160.012	2.941	DONMOL	176.229	3.421
DOPFAT	155.822	2.853	DOPFAT	169.706	3.151	DOPBIT	174.63	3.153	DOPBIT	154.778	3.334	DOPBIT	175.538	2.891
DOPSOR	153.369	3.019	DOPSOR	165.679	3.119	DOPQIM	153.848	3.039	DOPSOR	151.265	3.044	DOPSOR	154.065	3.457
DOQGUN	157.42	3.411	DOQGUN	166.929	3.318	DOPSOR	150.069	3.193	DOQGOG	160.336	3.212	DOQGUN	170.007	2.911
DOQJUR	167.131	3.233	DOQMED	164.412	2.81	DOQHAU	152.364	3.231	DOQHAU	150.931	2.622	DOQHAU	153.053	3.085
DOQMOL	155.962	2.77	DOQMOL	152.376	2.835	DOQMIF	162.988	3.112	DOQMIF	150.544	2.998	DOQMOL	158.036	2.925
DOQXEO	156.788	3.236	DOQXEO	171.566	3.002	DOQMOL	160.826	3.448	DOQSOS	155.545	3.212	DOQTOW	162.54	3.378
DORMOQ	161.83	3.378	DORREJ	162.467	3.15	DOQXEO	176.435	2.737	DORMOQ	156.83	3.27	DORMOQ	152.633	3.475
DORSIO	168.423	3.188	DORSOU	161.897	3.048	DORSIO	150.209	3.208	DORSIO	155.056	3.188	DORSIO	168.974	3.373
DORWAJ	166.719	3.023	DORXAK	156.33	3.144	DORSUA	165.624	3.025	DORSUA	168.453	2.719	DORSUA	152.379	3.427
DOSBET	152.93	2.986	DOSBET	163.461	3.038	DOSBET	158.576	3.488	DOSBET	166.716	3.251	DOSBET	158.078	3.333
DOSCUM	154.471	3.151	DOSCUM	165.913	3.272	DOSBET	174.085	3.297	DOSCUM	156.691	3.079	DOSCUM	169.757	2.982
DOSRAF	154.886	3.33	DOTDAU	155.117	3.381	DOSCUM	152.538	3.162	DOSCUM	166.157	2.91	DOSLED	158.063	3.444
DOTDIA	150.123	3.291	DOTDIA	164.813	3.263	DOTDAU	167.222	3.434	DOTDAU	154.308	3.434	DOTDIA	152.642	3.347
DOTPEK	163.13	3.064	DOTPEK	153.239	2.774	DOTMIJ	161.384	3.03	DOTPEK	161.447	3.036	DOTPEK	157.542	3.323
DOVHUT	172.506	3.284	DOVJEF	171.045	2.927	DOTPEK	152.294	2.825	DOTPEK	174.311	2.806	DOVHUT	178.009	3.45
DOVJIJ	158.467	3.485	DOVJOP	164.193	3.274	DOVJEF	164.64	3.379	DOVJIJ	162.874	2.962	DOVJIJ	157.129	3.211
DOVZUJ0	159.873	3.28	DOWMEH	160.542	3.425	DOVJOP	166.867	3.435	DOVJOP0	163.791	2.902	DOVZAR0	170.849	3.296
DOWMIN	157.28	3.301	DOWPIQ	167.115	3.233	DOWMEH	178.821	2.859	DOWMIN	173.887	3.074	DOWMIN	167.279	3.228
DOXYUL	155.483	3.468	DOYHUY	156.603	3.037	DOWQUD	157.875	3.094	DOWVOE	163.48	3.414	DOXFAZ	173.475	3.245
DOYJIM	177.519	2.885	DOYNEM	168.209	3.177	DOYHUY	150.849	2.874	DOYHUY	170.568	3.186	DOYJIM	159.668	3.271
DOYNEM	151.28	3.472	DOYNIQ	152.176	2.951	DOYNEM	161.953	3.047	DOYNEM	154.868	3.177	DOYNEM	156.698	3.03
DOZFIJ	162.143	3.428	DOZGUW	156.751	2.986	DOYPEM	150.106	3.177	DOYTOE	153.11	2.989	DOYZIC	159.942	3.415
DOZKEJ	164.301	3.442	DOZMEN	150.534	3.1	DOZGUW	172.579	2.876	DOZGUW	174.016	3.1	DOZGUW	166.897	2.92
DPFPPO	155.113	2.687	DPFPPO	153.189	3.172	DOZMEN	160.604	3.102	DOZQER	157.658	2.895	DOZQIV	152.891	3.37
DUBBAF	166.883	3.223	DUBPUO	172.926	2.971	DTFMHG0	162.421	3.145	DTFNPS	169.421	3.269	DTFNPS	167.3	3.269
DUCMIA	156.013	3.038	DUCPUO	153.237	3.409	DUBVUU	157.117	3.249	DUBXIH	150.777	3.427	DUBXIH	166.534	3.084
DUCTOO	173.778	3.47	DUCTOO	155.007	3.089	DUCPUO	157.339	2.995	DUCPUO	160.119	3.082	DUCPUO	151.092	2.831
DUDPID	175.251	2.841	DUDPOJ	155.842	3.347	DUCWEH	153.94	3.337	DUDDEP	165.523	2.955	DUDPID	155.828	3.333
DUDQAW	153.503	2.499	DUFGAN	157.733	3.415	DUDPOJ	175.712	2.847	DUDPUP	161.692	3.017	DUDPUP	152.161	2.554
DUFHAO	164.99	3.312	DUFHIW	159.978	3.241	DUFGER	152.101	3.224	DUFGIV	175.475	2.821	DUFGUH	165.798	3.221
DUFPUR	166.589	3.16	DUFPUR	151.457	2.636	DUFHIW	155.771	2.659	DUFHIW	158.594	3.245	DUFKEX	167.954	2.918
DUFREF	164.392	2.92	DUFREF	152.411	3.185	DUFREF	155.855	2.889	DUFREF	160.92	2.979	DUFREF	157.342	2.957
DUFREF	150.267	3.046	DUFREF	160.058	2.859	DUFREF	164.414	2.979	DUFREF	151.219	3.046	DUFREF	153.831	3.043
DUFWOR	155.054	3.358	DUFYUC	153.867	3.411	DUFREF	152.366	3.191	DUFREF	159.349	2.92	DUFREF	171.373	2.859
DUGDIT	174.255	3.144	DUGGEV	156.257	2.875	DUFYUC	163.407	3.262	DUGDEP	151.057	3.034	DUGDES	152.218	3.351
DUGNIF	155.229	3.266	DUGYIQ	165.056	2.67	DUGGEV	152.419	3.294	DUGMEY	156.407	3.049	DUGMEY	159.617	3.492
DUGYUC	164.018	3.135	DUGYUC	166.494	3.447	DUGYUC	165.338	3.205	DUGYUC	158.65	3.195	DUGYUC	169.664	3.446
DUHBUF	168.918	2.814	DUHDAP	154.552	3.151	DUGYUC	151.88	3.057	DUHBOZ	162.286	3.143	DUHBUF	160.595	3.152
DUHMEA	165.586	2.808	DUHMOK	155.172	2.86	DUHDAP	158.156	3.124	DUHKIE	160.326	3.316	DUHMEA	154.497	3.479
DUHQUT	157.635	3.184	DUHSEF	179.743	2.928	DUHMOK	173.419	2.776	DUHNAX	153.024	2.973	DUHNEB	152.389	3.003
DUJJUP	154.297	2.733	DUJJUP	150.403	3.042	DUJJUP	154.106	3.347	DUJJUP	160.972	2.856	DUJJUP	168.391	3.029
DUJJUP	161.313	3.373	DUJJUP	158.699	2.982	DUJJUP	153.326	2.982	DUJJUP	163.786	2.856	DUJJUP	174.671	2.884
DUJKEA	159.297	3.265	DUJKEA	154.105	2.829	DUJKEA	165.621	2.856	DUJKEA	164.167	3.388	DUJKEA	163.346	2.856
DUJKIE	163.024	3.311	DUJKOL	174.061	3.002	DUJKEA	154.051	2.881	DUJKIE	166.111	3.266	DUJKIE	175.147	2.785
DUJXEL	165.89	2.953	DUJXEL	158.182	2.987	DUJKOL	154.61	3.444	DUJKOL	150.455	3.279	DUJNIH	167.646	2.819
DUJXIR	167.448	3.309	DUKNAY	155.445	3.403	DUJXEL	170.017	2.953	DUJXEL	156.877	3.175	DUJXEL	153.259	2.987
DUKPUV	177.845	3.238	DUKPUV	152.875	3.215	DUKNAY	155.955	2.896	DUKNAY	157.025	3.369	DUKPUV	162.25	2.799
DUKWIQ	159.98	2.879	DUKWIQ	161.472	2.891	DUKPUV	150.175	3.113	DUKSEK	159.777	3.302	DUKTUB	154.835	3.077
DUKYAJ	152.351	3.089	DUKYAJ	159.036	3.089	DUKXEQ	153.055	2.979	DUKXEQ	172.963	2.922	DUKXEQ	159.097	3.445
DULBOF	154.026	2.602	DULBOF	150.052	3.41	DUKZER	158.55	3.075	DUKZER	160.62	3.227	DUKZER	159.632	2.912
DULDET	167.317	3.227	DULDET	164.112	2.873	DULBOF	160.349	3.387	DULBOF	162.588	2.602	DULBOF	154.267	3.037
DULHIE	170.335	3.029	DULMUW	157.998	3.122	DULDET	172.049	3.445	DULDET	161.538	2.873	DULFEZ	160.831	3.016
DULXUF	154.174	3.358	DUMDIC	154.149	3.072	DULQUA	174.238	3.17	DULWEO	155.729	3.037	DULWEO	164.374	3.188
DUMDIC	175.992	3.072	DUMDIC	150.422	2.949	DUMDIC	168.037	3.385	DUMDIC	156.776	2.774	DUMDIC	152.104	3.335
DUMDOI	159.995	2.853	DUMDOI	156.983	2.972	DUMDOI	163.109	3.312	DUMDOI	156.75	3.231	DUMDOI	152.549	2.853
DUMFOK	165.116	3.139	DUMFOK	163.037	3.086	DUMDOI	158.477	3.215	DUMDOI	169.938	3.216	DUMFOK	153.034	3.372
DUMKEF	167.152	3.253	DUMKEF	167.796	2.984	DUMFOK	155.49	3.264	DUMJII	177.138	3.043	DUMJII	161.669	3.129
DUMRIN	172.12	3.38	DUMTUA	163.479	3.104	DUMKEF	154.203	3.163	DUMKEF	154.48	3.253	DUMKEF	151.33	3.163
DUMYET	154.92	3.426	DUMYET	166.65	3.015	DUMTUA	172.65	3.119	DUMTUA	157.957	3.32	DUMYET	163.317	3.002
DUNMUX	166.779	2.796	DUNQOW	150.303	3.152	DUNCIY	166.677	2.804	DUNJED	170.275	3.32	DUNMUX	163.046	3.096
DUPHED	157.515	2.819	DUPPEL	161.184	3.35	DUNQOW	153.238	3.103	DUNXES	164.117	3.466	DUNXES	162.532	3.471
DUPQEN	153.304	2.936	DUPQEN	157.383	3.469	DUPQAJ	155.047	2.926	DUPQAJ0	155.343	2.928	DUPQEN	157.249	2.869

DUPQEN	155.789	3.057	DUPQEN	163.31	3.235	DUPQEN	159.611	2.933	DUPQEN	167.874	3.057	DUPQEN	151.216	3.277
DUPQEN	174.183	2.869	DUQBOJ	152.86	2.915	DUPQEN	159.852	3.05	DUPQEN	165.819	2.989	DUPQEN	156.777	3.395
DUQCEA	170.709	2.807	DUQDUS	166.886	2.877	DUQBOJ	150.257	2.931	DUQBOK	164.256	3.203	DUQCAW	162.616	2.86
DUQRIU	158.911	3.391	DUQRIU	173.118	2.841	DUQRIU	163.882	2.961	DUQRIU	169.839	2.919	DUQRIU	166.667	2.841
DUQZIA	161.408	3.115	DUQZIA	150.286	2.875	DUQRIU	155.743	3.46	DUQXAS	156.994	3.43	DUQXAS	175.876	3.085
DURKUY	167.923	3.314	DURKUZ	151.639	2.815	DURBAV	167.906	3.373	DURKUY	156.017	3.426	DURKUY	151.146	3.005
DURKUZ	162.544	3.397	DURLUB	170.683	3.3	DURKUZ	175.17	2.815	DURKUZ	154.274	2.76	DURKUZ	167.928	3.428
DURQOZ	151.531	3.341	DURQOZ	164.028	3.286	DURMAI	156.362	3.126	DURQOZ	171.521	3.204	DURQOZ	155.627	3.199
DURTUH	161.469	3.052	DURVIV	152.756	3.299	DURQOZ	163.512	3.122	DURRUE	152.009	3.052	DURRUE	173.836	3.386
DUSGUW	172.362	3.122	DUSGUW	162.182	3.371	DURWAR	151.526	3.398	DUSFUT	172.45	2.981	DUSGOQ	155.29	3.086
DUSHEH	160.219	2.972	DUSKAF	170.405	3.138	DUSHAD	150.95	3.159	DUSHEH	165.07	3.375	DUSHEH	154.912	2.972
DUSKEI	174.759	3.286	DUSKEI	167.59	3.182	DUSKAF	175.274	3.379	DUSKAF	157.052	3.072	DUSKAF	172.49	3.439
DUSKIN	152.907	3.211	DUSKUZ	163.63	3.488	DUSKEI	160.138	3	DUSKEI	152.321	2.786	DUSKEI	157.068	3.286
DUSLEM	163.543	3.359	DUSNAJ	155.106	3.271	DUSKUZ	156.006	3.221	DUSLAF	175.059	3.111	DUSLEM	154.919	2.862
DUSRIV	164.012	3.359	DUSRIV	154.479	3.175	DUSNEN	154.831	2.76	DUSNIO	150.165	3.458	DUSRAN0	160.804	3.378
DUTBON	169.145	2.88	DUTJAH	153.774	3.405	DUSSEQ	163.95	2.895	DUSWEW	152.866	3.289	DUTBON	164.537	2.88
DUTPUD1	171.739	3.195	DUTPUD1	163.176	3.205	DUTPUD1	169.784	3.162	DUTPUD1	172.165	3.469	DUTPUD1	168.959	2.787
DUTPUD1	174.915	3.49	DUTPUD1	168.436	2.809	DUTPUD1	176.379	2.836	DUTPUD1	161.799	3.09	DUTPUD1	167.647	2.863
DUTREQ	158.053	3.238	DUTZIC	176.405	3.008	DUTPUD1	171.571	3.403	DUTPUD1	167.911	2.741	DUTQEP	153.002	3.141
DUVBIF	153.191	3.084	DUVLEN	172.108	3.291	DUVBEF	155.873	3.092	DUVBEF	173.513	3.49	DUVBIF	172.228	3.473
DUVRUK	165.414	3.477	DUVRUK	168.575	3.088	DUVLEN	170.113	3.016	DUVNAN	165.889	2.874	DUVRUK	160.237	2.936
DUVRUK	167.347	2.773	DUVRUK	155.159	2.773	DUVRUK	155.839	2.737	DUVRUK	156.144	2.963	DUVRUK	168.49	3.408
DUWLUD	166.371	3.402	DUWLUD	173.853	2.942	DUVRUK	162.117	2.638	DUVRUK	173.474	2.974	DUVRUK	151.341	2.963
DUXRUL	168.52	2.813	DUXRUL	162.762	3.476	DUWLUD	159.458	2.852	DUWPOC	174.913	3.273	DUXRUL	157.74	3.023
DUXTUN	160.401	2.784	DUXWUS	160.397	2.781	DUXRUL	150.53	3.077	DUXRUL	170.669	2.927	DUXRUL	153.832	2.976
DUYCIL	151.75	3.334	DUYCIL	167.136	3.022	DUXWUS	162.686	2.781	DUYBUU	175.133	3.152	DUYCIL	158.959	2.84
DUYGUZ	161.037	2.932	DUYGUZ	159.506	3.488	DUYGOT	154.778	3.249	DUYGOT	153.172	2.915	DUYGOT	154.986	3.141
DUYGUZ	154.46	2.888	DUYGUZ	155.199	3.19	DUYGUZ	151.021	3.398	DUYGUZ	160.689	3.011	DUYGUZ	159.18	2.672
DUYPEW	169.541	3.458	DUYPEW	152.474	2.659	DUYLAL	163.371	3.464	DUYNUI	151.637	3.217	DUYPEW	166.531	2.761
DUYWUR(	155.512	3.122	DUYWUR(	175.468	3.108	DUYPEW	172.704	3.29	DUYVIE	150.443	2.922	DUYWIH	152.11	3.293
DUYXAY	175.19	3.489	DUYXAZ	154.992	3.479	DUYWUR(	162.408	3.269	DUYXAY	151.515	3.075	DUYXAY	151.696	3.056
DUYXUS	169.034	2.824	DUYXUS	154.204	3.021	DUYXAZ	153.338	3.442	DUYXOM	170.024	3.139	DUYXUS	150.607	3.037
DUZJUD	160.692	3.378	DUZMOB	172.158	3.166	DUYXUS	166.076	3.037	DUYZEE	162.175	3.461	DUYZEG	169.574	3.386
EABGUJ	161.187	3.215	EBABER	153.083	3.209	DUZPOG	158.899	2.675	DUZQAT	170.834	2.778	DUZVAY	151.799	3.333
EBANOO	158.823	3.228	EBANOO	155.827	3.107	EBACUG	167.585	3.26	EBACUG	166.642	2.836	EBAGAS	153.775	2.874
EBAPIK	168.756	2.835	EBAPIK	154.315	3.361	EBANOO	160.792	3.004	EBANOO	150.727	3.228	EBAPIK	154.989	3.202
EBEHIG	153.087	3.457	EBEMOR	161.49	3.241	EBAPIK	154.497	3.112	EBAVOT	150.975	3.083	EBEBIZ01	152.94	3.485
EBENOP	155.143	2.938	EBENUV	155.929	3.008	EBEMOR	152.485	3.214	EBENOP	170.887	3.28	EBENOP	170.082	2.882
EBENUV	153.098	2.84	EBEZOD	150.649	2.87	EBENUV	160.408	2.994	EBENUV	177.899	3.457	EBENUV	168.847	3.397
EBIBUQ	150.119	3.037	EBIGAX	152.101	2.871	EBIBEA	154.714	3.016	EBIBEA	153.046	3.1	EBIBUQ	152.503	3.013
EBIHIH	156.512	2.847	EBIHON	165.999	3.304	EBIGAX	150.571	3.072	EBIGAX	154.999	3.034	EBIHIH	170.399	3.265
EBIJAB	169.257	3.096	EBIJEF	164.154	2.832	EBIHON	155.625	2.941	EBIHON	153.024	2.888	EBIJAB	161.766	2.627
EBIJOP	154.209	2.798	EBIJOP	151.877	3.084	EBIJEF	170.499	3.319	EBIJEF	165.336	2.832	EBIJOP	158.351	2.872
EBININ	169.798	3.179	EBIRAI	164.67	2.917	EBIJOP	173.031	3.217	EBIJOP	153.198	2.872	EBININ	168.353	3.063
EBIRAI	161.066	3.159	EBIRAI	154.597	2.792	EBIRAI	164.115	3.006	EBIRAI	158.532	3.033	EBIRAI	172.6	2.959
EBIXEW	172.946	3.162	EBIXEW	167.696	2.793	EBIXEW	161.438	2.926	EBIXEW	154.792	2.915	EBIXEW	151.623	2.881
EBIYIB	158.903	3.356	EBIYIB	154.673	2.953	EBIYEX	155.146	3.069	EBIYEX	160.241	2.976	EBIYIB	169.447	2.738
EBOBED	174.903	3.268	EBOBED	167.957	3.332	EBIYUN	153.915	2.841	EBIYUN	165.368	2.784	EBIZIZ	169.91	3.248
EBOYOM	160.199	3.499	EBOYOM	174.955	3.11	EBOBED	160.901	3.338	EBOFAF	159.398	3.135	EBOXOL	157.997	2.972
EBOYOM	168.65	2.661	EBOZEA	150.927	3.49	EBOYOM	155.697	2.82	EBOYOM	151.606	3.292	EBOYOM	157.913	2.661
EBOZUQ	152.664	3.393	EBUBAE	156.794	2.899	EBOZOK	155.142	3.251	EBOZUQ	155.37	3.346	EBOZUQ	152.828	3.021
EBUNEY	160.132	3.453	EBUQOK	151.221	3.452	EBUBAE	158.071	3.211	EBUBAE	153.356	3.357	EBUBEI	162.927	2.902
EBUYIJ	156.011	2.944	ECALEA	164.932	3.081	EBUQOK	164.104	2.852	EBUQOL	155.549	3.021	EBUYEF	153.883	2.766
ECAMEE	156.922	3.194	ECAMEE	156.975	3.238	ECALOK	153.15	3.297	ECAMEB	155.421	3.372	ECAMEB	162.678	3.405
ECAVEL	176.318	3.21	ECEBUL	157.915	3.269	ECAMUU	159.067	3.03	ECAMUU	160.668	3.22	ECAMUU	157.304	3.125
ECEDID	150.629	3.29	ECEDID	158.759	3.293	ECEDEZ	155.818	2.985	ECEDEZ	162.878	3.161	ECEDID	160.791	3.197
ECEGOL	162.39	2.802	ECERIP	167.394	2.851	ECEDID	153.395	3.485	ECEGOL	162.869	3.074	ECEGOL	151.65	2.843
ECERIP01	153.945	3.421	ECERIP01	150.184	3.001	ECERIP	163.691	2.911	ECERIP	151.955	3.408	ECERIP	152.396	3.005
ECERIP01	165.927	2.713	ECERIP01	174.021	2.793	ECERIP01	150.839	3.38	ECERIP01	164.933	3.351	ECERIP01	170.999	3.394
ECERIP01	160.346	3.324	ECESAK	152.443	2.945	ECERIP01	172.666	3.094	ECERIP01	162.041	3.204	ECERIP01	158.985	3.287
ECIGEC	164.353	3.212	ECIGIH	157.37	3.173	ECESAK	150.162	3.343	ECEYAQ	163.448	3.285	ECIGEC	167.989	3.496
ECILAF	155.763	3.496	ECINAG	162.342	2.845	ECIGIH	166.09	3.038	ECIGON	150.964	2.869	ECIJUY	158.783	2.946
ECINAG	166.21	2.892	ECINAG	155.032	2.845	ECINAG	167.055	2.985	ECINAG	157.823	3.252	ECINAG	167.723	3.206
ECINAG	170.217	2.841	ECINUB	166.927	3.155	ECINAG	162.983	2.994	ECINAG	150.312	3.044	ECINAG	168.134	2.763
ECOBIL	165.289	2.912	ECOBIL	159.269	2.856	ECIWAS	154.855	3.38	ECIWAS	175.333	3.38	ECOBIL	170.757	2.794
ECUBEJ	153.788	3.27	ECUDIQ	151.412	3.106	ECOBIL	161.877	2.752	ECOBIL	163.03	3.251	ECOBIL	154.707	2.856
ECUDOW	171.369	2.83	ECUDOW	167.491	3.471	ECUDIQ	158.158	3.361	ECUDOW	153.356	2.738	ECUDOW	151.928	3.274
ECUFUE	162.564	3.478	ECUGAL	158.717	2.994	ECUDOW	161.709	3.438	ECUFUE	169.832	2.849	ECUFUE	152.567	2.813
ECUGAL	169.651	3.297	ECUGAL	167.556	2.812	ECUGAL	155.532	3.312	ECUGAL	157.451	2.975	ECUGAL	152.327	3.389
ECUGAL	176.421	2.904	ECUGOZ	166.224	2.824	ECUGAL	160.802	2.812	ECUGAL	155.363	3.442	ECUGAL	163.791	3.396
ECUGOZ	161.895	3.331	ECUGOZ	156.933	2.781	ECUGOZ	164.628	2.717	ECUGOZ	168.957	2.717	ECUGOZ	169.997	3.236
ECUVII	156.195	3.183	ECUYEI	166.178	2.98	ECUJAQ	165.234	3.323	ECUQID	150.091	3.379	ECUVII	154.965	2.868
EDAGUL	168.637	2.857	EDAMEC	161.99	3.39	EDAGIZ	162.383	3.282	EDAGIZ	163.5	3.275	EDAGUL	161.328	3.157
EDAMEC	157.193	3.064	EDAMEC	155.106	3.142	EDAMEC	160.491	2.718	EDAMEC	153.661	3.003	EDAMEC	171.395	2.967
EDASIM	150.464	3.047	EDASIM	157.097	2.988	EDAMEC	171.385	3.003	EDAMOM	161.522	3.488	EDASIM	171.284	3.007
EDASOS	151.924	3.425	EDASOS	158.602	3.271	EDASOS	168.95	3.272	EDASOS	158.26	3.328	EDASOS	169.905	2.957
EDAWAH	157.396	3.093	EDAWAH	152.838	3.456	EDASOS	160.631	3.333	EDATEM	150.493	3.36	EDATEM	151.403	3.245
EDEJIH	168.194	3.182	EDEJIH	164.408	2.712	EDAYOY	158.844	3	EDECAV	161.795	2.998	EDEJIH	160.753	2.876
EDEJIH	151.987	3.182	EDEJIH	153.291	2.877	EDEJIH	154.793	2.876	EDEJIH	157.712	3.462	EDEJIH	166.246	3.021

EDEJIH	159.721	3.462	EDENUA	151.548	2.945	EDEJIH	159.491	3.407	EDEJIH	169.006	3.033	EDEJIH	152.035	3.021
EDEYIY01	152.917	3.458	EDEYIY01	153.652	2.841	EDEPIM	162.747	3.328	EDEPIM	154.723	3.427	EDEPIM	156.615	3.451
EDEYOE0	170.931	2.901	EDICEZ	152.041	3.428	EDEYIY01	153.623	3.044	EDEYIY01	158.191	2.995	EDEYOE0	150.845	2.785
EDICID	157.643	3.055	EDICID	162.704	3.415	EDICID	159.974	3.41	EDICID	152.392	3.235	EDICID	168.216	2.851
EDINOV	160.45	2.718	EDISOA	154.238	3.218	EDIJAD	174.714	2.998	EDINOV	152.913	3.05	EDINOV	151.791	2.951
EDIXOI	150.401	3.236	EDOBAB	170.1	2.885	EDISOA	153.441	3.124	EDISOA	160.557	3.193	EDIVIW	158.529	2.911
EDOBUV	151.05	3.119	EDOFEJ	167.563	2.862	EDOBAB	170.66	3.27	EDOBAB	171.517	3	EDOBAB	152.314	3.162
EDOMUF	160.591	3.029	EDOMUF	155.793	2.975	EDOGIO	175.614	3.427	EDOMUF	154.28	3.023	EDOMUF	151.887	2.773
EDONAM	156.288	2.963	EDONAM	152.234	2.785	EDOMUF	152.31	2.953	EDOMUF	153.578	2.923	EDOMUF	161.494	2.677
EDONAM	152.482	2.941	EDONAM	152.324	2.906	EDONAM	160.325	3.029	EDONAM	155.337	2.977	EDONAM	150.765	2.98
EDONAM0	150.554	2.954	EDONAM0	155.104	2.969	EDONAM	161.103	2.69	EDONAM0	156.027	2.936	EDONAM0	152.355	2.876
EDONAM0	160.581	2.989	EDOSAT	164.126	2.986	EDONAM0	153.004	2.764	EDONAM0	161.23	2.663	EDONAM0	152.772	2.926
EDOSAT0	173.543	2.705	EDOSAT0	173.604	2.705	EDOSAT	169.599	2.986	EDOSAT0	170.002	3.47	EDOSAT0	176.525	3.421
EDOZON	166.062	3.175	EDOZON	174.293	2.781	EDOXEE	165.945	3.349	EDOZIG	170.382	3.443	EDOZON	155.585	2.942
EDOZUT	165.799	2.82	EDOZUT	171.943	3.045	EDOZON	154.068	2.955	EDOZON	169.534	3.024	EDOZUT	151.727	3.054
EDOZUT	150.212	2.867	EDOZUT	177.408	3.193	EDOZUT	173.624	3.054	EDOZUT	178.615	3.198	EDOZUT	153.665	3.186
EDUBAH	166.615	2.938	EDUBEL	177.711	3.118	EDUBAG	175.902	2.79	EDUBAH	160.288	3.373	EDUBAH	170.421	2.795
EDUBEL	178.485	2.886	EDUBIP	150.633	3.001	EDUBEL	164.893	3.087	EDUBEL	156.404	3.012	EDUBEL	158.742	3.273
EDUBIP	150.944	3.047	EDUCIP	153.057	3.145	EDUBIP	167.073	3.081	EDUBIP	167.462	2.731	EDUBIP	151.268	3.378
EDUYAH	162.504	3.095	EDUYEL	174.207	2.904	EDUCIP	153.418	3.25	EDUCIP	162.785	3.099	EDUCIP	164.721	2.83
EFAHAU	152.101	3.367	EFAHAU	155.489	3.314	EFACIX	158.128	3.217	EFACIX01	158.368	3.222	EFAHAU	150.3	3.212
EFAQOU	154.199	3.201	EFAQOU	152.317	3.08	EFAHAU	151.903	3.044	EFAHAU	161.76	2.877	EFAHAU	151.223	3.162
EFAQOU	151.872	3.459	EFAQOU	154.081	2.836	EFAQOU	162.707	3.249	EFAQOU	158.889	3.288	EFAQOU	155.808	2.781
EFAQOU0	153.576	3.193	EFAQOU0	151.915	3.062	EFAQOU	160.239	2.8	EFAQOU	152.422	3.288	EFAQOU0	158.699	3.235
EFAQOU0	153.483	2.824	EFAQOU0	156.655	2.753	EFAQOU0	169.856	3.493	EFAQOU0	152.966	3.235	EFAQOU0	159.779	2.778
EFAQUA	164.122	3.06	EFAQUA	153.642	3.032	EFAQOU0	151.12	3.461	EFAQOU0	159.056	3.493	EFAQOU0	161.204	3.187
EFAWOY	156.823	3.413	EFAWOY	159.093	3.181	EFATOU	168.617	2.919	EFATUD	150.199	3.074	EFAWOY	153.101	3.188
EFAWOY	166.719	3.471	EFAWOY	150.82	3.257	EFAWOY	155.858	3.036	EFAWOY	165.779	2.793	EFAWOY	165.165	2.954
EFAWOY	158.921	2.907	EFAWOY	157.882	3.276	EFAWOY	157.261	3.105	EFAWOY	153.071	2.724	EFAWOY	150.467	3.293
EFAZEQ	176.743	3.375	EFEGAB	157.977	3.103	EFAWOY	155.011	2.857	EFAYAL	163.65	2.805	EFAZAM	170.718	3.243
EFFACO	161.415	3.119	EFFACO	158.002	3.027	EFEVUH	153.926	3.466	EFEXEW	159.321	2.992	EFFACO	154.286	3.204
EFIKAH	167.09	3.179	EFIKAH	158.562	3.055	EFIKAH	150.437	3.121	EFIKAH	150.85	2.983	EFIKAH	165.123	2.849
EFIKAH	156.576	2.849	EFILUD	160.371	3.15	EFIKAH	153.408	2.786	EFIKAH	156.78	3.299	EFIKAH	159.243	2.759
EFINOX	160.962	3.351	EFIQAP	157.996	2.949	EFINOX	160.045	3.212	EFINOX	160.437	3.365	EFINOX	159.383	3.215
EFIWOG	151.909	3.381	EFIWOG	170.298	3.196	EFITAO	150.824	3.227	EFITAO	158.162	3.018	EFIWOG	153.784	2.771
EFIWOG	150.347	3.355	EFIWOG	161.348	3.238	EFIWOG	177.555	2.908	EFIWOG	157.853	3.109	EFIWOG	151.175	2.741
EFIWOG	151.279	2.77	EFIWOG	152.441	2.916	EFIWOG	165.982	3.099	EFIWOG	172.248	2.863	EFIWOG	170.068	2.703
EFOQAU	156.325	2.687	EFOQAU	156.506	2.687	EFONET	151.659	3.191	EFOQAU	161.185	3.415	EFOQAU	158.91	3.491
EFOZOP	152.759	3.356	EFOZOP	162.473	3.168	EFOSIE	155.578	3.103	EFOSIE	159.334	3.011	EFOZOP	154.782	3.095
EFOZOP	152.036	3.356	EFOZOP	158.137	3.415	EFOZOP	156.667	3.416	EFOZOP	156.859	3.388	EFOZOP	154.566	3.43
EFOZOP	156.757	3.079	EFOZOP	151.332	3.207	EFOZOP	158.171	3.018	EFOZOP	150.444	2.831	EFOZOP	157.535	3.163
EFUFAM	165.157	3.246	EFUFAO	161.812	2.71	EFOZOP	165.277	3.225	EFOZOP	166.902	2.94	EFOZOP	161.598	3.19
EFUFIW	159.807	3.064	EFUFOC	166.194	2.897	EFUFAO	154.753	2.722	EFUFAO	156.744	2.722	EFUFES	155.489	3.178
EFUGET	170.942	3.489	EFUGET	161.988	2.924	EFUFUI	161.265	3.109	EFUFUI	159.219	2.95	EFUGET	159.663	3.325
EFUJEU	161.494	2.927	EFUTUV	150.942	2.979	EFUJEU	165.527	3.219	EFUJEU	150.808	3.346	EFUJEU	162.179	3.398
EFUXIO	150.413	3.403	EFUZUD	168.472	3.1	EFUTUV	157.332	2.969	EFUXIO	162.634	2.861	EFUXIO	173.06	2.861
EGABOD	154.748	3.457	EGABOD	154.867	2.948	EGABOD	163.409	2.949	EGABOD	153.619	2.821	EGABOD	164.101	3.463
EGADOH	162.555	2.825	EGADOH	151.296	2.951	EGABOD	168.361	2.983	EGABOD	153.692	2.967	EGADOH	151.842	3.478
EGANIK	156.742	3.159	EGANOQ	153.06	2.828	EGAFOL	159.012	3.244	EGAJON	156.835	3.245	EGAJON	151.505	3.341
EGAQOS	171.249	3.343	EGAQOS	173.687	3.173	EGAPIL	157.925	3.393	EGAQOS	171.965	3.421	EGAQOS	153.812	3.136
EGAVUD	153.701	2.945	EGAWAK	166.953	3.069	EGAQOS	153.501	3.424	EGAQOS	163.295	3.178	EGAQOS	158.928	3.281
EGEBUR	160.849	3.452	EGEBUR	168.105	3.071	EGAWAK	151.177	2.805	EGAWAK	174.733	2.842	EGAWES	151.912	3.191
EGEXOE	152.848	3.307	EGEYAR	152.992	2.779	EGETOB	159.228	3.127	EGEXOE	152.865	2.895	EGEXOE	165.438	3.429
EGEZUO	154.404	3.064	EGOCOV	152.234	3.406	EGEYAR	171.585	3.163	EGEYAR	151.343	2.919	EGEYAR	152.91	2.791
EGOSIG	161.118	2.625	EGOYII	158.963	3.068	EGOJIU	173.429	3.185	EGOSIG	161.551	2.625	EGOSIG	156.016	3.015
EGUDUF	159.267	2.841	EGUDUF	172.388	3.068	EGOYIM	156.372	3.419	EGUBEO	153.722	3.44	EGUCES	155.085	3.11
EGUDUF	159.164	3.344	EGUDUF	155.373	2.875	EGUDUF	151.78	2.839	EGUDUF	158.947	2.974	EGUDUF	157.174	3.429
EGUDUF	162.927	2.996	EGUDUF	153.102	3.472	EGUDUF	173.835	3.403	EGUDUF	152.181	3.141	EGUDUF	162.309	3.267
EGUDUF	152.235	3.255	EGUFEU	150.214	2.901	EGUDUF	169.544	3.373	EGUDUF	165.578	2.87	EGUDUF	154.004	2.83
EGUFIY01	156.286	3.29	EGUFIY02	162.1	3.19	EGUFIY	162.121	3.183	EGUFIY	156.115	3.284	EGUFIY01	162.104	3.19
EGUHEX	157.802	3.362	EGUHEX	168.158	2.729	EGUFIY02	156.291	3.29	EGUFIY03	162.107	3.19	EGUFIY03	156.281	3.29
EGUXOU	158.717	3.272	EHAKIH	164.32	3.216	EGUMOI	172.114	2.859	EGUWEK	169.393	2.874	EGUWOU	156.263	2.926
EHARUC	160.206	2.888	EHARUC	158.178	3.155	EHAKON	158.81	3.123	EHAKON	164.831	3.399	EHAKON	161.197	3.399
EHAWAL	160.089	3.128	EHAWAL	168.257	3.049	EHARUC	169.481	3.278	EHARUC	170.081	3.263	EHARUC	151.941	3.018
EHAWIT	150.944	3.484	EHAWIT	157.681	3.295	EHAWIT	161.865	3.41	EHAWIT	156.02	2.973	EHAWIT	170.661	3.355
EHAWIT	151.051	3.128	EHAWIT	154.847	3.21	EHAWIT	157.558	3.285	EHAWIT	160.781	2.73	EHAWIT	165.013	3.102
EHAWIT	169.826	2.973	EHAWIT	157.558	2.886	EHAWIT	152.424	3.043	EHAWIT	153.261	3.48	EHAWIT	153.341	2.953
EHECAX	153.83	3.27	EHEDUR	173.135	2.804	EHAWIT	171.064	3.23	EHEBEA	166.124	3.487	EHECAX	161.038	3.212
EHEGIJ	151.406	3.412	EHEGIJ	150.263	3.372	EHEFED	157.3	2.605	EHEFED	156.417	3.151	EHEFED	159.747	2.929
EHEKIN	172.523	3.354	EHEKIN	175.346	3.438	EHEKIN	150.495	3.327	EHEKIN	170.099	2.88	EHEKIN	172.354	3.254
EHEROZ	155.083	3.4	EHESUG	152.457	2.755	EHEPEM	160.397	3.343	EHEPEM	160.564	2.956	EHEROZ	154.644	3.394
EHESUG	152.37	2.907	EHESUG	160.92	2.765	EHESUG	155.757	3.063	EHESUG	156.412	2.785	EHESUG	163.876	2.955
EHIJIQ	170.4	3.412	EHIJIQ	153.638	3.163	EHESUG	170.786	3.036	EHESUG	152.841	3.007	EHEXUK	151.34	3.346
EHIKOV	159.175	3.31	EHILEM	172.371	3.476	EHIJIQ	157.564	2.785	EHIJUB	172.221	3.157	EHIKOV	165.839	2.955
EHILIU	156.214	3.419	EHILIU	165.907	3.12	EHILEM	170.059	3.381	EHILIU	153.83	2.883	EHILIU	154.059	3.479
EHIVID	151.165	3.015	EHIVID	158.403	3.122	EHIMER	165.069	3.036	EHIVEZ	178.173	3.043	EHIVEZ	162.412	2.719
EHOVUV	172.345	3.28	EHOYEH	173.797	3.387	EHIVOJ	157.331	2.875	EHIWIB	152.397	2.896	EHOTEC	156.806	3.069
EHOYEH	169.994	3.209	EHOZOS	151.01	2.947	EHOYEH	159.553	3.132	EHOYEH	175.673	3.486	EHOYEH	157.641	3.119
EHUQOP	170.532	2.721	EHUQOP	168.057	2.769	EHOZOS	150.172	2.947	EHUFAR	153.265	3.341	EHUKOJ	153.193	2.837

EHUWEK	157.454	3.052	EHUXAH	158.494	2.995	EHUREG	168.851	2.994	EHUROQ	151.628	2.974	EHUWEK	172.155	3.052
EHUXAH	157.957	2.887	EHUXAH	155.534	2.9	EHUXAH	163.375	3.164	EHUXAH	170.025	3.056	EHUXAH	152.507	3.34
EHUXAH0	152.507	3.34	EHUXAH0	157.957	2.887	EHUXAH0	158.494	2.995	EHUXAH0	163.375	3.164	EHUXAH0	170.025	3.056
EJADOM	158.104	3.28	EJAHIK	175.484	3.493	EHUXAH0	155.534	2.9	EJADIF	153.686	2.935	EJADIF	158.768	2.846
EJAHIK	156.137	2.798	EJAHIK	160.566	3.173	EJAHIK	163.133	2.887	EJAHIK	154.415	3.049	EJAHIK	169.342	3.031
EJAVES	162.676	3.375	EJAXIA	166.048	2.978	EJAJUY	165.386	3.246	EJAKIN	169.498	2.77	EJAKIN	159.249	3.441
EJEGEI	162.047	2.778	EJEGEI	166.197	2.778	EJAZOG	175.177	3.411	EJEFAE	150.719	3.148	EJEFAE	155.605	3.148
EJEHEK	156.739	3.142	EJERUI	155.958	2.923	EJEGIM	161.378	2.914	EJEGIM	167.453	2.914	EJEHEK	152.722	3.219
EJEXOK	172.824	3.252	EJEXOK	174.744	3.353	EJERUI	158.127	2.923	EJEVEX	156.08	3.228	EJEVEX	157.919	3.228
EJEYUR	150.103	2.855	EJEYUR	152.576	3.314	EJEYUP	166.048	3.126	EJEYUP	170.032	2.637	EJEYUP	169.574	2.799
EJEYUR	154.876	3.257	EJEYUR	171.113	3.216	EJEYUR	155.282	2.855	EJEYUR	171.656	2.975	EJEYUR	177.618	2.975
EJIDUA	158.373	3.268	EJIDUA	164.014	3.483	EJEYUR	152.344	3.367	EJEZEC	158.797	3.35	EJEZEC	157.796	2.955
EJIHEO	156.734	2.971	EJIHEO	164.489	2.779	EJIDUA	175.664	3.204	EJIDUA	153.472	3.204	EJIDUY	173.181	3.199
EJIJEQ	153.395	2.981	EJIJEQ	152.503	2.981	EJIJEQ	165.477	3.282	EJIJEQ	176.378	3.374	EJIJEQ	163.869	3.125
EJIJIU	159.468	2.856	EJIJIU	171.465	3.376	EJIJEQ	168.275	2.806	EJIJIU	154.021	3.455	EJIJIU	154.039	2.787
EJIMES	164.191	3.245	EJIMIW	164.403	3.246	EJIMES	162.225	3.054	EJIMES	161.014	3.087	EJIMES	159.603	3.37
EJIPOD	153.471	2.711	EJIPOD	156.192	3.489	EJIMIW	159.517	3.367	EJIMIW	160.916	3.086	EJIMIW	162.122	3.054
EJIPOD01	159.06	3.249	EJIPOD01	168.426	3.341	EJIPOD	150.192	2.881	EJIPOD	167.074	2.711	EJIPOD	156.702	3.181
EJIQAQ	175.922	2.953	EJIQAQ	160.677	3.176	EJIPOD01	156.484	3.165	EJIPOD01	157.142	3.302	EJIQAQ	173.372	3.084
EJOGIU	168.409	3.168	EJOGIU	166.103	3.168	EJIVAV	164.798	3.173	EJIVAV	150.285	3.445	EJOBOY	172.203	3.247
EJOWEG	159.489	2.92	EJOWEG	161.191	2.92	EJOREA	163.449	2.828	EJOWEG	160.059	3.039	EJOWEG	151.321	3.039
EJUCIY	159.745	2.676	EJUCIY	163.929	2.799	EJOWIK	152.827	3.485	EJUCIY	168.174	2.62	EJUCIY	167.734	2.8
EJUCUK	154.343	2.978	EJUDAR	163.935	2.802	EJUCUK	166.983	3.408	EJUCUK	157.875	2.836	EJUCUK	151.459	3.226
EJUGOJ	160.331	3.377	EJUJUS	154.468	3.291	EJUDAR	161.43	2.663	EJUDAR	169.89	2.785	EJUDAR	166.511	2.624
EJUKOJ	174.598	3.191	EJUNOP	161.082	2.992	EJUJUS	153.442	3.024	EJUJUS	150.536	3.292	EJUJUS	169.406	2.739
EJUYEQ	158.372	2.754	EJUYEQ	165.826	3.096	EJUTEK	164.281	3.108	EJUVIS	152.803	3.214	EJUYAM	150.946	3.486
EKABEY01	156.401	3.258	EKABEY02	153.418	3.067	EJUZUF	176.978	2.786	EKABAU	166.329	3.094	EKABEY	156.332	3.272
EKABEY02	150.538	3.323	EKABEY02	163.819	3.005	EKABEY02	160.668	3.155	EKABEY02	174.388	3.009	EKABEY02	158.669	3.359
EKABEY04	158.651	3.028	EKABEY06	154.421	3.129	EKABEY03	173.344	3.241	EKABEY03	151.78	3.47	EKABEY03	159.677	3.059
EKABEY06	159.324	3.418	EKABEY06	151.049	3.413	EKABEY06	161.531	3.323	EKABEY06	173.297	3.106	EKABEY06	162.463	3.086
EKABIC	168.135	3.18	EKABOJ	159.393	3.444	EKABIC	152.621	2.985	EKABIC	152.172	3.059	EKABIC	170.52	3.29
EKADUS	161.074	2.875	EKAGEF	151.131	2.97	EKADUS	154.48	2.917	EKADUS	153.349	3.131	EKADUS	156.216	3.066
EKAHAC0	152.164	3.122	EKAHAC0	152.696	2.862	EKAHAC	165.536	3.334	EKAHAC0	159.962	3.122	EKAHAC0	158.082	2.73
EKAPAI	154.582	3.04	EKAPAI	160.651	2.846	EKAPAI	165.035	3.213	EKAPAI	174.974	3.074	EKAPAI	174.084	3.118
EKAPAI	162.457	2.868	EKAPAI	156.418	2.931	EKAPAI	172.691	2.999	EKAPAI	172.033	3.108	EKAPAI	160.666	3.006
EKAPAI	156.97	3.074	EKAPAI	163.861	3.196	EKAPAI	156.187	3.059	EKAPAI	172.922	3.104	EKAPAI	159.749	2.916
EKAPEM	155.026	3	EKAPEM	161.62	3.387	EKAPAI	154.579	3.211	EKAPAI	165.169	3.009	EKAPEM	174.736	3.197
EKAPEM	160.829	2.793	EKAPEM	152.991	3.058	EKAPEM	156.717	2.984	EKAPEM	173.172	3.191	EKAPEM	166.834	3.324
EKAPUE	152.182	3.216	EKAPUE	159.638	3.127	EKAPEM	154.37	3.366	EKAPEM	165.744	2.663	EKAPEM	155.063	3.219
EKAQIT	162.642	3.337	EKAQIT	157.436	3.498	EKAPUE	167.181	3.292	EKAPUE	170.585	3.133	EKAQIT	166.302	3.029
EKASEQ	154.274	2.83	EKASEQ	174.655	3.064	EKAQIT	166.913	3.14	EKAQIT	160.849	3.113	EKASEQ	157.76	3.347
EKATAP	171.533	3.216	EKAVAR	166.394	3.339	EKASEQ	152.308	3.495	EKASOC	157.967	2.971	EKASOC	155.805	3.47
EKEGIM	159.737	2.856	EKEGIM	166.33	3.099	EKAVAR	175.982	2.961	EKAVAR	152.506	2.999	EKAVAR	159.505	3.444
EKEGOS	155.669	3.437	EKEGUY	163.427	2.856	EKEGOS	158.424	3.319	EKEGOS	166.377	3.276	EKEGOS	162.612	3.276
EKEHAH	151.319	3.176	EKEHAH	151.997	3.199	EKEHAH	151.16	3.298	EKEHAH	150.885	3.054	EKEHAH	161.722	3.202
EKENOZ	153.004	3.17	EKENOZ	158.536	3.111	EKEHAH	155.917	3.338	EKEHAH	158.978	3.01	EKENOZ	152.308	2.996
EKENOZ	157.073	2.803	EKENOZ	152.679	3.341	EKENOZ	165.08	3.252	EKENOZ	152.908	3.17	EKENOZ	162.124	2.803
EKEPAN	161.669	3.272	EKEPOC	164.621	3.213	EKENOZ	152.701	3.393	EKENOZ	150.161	2.893	EKEPAN	170.007	3.137
EKEPOC	155.407	3.072	EKEPOC	151.44	3.245	EKEPOC	162.459	3.48	EKEPOC	169.471	3.213	EKEPOC	162.841	3.17
EKESIA	165.746	2.745	EKEWUP	150.811	3.35	EKEPOC	155.235	2.861	EKEQOE	156.805	3.042	EKEQOE	155.627	2.907
EKEZUR	153.305	3.047	EKEZUR	162.633	2.779	EKEWUP	165.648	3.165	EKEXID	152.919	2.825	EKEZUR	162.837	3.085
EKEZUR	154.055	3.116	EKIBIK	164.079	2.869	EKEZUR	158.609	3.22	EKEZUR	156.955	3.025	EKEZUR	155.94	2.924
EKIBIK	157.972	3.116	EKICAE	161.696	3.099	EKIBIK	172.542	3.338	EKIBIK	157.824	2.959	EKIBIK	174.537	3.282
EKICAE	155.62	2.947	EKICEI	170.994	2.811	EKICAE	166.232	3.099	EKICAE	155.419	3.237	EKICAE	151.655	2.836
EKICOS	165.742	2.879	EKICOS	151.559	3.16	EKICEI	164.406	3.281	EKICEI	158.714	2.97	EKICEI	169.507	2.906
EKICOS	155.266	3.297	EKICOS	169.519	3.355	EKICOS	158.992	3.401	EKICOS	169.222	2.903	EKICOS	163.02	3.434
EKICUY	173.15	2.982	EKICUY	165.259	2.985	EKICOS	158.633	3.346	EKICOS	152.883	3.373	EKICUY	154.962	3.289
EKICUY	170.579	3.041	EKICUY	162.791	3.103	EKICUY	161.187	2.692	EKICUY	177.679	2.937	EKICUY	174.767	3.067
EKICUY	158.944	2.907	EKICUY	150.52	2.787	EKICUY	159.887	2.692	EKICUY	171.048	2.755	EKICUY	168.741	2.766
EKIMUK	152.038	3.18	EKINER	158.986	3.054	EKICUY	169.982	2.942	EKIMER	167.917	3.114	EKIMER	150.413	3.114
EKISID	150.289	2.554	EKISID	170.623	2.554	EKIQEW	160.03	3.272	EKIQEW	169.747	3.097	EKIRIC	168.617	2.795
EKITEA	157.5	2.756	EKITIE	150.775	2.622	EKISID	152.889	2.855	EKISOJ	150.296	3.363	EKISUP	168.533	2.649
EKITUQ	179.037	3.439	EKITUQ	156.887	2.957	EKITIE	160.342	2.855	EKITIE	152.951	2.779	EKITIE	175.707	3.424
EKIZAA	156.614	2.953	EKIZAA	152.369	2.886	EKIYEC	157.648	3.086	EKIZAA	158.583	3.188	EKIZAA	150.117	3.484
EKIZAA	151.159	2.999	EKOCIT	150.313	3.214	EKIZAA	152.965	3.171	EKIZAA	161.827	3.445	EKIZAA	165.355	2.677
EKOCIT	166.927	3.11	EKOCIT	155.665	2.693	EKOCIT	161.518	3.41	EKOCIT	164.068	2.867	EKOCIT	164.791	2.917
EKOKAS	154.298	3.4	EKOLEW	164.832	3.174	EKOCIT	161.341	2.803	EKOCIT	165.532	3.474	EKOCIT	156.137	2.814
EKORII	154.851	3.005	EKOWOQ	160.431	3.084	EKORII	155.913	3.009	EKORII	150.466	3.11	EKORII	153.598	2.984
EKUBOB	157.73	2.784	EKUBOB	160.382	2.966	EKOWOQ	151.191	2.852	EKOWOQ	167.612	2.966	EKUBOB	163.814	3.142
EKUBOB	155.447	2.995	EKUBOB	156.905	3.209	EKUBOB	154.005	2.835	EKUBOB	155.042	2.89	EKUBOB	154.363	3.476
EKUBUH	154.153	3.186	EKUBUH	151.61	3.309	EKUBUH	153.36	3.072	EKUBUH	153.166	3.371	EKUBUH	155.637	3.105
EKUBUH	153.118	3.395	EKUBUH	153.807	3.448	EKUBUH	170.764	3.181	EKUBUH	150.161	2.827	EKUBUH	171.065	3.193
EKUQUZ	161.429	3.064	EKUQUZ	163.907	2.931	EKUCIY	169.039	3.406	EKULAY	155.166	2.859	EKULAY	154.345	3.155
EKUTEM	162.096	3.335	EKUTEM	174.792	3	EKUSOV	159.359	3.285	EKUTAI	156.815	3.151	EKUTAI	160.322	3.478
EKUTIR	151.504	2.904	EKUTIR	166.696	2.8	EKUTEM	172.38	3.337	EKUTIR	152.022	3.088	EKUTIR	151.787	3.366
ELACAV	156.295	2.945	ELACAV	150.173	2.739	EKUTIR	164.995	2.561	EKUVIS	153.121	2.746	EKUWEQ	154.625	2.79
ELAPOA	157.804	3.152	ELAPOA	158.687	3.374	ELACAV	166.152	3.481	ELAJEK	154.25	2.836	ELAJEK	167.042	3.217
ELAPUG	164.609	3.427	ELAPUG	160.06	2.864	ELAPOA	163.033	3.367	ELAPOA	169.136	3.338	ELAPOA	163.054	3.338
ELAQOX	156.334	3.233	ELAQOX	150.761	2.852	ELAQOX	168.476	3.148	ELAQOX	168.389	3.121	ELAQOX	152.405	2.977

ELAQOX	155.128	3.197	ELAQOX	151.3	3.377	ELAQOX	156.189	3.154	ELAQOX	168.291	3.33	ELAQOX	153.928	3.065
ELAREO	168.805	3.162	ELAREO	169.737	3.21	ELAQOX	152.243	3.368	ELAQOX	158.418	3.148	ELAQOX	163.701	3.163
ELAREO	161.575	3.162	ELAREO	157.917	3.274	ELAREO	155.317	2.834	ELAREO	167.954	3.162	ELAREO	158.932	3.274
ELEDIM	152.247	2.769	ELEFIK	157.533	3.265	ELEBED	150.369	2.847	ELEBIH	151.53	2.717	ELEBIH	153.233	3.461
ELEFIK	167.698	3.011	ELEFIK	160.451	2.769	ELEFIK	150.169	3.115	ELEFIK	163.502	3.011	ELEFIK	168.009	2.903
ELEFIK	161.456	3.417	ELEFIK	166.844	2.801	ELEFIK	159.328	3.439	ELEFIK	167.432	2.913	ELEFIK	153.809	2.733
ELEFOQ	175.284	2.744	ELEFOQ	153.812	3.009	ELEFOQ	164.634	2.931	ELEFOQ	153.164	3.496	ELEFOQ	154.784	2.978
ELEJUA	167.7	3.347	ELEKEP	158.612	2.812	ELEFOQ	150.778	2.744	ELEGAH	166.894	3.074	ELEJUA	152.049	3.295
ELELOA	172.462	2.82	ELEQAR	155.763	3.071	ELEKEP	155.897	3.225	ELEKEP	152.669	3.225	ELELOA	172.615	2.82
ELIDIP	157.825	3.003	ELIDIP	156.7	2.775	ELEQAR	166.699	3.134	ELEQAR	160.495	3.112	ELIDIP	153.786	3.16
ELIHUG	165.567	2.915	ELIHUG	162.715	2.927	ELIFOX	156.3	2.891	ELIHUG	157.011	3.461	ELIHUG	159.526	3.468
ELIMAQ	152.972	3.446	ELIVOL	175.332	3.353	ELIHUG	162.698	3.249	ELIHUG	158.954	3.309	ELIMAQ	167.508	3.446
ELIYIK	173.953	3.378	ELIYIK	155.766	3.444	ELIVOL	153.491	2.878	ELIYIK	153.128	3.075	ELIYIK	152.192	3.378
ELOHAQ	161.221	2.806	ELOHAQ	165.713	3.098	ELOHAP	166.031	3.326	ELOHAP0	165.708	3.446	ELOHAQ	159.947	3.282
ELOQEF	169.209	2.755	ELOQEF	162.528	3.158	ELOHAQ	163.218	2.806	ELOLEY	163.544	3.321	ELOLIC	161.459	3.304
ELORAC	150.249	2.786	ELORAC	167.498	3.138	ELOQEF	161.21	2.751	ELORAC	150.792	3.108	ELORAC	150.794	3.108
ELORAC	163.735	3.27	ELORAC	150.368	2.777	ELORAC	166.581	3.359	ELORAC	174.449	2.989	ELORAC	160.669	2.901
ELOTII	167.891	2.862	ELOTII	170.872	2.984	ELORAC	154.28	3.133	ELORAC	168.752	2.946	ELOTII	163.343	2.862
ELUHEC	156.134	2.705	ELUKAB	158.566	2.83	ELOTOO	154.581	3.292	ELOWUB	155.015	3.472	ELUHEC	154.014	2.696
ELULED	166.22	3.274	ELULON	150.904	3.003	ELUKAB	179.233	3.35	ELUKAB	156.591	3.031	ELUKAB	156.785	2.858
ELULUT	158.635	3.013	ELULUT	161.716	3.44	ELULUT	173.249	2.83	ELULUT	160.259	2.889	ELULUT	155.523	3.244
EMABEA	150.217	2.985	EMABED	160.46	2.967	ELULUT	158.438	3.187	ELUYET	157.299	3.147	EMABAW	161.935	3.456
EMABED	171.513	2.702	EMABED	164.37	2.622	EMABED	161.478	3.192	EMABED	165.27	3.286	EMABED	150.303	2.622
EMAFEG	169.992	3.318	EMAFEG	154.589	3.38	EMABED	156.811	3.378	EMAFEG	165.185	3.144	EMAFEG	160.187	2.811
EMAFEG	165.611	3.227	EMAFEG	156.573	3.462	EMAFEG	172.66	3.316	EMAFEG	160.413	2.867	EMAFEG	157.464	3.023
EMAFEG	158.932	3.318	EMAFEG	160.457	2.961	EMAFEG	157.592	2.801	EMAFEG	163.488	3.403	EMAFEG	158.345	3.19
EMAMEN	171.922	3.178	EMAMEN	155.178	2.854	EMAFEG	154.316	3.222	EMAFEG	161.021	3.051	EMAMEN	151.389	3.339
EMEGAE	156.745	2.667	EMEKAJ	162.773	3.399	EMAMIR	150.358	3.364	EMAMIR	157.971	2.875	EMAMIR	160.62	2.768
EMEKEN	150.645	3.04	EMEKIQ	171.936	3.324	EMEKEN	166.398	3.352	EMEKEN	154.21	3.04	EMEKEN	161.973	3.244
EMEKIQ	157.046	3.102	EMEKIR	155.007	3.406	EMEKIQ	168.841	3.483	EMEKIQ	158.059	3.424	EMEKIQ	179.5	3.277
EMEKUD	163.01	2.883	EMEMUG	165.774	2.934	EMEKUD	152.147	2.872	EMEKUD	165.375	3.025	EMEKUD	160.024	3.322
EMIBOS01	170.095	3.212	EMICEJ	153.746	2.931	EMEMUG	156.1	2.934	EMEZOL	154.217	3.471	EMIBOS	169.431	3.208
EMICIP	162.671	3.153	EMICIP	153.916	2.85	EMICEJ	156.824	3.105	EMICEJ	176.467	3.093	EMICEJ	160.239	3.105
EMICOU	172.632	3.479	EMIDAF	160.611	3.169	EMICIP	171.116	3.104	EMICOU	158.446	3.11	EMICOU	167.504	3.102
EMILIX	159.211	3.319	EMILIX	175.383	3.336	EMIDAF	165.227	2.866	EMIDAF	160.579	3.131	EMILIX	154.007	3.004
EMILIX	164.74	3.052	EMILIX	151.646	3.052	EMILIX	151.679	2.885	EMILIX	171.841	3.004	EMILIX	174.918	3.073
EMILIX01	151.838	2.976	EMILIX01	157.669	2.975	EMILIX01	163.399	3.252	EMILIX01	155.647	3.227	EMILIX01	150.94	2.967
EMOCIT	170.766	2.817	EMOCIT	169.633	2.972	EMILIX01	155.589	3.41	EMOCIT	175.253	3.08	EMOCIT	165.484	3.102
EMOCIT	174.05	3.08	EMOCIT	170.238	3.102	EMOCIT	166.532	2.833	EMOCIT	171.573	2.99	EMOCIT	161.547	3.078
EMOCIT	166.337	2.838	EMOMUQ	156.247	2.892	EMOCIT	160.418	2.833	EMOCIT	172.747	2.99	EMOCIT	155.879	2.838
EMUMAB	151.689	2.932	EMUNAD	166.792	3.472	EMOPUU	150.14	2.952	EMOPUU	153.396	3.356	EMUCOH	171.631	3.134
EMUVUG	163.137	2.922	EMUYUH	163.233	3.312	EMUSAG	173.505	3.167	EMUTEM	174.985	3.189	EMUVUG	159.955	2.922
ENACUU	161.714	3.28	ENAJEK	162.31	3.145	EMUYUH	171.351	2.923	EMUYUH	156.43	3.312	EMUZET	156.96	3.195
ENECUX	172.074	3.377	ENEDAF	171.106	2.838	ENAKEJ	164.698	3.299	ENAMAJ	155.395	2.822	ENAVAR	151.674	2.964
ENEDAF	153.08	3.29	ENEDAF	157.904	2.719	ENEDAF	151.672	2.748	ENEDAF	151.796	3.425	ENEDAF	173.05	2.768
ENEPOC0	161.565	2.987	ENEPOC0	154.853	3.298	ENEDAF	157.143	3.204	ENEPOC0	150.715	3.087	ENEPOC0	151.804	3.493
ENEROF	171.671	3.293	ENEROF	153.48	2.961	ENEPOC0	158.608	3.154	ENEPOC0	160.794	3.309	ENEPOC0	153.729	3.422
ENIDEM	159.071	2.986	ENIDEM	160.16	3.33	ENIDAI	154.899	3.418	ENIDAI	168.07	2.831	ENIDAI	168.66	3.209
ENIHIS	153.519	3.263	ENIHIU	170.435	3.244	ENIDEM	174.921	3.115	ENIDEM	157.541	3.181	ENIDEM	158.158	2.992
ENISAY	162.824	3.106	ENOCOC	154.528	3.004	ENIPUN	172.08	2.96	ENISAY	165.021	3.042	ENISAY	156.16	2.804
ENUJEF	156.342	2.944	ENUKII	167.863	3.225	ENOTEJ	160.626	2.651	ENUBOG	176.701	2.929	ENUJEF	161.581	3.175
ENUMOR	173.096	3.099	ENUQAI	159.97	3.134	ENUMOR	165.783	3.469	ENUMOR	162.259	2.683	ENUMOR	152.758	3.296
EPABUU	157.233	3.454	EPABUU	159.273	3.084	ENUQAI	164.466	3.227	EPABAA	157.938	3.073	EPABAA	156.476	3.169
EPAQUK	177.337	2.992	EPAVEX	160.848	2.76	EPAFUY	166.185	3.43	EPAFUY	153.023	3.06	EPANIU12	152.688	2.732
EPAVIB	165.325	3.437	EPAXUP	157.092	3.38	EPAVEX	152.176	3.21	EPAVEX	151.017	3.306	EPAVIB	157.234	2.913
EPEFOW	153.581	2.98	EPEFOW	170.705	3.455	EPAXUP	152.201	2.971	EPAYUQ	169.77	3.346	EPAYUQ	160.625	2.871
EPEFOX	150.538	2.727	EPEGAJ	171.624	3.449	EPEFOW	158.39	3.036	EPEFOW	155.593	3.186	EPEFOX	158.789	2.922
EPEHOZ	152.409	3.424	EPEHOZ	163.465	3.251	EPEGAJ	154.304	2.994	EPEGAJ	158.252	3.041	EPEGAJ	155.82	3.196
EPEVEC	161.378	3.395	EPEVEC	156.288	3.381	EPEHOZ	153.941	3.108	EPEMIX	155.289	3.164	EPERUP	157.035	2.888
EPEVEC	163.777	2.908	EPEVOM	154.561	2.865	EPEVEC	156.76	2.7	EPEVEC	173.638	2.785	EPEVEC	150.768	2.84
EPITEE	155.322	2.948	EPITII	158.32	3.493	EPEVOM	155.952	2.949	EPIBEN	151.754	3.454	EPIBEN	159.262	3.146
EPIVOP	157.026	2.848	EPIVOP	156.512	2.861	EPIVAC	154.102	3.461	EPIVAC	152.887	2.902	EPIVOP	156.997	2.848
EPIVOQ	154.972	3.123	EPIVOQ	156.304	2.996	EPIVOP	158.587	2.861	EPIVOQ	159.812	2.918	EPIVOQ	169.983	2.772
EPIVUV	175.845	2.769	EPIWAC	157.847	3.146	EPIVOQ	155.865	3.364	EPIVUV	166.929	2.948	EPIVUV	173.357	2.769
EPIWIL	155.731	3.115	EPOHEY	170.156	3.422	EPIWAC	157.871	2.89	EPIWEG	154.22	3.101	EPIWIL	150.411	3.381
EPOMEE	151.587	3.257	EPOMEE	156.401	3.257	EPOMEE	160.402	3.366	EPOMEE	158.701	2.879	EPOMEE	159.018	2.991
EPOMII	160.707	3.35	EPOMII	156.997	2.867	EPOMEE	159.208	3.366	EPOMEE	158.063	2.872	EPOMEE	160.585	3.002
EPOMII	159.077	2.981	EPOMII	156.638	3.287	EPOMII	160.924	2.974	EPOMII	160.139	3.35	EPOMII	158.886	2.869
EPOMOO	156.092	2.885	EPOMOO	157.756	2.825	EPOMII	150.022	3.287	EPOMOO	158.872	3.385	EPOMOO	161.058	2.937
EPOMOO	156.323	3.298	EPONAZ	172.197	2.975	EPOMOO	159.927	2.975	EPOMOO	160.145	3.385	EPOMOO	150.969	3.298
EPOTIN	173	2.872	EPOTIN	172.977	3.421	EPOTIN	171.443	3.427	EPOTIN	171.814	3.398	EPOTIN	168.343	3.295
EPOTOV	155.192	3.317	EPOTUZ	161.937	3.484	EPOTOV	158.332	3.4	EPOTOV	156.382	2.859	EPOTOV	161.055	2.926
EPOTUZ	161.295	2.811	EPOTUZ	160.633	2.741	EPOTUZ	150.512	3.412	EPOTUZ	176.252	3.29	EPOTUZ	160.397	3.065
EPUFIF	173.478	3.158	EPUHII	169.101	2.86	EPOTUZ	157.349	2.831	EPOTUZ	160.228	3.387	EPUFIF	167.219	3.151
EPUHOO	153.18	2.926	EPUHOO	166.546	3.156	EPUHOO	179.4	3.301	EPUHOO	155.4	3.163	EPUHOO	150.343	3.156
EPUKUY	174.27	2.951	EQADAD	163.372	2.713	EPUHOO	154.769	2.924	EPUHUU	171.776	2.846	EPUKOS	159.251	3.478
EQEFAK	160.222	3.077	EQEFAK	161.466	3.408	EQADEG	163.779	2.909	EQADUW	155.27	3.28	EQAKOX	167.415	3.313
EQEMIZ	167.264	2.876	EQEMIZ	159.625	3.358	EQEMIZ	168.182	2.872	EQEMIZ	157.741	3.423	EQEMIZ	166.404	2.89
EQEMIZ	167.25	2.95	EQEMIZ	158.42	3.175	EQEMIZ	164.919	2.897	EQEMIZ	156.131	3.263	EQEMIZ	171.227	2.971

EQEYOQ	173.068	3.136	EQEYOQ	165.815	3.06	EQENOG	150.954	3.433	EQENUK	169.289	3.087	EQEWII	171.163	3.154
EQIVIM	168.503	2.858	EQIZOV	174.136	3.048	EQEYOQ	164.137	2.822	EQEYOQ	159.529	2.822	EQIQOM	165.707	3.279
EQOHAT	152.654	2.835	EQOHAT	158.816	3.24	EQIZOV	153.988	3.1	EQIZOV	150.186	2.888	EQODIX	156.247	3.142
EQOKUQ	176.318	3.433	EQOKUQ	158.532	3.469	EQOHAT	154.434	3.022	EQOHAT	154.548	2.89	EQOHAT	156.797	2.89
EQOLIF	164.042	3.487	EQONIK	152.995	2.692	EQOKUQ	155.96	3.486	EQOLEB	177.223	2.599	EQOLEB	171.04	2.599
EQOTOU0	172.063	2.959	EQUBAT	174.179	2.905	EQONIK	158.836	3.022	EQONIK	153.754	3.102	EQOTOU	171.474	3.029
EQUGEF	150.617	3.072	EQUHII	167.97	3.418	EQUGEF	161.908	3.278	EQUGEF	167.277	3.118	EQUGEF	150.2	2.697
EQUXOF	162.616	2.938	EQUXOF	166.066	2.926	EQUHII	172.437	3.484	EQUXOF	154.428	2.926	EQUXOF	162.184	2.938
ERACOS	151.983	3.099	ERACOS	162.096	3.099	EQUZAR	166.214	2.805	EQUZAR	150.732	3.246	EQUZAR	172.364	2.991
ERAROH	151.652	3.239	ERAROH	164.528	3.335	ERADIN	159.152	2.906	ERADIN	175.909	3.311	ERAJIS	173.325	2.938
ERATAU	166.514	3.174	ERATAU	155.911	2.82	ERATAU	157.561	2.748	ERATAU	159.815	3.31	ERATAU	163.562	2.933
ERATOI	156.299	3.002	ERATOI	154.682	2.684	ERATAU	163.432	2.603	ERATOI	152.15	3.175	ERATOI	161.517	2.762
ERAWAY	151.476	3.309	ERAWAY	162.138	3.309	ERATOI	163.648	3.475	ERAVUO	151.139	3.034	ERAWAY	150.923	3.046
EREHOY	155.865	3.064	EREHOY	150.998	3.471	EREHAM	152.394	2.915	EREHAM	174.896	2.787	EREHIS	155.801	3.218
ERENEW	151.852	2.925	ERENEW	151.622	2.83	EREHOY	158.372	3.056	EREHUE	151.687	3.264	ERENEW	155.816	3.387
ERERIF	161.902	3.472	ERERIF	170.428	3.465	ERENEW	153.553	3.168	ERERAX	164.065	3.277	EREREB	151.34	3.038
ERIFIU	154.89	3.443	ERIFIU	171.985	2.844	ERERUR	152.569	3.115	EREWUU	170.672	3.002	EREWUU	168.983	2.808
ERIFIU	157.226	3.237	ERIFIU	151.3	2.974	ERIFIU	150.503	3.13	ERIFIU	155.279	2.844	ERIFIU	154.976	3.211
ERIROO	153.022	3.414	ERIROO	162.922	2.994	ERIFIU	156.976	3.421	ERIFIU	153.446	3.417	ERIFIU	170.502	3.083
EROPUX	160.054	3.339	EROPUX	156.918	2.918	ERIROO	156.039	3.078	EROLUS	168.11	3.49	EROPUX	157.47	3.306
EROPUX	163.098	3.3	EROPUX	163.351	3.434	EROPUX	161.88	2.918	EROPUX	151.092	3.136	EROPUX	156.174	3.154
ERUHEE	156.854	3.399	ERUHEE	157.433	3.493	EROPUX	151.245	3.169	EROXIU	170.125	3.36	ERUHEE	161.479	3.288
ERUJIM	157.371	3.024	ERUJIM	160.69	3.37	ERUHEE	154.574	2.745	ERUHII	161.952	3.423	ERUHUX	161.425	2.937
ERUJIM	152.483	2.827	ERUJIM	150.906	2.763	ERUJIM	158.324	3.313	ERUJIM	159.974	2.777	ERUJIM	162.03	3.491
ERUJIM	154.711	3.124	ERUJIM	150.515	2.716	ERUJIM	164.493	3.437	ERUJIM	157.188	3.249	ERUJIM	159.776	3.313
ERUJIM	168.288	3.184	ERUJIM	154.611	3.2	ERUJIM	153.037	3.184	ERUJIM	157.926	3.086	ERUJIM	154.573	2.935
ERUJUY	155.905	2.751	ERUJUY	160.062	3.287	ERUJIM	177.272	3.182	ERUJIM	170.744	3.405	ERUJUY	164.189	3.075
ERUJUY01	164.18	3.075	ERUJUY01	155.716	2.752	ERUJUY	168.953	3.287	ERUJUY	174.375	3.158	ERUJUY	156.957	2.877
ERUJUY01	156.987	2.876	ERUKAF	163.94	3.234	ERUJUY01	159.967	3.287	ERUJUY01	168.975	3.287	ERUJUY01	174.287	3.159
ERUKAF	172.602	3.138	ERUKAF	151.487	3.447	ERUKAF	156.398	3.181	ERUKAF	167.417	2.957	ERUKAF	160.996	3.151
ERUKAF	159.417	3.099	ERUKAF	163.717	3.203	ERUKAF	168.816	3.175	ERUKAF	164.736	3.351	ERUKAF	158.394	3.324
ERUKAF	170.767	2.902	ERUKEJ	163.69	3.278	ERUKAF	151.022	2.876	ERUKAF	156.235	3.431	ERUKAF	166.232	2.829
ERUKEJ	162.711	3.254	ERUKEJ	151.883	3.442	ERUKEJ	158.352	3.316	ERUKEJ	168.027	3.01	ERUKEJ	155.035	3.134
ERUKEJ	171.161	2.911	ERUKEJ	165.196	2.806	ERUKEJ	167.443	3.173	ERUKEJ	172.749	3.15	ERUKEJ	162.039	3.199
ERUPIT	150.145	3.292	ERUPIT	159.875	2.923	ERUKEJ	156.642	3.41	ERUKEJ	160.176	3.1	ERUKEJ	164.802	3.187
ERUWAR	153.631	3.241	ERUWAR	152.235	3.231	ERUPIT	150.145	3.023	ERUPIT	156.918	3.235	ERUWAR	177.683	3.129
ERUWAR	157.085	2.976	ERUWAR	155.673	2.743	ERUWAR	174.05	3.347	ERUWAR	153.263	2.929	ERUWAR	167.664	3.166
ERUWAR	151.217	2.937	ERUWAR	154.262	3.395	ERUWAR	164.867	3.407	ERUWAR	150.326	3.254	ERUWAR	151.989	3.39
ERUWAR	158.476	2.997	ERUWAR	155.456	3.39	ERUWAR	164.56	3.456	ERUWAR	158.246	3.294	ERUWAR	163.718	3.367
ERUZOJ	151.119	3.116	ERUZOJ	158.248	3.485	ERUZOJ	151.107	3.02	ERUZOJ	158.725	3.07	ERUZOJ	161.725	2.865
ESAXAA23	163.975	3.105	ESAXAA23	166.757	3.087	ESAPOG0	171.003	2.809	ESAPOG0	161.059	3.149	ESATOH	162.486	3.109
ESAXAA24	163.508	3.063	ESAXAA25	163.527	3.066	ESAXAA23	153.981	3.276	ESAXAA24	153.169	3.288	ESAXAA24	166.462	3.045
ESAXAA26	166.511	3.055	ESAXAA26	153.299	3.284	ESAXAA25	166.474	3.049	ESAXAA25	153.189	3.287	ESAXAA26	163.621	3.071
ESAXAA28	163.729	3.087	ESAXAA28	166.653	3.068	ESAXAA27	163.63	3.079	ESAXAA27	166.551	3.063	ESAXAA27	153.375	3.286
ESAXAA29	153.47	3.288	ESAXAA30	163.918	3.099	ESAXAA28	153.414	3.288	ESAXAA29	163.833	3.092	ESAXAA29	166.675	3.076
ESAXAA31	166.891	3.092	ESAXAA31	153.703	3.287	ESAXAA30	166.832	3.084	ESAXAA30	153.762	3.284	ESAXAA31	164.065	3.106
ESAXAA33	164.227	3.121	ESAXAA33	166.991	3.106	ESAXAA32	164.145	3.114	ESAXAA32	166.907	3.097	ESAXAA32	153.813	3.287
ESAXAA34	153.857	3.29	ESCUHM	152.288	3.232	ESAXAA33	153.904	3.287	ESAXAA34	164.29	3.128	ESAXAA34	167.007	3.112
ESESAX	160.656	3.127	ESEVEG	172.142	3.277	ESCUHM	154.78	2.919	ESCUHM	152.062	3.208	ESENAR	160.43	3.131
ESIFAQ	158.107	2.844	ESIFAQ	168.976	2.844	ESEVEG	156.071	3.325	ESEXOP	161.371	3.025	ESIDUI	156.149	3.422
ESIMEZ	150.094	3.076	ESIMIF	169.669	3.047	ESIGOF	151.514	3.223	ESILUO	158.903	3.236	ESILUO	154.876	2.637
ESIPUR02	163.328	3.329	ESIPUR02	160.056	3.11	ESIPUR	164.148	3.303	ESIPUR	159.916	3.096	ESIPUR02	163.623	3.01
ESISEG	161.26	2.725	ESISEG	167.398	2.842	ESIQOO	165.317	3.462	ESIRII	150.518	3.336	ESIRII	165.723	2.877
ESOCIA	175.845	3.471	ESODUO	165.245	2.725	ESIWOV	152.816	3.339	ESOCIA	150.315	3.413	ESOCIA	151.003	3.104
ESODUO	152.791	3.067	ESODUO	174.664	3.416	ESODUO	174.923	3.228	ESODUO	152.112	3.175	ESODUO	159.215	3.415
ESOTAI	158.166	2.868	ESOWUF	151.967	2.886	ESOKIJ	172.692	3.269	ESOKIJ	164.357	3.411	ESOKIJ	173.273	2.945
ESUCAY	156.321	2.843	ESUCAY	151.241	2.855	ESOWUF	156.116	3.312	ESOWUF	152.271	2.862	ESOWUF	154.461	3.466
ESUSER	154.47	3.254	ESUSER	162.568	2.84	ESUDAZ	171.874	3.283	ESULUZ	174.67	3.079	ESUQIV	157.077	3.417
ESUSIV	163.336	2.944	ESUSIV	162.597	3.237	ESUSER	154.688	2.984	ESUSER	167.444	2.938	ESUSER	161.483	2.938
ETACUY	159.559	3.325	ETACUY	167.227	3.098	ESUTAO	158.3	2.881	ETABUW	150.566	3.472	ETABUW	153.121	3.246
ETAKAL	172.067	3.067	ETAKAL	157.61	3.014	ETAJAL	157.859	3.444	ETAJAL	150.827	3.199	ETAJAL	170.173	3.444
ETAKAL	163.584	3.168	ETAZOO	160.845	3.235	ETAKAL	172.897	2.74	ETAKAL	164.166	2.956	ETAKAL	163.915	3.4
ETIKEZ	167.762	2.857	ETIMAX	161.618	3.485	ETEDEO	162.047	3.03	ETIFUJ	161.542	3.466	ETIHEV	152.659	3.165
ETIMAX	160.053	2.741	ETIMAX	174.198	3.151	ETIMAX	150.725	3.163	ETIMAX	179.335	3.038	ETIMAX	153.912	2.984
ETIMAX	165.218	2.749	ETIMAX	151.187	3.423	ETIMAX	160.82	2.861	ETIMAX	170.127	2.993	ETIMAX	160.065	2.733
ETOBOH	167.468	3.257	ETOCAU	172.19	3.025	ETIMAX	164.988	3.143	ETINUS	153.938	3.211	ETOBOH	156.979	3.331
ETOPAF	157.546	3.425	ETOPAF	156.075	3.451	ETOCOI	151.707	2.823	ETOJED	171.89	3.201	ETOKAA	157.158	3.34
EVADUD	168.544	3.354	EVADUD	164.646	3.126	ETUDII	154.319	3.065	ETUDII	162.403	3.403	EVADUD	175.432	2.602
EVADUD	159.182	3.468	EVAFAL	150.638	2.705	EVADUD	158.569	3.456	EVADUD	152.62	2.809	EVADUD	171.17	3.209
EVAFAL	155.295	3.225	EVAFEP	172.559	3.287	EVAFAL	171.381	3.414	EVAFAL	168.339	2.885	EVAFAL	171.772	3.097
EVAFEP	174.313	3.45	EVAFEP	153.881	3.161	EVAFEP	150.106	2.896	EVAFEP	151.442	2.864	EVAFEP	165.647	2.853
EVAHAM	154.6	3.152	EVAHEQ	150.716	3.235	EVAFEP	155.539	2.713	EVAFEP	168.577	3.401	EVAFEP	158.924	3.154
EVANOH	154.116	3.028	EVAQAT	166.33	3.239	EVAKIW	168.644	3.15	EVAKIW	164.603	3.203	EVAMUK	153.786	3.263
EVATIF	176.041	2.863	EVATIF	168.715	2.877	EVAQAT	154.324	2.855	EVAQAT	159.603	3.242	EVAQAT	154.975	3.155
EVATOL	155.676	2.89	EVEDEP	151.96	2.953	EVATIF	169.508	2.908	EVATIF	174.06	3.015	EVATOL	162.266	2.994
EVEVEI	168.511	3.258	EVEVEI	162.178	3.25	EVEDEP	161.296	3.205	EVEVEI	155.139	3.16	EVEVEI	161.19	3.477
EVIBUG	151.391	3.095	EVIBUG	165.354	3.443	EVEZUD	150.983	3.058	EVEZUD	162.486	3.006	EVIBUG	152.076	3.238
EVIHAU	170.858	3.246	EVIHAU	163.165	3.349	EVIBUG	164.881	2.929	EVIBUG	155.81	3.235	EVIBUG	165.815	3.334
EVIKOJ	166.582	2.818	EVIKOJ	165.049	2.852	EVIHAU	156.895	2.852	EVIKOJ	151.756	2.846	EVIKOJ	160.252	3.309

EVIQIM	164.141	3.248	EVIQIM	156.459	3.085	EVIKOJ	150.178	2.73	EVIKOJ	157.624	3.34	EVINIG	154.689	2.768
EVIQIM	164.534	2.927	EVIQOS	151.028	3.389	EVIQIM	153.91	3.037	EVIQIM	158.372	3.283	EVIQIM	162.53	2.878
EVIQOS	154.24	2.726	EVIQOS	157.122	3.104	EVIQOS	152.526	3.026	EVIQOS	152.753	3.039	EVIQOS	155.67	3.107
EVUBUS	155.799	3.407	EVUBUS	167.385	3.419	EVOHAB	160.956	3.493	EVOHAB	161.064	2.925	EVOHAB	165.108	3.414
EVUDIL	177.605	3.328	EVUDIL	152.285	3.113	EVUBUS	152.631	3.256	EVUBUS	163.52	3.34	EVUBUS	150.595	3.34
EVUNIU	156.175	2.915	EVUNIU	169.194	2.836	EVUNIU	153.532	2.919	EVUNIU	151.887	3.429	EVUNIU	150.358	3.145
EVUNIU	161.646	3.284	EVUTOF	156.706	3.207	EVUNIU	170.037	2.712	EVUNIU	155.455	3.068	EVUNIU	153.195	3.335
EVUVOJ	156.434	3.229	EVUVOJ	157.278	3.081	EVUTOF	156.162	3.189	EVUVOJ	156.429	3.051	EVUVOJ	162.387	2.894
EVUVOJ	156.583	3.135	EVUVUP	156.542	2.989	EVUVOJ	174.794	2.894	EVUVOJ	161.944	2.894	EVUVOJ	151.968	2.921
EVUVUP	159.937	2.646	EVUVUP	155.56	2.81	EVUVUP	165.777	2.646	EVUVUP	157.38	2.862	EVUVUP	153.899	3.302
EVUVUP	151.344	2.81	EVUWAW	151	3.424	EVUVUP	153.513	3.147	EVUVUP	159.218	2.828	EVUVUP	156.837	3.28
EVUWAW	172.162	3.239	EVUWAW	150.868	2.92	EVUWAW	157.402	2.602	EVUWAW	154.298	3.239	EVUWAW	165.683	2.996
EVUXUQ	169.858	3.456	EVUXUQ	171.428	3.475	EVUXEB	153.681	3.247	EVUXEB	160.789	3.05	EVUXUQ	154.233	2.989
EWACOV	164.515	3.067	EWACOV	159.713	3.123	EVUZAY	166.814	3.383	EWABIO	168.251	2.59	EWACEL	156.031	3.276
EWADEM	161.285	3.291	EWADEM	170.977	3.137	EWACOV	160.256	2.762	EWACOV	164.753	2.837	EWADEM	164.079	2.852
EWADOY	173.683	2.71	EWAFIS	163.261	3.382	EWADOY	160.157	2.726	EWADOY	174.167	3.202	EWADOY	156.52	3.406
EWANUO	155.905	3.256	EWANUO	175.976	3.493	EWAFIS	153.525	3.004	EWALUL	165.424	3.032	EWALUL	162.28	2.835
EWERAZ	168.885	3.023	EWESUV	157.373	3.142	EWAZEK	154.157	3.188	EWAZEK	174.211	3.199	EWERAZ	171.328	3.32
EWETAC	154.258	3.487	EWETAC	162.754	2.839	EWESUV	159.712	3.257	EWESUV	154.855	3.242	EWESUV	167.25	3.033
EWEWEJ	155.182	3.16	EWIJEC	168.045	3.446	EWETAC	154.066	3.113	EWETAC	151.053	3.398	EWETAC	154.737	2.754
EWIZAN	168.827	3.207	EWIZAN	169.619	3.403	EWIXEO	177.425	2.735	EWIXEO	168.397	2.757	EWIXEO	161.328	3.021
EWOVUJ	156.758	3.09	EWOVUJ	158.155	2.925	EWIZAN	167.12	3.072	EWOGAZ	151.444	3.29	EWOGIG	156.489	2.768
EWOWEV	150.124	2.995	EWOWEV	164.936	2.68	EWOVUK	163.298	3.42	EWOWEV	167.759	2.738	EWOWEV	159.167	2.911
EWUHAF0	150.32	3.308	EWUKUD	163.673	3.278	EWOWEV	165.72	2.992	EWOXEW	150.718	3.127	EWUGEJ	164.193	3.448
EWUNAL	158.274	2.898	EWUNAL	174.051	3.004	EWUKUD	156.782	3.054	EWUNAL	152.393	3.308	EWUNAL	158.398	3.022
EWUNAL	150.971	3.496	EWUNEP	152.717	2.792	EWUNAL	152.003	3.096	EWUNAL	163.009	3.111	EWUNAL	163.513	3.145
EWUNEP	154.48	3.181	EWUNIT	170.093	3.186	EWUNEP	156.959	3.483	EWUNEP	161.896	3.287	EWUNEP	156.592	2.746
EWURUL	165.508	3.17	EWURUL	158.503	3.029	EWUNIT	152.876	2.872	EWUNIT	155.081	3.075	EWURUL	171.281	2.878
EWURUL	155.857	3.011	EWUSAS	163.163	2.779	EWURUL	158.031	3.277	EWURUL	150.647	3.01	EWURUL	167.86	3.094
EWUSAS	163.877	2.742	EWUSAS	167.659	3.305	EWUSAS	165.725	3.211	EWUSAS	151.872	3.298	EWUSAS	153.263	2.781
EXACUE	165.518	3.015	EXADEM	152.981	3.123	EWUSAS	150.458	3.467	EWUSAS	153.561	3.238	EWUSAS	168.19	2.985
EXAHER	154.379	3.349	EXAHOD	172.775	3.26	EXADEM	174.711	3.134	EXAGIV	151.961	3.399	EXAGIV	159.512	3.487
EXAMUL	151.4	2.996	EXAMUL	167.773	3.391	EXAMOF	158.927	3.358	EXAMOF	158.341	3.281	EXAMUL	153.642	3.457
EXANUO	156.341	3.328	EXEHUM	174.329	3.476	EXANUO	166.151	3.268	EXANUO	156.929	3.484	EXANUO	156.91	2.965
EXEMOK	152.757	3.347	EXENAX	151.341	3.291	EXEHUM	150.835	3.476	EXEHUM	169.397	3.318	EXEHUM	150.367	3.221
EXEPOO	164.531	3.037	EXEQUV	155.496	3.466	EXENAX	157.777	3.422	EXENAX	168.486	3.004	EXEPOO	151.226	3.088
EXEZAK	151.475	3.265	EXEZAK	152.883	3.331	EXETUX	176.054	2.915	EXEXIR	152.679	2.934	EXEZAK	157.709	3.016
EXEZAK	151.308	3.05	EXEZAK	157.367	2.81	EXEZAK	162.428	2.795	EXEZAK	172.773	3.274	EXEZAK	168.143	3.469
EXEZAK	166.605	2.881	EXEZAK	164.347	2.902	EXEZAK	159.818	3.184	EXEZAK	150.147	3.348	EXEZAK	170.472	2.742
EXEZAK	159.998	3.488	EXEZAK	168.463	3.309	EXEZAK	156.167	3.31	EXEZAK	163.289	3.108	EXEZAK	151.058	2.795
EXEZAK	170.52	2.639	EXIFAT	160.07	2.904	EXEZAK	151.637	3.039	EXEZAK	152.484	3.119	EXEZAK	154.169	3.118
EXIHAU	173.605	3.213	EXOKIO	150.068	2.834	EXIFAT	156.166	2.99	EXIFEX	160.329	3.143	EXIHAU	179.193	3.118
EXONAJ	150.831	3.359	EXONAJ	150.627	2.762	EXOKIO	153.195	3.401	EXONAJ	150.979	3.272	EXONAJ	153.595	2.788
EXONAJ	171.882	3.165	EXONAJ	150.089	3.163	EXONAJ	166.117	2.762	EXONAJ	150.648	3.404	EXONAJ	158.816	2.788
EXUBAD	170.687	3.328	EXUBAD	159.063	3.215	EXONAJ	155.213	2.873	EXOXEU	152.702	3.02	EXUBAD	173.225	3.147
EXUPUK	151.756	3.451	EXUPUK	161.924	3.337	EXUJOZ	172.263	3.155	EXUJOZ	171.835	3.327	EXUJOZ	157.617	3.232
EXUQUL	162.75	3.348	EXURUN0	166.112	3.425	EXUPUK	151.887	3.45	EXUPUK	163.469	3.45	EXUPUK	163.164	3.126
EXUTOH	157.722	2.752	EXUTOH	150.06	3.034	EXURUN0	166.786	3.079	EXURUN0	157.414	3.294	EXURUN0	154.47	3.484
EYACAJ	157.949	3.068	EYAGUJ	156.292	3.5	EXUTOH	158.87	3.358	EXUTOH	164.832	2.752	EXUXIH	172.732	3.273
EYEJOI	163.01	3.298	EYEJOI	171.327	2.924	EYAGUJ	158.854	2.974	EYEHIB	156.68	3.11	EYEJOI	151.086	2.924
EYEJUO	157.942	3.448	EYEJUO	153.674	3.439	EYEJUO	154.894	3.138	EYEJUO	152.542	3.408	EYEJUO	158.445	3.032
EYEZOZ	171.011	2.954	EYEZUF	154.96	2.674	EYEJUO	151.198	3.448	EYETAF	163.257	3.376	EYEZOZ	159.232	3.407
EYICIY	150.548	3.263	EYICOE	150.745	2.942	EYEZUF	163.23	2.674	EYICIY	163.995	3.436	EYICIY	169.063	3.039
EYINAC	158.318	3.461	EYINAC	165.871	3.17	EYICOE	164.487	3.488	EYICOE	174.757	3.485	EYINAC	155.88	3.384
EYINAC	169.076	2.786	EYINOS	150.019	3.053	EYINAC	162.173	2.835	EYINAC	158.276	3.007	EYINAC	173.193	3.49
EYIQEK	159.512	3.396	EYIQEK	173.554	2.951	EYIQEK	170.166	3.003	EYIQEK	151.118	3.383	EYIQEK	150.612	3.1
EYITUC	154.601	3.075	EYIYIV	153.313	3.048	EYIROU	158.699	3.364	EYISAH	154.889	3.065	EYISAH	154.665	3.028
EYIYOB	165.091	3.073	EYIYUH	159.015	2.992	EYIYIV	157.048	3.094	EYIYOB	176.336	3.137	EYIYOB	177.867	3.292
EYIZAO	170.56	3.213	EYIZAO	178.734	3.02	EYIYUH	169.845	3.042	EYIZAO	150.469	3.438	EYIZAO	172.224	3.256
EYIZOC	167.561	3.152	EYIZOC	157.951	3.039	EYIZES	158.554	3.101	EYIZES	169.529	3.369	EYIZES	152.153	3.243
EYOSEQ	153.259	3.223	EYOSEQ	156.15	3.43	EYIZUI	160.254	3.221	EYIZUI	171.265	3.283	EYOSEQ	152.794	3.427
EYUCEJ	167.728	2.759	EYUDUZ	159.885	3.43	EYOXOH	156.429	2.755	EYOXOH	158.458	2.955	EYUCEH	161.161	3.246
EYUSOG	159.067	2.841	EYUSOG	156.889	2.939	EYUJIT	150.22	3.371	EYURUO	151.485	2.709	EYUSEZ01	151.306	3.248
EYUSOG	165.809	3.21	EYUSUM	164.257	3.061	EYUSOG	152.872	3.409	EYUSOG	150.671	3.226	EYUSOG	157.46	2.944
EZEDAP	158.389	2.879	EZETIM	156.235	3.366	EYUSUM	172.368	3.32	EYUSUM	167.081	3.401	EZAMAV	158.533	3.419
EZETIM	164.349	3.404	EZETIM	170.008	3.305	EZETIM	152.642	2.677	EZETIM	152.461	3.404	EZETIM	150.622	3.054
EZIMIM	176.5	3.412	EZIQEM	155.106	3.225	EZETIM	165.824	3.121	EZIMIM	167.14	2.811	EZIMIM	166.639	2.811
EZOHUW	150.139	3.129	EZOHUW	157.071	2.884	EZIQEM01	155.087	3.224	EZOBIF02	157.823	3.247	EZOBIF02	152.114	2.847
EZOHUW	162.052	2.995	EZOHUW	155.855	3.175	EZOHUW	169.692	2.966	EZOHUW	151.719	3.357	EZOHUW	164.925	3.275
EZOHUW	164.49	3.046	EZOHUW	168.072	3.345	EZOHUW	161.21	3.298	EZOHUW	157.076	3.058	EZOHUW	177.333	3.095
EZOTUJ	165.392	2.758	EZOVUL	164.207	3.231	EZOHUW	167.494	2.947	EZONOY	157.677	3.219	EZOPOZ	179.61	3.246
EZOWIA	162.698	3.002	EZOWIA	165.147	3.255	EZOVUL	154.673	3.071	EZOWEW	161.561	3.066	EZOWIA	172.834	2.821
EZOWIA	161.125	3.395	EZOWIA	159.963	2.973	EZOWIA	157.694	3.351	EZOWIA	157.639	3.488	EZOWIA	150.212	3.016
EZUXUS	169.044	3.069	EZUXUS	153.7	2.882	EZOWIA	167.033	3.332	EZUJEQ	164.283	2.753	EZUXUS	160.823	3.409
EZUXUS	152.357	3.094	EZUYAZ	151.186	3.332	EZUXUS	166.037	3.239	EZUXUS	169.516	3.391	EZUXUS	161.594	3.239
FABBAN	161.751	3.333	FABBOE	156.854	2.888	EZUYAZ	162.594	3.424	EZUZUX	159.157	3.421	EZUZUX	159.863	3.274
FABBOE	160.635	3.355	FABBOE	157.51	3.487	FABBOE	165.709	3.26	FABBOE	162.728	2.826	FABBOE	154.157	3.355
FABBOE	151.897	2.865	FABBOE	163.07	2.845	FABBOE	176.949	2.96	FABBOE	155.291	3.358	FABBOE	155.332	3.21
FABKOM	166.46	2.898	FABKOM	168.88	2.959	FABHEA	150.292	2.753	FABHEA	161.482	3.096	FABHEA	162.357	2.839

FABRAE	171.856	3.215	FABRAE	172.768	3.248	FABLUT	165.834	3.197	FABLUT	168.313	3.009	FABQEE	168.694	3.212
FABSOT	154.36	3.024	FABSOT	157.73	2.789	FABSOT	151.404	3.435	FABSOT	155.943	2.81	FABSOT	158.102	3.001
FABSOT	166.384	2.896	FABSOT	166.992	3.076	FABSOT	151.087	2.807	FABSOT	160.346	3.149	FABSOT	154.366	3.339
FABSUZ	151.247	3.433	FABSUZ	155.876	2.817	FABSOT	174.471	2.659	FABSOT	164.906	2.808	FABSOT	160.67	3.108
FABSUZ	150.753	2.815	FABSUZ	160.335	3.142	FABSUZ	158.077	3.003	FABSUZ	154.26	3.029	FABSUZ	158.002	2.794
FABSUZ	174.208	2.659	FABSUZ	164.824	2.808	FABSUZ	154.253	3.325	FABSUZ	166.487	2.896	FABSUZ	166.661	3.085
FABXIT	156.83	2.848	FABXIT	150.967	3.197	FABSUZ	160.358	3.115	FABXAK	158.52	3.498	FABXIT	159.938	3.275
FACCRE	150.2	3.385	FACCRE	165.392	2.93	FACAPR0	161.227	3.118	FACAPR0	164.443	2.97	FACCRE	156.045	3.419
FACFOE	171.547	2.857	FACGEV	162.723	3.421	FACCRE	167.28	2.949	FACETC0	165.784	3.061	FACETC1	165.601	3.131
FACJEB	151.272	2.888	FACJEB	151.319	2.943	FACHUP	164.07	3.16	FACJAU	160.871	3.201	FACJAU01	161.075	3.199
FACJEB	154.099	2.831	FACJEB	156.756	2.941	FACJEB	153.062	2.888	FACJEB	158.304	2.942	FACJEB	153.917	2.831
FACJIF	152.136	2.882	FACJIF	151.24	2.945	FACJEB	152.929	2.972	FACJEB	150	2.831	FACJEB	156.907	2.895
FACJIF	153.857	2.832	FACJIF	157.297	2.938	FACJIF	152.978	2.882	FACJIF	158.76	2.939	FACJIF	154.277	2.834
FACJUR	151.522	2.899	FACJUR	150.459	2.953	FACJIF	152.4	2.972	FACJIF	150.295	2.834	FACJIF	157.214	2.888
FACJUR	153.357	2.833	FACJUR	156.753	2.953	FACJUR	153.443	2.899	FACJUR	158.013	2.955	FACJUR	153.624	2.837
FACKEC	150.068	2.735	FACKEC	156.175	3.072	FACJUR	152.211	2.989	FACJUR	156.981	2.907	FACKEC	153.581	2.768
FACKEC	160.766	3.015	FACKEC	153.269	2.726	FACKEC	151.235	2.781	FACKEC	151.862	2.892	FACKEC	150.434	2.768
FACKOM	151.007	2.779	FACKOM	151.444	2.909	FACKEC	160.632	2.815	FACKOM	153.198	2.785	FACKOM	156.003	3.073
FACKOM	160.677	2.82	FACLAZ	157.839	2.796	FACKOM	150.697	2.785	FACKOM	160.772	3.02	FACKOM	153.019	2.736
FACLAZ	151.567	3.438	FACLAZ	151.119	2.812	FACLAZ	154.372	3.03	FACLAZ	157.987	3.01	FACLAZ	155.602	2.82
FACLAZ	167.153	3.083	FACLAZ	174.325	2.661	FACLAZ	160.346	3.143	FACLAZ	154.415	3.336	FACLAZ	166.42	2.902
FACLED	155.895	2.807	FACLED	158.118	3.008	FACLAZ	165.101	2.812	FACLAZ	160.725	3.111	FACLED	151.619	3.45
FACLED	160.391	3.169	FACLED	154.485	3.351	FACLED	154.449	3.032	FACLED	157.776	2.786	FACLED	151.677	2.798
FACLED	165.136	2.811	FACLED	161.085	3.109	FACLED	166.498	2.902	FACLED	167.234	3.079	FACLED	174.932	2.666
FACMOL	157.963	2.895	FACMOL	164.174	3.094	FACMOL	157.709	3.215	FACMOL	155.724	3.225	FACMOL	153.183	2.962
FACPOS	157.418	3.447	FACRIL	161.984	3.303	FACMOL	150.146	3.345	FACPAA0	163.348	3.38	FACPAA0	165.119	3.38
FACWUF	160.76	2.804	FACYER	161.102	3.247	FACRIL	152.053	2.682	FACTNS	163.223	3.007	FACTNS	153.663	3.181
FACYER	164.102	2.929	FACZUH	150.162	3.368	FACYER	156.523	3.045	FACYER	158.052	3.301	FACYER	165.829	3.302
FADFEY	152.601	3.103	FADHUN	165.277	3.374	FACZUH	168.567	2.997	FACZUH	156.487	3.157	FADBOE	159.384	3.335
FADXIV	163.511	2.867	FADXIV	151.419	2.844	FADJIH	163.218	3.031	FADXAN	159.631	3.383	FADXER	156.113	2.975
FAFQOW	167.047	3.388	FAFYUH	170.929	2.757	FADXIV	158.915	2.971	FADZEQ	161.517	2.651	FAFQOW	153.851	3.437
FAGDUO	159.049	2.737	FAGDUQ	151.368	2.695	FAFYUH	164.983	3.054	FAFZAQ	159.922	3.056	FAGDUO	166.383	3.411
FAHTER0	152.335	3.199	FAHTER0	164.722	3.15	FAGYOF	155.469	3.127	FAHGME1	175.9	2.688	FAHGME1	157.92	3.164
FAHTOB0	151.395	3.27	FAHTOZ	166.403	3.157	FAHTER0	162.89	2.901	FAHTER0	168.383	2.907	FAHTOB0	166.497	2.758
FAHWER	161.649	2.861	FAHWER	157.673	2.802	FAHTOZ	168.241	3.247	FAHVER	164.555	2.895	FAHVER	150.918	3.339
FAHWIV	150.559	2.916	FAHWIV	151.405	3.28	FAHWIV	166.153	3.328	FAHWIV	161.094	2.976	FAHWIV	160.657	3.435
FAHWIV	163.634	2.726	FAHWIV	153.533	3.095	FAHWIV	152.623	2.95	FAHWIV	157.006	3.103	FAHWIV	174.041	2.741
FAHWOB	159.017	2.84	FAHWOB	159.728	3.347	FAHWIV	157.043	3.419	FAHWOB	155.13	3.15	FAHWOB	155.735	2.781
FAHYIY	155.874	3.03	FAHYIY	151.444	3.421	FAHWOB	168.113	2.968	FAHYEU	154.183	2.773	FAHYEU	176.734	2.773
FAHZOE	152.014	3.365	FAHZOE	166.237	2.747	FAHYIY	151.993	2.95	FAHYIY	165.609	3.029	FAHZOE	155.211	2.753
FAJPEM0	175.105	3.071	FAJPEM0	162.214	3.008	FAJMOT	158.062	3.215	FAJMOT	154.744	3.002	FAJPEM0	169.112	3.057
FAJPEN	169.369	3.096	FAJPEN	150.515	3.451	FAJPEM0	169.728	3.184	FAJPEM0	162.491	3.184	FAJPEM0	155.378	2.882
FAKNUD	166.247	2.947	FAKNUD	173.374	2.866	FAJVER	170.943	3.341	FAJVER	175.543	3.341	FAKNUD	160.64	2.626
FAKPIT	151.888	3.031	FAKPIT	159.646	3.031	FAKNUD	162.296	2.76	FAKPAL	151.098	3.113	FAKPAL	152.743	3.272
FAKQIS	150.382	3.026	FAKQIS	162.761	3.326	FAKPIT	161.292	3.168	FAKPIT	164.397	3.168	FAKPIT	153.628	3.167
FAKQIS	168.132	2.944	FAKVAS	153.592	2.852	FAKQIS	157.92	3	FAKQIS	152.421	3.494	FAKQIS	164.368	3.359
FALSUG	167.71	3.448	FAMBOL	160.14	3.065	FAKVEW	158.308	2.75	FAKWEW	154.75	3.181	FAKWEW	152.573	3.268
FAMBOL	160.001	2.793	FAMBOL	151.599	3.065	FAMBOL	161.559	3	FAMBOL	175.39	3.434	FAMBOL	157.277	3.186
FAMBUR	163.605	2.808	FAMBUR	157.059	3.477	FAMBOL	161.993	3.186	FAMBOL	169.446	2.845	FAMBOL	150.728	3.177
FAMCUS	159.194	3.036	FAMDAZ	164.723	3.437	FAMBUR	173.188	3.437	FAMBUR	162.576	2.83	FAMCAB	160.98	3.082
FAMGEI	159.578	3.463	FAMGEI	161.299	3.483	FAMDAZ	162.223	3.437	FAMGEI	162.276	3.463	FAMGEI	151.668	2.94
FAMNAM	165.538	2.829	FANFAB	167.152	2.803	FAMLUC	161.671	2.942	FAMNAM	160.2	2.896	FAMNAM	154.451	3.25
FANKOX	164.909	2.903	FANKOX	171.693	2.672	FANFAB	155.117	2.874	FANFAB	151.889	3.309	FANKOX	168.027	3.136
FANKUD	155.701	3.063	FANLUE	166.512	2.988	FANKUD	166.081	2.952	FANKUD	175.028	2.976	FANKUD	160.01	3.092
FANNUE	159.287	3.104	FANNUE0	163.831	2.98	FANLUE	175.325	2.704	FANLUF	152.926	3.291	FANNUE	164.538	2.997
FANTAT	165.413	3.394	FANTAT	163.664	3.226	FANNUE0	160.412	2.888	FANPAM	161.624	2.948	FANPAM	161.05	3.053
FANXAV	164.873	3.327	FANXEZ	162.946	3.2	FANWOJ	153.174	2.79	FANWUO	174.462	2.72	FANWUO	157.643	2.872
FANYIG	171.34	2.933	FANYIG	154.51	3.133	FANXID	175.503	2.771	FANXID	152.985	3.111	FANXID	173.676	2.756
FANYIG	159.866	3.345	FANYIG	160.376	3.24	FANYIG	159.284	3.24	FANYIG	154.165	3.42	FANYIG	151.45	3.272
FANYIG	164.338	3.133	FANYIG	150.947	2.818	FANYIG	154.715	3.272	FANYIG	152.647	3.246	FANYIG	151.908	3.378
FANYOM	154.549	3.425	FANYOM	158.385	2.976	FANYIG	155.806	3.322	FANYOM	150.64	3.468	FANYOM	166.234	2.986
FANZED	166.128	3.439	FANZED	155.114	3.063	FANYOM	153.102	3	FANYOM	150.178	3.207	FANYOM	158.085	3.019
FANZED	151.295	3.113	FANZED	151.62	3.101	FANZED	151.408	3.076	FANZED	167.146	2.912	FANZED	156.489	2.626
FAPBAB	157.127	3.496	FAPGAH	159.352	3.483	FANZED	173.733	2.58	FANZED	170.677	2.58	FANZED	153.228	3.238
FAPGEK	153.592	2.946	FAPGEK	165.854	3.212	FAPGAH	152.84	3.435	FAPGEK	164.49	3.334	FAPGEK	158.085	2.806
FAPGEL	151.875	3.484	FAPGEL	178.654	3.271	FAPGEK	155.177	3.197	FAPGEL	151.101	3.435	FAPGEL	171.041	3.187
FAPGIO	163.502	3.201	FAPGIO	157.878	3.35	FAPGIO	162.085	3.285	FAPGIO	157.729	3.052	FAPGIO	174.858	3.315
FAPGUB	150.69	3.336	FAPLAN	157.079	2.838	FAPGIO	158.992	2.715	FAPGIO	157.548	3.043	FAPGOU	161.627	3.186
FAPZTH1	169.299	3.293	FAPZTH1	150.108	2.871	FAPLAN	152.121	3.386	FAPZTH1	167.94	2.834	FAPZTH1	151.478	2.826
FAQGOT	166.706	2.793	FAQGOT	163.16	2.848	FAPZTH1	159.626	3.355	FAPZTH1	162.201	3.272	FAPZTH1	150.141	3.383
FAQGOT	151.91	3.017	FAQGOT	161.755	3.268	FAQGOT	150.082	3.088	FAQGOT	159.706	2.793	FAQGOT	168.29	3.307
FAQNUG	164.183	3.009	FAQNUG	173.233	2.809	FAQGOT	176.383	3.017	FAQGOT	153.914	3.389	FAQGOT	168.978	3.312
FARMNC1	159.766	3.181	FARPEU	175.527	3.087	FAQREW	162.107	3.016	FARGEO	164.805	2.905	FARJUG	151.334	3.169
FASFOY	164.208	3.291	FASGAL	162.258	2.949	FARPEU	160.587	3.012	FARPOG	163.647	2.951	FASFIS	165.348	3.085
FASGEL	157.657	3.081	FASGEL	160.932	2.919	FASGAL	164.689	2.949	FASGAL0	164.686	2.954	FASGAL0	162.229	2.954
FASHAM	164.573	3.299	FASJES	166.636	3.422	FASGEL	152.671	3.11	FASGOZ	151.086	3.167	FASHAM	163.845	3.299
FASLIY	156.006	3.059	FASNET	168.341	3.203	FASKER	157.309	2.962	FASKER	156.607	2.884	FASKES	155.373	2.929
FASNIX	151.392	3.109	FASNIX	151.259	3.111	FASNET	150.407	3.033	FASNET	171.379	3.236	FASNEW	163.59	2.936
FATCUB	153.978	3.422	FATCUB	152.109	3.124	FASNOD	156.199	3.443	FASNOD	153.896	3.295	FASNUJ	163.565	2.918

FATMIX01	159.945	3.274	FATMIX01	159.663	3.286	FATMIX	155.04	3.09	FATMIX	161.328	3.148	FATMIX01	157.334	2.618
FATTIH	166.602	3.249	FATTIH	154.055	2.903	FATMIX01	157.426	3.107	FATMUJ	157.422	3.095	FATTIH	176.311	2.944
FATVUV	173.079	3.444	FATVUV	152.685	2.903	FATTIH	155.307	3.016	FATTIH	169.489	2.799	FATVUV	170.187	3.369
FATVUV	155.317	3.325	FATVUV	159.333	2.882	FATVUV	151.246	2.839	FATVUV	150.759	3.008	FATVUV	154.537	2.694
FATVUV	176.85	3.065	FATVUV	156.421	3.367	FATVUV	165.768	2.701	FATVUV	157.191	2.963	FATVUV	178.192	2.989
FAVJIZ	153.466	3.136	FAVJIZ	164.422	3.053	FATVUV	153.716	3.054	FAVJIZ	168.99	3.009	FAVJIZ	155.208	3.241
FAVJIZ	151.922	2.623	FAVJIZ	161.933	3.321	FAVJIZ	167.239	3.085	FAVJIZ	163.621	2.651	FAVJIZ	159.902	2.797
FAVJIZ	151.856	2.753	FAVJIZ	158.728	2.922	FAVJIZ	151.843	2.917	FAVJIZ	154.265	2.651	FAVJIZ	150.293	3.241
FAVLOF	160.517	3.127	FAVLOF	165.356	3.338	FAVJIZ	156.733	3.109	FAVJIZ	151.878	3.051	FAVLOF	156.089	3.338
FAWBIQ	155.019	3.028	FAWFIW	173.044	3.048	FAVLOF	152.518	3.031	FAVQIC	155.529	3.189	FAWBEM	166.01	3.463
FAWKIX01	155.094	2.94	FAWKIX01	153.069	3.012	FAWFIW	161.981	3.487	FAWFIW	157.058	3.487	FAWFOY0	150.559	3.376
FAWPUP	157.297	3.25	FAWREE	153.561	2.883	FAWPUP	172.606	3.005	FAWPUP	165.542	2.711	FAWPUP	153.602	3.189
FAWYEJ	156.692	2.946	FAWYEJ	150.294	2.946	FAWREE	151.165	3.213	FAWVOQ	163.576	3.188	FAWYEJ	151.282	2.984
FAXMAW	158.906	2.699	FAXZUB	162.085	3.366	FAXBEN	158.076	2.969	FAXKAR	163.03	3.412	FAXMAW	158.438	3.025
FAYBOA	158.059	3.486	FAYBOA	167.502	3.062	FAYBOA	164.096	3.286	FAYBOA	152.992	3.378	FAYBOA	152.297	2.701
FAZCUA	154.509	3.133	FAZFAN	157.072	3.187	FAYXAH	152.122	2.928	FAYXAH	174.172	2.843	FAZCUA	162.317	3.332
FAZGES02	167.243	3.031	FAZGES02	159.818	3.128	FAZFAN10	156.621	3.194	FAZGES01	167.35	3.059	FAZGES01	160.21	3.199
FAZHIX10	152.414	3.386	FAZHIX10	161.604	2.812	FAZHIX	152.414	3.386	FAZHIX	161.604	2.812	FAZHIX	152.213	3.342
FAZNAW	165.751	3.11	FAZRAC	170.324	2.862	FAZHIX10	152.213	3.342	FAZMIG	164.501	2.953	FAZMIG	152.627	3.196
FAZZAK	154.543	3.211	FAZZAK	157.168	3.098	FAZRIH	154.279	3.2	FAZVUA	172.675	2.917	FAZVUA	158.09	2.991
FAZZEO	150.229	3.398	FAZZEO	156.246	2.759	FAZZAK	150.047	3.152	FAZZAK	166.018	3.255	FAZZEO	155.645	3.344
FBYTMO	159.396	3.234	FEBHAY	175.48	2.944	FBYTMO	161.363	2.97	FBYTMO	155.959	3.426	FBYTMO	162	2.944
FEBHOM	151.202	3.481	FEBLUW	156.919	3.076	FEBHAY	154.799	3.121	FEBHAY	166.426	3.185	FEBHOM	158.422	3.254
FEBLUW	160.711	2.91	FEBNOS	171.69	2.742	FEBLUW	169.55	2.98	FEBLUW	170.174	3.057	FEBLUW	171.487	3.087
FEBNOS	164.188	2.838	FEBPIO	166.929	3.274	FEBNOS	161.938	3.327	FEBNOS	150.489	3.129	FEBNOS	165.104	3.013
FEBWIS	155.523	3.411	FEBWOY0	164.009	2.76	FEBROU	161.788	3.027	FEBROU	153.813	3.04	FEBWEO	156.756	2.868
FECDUM	164.675	3.488	FECDUM	159.08	3.249	FEBWOY0	152.752	2.871	FEBXIX	155.267	3.3	FECDUM	159.238	2.952
FEDFED	151.596	3.281	FEDFED	169.261	3.17	FECTAM	157.438	3.34	FEDFAV	154.741	3.295	FEDFAV	150.066	3.338
FEDNOR	159.96	3.382	FEDNOR	151.923	3.173	FEDNEH	153.55	3.119	FEDNEH	157.939	3.344	FEDNOR	159.791	3.087
FEDXIX	154.792	3.441	FEDXIX	168.962	3.295	FEDNOR	169.328	3.243	FEDNOR	161.645	3.382	FEDNOR	151.979	3.482
FEDZET	157.381	2.934	FEFFOM	150.358	2.956	FEDZET	155.797	3.27	FEDZET	152.929	3.148	FEDZET	151.083	3.333
FEFKOT	169.91	3.088	FEFKOT	154.074	3.131	FEFFOM	171.411	3.074	FEFFUS	155.046	3.161	FEFFUS	150.611	3.178
FEFSEP	151.149	3.207	FEFSEP	157.034	3.352	FEFKOT	154.282	3.173	FEFKOT	160.312	3.105	FEFKOT	156.905	3.414
FEFSIT	151.269	3.258	FEFSOC	157.653	3.076	FEFSEP	175.681	3.214	FEFSIT	170.084	2.759	FEFSIT	169.431	2.759
FEFSOC	152.113	3.162	FEFSOC	150.209	3.162	FEFSOC	150.804	2.806	FEFSOC	152.021	3.292	FEFSOC	162.941	3.475
FEFWUM	154.001	3.452	FEFWUM	159.079	3.452	FEFWOG	152.601	3.153	FEFWOG	151.611	2.792	FEFWOG	169.673	2.858
FEFXOH	167.317	3.277	FEFXOH	167.079	3.207	FEFXEX	162.475	3.16	FEFXOH	169.431	2.892	FEFXOH	173.276	3.277
FEGCOK	165.055	3.299	FEGKIL	152.747	2.877	FEFXOH	178.151	3.013	FEGBAW	151.746	3.27	FEGBAW	163.394	3.448
FEGKIL	159.18	3.069	FEGKIL	152.894	3.318	FEGKIL	150.987	2.773	FEGKIL	153.008	3.25	FEGKIL	158.333	3.35
FEGZEY	152.171	3.42	FEHCED	159.049	3.191	FEGKIL	159.952	3.305	FEGZEY	168.09	3.132	FEGZEY	161.272	2.823
FEHHUZ	154.616	2.761	FEHHUZ	165.148	2.761	FEHGUY	169.684	2.864	FEHHUZ	178.143	3.214	FEHHUZ	165.869	3.117
FEHRUG	154.916	2.694	FEHSAP	162.001	3.16	FEHRAN	157.105	3.313	FEHRAO	171.843	3.046	FEHRIW	166.991	3.057
FEHTUI	164.25	3.031	FEHVAQ	171.808	3.149	FEHTUI	155.515	3.495	FEHTUI	171.128	3.031	FEHTUI	153.242	3.495
FEHVUL01	154.161	3.326	FEHZIF	158.455	3.407	FEHVUL01	171	3.326	FEHVUL01	168.814	3.032	FEHVUL01	165.614	3.458
FEJMIU	166.459	3.472	FEJMIU	153.332	3.063	FEIRUD10	167.838	3.178	FEIRUD10	156.981	3.167	FEJMIU	159.378	3.331
FEJMIU	166.158	2.698	FEJMIU	154.514	2.953	FEJMIU	155.069	3.023	FEJMIU	171.163	3.053	FEJMIU	169.523	2.861
FEJMIU	150.599	3.364	FEJMIU	164.899	2.962	FEJMIU	151.197	2.993	FEJMIU	167.252	2.861	FEJMIU	155.805	3.14
FEJMIU	171.097	2.993	FEJMIU	165.613	3.467	FEJMIU	150.099	3.3	FEJMIU	151.871	2.98	FEJMIU	160.372	3.076
FEJQAN	172.381	2.922	FEJQAN	168.625	3.487	FEJQAN	159.581	3.241	FEJQAN	156.251	3.41	FEJQAN	150.644	3.122
FEJXAW	158.953	2.78	FEJXOK	158.199	3.213	FEJSAP	165.78	3.077	FEJWID	163.017	3.361	FEJWUP	166.143	2.867
FEKQOF	167.523	2.752	FEKSUN	163.345	3.173	FEJXOK	157.361	3.363	FEKDAA	174.049	3.139	FEKQOF	150.271	2.752
FELLOY	169.327	3.045	FELLOY	174.289	2.996	FEKWIC	151.084	3.223	FEKWIC	170.845	2.957	FEKWIC	153.263	3.238
FELRAQ	166.461	3.05	FELRAQ	154.075	2.995	FELNUF	159.002	3.142	FELQOD	170.336	3.455	FELQOD	155.24	3.357
FELRAQ	165.99	2.929	FELZIH	152.786	3.359	FELRAQ	151.834	3.263	FELRAQ	155.731	3.126	FELRAQ	151.333	3.126
FEMWAW	152.594	3.291	FEMXEA	173.155	3.318	FELZIH	160.378	2.897	FELZIH	153.075	3.067	FEMVUS	164.01	2.941
FEMXEA	169.634	2.978	FEMXEA	166.444	3.002	FEMXEA	155.395	3.371	FEMXEA	167.241	2.795	FEMXEA	168.523	2.951
FEMZIK	157.341	2.624	FEMZIK	152.088	3.109	FEMXEA	156.186	3.279	FEMZIK	162.281	2.624	FEMZIK	150.695	3.358
FENBUV	151.904	3.299	FENCOS	160.738	2.951	FEMZIK	150.59	3.151	FEMZIK	167.603	3.109	FENBUV	150.94	2.989
FENDUX1	165.273	3.403	FENDUX1	158.881	3.219	FENDUX	165.273	3.403	FENDUX	158.881	3.219	FENDUX	164.101	3.307
FENFAH01	165.022	3.239	FENFOV	153.688	3.115	FENDUX1	164.101	3.307	FENDUZ	171.89	3.102	FENFAH	165.001	3.24
FENMIY	176.207	3.219	FENMIY	171.768	2.938	FENFOV	154.489	3.31	FENKEP	167.365	3.428	FENKIT	176.332	3.289
FENMOB	162.684	3.31	FENMOB	163.95	3.221	FENMIY	161.617	3.14	FENMIY	162.528	3.055	FENMIY	155.854	3.37
FENNAR	155.446	2.933	FENNAR	152.14	2.927	FENMOB	158.642	3.376	FENMOB	165.519	3.379	FENNAR	161.338	3.396
FENNAR	156.71	3.376	FENNAR	155.59	3.396	FENNAR	165.295	3.134	FENNAR	159.666	3.18	FENNAR	158.53	2.9
FENNAR	150.09	3.214	FENSAW	153.031	3.442	FENNAR	159.505	2.968	FENNAR	167.571	2.903	FENNAR	150.718	2.9
FEPLES	159.575	3.216	FEPLES	173.742	2.878	FENTOI	156.876	3.04	FEPCIO	166.88	3.011	FEPKIW	159.681	3.386
FEPWUV	158.06	3.351	FEQBIP	167.769	3.434	FEPLOC	154.256	2.874	FEPLOF	164.706	3.467	FEPWOM	162.16	3.268
FEQVAC	157.684	3.247	FEQVAC	165.539	2.842	FEQPAV01	165.633	3.417	FEQPAV02	166.095	3.422	FEQVAC	158.666	3.352
FEQVAC0	158.231	3.333	FEQVAC0	157.786	3.231	FEQVAC	162.012	3.15	FEQVAC0	165.526	2.815	FEQVAC0	162.542	3.13
FEQZAD	165.344	2.974	FEQZAD	168.48	2.934	FEQVIJ	176.483	3.218	FEQXOQ	160.574	3.2	FEQXOQ	162.681	3.245
FERHIT	154.829	3.309	FERHIT	154.186	3.394	FEQZEH	166.387	2.931	FEQZEH	154.308	3.027	FEQZEH	152.619	2.931
FERNUN	158.039	3.449	FERPOL	152.128	3.307	FERJEU	150.536	3.325	FERJEU	166.805	3.272	FERJEU	161.753	3.388
FESFOB	152.821	3.041	FESJAS	154.385	3.475	FERRUT	171.056	3.408	FERRUT	165.282	3.165	FERYUY	150.745	3.003
FESPID	150.424	3.275	FETBAH	150.794	3.461	FESJAS	153.172	3.069	FESJEW	151.493	3.088	FESJEW	154.206	3.474
FETBAH	162.244	2.84	FETDUG	163.698	2.918	FETBAH	151.121	2.84	FETBAH	155.478	3.478	FETBAH	160.063	3.461
FETJIB02	161.17	3.282	FETWOU	162.009	3.167	FETJIB01	156.103	3.179	FETJIB01	158.192	3.348	FETJIB02	157.563	3.117
FETXOV	160.216	3.048	FETXOV	161.088	3.037	FETXOV	151.81	3.392	FETXOV	168.49	2.989	FETXOV	174.798	3.24
FEVHET	165.604	3.11	FEVHET	151.593	3.197	FETXOV	163.301	3.29	FETXOV	160.575	3.325	FEVHET	170.766	2.805
FEVHET	152.425	3.149	FEVKOI	152.451	3.025	FEVHET	165.727	2.818	FEVHET	164.424	3.049	FEVHET	158.223	3.217

FEVQUU	167.835	2.67	FEVQUU	163.65	3.419	FEVLUR	156.018	3.325	FEVQUU	161.706	2.855	FEVQUU	157.575	3.425
FEVSIL	159.628	3.331	FEVSIL	154.085	3.322	FEVRUW	150.695	2.677	FEVSAE	169.741	3.226	FEVSAE	153.457	3.226
FEVSUX01	167.832	3.296	FEWCIV01	158.586	3.442	FEVSUX	169.89	2.669	FEVSUX	161.653	2.669	FEVSUX01	162.844	3.296
FEWCUH0	158.336	3.47	FEWCUH0	177.903	2.779	FEWCUH0	154.712	3.35	FEWCUH0	154.682	3.095	FEWCUH0	166.95	3.128
FEWDES	156.427	2.824	FEWDES	154.18	3.28	FEWCUH0	153.369	2.859	FEWCUH0	159.141	2.99	FEWCUH0	174.399	3.267
FEWDES	164.516	3.016	FEWDES	158.22	3.24	FEWDES	154.927	3.099	FEWDES	157.318	3.363	FEWDES	163.508	2.947
FEWDIW0	161.016	3.493	FEWDIW0	169.486	3.037	FEWDES	168.623	3.259	FEWDES	154.633	2.858	FEWDES	173.594	3.081
FEWHEY	173.662	3.07	FEWHEY	175.141	2.767	FEWDIW0	161.91	3.111	FEWHEY	169.209	3.104	FEWHEY	170.768	2.843
FEWPUU	156.446	2.75	FEWPUU	153.359	2.748	FEWHOI	155.541	3.267	FEWHUO	160.465	2.844	FEWPAC	171.679	2.79
FEWPUU	157.793	3.199	FEWQIK	157.942	3.02	FEWPUU	150.907	3.297	FEWPUU	173.534	2.761	FEWPUU	160.439	2.75
FEXNIG	162.951	3.067	FEXNIG	158.175	3.028	FEWYOX	176.967	2.901	FEWYOX	160.519	2.85	FEWYOX	155.222	2.85
FEXNIG	159.936	3.07	FEXNIG	151.078	3.28	FEXNIG	160.688	3.493	FEXNIG	153.761	3.196	FEXNIG	171.21	3.48
FEXZIV	154.363	3.357	FEXZOZ	152.373	2.905	FEXNIG	164.591	3.16	FEXYAJ	172.027	3.24	FEXYAJ	167.189	3.488
FEXZOZ	155.91	3.338	FEXZOZ	153.602	2.926	FEXZOZ	164.356	3.2	FEXZOZ	154.074	2.804	FEXZOZ	163.437	2.897
FEXZOZ	157.148	3.106	FEXZOZ	153.276	3.114	FEXZOZ	176.068	2.953	FEXZOZ	162.579	2.909	FEXZOZ	165.576	3.424
FEXZOZ	150.44	2.97	FEXZOZ	172.139	3.088	FEXZOZ	150.125	3.065	FEXZOZ	164.119	3.307	FEXZOZ	163.258	2.926
FEYFEV	150.946	3.182	FEYFIC	150.128	3.277	FEXZUH	154.45	3.127	FEYFEV	170.234	3.032	FEYFEV	164.474	3.323
FEYGAV	179.449	2.94	FEYHEX	171.522	3.055	FEYFIC	164.544	3.09	FEYFIC	152.515	2.821	FEYGAV	150.001	2.851
FEYHIB	153.337	3.33	FEYHIB	155.747	3.447	FEYHIB	164.697	3.44	FEYHIB	150.467	3.439	FEYHIB	151.39	3.284
FEYHIB	166.278	3.307	FEYHIB	158.721	2.998	FEYHIB	154.361	2.854	FEYHIB	160.971	3.023	FEYHIB	164.017	3.261
FEYHIB	161.289	2.85	FEYHIB	153.716	2.959	FEYHIB	157.497	2.85	FEYHIB	156.729	3.162	FEYHIB	176.717	2.935
FEYHIC	163.375	2.941	FEYHIC	164.593	3.442	FEYHIC	155.836	3.338	FEYHIC	172.398	2.908	FEYHIC	159.724	3.374
FEYHIE	158.927	3.346	FEYHIE	158.741	2.942	FEYHIC	158.648	3.144	FEYHIE	157.828	3.088	FEYHIE	158.463	3.258
FEYHOI	157.711	3.131	FEYHOI	150.243	2.87	FEYHIE	150.032	3.219	FEYHIE	160.7	3.369	FEYHIE	163.786	2.671
FEYHOI	150.695	3.397	FEYHUO	157.634	3.289	FEYHOI	150.504	2.859	FEYHOI	152.816	3.257	FEYHOI	163.951	3.105
FEYHUO	166.883	3.075	FEYHUO	177.288	3.245	FEYHUO	170.511	2.928	FEYHUO	160.196	3.303	FEYHUO	169.574	3.44
FEYKON	157.09	2.751	FEYSOU	175.601	2.94	FEYHUO	163.715	3.284	FEYHUO	157.251	3.096	FEYJIG	157.053	2.599
FEYTUC	152.643	3.182	FEYZIS	159.305	3.244	FEYTAH	168.99	3.421	FEYTEK	157.601	3.443	FEYTIP	178.348	3.102
FEZDIA	160.032	3.229	FEZDIA	156.175	3.085	FEYZIS	155.469	2.879	FEZCIY	173.183	3.438	FEZCIY	174.548	3.334
FEZHEX	163.321	3.125	FEZHOH	169.453	3.006	FEZDIA	171.996	3.212	FEZDIA	153.728	3.028	FEZDIA	152.696	3.099
FGEBIA	162.017	2.716	FGEBIA	166.103	2.716	FEZJUS	169.972	3.227	FEZZIW	153.171	3.054	FGEBIA	159.808	2.792
FGEBIA	162.302	3.069	FGEBIA	153.256	2.751	FGEBIA	157.019	3.219	FGEBIA	162.277	2.792	FGEBIA	164.911	2.771
FGEBIA	158.658	2.898	FGEBIA	159.153	3.219	FGEBIA	166.25	2.903	FGEBIA	155.304	2.771	FGEBIA	156.956	2.869
FIBJUW	153.312	3.019	FIBJUW	152.575	3.372	FGEBIA	158.186	3.413	FIBJUW	152.838	2.988	FIBJUW	164.102	2.909
FIBSIT	155.577	2.675	FIBWAR	157.823	3.439	FIBJUW	169.779	2.927	FIBKOS	156.615	2.652	FIBLEK	160.369	3.129
FICDUS	167.952	3.01	FICDUS	159.991	3.32	FICBUR	156.974	3.156	FICDUS	168.987	3.01	FICDUS	169.069	3.201
FICGAB	159.204	2.771	FICGAB	162.271	2.773	FICGAB	159.663	3.121	FICGAB	154.049	3.418	FICGAB	169.061	3.25
FICGAB	155.569	2.952	FICNIR	171.651	3.366	FICGAB	155.775	2.791	FICGAB	156.572	2.878	FICGAB	169.935	3.053
FICTOD	150.873	3.422	FICTOD	167.223	3.422	FICNOX	171.661	3.356	FICROA	154.997	3.409	FICROA	169.142	3.43
FIDKUB	164.827	3.177	FIDLUD	153.852	2.773	FICZOK	154.073	2.839	FICZOK	163.209	3.21	FIDGEE	168.948	3.394
FIDNIT	159.069	3.005	FIDNOZ	167.599	3.01	FIDLUD	165.307	2.773	FIDLUD	167.85	3.447	FIDNIT	173.325	2.971
FIDZUQ	150.919	3.168	FIDZUQ	164.351	3.16	FIDNOZ	166.413	3.125	FIDZUQ	165.221	3.412	FIDZUQ	150.951	3.412
FIFDAZ	165.858	3.065	FIFDAZ	153.05	3.361	FIFDAZ	164.451	3.39	FIFDAZ	155.347	3.157	FIFDAZ	159.115	3.394
FIFKAH	157.709	3.463	FIFQEU	161.475	3.499	FIFDAZ	151.909	2.901	FIFDAZ	173.798	3.006	FIFDAZ	161.198	3.065
FIFQOE	165.467	3.34	FIGJEK	172.94	2.917	FIFQEU	151.091	3.253	FIFQOE	154.143	2.818	FIFQOE	164	3.143
FIGJEK	155.17	2.839	FIGKAH	153.733	3.295	FIGJEK	159.286	2.91	FIGJEK	161.256	2.839	FIGJEK	176.154	3.107
FIGZEC	157.088	3.058	FIGZEC	150.419	3.222	FIGWOH	161.828	3.133	FIGWOH	153.628	2.966	FIGYAV10	160.054	3.009
FIGZEC	153.3	2.884	FIGZEC	154.744	2.937	FIGZEC	154.51	2.97	FIGZEC	174.819	2.978	FIGZEC	169.899	3.054
FIGZEC	155.934	3.197	FIGZOL	154.64	3.073	FIGZEC	151.097	3.382	FIGZEC	150.279	3.198	FIGZEC	158.49	3.019
FIHGEI	157.333	3.06	FIHGEI	159.048	3.216	FIHGEI	159.83	3.133	FIHGEI	159.951	3.206	FIHGEI	159.808	3.057
FIHHIN	169.124	3.295	FIHJOZ	172.577	3.057	FIHGEI	163.838	3.206	FIHGEI	154.545	3.239	FIHGEI	172.302	3.487
FIHJOZ	165.705	2.926	FIHTEZ	168.909	3.303	FIHJOZ	170.505	3.096	FIHJOZ	161.187	3.025	FIHJOZ	159.323	2.859
FIJJAL	162.055	3.493	FIJKES	150.327	3.068	FIJGUE	160.515	3.169	FIJGUE	152.868	3.256	FIJJAL	164.117	3.069
FIJKES	152.447	2.904	FIJKES	153.442	2.801	FIJKES	152.121	3.091	FIJKES	168.649	2.869	FIJKES	156.183	2.883
FIKJIW	150.622	2.741	FIKSEY	154.259	3.153	FIJMIY	158.491	3.297	FIJMIY	175.305	3.15	FIJQUL	162.162	3.26
FIKWAB	177.117	2.783	FIKWAB	159.091	3.405	FIKSEY	154.17	3.076	FIKSEY	154.369	2.93	FIKWAB	157.247	3.291
FIKXIH	154.029	3.01	FIKXON	177.314	2.738	FIKWAB	162.355	3.043	FIKXAZ	150.489	3.23	FIKXIH	162.084	2.91
FILHUE	156.723	3.301	FILMEU	160.743	3.132	FIKXON	168.841	3.041	FIKXOP	161.515	2.943	FILHUE	150.756	3.487
FILTUR	169.46	2.856	FILTUR	153.433	3.25	FILMEU	157.185	3.064	FILTUR	166.096	3.314	FILTUR	166.252	3.377
FILTUR	166.449	2.854	FILTUR	168.55	3.061	FILTUR	165.773	2.826	FILTUR	157.227	3.47	FILTUR	151.633	3.375
FIMTON	156.968	3.228	FIMTON	162.786	3.107	FILZUX	155.703	3.012	FIMBIO	150.827	2.881	FIMNOH	151.628	3.285
FINDOV	158.475	3.204	FINDOV	153.047	3.018	FIMYEI	168.163	3.144	FIMYEI	163.636	2.714	FIMYEI	157.355	3.169
FINGER	167.16	3.427	FINPEZ	153.05	3.06	FINFIS	168.465	2.593	FINFIS	173.958	2.781	FINFIS	162.627	2.593
FINQAX	160.072	2.871	FINQAX	150.48	3.089	FINPEZ	161.361	3.158	FINPID	152.842	3.318	FINPID	164.035	3.497
FINQEB	175.182	3.386	FINQEB	167.692	2.692	FINQEB	158.568	3.085	FINQEB	155.942	3.085	FINQEB	156.274	2.764
FINQEB	155.424	3.175	FINQEB	175.115	3.31	FINQEB	150.169	3.365	FINQEB	150.251	2.93	FINQEB	154.489	3.23
FINQEB	158.625	3.222	FINQEB	162.932	2.882	FINQEB	169.937	2.757	FINQEB	158.08	3.057	FINQEB	155.076	3.148
FINQEB	171.945	3.424	FINQEB	165.915	3.33	FINQEB	166.471	3.135	FINQEB	155.004	3.182	FINQEB	154.524	3.288
FINQEB	170.332	3.087	FINQEB	151.736	2.89	FINQEB	151.812	3.19	FINQEB	153.304	3.208	FINQEB	155.53	2.955
FINQIF	152.474	3.039	FINQIF	171.275	3.491	FINQEB	153.427	3.111	FINQIF	153.338	3.035	FINQIF	167.722	2.709
FINQIF	164.094	3.437	FINQIF	150.345	2.827	FINQIF	159.619	3.491	FINQIF	175.009	2.698	FINQIF	163.445	3.365
FINRAY	159.116	2.751	FINRAY	152.419	2.824	FINQIF	167.711	2.698	FINQIF	163.296	3.058	FINRAY	160.723	3.351
FINRAY	163.27	3.388	FINRAY	164.552	2.89	FINRAY	161.553	3.092	FINRAY	166.518	3.175	FINRAY	156.546	2.953
FINRAY	151.654	3.351	FINRAY	156.434	3.307	FINRAY	153.028	2.833	FINRAY	158.707	2.926	FINRAY	158.585	3.49
FINRAY	165.369	3.175	FINRAY	159.149	3.388	FINRAY	164.814	2.833	FINRAY	176.095	2.876	FINRAY	158.628	3.408
FINROL	163.143	3.009	FINSEC	152.727	3.371	FINRAY	165.223	2.89	FINROL	153.276	3.379	FINROL	172.016	3.316
FIPHUI	158.469	3.086	FIPHUI	167.541	3.086	FINSEC	171.103	3.338	FINSEC	162.384	3.002	FINWOR	154.93	3.446
FIPZIP	156.388	3.477	FIPZIP	178.288	3.481	FIPHUI	152.519	3.077	FIPZEL	170.997	3.262	FIPZIP	162.199	3.456
FIPZOV	157.061	2.61	FIPZOV	154.79	3.421	FIPZOV	155.039	3.352	FIPZOV	155.201	2.803	FIPZOV	156.744	2.61

FIQTII	171.799	3.341	FIQTII	164.217	2.996	FIQTAA	155.467	2.894	FIQTAA	156.128	3.201	FIQTAA	167.364	2.888
FIRCEP	165.51	3.063	FIRCIT	177.066	3.494	FIRCEP	174.715	3.107	FIRCEP	161.763	3.483	FIRCEP	165.403	2.921
FIRCIT	164.346	3.354	FIRCIT	153.643	2.979	FIRCIT	169.82	2.834	FIRCIT	161.825	2.88	FIRCIT	170.816	3.104
FIRCIT	174.813	2.911	FIRCIT	156.245	2.956	FIRCIT	162.243	3.449	FIRCIT	152.125	3.216	FIRCIT	165.27	2.976
FIRZEM	163.422	3.414	FIRZEM	175.597	3.208	FIRLAU	150.865	2.854	FIRRON	155.111	3.426	FIRRON	162.668	2.834
FIRZEM	159.077	3.001	FIRZEM	165.729	3.098	FIRZEM	155.669	2.78	FIRZEM	159.345	2.982	FIRZEM	151.889	3.206
FIRZEM	159.672	3.375	FIRZEM	163.293	3.321	FIRZEM	166.017	3.479	FIRZEM	175.857	3.467	FIRZEM	171.845	2.708
FIRZEM	150.735	3.271	FIRZEM	156.018	2.703	FIRZEM	175.411	3.007	FIRZEM	176.61	3.32	FIRZEM	150.887	3.019
FIRZOW	155.801	3.383	FIRZOW	169.018	3.395	FIRZIQ	176.72	2.784	FIRZIQ	158.562	3.113	FIRZOW	156.744	3.087
FISFAO	151.611	3.375	FISFAO	150.224	3.159	FIRZOW	150.857	2.898	FIRZOW	154.089	3.075	FISFAO	150.114	3.427
FISJOJ	153.877	3.387	FISJOJ	175.664	3.027	FISFAO	166.259	3.472	FISFAO	154.543	3.109	FISFAO	157.121	3.109
FISQIH	168.95	3.14	FISQIH	161.342	2.621	FISNIG	158.591	2.789	FISNIG	163.194	3.27	FISNIG	170.192	2.789
FISWIP	160.444	3.162	FISWIP	153.484	2.858	FISTUY	154.714	3.174	FISTUY	163.563	3.174	FISWIP	157.725	2.811
FITHOG	154.633	3.28	FITHOG	152.516	3.163	FITGAR	155.154	2.932	FITGAR	161.271	3.191	FITHOG	151.851	3.481
FITHOG	154.513	3.227	FITHOG	161.946	3.177	FITHOG	168.86	3.059	FITHOG	164.13	2.939	FITHOG	172.945	3.174
FITJIB	151.442	3.31	FITJIB	156.14	3.206	FITHOG	152.296	2.939	FITHOG	172.985	3.437	FITHOG	156.474	3.28
FITXIR	156.334	3.292	FITYOX	171.427	2.963	FITJIB	167.35	3.133	FITVUD	170.548	2.991	FITVUZ	157.001	3.459
FITYOY	172.77	3.056	FITYOY	168.395	3	FITYOX	154.994	2.902	FITYOX	152.539	3.136	FITYOX	170.91	2.902
FIVCIZ01	162.487	3.1	FIVCIZ01	163.625	3.232	FITYUD	155.31	3.436	FITYUD	172.905	3.268	FIVBOF	155.847	3.44
FIVFUP	152.393	3.145	FIVFUP	160.132	3.351	FIVCIZ01	150.917	3.048	FIVCIZ01	170.425	3.306	FIVFUP	152.758	3.203
FIVNUW	150.852	3.405	FIVPAE	152.477	3.177	FIVFUP	151.46	3.189	FIVGEZ	173.093	3.003	FIVGID	151.528	3.232
FIVREK	157.035	3.373	FIVXIU	168.78	2.952	FIVREK	174.375	3.135	FIVREK	153.365	2.892	FIVREK	155.434	2.759
FIWJIH	154.268	3.367	FIWJIH	165.363	3.243	FIVXIU	176.678	3.264	FIWFAW	169.961	3.298	FIWJIH	160.734	3.195
FIWRAG	152.155	3.202	FIWTUC	173.195	2.499	FIWKIJ	161.034	3.268	FIWNEF	157.246	3.406	FIWQUZ	162.617	3.188
FIWZIX	156.084	2.674	FIWZIX	156.665	3.084	FIWZIX	150.896	3.221	FIWZIX	174.684	3.285	FIWZIX	151.179	3.388
FIXVUF	152.172	3.164	FIXXET	153.399	3.224	FIXGID	154.829	3.33	FIXGID	166.065	2.659	FIXGID	163.781	3.108
FIYDUN	161.782	3.382	FIYDUN01	160.537	3.308	FIXXET	155.327	2.995	FIXYIY	164.478	3.495	FIYDUN	160.404	3.266
FIYNIN	167.596	3.095	FIYNIN01	168.082	3.045	FIYDUN01	161.636	3.429	FIYNAE	157.958	2.895	FIYNAE	160.735	3.488
FIYQUA	163.237	2.833	FIYQUA	152.001	2.777	FIYNIN02	167.863	3.056	FIYNIN03	167.674	3.077	FIYPIO	151.289	2.975
FIYYAN	172.703	3.488	FIYZUL	160.935	3.069	FIYQUA	156.229	2.665	FIYYAN	172.524	2.867	FIYYAN	150.954	3.487
FIZMOR	153.494	3.01	FIZMOR	155.879	3.19	FIYZUL01	161.837	3.077	FIYZUL02	162.721	3.086	FIYZUL03	163.412	3.101
FIZPOX	152.347	3.226	FIZPOX01	166.982	2.935	FIZMOR	153.846	3.224	FIZNOT	150.806	3.499	FIZPOX	166.874	2.935
FIZWAN	155.693	3.151	FIZZIY	157.871	3.058	FIZPOX01	152.225	3.227	FIZSIT	150.529	3.064	FIZTAN	154.748	3.388
FLGEHG	156.838	3.413	FLGEHG	160.855	3.355	FIZZIY	154.638	3.16	FIZZIY	155.748	3.461	FLGEHG	155.504	3.19
FLGEHG	166.458	3.457	FLGEHG	153.817	3.156	FLGEHG	157.778	3.393	FLGEHG	165.99	3.302	FLGEHG	150.317	3.388
FLMECE	157.337	2.754	FLMECE	153.787	2.968	FLGEHG	157.543	3.388	FLGEHG	153.963	2.856	FLMECE	153.606	2.754
FLPCTD	159.539	3.204	FLPCTD	166.314	3.097	FLMECE	159.168	3.222	FLMECE	161.457	3.222	FLMECE	150.724	3.433
FLTHAN	166.453	3.064	FLTHAN	165.15	2.876	FLPCTD	152.692	3.079	FLTHAN	163.667	3.17	FLTHAN	151.457	3.252
FMAZPH	157.682	3.128	FMAZPH	164.592	2.963	FMALON2	174.267	3.089	FMAPNI	164.233	3.484	FMAZPH	161.743	3.387
FMEAMO1	165.001	3.468	FMEAMO1	154.056	3.079	FMBTMO	161.21	3.004	FMEAMO1	150.206	3.349	FMEAMO1	158.342	3.153
FMPENR	159.423	2.973	FMPNFE	152.302	3.287	FMEAMO1	154.587	3.499	FMHGAZ	168.549	3.379	FMHGAZ	160.978	3.252
FMPNFE	158.36	3.372	FMPNFE	153.049	3.318	FMPNFE	170.879	3.343	FMPNFE	166.143	3.149	FMPNFE	153.659	2.964
FMTCBW1	154.857	3.262	FMTCBW1	155.037	3.262	FMPNFE	159.973	2.776	FMTCBW	154.82	3.261	FMTCBW	155.015	3.261
FMTPHG	162.706	3.339	FMTPHG	154.056	3.445	FMTPHG	171.056	3.148	FMTPHG	159.211	3.125	FMTPHG	168.008	3.178
FNETAM0	151.562	2.766	FNETAM0	158.219	2.778	FMTPHG	166.228	3.148	FMTPHG	172.09	2.969	FMVPPT	156.793	3.366
FOBFOV	164.864	3.011	FOBFOV	151.532	2.855	FOBFIP	169.866	3.457	FOBFOV	166.3	3.011	FOBFOV	175.5	3.035
FOBLIS	153.478	3.455	FOBLIT	167.928	3.069	FOBFOV	151.323	2.907	FOBKEO	162.757	3.286	FOBLIS	158.599	3.026
FOBNOB	153.444	3.284	FOBNOB	159.217	3.11	FOBLIT	163.195	3.086	FOBLIT	163.878	3.15	FOBMIU01	163.124	3.266
FOBNUJ	156.759	2.871	FOBPAP	150.15	3.398	FOBNOB	157.717	3.417	FOBNUJ	153.227	2.791	FOBNUJ	154.285	3.301
FOBPIZ	165.715	3.385	FOBPIZ	162.443	3.472	FOBPAR	165.819	3.437	FOBPAR	173.711	3.287	FOBPAR	151.413	2.747
FOBREV	150.435	3.294	FOBRIZ	164.252	3.003	FOBPIZ	153.429	3.136	FOBPIZ	160.762	2.93	FOBPOC	166.201	2.842
FOBYUT	157.5	2.995	FOBYUT	159.544	3.08	FOBYIH	162.402	3.392	FOBYIH	160.831	2.759	FOBYIH	158.488	2.905
FOBZEE	163.013	3.236	FOBZEE	173.216	3.271	FOBZAA	153.913	2.984	FOBZEE	154.314	3.449	FOBZEE	159.431	2.772
FOCNAM	161.068	3.45	FOCNAM	164.825	2.908	FOCFAG	162.945	2.936	FOCNAM	162.385	2.953	FOCNAM	162.383	3.315
FOCNAM	161.074	2.924	FOCNAM	160.961	3.231	FOCNAM	158.834	2.904	FOCNAM	168.588	3.151	FOCNAM	173.184	3.417
FOCQUJ	158.91	2.912	FOCQUJ	152.811	2.924	FOCNAM	167.337	2.904	FOCNAM	178.256	2.924	FOCNAM	157.634	3.398
FODDOU	166.848	3.377	FODDOU	151.987	3.305	FOCQUJ	170.002	2.912	FOCQUN	151.258	3.202	FODCEK	150.46	3.345
FOFDUZ	151.622	3.426	FOFDUZ	152.954	3.141	FOFDEK	165.328	3.067	FOFDOV	162.845	3.058	FOFDUZ	153.619	2.81
FOFDUZ1	151.622	3.426	FOFDUZ1	159.001	3.426	FOFDUZ	159.001	3.426	FOFDUZ	160.06	3.294	FOFDUZ	163.697	3.495
FOFPAU	174.196	2.934	FOFTIE	160.537	3.048	FOFDUZ1	163.697	3.495	FOFDUZ1	160.06	3.294	FOFDUZ1	153.619	2.81
FOFXUW	163.395	3.484	FOFYAD	162.685	3.482	FOFTIE	159.256	3.253	FOFTIE	156.743	3.113	FOFTIE	156.004	2.928
FOFZOS	162.55	3.411	FOFZUV	173.381	3.007	FOFYIL	150.02	3.057	FOFYOR	155.697	2.969	FOFZOS	162.621	3.282
FOGBAE1	161.618	3.345	FOGBAH	163.385	3.484	FOFZUY	160.329	3.383	FOFZUY	161.863	3.309	FOGBAE	161.618	3.345
FOGFAL	158.078	2.593	FOGFEP	151.105	3.086	FOGDOX	161.339	2.791	FOGDOX	157.158	3.001	FOGDUD	173.089	3.231
FOGGOX	167.735	3.114	FOGGOX	154.029	3.154	FOGFEP	152.262	2.942	FOGGOX	160.201	3.285	FOGGOX	163.894	2.942
FOGGUD	170.292	3.444	FOGGUD	152.22	3.427	FOGGOX	155.953	2.976	FOGGOX	153.571	2.97	FOGGOX	150.352	3.154
FOGGUD	157.753	2.985	FOGGUD	151.844	3.308	FOGGUD	160.557	3.14	FOGGUD	163.969	3.116	FOGGUD	165.961	3.421
FOGLIZ	167.16	3.161	FOGLIZ	152.543	3.323	FOGGUD	167.604	3.294	FOGGUD	152.884	3.487	FOGLIZ	155.721	3.41
FOGLIZ	167.518	3.361	FOGLIZ	153.862	2.979	FOGLIZ	151.712	3.064	FOGLIZ	159.11	3.231	FOGLIZ	163.475	2.893
FOGLIZ	171.331	2.874	FOGLIZ01	158.404	3.194	FOGLIZ	162.345	2.888	FOGLIZ	162.789	2.684	FOGLIZ	172.124	2.978
FOGLIZ01	162.52	2.675	FOGLIZ01	162.883	2.856	FOGLIZ01	170.583	2.843	FOGLIZ01	171.984	2.893	FOGLIZ01	168.135	3.395
FOGLIZ01	167.852	3.085	FOGLIZ01	164.633	2.863	FOGLIZ01	150.896	3.297	FOGLIZ01	152.725	3.011	FOGLIZ01	156.46	3.413
FOGLOF	150.059	3.283	FOGLOF	165.125	3.439	FOGLIZ01	154.356	2.937	FOGLOF	154.327	2.965	FOGLOF	162.201	3.169
FOGLOF	165.12	2.986	FOGLOF	161.551	3.03	FOGLOF	172.137	3.439	FOGLOF	166.33	3.175	FOGLOF	164.382	2.968
FOGLOF	158.808	3.417	FOGLOF	166.646	3.43	FOGLOF	164.624	3.154	FOGLOF	165.78	3.15	FOGLOF	152.773	2.974
FOGLUL	158.793	3.128	FOGLUL	164.97	2.66	FOGLOG	153.511	3.144	FOGLUL	152.709	3.016	FOGLUL	173.276	2.852
FOGLUL	151.43	2.916	FOGMEW	170.463	3.095	FOGLUL	159.247	2.796	FOGLUL	165.662	3.327	FOGLUL	160.422	3.043
FOGNEV	153.747	3.321	FOGNEV	157.721	3.479	FOGMEW	169.635	3.126	FOGMEW	165.783	2.979	FOGMEW	167.967	3.269
FOGQAW	166.049	3.238	FOGQOK	154.492	2.887	FOGNIB	155.423	2.965	FOGNIB	160.465	3.43	FOGQAW	169.25	2.705

FOGVIH	167.304	3.398	FOGVIH	158.747	3.202	FOGQOK	155.325	2.841	FOGQOK	157.33	3.201	FOGQOK	151.442	3.205
FOHCOY	152.493	3.188	FOHFAN	151.189	3.006	FOGVIH	156.896	2.83	FOHCIR	168.919	3.245	FOHCOX	171.405	3.165
FOHFAN	157.141	3.002	FOHFAN	158.076	3.002	FOHFAN	155.023	2.651	FOHFAN	173.277	2.91	FOHFAN	172.171	2.936
FOHFER	158.538	2.844	FOHFER	173.592	2.898	FOHFER	151.552	3.284	FOHFER	162.016	2.844	FOHFER	154.663	3.374
FOHMEU	175.596	3.048	FOHMEU	168.94	3.01	FOHFER	163.636	2.794	FOHFUH	152.952	2.917	FOHFUH	172.102	3.217
FOHQEC	155.414	3.065	FOHQEC	177.089	2.992	FOHPOJ	152.603	3.056	FOHPOJ	152.877	3.177	FOHQEA	163.46	2.633
FOHQEC	150.738	2.833	FOHQEC	158.49	2.824	FOHQEC	153.336	3.065	FOHQEC	152.312	3.296	FOHQEC	150.738	3.126
FOHROK	151.228	3.18	FOHSIE	171.852	3.358	FOHRAX	162.119	3.451	FOHRAX	159.96	3.46	FOHROK	166.554	2.798
FOHZEL	164.76	3.01	FOJBAK	150.304	3.255	FOHSOK	155.104	3.014	FOHWEI	158.244	3.317	FOHXUV	164.253	3.409
FOJBAK	157.701	3.096	FOJBOY	156.744	3.411	FOJBAK	156.287	3.486	FOJBAK	157.106	3.015	FOJBAK	174.659	3.152
FOJCEP	156.901	2.885	FOJCEP	151.98	2.979	FOJBOY	165.091	3.44	FOJBOY	152.377	3.171	FOJCEP	157.876	3.063
FOJCEP	157.193	3.224	FOJCEP	165.143	2.772	FOJCEP	160.368	2.745	FOJCEP	165.19	3.01	FOJCEP	173.971	2.855
FOJCIT	154.238	3.384	FOJCIT	171.624	2.872	FOJCIT	156.579	2.855	FOJCIT	175.203	2.762	FOJCIT	162.374	2.88
FOJCOZ	158.242	3.23	FOJCOZ	173.326	3.05	FOJCIT	173.276	3.175	FOJCIT	164.139	3.332	FOJCOZ	150.295	2.848
FOJCOZ	150.239	2.778	FOJCOZ	151.997	2.96	FOJCOZ	177.501	2.772	FOJCOZ	174.469	3.251	FOJCOZ	152.414	3.345
FOJCUF	172.178	2.73	FOJCUF	174.761	3.114	FOJCOZ	154.187	3.18	FOJCUF	151.683	2.809	FOJCUF	179.551	3.072
FOJCUF	152.794	3.149	FOJDIU	170.601	3.049	FOJCUF	154.582	3.363	FOJCUF	154.694	2.818	FOJCUF	152.293	2.842
FOJFIU	156.935	3.228	FOJJAR	161.619	3.065	FOJDIU	157.847	3.062	FOJDIU	169.541	2.984	FOJDIU	161.067	2.836
FOJSIK	155.41	3.207	FOKGOC	155.207	3.249	FOJQOM	167.66	3.288	FOJQOM	161.178	3.195	FOJSAY	151.34	3.052
FOKHAP	171.706	3.102	FOKHIX	160.652	2.99	FOKGOC	165.785	3.148	FOKHAP	168.724	2.745	FOKHAP	162.189	2.951
FOKSUV	164.717	2.997	FOKZUD	167.622	2.937	FOKHIX	158.23	2.989	FOKHIX	160.323	2.872	FOKHIX	162.535	3.009
FOLDES	168.54	3.455	FOLDIW	168.856	3.46	FOKZUD	156.515	2.946	FOLCUG	153.488	3.023	FOLCUG	151.767	3.266
FOMBER	161.236	3.495	FOMDIV	169.676	2.976	FOLMOM	173.244	2.936	FOLMOM	163.473	3.467	FOLQUW	150.878	3.452
FOMNEC	166.832	2.941	FOMXIQ	152.679	2.875	FOMMUQ	167.914	3.453	FOMMUQ	150.499	3.381	FOMNEC	158.31	3.137
FOMYAJ	150.276	3.066	FONMEA	151.7	3.06	FOMXIQ	156.865	3.352	FOMXOW	153.572	3.182	FOMYAJ	150.561	3.462
FONRAE	150.342	3.219	FONRAE	160.541	3.216	FONMEA	155.856	3.397	FONMEA	151.722	3.222	FONRAE	150.967	3.192
FONRIM	154.247	3.028	FONRIM	153.482	3.047	FONRAE	154.878	3.214	FONRAE	154.44	3.216	FONRIM	170.888	2.853
FONROS	166.092	2.822	FONROS	150.903	3.079	FONRIM	161.148	2.883	FONROS	168.582	2.99	FONROS	155.648	2.96
FONRUY	171.324	3.147	FONRUY	173.083	2.972	FONROT	158.253	3.3	FONRUY	163.57	3.486	FONRUY	165.947	3.224
FONSAF	166.511	3.056	FONSAF	152.932	2.766	FONSAF	164.651	3.101	FONSAF	162.503	3.221	FONSAF	168.406	3.343
FONSOT	152.532	2.853	FONSOT	152.468	2.926	FONSEJ	172.797	3.097	FONSOT	170.359	2.926	FONSOT	150.037	2.867
FONTUB	165.622	2.889	FONVAI	172.945	3.036	FONTOU	161.743	3.128	FONTOU	157.94	3.123	FONTUB	160.238	2.966
FONVAI	164.626	3.46	FONVAI	157.56	3.036	FONVAI	175.223	3.241	FONVAI	151.124	3.392	FONVAI	158.179	3.121
FONYEP	166.73	3.262	FONYEP	150.316	3.016	FONVAI	164.734	3.08	FONVAI	152.745	3.103	FONYEP	175.303	3.01
FOPDEX	159.611	3.371	FOPJIE	157.397	3.3	FONYEP	154.244	2.707	FOPBOC	152.443	3.489	FOPBUJ	158.784	3.311
FOPMUW	158.766	3.429	FOPNEF	155.496	3.339	FOPJIE	170.041	3.122	FOPLON	174.1	2.722	FOPMUW	157.039	2.642
FOPNEF	159.862	3.272	FOPNEF	155.129	3.477	FOPNEF	156.475	2.918	FOPNEF	161.634	3.04	FOPNEF	153.371	3.104
FOPWIT	153.544	3.082	FOPWIT	159.956	3.191	FOPSOT	160.91	3.435	FOPVUB	155.956	3.341	FOPWIT	162.918	3.3
FOQJIH	156.866	3.09	FOQJIH	160.794	3.305	FOPXAM	154.555	3.459	FOPXAM	160.526	3.012	FOPXAM	160.372	2.895
FORHOJ	151.684	3.129	FORHOJ	152.488	3.49	FOQJIH	156.566	3.423	FORCOE	170.74	2.846	FORHOJ	154.255	3.176
FORLAC	157.489	3.485	FORLAC	159.889	3.224	FORHOJ	153.295	2.935	FORJAX	166.187	2.845	FORLAC	163.618	3.219
FORQUA	158.883	3.451	FORREL	151.557	2.788	FORLAC	170.331	3.425	FORLAC	157.984	3.066	FORMAB	170.163	3.008
FORSUD	157.519	3.024	FORSUD	168.803	3.023	FORSUD	172.814	3.326	FORSUD	167.269	3.353	FORSUD	167.42	2.964
FORSUD	164.435	2.94	FORSUD	150.028	3.166	FORSUD	152.826	3.002	FORSUD	165.88	3.44	FORSUD	154.312	3.415
FORSUD	157.607	3.305	FORSUD	159.448	3.326	FORSUD	153.317	3.313	FORSUD	151.017	3.042	FORSUD	171.419	2.962
FOSQOX	163.229	3.008	FOSZOE	158.797	3.408	FOSFEN1(	156.154	2.942	FOSNAD	175.455	3.442	FOSNAD	173.582	3.322
FOTMOQ	157.203	3.093	FOTMOQ	160.504	3.327	FOSZOE	167.54	2.811	FOTMOQ	151.962	3.491	FOTMOQ	159.026	3.316
FOTNEH	155.36	3.327	FOTPEJ	164.137	3.33	FOTMOQ	161.448	3.04	FOTMOQ	158.569	3.491	FOTNEH	169.985	3.012
FOVGEG	176.639	3.329	FOVHEH	163.461	2.951	FOTREL	159.622	3.441	FOVDUT	167.331	2.795	FOVFUV	150.494	3.212
FOVLOS	155.866	3.212	FOVMEM	153.604	3.278	FOVHEH	159.698	2.869	FOVHOR	158.136	3.022	FOVKEJ	150.985	3.257
FOWPEN	152.703	3.329	FOWRAK	154.835	2.924	FOVWOF	176.037	3.01	FOWCAX	164.72	2.686	FOWHEH	154.842	3.14
FOXFIL	167.776	3.093	FOXGAD	155.336	3.104	FOWYIZ	150.157	3.496	FOWYIZ	156.977	3.245	FOWYIZ	162.484	3.496
FOXMOW	152.032	2.976	FOXMOW	152.76	2.996	FOXKEM	173.795	3.05	FOXKEM	157.142	2.857	FOXMOW	171.193	2.976
FOXMUC	150.78	3.336	FOXMUE	152.905	3.163	FOXMUC	155.062	3.261	FOXMUC	157.69	2.771	FOXMUC	168.172	2.638
FOXROB	165.288	3.492	FOXYEX	151.433	2.971	FOXNAL	167.934	3.321	FOXNUE	150.128	3.078	FOXPAN	173.687	3.005
FOYCED	156.104	3.241	FOYCED	164.938	3.448	FOXZEY	161.891	3.361	FOXZEY	156.566	3.267	FOYCED	172.24	3.037
FOYJIQ	176.284	3.247	FOYJIQ	162.517	3.071	FOYFAB	162.536	3.371	FOYJAI	168.971	3.334	FOYJAI	166.131	2.807
FOYPUG	158.337	3.172	FOYPUG	167.803	2.933	FOYJOT	151.218	3.221	FOYJOT	170.171	3.175	FOYPUG	153.574	3.482
FOYQIV	169.544	2.798	FOYQIV	154.095	2.993	FOYQIV	158.086	3.004	FOYQIV	157.544	2.843	FOYQIV	159.851	2.883
FOYRAO	153.457	3.144	FOZHAH	176.605	2.596	FOYQIV	150.272	2.904	FOYRAO	160.844	3.194	FOYRAO	167.881	3.273
FOZKEL	161.426	3.401	FOZKOV	169.029	3.488	FOZHAH	154.998	2.596	FOZHEL	152.057	3.467	FOZKEL	153.441	3.498
FOZMIS	153.772	3.371	FOZMIS	171.123	3.066	FOZMAM	154.64	3.187	FOZMAM	157.184	3.088	FOZMIS	152.198	3.488
FOZMOZ0	167.598	3.475	FOZMOZ0	166.153	3.479	FOZMIS	169.281	3.308	FOZMOZ0	173.884	3	FOZMOZ0	166.83	3.047
FOZMOZ0	152.354	3.259	FOZMOZ0	151.301	3.379	FOZMOZ0	155.891	3.105	FOZMOZ0	165.555	3.281	FOZMOZ0	170.805	2.776
FOZMOZ0	164.84	3.179	FOZMOZ0	154.802	2.966	FOZMOZ0	166.556	3.114	FOZMOZ0	169.201	2.938	FOZMOZ0	157.059	3.259
FOZMOZ0	173.134	2.909	FOZMOZ0	170.412	2.868	FOZMOZ0	155.404	3.326	FOZMOZ0	171.096	3.273	FOZMOZ0	167.19	3.273
FOZMOZ0	167.311	3.469	FOZMOZ0	150.299	2.715	FOZMOZ0	150.646	3.367	FOZMOZ0	153.277	3.281	FOZMOZ0	167.331	3.214
FOZMOZ0	166.139	3.273	FOZMOZ0	168.202	3.214	FOZMOZ0	157.311	3.244	FOZMOZ0	170.195	3.279	FOZMOZ0	172.064	2.91
FOZMOZ0	155.351	3.335	FOZMOZ0	168.608	2.938	FOZMOZ0	152.677	3.282	FOZMOZ0	167.088	3.279	FOZMOZ0	154.678	2.962
FOZMOZ0	171.571	2.762	FOZMOZ0	151.352	3.381	FOZMOZ0	150.234	3.441	FOZMOZ0	173.666	2.986	FOZMOZ0	163.581	3.172
FOZMOZ0	165.644	3.464	FOZMOZ0	156.542	3.102	FOZMOZ0	166.402	3.112	FOZMOZ0	152.692	3.261	FOZMOZ0	166.254	3.056
FOZMOZ0	166.613	3.467	FOZMOZ0	166.446	3.044	FOZMOZ0	169.999	2.879	FOZMOZ0	166.267	3.28	FOZMOZ0	155.932	3.121
FOZMOZ0	170.915	3.28	FOZMOZ0	151.665	3.274	FOZMOZ0	167.151	3.472	FOZMOZ0	151.631	3.425	FOZMOZ0	167.668	2.95
FOZMOZ0	157.603	3.244	FOZMOZ0	164.293	3.163	FOZMOZ0	166.831	3.121	FOZMOZ0	170.329	2.774	FOZMOZ0	151.891	3.375
FOZMOZ0	172.538	2.9	FOZMOZ0	152.445	3.287	FOZMOZ0	154.648	2.971	FOZMOZ0	155.919	3.334	FOZMOZ0	169.511	2.886
FOZMOZ0	174.013	2.995	FOZMOZ0	150.862	2.708	FOZMOZ0	168.179	3.209	FOZMOZ0	150.19	3.366	FOZMOZ0	166.515	3.28
FOZRAP	150.207	3.407	FOZRIW	154.454	3.399	FOZQUH	167.569	2.817	FOZQUH	160.027	3.052	FOZQUH	154.036	3.088
FOZRIW	156.778	3.28	FOZRIW	161.78	2.843	FOZRIW	160.065	3.469	FOZRIW	152.672	3.172	FOZRIW	153.957	3.444
FPAMCA	155.541	3.454	FPAMCA1	162.229	2.813	FOZRIW	152.282	2.83	FOZROC	171.782	3.15	FOZROC	171.769	3.135

FPAMCA2	156.171	3.389	FPCYTP1	161.851	3.423	FPAMCA1	156.118	3.386	FPAMCA1	157.261	3.381	FPAMCA2	157.221	3.383
FPIRCO10	152.106	2.812	FPPBCU	160.856	3.079	FPFAZP	173.377	2.972	FPHBCU	150.39	3.179	FPHBCU	171.254	3.286
FPSULF1	152.158	3.358	FPSULF1	151.902	3.193	FPSULF1	153.368	3.124	FPSULF1	172.127	3.193	FPSULF1	151.994	3.049
FPTBCO	157.682	3.308	FPZCRU1	156.891	3.003	FPSULF1	153.325	3.392	FPSULF1	152.64	2.89	FPSULF1	159.508	3.083
FTHXCO	163.604	3.243	FTHXCO	165.364	3.172	FSEANT	157.824	2.951	FSEANT	154.022	3.387	FTHXCO	155.93	3.181
FTHXCO	164.061	2.768	FTHXCO	166.121	3.318	FTHXCO	155.411	3.099	FTHXCO	170.948	3.181	FTHXCO	163.651	2.768
FUBJOC	150.796	3.041	FUBLAQ	167.685	3.185	FTHXCO	158.499	3.185	FUBJAO	171.698	3.052	FUBJOB	160.145	3.363
FUBLIA	168.319	3.22	FUBLIY	150.913	3.12	FUBLEW	163.586	3.439	FUBLEW	167.65	3.343	FUBLIA	159.478	3.339
FUBLUM	164.161	3.021	FUBNAR	156.778	3.369	FUBLOD	150.562	3.23	FUBLOG	152.937	3.326	FUBLOG	159.183	3.427
FUBVOO	165.574	2.676	FUBVOO	167.387	3.358	FUBPID	174.302	3.171	FUBPID	154.036	3.486	FUBVAA	163.547	3.323
FUCXOQ	162.356	3.444	FUCXOQ	170.765	3.457	FUBVOO	151.212	2.998	FUCXOQ	155.136	3.408	FUCXOQ	166.643	2.847
FUDGUK	152.443	2.881	FUDGUK	155.406	3.145	FUCXOQ	177.305	2.847	FUCXOQ	156.48	3.358	FUCXOQ	154.031	3.408
FUDHUL	154.769	3.326	FUDHUL	157.79	3.07	FUDHEV	166.839	3.02	FUDHUL	157.116	2.941	FUDHUL	160.464	3.213
FUDHUL	155.083	3.078	FUDNUP	167.626	3.444	FUDHUL	165.953	3.07	FUDHUL	176.605	3.078	FUDHUL	150.524	3.033
FUDQOK	158.588	3.221	FUDWOT	161.162	2.95	FUDNUP	158.463	3.036	FUDNUP	163.162	3.209	FUDNUP	152.583	3
FUFJUN	175.272	3.296	FUFJUN	152.749	3.131	FUDWOT	165.628	3.402	FUFBEN	169.737	2.757	FUFJUN	165.503	3.154
FUFJUN	153.236	2.971	FUFJUN	156.422	3.211	FUFJUN	166.503	3.389	FUFJUN	157.961	2.99	FUFJUN	157.652	3.296
FUFJUN	159.741	2.892	FUFJUN	155.435	2.843	FUFJUN	155.42	2.867	FUFJUN	168.982	3	FUFJUN	166.086	3.231
FUFJUN	169.565	3.288	FUFJUN	151.95	3.18	FUFJUN	165.182	3.39	FUFJUN	164.248	3.009	FUFJUN	160.072	3.228
FUFKAU	159.555	2.863	FUFKAU	152.592	3.142	FUFJUN	156.485	2.996	FUFKAU	172.81	3.226	FUFKAU	160.163	3.293
FUGJIE	154.223	3.084	FUGJIE	156.433	3.268	FUFKAU	159.872	3.319	FUGJID	153.824	3.193	FUGJIE	151.46	2.897
FUGVAI	173.779	2.917	FUGVAI	161.535	3.348	FUGPUU	152.152	3.167	FUGQAB	160.389	3.366	FUGVAI	170.505	3.233
FUHMIH	153.532	3.48	FUHMIH	151.774	2.894	FUGVAI	174.432	2.971	FUGVAI	161.146	3.221	FUHJIB	153.823	3.19
FUHVOT	169.518	2.903	FUHZER	169.774	3.074	FUHMUT	154.708	2.749	FUHQEF	165.651	2.914	FUHVOT	162.71	3.437
FUJPAE	161.045	2.822	FUJTOU	155.487	3.467	FUHZER0	170.215	3.088	FUJKAA	172.694	3.303	FUJPAE	151.783	3.398
FULDIC	151.569	3.028	FULDIC	151.874	3.089	FUJTOU	167.64	3.321	FULCIA	166.733	2.783	FULDIC	155.107	3.034
FULGUQ	163.047	3.016	FULHIF	171.819	3.129	FULDIC	165.943	3.236	FULDIC	150.58	2.83	FULDIC	166.309	3.353
FUMBEX	171.866	3.497	FUMBUK	170.055	3.127	FULLIJ	152.584	3.115	FULLUV	157.805	3.236	FUMBEX	155.172	3.283
FUMCEZ	163.021	3.307	FUMCEZ0	171.252	3.315	FUMBUK	156.297	3.187	FUMCEZ	174.438	3.353	FUMCEZ	168.054	3.071
FUMKAB	154.107	3.499	FUMKEF	165.073	3.208	FUMFUS	168.532	3.49	FUMHAA	162.859	2.677	FUMHAA	161.386	2.85
FUMLUY	163.196	2.872	FUMRIR	155.33	2.931	FUMKEF	168.609	3.354	FUMLEI	157.157	3.236	FUMLEI	164.726	3.369
FUMTEP	158.724	2.821	FUMTEP	170.887	3.477	FUMRIR	152.859	2.931	FUMROX	173.674	2.709	FUMROX	156.235	2.87
FUMTEP0	175.09	2.946	FUMVUI	154.678	3.132	FUMTEP	160.52	3.054	FUMTEP0	168.527	3.428	FUMTEP0	166.069	3.263
FUMZAQ	160.295	3.432	FUMZAQ	168.541	2.851	FUMVUI	165.436	3.087	FUMVUI	174.406	3.132	FUMZAQ	162.302	3.12
FUMZAQ	171.739	2.851	FUMZAQ	152.763	3.089	FUMZAQ	162.338	3.089	FUMZAQ	157.754	3.021	FUMZAQ	150.666	3.28
FUNDAT	153.735	3.121	FUNDAT	150.791	3.311	FUNCOG	163.589	2.797	FUNCOG	158.881	3.377	FUNDAT	152.995	3.056
FUNDEX	151.872	2.761	FUNDEX1	151.872	2.761	FUNDAT1	152.995	3.056	FUNDAT1	153.735	3.121	FUNDAT1	150.791	3.311
FUNDIB	168.542	3.276	FUNDIB	158.36	2.892	FUNDIB	178.032	3.276	FUNDIB	163.491	3.232	FUNDIB	164.617	3.281
FUNDIB10	164.617	3.281	FUNDIB10	168.542	3.276	FUNDIB	158.149	2.781	FUNDIB10	178.032	3.276	FUNDIB10	163.491	3.232
FUNDOH1	166.225	3.172	FUNGII	152.676	3.255	FUNDIB10	158.36	2.892	FUNDIB10	158.149	2.781	FUNDOH	166.225	3.172
FUNHIH	165.781	3.066	FUNHIH	167.477	3.144	FUNHIH	165.854	3.074	FUNHIH	161.737	3.187	FUNHIH	151.509	3.212
FUNHIH	171.856	2.807	FUNHIH	154.727	3.028	FUNHIH	177.141	2.916	FUNHIH	163.358	3.05	FUNHIH	155.157	3.317
FUNHIH	163.897	2.966	FUNHIH	153.619	2.694	FUNHIH	167.633	3.068	FUNHIH	152.739	3.372	FUNHIH	152.704	3.239
FUNHIH	154.94	3.346	FUNHIH	150.177	3.003	FUNHIH	172.724	3.474	FUNHIH	168.679	3.127	FUNHIH	157.963	3.013
FUNHUT	153.248	2.728	FUNHUT	169.926	2.782	FUNHIH	159.557	3.132	FUNHIH	152.826	2.945	FUNHIH	163.648	2.833
FUNHUT	151.795	2.763	FUNHUT	159.877	2.782	FUNHUT	165.437	3.206	FUNHUT	155.4	2.748	FUNHUT	165.878	3.155
FUNJAB	154.423	2.774	FUNJAB	154.222	3.127	FUNHUT	157.478	3.427	FUNHUT	152.624	3.205	FUNJAB	150.177	3.193
FUNJAB	172.307	2.876	FUNJAB	169.73	3.419	FUNJAB	163.802	2.892	FUNJAB	162.722	2.965	FUNJAB	154.356	3.013
FUNJAB	158.657	3.19	FUNJAB	164.918	3.317	FUNJAB	154.3	2.894	FUNJAB	154.986	3.48	FUNJAB	156.281	3.083
FUNJAB	160.793	3.127	FUNJAB	150.312	3.011	FUNJAB	161.544	3.221	FUNJAB	159.29	2.954	FUNJAB	158.633	2.774
FUNMEH	172.864	2.916	FUNMUX	153.586	2.99	FUNJAB	170.937	3.329	FUNMEH	158.227	2.976	FUNMEH	160.698	3.033
FUNPIR	178.642	3.438	FUNPIR	153.004	2.962	FUNMUX	160.134	2.91	FUNMUX	157.833	3.115	FUNMUX	154.548	3.178
FUNTOZ	169.43	3.322	FUNXEV	156.051	3.156	FUNPOX	178.856	3.414	FUNPOX	152.581	2.967	FUNTOZ	167.101	3.387
FUPHUV	168.549	3.162	FUPHUV	153.09	3.435	FUPBOH	158.091	3.463	FUPGUS	162.354	2.808	FUPGUS	161.2	3.48
FUPJUW	151.513	2.857	FUPJUW	164.383	3.316	FUPJUW	156.566	2.962	FUPJUW	161.485	3.122	FUPJUW	164.982	3.027
FUQGIK	162.666	3.386	FUQGUW	166.379	2.754	FUPJUW	170.469	3.212	FUPJUW	169.379	3.354	FUPJUW	162.771	3.044
FUQLOT	151.504	3.073	FUQLOT	156.565	2.807	FUQLOT	151.861	2.807	FUQLOT	177.326	3.339	FUQLOT	154.12	2.941
FUQLOW	172.074	2.997	FUQQEQ	165.145	3.015	FUQLOV	172.527	3.427	FUQLOW	152.486	3.053	FUQLOW	154.834	2.873
FUQSIW	174.683	2.845	FUQVAS	153.648	3.159	FUQSEQ	157.907	2.826	FUQSEQ	166.732	3.138	FUQSEQ	151.126	2.847
FUQYEW	174.724	2.965	FUQYEW	156.202	3.091	FUQWOF	167.475	3.336	FUQYEW	176.974	3.071	FUQYEW	153.432	3.344
FURACL0	154.02	3.091	FURACL1	156.95	3.052	FURACL0	156.782	3.045	FURACL0	159.388	3.046	FURACL0	151.186	3.05
FURACL1	158.755	3.08	FURACL1	156.043	3.081	FURACL1	159.738	3.055	FURACL1	151.633	3.058	FURACL1	154.273	3.093
FURJIP	162.255	2.825	FURJIP	151.221	3.45	FURACL1	150.71	3.09	FURACL1	152.633	3.143	FURJIP	169.682	3.31
FURLOX	165.27	3.49	FURLUD	173.291	3.299	FURJIP	158.151	2.825	FURKAI	152.53	2.941	FURLOX	155.223	2.805
FURSIV01	166.55	3.164	FURSIV01	167.901	2.913	FURSAN	164.244	3.402	FURSIV	165.689	3.38	FURSIV	161.717	2.946
FUSFUX	167.088	3.305	FUSFUX	160.241	2.652	FURSUJ	169.08	3.011	FURVOG	168.991	3.481	FURVOG	150.783	2.694
FUSFUX0	160.475	2.653	FUSFUX0	167.102	3.423	FUSFUX0	168.496	3.456	FUSFUX0	153.065	2.68	FUSFUX0	166.43	3.322
FUSPAO	150.795	2.749	FUSTIW	165.432	2.725	FUSFUX0	155.256	2.679	FUSFUX0	167.54	3.482	FUSFUX0	152.463	2.697
FUTFAC	155.63	3.312	FUTFAC	165.686	3.407	FUSTIW	159.859	3.117	FUSTIW	165.339	2.725	FUTFAC	168.269	3.318
FUTLEM	172.362	3.395	FUTLEM	163.297	2.938	FUTHEL	151.032	2.78	FUTLAK	153.758	3.02	FUTLEM	150.394	3.406
FUTLEM	151.373	3.309	FUTMEN	151.776	3.013	FUTLEM	150.922	3.427	FUTLEM	153.266	3.454	FUTLEM	167.187	2.938
FUTMOZ	163.568	3.376	FUTMOZ	157.249	3.154	FUTMOZ	162.99	3.354	FUTMOZ	157.571	2.988	FUTMOZ	154.572	3.08
FUVJOY	169.388	3.12	FUVJOY	164.916	3.392	FUTROF	167.951	3.224	FUTROF	175.407	2.977	FUVJOY	160.675	3.458
FUVMUE	163.128	3.328	FUVMUE	156.262	2.897	FUVJOY	171.609	3.346	FUVMUE	161.551	3.341	FUVMUE	171.727	3.236
FUVSOG	153.358	3.167	FUWKEP	154.985	3.091	FUVNAL	152.506	3.255	FUVNIW	163.59	3.07	FUVNUI	158.895	3.347
FUWMIV	152.466	3.227	FUWMIV	164.806	3.247	FUWKIT	153.795	3.302	FUWKOZ	167.867	3.41	FUWMIV	165.473	3.278
FUWPOF	152.631	3.276	FUWPOF	171.924	3.276	FUWPEV	161.073	3.277	FUWPIZ	154.841	3.089	FUWPIZ	171.502	3.089
FUWREV	159.243	3.216	FUWREV	168.582	2.823	FUWPUL	152.84	2.956	FUWQEW	158.645	2.965	FUWQEW	156.727	3.151
FUWREV	154.18	3.068	FUWREV	162.723	3.177	FUWREV	152.003	3.292	FUWREV	154.305	3.217	FUWREV	168.947	3.427

FUWREV	152.737	2.675	FUWXOM	157.257	3.372	FUWREV	166.579	3.216	FUWREV	165.508	2.885	FUWREV	151.087	3.08
FUXCEI	164.392	3.029	FUXGEN	150.474	3.5	FUWXOM	163.392	3.235	FUWXOM	164.212	3.055	FUWXOM	153.919	3.35
FUXZEE	154.286	3.053	FUYDEK	162.812	3.146	FUXGEN	158.634	3.313	FUXGEN	155.326	3.271	FUXMEU	164.736	3.368
FUYFAI03	152.895	3.182	FUYFAI03	162.601	2.844	FUYDEK0	161.061	3.111	FUYFAI03	158.936	2.744	FUYFAI03	171.016	2.963
FUYFAI04	171.135	2.963	FUYFAI04	152.381	3.214	FUYFAI03	158.918	2.829	FUYFAI03	160.475	2.614	FUYFAI04	159.99	2.754
FUYFAI05	162.564	2.766	FUYFAI05	171.547	2.929	FUYFAI04	162.459	2.85	FUYFAI04	158.587	2.835	FUYFAI04	161.074	2.625
FUYFAI05	163.668	2.648	FUYFAI05	152.329	2.865	FUYFAI05	150.572	3.264	FUYFAI05	162.067	2.845	FUYFAI05	158.398	2.828
FUYMET	162.746	2.921	FUYPEW	168.405	2.707	FUYMET	150.58	3.202	FUYMET	170.676	3.471	FUYMET	162.54	2.847
FUYYAB	158.627	2.953	FUZCUC	167.912	3.069	FUYPEW	160.539	2.998	FUYPEW	169.354	2.682	FUYYAB	176.799	2.953
FUZCUC	161.315	3.285	FUZDAJ	159.989	3.124	FUZCUC	158.428	3.085	FUZCUC	166.083	3.085	FUZCUC	154.858	3.392
FUZXEH	150.729	2.8	FUZXEH	153.664	2.724	FUZDAJ	170.222	2.897	FUZXEH	155.521	3.407	FUZXEH	158.286	3.194
FUZYOS	150.703	3.003	FUZYUY	150.233	3.469	FUZYAE	163.725	3.181	FUZYOS	163.189	2.995	FUZYOS	152.903	2.995
GABBAQ	151.861	2.876	GABBAQ	158.985	2.935	FUZYUY	170.729	3.006	FUZYUY	155.734	3.21	GABBAQ	174.855	3.038
GABBEU	161.408	2.933	GABBEU	167.583	3.024	GABBAQ	161.251	3.372	GABBAQ	166.24	2.951	GABBEU	157.858	2.851
GABBEU	160.231	3.368	GABBEU	157.99	3.155	GABBEU	176.174	2.68	GABBEU	151.433	3.399	GABBEU	177.454	3.181
GABBUK	153.9	3.319	GABBUK	161.185	2.732	GABBEU	159.18	3.377	GABBEU	153.479	3.024	GABBEU	168.231	3.054
GABBUK	163.364	2.858	GABBUK	153.921	3.361	GABBUK	172.937	3.361	GABBUK	159.261	2.957	GABBUK	157.954	2.892
GABCOF	166.93	2.957	GABCOF	156.187	3.476	GABCEV	152.705	3.445	GABCEV	164.687	2.877	GABCEV	159.028	3.258
GABQUW	150.376	3.164	GABQUW	158.098	2.836	GABCOF	155.904	3.268	GABFAS	163.155	2.6	GABFAS	166.964	2.966
GABQUW	152.343	2.991	GABQUW	157.733	2.937	GABQUW	153.818	3.452	GABQUW	162.422	3.059	GABQUW	159.199	3.482
GABRAD	158.3	2.994	GABRAD	151.413	3.091	GABQUW	167.012	2.755	GABQUW	168.358	3.053	GABRAD	168.022	3.438
GABRAD	163.73	2.993	GABRIL	151.189	2.934	GABRAD	166.142	3.366	GABRAD	160.906	3.005	GABRAD	166.387	3.121
GABRIL	178.124	3.432	GABRIL	166.361	3.239	GABRIL	169.772	3.047	GABRIL	152.853	2.838	GABRIL	154.909	2.862
GABWAJ	167.424	3.054	GABWAJ	165.956	3.331	GABWAJ	152.017	3.268	GABWAJ	162.88	2.875	GABWAJ	157.17	2.922
GACFOJ	157.836	3.069	GACFOJ	171.762	3.073	GABXAL	151.703	2.98	GABZER	168.357	3.036	GACFOJ	152.18	2.78
GACGID	173.125	2.885	GACGOJ	151.326	3.212	GACFOJ	150.748	3.082	GACGAW	166.943	3.003	GACGID	151.806	3.226
GACZIY	158.719	2.982	GACZIY	159.844	3.318	GACGOJ	174.776	2.884	GACSIN	155.36	3.442	GACTUE	152.323	2.867
GADHOK	162.318	2.707	GADLIK	165.677	3.019	GADHOK	159.219	3.197	GADHOK	168.176	2.866	GADHOK	164.906	2.82
GADPOT	155.723	3.067	GADXIT	154.037	2.851	GADPOT	151.469	3.089	GADPOT	160.951	3.213	GADPOT	170.834	3.089
GADYAO	173.715	3.041	GADYAO	156.307	2.891	GADXIT	177.295	3.229	GADXIT	156.961	3.234	GADYAO	151.682	2.798
GAFHUR	166.422	3.22	GAFLAB	150.132	3.417	GADYAO	160.303	3.17	GADYAO	172.636	3.17	GADYAO	163.047	2.831
GAFLIJ	166.386	3.07	GAFLIN	160.779	2.915	GAFLEF	163.588	3.193	GAFLEF	167.267	3.315	GAFLIJ	158.564	3.077
GAFLUV	156.66	3.334	GAFMUY	160.977	2.953	GAFLOP	162.488	2.998	GAFLOP	160.968	2.838	GAFLOP	171.321	2.949
GAFQOY	168.972	2.749	GAFROU	159.442	3.101	GAFMUY	170.157	3.302	GAFMUY	151.279	3.011	GAFQOY	157.166	3.349
GAFXAO	158.6	2.859	GAFXAO	151.342	3.214	GAFROU	155.349	3.435	GAFXAO	153.912	3.421	GAFXAO	173.913	3.101
GAFZIX	152.571	3.429	GAGHEF	154.921	3.126	GAFXAO	165.62	3.042	GAFXAO	151.921	3.073	GAFXAO	151.517	2.859
GAGVAM	158.475	2.765	GAGVAM	161.139	2.721	GAGMUA	156.311	3.163	GAGPIO	155.699	3.278	GAGQUE	150.226	3.407
GAGXEU	155.816	3.117	GAGXEU	165.672	3.428	GAGVAM	154.006	3.008	GAGXEU	163.486	3.497	GAGXEU	155.705	3.147
GAHDAZ	176.912	3.156	GAHDAZ	176.215	2.954	GAGYOH	165.585	3.012	GAHBUR	162.567	3.365	GAHCIF	152.703	3.39
GAHDUT	179.721	3.389	GAHFOP	151.947	3.268	GAHDIH	171.58	2.975	GAHDIH	169.263	3.133	GAHDUT	174.314	3.119
GAHLAG	173.236	3.233	GAHLAG	164.823	2.927	GAHFUV	151.094	2.79	GAHHAD	166.523	3.327	GAHHAD	160.484	3.005
GAHLEK	159.315	2.82	GAHQIU	150.038	2.947	GAHLAG	153.429	3.066	GAHLAG	157.03	2.985	GAHLEK	159.235	3.063
GAHSES	156.178	3.161	GAJHEH	156.902	2.809	GAHQIU	171.943	3.494	GAHSAO	160.841	2.697	GAHSES	157.273	3.238
GAJRER	159.336	3.394	GAJRER	150.763	2.977	GAJRAN	173.83	3.401	GAJRER	160.127	2.706	GAJRER	160.247	2.706
GAJWEX	169.896	2.928	GAJWEX	152.662	3.081	GAJRIV	159.716	2.807	GAJRIV	177.754	3.323	GAJWEX	175.167	2.917
GAJWEX	163.013	2.838	GAJWEX	160.945	2.926	GAJWEX	150.055	3.309	GAJWEX	172.16	2.768	GAJWEX	159.594	2.979
GAJZAX	150.539	3.222	GAJZAX	169.252	2.912	GAJWEX	167.122	2.762	GAJWEX	156.655	3.103	GAJWEX	170.284	2.926
GAJZID	152.376	3.076	GAJZID	170.9	3.462	GAJZAX	155.834	3.385	GAJZID	167.379	3.177	GAJZID	166.751	3.197
GAKHUX	168.023	3.367	GAKKAI	162.522	2.858	GAJZID	171.881	3.32	GAKCEA	165.751	3.257	GAKDUT	159.73	3.154
GAKKIQ	154.4	3.234	GAKKIQ	161.898	3.046	GAKKAI	150.164	2.916	GAKKEM	156.329	2.824	GAKKIQ	165.493	2.997
GAKTUK	177.81	3.146	GAKTUK	175.61	3.487	GAKKIQ	157.467	3.146	GAKLUC	154.045	2.737	GAKTUK	167.9	3.403
GAKVIA	177.401	3.054	GAKVOG	173.504	3.224	GAKTUK	174.674	3.248	GAKVIA	161.028	3.404	GAKVIA	163.481	3.117
GAKWOH	160.135	2.693	GAKWOH	153.96	3.438	GAKVUM	153.657	2.983	GAKWOH	165.782	3.401	GAKWOH	152.711	2.848
GAKWUN	157.976	3.085	GAKWUN	150.017	3.363	GAKWOH	162.65	2.693	GAKWUN	172.906	3.177	GAKWUN	154.081	3.353
GALBAA	153.865	3.303	GALBAA	171.544	3.331	GAKWUN	161.666	2.817	GALBAA	161.697	3.085	GALBAA	163.301	3.423
GALDED	156.955	3.396	GALDED	166.781	3.061	GALBEE	154.86	2.963	GALBEE	155.759	2.988	GALBEE	150.93	3.001
GALJEL	150.649	3.15	GALJEL	157.983	2.745	GALDED	169.628	3.489	GALJEI	160.925	2.85	GALJEI	167.459	2.907
GALNAM0	157.165	3.478	GALNAM0	156.107	3.459	GALJUC	156.564	3.365	GALJUC	155.096	3.2	GALNAM0	155.083	3.484
GALNUG	150.933	3.328	GALNUG0	150.955	3.304	GALNAM0	157.949	3.45	GALNAM0	159.301	3.415	GALNAM0	161.418	3.399
GALQET	156.787	3.352	GALQET	167.479	2.991	GALNUG0	151.265	3.278	GALNUG0	151.322	3.254	GALQET	156.068	2.957
GAMKEO	155.99	3.21	GAMKEO	154.404	2.845	GALSEU	161.993	3.047	GALSEU	154.693	3.132	GAMKEO	157.102	2.845
GAMPEP	165.526	3.154	GAMPEP	150.912	3.498	GAMPEP	162.232	2.81	GAMPEP	157.046	3.373	GAMPEP	175.633	2.847
GANBOO	152.419	3.25	GANFIK	155.721	2.878	GAMXEB	152.67	3.382	GANBAA	163.685	3.104	GANBEF	174.914	2.663
GANWAX	163.871	2.814	GANYUP	159.653	3.002	GANFIK	153.031	2.923	GANFIK	155.402	3.305	GANWAX	150.481	2.918
GANYUP	153.504	2.84	GANYUP	178.862	2.682	GANYUP	155.535	3.421	GANYUP	157.653	3.366	GANYUP	169.18	3.166
GANYUP	151.981	3.482	GANYUP	164.294	3.178	GANYUP	169.333	2.922	GANYUP	161.543	3.472	GANYUP	159.857	3.391
GAPHOY	158.441	2.907	GAPHOY	168.665	2.806	GANZAW	151.69	3.07	GAPHOY	160.052	2.842	GAPHOY	157.266	2.913
GAPHOY	168.528	3.159	GAPHOY	158.121	2.85	GAPHOY	155.741	2.901	GAPHOY	158.852	3.304	GAPHOY	156.356	2.776
GAPJAM	172.515	3.171	GAPJAM	156.344	3.24	GAPHOY	153.119	3.339	GAPJAM	168.01	3.284	GAPJAM	166.753	2.875
GAPJAM	155.707	2.966	GAPJAM	162.074	3.001	GAPJAM	158.457	2.893	GAPJAM	152.083	2.777	GAPJAM	152.185	3.112
GAPJAM	163.431	3.412	GAPVID	152.778	3.237	GAPJAM	165.22	3.389	GAPJAM	150.002	3.021	GAPJAM	163.422	3.446
GAQHUF	156.227	3.342	GAQKAN	170.688	2.77	GAQCOS	155.082	3.44	GAQDOV	152.404	3.047	GAQDOV	159.1	3.3
GARJAO	175.96	2.843	GARJAO	157.49	3.303	GARHIR	155.877	3.405	GARJAM	159.721	2.891	GARJAM	176.073	2.873
GARJES	151.614	3.186	GARJES	155.41	3.053	GARJES	162.954	2.952	GARJES	156.403	3.484	GARJES	154.615	3.186
GARJES	167.984	3.073	GARJES	156.183	3.125	GARJES	151.222	3.262	GARJES	159.434	3.315	GARJES	157.617	2.899
GARYEG	162.43	3.317	GASFUD	156.81	2.889	GARJES	172.999	2.992	GARJES	150.582	2.933	GARJES	164.314	2.892
GASFUD	153.385	2.816	GASFUD	156.008	3.312	GASFUD	150.966	2.828	GASFUD	160.93	2.669	GASFUD	157.389	2.6
GASPIB	160.062	2.897	GASPIB	153.837	3.356	GASFUD	157.378	2.87	GASKOE	151.491	3.112	GASMOG	162.96	3.263
GATJEU	150.364	2.796	GATKUI	153.766	3.19	GASPIB	161.93	2.92	GATDIP	173.576	3.109	GATFIR	153.262	2.687

GATSIE	159.555	2.988	GATSIE	150.47	3.094	GATKUI	150.163	3.383	GATKUI	152.035	3.365	GATKUI	168.63	3.419
GATSIE	171.504	3.367	GATSOK	169.649	3.166	GATSIE	155.324	3.147	GATSIE	159.559	3.316	GATSIE	158.591	3.494
GAVCAK	153.981	3.219	GAVMEZ	177.039	3.443	GATSOK	162.003	3.305	GAVBIR	162.086	3.378	GAVCAK	151.446	3.15
GAVMOJ	158.102	2.888	GAVQAY	167.226	3.497	GAVMIB	177.31	3.468	GAVMID	156.503	2.698	GAVMID	168.152	3.188
GAVQED	154.845	2.901	GAVRAZ	153.932	3.172	GAVQAY	152.976	3.096	GAVQED	166.397	2.917	GAVQED	166.312	3.445
GAVXEG	152.566	2.943	GAWCAJ	167.785	3.086	GAVRAZ	151.945	2.97	GAVXEG	162.956	3.485	GAVXEG	152.752	2.959
GAWQAA	154.481	3.156	GAWQEE	154.877	3.123	GAWCAJ	159.885	3.373	GAWCAJ	155.804	2.906	GAWFUI	161.431	2.957
GAWWAE	164.174	3.476	GAWWOS	159.501	3.43	GAWRUT	154.663	2.889	GAWTOQ	162.648	3.214	GAWWAE	160.439	3.476
GAXFIW	154.599	3.105	GAXFIW	172.664	3.252	GAWWOS	164.701	3.43	GAWXOU	170.262	2.946	GAXDIW	156.01	3.335
GAXFIW	170.411	2.893	GAXFIW	159.842	3.032	GAXFIW	154.177	3.465	GAXFIW	178.561	3.046	GAXFIW	157.116	3.252
GAXGIZ	151.914	2.92	GAXVOT0	164.633	3.016	GAXFIW	156.812	3.106	GAXFIW	155.912	3.003	GAXFIW	173.233	3.13
GAXVOT0	167.399	3.016	GAXVOT0	164.018	3.071	GAXVOT0	154.404	2.979	GAXVOT0	157.575	3.223	GAXVOT0	176.844	3.372
GAZBAM	162.868	3.247	GAZGOG	168.081	3.042	GAXXOU	165.277	3.06	GAXYOV	160.268	2.916	GAYBUG	162.247	3.267
GAZPOQ	172.283	3.259	GAZPOQ	155.478	3.023	GAZJAS	154.334	3.38	GAZKOK	168.163	2.936	GAZPOQ	163.462	2.853
GAZQIL	173.073	3.221	GAZQIL	152.824	3.133	GAZQAD	164.553	2.874	GAZQAD	154.068	3.192	GAZQAD	173.112	3.254
GEBBUN	151.236	3.405	GEBBUN	160.651	3.445	GAZQIL	164.09	2.89	GAZRUX	152.116	3.145	GEBBOH	159.854	3.346
GEBCIB	156.103	2.924	GEBFAW	161.423	2.953	GEBCEX	168.034	2.995	GEBCEX	159.553	3.282	GEBCIB	167.01	3.159
GEBJOP	170.12	3.483	GEBJOP	163.217	3.483	GEBFAW	167.824	2.953	GEBFAW	158.273	2.966	GEBFAW	150.974	2.988
GEBNOS	160.957	2.749	GEBNOS	151.696	3.372	GEBNAE	154.937	3.424	GEBNAE	168.321	2.754	GEBNAE	174.711	3.093
GECFIH	163.603	2.958	GECFIH	170.065	2.713	GEBPEM	151.245	2.849	GECCOK	173.915	3.018	GECCOK	166.659	3.018
GECGOO	156.663	2.859	GECGOO	163.543	2.975	GECFON	170.261	2.789	GECGEE	159.893	3.43	GECGOO	156.165	3.058
GECGUU	156.092	2.887	GECGUU	160.011	3.379	GECGOO	152.497	2.733	GECGOO	154.742	2.717	GECGOO	155.572	2.975
GECKAE	158.155	2.638	GECKAE	169.116	2.955	GECKAE	163.949	2.684	GECKAE	151.069	3.096	GECKAE	151.193	3.211
GECKIM	156.683	3.103	GECLIL	151.666	3.336	GECKEI	151.283	2.473	GECKEI	155.743	3.397	GECKIM	155.211	3.103
GECZIZ	177.567	3.255	GEDFAA	163.325	3.183	GECVES	173.665	3.08	GECVES	151.752	3.119	GECYEU	165.525	3.319
GEDHAC	164.259	3.041	GEDJIM	153.527	2.977	GEDFAA	169.811	2.787	GEDFOO	162.886	3.109	GEDHAC	172.64	3.202
GEDLIL	157.79	3.36	GEDLIL	158.574	3.001	GEDJIM	153.529	2.977	GEDKOT	151.967	3.026	GEDLEH	163.462	2.844
GEDRAL0	160.646	3.031	GEDRAL0	169.926	2.865	GEDLOR	160.285	3.374	GEDMUX	171.75	3.197	GEDNUY	154.491	2.8
GEDRAL0	161.586	2.904	GEDRAL0	162.125	3.057	GEDRAL0	165.25	2.835	GEDRAL0	166.597	3.147	GEDRAL0	173.112	2.84
GEDSAM0	166.345	2.963	GEDSIU	167.667	3.161	GEDROA	176.371	2.956	GEDROA	158.876	3.256	GEDSAM	162.017	3.45
GEFCAX	162.607	3.177	GEFCAX	150.609	2.901	GEDTER	157.797	3.015	GEFCAX	151.933	3.336	GEFCAX	161.744	2.959
GEFCIF	161.522	3.302	GEFLUZ	157.587	2.683	GEFCEB	152.763	2.885	GEFCEB	162.045	3.307	GEFCEB	163.565	3.234
GEFWEX	169.609	3.131	GEFWEX	152.324	2.729	GEFLUZ	166.921	3.079	GEFLUZ	154.071	2.871	GEFLUZ	156.59	2.84
GEGDII	156.186	3.254	GEGDOO	157.652	3.03	GEFWOH	153.173	3.433	GEFWOH	170.938	3.188	GEGDII	170.141	3.374
GEGWOI	171.118	3.171	GEGWOI	152.553	3.459	GEGDOO	151.964	2.844	GEGLOV	158.898	2.92	GEGVUM	152.898	3.3
GEGXAV	161.063	3.209	GEGXAV	158.195	3.217	GEGWOI	159.801	3.384	GEGWOI	156.786	2.758	GEGXAV	161.964	2.835
GEGXOG	153.956	3.06	GEGXOG	156.174	2.768	GEGXAV	166.613	3.052	GEGXOG	163.825	3.322	GEGXOG	168.111	3.059
GEGXUM	165.969	3.34	GEGXUM	174.356	3.46	GEGXUM	152.227	2.713	GEGXUM	164.667	3.448	GEGXUM	159.992	2.85
GEGYUM	170.61	3.039	GEGYUM	153.216	2.998	GEGXUM	152.045	2.842	GEGXUM	158.567	2.816	GEGYUM	155.813	3.117
GEHBEB	172.665	2.96	GEHBEB	173.657	3.442	GEGYUM	153.168	3.292	GEHBAW	153.794	2.816	GEHBEB	163.434	2.96
GEHCAX	166.871	3.46	GEHCUU	160.681	3.017	GEHBEB	169.831	3.24	GEHBEB	157.673	3.359	GEHCAX	150.213	2.969
GEHLUD	151.49	3.289	GEHMOZ	156.475	2.795	GEHHAF	150.792	3.326	GEHLOX	155.224	3.359	GEHLUD	150.941	3.181
GEHNAM	154.475	2.772	GEHNOA	173.207	3.344	GEHMOZ	158.221	2.795	GEHNAM	154.922	3.205	GEHNAM	167.454	3.292
GEHNOA	166.561	2.859	GEHNOA	158.657	3.097	GEHNOA	161.443	3.007	GEHNOA	163.011	2.946	GEHNOA	176.279	2.992
GEHPAO	158.149	2.573	GEHPAO	153.535	3.266	GEHNOA	164.827	3.194	GEHNOA	173.457	3.176	GEHPAO	157.821	3.052
GEHSIW	166.653	3.119	GEJKAL	159.344	2.988	GEHPAO	175.227	3.159	GEHPAO	157.869	3.298	GEHSIW	151.424	3.416
GEJKAL	156.102	3.255	GEJMIT	154.181	3.048	GEJKAL	150.57	3.282	GEJKAL	162.513	3.053	GEJKAL	166.937	3.16
GEJMOB	165.81	3.115	GEJMOB	171.923	3.088	GEJMOB	167.037	3.252	GEJMOB	153.628	2.875	GEJMOB	154.779	2.95
GEJQUJ	160.485	2.852	GEJQUJ	160.935	3.074	GEJMOB	155.423	3.115	GEJMOB	163.455	3.135	GEJMOZ	169.929	3.229
GEJSAR	163.26	3.195	GEJSEV	152.666	3.372	GEJQUJ	151.022	2.836	GEJQUJ	169.081	2.999	GEJQUJ	166.516	2.923
GEJSEV	170.44	3.335	GEJSEV	152.408	3.445	GEJSEV	156.78	3.052	GEJSEV	173.526	3.09	GEJSEV	151.511	3.189
GEJYUP	154.368	3.107	GEKCEI	150.016	3.133	GEJSEV	153.193	3.161	GEJWUP	165.862	3.11	GEJYUP	153.546	3.458
GEKCEI	168.939	2.856	GEKCEI	163.543	3.477	GEKCEI	156.323	2.804	GEKCEI	159.487	2.9	GEKCEI	178.962	2.847
GEKGOV	159.897	3.357	GEKLIU	167.196	3.112	GEKDEJ	150.537	3.127	GEKGIP	170.942	3.477	GEKGOV	159.057	3.267
GELHEM	155.114	3.039	GELHEM	153.589	3.276	GEKZOO	150.885	3.286	GELFAG	163.8	3.478	GELFAG	172.079	3.084
GELPOG	162.855	2.878	GELPOG	152.467	3.158	GELHEM	155.215	2.876	GELHEM	151.784	3.043	GELHEM	153.334	3.473
GELPOG	150.542	2.946	GELQAT	160.309	3.155	GELPOG	166.53	2.878	GELPOG	170.888	2.703	GELPOG	158.008	3.47
GELRIC	150.777	2.928	GELRIC	172.356	2.771	GELQEX	151.589	3.478	GELQEX	160.785	3.247	GELQEX	165.997	3.237
GELRIC	163.9	2.928	GELZAB	169.11	3.478	GELRIC	170.623	2.968	GELRIC	163.28	3.099	GELRIC	157.479	2.847
GEMFOW	161.897	3.04	GEMFOW	178.6	2.941	GELZAB	150.641	2.868	GELZAB	155.144	2.862	GELZAB	171.999	2.953
GEMFUB	160.816	3.405	GEMGEN	152.228	3.309	GEMFOW	155.994	2.941	GEMFUB	163.623	3.071	GEMFUB	150.563	3.318
GEMGEN	165.399	2.903	GEMLUI	157.052	3.119	GEMGEN	156.584	2.705	GEMGEN	152.836	3.253	GEMGEN	152.928	2.83
GEMNEU	174.871	2.948	GEMNEU	155.634	3.011	GEMMUJ	164.105	2.95	GEMNAQ	168.299	2.892	GEMNAQ	152.299	2.734
GEMNIY	159.461	3.131	GEMNIY	160.184	3.133	GEMNEU	150.103	2.754	GEMNIY	160.539	2.732	GEMNIY	163.717	3.032
GEMNOB	154.156	3.104	GEMNOB	158.947	3.363	GEMNIY	152.319	3.336	GEMNOB	157.586	3.018	GEMNOB	150.238	3.257
GENJUI	164.673	3.025	GENKAP	162.518	3.282	GEMNOB	161.518	3.01	GEMNOB	157.746	3.424	GENCIN	166.031	3.204
GENKAP	158.248	2.983	GENKOZ	162.038	2.913	GENKAP	152.795	3.191	GENKAP	153.881	3.067	GENKAP	156.263	3.282
GENQOI	168.586	3.323	GENVOM	172.894	2.959	GENKOZ	152.606	2.772	GENKOZ	155.088	2.85	GENQEZ	162.145	3.234
GENXAB	160.979	3.109	GENXUT	150.473	2.713	GENVOM	176.516	3.197	GENVOM	154.967	3.339	GENXAB	166.637	3.448
GEPRUS	164.656	2.877	GEPRUS	157.382	2.95	GEPCUA	162.585	3.15	GEPPIA	165.893	3.092	GEPROM	160.547	3.433
GEQCUC	170.672	3.115	GEQCUC	169.494	3.198	GEQCUC	152.566	2.927	GEQCUC	152.881	2.922	GEQCUC	167.732	3.345
GEQCUC	163.583	2.746	GEQCUC	167.249	2.739	GEQCUC	171.277	3.028	GEQCUC	154.377	3.35	GEQCUC	158.754	2.813
GEQDAJ	152.472	3.049	GEQDAJ	154.252	3.316	GEQCUC	167.667	3.474	GEQCUC	155.487	2.976	GEQCUC	165.119	3.242
GEQDEN	165.21	2.716	GEQDEN	172.643	2.716	GEQDAJ	160.801	3.082	GEQDAJ	166.05	2.954	GEQDAJ	164.227	2.954
GEQHAN	176.85	3.131	GEQHAN	150.778	3.216	GEQDUF	157.717	3.363	GEQHAN	152.643	2.814	GEQHAN	158.311	3.358
GEQHAN	161.683	3.447	GEQHAN	156.877	2.972	GEQHAN	152.411	2.841	GEQHAN	166.114	3.167	GEQHAN	152.801	2.905
GEQHER	152.182	3.007	GEQHER	176.063	3.29	GEQHAN	160.232	2.761	GEQHAN	154.578	3.056	GEQHAN	156.776	2.996
GEQHER	158.085	3.425	GEQHER	161.695	3.111	GEQHER	150.993	3.249	GEQHER	153.273	3.234	GEQHER	152.042	2.942
GEQHER	151.731	3.111	GEQHER	153.312	3.118	GEQHER	152.166	2.98	GEQHER	165.729	3.368	GEQHER	155.845	2.638

GEQHER	154.578	2.982	GEQHER	150.271	2.902	GEQHER	150.274	3.07	GEQHER	151.662	3.496	GEQHER	151.522	2.982
GEQHER	164.059	3.198	GEQHER	156.073	3.274	GEQHER	169.865	3.067	GEQHER	159.814	3.439	GEQHER	161.514	3.146
GEQHIV	151.559	3.164	GEQHIV	159.885	3.447	GEQHER	153.615	2.792	GEQHER	171.347	3.067	GEQHIV	157.31	2.961
GEQHIV	169.012	2.811	GEQHIV	167.322	3.394	GEQHIV	162.729	2.97	GEQHIV	154.817	2.975	GEQHIV	153	3.184
GEQHIV	154.096	3.046	GEQHIV	151.308	3.364	GEQHIV	166.923	3.132	GEQHIV	153.497	2.965	GEQHIV	153.05	2.97
GEQJAP	157.415	3.456	GEQJAP	163.766	2.994	GEQHIV	166.199	3.217	GEQJAP	151.013	2.93	GEQJAP	168.33	3.117
GEQJAP	162.79	3.363	GEQJAP	151.49	3.184	GEQJAP	166.602	3.142	GEQJAP	158.552	3.248	GEQJAP	158.168	3.312
GEQJAP	150.819	2.892	GEQMOH	152.774	2.86	GEQJAP	160.827	3.188	GEQJAP	151.779	3.437	GEQJAP	166.435	3.173
GEQNEY	160.772	3.021	GEQNEY	165.422	3.2	GEQMOH	152.387	3.192	GEQMOH	167.423	3.189	GEQMOH	161.048	3.041
GEQTUV	160.898	3.201	GEQTUV	161.97	2.874	GEQNEY	153.835	2.89	GEQNEY	153.086	3.288	GEQTUV	175.726	3.408
GERBEO	165.057	3.228	GERCUC	164.625	2.926	GEQTUV	162.852	2.838	GEQZEL	161.678	3.3	GEQZEL	158.627	3.055
GERKOH	154.131	2.903	GERKOH	158.949	3.174	GERCUC	156.998	2.943	GERKOH	150.281	2.782	GERKOH	164.411	3.186
GERKUN	170.663	2.851	GERKUN	159.426	3.279	GERKOH	170.026	2.836	GERKOH	167.212	2.982	GERKOH	163.402	2.988
GERLAU	157.263	3.202	GERLAU	151.39	3.067	GERKUN	158.886	3.218	GERKUN	153.33	2.757	GERKUN	161.488	3.276
GERLAU	150.163	2.893	GERLAU	151.266	2.893	GERLAU	167.206	3.168	GERLAU	150.792	2.751	GERLAU	164.94	2.993
GERLAU	165.815	2.94	GERNOK	152.24	3.097	GERLAU	164.562	3.04	GERLAU	162.199	2.899	GERLAU	172.896	3.235
GESCEQ	152.903	3.097	GESCIU	152.737	3.03	GERQAX	154.524	3.192	GERYAE	160.171	2.947	GERYAE	163.014	2.947
GESZIP	160.014	3.11	GESZIP	153.875	3.003	GESYUA	151.794	3.265	GESYUA	157.441	2.928	GESZIP	160.443	2.908
GETJOF	151.139	3.058	GETJOF	155.643	3.234	GESZIP	157.786	3.2	GESZIP	172.069	2.797	GETJOF	161.906	3.147
GETJOF	151.976	3.227	GETJOF	150.09	3.005	GETJOF	158.089	3.351	GETJOF	162.825	2.898	GETJOF	168.854	3.346
GETJUL	152.234	2.969	GETJUL	151.943	3.498	GETJOF	153.799	3.034	GETJUL	150.557	3.315	GETJUL	161.348	3.481
GETJUL	160.263	2.729	GETJUL	153.674	3.277	GETJUL	150.719	3.273	GETJUL	154.286	3.255	GETJUL	159.57	3.321
GETJUL	168.18	3.255	GETJUL	169.521	3.092	GETJUL	155.719	3.239	GETJUL	167.028	3.062	GETJUL	175.097	3.336
GETKAS	164.354	2.799	GETKAS	159.638	3.366	GETJUL	150.403	2.934	GETJUL	152.687	3.118	GETJUL	161.105	3.117
GETKAS	158.71	3.193	GETKAS	162.286	2.999	GETKAS	159.31	3.019	GETKAS	159.534	3.287	GETKAS	160.328	3.345
GETKAS	160.279	3.072	GETKAS	165.557	2.963	GETKAS	164.553	3.01	GETKAS	157.897	2.74	GETKAS	171.456	2.736
GETMAU	163.054	3.379	GETMAU	158.983	3.203	GETKAS	151.028	3.348	GETKAS	151.139	3.154	GETKAS	154.055	2.786
GETPIF	151.202	3.241	GETQAY	169.07	3.084	GETMAU0	162.705	3.383	GETMAU0	159.719	3.212	GETPIF	159.857	3.459
GEVCIT	170.844	3.073	GEVCUF	166.656	3.373	GETVAD	162.413	2.962	GEVBAO	163.609	3.151	GEVBAO	164.072	3.014
GEVFEU	155.775	3.006	GEVFEU	173.097	2.659	GEVFEU	165.93	2.862	GEVFEU	164.205	2.659	GEVFEU	151.022	2.94
GEVFIY	167.661	3.208	GEVFIY	161.245	2.899	GEVFEU	153.213	3.486	GEVFEU	163.293	2.862	GEVFIY	166.052	3.234
GEVFOE	162.442	2.934	GEVFOE	162.75	2.934	GEVFIY	162.694	2.899	GEVFOE	166.37	3.237	GEVFOE	165.966	3.288
GEVJOK	170.149	2.777	GEVJOK	167.746	3.241	GEVGUK	151.357	2.912	GEVJOK	154.064	3.173	GEVJOK	152.559	3.439
GEVPUS	171.327	2.897	GEVPUS	174.453	3.267	GEVJOK	157.439	2.777	GEVPIG	170.609	3.101	GEVPIG	173.801	3.202
GEWJID	163.89	2.785	GEWJID	151.972	2.876	GEVPUS	168.496	3.049	GEVPUS	167.054	3.069	GEWHUP	158.674	3.204
GEXFIB	163.02	3.093	GEXFUN	165.595	3.243	GEWWUD	175.742	3.014	GEXCOE	155.956	2.715	GEXFIB	173.285	3.077
GEXLUT	156.784	2.99	GEXLUT	177.739	2.924	GEXLIH	154.326	3.334	GEXLIH	160.239	2.981	GEXLON	164.402	3.139
GEYLOL0	160.603	3.213	GEYLOL0	157.4	2.862	GEYLOL	158.048	3.246	GEYLOL	158.099	2.946	GEYLOL	160.651	3.269
GEYLOL0	156.844	2.852	GEYMAA	152.155	3.465	GEYLOL0	158.019	3.083	GEYLOL0	157.978	3.08	GEYLOL0	160.552	3.21
GEYYAO	166.386	2.714	GEYYAO	165.329	3.195	GEYYAO	151.106	3.23	GEYYAO	155.681	3.073	GEYYAO	154.088	2.848
GEYYAO	159.872	2.926	GEYYAO	172.819	3.063	GEYYAO	157.38	3.206	GEYYAO	165.307	3.187	GEYYAO	166.226	3.216
GEYYAO	160.935	3.122	GEYYAO	154.94	3.231	GEYYAO	170.357	3.051	GEYYAO	158.373	3.206	GEYYAO	156.392	3.384
GEYYAO	153.403	2.956	GEYYAO	161.945	3.061	GEYYAO	169.958	3.009	GEYYAO	161.426	3.134	GEYYAO	150.534	3.057
GEZVIS	153.438	3.35	GEZVIS	152.975	2.763	GEYYAO	170.975	3.146	GEYYAO	160.231	3.318	GEZVEO	164.397	3.406
GEZVUE	167.215	3.211	GEZWOZ	160.617	2.807	GEZVOY	165.076	3.094	GEZVOY	155.9	2.768	GEZVUE	157.837	2.932
GIBREQ	155.337	2.936	GIBREQ	167.649	3.052	GEZXAM	159.677	2.768	GIBRAM	158.398	3.462	GIBRAM	154.36	2.854
GIBRUI	151.106	2.855	GIBVES	176.934	3.209	GIBREQ	160.39	3.282	GIBRIU	154.631	3.043	GIBRUI	160.782	2.855
GIBXUO	160.077	3.24	GIBZUQ	161.137	3.011	GIBVES	162.002	3.39	GIBXEY	155.803	3.102	GIBXUO	158.46	3.11
GIBZUQ	156.477	3.054	GIBZUQ	150.54	3.326	GIBZUQ	168.723	2.742	GIBZUQ	160.219	2.734	GIBZUQ	173.146	2.754
GICKAF	171.513	3.305	GICKAF	166.073	3.352	GICBAY	165.754	3.215	GICGEH	153.951	3.435	GICJIM	168.294	3.106
GICKUC	159.13	3.481	GICLAJ	159.469	3.479	GICKAF	159.971	3.059	GICKAF	154.139	3.293	GICKAF	159.026	3.185
GIDVUN	151.896	3.265	GIFDOO	153.781	3.376	GICLIR	173.199	3.407	GICSEU	150.196	3.126	GIDMEM	164.842	2.994
GIFHOS	162.944	3.345	GIFHOS	169.143	3.117	GIFDOO	151.636	3.387	GIFDUU	167.46	2.807	GIFDUU	150.682	3.067
GIFKEM	174.933	2.775	GIFPEU	152.049	3.25	GIFHOS	155.954	3.162	GIFHOS	171.205	3.05	GIFKEM	158.07	3.06
GIFXAV	163.497	2.869	GIFXAV	155.508	2.781	GIFPIY	153.277	3.036	GIFXAV	162.856	3.16	GIFXAV	166.082	2.779
GIFXAV	158.832	3.107	GIFXAV	173.871	2.926	GIFXAV	160.17	3.062	GIFXAV	159.039	2.779	GIFXAV	162.647	3.24
GIFXOJ	167.31	3.025	GIFXOJ	154.788	2.991	GIFXAV	164.478	2.864	GIFXOJ	157.242	2.96	GIFXOJ	169.761	3.054
GIHHIR	159.453	2.953	GIHKIS	150.766	3.299	GIFXOJ	153.449	3.474	GIGGEM	160.534	3.095	GIGGEM	164.192	3.176
GIHKIS	156.121	2.779	GIHKIS	152.586	2.615	GIHKIS	158.248	3.251	GIHKIS	155.597	3.224	GIHKIS	156.971	3.17
GIHKOY	158.614	3.26	GIHKOY	154.199	3.217	GIHKIS	160.807	3.331	GIHKIS	166.392	2.615	GIHKOY	152.205	3.285
GIHKOY	159.319	3.376	GIHKOY	165.288	2.617	GIHKOY	156.845	3.149	GIHKOY	156.397	2.779	GIHKOY	151.335	2.617
GIHKOY0	154.269	3.154	GIHKOY0	156.109	3.243	GIHKOY0	157.704	2.729	GIHKOY0	151.327	3.16	GIHKOY0	156.72	3.078
GIHKOY0	164.659	2.55	GIHLAL	157.379	3.245	GIHKOY0	150.755	3.363	GIHKOY0	150.312	2.55	GIHKOY0	154.303	3.223
GIHLAL	157.155	3.186	GIHLAL	154.237	2.624	GIHLAL	157.521	3.245	GIHLAL	168.732	2.624	GIHLAL	156.071	2.784
GIHMIV	170.884	3.307	GIHPIA	170.744	3.236	GIHLAL	162.183	3.257	GIHMIV	160.51	2.94	GIHMIV	158.05	3.103
GIHWIF	163.891	2.554	GIHYOP	166.69	3.186	GIHRUN	168.541	3.423	GIHRUN	155.316	3.183	GIHVOM	159.947	2.64
GIJGUC	159.063	2.943	GIJGUC	160.046	3.405	GIHZAB	151.132	3.445	GIHZAB	169.971	3.399	GIJGUC	154.761	3.439
GIKWAB	158.044	2.859	GIKWAB	161.819	2.817	GIJHOA	157.698	2.749	GIKGIT	155.294	3.249	GIKGIT	155.438	3.299
GIKZOR	167.946	2.907	GIKZOR	159.808	3.148	GIKWAB	161.236	3.018	GIKWAB	154.202	3.018	GIKXOQ	157.482	3.153
GIKZUX	152.645	3.239	GILBIO	157.688	3.021	GIKZOR	153.014	3.214	GIKZOR	166.222	3.217	GIKZOR	169.958	3.491
GILGUH	161.433	3.328	GILPEZ	158.604	3.342	GILGIV	167.059	3.131	GILGUH	150.761	3.236	GILGUH	161.88	3.406
GILPEZ	151.144	3.446	GILPEZ	156.575	3.056	GILPEZ	163.656	3.342	GILPEZ	150.688	3.329	GILPEZ	158.097	3.003
GILWON	167.708	3.361	GILYOR	161.206	2.776	GILPEZ	157.57	3.182	GILPEZ	152.003	2.839	GILWAD	151.614	2.893
GILZUY	166.629	3.311	GILZUY	153.277	2.703	GILYOR	157.222	3.427	GILYOR	158.732	2.835	GILYOR	163.407	3.15
GIMDIS	161.965	2.948	GIMDIS	152.98	3.099	GIMBEL	174.922	3.054	GIMBEL	154.995	3.121	GIMDIS	154.696	3.498
GIMQEY	171.822	2.703	GIMQEY	155.314	2.952	GIMDIS	162.704	3.277	GIMFAM	156.177	3.244	GIMFUF	150.061	3.106
GIMXEH	159.991	3.155	GIMXUY	167.944	3.162	GIMQIC	162.46	3.134	GIMRUQ	165.779	2.87	GIMRUQ	167.596	2.862
GIMYAF	172.414	3.201	GIMYAF	159.637	2.807	GIMYAF	156.656	2.807	GIMYAF	153.335	2.885	GIMYAF	175.293	3.137
GINDIU	163.134	3.02	GINDIU	172.238	3.074	GIMYAF	159.63	2.885	GINDIU	158.436	2.878	GINDIU	156.781	3.285

GINFAO	155.668	2.774	GINGAO	160.609	3.15	GINFAO	157.419	2.627	GINFAO	158.935	2.951	GINFAO	150.754	3.419
GINHAQ	150.212	2.911	GINHAQ	166.338	2.928	GINHAQ	169.902	2.928	GINHAQ	169.387	2.911	GINHAQ	179.294	2.703
GINHAQ	157.521	3.256	GINHAQ	158.861	3.284	GINHAQ	150.652	3.13	GINHAQ	152.701	3.186	GINHAQ	150.844	2.712
GINLOE01	163.906	2.984	GINLOE01	150.217	3.139	GINHAQ	151.657	2.703	GINLOE01	154.776	3.073	GINLOE01	161.031	3.308
GINLOE01	150.314	3.085	GINMAV	166.122	2.781	GINLOE01	160.528	3.292	GINLOE01	162.806	3.073	GINLOE01	155.148	3.147
GINMOJ	163.046	3.127	GINPAY	163.114	3.162	GINMAV	155.133	2.781	GINMEZ	156.457	3.044	GINMID	160.885	2.814
GIPCEP	162.364	3.091	GIPCEP	165.249	3.261	GINPUR	171.086	3.417	GINPUR	160.451	2.884	GIPCEP	155.629	3.319
GIPCEP	175.882	3.438	GIPNAU	155.237	3.021	GIPCEP	157.552	3.058	GIPCEP	150.325	3.473	GIPCEP	153.006	3.006
GIPNAU	153.686	3.417	GIPQEE	161.966	3.471	GIPNAU	161.124	2.855	GIPNAU	159.303	3.021	GIPNAU	167.241	3.405
GIPSEF	152.339	3.008	GIPSEF	159.583	3.36	GIPRIG	163.28	3.177	GIPRIG	156.684	3.396	GIPSEF	160.752	2.86
GIPSUU	153.861	2.698	GIPSUU	164.528	3.279	GIPSUU	152.56	3.369	GIPSUU	150.257	3.122	GIPSUU	150.416	3.054
GIPVUA	164.93	3.484	GIPVUA	150.024	3.167	GIPSUU	161.053	3.358	GIPSUU	170.387	2.983	GIPSUU	151.168	3.344
GIPVUA	157.851	2.862	GIPVUA	156.618	2.853	GIPVUA	172.856	3.025	GIPVUA	159.927	2.949	GIPVUA	151.019	3.094
GIPVUA	153.343	2.937	GIPWEL	160.315	2.879	GIPVUA	152.392	3.067	GIPVUA	156.003	2.845	GIPVUA	157.785	2.826
GIPWEL	161.541	3.019	GIPWEL	158.434	3.155	GIPWEL	151.569	3.047	GIPWEL	170.169	2.805	GIPWEL	155.018	2.825
GIPWEL	167.554	3.189	GIPWEL	162.192	3.231	GIPWEL	165.975	3.151	GIPWEL	165.452	3.113	GIPWEL	156.147	2.858
GIPXAG	154.674	3.071	GIPXAG	153.511	3.448	GIPXAG	163.828	2.898	GIPXAG	172.204	2.71	GIPXAG	152.278	2.943
GIQCIT	157.772	3.482	GIQCOZ	163.355	3.254	GIPZOY	151.437	3.076	GIPZOY	152.829	2.799	GIQBIV	170.8	3.426
GIQFER	158.328	3.242	GIQFER	168.583	3.481	GIQCUF	165.324	3.421	GIQDAM	150.924	2.703	GIQFER	157.675	3.376
GIQFER	159.807	3.062	GIQFER	157.269	3.15	GIQFER	161.684	3.176	GIQFER	170.479	3.402	GIQFER	161.027	3.488
GIQGUM	150.492	2.926	GIQHEU	162.218	3.11	GIQFER	151.404	3.182	GIQGOG	171.48	3.393	GIQGUM	179.531	3.352
GIQJEW	153.336	3.445	GIQJEY	164.491	3.224	GIQHUM	166.95	2.74	GIQHUM	153.965	3.308	GIQJEW	154.5	3.255
GIQJIB01	152.242	3.173	GIQJIB01	155.106	3.365	GIQJEY	166.382	3.165	GIQJIB01	155.128	3.106	GIQJIB01	151.317	2.701
GIQJID	156.771	2.845	GIQJOH01	152.243	3.16	GIQJID	158.469	3.222	GIQJID	155.494	3.378	GIQJID	163.104	2.845
GIQMUO	159.233	3.395	GIQMUO	160.476	3.467	GIQJOH01	155.232	3.338	GIQJOH01	154.89	3.094	GIQJOH01	151.426	2.698
GIQPUS	176.156	3.383	GIQPUS	157.251	2.938	GIQMUO	154.77	2.998	GIQPON	151.968	2.984	GIQPON	154.917	3.305
GIRFUM	166.723	3.203	GIRMAV	167.131	2.79	GIQQII	173.017	3.447	GIQVIL	172.963	3.122	GIQYOX	155.514	3.04
GIRMAV	152.835	3.232	GIRMIH	154.033	2.816	GIRMAV	175.038	3.064	GIRMAV	165.557	2.797	GIRMAV	156.09	2.77
GIRSAE	179.108	2.818	GIRSAE	150.594	2.608	GIRQIL	158.268	3.316	GIRQIL	163.767	3.316	GIRSAE	162.946	2.729
GIRSAE	155.301	2.608	GIRSAE	176.658	3.039	GIRSAE	164.431	2.841	GIRSAE	161.698	2.912	GIRSAE	161.548	3.052
GIRSAE	159.117	3.031	GIRSAE	166.15	3.162	GIRSAE	172.076	3.137	GIRSAE	176.549	3.145	GIRSAE	153.455	3.011
GIRSAE	169.421	3.418	GIRSAE	151.608	2.956	GIRSAE	174.508	2.817	GIRSAE	157.888	3.298	GIRSAE	158.382	3.326
GIRSOS	176.575	3.28	GIRVUY01	161.438	3.012	GIRSAE	166.015	3.497	GIRSAE	163.548	3.25	GIRSAE	158.846	2.764
GIRXAJ	153.327	3.28	GIRXEN	154.922	3.276	GIRVUY01	151.089	3.012	GIRVUY01	160.992	3.101	GIRXAJ	151.987	3.374
GIRZUE	164.875	3.12	GIRZUE	171.703	3.029	GIRZIS	157.999	3.339	GIRZIS	155.644	3.293	GIRZUE	152.558	3.045
GISRIL	167.272	3.292	GITCEU	154.151	3.281	GISPOQ	157.157	3.339	GISQEH	165.308	2.954	GISRIL	155.308	3.449
GITCOC	157.578	2.91	GITCOC	153.246	3.403	GITCIY	166.575	3.387	GITCOC	162.895	2.981	GITCOC	156.945	3.282
GITPOO	160.592	2.995	GITPOO	151.979	3.24	GITCOC	158.408	3.195	GITLUS	150.031	3.47	GITLUS	153.774	3.279
GITPOO	162.778	2.879	GITPOO	163.295	2.936	GITPOO	151.934	3.214	GITPOO	167.845	3.064	GITPOO	152.195	2.984
GITYOB	161.788	3.49	GITYOB	164.676	2.828	GITPOO	171.339	3.468	GITPOO	169.978	3.079	GITPOO	153.715	3.035
GITZIW	163.679	3.296	GITZIW	161.554	3.137	GITYOB	160.635	3.297	GITZIW	155.648	3.101	GITZIW	164.216	2.832
GIVBAS	152.639	3.461	GIVBAS	167.464	3.393	GITZIW	165.739	3.023	GITZIW	163.581	2.832	GITZIW	158.75	2.759
GIVCAT	168.286	3.349	GIVNUU	172.343	3.077	GIVBAS	167.158	3.393	GIVBAS	160.685	2.899	GIVBIZ	167.001	3.27
GIVSOV	151.209	3.009	GIWHID	160.292	3.027	GIVNUU	165.033	3.441	GIVRUA01	167.239	3.332	GIVSOV	177.71	3.366
GIWHID	166.937	2.927	GIWHID01	164.528	2.843	GIWHID	169.672	2.927	GIWHID	170.013	3.482	GIWHID	155.449	3.027
GIWJAX	150.787	3.224	GIWMOS	163.169	3.102	GIWHID01	158.335	2.806	GIWHID01	165.974	3.4	GIWHID01	171.138	2.843
GIWTIT	168.672	3.18	GIWTIT	159.664	3.18	GIWMOS	174.865	2.92	GIWPIO	171.031	3.375	GIWPIO	163.69	3.127
GIWWOY	154.406	2.863	GIWXAL	174.083	2.783	GIWWOY	169.972	2.863	GIWWOY	176.183	2.909	GIWWOY	169.161	2.832
GIWXAL	151.91	3.048	GIWXAL	160.841	3.098	GIWXAL	164.201	2.923	GIWXAL	175.378	3.33	GIWXAL	173.083	3.042
GIWXAL01	157.641	3.134	GIWXAL01	155.653	3.497	GIWXAL	156.67	2.919	GIWXAL01	156.701	2.906	GIWXAL01	168.227	2.998
GIWXAL02	152.422	3.077	GIWXAL02	161.065	3.11	GIWXAL01	160.559	3.153	GIWXAL01	151.574	2.995	GIWXAL01	157.993	3.208
GIWXAL02	176.107	3.327	GIWXAL02	164.778	2.957	GIWXAL02	172.583	3.068	GIWXAL02	174.387	2.804	GIWXAL02	157.224	2.944
GIXGEZ	170.451	3.057	GIXGEZ	172.36	3.356	GIXFIE	159.66	3.171	GIXFUQ	158.199	2.951	GIXFUQ	165.775	2.887
GIXGUP01	173.519	3.17	GIXGUP01	170.034	2.654	GIXGEZ	162.93	3.057	GIXGUP	152.493	2.699	GIXGUP01	170.474	2.939
GIXHUQ	159.151	2.996	GIXHUQ	163.438	3.127	GIXHUQ	151.442	3.004	GIXHUQ	154.958	2.738	GIXHUQ	175.802	3.495
GIXKOR	163.629	2.866	GIXKOR	158.651	2.68	GIXHUQ	155.203	3.236	GIXKEH	152.014	3.182	GIXKOR	154.846	2.947
GIXLEI	173.387	2.921	GIXLEI	158.995	2.965	GIXLEI	171.041	2.866	GIXLEI	161.085	2.985	GIXLEI	158.511	3.048
GIXXAQ	150.754	3.059	GIXXAQ	153.693	3.212	GIXLEI	157.553	3.121	GIXQIR	173.719	2.698	GIXSIR	159.333	3.325
GIXXAQ	157.618	3.238	GIXXAQ	175.406	3.254	GIXXAQ	155.371	3.381	GIXXAQ	157.197	3.279	GIXXAQ	164.836	3.279
GIYBOF	160.372	2.91	GIYBOF	156.488	3.142	GIXYET	171.599	2.662	GIXZEV	156.132	3.27	GIXZIW	158.458	3.367
GIYBOF01	151.102	3.489	GIYBOF01	155.437	3.158	GIYBOF	172.17	3.166	GIYBOF01	158.058	2.695	GIYBOF01	172.68	3.254
GIYDIC01	174.069	3.495	GIYJAC	159.908	3.145	GIYBOF01	156.971	3.014	GIYDIC	164.941	3.143	GIYDIC	151.96	2.987
GIYLUZ01	152.16	2.857	GIYLUZ02	153.046	2.872	GIYKIJ	163.828	3.126	GIYKIJ	151.717	3.456	GIYLUZ	152.142	2.858
GIYZAR	158.4	3.089	GIZCOH	167.661	3.132	GIYQEM	158.381	3.033	GIYVIU	173.481	2.923	GIYZAR	161.672	3.089
GIZQIP	169.566	3.153	GIZYOE	169.842	3.357	GIZDEA	152.939	3.1	GIZDEA	153.606	2.761	GIZDEA	174.832	2.938
GIZYOE	174.827	2.71	GIZYOE01	165.522	3.481	GIZYOE	150.707	2.56	GIZYOE	165.98	3.498	GIZYOE	164.183	3.368
GIZYOE01	171.61	3.314	GIZYUK	175.509	2.908	GIZYOE01	177.062	2.672	GIZYOE01	152.331	2.527	GIZYOE01	162.73	3.364
GIZZAR	152.518	3.479	GIZZAR	170.188	2.952	GIZYUK	161.45	2.764	GIZYUK	158.579	2.892	GIZZAR	174.218	3.069
GIZZAR	170.046	3.182	GIZZAR	176.594	3.194	GIZZAR	176.925	3.239	GIZZAR	174.086	3.13	GIZZAR	150.906	2.981
GIZZEV	163.868	3.075	GIZZEV	173.091	3.188	GIZZEV	151.34	3.426	GIZZEV	169.807	3.048	GIZZEV	163.963	3.088
GIZZIZ	157.909	3.171	GOBBUW	152.127	2.905	GIZZIZ	162.517	3.371	GIZZIZ	152.587	3.198	GIZZIZ	161.569	3.481
GOBDEG	150.274	3.441	GOBFAF	157.182	3.38	GOBCIL	155.306	2.814	GOBDEG	160.203	2.914	GOBDEG	163.779	2.96
GOBHOV	157.253	2.976	GOBHOV	161.727	2.926	GOBFAF	175.271	2.917	GOBFAF	163.945	3.243	GOBFAF	158.428	2.944
GOCGUC	160.681	2.913	GOCGUC	151.781	3.222	GOBHOV	167.246	3.061	GOBHOV	174.455	3.061	GOCGOW	163.76	3.419
GOCPIA	164.961	3.196	GOCXEC	174.141	3.139	GOCMET	153.203	2.872	GOCMUJ	162.14	3.306	GOCNOE	170.076	2.783
GODHAM	152.55	2.749	GODHAM	170.686	3.172	GOCXEC	159.708	2.995	GOCXEC	150.478	2.777	GOCXEC	162.075	3.488
GODHOZ	159.405	2.945	GODHOZ	158.403	3.45	GODHAM	163.803	3.15	GODHAM	165.216	3.496	GODHAM	167.305	3.233
GODMEV	160.564	2.865	GODMEV	168.534	2.865	GODJIW	152.322	2.752	GODJIW	151.868	2.753	GODJIW	169.482	2.752
GOFCIP	157.779	2.904	GOFCIP	165.047	3.46	GODMIV	151.857	3.346	GOFBUB	163.869	2.788	GOFCIP	172.913	3.108

GOFHER	167.086	2.795	GOFHER	154.345	3.299	GOFCIP	158.161	3.333	GOFDEM	164.158	3.14	GOFHER	153.466	3.344
GOFHER	157.737	3.192	GOFPIE	176.388	3.273	GOFHER	152.283	2.985	GOFHER	151.145	3.28	GOFHER	159.7	3.299
GOFVEF	150.007	3.3	GOGJER	159.617	3.21	GOFPOK	174.349	3.241	GOFVEF	159.318	3.015	GOFVEF	177.267	2.837
GOGJER	158.965	3.164	GOGJER	155.065	3.336	GOGJER	173.78	3.286	GOGJER	173.336	3.297	GOGJER	155.495	3.365
GOGJER	156.358	3.099	GOGJER	165.365	3.118	GOGJER	150.073	3.389	GOGJER	165.946	3.121	GOGJER	176.661	3.274
GOGZAF	164.691	2.984	GOGZAF	164.886	3.042	GOGJER	156.436	3.353	GOGJER	168.054	3.133	GOGYAE	157.382	2.912
GOGZAF	157.192	2.845	GOGZAF	175.761	2.739	GOGZAF	165.505	2.739	GOGZAF	151.473	2.98	GOGZAF	165.19	2.953
GOHDOZ	160.849	2.835	GOHDOZ	163.311	2.903	GOGZAF	158.997	2.711	GOGZAF	157.292	3.212	GOHBAI	163.251	2.903
GOHDOZ	164.704	3.034	GOHDOZ	163.113	2.811	GOHDOZ	170.301	2.942	GOHDOZ	168.529	3.043	GOHDOZ	164.25	2.954
GOHQOM	160.649	3.345	GOHQON	157.818	2.62	GOHDOZ	162.419	2.887	GOHFOB	158.367	3.245	GOHJEU	169.038	3.064
GOHZUZ	157.997	2.803	GOHZUZ	170.844	3.044	GOHQON	161.092	2.665	GOHZUZ	160.568	2.803	GOHZUZ	157.924	3
GOJSIK	158.09	3.384	GOKBER	164.244	3.091	GOJQII	161.666	3.257	GOJSIK	166.641	3.384	GOJSIK	167.985	2.743
GOKMAX	162.103	2.99	GOKMAX	166.516	3.341	GOKBER	160.804	3.392	GOKHIB	150.276	2.986	GOKHIB	158.682	2.989
GOKMEB	163.482	3.325	GOKSUX	157.49	3.291	GOKMEB	150.273	2.907	GOKMEB	152.052	3.015	GOKMEB	155.69	2.877
GOKSUX	168.209	2.871	GOKSUX	151.417	2.718	GOKSUX	164.75	3.491	GOKSUX	175.127	2.998	GOKSUX	170.77	3.404
GOKYEL	176.033	2.918	GOKYEL	173.137	3.284	GOKTOR	163.035	2.8	GOKVUZ	159.171	3.324	GOKVUZ	162.737	3.324
GOLKAT	166.716	3.126	GOLKAT	159.016	3.257	GOKYEL	157.444	3.376	GOLKAT	152.627	3.155	GOLKAT	155.999	2.895
GOLKAT	154.56	3.208	GOLKAT	159.858	3.492	GOLKAT	173.979	3.285	GOLKAT	173.481	3.379	GOLKAT	158.501	3.111
GOLPOO	153.187	3.445	GOLXIR	157.251	3.492	GOLKAT	160.299	2.71	GOLKAT	151.891	3.367	GOLPOO	158.509	3.042
GOMROQ	155.206	3.069	GOMROQ	162.28	3.036	GOLXUD	150.294	2.871	GOMHUL	160.844	3.42	GOMRAB	166.176	3.312
GOMROQ	162.593	2.849	GOMROQ	151.058	3.08	GOMROQ	165.385	2.845	GOMROQ	166.999	3.439	GOMROQ	175.082	3.091
GOMSIO0	170.077	2.8	GOMSIO0	170.304	2.821	GOMROQ	150.153	3.145	GOMSIO	169.523	2.817	GOMSIO	169.364	2.838
GOMSIO0	171.469	2.785	GOMSIO0	171.484	2.748	GOMSIO0	170.946	2.784	GOMSIO0	170.729	2.804	GOMSIO0	171.573	2.764
GOMVEJ	169.602	3.498	GONJAX	150.279	2.963	GOMSIO0	171.664	2.775	GOMSIO0	171.905	2.736	GOMSIO0	171.814	2.756
GONJEA	157.345	3.467	GONJEA	152.872	3.028	GONJAX	160.027	2.622	GONJAX	152.803	3.235	GONJEA	170.717	3.162
GONTAE	154.872	3.124	GONTEK	156.042	3.103	GONJEA	165.324	3.161	GONNII	171.522	3	GONTAE	150.041	2.975
GOPBEW	157.072	2.972	GOPQOU	158.946	3.36	GONTEK	161.966	3.478	GONXEQ	167.926	3.105	GONXEQ	169.82	3.204
GOPWAJ	168.101	2.644	GOPWAJ	170.551	3.478	GOPVAM	161.103	3.306	GOPWAJ	165.443	3.36	GOPWAJ	174.357	3.36
GOPWEN	172.49	2.848	GOPWEN	161.352	3.283	GOPWAJ	161.217	3.478	GOPWAJ	150.149	3.329	GOPWEN	157.459	3.124
GOPWEN	157.215	3.126	GOPWEN	161.656	3.369	GOPWEN	154.478	2.884	GOPWEN	166.868	3.149	GOPWEN	175.941	3.273
GOPWIR	151.23	3.264	GOPWIR	156.214	3.053	GOPWEN	162.017	3.186	GOPWIR	166.529	3.052	GOPWIR	153.009	2.95
GOQBOG	152.489	3.055	GOQBOG	163.525	2.834	GOQBOG	160.866	2.834	GOQBOG	174.858	3.456	GOQBOG	153.899	2.964
GOQCOH	162.866	2.987	GOQCOH	166.489	2.987	GOQCOH	172.826	2.737	GOQCOH	152.116	3.42	GOQCOH	158.159	2.694
GOQQEM	167.766	2.925	GOQQEM	171.619	3.007	GOQCOH	157.115	2.827	GOQNAE	160.655	3.313	GOQNAE0	160.526	3.23
GOQQIQ	160.879	3.347	GOQQIQ	150.38	3.295	GOQQEM	150.212	2.924	GOQQIQ	158.895	2.999	GOQQIQ	158.886	3.248
GOQXIW	159.104	3.065	GOQXOC	159.187	3.073	GOQQIQ	156.406	3.203	GOQQIQ	152.524	3.025	GOQXES	159.037	3.069
GORKAZ	157.049	2.737	GORPUA	162.601	2.986	GORBOE	156.766	3.192	GORBOE	157.501	3.242	GORKAZ	166.918	3.25
GORSAK	162.585	3.032	GORSAK	170.198	2.823	GORSAK	160.88	2.795	GORSAK	172.028	2.795	GORSAK	157.418	2.823
GORSEN	162.862	3.463	GORSIS	168.131	3.435	GORSEN	158.965	3.224	GORSEN	170.284	3.212	GORSEN	167.322	3.142
GOSCIA	153.87	3.367	GOSHAX	152.097	3.399	GORSIS	166.032	3.322	GORWUI	154.335	2.751	GORXAP	156.595	3.417
GOSHIF	165.097	2.859	GOSHON	162.9	3.119	GOSHAX	155.704	3.384	GOSHAX	176.895	3.371	GOSHIF	162.753	2.859
GOSQOY	157.881	3.32	GOSQOY	164.814	2.838	GOSJOP	152.967	3.201	GOSMUZ	151.484	3.477	GOSQOY	158.496	3.383
GOSQOY	153.288	3.256	GOSQOY	161.706	3.381	GOSQOY	160.065	3.437	GOSQOY	158.608	2.842	GOSQOY	164.235	2.842
GOSSOY	160.002	3.065	GOSTAL	153.764	3.245	GOSQOY	170.191	3.102	GOSQOY	151.379	3.414	GOSQOY	164.421	3.053
GOTKAB	170.544	2.893	GOTKEF	154.2	3.274	GOSVER	159.172	3.359	GOSVOB	168.929	3.139	GOTKAB	157.416	2.893
GOTQEL0	151.516	2.743	GOTQEL0	159.922	2.939	GOTQEL0	166.743	3.238	GOTQEL0	167.185	3.291	GOTQEL0	167.474	3.306
GOTQUE	155.653	3.21	GOTSUD	170.544	3.451	GOTQEL0	170.763	3.199	GOTQEL0	155.763	3.135	GOTQEL0	161.375	3.269
GOVFOQ	150.071	3.258	GOVFOQ	158.079	3.048	GOTSUD	176.914	3.152	GOTSUD	172.542	3.337	GOVFEG	154.85	3.252
GOVMEN	158.032	3.465	GOVMEN	157.911	3.213	GOVFOQ	165.818	3.258	GOVMEN	156.772	3.059	GOVMEN	168.583	3.017
GOVMIR	154.157	3.258	GOVMIR	150.846	3.053	GOVMIR	167.588	3.014	GOVMIR	152.125	3.053	GOVMIR	157.249	3.487
GOVMOX	151.487	3.159	GOVMOX	157.07	3.226	GOVMOX	163.583	3.061	GOVMOX	151.36	3.284	GOVMOX	150.78	3.159
GOVMUD	159.36	3.173	GOVPEO	165.118	3.372	GOVMUD	159.504	3.113	GOVMUD	157.91	3.405	GOVMUD	167.149	3.016
GOVPEQ	168.397	3.252	GOVREQ	152.767	2.962	GOVPEO	169.217	2.816	GOVPEQ	153.599	3.064	GOVPEQ	157.309	3.252
GOVVAS	169.593	3.144	GOWBOL	163.451	3.479	GOVREQ	168.948	3.2	GOVREQ	151.059	3.162	GOVREQ	161.03	2.825
GOWPIR	156.248	3.487	GOXBII	150.918	3.096	GOWPIR	154.251	3.165	GOWPIR	157.044	3.449	GOWPIR	155.739	3.226
GOXCUU	157.038	3.22	GOXCUU	156.722	3.307	GOXBII	156.932	2.983	GOXBII	174.456	2.858	GOXCUU	153.056	3.045
GOYDOR	159.611	3.198	GOYDOR	156.174	2.892	GOXCUU	152.547	3.124	GOXCUU	158.707	3.068	GOXKEM	160.702	3.372
GOZDOS	153.704	3.467	GOZGIO	156.275	3.093	GOYWEY	152.953	3.282	GOYWEY	157.193	2.767	GOZDOS	157.087	3.155
GOZTEY	159.146	3.044	GOZTEY	163.137	3.109	GOZGIO0	156.275	3.093	GOZTEY	176.597	2.854	GOZTEY	152.337	3.31
GOZWAW	160.963	2.618	GOZWIE	158.501	3.173	GOZTEY	155.313	2.708	GOZTEY	154.613	3.115	GOZTEY	154.368	3.143
GOZWIE0	168.934	2.935	GOZWIE0	151.105	2.923	GOZWIE	168.781	2.937	GOZWIE	151.957	2.928	GOZWIE0	158.639	3.169
GOZWIE0	152.015	2.92	GOZWIE0	168.526	2.932	GOZWIE0	158.434	3.164	GOZWIE0	169.001	2.928	GOZWIE0	151.367	2.921
GUBFER	151.146	3.361	GUBFOB	160.619	2.94	GOZWIE0	158.405	3.167	GUBBOW	167.063	3.378	GUBFER	162.387	2.948
GUBHAQ	178.837	2.757	GUBHAQ	156.532	3.09	GUBGUJ	170.49	3.002	GUBGUJ	154.788	3.007	GUBGUJ	176.774	2.915
GUBHAQ	152.481	2.903	GUBHAQ	151.049	2.716	GUBHAQ	156.643	2.902	GUBHAQ	158.925	3.09	GUBHAQ	165.632	2.952
GUBHAQ	155.348	2.716	GUBHEU	157.237	3.243	GUBHAQ	151.154	3.104	GUBHAQ	160.013	3.082	GUBHAQ	153.981	3.104
GUBHIY	162.251	3.082	GUBHIY	161.672	2.711	GUBHEU	160.3	3.243	GUBHEU	157.169	3.024	GUBHIY	168.956	2.811
GUBLIC	167.471	3.044	GUBMUM	154.937	3.237	GUBHOE	172.854	2.908	GUBHOE	168.425	3.287	GUBKEX	163.922	3.143
GUBVOQ	175.571	3.49	GUCBEP	162.531	3.39	GUBNEX	166.734	3.179	GUBSUT	162.89	3.242	GUBVOQ	150.104	3.321
GUCNAT	169.391	2.968	GUCNAT	164.518	3.473	GUCBEP	157.797	3.072	GUCBEP	155.869	3.08	GUCBIR	164.826	2.814
GUCNAT	168.027	3.166	GUCNAT	172.373	3.432	GUCNAT	167.311	3.317	GUCNAT	157.463	3.053	GUCNAT	151.898	3.252
GUCNAT	156.022	3.109	GUCNAT	166.062	2.914	GUCNAT	175.413	3.392	GUCNAT	160.721	2.844	GUCNAT	154.771	2.839
GUCNEX	164.562	3.015	GUCNEX	164.15	2.862	GUCNAT	166.661	2.912	GUCNEX	151.041	3.114	GUCNEX	159.459	3.25
GUCNEX	164.1	3.029	GUCNEX	158.387	3.427	GUCNEX	167.795	2.829	GUCNEX	162.391	3.204	GUCNEX	169.633	3.271
GUCNEX	163.722	3.095	GUCNEX	152.311	3.016	GUCNEX	172.067	3.205	GUCNEX	151.239	3.156	GUCNEX	168.611	3.223
GUCPIH	174.147	3.179	GUCPIH	170.933	2.925	GUCNOL	167.992	3.146	GUCNOL	166.718	3.054	GUCPIH	159.745	2.937
GUCQIG	153.573	3.199	GUCTEG	155.998	2.804	GUCPIH	156.588	2.767	GUCPIH	160.805	3.366	GUCPUP	167.152	3.446
GUCWUW	150.894	2.825	GUCWUW	164.62	2.905	GUCTEG	159.17	3.417	GUCTEG	163.865	3.195	GUCWUW	157.143	2.151
GUCXAD	165.799	3.445	GUCXUA	150.229	3.244	GUCWUW	151.809	3.018	GUCXAD	151.679	2.275	GUCXAD	171.677	3.055

GUCYAH	159.762	2.874	GUCYIM	158.164	3.158	GUCXUA	162.524	3.028	GUCYAE	164.605	3.186	GUCYAE	167.282	3.089
GUDCAM	158.199	3.187	GUDCAM	172.994	3.419	GUCYIM	169.984	3.368	GUCYIM	153.561	2.953	GUCYIM	156.031	3.002
GUFKID	164.61	3.239	GUFKOJ	160.378	3.151	GUDCAM	159.35	3.45	GUDCUG	164.168	3.176	GUDWAD	164.422	3.462
GUFMUS	154.638	2.865	GUFMUS	158.758	3.496	GUFMAV	159.22	2.879	GUFMUS	167.46	2.939	GUFMUS	167.886	3.482
GUFSAE	152.913	3.422	GUFVOV	163.687	2.603	GUFMUS	150.311	3.438	GUFQAA	150.673	3.136	GUFQAA	171.004	3.236
GUFZIR	153.516	3.002	GUFZIR	150.958	3.112	GUFWOU	151.662	3.121	GUFZIR	167.193	3.254	GUFZIR	153.057	3.357
GUFZIR	173.479	2.773	GUFZIR	170.642	3.062	GUFZIR	162.092	2.769	GUFZIR	169.252	2.719	GUFZIR	160.252	2.719
GUFZIR	151.897	3.351	GUFZUD	152.35	3.353	GUFZIR	157.823	2.916	GUFZIR	157.827	2.943	GUFZIR	166.827	3.429
GUFZUD	157.325	3.424	GUFZUD	158.432	3.059	GUFZUD	168.227	2.979	GUFZUD	169.073	2.973	GUFZUD	157.848	3.33
GUFZUD	171.366	3.202	GUGHEX	168.402	2.9	GUFZUD	160.673	2.981	GUFZUD	166.299	2.836	GUFZUD	151.596	3.126
GUGHOH	167.789	2.905	GUGHOH	167.951	2.894	GUGHEX	171.161	2.912	GUGHOH	170.807	2.887	GUGHOH	169.335	2.917
GUGRIM	176.587	2.994	GUGRUY	151.507	3.442	GUGNUS	154.149	3.079	GUGQAD	158.405	3.072	GUGQAD	156.513	2.944
GUGSEJ	150.128	3.406	GUGSEJ	151.654	3.406	GUGRUY	153.502	3.322	GUGSAF	155.854	3.161	GUGSAF	157.792	3.167
GUGYOW	161.563	3.078	GUGYUC	170.483	3.192	GUGYIQ	169.99	3.271	GUGYIQ	161.813	3.129	GUGYOW	170.721	3.24
GUHTAH	161.961	3.491	GUHTAH	163.527	2.895	GUGYUC	162.253	3.108	GUGZUF	161.123	3.483	GUHCAP	158.09	3.429
GUHYOZ	170.558	2.53	GUHYOZ	166.924	2.53	GUHYIV	170.518	3.445	GUHYIV	151.22	3.178	GUHYIV	150.581	2.871
GUJLED	151.612	3.361	GUJLOQ	157.066	3.083	GUHYUH	160.832	2.784	GUHYUH	165.799	2.809	GUJDAU	153.526	2.964
GUJXUG	155.603	3.178	GUKDUO	151.321	3.282	GUJMOR	172.367	3.395	GUJMOR	160.936	3.333	GUJXUG	158.268	3.27
GUKSUC	155.977	2.96	GUKSUC	160.626	2.896	GUKKUW	160.222	2.935	GUKKUW	172.068	3.424	GUKQEI	162.012	2.819
GUKTIR	166.37	3.088	GUKTIR	163.682	3.153	GUKTEN	173.135	3.146	GUKTEN	153.469	2.887	GUKTEN	153.218	2.887
GULGOL	154.966	2.903	GULHAY	162.371	3.012	GUKTIR	151.797	2.968	GUKTIR	173.158	3.345	GULGOL	165.662	2.992
GULRAG	176.084	2.895	GULSIR	163.058	3.027	GULHAY	166.031	2.946	GULHEC	170.968	2.988	GULHEC	170.279	2.83
GULSOX	155.576	3.268	GULTAK	169.101	3.208	GULSOX	164.309	2.878	GULSOX	150.509	3.313	GULSOX	161.744	2.82
GULXIY	157.708	3.47	GULXIY	152.427	2.739	GULTAK	158.139	3.3	GULVES	172.098	3.296	GULWOC	164.156	3.083
GUMDUQ	159.566	3.133	GUMDUQ	151.576	3.456	GULXIY	178.249	3.01	GULXUG	164.551	3.245	GULXUG	163.427	3.252
GUMHAZ	163.782	3.394	GUMHAZ	159.68	3.24	GUMDUQ	162.487	2.946	GUMDUQ	150.865	3.167	GUMHAZ	157.035	3.241
GUMWOB	155.833	3.132	GUMWOB	166.599	2.629	GUMNUA	170.823	2.768	GUMPAF	164.198	3.406	GUMWOB	162.903	2.658
GUMWOB	158.932	2.872	GUMZIB	155.845	2.969	GUMWOB	153.955	3.346	GUMWOB	157.645	3.428	GUMWOB	153.362	3.329
GUNHON	166.853	2.823	GUNHON	152.736	2.998	GUNFEC	159.305	3.409	GUNHON	155.432	3.215	GUNHON	171.87	3.175
GUNHON	155.601	3.206	GUNHON	150.969	3.444	GUNHON	166.611	2.816	GUNHON	171.151	3.009	GUNHON	153.219	2.818
GUNPEN	154.825	3.338	GUNPEN	169.379	3.462	GUNJAB	156.044	2.735	GUNJAB	150.069	3.056	GUNKEI	156.792	2.965
GUNVUJ	159.081	3.096	GUNVUJ	173.203	2.884	GUNPIR	162.076	3.282	GUNTEO	154.738	2.931	GUNTUI	150.01	3.435
GUNVUJ	161.339	3.155	GUNVUJ	157.438	3.012	GUNVUJ	170.207	3.176	GUNVUJ	175.236	2.874	GUNVUJ	154.439	2.808
GUPNIR	163.376	2.984	GUPNIR	150.326	3.38	GUNVUJ	161.326	2.727	GUPCEC	179.904	3.176	GUPNIR	168.094	3.181
GUQBUQ	159.39	2.936	GUQCAX	173.058	3.15	GUPWIA	171.544	3.25	GUQBEA	167.248	3.27	GUQBEA	169.485	2.857
GUQMAH	163.053	3.451	GUQNOZ	166.809	3.15	GUQLOV	163.15	2.818	GUQLOV	162.424	3.223	GUQLOV	162.857	3.372
GUQNOZ	163.789	3.112	GUQWIA	161.924	3.092	GUQNOZ	172.859	2.95	GUQNOZ	164.016	3.083	GUQNOZ	156.516	3.225
GURLOX	171.782	3.212	GURNUD	156.267	3.429	GURDEF	155.37	3.275	GURDEF	150.271	3.275	GURLOX	157.443	3.202
GURPEQ	163.46	3.149	GURPEQ	169.545	3.183	GURNUD	157.151	2.863	GURNUD	156.294	3.307	GURPEQ	156.776	3.153
GURPEQ	158.475	2.876	GURPEQ	164.585	3.191	GURPEQ	150.838	2.858	GURPEQ	150.088	3.194	GURPEQ	161.062	3.428
GURPUH	152.686	3.192	GURPUH	158.765	3.358	GURPEQ	168.059	2.552	GURPUH	161.635	3.273	GURPUH	160.423	3.44
GURZUS	154.053	3.088	GUSCEE	161.174	3.384	GURZUS	154.34	3.091	GURZUS	157.596	3.312	GURZUS	154.406	3.441
GUSHEK	157.557	3.205	GUSHEK	171.449	3.078	GUSCEE	170.282	3.133	GUSCEE	151.576	3.003	GUSHEK	160.678	3.145
GUSKOU	160.099	3.18	GUSNOA	150.242	3.417	GUSJEK	165.758	3.189	GUSKEK	157.133	2.975	GUSKIO	160.368	3.177
GUSTOE	164.291	3.079	GUSVAR	155.316	2.84	GUSQOD	164.083	3.385	GUSRAN	158.674	3.199	GUSRAN	159.138	3.309
GUSVIC	159.48	3.209	GUSVIC	166.409	2.935	GUSVEZ	157.324	3.336	GUSVIA	159.822	3.38	GUSVIA	161.294	3.348
GUSVOG	163.967	2.984	GUSVOG	159.139	3.125	GUSVIC	164.762	3.011	GUSVIC	159.386	3.393	GUSVOG	163.812	3.005
GUSYUR	152.252	3.311	GUSYUR	157.754	3.465	GUSYUR	169.829	2.982	GUSYUR	172.753	3.199	GUSYUR	160.196	3.1
GUSYUR	154.724	3.455	GUSYUR	152.435	3.265	GUSYUR	161.244	3.465	GUSYUR	162.711	2.774	GUSYUR	150.201	3.453
GUSZAY	157.085	2.919	GUSZAY	153.112	3.164	GUSYUR	163.429	2.935	GUSZAY	154.704	3.341	GUSZAY	151.66	3.046
GUSZEC	158.227	2.961	GUSZEC	151.95	3.241	GUSZEC	160.638	3.234	GUSZEC	160.898	3.405	GUSZEC	150.782	3.367
GUSZEC	155.775	3.264	GUSZEC	169.081	3.112	GUSZEC	158.265	3.333	GUSZEC	155.383	3.112	GUSZEC	158.078	2.845
GUTKIR	155.281	3.073	GUTLAL	167.086	2.98	GUSZEC	168.528	2.876	GUSZEC	171.7	3.467	GUTFAG	158.538	3.119
GUTLAL	152.069	2.879	GUTLAL	160.688	3.168	GUTLAL	155.483	3.077	GUTLAL	165.298	2.994	GUTLAL	155.574	3.073
GUTLEQ	161.691	3.029	GUTLEQ	167.363	2.893	GUTLAL	156.044	3.345	GUTLAL	161.122	3.177	GUTLAL	155.05	3.345
GUTLIU	172.428	3.048	GUTLOZ	168.595	3.26	GUTLEQ	173.857	2.893	GUTLIT	157.39	2.799	GUTLIT	156.883	2.777
GUTNER	153.425	3.049	GUTPUJ	164.084	3.09	GUTLOZ	170.299	3.26	GUTLOZ	155.445	3.361	GUTLOZ	154.57	3.378
GUTQOB	178.027	3.224	GUTQOE	158.335	3.205	GUTQEU	171.15	3.076	GUTQIZ	159.172	3.19	GUTQOB	172.105	3.224
GUTRUJ	162.448	2.726	GUTRUJ	150.292	3.251	GUTQUH	171.544	3.415	GUTQUH	176.496	3.415	GUTRUJ	153.37	3.016
GUTXOK	168.01	2.668	GUTXOK	153.476	2.94	GUTSAT	162.146	2.859	GUTXOK	167.863	3.437	GUTXOK	152.742	2.668
GUVDOU	175.527	2.737	GUVFAI	154.43	2.987	GUTXOK	166.81	3.437	GUVDIO	162.307	3.427	GUVDIO	158.153	3.427
GUVGAH	167.73	3.358	GUVGOV	163.706	2.899	GUVGAH	165.963	3.026	GUVGAH	153.385	2.978	GUVGAH	150.786	3.026
GUVGOV	168.799	3.278	GUVGOV	157.325	3.088	GUVGOV	158.459	3.422	GUVGOV	168.603	3.19	GUVGOV	150.203	3.276
GUWNUL	152.369	3.111	GUWSOH	158.743	3.229	GUVGOV	175.824	3.056	GUVHUE	161.351	2.812	GUVHUE	163.969	2.741
GUWVUP	167.085	2.774	GUWVUP	162.35	3.28	GUWSOH	152.716	3.193	GUWVUP	164.851	3.076	GUWVUP	153.5	2.826
GUYGAM	170.99	2.885	GUYGAM	152.999	3.362	GUXNIA	155.981	3.002	GUXNIA	159.684	3.376	GUXVIF	153.439	3.012
GUYJOC	152.215	3.283	GUYJOC0	152.345	3.281	GUYHEN	163.765	3.141	GUYHEN	165.093	2.751	GUYJAL	161.388	3.485
GUYNUL	154.908	3.448	GUYRUO	151.989	2.786	GUYKEQ	151.862	3.317	GUYNEW	157.203	3.181	GUYNOF	154.043	3.22
GUYRUO	167.411	2.817	GUYRUO	167.372	2.733	GUYRUO	155.289	2.786	GUYRUO	158.223	3.185	GUYRUO	156.23	2.733
GUYRUO	156.179	2.781	GUYRUO	161.914	3.098	GUYRUO	169.606	3.062	GUYRUO	161.253	3.172	GUYRUO	153.424	2.932
GUYSAV	157.802	2.992	GUYSAV	150.858	3.265	GUYRUO	162.023	2.79	GUYSAV	165.319	3.371	GUYSAV	173.256	2.896
GUZLAR	162.024	2.963	GUZNUL	154.13	2.958	GUYSAV	164.003	2.912	GUYVEF	154.39	3.271	GUZDUB	153.279	3.324
GUZRUO	151.405	3.268	GUZRUO	162.552	3.087	GUZPAW	160.878	2.871	GUZPAW	165.766	2.985	GUZPAW	150.876	3.431
GUZZOT	160.853	3.387	GUZZOT	176.673	2.846	GUZZAD	153.732	3.093	GUZZOT	155.119	3.192	GUZZOT	161.579	3.239
GUZZUA	157.914	2.661	GUZZUZ	160.61	3.103	GUZZOT	159.612	3.239	GUZZUA	160.231	3.393	GUZZUA	157.7	3.434
GUZZUZ	167.805	3.38	GUZZUZ	167.055	2.929	GUZZUZ	152.805	3.066	GUZZUZ	157.273	3.018	GUZZUZ	159.391	3.464
HABGEZ	166.332	2.933	HABGEZ	155.532	3.09	HABBIZ	166.891	3.072	HABBIZ	173.988	3.115	HABGEZ	169.626	3.436
HABKUT	158.511	3.499	HABMUX	166.841	3.413	HABGEZ	153.408	3.118	HABGEZ	151.496	3.388	HABJUS	153.989	3.378
HABQAG	159.886	2.735	HABQAG	155.012	3.142	HABQAF	153.654	2.956	HABQAG	150.486	2.895	HABQAG	156.044	3.295

HABQAG	173.495	3.454	HABQUA	152.606	2.793	HABQAG	160.173	2.944	HABQAG	162.81	3.295	HABQAG	172.103	2.766
HACDAU	151.931	3.206	HACDOG	175.496	2.975	HABREK	158.572	3.136	HACDAU	155.841	3.129	HACDAU	166.601	3.459
HACGEZ	161.04	2.99	HACGEZ	154.512	3.085	HACGEZ	151.598	3.444	HACGEZ	170.337	3.144	HACGEZ	165.646	3.125
HADHID	162.463	3.295	HADHID	160.345	3.266	HACTIQ	150.588	3.174	HADHID	156.729	3.125	HADHID	154.172	2.811
HADHID	162.446	2.707	HADHID	150.334	3.218	HADHID	167.379	3.403	HADHID	154.632	3.077	HADHID	154.288	2.787
HADHID	164.35	2.832	HADHID	157.202	3.384	HADHID	158.544	2.897	HADHID	157.204	3.461	HADHID	153.274	3.302
HADQEI	154.392	3.451	HAFDEZ	176.486	3.228	HADHID	160.754	2.962	HADHID	151.727	3.313	HADNOS	169.036	2.977
HAFDEZ	158.943	3.306	HAFDEZ	165.42	3.085	HAFDEZ	164.573	3.435	HAFDEZ	151	3.226	HAFDEZ	156.278	2.818
HAFFAX	156.077	2.861	HAFFAX	170.302	2.763	HAFDEZ	151.303	3.227	HAFDEZ	166.557	2.71	HAFDEZ	176.7	3.472
HAFFIF	160.911	2.954	HAFFIF	150.676	3.131	HAFFAX	152.217	3.351	HAFFAX	150.868	3.251	HAFFAX	177.85	2.853
HAFFOL	151.027	2.857	HAFFOL	167.9	2.784	HAFFIF	160.384	3.269	HAFFOL	161.881	3.031	HAFFOL	165.815	2.713
HAFNAG	161.681	3.145	HAFNUA	159.466	3.172	HAFJUW	154.761	3.269	HAFNAE	151.262	3.24	HAFNAG	160.587	3.145
HAGGAB	163.634	3.107	HAGHED	166.605	3.263	HAFRAK	170.483	3.311	HAFRUD	152.077	3.153	HAFRUD	152.309	3.136
HAGQOW	166.04	3.405	HAGQOW	158.82	2.882	HAGNOV	150.484	2.974	HAGNOV	162.098	2.978	HAGNOV	150.849	3.485
HAGRAJ	150.344	3.032	HAGREN	151.854	3.118	HAGQOY	157.287	3.289	HAGQUC	153.22	3.32	HAGQUC	160.748	2.821
HAGSAK	155.973	3.295	HAGYAP	153.579	2.988	HAGREN	166.497	2.827	HAGREN	150.027	3.486	HAGSAK	163.273	3.494
HAGYAP	167.273	2.793	HAGYAP	171.265	2.915	HAGYAP	164.545	3.145	HAGYAP	151.873	3.05	HAGYAP	151.653	3.138
HAGYAP	150.675	3.063	HAGYAP	151.782	2.842	HAGYAP	152.011	3.148	HAGYAP	152.813	2.793	HAGYAP	157.852	3.163
HAHCIE	161.415	3.228	HAHCIE	150.827	3.272	HAGYAP	163.638	3.416	HAGYAP	151.182	3.289	HAHBUP	157.157	3.386
HAHMUA	152.821	3.137	HAHNUB	174.255	3.25	HAHGAC	153.605	3.221	HAHGIJ	163.782	2.941	HAHMEJ	163.233	2.976
HAHNUB	157.767	3.436	HAHNUB	167.657	3.009	HAHNUB	163.934	3.228	HAHNUB	174.57	3.457	HAHNUB	157.52	3.457
HAJMEN	161.76	3.187	HAJVUJ	156.283	3.404	HAHNUB	160.935	2.921	HAJFIL	153.088	3.033	HAJFIL	159.651	3.304
HAJWUK1	153.248	3.333	HAJWUK1	151.026	3.437	HAJWUK	153.248	3.333	HAJWUK	151.026	3.437	HAJWUK	155.508	3.314
HAKBEE	161.252	3.356	HAKJOS	162.894	3.381	HAJWUK1	155.508	3.314	HAJZEX	169.155	3.477	HAJZEX10	169.155	3.477
HAKQIV	166.55	3.393	HAKSIZ	164.251	3.254	HAKMIS	170.271	2.996	HAKMIS	163.838	3.258	HAKMOZ	164.196	2.711
HAKXUO	170.663	3.345	HAKYAU	160.203	2.829	HAKSIZ	159.882	3.283	HAKSIZ	175.381	3.014	HAKSIZ	172.085	3.008
HAKYAU	164.127	3.381	HAKYAU	155.561	2.694	HAKYAU	150.437	3.13	HAKYAU	152.457	2.9	HAKYAU	150.025	2.829
HAKYOI	154.684	3.449	HAKYOI	152.843	2.792	HAKYAU	159.57	2.781	HAKYAU	161.241	2.694	HAKYOI	169.36	3.044
HALLOX	165.02	3.299	HALLOX	156.12	3.117	HALDUV	153.019	3.5	HALFAD	155.656	3.411	HALFAD	164.24	3.423
HALMEP	159.165	3.332	HALMEP	155.136	3.053	HALLOX	156.758	3.432	HALMAL	151.198	3.042	HALMAL	166.036	2.882
HALRIZ	165.47	2.974	HALVAT	170.544	3.293	HALQES	161.805	3.058	HALREV	159.321	3.376	HALREV	170.592	2.943
HALVOJ	168.698	3.322	HALVOJ	163.714	2.972	HALVEZ	156.855	3.464	HALVEZ	169.333	3.034	HALVOJ	156.816	3.024
HALWUP	165.589	3.367	HALWUP	155.341	2.886	HALWAW	158.654	3.455	HALWAW	168.445	3.052	HALWEA	173.766	2.936
HAMMOB	164.392	3.204	HAMMOB	166.314	2.562	HALWUP	167.197	2.848	HALWUP	157.5	2.886	HAMMOB	165.453	2.562
HAMMUH	158.978	2.797	HAMMUH	151.957	3.48	HAMMUH	152.234	3.05	HAMMUH	162.289	2.757	HAMMUH	157.156	2.952
HAMNIW	150.505	3.076	HAPMUG	150.271	3.197	HAMNES	171.095	2.934	HAMNES	150.033	3.002	HAMNIW	166.948	3.401
HAPNIZ	160.855	3.366	HAPYIJ	156.955	3.387	HAPNER	165.06	2.627	HAPNER	150.09	3.344	HAPNER	170.868	2.627
HAQDAH	154.502	2.972	HAQDEL	170.213	3.285	HAQDAH	155.997	2.757	HAQDAH	165.712	3.496	HAQDAH	155.104	3.128
HAQYIK	165.507	3.29	HARSIC	168.565	3.335	HAQDEL	169.331	3.291	HAQDEL	159.941	3.118	HAQYIK	150.465	3.345
HARWEE	173.298	2.961	HARXAB	169.115	3.109	HARSOI	153.855	3.115	HARSOI	163.419	2.832	HARSOI	169.18	2.993
HASBOU	155.324	3.301	HASBOU	152.282	2.959	HASBIP	154.025	3.428	HASBIP	159.133	3.306	HASBOU	164.737	3.103
HASGAL	166.541	2.836	HASGAL	174.614	3.154	HASBOU	151.801	3.254	HASBOU	161.295	3.17	HASBOU	160.487	3.38
HASMUJ	163.347	2.883	HASMUJ	168.114	3.383	HASGIT	160.035	2.879	HASJAQ	164.122	3.412	HASMUJ	164.689	3.261
HASZIM	155.809	2.792	HASZIM	163.258	3.029	HASQUR	175.124	3.447	HASTUQ	163.477	3.081	HASZIM	162.438	3.416
HASZOS	160.567	3.339	HATBAI	158.655	2.861	HASZOS	153.903	3.272	HASZOS	163.372	2.746	HASZOS	172.852	2.992
HATNOI	151.225	3.339	HATNOI	177.748	3.231	HATLEX	152.705	3.315	HATNAS	164.276	3.183	HATNOI	150.898	3.042
HATQAU	151.689	3.288	HATQAU	152.886	3.166	HATNOI	156.308	3.231	HATNOI	173.445	2.881	HATQAU	152.678	3.19
HAVCOW	157.548	2.8	HAVCUF	169.567	3.082	HATVAC	154.582	3.052	HATYEJ	166.869	3.007	HATYEJ	167.228	3.007
HAVCUF	153.39	2.986	HAVLUM	166.782	2.91	HAVCUF	171.199	2.841	HAVCUF	164.666	3.082	HAVCUF	155.511	2.862
HAWCEQ	154.722	3.192	HAWCEQ	167.148	2.979	HAVSAC	152.129	3.488	HAVSIH	153.512	2.942	HAVTOO	167.48	2.959
HAWJAR	150.519	2.872	HAWJAR	164.298	3.495	HAWJAR	163.948	3.006	HAWJAR	152.255	2.844	HAWJAR	165.572	2.962
HAWQOM	161.921	3.37	HAWSUT	154.652	2.736	HAWJOE0	155.004	2.898	HAWMOK	171.07	3.079	HAWMOK	173.926	3.474
HAWVAF	154.834	3.197	HAWVAF	155.328	3.14	HAWTAA	159.448	3.113	HAWTAA	156.457	2.616	HAWTAA	154.178	3.248
HAWXAG	162.741	3.124	HAWXEK	151.269	3.11	HAWVAF	159.112	3.282	HAWXAG	169.028	3.381	HAWXAG	163.127	3.059
HAWXIO	153.461	2.974	HAWXIO	152.689	3.017	HAWXEK	154.703	2.898	HAWXEK	154.628	2.898	HAWXIO	173.863	3.037
HAWYIP	167.622	2.838	HAWYIP	158.948	3.141	HAWXUA	161.124	3.066	HAWXUA	162.899	3.003	HAWXUA	168.989	3.446
HAWYIP	156.192	2.954	HAWYIP	158.415	3.192	HAWYIP	163.427	3.08	HAWYIP	155.07	3.423	HAWYIP	161.806	3.033
HAWYIP	151.514	2.997	HAWYOV	160.106	3.055	HAWYIP	165.365	3.005	HAWYIP	159.956	3.127	HAWYIP	150.672	3.341
HAWYOV	162.165	3.064	HAWYOV	154.181	3.207	HAWYOV	157.044	3.307	HAWYOV	159.5	2.962	HAWYOV	151.463	3.344
HAXMAY	154.484	3.082	HAXMAY	158.567	3.082	HAWYOV	157.462	3.04	HAWYOV	156.343	2.962	HAXMAY	176.361	3.433
HAXNAY	160.371	3.037	HAXNAY	163.057	3.078	HAXMIF	178.368	3.307	HAXMIF	173.374	3.307	HAXMUR	163.643	3.371
HAXPAZ	151.861	2.85	HAXPAZ	150.529	2.85	HAXNAY	161.634	2.875	HAXNAZ	163.38	3.028	HAXNAZ	168.306	2.733
HAXROR	155.779	2.77	HAXROR	150.652	3.5	HAXRAC	150.377	3.122	HAXREH	164.717	3.095	HAXREH	154.376	3.318
HAXRUT10	157.246	3.399	HAXWOU	151.723	2.897	HAXROR	159.489	2.95	HAXROR	152.677	3.091	HAXRUT	157.419	3.372
HAXZEM	171.391	3.227	HAXZEM	155.36	2.968	HAXWOU	153.387	2.916	HAXWOU	153.567	2.926	HAXWOU	150.349	2.904
HAYPED	162.839	2.979	HAYPED	156.463	3.402	HAYFET	152.478	3.143	HAYFET	159.446	3.43	HAYJOI	165.16	3.193
HAYWIP	166.706	3.493	HAYWIP	156.781	3.484	HAYPED	152.546	2.979	HAYREH	173.549	3.461	HAYWIP	153.954	3.107
HAZHEW	156.805	3.321	HAZHEW	173.157	3.278	HAYWIP	171.017	3.391	HAYYAJ	156.897	3.204	HAYYAJ	152.254	3.057
HAZHUM	164.686	3.158	HAZHUM	151.679	3.073	HAZHEW	170.915	3.198	HAZHUM	166.69	3.053	HAZHUM	165.994	2.831
HAZHUM	174.657	2.972	HAZHUM	165.239	2.955	HAZHUM	170.298	3.241	HAZHUM	162.501	3.284	HAZHUM	163.912	2.947
HAZHUM	156.873	2.892	HAZHUM	155.893	3.05	HAZHUM	163.939	3.277	HAZHUM	168.523	3.295	HAZHUM	150.208	3.359
HAZHUM	152.44	3.346	HAZHUM	173.364	2.843	HAZHUM	167.446	3.19	HAZHUM	160.939	3.093	HAZHUM	168.943	2.922
HAZHUM	165.393	3.47	HAZHUM	155.765	2.971	HAZHUM	175.248	3.004	HAZHUM	168.676	2.892	HAZHUM	156.998	2.808
HAZHUM	176.977	2.805	HAZHUM	158.568	3.137	HAZHUM	156.878	2.87	HAZHUM	157.008	3.118	HAZHUM	155.773	3.237
HAZHUM	171.962	3.454	HAZHUM	162.157	2.603	HAZHUM	150.978	2.896	HAZHUM	161.051	3.298	HAZHUM	164.393	2.825
HAZHUM	151.96	3.199	HAZHUM	151.869	3.248	HAZHUM	158.99	2.867	HAZHUM	151.689	2.933	HAZHUM	155.461	3.054
HAZHUM	157.015	3.415	HAZHUM	153.107	3.373	HAZHUM	167.584	3.408	HAZHUM	160.601	3.407	HAZHUM	153.235	3.224
HAZRIL	150.149	3.144	HEBFUT	152.469	2.996	HAZHUM	163.399	2.707	HAZRAE	164.375	3.353	HAZRIL	173.186	3.019
HECJEG	154.093	3.303	HECJEG	170.939	2.955	HECHIK	155.938	3.103	HECHIK	150.206	3.269	HECJEG	164.565	3.447

HECJEG	157.601	3.409	HECJEG	153.699	3.482	HECJEG	158.494	2.939	HECJEG	170.269	2.986	HECJEG	152.766	2.967
HECMUY	167.777	3.04	HECMUY	156.699	2.846	HECJEG	150.767	3.415	HECJEG	152.394	3.11	HECLAF	172.426	3.439
HEDDAY	158.426	3.448	HEDNUC	171.133	3.182	HEDCID	170.518	3.035	HEDCIF	154.367	3.158	HEDCIF01	154.374	3.152
HEDPUE	174.968	3.423	HEDPUE	171.3	3.173	HEDNUC	168.924	3.411	HEDNUC	175.144	3.438	HEDPAK	174.002	3.476
HEDSOB	171.016	2.845	HEDSOB	162.445	2.845	HEDPUE	169.11	3.4	HEDROA	154.373	2.941	HEDROA	168.28	3.265
HEFBIG	155.672	2.717	HEFCAZ	156.871	3.162	HEDSUH	150.032	3.288	HEFBIG	158.329	3.381	HEFBIG	159.498	3.081
HEFCAZ	154.739	3.485	HEFCAZ	154.998	3.294	HEFCAZ	169.922	2.703	HEFCAZ	170.021	2.703	HEFCAZ	150.701	3.424
HEFCAZ	164.882	3.068	HEFCAZ	170.876	2.765	HEFCAZ	160.7	2.966	HEFCAZ	152.464	2.66	HEFCAZ	152.54	3.384
HEFCAZ	163.793	2.895	HEFJIO	160.553	3.168	HEFCAZ	164.686	2.739	HEFCAZ	156.496	2.994	HEFCAZ	166.699	2.739
HEFQUH	164.621	2.926	HEFQUH	161.687	2.899	HEFJIO	158.125	2.922	HEFMOX	154.494	3.096	HEFQUH	161.178	2.901
HEFSOC	158.668	2.916	HEFTUH	150.213	3.119	HEFQUH	165.833	2.919	HEFROD	167.535	3.124	HEFROD	162.902	3.298
HEFTUH	163.442	3.191	HEFTUH	162.744	3.221	HEFTUH	169.036	3.049	HEFTUH	155.374	3.023	HEFTUH	171.182	3.063
HEGNAL	153.717	3.498	HEGNAL	152.067	3.19	HEFTUH	150.759	3.303	HEFTUH	154.009	3.049	HEGMAK	155.736	3.422
HEGRAP	169.001	3.007	HEGRAP	163.402	2.806	HEGPUE	172.675	2.907	HEGPUE	155.33	3.356	HEGPUE	159.133	2.999
HEGWEZ	154.186	3.174	HEGWID	153.477	3.004	HEGRAP	161.602	3.435	HEGRAP	158.329	3.007	HEGRUG	156.582	2.938
HEHJIR	166.735	3.26	HEHJIR	159.552	3.431	HEHJIN	160.032	3.389	HEHJIR	164.006	2.964	HEHJIR	166.528	3.043
HEHJOX	160.27	3.437	HEHJUD	159.549	3.413	HEHJOX	164.658	3.068	HEHJOX	170.116	3.298	HEHJOX	163.891	3.016
HEHMUC	161.09	3.129	HEHMUC	155.052	3.262	HEHJUD	164.168	2.972	HEHJUD	167.112	3.27	HEHJUD	164.553	3.05
HEJCUY	154.338	2.77	HEJRAR	160.354	3.097	HEHMUC	156.184	3.129	HEJBOP	163.524	3.479	HEJCUY	153.833	2.77
HEKCAE	157.36	2.804	HEKCAE	151.134	3.366	HEJRAR	153.136	3.277	HEJREX	153.06	3.311	HEJVUR	151.545	2.884
HEKCEI	152.585	3.088	HEKKAM	156.658	3.28	HEKCAE	160.076	3.05	HEKCAE	167.075	3.492	HEKCEI	167.627	3.422
HEKZEC	163.107	3.473	HEKZEC	164.587	3.229	HEKTIA01	157.96	2.821	HEKZEC	165.273	3.457	HEKZEC	158.259	3.313
HELGUC	171.195	3.158	HELGUC	171.587	3.162	HELFUD	152.051	3.085	HELFUD	161.745	3.455	HELFUD	157.907	3.173
HELHOV	155.022	2.826	HELHOV	173.22	3.316	HELGUC	178.151	3.484	HELGUC	176.571	3.314	HELGUC	176.262	3.318
HELSEW	159.935	2.832	HELSEW	169.017	3.056	HELHOV	150.751	2.826	HELHOV	167.986	2.87	HELSEW	155.27	3.163
HEMBEK	166.252	3.344	HEMBEK	164.974	3.236	HELTIE	154.001	3.072	HEMBEK	158.367	3.458	HEMBEK	160.606	3.297
HEMDOS	156.917	3.095	HEMHAM	161.094	3.188	HEMBIO	164.934	3.004	HEMBIO	164.006	3.118	HEMDOS	156.61	3.371
HENMAR	150.19	3.44	HENMEW	151.603	3.251	HEMWIG	159.274	3.313	HENMAR	159.729	3.045	HENMAR	157.598	3.403
HENVAA	153.647	3.307	HENXIH01	161.313	3.374	HENMOG	170.81	3.031	HENRIB	160.176	3.294	HENTAZ	174.041	2.91
HEPJOF	163.811	3.079	HEPMEY	169.859	3.036	HENXIH01	154.263	3.28	HENYUU	161.329	3.186	HENZIJ	151.32	3.049
HEPVUT	151.335	3.498	HEPVUT	162.093	3.297	HEPMUL	157.15	2.982	HEPMUL	165.343	2.904	HEPRUQ	161.288	2.992
HEQFOC	153.116	3.444	HEQFOC	172.914	2.592	HEQFOC	156.892	3.389	HEQFOC	166.242	2.796	HEQFOC	170.517	2.592
HEQPID	156.585	2.832	HEQPID	161.504	3.302	HEQFOC	167.536	3.338	HEQFOC	169.381	3.3	HEQFOC	158.684	2.997
HEQSUT	154.27	3.314	HEQYOU	156.081	3.314	HEQPOJ	151.508	2.911	HEQPOJ	165.757	3.31	HEQSUT	174.605	2.7
HEQZEI	172.155	2.913	HEQZEI	151.069	2.789	HEQZEI	158.73	3.109	HEQZEI	174.462	2.953	HEQZEI	152.702	2.833
HERVOP	158.279	3.179	HERVOP	167.429	3.317	HERHOC0	169.367	2.777	HERPAZ	160.975	3.122	HERSUS	169.837	3.441
HESZEO	157.514	3.402	HESZEO	158.878	3.298	HERVOP	158.654	2.988	HERVOP	162.883	3.092	HESDOB	155.176	3.1
HETZUF	159.092	3.474	HEWFAQ	167.791	3.243	HETBOA	151.958	3.279	HETBOA0	152.229	3.271	HETQOM	151.587	3.151
HEWGID	161.044	3.281	HEWKUS	171.745	2.719	HEWGID	162.976	3.496	HEWGID	169.761	3.005	HEWGID	158.576	3.397
HEWLUT	150.006	3.05	HEWLUU	150.53	3.436	HEWKUS	151.296	3.433	HEWKUS	176.143	2.632	HEWKUS	163.226	3.433
HEWXUG	157.012	3.494	HEWXUG	150.083	2.804	HEWSIO	152.517	2.957	HEWWOW	158.499	3.269	HEWXUG	150.305	3.131
HEXGUM	152.826	3.315	HEXGUM	151.045	3.417	HEWXUG	155.842	3.169	HEWXUG	159.58	3.136	HEWXUG	176.971	3.105
HEXGUM	165.681	3.335	HEXGUM	161.899	2.669	HEXGUM	162.343	3.009	HEXGUM	160.241	3.355	HEXGUM	156.333	2.841
HEXYAK	164.792	3.369	HEXYAK	157.046	3.369	HEXGUM	155.171	2.852	HEXWOY	168.426	3.266	HEXYAK	179.258	2.826
HEXYAK	156.498	3.075	HEXYAK	160.426	2.382	HEXYAK	172.363	3.151	HEXYAK	153.483	2.986	HEXYAK	153.113	3.151
HEYLAC	168.538	3.262	HEYMUX	162.421	3.494	HEXYIT	159.166	3.452	HEXZAM	155.513	3.425	HEXZEQ	150.116	3.198
HEZHON	155.199	2.732	HEZHON	152.662	3.466	HEYRUC	161.915	3.182	HEYYAN	160.279	2.924	HEZDAU	156.187	3.348
HFAESC	154.164	3.143	HFBENZ02	161.451	3.098	HEZHON	164.547	2.99	HEZHON	153.213	3.451	HEZHON	154.315	2.921
HFBENZ02	154.223	3.027	HFBENZ15	171.55	2.913	HFBENZ02	157.732	3.137	HFBENZ02	169.358	3.137	HFBENZ02	162.138	2.798
HFBENZ16	173.582	2.861	HFBENZ16	167.678	2.927	HFBENZ15	173.567	2.882	HFBENZ15	167.697	2.937	HFBENZ16	171.611	2.898
HFBENZ18	172.385	2.818	HFBENZ18	174.537	2.791	HFBENZ17	171.291	2.882	HFBENZ17	173.364	2.842	HFBENZ17	167.355	2.913
HFBENZ19	168.804	2.806	HFBOCP	164.131	3.204	HFBENZ18	167.99	2.848	HFBENZ19	173.057	2.794	HFBENZ19	175.145	2.748
HFMPDC1	153.925	3.008	HFMPDC1	150.228	3.255	HFMBPO1	164.432	3.013	HFMPDC1	154.659	2.959	HFMPDC1	165.394	3.054
HFMPHE	155.395	3.391	HFMPHE	166.452	3.077	HFMPHE	161.978	3.283	HFMPHE	154.643	3.352	HFMPHE	161.161	3.419
HFMPHE	151.247	3.193	HFMPHE	158.868	3.249	HFMPHE	152.427	3.352	HFMPHE	160.797	3.06	HFMPHE	155.447	3.467
HIBGOP	164.065	3.453	HIBGOP	161.186	3.475	HFPCDN	175.437	3.036	HFPCDN	165.913	3.11	HIBGOP	170.115	3.007
HICBAW	152.849	2.987	HICBAW	151.081	2.987	HIBGOP	169.365	3.112	HIBGOP	160.023	2.89	HIBGOP	164.741	3.081
HICJAF	150.302	3.269	HICQER	152.016	2.828	HICJAF	152.264	3.158	HICJAF	169.502	3.119	HICJAF	166.333	3.485
HIDGIM	158.997	3.21	HIDGOU	161.48	2.959	HICQER	169.911	3.089	HIDCAZ	158.954	2.8	HIDCAZ	152.737	2.709
HIDNOB	160.881	3.014	HIDNOB	154.444	2.927	HIDGOU	173.668	3.225	HIDKAH	176.725	3.451	HIDLIT	162.885	3.195
HIDREU	162.89	2.836	HIDRIY	151.371	3.345	HIDNOB	151.043	3.424	HIDNOB	154.607	3.331	HIDREU	155.344	3.094
HIFCUV	156.316	3.112	HIFJEL01	160.906	3.225	HIDTUL	161.692	3.261	HIDTUL	161.916	3.31	HIDXEX	159.708	3.166
HIFJEL01	161.616	3.075	HIFJEL01	174.668	3.4	HIFJEL01	160.183	2.78	HIFJEL01	158.226	3.089	HIFJEL01	151.446	2.893
HIGCOT	162.309	3.454	HIGCOT	151.818	2.706	HIFJEL01	157.306	2.931	HIFZED	157.787	3.141	HIGBIJ	158.919	3.148
HIGKEP	154.56	2.788	HIGKEP	164.915	2.943	HIGCOT	156.855	3.096	HIGCOT	161.909	3.418	HIGFAI	173.928	2.826
HIGLAL	174.011	3.487	HIGLUF	160.246	3.29	HIGLAL	163.981	2.979	HIGLAL	167.078	3.373	HIGLAL	160.67	3.212
HIGTID	160.423	3.451	HIGTID	151.151	2.934	HIGPAP	173.43	3.354	HIGSUP	156.32	3.072	HIGSUP	163.874	2.976
HIGVAX	164.281	2.942	HIGVAX	163.311	2.96	HIGVAX	160.056	2.817	HIGVAX	160.561	2.852	HIGVAX	165.092	2.937
HIGVAX	161.436	2.811	HIGVAX	161.597	2.828	HIGVAX	161.117	2.964	HIGVAX	156.067	3.037	HIGVAX	155.281	3.043
HIGVAX	158.084	2.998	HIGVAX	153.131	3.024	HIGVAX	167.13	2.946	HIGVAX	165.907	2.951	HIGVAX	161.534	2.965
HIGVAX	166.528	2.943	HIGVAX	165.514	2.951	HIGVAX	156.174	3.017	HIGVAX	160.937	2.771	HIGVAX	161.061	2.792
HIGVAX	152.938	3.104	HIGVOL	151.12	3.114	HIGVAX	161.427	2.942	HIGVAX	161.1	2.947	HIGVAX	154.249	3.069
HIHLIX	161.039	2.862	HIHREX	170.114	2.876	HIGWUT	155.734	2.844	HIGXAA	168.476	2.704	HIGXAA	170.862	2.704
HIHTUP	164.641	3.057	HIHVAX	153.764	3.237	HIHREX	157.454	3.051	HIHRUN	154.915	3.028	HIHSAU	163.722	3.239
HIJJAO	162.676	2.921	HIJJAO	152.812	3.123	HIHVAX	158.075	3.305	HIJGOZ	152.848	2.779	HIJGOZ	174.912	2.779
HIJJAO	165.7	2.798	HIJJAO	154.159	3.483	HIJJAO	167.706	2.813	HIJJAO	167.48	3.097	HIJJAO	177.897	2.813
HIJXAB	162.757	3.198	HIJXAB	152.898	3.296	HIJJAO	155.609	2.874	HIJJIW	160.611	3.186	HIJLIY	162.658	2.969
HIJZAD	155.895	3.089	HIKCOV	168.317	3.01	HIJXAB	157.367	3.085	HIJXAB	161.695	3.184	HIJZAD	159.66	3.324
HIKJAM	155.378	3.074	HIKJAM	156.172	3.202	HIKDAG	157.159	3.381	HIKDAG	161.892	3.092	HIKGOB	165.99	2.905

HILBIQ	165.019	3.106	HILBOT	172.787	2.978	HIKJAM	173.275	3.453	HIKKOE	152.11	2.665	HIKMIX	153.109	3.194
HILJAQ	166.094	2.965	HILJAQ	158.251	2.786	HILCUB	170.068	3.179	HILGES	163.419	3.07	HILJAQ	151.81	2.846
HILJAQ	159.842	2.882	HILJIY	166.785	3.194	HILJAQ	172.387	3.111	HILJAQ	153.639	2.845	HILJAQ	173.261	3.153
HILJOE	166.842	2.725	HILJOE	176.217	3.376	HILJOE	168.572	2.703	HILJOE	166.214	3.035	HILJOE	165.469	2.733
HILKIZ	163.154	3.083	HILKIZ	166.864	3.249	HILJOE	165.076	2.776	HILKIZ	172.721	3.328	HILKIZ	157.461	2.93
HILKOF	167.577	3.205	HILKOF	161.14	3.164	HILKIZ	166.387	2.964	HILKOF	165.507	2.778	HILKOF	156.21	2.896
HILLEV	161.665	3.102	HILQOJ	150.803	3.476	HILKOF	177.143	3.344	HILLEV	151.007	3.38	HILLEV	153.47	3.061
HIMCOY	169.807	3.082	HIMDIT	153.242	2.911	HILZIN	154.724	3.283	HIMBIO	162.919	3.364	HIMCEM	168.495	3.297
HIMHUI	165.515	3.193	HIMHUI	170.421	2.942	HIMDIT	171.092	3.028	HIMDOX	153.54	2.995	HIMFUE01	168.183	3.299
HIMKAR	166.369	3.47	HIMKAR	165.664	3.001	HIMKAR	152.389	3.471	HIMKAR	169.347	2.754	HIMKAR	155.213	3.217
HIMKIZ	152.095	3.303	HIMKIZ	175.804	2.838	HIMKEV	152.745	3.072	HIMKEV	154.137	3.09	HIMKIZ	152.74	3.259
HIMTAA	161.961	3.136	HINCEP	167.531	2.801	HIMLEY	167.896	2.777	HIMTAA	150.385	3.336	HIMTAA	166.216	2.878
HINPEZ	158.903	3.235	HINPEZ	153.875	2.909	HINCEP	174.87	2.801	HINGUI	163.037	3.38	HINNAT	150.117	3.334
HIPQEE	156.734	3.146	HIPQEE	158.058	3.147	HINPEZ	166.016	2.817	HIPCAK	161.427	3.405	HIPFOE	162.575	2.729
HIPQEE	164.231	2.69	HIPQEE	170.88	3.206	HIPQEE	150.464	2.998	HIPQEE	153.724	3.267	HIPQEE	173.124	3.092
HIQPUW	174.409	3.466	HIQVAG	164.884	3.064	HIPXUA	153.169	3.051	HIPXUA	162.047	3.051	HIPXUA	150.756	3.38
HIRCIU	172.327	3.334	HIRCIU	162.802	3.414	HIQVUC	164.209	3.131	HIQVUC	167.967	2.953	HIRCIU	150.386	3.388
HIRGOE	170.333	3.369	HIRGOE	169.864	2.79	HIRCIU	154.725	3.111	HIRCIU	156.96	3.369	HIRFOH	159.067	3.332
HIRKIG	174.465	3.359	HIRKIG	173.678	3.364	HIRKAY	161.33	3.324	HIRKIG	150.312	3.098	HIRKIG	157.084	3.091
HIRKOJ	160.737	3.446	HIRMAY	154.722	3.034	HIRKOJ	165.833	3.412	HIRKOJ	174.934	3.468	HIRKOJ	160.804	3.457
HIRMEC	150.179	3.37	HIRMUT	164.27	2.925	HIRMEC	153.869	3.201	HIRMEC	156.027	3.442	HIRMEC	151.455	3.419
HIRTUY	157.317	3.163	HIRTUY	153.902	3.421	HIRNIG01	163.176	2.898	HIRTUY	162.857	3.069	HIRTUY	165.935	2.648
HISFEX	155.921	3.095	HISHIE	161.636	3.168	HIRTUY	156.75	3.066	HIRXEM	161.784	3.49	HIRXEM	174.714	3.107
HISKEB	171.327	3.473	HISKIF	154.656	3.088	HISHOK	161.164	3.168	HISHOK	156.779	3.469	HISKEB	176.722	3.355
HISKIF	159.71	2.884	HISKIF	170.843	3.221	HISKIF	151.59	3.065	HISKIF	161.918	3.389	HISKIF	163.343	2.712
HISNAZ	167.525	3.08	HITBUH	172.41	3.339	HISKIF	161.043	3.471	HISKIF	160.919	2.995	HISLOM	161.008	3.344
HITPEF	159.194	3.259	HITPUV	169.897	2.918	HITGIA	163.044	3.365	HITGIA	159.051	2.971	HITGIA	151.695	3.278
HITPUV	165.313	2.751	HITPUV	155.725	3.27	HITPUV	177.406	3.4	HITPUV	169.013	3.163	HITPUV	177.889	3.134
HITVAK	167.589	3.178	HITVUC	152.657	2.666	HITSEM	166.366	3.415	HITSEM	169.188	3.371	HITVAK	156.033	2.995
HITXIS02	150.811	3.272	HITXIS02	150.439	3.398	HITVUC	172.453	3.354	HITVUC	161.588	2.666	HITWUF	162.767	3.361
HITXIS03	150.99	3.397	HITXIS03	165.612	3.135	HITXIS02	157.049	3.109	HITXIS02	163.186	3.416	HITXIS03	167.031	3.341
HITYIW	164.057	3.056	HITYIW	168.792	3.015	HITXIS03	158.953	3.211	HITXIS03	151.495	3.288	HITYIW	156.228	3.223
HITZIX	161.888	2.936	HITZIX	156.781	3.042	HITYIW	150.305	3.352	HITYIW	151.814	3.294	HITZIX	168.703	2.795
HIVCEV	152.152	3.257	HIVDEV	178.237	2.903	HITZIX	160.741	2.95	HITZOD	162.827	3.171	HITZOD	154.029	3.286
HIVDEV	150.644	3.21	HIVDEV	169.272	3.043	HIVDEV	167.359	3.118	HIVDEV	161.752	3.045	HIVDEV	153.356	3.213
HIVDIA	176.381	2.807	HIVDUN	157.468	3.054	HIVDEV	162.355	3.062	HIVDEV	154.651	3.255	HIVDEV	155.314	3.337
HIVSUC	155.006	3.173	HIVWUH	153.397	3.042	HIVGOK	156.21	2.886	HIVSUC	164.759	3.106	HIVSUC	173.636	3.173
HIVZOB	160.439	3.465	HIVZOB	159.758	3.388	HIVXES	156.864	2.665	HIVXIW	177.192	3.06	HIVYUI	159.561	2.978
HIVZOB	152.804	3.389	HIWBEU	163.728	3.151	HIVZOB	159.057	3.203	HIVZOB	156.723	3.089	HIVZOB	176.269	3.388
HIWBEU	162.126	3.114	HIWKAZ	155.497	2.979	HIWBEU	175.466	3.414	HIWBEU	150.243	3.125	HIWBEU	152.259	2.912
HIWVOA	153.006	2.766	HIWVOA	151.669	2.956	HIWNOT	152.919	3.437	HIWPUC	173.027	2.811	HIWTAM	155.421	3.428
HIWYAR	172.071	2.776	HIWYAR	165.3	3.032	HIWXOE	151.608	3.476	HIWXOE	162.566	3.246	HIWYAR	159.357	2.665
HIXGOO	156.253	3.343	HIXGOO	167.286	3.235	HIXFIF	164.584	3.371	HIXGOO	160.813	2.9	HIXGOO	152.211	3.235
HIXLOR	173.982	3.262	HIXLOR	162.025	2.91	HIXGOO	155.338	2.918	HIXJOP	156.301	3.195	HIXJOP	159.347	2.901
HIXNIL	157.812	3.365	HIXSIR	152.619	3.011	HIXLOR	155.592	3.277	HIXLOR	160.352	3.325	HIXNEL	152.744	3.29
HIYCOK	158.635	2.896	HIYCOK	154.397	3.075	HIXSUD	170.637	2.799	HIXSUD	174.135	2.874	HIXSUD	158.23	3.295
HIYVUG02	151.566	2.869	HIYVUG02	153.983	3.103	HIYMAD	157.321	2.999	HIYVUG	154.659	3.128	HIYVUG02	154.166	2.978
HIZCUQ	166.025	3.459	HIZHEG	178.089	3.464	HIZBEY	159.347	2.804	HIZCUQ	151.657	2.917	HIZCUQ	158.007	2.917
HIZJIK	166.344	2.829	HIZKUA	158.317	3.305	HIZHUW	153.381	3.331	HIZHUW	169.352	3.409	HIZJIK	158.187	3.116
HIZLAH	158.024	2.926	HIZLAH	160.934	2.952	HIZKUA	152.281	2.951	HIZKUA	165.048	3.496	HIZKUA	158.327	2.96
HIZQIS	154.884	3.086	HIZQIS	150.149	2.89	HIZLAH	164.98	3.224	HIZLAH	158.931	3.345	HIZQIS	154.446	3.021
HIZQIS	153.006	2.781	HIZQIS	160.682	2.657	HIZQIS	159.789	2.657	HIZQIS	161.367	2.683	HIZQIS	151.937	2.777
HIZQOY	150.132	3.123	HIZQOY	156.735	3.216	HIZQIS	162.105	2.683	HIZQIS	151.035	2.777	HIZQIS	154.808	2.781
HIZRIS	156.937	3.139	HIZRIS	173.104	3.358	HIZREP	157.107	2.899	HIZREP	167.072	3.127	HIZRIS	151.992	3.136
HIZRIT	159.767	3.397	HIZWAQ	151.361	2.924	HIZRIT	174.618	2.697	HIZRIT	159.808	3.122	HIZRIT	155.022	3.349
HOBBIK	165.85	3.299	HOBBIK	150.386	2.914	HOBBAC	168.743	3.498	HOBBEG	167.051	3.305	HOBBEG	161.994	3.259
HOBBUW	150.206	3.264	HOBBUW	175.454	2.888	HOBBIK	156.085	3.463	HOBBOQ	156.296	2.766	HOBBOQ	170.445	2.881
HOBGOU	171.565	3.186	HOBHOZ	167.275	3.145	HOBBUW	173.369	2.908	HOBCAD	163.116	2.871	HOBCAD	166.434	2.871
HOBZIL	168.386	3.279	HOBZIL	150.282	3.138	HOBHOZ	152.644	3.12	HOBKEQ	156.653	3.128	HOBYAZ	167.623	3.187
HOCWEC	152.829	3.146	HOCWEC	170.892	3.191	HOCJES	158.709	3.488	HOCRAS	167.338	3.195	HOCRAS	160.783	2.86
HOCWEC	177.468	3.164	HOCWEC	168.219	2.997	HOCWEC	174.583	3.372	HOCWEC	154.128	3.462	HOCWEC	174.749	2.83
HODNAS	159.032	3.329	HODPAU	168.556	3.405	HOCWEC	166.545	3.09	HODBEH	150.16	3.409	HODNAS	156.717	3.277
HODPAU	153.842	2.898	HODPAU	155.123	3.345	HODPAU	169.287	2.973	HODPAU	168.356	2.884	HODPAU	159.275	3.301
HODXEG	169.124	3.074	HODXEG	155.445	2.977	HODPAU	160.17	3.203	HODPEY	151.638	3.027	HODPIC	153.994	3.374
HOFBOW	152.141	2.819	HOFBOW	168.766	2.892	HODXEG	154.761	2.954	HODXEG	169.034	3.041	HOFBAJ	151.864	2.725
HOFCAH	160.879	3.107	HOFCAH	158.204	3.144	HOFCAH	168.467	3.468	HOFCAH	153.593	3.409	HOFCAH	169.385	3.46
HOFCIR	151.647	2.918	HOFCIR	153.269	2.876	HOFCIR	168.775	2.955	HOFCIR	154.438	2.895	HOFCIR	163.537	3.149
HOFTIG	167.305	3.229	HOFVUU	150.24	3.439	HOFJAP	160.78	3.454	HOFJAP	161.008	3.221	HOFJAP	155.599	3.043
HOGDUC	154.609	2.893	HOGDUC	164.096	3.141	HOFXAC	154.692	3.333	HOGDUC	175.119	3.308	HOGDUC	156.641	3.375
HOGHIV	153.156	3.3	HOGHIV	153.023	3.037	HOGDUC	163.183	3.208	HOGHIV	153.694	3.105	HOGHIV	160.764	3.476
HOGHIV	158.802	3.025	HOGHIV	159.29	2.788	HOGHIV	163.754	3.208	HOGHIV	156.227	3.445	HOGHIV	154.483	2.895
HOGSEB	154.143	3.456	HOGSEF	154.252	3.118	HOGHIV	158.055	2.895	HOGHIV	152.141	3.208	HOGNIB	161.238	3.286
HOHHER	152.68	3.106	HOHHER	161.974	3.355	HOGZEK	152.821	2.74	HOGZEM	151.953	3.402	HOHHER	167.055	3.065
HOHHER	163.929	3.179	HOHHER	162.235	2.992	HOHHER	155.635	3.451	HOHHER	152.667	2.87	HOHHER	158.223	3.312
HOHJIB	150.725	3.146	HOHJIB	161.468	3.226	HOHHER	154.837	3.007	HOHHER	161.877	2.909	HOHHER	159.496	3.194
HOHJIB	160.425	3.173	HOHJOH	150.452	3.081	HOHJIB	162.34	3.148	HOHJIB	155.992	3.319	HOHJIB	171.126	2.885
HOHJOH	171.205	2.798	HOHJOH	168.938	3.436	HOHJOH	160.959	3.182	HOHJOH	162.778	3.08	HOHJOH	158.613	3.178
HOHJUJ	157.185	3.025	HOHJUJ	159.337	3.09	HOHJOH	158.434	3.157	HOHJUJ	170.359	2.723	HOHJUJ	156.747	2.835
HOHJUJ	160.363	3.433	HOHKAQ	155.379	3.087	HOHJUJ	156.974	2.823	HOHJUJ	152.293	3.043	HOHJUJ	154.575	3.353

HOHKAS	167.119	3.293	HOHKAS	153.573	3.081	HOHKAQ	150.708	3.042	HOHKAQ	157.044	3.419	HOHKAS	165.421	3.493
HOHKAS	151.3	3.27	HOHKAS	177.734	3.161	HOHKAS	173.849	2.852	HOHKAS	156	3	HOHKAS	152.171	3.313
HOHKUM	155.701	3.262	HOHKUM	156.905	3.351	HOHKAS	152.096	3.326	HOHKAS	164.331	2.852	HOHKAS	151.894	3.15
HOHKUM	164.612	3.012	HOHKUM	157.674	3.465	HOHKUM	166.891	3.144	HOHKUM	162.847	3.273	HOHKUM	171.731	3.135
HOHLEX	157.323	3.33	HOHLEX	168.519	3.104	HOHKUM	152.481	3.202	HOHKUM	161.4	3.287	HOHKUM	153.969	3.3
HOHLEX	153.283	3.4	HOHLEX	171.087	3.35	HOHLEX	157.307	3.339	HOHLEX	172.924	3.291	HOHLEX	156.757	2.911
HOHLEX	161.585	3.214	HOHLIB	152.964	3.28	HOHLEX	150.546	3.172	HOHLEX	153.362	3.015	HOHLEX	167.033	3.214
HOHLIB	167.122	3.21	HOHLIB	156.509	2.97	HOHLIB	176.909	3.383	HOHLIB	169.489	3.315	HOHLIB	152.604	3.197
HOHLIB	169.912	3.132	HOHLIB	151.313	2.78	HOHLIB	153.973	3.364	HOHLIB	173.728	3.284	HOHLIB	159.376	3.314
HOHYUY	172.572	2.886	HOHYUY	165.969	3.233	HOHLIB	162.061	3.21	HOHTIL	164.933	3.077	HOHXEL	157.519	2.946
HOJTUX	167.596	3.432	HOJXIR	159.707	3.467	HOJGOE	158.391	3.249	HOJGUK	150.543	2.951	HOJGUK	153.018	3.463
HOKKUO	156.463	3	HOKLOJ	177.261	2.692	HOJZUB	155.6	3.093	HOKBOA	158.092	3.099	HOKKEA	164.438	3.233
HOKMAW	178.511	2.811	HOKMAW	166.42	2.913	HOKLUP	151.299	2.785	HOKMAW	152.478	2.71	HOKMAW	169.197	2.71
HOKMIE	163.892	3.242	HOKMIE	152.645	2.836	HOKMAW	156.141	2.733	HOKMIE	164.541	2.944	HOKMIE	154.144	2.91
HOKMOK	175.535	3.111	HOKMOK	158.551	3.435	HOKMOK	156.614	3.224	HOKMOK	172.511	3.296	HOKMOK	163.53	2.831
HOKMUQ	156.447	2.849	HOKNAX	170.518	2.736	HOKMUQ	150.734	2.923	HOKMUQ	152.547	2.876	HOKMUQ	155.196	3.016
HOKVIQ	156.23	2.924	HOKVIQ	165.255	2.753	HOKNAX	153.247	3.032	HOKNAX	152.355	2.927	HOKNAX	155.032	2.99
HOKVIQ	152.463	3.262	HOKVIQ	152.628	3.457	HOKVIQ	168.263	2.759	HOKVIQ	150.114	3.018	HOKVIQ	152.996	3.021
HOKZUD	154.662	2.954	HOKZUD	158.522	3.027	HOKVIQ	157.519	2.96	HOKYIS	154.825	3.25	HOKYIS	164.965	3.25
HOLGEY	169.821	2.733	HOLGEY	155.593	2.84	HOLGEY	154.743	3.072	HOLGEY	164.239	2.998	HOLGEY	164.265	3.329
HOLGEY	155.03	3.137	HOLJAX	161.562	3.431	HOLGEY	151.937	3.373	HOLGEY	159.641	3.213	HOLGEY	152.504	3.141
HOMNEE	154.572	3.341	HOMNEE	161.242	3.252	HOLKUQ	159.566	3.256	HOLKUQ	151.007	3.426	HOMJIE	150.678	3.12
HOMZOC	169.1	3.093	HOMZOC	165.446	3.198	HOMNEE	162.893	2.97	HOMZIW	169.028	3.089	HOMZIW	165.202	3.201
HONDEW	165.183	3.24	HOPCEW	156.319	3.214	HONBOF	171.931	3.297	HONBUL	171.594	3.295	HONDEW	165.959	3.135
HOPCEW	172.813	3.406	HOPCEW	155.516	2.869	HOPCEW	173.747	2.957	HOPCEW	160.754	3.104	HOPCEW	151.641	2.613
HOPGOL	168.717	2.784	HOPGOL	161.112	2.784	HOPCIA	155.89	3.317	HOPCIA	173.053	2.937	HOPCIA	155.416	2.926
HOPLUV	166.492	2.834	HOPLUV	171.944	3.172	HOPGOL	163.362	2.819	HOPLOP	159.019	3.359	HOPLOP	165.958	3.263
HOPTUD	167.111	3.292	HOPTUD	154.706	2.825	HOPNUW	153.322	3.281	HOPTUD	162.398	3.065	HOPTUD	154.032	3.054
HOQXUH	151.11	3.01	HOQXUH	174.989	2.977	HOPTUD	169.582	2.88	HOQLUY	150.487	3.191	HOQLUY	157.394	3.191
HORVET	151.943	3.267	HORVET	163.364	3.267	HORKUY	158.037	3.293	HORQOU	157.396	3.434	HORQOU	160.419	3.419
HOSDID	151.865	2.928	HOSRAM	161.637	3.353	HORVOA	168	3.211	HORVUJ	161.819	3.169	HORYAS	152.768	3.432
HOSTOB	162.758	2.932	HOSTOB	153.314	2.758	HOSTOB	161.684	3.065	HOSTOB	150.642	3.125	HOSTOB	166.787	2.972
HOTRUD	156.624	3.154	HOTRUD	152.926	3.49	HOSWOF	153.54	3.074	HOTKOR	169.066	3.273	HOTRUD	155.568	2.748
HOTRUD0	157.455	2.968	HOTRUD0	152.09	3.486	HOTRUD	165.619	3.297	HOTRUD	174.476	3.334	HOTRUD0	152.253	2.686
HOTTIW	162.651	3.255	HOTTIW	160.078	3.44	HOTRUD0	174.467	3.17	HOTRUD0	165.306	3.268	HOTTIW	165.036	2.898
HOVFEF	155.238	3.251	HOVFEG	155.899	2.911	HOTTIW	157.936	2.67	HOTVIY	153.01	3.092	HOVDUT	155.38	3.499
HOVLIR	166.573	3.036	HOVLIR	166.846	3.338	HOVFEG	167.871	2.929	HOVKOW	167.345	3.103	HOVKOW	159.371	3.353
HOVMOY	174.455	3.083	HOVMOY	158.878	2.746	HOVLUD	151.013	3.064	HOVLUD	166.658	2.99	HOVMAK	153.397	3.043
HOVNIR	163.758	2.812	HOVNIR	163.615	2.882	HOVMOY	156.801	3.156	HOVMOY	160.815	3.356	HOVNIR	158.492	2.997
HOVQAN	152.499	3.306	HOVQIV	163.75	3.184	HOVPUG	169.83	2.959	HOVPUG	154.022	3.279	HOVQAN	154.581	3.182
HOVZEX	171.064	3.355	HOVZEX	153.209	3.194	HOVROC	171.722	2.632	HOVYIC	158.856	3.068	HOVZAT	162.402	3.434
HOWGAC	151.728	3.147	HOWGEG	161.377	2.848	HOVZIB	157.185	2.929	HOWGAC	164.099	3.42	HOWGAC	159.377	3.019
HOWPEQ	172.69	3.345	HOWPEQ	150.068	3.128	HOWGEG	158.843	2.825	HOWNUF	171.543	3.473	HOWPEQ	151.484	2.853
HOXQEU	175.538	2.765	HOXQEU	173.721	2.655	HOWWOH	156.155	3.418	HOWWOH	171.004	3.425	HOXJEN	152.479	3.414
HOXQOB	158.03	3.279	HOXQOB	153.626	3.133	HOXQEU	172.332	2.765	HOXQEU	175.814	2.655	HOXQOB	167.969	2.821
HOYBED	154.104	2.984	HOYDUY	175.797	2.769	HOXQOB	167.037	2.821	HOYBED	170.398	2.792	HOYBED	161.348	2.792
HOYJOX	158.442	3.099	HOYKEP	156.633	2.958	HOYDUY	174.032	2.665	HOYDUY	172.579	2.769	HOYDUY	174.888	2.665
HOYPOB	174.143	3.239	HOYPOB	164.007	3.307	HOYKEP	169.78	2.752	HOYPOB	154.263	3.009	HOYPOB	150.786	3.221
HOYQAO	164.295	2.742	HOYQAO	174.84	2.72	HOYPOB	160.006	2.795	HOYPOB	166.215	3.183	HOYPOB	170.934	2.882
HOYQAO	160.128	3.284	HOYQAO	150.556	2.928	HOYQAO	158.125	3.284	HOYQAO	172.525	2.808	HOYQAO	164.283	2.772
HOYSET	167.221	3.318	HOYSET	158.839	3.371	HOYQAO	166.761	2.853	HOYSET	152.133	2.961	HOYSET	164.692	3.446
HOYSUM	168.506	3.423	HOYSUM	167.281	3.397	HOYSUM	175.278	3.157	HOYSUM	162.389	3.157	HOYSUM	159.281	2.91
HOYSUM	163.197	2.859	HOYSUM	166.579	3.492	HOYSUM	153.517	3.295	HOYSUM	158.778	2.986	HOYSUM	153.313	3.197
HOYZOL	164.177	3.366	HOYZOL	168.18	2.947	HOYVAS	166.603	3.198	HOYVAS	164.127	3.166	HOYZOL	156.92	3.009
HOZMIW	161.053	3.354	HOZQAS	156.429	3.442	HOZCAB	157.65	3.433	HOZCAB	157.934	2.941	HOZCAB	164.776	2.941
HTFMBZ	152.808	2.956	HTFMBZ	168.463	3.12	HOZYED	168.174	3.097	HTFMBZ	169.816	3.12	HTFMBZ	161.058	3.094
HUBGUH	151.811	2.965	HUBGUH	175.312	3.035	HTFMBZ	151.202	3.091	HTFMBZ	156.455	3.171	HUBGUH	151.124	2.922
HUBTOR	161.213	3.08	HUBTOR	167.761	2.971	HUBJAS	161.498	2.936	HUBJAS	170.338	3.09	HUBJAS	154.549	3.208
HUBTOR0	167.872	3.028	HUBTOR0	161.73	3.094	HUBTOR0	162.84	3.119	HUBTOR0	168.019	3.001	HUBTOR0	164.471	3.151
HUBVAB	173.64	3.203	HUBVUV	166.143	2.749	HUBTOR0	167.975	2.984	HUBTOR0	163.644	3.135	HUBTOR0	167.951	3.013
HUBWAC	162.51	3.358	HUBWAC	153.4	3.242	HUBVUV	153.656	2.751	HUBVUV	150.044	2.751	HUBWAC	162.811	2.947
HUBYUC	157.222	3.199	HUCLUM	173.25	3.128	HUBWAC	153.696	2.972	HUBWEG	154.446	3.459	HUBYUC	152.105	3.254
HUCLUM	153.946	2.803	HUCVEK	155.418	2.574	HUCLUM	171.21	3.07	HUCLUM	151.191	3.015	HUCLUM	153.359	3.267
HUDCIT	152.758	3.163	HUDGAQ	157.904	3.122	HUDCAM	165.064	3.331	HUDCAM	162.298	3.135	HUDCAM	153.951	3.208
HUFHOI	167.033	2.813	HUFJIF	159.948	3.441	HUDLEB	161.044	3.411	HUDRIK	152.707	3.218	HUFBOB	156.902	3.088
HUGFEU	154.898	3.391	HUGGOJ	154.99	3.47	HUFLIF	157.245	2.974	HUFROS	158.744	3.396	HUFVEJ	150.35	2.987
HUGSEH	150.173	3.288	HUGSEH	166.235	2.974	HUGHAU	156.005	3.459	HUGHIE	167.75	3.096	HUGSEH	156.797	3.411
HUGSEL	162.962	3.309	HUGSIL	174.214	3.38	HUGSEH	173.501	2.683	HUGSEH	167.588	3.268	HUGSEL	168.982	3.016
HUGYOZ	159.788	3.371	HUHGAV	152.989	3.4	HUGSIL	177.638	3.147	HUGWOZ	151.839	3.267	HUGWOZ	153.761	2.998
HUHJAZ	151.344	2.911	HUHXAK	168.914	2.794	HUHGAV	153.439	3.473	HUHGEA	165.67	3.064	HUHHOH	151.146	3.439
HUJJOP	160.881	3.037	HUJJOP	166.302	3.477	HUHXEO	160.563	3.31	HUJJOP	159.008	3.062	HUJJOP	151.446	3.219
HUKGIG	155.724	2.761	HUKGIG	160.978	3.073	HUKFIE	162.53	3.243	HUKFIE	169.06	2.987	HUKFIE	151.714	3.117
HUKSOY	150.568	3.188	HUKSOY	157.858	3.036	HUKGIG	169.453	3.391	HUKJAC	168.356	2.957	HUKLEF	150.896	3.347
HULHEC	157.381	3.473	HULHEC	161.482	3.067	HUKSOY	162.701	3.443	HULDAT	174.379	3.421	HULHEC	168.146	3.444
HULPEK	175.671	3.105	HULPIO	151.462	3.089	HULPEK	177.413	3.087	HULPEK	174.188	3.105	HULPEK	178.887	3.087
HULRIQ	152.021	3.399	HULWAQ	154.287	2.735	HULPIO	163.265	3.432	HULPIO	166.841	2.852	HULPIO	150.561	3.38
HUMQAI	161.755	3.013	HUMQAI	169.547	3.106	HULWAQ	162.394	3.465	HUMPOY	157.262	3.293	HUMQAI	159.841	2.948
HUNBIF	151.365	2.972	HUNDIF	155.175	2.874	HUMQAI	161.243	3.177	HUMQAI	160.744	2.845	HUMQUD	175.863	3.445
HUNDIF	152.323	3.323	HUNDIF	154.119	2.847	HUNDIF	152.942	3.03	HUNDIF	168.232	3.323	HUNDIF	157.092	3.145

HUNDOL	166.408	2.958	HUNDUP	167.71	3.386	HUNDOL	171.867	2.97	HUNDOL	155.796	3.304	HUNDOL	162.71	3.125
HUNDUR	163.215	3.15	HUNDUR	155.495	3.293	HUNDUP	150.194	2.843	HUNDUP	162.86	3.1	HUNDUR	165.792	2.951
HUNFAX	155.505	2.782	HUNJEG	162.246	3.496	HUNDUR	171.329	2.972	HUNFAX	159.651	2.782	HUNFAX	150.483	3.439
HUNVOF	159.014	3.184	HUNVOF	167.65	3.204	HUNPEL	152.838	3.433	HUNPIP	153.898	3.16	HUNVOF	159.757	3.014
HUNVOF	170.887	2.998	HUNVOF	161.647	3.018	HUNVOF	165.001	3.014	HUNVOF	167.662	2.817	HUNVOF	166.486	3.351
HUNVOF0	162.601	3.202	HUNVOF0	156.391	3.484	HUNVOF0	161.592	3.472	HUNVOF0	161.649	3.009	HUNVOF0	153.83	3.009
HUNVOF0	169.459	2.991	HUNVOF0	167.984	3.364	HUNVOF0	164.168	3.18	HUNVOF0	162.941	3.202	HUNVOF0	171.115	3.141
HUNWOG	157.582	2.763	HUNWOG	161.708	2.953	HUNWAS	156.293	3.397	HUNWOG	156.164	3.474	HUNWOG	154.823	2.953
HUPSIU	154.058	3.298	HUPSIU	151.557	3.036	HUNWUM	156.656	2.952	HUPBEZ	159.209	2.937	HUPBEZ	162.346	2.668
HUPXEY	156.787	3.441	HUPYUM	161.406	3.429	HUPXEY	172.481	3.456	HUPXEY	150.286	2.724	HUPXEY	154.989	2.961
HUPYUM	150.505	2.928	HUPYUM	172.948	3.07	HUPYUM	153.888	3.456	HUPYUM	174.014	2.844	HUPYUM	167.088	2.905
HUPZAT	156.806	3.413	HUPZAT	157.449	3.437	HUPYUM	156.164	2.914	HUPYUM	167.158	2.99	HUPZAT	151.164	3.235
HUQFUU	164.169	3.149	HUQFUU	158.88	3.48	HUPZAT	150.895	2.896	HUPZAT	153.048	3.19	HUQFUU	171.994	3.101
HUQGEF	160.446	2.901	HUQGEF	153.072	3.46	HUQFUU	153.791	3.259	HUQFUU	169.74	3.219	HUQGEF	172.591	3.226
HUQRAQ	165.834	3.065	HUQTEW	164.567	3.1	HUQGEF	154.125	3.449	HUQGOQ	163.765	3.232	HUQNOY	158.337	2.856
HUQYEZ	167.3	3.436	HUQYEZ	150.24	3.442	HUQTEW	157.777	2.75	HUQTEW	171.391	3.267	HUQTEW	163.064	2.865
HURSES	155.3	2.826	HURWAV	152.786	2.934	HURDUX	150.353	2.872	HURDUX	150.41	2.873	HURLAK	170.34	2.84
HUSDEF	169.567	3.128	HUSDUY	151.952	2.896	HUSBOQ	163.777	3.119	HUSBOQ	155.098	3.324	HUSCON	150.033	3.18
HUTHEL	171.08	3.431	HUTHEN	168.01	3.25	HUSDUY	151.318	3.057	HUSWOJ	160.106	2.838	HUTHEL	158.94	2.964
HUTSIZ	151.491	3.1	HUTWAU	169.737	2.993	HUTLOB	162.331	3.097	HUTSEV	163.174	3.266	HUTSIZ	154.26	2.923
HUTWEZ	179.147	3.265	HUTXUP	161.977	2.956	HUTWAU	154.345	3.345	HUTWAU	159.037	3.178	HUTWAU	177.841	2.994
HUVDAH	156.254	3.454	HUVDAH	164.182	3.321	HUTXUP	150.534	2.994	HUVCAC	158.167	3.02	HUVDAH	157.546	3.166
HUVPAS	169.721	2.684	HUWCAF	156.058	2.905	HUVLOB	166.543	3.392	HUVNIY	163.102	3.067	HUVNIY	160.168	3.226
HUXKEQ	150.707	3.333	HUXKEQ	153.742	3.012	HUWCAF	154.7	2.905	HUXHAK	163.054	3.248	HUXKEQ	170.302	3.012
HUXLUJ	171.088	3.268	HUXLUJ	166.681	3.283	HUXKER	165.355	2.887	HUXLUJ	154.986	3.133	HUXLUJ	154.122	2.872
HUXMIY	158.994	3.24	HUXMIY	166.322	3.435	HUXLUJ	150.381	2.936	HUXLUJ	175.178	3.448	HUXMIY	160.474	2.879
HUXQAT	171.406	3.068	HUXQAT	160.889	2.806	HUXMIY	169.092	3.327	HUXMIY	171.072	3.02	HUXMIY	159.995	3.327
HUXSIC	152.141	3.231	HUXSID	159.572	3.094	HUXQAT	167.408	2.837	HUXREB	169.543	3.145	HUXSIC	160.732	2.91
HUXVOO	169.84	2.771	HUXWIJ	167.239	3.338	HUXVOO	151.45	2.771	HUXVOO	154.693	2.841	HUXVOO	153.723	2.819
HUYHUG	150.24	3.355	HUYHUG	159.594	3.015	HUXWIJ	165.999	3.229	HUXWIJ	150.277	2.792	HUXYEE	161.512	3.376
HUYMOG	155.614	2.974	HUYNIZ	167.45	2.906	HUYJAO	166.218	3.237	HUYJAO	155.331	3.343	HUYMOD	169.874	2.928
HUYQUN	156.714	3.079	HUYQUN	171.468	3.462	HUYNIZ	159.907	3.014	HUYNIZ	152.354	3.13	HUYQUN	164.187	2.825
HUYQUN	154.613	3.328	HUYQUN	158.996	3.435	HUYQUN	151.061	3.433	HUYQUN	155.326	2.942	HUYQUN	151.212	3.457
HUYREY	157.098	2.935	HUYREY	159.624	3.199	HUYQUN	165.292	3.486	HUYQUN	166.939	3.33	HUYQUN	155.835	2.79
HUYWOQ	150.833	3.268	HUYWOQ	154.088	3.375	HUYREY	158.308	2.851	HUYREY	170.255	2.989	HUYREY	167.083	2.836
HUZHIV	156.615	3.5	HUZHIV	170.41	3.433	HUYXUU	173.82	3.352	HUZFUH	150.599	2.886	HUZFUH	158.753	2.886
HUZLAR	155.714	3.137	HUZLAR	159.456	3.158	HUZHIV	154.9	2.916	HUZLAR	162.941	3.341	HUZLAR	161.172	2.838
HUZLOF	154.352	3.114	HUZRON	170.462	2.847	HUZLAR	154.609	3.072	HUZLAR	152.293	3.084	HUZLOF	151.297	3.458
HUZWAD	151.964	2.746	HUZWAD	150.464	2.925	HUZTUU	166.043	3.232	HUZVUX	154.936	3.032	HUZWAD	151.302	2.908
HUZXEJ	165.129	2.834	HUZXIN	160.935	2.793	HUZWAD	151.955	2.841	HUZWAE	163.91	2.85	HUZXEJ	167.58	2.834
HUZXUY	165.95	3.129	HUZYOU	168.143	3.271	HUZXIN	162.801	2.793	HUZXUY	157.332	3.317	HUZXUY	152.102	3.119
HUZZAH	163.004	3.352	HUZZAH	161.919	3.247	HUZYOU	163.698	2.828	HUZYOU	154.145	2.718	HUZYOU	158.98	2.951
HUZZEJ	151.597	3.042	HUZZEJ	156.899	3.14	HUZZEJ	157.02	3.157	HUZZEJ	160.902	3.294	HUZZEJ	164.661	3.067
HUZZOV	175.285	2.806	HUZZOV	151.32	2.91	HUZZEJ	167.744	2.888	HUZZEJ	150.619	3.127	HUZZIP	151.543	3.268
HUZZUB	176.496	2.857	HUZZUB	166.239	3.085	HUZZOV	156.929	2.968	HUZZUB	157.306	2.929	HUZZUB	159.109	3.37
HUZZUB	151.638	3.421	IBABUK	151.789	3.169	HUZZUB	174.626	3.114	HUZZUB	165.145	2.923	HUZZUB	158.313	2.717
IBABUK	153.532	2.897	IBABUK	167.594	2.823	IBABUK	150.151	3.186	IBABUK	164.957	3.088	IBABUK	151.386	2.672
IBAGEZ	152.388	3.277	IBARUC	156.236	2.991	IBADAR	162.089	3.265	IBADAR	159.911	3.413	IBADOF	157.151	3.32
IBAZAN	170.815	3.466	IBAZAN	153.316	3.46	IBASAJ	164.64	3.049	IBATIS	152.616	3.429	IBAZAN	151.803	2.945
IBEQOW	156.976	2.869	IBEQOW	160.194	3.312	IBAZAN	161.158	2.97	IBECEB	156.565	3.349	IBECEB	151.294	3.071
IBEQUC	150.608	3.052	IBERAJ	159.954	3.28	IBEQOW	155.505	3.373	IBEQUC	160.271	3.066	IBEQUC	160.728	3.328
IBEREN	151.987	2.831	IBEREN	157.398	2.975	IBERAJ	161.935	3.213	IBERAJ	176.76	3.263	IBERAJ	154.737	3.387
IBEREN	152.197	2.721	IBEREN	155.055	3.388	IBEREN	151.956	3.312	IBEREN	171.257	3.461	IBEREN	171.388	3.358
IBESIS	172.736	2.99	IBEZEW	160.091	2.735	IBESIS	171.935	3.094	IBESIS	169.847	3.184	IBESIS	173.392	3.184
IBEZEW	171.933	3.083	IBEZEW	150.204	3.328	IBEZEW	157.075	2.899	IBEZEW	166.589	2.986	IBEZEW	164.521	2.829
IBIRER	152.865	3.179	IBIRER	163.245	3.179	IBEZEW	167.189	3.05	IBIRAN	175.958	3.217	IBIRAN	157.577	3.117
IBOFAK	153.031	3.038	IBOKET	154.386	2.982	IBITOG	164.02	3.13	IBIYUN	161.157	2.925	IBODUC	151.361	3.235
IBONES	158.314	3.256	IBONES	168.587	2.953	IBOKET	153.33	2.701	IBOKET	173.335	3.246	IBONES	169.676	3.389
IBONES	175.291	3.335	IBONES	160.952	3.389	IBONES	154.956	2.932	IBONES	151.719	3.346	IBONES	153.837	3.26
IBOTEC	168.563	2.661	IBOTEC	164.435	3.178	IBONES	167.972	2.923	IBOSEZ	152.409	3.441	IBOSUR	152.404	3.39
IBUHEV	168.486	2.927	IBUHEV	153.074	2.856	IBOZIL	159.953	3.446	IBOZIL	159.72	3.108	IBUCER	176.826	3.1
IBUNOI	167.883	3.427	IBUNOI	168.716	3.368	IBUHEV	156.856	3.407	IBUKOH	165.596	3.29	IBUKOH	156.505	3.397
ICAHEA	152.258	3.488	ICAHOK	174.746	3.404	IBURUW	163.865	3.094	ICAHAX	154.346	2.9	ICAHAX	156.637	3.42
ICANOT	172.451	3.474	ICANOT	156.046	3.254	ICAHOK	163.364	3.236	ICANOT	171.888	3.254	ICANOT	169.11	3.015
ICAXIT	156.422	2.994	ICEDAY	153.624	3.325	ICAQEK	171.677	3.03	ICAQEK	157.272	3.483	ICAQEK	153.356	3.269
ICEFUT	168.122	2.983	ICEFUT	161.325	2.852	ICEFOL	153.711	3.424	ICEFOL	164.995	2.9	ICEFON	172.977	2.861
ICETIX	151.462	3.229	ICEWUL	170.439	3.114	ICEFUT	159.329	2.662	ICEFUT	156.744	3.306	ICEGOM	152.361	3.227
ICIHIO	163.104	3.148	ICIKAJ	167.558	2.747	ICIHEK	150.297	3.085	ICIHEK	159.075	2.979	ICIHIO	169.748	3.01
ICINEP	161.858	2.823	ICIYAV	172.374	2.807	ICIKAJ	157.369	2.965	ICIKAJ	155.233	2.805	ICIKAJ	165.289	2.747
ICIYUQ	155.298	2.891	ICIYUQ	160.735	3.178	ICIYEZ	173.48	2.942	ICIYEZ	151.065	2.958	ICIYUQ	162.246	3.178
ICOBEJ	151.914	3.338	ICOMEW	158.852	3.09	ICIYUQ	155.836	2.891	ICIYUQ	168.475	3.383	ICIYUQ	154.479	3.381
ICOVUV	151.738	2.91	ICOVUV	152.746	3.323	ICOMEW	163.932	3.095	ICOPIC	164.901	3.066	ICOTAV	150.662	2.995
ICOWOM	151.503	2.951	ICOWOM	172.686	3.022	ICOVUV	165.661	3.139	ICOVUV	150.559	2.988	ICOVUV	162.205	3.278
ICUCEQ	160.832	2.954	ICUCEQ	161.776	3.396	ICUCEQ	172.211	2.895	ICUCEQ	170.133	3.212	ICUCEQ	167.067	3.398
ICUCEQ	169.095	2.895	ICUSOQ	172.845	3.214	ICUCEQ	168.527	3.35	ICUCEQ	153.025	3.39	ICUCEQ	153.609	3.215
IDAKUW	151.991	3.305	IDAKUW	151.391	3.037	ICUTAD	169.146	3.384	IDACEX	164.247	2.918	IDAJOO	164.959	3.329
IDALAA	151.965	2.935	IDALAA	162.525	3.162	IDAKUW	161.686	3.276	IDALAA	158.714	3.146	IDALAA	152.449	2.915
IDALII	166.588	3.025	IDALII	158.726	2.901	IDALEE	150.342	3.422	IDALEE	156.189	2.968	IDALEF01	152.692	3.257
IDAPUB	158.871	2.983	IDAPUB	151.219	3.5	IDANIL	150.166	3.308	IDAPUB	153.936	3.132	IDAPUB	159.169	3.033

IDAWOC	168.4	2.862	IDAWOC	172.614	3.016	IDAVIW	177.224	2.99	IDAWES	157.62	3.442	IDAWES	166.4	3.331
IDETIY	168.027	3.261	IDEWUJ	158.752	3.071	IDAWOC	159.418	3.178	IDAWUI	171.274	3.109	IDEBEB	163.822	3.294
IDEWUJ	153.693	2.904	IDEWUJ	162.699	2.975	IDEWUJ	160.462	3.195	IDEWUJ	173.356	3.247	IDEWUJ	158.484	3.101
IDEXEU	164.988	2.884	IDEXEU	150.402	3.16	IDEWUJ	152.326	3.206	IDEXEU	163.178	3.083	IDEXEU	154.388	2.934
IDEZUM	166.605	3.371	IDEZUQ	169.623	3.224	IDEXEU	152.28	3.3	IDEXEU	152.31	3.32	IDEXOE	157.625	3.486
IDIHUZ	167.89	2.908	IDIHUZ	167.933	2.931	IDIBAC	153.835	3.135	IDICEG	151.885	3.186	IDIDOP	172.295	3.227
IDINEO	162.953	2.937	IDINEO	161.369	2.911	IDIHUZ	152.938	3.236	IDINEO	170.591	2.883	IDINEO	156.087	2.826
IDINEO	152.89	3.323	IDINEO	160.385	3.385	IDINEO	165.861	3.274	IDINEO	175.437	3.49	IDINEO	158.931	3.128
IDIPUI	175.893	2.827	IDIPUI	161.363	3.344	IDINIW	169.242	3.282	IDINUI	162.767	3.244	IDIPUI	172.922	2.827
IDIYUP	162.576	3.074	IDOFOY	152.711	3.143	IDIREW	157.074	3.321	IDIXEY	163.9	2.975	IDIYUP	161.847	3.074
IDOSEB	150.766	3.209	IDUMUS	164.071	2.871	IDOPEX	171.634	3.333	IDORUS	172.268	3.089	IDORUS	156.225	3.025
IDUPOP	164.061	3.134	IDUPOP	153.279	3.302	IDUNUS	164.693	3.3	IDUNUS	166.134	3.03	IDUPAY	173.425	3.221
IDUSUV	164.026	3.094	IDUWOX	157.691	3.056	IDUPOP	150.446	3.1	IDUPOP	159.195	2.877	IDUQIK	151.985	3.094
IFABOI	154.764	2.796	IFABOI	154.634	2.97	IFABOI	151.762	3.333	IFABOI	156.528	3.321	IFABOI	154.747	3.315
IFACEZ	150.469	2.87	IFAMUB	175.782	2.753	IFABOI	158.642	2.829	IFACEZ	165.582	2.733	IFACEZ	158.621	3.093
IFAPUD	172.805	3.161	IFASUF	163.756	3.076	IFAMUB	174.127	3.301	IFAPUD	172.997	3.206	IFAPUD	159.723	2.936
IFASUF	165.33	3.494	IFAVIY	164.445	3.188	IFASUF	156.091	3.013	IFASUF	154.201	2.836	IFASUF	162.832	3.218
IFAVOE	157.497	2.874	IFAVOE	163.181	3.105	IFAVOE	155.172	3.083	IFAVOE	151.037	3.408	IFAVOE	153.705	2.824
IFAVOE	151.82	2.884	IFAVOE	168.644	2.976	IFAVOE	152.791	2.852	IFAVOE	157.666	3.025	IFAVOE	162.185	3.114
IFAVOE	162.51	3.143	IFAVOE	153.07	3.336	IFAVOE	159.339	2.903	IFAVOE	168.405	2.653	IFAVOE	170.895	3.01
IFAWEV	150.461	3.196	IFAWEV	165.95	3.012	IFAVOE	169.22	3.421	IFAVOE	151.782	3.131	IFAWEV	159.171	3.168
IFECAX	160.017	3.079	IFECAX	171.822	3.399	IFAWEV	154.217	3.316	IFECAX	168.352	3.155	IFECAX	158.009	3.072
IFEXOI	150.947	3.058	IFEZEA	155.433	3.458	IFEJAH	166.567	3.177	IFEJAH	164.003	3.177	IFEJUA	153.672	2.945
IFEZUS	153.067	3.368	IFIBEI	153.736	2.889	IFEZEA	153.315	3.451	IFEZUS	160.447	2.772	IFEZUS	157.749	2.931
IFIBEI	154.588	2.961	IFIBEI	169.993	3.416	IFIBEI	155.167	3.456	IFIBEI	160.245	2.918	IFIBEI	172.627	3.275
IFILAM	170.458	3.147	IFILAO	154.213	2.867	IFIBEI	168.417	2.609	IFIBOQ	150.118	3.414	IFIKOZ	151.351	2.753
IFITUP	160.846	2.967	IFITUP	155.6	3.125	IFITUP	176.506	3.053	IFITUP	164.446	3.472	IFITUP	152.377	3.346
IFOFUF	163.674	3.208	IFOFUF01	163.4	3.199	IFITUP	158.43	3.119	IFOFER	152.944	3.261	IFOFER	163.764	3.043
IFOGEQ	171.253	3.414	IFOGEQ	165.636	3.451	IFOFUI	155.414	2.863	IFOGAM01	154.407	3.078	IFOGAM01	162.942	3.252
IFOHAQ	150.834	3.189	IFOHAQ	156.188	3.32	IFOHAO	173.478	3.103	IFOHAQ	163.629	3.306	IFOHAQ	172.379	2.834
IFOJET	150.596	3.165	IFOJET	152.04	3.237	IFOHAQ	155.505	3.14	IFOHIV	162.454	3.226	IFOJET	159.996	3.144
IFOJIX	158.375	3.31	IFOWAF	158.734	3.431	IFOJET	170.973	2.961	IFOJIX	152.144	3.205	IFOJIX	150.479	3.119
IFOZEJ	161.184	2.933	IFOZIQ	169.416	2.988	IFOWAF	153.659	3.045	IFOZAF	176.311	3.272	IFOZEJ	156.701	3.006
IFUBAN	152.077	3.023	IFUCEU	157.897	3.175	IFOZIQ	160.948	3.154	IFOZIQ	159.685	2.896	IFPPAU	171.425	3.439
IFUCOD	163.125	2.893	IFUCOD	157.478	3.477	IFUCIY	165.257	3.014	IFUCIY	157.777	3.014	IFUCOD	152.728	3.11
IFUCOD	170.46	3.117	IFUCUJ	168.902	3.288	IFUCOD	162.021	2.674	IFUCOD	178.377	3.073	IFUCOD	163.4	3.011
IFUDAQ	150.396	3.32	IFUDAQ	167.299	3.32	IFUCUJ	158.06	3.208	IFUCUJ	166.476	3.121	IFUCUJ	169.639	2.912
IFUDAQ	174.49	3.013	IFUDAQ	159.818	2.855	IFUDAQ	155.155	2.739	IFUDAQ	171.218	2.739	IFUDAQ	151.429	2.855
IFUDEU	161.334	2.999	IFUDIY	163.867	2.886	IFUDAQ	169.622	3.079	IFUDAR	157.813	3.196	IFUDAR	176.246	2.84
IFUDIY	151.662	3.476	IFUDIY	155.405	2.7	IFUDIY	171.145	3.074	IFUDIY	171.46	2.7	IFUDIY	172.996	2.987
IFUDUK	164.289	3.136	IFUDUK	160.152	2.857	IFUDIY	168.245	3.324	IFUDUK	174.296	3.065	IFUDUK	167.038	3.138
IFUDUL	172.064	3.073	IFUDUL	157.44	3.436	IFUDUK	150.101	3.107	IFUDUK	159.357	3.2	IFUDUK	169.729	3.099
IFUDUL	154.152	3.471	IFUDUL	169.467	3.045	IFUDUL	161.761	3.436	IFUDUL	176.443	2.731	IFUDUL	169.936	3.005
IFUFOG	156.635	3.223	IFUFOG	170.938	2.831	IFUDUL	159.283	3.471	IFUFOG	156.802	3.42	IFUFOG	161.11	3.291
IFUJOI	162.487	3.391	IFUJOI	156.409	2.768	IFUJAY	171.427	2.88	IFUJAY	154.143	2.898	IFUJOI	168.572	2.883
IFUJUO	177.283	3.133	IFUKEZ	151.048	3.014	IFUJOI	173.716	2.985	IFUJUO	158.097	2.906	IFUJUO	162.704	3.205
IFULEC	153.321	2.568	IFULEC	166.234	2.486	IFUKEZ	169.434	3.365	IFUKEZ	153.101	3.192	IFULEC	152.142	2.568
IFURAF	165.509	3.175	IFURAF	158.775	3.405	IFULEC	167.053	2.486	IFURAF	170.147	3.074	IFURAF	156.191	3.316
IGAGEC	153.337	3.164	IGAGEC	167.99	2.874	IFUVEO	176.102	3.105	IFUWIQ	167.546	3.046	IGAGEC	156.706	3.369
IGAGEC	155.988	2.975	IGAGEC	159.256	3.052	IGAGEC	167.732	3.052	IGAGEC	155.841	3.391	IGAGEC	162.843	2.875
IGAMIN	162.836	2.997	IGAPUC	167.638	3.108	IGAGEC	154.105	3.038	IGAGOP	164.978	3.352	IGAMIN	163.394	3.138
IGATES	163.474	3.156	IGATES	152.427	2.978	IGAPUC	175.596	3.086	IGARIV	150.105	3.132	IGATES	150.209	3.172
IGEGAG	157.303	2.985	IGEQUJ	168.678	3.427	IGATES	152.803	3.437	IGATES	168.768	3.112	IGATES	158.005	2.991
IGEWID02	166.91	3.342	IGEWID03	167.509	3.368	IGETEW	157.943	2.715	IGEWID	166.861	3.312	IGEWID01	168.155	3.439
IGIPIZ	174.037	2.993	IGITIC	151.743	3.473	IGEZEB	163.748	3.073	IGIPAR	150.29	2.738	IGIPAR	156.756	3.453
IGIYEG	162.113	2.852	IGIYEG	177.779	2.872	IGITIC	150.27	2.779	IGIXED	153.178	3.156	IGIXED	162.793	3.017
IGIYEG	167.15	3.407	IGIYEG	152.467	3.026	IGIYEG	177.894	3.474	IGIYEG	157.047	2.905	IGIYEG	170.548	3.331
IGIYEG	154	3.16	IGIYEG	150.408	3.032	IGIYEG	169.526	3.081	IGIYEG	173.263	3.256	IGIYEG	158.143	3.081
IGUDUJ	175.235	3.318	IGUDUJ	165.968	3.395	IGOBAI	158.799	3.368	IGOJEU	161.214	2.74	IGOVOP	159.995	2.973
IGUDUJ	154.35	3.116	IGUDUJ	162.257	3.204	IGUDUJ	159.388	3.331	IGUDUJ	168.906	3.263	IGUDUJ	166.377	3.331
IGUDUJ	158	3.081	IGUQEG	171.227	2.92	IGUDUJ	151.769	2.862	IGUDUJ	158.864	2.989	IGUDUJ	156.47	2.722
IGURAH	166.4	3.044	IGUWIQ	151.985	3.199	IGUQEG	156.071	2.92	IGUQEG	169.561	3.363	IGUQEG	153.207	3.395
IGUWIQ	160.368	3.024	IGUWIQ	150.519	3.065	IGUWIQ	163.768	2.784	IGUWIQ	156.806	2.806	IGUWIQ	163.509	2.925
IGUWUC	161.107	3.046	IHALEJ	150.821	3.428	IGUWIQ	153.42	3.264	IGUWOW	154.055	3.09	IGUWUC	163.474	3.189
IHAZOI	153.931	2.896	IHEDOO	152.07	2.978	IHAWOH	155.564	3.478	IHAWOH	161.302	3.227	IHAXEW	171.639	2.878
IHEGIM	165.594	3.276	IHEGIM	165.056	2.721	IHEFEK	161.056	3.147	IHEFEK	158.031	3.147	IHEGIM	155.559	2.861
IHELIU	174.925	3.011	IHENES	171.447	2.78	IHEGIM01	163.407	2.841	IHEGIM01	167.312	3.153	IHEHIM	175.574	2.987
IHENOC	175.299	3.338	IHEPAP	153.481	3.389	IHENES	166.449	2.78	IHENES	150.516	3.469	IHENIS	155.594	3.276
IHEPUJ	157.766	3.484	IHEPUJ	161.928	2.742	IHEPAP	158.895	3.255	IHEPES	170.705	3.272	IHEPES	173.927	2.99
IHEPUJ	162.437	2.742	IHIJIW	163.421	3.063	IHEPUJ	162.672	3.118	IHEPUJ	151.107	3.361	IHEPUJ	159.225	3.321
IHIJIW	164.88	3.034	IHIJIW	170.754	3.453	IHIJIW	152.069	3.315	IHIJIW	152.76	3.258	IHIJIW	152.331	3.235
IHINAP	169.55	2.821	IHINAP	163.723	2.973	IHIJIW	162.486	3.122	IHINAP	164.104	2.992	IHINAP	159.194	3.406
IHINET	171.352	2.834	IHINET	171.805	2.851	IHINAP	166.43	2.992	IHINAP	170.359	2.893	IHINET	171.649	2.81
IHINOD	165.668	2.992	IHINOD	172.752	2.989	IHINOD	164.697	2.922	IHINOD	171.96	2.96	IHINOD	173.713	2.832
IHIQIB	151.851	3.278	IHIQIB	171.356	3.488	IHINOD	170.191	3.101	IHINOD	155.733	3.123	IHIQIB	157.697	3.011
IHIVUT	164.259	3.468	IHIWAA	171.066	3.141	IHIQIB	153.583	3.169	IHIQIB	167.856	3.1	IHISIE	150.64	2.85
IHIZUY	159.687	3.399	IHOCIR	168.157	2.924	IHIZUY	157.828	3.117	IHIZUY	157.111	2.946	IHIZUY	168.712	3.229
IHOHUM	172.581	3.418	IHOQOL	167.166	3.478	IHOCOX	161.813	3.407	IHODER	175.732	3.377	IHOFAO	159.856	2.954
IHOTAD	163.503	2.888	IHUDOI	155.5	3.382	IHORAC	158.843	2.801	IHOSED	163.547	2.951	IHOTAD	163.862	2.888

IHUHUQ	170.772	2.965	IHUJIE	151.723	3.353	IHUDOI	150.914	3.086	IHUFAW	178.361	2.947	IHUFAW	156.778	2.573
IHUJIE	174.001	3.192	IHUJIE	167.376	3.089	IHUJIE	164.658	3.175	IHUJIE	177.256	3.233	IHUJIE	164.2	3.2
IHUJOK	151.964	2.881	IHUJOK	155.617	2.823	IHUJIE	157.243	2.742	IHUJIE	176.9	2.801	IHUJOK	150.984	2.962
IHUJOK	153.967	2.791	IHUJOK	153.527	3.178	IHUJOK	168.145	3.175	IHUJOK	151.417	2.882	IHUJOK	161.244	3.012
IHUJOK01	155.61	2.816	IHUJOK01	151.576	2.876	IHUJOK	155.442	3.24	IHUJOK01	150.372	2.888	IHUJOK01	150.889	2.95
IHUJOK01	153.706	2.781	IHUJOK01	152.599	3.148	IHUJOK01	168.172	3.154	IHUJOK01	151.496	2.87	IHUJOK01	161.522	3.005
IHUQUX01	169.207	3.19	IHUQUX01	150.404	3.03	IHUJOK01	155.654	3.214	IHUMEG	152.359	3.135	IHUMII	170.344	2.924
IHUQUX01	163.189	3.275	IHUSUC	155.546	3.399	IHUQUX01	150.726	3.385	IHUQUX01	152.293	3.31	IHUQUX01	170.84	3.123
IHUXUG	154.742	3.049	IHUXUG	158.763	2.913	IHUTOY	164.365	2.633	IHUTOY	161.283	2.633	IHUXUG	167.376	3.492
IHUXUG	164.799	3.345	IHUXUG	155.375	3.017	IHUXUG	173.302	3.053	IHUXUG	153.026	3.017	IHUXUG	155.752	3.049
IHUZAP	160.361	3.033	IHUZAP	176.678	3.308	IHUXUG	160.316	3.103	IHUYOC	175.052	3.245	IHUZAP	174.755	2.91
IJACUT	171.853	3.16	IJAGEG	150.271	2.912	IJACIJ	151.994	3.105	IJACON	158.998	3.15	IJACUT	161.968	3.454
IJAJIQ	165.073	3.239	IJASUH	151.904	3.357	IJAJAI	156.584	3.212	IJAJIQ	151.003	2.768	IJAJIQ	157.948	3.241
IJECEI	166.96	3.454	IJECEI	162.133	2.899	IJASUH	151.208	3.308	IJASUH	169.791	3.308	IJECEI	162.482	2.861
IJEGIN	150.345	3.267	IJEJUE	176.219	3.131	IJECIM01	150.015	3.489	IJECIM01	157.666	3.495	IJEGEJ	162.125	2.907
IJENES	163.317	3.158	IJENES	158.872	3.23	IJELIT	155.345	2.785	IJELUF	160.468	2.954	IJENAO	150.549	3.275
IJEPAQ	174.072	2.943	IJEPAQ	156.615	2.957	IJENES	161.464	3.278	IJENES	152.415	2.957	IJEPAQ	164.636	3.17
IJEROG	168.27	2.861	IJEROG	153.721	3.214	IJEPEU	153.859	3.02	IJEPEU	151.24	2.96	IJEROG	152.722	3.478
IJEROG	150.854	3.187	IJEROG	152.473	2.867	IJEROG	162.214	3.101	IJEROG	166.218	2.896	IJEROG	152.228	3.436
IJESIC	156.735	2.939	IJESUO	156.902	2.979	IJEROG	152.875	3.286	IJEROG	158.94	3.219	IJEROG	174.184	3.315
IJETEZ	166.847	3.355	IJETEZ	154.466	2.658	IJETEZ	159.224	3.248	IJETEZ	164.439	3.245	IJETEZ	153.663	3.426
IJIDUD	168.677	2.954	IJIFEP	164.65	2.931	IJICEN	174.713	3.218	IJIDAH	174.621	2.62	IJIDAH	151.134	3.233
IJIKET	165.469	2.732	IJIPIC	154.442	3.498	IJIHEQ	169.706	3.37	IJIKET	164.184	3.057	IJIKET	151.877	3.352
IJOHAQ	161.254	2.924	IJOHAQ	157.815	2.926	IJIYAD	164.33	3.262	IJIYAD	150.242	3.431	IJIYAD	153.329	2.968
IJOHIY	152.821	2.921	IJOHIY	158.12	3.187	IJOHAQ	158.943	2.883	IJOHAQ	151.879	2.901	IJOHIA	159.815	3.392
IJOQOR	151.464	3.296	IJOQOR	164.841	3.443	IJOHIY	167.565	3.437	IJOQAD	161.02	3.373	IJOQAD	162.81	3.336
IJUVAN	166.919	3.214	IJUVAN	160.436	3.108	IJOREF	154.652	3.445	IJUBOI	153.083	3.49	IJUGUS03	150.183	2.725
IJUXAQ	152.045	3.353	IJUXAQ	164.735	2.762	IJUXAQ	151.688	3.392	IJUXAQ	155.978	3.154	IJUXAQ	152.733	3.404
IJUXAQ	157.999	2.986	IJUXAQ	150.528	3.255	IJUXAQ	154.006	3.224	IJUXAQ	164.811	2.762	IJUXAQ	160.099	3.494
IKAYEZ	155.411	2.885	IKAYEZ	159.126	3.26	IJUXAQ	151.435	2.647	IKAQOE	153.201	2.729	IKAXUP	159.917	2.945
IKEKAL	160.749	2.846	IKEMER	150.147	3.137	IKEBUW	165.983	2.857	IKEDUZ	157.453	3.105	IKEKAL	153.754	3.097
IKEWAA	168.046	3.019	IKEWAA	166.113	2.936	IKESOK	161.975	3.401	IKETOK	170.496	2.912	IKETUQ	172.087	3.063
IKEWEE	173.077	3.2	IKEWIF	165.288	3.288	IKEWAA	168.673	3.031	IKEWEE	171.034	3.19	IKEWEE	168.734	2.863
IKEWII	164.468	2.897	IKEYUS	160.33	2.943	IKEWIF	163.065	3.481	IKEWII	165.042	2.903	IKEWII	163.425	2.871
IKIHIT	162.265	3.351	IKIHIT	157.201	2.881	IKEYUS	150.048	3.096	IKIFOX02	150.358	2.791	IKIHIT	150.455	3.317
IKIHIT	167.134	3.459	IKIKOE	158.049	2.783	IKIHIT	150.403	3.188	IKIHIT	153.547	2.927	IKIHIT	162.856	2.91
IKIYAE	150.521	2.804	IKIYAE	162.638	2.804	IKIXOR	173.208	3.179	IKIXOR	150.269	3.472	IKIXOR	158.099	2.851
IKOBOZ	154.43	3.141	IKOBOZ	153.441	3.191	IKIYAE	167.824	3.097	IKIYAE	156.449	3.133	IKIZAH	171.466	3.07
IKOBOZ	157.199	2.92	IKOBOZ	165.933	3.31	IKOBOZ	150.194	3.053	IKOBOZ	158.561	2.818	IKOBOZ	154.513	2.994
IKOBOZ	158.209	3.16	IKODAN	168.298	2.818	IKOBOZ	151.734	2.92	IKOBOZ	157.198	3.265	IKOBOZ	160.462	2.844
IKOFIY	153.679	2.81	IKOFIY	161.022	2.931	IKODIZ	164.44	3.488	IKOFAT	163.742	3.256	IKOFAT	150.526	3.232
IKOKUP	175.069	3.075	IKOKUP	158.607	3.051	IKOFOG	157.889	3.111	IKOFOG	152.352	3.004	IKOGEX	170.447	2.957
IKOLAW	160.619	3.064	IKOLAW	151.976	2.955	IKOKUP	164.293	3.487	IKOKUP	169.014	3.215	IKOLAW	174.101	3.028
IKOLEA	160.849	3.085	IKOLEA	165.619	3.163	IKOLAW	169.334	3.388	IKOLEA	156.086	3.132	IKOLEA	153.816	3.415
IKOLOK	166.357	3.164	IKOLOK	156.293	3.13	IKOLEA	153.914	3.222	IKOLEA	158.643	3.171	IKOLOK	175.388	3.318
IKOLUQ	150.871	3.279	IKOLUQ	153.946	2.869	IKOLOK	152.496	3.227	IKOLUQ	176.781	3.279	IKOLUQ	153.478	2.971
IKOMEB	164.085	2.93	IKOMEB	162.872	2.718	IKOMAX	174.697	3.401	IKOMAX	160.796	2.885	IKOMAX	175.714	3.324
IKORUZ	161.557	2.809	IKORUZ	177.353	3.025	IKOMEB	153.496	2.931	IKORUZ	152.001	2.809	IKORUZ	150.777	2.767
IKOXIP	157.737	3.193	IKOZAM	153.42	3.399	IKORUZ	158.512	2.89	IKOXEL	158.027	3.374	IKOXEL	152.74	2.988
IKOZAM	154.418	2.819	IKOZEQ	163.032	3.044	IKOZAM	173.303	3.325	IKOZAM	156.745	2.985	IKOZAM	151.221	3.086
IKOZOA	153.084	3.099	IKOZOA	153.654	3.057	IKOZEQ	173.064	3.471	IKOZIU	158.764	2.704	IKOZOA	169.177	2.757
IKUCEZ	154.991	3.43	IKUCOJ	178.428	2.859	IKOZOA	150.34	3.326	IKUBAV	159.338	2.941	IKUBAV	159.866	2.941
IKUKUV	151.59	3.138	IKULOQ	151.484	3.225	IKUCOJ	156.166	2.859	IKUFIH	150.343	3.477	IKUJON	161.546	2.881
IKUSAJ	169.98	2.832	IKUSEN	169.125	2.861	IKUMEH	167.412	3.195	IKUMIL	168.113	3.41	IKUNEL	165.574	3.303
ILAGOU	150.865	2.905	ILAGOU	163.533	3.23	IKUYEV	169.636	3.251	IKUYUK	166.205	2.973	IKUYUK	160.529	3.421
ILALEQ	174.341	3.443	ILANAM	169.877	3.167	ILAGUA	159.649	2.858	ILAGUA	166.168	3.437	ILAGUA	169.085	3.334
ILANIU	168.101	3.105	ILATIA	152.294	3.472	ILANEQ	152.418	2.805	ILANEQ	163.329	3.157	ILANIU	166.928	3.31
ILAWEY	162.043	3.026	ILAWEY	163.046	3.036	ILAVIA	162.225	3.278	ILAWEY	160.924	3.479	ILAWEY	154.817	3.007
ILAWEY	152.442	2.869	ILAWEY	157.004	3.383	ILAWEY	169.678	3.482	ILAWEY	163.348	3.103	ILAWEY	152.389	2.845
ILAWEY	159.576	3.422	ILAWEY	161.112	3.122	ILAWEY	150.15	2.822	ILAWEY	154.661	2.7	ILAWEY	161.463	2.799
ILAWEY	157.811	3.309	ILAWEY	157.575	2.99	ILAWEY	158.817	3.2	ILAWEY	167.21	3.167	ILAWEY	153.842	3.079
ILAWEY	153.95	2.709	ILAWEY	178.65	3.203	ILAWEY	153.881	3.07	ILAWEY	154.153	3.079	ILAWEY	165.645	3.309
ILAWEY	167.166	3.01	ILAWEY	154.414	3.159	ILAWEY	163.928	3.413	ILAWEY	177.501	3.295	ILAWEY	161.946	2.935
ILAWEY	168.472	3.363	ILAWEY	163.36	3.17	ILAWEY	158.095	3.434	ILAWEY	174.474	2.808	ILAWEY	166.396	3.272
ILAWEY	160.823	3.328	ILAXAX	165.627	3.287	ILAWEY	163.463	2.775	ILAWEY	152.329	3.177	ILAWEY	155.418	3.352
ILAYIG	167.948	3.128	ILAYOM	162.355	3.314	ILAXAX	153.429	2.949	ILAXAX	178.447	2.886	ILAYIG	159.195	3.145
ILEJEO	158.704	2.931	ILEJEO	170.265	2.825	ILAYOM	156.372	3.39	ILEHAI	162.989	3.328	ILEJEO	154.717	2.825
ILEMAO	168.404	2.741	ILEMIW	157.005	2.89	ILEJOA	157.156	3.473	ILELAN	152.611	3.029	ILELUH	160.361	3.271
ILESOK	167.899	3.384	ILESOK	155.595	3.36	ILERUP	153.22	3.132	ILESOK	172.717	3.24	ILESOK	150.867	3.384
ILESOK	174.08	3.124	ILESOK	150.557	2.781	ILESOK	155.326	2.883	ILESOK	163.821	3.303	ILESOK	161.679	2.858
ILIHET	170.57	2.963	ILIQID	151.981	3.164	ILESOK	150.281	2.919	ILESOK	152.115	2.742	ILESOK	154.678	2.858
ILOCIY	169.439	3.021	ILOCIY	156.252	3.364	ILISUU	151.16	3.175	ILIVIL	171.383	3.29	ILOCIY	150.103	3.148
ILOQIN	173.955	3.114	ILOQIN	150.162	3.284	ILOCIY	174.734	3.298	ILOCIY	165.774	3.483	ILOCIY	150.139	2.995
ILOQIN01	173.92	3.109	ILOQIN01	150.287	3.278	ILOQIN	150.73	2.846	ILOQIN	166.992	2.85	ILOQIN	168.542	2.858
ILOQOT	155.874	2.978	ILOQOT	155.813	2.994	ILOQIN01	150.76	2.845	ILOQIN01	167.02	2.847	ILOQIN01	168.821	2.854
ILOQOT	162.783	3.428	ILUBIC	152.102	2.848	ILOQOT	158.025	2.617	ILOQOT	167.599	2.886	ILOQOT	162.295	2.829
ILUDUQ	171.247	3.215	ILUDUQ	154.56	3.331	ILUBOI	158.316	3.218	ILUDUQ	169.397	3.115	ILUDUQ	158.172	2.709
ILUDUQ	159.97	2.939	ILUDUQ	169.106	3.18	ILUDUQ	157.155	3.158	ILUDUQ	164.045	3.265	ILUDUQ	152.456	2.74
ILUDUQ	158.775	3.457	ILUDUQ	156.439	2.931	ILUDUQ	173.201	2.993	ILUDUQ	159.076	2.918	ILUDUQ	174.937	2.682

ILUDUQ	170.255	3.38	ILUDUQ	152.548	2.816	ILUDUQ	153.491	2.709	ILUDUQ	169.757	2.727	ILUDUQ	157.319	2.931
ILUDUQ01	175.496	2.663	ILUDUQ01	153.609	2.693	ILUDUQ	159.183	3.02	ILUDUQ01	163.713	3.195	ILUDUQ01	158.508	3.445
ILUDUQ01	159.899	2.99	ILUDUQ01	156.362	3.08	ILUDUQ01	157.006	2.931	ILUDUQ01	170.067	2.707	ILUDUQ01	150.092	2.894
ILUDUQ01	154.526	2.722	ILUDUQ01	168.948	3.194	ILUDUQ01	171.079	3.194	ILUDUQ01	169.178	3.109	ILUDUQ01	170.396	3.353
ILUDUQ01	158.307	2.693	ILUDUQ01	154.708	3.314	ILUDUQ01	151.545	2.779	ILUDUQ01	156.333	2.931	ILUDUQ01	159.05	2.875
ILUJEH	165.023	2.69	ILUJEH	165.87	3.244	ILUDUQ01	173.725	2.934	ILUDUQ01	160.408	2.89	ILUJEH	154.417	2.872
ILUJEH	150.074	2.828	ILUJEH	151.374	2.879	ILUJEH	159.393	3.344	ILUJEH	157.223	3.229	ILUJEH	160.652	3.498
ILUJEH	150.689	2.862	ILUJEH	167.918	3.391	ILUJEH	158.735	2.777	ILUJEH	160.344	2.73	ILUJEH	156.33	3.492
ILUJOR	156.314	2.736	ILUJOR	152.079	3.494	ILUJEH	167.621	2.916	ILUJOR	158.184	2.781	ILUJOR	155.812	3.3
ILUJOR	175.403	3.468	ILUJOR	156.099	3.24	ILUJOR	167.31	3.084	ILUJOR	158.34	2.82	ILUJOR	155.299	2.781
ILUJUX	172.697	2.98	ILUJUX	167.425	3.367	ILUJOR	150.107	2.96	ILUJOR	170.982	2.993	ILUJUX	172.532	3.039
ILUJUX	170.233	3.189	ILUJUX	152.888	2.862	ILUJUX	159.205	2.923	ILUJUX	156.553	2.862	ILUJUX	164.173	3.456
ILUKAE	161.725	2.822	ILUKAE	154.695	3.366	ILUJUX	175.329	3.354	ILUJUX	174.263	3.166	ILUJUX	173.191	3.15
ILUKAE	170.303	2.822	ILUKAE	169.804	3.297	ILUKAE	173.823	3.189	ILUKAE	158.696	2.854	ILUKAE	160.764	3.198
ILUVIX	162.336	2.889	ILUVIX	176.711	2.928	ILUKAE	175.44	3.1	ILUKIJ	169.158	2.742	ILUKIJ01	169.598	2.71
ILUZEV	154.373	3.081	ILUZIZ	154.655	3.091	ILUVIX	157.115	3.069	ILUVIX	150.347	3.14	ILUYAR	159.114	3.232
IMADEF	163.557	3.018	IMAFAD	162.288	3.196	IMADEF	171.264	3.297	IMADEF	151.714	2.676	IMADEF	169.475	3.077
IMAFAD	164.248	2.91	IMAFAD	163.958	3.39	IMAFAD	165.18	2.816	IMAFAD	162.836	3.044	IMAFAD	175.945	2.983
IMARUK	165.624	3.267	IMAXAZ	166.713	3.136	IMAFAD	158.282	3.196	IMAFAD	168.832	3.001	IMARUK	166.66	3.493
IMECAI	164.632	3.232	IMECAI	158.093	3.453	IMECAI	168.639	2.77	IMECAI	162.685	2.769	IMECAI	151.15	2.801
IMEJIX	153.699	3.329	IMEJOB	160.796	2.739	IMEFEN	168.139	3.119	IMEJAL	162.602	2.898	IMEJIT	166.094	2.939
IMEPAU	170.53	3.134	IMEPAU	165.693	2.981	IMEJOB	150.151	3.31	IMENUM	162.756	2.911	IMENUM	172.145	3.095
IMEPAU	153.045	3.034	IMEPAU	162.572	3.087	IMEPAU	164.431	2.981	IMEPAU	158.222	2.979	IMEPAU	163.461	3.28
IMESAV	161.312	3.461	IMESAV	159.186	2.888	IMESAV	172.291	2.823	IMESAV	162.188	3.398	IMESAV	178.906	2.93
IMEVAZ	162.185	3.074	IMIBOY	157.005	3.341	IMESEZ	179.134	3.174	IMESEZ	160.891	3.103	IMESEZ	172.452	2.768
IMIGIV	164.792	3.148	IMIGIV	154.045	2.872	IMICUC	156.105	3.122	IMIGIV	159.415	3.448	IMIGIV	156.554	2.971
IMIGOB	170.967	3.341	IMIGUH	161.131	2.828	IMIGOB	159.844	3.085	IMIGOB	172.881	3.292	IMIGOB	159.553	3.152
IMIGUH	156.112	2.828	IMIHAO	159.34	3.222	IMIGUH	158.251	2.882	IMIGUH	154.266	3.106	IMIGUH	168.46	3.417
IMIJAQ	164.734	3.333	IMIJAQ	173.801	3.403	IMIHAO	155.277	3.105	IMIHAO	167.486	3.114	IMIJAQ	175.953	3.15
IMINUN	170.692	2.773	IMINUN	168.476	3.232	IMINUN	150.144	2.861	IMINUN	154.049	2.769	IMINUN	150.956	2.817
IMINUN	152.672	3.217	IMINUN	154.413	3.477	IMINUN	156.683	2.95	IMINUN	163.078	2.674	IMINUN	167.255	2.961
IMINUN	173.489	3.249	IMINUN	177.227	2.911	IMINUN	153.022	2.674	IMINUN	151.592	2.721	IMINUN	166.325	2.895
IMINUN	174.037	2.906	IMINUN	152.193	3.075	IMINUN	167.748	2.81	IMINUN	162.627	2.93	IMINUN	166.294	3.083
IMIWOT	173.094	2.781	IMIWOT	160.027	2.964	IMINUN	153.408	2.925	IMINUN	153.27	2.851	IMISIJ	167.61	2.992
IMIZEL	163.931	2.969	IMIZEL	153.023	2.93	IMIZEL	164.417	2.945	IMIZEL	170.511	3.123	IMIZEL	156.717	2.706
IMIZEL	170.427	2.931	IMIZOV	152.252	3.192	IMIZEL	167.963	3.294	IMIZEL	151.981	3.493	IMIZEL	154.195	2.803
IMOBOC	162.393	3.454	IMOBOC	154.385	3.044	IMOBES	152.092	3.161	IMOBES	158.197	3.382	IMOBIY	160.654	3.377
IMOBUK	150.231	3.049	IMOCAP	160.248	3.324	IMOBOC0	162.662	3.421	IMOBOC0	155.085	3.009	IMOBOE	150.309	3.076
IMOCIX	167.792	3.433	IMOCIZ	163.391	2.887	IMOCAP	153.557	2.81	IMOCAP	155.521	3.11	IMOCAP	166.628	2.833
IMONIJ	159.309	3.147	IMONIJ	150.426	3.031	IMOCIZ	166.476	2.887	IMODAS	159.967	3.368	IMOMAZ	153.66	3.265
IMONIJ	158.89	3.465	IMONIJ	166.459	3.38	IMONIJ	151.047	3.493	IMONIJ	151.11	3.013	IMONIJ	153.835	3.361
IMONOP	155.991	2.957	IMONOP	179.201	2.685	IMONIJ	156.974	3.195	IMONIJ	154.07	2.901	IMONOP	161.586	3.092
IMONOP	150.216	3.26	IMONOP	172.418	3.182	IMONOP	161.665	3.337	IMONOP	170.973	3.153	IMONOP	151.836	3.124
IMOVUC	157.643	3.342	IMOYIT	175.89	3.484	IMONOP	157.295	2.921	IMOQOU	158.838	2.927	IMOQOU	153.478	3.058
IMUCAX	171.627	3.152	IMUCAX	151.64	2.856	IMUCAX	150.134	3.412	IMUCAX	169.434	3.296	IMUCAX	162.077	3.334
IMUMEK	150.136	3.082	IMUMEK	165.163	3.031	IMUDEC	156.306	3.267	IMUDEC	157.978	3.06	IMUDEC	168.33	2.995
INASOG	171.678	3.005	INDRHB	156.248	3.483	IMUMEK	155.265	3.444	IMUTOA	163.764	2.757	INAGUZ	152.096	3.412
INEBAE	163.357	3.444	INEFIQ01	165.341	3.476	INEBAE	170.08	3.059	INEBAE	173.31	3.219	INEBAE	150.839	3.029
INEKIX	154.711	3.145	INEKIX	150.459	3.239	INEFIQ01	162.625	2.913	INEKIX	168.661	3.383	INEKIX	168.646	3.236
INEZUZ	172.131	3.053	INIMIC	151.37	3.247	INEKIX	151.341	3.09	INEKIX	172.984	2.857	INELUK02	154.96	2.699
INOBIW	150.395	3.271	INOKAY	165.005	3.392	INIXUY	156.366	3.467	INOBEU	151.491	2.995	INOBEU	168.57	3.241
INOKIG	151.791	3.065	INOLOP	157.883	3.332	INOKAY	174.858	2.893	INOKAY	157.665	2.932	INOKIG	155.096	2.941
INOPAE	165.485	3.218	INOPIN	153.799	3.188	INONAC	156.324	3.478	INONOQ	152.691	3.282	INONUW	166.384	3.446
INUZIC	170.831	3.053	IPABEG	167.128	2.921	INUKUX	153.784	3.324	INUKUX	155.209	3.38	INUTOC	161.663	2.612
IPAJIT	153.703	2.736	IPAJUF	164.051	2.818	IPAFOV	166.654	3.493	IPAJIT	158.363	2.736	IPAJIT	150.216	3.242
IPAJUF	164.433	3.331	IPAKEQ	159.046	3.137	IPAJUF	158.915	3.331	IPAJUF	152.96	2.818	IPAJUF	171.501	3.008
IPAKIU	152.944	2.806	IPAKIU	163.488	2.806	IPAKEQ	164.792	3.137	IPAKIU	163.017	3.323	IPAKIU	170.866	2.979
IPAPIA	160.834	2.828	IPAPIA	162.102	2.733	IPAKIU	159.017	3.323	IPAPIA	164.755	2.733	IPAPIA	168.434	3.424
IPAPOG	164.349	2.872	IPAPOG	151.377	3.127	IPAPIA	154.564	2.828	IPAPIA	162.656	2.692	IPAPIA	161.475	2.887
IPASAW	165.258	3.491	IPASAW	151.702	3.328	IPAPOG	152.238	3.455	IPAPOG	153.559	3.045	IPARAU	169.483	2.924
IPAWII	162.592	3.173	IPAWII	171.491	2.742	IPASAW	157.217	3.379	IPASIE	163.933	3.237	IPASIE	156.42	3.347
IPENEZ	164.322	3.381	IPENEZ	156.735	3.443	IPELUK	165.927	3.21	IPEMIZ	159.676	3.093	IPEMIZ	156.792	3.037
IPIFUI	158.266	3.317	IPIFUI	150.287	3.098	IPIDIV	153.653	3.059	IPIDIV	159.681	3.059	IPIDIV	171.045	3.497
IPIGAP	155.121	3.116	IPIGET	164.35	3.231	IPIGAP	161.444	3.145	IPIGAP	173.333	2.956	IPIGAP	171.925	3.433
IPIRAC	175.867	2.911	IPIRAC	162.695	3.03	IPIGET	174.761	3.113	IPIGUJ	152.026	3.419	IPIGUJ	156.002	3.449
IPOSOX	158.281	3.234	IPOSOX	166.867	3.366	IPIROO	152.391	3.146	IPONUX	156.452	2.904	IPONUX	169.851	2.904
IPOSOX	168.22	2.856	IPOSOX	167.232	3.321	IPOSOX	151.786	3.234	IPOSOX	161.309	2.835	IPOSOX	151.295	2.768
IPOSOX	160.005	2.894	IPOSUD	157.447	3.025	IPOSOX	160.838	3.431	IPOSOX	157.369	3.231	IPOSOX	164.592	3.35
IPOVUF	172.829	3.363	IPUDEE	150.155	3.016	IPOSUD	161.587	3.007	IPOSUD	151.204	2.896	IPOSUD	157.141	3.071
IPUDEE	168.18	2.83	IPUDEE	152.741	2.938	IPUDEE	156.321	3.208	IPUDEE	153.721	3.226	IPUDEE	169.34	3.142
IPUFUX	164.417	2.871	IPUFUX	166.426	3.205	IPUDEE	168.481	3.04	IPUDII	153.116	3.18	IPUDII	153.515	2.868
IPUHIL	151.129	3.256	IPUHIL	168.977	3.365	IPUFUX	163.995	2.871	IPUGAE	151.414	3.318	IPUGAE	154.144	3.362
IPUSIW	154.874	2.87	IPUYOH	167.21	3.427	IPUJOV	155.921	3.04	IPUJOV	154.42	3.4	IPUSIW	166.886	2.673
IQAJOZ	150.977	3.494	IQAJOZ	166.392	3.261	IPUYOH	167.902	3.427	IQABEH	151.053	3.448	IQAJOZ	160.939	2.802
IQAJOZ01	166.459	3.275	IQAJOZ01	173.421	3.12	IQAJOZ	156.036	3.205	IQAJOZ	173.179	3.124	IQAJOZ	154.854	2.849
IQALOD	157.141	2.791	IQAPOH	150.934	3.329	IQAJOZ01	156.039	3.217	IQAJOZ01	154.827	2.868	IQAJOZ01	161.005	2.814
IQEBEL	165.024	3.086	IQEBEL	155.409	3.343	IQATUS	170.432	3.275	IQAZOQ	161.293	3.374	IQAZOQ	157.73	3.457
IQEPEA	175.582	3.121	IQEPEA	165.109	3.124	IQELIA	164.053	2.929	IQEPEA	161.786	3.246	IQEPEA	164.965	2.769
IQETAB	165.293	2.728	IQEVOS	168.458	3.169	IQEPEA	163.905	2.917	IQEQIH	160.132	3.425	IQEQIH	153.673	2.811

IQEWUX	161.381	3.04	IQICER	156.498	3.233	IQEWIK	169.172	2.821	IQEWIM	172.78	3.117	IQEWIM	151.852	3.275
IQIDOD	161.814	3.276	IQIDOD	161.594	2.98	IQICER	150.41	3.001	IQICER	155.37	3.311	IQIDOD	151.707	3.346
IQIDOD	179.64	2.987	IQIMAY	151.811	3.056	IQIDOD	163.354	2.838	IQIDOD	168.593	3.025	IQIDOD	170.853	2.86
IQIQUW	158.528	2.907	IQIQUW	154.931	2.898	IQIMAY	172.268	3.15	IQIQUW	172.232	2.803	IQIQUW	169.009	2.897
IQIZIR	160.371	2.78	IQIZIR	162.692	3.477	IQIRIL	156.187	3.47	IQIRIL	151.459	2.756	IQIZIR	174.921	2.78
IQIZOA	155.831	2.798	IQIZOA	166.432	3.078	IQIZIR	156.866	2.864	IQIZIR	164.244	3.161	IQIZOA	150.289	2.795
IQIZOA	165.072	3.077	IQIZOX	160.876	3.482	IQIZOA	161.225	3.131	IQIZOA	152.943	2.694	IQIZOA	151.198	2.795
IQOYEV	155.315	3.025	IQUCII	152.29	3.283	IQOGUS	172.034	3.47	IQOLEF	156.988	3.348	IQOLEF	162.376	3.221
IQUROD	159.236	2.953	IQUZID	150.611	3.358	IQUCII	163.674	3.283	IQUCII	153.684	3.048	IQUROD	152.496	2.943
IRAKES	169.515	3.326	IREDUE	153.378	3.383	IQUZOJ	152.236	3.236	IRAKES	153.619	2.98	IRAKES	174.765	3.253
IREFEQ	150.041	2.962	IREFEQ	159.902	3.247	IREDUE	165.749	3.093	IREDUE	163.72	2.999	IREDUE	161.354	3.234
IREYUC	164.9	3.093	IREYUC	150.37	3.498	IREFEQ	151.18	2.977	IREQIG	163.808	3.12	IREYEJ	169.495	3.278
IROBAU	168.702	3.416	IROCAV	172.872	2.937	IRIFEU	176.693	3.041	IRIFEU	159.591	3.432	IRILAW	164.396	3.351
IROCEZ	155.695	2.761	IROCEZ	151.631	2.799	IROCEZ	156.602	2.761	IROCEZ	159.083	2.852	IROCEZ	158.718	2.832
IROCOJ	176.67	3.044	IROCOJ	158.807	2.762	IROCEZ	153.046	3.363	IROCIE	151.496	3.126	IROCOJ	163.761	3.499
IROCOJ	159.23	2.762	IROCOJ	155.928	3.295	IROCOJ	155.5	3.227	IROCOJ	174.207	3.149	IROCOJ	151.844	3.158
IROQIS	171.337	3.211	IROYET	176.505	3.216	IROCOJ	155.541	2.991	IROCOJ	150.496	3.14	IROGUU	166.816	3.149
IROYET	152.886	2.901	IROYET	171.188	3.158	IROYET	151.737	3.023	IROYET	161.343	3.028	IROYET	151.085	3.222
IROYET01	161.318	3.029	IROYET01	151.123	3.22	IROYET	170.492	3.272	IROYET01	176.436	3.213	IROYET01	151.649	3.025
IRUBOP	170.185	2.962	IRUCEF	159.581	3.469	IROYET01	152.781	2.899	IROYET01	171.266	3.158	IROYET01	170.494	3.272
IRUFAE	151.85	3.128	IRUFAE	150.62	2.646	IRUCEF	151.97	3.26	IRUCEF	152.074	3.223	IRUDUW	155.348	3.092
IRUMEN	177.882	3.07	IRUMEN01	159.569	3.177	IRUFAE	173.459	3.203	IRUKAK	167.507	3.414	IRUKAK	164.161	2.875
IRUREU	169.67	3.047	IRUREU	150.435	3.429	IRURAQ	166.611	2.758	IRURAQ	167.079	2.758	IRUREU	162.712	2.932
IRUZIH	157.91	2.994	IRUZIH	167.037	3.017	IRUZAZ	158.767	3.164	IRUZED	156.9	3.22	IRUZIH	162.781	3.307
IRUZON	160.939	3.385	IRUZON	174.562	2.716	IRUZIH	172.335	2.672	IRUZON	165.893	3.044	IRUZON	159.531	3.056
IRUZUT	152.283	3.125	ISABAI	160.745	3.388	IRUZON	150.485	2.824	IRUZUT	174.222	3.427	IRUZUT	171.689	3.19
ISABAI	150.013	2.834	ISADIP	171.681	3.247	ISABAI	160.341	3.062	ISABAI	165.658	3.051	ISABAI	174.615	2.723
ISAHEP	158.514	3.346	ISANAS	156.661	2.899	ISADIP	154.021	3.164	ISADOV	158.376	3.374	ISADOV	163.266	3.296
ISANIB	152.836	3.487	ISANIB	150.859	2.869	ISANAS	155.393	2.926	ISANAS	155.037	2.729	ISANIB	162.032	2.707
ISAYEJ	160.491	3.103	ISAYEJ	173.585	3.472	ISANIB	154.286	3.343	ISAQUQ	151.057	3.158	ISAYEJ	151.465	3.199
ISAYEJ	176.669	3.01	ISAYEJ	177.968	3.385	ISAYEJ	171.141	3.005	ISAYEJ	165.705	3.204	ISAYEJ	160.276	3.364
ISIBOE	164.537	3.494	ISIBOE	165.354	2.901	ISEBOZ	158.95	3.162	ISEGUJ	155.418	3.307	ISEHUK	159.344	3.281
ISICEV	161.607	2.742	ISICEV	151.223	2.972	ISIBOE	153.183	3.196	ISICAR	151.385	2.995	ISICAR	163.484	2.81
ISICIZ	162.545	2.898	ISIDAS	152.426	3.207	ISICEV	155.317	3.346	ISICEV	162.883	3.321	ISICEV	156.688	3.209
ISIDEW	160.791	2.86	ISIDEW	162.596	2.874	ISIDAS	150.666	3.142	ISIDEW	163.022	2.798	ISIDEW	153.704	3.126
ISIDIA	156.632	2.845	ISIDIA	155.856	2.875	ISIDEW	175.9	3.083	ISIDEW	166.093	3.269	ISIDIA	169.836	3.127
ISIKAY	152.832	3.362	ISIPIJ	168.76	3.49	ISIJEZ	165.34	3.153	ISIJEZ01	165.05	3.3	ISIJOJ	177.532	3.425
ISOTES	161.906	2.903	ISOTES	166.121	3.061	ISIPIJ	170.435	3.428	ISOJEI	151.322	3.031	ISOTES	150.85	2.903
ISOZOI	156.871	3.252	ISUBOQ	164.303	3.429	ISOZIB	150.646	3.092	ISOZIB	165.213	3.014	ISOZIB	151.495	3.17
ISUSUK	157.962	3.182	ISUZUT	165.978	3.176	ISUBUW	161.764	3.116	ISUCAD	157.527	2.7	ISUCIL	164.707	3.01
ISUZUT	161.619	3.174	ISUZUT	168.965	3.102	ISUZUT	154.036	2.97	ISUZUT	151.344	2.939	ISUZUT	160.323	3.216
ISUZUT01	155.633	2.909	ISUZUT01	160.037	2.982	ISUZUT	159.513	3.304	ISUZUT	154.368	3.46	ISUZUT01	168.822	3.041
ITAJIX	156.508	3.203	ITAJIX	154.81	2.943	ISUZUT01	154.488	2.73	ISUZUT01	150.745	3.09	ITAFEO	160.816	3.246
ITAJIX	154.037	2.899	ITAJIX	177.02	2.851	ITAJIX	150.456	2.787	ITAJIX	161.363	2.806	ITAJIX	168.067	3.334
ITAJIX	152.999	3.132	ITAJIX01	165.614	3.456	ITAJIX	158.252	3.324	ITAJIX	179.191	3.098	ITAJIX	168.017	3.274
ITAJIX01	172.682	3.024	ITAJIX01	175.197	2.968	ITAJIX01	163.623	3.126	ITAJIX01	161.91	3.405	ITAJIX01	163.496	2.968
ITAJIX01	170.443	2.722	ITAJIX01	171.283	3.072	ITAJIX01	166.442	2.992	ITAJIX01	165.652	2.924	ITAJIX01	167.982	3.398
ITAJIX02	161.347	3.451	ITAJIX02	163.249	3.028	ITAJIX01	165.033	3.145	ITAJIX02	168.949	3.484	ITAJIX02	165.751	3.144
ITAJIX02	166.369	2.97	ITAJIX02	169.18	3.416	ITAJIX02	170.862	3.074	ITAJIX02	174.094	2.985	ITAJIX02	168.265	2.979
ITAJIX02	169.966	3.109	ITAJIX02	165.363	3.121	ITAJIX02	151.369	3.402	ITAJIX02	167.656	3.426	ITAJIX02	169.795	2.747
ITAQOJ	161.272	3.095	ITAQOJ	154.702	3.222	ITAMEX	150.591	3.224	ITAQOJ	154.836	3.027	ITAQOJ	161.07	3.079
ITAQOJ	150.622	2.932	ITARIF	159.859	3.479	ITAQOJ	163.374	3.164	ITAQOJ	152.257	3.303	ITAQOJ	169.907	3.413
ITEBUF	150.528	3.028	ITEBUF	172.325	3.215	ITAWAD	163.011	3.381	ITAWAD	162.476	3.084	ITEBUF	155.688	3.125
ITEDUJ	158.906	3.231	ITEDUJ	175.286	2.96	ITEDUJ	159.032	3.445	ITEDUJ	161.213	3.059	ITEDUJ	151.182	2.877
ITEFAR	163.984	3.178	ITEHAS	170.232	3.343	ITEFAP	161.661	3.497	ITEFAR	156.009	3.054	ITEFAR	175.173	3.088
ITEKOI	169.597	3.059	ITEKOI	154.115	2.995	ITEKIC	155.053	3.354	ITEKIC	173.208	2.93	ITEKOI	172.753	2.808
ITEKUO	162.935	2.809	ITEKUO	154.567	3.399	ITEKUO	151.395	3.048	ITEKUO	162.915	3.048	ITEKUO	162.727	2.811
ITENUT	160.367	3.048	ITENUT	159.989	2.873	ITEKUO	164.974	2.884	ITEMEA	167.594	2.991	ITENUT	156.507	3.323
ITEPAB	175.903	3.284	ITEPAB	161.399	3.018	ITENUT	154.645	3.02	ITENUT	153.912	2.958	ITENUT	174.422	3.21
ITEPAB	161.193	3.409	ITEPAB	168.994	2.955	ITEPAB	169.336	3.078	ITEPAB	159.537	2.793	ITEPAB	161.51	3.213
ITEPEF	161.935	3.201	ITEPEF	169.052	3.498	ITEPAB	162.821	3.465	ITEPEF	153.34	3.188	ITEPEF	168.816	2.919
ITEPIJ	175.644	3.313	ITEPIJ	173.687	2.707	ITEPEF	170.292	3.032	ITEPIJ	154.086	2.885	ITEPIJ	157.935	3.354
ITEPIJ	155.62	2.949	ITEPIJ	152.871	3.193	ITEPIJ	166.456	3.119	ITEPIJ	160.119	3.472	ITEPIJ	151.104	3.053
ITEPOP	152.104	3.474	ITEPOP	167.371	2.917	ITEPOP	153.312	3.213	ITEPOP	154.428	3.012	ITEPOP	163.526	3.239
ITERUV	175.666	3.348	ITERUV	157.353	2.851	ITEROP	173.734	3.266	ITEROP	150.866	2.831	ITEROP	158.069	3.358
ITESAC	158.239	3.389	ITESAC01	176.707	3.187	ITERUV	152.052	3.337	ITESAC	176.707	3.187	ITESAC	151.405	2.769
ITEXUB01	152.728	3.221	ITEXUB01	151.674	3.441	ITESAC01	151.405	2.769	ITESAC01	158.239	3.389	ITESAE	151.825	3.062
ITEYEO	180	2.971	ITEYEO	180	2.971	ITEXUB01	151.087	3.154	ITEXUB01	150.139	3.468	ITEXUB03	152.171	3.094
ITICOD	157.315	3.21	ITICOD	166.625	2.849	ITEYUE	157.169	3.285	ITEYUE	166.479	3.127	ITICOD	167.176	2.834
ITICOD	162.092	2.926	ITIFEZ	151.972	2.81	ITICOD	152.624	3.366	ITICOD	151.146	3.491	ITICOD	178.576	2.792
ITIFEZ	168.533	2.684	ITIFEZ	163.991	3.137	ITIFEZ	167.909	3.45	ITIFEZ	154.266	3.159	ITIFEZ	167.223	2.815
ITIFEZ	150.612	3.324	ITIFEZ	154.884	2.749	ITIFEZ	152.915	3.326	ITIFEZ	154.371	3.338	ITIFEZ	167.039	3.345
ITIRUA	151.851	3.4	ITIRUA	152.972	3.349	ITIKEB	170.822	2.723	ITINEF	152.31	3.449	ITINIK	172.787	3.01
ITIZET	161.413	3.002	ITIZET	172.215	3.171	ITIRUA	169.409	3.4	ITIZAP	167.113	3.385	ITIZET	165.774	2.964
ITOLUB	156.817	2.686	ITOLUB	163.761	3.325	ITIZET	155.64	3.182	ITODIH	170.488	3.263	ITOLUB	159.036	2.686
ITONIP	150.55	2.938	ITONIP	157.41	2.868	ITOLUB	150.212	2.969	ITOMAH	162.312	3.37	ITONIP	164.466	3.237
ITONOV	150.845	3.208	ITONOV	165.646	3.304	ITONIP01	164.472	3.239	ITONIP01	157.404	2.869	ITONIP01	150.534	2.939
ITONOV	172.864	2.988	ITONOV	152.627	2.845	ITONOV	164.224	3.087	ITONOV	161.016	3.356	ITONOV	158.2	2.763
ITONOV01	165.621	3.3	ITONOV01	152.771	2.813	ITONOV	176.378	2.764	ITONOV01	150.914	3.185	ITONOV01	156.498	2.744

ITONOV01	161.187	3.304	ITONOV02	153.87	2.61	ITONOV01	173.104	2.971	ITONOV01	176.332	2.738	ITONOV01	164.663	3.068
ITONOV02	158.111	2.786	ITOPEN	164.188	3.339	ITONOV02	152.064	3.442	ITONOV02	174.416	3.411	ITONOV02	159.33	3.383
ITUBIK	153.671	3.32	ITUBUX	163.591	2.875	ITOPEN	155.142	3.403	ITUBAC	158.98	3.077	ITUBAC	150.864	3.311
ITUGEL	167.92	3.224	ITUGEL	150.348	3.353	ITUCAE	167.456	3.189	ITUCUY	150.997	2.972	ITUFUZ	151.325	3.193
ITUPOD	158.82	3.447	ITUPOD	165.383	3.447	ITULIU	165.602	3.208	ITULIU01	165.674	3.205	ITUPAP	155.457	2.904
ITUQOE	160.998	3.38	ITUSUL	164.22	3.059	ITUQAQ	167.185	3.158	ITUQAQ	170.339	3.158	ITUQOE	167.686	3.496
ITUZIG01	174.397	2.926	IVADOA	150.999	3.393	ITUSUL	152.584	3.272	ITUSUL	175.136	3.054	ITUZIG	177.387	2.927
IVADOA	157.98	3.013	IVADOA	150.051	2.8	IVADOA	158.017	3.001	IVADOA	156.72	3.184	IVADOA	151.156	3.231
IVADOA	169.103	3.051	IVADOA	154.943	2.9	IVADOA	154.788	2.953	IVADOA	176.075	2.977	IVADOA	153.322	2.877
IVAREE	174.757	2.636	IVAREE	170.779	3.055	IVAKIB	162.412	3.449	IVAQEE	167.092	3.235	IVAREE	159.034	3.186
IVEDIX	171.173	3.078	IVESAF	166.321	3.241	IVAREE	166.557	3.218	IVAWIN	165.592	2.927	IVEDIX	154.365	3.034
IVETIM	166.497	3.402	IVETIM	169.196	3.117	IVESAF	158.96	3.241	IVETIM	156.718	2.876	IVETIM	171.974	3.384
IVETOS	167.154	3.478	IVETOS	158.638	3.262	IVETIM	152.839	2.971	IVETOS	154.829	3.197	IVETOS	160.225	3.17
IVETOS	161.644	3.243	IVETOS	157.054	3.331	IVETOS	162.961	3.254	IVETOS	165.219	3.265	IVETOS	156.609	3.171
IVETUY	162.39	3.373	IVETUY	167.406	3.221	IVETOS	162.923	2.873	IVETOS	154.161	3.134	IVETUY	161.511	3.412
IVETUY	155.437	3.223	IVETUY	166.388	3.32	IVETUY	170.049	3.293	IVETUY	167.744	3.334	IVETUY	161.465	3.409
IVETUY	151.028	2.933	IVETUY	153.896	3.169	IVETUY	157.918	2.933	IVETUY	169.166	3.34	IVETUY	163.927	2.665
IVETUY	158.91	3.164	IVEXIR	170.782	3.116	IVETUY	166.365	3.051	IVETUY	152.747	3.067	IVETUY	157.622	3.299
IVEYIS	152.106	3.174	IVIBEU	160.502	3.423	IVEXOX	164.627	3.338	IVEXUD	150.994	3.353	IVEYIS	153.806	2.599
IVOBIF	154.628	3.375	IVOBIF	152.244	3.005	IVIBEU	152.539	3.462	IVIHON	159.496	3.401	IVOBAY	150.165	3.297
IVOLIO	150.024	2.834	IVOLIO	160.798	2.855	IVOFAA	154.074	3.476	IVOLIO	167.594	2.957	IVOLIO	153.739	3.188
IVOLIO	162.139	3.003	IVOYIE	160.567	3.422	IVOLIO	151.732	2.92	IVOLIO	156.951	2.949	IVOLIO	163.084	3.032
IVUHOW	167.828	3.234	IVUQIZ	164.96	3.41	IVUHIS	167.852	3.247	IVUHIS01	170.565	3.345	IVUHIS01	150.676	3.069
IVUQIZ04	163.052	3.292	IVUQIZ05	163.049	3.292	IVUQIZ01	163.067	3.289	IVUQIZ02	163.121	3.288	IVUQIZ03	163.041	3.292
IVUQOF07	162.243	3.077	IVUVAY	172.294	2.996	IVUQIZ06	165.106	3.418	IVUQOF01	154.084	3.145	IVUQOF01	160.672	3.322
IWAHIY01	157.976	3.194	IWAMEY	160.199	3.179	IVUVAY01	172.543	2.993	IVUZAB	158.617	3.116	IVUZAB	164.413	3.323
IWEDUL	164.476	3.16	IWEDUL	150.898	3.102	IWASOP	159.992	2.918	IWAXIQ	163.055	2.937	IWAZAJ	169.08	3.371
IWENEF	172.901	3.495	IWENEF	175.18	2.791	IWENAB	169.789	3.309	IWENAB	169.237	2.999	IWENEF	165.984	3.084
IWENIJ	157.068	3.235	IWENIJ	168.279	3.358	IWENEF	164.266	3.084	IWENEF	177.269	3.495	IWENIJ	159.897	3.073
IWENOP	158.644	3.197	IWEPIL	171.187	3.187	IWENIJ	171.07	3.238	IWENIJ	172.215	2.701	IWENOP	161.977	2.881
IWISUE	152.03	2.848	IWITOA	155.075	3.31	IWEPIL	158.619	3.494	IWEPIL	153.099	3.126	IWETIQ	164.916	2.851
IWOMUC	160.106	3.223	IWOTEV	150.382	3.218	IWIZAR	152.897	2.832	IWOBEE	161.442	3	IWOLIP	156.736	3.29
IWOXUP	157.142	2.757	IWOZAV	167.251	3.236	IWOTEV	156.968	2.889	IWOTEV	160.831	2.889	IWOVUN	156.783	2.9
IWUNOD	150.781	3.043	IWUNOD	159.851	3.023	IWUJER	165.672	3.356	IWUNOD	153.745	3.349	IWUNOD	160.434	2.875
IWUNOD	176.003	2.853	IWUNOD	155.463	2.891	IWUNOD	151.555	2.939	IWUNOD	157.133	2.937	IWUNOD	153.795	3.384
IXAPEE01	156.209	3.319	IXAPEE01	154.007	3.412	IWUNOD	151.154	2.806	IWUNOD	154.148	2.99	IWUROK	163.87	3.484
IXECEW	158.526	3.06	IXEFUP01	171.127	2.922	IXAPEE01	158.777	2.795	IXECEW	171.557	3.431	IXECEW	159.41	3.074
IXEMUW	173.635	3.485	IXENAD	150.165	3.431	IXEFUP01	158.645	2.777	IXEFUP01	167.362	2.777	IXEFUP01	153.303	3.007
IXENUX	161.855	3.39	IXEPEG	156.422	3.167	IXENAD	154.875	3.234	IXENUX	151.989	3.141	IXENUX	172.589	3.397
IXEPEG	166.386	3.282	IXEPEG	170.902	3.302	IXEPEG	160.302	3.253	IXEPEG	158.611	3.452	IXEPEG	169.802	2.916
IXOBAZ	166.397	3.177	IXOGIL	155.181	3.123	IXIHIH	151.057	3.016	IXOBAZ	155.207	3.122	IXOBAZ	171.021	3.07
IXOYUR	168.909	3.027	IXUMOD	153.708	3.274	IXOKAH	164.065	3.294	IXOMIT	151.679	2.894	IXOMIT	163.974	2.67
IXURIF	158.561	3.081	IXUSAV	164.972	3.024	IXUMOD	168.964	3.377	IXUPID	150.685	2.999	IXURIF	159.31	3.082
IXUZOT	155.205	3.268	IXUZOT	173.865	3.268	IXUSAV	150.137	2.687	IXUTOM	153.731	3.367	IXUVEE	161.635	3.061
IYALAW	164.449	2.958	IYALEA	154.953	3.104	IYALAW	157.407	3.224	IYALAW	161.384	2.89	IYALAW	167.359	3.279
IYALEA	157.544	3.382	IYALEA	174.433	3.296	IYALEA	162.147	3.081	IYALEA	158.606	3.081	IYALEA	172.807	3.185
IYALOK	157.862	2.951	IYALOK	159.658	2.824	IYALOK	153.675	2.929	IYALOK	150.173	3.402	IYALOK	152.379	2.833
IYALOK	150.269	2.833	IYALOK	161.53	3.355	IYALOK	151.953	3.032	IYALOK	169.696	3.004	IYALOK	173.265	3.356
IYALOK	170.903	3.145	IYALUQ	170.34	2.99	IYALOK	169.307	3.4	IYALOK	163.481	2.769	IYALOK	160.509	3.302
IYAZUE	158.739	3.128	IYEDUO	151.518	2.923	IYALUQ	155.028	3.437	IYALUQ	156.8	2.99	IYALUQ	174.151	2.878
IYEGEB01	152.614	2.656	IYEGEB01	153.946	2.656	IYEFEA	166.113	3.279	IYEFOK	167.469	2.967	IYEFOK	154.224	2.777
IYEPAD	150.976	3.268	IYEPOR	151.293	3.335	IYEGOJ	173.248	3.12	IYEGOJ	166.072	3.178	IYEPAD	156.804	3.379
IYEREK	150.3	3.385	IYESOX	153.426	3.209	IYEQUY	169.638	3.266	IYEQUY	156.512	3.132	IYEQUZ	168.751	3.425
IYICAU	168.377	3.036	IYICAU	156.511	2.966	IYEXOC	164.02	3.454	IYICAU	162.123	3.053	IYICAU	150.917	3.374
IYICAU	155.423	3.325	IYICAU	154.799	3.464	IYICAU	154.033	2.825	IYICAU	165.535	2.973	IYICAU	160.219	2.989
IYICIE	159.283	3.257	IYICIE	154.392	3.271	IYICEY	165.528	3.273	IYICIE	159.754	3.154	IYICIE	157.639	3.084
IYICOL	164.459	2.695	IYICOL	155.218	3.112	IYICIE	162.55	2.833	IYICOL	151.981	3.278	IYICOL	158.167	3.112
IYILOU	168.062	3.439	IYILOU	164.343	3.383	IYICOL	161.59	3.356	IYICOL	159.718	3.443	IYILOU	155.885	2.699
IYILOU01	167.296	3.024	IYILOU01	171.407	2.892	IYILOU	161.89	3.383	IYILOU01	174.318	2.885	IYILOU01	166.131	3.001
IYILOU01	160.415	2.897	IYILOU01	162.57	3.011	IYILOU01	152.926	3.25	IYILOU01	154.605	3.32	IYILOU01	161.203	3.011
IYILOU01	170.154	3.102	IYILOU01	151.443	3.374	IYILOU01	166.718	2.897	IYILOU01	162.132	3.023	IYILOU01	153.947	3.224
IYILOU02	159.917	2.876	IYILOU02	168.137	3.022	IYILOU02	174.342	2.875	IYILOU02	166.58	2.987	IYILOU02	154.273	3.337
IYILOU02	162.019	2.994	IYILOU02	167.582	2.876	IYILOU02	170.524	2.899	IYILOU02	152.081	3.247	IYILOU02	161.548	2.994
IYILOU02	152.042	3.332	IYIMAF01	160.616	3.188	IYILOU02	161.432	3.005	IYILOU02	152.974	3.209	IYILOU02	169.017	3.095
IYIMAF01	168.61	2.917	IYIMAF01	158.628	3.208	IYIMAF01	153.33	3.293	IYIMAF01	160.339	3.116	IYIMAF01	153.775	3.383
IYIVUI	164.79	3.164	IYIYAQ	177.031	3.343	IYIMAF01	159.491	3.03	IYIMAF01	165.191	3.039	IYIRUD	155.645	2.802
IYOWAW	160.392	3.471	IYOWAW	163.767	3.019	IYOGEL	151.028	3.359	IYOSAT	156.137	3.286	IYOVUP	163.642	2.846
IYOWOK	167.815	2.89	IYOZEB	152.948	3.325	IYOWAW	162.895	2.826	IYOWEA	171.726	3.01	IYOWIE	172.656	2.933
IYUCAG	168.745	2.856	IYUCAG	170.775	3.233	IYOZEB	154.337	3.055	IYOZEB	155.072	3.277	IYOZEB	171.792	2.797
IYUGOY	156.071	2.92	IYUGUE	174.159	2.858	IYUCAG	169.154	3.352	IYUFOA	151.099	3.398	IYUGIS	156.832	2.951
IZAGIA	155.323	3	IZAGIA	153.361	2.965	IYUQUP	162.359	3.308	IYURAW	153.906	3.055	IZADUI	156.838	2.873
IZAGIA	152.399	2.862	IZAGIA	150.76	2.989	IZAGIA	151.334	2.965	IZAGIA	157.396	2.952	IZAGIA	154.415	3.003
IZAGIA	150.575	2.862	IZAGIA01	151.121	2.93	IZAGIA	151.272	2.87	IZAGIA	152.945	3.04	IZAGIA	151.278	2.87
IZAGIA01	157.223	2.976	IZAGIA01	154.135	2.835	IZAGIA01	150.047	2.993	IZAGIA01	150.607	2.95	IZAGIA01	153.159	2.93
IZAGIA01	156.137	2.926	IZAHIB	165.59	2.939	IZAGIA01	152.632	2.829	IZAGIA01	156.126	2.975	IZAGIA01	151.768	3.009
IZAJEB01	164.004	2.739	IZAZEQ	159.24	2.823	IZAHIB	167.734	2.939	IZAHIB	166.043	3.311	IZAJEB	164.003	2.739
IZAZEQ	161.765	3.08	IZAZEQ	154.874	2.946	IZAZEQ	171.492	3.323	IZAZEQ	152.032	2.882	IZAZEQ	151.435	2.994
IZEQAG	162.968	3.123	IZEQAG	157.107	3.438	IZAZEQ	163.641	2.848	IZAZEQ	152.38	2.986	IZAZEQ	169.697	3.437
IZETAL	158.089	3.041	IZETAL	159.406	3.041	IZESEN	163.042	3.144	IZESUE	157.577	3.044	IZESUE	159.281	3.044

IZICAX	157.712	3.005	IZICAX	167.675	3.282	IZEVEP	157.293	2.745	IZEVEP01	156.914	2.795	IZEVUF	164.711	3.22
IZIQOZ	165.861	3.029	IZIQOZ	151.685	3.322	IZICAX	154.035	3.064	IZIJUY	169.152	3.111	IZIJUY	159.786	3.111
IZITAO	157.035	2.934	IZITAO	150.731	3.016	IZIQOZ	153.161	3.481	IZITAO	166.861	3.095	IZITAO	151.307	2.705
IZITAO	154.548	2.915	IZITAO	150.218	2.928	IZITAO	167.205	3.204	IZITAO	153.219	2.817	IZITAO	155.1	2.817
IZIYAU	158.027	2.951	IZIZAU	173.512	3.46	IZITAO	159.3	3.287	IZITAO	158.358	3.06	IZITAO	164.137	2.705
IZUWAD	153.477	3.024	IZUWAD	158.354	2.884	IZUQID	154.071	3.289	IZUQOJ	177.474	3.212	IZUVUW	150.019	3.307
JABBET	159.148	2.778	JABBET	159.095	3.453	IZUWAD	155.183	3.421	IZUWAD	162.796	2.976	IZUWAD	171.136	3.137
JABPUC	153.809	3.494	JABVER	167.868	2.88	JABBET	167.584	2.933	JABBET	152.503	2.924	JABBIX	162.093	3.026
JABZIY	153.61	2.928	JABZIY	162.217	2.955	JABVER	157.629	3.436	JABVER	171.742	3.085	JABVER	167.86	2.915
JACFID	159.15	2.911	JACFID	159.675	2.905	JABZIY	150.553	2.928	JACFID	169.981	2.836	JACFID	163.23	2.957
JACFOJ	153.884	3.464	JACFOJ	165.257	2.97	JACFID	170.941	2.905	JACFID	154.968	2.798	JACFID	150.378	3.016
JACZEX	155.038	3.077	JACZEX	150.471	2.834	JACVIV	152.137	2.918	JACVIW	155.515	3.002	JACYIW	154.891	3.334
JADGOO	153.188	2.892	JADGOO	152.31	3.057	JACZEX	176.011	3.088	JACZEX	152.504	3.218	JADGII	151.605	3.303
JADGUU	170.386	2.923	JADHEF	158.858	2.918	JADGOO	169.173	2.904	JADGUU	151.588	2.958	JADGUU	152.24	3.24
JADHEF	163.664	3.36	JADHEF	152.912	3.316	JADHEF	163.82	2.897	JADHEF	160.831	3.127	JADHEF	151.01	3.367
JADTER	171.069	2.863	JADYAS	158.425	3.24	JADHEF	167.905	3.032	JADHEF	159.193	2.89	JADHEF	151.998	3.259
JAFXAS	157.961	3.102	JAGBIF	157.644	2.878	JADYAS01	156.87	3.271	JAFFOQ	169.055	3.099	JAFFOQ02	168.493	2.99
JAGCON	156.38	3.471	JAGCON	163.817	2.86	JAGBIF	162.054	2.878	JAGBIF	159.999	3.058	JAGBIF	173.913	3.489
JAGDII	171.053	3.323	JAGDOO	173.069	2.824	JAGCON	172.212	3.105	JAGCON	161.259	3.039	JAGCON	175.892	2.75
JAGDOO	168.583	3.122	JAGDOO	167.751	3.177	JAGDOO	160.027	2.877	JAGDOO	153.343	3.025	JAGDOO	153.834	3.375
JAGXAT	152.218	3.435	JAHFAD	166.192	3.382	JAGHAF	157.567	2.851	JAGXAR	155.605	3.403	JAGXAR	158.797	3.344
JAHVEX	150.326	2.956	JAHVIB	161.199	3.482	JAHFAD	156.158	2.634	JAHHOQ	171.9	3.299	JAHVEX	161.297	3.366
JAHWIB	172.196	2.953	JAHWIB	160.309	3.081	JAHVIB	152.293	2.979	JAHWEX	164.297	3.324	JAHWEX	167.113	3.312
JAHYID	153.477	2.929	JAHYID	151.346	2.923	JAHWIB	156.213	2.948	JAHYEZ	166.074	2.812	JAHYEZ	152.614	2.751
JAHYID	150.261	3.153	JAHYOJ	153.679	3.24	JAHYID	173.403	2.965	JAHYID	166.563	3.279	JAHYID	172.25	3.243
JAHYUP	153.193	3.034	JAHYUP	165.15	2.902	JAHYOJ	161.069	3.07	JAHYUP	152.082	3.083	JAHYUP	157.101	3.221
JAHYUP	154.745	3.063	JAHYUP	163.187	2.968	JAHYUP	160.669	2.844	JAHYUP	151.062	3.092	JAHYUP	158.714	3.263
JAJDUT	165.327	3.44	JAJGUX	153.047	3.222	JAHYUP	158.31	2.893	JAJDUT	154.782	3.111	JAJDUT	151.661	3.018
JAKMIV	155.081	2.904	JAKMIV	167.42	3.286	JAJGUX	162.25	3.26	JAKHUZ	162.598	3.401	JAKHUZ	151.152	3.373
JAKMIV	154.217	3.313	JAKMIV	158.555	2.916	JAKMIV	161.089	3.116	JAKMIV	167.513	3.209	JAKMIV	157.327	3.014
JALKUD	168.794	2.569	JALKUD	160.652	2.731	JAKMIV	155.724	2.921	JAKMIV	162.765	3.277	JAKMIV	157.087	2.951
JAMPEV	166.59	3.245	JAMSIZ	162.715	2.937	JALKUD	154.053	3.081	JALLAK	156.226	2.906	JALLIS	170.867	3.419
JAMTUN	165.085	2.726	JAMTUN	150.165	3.114	JAMTUN	160.454	3.254	JAMTUN	160.584	3.162	JAMTUN	153.868	2.92
JAMTUN	155.18	2.726	JAMZOQ	151.808	3.468	JAMTUN	160.958	3.336	JAMTUN	165.03	3.114	JAMTUN	151.945	3.04
JAMZOQ	150.195	3.322	JANBAF	176.266	3.004	JAMZOQ	157.756	3.479	JAMZOQ	150.879	3.455	JAMZOQ	168.634	2.944
JANBAF	159.241	3.004	JANBEJ	170.26	2.822	JANBAF	172.013	2.714	JANBAF	173.602	3.399	JANBAF	158.029	3.174
JANBIN	156.553	2.782	JANBIN	161.754	3.365	JANBEJ	155.732	2.779	JANBEJ	160.676	3.298	JANBIN	169.494	2.796
JANBOQ	169.061	2.954	JANBOQ	161.662	2.799	JANBIN	152.781	2.647	JANBIN	159.829	2.647	JANBIN	153.804	2.854
JANBOT	162.796	2.755	JANPIX	162.103	3.058	JANBOT	163.398	3.057	JANBOT	157.555	3.228	JANBOT	165.226	3.021
JANTAW	152.932	3.402	JANXOM	170.467	3.062	JANQUL	154.438	3.345	JANQUL01	154.938	3.304	JANTAW	169.393	3.105
JAPHEQ	160.411	3.284	JAPHEQ	159.707	3.036	JANXOM	167.83	3.089	JANXOM	166.242	3.082	JANYAC	163.85	2.897
JAPKIX	159.671	3.128	JAPKIX	166.345	3.047	JAPHEQ	158.752	3.034	JAPKIX	151.026	3.427	JAPKIX	160.133	3.047
JAPKIX	161.221	3.318	JAPLUL	165.605	2.71	JAPKIX	168.773	2.962	JAPKIX	170.997	2.804	JAPKIX	167.385	3.413
JAPMUJ	177.296	3.085	JAPMUJ	176.625	3.085	JAPLUL	164.934	2.826	JAPLUL	154.789	3.282	JAPLUL	154.172	2.95
JAPQAT	166.653	2.846	JAQCOT	173.837	3.082	JAPNAQ	154.484	3.356	JAPQAT	172.771	2.9	JAPQAT	157.768	2.921
JAQCOT	154.016	2.917	JAQFAM	162.07	2.907	JAQCOT	165.132	3.358	JAQCOT	162.683	2.734	JAQCOT	179.746	3.112
JAQQOL	159.81	2.915	JAQROL	170.781	2.647	JAQFAM	159.632	2.857	JAQMIY	166.454	3.466	JAQMIY01	167.925	3.413
JAQSIG	152.936	2.898	JAQSIG	156.562	3.28	JAQSIG	171.212	3.023	JAQSIG	169.38	3.226	JAQSIG	155.742	3.438
JARBUC	172.662	3.385	JARCUD01	160.756	3.045	JAQSIG	152.672	3.199	JARBOW	157.664	3.319	JARBOW	166.839	3.368
JARNOI	177.792	3.348	JARNOI	150.747	3.15	JARLIB	152.733	3.465	JARLIB	176.111	3.171	JARNOI	155.015	3.284
JASFOY	152.096	3.069	JASHOA	150.637	3.315	JARWEI	162.786	3.021	JARWEI	159.78	3.108	JARZOS01	170.926	3.441
JASWEJ	150.276	2.831	JASWEJ	169.162	3.035	JASWEJ	173.107	2.942	JASWEJ	163.674	3.301	JASWEJ	162.295	3.208
JAVLOI	172.929	3.154	JAVMEZ	154.252	3.225	JATFUI	165.819	3.347	JATGAP	168.9	3.08	JATRUU	170.987	3.064
JAWCAO	159.334	2.928	JAWCIW	173.354	2.843	JAVMEZ	160.569	3.094	JAVWIN01	162.96	3.245	JAWCAO	159.692	2.928
JAWHAR	175.865	2.996	JAWHAR	153.679	2.954	JAWCUI	157.92	3.061	JAWCUI	162.323	3.061	JAWHAR	160.022	3.474
JAWKIF	155.178	3.416	JAWPEG	154.739	3.496	JAWHAR	175.845	3.079	JAWHEV	152.291	3.056	JAWKIF	170.913	3.037
JAWPEG	178.677	2.74	JAWPEG	150.862	3.401	JAWPEG	164.917	2.943	JAWPEG	151.748	2.657	JAWPEG	152.524	3.418
JAWPEG	176.157	3.404	JAWPEG	159.667	3.045	JAWPEG	166.887	3.003	JAWPEG	165.046	3.045	JAWPEG	156.261	3.059
JAWPIK	152.164	2.772	JAXJAT	153.73	2.847	JAWPEG	162.017	3.004	JAWPIK	151.587	2.712	JAWPIK	152.528	2.706
JAXMEA	167.213	2.944	JAXMEA	151.867	3.471	JAXLEZ	176.168	3.374	JAXLEZ	172.429	3.184	JAXLEZ	172.192	3.371
JAXMII	153.165	2.79	JAXMII	151.701	2.994	JAXMEA	152.061	3.276	JAXMII	151.368	2.964	JAXMII	177.884	3.089
JAXPAD	162.617	3.217	JAYDOF	153.492	2.927	JAXMII	158.964	3.3	JAXMUQ	162.191	3.117	JAXMUQ	157.527	3.12
JAYGEZ01	165.065	3.442	JAYGEZ02	151.619	3.363	JAYDOF	154.349	2.717	JAYDOF	151.254	2.717	JAYGEZ	165.639	3.425
JAYJUO	154.518	3.325	JAYYOB	154.221	3.482	JAYGEZ03	164.564	3.456	JAYJUO	162.017	2.925	JAYJUO	150.228	2.925
JAYZAO	151.499	3.272	JAYZAO	156.832	3.127	JAYYUH	157.757	2.889	JAYYUH	173.591	2.918	JAYZAO	169.903	3.043
JAZBOF	170.742	3.135	JAZBOF	178.052	3.135	JAYZEP	150.232	3.148	JAZBEV	150.075	2.793	JAZBOF	154.493	3.423
JAZJAZ	158.002	3.257	JAZJAZ	170.995	2.828	JAZBOF	160.649	3.368	JAZBOF	166.089	3.234	JAZFAS	166.589	3.358
JAZJED	152.607	2.888	JAZJON	150.429	3.085	JAZJAZ	152.124	3.359	JAZJED	152.801	3.077	JAZJED	168.435	2.888
JAZVEO	166.12	2.867	JAZXOX	164.064	3.144	JAZJON	162.653	3.325	JAZMEC	154.638	2.959	JAZTOW	167.673	3.472
JEBPIQ	151.246	3.117	JEBPOW	151.392	3.099	JAZXOX	153.026	3.336	JEBMAH	159.595	3.477	JEBMAH	160.985	3.179
JEBWUJ	164.311	2.842	JEBWUJ	157.113	2.819	JEBPOW	150.115	3.112	JEBWOD	166.759	2.718	JEBWUJ	160.845	2.819
JEBXIY	150.958	3.189	JEBXIY	165.751	3.259	JEBWUJ	152.317	2.842	JEBXIY	159.471	3.329	JEBXIY	155.408	3.189
JECLUY	159.412	3.49	JECPOA	152.558	3.056	JEBZEZ	158.198	3.44	JECJOT	153.853	2.902	JECLUY	161.66	3.061
JEDLEN	163.859	3.034	JEDLEN	151.43	3.443	JECRIT	167.602	2.849	JEDDEE	150.304	2.94	JEDLEN	152.394	3.049
JEDPOB	161.212	2.634	JEDPUH	161.019	3.238	JEDLEN	150.354	3.093	JEDLIQ	168.275	2.898	JEDLIQ	166.089	3.222
JEDSUH	151.088	3.199	JEDSUH	159.402	2.962	JEDPUH	171.301	2.746	JEDSAN	155.962	3.05	JEDSOB	169.092	3.102
JEFCUW	161.45	3.436	JEFKAH	157.505	3.205	JEDTES	160.546	3.258	JEDTIW	171.299	3.226	JEFCUW	155.728	2.984
JEFLEL	169.311	2.876	JEFMIR	156.971	3.473	JEFKAH	158.716	2.695	JEFLEL	158.044	3.236	JEFLEL	166.626	2.876
JEFZUT	159.854	3.219	JEGBAC	156.097	3.226	JEFPOA	162.693	2.887	JEFZUT	150.297	3.246	JEFZUT	150.267	3.404

JEGDIM	171.856	3.351	JEGDIM	155.337	3.171	JEGBEG	172.623	3.461	JEGDEI	159.932	3.091	JEGDIM	156.251	3.171
JEGDOS	158.581	3.367	JEGKUC	173.27	2.75	JEGDIM	168.776	3.343	JEGDIM	154.061	3.433	JEGDOS	167.167	3.127
JEGKUC	160.691	2.772	JEGKUC	160.418	3.173	JEGKUC	163.306	3.154	JEGKUC	176.751	2.798	JEGKUC	160.456	3.178
JEGKUC	173.38	2.816	JEGKUC	154.014	3.311	JEGKUC	155.733	3.178	JEGKUC	156.069	3.259	JEGKUC	159.952	2.72
JEGKUC	172.347	2.871	JEGLIR	167.329	2.792	JEGKUC	158.05	3.338	JEGKUC	162.153	2.772	JEGKUC	169.312	2.789
JEGRIZ	150.206	2.625	JEGRIZ	150.939	2.935	JEGLIR	161.548	2.865	JEGLIR	164.673	2.911	JEGLIR	162.945	2.924
JEGRIZ	163.304	3.294	JEGRIZ	169.091	2.74	JEGRIZ	177.571	3.118	JEGRIZ	165.999	2.89	JEGRIZ	155.54	2.625
JEHKIR	165.022	3.232	JEHKIR	151.76	3.185	JEGRIZ	159.929	2.809	JEHCIJ	157.652	3.345	JEHHAG	170.995	3.298
JEJBEI	150.273	3.171	JEJBIM	153.737	3.109	JEHRAQ	167.549	2.704	JEHSIZ	159.925	2.943	JEHYIF	162.525	3.309
JEJDIL01	161.104	3.487	JEJFOT	155.175	2.99	JEJBIM	165.765	3.126	JEJDIL01	161.75	3.094	JEJDIL01	158.5	2.839
JEKJUE	158.739	2.88	JEKXEF	150.219	2.953	JEJJOA	163.87	3.475	JEJTOI	169.331	3.457	JEJXOP	159.717	2.586
JELBAE	158.343	3.265	JELBAE	154.404	3.293	JEKXEF	166.89	2.901	JEKXOP	162.455	3.066	JEKXOP	159.336	3.005
JELKIU	162.188	3.234	JELKIU	162.593	3.077	JELBIP	166.298	3.324	JELBIP	157.761	3.06	JELKIU	157.479	3.404
JELKIU	155.709	3.368	JELKIU	155.395	3.114	JELKIU	151.485	3.27	JELKIU	158.35	3.384	JELKIU	158.475	2.727
JELXIK	161.802	3.291	JELXUX	160.756	2.894	JELNIB	150.219	3.379	JELREA	154.398	2.996	JELXIK	154.995	3.408
JEMVUT	151.366	3.471	JEMVUT	156.659	3.489	JELXUX	155.167	2.894	JEMGEQ	151.125	3.121	JEMVOQ	153.481	2.855
JEMWUT	155.374	2.76	JEMWUT	173.813	2.8	JEMWOQ	157.811	2.827	JEMWUT	154.886	3.106	JEMWUT	167.69	2.86
JEMXAA	152.555	2.919	JENGUF	167.903	3.311	JEMXAA	151.895	3.216	JEMXAA	172.557	3.132	JEMXAA	151.398	3.216
JENGUF0	168.186	3.319	JENGUF0	164.736	2.835	JENGUF	164.326	2.778	JENGUF	159.192	3.163	JENGUF	174.506	3.284
JENJAR	156.416	2.821	JENTAY	169.912	2.892	JENGUF0	160.08	3.18	JENGUF0	174.948	3.305	JENJAR	154.17	3.309
JENTAY	153.653	3.253	JENTAY	171.51	3.055	JENTAY	156.364	3.027	JENTAY	156.632	2.966	JENTAY	155.018	2.959
JENTAY	155.663	3.301	JENTAY	156.324	3.276	JENTAY	152.714	3.086	JENTAY	155.425	2.892	JENTAY	172.971	2.964
JEPFET	167.368	3.497	JEPFET	166.959	2.849	JENTAY	172.772	3.486	JEPFAP	158.212	3.116	JEPFAP	175.424	3.387
JEPKUL	154.248	3.304	JEPKUL	175.602	3.072	JEPFET	167.841	2.878	JEPKUL	153.98	2.824	JEPKUL	162.652	2.766
JEPKUL	164.421	3.474	JEPKUL	167.293	3.335	JEPKUL	171.493	3.304	JEPKUL	165.905	3.439	JEPKUL	160.527	2.814
JEPNEA	174.819	2.765	JEPNEA	165.553	2.765	JEPLUL	159.83	2.86	JEPLUL	156.095	3.194	JEPLUL	150.038	3.055
JEPSOM	157.956	3.171	JEPSOM	151.894	3.262	JEPNEA	159.757	3.057	JEPNOK	156.418	3.268	JEPNUQ	155.233	3.32
JERCIW	158.545	3.25	JERCIW	167.452	2.816	JEPTOQ	153.938	3.245	JEQCIS	153.076	2.872	JEQCIS	152.371	2.899
JERMOI	162.291	3.275	JERMUO	168.267	3.396	JERGES	153.085	3.129	JERMOI	155.179	3.213	JERMOI	150.273	3.163
JERMUO	161.223	2.773	JERMUO	157.68	2.789	JERMUO	164.895	2.975	JERMUO	162.055	2.935	JERMUO	152.275	2.773
JESKIC	150.021	3.042	JESZAL	150.607	3.334	JERMUO	151.953	2.789	JERMUO	165.29	2.935	JERXUA	169.624	2.705
JETBIT	166.27	3.471	JETBIT	153.354	3.336	JETBIT	162.577	2.872	JETBIT	162.711	3.471	JETBIT	157.427	3.016
JETTOU	158.045	3.149	JETTOU	167.868	2.68	JETBIT	169.481	2.979	JETBIT	177.999	3.205	JETFOH	174.162	2.895
JEVGAS	163.909	3.325	JEVHOK	150.259	3.167	JETXAH	157.173	3.092	JEVFEY	157.08	3.069	JEVGAS	151.064	3.234
JEWBIA	158.088	3.026	JEWCOH	169.654	3.227	JEVHOK	168.115	3.038	JEVVIQ	162.824	3.207	JEVWIT	162.851	3.24
JEWGIF	152.37	3.172	JEWGIF	156.227	2.928	JEWCUN	151.893	3.318	JEWCUN	167.373	3.179	JEWGIF	170.32	3.28
JEWGOI	157.523	3.034	JEWGOI	156.171	2.836	JEWGIF	156.298	2.704	JEWGIF	160.159	3.092	JEWGIF	169.666	3.15
JEWGOI	178.975	2.95	JEWGOI	157.165	3.455	JEWGOI	166.783	3.37	JEWGOI	152.589	3.183	JEWGOI	151.844	3.267
JEWGOI	150.835	3.019	JEWNEF	175.436	3.391	JEWGOI	175.528	2.951	JEWGOI	154.984	3.084	JEWGOI	150.461	3.192
JEXVIU	169.624	3.101	JEXXAP	152.688	3.332	JEWQAE	174.226	2.938	JEWWOY	153.286	3.4	JEXCUN	155.074	3.173
JEYLOO	155.778	2.823	JEYLOO	154.227	2.739	JEXXAP	154.068	3.106	JEYKOO	153.578	3.148	JEYKUU	168.058	3.21
JEYLUU	160.066	2.93	JEYLUU	154.256	3.276	JEYLOO	154.218	3.129	JEYLOO	165.841	2.823	JEYLUU	154.307	3.002
JEYSAL	161.364	3.446	JEYSAL	150.028	3.446	JEYLUU	158.291	3.129	JEYLUU	155.283	3.125	JEYLUU	164.457	3.073
JEYZIZ	158.063	3.341	JEZHUV	164.203	3.311	JEYTUG	154.673	3.357	JEYXIY	169.815	2.85	JEYZEV	152.725	3.325
JEZTUH	167.352	3.201	JEZTUH	161.813	2.69	JEZKOS	170.196	2.879	JEZKOS	165.529	2.879	JEZROZ	153.051	3.13
JICVEA	174.859	3.234	JICWEA	152.991	3.494	JIBHIO	152.375	2.77	JIBHOU	154.233	2.903	JICSIX	151.219	3.188
JICWOK	154.611	3.467	JICWUN	153.037	2.888	JICWIE	159.329	3.309	JICWOK	153.141	3.434	JICWOK	151.034	3.127
JICWUN	150.093	2.912	JICWUN	164.231	3.178	JICWUN	162.628	3.082	JICWUN	171.293	2.78	JICWUN	171.644	3.236
JICXOI	162.334	3.245	JICXOI	152.924	3.023	JICXOI	176.796	3.307	JICXOI	156.632	2.92	JICXOI	167.312	3.439
JICXOI	168.447	3.229	JICXOI	166.242	3.333	JICXOI	173.312	3.322	JICXOI	169.43	2.815	JICXOI	171.399	3.151
JICXOI	172.422	3.106	JICXOI	155.213	2.955	JICXOI	156.068	2.955	JICXOI	157.32	3.023	JICXOI	177.814	3.318
JICXOI10	176.796	3.307	JICXOI10	156.632	2.92	JICXOI	169.599	3.258	JICXOI	150.366	3.33	JICXOI	158.93	3.026
JICXOI10	173.312	3.322	JICXOI10	169.43	2.815	JICXOI10	167.312	3.439	JICXOI10	162.334	3.245	JICXOI10	152.924	3.023
JICXOI10	156.068	2.955	JICXOI10	157.32	3.023	JICXOI10	171.399	3.151	JICXOI10	168.447	3.229	JICXOI10	166.242	3.333
JICXOI10	169.599	3.258	JICXOI10	150.366	3.33	JICXOI10	177.814	3.318	JICXOI10	172.422	3.106	JICXOI10	155.212	2.955
JIDBIK	164.492	3.419	JIDBIK01	166.176	3.332	JICXOI10	158.93	3.026	JICYUT	165.977	3.099	JIDBIK	164.368	3.391
JIDCAE	152.794	3.316	JIDCAE	150.559	3.097	JIDBIK01	166.566	3.364	JIDBIK03	164.479	3.42	JIDBIK03	164.258	3.39
JIDPOB	160.754	3.04	JIDPOB	168.616	3.457	JIDCAE	158.571	3.114	JIDCAE	165.126	2.919	JIDLOA	170.022	2.886
JIFKIV	159.045	2.938	JIFKIV	152.315	2.861	JIDWOL	171.538	2.905	JIDWOL	166.892	3.488	JIFKIV	159.486	2.861
JIFREZ	166.499	3.229	JIFREZ	164.286	3.312	JIFKIV	151.607	2.938	JIFPUN	167.185	2.859	JIFREZ	174.57	3.062
JIFVEC	173.107	2.986	JIFVEC	172.613	2.714	JIFVAY	166.996	3.013	JIFVAY	172.221	2.773	JIFVEC	175.578	2.714
JIGGOZ	164.609	3.498	JIGGOZ	169.126	3.498	JIFVEC	174.733	2.986	JIGGOZ	176.78	3.327	JIGGOZ	159.114	3.248
JIGGUF	163.911	3.494	JIGGUF	171.032	3.494	JIGGUF	160.847	3.469	JIGGUF	176.464	3.317	JIGGUF	159.848	3.113
JIGZOO	150.776	3.089	JIHNOG	161.522	3.39	JIGNEW	151.597	3.286	JIGNEW	169.002	2.95	JIGNEW	160.627	3.327
JIJKIA	162.673	3.159	JIJKIA	160.959	3.376	JIHNOG	172.999	3.374	JIHNOG	162.698	3.5	JIJKIA	156.414	3.451
JIJTEE	162.153	3.333	JIJTEE	171.88	2.807	JIJKIA	169.41	3.084	JIJKIA	161.477	3.376	JIJTEE	159.767	2.807
JIJTEE01	160.864	3.096	JIJTEE01	159.767	2.813	JIJTEE	160.858	3.089	JIJTEE	166.383	2.845	JIJTEE01	171.862	2.813
JIKBUA	163.568	3.318	JIKGAO	158.142	3.426	JIJTEE01	162.081	3.338	JIJTEE01	166.358	2.854	JIJVAD	155.06	3.099
JIKGAO	162.255	3.462	JIKGAO	164.698	3.211	JIKGAO	155.263	2.778	JIKGAO	157.042	2.957	JIKGAO	163.203	3.303
JIKGAO	172.607	3.006	JIKGAO	161.542	3.169	JIKGAO	160.146	2.705	JIKGAO	164.833	3.131	JIKGAO	155.379	3.293
JIKGAO	152.315	3.151	JIKGAO	158.668	3.129	JIKGAO	150.555	3.357	JIKGAO	152.133	2.858	JIKGAO	154.702	3.175
JIKGAO	165.669	3.472	JIKGAO	155.894	3.282	JIKGAO	157.179	3.187	JIKGAO	157.312	2.79	JIKGAO	151.464	2.758
JIKGAO	151.828	3.009	JILGAP	150.665	3.063	JIKGAO	153.692	2.986	JIKGAO	155.125	3.006	JIKGAO	174.163	3.19
JILGET	154.941	2.945	JILGET	152.846	3.039	JILGAP	150.163	2.776	JILGET	162.603	3.133	JILGET	160.139	3.097
JILKUN	165.648	2.969	JILNOL	150.098	2.978	JILGET	156.629	2.949	JILGET	154.93	3.337	JILKIB	150.255	2.95
JILZOT	158.77	3.432	JILZOT	153.201	2.785	JILRIJ	152.006	3.219	JILYUC	150.65	2.721	JILYUC	156.801	3.024
JILZUZ	166.327	3.154	JILZUZ	153.603	3.071	JILZOT	150.574	2.9	JILZOT	173.996	2.785	JILZUZ	164.972	3.083
JIMBAI	173.178	3.425	JIMBAI	151.75	3.116	JILZUZ	161.213	2.865	JILZUZ	153.598	3.429	JILZUZ	166.933	3.138
JIMBEM	158.915	3.457	JIMBEM	171.566	2.944	JIMBAI	164.906	3.425	JIMBEM	165.117	2.937	JIMBEM	172.665	3.397

JIMBEM	156.917	2.947	JIMBEM	156.952	3.388	JIMBEM	166.36	3.314	JIMBEM	174.153	2.772	JIMBEM	154.36	3.095
JIMBEM	157.691	3.264	JIMBEM	153.742	3.311	JIMBEM	163.235	3.457	JIMBEM	155.97	3.097	JIMBEM	153.133	3.282
JIMDAN	166.078	2.963	JIMDEP	167.203	3.358	JIMCAN	150.946	2.953	JIMDAL	174.389	3.339	JIMDAN	156.827	2.957
JIMTEF	170.735	3.065	JIMTEF	174.176	3.447	JIMQAB	151.18	3.45	JIMTEF	178.183	3.484	JIMTEF	151.15	3.112
JIMTIJ	173.014	3.132	JIMWAE	164.698	3.362	JIMTEF	163.371	2.824	JIMTEF	168.765	3.329	JIMTIJ	172.129	3.117
JINDOC	161.567	3.332	JINDOC	158.394	2.893	JINCEO	167.74	3.34	JINCEO20	167.74	3.34	JINDOC	161.398	2.687
JINDOC	150.598	3.064	JINDOC	150.944	2.802	JINDOC	150.993	3.367	JINDOC	164.595	3.105	JINDOC	161.364	3.261
JINDUI	157.024	3.398	JINDUI	156.352	3.303	JINDOC	177.062	3.189	JINDOC	164.689	3.347	JINDOC	155.791	2.864
JINFAQ	163.134	3.47	JINFAQ	157.715	2.945	JINDUI	156.552	3.005	JINFAQ	153.94	3.081	JINFAQ	151.904	2.774
JINFAQ	158.399	2.969	JINFAQ	158.15	2.932	JINFAQ	158.46	3.495	JINFAQ	152.022	2.734	JINFAQ	150.232	2.734
JINFAQ	151.683	2.723	JINGAR	164.774	2.814	JINFAQ	150.05	3.252	JINFAQ	157.1	3.031	JINFAQ	151.935	2.723
JINGUJ	155.247	3.46	JINGUJ	173.879	2.68	JINGEV	164.629	2.836	JINGEV	165.326	2.988	JINGIZ	170.974	3.017
JINGUL	158.842	3.03	JINGUL	173.207	3.457	JINGUL	153.495	2.937	JINGUL	152.092	3.453	JINGUL	158.024	3.004
JINGUL	166.936	2.923	JINGUL	159.672	2.762	JINGUL	162.347	3.189	JINGUL	150.42	2.906	JINGUL	160.294	3.297
JINGUL	162.152	3.452	JINGUL	154.518	2.99	JINGUL	153.696	2.792	JINGUL	152.434	2.888	JINGUL	165.161	3.163
JINMAX	152.29	3.145	JINMAX	159.103	3.005	JINGUL	150.22	2.888	JINGUL	154.553	2.906	JINGUL	156.893	2.943
JINNIG	164.825	3.23	JINNIG	159.045	3.193	JINMOL	168.056	3.295	JINNAW	150.823	3.119	JINNAW	150.804	3.098
JINTUY	160.371	3.107	JINTUY	153.931	3.199	JINNIG	156.395	3.228	JINTUY	159.223	2.708	JINTUY	162.781	3.158
JINVEL	169.503	2.852	JIPGUN	150.069	3.058	JINTUY	157.39	2.933	JINVEL	163.382	3.016	JINVEL	174.472	3.291
JIRQEJ	164.468	3.176	JIRQEJ	178.992	3.339	JIQTUB	177.539	3.184	JIQTUB	157.072	2.896	JIRHIF	166.101	3.061
JISCEU	167.453	2.892	JISCEU	159.497	2.929	JIRQEJ	171.172	3.356	JISCEU	164.595	2.892	JISCEU	163.131	3.01
JISSOT	167.007	3.409	JISZER	154.99	2.616	JISCEU	164.871	2.866	JISCEU	155.871	2.94	JISJEB	162.33	2.907
JISZER	160.701	2.684	JITDAU	161.028	3.098	JISZER	152.993	3.477	JISZER	170.192	3.125	JISZER	153.532	3.192
JITMOR	173.802	3.394	JITMOR	151.088	2.939	JITLON	151.023	3.041	JITLON	163.654	2.991	JITMOR	160.684	2.97
JIVDUQ	158.067	3.212	JIVDUQ	154.252	3.497	JITNEG	166.401	3.321	JITNEG	163.791	3.321	JIVDUQ	154.683	2.89
JIVHEF	156.264	2.983	JIVKEI	154.876	3.471	JIVDUQ	151.317	2.653	JIVDUQ	150.414	2.979	JIVGED	154.17	3.184
JIWVIU	165.941	3.169	JIWVIU	161.086	3.149	JIVYEW	155.879	3.446	JIVYIZ	169.774	2.838	JIVZEX	164.818	2.946
JIWVIU	156.81	2.773	JIWVIU	153.533	3.238	JIWVIU	164.526	2.75	JIWVIU	151.405	3.472	JIWVIU	162.853	2.922
JIWWES	150.332	3.497	JIWZEX	169.085	2.417	JIWVIU	151.627	3.301	JIWWES	152.981	3.446	JIWWES	153.986	2.928
JIXBUR	153.16	3.1	JIXBUR	154.941	3.194	JIWZEX	160.009	2.417	JIWZEX	152.018	3.2	JIWZIZ	162.194	3.09
JIXGED	152.525	2.788	JIXGED	163.857	3.461	JIXFII	152.226	3.092	JIXFII	175.35	3.376	JIXFOO	158.315	3.177
JIXMUB	169.234	2.88	JIXQAJ	151.449	3.361	JIXGED	154.617	3.362	JIXHON	159.966	3.215	JIXHON10	159.966	3.215
JIXQAJ	178.419	2.844	JIXQAJ	178.587	2.857	JIXQAJ	150.66	3.091	JIXQAJ	151.091	3.357	JIXQAJ	150.377	3.104
JIYBEZ	162.381	2.79	JIYBEZ	162.823	2.79	JIXTAO	165.364	3.203	JIXTES	167.186	3.281	JIYBEZ	155.74	3.384
JIYDUT	173.681	2.899	JIYFON	156.993	2.824	JIYDON	152.389	2.982	JIYDON	154.841	3.423	JIYDON	159.725	2.907
JIYFON	158.572	2.998	JIYJIO	154.718	3.13	JIYFON	170.292	3.105	JIYFON	160.222	2.756	JIYFON	160.559	3.03
JIYMUC	163.441	3.276	JIYNIR	158.748	2.84	JIYJIO	157.003	3.197	JIYJIO	176.338	3.405	JIYJIO	173.472	3.401
JIYNUD	177.877	3.366	JIYNUD	160.839	3.159	JIYNIR	163.251	2.777	JIYNUD	159.945	3.326	JIYNUD	151.246	3.056
JIYPOZ	150.732	3.138	JIYPOZ	163.713	2.818	JIYNUD	151.56	2.851	JIYNUD	162.451	3.146	JIYPOZ	160.724	2.989
JIYQEQ	158.384	3.123	JIYQEQ	156.929	2.796	JIYPOZ	150.124	3.483	JIYPUF	160.993	2.918	JIYQEQ	154.809	3.284
JIYSAO	159.629	3.158	JIYVAP	162.107	3.374	JIYQIU	152.155	3.294	JIYQIU	152.115	3.493	JIYSAO	159.747	2.923
JIYZAU	168.599	2.963	JIYZUP	151.625	2.815	JIYVAP	157.591	3.068	JIYWOF	175.825	2.933	JIYYUN	163.354	3.028
JIYZUP	150.411	2.859	JIYZUP	172.197	3.038	JIYZUP	152.527	2.91	JIYZUP	158.798	2.756	JIYZUP	160.775	3.473
JIYZUP	151.693	2.875	JIZBAZ	150.602	3.085	JIYZUP	158.524	3.329	JIYZUP	156.772	3.401	JIYZUP	153.884	2.923
JIZBIG	163.462	3.184	JIZBIG	150.691	3.45	JIZBED	154.505	2.888	JIZBED	155.53	2.883	JIZBIG	150.027	3.12
JIZBIG	157.158	2.722	JIZBIG	157.43	3.257	JIZBIG	159.5	3.141	JIZBIG	165.567	3.392	JIZBIG	168.438	3.264
JIZBIG	164.449	3.151	JIZBIG	158.911	3.102	JIZBIG	158.157	2.987	JIZBIG	153.882	3.151	JIZBIG	159.551	2.815
JIZGOR	163.559	3.023	JIZGOR	154.378	3.262	JIZDIJ	150.77	3.416	JIZGOR	151.566	3.272	JIZGOR	173.457	2.799
JIZPEO	158.202	2.744	JIZPEO	156.274	2.744	JIZGOR	160.981	3.074	JIZHAE	170.398	3.016	JIZMUB	163.831	3.463
JOBBUA	162.461	2.637	JOBGEN	150.979	3.227	JIZPOY	151.759	3.46	JIZQIS01	160.318	2.815	JIZQUE	161.117	3.161
JOBVEE	161.339	3.316	JOBXEE	152.845	3.071	JOBGEN	168.534	3.389	JOBQID	160.191	3.375	JOBVEE	161.937	2.969
JOBXEE	150.312	3.424	JOBXEE	150.67	3.197	JOBXEE	164.791	3.234	JOBXEE	164.568	2.835	JOBXEE	163.903	3.234
JOBXUW	159.603	2.824	JOBXUW	151.366	2.842	JOBXII	157.967	3.113	JOBXUW	165.404	3.217	JOBXUW	156.925	2.835
JOCFEO	166.026	2.79	JOCFEO	152.287	2.79	JOBXUW	169.557	2.915	JOBXUW	156.807	2.621	JOCBAI	155.853	2.924
JOCFIS	172.968	3.498	JOCFUE	151.955	3.301	JOCFIS	178.123	3.419	JOCFIS	156.495	3.27	JOCFIS	157.16	3.27
JOCHEQ	173.878	3.402	JOCHEQ	152.308	2.913	JOCHAM	171.429	2.902	JOCHAM	155.238	2.902	JOCHEQ	157.578	2.925
JOCHOA	162.186	2.865	JOCHUG	163.849	2.964	JOCHOA	163.317	2.85	JOCHOA	162.274	2.85	JOCHOA	163.801	2.858
JOCKIV	152.303	3.452	JOCKIV	158.359	3.124	JOCHUG	159.463	2.957	JOCHUG	173.555	2.979	JOCKIV	163.392	2.674
JOCKIV	167.47	3.495	JOCKIV	161.67	3.497	JOCKIV	161.101	3.281	JOCKIV	160.415	3.076	JOCKIV	167.608	2.674
JODBEK	160.544	3.404	JODBEK	156.278	3.084	JOCLUK	167.652	3.381	JOCMEV	172.15	3.098	JOCVAB	172.388	3.192
JODBEK	169.193	3.113	JODBEK	154.574	3.262	JODBEK	150.326	3.242	JODBEK	153.689	3.203	JODBEK	165.746	3.007
JODBEK	167.308	3.293	JODDAJ	159.521	3.271	JODBEK	167.056	3.159	JODBEK	165.851	2.652	JODBEK	156.657	3.289
JODLUL	152.235	2.724	JODLUL	154.961	3.371	JODDAJ	160.189	3.138	JODDAJ	151.311	3.138	JODLUL	178.327	2.699
JODMEW	166.692	2.783	JODMEW	157.481	2.85	JODLUL	172.163	2.699	JODLUL	155.525	2.883	JODLUL	163.472	2.754
JODMIA	161.733	2.902	JODMIA	150.469	2.828	JODMEW	172.835	2.76	JODMEW	170.081	2.76	JODMIA	157.172	2.902
JODMIA	152.606	3.022	JODMIA	165.531	2.827	JODMIA	158.331	2.839	JODMIA	153.98	2.85	JODMIA	154.61	2.947
JODQEZ	167.812	3.128	JODWAB	154.853	3.292	JODMIA	155.028	2.863	JODMIA	159.935	2.828	JODMIA	168.401	2.852
JODZAF	157.066	3.088	JODZAF	159.96	2.787	JODWAB	161.668	3.363	JODZAF	155.97	3.455	JODZAF	156.438	2.933
JOFDIV	151.441	3.124	JOFFAO	158.17	2.784	JODZAF	151.724	3.497	JODZAF	155.086	2.803	JOFDIU	154.544	3.178
JOFFIW	168.654	2.866	JOFFIW	157.87	2.791	JOFFAO	167.823	2.838	JOFFES	152.029	2.887	JOFFES	169.574	2.819
JOFHIY	155.916	3.243	JOFXAE	166.942	2.671	JOFFIW	151.561	3.48	JOFHIY	159.788	3.419	JOFHIY	162.73	3.21
JOGBUD	170.787	3.134	JOGBUD	161.453	3.296	JOFXAE	160.398	3.283	JOFZAG	153.568	3.1	JOGBUD	154.883	3.498
JOGFUH	158.129	2.968	JOGFUH	164.167	2.968	JOGBUD	151.008	3.214	JOGCIR	160.848	3.203	JOGFAP	170.368	3.292
JOGKOI	158.706	2.983	JOGKOI	150.38	3.025	JOGHOE	153.417	3.143	JOGHOE	158.715	3.026	JOGKOI	151.66	3.16
JOGLIB	167.107	2.961	JOGLIB	161.55	3.23	JOGLIB	156.158	3.49	JOGLIB	163.535	3.495	JOGLIB	151.052	3.184
JOGLOH	160.895	3.136	JOGLOH	174.181	2.952	JOGLIB	162.984	3.024	JOGLIB	164.349	3.32	JOGLIB	167.036	3.042
JOGLOH	164.581	3.475	JOGLOH	155.721	2.749	JOGLOH	163.126	3.023	JOGLOH	157.841	3.079	JOGLOH	162.124	3.317
JOGNAW	150.823	2.917	JOGNAW	173.126	3.044	JOGNAW	156.149	2.92	JOGNAW	151.533	2.947	JOGNAW	171.853	3.056
JOGNEA	152.019	3.023	JOGNEA	154.878	3.229	JOGNEA	150.07	3.354	JOGNEA	165.859	3.153	JOGNEA	168.055	3.269

JOGZOY	152.962	3.281	JOHCUI	164.641	3.414	JOGSII	175.964	2.874	JOGSII	165.419	3.442	JOGSII	155.356	2.819
JOHMAU	172.118	3.103	JOHMAU	172.815	3.254	JOHDIV	156.374	3.135	JOHGIX	171.561	3.159	JOHMAU	163.381	2.872
JOHMAU	152.88	3.404	JOHMAU	165.753	3.309	JOHMAU	157.445	3.053	JOHMAU	165.609	3.103	JOHMAU	165.147	3.372
JOJGIC	169.107	3.396	JOJGOF	156.67	3.443	JOHMAU	169.462	3.416	JOHMAU	172.695	2.91	JOHMAU	159.184	3.107
JOJGOF	157.766	3.472	JOJGOF	154.194	3.23	JOJGOF	157.215	3.456	JOJGOF	167.405	2.85	JOJGOF	173.795	3.002
JOJGOF	173.751	2.75	JOJGOF	153.985	2.843	JOJGOF	158.078	2.67	JOJGOF	165.743	2.646	JOJGOF	170.8	3.483
JOJGOF	164.804	3.24	JOJGOF	169.891	3.176	JOJGOF	159.649	3.382	JOJGOF	154.181	3.085	JOJGOF	159.14	3.443
JOJGOF	151.063	3.238	JOJGOF	158.452	2.975	JOJGOF	154.636	2.895	JOJGOF	150.901	3.423	JOJGOF	152.558	2.964
JOJHIA	173.452	3.31	JOJHIA	150.933	2.894	JOJGOF	154.379	2.726	JOJGOF	154.527	2.987	JOJGOF	166.578	3.378
JOJPII	171.426	3.334	JOJPII	151.223	3.099	JOJNUS	152.711	3.007	JOJNUS	160.804	2.933	JOJPII	163.193	3.015
JOJXUD	158.246	3.437	JOKFIZ	166.291	3.332	JOJQUX	169.074	3.077	JOJXOZ	161.228	3.162	JOJXUD	165.425	2.907
JOKRUA	169.757	3.342	JOKSAH	154.279	3.077	JOKFIZ	151.826	3.208	JOKFOF	156.94	3.266	JOKREH	153.996	3.487
JOKSAH	161.874	2.898	JOKSAH	157.613	3.483	JOKSAH	155.894	2.781	JOKSAH	175.973	3.405	JOKSAH	162.184	3.272
JOKTUZ	164.116	3.249	JOKTUZ	158.161	3.495	JOKSAH	165.156	3.408	JOKSAH	161.983	3.262	JOKSAH	153.658	2.79
JOKZUE	167.407	2.93	JOLJON	159.408	2.816	JOKTUZ	160.777	3.14	JOKTUZ	153.756	3.317	JOKTUZ	166.289	3.178
JOLQUA	170.156	3.066	JOLZAL	165.222	3.213	JOLJON	165.912	2.804	JOLJON	175.005	2.816	JOLQUA	159.555	3.118
JOMNUW	160.406	3.483	JOMNUW	151.127	3.016	JOLZAL	158.249	3.414	JOLZAL	154.071	3.213	JOMDIA	150.943	3.046
JOMPUA	155.913	2.972	JONGEC	159.985	3.044	JOMPIN	174.872	2.847	JOMPIN	162.837	2.805	JOMPIN	160.299	3.114
JONLEF	153.316	2.672	JONLEF	157.318	2.969	JONGEC	156.114	3.249	JONLEF	155.782	3.236	JONLEF	162.433	3.035
JONNIJ	168.177	2.917	JONNIJ	154.039	3.102	JONLEF	158.57	3.026	JONNIJ	150.674	3.478	JONNIJ	158.646	3.306
JONNIJ01	154.241	3.231	JONNIJ01	153.929	2.907	JONNIJ	164.832	2.994	JONNIJ	161.714	2.878	JONNIJ01	158.185	3.06
JONNUV	158.105	3.497	JONNUV	159.33	3.303	JONNIJ01	166.079	2.915	JONNIJ01	171.566	3.202	JONNUV	152.042	2.886
JONNUV	152.726	2.784	JONNUV	156.038	3.303	JONNUV	160.737	3.219	JONNUV	150.891	3.199	JONNUV	164.151	2.94
JONNUV0	172.072	2.78	JONREJ	171.612	2.891	JONNUV0	150.934	3.229	JONNUV0	166.466	3.286	JONNUV0	156.83	3.374
JONREJ	171.676	2.891	JONSIO	160.182	3.033	JONREJ	154.217	3.127	JONREJ	153.231	3.01	JONREJ	152.116	3.01
JOPFAV	150.155	3.1	JOPFAV	156.583	3.141	JONSIO	155.788	2.946	JONVAL	165.188	2.84	JOPFAV	154.346	3.177
JOPFOJ	168.334	2.883	JOPFOJ	157.316	2.778	JOPFAV	164.194	3.271	JOPFID	163.432	3.078	JOPFID	150.089	3.118
JOPMIN	168.565	2.921	JOPMIN	177.621	2.95	JOPFOJ	155.735	3.006	JOPFOJ	154.469	3.051	JOPFOJ	167.666	2.783
JOPTIR	160.859	2.964	JOPVUI	169.271	3.266	JOPMUZ	158.993	3.239	JOPMUZ	161.9	2.51	JOPNAG	171.635	2.831
JOPYOC	158.39	3.014	JOPYOC	157.592	2.818	JOPYES	160.67	3.122	JOPYES	151.841	3.122	JOPYES	162.69	2.893
JOPZAT	168.596	3.437	JOPZAT	152.006	3.494	JOPYUM	168.201	3.434	JOPYUM	152.01	3.484	JOPYUM	154.064	2.74
JOQBIE	153.253	2.747	JOQBUQ	153.312	3.337	JOPZAT	154.079	2.75	JOQBIE	151.535	3.499	JOQBIE	168.101	3.449
JOQCOH0	166.59	2.897	JOQCOH0	160.99	2.754	JOQCIF	153.863	3.33	JOQCIF	153.834	3.123	JOQCOH0	156.979	2.972
JOQCOH0	163.405	3.354	JOQCOH0	150.403	2.938	JOQCOH0	163.775	3.232	JOQCOH0	156.72	3.253	JOQCOH0	159.449	3.248
JOQCOH0	161.853	3.41	JOQCOH0	159.605	3.421	JOQCOH0	155.235	3.37	JOQCOH0	155.774	2.638	JOQCOH0	152.34	3.116
JOQMIN	151.536	3.326	JOQMIN	151.603	3.305	JOQCOL	172.897	2.653	JOQJII	166.63	3.458	JOQMIN	150.61	3.217
JORLOR	152.474	3.135	JORLOR	152.084	3.447	JOQVEU	170.857	3.223	JOQVEU	158.547	3.284	JORLOR	161.595	3.469
JOSFAC	159.816	3.022	JOSFAC	152.356	3.319	JORMUC	160.09	3.256	JORMUC	160.134	3.242	JOSFAC	168.371	2.866
JOSMIN	153.115	3.187	JOSMIN	164.149	3.275	JOSFAC	156.584	2.854	JOSFAC	154.894	3.134	JOSMIN	150.587	2.933
JOSXIY	150.426	3.413	JOSZUP	159.567	3.437	JOSMIN	150.349	3.275	JOSQUG	157.538	3.418	JOSQUG	162.836	2.812
JOTKEL	155.906	2.757	JOTXEX	152.399	2.866	JOTDEF	154.476	3.486	JOTDEF	159.999	3.486	JOTKAH	160.177	3.056
JOVHOT	168.287	3.137	JOVHUZ	169.611	3.182	JOTYEZ	173.892	3.314	JOTYEZ	163.83	3.413	JOVHOT	152.586	3.325
JOVHUZ	158.015	3.205	JOVJEL	176.658	3.315	JOVHUZ	176.084	3.385	JOVHUZ	152.463	3.182	JOVHUZ	162.277	3.196
JOVJEL	170.264	2.987	JOVJEL	173.153	3.059	JOVJEL	169.538	2.863	JOVJEL	169.161	3.022	JOVJEL	153.865	3.153
JOVJIP	176.142	3.342	JOVJIP	164.298	3.346	JOVJEL	172.388	3.057	JOVJEL	164.911	2.878	JOVJIP	159.507	3.286
JOVVIB	170.037	2.957	JOVVOH	158.039	3.114	JOVVIB	155.167	2.792	JOVVIB	176.119	2.885	JOVVIB	150.49	2.86
JOVVOH	158.847	3.034	JOWBAY	156.933	3.323	JOVVOH	161.974	2.928	JOVVOH	153.231	3.202	JOVVOH	166.911	2.865
JOWHUB	150.72	2.773	JOWHUB	170.191	2.664	JOWBAY	152.721	3.082	JOWBAY	168.222	3.076	JOWBAY	173.245	3.148
JOWKAL	166.844	2.901	JOWKOZ	158.89	2.676	JOWJIS	160.917	3.082	JOWJIS	150.67	3.082	JOWJUE	153.854	2.931
JOWMIV	153.236	3.407	JOWMIV	165.349	2.799	JOWLAM	156.073	2.668	JOWLAM0	163.785	3.119	JOWLUG	154.331	3.426
JOXZUV	153.701	3.323	JOXZUV	161.351	3.147	JOWREW	163.109	2.99	JOXBED	157.993	3.145	JOXZUV	155.936	2.969
JOYHEO	159.918	3.292	JOYHEO	159.842	3.296	JOYHAK	157.071	2.779	JOYHAK	160.394	3.063	JOYHEO	161.131	3.043
JOYZEG	157.144	3.302	JOZBAE	168.172	2.804	JOYHEO	157.583	2.74	JOYHEO	168.194	3.173	JOYZAB	174.076	3.204
JUBCAM	163.939	3.106	JUBCAM	169.782	2.809	JOZKIS	166.701	3.152	JOZKIS	153.355	3.172	JOZZUX	158.838	3.37
JUBCAM	163.213	3.23	JUBCAM	150.788	3.142	JUBCAM	153.924	2.809	JUBCAM	150.22	2.94	JUBCAM	169.404	2.994
JUBJEZ	153	3.46	JUBKIA	156.028	3.177	JUBJAR	178.09	3.09	JUBJEZ	158.357	2.86	JUBJEZ	158.184	2.798
JUBQAB0	161.871	3.33	JUBQAB0	162.197	3.327	JUBKIA	177.082	2.887	JUBKIA01	156.374	3.154	JUBKIA01	177.46	2.862
JUBQOQ	160.218	2.95	JUBQOQ	165.61	3.324	JUBQAC	167.151	2.814	JUBQAC	161.251	2.879	JUBQOQ	163.318	3.09
JUBQUW	166.562	2.746	JUBQUW	168.748	3.302	JUBQOQ	162.731	2.958	JUBQUW	153.338	3.457	JUBQUW	167.094	3.335
JUBTOR	155.149	3.199	JUBVAF	150.582	3.063	JUBTOR	150.399	3.26	JUBTOR	157.626	2.647	JUBTOR	163.268	3.491
JUCWIR	174.787	3.404	JUCWUD	172.838	3.478	JUBVEJ	155.77	2.965	JUCQEF	157.165	3.105	JUCWIP	161.198	3.356
JUDCAQ	171.493	3.084	JUDCAQ	150.936	3.236	JUCXEO	161.067	3.093	JUCXIS	155.71	2.818	JUCXIS	161.592	3.439
JUDMED	164.337	2.931	JUDMUQ	153.223	3.475	JUDCAQ	176.553	2.962	JUDLAX	153.531	3.336	JUDMED	161.045	2.931
JUDMUT	160.698	3.206	JUDPIH	160.863	2.901	JUDMUQ	157.655	3.033	JUDMUQ	170.182	2.786	JUDMUT	158.72	3.205
JUDQEE	165.845	2.699	JUDSIM	171.691	3.332	JUDPIH	151.251	3.077	JUDQEE	153.547	3.303	JUDQEE	170.406	2.908
JUDYAK	159.617	2.989	JUDYAK	167.758	3.369	JUDYAK	152.497	3.353	JUDYAK	156.733	3.096	JUDYAK	171.543	3.415
JUFMAC	165.866	3.388	JUFMAC	158.824	3.319	JUDYAK	166.704	2.831	JUDZAL	173.233	3.336	JUFMAC	156.694	2.652
JUFWAK	165.835	3.296	JUFWAK	172.357	3.363	JUFMOQ	158.148	2.635	JUFMOQ	166.903	3.469	JUFMOQ	161.544	3.422
JUFWEO	167.454	3.109	JUFWEO	163.706	3.086	JUFWAK	156.775	2.979	JUFWAK	150.335	3.362	JUFWAK	159.455	2.849
JUFWIS	153.193	3.46	JUFWIS	165.619	3.461	JUFWEO	163.005	3.139	JUFWEO	159.167	2.868	JUFWEO	153.224	2.809
JUFWOY	158.963	3.106	JUFWOY	167.48	2.954	JUFWIS	163.778	3.313	JUFWIS	152.033	2.865	JUFWIS	164.775	2.776
JUGQIL	162.287	3.252	JUGWEN	159.113	3.245	JUFWOY	160.573	3.266	JUGGIB	157.955	3.36	JUGGUN	167.249	2.873
JUHSEN	157.937	2.84	JUHSEN	159.381	3.356	JUGWEN	169.447	2.892	JUGWEN	164.562	3.44	JUHBUL	155.527	2.898
JUHSEN	166.088	3.15	JUHSEN	164.271	2.967	JUHSEN	152.109	3.08	JUHSEN	158.384	2.895	JUHSEN	158.66	2.782
JUHSEN	165.146	3.08	JUHSEN	155.309	3.022	JUHSEN	160.586	2.998	JUHSEN	158.73	3.356	JUHSEN	155.38	3.048
JUHTIS	160.329	3.37	JUHTIS	160.08	3.379	JUHTAK	161.514	2.873	JUHTAK	165.492	3.469	JUHTAK	175.108	3.281
JUHXES	152.539	2.642	JUHXOC	157.458	3.47	JUHVIT	159.502	2.828	JUHWOY	161.044	2.881	JUHXES	150.912	2.732
JUJGIG	150.145	2.963	JUJGIG	150.227	3.269	JUHXOC	151.324	2.914	JUJGIG	154.65	3.058	JUJGIG	152.547	2.809
JUJRUD	157.986	3.389	JUJRUD	150.405	2.898	JUJJUW	170.461	3.354	JUJJUW	153.392	3.182	JUJMAE	153.564	3.133

JUKGIJ01	168.703	3.238	JUKGIJ01	162.721	2.909	JUKBEA	163.141	2.995	JUKBEA	176.271	2.995	JUKGIJ01	150.542	2.779
JUKGIJ01	159.36	2.907	JUKGIJ01	162.6	2.909	JUKGIJ01	160.929	2.831	JUKGIJ01	154.913	2.713	JUKGIJ01	156.465	3.423
JUKGIJ01	159.483	3.24	JUKGIJ01	156.358	2.831	JUKGIJ01	166.64	3.065	JUKGIJ01	154.6	3.296	JUKGIJ01	176.546	3.352
JUKGIJ01	151.992	2.801	JUKGUT	167.951	2.81	JUKGIJ01	153.854	3.24	JUKGIJ01	174.57	3.125	JUKGIJ01	158.412	3.191
JUKHAA	168.42	3.082	JUKHEE	161.807	2.716	JUKGUT	150.156	2.947	JUKGUT	157.059	3.456	JUKHAA	157.973	3.346
JULQUE	158.507	3.041	JULREP	167.048	3.41	JUKHII	162.165	3.333	JUKMIO	153.572	3.369	JULQOY	158.432	3.481
JUMGAB	155.855	3.262	JUMGAB	163.025	3.38	JULREP	174.625	3.038	JULWOC	165.568	3.167	JULWUI01	160.765	3.306
JUMMEM	163.315	2.784	JUMYIZ	167.969	2.843	JUMJOT	166.907	3.428	JUMJOT	156.272	3.428	JUMJOT	169.144	3.246
JUNMOY	169.237	2.917	JUNMOY	157.561	3.269	JUNFAC	153.044	3.111	JUNMOY	157.683	2.933	JUNMOY	169.52	3.243
JUNMOY	152.412	3.015	JUNMOY	153.06	2.818	JUNMOY	157.785	3.168	JUNMOY	153.084	3.49	JUNMOY	155.897	2.818
JUNMOY	153.725	3.102	JUNMOY	167.738	3.393	JUNMOY	157.012	2.996	JUNMOY	177.546	2.812	JUNMOY	163.603	2.914
JUNMOY	150.183	3.019	JUNMOY	166.382	2.98	JUNMOY	162.305	2.928	JUNMOY	166.664	2.933	JUNMOY	152.143	2.97
JUNNOZ	169.09	3.064	JUNNOZ	161.009	2.997	JUNNOZ	150.69	3.301	JUNNOZ	171.57	2.898	JUNNOZ	150.703	2.849
JUNVET	153.781	3.006	JUPWID	158.222	3.366	JUNNOZ	152.317	2.741	JUNNOZ	163.438	3.269	JUNVET	157.909	2.961
JUPWOK	168.236	3.192	JUPWOK	161.77	3.15	JUPWOK	166.315	2.796	JUPWOK	168.263	2.796	JUPWOK	153.692	3.151
JUPYIC	151.846	3.497	JUPYOI	170.852	3.147	JUPXUN	167.141	3.375	JUPXUN	162.797	2.9	JUPYEA	172.787	3.185
JUQGAD	152.015	3.239	JUQKUD	169.149	3.475	JUPYOI	175.838	2.878	JUPZUR	152.975	3.431	JUQGAD	165.732	3.293
JURHAF	160.661	2.702	JURHAF	160.215	2.826	JUQPOE	160.05	2.985	JUQXIF	154.301	3.243	JUQXIF	155.681	3.321
JURHAF	154.452	2.702	JURHAF	163.518	3.22	JURHAF	166.866	3.222	JURHAF	165.715	3.22	JURHAF	154.856	3.389
JURWAU	152.338	3.098	JURWAU	177.884	3.154	JURHEJ	157.517	3.233	JURSUK	166.774	3.128	JURSUK	168.38	3.376
JUSWID	150.725	3.026	JUSWID	163.772	2.919	JURWAU	154.437	3.154	JUSTIC	168.639	3.314	JUSTIC	166.715	3.489
JUTCAF	159.319	3.199	JUTJOZ	174.695	3.038	JUSWID	169.943	2.937	JUSWID	162.118	3.413	JUTCAF	169.709	2.838
JUTQAQ	156.609	3.25	JUTSAW	159.679	3.051	JUTKUE	169.133	3.36	JUTKUE	159.659	2.894	JUTKUE	154.933	2.894
JUVQAV	151.95	3.01	JUVQAV	166.243	2.706	JUVQAV	161.833	3.108	JUVQAV	150.764	2.722	JUVQAV	164.156	3.021
JUWJUI	157.195	3.436	JUWJUI	161.501	3.113	JUVQAV	153.927	3.208	JUVQAV	153.005	2.932	JUWJUI	155.723	2.71
JUWKIX	168.61	3.436	JUWKIX	152.14	2.818	JUWJUI	165.734	3.102	JUWKAP	162.201	3.209	JUWKAP	164.333	3.47
JUWKUJ	150.223	2.693	JUWKUJ	160.391	3.334	JUWKUJ	174.5	3.204	JUWKUJ	157.994	3.425	JUWKUJ	174.553	3.21
JUWLAQ	150.723	3.258	JUWLAQ	165.996	3.149	JUWKUJ	151.458	2.949	JUWKUJ	150.291	3.165	JUWLAQ	165.13	3.106
JUWLAQ	151.982	3.254	JUWLAQ	159.049	3.424	JUWLAQ	166.645	2.91	JUWLAQ	152.536	2.97	JUWLAQ	160.2	3.286
JUXCEM	163.587	2.909	JUXCEM	155.56	3.013	JUWLAQ	152.643	2.856	JUWTOK	151.394	3.376	JUXBOW	172.03	3.039
JUXCIQ	162.944	3.244	JUXCIQ	151.765	2.983	JUXCIQ	165.22	2.941	JUXCIQ	151.793	3.182	JUXCIQ	164.98	3.17
JUXCOW	158.246	3.391	JUXCOW	175.912	2.879	JUXCIQ	155.351	2.811	JUXCOW	154.103	3.364	JUXCOW	169.605	3.41
JUXCOW	150.299	3.351	JUXCOW	170.44	3.036	JUXCOW	151.917	2.995	JUXCOW	151.07	3.453	JUXCOW	150.835	3.212
JUXNEX	161.686	3.17	JUXNEX	157.069	3.401	JUXGUG	168.463	3.104	JUXHAN	151.442	3.17	JUXNEX	159.93	3.187
JUXNEX	158.982	3.233	JUXNEX	152.67	2.786	JUXNEX	163.535	3.026	JUXNEX	166.402	3.06	JUXNEX	152.457	3.051
JUXWEG	154.011	3.14	JUXWIK	150.855	3.173	JUXPEA	152.425	3.279	JUXRON	173.663	3.255	JUXRON	157.764	3.353
JUXZEJ	172.32	2.725	JUXZEJ	163.679	3.481	JUXXEH	163.813	2.95	JUXZEJ	157.825	2.885	JUXZEJ	157.826	2.983
JUXZEJ	154.393	3.239	JUXZUB	161.955	2.819	JUXZEJ	163.499	3.176	JUXZEJ	171.451	3.38	JUXZEJ	163.297	2.92
JUYCUD	153.037	3.18	JUYFIS	155.896	3.398	JUYBEO	158.306	3.107	JUYBEO	157.835	2.837	JUYBEO	170.621	3.145
JUYGOC	170.693	3.476	JUYLEX	172.498	2.755	JUYFIS	154.749	3.264	JUYFIS	170.716	3.249	JUYFIS	175.564	3.278
JUZCAM	178.007	3.336	JUZCIU	165.254	3.481	JUYNOI	166.231	3.347	JUYNOI	156.37	2.685	JUYNOI	167.031	2.875
JUZMAS	171.445	3.161	JUZMAS	165.008	2.832	JUZDAN	177.059	3.361	JUZDER	170.084	3.408	JUZMAS	153.217	2.832
KABHAX	153.882	3.498	KABHAX	156.964	2.735	JUZVAE	166.582	3.001	KABCUQ0	165.493	2.996	KABDEY	154.249	3.177
KABHEB	153.751	2.958	KABHOM	150.31	3.139	KABHAX	167.644	2.735	KABHAX	153.957	3.328	KABHEB	154.059	2.94
KABKEI	152.881	3.199	KABMEK	168.941	3.247	KABHOM	156.03	3.113	KABHOM	157.086	3.017	KABKEI	162.509	3.475
KABSOW	155.166	3.138	KABWAL	158.648	3.391	KABSEQ	162.093	3.281	KABSOW	152.908	2.927	KABSOW	169.987	3.05
KABWAL	162.922	3.023	KABWAL	167.527	2.985	KABWAL	160.964	2.961	KABWAL	161.283	3.333	KABWAL	160.169	3.017
KACGOM	153.562	3.392	KACHON	164.409	3.306	KABWAL	163.489	3.341	KACGOM	158.677	2.728	KACGOM	151.316	3
KACHON	158.506	3.306	KACHON	168.085	3.201	KACHON	156.255	2.757	KACHON	171.162	2.843	KACHON	164.208	3.348
KACVEQ	156.45	3.387	KACVEQ	160.28	2.996	KACKOR	164.649	3.354	KACKOR	169.163	3.354	KACKOR	163.825	3.016
KACWIX	170.692	3.384	KACWIX	166.835	3.384	KACVEQ	175.485	2.688	KACVEQ	173.257	3.289	KACWIX	164.542	2.998
KACWUJ	160.848	3.375	KACWUJ	168.865	3.375	KACWOD	165.212	3.031	KACWOD	163.669	3.373	KACWOD	169.987	3.373
KACXAQ	165.884	3.025	KADSIU	165.808	2.798	KACWUJ	165.404	2.999	KACXAQ	169.434	3.389	KACXAQ	159.204	3.389
KAFDOK	168.908	3.367	KAFDOK	154.829	3.249	KAFDOK	150.779	3.345	KAFDOK	158.173	3.367	KAFDOK	157.843	3.27
KAFNOV	159.106	2.838	KAFPIQ	173.071	3.092	KAFGEE	150.445	3.479	KAFHIK	159.864	3.091	KAFHIK	158.086	2.961
KAFYOI	171.375	3.186	KAGKEI	151.674	3.039	KAFPOZ	157.851	3.475	KAFVOD	169.674	3.194	KAFYOI	150.032	3.248
KAGYIA	160.781	2.923	KAGYIA	160.985	3.072	KAGKEI	151.184	3.22	KAGRAQ	169.295	3.446	KAGXIC	163.12	3.354
KAHGAF	156.43	2.966	KAHGAF	158.143	3.44	KAGYIA	156.198	3.283	KAGYIF	150.079	3.441	KAGYIF	167.533	3.239
KAHGIN	174.999	3.329	KAHGOT	162.869	3.334	KAHGAF	155.594	3.012	KAHGAF	154.692	3.425	KAHGEK	169.916	2.761
KAHGOT	168.66	3.193	KAHGOT	175.269	3.059	KAHGOT	157.158	3.285	KAHGOT	157.536	3.364	KAHGOT	162.49	3.233
KAHLIR	152.825	3.285	KAHLIR	152.783	3.089	KAHLIR	156.542	3.226	KAHLIR	168.87	2.909	KAHLIR	178.954	3.226
KAHXUM	167.64	3.478	KAJKIQ	168.604	2.939	KAHXIA	158.265	3.361	KAHXIA	152.022	3.302	KAHXIA	154.549	2.999
KAJKIQ	175.176	3.208	KAJKIQ	167.691	3.431	KAJKIQ	172.933	3.479	KAJKIQ	161.296	2.895	KAJKIQ	163.51	3.467
KAJNIV	158.608	3.363	KAJNIV	166.344	3.11	KAJNAK	176.364	3.162	KAJNAK	152.576	3.425	KAJNAK	151.408	3.024
KAJNIV	152.373	3.058	KAJNOC	167.315	3.427	KAJNIV	152.46	3.166	KAJNIV	166.14	3.363	KAJNIV	163.933	2.889
KAJWOJ	155.999	2.863	KAJYUS	164.714	3.015	KAJPAM	176.027	3.025	KAJTOG	162.005	2.911	KAJWOJ	151.736	2.596
KAJZAZ	155.411	2.894	KAJZAZ	155.198	3.21	KAJYUS	154.968	3.236	KAJYUS	161.715	3.205	KAJZAZ	156.737	2.88
KAJZAZ	169.433	2.982	KAJZAZ	170.846	3.269	KAJZAZ	153.844	3.151	KAJZAZ	153.904	3.093	KAJZAZ	162.437	2.769
KAJZAZ	151.402	3.119	KAJZAZ	169.632	2.921	KAJZAZ	162.776	3.151	KAJZAZ	153.715	3.229	KAJZAZ	162.375	2.803
KAJZAZ	153.672	2.949	KAJZAZ	166.201	3.02	KAJZAZ	160.984	2.769	KAJZAZ	166.167	3.057	KAJZAZ	151.887	3.147
KAJZAZ	162.721	3.128	KAJZAZ	158.801	2.749	KAJZAZ	154.857	3.369	KAJZAZ	171.436	3.102	KAJZAZ	163.969	2.875
KAKFUX	174.062	2.982	KAKFUX	155.192	3.116	KAJZAZ	170.824	2.923	KAKBIJ	151.185	3.497	KAKBIJ	171.833	3.486
KAKGUZ	169.654	2.892	KAKGUZ	168.446	3.139	KAKFUX	172.02	3.01	KAKGUZ	175.941	2.736	KAKGUZ	153.56	2.823
KAKKOW	150.911	2.954	KAKKOW	150.766	2.946	KAKGUZ	165.985	2.736	KAKKIQ	153.536	3.342	KAKKIQ	152.897	3.353
KALTAS	150.337	2.815	KALTOH	154.506	3.37	KAKTAS0	152.349	2.969	KAKVUN	156.255	3.05	KAKXID	157.909	3.001
KAMMUK	168.569	3.156	KAMMUK	152.054	3.32	KALTOH	150.556	2.986	KAMMUK	161.6	3.228	KAMMUK	159.975	2.85
KAMTUO	170.575	3.122	KAMVOL	160.91	2.643	KAMMUK	169.47	3.29	KAMMUK	155.139	2.858	KAMTUO	174.657	2.854
KAMWEC	174.486	3.016	KAMWEC	154.551	3.181	KAMVOL	151.951	2.953	KAMVOL	152.969	2.794	KAMWAY	157.393	3.004
KAMYII	156.904	3.497	KAMYII	151.423	3.04	KAMWEC	163.146	3.336	KAMWEC	166.327	2.775	KAMWEC	156.639	2.853

KANCIP	154.973	3.261	KANCIP	161.071	3.302	KAMYII	152.167	3.096	KAMYOQ	155.511	3.258	KAMYOQ	154.157	3.29
KANHIS	168.391	2.894	KANHIS	176.04	2.897	KANDEM	155.524	3.076	KANHIS	164.423	2.776	KANHIS	163.368	2.749
KANHIS	152.608	3.464	KANHIS	163.159	3.129	KANHIS	159.719	2.855	KANHIS	166.623	3.125	KANHIS	171.305	3.465
KANHIS	150.736	3.464	KANHOY	162.407	2.778	KANHIS	160.4	2.868	KANHIS	151.575	2.931	KANHIS	172.555	3.275
KANHOY	159.749	2.835	KANHOY	166.826	3.122	KANHOY	163.435	2.741	KANHOY	167.813	2.885	KANHOY	175.894	2.896
KANHOY	164.037	2.891	KANHOY	151.437	2.946	KANHOY	171.768	3.459	KANHOY	151.399	3.43	KANHOY	163.274	3.191
KANYIL	169.139	3.278	KANYIL	164.135	2.868	KANHOY	171.699	3.275	KANHOY	152.968	3.43	KANSOI	155.353	2.783
KAPROJ	154.725	3.016	KAPROJ	151.54	3.395	KAPROJ	168.475	2.839	KAPROJ	176.509	2.839	KAPROJ	160.185	2.834
KAPRUR	165.4	3.202	KAPRUR	160.482	3.462	KAPROJ	173.819	2.836	KAPROJ	177.479	2.824	KAPRUR	157.268	2.819
KAPSIE	162.586	3.405	KAPSIE	160.425	2.962	KAPRUR	161.053	2.819	KAPRUR	173.407	3.125	KAPRUR	165.42	3.155
KAQBEM	161.282	2.84	KAQBEM	167.15	3.264	KAPSIE	161.02	3.039	KAQBEM	158.684	3.024	KAQBEM	155.246	3.219
KAQBEM	153.122	2.933	KAQBEM	154.508	3.427	KAQBEM	160.356	3.445	KAQBEM	159.588	2.69	KAQBEM	154.37	3.451
KAQBEM	169.203	2.799	KAQBEM	164.708	2.759	KAQBEM	157.857	3.404	KAQBEM	158.291	3.359	KAQBEM	159.639	2.952
KAQBUA	157.876	3.094	KAQBUA	156.756	2.811	KAQBEM	154.737	3.203	KAQBUA	150.815	2.979	KAQBUA	155.046	2.934
KAQJUJ	156.545	3.07	KAQJUK	155.043	3.095	KAQJUJ	152.044	2.952	KAQJUJ	168.054	3.378	KAQJUJ	152.242	3.07
KAQJUK	154.013	2.951	KAQJUK	154.302	3.165	KAQJUK	159.479	3.06	KAQJUK	151.978	3.14	KAQJUK	154.663	3.095
KAQKEV	150.492	3.11	KAQKEV	163.324	3.162	KAQJUK	164.545	2.949	KAQJUK	157.139	3.067	KAQJUK	166.514	3.025
KAQNOG	158.104	2.695	KAQNOG	154.109	3.022	KAQMUK	169.387	3.346	KAQMUK	161.022	3.34	KAQNAV	161.576	2.885
KAQNOG	156.714	2.695	KAQZAH	174.346	3.237	KAQNOG	158.314	3.45	KAQNOG	172.047	3.369	KAQNOG	170.553	3.443
KARLUN	164.365	3.007	KARVOR	158.353	3.361	KARCIQ	162.011	3.331	KARCIQ01	161.924	3.306	KARHIU01	151.762	3.416
KARYUZ	157.756	3.187	KASCUD	157.843	3.19	KARVOR	153.088	2.954	KARVOR	175.16	3.213	KARYOT	165.881	3.444
KASCUD	154.571	3.47	KASGIY	165.005	2.941	KASCUD	161.671	3.255	KASCUD	153.789	2.914	KASCUD	162.511	2.936
KASGIY	161.471	2.908	KASGIY	167.052	3.218	KASGIY	152.038	2.989	KASGIY	155.844	2.96	KASGIY	152.083	2.892
KATCAN	154.272	3.042	KATDIV	157.105	3.159	KASQOM	154.483	2.639	KASQOM	173.222	3.224	KASQOM	165.93	3.018
KATMEZ	157.152	3.008	KATMEZ	154.179	2.87	KATDIV	171.669	3.044	KATFAP	150.295	2.908	KATFAP	155.492	2.779
KATNAV	162.292	2.943	KATNAV	153.758	2.915	KATMEZ	156.647	3.278	KATNAV	162.8	3.289	KATNAV	158.56	3.193
KAWBUJ	167.129	3.243	KAWBUJ	153.582	3.256	KAVQUX	151.537	2.972	KAVVEJ	161.62	3.081	KAWBUJ	156.032	3.19
KAWHOJ	152.078	3.062	KAWHOJ	169.929	2.924	KAWFOF	169.463	3.419	KAWHOJ	170.326	2.746	KAWHOJ	167.254	2.746
KAXFEU	153.374	2.702	KAXFEU	171.281	2.88	KAWKUQ	155.556	3.216	KAWSUY	167.81	3.467	KAXDIW	151.067	3.349
KAYGOK	158.398	2.933	KAYLEC	153.831	3.13	KAXLAY	158.856	3.461	KAXLAY	156.251	2.978	KAXLAY	169.295	3.145
KAYLIG	153.724	3.313	KAYLIG	173.259	2.919	KAYLEC	153.478	3.254	KAYLEC	159.311	3.279	KAYLEC	163.344	3.339
KAYWAK	170.005	2.796	KAYWEO	168.462	2.821	KAYLIG	152.468	3.084	KAYLIG	157.268	2.916	KAYREH	151.616	2.954
KAZNAD	150.573	3.087	KAZREL	159.058	2.979	KAZKOP	163.393	3.355	KAZKOP	157.93	3.059	KAZNAD	154.681	2.997
KAZWOA	169.779	2.777	KAZWOA	169.725	2.777	KAZSAJ	169.512	2.923	KAZSUD	155.63	3.324	KAZSUD	155.244	3.231
KEBQOZ	154.841	3.235	KECBAV	158.763	3.282	KEBHOR	152.201	3.362	KEBHOR	171.433	2.953	KEBQAN	151.093	2.904
KECNUE	151.133	2.81	KECPIU	156.456	3.085	KECBAV	163.578	3.402	KECNUE	151.147	2.794	KECNUE	153.514	2.76
KECQIV	173.179	2.929	KECQIV	153.55	3.408	KECQER	156.793	3.079	KECQIV	153.677	3.26	KECQIV	156.456	2.747
KECROA	157.581	2.727	KECROA	161.903	2.727	KECQIV	153.679	2.747	KECROA	157.32	3.49	KECROA	154.131	3.165
KECVEW	156.458	2.998	KECXAR	165.746	3.118	KECSIU	153.304	3.368	KECSIU	164.013	3.467	KECVEW	170.75	2.895
KECXEV	150.342	3.227	KEDCAB	161.636	3.08	KECXAR	169.325	3.233	KECXAR	153.571	3.363	KECXEV	156.784	3.259
KEDPIT	150.698	3.227	KEDPIT	156.928	3.268	KEDGUZ	151.809	3.214	KEDGUZ	166.518	3.179	KEDNUE	157.873	2.943
KEDPIT	165.412	2.966	KEDPIT	150.295	2.949	KEDPIT	160.268	3.047	KEDPIT	152.822	3.265	KEDPIT	164.05	2.944
KEDPOZ	162.333	2.876	KEDPOZ	163.604	3.076	KEDPIT	157.592	3.495	KEDPOZ	151.919	2.818	KEDPOZ	167.004	2.83
KEDPOZ	170.705	3.018	KEDPUF	160.134	3.362	KEDPOZ	155.064	2.999	KEDPOZ	161.424	3.219	KEDPOZ	151.746	2.818
KEDPUF	174.833	3.228	KEDPUF	171.112	3.032	KEDPUF	171.996	3.214	KEDPUF	158.589	2.735	KEDPUF	166.244	3.189
KEDWEW	155.633	3.364	KEDWEW	164.32	3.073	KEDPUF	156.09	3.243	KEDPUF	168.975	3.137	KEDPUF	164.901	2.513
KEDWEW	152.455	2.906	KEDWEW	150.935	2.887	KEDWEW	165.592	3.073	KEDWEW	155.253	3.073	KEDWEW	163.124	3.069
KEDWIA	154.779	3.282	KEDWIA	166.957	2.942	KEDWEW	152.202	3.398	KEDWEW	161.302	2.958	KEDWIA	160.57	3.434
KEFHUZ	159.399	2.683	KEFHUZ	151.337	3.085	KEDWIA	164.936	3.184	KEDWIA	156.124	3.085	KEDWIA	151.657	3.228
KEFJIO	150.119	3.362	KEFJIO	160.015	3.01	KEFJIO	156.028	3.425	KEFJIO	158.927	3.447	KEFJIO	169.113	3.447
KEFRES	165.11	2.965	KEFRES	154.471	3.179	KEFMIV	159.066	3.102	KEFQAR	172.778	3.28	KEFRES	155.446	2.995
KEFZON	151.32	3.491	KEGBON	160.248	3.286	KEFXOI	154.076	3.17	KEFXOI	164.349	3.161	KEFXOI	166.811	3.19
KEGKIR	153.033	2.996	KEGKIR	156.883	2.947	KEGBUW	167.772	3.137	KEGGUY	165.027	3.323	KEGKEN	171.197	2.989
KEGMAK0	164.623	2.948	KEGMAK0	168.806	2.948	KEGMAK	150.053	2.846	KEGMAK	166.76	3.498	KEGMAK	153.403	3.232
KEGMEO	158.694	3.454	KEGMOY	172.961	2.757	KEGMAK0	151.105	2.968	KEGMAK0	162.499	2.706	KEGMEO	162.274	3.375
KEGMUE	156.404	2.752	KEGMUE	160.327	3.3	KEGMOY	156.638	2.852	KEGMOY	165.976	3.275	KEGMOY	150.684	3.397
KEGSEW	153.913	3.055	KEGSEW	163.383	3.347	KEGMUE	163.157	3.149	KEGSEW	161.558	2.8	KEGSEW	157.516	3.298
KEGWIF	169.168	3.351	KEGWOL	151.088	3.041	KEGSEW	168.652	3.456	KEGSOF	169.204	3.185	KEGSOH	165.623	3.287
KEGZAZ	162.218	2.823	KEGZAZ	151.587	2.733	KEGWOL	154.369	3.05	KEGZAZ	151.481	3.269	KEGZAZ	161.485	3.384
KEGZIH	162.571	3.49	KEGZIH	167.492	3.083	KEGZED	172.216	3.295	KEGZED	157.11	2.83	KEGZED	173.529	3.001
KEGZIH	158.08	3.27	KEGZIH	164.499	2.874	KEGZIH	160.555	3.083	KEGZIH	153.366	3.235	KEGZIH	158.14	3.109
KEGZUV	163.782	3.113	KEHBEG	152.094	2.86	KEGZIH	163.301	2.659	KEGZUV	168.077	2.954	KEGZUV	169.505	3.176
KEHKAM	171.283	3.485	KEHKEQ	153.917	2.864	KEHGOW	171.923	2.748	KEHGOW	159.422	2.717	KEHJUE	155.752	3.479
KEHYON	166.761	3.212	KEHZUT	155.395	3.139	KEHRAR	162.612	3.269	KEHRAR	156.78	3.397	KEHWUQ	152.457	3.463
KEJLEQ	169.405	3.268	KEJYII	169.542	3.024	KEHZUT	159.821	2.81	KEJLAO	167.059	2.535	KEJLEQ	169.719	2.856
KEJZEF	163.847	3.225	KEJZEF	169.689	3.247	KEJYII	158.92	2.669	KEJYII	160.54	3.25	KEJZEF	172.663	3.236
KEJZIJ	172.811	3.315	KEJZIJ	170.408	3.32	KEJZEF	173.639	2.905	KEJZEF	171.929	3.151	KEJZEF	154.178	3.433
KEJZOR	153.346	3.175	KEJZOR	160.278	3.014	KEJZIJ	173.95	3.012	KEJZIJ	164.49	3.397	KEJZIJ	170.783	3.289
KEKLAN	161.833	3.242	KEKNUM	163.676	3.479	KEKBAG	156.88	3.251	KEKBAG	160.81	3.255	KEKGIT	163.317	2.899
KELBUZ	175.421	3.139	KELDAI	164.553	3.483	KEKPAU	153.16	3.283	KEKPIC	151.677	3.293	KELBUZ	160.571	3.196
KELYUX	155.175	2.974	KELYUX	170.101	3.471	KELNEV	164.723	2.743	KELNEV	164.183	2.743	KELYUX	170.41	2.893
KELYUX	162.949	3.069	KELYUX	151.205	2.997	KELYUX	155.67	3.381	KELYUX	151.132	3.338	KELYUX	158.689	3.414
KELZAE	152.499	3.37	KELZAE	162.422	3.138	KELYUX	155.53	3.414	KELYUY	156.353	3.319	KELYUY	172.247	3.34
KELZAE	151.085	2.894	KELZAE	161.74	2.972	KELZAE	171.982	2.833	KELZAE	152.435	3.188	KELZAE	152.938	2.991
KELZAE	156.12	2.837	KELZAE	151.981	3.051	KELZAE	169.628	3.131	KELZAE	152.895	2.965	KELZAE	168.176	3.148
KELZAE	161.769	3.404	KELZAE	166.895	3.198	KELZAE	157.643	2.743	KELZAE	157.041	3.328	KELZAE	176.336	2.866
KEMVIJ	158.958	3.286	KEMVIJ	163.605	2.888	KELZAE	158.726	2.82	KEMDAK	157.661	3.176	KEMVAC	158.81	2.816
KEMVIJ	157.173	3.086	KEMVIJ	157.609	3.413	KEMVIJ	156.615	2.977	KEMVIJ	153.236	3.413	KEMVIJ	157.57	3.286
KENDEP	151.782	3.017	KENDEP	172.91	3.09	KEMVIJ	162.871	3.152	KENDEP	160.09	2.988	KENDEP	164.469	3.158

KENDIT	152.827	3	KENDIT	168.743	3.015	KENDEP	168.344	3.245	KENDIT	164.592	3.394	KENDIT	154.784	3
KENDOZ	154.831	2.778	KENDUF	160.006	2.694	KENDOZ	166.097	2.833	KENDOZ	161.301	2.759	KENDOZ	173.708	3.462
KENDUF	159.474	2.989	KENDUF	157.154	2.9	KENDUF	157.56	2.694	KENDUF	150.25	2.875	KENDUF	154.665	2.875
KENMOI	167.735	3.414	KENMOI	160.171	3.19	KENGER	166.487	3.476	KENJAS	151.035	2.961	KENJAS	174.706	3.161
KENMUO	150.813	3.433	KENMUO	161.216	3.447	KENMOI	153.631	3.033	KENMUO	150.822	3.099	KENMUO	152.787	3.356
KENNEZ	153.554	2.949	KENNEZ	167.484	3.27	KENMUO	162.67	3.327	KENNEZ	170.616	3.111	KENNEZ	169.192	2.934
KENNEZ	161.405	2.999	KENNEZ	159.781	3.063	KENNEZ	155.67	3.145	KENNEZ	153.893	3.188	KENNEZ	165.784	3.063
KENNEZ	169.74	3.388	KENNEZ	171.104	3.458	KENNEZ	169.548	3.404	KENNEZ	163.115	2.964	KENNEZ	150.756	3.129
KENVEF	170.767	3.365	KENVEF	161.422	3.336	KENNEZ	164.215	2.958	KENSIH	161.934	3.285	KENVEF	151.812	3.174
KENZAF	159.273	3.221	KENZAF	158.167	3.485	KENVEF	164.791	2.976	KENVEF	150.313	2.847	KENWOR	170.604	3.269
KEPPUS	162.259	3.467	KEPQIH	161.49	3.17	KEPDES	153.072	3.346	KEPDES	164.868	2.89	KEPDOB0	150.065	3.285
KEPQON	157.834	3.084	KEPQON	168.771	3.381	KEPQIH	153.886	3.431	KEPQIH	159.31	2.957	KEPQON	170.873	3.456
KEPXAI	152.637	2.88	KEQLEZ	154.346	3.354	KEPQON0	157.66	3.093	KEPQON0	171.092	3.462	KEPQON0	168.793	3.388
KEQLEZ	151.665	3.155	KEQLEZ	152.733	3.246	KEQLEZ	155.15	3.155	KEQLEZ	166.399	3.373	KEQLEZ	152.556	2.826
KEQLEZ	159.442	3.039	KEQLEZ	150.896	3.426	KEQLEZ	150.853	3.201	KEQLEZ	153.18	2.959	KEQLEZ	160.986	3.225
KEQQAA	168.084	3.104	KEQXUA	152.154	2.889	KEQLID	152.275	2.821	KEQLID	167.453	3.207	KEQNAW	161.391	3.067
KEQXUA	169.492	3.301	KEQXUA	171.331	3.431	KEQXUA	151.662	2.887	KEQXUA	151.044	3.133	KEQXUA	151.231	2.887
KERLOI	168.681	2.981	KERLOI	159.615	2.981	KEQXUA	157.312	3.069	KERJUQ	161.225	3.471	KERJUQ	171.182	2.641
KERSOQ	163.909	3.464	KERSOQ	152.175	3.005	KERQAD	164.503	2.866	KERSEH	158.455	3.32	KERSOQ	159.549	3.111
KERVEM	169.827	3.277	KERWAJ	170.128	3.334	KERSOQ	176.239	3.023	KERSOQ	166.269	2.718	KERVEM	169.471	3.277
KERWIR	168.627	3.202	KESDER	151.619	3.437	KERWAJ	172.114	3.334	KERWAJ	152.187	3.091	KERWIR	170.699	3.202
KETDIW01	154.152	2.906	KETDIW01	162.359	3.257	KESFIY	178.217	3.319	KESNUU	151.159	2.875	KESPEE	155.541	3.163
KETNEF01	150.265	3.214	KETNOP	170.156	3.069	KETFUM	160.369	3.127	KETGAT	170.277	3.127	KETGID	176.831	2.914
KETSOV	167.107	3.475	KETSOV	161.71	3.471	KETPUU	152.856	3.468	KETPUU	150.194	3.459	KETQAE	174.654	3.345
KETVOW	151.604	3.18	KETVOW	155.145	3.411	KETTAF	157.866	3.046	KETTAF	168.628	2.613	KETTAF	154.197	3.14
KETVUC	174.157	2.668	KETVUC0	158.593	2.967	KETVOW	156.26	2.744	KETVUC	158.522	2.976	KETVUC	162.475	3.284
KETWEL0	161.491	2.607	KETWEO	153.791	2.915	KETVUC01	173.543	2.664	KETVUC01	162.81	3.286	KETWEL	161.453	2.607
KETXAL	157.648	3.463	KETXAL	156.455	3.365	KETWIS	163.875	3.061	KETXAL	172.724	3.105	KETXAL	151.459	3.436
KETXEP	169.329	2.933	KEVMAC	162.466	3.138	KETXAL	170.15	3.105	KETXAL	164.308	3.387	KETXEP	171.717	2.757
KEVQOS	152.472	3.203	KEVQOT	154.938	3.266	KEVMOQ	166.217	2.931	KEVMOQ	156.219	3.206	KEVMUW	153.937	3.463
KEVQUZ	167.356	3	KEVQUZ	156.378	2.974	KEVQOT	173.654	3.155	KEVQOT	153.926	3.238	KEVQOT	169.369	2.928
KEWCAT	166.451	3.43	KEWCIA	162.942	3.479	KEVRAG	158.747	2.982	KEVRAG	167.931	2.922	KEVSAJ	176.748	2.976
KEWHEB	151.228	2.756	KEWHEB	168.438	3.205	KEWDOJ	168.698	3.107	KEWFIF	160.66	2.883	KEWGID	165.47	3.458
KEWKIK	150.351	3.48	KEWKIK	159.135	3.19	KEWHEB	172.831	3.241	KEWHOL	166.448	2.945	KEWHOL	163.46	3.158
KEWNEI	166.845	3.044	KEWNEI	152.371	2.812	KEWNAE	161.609	3.4	KEWNEI	154.752	2.812	KEWNEI	158.019	2.697
KEWSOW	167.528	3.267	KEWWIU	170.64	3.383	KEWNEI	160.706	2.697	KEWPEK	170.561	2.861	KEWRIN	176.211	3.409
KEXCAR	153.066	3.285	KEXCAR	158.954	3.285	KEWWIU	152.345	3.482	KEWWIU	159.808	3.228	KEWWUG	157.487	3.127
KEXVEQ	157.504	3.164	KEXVEQ	169.426	3.217	KEXGAW	151.668	3.07	KEXPIQ	152.946	3.32	KEXVEQ	158.655	3.307
KEXVIU	150.14	3.128	KEXVOA	152.723	3.257	KEXVEQ	159.048	3.273	KEXVEQ	161.486	3.442	KEXVIU	156.691	3.199
KEYGOK	154.906	3.424	KEYGOK	163.933	3.32	KEYGOK	156.171	2.858	KEYGOK	157.794	3.354	KEYGOK	156.852	3.489
KEYKAD	159.119	2.894	KEYSIQ	161.488	3.31	KEYKAD	163.985	2.785	KEYKAD	150.204	3.151	KEYKAD	158.918	2.785
KEZDIE	162.808	3.278	KEZPUC	151.05	3.124	KEYSIQ	166.443	3.362	KEYWAQ	156.49	3.21	KEZDIE	151.07	2.847
KHDFMA	156.626	3.31	KHDFMA0	156.194	3.252	KEZRIS	166.198	3.095	KEZSAK	174.013	3.47	KHDFMA	151.829	2.986
KIBDOQ	162.945	3.464	KIBDOQ	153.842	2.995	KHDFMA0	152.017	2.934	KHFLAC02	165.91	3.199	KIBDOQ	169.142	3.48
KIBKOZ	164.912	2.923	KIBKOZ	163.325	3.049	KIBDOQ	158.599	2.985	KIBDOQ	158.383	3.101	KIBDOQ	160.078	2.949
KIBKOZ	151.696	3.242	KIBKOZ	154.043	3.28	KIBKOZ	158.92	3.132	KIBKOZ	159.772	3.135	KIBKOZ	158.662	3.179
KIBQOC	175.24	3.262	KIBQOC	161.391	2.958	KIBKOZ	155.903	3.281	KIBQOC	166.664	3.267	KIBQOC	168.208	3.359
KIBQOC	179.383	3.038	KIBQOC	159.222	2.929	KIBQOC	173.047	3.497	KIBQOC	163.409	2.958	KIBQOC	174.157	3.171
KIBQOC01	159.664	3.011	KIBQOC01	174.184	3.239	KIBQOC01	174.653	3.163	KIBQOC01	178.341	3.099	KIBQOC01	161.493	2.993
KIBQOC01	163.035	2.993	KIBQOC01	174.572	3.189	KIBQOC01	178.131	3.173	KIBQOC01	162.462	2.993	KIBQOC01	176.637	3.236
KIBQOC01	163.258	2.993	KIBQOC01	163.728	3.011	KIBQOC01	172.963	3.208	KIBQOC01	173.557	3.31	KIBQOC01	179.583	3.059
KIBQOC01	168.935	3.246	KIBQOC01	158.459	2.943	KIBQOC01	177.811	3.046	KIBQOC01	173.347	3.356	KIBQOC01	165.688	3.305
KICCIL	173.623	3.418	KICDEH	169.313	2.936	KIBQOC01	157.328	2.947	KIBQOC01	172.011	3.157	KIBQOC01	168.134	3.386
KICDIL	173.874	3.474	KICDIL	150.189	3.371	KICDEH	160.93	3.314	KICDEH	164.423	3.461	KICDEH	165.365	2.845
KICDOR	174.01	2.804	KICDOR	155.664	2.686	KICDIL	176.438	3.06	KICDOR	152.201	2.765	KICDOR	170.947	2.756
KICMUG	151.722	3.338	KICMUG	173.023	3.217	KICDUX	173.4	3.407	KICDUX	173.449	3.362	KICFAD	162.524	3.108
KIDCIL	171.42	3.061	KIDCOR	155.986	2.549	KICZIH	172.998	3.235	KICZIH	150.018	3.343	KIDCIL	167.329	3.324
KIDJEN	164.531	3.337	KIDJEN	150.91	3.04	KIDDIL	151.031	3.429	KIDFAF	162.713	3.231	KIDJEN	155.433	2.823
KIDLIS	156.805	3.485	KIDLIS	156.59	3.226	KIDJEN	163.249	3.433	KIDJEN	158.656	2.976	KIDJEN	166.693	3.04
KIDSIC	166.103	3.005	KIDSIC	161.735	3.255	KIDREW	158.138	2.998	KIDSEY	153.919	3.451	KIDSEY	153.214	2.724
KIDTUO	153.595	3.231	KIDVOL	166.294	3.454	KIDSIC	151.28	3.202	KIDSIC	153.78	3.218	KIDTUO	163.475	2.992
KIFYAC	163.572	3.433	KIGFUF	154.356	3.016	KIFFIQ	169.187	2.92	KIFFIQ	151.274	3.312	KIFNEU	159.537	3.451
KIGFUF	164.071	2.616	KIGFUF	162.309	2.696	KIGFUF	153.666	2.696	KIGFUF	156.33	3.152	KIGFUF	155.11	2.857
KIGKOC	153.64	3.353	KIGKOC	151.448	3.076	KIGJES	165.653	3.363	KIGKOC	152.153	3.235	KIGKOC	161.806	3.377
KIGKOC	153.632	2.824	KIGKOC	152.382	3.455	KIGKOC	154.765	3.128	KIGKOC	152.484	3.205	KIGKOC	151.951	3.235
KIGKOC	156.58	3.368	KIGLIZ	154.303	3.356	KIGKOC	153.402	3.256	KIGKOC	153.77	3.2	KIGKOC	155.574	3.368
KIGQAU	165.023	3.252	KIGQAU	164.846	3.252	KIGLIZ	155.765	2.814	KIGLIZ	153.208	2.826	KIGPUL	170.849	2.932
KIHHIT	154.131	3.083	KIHHIT	154.069	3.336	KIHGEQ	152.13	2.791	KIHHIT	176.585	3.009	KIHHIT	158.734	2.994
KIHREB	172.295	3.31	KIHRUQ	167.222	2.825	KIHHIT	160.813	3.487	KIHJOB	150.213	3.086	KIHMUM	156.88	3.477
KIHVAC	169.915	3.386	KIHXUW	165.057	2.934	KIHTOO	150.4	3.201	KIHTUU	150.771	3.266	KIHTUU	153.915	3.324
KIJKIX	159.333	3.025	KIJKIX	154.231	2.889	KIJBUD	151.564	3.222	KIJCAK	152.672	3.316	KIJKIX	169.285	2.725
KIJKIX	173.825	3.287	KIJKIX	175.327	2.845	KIJKIX	178.967	3.276	KIJKIX	154.469	3.452	KIJKIX	153.54	3.371
KIJXIN	163.266	3.094	KIKSUW	153.586	3.015	KIJLOF	155.191	3.065	KIJLOF	151.991	3.478	KIJXIN	156.253	3.211
KIKXEJ01	157.957	3.428	KIKXEJ01	156.462	3.406	KIKSUW	159.08	3.187	KIKVUV	171.293	2.858	KIKXEJ01	159.002	3.391
KIKYIN	157.203	3.38	KIKYOT	163.975	3.397	KIKYEJ	152.904	2.834	KIKYIN	162.313	3.349	KIKYIN	168.111	2.982
KIKYUZ	167.932	2.993	KIKYUZ01	162.394	3.353	KIKYOT	171.858	3.009	KIKYUZ	156.788	3.418	KIKYUZ	162.887	3.379
KILHAO	167.224	3.09	KILHAO	173.225	3.23	KIKYUZ01	168.421	2.989	KIKYUZ01	157.405	3.369	KILHAO	156.129	2.913
KILHAO	169.886	2.93	KILHAO	151.359	3.023	KILHAO	153.153	3.361	KILHAO	160.743	3.363	KILHAO	160.34	3.161
KILMEX	156.509	3.329	KILMEX	164.804	3.486	KILHAO	153.008	2.896	KILHAO	159.111	3.23	KILHAO	158.543	3.188

KILPOK	159.976	3.168	KILPOK10	159.976	3.168	KILMEX	154.712	2.974	KILMEX	159.247	3.144	KILMEX	152.016	3.162
KILSAB	157.803	3.296	KIMBOB	155.702	3.02	KILSAB	154.142	2.713	KILSAB	168.907	2.901	KILSAB	155.183	3.365
KIMGAS	166.153	3.369	KIMGAS	169.808	3.133	KIMBOB	162.74	3.02	KIMFOF	156.865	2.939	KIMGAS	163.317	3.245
KIMGIA	168.39	3.069	KIMGIA	168.95	2.836	KIMGAS	167.178	3.287	KIMGEU	159.987	3.217	KIMGIA	154.15	2.715
KIMJUN	175.375	2.94	KIMMUO	151.793	3.175	KIMGIY	156.892	2.999	KIMGOG	161.837	2.945	KIMJUN	155.965	3.494
KIMWUC	160.29	3.015	KIMWUC	157.093	2.849	KIMMUO	153.139	2.824	KIMMUO	159.223	2.722	KIMQEE	168.995	3.189
KIMWUC	154.503	3.069	KIMWUC	152.508	3.391	KIMWUC	170.566	3.354	KIMWUC	156.89	3.259	KIMWUC	164.395	2.899
KIMWUC	157.304	3.226	KIMWUC	150.01	3.285	KIMWUC	161.516	3.015	KIMWUC	157.745	3.009	KIMWUC	164.325	3.432
KIMXAJ	150.524	2.858	KIMXIR	156.966	3.164	KIMWUC	152.457	3.378	KIMWUC	156.772	3.026	KIMXAJ	173.616	3.337
KIMXUD	166.194	2.954	KIMXUD	151.744	3.452	KIMXIR	152.547	2.96	KIMXIR	159.108	2.96	KIMXUD	164.059	3.43
KIMYAK	160.634	3.273	KIMZEL	163.267	3.01	KIMXUD	161.57	2.954	KIMYAK	177.635	3.161	KIMYAK	152.258	3.493
KIMZEL	150.157	2.937	KIMZEL	153.979	2.845	KIMZEL	158.449	2.852	KIMZEL	162.569	3.121	KIMZEL	161.746	2.614
KIMZEL	152.322	3.427	KIMZEL	160.091	2.971	KIMZEL	151.31	3.099	KIMZEL	156.262	2.979	KIMZEL	153.539	2.777
KINPEE	157.703	3.04	KINPEE	166.322	3.094	KIMZEL	161.872	3.159	KINPEE	158.106	3.442	KINPEE	160.971	3.369
KINQUX	156.128	3.304	KINQUX	160.471	3.143	KINQOR	155.965	3.277	KINQOR	158.992	3.137	KINQOR	158.124	3.477
KINRAE	161.584	3.143	KINRAE	157.657	3.457	KINQUX	158.031	3.47	KINRAE	155.714	3.296	KINRAE	153.725	2.742
KINTEK	150.904	2.781	KINWIN	159.55	2.731	KINSOT	158.4	3.331	KINTEK	154.013	3.416	KINTEK	175.009	2.849
KIPCOE02	156.422	3.109	KIPCOE03	157.245	3.118	KIPBAQ	157.942	2.934	KIPCOE	158.414	3.13	KIPCOE01	155.781	3.101
KIPJAX	150.055	2.966	KIPJAX	150.221	2.892	KIPCOE04	158.275	3.13	KIPGAR	151.415	3.151	KIPHAS	176.507	2.88
KIPKAX	159.694	3.074	KIPKAX	158.254	3.006	KIPJAX	151.608	3.426	KIPJAX	152.139	3.106	KIPJAX	153.452	3.041
KIQNAZ01	167.6	2.978	KIQRIO	160.396	3.473	KIPKAX	150.581	2.711	KIPKAX	154.358	3.033	KIQNAZ	167.595	2.979
KIQSUA	162.425	2.985	KIQSUA	160.08	3.405	KIQRIO	151.393	3.267	KIQROU	154.839	3.272	KIQROU	150.385	3.485
KIQTAJ01	170.169	3.282	KIQTAJ01	174.723	3.105	KIQSUA	160.406	2.985	KIQTAJ01	163.511	2.844	KIQTAJ01	171.235	3.231
KIQTAJ01	160.844	3.231	KIQTAJ01	160.881	3.105	KIQTAJ01	154.262	2.869	KIQTAJ01	160.232	3.491	KIQTAJ01	163.642	2.844
KIQWAK	152.22	3.161	KIQWAK	156.771	3.034	KIQTAJ01	168.899	3.369	KIQWAK	161.358	3.001	KIQWAK	162.174	3.172
KIQZIV	162.216	3.047	KIQZIV	152.444	3.078	KIQYAN	167.751	2.884	KIQYAN	169.787	3.154	KIQZIV	156.053	3.316
KIQZOB	152.769	3.217	KIQZOB	168.901	2.88	KIQZIV	152.575	3.316	KIQZIV	162.399	2.891	KIQZIV	163.316	2.695
KIQZOB	164.801	3.331	KIQZOB	157.818	2.793	KIQZOB	151.458	2.933	KIQZOB	163.235	2.933	KIQZOB	172.581	3.169
KIRDIA	150.835	3.157	KIRDIA	157.733	3.181	KIRDIA	153.997	3.111	KIRDIA	170.899	3.139	KIRDIA	157.869	3.495
KIRGOJ	165.671	3.053	KIRGOJ	150.247	2.737	KIRDIA	176.898	3.176	KIRDUM	152.497	2.948	KIRGOJ	165.604	3.414
KIRHEA02	152.045	3.472	KIRHEA02	167.399	2.735	KIRGUP	172.026	2.872	KIRHEA02	152.249	2.746	KIRHEA02	160.851	3.472
KIRHOK	159.243	3.4	KIRHOK	154.116	3.312	KIRHEA02	159.299	3.395	KIRHEA02	177.126	2.735	KIRHOK	150.81	3.078
KIRLEF	159.694	3.208	KIRLEF	152.746	3.479	KIRLEF	160.309	2.914	KIRLEF	166.22	3.348	KIRLEF	154.202	3.473
KIRLEF	157.432	3.164	KIRLEF	161.765	3.312	KIRLEF	175.228	2.998	KIRLEF	164.384	3.437	KIRLEF	153.152	3.212
KIRLEF	162.828	2.914	KIRNEH	154.839	3.216	KIRLEF	157.084	3.053	KIRLEF	155.053	3.425	KIRLEF	155.568	3.119
KIRNEH	151.941	3.404	KIRNIL	156.925	2.794	KIRNEH	166.538	3.24	KIRNEH	152.263	2.85	KIRNEH	153.627	2.802
KIRXAM	160.526	3.388	KIRXAM	152.181	2.855	KIRNIL	159.689	3.007	KIRNIL	152.847	3.27	KIRXAM	157.759	3.13
KISCIA	157.609	3.174	KISHIG	156.441	2.776	KIRXEP	160.16	2.76	KIRYOZ	161.25	2.723	KIRYOZ10	161.25	2.723
KISPUY	156.216	2.973	KISQEJ01	163.571	3.426	KISPUY	157.186	2.619	KISPUY	173.431	3.486	KISPUY	150.632	3.423
KISVOZ	151.464	2.922	KISVUF	151.212	3.485	KISVAK	153.464	2.906	KISVAK	151.503	3.266	KISVEP	164.497	3.433
KITBIA	152.979	3.165	KITBIC	158.189	2.833	KISWUG	155.229	3.488	KISWUG	154.22	3.177	KISYES	170.486	3.023
KITMAD	174.27	2.984	KITMAD	150.116	3.05	KITBIC	154.619	3.299	KITJAZ	152.243	3.49	KITJAZ	151.488	3.481
KITMAD	152.547	3.158	KITSOV	170.77	3.475	KITMAD	166.269	3.235	KITMAD	176.191	2.889	KITMAD	169.726	2.889
KIVJOO	153.925	3.203	KIVLAF	160.945	2.904	KITXOA	154.627	2.943	KITXUG	157.319	2.95	KITXUG	154.732	3.102
KIVLAF	152.037	3.081	KIVRIT01	151.68	2.991	KIVLAF	162.286	3.1	KIVLAF	173.391	2.904	KIVLAF	166.516	3.458
KIVRIT01	154.551	3.324	KIVSEQ	156.286	2.924	KIVRIT01	150.937	3.073	KIVRIT01	156.92	3.076	KIVRIT01	162.749	2.975
KIVWAP	165.272	3.42	KIWJEI	158.179	2.982	KIVSEQ	168.415	2.795	KIVSEQ	163.466	2.795	KIVWAP	152.565	2.831
KIWJIM	175.103	3.203	KIWJIM	153.658	3.136	KIWJIM	154.781	3.115	KIWJIM	169.858	2.887	KIWJIM	154.287	3.112
KIWJIM	154.456	3.121	KIWJUY	151.499	3.259	KIWJIM	150.064	2.868	KIWJIM	165.537	3.424	KIWJIM	151.41	3.035
KIWQEN	150.626	3.377	KIWQEN	152.831	3.068	KIWPIP	153.729	3.394	KIWQEN	168.472	2.704	KIWQEN	174.485	2.725
KIXFOO	161.272	2.975	KIXFOO	155.709	3.012	KIWQEN	160.192	3.123	KIXBOI	168.829	3.023	KIXFOO	154.641	3.207
KIYDUV	153.582	3.124	KIYGAA	154.774	3.121	KIXQIR	169.957	3.306	KIYDUV	153.278	3.352	KIYDUV	153.639	2.81
KIYLAG	161.428	2.857	KIYMUB	150.469	3.139	KIYGAA	159.019	3.048	KIYGAA	162.767	3.146	KIYHUW	160.367	3.278
KIYQAN	170.262	3.285	KIYQAN	155.422	3.463	KIYPIR	156.098	2.821	KIYPIR	165.209	3.185	KIYQAN	150.601	3.302
KIYXIZ	162.469	3.391	KIYXIZ	159.637	3.246	KIYQAN	153.472	2.808	KIYQAN	156.217	2.97	KIYRIX	151.603	3.462
KIZDUW	150.125	2.837	KIZDUW	158.892	3.073	KIZBAX	162.684	3.304	KIZBAX	164.746	3.373	KIZBAX	168.32	3.142
KIZDUW	161.591	2.929	KIZDUW	168.775	3.123	KIZDUW	156.673	3.075	KIZDUW	156.607	3.162	KIZDUW	163.12	3.472
KIZGAB	168.427	3.445	KIZGAB	151.89	3.081	KIZDUW	156.37	2.648	KIZDUW	157.742	2.648	KIZGAB	172.717	2.999
KIZHAE	159.754	3.274	KIZHAE	162.593	2.917	KIZGAB	169.219	3.3	KIZGAB	168.326	3.135	KIZGOR	160.928	3.415
KIZLOV	177.511	3.005	KIZLOV	174.089	3.475	KIZLOV	162.804	3.084	KIZLOV	151.426	3.084	KIZLOV	151.244	2.957
KOBGOZ	172.036	3.093	KOBGOZ	154.724	3.122	KIZLOV	158.707	2.691	KIZRIU	161.704	3.444	KIZZEZ	160.317	3.49
KOBYUW	156.092	2.828	KOBZAD	171.598	3.319	KOBGOZ	174.752	2.683	KOBYUW	160.462	3.447	KOBYUW	161.612	3.21
KOCCOV	173.26	3.4	KOCCOV	150.019	3.067	KOBZAD	163.835	3.207	KOBZOT0	167.057	3.26	KOCCOV	154.551	2.878
KOCDAI	164.603	3.033	KOCDAI	161.231	3.44	KOCCOV	155.042	3.304	KOCCUB	151.772	2.916	KOCCUB	155.72	3.218
KOCPIB	152.425	2.958	KOCYOU	179.042	2.977	KOCDAI	166.309	2.816	KOCDAI	162.369	2.96	KOCDAI	173.892	2.772
KODREA	158.16	3.163	KODREA	156.644	3.432	KOCYOU	151.683	2.958	KODREA	170.42	3.228	KODREA	153.937	2.965
KODSIJ	167.177	3.1	KODSIJ	153.166	3.063	KODREA	164.919	3.33	KODSIJ	161.103	3.185	KODSIJ	152.064	3.175
KODSIJ	157.878	3.183	KODTON	160.579	3.023	KODSIJ	160.614	3.219	KODSIJ	168.946	3.029	KODSIJ	158.158	2.892
KOGHIZ	162.072	2.939	KOGHIZ	165.275	2.659	KOFFER	155.662	3.473	KOGHIZ	158.632	2.776	KOGHIZ	175.166	3.467
KOGHIZ	155.691	3.212	KOGHIZ	171.027	3.398	KOGHIZ	151.999	2.659	KOGHIZ	166.484	3.071	KOGHIZ	166.389	3.474
KOGLEZ	154.85	3.434	KOGLEZ	151.185	2.965	KOGLEZ	151.884	2.814	KOGLEZ	168.039	2.691	KOGLEZ	160.759	2.781
KOHCUI	157.024	3.31	KOHCUI	155.617	3.263	KOGNIH	159.757	3.133	KOGNIH	154.121	3.005	KOHCUI	164.46	3.213
KOHNOK	164.238	3.038	KOHNOK	159.719	3.038	KOHCUI01	158.566	3.168	KOHCUI01	178.709	3.055	KOHDER	159.406	3.332
KOJCIY	165.916	3.071	KOJPAE	157.461	3.17	KOHROP	168.172	3.468	KOHROP	164.388	2.541	KOHVAI	162.29	3.101
KOKDUJ	153.107	2.737	KOKKEC	156.353	3.004	KOJXIQ	167.889	3.288	KOJXIQ	165.324	2.903	KOJZUH	163.649	3.118
KOKWUG	157.921	3.171	KOLWUG	164.636	2.668	KOKKUQ	155.018	2.669	KOKNOQ	151.898	3.394	KOKWOA	152.44	3.152
KOLXAM	162.812	2.907	KOLXAM	162.044	2.907	KOLWUG	163.274	3.343	KOLXAM	166.292	3.214	KOLXAM	165.086	3.29
KOLXER	161.098	3.491	KOLXUH	174.19	2.718	KOLXEQ	154.643	3.17	KOLXER	175.652	2.802	KOLXER	167.528	2.596
KOLXUH	166.912	2.759	KOLXUH	152.691	3.488	KOLXUH	165.89	2.832	KOLXUH	156.482	2.832	KOLXUH	166.885	3.05

KOMNAF	154.323	3.423	KOMNAF	161.612	2.919	KOLXUH	164.945	3.027	KOMMIM	161.888	3.188	KOMMIM	154.748	3.188
KOMSAJ	155.83	2.988	KOMSAJ	169.896	3.058	KOMROW	152.341	3.152	KOMROW	166.217	2.87	KOMROW	163.496	3.034
KOMWIS	168.753	3.041	KOMWIS0	172.073	3.49	KOMWIS	175.19	2.944	KOMWIS	151.403	3.116	KOMWIS	155.381	2.867
KOMWIS0	169.474	2.815	KOMZIX	167.113	2.782	KOMWIS0	162.988	2.959	KOMWIS0	158.205	3.063	KOMWIS0	153.683	3.301
KONZOE	157.341	3.184	KONZOE	171.199	2.983	KOMZIZ	159.702	3.29	KONJOM	154.894	3.27	KONKED	153.171	3.429
KOPBAW	173.235	3.362	KOPBAW	161.955	3.424	KOPBAU	162.759	3.283	KOPBAW	178.163	3.207	KOPBAW	165.059	2.77
KOPJAC	168.219	3.141	KOPREM	172.942	3.137	KOPBOK	153.115	3.075	KOPFED	170.61	2.93	KOPFED	173.65	2.624
KORPIT	162.466	2.806	KORWOF	166.208	3.27	KOQJOS	170.071	3.468	KORPIT	150.305	2.872	KORPIT	172.702	3.499
KOTHIK	170.392	3.24	KOTHIK	161.04	3.078	KORYUP	177.273	2.963	KOSMIO	164.752	2.927	KOTHEG	170.786	3.385
KOTTAR0	155.124	2.898	KOTTAR0	155.338	2.9	KOTHIK	150.04	3.456	KOTHIK	174.47	3.423	KOTTAR0	152.907	2.9
KOTTIZ	165.276	3.125	KOTTIZ	155.101	2.898	KOTTAR0	165.883	3.149	KOTTAR0	167.46	3.208	KOTTAR0	154.473	3.13
KOTTIZ	154.091	2.867	KOTTUL	161.968	3.496	KOTTIZ	152.91	2.867	KOTTIZ	154.398	3.136	KOTTIZ	167.85	3.161
KOVBAB	154.183	2.885	KOVCAZ	164.316	2.925	KOTTUL	158.561	3.054	KOTTUL	163.934	3.289	KOTTUL	152.068	3.274
KOVDOS	152.226	3.237	KOVDOS	162.291	2.879	KOVCUX	162.12	3.252	KOVCUX	158.044	2.904	KOVDOS	161.612	3.142
KOVZON	171.198	2.879	KOVZON	162.853	3.476	KOVDUY	152.266	2.941	KOVMAJ	167.28	3.476	KOVRUL	157.092	2.854
KOVZON	151.671	3.484	KOVZON	155.959	3.004	KOVZON	156.227	3.071	KOVZON	151.346	3.152	KOVZON	172.728	3.265
KOVZON	161.492	3.469	KOWMOB	158.143	2.934	KOVZON	152.337	3.294	KOVZON	150.467	3.293	KOVZON	150.198	3.283
KOWMOB	150.498	3.374	KOWMOB	153.564	2.961	KOWMOB	161.109	2.934	KOWMOB	162.308	3.033	KOWMOB	160.8	3.161
KOWMOB	154.358	3.183	KOWTIB	154.317	3.353	KOWMOB	160.27	3.086	KOWMOB	159.47	3.414	KOWMOB	150.119	2.773
KOXJUF	173.888	2.948	KOXJUF	158.341	2.993	KOWXIF	160.949	3.48	KOWXIF0	160.38	3.445	KOXJUF	151.391	2.948
KOXNIX	161.232	2.914	KOXNIX	154.413	2.914	KOXMIX	170.398	2.936	KOXMUJ	154.573	2.914	KOXMUJ	167.393	2.982
KOXTIE	169.976	3.489	KOXTIE	154.365	3.206	KOXNOE	150.004	3.124	KOXTAW	154.487	3.343	KOXTEA	156.067	3.232
KOXVEY	162.32	3.453	KOXVEY	161.751	3.058	KOXTUP	158.152	3.306	KOXVEY	171.043	3.351	KOXVEY	151.949	3.316
KOXVEY	155.098	3.492	KOXVEY	163.055	3.467	KOXVEY	164.593	3.43	KOXVEY	157.26	3.299	KOXVEY	161.91	3.02
KOXVIC	166.643	2.815	KOXVIC	166.304	3.34	KOXVEY	166.637	3.395	KOXVEY	150.309	3.15	KOXVEY	154.861	3.213
KOXVIC	163.662	2.887	KOXVIC	156.006	3.27	KOXVIC	167.85	3.142	KOXVIC	154.814	3.207	KOXVIC	151.302	2.982
KOXVIC	158.307	3.363	KOXVIC	163.385	3.206	KOXVIC	157.014	2.988	KOXVIC	154.153	3.055	KOXVIC	166.372	3.164
KOXWUS	170.825	2.894	KOXXAZ	161.352	2.913	KOXVIC	151.105	3.115	KOXVIC	167.336	3.017	KOXWUS	168.44	2.903
KOYGIO	166.05	3.327	KOYGIO	163.32	2.964	KOXXAZ	170.155	2.884	KOYBEH	156.921	2.795	KOYDOV	158.92	3.49
KOYHIT	152.242	3.41	KOYHUF	162.539	2.779	KOYGIO	152.663	3.146	KOYGIO	168.71	3.5	KOYHIT	162.636	2.785
KOYJEN	157.262	2.911	KOYJOB	162.613	2.777	KOYHUF	152.78	3.395	KOYJEN	152.884	3.364	KOYJEN	158.715	3.446
KOYKAN	161.111	3.101	KOYKER	174.4	2.731	KOYJOB	152.012	3.393	KOYKAN	155.429	3.211	KOYKAN	150.609	3.126
KOYKIV	155.838	2.97	KOYKIV	157.191	3.285	KOYKIV	158.39	3.363	KOYKIV	156.055	3.285	KOYKIV	159.232	3.363
KOZHAL	169.559	2.843	KOZHEP	151.47	3.299	KOYYEF	160.764	3.108	KOYYOP	159.932	3.268	KOYYOP	151.89	3.243
KOZQAT	153.529	3.213	KOZQAT	164.125	3.368	KOZHEP	170.682	2.806	KOZHIT	169.704	2.866	KOZLOE	156.636	3.227
KTFPHT0	159.185	2.71	KUBHEX	156.403	2.792	KOZQAT	168.86	2.746	KOZQAV	153.018	2.926	KOZQAV	150.954	3.497
KUBRIK	161.814	3.386	KUBRIK	158.74	3.141	KUBHEX	164.334	2.467	KUBPOO	173.066	3.056	KUBREG	155.344	2.972
KUBXAJ	168.634	2.677	KUCCES	160.29	2.824	KUBRIK	157.346	3.063	KUBVEJ	155.208	3.183	KUBVIN	171.54	3.094
KUCCIW	164.927	3.225	KUCCOC	170.663	2.872	KUCCES	162.404	2.699	KUCCES	163.927	2.699	KUCCIW	160.189	2.656
KUCGAR	160.691	2.699	KUCGAR	175.526	3.029	KUCCOC	152.962	3.262	KUCCUI	151.59	2.884	KUCFEW	160.648	3.471
KUCNAY	157.016	3.003	KUCNAY	156.41	3.157	KUCGUL	167.311	3.432	KUCGUL	162.535	3.083	KUCGUL	157.106	3.258
KUDJUO	154.871	3.047	KUDLEB	168.435	2.99	KUCNAY	153.658	3.209	KUCNIG	153.561	3.277	KUDJIG	159.063	3.49
KUDYEO	154.232	2.96	KUDYEO	162.945	3.219	KUDLEB	156.523	3.132	KUDTUA	154.274	3.384	KUDYEO	150.568	3.219
KUDZUH	151.091	3.29	KUFHID	151.866	3.068	KUDYEO	167.238	3.023	KUDYIS	171.005	3.385	KUDZER	167.52	2.832
KUFHID	172.515	2.914	KUFHID	153.712	2.687	KUFHID	170.515	2.975	KUFHID	163.825	3.181	KUFHID	166.122	3.439
KUFXIT	150.748	3.082	KUGCUN	151.449	2.897	KUFRUY	173.059	3.058	KUFXAN	163.179	3.479	KUFXAN	155.674	3.183
KUGGIG	164.412	3.232	KUGGIG	163.355	3	KUGDAV0	156.653	3.353	KUGGIG	164.038	3.397	KUGGIG	150.583	3
KUGHUT	171.858	3.282	KUGHUT	178.121	3.259	KUGGIG	172.919	3.012	KUGHON	161.539	2.737	KUGHON	171.253	2.737
KUGJAY	158.438	2.694	KUGJAY	154.974	3.412	KUGJAB	178.271	3.353	KUGJAB	150.52	3.436	KUGJAY	160.747	2.752
KUGJEC	166.041	3.36	KUGJEC	159.386	2.826	KUGJAY	162.175	2.931	KUGJAY	163.212	2.694	KUGJEC	150.398	3.36
KUGTAL	154.48	3.04	KUGWUE	168.313	3.24	KUGJEC	162.05	2.763	KUGJEF	175.19	3.462	KUGMOO	162.869	3.079
KUHJII	174.976	2.856	KUHJII	171.429	2.833	KUHCAV	161.164	2.854	KUHJII	176.599	2.84	KUHJII	171.268	2.84
KUHJII	167.452	3.038	KUHZAR	161.43	2.951	KUHJII	164.727	2.958	KUHJII	169.171	3.066	KUHJII	167.688	2.966
KULBOL	157.531	3.198	KULBUS	150.352	2.863	KUJSOX	151.98	3.19	KUKHIL	167.818	2.913	KULBOL	160.572	3.198
KULCAZ	161.251	2.907	KULCAZ	160.644	2.981	KULBUS	150.511	3.153	KULBUS	155.833	3.122	KULCAZ	174.937	2.95
KULCED	161.539	2.587	KULCED	150.447	2.877	KULCAZ	154.975	3.023	KULCAZ	154.007	3.023	KULCED	154.635	3.074
KULHIK	166.068	2.923	KULQAL	153.672	3.443	KULCUT	158.028	3.144	KULCUT	167.324	2.997	KULFUU	162.191	3.009
KULVOE	163.956	3.137	KUMKIM	168.732	3.223	KULVIY	150.902	2.698	KULVIY	153.331	3.209	KULVOE	151.617	3.071
KUNBIE	152.724	3.202	KUNGEJ	174.932	3.457	KUMMAI	164.188	3.158	KUMXIB	167.221	2.944	KUMXOF	150.038	2.923
KUNLAK	153.11	3.325	KUNLAK	152.322	3.369	KUNKEL	161.811	2.855	KUNKEL	157.71	3.332	KUNKEL	154.848	3.445
KUNLEM	153.89	3.078	KUNSOD	167.421	3.211	KUNLEM	175.696	3.121	KUNLEM	157.442	3.063	KUNLEM	163.142	3.121
KUNYEA	150.494	3.198	KUNYUQ	154.449	2.896	KUNSOD	157.478	3.07	KUNYEA	150.757	2.907	KUNYEA	158.076	2.749
KUPKIQ	166.302	3.307	KUPKIQ	162.721	3.453	KUNYUQ	153.982	2.896	KUNZAX	151.137	3.474	KUPBAB	157.734	3.378
KUPYON	155.138	3.118	KUPYON	151.556	3.118	KUPKOW	168.119	3.361	KUPKOW	162.201	3.497	KUPXUQ	151.875	3.474
KUPYUT	154.777	3.452	KUPZUS	156.323	2.884	KUPYUP	153.844	3.206	KUPYUP	151.568	3.101	KUPYUT	154.94	3.487
KUQHIR	151.356	3.075	KUQMAL	151.689	3.456	KUQDOT	162.673	3.433	KUQDUX	160.053	3.149	KUQDUX	157.59	3.079
KUQSUL	151.108	3.112	KUQYOL	152.262	3.23	KUQNET	162.088	2.987	KUQRIB	150.82	3.325	KUQSOF	161.478	3.426
KURDEJ	150.963	3.269	KURDEJ	155.054	3.115	KURBEG	170.505	3.147	KURBEG	150.405	3.012	KURDAD	150.141	3.067
KURVAY	166.821	2.918	KURVAY	155.342	3.249	KURDEJ	165.561	2.846	KURGAF	151.826	3.239	KURPIW	153.828	3.069
KURVAY	160.047	2.898	KURVAY	158.413	3.409	KURVAY	162.96	3.058	KURVAY	155.529	3.144	KURVAY	152.435	3.231
KURVAY	150.618	3.477	KURVAY	152.912	2.693	KURVAY	158.634	3.22	KURVAY	167.815	3.227	KURVAY	169.998	2.93
KUSGEL	157.164	3.079	KUSGEL	175.514	3.487	KURVAY	152.803	3.313	KURVAY	150.317	3.386	KURYUV	171.472	3.348
KUSJIT	150.548	3.283	KUSJIT	156.471	3.394	KUSGIR	173.925	3.421	KUSHUD	150.903	3.232	KUSJAL	160.712	3.45
KUSNET	172.512	3.108	KUSPEX	154.942	2.919	KUSJOZ	174.732	3.204	KUSNET	163.112	3.463	KUSNET	175.948	3.018
KUSVAW0	150.191	3.291	KUSVAW0	150.188	3.291	KUSQIA	156.681	3.172	KUSRIC	150.735	3.33	KUSSOG	151.113	3.31
KUTCAE	161.734	3.284	KUTGIS	166.309	3.006	KUSVON	161.534	3.206	KUSZUT0	177.903	3.213	KUSZUT0	152.39	3.467
KUTKUF	157.178	2.9	KUTKUF	169.565	3.422	KUTGIS	155.094	3.216	KUTGIS01	165.995	2.969	KUTGIS01	155.324	3.183
KUTLIU	173.786	2.783	KUTTAV	151.539	3.428	KUTLIU	150.531	3.472	KUTLIU	170.213	3.407	KUTLIU	169.417	3.227
KUVKEV	163.623	2.731	KUVQAW	160.707	2.975	KUVKEV	158.522	2.731	KUVKEV	150.583	2.776	KUVKEV	165.833	2.873

KUWBUB	163.339	3.4	KUWFAM	162.544	3.314	KUVQAW	158.801	3.212	KUVVAC	159.458	3.252	KUVVAC	154.555	3.251
KUWFAM	162.193	3.119	KUWFAM	155.305	3.236	KUWFAM	156.744	3.045	KUWFAM	164.898	3.19	KUWFAM	159.998	3.042
KUWFAM	158.034	3.494	KUWGIW	173.901	2.949	KUWFAM	153.09	3.261	KUWFAM	162.693	3.421	KUWFAM	156.914	3.497
KUWJUK	153.253	2.926	KUWKAR	152.994	2.931	KUWGIW	157.101	2.878	KUWGIW	154.037	3.439	KUWJOE	153.387	2.913
KUWKUL	153.454	2.942	KUWLEW	153.159	2.951	KUWKEV	153.23	2.931	KUWKIZ	153.635	2.908	KUWKOF	153.411	2.94
KUWPEZ	171.359	3.303	KUWPEZ	164.738	2.653	KUWLIA	153.104	2.953	KUWLOG	152.963	2.954	KUWLUM	152.461	2.955
KUWREB	155.869	2.856	KUWRIH	152.007	3.425	KUWQOK	150.648	3.258	KUWRAX	158.616	3.44	KUWREB	157.572	2.856
KUWXUX	159.823	3.263	KUWXUX	159.519	3.232	KUWWOC	166.365	2.726	KUWWOC	167.406	2.882	KUWWOC	167.035	3.414
KUWXUX	174.554	2.888	KUWXUX	173.186	2.821	KUWXUX	158.288	3.435	KUWXUX	162.128	2.9	KUWXUX	157.737	2.96
KUWZAG	168.99	2.735	KUWZEK	155.008	3.191	KUWZAG	155.493	3.222	KUWZAG	156.241	2.749	KUWZAG	159.35	3.434
KUXJUK	151.784	3.104	KUXJUK	154.363	2.972	KUXHAP	165.43	3.246	KUXJUK	166.481	3.4	KUXJUK	163.885	2.932
KUXNAU	159.389	2.93	KUXNAU	158.007	2.723	KUXJUK	150.511	3.381	KUXNAU	162.983	3.058	KUXNAU	166.139	2.723
KUXQUT	167.715	2.857	KUXWEF	167.823	2.69	KUXNAU	153.356	2.984	KUXNAU	151.022	3.139	KUXQUT	168.692	2.76
KUYFAP	160.228	3.431	KUYFAP	160.264	3.376	KUYFAL	157.391	2.794	KUYFAL	156.736	3.199	KUYFAL	158.103	3.199
KUYNAW	168.452	3.28	KUYREF	155.8	2.983	KUYGAO	162.225	3.159	KUYNAW	155.872	3.365	KUYNAW	157.064	3.392
KUYVOQ	176.589	2.946	KUYYIO	156.474	3.276	KUYSEF	153.536	3.344	KUYTAD	173.364	3.419	KUYTAD	164.943	2.884
KUZGEV	153.755	3.142	KUZGEV	157.524	2.933	KUYYIO	157.785	3.371	KUZGEV	175.793	2.933	KUZGEV	155.351	2.971
KUZRUV	157.714	2.955	KUZRUV	162.09	2.955	KUZGUL	158.703	3.145	KUZGUL	168.101	3.137	KUZJEX	151.139	2.991
KUZWAF	168.58	3.153	KUZWAF0	155.189	3.418	KUZRUV	150.911	3.186	KUZRUV	160.883	2.991	KUZRUV	150.043	3.243
LABBEY	161.885	2.643	LABDUR	150.909	3.257	KUZWAF0	174.73	3.055	LABBEY	162.36	2.643	LABBEY	171.496	3.456
LABDUR	172.455	3.109	LABFIF	164.087	2.905	LABDUR	173.656	3.086	LABDUR	167.028	2.969	LABDUR	168.058	2.853
LABRIU	160.357	3.436	LABRUG	151.008	2.967	LABFIF	160.909	3.036	LABJAE	158.449	2.957	LABRIU	151.738	3.017
LABYEX	165.809	3.191	LABYIB	157.408	3.083	LABRUG	166.009	2.92	LABRUG	150.33	2.974	LABYEX	164.659	3.491
LACCAW	161.177	3.2	LACCAW	150.085	3.312	LABYIB	164.968	3.023	LABYIB	168.69	3.163	LACCAW	158.468	2.988
LACCOK	174.507	3.267	LACCOK	164.038	3.287	LACCAW	158.655	3.344	LACCAW	162.509	3.312	LACCOK	164.115	2.916
LACQIT	150.676	2.938	LACQIT	168.289	3.411	LACFUU	150.42	3.133	LACFUU	152.564	3.133	LACGAB	154.344	3.087
LACRIU	151.87	2.85	LACRIU	164.621	3.222	LACRIU	162.075	3.43	LACRIU	167.14	3.222	LACRIU	155.624	3.244
LACWER	161.48	3.006	LACWIV	163.205	3.194	LACRIU	167.285	3.218	LACWER	152.706	2.884	LACWER	173.233	2.884
LADGIK	150.537	3.123	LADHOO	163.338	3.338	LACWOD	150.926	3.221	LACWOD	156.315	3.177	LACZOF	154.11	3.215
LADTAM	166.382	3.343	LADTAM	151.968	3.173	LADHOO	170.886	3.436	LADJAC	155.821	3.176	LADTAM	153.805	3.095
LADTAM	157.583	3.223	LADTAM	158.598	2.971	LADTAM	162.82	3.167	LADTAM	155.17	2.703	LADTAM	154.207	3.452
LADTEQ	166.397	3.091	LADVEV	164.083	3.38	LADTAM	164.587	2.87	LADTAM	164.575	2.849	LADTEQ	151.462	3.274
LADZAR	157.169	3.308	LADZAR	169.075	3.189	LADWEW	174.576	2.696	LADZAR	173.176	3.189	LADZAR	162.482	2.866
LADZAR	167.677	3.398	LADZAR	153.227	3.346	LADZAR	162.755	2.965	LADZAR	160.845	3.227	LADZAR	150.514	3.308
LAFVUO	167.419	3.149	LAFVUO	174.428	2.969	LAFMUC	151.377	3.359	LAFNEP	161.955	3.097	LAFTIA	167.29	2.886
LAFZIC	159.82	2.888	LAGFOQ	171.034	2.746	LAFWAQ	162.697	3.445	LAFXOJ	158.786	3.097	LAFXOJ	166.337	2.925
LAGGOT	152.079	3.488	LAGGOT	155.865	2.963	LAGFUW	155.061	3.086	LAGGAD	167.625	2.872	LAGGAD	165.917	2.883
LAGJEK02	150.969	3.231	LAGJEK02	161.007	3.349	LAGHUA	156.48	3.49	LAGHUA	171.007	2.883	LAGJEK02	150.343	3.458
LAGKUD0	168.478	2.912	LAGKUD0	168.421	2.913	LAGJEK02	163.057	3.343	LAGJEK02	155.967	2.831	LAGKUD	168.712	2.909
LAGVEY	154.204	3.433	LAHBOM	173.002	2.936	LAGKUD0	167.922	2.824	LAGNEQ	157.407	2.866	LAGNEQ	158.953	2.883
LAHFUZ	157.524	3.14	LAHFUZ	162.984	3.155	LAHBOM	150.396	3.335	LAHCAZ	161.265	2.961	LAHFEJ01	160.42	3.437
LAHPIV	166.778	3.033	LAHQET	156.86	3.399	LAHFUZ	170.881	3.323	LAHFUZ	166.658	2.891	LAHPIV	163.581	3.186
LAHXUO	160.109	3.032	LAHXUO	157.351	3.325	LAHQET	162.341	3.308	LAHQET	154.966	3.479	LAHQET	154.954	3.323
LAHYAV	156.167	2.898	LAHYAV	176.761	2.913	LAHXUO	167.635	2.848	LAHXUO	157.167	2.784	LAHXUO	150.049	2.973
LAJFAI	177.324	2.9	LAJLAM	169.738	2.905	LAHYAV	173.385	3.332	LAJFAI	155.814	3.092	LAJFAI	160.654	3.236
LAJMIV	168.214	2.916	LAJMIV	152.7	3.021	LAJLAM	171.217	2.889	LAJLAM	153.086	2.986	LAJMIV	167.015	2.933
LAJWON	154.717	2.949	LAJWON	165.952	2.983	LAJPET	154.148	3.462	LAJTIA	165.756	3.439	LAJTIA	160.643	2.799
LAKBEJ	161.437	3.396	LAKBEJ	167.155	3.041	LAJXAA	162.418	3.249	LAJZUW	159.993	3.219	LAKBEJ	156.198	2.659
LAKBIN	153.729	3.156	LAKHAG	158.752	3.098	LAKBEJ	153.096	2.814	LAKBEJ	169.031	2.865	LAKBIN	165.53	3.156
LAKHUF	173.477	2.985	LAKJIV	152.285	3.251	LAKHEP	151.379	2.967	LAKHOU	151.876	3.212	LAKHUF	169.56	3.276
LAKKAO	171.72	3.101	LAKKUD	162.538	3.066	LAKJIV	158.671	3.148	LAKKAO	162.096	3.442	LAKKAO	167.528	2.733
LAKMIY	176.008	2.835	LAKNUI	165.242	2.899	LAKKUD	155.599	3.29	LAKKUD	170.519	3.035	LAKMIY	160.382	3.284
LAKREZ	161.772	3.163	LAKRUL	164.251	3.066	LAKNUI	151.067	2.993	LAKPAQ	159.848	3.472	LAKPOG	158.632	3.233
LAKZII	161.645	3.095	LALHUC	156.167	3.126	LAKTEB	153.62	3.314	LAKWON	169.162	2.949	LAKXOK	168.687	3.091
LALMIY	169.099	3.28	LALROF	157.522	3.177	LALHUC	153.972	2.859	LALHUC	158.548	3.01	LALHUC	163.282	3.208
LALROF	150.671	2.729	LALROF	155.319	3.003	LALROF	165.076	2.901	LALROF	151.837	3.132	LALROF	157.124	2.832
LALYED	154.286	3.129	LAMCIN	153.197	3.495	LALROF	169.364	2.707	LALROF	156.198	2.846	LALXUS	151.818	2.788
LAMRAW	167.304	3.009	LAMRAW	150.247	3.034	LAMNOF	150.871	3.493	LAMRAW	161.088	3.472	LAMRAW	160.612	3.36
LANFOV	153.001	3.014	LANFUB	169.058	3.458	LAMRAW	165.02	2.665	LAMSAX	157.235	3.02	LANFOV	151.477	3.014
LANJOC	169.373	3.365	LANLET	152.011	3.463	LANJAN	176.101	3.175	LANJES	160.267	3.2	LANJES	169.153	3.079
LANQEX	163.752	2.495	LANQEX	173.938	2.495	LANLET02	166.082	2.805	LANLET04	154.392	2.695	LANLET04	165.297	3.133
LANSEZ	160.45	3.168	LANSUP	155.3	3.474	LANROL	155.985	3.461	LANRUP	162.206	3.063	LANSEZ	156.771	3.151
LANSUP	154.238	2.997	LANSUP	161.557	3.057	LANSUP	158.388	3.213	LANSUP	156.601	3.384	LANSUP	158.354	3.384
LANSUP	164.498	3.339	LANSUQ	153.424	3.31	LANSUP	156.672	3.114	LANSUP	153.755	2.883	LANSUP	156.852	2.888
LAPKIW	163.106	3.263	LAPKIW	178.243	3.269	LAPFAM	155.672	3.073	LAPJOF	171.879	2.763	LAPJOF	171.334	2.599
LAPPUR	159.051	2.807	LAPPUR	170.536	3.191	LAPKIW	155.299	3.462	LAPPOL	155.379	3.152	LAPPOL	167.239	2.975
LAPSUT	151.449	3.398	LAPSUT	170.769	3.398	LAPPUR	158.356	3.34	LAPSEB	153.819	3.119	LAPSUT	169.249	3.398
LAPTEE	155.995	3.133	LAPTUT	161.883	3.173	LAPTEE	152.194	3.472	LAPTEE	162.943	3.101	LAPTEE	161.811	3.406
LAPYOQ	164.893	2.963	LAPYOQ	159.156	3.164	LAPVII	168.42	2.777	LAPVII	164.468	2.777	LAPXAD	158.682	3.489
LAPYOQ	151.718	3.158	LAQBUB	174.028	2.975	LAPYOQ	156.658	2.92	LAPYOQ	157.542	2.963	LAPYOQ	173.617	2.902
LAQFIV	153.074	2.971	LAQFIV	159.468	3.348	LAQFIV	153.718	3.045	LAQFIV	172.643	3.191	LAQFIV	169.773	3.434
LAQQEA	150.227	3.179	LAQTOM	170.111	3.25	LAQFIV	167.02	3.008	LAQFIV	169.14	3.017	LAQKEV	150.556	3.025
LAQTOM	152.391	3.356	LAQTOM	166.898	3.392	LAQTOM	169.383	2.871	LAQTOM	168.529	2.978	LAQTOM	176.856	3.356
LAQWOS	168.892	3.351	LAQWOS	153.003	3.114	LAQWOS	167.526	2.987	LAQWOS	151.742	3.184	LAQWOS	154.073	3.275
LAQWOS	165.604	3.275	LAQWOS	162.868	3.193	LAQWOS	171.557	3.147	LAQWOS	173.649	2.962	LAQWOS	160.869	3.16
LARHES	171.169	2.961	LARHES	164.28	3.333	LAQWOS	151.395	3.222	LAQWOS	154.2	2.922	LARHES	169.179	3.045
LARTAA	161.109	3.132	LARTAA	152.525	2.982	LARQON	150.534	3.26	LARSON	151.072	3.493	LARSON	157.633	2.708
LARTAA	154.326	3.077	LARYEM	175.377	3.392	LARTAA	153.459	3.466	LARTAA	151.751	3.314	LARTAA	166.4	2.838
LASHIY	169.91	3.125	LASJAS	155.123	3.263	LASBIU	171.224	3.4	LASBIU	170.988	3.121	LASBIU	150.113	3.042

LASVEJ	156.531	2.91	LASVUZ	150.073	3.186	LASJAS	154.068	3.347	LASRAZ	157.628	3.223	LASRAZ	154.013	3.084
LATBEO	171.803	3.331	LATBEO	167.627	3.21	LASYEK	163.473	2.719	LASYEK	164.401	2.719	LATBAK	170.813	3.101
LATLEA	161.492	3.221	LATLIE	160.162	2.941	LATBEO	150.905	3.453	LATBEO	154.642	3.47	LATBEO	157.72	3.365
LATSEE	163.57	2.925	LATSEE	157.544	2.877	LATMUP	151.797	3.398	LATMUP	152.248	2.888	LATSEE	153.137	3.413
LATSEE	160.463	2.759	LATSEE	155.571	3.084	LATSEE	152.333	3.015	LATSEE	152.228	2.978	LATSEE	164.867	2.759
LAVKUP	168.335	3.471	LAVLOK	161.695	2.865	LATSEE	164.643	2.639	LAVBAL	173.703	3.04	LAVJUR	163.411	3.365
LAVNEC	156.16	3.366	LAVRIN	170.392	3.079	LAVLOK	163.042	2.975	LAVNAZ	153.931	3.168	LAVNAZ	158.326	3.168
LAWBIU	168.537	3.014	LAWBIU	164.245	3.178	LAVRIN	171.379	3.48	LAVRIN	177.197	3.407	LAVRIN	150.395	3.079
LAWDEW	166	3.202	LAWFIC	151.479	3.413	LAWBIU	161.299	2.961	LAWBIU	169.293	3.108	LAWBIU	157.749	3.342
LAWLAW	169.902	3.036	LAWLAW	162.677	3.002	LAWFIC	150.883	3.165	LAWJUO	153.525	3.311	LAWJUQ	150.351	3.402
LAWQUV0	155.22	3.056	LAWQUV0	170.579	2.783	LAWLAW	170.319	2.876	LAWLAW	174.787	2.932	LAWQUV0	150.811	3.428
LAWQUV0	158.003	3.282	LAWQUV0	158.924	3.183	LAWQUV0	153.311	3.17	LAWQUV0	159.027	3.307	LAWQUV0	163.14	2.81
LAWXOX	159.192	3.188	LAWXOX	157.568	2.681	LAWQUV0	151.987	2.82	LAWXOX	153.534	3.39	LAWXOX	175.548	2.757
LAWYOA	164.87	2.932	LAXCAR	173.329	3.036	LAWXOX	168.868	3.193	LAWYEQ	169.895	3.442	LAWYOA	152.83	3.001
LAXFUN	156.275	3.055	LAXGAU	164.996	3.075	LAXCAR	166.506	2.901	LAXFOH	163.895	3.182	LAXFUN	163.005	2.946
LAXLEC	150.233	3.138	LAXNEG	167.133	3.18	LAXGAU	162.114	2.879	LAXGAU	153.982	2.879	LAXKUU	152.231	3.207
LAXSEJ	170.507	3.231	LAXSEK	160.094	3.017	LAXNEG	160.561	3.18	LAXNEG	150.703	3.175	LAXNEG	166.413	3.394
LAXSOT	156.143	3.467	LAXVAK	163.539	3.078	LAXSIN	171.038	3.033	LAXSIN	159.463	2.882	LAXSOT	158.209	3.289
LAYJON	164.325	2.886	LAZBET	165.659	3.474	LAXVAL	154.584	3.455	LAXVUB	171.025	3.219	LAYHUQ	162.008	3.284
LAZBET	154.801	2.998	LAZGUO0	152.86	3.147	LAZBET	153.594	2.984	LAZBET	152.288	3.023	LAZBET	160.42	3.307
LAZHID02	150.736	3.199	LAZHID02	151.799	3.126	LAZGUO0	167.808	3.008	LAZGUO0	166.174	2.817	LAZGUO0	152.542	3.088
LAZREK01	163.283	3.004	LAZREK01	153.039	3.004	LAZHID03	152.231	3.243	LAZHID03	151.588	3.156	LAZJUU	159.16	3.254
LEBBON	154.567	3.12	LEBFIH	155.987	3.111	LAZVAM	163.027	2.852	LAZVAM	160.333	3.021	LAZXAL	155.435	2.969
LEBHIK	174.303	3.481	LEBHOQ	173.512	3.466	LEBFIH	163.487	2.976	LEBFIH	167.912	3.463	LEBHAC	175.151	3.483
LEBVIY	156.604	3.422	LEBYIE	163.392	3.392	LEBVIY	176.279	3.303	LEBVIY	161.793	3.086	LEBVIY	156.345	3.062
LECLUD07	150.742	3.415	LECLUD14	153.938	3.206	LEBYIE	155.061	2.983	LECLUD01	156.462	2.876	LECLUD01	152.471	3.386
LEDBUS	151.569	2.987	LEDBUS	152.855	3.005	LECVUM	169.067	3.073	LECVUM	150.541	3.035	LECYAX	159.193	3.065
LEDFEJ	150.386	3.235	LEDFEJ	165.513	3.007	LEDBUS	164.647	3.322	LEDBUS	156.152	2.92	LEDBUS	166.792	3.337
LEDTIX	172.964	3.266	LEDTIX	163.023	3.426	LEDFOT	150.75	3.481	LEDTIX	162.644	2.891	LEDTIX	150.067	3.381
LEDWIB	154.084	3.181	LEDWIB	163.237	2.751	LEDTIX	156.563	2.926	LEDTIX	161.017	3.285	LEDWIB	150.386	3.181
LEFCIL	159.335	3.304	LEFCIM	150.353	3.034	LEDXEY	156.149	2.886	LEDXEY	162.652	3.369	LEDZUR	151.691	3.054
LEFCIM	151.461	3.161	LEFCIM	166.399	2.936	LEFCIM	175.166	2.941	LEFCIM	153.61	3.106	LEFCIM	167.453	2.918
LEFDEG	159.568	3.356	LEFDEI	164.594	3.293	LEFCIM	158.851	3.052	LEFCOR	155.576	3.099	LEFDEG	162.849	2.89
LEFZEE	165.2	3.331	LEFZII	166.83	2.994	LEFDEI	151.881	3.356	LEFHUD	151.38	2.981	LEFZEE	163.926	2.915
LEGVEC	152.35	2.9	LEGVEC	155.781	3.345	LEFZII	161.52	3.297	LEGRAU	166.353	3.365	LEGVEC	167.04	2.979
LEGVEC	164.43	2.768	LEGVEC	154.014	3.401	LEGVEC	152.47	3.108	LEGVEC	152.207	2.878	LEGVEC	161.444	3.223
LEHTOI	166.189	3.425	LEHTOI	154.254	3.001	LEHMUI	153.72	3.354	LEHMUI	157.84	3.117	LEHTOI	164.975	2.898
LEHTOI	152.491	2.968	LEHTOI	152.744	2.796	LEHTOI	158.902	3.413	LEHTOI	161.426	3.137	LEHTOI	150.962	2.888
LEJCIP	160.316	3.238	LEJFIQ	161.88	3.049	LEJCAF	158.414	3.048	LEJCAF	153.464	3.296	LEJCIP	151.256	3.066
LEJFIQ	150.935	3.378	LEJFIQ	155.959	3.049	LEJFIQ	153.589	2.927	LEJFIQ	156.782	3.262	LEJFIQ	151.802	3.127
LEJWAC	164.974	2.888	LEJYOS	171.194	3.274	LEJHIV	164.512	2.998	LEJLUJ	156.314	3.149	LEJWAC	161.964	2.919
LEKPEX	164.924	3.363	LEKPEX	159.129	2.789	LEKNOF	168.839	2.708	LEKPEX	174.389	2.774	LEKPEX	152.036	3.433
LELCAH	159.477	2.751	LELCAH	158.168	3.107	LEKQEA	150.215	2.958	LELBUC	174.659	2.948	LELCAH	168.517	3.476
LELCIP	156.698	3.023	LELCIP	156.783	2.902	LELCAH	170.016	2.932	LELCAH	151.434	3.238	LELCAH	156.391	3.139
LELLUL	154.162	2.784	LELLUL	157.497	2.979	LELCIP	157.546	2.934	LELCIP	158.495	3.062	LELCIP	155.805	2.804
LELZUZ	152.603	3.127	LEMCAJ	170.711	2.974	LELQID	154.271	3.432	LELQID	159.68	2.869	LELYIO	159.758	3.492
LEMGUJ	159.478	3.169	LEMSOL	155.113	2.956	LEMCEP	154.721	3.208	LEMCEP	167.247	3.109	LEMGOD	152.682	3.449
LENHOC	160.065	3.251	LENHOC	158.059	3.409	LEMSOL	163.012	2.864	LENBUD	150.737	2.947	LENBUD	159.641	3.356
LENJAQ	152.351	2.931	LENJEU	156.512	3.263	LENHUI	166.951	3.227	LENHUI	156.418	3.322	LENHUI	161.972	2.925
LENJEU	166.056	3.048	LENJEU	163.512	3.281	LENJEU	169.674	3.393	LENJEU	151.325	3.238	LENJEU	161.724	3.238
LEQJEX	151.723	3.475	LEQJEX	156.939	3.316	LENLEW	172.192	3.399	LEPTAB	167.872	3.251	LEQCIV	158.453	3.157
LEQKOL	164.489	2.909	LEQKOL	169.56	3.062	LEQJEX	159.543	3.328	LEQKOL	150.239	3.184	LEQKOL	153.26	3.329
LERJAV	164.467	3.158	LERJAV	152.352	3.459	LEQKOL	150.251	3.237	LEQQAB	152.287	3.334	LEQQEF	157.12	3.258
LERZOA	155.314	2.801	LESCOD	161.255	3.078	LERNOO0	159.663	3.286	LERXOY	151.204	2.729	LERXOY	166.829	2.771
LESDAP	169.385	2.903	LESDUJ	166.183	2.986	LESCOF	172.709	3.019	LESCOF	166.591	2.854	LESDAP	152.869	2.783
LESGAT	159.499	3.118	LESGAT	171.319	2.941	LESGAT	150.005	3.304	LESGAT	172.278	3.221	LESGAT	154.3	3.207
LESQUY	150.05	2.875	LESQUY	159.546	3.247	LESHEY	151.639	3.291	LESJUP	163.378	2.953	LESMON	152.748	3.432
LETFEV	160.187	3.465	LETQEJ	158.618	3.327	LESQUY	161.347	3.269	LESRAE	170.099	3.331	LESREI	150.703	3.481
LEVBOF	163.163	2.977	LEVBOF	156.128	3.253	LETQEJ	151.671	3.418	LEVBOF	168.695	2.89	LEVBOF	154.742	2.89
LEVBOF	153.257	3.481	LEVBUL	166.627	2.732	LEVBOF	153.8	3.136	LEVBOF	152.604	2.869	LEVBOF	163.406	2.931
LEVDOH	151.388	2.971	LEVDOH	150.792	2.769	LEVCAU	158.359	3.093	LEVCAU	168.68	2.878	LEVCUO	153.813	3.146
LEVFEZ	161.998	3.205	LEVFEZ	169.88	3.391	LEVDOH	153.567	2.811	LEVFEZ	153.236	2.861	LEVFEZ	165.708	3.03
LEVJON	167.333	2.883	LEVMOQ	153.226	3.442	LEVFEZ	162.705	3.466	LEVFEZ	151.238	3.339	LEVFEZ	165.284	2.732
LEVXOZ	168.183	3.15	LEVXOZ	161.382	2.95	LEVMOQ	154.399	3.408	LEVRIR	155.281	3.408	LEVXOZ	169.157	3.238
LEVYAM	153.109	3.019	LEVYAM	154.832	3.071	LEVXOZ01	168.466	3.149	LEVXOZ01	169.896	3.094	LEVXOZ01	161.253	2.899
LEWSEP	165.463	2.845	LEWSEP	161.017	2.89	LEVYAM	156.755	2.755	LEVYAM	165.349	2.938	LEWSEP	167.787	2.753
LEWSEP	161.323	3.5	LEWZIX	171.341	2.813	LEWSEP	150.432	2.949	LEWSEP	150.774	3.499	LEWSEP	152.223	3.183
LEXBUN	168.414	3.406	LEXBUN	150.648	3.176	LEWZIX	152.41	3.032	LEXBAT	151.136	3.261	LEXBOH	155.881	3.442
LEXRIT	164.317	2.84	LEXRIT	160.003	2.94	LEXMOR	159.172	3.162	LEXRIT	157.817	3.031	LEXRIT	158.054	2.73
LEYQEN	150.15	3.486	LEYRAL	173.668	3.255	LEXRIT	166.403	2.963	LEYDIG	157.804	3.373	LEYDIG	163.346	3.185
LEZHII	164.157	3.107	LEZHII	156.028	3.304	LEYSAM	164.298	2.891	LEYXIA	160.244	2.993	LEYYOD	169.189	3.253
LEZQAJ	150.813	2.902	LEZTAP	153.516	2.921	LEZHII01	166.585	3.001	LEZHII01	154.567	3.142	LEZPUD	152.717	3.146
LEZZOI	167.232	2.773	LIBCIM	175.038	3.175	LEZTET	160.111	3.17	LEZVAQ	152.859	3.015	LEZVAQ	157.835	3.335
LIBFIO	153.351	3.123	LIBFIO	154.134	2.974	LIBCOS	150.555	3.387	LIBDEG	156.211	2.678	LIBFIO	153.293	3.318
LIBFOU	161.48	2.624	LIBGIP	153.672	2.924	LIBFIO	163.925	3.044	LIBFOU	153.83	2.86	LIBFOU	155.291	2.624
LIBGIP	162.88	2.981	LIBGUY	168.657	2.934	LIBGIP	170.528	2.921	LIBGIP	155.223	2.921	LIBGIP	160.958	3.213
LIBLUG	158.198	3.242	LIBMOY	156.963	3.379	LIBGUY	161.514	3.415	LIBHOW	159.897	3.177	LIBLUG	166.482	3.379
LIBWEZ	155.276	3.15	LICDUZ	162.102	3.283	LIBMOY	162.283	3.379	LIBSOI	173.522	3.48	LIBSOI	151.198	2.985
LICLUI	166.744	3.215	LICLUI	156.614	3.156	LICDUZ	151.855	3.405	LICLIW	152.197	3.233	LICLIW	151.639	3.225

LICMAP	162.347	3.205	LICMAP	158.669	3.04	LICLUI	171.494	3.358	LICLUI	156.344	3.057	LICMAP	173.237	3.218
LICWUR	151.074	3.099	LICWUR	158.112	3.323	LICMAP	159.662	3.288	LICTAU	165.138	3.212	LICWUR	167.698	3.45
LICXAX	158.313	3.176	LICXEE	172.593	2.7	LICXAA	174.664	3.036	LICXAA	155.331	3.119	LICXAX	151.396	2.999
LICXEE	151.58	3.192	LICYAX	177.197	3.324	LICXEE	177.228	2.788	LICXEE	155.228	2.743	LICXEE	160.37	2.898
LIDGEL	153.8	3.483	LIDGEL	161.176	2.986	LICYAX	164.287	3.143	LIDGEL	156.643	2.986	LIDGEL	164.889	2.821
LIDJUF	155.445	3.327	LIDQIZ	160.067	3.476	LIDGEL	161.686	2.821	LIDHOW	156.207	3.038	LIDHOW	165.819	3.077
LIFSOK	158.18	3.255	LIFSOK	166.139	2.732	LIDYEG	170.266	2.796	LIFDEJ	157.471	3.007	LIFSOK	155.051	3.081
LIFSOK	172.478	2.903	LIFSOK	162.341	2.736	LIFSOK	158.72	2.807	LIFSOK	164.284	2.9	LIFSOK	166.207	2.89
LIFSOK	171.737	3.239	LIFSOK	160.73	3.178	LIFSOK	157.586	3.016	LIFSOK	155.932	3.083	LIFSOK	157.242	2.807
LIGFAL	165.002	2.855	LIGFAL	158.749	3.42	LIFSOK	153.486	2.893	LIGBIQ	150.491	3.419	LIGCAJ	166.891	3.087
LIGFOW	169.478	3.452	LIGGIV	175.311	3.451	LIGFAL	158.326	3.477	LIGFAL	171.299	3.44	LIGFAL	158.467	3.384
LIGGUG	160.893	3.173	LIGGUG	164.396	2.752	LIGGIV	165.274	3.107	LIGGIV	169.524	3.303	LIGGUG	155.894	2.778
LIGLOG	163.026	3.011	LIGLOG	151.326	3.087	LIGGUG	160.181	3.081	LIGGUG	176.982	2.803	LIGGUG	163.582	3.137
LIGLOG	153.787	3.259	LIGLOG	155.506	3.199	LIGLOG	160.478	2.927	LIGLOG	153.957	3.255	LIGLOG	150.436	3.036
LIGLOG	153.142	2.939	LIGLOG	150.218	2.967	LIGLOG	153.529	3.28	LIGLOG	163.655	3.459	LIGLOG	155.125	2.844
LIGLOG	156.03	3.064	LIGLOG	152.986	2.802	LIGLOG	151.866	3.026	LIGLOG	176.778	3.053	LIGLOG	152.02	2.893
LIGLOG	158.984	3.117	LIGLOG	153.26	2.923	LIGLOG	159.12	2.957	LIGLOG	160.317	2.98	LIGLOG	154.238	3.082
LIGLOG	156.921	2.882	LIGLOG	173.64	3.081	LIGLOG	161.512	2.869	LIGLOG	151.037	2.953	LIGLOG	152.202	3.023
LIGLOG	153.336	3.261	LIGLOG	173.694	3.327	LIGLOG	166.338	2.828	LIGLOG	151.422	2.883	LIGLOG	176.881	3.062
LIGLOG	157.093	2.966	LIGLOG	156.962	3.16	LIGLOG	161.19	2.915	LIGLOG	158.658	3.283	LIGLOG	158.162	3.069
LIGLOG	153.302	3.169	LIGLOG	150.868	3.322	LIGLOG	169.718	2.737	LIGLOG	168.916	3.296	LIGLOG	153.636	2.987
LIGLOG	154.2	3.229	LIGLOG	166.3	3.228	LIGLOG	150.643	3.107	LIGLOG	163.854	3.421	LIGLOG	151.609	3.008
LIGNEY	161.05	3.051	LIGNEY	154.545	2.94	LIGNEY	160.82	3.433	LIGNEY	168.126	3.188	LIGNEY	152.419	3.357
LIGNEY	163.575	3.222	LIGNEY	153.813	2.927	LIGNEY	170.952	3.433	LIGNEY	163.408	3.039	LIGNEY	164.031	3.061
LIGNEY	157.291	3.114	LIGTUS	157.937	3.351	LIGNEY	164.155	3.082	LIGNEY	163.057	2.861	LIGNEY	160.446	3.158
LIHQEZ	158.412	2.933	LIHQEZ	157.143	3.146	LIHDOY	160.386	2.879	LIHNAT	159.626	3.271	LIHNUO	154.863	2.96
LIHZEH	151.9	3.077	LIHZEH	161.255	3.163	LIHQID	169.294	3.115	LIHRAY	172.844	2.994	LIHRAY	170.721	2.671
LIJDOZ	151.662	3.053	LIJDUG	154.867	3.485	LIHZOR	152.221	3.079	LIJBEL	154.392	2.886	LIJBEL	160.6	3.181
LIJMUO	150.689	2.837	LIJMUO	153.817	3.001	LIJDUG	158.883	3.347	LIJFES	151.962	2.922	LIJLUM	154.011	2.766
LIJMUO	163.098	3.19	LIJMUO	162.593	3.49	LIJMUO	159.039	3.064	LIJMUO	166.642	2.959	LIJMUO	156.966	2.949
LIJMUO	153.925	3.092	LIJMUO	158.114	2.97	LIJMUO	159.29	3.149	LIJMUO	160.853	3.149	LIJMUO	159.239	2.913
LIJMUO	153.342	2.837	LIJPIE	158.44	3.084	LIJMUO	156.711	3.013	LIJMUO	153.454	2.94	LIJMUO	152.337	2.805
LIJPIE	168.127	3.034	LIJPIE	152.755	2.923	LIJPIE	167.879	2.891	LIJPIE	172.702	2.939	LIJPIE	150.733	3.376
LIJTOQ	155.333	2.776	LIJTOQ	171.111	3.339	LIJPIE	160.965	3.084	LIJTOQ	152.149	2.963	LIJTOQ	155.152	2.782
LIJTOQ	150.422	2.999	LIJTOQ	157.957	3.071	LIJTOQ	174.17	2.997	LIJTOQ	150.968	3.178	LIJTOQ	155.968	2.746
LIJTOQ	151.701	3.001	LIJTOQ	161.462	2.985	LIJTOQ	156.463	2.79	LIJTOQ	167.812	3.176	LIJTOQ	150.88	2.956
LIJZOX	161.176	3.021	LIJZOX	159.133	3.168	LIJTOQ	174.52	2.776	LIJTOQ	150.461	2.999	LIJZOX	157.857	3.108
LIKPOL	162.784	3.093	LIKPOL	167.642	3.231	LIKKUM	161.472	3.26	LIKLAW	159.194	2.942	LIKPIF	152.035	2.864
LIKZOU	164.365	3.087	LILBEP	156.406	2.945	LIKPOL	172.576	3.093	LIKTUX	154.54	2.824	LIKZOU	167.072	3.352
LILBEP	165.888	3.189	LILFAO	173.268	3.429	LILBEP	175.218	2.809	LILBEP	152.416	2.975	LILBEP	170.337	2.983
LILMAX	166.678	3.209	LILMEA	178.808	2.553	LILFAO	153.344	2.729	LILFAO	153.924	2.648	LILGAQ	151.582	3.252
LILMIE	158.714	2.517	LILMOK	160.298	3.177	LILMEA	173.989	2.553	LILMEA01	167.135	3.217	LILMEB	156.885	3.316
LILZEM	155.422	3.022	LILZIT	158.639	2.999	LILMOK	153.713	3.121	LILMOK	160.265	3.182	LILMOK	151	3.116
LIMKIE	171.66	3.457	LIMKIE	160.745	3.319	LIMDAP	154.947	3.494	LIMDAP	160.042	3.302	LIMKIE	151.163	3.275
LIMLOM	160.931	2.848	LIMMAZ	165.754	3.186	LIMKIE	171.957	2.651	LIMLOM	158.401	2.848	LIMLOM	150.801	2.834
LIMMUT	153.953	2.872	LIMMUT	161.976	3.239	LIMMUT	153.81	3.122	LIMMUT	154.778	3.122	LIMMUT	159.726	3.123
LIMTAE	156.076	3.399	LIMTEJ	154.284	3.014	LIMMUT	164.708	3.37	LIMTAE	162.878	3.488	LIMTAE	152.906	3.336
LINLUT	158.526	3.108	LINLUT	157.45	2.951	LINFIX	161.53	3.145	LINFIX	164.063	3.127	LINGAU	163.793	3.466
LINTUZ	152.982	2.879	LINXIS	166.162	2.959	LINLUT	152.657	3.17	LINPAC	153.158	2.945	LINSAC	168.341	3.128
LINXOY	157.407	3.309	LINXOY	153.163	3.425	LINXIS	162.189	3.315	LINXIS	167.583	3.385	LINXIS	152.683	3.385
LIPZAP	162.381	3.335	LIPZAP	153.575	3.022	LIPLEB	174.694	3.401	LIPLEB	164.853	3.031	LIPMAC	154.537	3.31
LIPZAP	165.511	3.477	LIPZAP	159.344	3.404	LIPZAP	163.374	3.326	LIPZAP	154.507	3.24	LIPZAP	163.213	3.228
LIQGUQ	162.712	3.092	LIQLII	150.985	3.347	LIQGUQ	165.83	3.133	LIQGUQ	154.143	3.089	LIQGUQ	152.024	3.21
LIQVOZ	164.169	3.105	LIQVUF	151.976	3.049	LIQSEN	167.772	3.492	LIQSEN	167.772	2.73	LIQVOZ	151.002	3.041
LIRMEE	159.99	3.238	LIRMEE	151.951	3.467	LIQVUF	164.262	3.103	LIRLUV	156.565	2.995	LIRLUV	179.353	3.399
LIRMEE	151.952	3.07	LIRMII	168.119	3.068	LIRMEE	155.063	3.462	LIRMEE	152.585	3.208	LIRMEE	159.742	3.104
LIRMOO	171.927	3.407	LIRMOO	155.905	3.124	LIRMII	150.387	2.875	LIRMII	167.909	3.498	LIRMII	150.938	2.943
LIRZUL	151.424	3.434	LIRZUL	150.05	3.166	LIRVIR	161.877	3.414	LIRVIR	153.413	2.847	LIRZUL	162.732	3.391
LIRZUL	157.055	2.855	LISBAU	166.686	2.693	LIRZUL	151.293	2.848	LIRZUL	168.254	2.779	LIRZUL	158.603	2.989
LISFEC	162.135	2.967	LISFEC	159.87	2.947	LISCEY	172.125	3.231	LISFEC	153.209	2.73	LISFEC	152.724	3.381
LISNAE	176.962	3.328	LISPAF	168.812	3.377	LISFEZ	169.34	3.223	LISFEZ	164.031	3.305	LISFIF	151.514	3.402
LITJUV	160.909	2.914	LITLUY	166.923	3.191	LISWEQ	168.854	3.103	LISWOZ	153.476	2.576	LITBID	156.948	3.357
LITWEU	158.002	3.152	LITWEU	150.041	3.036	LITMOU	153.485	2.911	LITTUD	177.514	3.015	LITTUD	170.041	3.467
LIVCOM	171.845	3.437	LIVCOM	152.398	2.647	LITWEU	168.869	3.459	LITZAR	154.186	2.988	LITZOE	161.689	3.068
LIVYAQ	171.28	3.265	LIVYAQ	155.979	2.773	LIVJAB	155.977	2.888	LIVVUH	158.957	2.762	LIVVUH	163.884	3.083
LIVYAQ	161.724	2.945	LIVYAQ	161.979	2.773	LIVYAQ	155.783	3.234	LIVYAQ	169.042	3.077	LIVYAQ	177.256	2.898
LIWGIK	151.043	3.251	LIWGIK	170.28	3.059	LIVYAQ	150.561	3.234	LIVZEV	175.271	2.905	LIVZEV	152.054	2.905
LIWPIU	150.636	3.211	LIWPIU	157.163	3.115	LIWJIL	169.89	2.879	LIWJIL	154.548	3.16	LIWJIL	171.398	3.01
LIWZUP01	151.316	3.279	LIXBEC	160.81	3.076	LIWPUG	160.068	2.726	LIWQAN	151.145	2.981	LIWZUP	151.637	3.305
LIXMUA	176.536	3.008	LIXPUH	150.349	2.966	LIXBEC	156.559	3.052	LIXDAA	155.154	2.956	LIXFAC	155.99	3.326
LIXPUH	156.162	3.454	LIXPUH	153.203	3.471	LIXPUH	169.569	2.983	LIXPUH	160.021	3.471	LIXPUH	150.796	2.831
LIXTAP	177.083	3.283	LIYHOR	166.368	3.272	LIXRAM	160.586	3.063	LIXRIU	168.022	2.751	LIXSAO	152.495	3.027
LIYHOR	177.543	2.926	LIYHOR	163.586	3.088	LIYHOR	167.644	2.926	LIYHOR	160.094	3.17	LIYHOR	158.568	3.034
LIYHUX	172.912	3.159	LIYHUX	176.989	3.005	LIYHUX	176.937	2.989	LIYHUX	168.738	3.126	LIYHUX	167.69	2.913
LIYJIO	157.47	2.765	LIYJUY	168.098	3.454	LIYHUX	157.58	3.179	LIYHUX	166.485	2.926	LIYHUX	162.516	3.164
LIYKAF	159.553	3.301	LIYKAF	155.407	3.423	LIYJUY	155.278	3.385	LIYJUY	163.847	2.836	LIYKAF	152.003	3.15
LIYNOY	167.718	3.356	LIYPOA	153.047	3.198	LIYKAF	166.557	3.16	LIYNOY	160.097	3.45	LIYNOY	157.989	3.341
LIYZAU	158.943	2.848	LIYZUS	168.923	2.911	LIYPOA	168.341	2.953	LIYVUL	174.529	3.367	LIYYEY	164.94	3.229
LIZJUC	152.757	3.348	LIZKAJ	169.18	3.325	LIZJUC	153.174	3.127	LIZJUC	162.502	3.018	LIZJUC	153.798	3.362

LIZKAJ	171.102	2.922	LIZLUF	169.673	2.868	LIZKAJ	151.627	3.098	LIZKAJ	157.89	2.958	LIZKAJ	172.125	3.016
LIZYEZ	164.025	3.265	LIZYEZ	169.751	2.911	LIZLUF	159.118	3.27	LIZSUL	157.927	3.45	LIZSUL	161.21	3.381
LIZYEZ	153.097	2.712	LIZYEZ	159.585	2.894	LIZYEZ	152.122	3.312	LIZYEZ	161.321	3.302	LIZYEZ	159.979	3.312
LOBCOV	155.251	3.428	LOBCOV	152.293	3.237	LIZYEZ	153.302	2.911	LIZYEZ	173.812	3.012	LIZYEZ	150.834	2.704
LOBJOB	154.097	3.202	LOBJOB	156.751	2.762	LOBCUB	174.39	3.25	LOBJOB	162.061	3.476	LOBJOB	156.28	2.854
LOCKEU	159.897	3.327	LOCKEU	152.947	3.081	LOBPEB	176.364	3.07	LOBPEB	155.978	3.241	LOCGAM	159.617	3.072
LOCVUV	160.029	3.476	LOCXUZ	150.208	2.842	LOCKEU	167.553	3.166	LOCPID	162.889	3.381	LOCQAW	167.545	3.419
LOCYUX	152.443	3.289	LOCYUX	175.895	3.176	LOCXUZ	159.333	3.35	LOCXUZ	151.063	3.025	LOCYEK	158.572	3.28
LODGUH	152.388	3.105	LODGUH	151.001	3.361	LOCZAE	173.003	2.917	LODGUH	154.481	3.318	LODGUH	152.805	3.373
LODKUL	157.497	2.984	LODLAS	158.111	3.364	LODKOF	163.576	3.059	LODKOF	156.628	3.062	LODKUL	161.877	3.015
LODLUN	177.008	3.294	LODPAZ	154.405	2.984	LODLAS	150.354	3.218	LODLEW	157.247	3.366	LODLEW	151.657	3.205
LODRIJ	157.356	3.386	LODRIJ	153.518	3.329	LODRIJ	150.569	3.092	LODRIJ	151.846	2.876	LODRIJ	160.703	2.89
LODYEL	158.095	3.333	LOFDAM	165.321	2.588	LODRIJ	161.753	3.361	LODRIJ	154.699	2.876	LODVUZ	163.55	3.002
LOFMEB	162.363	2.77	LOFNEB	167.111	3.294	LOFDEQ	163.784	2.527	LOFKEZ	153.303	3.281	LOFKEZ	160.523	3.307
LOFQOO	151.487	3.406	LOFQOO	152.739	2.975	LOFQEE	174.298	2.954	LOFQOO	150.949	3.184	LOFQOO	170.188	3.195
LOFWOU	166.232	2.964	LOGDIW	174.282	2.838	LOFQOO	159.429	3.456	LOFWOU	172.034	3.273	LOFWOU	150.546	3.315
LOGKAU	155.076	2.94	LOGKAU	161.443	3.009	LOGJEX	174.619	3.353	LOGJEX	154.986	3.353	LOGJUN	166.957	2.95
LOGKIC	174.998	2.738	LOHCIV	161.366	3.044	LOGKEY	168.709	3.076	LOGKIC	154.671	2.738	LOGKIC	155.319	3.365
LOHCIV	169.707	3.224	LOHJIB	165.517	3.026	LOHCIV	159.038	3.079	LOHCIV	155.879	2.997	LOHCIV	154.149	3.137
LOHTIL	158.571	3.376	LOJJAW	169.152	3.437	LOHJIB	150.379	2.947	LOHPED	167.08	3.345	LOHTIL	161.7	3.376
LOJLAA	152.097	2.758	LOJWIU	173.263	3.474	LOJKEB	163.144	3.328	LOJKEB	156.117	3.175	LOJKEB	170.446	3.273
LOKLIG	150.416	3.207	LOKLIG	151.246	3.401	LOJWIU	165.047	3.345	LOJWOA	154.833	3.124	LOJWOX	157.632	3.045
LOKPOS	168.815	2.711	LOKPOS	152.288	2.711	LOKLIG	159.739	3.147	LOKLIG	159.39	3.404	LOKPOS	150.68	2.706
LOKPOS	178.867	3.368	LOKRIN	154.715	2.744	LOKPOS	172.994	2.773	LOKPOS	165.263	2.968	LOKPOS	156.768	2.781
LOKVOX	159.827	3.162	LOKVOX	170.262	3.044	LOKRIN	156.797	3.129	LOKRIN	155.486	3.302	LOKRIN	162.663	3.205
LOKWAK	160.84	3.468	LOKWAK	151.274	3.282	LOKVOX	172.45	3.23	LOKVOX	172.298	2.909	LOKWAK	150.143	3.468
LOLFAX	157.327	3.188	LOLGUQ	157.93	3.414	LOLCUN	169.506	2.966	LOLDEZ	160.76	3.382	LOLDID	161.967	2.948
LOLHIG04	150.12	3.339	LOLREJ	164.471	3.216	LOLHED	175.041	3.23	LOLHIG02	153.764	3.463	LOLHIG03	150.12	3.252
LOLVAK	152.149	2.608	LOLVAK	155.635	2.745	LOLTEM	155.213	3.12	LOLTIQ	150.506	3.352	LOLTOW	159.237	2.71
LOMBAS	168.259	2.918	LOMDAW	172.094	3.26	LOLWOC	150.738	3.083	LOLWOC	160.843	3.173	LOLWOC	157.137	2.982
LOMQOW	168.274	2.831	LOMQOW	152.086	3.19	LOMDAW	162.297	3.493	LOMDAW	169.369	3.5	LOMGIE	165.978	2.996
LOMTOY	164.579	3.069	LOMZAP	161.808	3.345	LOMQOW	157.441	3.039	LOMTOX	154.703	3.237	LOMTOY	150.492	3.06
LONMIN	166.491	3.171	LONTIS	158.004	3.315	LOMZAP	172.621	3.269	LONCID	156.919	2.778	LONMIN	168.132	3.039
LONXIW	153.359	3.099	LONYEW	154.356	3.05	LONTIS	179.552	3.252	LONXES	155.471	3.059	LONXIW	152.81	3.278
LONYEW	159.284	2.722	LONYEW	161.078	2.904	LONYEW	174.314	2.837	LONYEW	159.269	2.71	LONYEW	164.931	3.104
LONYEW	153.057	3.497	LONYOD	169.356	3.054	LONYEW	153.411	2.716	LONYEW	163.836	2.784	LONYEW	160.83	2.789
LOPCIG	154.285	2.828	LOPCIG	154.795	2.828	LONYUJ	161.365	3.02	LONYUJ	152.515	3.32	LOPBUP	179.315	2.701
LOPXOD	162.517	3.137	LOPXOD	152.047	3.358	LOPLEK	170.457	3.119	LOPNOW	161.095	3.063	LOPNOW	167.145	3.456
LOQLUA	157.666	3.233	LOQLUA	163.047	3.233	LOPXOD	154.224	3.157	LOQBEZ	161.822	3.444	LOQLEK	156.999	3.147
LOQQAN	175.71	3.499	LORHAE	168.97	3.369	LOQNAI	150.553	3.494	LOQNAI01	165.904	2.971	LOQQAN	179.076	3.385
LORYOI	159.753	3.304	LORZUP	167.367	3.027	LORKIQ	162.958	2.54	LORQUH	167.853	2.754	LORWAU	150.074	3.467
LOSFII	156.682	3.201	LOSFII	155.037	3.08	LORZUP	159.053	3.027	LOSDAZ	160.206	3.108	LOSDAZ	152.524	3.406
LOSJAH	162.149	3.152	LOSJAH	170.658	3.491	LOSJAH	156.176	3.25	LOSJAH	153.974	3.419	LOSJAH	153.051	3.153
LOSLOX	165.942	3.484	LOSLOX	168.37	2.647	LOSJAH	151.726	2.904	LOSJAH	162.32	3.072	LOSJAH	162.959	3.487
LOSMAK	152.453	3.13	LOSMAK	160.009	3.092	LOSLOX	152.77	3.414	LOSLUD	166.659	2.817	LOSLUD	165.386	3.441
LOSTOC	154.099	3.364	LOSZAW	153.202	2.865	LOSMAK	172.259	3.201	LOSMAK	153.936	3.404	LOSNOX	157.094	3.217
LOSZOK	159.73	3.433	LOSZOK	176.562	3.019	LOSZAW	153.712	2.998	LOSZIE	152.98	3.208	LOSZIE	161.835	3.283
LOTVOJ	164.951	3.464	LOTVOJ	151.513	2.945	LOSZOK	177.138	3.038	LOTBED	173.267	2.795	LOTHEH	150.448	3.287
LOWHOX	155.133	3.431	LOWHUD	167.347	3.036	LOVQIA	177.573	2.792	LOVWIC	170.352	2.866	LOWBEE	173.764	3.263
LOWKAN	162.204	3.033	LOWKAN	175	2.799	LOWHUD	165.847	2.986	LOWHUD	155.315	3.443	LOWKAN	154.943	3.407
LOWLAM	158.859	3.08	LOWLAM	161.019	3.121	LOWKAN	156.224	3.357	LOWKAN	150.296	2.865	LOWLAM	165.747	3.311
LOWMOD	150.527	3.21	LOWMUJ	152.065	3.123	LOWLES	160.282	2.98	LOWLOC	174.576	3.492	LOWMAP	167.511	2.885
LOWNOE	157.854	3.29	LOWPAQ	160.909	3.257	LOWMUJ	163.505	3.314	LOWMUJ	164.152	3.219	LOWNAQ	165.252	3.051
LOWTUP	169.565	3.115	LOWTUP	158.555	2.655	LOWPUM	168.738	3.377	LOWTUP	157.581	3.401	LOWTUP	153.839	3.395
LOWTUP	160.738	3.42	LOWTUP	166.652	2.955	LOWTUP	153.637	3.301	LOWTUP	150.952	3.476	LOWTUP	150.722	2.928
LOWZAC	152.139	3.443	LOWZEG	151.12	3.408	LOWTUP	168.08	2.869	LOWTUP	152.565	3.039	LOWTUP	155.521	3.416
LOXCIO	175.822	2.99	LOXCOT	151.759	3.238	LOWZOQ	157.446	3.473	LOWZUW	157.572	3.5	LOXBAF	150.132	3.402
LOXFIP	163.429	3.105	LOXLOB	154.265	2.956	LOXCOT	166.987	2.929	LOXFEL	155.683	3.335	LOXFEL	153.502	2.807
LOXMIW	152.575	3.478	LOXMIW	155.674	3.473	LOXLOB	168.213	2.945	LOXLOB	160.822	2.891	LOXMIW	163.614	3.259
LOXPOF	153.281	3.074	LOXWOK	153.02	3.069	LOXNIZ	151.829	3.172	LOXNIZ	161.426	3.07	LOXPOF	162.303	2.853
LOYGEL	166.159	3.276	LOYGEL	150.977	3.252	LOXYII	158.709	3.187	LOXYOQ	174.067	3.045	LOYGEL	155.519	3.08
LOZDAJ	153.112	3.089	LOZDAJ	152.082	3.168	LOYGEL	162.892	3.117	LOYGEL	150.174	3.252	LOYGEL	160.585	3.08
LOZKOE	160.097	3.195	LOZQUP	158.108	3.329	LOZDUD	151.077	3.184	LOZDUD	154.194	3.358	LOZKOE	176.181	3.195
LUBFUM	165.888	3.01	LUBFUM	159.61	3.015	LOZQUP	161.785	3.227	LOZROK	153.144	3.487	LOZROK	150.614	2.943
LUBMOO	176.067	3.414	LUBMOO	163.402	3.355	LUBJEA	162.209	3.188	LUBJOK	160.167	3.11	LUBMOO	160.881	3.272
LUCDOG	153.351	3.117	LUCDOG	169.469	3.116	LUBTOR	150.876	2.884	LUBTUZ	159.617	2.95	LUCBUK	171.403	3.209
LUCHAW	156.526	2.889	LUCHAW	157.326	3.495	LUCHAW	152.846	2.83	LUCHAW	154.261	3.246	LUCHAW	165.155	2.652
LUCZAM	155.862	3.393	LUDRUA	176.99	3.275	LUCHAW	157.687	2.978	LUCHAW	172.192	3.274	LUCZAM	154.653	3.193
LUFBUK	165.017	3.29	LUFBUK	158.714	2.928	LUFBUK	158.54	3.499	LUFBUK	154.656	3.241	LUFBUK	171.506	2.867
LUFLEE	154.996	3.033	LUFLEE	159.866	2.969	LUFBUK	159.345	3.306	LUFBUK	154.604	2.702	LUFBUK	152.553	3.131
LUFQIQ	150.072	3.382	LUFQIQ	174.502	3.276	LUFLII	158.764	3.108	LUFLII	159.976	3.115	LUFLOO	165.104	3.183
LUFQUC	164.773	3.278	LUFQUC	177.407	2.88	LUFQUC	158.263	3.372	LUFQUC	176.848	2.878	LUFQUC	157.511	3.371
LUFROX	165.881	3.29	LUGKOQ	153.513	2.725	LUFQUC	165.234	3.287	LUFRAJ	155.007	2.979	LUFROX	153.632	2.839
LUGLIJ	166.061	2.696	LUGLOP	162.196	3.264	LUGLIJ	155.735	2.696	LUGLIJ	166.343	3.372	LUGLIJ	157.655	3.39
LUGLUV	167.131	3.427	LUGLUV	160.555	2.683	LUGLOP	163.558	2.731	LUGLOP	166.665	3.461	LUGLOP	174.586	2.731
LUGTOY	164.481	2.763	LUGVIW	152.001	3.148	LUGLUV	156.632	3.142	LUGLUV	174.082	2.683	LUGTOY	150.433	3.392
LUHFUT	152.107	3.261	LUHJOQ	152.093	3.121	LUGZET	166.861	3.085	LUGZUK	152.303	2.952	LUHBOJ	152.249	3.499
LUHQAJ	161.765	2.829	LUHQAJ	171.016	3.162	LUHNOT	169.237	2.917	LUHNOT	157.821	3.387	LUHNUZ	168.451	2.899
LUHSEO	152.836	2.911	LUHTEQ	171.299	3.099	LUHQAJ	163.501	3.5	LUHQAJ	170.247	3.32	LUHSEO	173.23	3.495

LUJNOU	157.179	3.184	LUJROX	170.429	3.319	LUHWIV	161.106	3.473	LUHWOC	150.353	2.913	LUJGUS	155.355	3.104
LUKHAD	151.547	2.961	LUKHAD	165.444	3.286	LUKHAD	171.617	3.497	LUKHAD	167.708	3.481	LUKHAD	153.867	2.737
LUKMOT	173.386	3.09	LUKMOT	153.142	2.913	LUKHAD	154.494	2.82	LUKHAD	156.049	3.118	LUKLAI	158.307	3.102
LULBON	160.031	3.082	LULBON	170.958	2.924	LUKQOX	155.625	3.271	LUKYEZ	164.344	3.06	LULBIH	176.699	3.039
LULBUT	172.25	2.739	LULCAA	166.317	3.401	LULBON	161.946	3.124	LULBON	168.825	2.813	LULBUT	153.362	2.725
LULHEJ	164.877	3.497	LULHEJ	153.533	3.094	LULDEC	155.354	3.144	LULDEC	155.159	3.144	LULHEH	153.577	3.249
LULRAL	153.744	2.844	LULREP	158.581	3.276	LULKOS	154.335	2.986	LULKOS	151.246	3.157	LULQOA	159.32	3.22
LUMCOL	159.163	3.463	LUMCOL	157.64	3.176	LULRIT	150.726	3.184	LULROZ	155.884	3.494	LULXUM	162.18	3.305
LUMCUR	157.478	3.217	LUMCUR	154.874	3.345	LUMCOL	164.532	2.965	LUMCOL	162.068	3.436	LUMCOL	158.697	2.807
LUMCUR	154.658	3.021	LUMCUR	158.807	2.914	LUMCUR	164.044	3.154	LUMCUR	154.944	3.475	LUMCUR	157.329	3.41
LUMGAF	170.975	2.824	LUMGAF	176.855	2.92	LUMCUR	168.126	2.935	LUMCUR	160.555	3.034	LUMFOS	156.858	3.368
LUMKEM	163.215	3.141	LUMKEM	164.96	2.971	LUMGAF	154.751	3.344	LUMGAF	164.885	2.92	LUMKEM	155.814	2.739
LUMKEM	155.303	3.059	LUMNIS	151.129	3.058	LUMKEM	154.535	2.93	LUMKEM	151.529	3.105	LUMKEM	160.618	2.739
LUMNIT	173.097	2.81	LUMNIT	172.962	3.08	LUMNIT	154.318	3.162	LUMNIT	155.432	3.334	LUMNIT	156.82	3.178
LUMNOZ	150.16	2.845	LUMNOZ	173.178	3.085	LUMNIT	150.184	2.837	LUMNIT	152.513	2.873	LUMNOZ	152.726	2.873
LUMNOZ	154.226	3.169	LUMPAN	154.57	3.203	LUMNOZ	173.443	2.822	LUMNOZ	156.86	3.186	LUMNOZ	155.536	3.342
LUMPAN	173.113	3.104	LUMPAN	150.561	2.864	LUMPAN	155.512	3.373	LUMPAN	157.142	3.218	LUMPAN	173.741	2.837
LUMPER	151.819	3.3	LUMPER	161.429	3.038	LUMPAN	152.804	2.889	LUMPER	175.327	2.999	LUMPER	153.229	3.203
LUMPER	153.909	2.996	LUMPER	167.684	2.972	LUMPER	159.257	3.297	LUMPER	173.125	2.994	LUMPER	160.206	3
LUMPER	177.752	3.069	LUMQIW	170.923	3.284	LUMPER	170.347	3.094	LUMPER	157.91	2.994	LUMPER	160.611	3.298
LUMQIW	165.454	3.199	LUMQIW	174.533	3.414	LUMQIW	153.227	3.113	LUMQIW	155.333	3.172	LUMQIW	151.514	3.112
LUMQIW	159.814	2.95	LUMQIW	159.63	3.261	LUMQIW	153.953	3.254	LUMQIW	156.054	3.06	LUMQIW	150.594	2.785
LUMQIW	159.898	2.907	LUMQIW	153.158	3.476	LUMQIW	169.678	2.937	LUMQIW	156.617	3.204	LUMQIW	157.398	3.289
LUMQIW	159.409	3.102	LUMQIW	150.38	2.772	LUMQIW	162.96	2.839	LUMQIW	155.677	3.359	LUMQIW	161.287	3.004
LUMQOC	151.661	2.956	LUMQOC	150.106	2.999	LUMQIW	157.171	3.284	LUMQIW	153.391	2.913	LUMQIW	167.82	2.806
LUMQOC	152.607	3.066	LUMQOC	151.793	3.09	LUMQOC	155.696	2.845	LUMQOC	154.129	3.067	LUMQOC	150.076	3.197
LUMQOC	155.248	2.933	LUMQOC	166.215	3.053	LUMQOC	161.694	3.226	LUMQOC	155.961	3.345	LUMQOC	150.381	2.919
LUMQOC	160.036	2.983	LUMQOC	170.156	2.891	LUMQOC	152.086	3.127	LUMQOC	172.218	2.858	LUMQOC	156.196	3.397
LUMQOC	155.005	3.393	LUMQOC	167.553	3.014	LUMQOC	157.579	3.43	LUMQOC	153.97	3.157	LUMQOC	166.309	2.937
LUMQOC	155.206	2.99	LUMQOC	156.111	2.936	LUMQOC	161.341	2.808	LUMQOC	170.694	3.119	LUMQOC	161.188	3.075
LUNPOC	167.674	3.263	LUNPOC	165.779	3.091	LUMQOC	175.183	3.178	LUMQOC	150.222	3.081	LUMQOC	153.58	3.438
LUNPOC	163.142	3.305	LUNPOC	166.412	3.457	LUNPOC	163.85	3.474	LUNPOC	159.957	3.195	LUNPOC	150.392	3.348
LUNTAP	150.906	2.835	LUNTAP	150.271	3.219	LUNQOD	153.715	3.48	LUNQOD	175.622	3.409	LUNTAP	162.724	3.12
LUNXIB01	154.556	3.238	LUNXIB01	150.959	3.369	LUNTAP	153.858	3.476	LUNTAP	157.132	2.78	LUNTAP	155.231	2.854
LUNXUR	170.029	3.245	LUNXUR	161.855	3.084	LUNXIB01	161.664	3.037	LUNXUR	152.317	3.313	LUNXUR	150.607	3.004
LUPBAD	156.773	3.327	LUPBAD	155.996	3.393	LUNXUR	174.467	3.243	LUNXUR	152.754	3.331	LUPBAD	170.796	3.34
LUPFOU	159.966	2.931	LUPFOU	165.915	2.949	LUPBAD	153.07	3.458	LUPCEH	151.113	2.975	LUPCEH	158.495	2.8
LUPGEL	160.159	3.171	LUPGEL	165.784	3.069	LUPFOU	174.421	3.268	LUPFOU	160.098	2.975	LUPFOU	164.921	2.984
LUPPIV03	156.742	3.325	LUPPIV03	159.231	3.116	LUPJOX	154.143	2.882	LUPJUB	153.644	3.251	LUPNUH	168.089	2.96
LUPWAX	162.968	3.456	LUPXED	158.667	3.176	LUPPIV04	157.679	3.344	LUPPIV04	159.218	3.136	LUPTIC	153.161	3.036
LUQMOC	160.388	2.848	LUQNIU	151.285	2.917	LUQHIQ	175.418	3.141	LUQHIQ	155.813	3.366	LUQMOC	159.072	2.848
LURBEF	165.205	3.028	LURBEF	163.744	2.881	LURBEF	150.154	3.061	LURBEF	160.972	3.239	LURBEF	152.849	3.061
LURNOB	170.597	2.783	LURNOB0	170.981	2.815	LURFEJ	161.691	3.052	LURFEJ	159.431	3.133	LURFEJ	166.766	3.365
LURSUO	151.756	3.167	LURXED	163.71	3.44	LURNOB0	170.428	2.771	LURSAS	157.926	3.366	LURSOI	170.993	3.378
LUSDUY	163.646	2.905	LUSDUY	153.821	3.221	LUSCUZ	155.284	3.268	LUSDOS	162.555	3.005	LUSDOS	159.076	3.455
LUSLAP	150.975	3.133	LUSLAP	155.275	2.968	LUSKES	160.103	3.038	LUSKIW	164.053	2.843	LUSKOC	166.959	3.024
LUSROG	163.431	2.93	LUSROG	155.136	2.974	LUSLET	169.849	3.002	LUSLUJ	173.308	3.453	LUSLUJ	157.856	3.436
LUSROG	154.26	2.945	LUSTOM	152.729	3.208	LUSROG	155.856	2.951	LUSROG	162.072	2.922	LUSROG	158.146	2.951
LUSYAZ	153.524	3.043	LUSYAZ	161.249	2.7	LUSYAZ	151.524	3.353	LUSYAZ	150.099	3.319	LUSYAZ	157.022	3.015
LUSYAZ	162.96	2.694	LUSYAZ	150.661	3.392	LUSYAZ	158.958	2.694	LUSYAZ	155.283	2.9	LUSYAZ	165.526	2.7
LUTHOZ	168.983	3.348	LUTHUF	164.013	2.958	LUTHOZ	153.415	3.178	LUTHOZ	166.75	2.961	LUTHOZ	151.128	3.086
LUTLIV	151.945	2.977	LUTLIV	165.548	2.828	LUTHUF	168.29	3.095	LUTHUF	160.968	3.243	LUTJAN	173.597	3.456
LUVHET	157.167	2.969	LUVHET	168.531	2.727	LUTLIV	163.726	3.363	LUTPIB	151.483	3.419	LUTPIB	166.733	3.297
LUVHOD	171.761	2.807	LUVHOD	150.748	2.782	LUVHIX	156.383	3.008	LUVHIX	170.153	2.761	LUVHOD	155.752	3.078
LUVJIY	154.174	2.996	LUVJIY	175.493	3.074	LUVHUJ	173.624	2.843	LUVHUJ	155.453	3.131	LUVHUJ	150.148	2.82
LUVJUL	169.253	3.009	LUVJUL	166.508	2.918	LUVJIY	163.771	3.165	LUVJUI	162.989	3.012	LUVJUL	172.519	3.294
LUVJUL	152.665	2.693	LUVJUL	157.042	2.972	LUVJUL	155.68	3.334	LUVJUL	150.216	2.781	LUVJUL	159.391	3.085
LUVQID	151.357	3.197	LUVQID	153.421	2.915	LUVJUL	155.931	2.824	LUVJUL	161.984	2.705	LUVQEB	153.135	3.035
LUWKEX	161.338	2.897	LUWMUL	152.957	3.043	LUVQID	152.028	3.361	LUVQID	157.309	3.339	LUWGAP	167.475	3.374
LUWRIE	159.741	3.299	LUWRUQ	166.81	2.983	LUWMUL	168.101	3.138	LUWMUL	150.533	3.038	LUWRIE	151.277	2.994
LUWYEI	161.473	3.195	LUXJOF	151.358	2.874	LUWVAD	167.379	2.927	LUWXAG	150.752	2.952	LUWXEK	170.924	2.925
LUXNAT	153.41	2.969	LUXNAT	153.56	2.953	LUXJOF	166.854	3.035	LUXJOF	151.199	3.468	LUXJUJ	161.239	2.999
LUYKIZ	155.41	2.973	LUYLEX	169.521	3.085	LUXWEK	156.75	3.336	LUYKIZ	167.805	2.943	LUYKIZ	152.248	3.463
LUYLEX	166.416	2.756	LUYMOH0	155.687	3.233	LUYLEX	150.702	3.034	LUYLEX	158.083	2.893	LUYLEX	175.685	2.912
LUZJIC	150.065	3.041	LUZJIC	152.524	3.168	LUYMOH0	178.24	2.822	LUYMOH0	155.385	3.249	LUZDOA	163.334	3.158
LUZXEM0	153.295	3.453	LUZXEM0	154.909	3.476	LUZJIC	152.904	3.226	LUZJIC	151.08	3.043	LUZKOG	163.013	3.119
MABFUU	156.4	3.38	MABLIL	169.431	3.162	LUZXOW	152.729	3.418	LUZXOW	150.962	3.079	MABBAX	156.285	3.289
MABQEO	151.051	3.259	MABQEO	157.061	3.426	MABQAI	178.134	3.082	MABQAI	150.056	3.359	MABQEO	164.754	3.142
MACNEN	150.56	3.14	MACNEN	150.542	3.293	MABQEO0	151.904	3.161	MABQEO0	158.81	3.329	MABQEO0	150.012	3.035
MACVAQ0	151.712	2.911	MACVAQ0	163.221	2.928	MACNEN	162.412	2.79	MACNEN	151.438	3.25	MACNEN	151.492	3.25
MACVAQ0	156.06	3.434	MACVAQ0	154.379	2.925	MACVAQ0	154.307	3.374	MACVAQ0	152.675	2.921	MACVAQ0	163.647	2.959
MACWUJ	165.832	3.226	MACWUJ	170.886	3.362	MACVAQ0	164.806	3.002	MACWUJ	156.423	2.845	MACWUJ	161.923	2.842
MACWUJ	172.997	2.696	MACWUJ	151.225	3.142	MACWUJ	156.947	3.264	MACWUJ	159.313	2.924	MACWUJ	161.972	3.487
MADKEK	157.377	3.066	MADKEK	168.22	3.019	MACZOG	158.497	3.046	MADBEZ	167.224	3.03	MADHUV	176.662	2.73
MADKEK	160.213	3.229	MADRIT	172.031	3.322	MADKEK	158.893	3.061	MADKEK	152.113	3.061	MADKEK	158.07	3.066
MAFCON	173.429	2.736	MAFCUT	154.997	3.104	MADWEW	154.539	3.404	MAFCON	157.536	3.104	MAFCON	160.283	3.096
MAFMAL	157.166	2.951	MAFMEM	167.833	3.097	MAFCUT	158.194	3.416	MAFHAE	171.15	3.436	MAFMAL	157.301	2.538
MAFMOZ0	161.129	2.607	MAFMOZ0	156.137	2.85	MAFMOZ	162.934	2.601	MAFMOZ	154.631	2.778	MAFMOZ	152.744	2.809
MAFNIU	164.428	2.6	MAFTAQ	161.774	3.368	MAFMOZ0	159.09	2.859	MAFNEQ	160.255	2.976	MAFNIU	159.99	2.6

MAFYOK	151.651	2.943	MAGDII	164.902	3.089	MAFTAQ	164.73	2.974	MAFTEU	167.976	3.08	MAFXOJ	155.964	2.919
MAGDOO	152.333	3.364	MAGDOO	159.254	3.277	MAGDII	161.052	3.433	MAGDII	166.936	3.264	MAGDOO	152.915	3.164
MAGNUE	165.788	2.883	MAGNUE	166.09	2.883	MAGDOO	168.411	3.478	MAGGUZ	164.324	2.865	MAGNUE	154.881	3.027
MAGPAM	151.008	2.986	MAGPAM	160.567	2.954	MAGPAM	157.861	3.343	MAGPAM	154.67	3.482	MAGPAM	153.984	3.407
MAGPAM	164.187	2.952	MAGQER	176.313	3.03	MAGPAM	153.814	3.093	MAGPAM	151.246	3.136	MAGPAM	152.693	3.215
MAGRAQ	168.994	2.698	MAGRET	151.341	3.019	MAGRAP	160.489	3.191	MAGRAP	165.479	2.96	MAGRAP	176.038	3.191
MAGROE	166.776	3.132	MAGYAX	170.859	3.482	MAGREU	172.414	3.418	MAGRIY	154.562	3.254	MAGRIY	159.724	2.976
MAHBUU	162.752	3.341	MAHFEI	167.615	3.165	MAHBEE	168.106	2.979	MAHBII	151.548	3.112	MAHBII	169.214	3.403
MAHNUG	158.449	2.734	MAHNUG	163.582	3.062	MAHKEP	154.386	3.307	MAHKEP	166.356	3.073	MAHNUG	156.94	2.847
MAHSOE	153.392	2.75	MAJFOW	156.275	3.498	MAHNUG	153.563	2.734	MAHQEV	173.216	3.253	MAHSOE	152.425	2.767
MAJSUO	160.23	2.813	MAJWUT	160.76	3.003	MAJLOZ	167.656	3.307	MAJLOZ	154.308	2.982	MAJSUO	164.176	2.82
MAKCOR	150.357	2.919	MAKCUA	152.968	3.362	MAKBEJ	160.464	2.919	MAKCEK	165.142	2.996	MAKCEK	165.533	2.864
MAKDAH	163.49	3.12	MAKJUD	152.088	3.115	MAKCUA	159.913	2.988	MAKCUA	155.034	2.907	MAKCUA	153.708	2.926
MAKLUI	154.53	3.145	MAKLUI	153.76	3.485	MAKLUI	151.464	3.063	MAKLUI	171.412	2.793	MAKLUI	165.245	3.177
MAKLUI	150.066	3.006	MAKLUI	172.712	2.793	MAKLUI	152.498	3.479	MAKLUI	161.918	3.155	MAKLUI	161.632	3.386
MAKYAZ0	162.751	3.002	MALDEK	154.459	3.251	MAKQIZ	151.572	2.838	MAKQOF	169.685	3.139	MAKYAZ	151.719	3.427
MALDEK	155.932	3.056	MALDEK0	162.417	3.424	MALDEK	158.04	3.039	MALDEK	156.728	3.036	MALDEK	160.375	3.381
MALDEK0	155.852	3.113	MALDEK0	160.552	3.417	MALDEK0	156.212	3.12	MALDEK0	153.265	3.252	MALDEK0	157.645	3.053
MALDEK0	155.81	3.092	MALDEK0	160.895	3.389	MALDEK0	156.435	3.088	MALDEK0	154.814	3.267	MALDEK0	158.273	3.056
MALDEK0	156.178	3.046	MALDEK0	160.476	3.37	MALDEK0	155.846	3.065	MALDEK0	154.8	3.25	MALDEK0	157.901	3.044
MALDEK0	158.321	3.03	MALDEK0	156.062	3.018	MALDEK0	155.789	3.044	MALDEK0	150.324	3.48	MALDEK0	154.794	3.23
MALDIO	159.497	3.489	MALDIO	163.914	3.205	MALDIO	168.812	3.342	MALDIO	163.145	3.413	MALDIO	165.305	3.495
MALDIO	176.427	3.413	MALDIO	167.926	2.734	MALDIO	151.885	3.228	MALDIO	159.746	2.895	MALDIO	156.823	3.239
MALKER	164.102	2.915	MALKER	155.122	2.915	MALFAK	163.249	3.291	MALKER	165.73	2.969	MALKER	169.716	3.471
MALKER	158.169	3.046	MALKER	153.653	2.879	MALKER	159.395	3.013	MALKER	152.191	3.296	MALKER	165.672	3.433
MALPIY	164.707	3.216	MALPIY	160.53	3.321	MALPIY	157.213	3.245	MALPIY	151.578	3.195	MALPIY	162.031	3.315
MALPIY	157.304	3.348	MALPIY	155.58	2.884	MALPIY	150.668	3.349	MALPIY	156.171	3.239	MALPIY	157.452	3.225
MAMFUB	160.241	3.359	MAMGAL	162.029	3.174	MALPIY	172.997	3.367	MALPIY	166.733	3.3	MALPIY	157.45	3.36
MAMGIT	164.672	2.882	MAMGIT	151.296	2.882	MAMGAL	157.14	2.712	MAMGAL	158.774	2.911	MAMGEQ	168.727	3.207
MAMGIT	174.749	3.302	MAMHIV	163.081	3.266	MAMGIT	160.693	3.004	MAMGIT	155.077	3.278	MAMGIT	157.642	3.278
MAMKUI	163.462	3.491	MAMKUI	174.782	3.493	MAMHIV	170.702	3.266	MAMKUI	155.844	3.195	MAMKUI	165.939	2.94
MAMKUI	167.106	3.099	MAMKUI	150.208	3.174	MAMKUI	166.441	2.987	MAMKUI	166.423	3.423	MAMKUI	157.771	2.682
MAMLAP	166.97	3.35	MAMLAP	168.147	2.714	MAMKUI	153.258	3.265	MAMKUI	160.386	3.044	MAMKUI	174.016	3.315
MAMLAP	166.832	3.229	MAMLAP	164.728	2.906	MAMLAP	151.97	2.812	MAMLAP	151.095	3.291	MAMLAP	151.11	3.268
MAMLEV	161.958	2.825	MAMTOL	155.653	3.178	MAMLAP	168.929	3.33	MAMLEV	158.363	3.437	MAMLEV	161.183	3.024
MAMTOL	162.612	3.488	MAMTUR	156.374	3.186	MAMTOL	156.109	3.15	MAMTOL	157.171	2.926	MAMTOL	151.908	2.863
MAMVOO	166.705	3.077	MAMWAZ	163.697	3.037	MAMTUR	164.18	3.207	MAMTUR	160.316	2.919	MAMTUR	162.749	3.468
MAMWIH	152.897	3.464	MAMWIH	155.687	3.046	MAMWAZ	152.438	3.02	MAMWAZ	157.981	3.164	MAMWAZ	162.468	3.232
MAMWOP	156.208	2.984	MANHIW	156.824	3.277	MAMWIH	151.653	2.928	MAMWIH	158.291	3.11	MAMWOP	165.911	3.487
MANLIY	169.19	2.797	MANMIB	157.626	3.184	MANHOZ	157.271	3.412	MANHOZ	158.824	3.003	MANJOB0	151.364	3.463
MANRAU	154.243	3.417	MANRAU	166.447	3.103	MANMIZ	165.624	3.482	MANMIZ	153.044	3.482	MANMIZ	177.31	2.996
MANTAA	169.848	2.785	MANVOM	150.394	2.853	MANRAU	159.941	3.081	MANRAU	172.387	3.154	MANSON	169.614	2.788
MANVOM	150.163	3.465	MANYOP	150.14	3.33	MANVOM	158.519	2.982	MANVOM	153.557	3.151	MANVOM	155.142	2.757
MANYOP	161.049	2.936	MANYOP	168.727	3.172	MANYOP	156.347	3.49	MANYOP	155.542	3.147	MANYOP	155.609	2.681
MANYOP	158.859	3.147	MANYOP	163.055	3.351	MANYOP	163.006	3.417	MANYOP	165.473	3.109	MANYOP	159.315	2.979
MANYOP0	155.342	3.152	MANYOP0	161.429	2.93	MANYOP	159.42	2.882	MANYOP0	162.877	3.353	MANYOP0	159.328	2.88
MANYOP0	165.644	3.103	MANYOP0	159.38	2.977	MANYOP0	168.595	3.165	MANYOP0	155.179	2.681	MANYOP0	162.931	3.419
MANYOP0	160.359	2.944	MANYOP0	158.55	2.817	MANYOP0	158.794	3.152	MANYOP0	156.56	3.496	MANYOP0	161.794	3.371
MANYOP0	162.869	2.883	MANYOP0	165.3	3.022	MANYOP0	157.073	2.651	MANYOP0	163.778	3.311	MANYOP0	150.896	3.329
MANYUV	157.379	3.467	MANYUV	152.096	3.335	MANYOP0	170.367	3.098	MANYOP0	158.538	3.128	MANYOP0	153.635	3.128
MANZAC	159.782	3.06	MANZAC	163.841	3.126	MANYUV	150.183	3.308	MANYUV	162.953	3.194	MANZAC	172.583	3.482
MANZAC	161.946	3.024	MANZAC	163.587	2.889	MANZAC	169.386	3.099	MANZAC	151.831	3.5	MANZAC	157.516	3.031
MANZEG	171.439	3.054	MANZIK	157.89	3.22	MANZEG	166.595	2.811	MANZEG	161.791	2.918	MANZEG	152.048	3.289
MAPHOD	173.889	3.38	MAPHOD	156.795	3.044	MANZIK	150.783	2.859	MAOPHS	157.147	3.447	MAPHOD	156.648	2.873
MAPJIZ	159.798	2.978	MAPLAR	153.225	2.703	MAPHUJ	151.796	3.079	MAPJAO	168.505	2.961	MAPJIZ	158.723	3.016
MAPLAR	166.531	3.238	MAPLAR	153.915	3.31	MAPLAR	165.606	3.404	MAPLAR	154.302	3	MAPLAR	150.121	3.251
MAPLAR	161.425	2.877	MAPLAR	163.724	3.135	MAPLAR	162.143	3.496	MAPLAR	161.804	3.242	MAPLAR	167.525	2.924
MAPLAR	167.517	2.924	MAPLAR0	162.08	2.897	MAPLAR	158.219	2.967	MAPLAR	159.525	3.171	MAPLAR	161.038	3.374
MAPLAR0	164.985	3.42	MAPLAR0	153.323	3.336	MAPLAR0	154.258	2.7	MAPLAR0	166.832	3.239	MAPLAR0	163.625	3.135
MAPLAR0	167.285	2.936	MAPLAR0	160.585	3.359	MAPLAR0	158.088	2.959	MAPLAR0	154.511	3.005	MAPLAR0	162.555	3.245
MAPSOP	161.586	3.19	MAPSOP	167.642	2.806	MAPLAR0	167.867	2.936	MAPLAR0	158.284	3.17	MAPPAY	169.098	3.1
MAPSOP	150.613	2.755	MAPSOP	173.473	3.201	MAPSOP	163.605	3.149	MAPSOP	162.872	3.149	MAPSOP	164.716	3.443
MAPWIJ	167.98	3.102	MAPWIJ	172.466	2.849	MAPSOP	175.32	3.394	MAPTEE	166.024	3.269	MAPWIJ	154.42	2.875
MAPWOR	157.79	2.935	MAPWOR	153.627	2.993	MAPWIJ	151.305	3.027	MAPWOR	154.141	2.968	MAPWOR	160.461	3.339
MAPXUY	163.721	2.935	MAPYAF	161.558	3.238	MAPXUY	162.701	3.144	MAPXUY	166.818	2.98	MAPXUY	150.346	3.441
MAPYOT	153.867	3.376	MAPYOT	152.488	3.33	MAPYAF	168.449	3.15	MAPYEJ	151.229	3.002	MAPYIN	153.124	2.928
MAPYOT	162.652	3.034	MAPYOT	162.305	3.319	MAPYOT	157.286	2.955	MAPYOT	168.469	3.419	MAPYOT	164.355	3.306
MAQKIB	171.828	2.894	MAQKIB	170.218	2.919	MAPYOT	150.03	3.011	MAPYOT	154.006	3.421	MAPYOT	156.012	3.179
MAQVOS	166.558	2.866	MAQWIK	169.101	3.088	MAQKIB	152.314	2.781	MAQKIB	151.365	3.285	MAQVOS	158.249	2.963
MARGUL0	163.886	3.465	MARJUL	158.226	3.298	MARGOF	155.886	3.313	MARGUL	163.844	3.459	MARGUL0	163.887	3.457
MARSAB	176.64	2.965	MARSAB	169.021	3.048	MARKAS	152.891	2.673	MARLUQ	180	2.532	MARQOL	150.671	2.813
MARXEJ	150.707	3.064	MASSAB	158.072	2.884	MARTIJ	154.333	3.202	MARTIJ	151.995	3.202	MARXEJ	157.27	3.145
MATDAQ0	156.286	2.719	MATDAQ0	166.965	3.334	MASXIO	152.445	3.117	MASXIO	154.359	3.112	MATDAQ	154.66	3.491
MATHEX	153.934	2.747	MATHEX	153.321	3.237	MATFUL	153.07	3.225	MATFUL	153.164	2.742	MATGEW	170.517	3.044
MATSEG	163.548	3.126	MATSIK05	155.496	3.055	MATNEE	150.199	3.433	MATNEE	154.843	3.273	MATNEE	157.816	2.605
MATSIK05	156.821	2.915	MATSIK05	152.896	3.01	MATSIK05	151.589	3.245	MATSIK05	150.645	2.867	MATSIK05	150.711	3.286
MATSIK05	164.695	2.825	MATSIK05	157.407	2.896	MATSIK05	152.937	3.445	MATSIK05	160.924	2.781	MATSIK05	152.895	3.13
MAVKUP	163.348	2.878	MAVKUP	161.727	2.936	MATSIK05	150.414	3.286	MATSIK05	155.074	3.018	MAVBOB	152.92	3.04
MAVMIG	155.977	3.266	MAVQAC	171.22	3.392	MAVKUT	161.074	3.425	MAVKUT	154.192	3.344	MAVKUT	160.636	2.89

MAVQOQ	176.674	2.946	MAVQOQ	167.201	2.903	MAVQAC	152.255	3.099	MAVQOQ	151.712	2.641	MAVQOQ	151.246	3.237
MAWDOH	163.735	2.871	MAWDOH	160.039	3.302	MAVQOQ	151.249	3.071	MAVRAD	167.029	3.033	MAVRAD	170.309	3.317
MAWDOH	154.328	2.944	MAWDOH	150.6	3.364	MAWDOH	151.422	2.874	MAWDOH	158.606	3.02	MAWDOH	158.764	3.073
MAWREL	154.499	3.081	MAWREL	153.668	2.909	MAWMEC	154.059	3.433	MAWREL	167.215	2.942	MAWREL	153.486	3.231
MAXGOK	160.989	3.403	MAXGOK	173.375	3.081	MAWREL	153.328	3.127	MAXGAX	156.927	3.482	MAXGAX	158.416	3.22
MAXGOK	165.621	2.938	MAXGOK	159.041	3.258	MAXGOK	177.416	2.787	MAXGOK	153.964	3.482	MAXGOK	163.874	2.817
MAXLIJ	156.224	3.072	MAXLIJ	156.993	3.212	MAXGOK	152.192	2.702	MAXLIJ	174.057	3.411	MAXLIJ	172.74	3.33
MAXWOY	174.906	3.262	MAXXAM	165.235	2.926	MAXQIO	150.561	3.132	MAXWEP	163.172	2.706	MAXWEP	151.153	3.172
MAYMEI	150.182	3.427	MAYVIS	157.645	3.105	MAYHAV	165.785	3.368	MAYJEC	169.929	3.374	MAYKUS	173.948	3.262
MAZZIX	155.252	3.413	MAZZIX	155.146	3.302	MAZLEF	155.716	3.421	MAZQIP	160.823	2.85	MAZQIP	154.436	2.85
MEBBAX	158.804	2.786	MEBBAX0	160.291	2.733	MBFYPT	151.098	3.172	MBFYPT	169.627	3.318	MEBBAX	164.066	2.567
MEBBIG	153.164	3.288	MEBBIG	160.813	2.668	MEBBAX0	164.673	2.548	MEBBAX0	160.577	3.423	MEBBAX0	162.859	3.423
MEBBOL0	160.807	3.234	MEBBOL0	167.405	2.827	MEBBOL0	162.08	3.011	MEBBOL0	155.842	2.912	MEBBOL0	172.518	3.109
MEBBOL0	151.766	3.15	MEBBOL0	178.476	3.217	MEBBOL0	166.238	3.311	MEBBOL0	155	3.321	MEBBOL0	163.353	3.051
MEBBOL0	156.105	3.219	MEBBOL0	166.027	2.85	MEBBOL0	154.251	3.022	MEBBOL0	151.061	3.064	MEBBOL0	151.396	3.045
MEBCAX	161.443	3.11	MEBCAX	173.761	2.807	MEBBOL0	159.88	2.929	MEBBOL0	165.184	3.028	MEBBOL0	159.197	3.101
MEBHIK	162.36	2.73	MEBQIU	155.383	3.22	MEBCAZ	160.097	3.449	MEBCON	159.887	3.42	MEBHIK	153.426	2.73
MEBQUG	166.387	3.292	MEBXID	170.157	3.36	MEBQIU	165.419	3.22	MEBQIU	167.252	3.124	MEBQUG	156.059	3.125
MEBXID04	169.901	3.388	MECFIJ	172.146	3.13	MEBXID01	169.83	3.377	MEBXID02	170.572	3.399	MEBXID03	170.621	3.375
MECPOA	157.144	3.235	MECPOA	161.49	3.197	MECFIJ	155.108	3.349	MECHOU	154.503	2.835	MECJEN	169.372	3.385
MECVIB	151.843	2.54	MECXAT	156.119	3.1	MECTUM	173.206	3.225	MECTUM	162.743	3.098	MECTUM	162.641	2.797
MECXAT	152.371	3.026	MECXUN	150.134	2.902	MECXAT	151.04	2.859	MECXAT	173.561	3.103	MECXAT	163.47	2.898
MECXUN	150.738	3.079	MECXUN	151.027	2.997	MECXUN	164.537	2.955	MECXUN	165.132	3.371	MECXUN	155.033	2.997
MEDPAP	160.203	3.137	MEDPAP	175.271	3.137	MECXUN	160.87	2.902	MEDCEF	156.546	3.447	MEDJEO	155.136	3.253
MEDQUL	156.578	3.324	MEDZAA	166.028	3.112	MEDPIX	150.759	3.125	MEDPIX	150.773	3.489	MEDQUL	167.492	3.089
MEFMET	171.018	2.913	MEFMET	162.465	3.094	MEFHOW	160.873	2.994	MEFMAP	153.586	3.238	MEFMET	163.249	3.04
MEFREY	169.76	3.072	MEFVAV	154.254	2.897	MEFMET	163.651	2.943	MEFMET	153.35	2.961	MEFNIU	160.651	3.462
MEFVEZ	158.617	3.336	MEFWAW	150.395	3.09	MEFVEZ	165.255	3.033	MEFVEZ	151.914	3.336	MEFVEZ	171.415	3.247
MEGHOV	160.954	3.322	MEGMEQ	157.288	3.296	MEFYUU	157.733	2.713	MEGDEJ	159.337	3.478	MEGHOV	154.402	3.321
MEGXAB	160.697	3.362	MEGXAB	152.798	3.205	MEGMEQ	171.336	3.374	MEGMIW	173.372	3.468	MEGXAB	160.01	3.291
MEGXEF	161.47	3.209	MEGXEF	155.776	3.033	MEGXAB	151.875	3.483	MEGXEF	169.024	2.842	MEGXEF	154.984	2.895
MEGXOP	156.14	3.428	MEGXOP	177.665	2.925	MEGXEF	157.132	3.107	MEGXEF	152.186	3.107	MEGXEF	156.067	3.112
MEGXOP	154.274	2.823	MEGXOP	175.081	2.918	MEGXOP	161.622	3.118	MEGXOP	163.798	3.487	MEGXOP	160.32	3.384
MEGZOR	172.582	3.368	MEHCOS	159.572	3.203	MEGZOR	155.734	2.935	MEGZOR	157.276	3.36	MEGZOR	173.057	3.123
MEHCOS	162.916	2.572	MEHCOS	153.471	2.995	MEHCOS	151.505	2.904	MEHCOS	155.96	2.995	MEHCOS	153.791	3.203
MEHQUM	157.071	3.361	MEHSOI	151.019	3.352	MEHKAP	157.572	3.302	MEHPIB	166.838	2.995	MEHQEW	151.497	3.055
MEHTAV	153.197	3.332	MEHTOJ	167.148	3.003	MEHSOI	157.631	3.119	MEHTAV	174.822	2.874	MEHTAV	150.191	3.023
MEJJET	166	2.94	MEJJET	159.364	2.907	MEHTOJ	170.262	3.003	MEJBAI	157.113	3.407	MEJJET	170.192	3.324
MEJKER	170.64	3.134	MEJKER	168.111	3.134	MEJKER	152.306	3.263	MEJKER	165.599	3.182	MEJKER	161.082	3.311
MEJMAR	157.823	3.479	MEJMEX	158.363	3.197	MEJMAR	154.089	3.108	MEJMAR	156.355	2.793	MEJMAR	175.041	2.793
MEJTAX	159.57	3.14	MEJVAZ	157.571	3.411	MEJMEX	161.879	3.124	MEJMEX	150.253	3.208	MEJSIG	150.264	3.278
MEJWOQ	162.295	3.434	MEKBOT	151.422	3.094	MEJWAC	151.448	2.875	MEJWAC	155.216	2.858	MEJWOQ	164.811	2.892
MEKGIS	162.295	2.857	MEKGIS	151.821	3.085	MEKCIO	156.759	3.084	MEKCIO	153.933	3.169	MEKGIS	155.551	3.419
MEKGOZ	163.422	3.143	MEKGOZ	152.9	3.049	MEKGIS	178.611	3.051	MEKGIS	174.192	2.794	MEKGIS	153.785	2.997
MEKHIT	160.771	3.164	MEKHOZ	163.92	3.011	MEKHIT	161.168	3.016	MEKHIT	178.231	3.417	MEKHIT	155.923	2.693
MEKKIZ	166.543	3.046	MEKKIZ	154.27	2.956	MEKHOZ	157.656	2.902	MEKHOZ	152.584	3.051	MEKHOZ	153.656	3.051
MEKLAP	150.913	3.421	MEKLAP	165.73	2.76	MEKKIZ	152.214	2.954	MEKKIZ	171.639	3.017	MEKLAP	150.914	2.953
MEKLAP	156.199	2.786	MEKLAP	151.201	2.91	MEKLAP	159.354	3.346	MEKLAP	163.181	3.146	MEKLAP	150.681	2.821
MELLAU01	176.956	2.992	MELLAU01	177.194	2.992	MEKMOF	167.176	3.182	MELLAU	176.98	3.01	MELLAU	177.059	3.01
MELMOF	155.79	2.736	MELNOJ	176.068	3.134	MELMOF	156.461	2.987	MELMOF	152.311	2.736	MELMOF	154.377	2.886
MELNOJ	169.797	2.869	MELNOJ	155.92	2.682	MELNOJ	169.229	3.483	MELNOJ	162.594	3.333	MELNOJ	150.962	2.956
MELNUP	155.62	2.683	MELNUP	169.385	2.881	MELNUP	177.188	3.123	MELNUP	168.779	3.458	MELNUP	161.85	3.34
MELPAX	169.725	2.874	MELPAX	150.531	2.956	MELPAX	169.038	3.48	MELPAX	162.196	3.33	MELPAX	176.655	3.131
MELPEB	176.195	3.469	MELPIF	167.085	3.092	MELPAX	155.647	2.685	MELPEB	168.142	3.08	MELPEB	162.952	2.988
MELPOL	161.083	3.225	MELPOL	166.017	3.494	MELPIF	163.465	2.997	MELPIF	175.919	3.449	MELPOL	150.871	3.233
MELTIG	153.591	3.14	MELTIG	153.952	3.109	MELPOL	167.06	3.238	MELTIG	150.719	3.143	MELTIG	157.931	3.095
MELWEI	157.076	3.349	MELWEI	150.02	3.23	MELTIG	165.846	3.109	MELVAA	158.804	2.794	MELWEI	160.031	3.339
MELWEI	165.235	3.007	MELWEI	156.876	3.455	MELWEI	171.695	3.158	MELWEI	156.141	3.273	MELWEI	155.682	3.246
MELWUY	163.955	2.839	MELWUY	173.52	2.839	MELWEI	167.991	3.097	MELWEI	151.017	3.234	MELWEI	162.729	3.3
MELWUY	152.291	3.318	MELWUY	167.844	3.289	MELWUY	152.925	3.315	MELWUY	175.576	3.481	MELWUY	151.777	3.144
MELWUY	177.21	3.173	MELWUY	150.081	2.83	MELWUY	153.139	3.113	MELWUY	151.362	3.303	MELWUY	155.094	2.997
MELXAF	155.051	3.093	MELXAF	164.388	2.809	MELXAF	179.454	3.401	MELXAF	158.668	2.945	MELXAF	165.79	3.252
MELXAF	151.054	3.092	MELXAF	175.405	3.229	MELXAF	150.825	3.363	MELXAF	152.825	3.362	MELXAF	153.279	3.276
MELYIO	167.394	2.951	MELYIO	154.216	2.851	MELXAF	150.743	2.817	MELXAF	173.56	2.809	MELYIO	161.36	2.951
MELYUA	169.852	2.736	MELYUB	164.612	3.108	MELYUA	154.209	3.002	MELYUA	173.767	3.429	MELYUA	173.739	3.335
MEMGOA	169.171	3.157	MEMNEY	169.581	3.204	MELZAI	159.567	3.204	MELZAI	160.392	3.287	MELZUC	152.961	3.437
MEMPAW	161.516	2.879	MEMPAW	150.596	3.074	MEMNEY	155.002	3.201	MEMNUN	168.162	3.181	MEMNUN	173.334	3.143
MEMPIE	154.578	3.049	MEMPIE	158.7	3.468	MEMPAW	150.652	2.865	MEMPED	151.122	2.792	MEMPIE	156.995	3.099
MEMPOK	163.76	2.769	MEMQAA	150.259	2.94	MEMPIH	150.418	2.806	MEMPOK	168.664	3.119	MEMPOK	166.969	2.788
MEMTAA0	164.06	2.942	MEMTAA0	169.389	3.166	MEMQAX	151.156	2.816	MEMQAX	165.324	3.26	MEMQAX	160.525	3.373
MENCOA	155.453	3.118	MENFOD	160.761	3.178	MEMTAA0	150.381	2.796	MENBEP	153.768	2.758	MENCOA	170.671	3.218
MENGIZ	152.1	3.165	MENGOF	168.669	3.156	MENFOD	154.192	3.334	MENGEV	159.736	3.1	MENGIZ	172.471	3.457
MENRUV	166.564	3.23	MENRUV	168.008	2.755	MENMOL	150.037	3.376	MENRIJ	170.799	3.078	MENRIJ	156.186	3.48
MENRUV	159.579	3.212	MENSUX	177.283	3.242	MENRUV	155.391	2.833	MENRUV	150.863	2.865	MENRUV	162.099	2.694
MENTIM	150.025	2.988	MENYIQ	163.369	3.092	MENTEI	155.364	3.367	MENTEI	173.766	3.367	MENTEI	156.405	3.125
MEPKAU	156.134	3.019	MEPKIC	160.665	2.93	MENYIQ	162.723	3.459	MEPKAU	162.104	3.166	MEPKAU	173.296	2.991
MEPPAB	161.873	2.966	MEPPEF	156.559	3.459	MEPKIC	170.4	2.88	MEPPAB	150.703	3.404	MEPPAB	172.998	3.081
MEQTEK	152.242	2.693	MERCAO	164.813	3.238	MEPYEP	164.702	2.881	MEQTAE	156.574	2.87	MEQTEK	163.723	2.693
MERFIZ	161.461	2.848	MERFIZ	156.082	3.473	MERCAO	157.781	3.348	MERCAO	150.971	3.116	MERFIZ	161.59	2.798

MERFIZ	158.065	2.803	MERFIZ	152.276	2.946	MERFIZ	151.286	2.941	MERFIZ	172.282	3.3	MERFIZ	156.339	2.919
MERQIN	151.56	3.044	MERQIN	163.61	3.182	MERFIZ	152.927	2.848	MERQEG	170.523	3.016	MERQIN	153.617	2.879
MERQIN	153.394	3.231	MERQIN	158.669	3.074	MERQIN	157.148	2.752	MERQIN	150.022	3.32	MERQIN	155.952	3.272
MERYAJ	151.127	3.374	MESBIZ	172.219	2.975	MERQIN	166.375	2.91	MERQIN	165.473	3.294	MERYAJ	158.699	3.03
METHEB	160.842	2.899	METJAX	158.783	3.391	MESQAG	159.591	2.888	METGUR	155.81	3.358	METHAV	164.15	2.975
MEVJIJ	152.544	3.164	MEVJIJ	166.025	3.043	METKAY	154.532	3.488	METYIW	150.978	3.107	MEVJIJ	158.163	2.871
MEVJIJ	159.811	3.273	MEVJIJ	160.529	3.191	MEVJIJ	150.366	3.245	MEVJIJ	169.84	2.927	MEVJIJ	157.051	3.151
MEVKIK	165.584	3.115	MEVKOQ	164.799	3.392	MEVKEG	155.587	3.298	MEVKEG	164.707	3.076	MEVKIK	155.285	3.425
MEWROX	176.889	2.763	MEWTAL	169.328	3.293	MEVKOQ	161.747	3.462	MEWNUX	162.39	2.978	MEWNUX	166.651	2.978
MEWYIW	157.03	3.353	MEWYIW	170.167	3.32	MEWTIU	161.309	3.31	MEWTIU	165.11	3.166	MEWVER	153.598	3.34
MEWZOF	156.075	3.115	MEWZOF	159.893	3.27	MEWYIW	167.609	2.94	MEWZIZ	160.128	2.917	MEWZOF	160.611	2.817
MEXQEN	159.865	3.239	MEXXEW	154.24	3.385	MEXBOI	155.587	3.2	MEXBOI	178.772	3.212	MEXHIJ	172.938	2.859
MEYTES	160.226	3.288	MEYZUN	156.282	2.929	MEXZUN	158.769	3.25	MEYDAZ	162.419	3.428	MEYSUG	150.844	2.969
MEYZUN	156.999	3.196	MEYZUN	154.158	3.453	MEYZUN	166.757	2.953	MEYZUN	159.712	3.186	MEYZUN	170.979	3.167
MEZLEK	168.09	2.883	MEZLEK	166.918	3.364	MEYZUN	156.983	3.412	MEYZUN	166.592	3.483	MEZLEK	154.927	2.927
MEZRIW	152.963	3.496	MEZVOG	155.35	2.899	MEZLEK	157.682	2.792	MEZLEK	166.007	3.498	MEZNUE	159.884	2.9
MEZYEX	158.884	2.981	MEZYEX	150.697	2.87	MEZVUL	151.049	3.334	MEZYEX	161.515	2.988	MEZYEX	158.054	2.717
MHCOFE	154.037	3.272	MIBNUK	171.364	3.456	MEZYEX	152.145	3.331	MEZYEX	153.279	2.73	MHCOFE	168.321	2.871
MIBQUM	174.077	3.233	MIBQUM	169.376	3.127	MIBQUM	176.823	2.874	MIBQUM	158.629	2.99	MIBQUM	158.363	2.852
MIBRIB	154.987	3.226	MIBRIB	163.863	3.226	MIBQUM	170.638	2.798	MIBQUM	171.222	2.798	MIBQUM	163.961	2.852
MIBRIB	157.77	3.328	MIBRIB	157.638	3.447	MIBRIB	157.834	3.428	MIBRIB	167.619	3.263	MIBRIB	166.462	3.197
MIBRIB	155.349	3.447	MICJOY	163.688	3.496	MIBRIB	153.368	3.33	MIBRIB	172.328	3.091	MIBRIB	153.522	3.197
MICPEU	155.33	3.478	MICPIY	168.367	2.798	MICMER	158.742	2.857	MICMER	161.964	2.68	MICNIW	150.521	3.378
MICPUK	154.833	2.699	MICQAR	154.447	3.259	MICPIY	167.096	3.446	MICPIY	166.733	2.659	MICPUK	157.619	3.444
MICROF	171.45	3.059	MICRUL	171.561	3.053	MICROF	158.183	2.896	MICROF	176.042	2.736	MICROF	163.188	3.154
MICRUL	150.04	3.093	MICRUL	162.383	3.293	MICRUL	160.931	3.426	MICRUL	151.659	3.412	MICRUL	174.85	3.072
MICSAS	151.122	2.77	MICSAS	162.868	3.076	MICSAS	171.365	2.944	MICSAS	158.336	3.478	MICSAS	152.7	3.469
MICSIE	151.172	3.119	MICSIE	158.526	2.796	MICSAS	167.434	3.47	MICSAS	151.419	3.363	MICSAS	151.325	3.495
MICSOH	153.361	2.831	MICSOH	152.97	2.996	MICSIE	169.236	2.804	MICSOH	158.995	2.831	MICSOH	154.386	2.939
MICWEB	168.889	3.218	MICWEB	158.651	3.129	MICSOH	150.456	2.823	MICSOH	157.301	2.939	MICWEB	171.444	3.205
MICWEB	152.081	3.404	MICWEB	152.361	3.44	MICWEB	156.684	3.206	MICWEB	154.423	3.45	MICWEB	158.385	3.101
MICWIF	158.751	3.007	MICWIF	158.131	3.374	MICWEE	173.168	2.825	MICWEE0	173.126	2.821	MICWIF	158.024	3.369
MICXUV	168.309	3.395	MIDBAE	169.502	3.099	MICWIF	168.229	3.284	MICWII	165.177	2.907	MICWII01	165.317	2.908
MIDDEK	150.294	2.897	MIDDEK	159.864	3.489	MIDCAE	165.035	3.298	MIDDEK	152.099	3.154	MIDDEK	159.032	2.825
MIDDEK	164.472	2.856	MIDDEK	170.633	2.904	MIDDEK	160.613	3.045	MIDDEK	153.729	2.966	MIDDEK	161.686	3.087
MIDDEK	157.148	3.212	MIDDEK	155.197	2.987	MIDDEK	167.685	3.144	MIDDEK	156.613	3.347	MIDDEK	166.255	3.434
MIDJAK01	161.316	3.228	MIDJAK01	165.207	2.908	MIDDEK	150.222	3.434	MIDDEL	150.867	3.326	MIDHOW	163.695	3.101
MIDJAK01	151.701	3.114	MIDJAK01	159.798	3.085	MIDJAK01	155.151	3.439	MIDJAK01	165.035	3.277	MIDJAK01	163.512	3.141
MIDJAN	168.194	2.794	MIDLUK	169.664	3.041	MIDJAK01	154.107	3.491	MIDJAK01	170.802	3.04	MIDJAK01	161.703	3.023
MIDPEW	167.138	3.232	MIDXIH	170.592	3.424	MIDNIX	157.284	2.95	MIDNUK	171.765	3.21	MIDPAS	163.027	3.198
MIFQAU	161.626	3.468	MIFQAU	165.649	2.932	MIDXIH01	170.419	3.327	MIFDIP	154.944	3.077	MIFDIP	159.443	2.817
MIFQAU	168.66	3.39	MIFSUS02	162.058	3.265	MIFQAU	150.798	2.982	MIFQAU	154.718	3.468	MIFQAU	151.737	3.469
MIGCAJ	161.297	3.423	MIGCAJ	166.21	2.852	MIFSUS02	150.31	3.21	MIGBOW	152.909	3.279	MIGCAJ	153.048	2.638
MIGCEN	163.988	2.543	MIGGIT	168.792	3.484	MIGCAJ	164.398	2.852	MIGCAJ	160.204	2.826	MIGCEN	156.047	3.1
MIGKIY	151.631	2.907	MIGKIY	151.784	3.028	MIGGIT	162.41	3.421	MIGGIU	168.368	3.098	MIGKIY	169.823	3.054
MIGKIY03	170.011	3.058	MIGKIY03	151.481	3.025	MIGKIY02	169.79	3.054	MIGKIY02	151.678	3.029	MIGKIY02	151.599	2.906
MIGKIY07	151.633	2.907	MIGKIY08	170.018	3.058	MIGKIY03	151.619	2.907	MIGKIY07	170.024	3.058	MIGKIY07	151.474	3.025
MIGKIY09	151.486	3.024	MIGKIY09	151.642	2.907	MIGKIY08	151.473	3.025	MIGKIY08	151.632	2.907	MIGKIY09	170.019	3.059
MIGKIY11	170.021	3.058	MIGKIY11	151.472	3.026	MIGKIY10	170.025	3.059	MIGKIY10	151.474	3.026	MIGKIY10	151.645	2.907
MIGKIY12	151.64	2.907	MIGKIY13	170.008	3.058	MIGKIY11	151.632	2.907	MIGKIY12	170.004	3.058	MIGKIY12	151.472	3.024
MIGKIY14	151.47	3.025	MIGKIY14	151.646	2.907	MIGKIY13	151.462	3.026	MIGKIY13	151.636	2.907	MIGKIY14	170.014	3.058
MIGKIY16	170.027	3.059	MIGKIY16	151.494	3.026	MIGKIY15	170.008	3.058	MIGKIY15	151.47	3.025	MIGKIY15	151.642	2.907
MIGKIY17	151.625	2.907	MIGPEZ	151.285	3.341	MIGKIY16	151.628	2.907	MIGKIY17	170.03	3.058	MIGKIY17	151.499	3.026
MIGVEF	166.13	3.446	MIGVEF	161.219	3.378	MIGPEZ	152.859	3.41	MIGREC	161.796	2.923	MIGREC	161.767	2.923
MIGWUY	180	2.641	MIGZIO	160.758	3.124	MIGVUX	168.22	3.291	MIGVUX	151.662	3.135	MIGVUX	163.782	2.849
MIGZUA	167.988	3.329	MIGZUA	156.183	3.081	MIGZIO	168.885	3.322	MIGZOU	157.762	3.013	MIGZOU	170.214	3.358
MIHFEO	152.646	2.89	MIHFEO	151.939	2.792	MIHBAJ	156.754	3.065	MIHBAJ	168.605	3.342	MIHFEO	150.625	2.878
MIHGET	168.682	3.068	MIHHES	164.114	2.999	MIHGET	153.555	2.957	MIHGET	152.062	3.062	MIHGET	158.604	2.957
MIHLIY	159.63	2.923	MIHLIY	154.143	2.967	MIHKAR	162.299	3.337	MIHKIA	155.8	3.341	MIHKIA	171.227	3.49
MIHPAU	150.167	2.78	MIHPAU	176.106	3.206	MIHLIY	152.445	3.321	MIHNOG	171.622	3.147	MIHNUM	174.006	3.168
MIHPOI	153.242	2.891	MIHPOI	161.092	2.982	MIHPEY02	161.564	3.376	MIHPIC03	162.582	3.452	MIHPOI	156.118	2.993
MIHPUO	167.651	3.498	MIHPUO	157.273	3.347	MIHPUO	156.389	3.103	MIHPUO	175.141	3.424	MIHPUO	154.727	3.451
MIHPUO	169.441	3.118	MIHPUO	157.431	2.879	MIHPUO	155.61	3.451	MIHPUO	162.792	3.347	MIHPUO	150.193	2.846
MIHTOP	151.102	3.49	MIHTOP	157.154	3.491	MIHQEC	152.76	3.392	MIHTIJ	176.464	3.001	MIHTOP	150.573	3.142
MIHWOQ	151.588	3.121	MIHWOQ	169.348	3.097	MIHTOP	155.446	3.045	MIHTUV	158.395	2.987	MIHWOQ	151.632	3.006
MIHXAD	156.105	3.355	MIHXIO	151.694	3.135	MIHWOQ	153.202	2.952	MIHWUW	162.304	2.653	MIHXAD	171.955	2.894
MIHYIO	162.593	3.365	MIJBOA	150.375	3.408	MIHXIO	161.508	3.009	MIHYIO	151.817	3.127	MIHYIO	155.972	3.261
MIJDOA	160.939	3.22	MIJHOE	168.36	3.246	MIJCUE	177.131	2.911	MIJCUE	155.084	2.898	MIJDOA	158.652	3.22
MIJJIY	165.544	3.185	MIJJUN	170.354	3.49	MIJHOE	151.427	3.403	MIJJIY	150.884	3.438	MIJJIY	166.334	2.882
MIJLAU	174.775	3.127	MIJNUP	170.855	2.869	MIJKEY	158.709	3.191	MIJKIB	176.238	3.19	MIJKUN	174.626	3.107
MIJPUQ	160.361	3.01	MIJQUR	164.763	3.309	MIJNUP	161.879	3.472	MIJNUP	151.705	3.133	MIJPOK	157.936	2.971
MIJYEL	158.012	2.862	MIJYEL	157.161	2.857	MIJQUR	160.416	3.407	MIJQUR	159.769	3.301	MIJQUR	158.574	3.424
MIKDAN	175.157	3.004	MIKDAN	165.604	3.367	MIJYEN	168.856	3.24	MIKDAN	164.353	3.007	MIKDAN	163.415	2.877
MIKNUT	170.444	2.999	MIKYAG	160.331	3.307	MIKDAN	165.572	2.926	MIKDEP	170.228	2.985	MIKNUT	156.095	3.022
MIKYUE	170.01	3.441	MIKYUE	158.091	3.388	MIKYAH	158.429	3.076	MIKYAH	161.526	3.292	MIKYAH	153.408	3.468
MILBOC	157.324	3.422	MILBOC	160.572	3.303	MIKYUE	167.614	3.454	MIKZUC	153.173	2.862	MILBES	154.647	2.875
MILPAY	164.448	3.016	MILPAY	151.512	2.761	MILDAQ	157.84	2.852	MILDAQ	162.909	2.961	MILNUQ	156.851	2.915
MILWOW	151.884	2.995	MILWUC	157.766	3.143	MILWIN	163.979	3.367	MILWIN	157.311	3.388	MILWOW	162.854	3.386
MIMCOB	155.432	2.7	MIMHAT	160.738	3.414	MILWUC	154.556	2.634	MIMCOB	153.32	3.338	MIMCOB	163.654	2.917

MIMLAX	151.305	3.363	MIMRIN	167.047	3.246	MIMHEV	170.143	3.347	MIMJIC	173.523	3.155	MIMLAX	152.017	3.216
MIMRIN	150.607	3.317	MIMTUB	167.702	2.988	MIMRIN	150.638	3.14	MIMRIN	170.245	2.785	MIMRIN	150.991	2.862
MIMTUB	172.395	2.858	MIMTUB	153.785	2.816	MIMTUB	174.316	2.99	MIMTUB	153.892	3.476	MIMTUB	163.184	2.988
MIMVAJ	164.979	2.753	MIMVAJ	151.244	3.291	MIMTUB	151.793	2.932	MIMVAJ	172.918	3.266	MIMVAJ	159.628	3.388
MIMVEN	173.103	3.471	MIMVEN	162.261	2.969	MIMVAJ	164.295	2.776	MIMVAJ	159.85	3.41	MIMVAJ	172.81	3.103
MIMVEN	177.715	3.41	MIMVEN	160.341	2.766	MIMVEN	162.198	2.961	MIMVEN	177.855	2.717	MIMVEN	160.452	3.123
MINHUO	155.453	2.957	MINLEE	150.651	2.89	MIMVEN	158.759	3.131	MINDUI	169.468	3.235	MINDUI	169.142	3.235
MINTEJ	156.868	2.848	MINTEJ	150.004	3.273	MINLEE	165.033	2.89	MINMAZ	167.674	3.013	MINRAG	150.889	3.346
MINTEJ	151.227	2.935	MINTEJ	151.2	2.971	MINTEJ	164.167	3.345	MINTEJ	161.176	2.971	MINTEJ	154.893	2.935
MINTIQ	165.758	2.676	MINTIQ	151.721	2.676	MINTEJ	164.318	2.918	MINTEJ	160.909	2.982	MINTIQ	160.023	2.983
MINTUZ	163.732	2.92	MINTUZ	168.19	3.03	MINTUZ	156.434	3.312	MINTUZ	156.941	3.275	MINTUZ	159.354	2.983
MINTUZ	161.24	2.946	MINVAH	169.448	2.676	MINTUZ	157.034	3.276	MINTUZ	166.024	3.41	MINTUZ	155.553	3.042
MINVIR	157.007	2.996	MINVIR	177.436	2.854	MINVAH	150.845	2.706	MINVAH	169.131	2.848	MINVIR	166.682	3.032
MIPCIA	154.32	2.676	MIPGOL	153.403	3.084	MINVIR	155.392	3.078	MINYIU	166.477	3.058	MIPCIA	175.908	2.676
MIPHAU	152.612	3.13	MIPHAU	151.235	3.496	MIPHAU	162.006	2.957	MIPHAU	150.443	3.246	MIPHAU	170.315	3.154
MIPHAU	158.161	3.231	MIPHAU	153.586	3.443	MIPHAU	154.893	3.231	MIPHAU	160.357	2.893	MIPHAU	175.221	2.706
MIPHAU	158.926	3.459	MIPHAU	160.058	2.957	MIPHAU	174.512	2.893	MIPHAU	154.978	3.344	MIPHAU	155.207	3.08
MIPHAU	152.971	3.331	MIPHEY	160.978	2.972	MIPHAU	163.36	3.04	MIPHAU	153.193	3.416	MIPHAU	154.872	3.117
MIPHEY	162.306	3.426	MIPHUQ	154.19	3.186	MIPHEY	156.178	2.972	MIPHEY	152.367	3.432	MIPHEY	161.222	3.302
MIPHUQ	150.032	3.066	MIPHUQ	160.524	2.822	MIPHUQ	155.442	3.455	MIPHUQ	154.02	2.833	MIPHUQ	152.089	2.94
MIPHUQ	163.941	2.972	MIPHUQ	165.044	3.038	MIPHUQ	157.093	3.385	MIPHUQ	162.496	3.405	MIPHUQ	156.639	2.919
MIPHUQ	162.174	2.966	MIPHUQ	151.458	2.653	MIPHUQ	154.972	2.976	MIPHUQ	161.464	3.156	MIPHUQ	156.148	3.298
MIPKON	164.154	3.447	MIPLAA	159.382	3.417	MIPJIE	165.288	3.14	MIPJOL	177.701	3.365	MIPKON	158.586	3.155
MIPQEL	162.736	3.258	MIPVUG	150.591	3.377	MIPLAA	161.833	3.015	MIPLAA	158.846	2.886	MIPMAB	154.805	3.217
MIQFAX	154.268	3.164	MIQFAX	162.126	2.999	MIPVUG	174.164	2.903	MIPXES	161.852	3.306	MIPXES	153.778	3.482
MIQFAX	159.051	2.944	MIQFAX	151.337	2.957	MIQFAX	176.97	2.729	MIQFAX	154.701	3.2	MIQFAX	173.273	3.164
MIQFAX	167.888	2.999	MIQFAX	157.931	3.255	MIQFAX	155.662	2.995	MIQFAX	166.038	3.462	MIQFAX	157.022	2.729
MIQFOL	157.798	2.945	MIQFOL	166.262	2.966	MIQFIF	151.231	3.095	MIQFIF	159.816	3.108	MIQFOL	163.056	2.945
MIRFEZ	154.168	3.211	MIRJIG	167.674	3.137	MIQWEO	155.384	2.623	MIQWEO	163.984	3.157	MIRFEZ	157.236	3.399
MIRLIL	171.825	3.097	MIRLIL	166.572	3.319	MIRJIG	170.348	3.151	MIRKOQ	151.829	2.84	MIRLIL	163.926	3.319
MIRNAF	156.336	3.49	MIRNAF	160.796	3.077	MIRLOR	153.768	2.941	MIRMAE	151.74	2.842	MIRMIM	174.61	3.027
MIRPEL	165.245	3.253	MIRPIO	166.596	3.328	MIRNOT	166.511	3.172	MIRPAH	166.475	3.206	MIRPAH	158.06	3.44
MISXUI	164.903	3.331	MISYIW02	170.86	2.659	MIRTUF	155.511	2.955	MIRZAO	156.775	3.209	MISHED	168.459	3.001
MITJEI	154.065	3.375	MITJIM	152.19	3.34	MITCUO	158.831	3.272	MITDUS	163.711	3.476	MITDUS	151.752	3.476
MITKEF	155.785	3.375	MITKEF	151.769	3.061	MITJIM	162.587	3.176	MITKEF	150.824	2.815	MITKEF	150.706	2.858
MITSAN	163.313	3.366	MITVAQ	160.267	3.017	MITKEF	152.725	2.769	MITRUG	162.551	2.684	MITRUG	156.734	2.684
MITVAQ	162.975	2.989	MITVAQ	159.967	2.835	MITVAQ	167.225	2.746	MITVAQ	157.701	2.797	MITVAQ	151.425	2.835
MITYIZ	174.209	2.566	MITZOG	153.298	3.425	MITVAQ	164.825	2.742	MITVAQ	175.976	2.906	MITYIZ	162.148	2.566
MIVMAF	173.287	3.396	MIVMAF	158.965	2.932	MIVBAW	160.289	3.127	MIVFEC	177.681	3.089	MIVKEI	173.058	3.282
MIVMAJ	164.713	3.313	MIVMAJ	150.113	2.911	MIVMAF	155.48	3.209	MIVMAF	175.367	2.899	MIVMAF	155.848	3.227
MIVMAJ	163.426	3.448	MIVMAJ	150.207	2.981	MIVMAJ	160.659	2.802	MIVMAJ	167.949	2.981	MIVMAJ	176.432	3.143
MIVMAJ	171.158	3.313	MIVMAJ	151.098	3.4	MIVMAJ	160.224	3.084	MIVMAJ	173.2	3.168	MIVMAJ	173.149	3.16
MIVMAJ	164.605	3.084	MIVMAJ	151.731	2.905	MIVMAJ	151.856	3.3	MIVMAJ	175.319	3.348	MIVMAJ	155.535	3.111
MIVMAJ	163.606	3.3	MIVMAJ	150.371	3.129	MIVMAJ	163.112	3.04	MIVMAJ	163.016	3.138	MIVMAJ	154.179	3.4
MIVMEJ	151.485	3.328	MIVMEJ	162.419	2.847	MIVMAJ	154.349	3.169	MIVMEJ	157.461	2.847	MIVMEJ	157.766	3.153
MIVMIN	169.88	3.194	MIVMOT	166.452	2.973	MIVMIN	171.005	3.466	MIVMIN	166.605	3.451	MIVMIN	158.626	3.329
MIVSEP	151.887	3.189	MIVSEP	157.419	3.495	MIVMOT	159.254	2.895	MIVMOT	161.077	3.395	MIVMOT	167.563	3.408
MIWFUV	168.364	3.183	MIWGEE	159.906	3.475	MIVSEP	169.739	2.784	MIWBIF	159.177	3.29	MIWFUV	160.448	3.183
MIWLAI	169.929	3.321	MIWLUA	161.665	2.825	MIWJAE	159.197	3.405	MIWJAE	164.033	3.469	MIWLAI	169.624	3.223
MIWNAK	174.359	3.458	MIWNAK	160.763	3.472	MIWLUA	160.581	3.278	MIWMAH	174.602	3.495	MIWMAH	171.28	3.321
MIXFAB	162.298	3.24	MIXJOU	172.299	3.437	MIXBAW	154.528	3.065	MIXBAW	155.988	3.288	MIXBAW	151.295	3.335
MIYGOT	152.092	3.247	MIYKOU	161.277	3.128	MIXJOU	168.591	3.15	MIYBEE	169.463	2.776	MIYGOT	152.249	3.262
MIYMEQ	165.438	3.173	MIYMEQ	161.636	3.434	MIYKOU	156.898	3.465	MIYKOU0	164.732	3.121	MIYKOU0	157.63	3.321
MIYQUK	158.325	2.913	MIYTUK	159.245	2.763	MIYMEQ	167.072	3.392	MIYMEQ	177.428	3.229	MIYMEQ	169.426	3.196
MIYWAU	164.346	3.036	MIYWEW	161.653	3.43	MIYVAU	159.435	2.797	MIYWAU	168.343	3.499	MIYWAU	173.019	2.784
MIYXOK	164.855	2.809	MIYXOK	159.098	2.939	MIYXAW	150.43	3.124	MIYXIE	159.191	3.185	MIYXIE	153.806	2.965
MIYZEB	157.222	3.477	MIYZEB	163.657	3.462	MIYXOK	160.718	2.832	MIYXOK	152.098	3.054	MIYYIF	163.466	2.997
MIZBAY	163.692	2.921	MIZBAY	160.462	3.114	MIZBAY	159.031	3.153	MIZBAY	159.846	3.067	MIZBAY	153.315	3.414
MIZQUI	172.028	2.539	MIZQUI	176.353	2.539	MIZCIH	174.457	2.954	MIZQUI	158.223	2.776	MIZQUI	153.882	3.273
MIZQUI	151.05	3.433	MIZQUI	160.756	2.765	MIZQUI	153.382	2.774	MIZQUI	156.448	2.882	MIZQUI	158.381	3.031
MIZQUI	150.837	2.931	MIZQUI	158.969	3.102	MIZQUI	156.514	3.476	MIZQUI	150.317	3.107	MIZQUI	156.036	2.983
MIZQUI	172.189	2.946	MIZQUI	150.275	3.063	MIZQUI	158.717	2.96	MIZQUI	152.536	3.07	MIZQUI	162.842	3.116
MIZQUI	157.228	2.754	MIZQUI	150.3	3.233	MIZQUI	164.991	3.102	MIZQUI	159.78	3.44	MIZQUI	152.632	3.273
MIZRAP	157.647	2.925	MIZRAP	178.094	2.799	MIZQUI	152.139	2.765	MIZRAP	168.162	3.173	MIZRAP	174.278	2.69
MIZRET	150.5	3.009	MIZVAU	162.079	3.044	MIZRAP	163.747	3.002	MIZRAP	160.923	3.458	MIZRET	168.753	3.108
MIZVEY	151.558	3.049	MIZVEY	151.137	3.186	MIZVAU	175.867	3.195	MIZVAU	166.679	3.312	MIZVEY	160.471	3.077
MIZWID	166.078	2.935	MIZWID	157.649	3.242	MIZVEY	161.706	3.019	MIZVEY	151.174	3.077	MIZVEY	171.779	3.006
MOBBAK	163.765	3.418	MOBTAA	164.945	2.894	MIZWID	158.33	3.211	MIZZON	150.362	3.263	MOBBAK	170.87	2.754
MOBTAA	175.859	2.778	MOBWAE	153.595	2.763	MOBTAA	155.871	2.872	MOBTAA	163.591	3.378	MOBTAA	154.83	2.826
MOBWAE	153.797	2.877	MOBWAE	155.422	2.903	MOBWAE	153.242	2.763	MOBWAE	167.021	3.026	MOBWAE	151.914	2.789
MOCMAS	166.631	3.409	MOCMIA	165.478	3.031	MOCBIQ	172.095	3.102	MOCBOW	175.328	3.304	MOCMAS	157.373	3.409
MOCNEX	155.611	3.184	MOCNEX	151.871	2.862	MOCMIA	172.968	3.143	MOCMOG	150.608	3.378	MOCMUM	161.878	2.745
MOCREB	153.595	3.428	MOCREB	153.616	3.045	MOCNEX	166.632	3.449	MOCNEX	161.888	2.731	MOCNEX	155.35	3.114
MOCXUZ	172.364	3.434	MOCXUZ	166.486	2.897	MOCREB	160.145	2.867	MOCSAA	168.946	3.147	MOCXUZ	154.948	3.194
MOCYEK	151.801	3.151	MOCYEK	156.016	3.422	MOCXUZ	151.842	3.191	MOCXUZ	158.323	3.239	MOCYEK	150.309	3.479
MODMUP	161.857	3.394	MODNEA	157.781	3.449	MODFIX	153.6	2.828	MODFUI	158.996	3.202	MODGAP	150.46	3.243
MODPAY	154.105	2.947	MODPAY	156.726	2.746	MODNEA	161.913	3.261	MODNIE	174.509	3.424	MODNIE	153.194	3.181
MODSAC	163.058	3.088	MODSAC	164.379	2.966	MODQAX	157.753	3.169	MODQAX	150.081	3.293	MODSAC	163.75	3.276
MODTUU	153.404	3.266	MODTUU	171.532	3.487	MODSAC	175.3	3.467	MODSEF	173.382	3.366	MODTUU	173.606	3.058

MOFBER0	168.821	3.187	MOFCAO	162.059	3.489	MODTUU	166.962	2.94	MOFBER0	156.837	3.088	MOFBER0	164.922	3.064
MOFHOF	163.449	3.218	MOFHOF	164	2.985	MOFDET	174.066	3.016	MOFHOF	154.494	3.102	MOFHOF	157.269	2.806
MOFHOF	151.304	3.324	MOFHOF	153.819	2.806	MOFHOF	160.466	3.126	MOFHOF	155.977	3.234	MOFHOF	153.258	3.041
MOFHOF	152.995	2.892	MOFHUL	153.747	2.984	MOFHOF	171.189	2.806	MOFHOF	159.801	3.228	MOFHOF	165.436	2.907
MOFHUL	165.147	3.261	MOFHUL	152.371	3.286	MOFHUL	158.845	3.267	MOFHUL	173.602	2.908	MOFHUL	156.148	3.38
MOFHUL	171.86	3.232	MOFJEX	163.058	3.434	MOFHUL	155.556	3.344	MOFHUL	161.669	2.976	MOFHUL	151.867	3.011
MOFSIK	163.088	3.367	MOFSIK	153.929	3.158	MOFPON	151.257	3.053	MOFQEG	159.727	3.262	MOFQEG	158.719	2.81
MOGLOL	155.77	3.029	MOGLOL	151.981	3.392	MOFTAF	151.128	3.303	MOGLAV	156.822	3.095	MOGLID	151.259	3.22
MOGPUU	167.843	2.682	MOGQOO	153.317	2.939	MOGMAY	162.434	3.073	MOGPUU	164.805	2.682	MOGPUU	155.172	2.603
MOGRUX	165.72	3.001	MOGSAG	163.381	2.947	MOGQOO	153.013	3.29	MOGRUX	162.73	2.906	MOGRUX	156.178	3.212
MOHJEZ	160.169	3.15	MOHJEZ	150.114	3.014	MOHJAV	163.02	3.43	MOHJAV	160.163	3.153	MOHJEZ	153.289	3.408
MOHTUY	161.439	3.186	MOHTUY	165.134	2.943	MOHJEZ	150.75	3.014	MOHJEZ	167.462	3.177	MOHPAB	170.046	3.057
MOJDAR	166.733	3.307	MOJDAR	157.673	3.293	MOHVUA	158.172	2.86	MOJDAR	153.439	3.446	MOJDAR	176.349	3.219
MOJFUP	158.264	3.386	MOJLUW	162.427	3.326	MOJDAR	160.544	3.262	MOJFUN	162.869	3.031	MOJFUP	164.521	3.367
MOKCOF	151.45	3.428	MOKGID	156.067	3.34	MOJLUW	173.705	3.393	MOJLUW	157.078	3.125	MOJLUW	154.042	3.178
MOKKON(	162.898	2.91	MOKLUU	150.94	3.424	MOKGOJ	171.607	3.366	MOKHOK	151.598	3.301	MOKKON(	151.096	2.91
MOKPOV	152.834	2.947	MOKPUB	159.141	3.134	MOKPOV	159.045	2.927	MOKPOV	151.258	3.307	MOKPOV	169.227	2.981
MOKVIV	157.252	3.407	MOLFUQ	160.977	3.06	MOKPUB	160.17	3.06	MOKPUB	155.452	3.243	MOKVIV	152.075	3.491
MOLGEB	163.933	3.038	MOLGEB	161.616	3.455	MOLFUQ	151.258	3.302	MOLGAX	158.912	3.04	MOLGAX	168.27	3.3
MOLGIF	165.598	3.474	MOLGIF	170.995	3.161	MOLGEB	162.763	3.405	MOLGEB	151.882	2.955	MOLGEB	154.396	3.394
MOLGUR	155.024	2.757	MOLGUR	159.004	3.262	MOLGIF	157.346	3.124	MOLGUR	155.984	3.297	MOLGUR	168.097	3.362
MOLHEC	153.032	3.21	MOLHEC	160.025	3.199	MOLHEC	160.518	3.199	MOLHEC	160.292	3.014	MOLHEC	156.684	2.919
MOLHUS	154.078	2.754	MOLHUS	152.94	2.832	MOLHIG	165.305	2.944	MOLHUS	164.6	2.906	MOLHUS	154.115	2.754
MOLJAA	151.52	3.006	MOLJEE	162.941	3.143	MOLJAA	159.27	2.792	MOLJAA	158.742	2.814	MOLJAA	153.597	3.052
MOLJII	155.79	2.96	MOLJII	156.667	3.116	MOLJEE	151.256	3.02	MOLJII	157.627	3.44	MOLJII	151.918	2.841
MOLJOO	158.664	2.921	MOLJOO	156.587	2.857	MOLJII	152.062	3.03	MOLJII	151.558	3.31	MOLJII	150.082	2.841
MOLJUU	153.038	2.934	MOLJUU	161.354	3.452	MOLJOO	150.643	3.231	MOLJOO	153.503	2.876	MOLJUU	166.687	3.077
MOLJUU	173.163	2.93	MOLPEI	161.983	3.48	MOLJUU	165.563	3.108	MOLJUU	167.48	2.884	MOLJUU	162.596	3.256
MOLPOS	150.12	3.361	MOLPOS	153.466	3.456	MOLPEI	171.351	3.362	MOLPEI	150.909	3.284	MOLPEI	155.629	2.981
MOLRAI	171.673	3.092	MOLRAI	174.765	3.111	MOLPOS	163.431	3.18	MOLPOS	178.239	2.812	MOLPOS	150.483	2.735
MOLREM	157.609	3.088	MOLRIQ	156.527	3.252	MOLRAI	162.398	2.941	MOLREM	155.853	2.709	MOLREM	157.59	2.889
MOMBEV	155.33	3.319	MOMBEV	171.678	3.433	MOLYAP	154.876	2.886	MOMBAR	153.593	3.364	MOMBAT	170.84	2.875
MOMBUL	153.861	3.179	MOMLUX	168.555	2.962	MOMBEX	178.657	2.669	MOMBEX	174.295	2.669	MOMBUL	152.035	3.179
MOMRUB	150.047	3.391	MOMYIA	174.36	3.08	MOMLUX	160.669	2.983	MOMLUX	161.742	3.029	MOMNIL	151.361	3.009
MONHIK	157.075	3.446	MONNEK	153.087	3.26	MOMYIA	171.957	3.269	MOMZEX	171.819	3.123	MOMZUN	154.761	3.205
MONYOD	167.78	3.473	MONYOD	151.197	2.726	MONPEM	170.403	2.801	MONPIQ	151.713	3.11	MONXIX	155.872	3.448
MONZIY	161.957	3.076	MONZIY	153.397	2.862	MONYOD	160.363	3.27	MONYOD	152.929	2.709	MONZIY	160.148	3.178
MOPNEL	169.984	3.036	MOPNEL	156.181	2.628	MONZIY	166.582	2.993	MOPCOK	154.236	3.254	MOPJAE	160.932	3.18
MOPNEL	155.54	3.238	MOPNEL	154.945	3.004	MOPNEL	151.952	2.777	MOPNEL	155.059	3.124	MOPNEL	159.098	2.777
MOPNEL	169.904	3.018	MOPNEL	173.884	3.462	MOPNEL	163.85	2.725	MOPNEL	172.073	3.333	MOPNEL	171.305	3.053
MOPNOV	150.974	3.431	MOPNOV	151.09	3.31	MOPNEL	157.506	2.782	MOPNIP	151.069	3.278	MOPNIP	153.753	3.356
MOQFIL	152.98	3.149	MOQGAE	156.584	2.839	MOPNOV	155.066	3.304	MOPZEX	162.257	3.196	MOQFIL	156.937	3.47
MOQNOZ	158.312	3.398	MOQNUF	151.651	3.198	MOQMOY	152.074	3.256	MOQMOY	160.3	3.484	MOQNAL	175.026	3.416
MORDIG	154.924	3.178	MORFOS	151.714	3.014	MOQVIB	151.183	3.106	MOQVIB	162.947	3.166	MOQVIB	164.486	3.461
MORKOU	159.175	3.343	MORMUF	152.937	2.878	MORKOU	177.107	2.646	MORKOU	160.63	2.646	MORKOU	155.614	3.194
MOSBEB	153.324	2.993	MOSBEB	158.593	3.185	MORMUF	167.426	3.489	MORMUF	172.536	3.489	MORSOB	165.549	3.357
MOSBEB0	154.439	3.042	MOSGEJ	168.137	3.133	MOSBEB0	158.878	3.163	MOSBEB0	152.617	2.976	MOSBEB0	158.25	3.258
MOTSUJ	159.851	2.775	MOTSUJ	159.691	3.21	MOSZAX	159.015	2.963	MOTSUJ	157.847	3.275	MOTSUJ	162.957	3.47
MOTZEE	160.513	3.499	MOTZUT	163.506	2.797	MOTSUJ	170.8	2.775	MOTSUJ	150.834	3.492	MOTSUJ	161.617	2.994
MOTZUT	155.188	3.468	MOVBEI	163.978	2.878	MOTZUT	168.863	2.797	MOTZUT	164.307	3.014	MOTZUT	163.357	3.405
MOVNEQ	157.165	3.205	MOVNEQ	157.199	3.124	MOVNEQ	153.079	3.301	MOVNEQ	162.905	2.771	MOVNEQ	153.669	3.337
MOVQOH	158.185	3.038	MOVQUN	151.234	3.347	MOVNEQ	166.533	3.099	MOVNEQ	151.344	2.978	MOVPUJ	157.598	3.047
MOWBIJ	152.081	3.018	MOWDIO	164.755	3.005	MOVQUN	150.253	3.082	MOVZUV	150.443	3.015	MOWBIJ	163.148	2.899
MOWRAR	161.217	3.41	MOWREY	158.98	3.259	MOWDIO	154.373	3.298	MOWLIU	164.439	3.313	MOWNAR	170.479	3.028
MOWTAX	166.484	3.129	MOWTAX	172.184	3.029	MOWRIZ	167.174	2.807	MOWRIZ	157.079	2.997	MOWRIZ	164.169	2.807
MOWZUU	154.163	2.935	MOWZUU	154.621	2.695	MOWTEB	171.501	2.978	MOWTEB	169.817	2.771	MOWZAA	150.011	3.47
MOXBAD	170.316	2.837	MOXBAD	150.141	2.717	MOWZUU	153.188	3.061	MOWZUU	150.874	2.931	MOXBAD	161.369	2.604
MOXBAD	166.593	2.604	MOXBAD	178.212	2.837	MOXBAD	157.622	2.982	MOXBAD	157.378	3.205	MOXBAD	150.257	2.951
MOXBAD	151.889	3.167	MOXBAD	154.176	3.182	MOXBAD	158.175	3.455	MOXBAD	151.679	2.913	MOXBAD	150.493	2.566
MOXBEH	168.474	2.996	MOXBEH	150.041	3.165	MOXBAD	161.765	3.379	MOXBEH	158.821	3.391	MOXBEH	163.595	3.047
MOXBEH	152.308	3.367	MOXBEH	163.299	2.992	MOXBEH	175.835	2.65	MOXBEH	173.982	3.05	MOXBEH	151.591	3.157
MOXBEH	154.098	3.164	MOXBEH	166.423	2.959	MOXBEH	154.923	3.449	MOXBEH	161.569	2.792	MOXBEH	161.518	3.233
MOXBIL	155.969	3.198	MOXBOR	150.612	3.167	MOXBIL	155.271	2.944	MOXBIL	152.866	3.046	MOXBIL	150.261	3.26
MOXBOR	172.635	3.403	MOXBOR	153.639	3.387	MOXBOR	152.364	2.729	MOXBOR	152.661	3.261	MOXBOR	168.756	3.093
MOXBOR	170.257	3.443	MOXBOR	150.392	3.248	MOXBOR	177.997	2.674	MOXBOR	156.892	3.219	MOXBOR	169.903	2.687
MOXBOR	157.182	2.787	MOXBOR	168.147	3.162	MOXBOR	150.585	2.773	MOXBOR	169.67	3.366	MOXBOR	150.135	3.153
MOXCEI	178.048	3.296	MOXCEI	169.548	3.345	MOXBOR	154.496	3.007	MOXBOR	158.076	3.089	MOXBOR	151.236	3.201
MOXCEI	151.913	3.279	MOXCEI	151.162	3.079	MOXCEI	164.452	3.249	MOXCEI	160.897	3.385	MOXCEI	159.882	2.933
MOXCUY	168.185	2.692	MOXCUY	156.648	3.081	MOXCEI	166.068	3.316	MOXCEI	170.553	2.833	MOXCUY	152.44	2.765
MOXLET	161.2	3.059	MOXMAN	160.266	3.098	MOXFUE	163.103	3.272	MOXFUE	169.187	2.651	MOXFUE	170.988	3.161
MOXMAN	166.366	3.151	MOXMAN	167.158	3.107	MOXMAN	154.074	2.868	MOXMAN	154.771	3.453	MOXMAN	167.32	3.117
MOXMAN(	164.074	2.806	MOXMAN(	164.561	2.806	MOXMAN	157.301	2.868	MOXMAN	161.89	2.942	MOXMAN	169.047	3.117
MOXMAN(	160.033	2.849	MOXMAQ	164.032	3.026	MOXMAN(	164.021	2.785	MOXMAN(	165.958	2.785	MOXMAN(	161.546	2.849
MOXMAQ	150.493	3.408	MOXMEU	154.288	3.283	MOXMAQ	152.591	3.442	MOXMAQ	172.107	3.442	MOXMAQ	172.008	3.141
MOXMEU	160.991	3.167	MOXPOH	150.056	3.47	MOXMEU	162.054	3.021	MOXMEU	170.601	3.488	MOXMEU	158.824	3.426
MOXPOH	150.981	3.081	MOXPOH	153.876	2.92	MOXPOH	163.047	2.822	MOXPOH	176.346	2.934	MOXPOH	163.105	3.093
MOXYEF	151.511	3.396	MOXYOP	171.613	3.307	MOXYEF	166.451	2.791	MOXYEF	173.466	2.791	MOXYEF	159.553	3.087
MOXYOP	156.279	2.85	MOXYUV	168.433	2.854	MOXYOP	161.008	2.678	MOXYOP	159.931	2.7	MOXYOP	157.554	3.19
MOXYUV	163.654	3.115	MOYFEL	168.201	3.37	MOXYUV	155.86	3.021	MOXYUV	169.635	3.423	MOXYUV	150.079	3.412

MOYSAU0	156.771	2.839	MOYSAU0	153.296	2.769	MOYJAN	152.617	3.296	MOYMOE	177.908	2.926	MOYSAU0	163.461	2.733
MOYSAU0	162.733	3.406	MOYSAU0	164.968	3.273	MOYSAU0	158.207	2.789	MOYSAU0	172.048	3.087	MOYSAU0	155.35	2.901
MOYSAU0	164.037	3.096	MOYSAU0	151.835	3.095	MOYSAU0	159.479	2.893	MOYSAU0	150.556	2.923	MOYSAU0	155.766	3.008
MOYSAU0	153.927	3.265	MOYSAU0	156.673	3.187	MOYSAU0	154.935	3.348	MOYSAU0	150.039	2.985	MOYSAU0	162.618	3.343
MOYSAU0	158.149	2.86	MOYSAU0	163.156	3.355	MOYSAU0	152.256	3.128	MOYSAU0	150.254	3.087	MOYSAU0	154.579	2.86
MOYSAU0	161.642	2.902	MOYSAU0	164.992	3.248	MOYSAU0	174.994	2.88	MOYSAU0	159.838	2.735	MOYSAU0	168.328	2.742
MOYSAU0	162.476	2.863	MOYSAU0	160.787	3.098	MOYSAU0	158.063	2.982	MOYSAU0	160.761	3.225	MOYSAU0	152.977	3.409
MOYSAU0	163.426	3.326	MOYSAU0	162.526	3.446	MOYSAU0	152.208	2.815	MOYSAU0	150.706	3.104	MOYSAU0	172.037	3.379
MOYSAU0	156.141	3.184	MOYSAU0	176.395	2.96	MOYSAU0	150.964	2.929	MOYSAU0	160.279	2.819	MOYSAU0	163.652	2.803
MOYSAU0	157.914	2.752	MOYSAU0	158.721	2.815	MOYSAU0	170.994	3.152	MOYSAU0	157.469	3.139	MOYSAU0	158.03	2.739
MOYSAU0	159.689	3.092	MOYSAU0	164.92	3.138	MOYSAU0	155.597	2.948	MOYSAU0	169.668	3.212	MOYSAU0	163.205	3.166
MOYSAU0	150.664	3.099	MOYSAU0	172.566	2.759	MOYSAU0	154.215	3.274	MOYSAU0	152.215	3.258	MOYSAU0	161.304	2.831
MOYSAU0	156.017	3.433	MOYSAU0	156.575	2.769	MOYSAU0	152.756	3.113	MOYSAU0	156.167	2.862	MOYSAU0	156.841	3.063
MOYSAU0	161.752	3.379	MOYSAU0	154.693	3.14	MOYSAU0	156.421	2.826	MOYSAU0	150.677	2.802	MOYSAU0	156.141	2.987
MOYSAU0	170.548	3.147	MOYSAU0	152.006	3.121	MOYSAU0	151.184	3.114	MOYSAU0	152.265	3.107	MOYSAU0	172.898	2.838
MOYSAU0	174.636	3.402	MOYSAU0	155.775	3.007	MOYSAU0	164.724	3.272	MOYSAU0	153.346	3.239	MOYSAU0	151.436	3.312
MOYSAU0	154.542	3.364	MOYSAU0	155.58	3.018	MOYSAU0	151.058	2.752	MOYSAU0	151.898	2.893	MOYSAU0	162.639	3.282
MOYSAU0	159.552	3.24	MOYSAU0	162.67	2.841	MOYSAU0	153.338	3.129	MOYSAU0	153.155	3.189	MOYSAU0	165.361	3.149
MOYSEY	166.559	3.332	MOYSEY	151.92	2.961	MOYSAU0	155.631	2.745	MOYSAU0	170.901	2.892	MOYSAU0	169.703	3.492
MOYSEY	153.14	2.659	MOYSEY	154.495	3.121	MOYSEY	151.561	3.018	MOYSEY	150.733	3.432	MOYSEY	152.785	3.092
MOYZOR	154.842	2.968	MOYZOR	161.66	2.837	MOYSEY	167.644	3.157	MOYSEY	150.378	3.084	MOYYIK	157.224	3.396
MOZFEP	170.319	2.899	MOZFIS	163.363	3.322	MOYZOR	155.522	2.969	MOZDOW	150.02	3.305	MOZDUC	167.962	3.204
MOZQEX	153.38	2.855	MOZQEX	156.01	3.492	MOZFIS	167.453	3.008	MOZKAO	155.57	3.466	MOZKEV	155.962	3.232
MOZQEX	159.828	3.113	MOZTIE	159.911	2.88	MOZQEX	156.48	3.168	MOZQEX	155.003	3.438	MOZQEX	156.469	2.903
MOZXUW	171.077	3.452	MOZXUW	152.475	3.015	MOZTIE	170.896	3.41	MOZVEE	159.279	3.256	MOZWOQ	151.964	3.066
MPNCHP1	150.092	3.137	MSEFCQ1	156.877	3.193	MPFPNI	152.906	2.902	MPNCHP1	155.946	2.644	MPNCHP1	169.158	3.028
MSORHB	165.409	2.986	MSORHB	169.78	3.476	MSNFMS1	161.203	3.322	MSNFMS1	164.892	2.84	MSNFMS1	159.887	3.221
MTFEFE	155.833	3.002	MTFEFE	157.16	3.365	MSORHB0	162.187	3.134	MSORHB0	157.066	3.405	MTFEFE	150.281	3.365
MUBBOA	172.694	3.091	MUBBUG	161.046	3.28	MTFEFE	160.907	3.218	MTFEFE	160.741	2.913	MTYHFB0	153.403	3.345
MUBFAU	164.261	3.04	MUBFAU	170.858	2.831	MUBFAU	167.337	2.796	MUBFAU	159.135	3.232	MUBFAU	153.23	3.085
MUBFOI	157.002	3.043	MUBFOI	160.885	3.053	MUBFAU	155.168	3.156	MUBFAU	155.4	3.106	MUBFAU	156.59	3.031
MUBFOI	162.728	2.943	MUBFOI	156.757	2.713	MUBFOI	174	2.73	MUBFOI	154.293	3.036	MUBFOI	169.036	2.926
MUBFOI	153.046	3.002	MUBFOI	157.078	2.993	MUBFOI	158.475	2.861	MUBFOI	156.729	3.042	MUBFOI	167.762	3.189
MUBGAT	169.577	2.95	MUBGEX	152.802	2.948	MUBFOI	150.816	2.943	MUBGAT	158.395	3.334	MUBGAT	156.169	3.132
MUBGEX	153.275	2.951	MUBGEX	153.446	3.406	MUBGEX	153.48	3.454	MUBGEX	154.358	2.806	MUBGEX	159.412	2.908
MUBSEH	164.328	2.985	MUBSIL	157.62	2.779	MUBGOH	159.236	3.011	MUBGOH	167.551	2.993	MUBSEH	157.638	2.758
MUCGEY	155.391	3.077	MUCVUD	171.735	3.476	MUBSIL	176.014	3.224	MUBSIL	160.538	2.946	MUBVIP	155.893	3.19
MUDKOO	166.914	3.331	MUDKOO	151.488	2.865	MUCZIV	153.564	3.473	MUDCOH	168.297	3.104	MUDKOO	152.969	3.332
MUDKUU	168.264	3.242	MUDKUU	174.568	3.062	MUDKOO	164.336	2.982	MUDKUU	157.385	3.327	MUDKUU	162.024	2.95
MUDPIN	162.916	2.903	MUDPIN	153.914	3.141	MUDPIN	172.663	3.375	MUDPIN	160.294	3.091	MUDPIN	154.795	2.811
MUFPIQ	156.27	3.391	MUGBAU	172.007	3.388	MUDROT	173.946	3.43	MUDWOY	159.673	3.205	MUFMIN	156.088	3.34
MUGKUY	172.322	3.232	MUGPUC	155.734	3.013	MUGCIA	169.374	3.245	MUGCIA	166.997	3.446	MUGCOG	162.26	2.85
MUGYAP	152.113	3.077	MUGYAP	169.105	3.087	MUGQUB	162.795	3.293	MUGREO	174.271	3.246	MUGYAP	156.358	3.313
MUHNAI	172.328	2.598	MUHNAI	159.236	2.998	MUGYAP	173.829	3.135	MUHMOU	152.533	3.294	MUHNAI	153.899	3.071
MUHNAI	160.235	3.012	MUHNAI	151.266	3.068	MUHNAI	156.593	2.907	MUHNAI	172.245	3.371	MUHNAI	156.092	2.75
MUHNAI	175.985	3.147	MUHNAI	153.95	3.111	MUHNAI	165.991	3.168	MUHNAI	159.663	2.858	MUHNAI	158.846	2.992
MUHREP	152.374	2.852	MUHREP	159.315	2.635	MUHNAI	163.004	3.071	MUHQUE	168.574	2.859	MUHRAL	167.512	3.5
MUHVUK	159.685	3.345	MUHVUK	160.952	3.303	MUHVUK	160.776	3.04	MUHVUK	164.52	3	MUHVUK	167.308	3.443
MUJJAE	158.975	3.27	MUJLAG	157.511	2.816	MUJFIJ01	173.733	3.387	MUJFIJ01	150.95	3.052	MUJJAE	166.711	3.137
MUJLAG	171.5	3.006	MUJPIU	156.092	2.97	MUJLAG	166.653	3.252	MUJLAG	153.584	3.306	MUJLAG	176.762	3.302
MUJQAJ01	159.351	3.191	MUJVAS	156.055	2.984	MUJPIU	155.212	3.082	MUJQAJ01	155.716	2.877	MUJQAJ01	155.051	3.164
MUKGIJ	153.005	2.893	MUKGIJ	151.015	2.977	MUJYEZ	162.247	3.227	MUKGIJ	166.907	3.112	MUKGIJ	167.375	3.102
MUKMEM	169.178	3.319	MUKMIP	162.424	3.314	MUKGIJ	171.316	3.398	MUKLIP	159.993	2.945	MUKMEM	171.139	3.301
MUKPIV	168.217	3.14	MUKPIV	158.232	3.184	MUKPIV	177.326	3.469	MUKPIV	169.088	2.947	MUKPIV	151.771	3.383
MUKVIA	155.713	3.106	MUKVIA	153.57	3.193	MUKPIV	160.892	3.381	MUKVEW	155.31	3.445	MUKVEW	155.104	3.138
MUKWOF	152.438	3.172	MUKWOF	154.184	3.172	MUKVUM	151.335	3.242	MUKVUM	169.875	2.987	MUKVUM	158.147	2.728
MUKWUN	157.939	3.002	MUKWUN	164.541	3.477	MUKWOF	163.339	3.239	MUKWUN	154.952	3.122	MUKWUN	161.315	3.454
MUKWUN	164.442	3.095	MUKXAU	156.917	2.968	MUKWUN	169.247	2.999	MUKWUN	167.403	3.056	MUKWUN	161.108	3.171
MULHAF	162.593	2.952	MULHAF	170.094	2.826	MULDUV	155.626	3.446	MULFEG	154.305	3.207	MULFEG	152.754	3.206
MULHUW	155.592	3.325	MULJAE	168.368	3.002	MULHAF	168.123	2.826	MULHAF	160.709	3.002	MULHOQ	171.581	3.202
MULLIP	155.628	3.28	MULLUB	165.264	3.328	MULJAE	162.079	3.393	MULJUA	171.583	2.785	MULLIP	162.667	3.473
MULTEW	161.831	3.281	MULTEW	166.614	3.2	MULPEP	161.797	3.481	MULROE	155.141	3.44	MULTAR	156.251	2.919
MULVOH	151.888	3.263	MUMBEE	150.91	3.146	MULTEW	150.475	3.419	MULTEW	159.43	3.064	MULVOH	158.717	3.014
MUMDON	158.291	3.444	MUMDON	166.519	2.871	MUMBEE	168.728	2.903	MUMBII	153.48	3.363	MUMDON	170.924	3.003
MUMDUT	150.544	3.344	MUMDUT	172.604	3.311	MUMDON	161.275	3.082	MUMDON	159.392	2.975	MUMDON	169.875	3.04
MUMFEF	161.791	2.916	MUMFEF	168.636	2.862	MUMFAE	156.332	3.16	MUMFEF	155.042	3.179	MUMFEF	161.503	2.862
MUMFIJ	170.549	2.96	MUMHEJ	156.493	3.399	MUMFIJ	157.321	3.084	MUMFIJ	163.665	2.96	MUMFIJ	162.518	2.956
MUNMUD	173.878	3.326	MUNMUF	176.782	3.089	MUMSET	173.122	3.152	MUMSET	177.946	2.902	MUMZED	161.644	3.409
MUNNAK	152.601	3.23	MUNNAK	155.366	3.06	MUNMUF	163.443	3.364	MUNNAK	162.825	2.952	MUNNAK	174.655	3.379
MUNZON	154.341	2.813	MUNZON	172.089	2.86	MUNQEU	160.644	3.209	MUNSUM	160.159	3.492	MUNZON	165.933	2.86
MUNZUT	153.518	3.127	MUNZUT	153.588	3.069	MUNZON	162.619	3.209	MUNZON	170.594	3.333	MUNZON	172.675	3.363
MUPPUM	156.904	3.202	MUPQAT	152.771	2.918	MUPKER	167.886	3.233	MUPKIV	173.209	3.123	MUPPUM	155.121	3.202
MUPQAT	169.654	3.088	MUPQAT	157.806	2.733	MUPQAT	159.402	3.044	MUPQAT	171.529	2.733	MUPQAT	161.163	2.878
MUPQAT	169.038	3.246	MUPQAT	163.12	2.955	MUPQAT	158.514	3.088	MUPQAT	155.456	2.64	MUPQAT	173.488	3.023
MUPQAT	156.715	3.178	MUPQAT	157.421	2.755	MUPQAT	158.296	3.316	MUPQAT	166.122	2.734	MUPQAT	151.058	3.124
MUPQAT	158.556	3.316	MUPQAT	158.425	2.64	MUPQAT	159.844	2.914	MUPQAT	151.469	3.175	MUPQAT	159.771	2.957
MUPQAT	153.543	3.178	MUPQEX	153.151	3.118	MUPQAT	150.858	3.082	MUPQAT	151.484	3.427	MUPQAT	163.731	3.371
MUPQEX	159.777	3.118	MUPQIB	171.398	3.222	MUPQEX	158.62	2.606	MUPQEX	167.082	3.363	MUPQEX	161.76	2.786
MUPQOD	160.429	2.839	MUPQOH	152.843	3.41	MUPQIB	154.496	2.648	MUPQIB	161.536	2.695	MUPQIB	152.781	2.998

MUPQOH	160.074	3.012	MUPWUP	177.685	3.23	MUPQOH	166.947	2.806	MUPQOH	159.679	2.778	MUPQOH	165.004	3.179
MUPXAW	172.865	3.266	MUPXAW	167.381	3.267	MUPWUP	175.672	3.261	MUPWUP	175.335	3.261	MUPWUP	160.01	3.038
MUPXIE	151.747	2.91	MUPXIE	171.392	2.758	MUPXAW	159.837	3.472	MUPXAW	156.777	3.233	MUPXIE	151.193	2.848
MUQKOC	160.04	2.996	MUQKOC	174.439	3.494	MUQBUX	164.71	3.297	MUQBUX	161.046	3.297	MUQCEH	154.831	2.783
MUQKUI	156.197	3.312	MUQKUI	178.624	3.046	MUQKUI	167.778	3.327	MUQKUI	151.364	2.854	MUQKUI	155.85	3.267
MUQLAP	155.575	3.48	MUQLAP	151.073	2.834	MUQKUI	158.731	3.439	MUQKUI	150.13	3.142	MUQLAP	154.061	2.879
MUQLAP	154.628	2.893	MUQLAP	151.455	2.834	MUQLAP	161.255	3.22	MUQLAP	156.088	3.134	MUQLAP	164.068	3.343
MUQLAP	154.358	3.343	MUQLAP	158.701	3.193	MUQLAP	160.465	3.363	MUQLAP	175.745	3.231	MUQLAP	157.375	3.064
MUQMAM	166.17	2.639	MUQMAM	157.565	3.298	MUQLEQ	163.123	2.821	MUQLEQ	153.502	3.001	MUQLEQ	153.738	3.099
MUQXEF	168.671	2.945	MUQXEF	170.877	3.454	MUQNUL	157.52	2.714	MUQXEF	166.111	2.878	MUQXEF	171.074	2.998
MUQXEF	155.574	3.213	MUQXEF	155.365	3.484	MUQXEF	152.92	2.894	MUQXEF	172.15	3.474	MUQXEF	157.79	3.158
MURGOV	161.709	3.051	MURGUB	163.934	3.146	MUQYIJ	176.41	3.302	MURGIP	153.486	3.212	MURGIP	172.056	3.088
MURJIT	163.417	3.308	MURJOZ0	157.183	3.319	MURGUB	166.301	3.33	MURJIT	152.736	3.182	MURJIT	167.348	3.337
MURRAS	152.247	3.203	MURRAS	164.713	3.243	MURJUF	155.749	3.387	MURJUF	152.797	3.075	MURLOD	152.949	2.931
MURRAS	160.019	3.085	MURRAS	150.583	2.923	MURRAS	150.465	3.057	MURRAS	151.413	2.881	MURRAS	161.186	3.263
MURVAX	150.535	3.266	MURXUV	153.228	3.053	MURRAS	176.704	2.923	MURTUP	169.651	3.224	MURVAX	172.028	3.024
MUSNIA	172.659	3.081	MUSNIA	150.369	3.257	MUSCOV	166.794	3.335	MUSHAM	175.192	3.311	MUSNIA	152.662	3.138
MUSROI	167.529	2.868	MUSROI	165.883	2.954	MUSNIA	163.736	2.836	MUSROI	162.821	2.87	MUSROI	169.129	2.859
MUSSID	158.674	3.108	MUSVUV	165.629	2.733	MUSRUO	153.494	2.951	MUSRUO	153.801	2.951	MUSRUO	171.301	2.613
MUTRUQ	153.083	2.818	MUTRUQ	166.038	2.956	MUTCEM	173.692	3.115	MUTKER	166.982	3.262	MUTNAQ	151.614	2.58
MUVGIT	163.808	2.841	MUVGIT	150.509	3.417	MUTSIF	155.958	3.435	MUTSIF	157.517	3.115	MUVFIV	157.113	3.136
MUVGIT	162.952	3.346	MUVGIT	152.03	3.135	MUVGIT	161.688	3.294	MUVGIT	171.861	3.082	MUVGIT	163.052	2.939
MUVHOE	159.682	3.022	MUVHOE	178.217	3.125	MUVHIY	159.137	2.872	MUVHIY	164.792	3.199	MUVHOA	153.311	2.676
MUVSIF	155.453	3.079	MUVSIF	157.605	2.995	MUVHUK	173.517	3.449	MUVSIF	170.264	2.887	MUVSIF	151.919	2.84
MUVSIF	154.268	3.413	MUVSIF	174.759	3.14	MUVSIF	167.782	2.82	MUVSIF	150.508	2.868	MUVSIF	152.2	3.191
MUVSIF	157.364	3.131	MUVSIF	154.767	3.424	MUVSIF	159.961	2.857	MUVSIF	167.285	2.845	MUVSIF	168.559	3.117
MUVVEI	160.694	3.072	MUVVEI	168.03	3.01	MUVTEG	155.44	2.968	MUVTEG	151.48	3.226	MUVVEI	158.368	3.271
MUVXIN	167.549	3.343	MUVXIN	162.769	2.754	MUVVEI	162.008	3.271	MUVXIN	175.317	3.258	MUVXIN	170.572	3.25
MUWCEM	176.674	2.992	MUWPAX	171.316	3.477	MUVXIN	161.444	3.302	MUVXIN	151.171	3.426	MUWCEM	151.856	3.015
MUWPEB	157.048	3.307	MUWPEB	157.989	3.462	MUWPAX	178.792	3.497	MUWPAX	174.861	3.369	MUWPEB	163.276	3.358
MUWPIF	171.631	3.169	MUWPIF	164.237	3.093	MUWPEB	151.827	3.387	MUWPIF	151.654	3.27	MUWPIF	169.79	3.349
MUWSED	166.102	3.259	MUWSED	164.996	3.458	MUWPIF	152.762	3.48	MUWSED	160.95	3.325	MUWSED	170.211	2.866
MUWSED	160.409	3.448	MUWSED	152.608	2.935	MUWSED	164.022	3.031	MUWSED	156.64	3.237	MUWSED	157.53	3.141
MUWSED	152.501	3.095	MUWSED	162.747	2.985	MUWSED	156.162	3.346	MUWSED	164.251	3.128	MUWSED	166.278	3.259
MUWSED	163.02	2.858	MUWSED	150.983	3.399	MUWSED	165.367	3.115	MUWSED	157.784	3.419	MUWSED	153.967	2.858
MUXJUL	154.914	2.853	MUXJUL	156.467	2.931	MUWSON	173.565	2.921	MUWTEE	163.814	3.208	MUWTEE	161.238	3.39
MUXJUL	166.738	2.729	MUXJUL	153.569	3.388	MUXJUL	167.219	3.407	MUXJUL	171.586	3.407	MUXJUL	158.362	2.87
MUXKAS	150.996	2.829	MUXKAS	156.646	3.059	MUXKAS	164.062	2.829	MUXKAS	167.083	3.259	MUXKAS	153.707	3.057
MUXKIA	158.809	2.945	MUXQED	162.537	2.89	MUXKAS	177.303	3.242	MUXKIA	164.148	2.992	MUXKIA	178.764	2.992
MUXQED	156.513	3.227	MUXTIM	153.194	3.498	MUXQED	167.795	3.113	MUXQED	173.144	2.89	MUXQED	162.666	2.988
MUYJOI	151.246	3.35	MUYMIE	152.482	3.384	MUXYAI	150.091	3.298	MUYBEN	161.547	3.038	MUYHUM	166.327	3.468
MUYNIG	165.451	3.126	MUYQAA	157.415	2.914	MUYMIE	168.616	2.967	MUYMOK	169.276	2.906	MUYMOK	158.986	2.968
MUZNAZ	150.548	3.331	MUZRAB	155.006	2.833	MUYSOP	158.822	3.099	MUYSOP0	150.224	2.929	MUZBUI	153.19	2.809
MUZTUY	152.827	3.35	MUZTUY	157.509	3	MUZSOT	156.105	2.826	MUZTUY	161.74	2.753	MUZTUY	164.615	2.753
MUZXAG	175.636	2.927	MUZXAG	156.078	3.115	MUZXAG	154.997	3.227	MUZXAG	163.113	3.339	MUZXAG	157.171	2.779
MUZZUG	156.074	3.244	NABPOA	163.627	3.441	MUZXAG	155.111	2.814	MUZXEK	157.085	3.14	MUZYEP	155.819	3.288
NABRUG	166.585	3.418	NABSER	161.337	2.729	NABRAM	158.525	2.715	NABRAM	159.725	2.715	NABROA	157.522	2.898
NABSUJ	160.998	2.766	NABSUJ	160.894	2.766	NABSER	163.736	2.729	NABSOB	157.25	2.82	NABSOB	155.009	2.82
NABTEU	166.389	3.35	NABTEU	166.323	3.084	NABTEU	173.57	2.81	NABTEU	153.184	3.178	NABTEU	168.122	3.068
NABTEU	151.692	2.989	NABXOE	162.49	3.374	NABTEU	159.387	2.81	NABTEU	159.504	3.178	NABTEU	171.493	2.898
NABYAR	154.999	2.887	NABYAR	154.219	3.296	NABXOE	156.613	3.171	NABYAR	163.732	3.309	NABYAR	152.923	3.313
NACPEN	152.074	3.201	NACPEN	170.696	2.867	NACDIH	165.326	3.049	NACKUB	176.175	2.667	NACPEN	178.932	2.867
NACPIR	161.143	3.4	NACPIR	175.335	3.068	NACPEN	153.185	3.202	NACPEO	157.544	3.423	NACPEO	154.262	3.494
NACPIR	155.579	2.974	NACPUD	159.121	2.944	NACPIR	172.527	3.389	NACPIR	158.474	3.055	NACPIR	167.552	2.953
NACPUD	150.272	3.2	NACPUD	157.877	2.808	NACPUD	163.546	3.04	NACPUD	151.95	3.482	NACPUD	157.029	3.079
NACPUD	172.075	2.903	NACPUD	161.947	3.44	NACPUD	158.901	3.073	NACPUD	166.104	3.079	NACPUD	159.229	2.808
NACQIS	166.735	3.233	NACQOY	163.452	3.069	NACQIS	154.6	3.154	NACQIS	160.113	3.178	NACQIS	157.7	3.002
NACSIX	177.349	3.207	NACSIX	165.103	3.497	NACQOY	155.114	2.715	NACQUE	153.241	3.249	NACREO	155.699	3.312
NADHUV	164.349	3.485	NADREP	171.148	3.275	NACSIX	155.688	3.407	NADHUV	151.162	2.925	NADHUV	163.715	2.925
NADTUM	167.529	2.814	NADTUM	176.141	2.808	NADRIU	168.211	3.344	NADSUI	165.779	2.865	NADSUI	173.516	3.414
NAFBEE	150.61	3.125	NAFHEM	164.346	2.889	NADTUM	155.751	3.49	NADTUM	158.899	2.937	NADYOL	170.156	3.293
NAFKAJ	156.781	3.157	NAFSAT	172.259	3.447	NAFJAF	160.069	3.225	NAFKAJ	163.621	3.022	NAFKAJ	168.736	2.947
NAFWIC	153.174	3.012	NAFWIC	155.421	3.024	NAFSAT	154.211	3.156	NAFSOH	172.797	3.097	NAFSOH	161.099	3.491
NAFXEA	151.051	3.41	NAFXEC	151.068	2.879	NAFWIC	171.802	3.032	NAFWIC	158.275	3.07	NAFWIC	168.24	2.938
NAGQAO0	163.19	3.006	NAGQER	161.653	3.182	NAFXUQ	170.729	3.491	NAGMOA	173.985	2.849	NAGQAO	162.759	3.012
NAGQER	165.983	3.205	NAGVOL	160.169	3.022	NAGQER	159.32	3.221	NAGQER	154.074	3.025	NAGQER	162.836	3.376
NAGXEZ	152.22	3.066	NAGXID	152.904	3.31	NAGVUM	175.32	3.179	NAGVUM	173.217	3.202	NAGWAT	164.119	2.977
NAHKUF	156.976	3.109	NAHPEW	157.386	3.199	NAGYAW	161.258	3.179	NAHJIU	153.37	3.207	NAHJIU	153.276	3.236
NAJLEQ01	168.662	3.436	NAJLEQ01	174.704	3.005	NAJGUC	159.906	3.012	NAJGUC	153.023	2.931	NAJJOY	150.233	3.121
NAJLEQ01	153.059	3.009	NAJLEQ01	152.448	2.83	NAJLEQ01	173.835	3.406	NAJLEQ01	171.497	3.069	NAJLEQ01	156.863	2.65
NAJPAR	153.877	3.017	NAJPAR	169.397	3.201	NAJLEQ01	170.484	2.786	NAJLEQ01	171.479	3.131	NAJPAR	150.384	3.017
NAJPEV	157.11	2.926	NAJPEV	157.479	3.235	NAJPAR	157.607	3.016	NAJPEV	152.093	3.063	NAJPEV	154.492	2.731
NAJPIZ	159.679	3.499	NAJQIA	174.175	3.082	NAJPEV	156.719	2.926	NAJPIZ	179.668	2.744	NAJPIZ	156.009	2.744
NAJROK	161.443	3.047	NAJXAZ	160.55	3.129	NAJROK	152.596	2.917	NAJROK	152.989	3.105	NAJROK	154.696	3.468
NAKSAY	151.165	3.077	NAKSAY	166.31	3.088	NAKFOW	174.528	3.046	NAKHEM	157.726	3.443	NAKHEP	165.659	3.431
NAKYOO	167.247	3.392	NAKZUX	171.804	3.009	NAKWUS	164.688	2.925	NAKXOM	174.018	3.362	NAKXOQ	151.822	3.492
NALNUK	168.283	3.494	NAMBAH	162.542	3.002	NALBAH	159.41	3.338	NALBAH	161.472	3.166	NALHUH	155.553	3.247
NAMJES	162.319	3.22	NAMJUK	159.678	3.127	NAMGIT	163.506	3.383	NAMJES	158.314	2.896	NAMJES	153.268	3.199
NAMWUU	155.18	3.264	NAMWUU	165.81	3.436	NAMQIB	158.444	3.075	NAMSIG	151.207	2.97	NAMWON	150.777	3.231

NAMWUU	156.217	3.279	NANCOX	167.33	3.088	NAMWUU	169.259	2.921	NAMWUU	158.38	3.279	NAMWUU	173.475	2.875
NANCOX	170.175	3.241	NANCOX	167.51	3.494	NANCOX	166.375	3.063	NANCOX	150.864	2.833	NANCOX	175.017	2.711
NANJEV01	162.089	3.242	NANJEV01	157.412	3.262	NANCOX	156.65	3.445	NANCOX	159.992	2.829	NANJEV01	152.792	3.111
NANJEV01	152.368	3.065	NANJEV01	151.113	3.005	NANJEV01	152.134	3.36	NANJEV01	151.011	3.213	NANJEV01	152.191	3.042
NANJEV01	158.973	3.123	NANJEV01	154.19	3.046	NANJEV01	156.241	2.977	NANJEV01	154.663	3.13	NANJEV01	150.502	3.194
NANJEV02	150.249	3.167	NANJEV02	160.971	3.203	NANJEV02	154.575	3.02	NANJEV02	158.607	3.092	NANJEV02	153.613	3.108
NANJEV02	152.388	3.377	NANJEV02	151.708	3.027	NANJEV02	156.706	3.225	NANJEV02	152.854	3.077	NANJEV02	151.741	3.249
NANJOF	168.11	3.39	NANJOF	151.341	3.016	NANJEV02	151.764	3.057	NANJEV02	151.54	2.978	NANJEV02	156.532	2.946
NANJOF	156.045	3.246	NANJOF	167.233	3.238	NANJOF	154.207	3.066	NANJOF	156.995	3.408	NANJOF	158.006	3.269
NANJOF	158.305	3.011	NANJOF	172.065	3.408	NANJOF	151.898	3.112	NANJOF	150.859	2.972	NANJOF	153.356	3.362
NANLOF	165.285	2.8	NANLOF	160.067	3.427	NANKOF	166.378	3.041	NANKUI	157.192	3.277	NANLOF	163.795	2.826
NANVAC	156.008	2.602	NANVAC	167.862	3.216	NANTEE	150.961	3.342	NANTEE	152.234	3.202	NANVAC	173.718	2.7
NANVOQ	166.972	3.437	NANVOQ	153.179	2.596	NANVAC	166.368	3.349	NANVIK	164.778	3.434	NANVIK	178.067	3.313
NAPGAP	152.078	3.224	NAPGET	150.337	3.37	NANVOQ	167.18	2.753	NANVOQ	170.101	3.316	NAPFAM	171.171	2.895
NAPPEC	156.69	2.85	NAPPEC	172.128	2.72	NAPGET	155.342	2.931	NAPPEC	173.08	3.132	NAPPEC	156.777	3.042
NAQDAL	158.752	2.952	NAQDAL	154.151	3.118	NAPYEM	150.491	3.419	NAPYEM	159.037	3.49	NAPYEM	161.047	3.268
NAQMUQ0	158.349	2.996	NAQNEB	150.658	3.388	NAQFAO	151.647	3.269	NAQFAO	163.016	3.045	NAQMUQ	158.179	2.99
NAQPUT	157.596	2.889	NAQSIK	169.002	2.756	NAQNEB	150.335	3.176	NAQNOM	150.708	2.87	NAQPUT	167.14	2.889
NARKIA	151.96	2.808	NARQAB	153.059	2.913	NAQSOQ	170.212	2.772	NAQVEH	150.38	2.999	NARBUG	167.765	3.305
NARQUV	153.874	2.939	NARRAC	153.023	2.932	NARQEF	153.238	2.929	NARQIJ	152.895	2.927	NARQOP	153.077	2.918
NARRUW	153.843	2.941	NARTUV	152.532	3.072	NARREG	153.181	2.937	NARRIK	153.001	2.941	NARROQ	153.152	2.959
NARYEO	155.784	2.98	NARYEO	164.178	3.278	NARTUV	167.368	3.072	NARVAD	153.931	3.312	NARYAI	154.234	2.98
NASBIU	157.123	3.377	NASNEA	171.582	2.914	NARZUD	169.887	3.408	NASBIU	170.749	3.291	NASBIU	162.001	2.901
NATLIG	170.289	2.928	NATLIG	173.623	3.327	NASNEA	177.506	3.354	NASQUW	175.609	2.541	NASQUW	179.868	2.541
NATLIH	166.389	3.207	NATLIH	154.907	2.881	NATLIG	161.892	2.902	NATLIH	170.961	2.943	NATLIH	156.578	3.207
NATPOO	177.566	3.486	NATPOO	152.399	3.486	NATMON	166.502	2.74	NATNEF	156.682	3.03	NATNIJ	153.548	3.23
NATVOT	156.759	3.242	NATVOT	174.866	3.081	NATPOO	164.384	2.985	NATVOT	151.301	3.283	NATVOT	161.706	3.116
NATVOT	163.029	3.32	NATVOT	153.078	2.98	NATVOT	151.382	2.815	NATVOT	153.77	2.815	NATVOT	168.748	3.09
NATXOW	170.066	3.474	NAVCIX	160.968	2.877	NATVOT	157.559	3.486	NATVOT	166.009	3.043	NATVOT	155.647	2.741
NAVCIX	154.656	2.995	NAVFEV	150.619	3.486	NAVCIX	160.54	3.494	NAVCIX	173.406	2.989	NAVCIX	171.772	3.332
NAVHIF	161.966	3.468	NAVHIF	151.245	3.393	NAVGOJ	154.147	3.357	NAVGOJ	164.488	3.328	NAVGOJ	172.71	3.219
NAVHOL	161.517	2.944	NAVJON	152.698	3.26	NAVHOL	158.044	3.382	NAVHOL	155.673	3.159	NAVHOL	166.991	3.175
NAVRUA	156.033	3.105	NAVTAG	160.849	3.196	NAVJON	162.835	3.26	NAVLOP	152.65	2.995	NAVRUA	161.869	3.239
NAVTAG	159.006	3.363	NAVTAG	156.125	2.987	NAVTAG	165.099	2.826	NAVTAG	157.275	2.733	NAVTAG	157.781	3.498
NAVTAG	170.268	3.246	NAWBOE	150.475	2.882	NAVTAG	169.723	3.246	NAVTAG	166.176	2.858	NAVTAG	154.154	2.836
NAWJUT	151.797	2.839	NAWJUT	174.846	3.241	NAWBOE	152.605	2.632	NAWBOE	151.152	2.896	NAWBOE	162.544	3.295
NAWJUT	156.867	3.381	NAWJUT	151.073	3.404	NAWJUT	168.614	3.309	NAWJUT	157.881	3.388	NAWJUT	157.939	3.346
NAWKAA	169.465	3.339	NAWKAA	155.451	3.364	NAWJUT	165.025	3.259	NAWKAA	150.273	2.854	NAWKAA	172.05	3.263
NAWPAE	158.685	3.064	NAWPAE	157.874	2.945	NAWKAA	156.451	3.388	NAWKAA	155.322	3.469	NAWKAA	168.627	3.192
NAWPAE0	158.786	3.066	NAWPAE0	158.758	2.975	NAWPAE	158.737	2.978	NAWPAE	168.303	3.151	NAWPAE0	157.689	2.938
NAWQAF	155.188	2.805	NAWQEJ	175.121	3.198	NAWPAE0	168.27	3.144	NAWQAF	156.125	2.918	NAWQAF	163.051	2.985
NAWQEJ	171.693	2.792	NAWQEJ	160.121	2.849	NAWQEJ	156.949	2.97	NAWQEJ	160.992	2.792	NAWQEJ	167.718	3.165
NAWQEJ	152.296	3.483	NAWQEJ	171.938	3.355	NAWQEJ	152.03	3.071	NAWQEJ	154.52	3.075	NAWQEJ	154.254	2.933
NAWQIN	162.841	3.224	NAWQIN	152.11	3.211	NAWQEJ	154.874	3.058	NAWQEJ	150.47	3.253	NAWQEJ	158.127	3.392
NAWQOT	171.765	2.952	NAWQOT	154.378	2.837	NAWQIN	163.09	3.378	NAWQIN	158.612	3.215	NAWQOT	164.481	2.941
NAWQUZ	161.364	2.867	NAWQUZ	154.041	2.646	NAWQOT	161.324	3.001	NAWQOT	158.438	2.802	NAWQOT	168.789	3.464
NAWSAH	165.76	2.894	NAWSAH	156.53	3.356	NAWQUZ	152.592	2.833	NAWSAH	151.253	3.435	NAWSAH	171.123	2.987
NAWSAH	169.056	2.686	NAWSAH	169.722	3.382	NAWSAH	156.46	3.423	NAWSAH	153.681	2.966	NAWSAH	170.393	3.34
NAWSAH	155.971	3.267	NAWSAH	169.198	3.022	NAWSAH	154.885	2.758	NAWSAH	172.04	2.905	NAWSAH	163.077	3.021
NAWSAH	155.25	3.496	NAWSAH	154.454	2.872	NAWSAH	157.188	2.795	NAWSAH	157.858	3.117	NAWSAH	165.874	3.449
NAXDAS	166.309	2.779	NAXDAS	176.413	3.315	NAWSAH	166.142	2.993	NAWXEP	162.056	2.882	NAWXUE	167.177	3.418
NAXHOJ	152.984	3.032	NAXHUP	175.268	3.089	NAXHOJ	169.661	3.489	NAXHOJ	160.589	3.334	NAXHOJ	164.021	3.408
NAXTUF	150.64	3.192	NAXXOB	172.921	3.49	NAXHUP	158.72	2.81	NAXPOS	173.376	2.967	NAXQOS	160.428	3.236
NAXXUH	157.178	3.181	NAXXUH	152.871	2.968	NAXXUH	171.569	3.181	NAXXUH	153.548	3.316	NAXXUH	154.458	2.82
NAZLIM	154.739	2.854	NAZLIM	161.34	2.797	NAXXUH	161.326	3.389	NAXXUH	165.816	2.968	NAZLIM	169.18	2.754
NAZQOY	176.827	3.161	NAZWEQ	151.519	3.047	NAZLIM	174.077	3.473	NAZQIS	166.645	3.478	NAZQOY	154.948	2.851
NEBCON	172.302	3.068	NEBDAA	169.246	2.785	NAZZAR	154.712	3.443	NAZZAR	152.476	3.41	NAZZEW	166.395	2.692
NEBVUO	166.807	3.263	NEBWOJ	153.85	2.86	NEBHOR	153.124	2.929	NEBLIT	150.813	2.837	NEBVUO	161.143	2.803
NECCIK	153.776	3.025	NECCIK	178.015	2.749	NEBXIE	153.794	3.283	NEBYOL	160.203	2.956	NECCIK	154.561	3.081
NECFAD	153.275	3.214	NECFOR	172.489	3.307	NECCOQ	155.536	3.213	NECFAD	154.538	2.925	NECFAD	161.167	3.065
NECFUX	165.152	3.09	NECFUX	155.985	3.484	NECFOR	150.008	3.439	NECFOR	152.365	3.31	NECFOR	173.355	3.331
NECGAG	151.598	2.648	NECGAG	157.817	2.714	NECFUX	156.524	2.979	NECGAE	164.893	3.376	NECGAE	162.932	3.23
NECGAG	172.21	3.05	NECGAG	160.041	2.88	NECGAG	165.679	3.318	NECGAG	156.941	3.318	NECGAG	166.904	3.05
NECGEK	162.591	2.775	NECGEK	158.239	2.833	NECGEI01	155.791	2.986	NECGEK	169.9	2.775	NECGEK	166.163	3.353
NECGIO	150.241	3.416	NECGIO	162.162	3.058	NECGEK	165.72	2.833	NECGEK	169.346	2.856	NECGEK	159.03	2.818
NECGIO	172.086	2.845	NECGIO	170.153	3.172	NECGIO	150.545	2.825	NECGIO	151.866	3.322	NECGIO	156.586	3.123
NECGIO	155.969	2.961	NECHAG	160.939	2.878	NECGIO	162.282	3.094	NECGIO	157.694	3.337	NECGIO	156.336	3.119
NECXIG	157.202	3.193	NECYED	162.462	3.058	NECHAG	150.973	3.261	NECLUF	160.687	3.291	NECXIG	157.057	3.065
NEDDOR	171.775	3.239	NEDDOR	151.323	2.966	NEDBIH	152.463	3.391	NEDDAD	172.214	3.49	NEDDAD	156.351	3.461
NEDHIR	156.181	3.399	NEDJUF	160.281	3.099	NEDDOR	178.22	2.966	NEDDUX	177.659	2.991	NEDHIR	155.415	3.321
NEDMAM	150.76	3.414	NEDMAM	150.45	2.965	NEDKOA	150.25	2.783	NEDMAM	162.85	2.953	NEDMAM	173.722	2.956
NEDREW	151.001	3.264	NEDREW	151.305	3.201	NEDMAM	150.029	2.848	NEDNUJ	153.494	3.352	NEDPOD	157.053	3.288
NEDRIA	157.73	2.809	NEDRIA	156.632	2.862	NEDREW	156.178	2.836	NEDREW	159.677	2.94	NEDRIA	151.889	3.266
NEDRUM	162.551	2.882	NEDRUM	171.183	2.853	NEDRUM	163.75	3.478	NEDRUM	164.882	2.998	NEDRUM	172.914	3.383
NEDSOI	168.909	3.29	NEDSOI	164.03	3.491	NEDRUM	165.893	2.936	NEDSOI	157.066	3.001	NEDSOI	158.343	3.394
NEDXAY	154.447	3.207	NEDXAY	150.317	3.352	NEDVAX	153.906	3.271	NEDWOL	166.594	2.85	NEDWUR	165.401	3.091
NEFBAE	177.05	3.024	NEFBAE	153.112	2.979	NEDXAY	153.849	3.207	NEDXAY	169.573	2.995	NEDYOO	151.921	3.346
NEFLAN	158.225	2.944	NEFLAN	155.912	3.066	NEFBAE	176.551	3.135	NEFBAE	174.596	3.135	NEFCOT	150.417	3.414
NEFLER	163.863	3.306	NEFLER	172.199	3.281	NEFLER	166.777	3.036	NEFLER	166.778	3.055	NEFLER	162.134	3.275

NEFNER	151.145	2.819	NEFQOH	155.519	2.96	NEFLER	170.728	3.295	NEFMIU	153.955	3.268	NEFNER	165.072	3.366
NEFRIA	153.982	3.336	NEFRIZ	153.796	2.982	NEFQOH	163.627	3.237	NEFQOH	159.545	2.908	NEFRIA	154.226	3.23
NEFTUQ	165.761	2.957	NEGBEH	153.818	3.364	NEFRIZ01	152.076	2.933	NEFTUQ	157.117	3.063	NEFTUQ	157.381	2.974
NEGHAK	167.544	3.221	NEGHAK	157.695	3.109	NEGCIL	171.161	3.094	NEGCIL	151.192	3.266	NEGHAK	173.855	3.203
NEGHEO	159.902	3.31	NEGHEO	150.98	2.593	NEGHAK	169.442	3.187	NEGHAK	153.588	3.101	NEGHEO	166.972	2.593
NEGHIS	150.021	3.442	NEGHOY	150.396	3.097	NEGHIS	155.666	3.498	NEGHIS	162.881	2.761	NEGHIS	169.244	2.771
NEGKET	164.71	2.837	NEGKET	158.55	3.194	NEGHOY	153.592	3.138	NEGHOY	161.942	2.639	NEGKET	156.309	2.967
NEGKIX	175.784	2.983	NEGKIX	158.334	3.298	NEGKET	164.596	3.091	NEGKET	165.934	3.418	NEGKET	154.862	2.916
NEGLEU	154.951	3.314	NEGLEU	158.547	3.177	NEGKIX	155.183	2.983	NEGLAQ	172.688	3.416	NEGLAQ	151.486	3.242
NEGLEU	164.817	3.224	NEGLIW	155.604	3.164	NEGLEU	158.994	3.451	NEGLEU	150.375	2.7	NEGLEU	160.964	3.178
NEGLIY	151.02	3.052	NEGLIY	156.809	3.005	NEGLIY	153.457	3.224	NEGLIY	165.011	2.769	NEGLIY	151.707	2.906
NEGLOE	153.856	3.03	NEGLUK	152.013	3.347	NEGLOE	158.769	2.744	NEGLOE	161.069	3.225	NEGLOE	158.651	3.437
NEGZAD	155.262	3.098	NEHFAK	151.167	2.901	NEGMAP	174.223	2.78	NEGMAP	161.158	2.844	NEGZAD	151.583	3.14
NEHFAK	170.013	2.952	NEHFAK	154.902	3.017	NEHFAK	150.779	3.183	NEHFAK	167.379	2.899	NEHFAK	159.149	3.117
NEHYUY	158.403	3.053	NEJPUO	151.008	2.994	NEHFAK	161.953	2.914	NEHSEA	157.115	3.143	NEHYOQ	153.536	3.133
NEJQEA	163.481	3.398	NEJQEA	153.096	3.106	NEJPUO	160.429	3.02	NEJPUO	158.205	3.493	NEJPUO	152.779	2.889
NEJQIE	162.171	3.069	NEJQIE	153.729	3.306	NEJQEA	170.185	2.842	NEJQEA	152.42	3.076	NEJQEA	165.002	3.388
NEJQIE	155.822	3.167	NEJQIE	151.911	3.066	NEJQIE	154.7	3.386	NEJQIE	160.607	2.902	NEJQIE	160.409	3.386
NEJRAW	162.928	2.695	NEJRAW	158.184	2.914	NEJQIE	167.93	3.267	NEJRAW	175.344	3.071	NEJRAW	172.763	3.082
NEJRAW	151.658	3.111	NEJWUX	152.749	3.009	NEJRAW	161.172	3.231	NEJRAW	172.571	2.725	NEJRAW	152.89	3.274
NEKNIE	169.722	2.931	NEKNOK	170.169	2.924	NEKNIE	167.837	2.931	NEKNIE	166.553	3.148	NEKNIE	151.327	3.148
NEKSII	151.927	3.261	NEKXUX	155.425	3.014	NEKNOK	163.858	3.29	NEKNOK	160.22	2.924	NEKNOK	161.705	3.29
NELFER	169.828	2.728	NELFER	156.208	3.133	NEKXUX	160.705	3.014	NELDIS	161.586	3.46	NELFAN	156.282	2.746
NELFER	159.282	3.195	NELFER	160.183	3.034	NELFER	164.385	2.852	NELFER	161.866	3.416	NELFER	152.334	3.247
NELMIE	177.58	2.782	NELVOP	161.72	2.915	NELGAM	158.534	3.342	NELKOF	169.42	3.097	NELKOF	154.36	3.088
NEMDAM	164.467	3.288	NEMDAM	163.594	3.4	NELYAF	157.805	3.154	NEMCUH	150.171	3.418	NEMCUH	159.212	3.414
NEMHOC	164.018	2.982	NEMHUI0	155.663	3.431	NEMHOC	164.295	3.363	NEMHOC	164.55	3.406	NEMHOC	150.549	3.008
NEMJIC	169.138	2.995	NEMJIC	159.445	2.965	NEMJIC	158.383	2.808	NEMJIC	152.942	3.183	NEMJIC	155.322	2.808
NEMJOI	151.236	3.496	NEMJOI	150.49	2.948	NEMJIC	151.126	3.183	NEMJOI	166.731	3.265	NEMJOI	168.662	3.352
NEMTEG	153.47	3.112	NEMTEG	153.496	3.369	NEMJOI	161.236	3.074	NEMKID	157.986	3.017	NEMTAC	151.42	3.006
NEMYAI	167.614	3.173	NEMYAI	160.334	3.128	NEMTEG	150.001	2.759	NEMVOU	165.176	3.079	NEMWEL	160.132	3.471
NEMYAI	161.799	3.212	NEMYAI	161.225	3.094	NEMYAI	173.834	2.808	NEMYAI	156.58	3.143	NEMYAI	172.154	3.094
NEMYEM	163.538	3.121	NEMYEM	154.919	3.396	NEMYAI	164.128	2.916	NEMYAI	151.036	3.052	NEMYAI	157.358	3.282
NEMYEM	163.505	2.987	NEMYEM	170.871	2.777	NEMYEM	154.58	3.228	NEMYEM	156.178	2.906	NEMYEM	163.766	3.435
NEMYEM	158.274	3.446	NEMYEM	157.185	3.409	NEMYEM	176.821	3.174	NEMYEM	158.965	3.261	NEMYEM	172.934	3.41
NEMYUA	165.496	3.38	NEMYUA	153.689	3.161	NEMYEM	151.889	2.769	NEMYEM	166.222	2.777	NEMYEM	158.331	2.736
NEMYUC	161.964	3.116	NEMYUC	168.554	2.91	NEMYUC	159.861	3.156	NEMYUC	162.611	2.768	NEMYUC	164.436	2.768
NENGUM	156.981	3.4	NENHUM	159.38	3.298	NEMYUC	153.51	3.271	NEMYUC	152.005	3.086	NENGUM	158.433	3.088
NENHUM	167.87	2.992	NENHUM	163.192	3.439	NENHUM	167.848	3.461	NENHUM	171.539	3.07	NENHUM	175.489	2.717
NENPII	150.835	3.495	NENPII	152.56	2.896	NENPII	173.72	3.113	NENPII	157.581	3.269	NENPII	178.469	2.926
NENPOO	159.148	3.39	NENPOO	177.593	2.947	NENPII	157.736	3.495	NENPOO	165.908	3.426	NENPOO	166.584	2.899
NEPHUP	162.995	3.01	NEPLOJ	151.304	2.935	NENQUT	155.629	3.44	NENVAH	160.619	2.81	NENVOT	155.969	3.435
NEPNEC	158.012	3.041	NEPNEE	169.922	3.462	NEPLOJ	159.309	2.802	NEPLOJ	172.879	3.252	NEPLOJ	150.025	3.189
NEPTOT	155.722	3.294	NEPTUB	168.581	3.051	NEPNIG	168.672	3.323	NEPQAA	150.582	2.866	NEPQAA	150.747	3.458
NEPVEM	175.245	3.278	NEPVIQ	154.849	3.394	NEPTUB	170.141	2.964	NEPTUB	174.135	3.051	NEPVEM	170.602	3.417
NEPVIR	165.251	2.866	NEPVIR	156.096	3.194	NEPVIQ	154.348	3.239	NEPVIQ	162.344	3.321	NEPVIQ	153.347	3.48
NEPVOW	151.708	3.334	NEPVUC	168.649	3.145	NEPVIR	160.89	3.215	NEPVOW	162.793	2.946	NEPVOW	160.293	3.284
NEPWUD	166.6	3.169	NEQDOG	165.785	2.984	NEPVUC	150.738	3.199	NEPVUC	169.421	3.291	NEPWEN	159.924	2.879
NEQDOG0	171.358	2.927	NEQDOG0	160.225	2.947	NEQDOG0	165.495	2.997	NEQDOG0	166.898	2.918	NEQDOG0	165.771	2.952
NEQTUB	154.875	3.36	NEQVIQ	156.48	2.991	NEQKAY	174.311	3.426	NEQPEE	160.093	3.387	NEQSIM	164.103	3.481
NEQZES	150.42	3.107	NEQZES	156.943	3.046	NEQVIQ	159.427	3.221	NEQWEO	165.01	3.413	NEQZES	154.184	3.175
NERFID	170.477	3.473	NERHOK	159.543	3.39	NERBUI	160.048	3.342	NERBUI	164.368	3.138	NERBUI	157.668	3.138
NERHOK	166.548	2.998	NERHOK	173.806	3.29	NERHOK	164.823	3.165	NERHOK	157.846	2.878	NERHOK	152.154	3.101
NERSUZ	153.798	3.139	NERSUZ	156.094	3.292	NERMUT	155.859	3.327	NERSAI	180	3.128	NERSUZ	155.854	3.391
NESCAS	160.735	3.326	NESCEV	175.832	3.409	NERSUZ	150.294	3.292	NERSUZ	176.216	2.867	NESCAR	155.999	3.023
NESHEA	159.015	3.311	NESHEA	159.739	2.998	NESDIY	169.942	3.021	NESDIY	153.443	3.421	NESHEA	154.342	2.802
NESJEB	153.32	3.313	NESLIH	159.509	3.408	NESHEA	163.512	3.027	NESHOK	169.777	3.198	NESJEB	156.575	3.067
NESSOW	157.518	3.431	NETFEA	155.418	2.896	NESLIH01	153.142	3.459	NESLIH01	154.262	3.233	NESMIH	152.698	2.98
NETLIK	151.866	3.18	NETNEJ	170.492	3.112	NETFEA	174.591	2.94	NETFEA	170.666	2.907	NETLIK	170.441	3.329
NEVHAB	157.377	2.888	NEVHAB	166.821	2.865	NETPOR	152.336	2.686	NETQUZ	151.673	3.167	NEVCEY	154.323	3.222
NEVLEI	155.859	3.102	NEVPUB	155.408	2.799	NEVLEI	157.76	2.858	NEVLEI	168.63	3.163	NEVLEI	159.146	2.858
NEVQAI	151.31	3.2	NEVQEM	154.99	3.224	NEVQAI	173.305	3.084	NEVQAI	150.216	2.978	NEVQAI	156.583	3.145
NEVQEM	173.65	3.17	NEVQEM	167.756	3.046	NEVQEM	155.187	3.431	NEVQEM	153.462	2.781	NEVQEM	161.947	2.976
NEVQUB	162.54	3.094	NEVQUC	173.9	3.1	NEVQEM	152.494	3.244	NEVQEM	159.069	3.15	NEVQUB	163.306	3.203
NEVREP	156.235	2.871	NEVRIR	176.188	2.893	NEVREP	155.182	3.346	NEVREP	157.067	3.302	NEVREP	158.933	3.014
NEWDIG	164.731	3.335	NEWDIG0	164.431	3.357	NEVRIR	164.173	2.884	NEVROX	151.469	3.176	NEVWOA	153.235	3.273
NEWKUV	170.418	3.295	NEWQUD	168.124	2.882	NEWGON	164.346	3.35	NEWKAD	162.764	3.172	NEWKAD	153.595	2.805
NEXNIO	156.949	2.839	NEXNIO	158.97	3.157	NEWVAQ	166.506	3.261	NEXKIN	164.871	3.197	NEXNIO	164.442	2.76
NEXNIO	150.4	2.834	NEXNIO	170.974	3.074	NEXNIO	153.176	2.76	NEXNIO	159.072	2.928	NEXNIO	166.356	3.235
NEXNIO	156.433	3.233	NEXNIO	167.11	2.791	NEXNIO	150.419	2.954	NEXNIO	164.709	2.778	NEXNIO	155.613	3.276
NEXNIO	159.816	2.954	NEXNIO	170.109	3.146	NEXNIO	170.82	2.792	NEXNIO	160.053	3.226	NEXNIO	161.156	3.116
NEXWUL	157.285	2.971	NEXWUL	178.978	2.98	NEXNIO	164.957	3.119	NEXWUL	156.209	2.785	NEXWUL	162.372	2.999
NEXWUL	151.702	3.465	NEXWUL	150.912	3.391	NEXWUL	176.062	3.192	NEXWUL	153.212	3.113	NEXWUL	159.302	2.97
NEXWUL	153.953	2.971	NEXWUL	161.688	2.9	NEXWUL	163.989	3.026	NEXWUL	173.327	3.293	NEXWUL	174.794	3.302
NEXWUL	176.837	3.466	NEXWUL	158.47	3.069	NEXWUL	165.413	2.591	NEXWUL	152.383	2.959	NEXWUL	156.055	3.215
NEXWUL	156.619	3.296	NEXWUL	160.722	3.466	NEXWUL	172.87	2.932	NEXWUL	164.097	2.914	NEXWUL	162.991	3.46
NEXWUL	165.674	2.89	NEXWUL	170.474	2.971	NEXWUL	151.3	3.247	NEXWUL	175.784	3.273	NEXWUL	164.235	2.894
NEXXUM	150.78	2.89	NEXXUM	156.125	3.104	NEXWUL	167.381	3.13	NEXWUL	151.897	3.14	NEXWUL	178.529	2.679
NEXXUM	169.512	2.883	NEXXUM	172.945	3.104	NEXXUM	151.608	3.413	NEXXUM	161.859	2.737	NEXXUM	153.82	2.786

NEXXUM	157.108	3.234	NEXXUM	153.056	3.366	NEXXUM	150.906	3.205	NEXXUM	150.133	3.269	NEXXUM	162.204	3.44
NEXXUM	150.955	3.102	NEXXUM	156.736	2.883	NEXXUM	165.667	2.941	NEXXUM	158.207	3.159	NEXXUM	151.022	3.045
NEYDIG	165.62	3.316	NEYDIG	167.998	2.867	NEYDIG	160.871	3.167	NEYDIG	170.914	2.852	NEYDIG	160.509	3.08
NEYDIG	153.185	3.112	NEYDUS	167.118	2.637	NEYDIG	169.545	3.277	NEYDIG	153.374	3.112	NEYDIG	159.18	3.199
NEYDUS	159.062	2.957	NEYDUS	158.941	3.275	NEYDUS	154.232	3.052	NEYDUS	168.385	3.345	NEYDUS	165.983	2.637
NEYFEE	164.66	2.597	NEYFII	157.245	3.091	NEYDUS	170.79	3.135	NEYDUS	152.833	3.301	NEYFAA	168.104	3.332
NEYPOY	167.742	3.052	NEYQAL	154.955	3.22	NEYFII	151.904	2.646	NEYFII	156.22	3.375	NEYKUX	160.996	3.397
NEYWEV	164.401	3.055	NEYWEV	151.595	3.06	NEYTUI	154.776	3.263	NEYVUK	173.399	3.389	NEYVUK	163.506	3.469
NEYWUJ	150.073	3.441	NEYWUJ	160.015	3.271	NEYWEV	154.73	2.806	NEYWEV	159.628	3.269	NEYWIZ	159.207	3.445
NEYXAS	151.25	2.984	NEYXAS	160.931	3.437	NEYXAS	151.526	3.241	NEYXAS	156.926	3.278	NEYXAS	166.726	3.239
NEYYEV	155.401	3.295	NEYYEV	157.252	3.287	NEYXAS	162.932	3.365	NEYYAR	161.036	3.087	NEYYEV	157.407	3.293
NEZBUS	153.904	2.907	NEZBUS	167.605	3.128	NEYYEV	168.742	3.361	NEZBUS	150.678	3.196	NEZBUS	156.336	3.015
NEZNUC	156.72	2.968	NEZNUC	163.392	3.211	NEZBUS	156.148	3.27	NEZBUS	176.905	3.078	NEZNUC	155.671	3.185
NEZPAK	154.043	3.092	NEZPAK	158.027	3.016	NEZNUC	159.348	2.926	NEZNUC	153.096	3.393	NEZPAK	177.981	3.478
NEZPAK	167.079	3.185	NEZPAK	152.657	2.821	NEZPAK	158.845	3.304	NEZPAK	167.678	2.933	NEZPAK	151.803	2.857
NEZYAT	161.288	3.05	NIBFEL	158.338	3.413	NEZSIW	151.848	3.358	NEZVOG	158.834	3.409	NEZXUL	173.562	3.368
NIBFUB	158.057	2.817	NIBGAI	164.027	3.198	NIBFEL	157.643	3.426	NIBFUB	155.395	3.073	NIBFUB	172.064	3.436
NIBHEM	170.248	3.156	NIBHEM	159.013	3.201	NIBHEM	171.887	3.269	NIBHEM	151.964	3.341	NIBHEM	156.087	3.151
NIBXUS	157.986	2.878	NIBYAZ	150.298	2.831	NIBJEQ	150.504	3.324	NIBTEA	151.348	3.426	NIBXUS	154.335	3.382
NICFIQ	161.472	3.018	NICFIQ	165.745	2.895	NIBYED	154.712	3.176	NICFEK	156.095	3.327	NICFIQ	155.972	2.984
NICGOY	153.535	3.188	NICGUE	157.164	3.168	NICFIQ	169.877	3.267	NICGEO	156.173	3.043	NICGEO	163.941	2.971
NICLUJ	154.382	2.984	NICNOD	158.753	3.467	NICGUE	157.37	3.381	NICHAL	169.365	3.306	NICHAL	157.025	3.167
NICSOJ	172.684	2.764	NICTAW	162.128	2.653	NICNUJ	170.196	2.974	NICNUJ	165.381	3.121	NICSIF	170.901	3.303
NICVIH	160.659	3.493	NIDHAL	162.914	3.231	NICTAW01	158.445	2.626	NICTIF	152.731	3.18	NICVIH	152.74	3.119
NIDHAL	151.045	3.115	NIDHOZ	160.635	2.924	NIDHAL	162.016	3.298	NIDHAL	158.145	3.298	NIDHAL	177.907	2.911
NIDJAN	155.728	2.773	NIDJAN	150.06	2.967	NIDHOZ	162.426	2.9	NIDHOZ	171.575	2.962	NIDHOZ	157.088	3.116
NIDJAN	167.085	2.957	NIDJIV	162.875	3.378	NIDJAN	150.245	2.905	NIDJAN	151.831	3.353	NIDJAN	162.098	2.804
NIDQOK	156.716	2.888	NIDTOL	155.318	2.9	NIDJIV	172.915	3.261	NIDQEA	155.659	2.926	NIDQOK	162.485	3.052
NIDTUR	160.551	2.803	NIDVAZ	150.506	3.01	NIDTOL	150.189	3.254	NIDTUR	153.45	3.11	NIDTUR	154.991	3.32
NIDVED	161.224	2.774	NIDVED	154.983	3.291	NIDVAZ	175.884	3.312	NIDVAZ	157.1	2.683	NIDVED	162.399	3.417
NIDVIH	159.466	3.341	NIDVUT	156.187	3.255	NIDVED	175.426	2.8	NIDVED	159.577	3.327	NIDVED	161.033	3.479
NIFFUD	151.696	3.241	NIFFUD	165.746	2.934	NIDZAF	158.06	3.178	NIDZIN	157.852	2.774	NIFBAG	163.294	2.979
NIFLAS	152.408	3.494	NIFLEW	171.78	3.141	NIFHET01	153.89	3.134	NIFJOE	158.403	3.499	NIFJOE	159.269	3.403
NIFSAA	157.189	3.009	NIFSAA	156.611	2.945	NIFLEW	151.694	3.141	NIFLUN	175.814	2.973	NIFLUN	153.133	2.987
NIGMUN	162.112	3.264	NIGNEW	159.127	3.283	NIFSAA	157.301	3.001	NIFSEE	172.099	2.956	NIGMOH	165.616	3.357
NIGPUS	153.195	2.814	NIGPUS	151.928	2.889	NIGNEW	156.152	3.163	NIGNEW	152.198	3.491	NIGNEW	171.03	3.163
NIHCIR	156.577	3.414	NIHCIR	157.945	2.82	NIGYUB	175.917	2.985	NIHCIR	161.124	2.73	NIHCIR	152.413	2.895
NIHDIS	173.604	2.831	NIHDIS	156.028	2.892	NIHCIR	164.582	2.837	NIHCIR	158.521	2.895	NIHCIR	172.135	2.896
NIHHES	151.353	3.329	NIHHES	160.16	3.312	NIHDIS	156.319	3.449	NIHDIS	159.042	2.725	NIHHES	158.356	2.922
NIHHES	173.337	2.871	NIHHES	150.082	3.157	NIHHES	158.641	2.97	NIHHES	150.167	3.292	NIHHES	165.97	3.453
NIHREE	160.898	3.149	NIHYIQ	153.02	3.131	NIHHEV	162.396	2.805	NIHJUM	153.424	3.455	NIHJUM	153.795	3.416
NIJDOC	163.579	3.116	NIJDOC	151.716	3.445	NIHYIQ	164.275	2.944	NIJDOC	158.356	2.842	NIJDOC	168.533	2.997
NIJFOC	156.679	3.432	NIJFOC	151.249	3.283	NIJDOC	164.311	3.336	NIJFEU	159.167	3.337	NIJFOC	171.071	3.184
NIJGAS	167.898	3.108	NIJJAV	150.883	3.237	NIJFOC	152.55	3.2	NIJFOC	167.326	3.34	NIJGAS	160.437	3.294
NIJLUP	153.294	3.284	NIJLUP	172.435	3.073	NIJJAV	158.731	3.23	NIJKOK	170.546	2.945	NIJKOK	157.346	3.037
NIKPON	163.815	3.349	NIKPON	169.203	2.975	NIJNOK	179.849	2.786	NIJNUQ	167.018	3.199	NIKLIF	151.766	3.014
NIKQAA	173.52	3.255	NIKQEE	166.093	3.274	NIKQAA	174.011	3.105	NIKQAA	171.16	3.273	NIKQAA	157.362	3.426
NIKTEJ	152.753	3.227	NIKTEJ	154.089	3.129	NIKQEE	171.649	3.011	NIKQEE	161.516	3.109	NIKTEJ	167.728	2.923
NILLAX	173.697	3.412	NILLAX	166.736	3.426	NIKTEJ	155.085	2.927	NIKTEJ	158.173	2.913	NILFAR	150.949	3.358
NILQUU	165.989	2.983	NIMBIY	170.898	3.445	NILLEB01	155.565	3.106	NILNAB	163.384	3.341	NILNAB	157.156	3.341
NIMCAR	165.855	3.009	NIMPOO	152.725	3.12	NIMBUK	162.073	3.022	NIMBUK	155.412	3.114	NIMCAR	152.488	3.394
NIMVOW	153.663	3.101	NIMZUI	158.803	3.345	NIMPOO	162.534	3.254	NIMTEI	153.648	3.33	NIMTEI	169.522	3.301
NINBIX	161.985	3.401	NINBIX	167.751	3.401	NIMZUI	162.462	3.436	NINBEV	151.712	2.836	NINBEV	159.971	2.935
NINLII	161.707	2.724	NINLOO	162.195	3.371	NINBOF	156.139	3.168	NINDUN	169.689	3.003	NINKUT	167.199	3.042
NINMEF	153.058	3.493	NINPEJ	173.678	3.258	NINLOO	157.653	3.205	NINMAB	173.423	2.954	NINMAB	172.149	2.877
NINTUZ	178.089	3.463	NINVUE	153.274	3.234	NINPUZ	155.21	2.983	NINPUZ	151.872	3.382	NINSUB	161.038	2.885
NIPDOH	162.286	3.016	NIPDOH	157.532	3.069	NINVUE	156.778	3.435	NINYEQ	157.784	3.04	NIPBAP	169.623	3.064
NIPTUC	177.215	3.124	NIPVIS	157.349	2.945	NIPFUN	161.294	2.897	NIPKUU	164.945	3.326	NIPKUU	152.508	2.831
NIQBEU	171.97	3.469	NIQDUQ	158.158	3.337	NIPVOY	177.348	2.88	NIPYUJ	154.394	3.383	NIPZOE	150.806	3.152
NIQFAY	154.538	3.289	NIQFAY	162.334	3.349	NIQDUQ	151.522	3.388	NIQDUQ	173.702	3.196	NIQDUQ	160.51	3.027
NIQHIG01	151.363	3.034	NIQHUQ	166.186	2.952	NIQHIG01	165.944	3.034	NIQHIG01	174.567	3.443	NIQHIG01	154.51	3.418
NIQQUD	153.605	3.445	NIQQUD01	159.641	3.161	NIQHUQ	166.02	2.988	NIQMIK	154.287	3.174	NIQQUD	159.79	3.162
NIRKOR02	164.254	3.144	NIRKOR02	157.074	3.256	NIQQUD01	153.621	3.434	NIQSOW	171.797	2.986	NIRDAX	160.007	3.339
NIRPOV	153.978	3.067	NIRPOV	168.025	3.315	NIRPOV	173.354	3.447	NIRPOV	161.08	3.289	NIRPOV	162.34	3.002
NIRQOV	164.221	2.913	NIRQUB	154.182	3.387	NIRQOV	161.09	2.764	NIRQOV	159.264	3.224	NIRQOV	153.32	2.763
NIRQUB	154.677	3.161	NIRRAI	154.124	3.075	NIRQUB	166.662	2.982	NIRQUB	156.836	3.122	NIRQUB	162.525	2.819
NIRWOC	154.867	2.78	NIRXUJ	164.779	3.123	NIRRIQ	157.388	3.419	NIRWOC	155.37	2.856	NIRWOC	168.685	2.702
NISNEI	167.071	3.26	NISNEK	154.639	3.025	NIRXUJ	159.892	3.123	NISBOH	154.69	3.314	NISBOH	160.49	2.807
NISZAR	177.805	3.413	NISZIZ	163.642	3.009	NISNOU	161.713	3.13	NISWOD	172.365	3.406	NISYUK	159.608	3.062
NITHON	160.534	3.204	NITHON	160.12	3.312	NISZOF	160.126	2.946	NISZOF	160.768	3.169	NISZOF	155.96	3.207
NIVBEA	173.362	3.277	NIVBEA	154.448	3.277	NITLEK	157.726	3.127	NITNEN	161.617	2.96	NITRUH	175.178	3.273
NIVMEO	172.167	3.389	NIVMEO	162.975	3.448	NIVGOQ	169.382	3.228	NIVJAH	153.505	2.976	NIVMEO	150.123	2.994
NIVTIW	151.312	3.385	NIWFIK	171.507	2.708	NIVQEO	162.04	3.093	NIVQEO	151.814	3.401	NIVTIW	150.662	3.3
NIWFIK	176.403	3.373	NIWFIK	163.233	2.951	NIWFIK	168.03	2.869	NIWFIK	166.84	2.913	NIWFIK	168.838	3.239
NIWFIL	172.754	3.079	NIWFIL	150.395	2.845	NIWFIL	155.266	3.121	NIWFIL	154.824	3.271	NIWFIL	153.298	3.166
NIWRAN	164.101	3.492	NIWSER	163.045	2.923	NIWFIL	152.275	2.883	NIWFIL	173.303	2.802	NIWQIU	164.226	3.435
NIXFEJ	154.916	3.237	NIXKIS	162.367	3.324	NIWSER01	163.045	2.923	NIWZOI	158.167	2.97	NIXFEJ	152.076	3.254
NIXNER	178.278	3.016	NIXREV	150.81	3.054	NIXKIS	162.252	3.24	NIXMIR	156.241	2.86	NIXMIR	162.863	3.433
NIXRIZ	177.333	3.282	NIXRIZ	164.059	3.024	NIXREV	165.563	2.978	NIXREV	175.573	3.226	NIXRIZ	160.998	3.488

NIXSIA	156.315	3.05	NIXSIA	151.274	3.242	NIXSIA	150.76	3.309	NIXSIA	152.838	3.247	NIXSIA	153.471	3.421
NIXSIA	152.414	3.164	NIXSIA	159.408	2.931	NIXSIA	152.104	2.816	NIXSIA	160.433	3.228	NIXSIA	153.014	2.799
NIYQUL	153.693	3.358	NIYSUL	173.413	3.056	NIYHIM	156.916	3.067	NIYJIR	154.97	3.102	NIYJIR	161.713	3.165
NIZHAI	165.218	3.307	NIZHAI	155.846	2.744	NIYVUQ	162.798	3.188	NIZHAI	153.895	2.744	NIZHAI	174.441	3.236
NIZPUK	160.266	2.763	NIZPUK	158.617	3.459	NIZHEM	160.041	2.752	NIZHEM	166.347	2.752	NIZPUK	161.264	3.389
NIZQAR	162.21	2.76	NIZQAR	156.62	3.418	NIZPUK	157.612	3.347	NIZPUK	168.525	3.266	NIZQAR	162.542	3.362
NIZQEV	158.595	3.428	NIZQEV	158.038	3.388	NIZQAR	168.238	3.334	NIZQEV	162.189	3.316	NIZQEV	161.736	2.788
NIZQIZ	157.767	3.418	NIZQIZ	157.961	3.385	NIZQEV	168.405	3.365	NIZQIZ	162.442	3.26	NIZQIZ	164.094	2.793
NIZQOF	177.508	3.366	NIZTIA	159.108	3.204	NIZQIZ	168.78	3.47	NIZQOF	174.878	3.067	NIZQOF	162.26	3.074
NIZTID	169.158	3.337	NIZVAU01	154.638	3.103	NIZTIA	175.103	2.945	NIZTID	165.871	3.016	NIZTID	150.208	3.245
NOBDAJ	155.741	3.497	NOBDAJ	156.711	3.095	NIZVUP	158.218	3.138	NIZXUS	174.162	3.436	NOBDAJ	172.335	3.445
NOBDAJ	151.672	3.381	NOBDEN	156.888	3.232	NOBDAJ	158.12	2.943	NOBDAJ	154.701	2.967	NOBDAJ	169.734	3.47
NOBDEN	152.178	3.29	NOBDEN	158.408	3.097	NOBDEN	152.655	3.1	NOBDEN	153.491	3.167	NOBDEN	174.55	3.49
NOBDEN	160.585	3.483	NOBDOX	166.594	2.966	NOBDEN	162.444	3.19	NOBDEN	157.981	3.235	NOBDEN	161.522	3.235
NOBDOX	162.506	2.789	NOBDOX	160.83	3.102	NOBDOX	150.56	3.431	NOBDOX	167.456	3.397	NOBDOX	155.085	3.217
NOBDOX	153.581	2.978	NOBDOX	151.442	3.065	NOBDOX	152.518	3.089	NOBDOX	163.187	3.156	NOBDOX	154.297	3.175
NOBKAQ	155.671	3.483	NOBKAR	160.277	3.345	NOBJIX	165.965	3.462	NOBJOF	164.301	3.083	NOBJOF	177.573	3.43
NOBKAS	158.441	3.162	NOBKIA	151.602	2.795	NOBKAR	162.335	3.25	NOBKAR	168.696	3.091	NOBKAR	151.025	3.469
NOBKOG	168.64	3.018	NOBQOM	171.562	2.97	NOBKIA	150.348	3.195	NOBKOG	150.514	3.479	NOBKOG	164.621	3.018
NOCNOL	171.256	3.257	NOCNOL	156.255	2.808	NOCGUK	168.41	2.824	NOCGUK	171.069	3.013	NOCMUQ	163.484	3.164
NOCSOR	154.884	3.151	NOCTAE	165.087	3.266	NOCSOR	157.978	3.038	NOCSOR	155.282	3.152	NOCSOR	158.952	3.03
NOCWEJ	177.142	2.894	NOCWEJ	152.666	3.067	NOCTAE	159.096	2.839	NOCTAE	157.118	3.035	NOCTAE	151.469	3.42
NOCWEJ	165.749	2.781	NOCWEJ	154.7	3.059	NOCWEJ	161.643	3.176	NOCWEJ	159.72	3.13	NOCWEJ	151.15	2.833
NOCWOS	174.211	2.881	NODBAJ	156.481	2.887	NOCWEJ	153.235	3.107	NOCWEJ	154.448	3.241	NOCWEJ	163.539	2.781
NODDUF	151.085	2.722	NODDUF	155.347	2.728	NODBAJ	157.796	3.313	NODBUG	156.663	2.854	NODDUF	167.778	2.728
NODFES	153.171	2.979	NODFES	156.173	2.862	NODFER	154.731	3.055	NODFES	153.015	3.498	NODFES	152.959	3.095
NODFES0	165.497	3.175	NODFES0	155.1	2.911	NODFES	165.125	3.115	NODFES0	154.963	3.116	NODFES0	151.909	3.02
NODWEM	151.284	3.068	NODWEM	166.939	3.313	NODLOK	177.925	2.827	NODLOK	162.988	3.104	NODRIK	155.134	2.875
NODXUD	155.349	3.121	NODXUD	167.013	2.896	NODXUA	165.177	3.382	NODXUD	168.994	2.668	NODXUD	167.69	2.896
NOFKOM	174.741	3.272	NOFSAF	151.365	3.008	NOFBAP	174.538	3.1	NOFCOE	154.777	2.937	NOFGAT	155.263	3.104
NOGZOB	166.638	3.131	NOGZOB	173.776	3.426	NOGBIY	163.728	3.206	NOGZOB	160.318	2.951	NOGZOB	176.477	3.139
NOHCEW	163.387	3.218	NOHDEX	156.411	2.948	NOHBES	169.751	3.041	NOHBES	171.418	3.433	NOHCEW	156.603	3.498
NOHDEX	151.647	3.404	NOHHOK	154.107	3.176	NOHDEX	153.613	3.446	NOHDEX	153.459	3.458	NOHDEX	159.114	2.948
NOHLUR	159.829	2.957	NOHPUW	177.005	3.013	NOHLUR	166.557	3.351	NOHLUR	153.592	2.84	NOHLUR	166.267	3.152
NOHRAG0	152.81	3.47	NOHRAG0	159.257	3.058	NOHQOT	150.919	2.819	NOHQOT	159.735	3.04	NOHRAG0	154.284	2.865
NOJHIE	153.428	3.194	NOJHIE	152.764	3.206	NOHREK	151.123	3.136	NOHREK	156.459	3.03	NOHRIM	177.765	2.829
NOJTAH	155.949	3.185	NOJTAH	174.932	3.082	NOJNOR	150.569	3.276	NOJNOR	175.191	3.229	NOJRIQ	152.381	3.097
NOJTEL	159.167	3.28	NOJXIT	155.3	3.498	NOJTAH	168.933	3.145	NOJTEL	152.427	3.297	NOJTEL	150.476	3.28
NOJXOZ	168.312	2.939	NOJXOZ	150.331	3.256	NOJXIT	154.093	3.297	NOJXIT	157.692	3.185	NOJXOZ	153.013	3.076
NOJZES	160.448	2.86	NOJZES	156.916	3.15	NOJXOZ	154.627	3.346	NOJYUG	167.963	3.328	NOJZES	166.738	3.086
NOJZIW	153.77	2.788	NOJZIW	154.964	3.319	NOJZES	161.253	2.884	NOJZIW	176.7	2.841	NOJZIW	166.152	3.154
NOJZIW	151.218	2.97	NOJZIW	169.811	2.952	NOJZIW	171.629	2.841	NOJZIW	166.083	3.296	NOJZIW	163.23	2.678
NOKHED	171.482	3.128	NOKLEG	174.053	3.306	NOKHED	170.203	3.045	NOKHED	175.339	2.694	NOKHED	158.962	2.694
NOKPEI	152.401	3.24	NOKPEI	155.853	3.116	NOKLEG	170.557	3.403	NOKPEI	159.045	3.301	NOKPEI	150.862	3.362
NOKSEP	158.376	2.869	NOKSEP	156.496	3.337	NOKQAI	159.857	2.911	NOKSAL	159.031	3.007	NOKSAL	160.79	3.246
NOKXUH	170.024	3.091	NOKXUH	156.619	3.251	NOKSEP	163.18	3.299	NOKSEP	152.707	3.352	NOKSUB	161.1	3.316
NOLKUU	156.731	3.481	NOLKUU	157.092	2.781	NOKYES	155.378	3.157	NOKYES	164.428	3.108	NOKZOG	150.387	2.985
NOLXUI01	167.987	3.138	NOMBAU	163.031	2.878	NOLNUZ	168.254	3.18	NOLROW	152.976	2.856	NOLXUI	170.629	3.071
NOMBOH	150.084	3.069	NOMBOH	161.461	3.359	NOMBAU	160.16	3.381	NOMBOH	151	3.11	NOMBOH	160.098	3.395
NOMKEI	164.104	3.165	NOMKEI	152.87	3.051	NOMBOH	161.746	2.887	NOMKEI	162.769	3.025	NOMKEI	175.104	3.399
NOMNOW	150.785	3.028	NOMNOW	152.904	2.985	NOMKEI	154.072	3.259	NOMKEI	171.368	3.361	NOMLAD	157.26	2.797
NOMRUE	173.392	3.22	NOMRUE	160.911	2.651	NOMNOW	154.953	3.187	NOMRUE	169.819	3.393	NOMRUE	163.796	3.029
NOMYAS	163.223	3.229	NOMYAS	152.817	2.561	NOMYAS	161.626	2.652	NOMYAS	151.105	2.917	NOMYAS	171.128	2.548
NOMYAS	171.97	2.788	NOMYAS	162.668	2.952	NOMYAS	155.021	3.463	NOMYAS	155.872	3.471	NOMYAS	174.815	3.139
NOMYAS	155.532	3.07	NONGOO	152.247	3.218	NOMYAS	159.173	3.463	NOMYAS	153.458	2.767	NOMYAS	155.01	3.2
NONJUX	156.13	2.736	NONKAE	156.108	2.899	NONJIL	156.034	2.669	NONJIL	153.774	2.669	NONJOR	157.459	3.13
NONPUD	165.689	3.294	NONSIW	156.636	2.993	NONPUD	150.518	3.295	NONPUD	162.895	3.096	NONPUD	168.771	3.471
NOPQIV	163.728	2.746	NOPQOB	165.785	2.891	NONYIY	167.119	3.307	NONYIY	175.043	3.093	NOPQIV	155.365	2.746
NOPQUH	156.359	2.977	NOQBAA	163.571	3.285	NOPQOB	150.343	2.806	NOPQOB	162.615	3.098	NOPQUH	154.207	3.048
NORBEF	150.445	3.313	NORBEF	168.186	2.926	NOQCEC	163.167	3.151	NOQMEP	160.87	2.988	NOQNUG	164.296	3.43
NORQEU	165.351	3.012	NORWAS	159.045	3.093	NORNIT	155.271	3.351	NORPEP	163.144	3.136	NORQEU	159.292	3.338
NOSDEI	152.6	3.446	NOSDEI	171.759	3.179	NOSCOR	154.532	3.29	NOSCOR	166.761	2.926	NOSCOR	174.232	3.15
NOSRIZ	163.295	3.318	NOSRIZ	167.948	3.406	NOSDUY	156.257	3.472	NOSNOY	170.198	3.078	NOSNOY	151.762	3.431
NOTJAL	157.341	3.127	NOTJEP	150.421	3.194	NOSRIZ	153.483	3.249	NOSZAY	160.05	3.366	NOTBUX	152.743	3.467
NOTKUE	164.86	3.056	NOTNIT	156.734	3.31	NOTKUE	163.581	3.111	NOTKUE	158.765	3.056	NOTKUE	158.522	3.384
NOTPUK	162.273	3.304	NOTPUK	157.961	2.835	NOTNIT	158.871	3.08	NOTNOC	150.214	3.328	NOTPUK	157.592	3.154
NOTZAB	176.425	3.016	NOTZAB	166.47	3.028	NOTPUK	150.314	2.835	NOTPUK	150.718	3.5	NOTPUK	151.375	2.841
NOVFIP	150.703	2.994	NOVMAO	162.907	3.445	NOTZAB	157.066	3.49	NOVFIP	164.588	2.851	NOVFIP	152.379	2.976
NOVQOI	165.643	3.167	NOVWIG	176.661	2.775	NOVQAU	167.965	3.281	NOVQOI	168.392	2.991	NOVQOI	159.956	2.972
NOVWIG	155.177	3.447	NOVWIG	156.192	3.184	NOVWIG	165.789	2.821	NOVWIG	163.431	2.868	NOVWIG	161.668	3.345
NOVWIG	164.759	3.158	NOVWIG	157.431	2.745	NOVWIG	152.169	3.049	NOVWIG	161.672	3.222	NOVWIG	156.337	2.89
NOVWOM	175.823	2.864	NOVWOM	167.874	2.775	NOVWIG	177.841	2.814	NOVWOM	154.135	2.988	NOVWOM	168.475	3.062
NOVXON	158.533	2.954	NOVYAA	170.139	2.798	NOVWOM	175.082	2.754	NOVWOM	171.185	2.887	NOVXON	159.575	3.198
NOVYEE	159.178	2.831	NOVYEE	163.451	3.046	NOVYEE	162.33	3.073	NOVYEE	160.539	3.227	NOVYEE	153.306	2.946
NOVZUV	155.656	3.4	NOVZUV	150.229	3.018	NOVZEF	155.728	3.173	NOVZEF	160.808	3.222	NOVZEF	162.389	2.931
NOWBIM	162.356	3.333	NOWBIM	162.038	3.25	NOVZUV	157.046	3.462	NOVZUV	156.016	3.37	NOWBIM	157.205	3.333
NOWBOS	150.821	2.808	NOWBOS	157.943	3.315	NOWBIM	162.978	3.493	NOWBOS	172.608	3.34	NOWBOS	165.575	3.202
NOWHUE	171.234	3.273	NOWHUE	152.552	2.937	NOWBOS	169.594	3.005	NOWFEN	157.651	3.169	NOWHUE	171.556	2.946
NOWJUG	161.964	3.022	NOWJUG	163.692	2.647	NOWJOA	157.39	2.783	NOWJOA	167.896	3.277	NOWJOA	165.16	3.059

NOWWED	162.42	3.306	NOWWED	151.29	3.306	NOWKAN	161.014	3.443	NOWKAN	163.594	2.888	NOWKAN	170.831	3.16
NOXBOU	157.746	3.383	NOXBOU	164.585	3.359	NOWWED	150.221	3.365	NOWWED	164.393	3.365	NOXBOU	160.756	2.695
NOXDOW	162.093	2.858	NOXDOW	164.32	2.768	NOXBOU	162.755	3.179	NOXBOU	161.765	3.053	NOXBOU	151.171	3.25
NOXWEF	167.918	3.451	NOXXUV	164.879	3.347	NOXPIC	160.803	3.383	NOXSOK	151.201	3.161	NOXSOK	150.349	3.146
NOYYOT	150.635	3.185	NOYYOT	164.182	3.117	NOXZEH	157.691	3.053	NOYGUD	161.943	2.87	NOYGUD(	163.468	2.878
NOYYOT	162.832	3.129	NOYYOT	151.659	3.071	NOYYOT	167.243	3.064	NOYYOT	158.729	2.868	NOYYOT	160.043	3.234
NOZVUX	165.539	3.149	NOZVUX	178.184	3.135	NOYYOT	157.571	3.307	NOYYOT	152.203	3.488	NOZVOR	155.874	3.46
NPOFNP0	151.016	3.442	NTNFBP1(	163.529	3.058	NOZWAD	157.885	2.854	NOZWAD	151.928	2.854	NOZWEI	154.413	3.274
NTNFBP1(	173.026	3.49	NUBDAP	159.746	3.049	NTNFBP1(	152.115	3.018	NTNFBP1(	165.378	2.883	NTNFBP1(	151.399	3.043
NUBROU	160.373	3.457	NUBROU	152.065	2.912	NUBGIA	159.236	3.458	NUBLOO	160.193	3.35	NUBLOO	154.102	3.451
NUBYIU	160.443	2.975	NUBYIU	158.542	2.957	NUBSIM	159.978	3.466	NUBSIM	162.304	3.144	NUBSIM	157.249	3.135
NUBYIU	170.655	3.189	NUBYIU	174.992	2.86	NUBYIU	169.05	2.769	NUBYIU	157.93	2.731	NUBYIU	151.285	2.837
NUCMAA	158.193	3.439	NUCMAA	170.542	2.925	NUBYIU	169.529	3.377	NUBYIU	174.645	3.032	NUCMAA	168.26	3.431
NUCMAA	168.592	2.925	NUCMAA	169.804	3.362	NUCMAA	150.436	3.342	NUCMAA	151.606	3.431	NUCMAA	172.447	3.193
NUDCEW	165.451	3.164	NUDCEW	160.266	3.171	NUCMAA	162.108	3.047	NUCMAA	164.832	3.395	NUCXOC	155.914	3.28
NUDNIM	171.305	3.238	NUDNUY	152.217	2.987	NUDJIJ	161.682	2.909	NUDKEG	157.568	2.823	NUDNIM	157.315	3.492
NUFNEL	173.471	2.91	NUFNEL	167.564	3.386	NUDNUY	161.613	2.965	NUDNUY	160.357	3.336	NUFNEL	164.968	3.128
NUFXOF0	160.384	2.743	NUFXOF0	155.938	3.219	NUFXOF	168.043	2.877	NUFXOF	156.16	3.215	NUFXOF	160.282	2.743
NUGPIP	156.692	3.487	NUGPIP	150.074	3.157	NUFXOF0	168.003	2.881	NUGPIP	172.879	2.936	NUGPIP	163.813	2.813
NUGVEQ	161.327	3.035	NUGVEQ	172.122	3.489	NUGPOV	156.825	2.948	NUGPUB	152.02	3.181	NUGSUF	155.107	3.122
NUHDOL	153.985	3.142	NUHDOL	159.638	3.205	NUHDOL	159.903	3.13	NUHDOL	165.539	3.193	NUHDOL	153.843	3.092
NUHYIZ	156.31	3.23	NUHYOF	162.949	2.758	NUHDOL	162.481	3.257	NUHHED	154.012	3.449	NUHRAN	159.842	3.492
NUJGAE	162.186	2.751	NUJGEI	156.864	3.444	NUJFOO	151.156	2.911	NUJGAE	166.613	3.143	NUJGAE	152.329	3.143
NUJLAH	172.032	3.019	NUJLIR	157.548	3.388	NUJGEI	161.665	3.157	NUJGEI	174.184	3.209	NUJGEI	153.837	2.803
NUJNEP	154.957	3.278	NUJNEP	161.237	3.133	NUJLIR	150.864	2.855	NUJNEP	154.555	3.439	NUJNEP	153.183	3.282
NUJRAM	159.867	3.262	NUKMAL	161.972	2.878	NUJNEP	157.22	2.883	NUJPAN	156.104	3.178	NUJRAM	153.233	3.327
NUKSOD	163.642	2.75	NUKSOD	153.561	2.603	NUKMAL	168.453	2.878	NUKROA	158.666	3.481	NUKROA	154.535	3.221
NULYIF	151.302	3.094	NUMCOQ	156.036	3.352	NUKSOD	151.562	2.663	NUKVIB	161.903	3.186	NULGAF	162.993	2.968
NUMGEH(	168.536	3.401	NUMLAL	151.212	3.288	NUMCUW	152.908	2.937	NUMCUW	152.919	2.933	NUMGEH(	150.423	3.214
NUMNER	164.157	2.828	NUMPUG	152.491	2.872	NUMLAL	170.962	3.21	NUMLAL0	150.885	3.269	NUMLAL0	170.813	3.193
NUMQAQ	157.319	2.662	NUMQAQ	163.378	3.411	NUMPUG	153.288	2.762	NUMQAQ	156.875	2.662	NUMQAQ	172.325	3.209
NUMQUK	153.002	3.014	NUMQUK	172.427	3.358	NUMQUK	151.536	3.19	NUMQUK	169.376	2.941	NUMQUK	154.237	2.963
NUMVUO	174.288	2.781	NUMXOL	156.106	3.468	NUMQUK	166.322	2.684	NUMVUO	170.464	3.014	NUMVUO	156.392	3.049
NUMYOM	155.308	3.417	NUMZAZ	152.687	3.301	NUMXOL	166.096	3.261	NUMYOJ	159.432	3.454	NUMYOJ	169.975	3.379
NUNJAI02	169.367	3.439	NUNMAL	155.861	2.922	NUMZAZ	169.15	2.969	NUMZIH	156.67	3.182	NUNJAI02	155.615	2.849
NUPCOT	151.582	3.379	NUPDAG	152.354	3.396	NUNMAL	173.634	3.152	NUNMAL	161.546	3.152	NUNMAL	162.846	3.1
NUPJEP	164.565	3.467	NUPJEP	154.577	2.89	NUPDEK	166.968	3.297	NUPGIR	160.258	3.359	NUPGIR	150.23	2.943
NUPSUM	172.778	3.083	NUPSUM	153.312	2.907	NUPJIT	155.263	3.025	NUPSUM	155.99	2.859	NUPSUM	151.13	2.907
NUPSUM(	172.835	3.096	NUPVUP	155.357	3.068	NUPSUM0	150.84	2.913	NUPSUM0	153.467	2.913	NUPSUM0	155.588	2.873
NUPVUP	157.132	3.263	NUPVUP	154.6	3.411	NUPVUP	150.977	2.986	NUPVUP	150.846	3.21	NUPVUP	169.773	3.411
NUQNIW0	150.225	2.739	NUQNIW0	175.489	3.224	NUPVUP	167.36	3.043	NUPVUP	171.107	3.263	NUQGIQ	158.865	3.078
NUQNOC(	156.173	3.396	NUQNOC(	169.078	3.206	NUQNIW0	159.758	3.388	NUQNIW0	171.289	3.157	NUQNOC(	171.173	3.213
NURREA	168.256	3.118	NURVON	150.025	3.428	NUQNOC(	158.094	3.399	NUQQIB	167.6	3.464	NURNOH	160.763	3.331
NURXON	164.861	2.985	NUSFOA	152.719	2.906	NURWAA	154.032	2.707	NURWAA	161.38	3.421	NURXON	157.116	3.089
NUSREZ	168.634	2.944	NUTHAO	171.379	2.97	NUSKOE	155.44	3.055	NUSQOL	158.642	3.233	NUSREZ	156.931	2.944
NUTXEJ	174.285	3.474	NUTXEJ	157.279	3.219	NUTHAO	153.451	2.84	NUTPAW	159.316	3.04	NUTVEG	164.902	3.001
NUVFIU	158.064	2.942	NUVFIU01	158.676	2.91	NUVCUF	174.249	3.178	NUVDIU	159.509	3.192	NUVFAO	167.138	3.034
NUVMUN	157.241	3.038	NUVMUN	161.672	3.136	NUVHUL	166.279	2.78	NUVKIC01	177.963	3.253	NUVMUN	165.722	2.826
NUVNUP	150.275	2.89	NUVVOS	169.63	3.292	NUVNUP	173.217	2.882	NUVNUP	161.605	3.072	NUVNUP	160.409	3.272
NUVVOS	164.806	3.331	NUVVUY	150.287	3.23	NUVVOS	173.563	2.918	NUVVOS	157.501	2.718	NUVVOS	170.742	2.894
NUWBIS	174.754	2.866	NUWBIS	162.507	3.097	NUVVUY	167.298	2.862	NUVVUY	151.452	2.961	NUVZEN	163.113	3.28
NUWDUE	154.871	3.029	NUWDUE	155.217	3.215	NUWBIS	160.934	3.311	NUWDER	150.018	3.212	NUWDUE	156.156	2.852
NUWFAM	156.788	3.351	NUWFAM	159.142	3.037	NUWDUE	171.57	3.01	NUWFAM	153.283	2.783	NUWFAM	153.962	2.941
NUWHEW	159.673	2.874	NUWHEW	156.612	3.361	NUWFAP	156.979	3.125	NUWHEW	150.195	3.091	NUWHEW	157.902	3.245
NUWLEW	153.924	3.418	NUWLEW	154.048	2.921	NUWHIA	158.636	3.107	NUWLEW	152.706	3.229	NUWLEW	150.435	2.947
NUWLIA	158.472	3.089	NUWLUN	171.613	2.668	NUWLEW	164.448	2.892	NUWLIA	161.825	2.803	NUWLIA	152.24	2.913
NUWMUN	151.285	3.282	NUWMUN	159.737	3.066	NUWLUN	164.606	2.867	NUWLUN(	177.316	2.747	NUWLUN(	158.749	2.94
NUWMUN	162.475	3.314	NUWMUN	158.805	3.314	NUWMUN	157.446	3.066	NUWMUN	172.57	2.975	NUWMUN	168.434	2.905
NUWTUW	168.609	3.152	NUWVEG	154.321	3.338	NUWTAC	167.748	3.045	NUWTAC	150.463	3.173	NUWTAC	165.363	3.233
NUXFEV	170.337	3.007	NUXFEV	173.578	2.989	NUWVEG	156.507	2.812	NUWVIK	151.245	3.108	NUXFEV	164.231	3.005
NUXFIZ	159.881	2.846	NUXFIZ	164.062	3.216	NUXFEV	171.199	2.87	NUXFIZ	173.313	3.302	NUXFIZ	161.195	2.787
NUXFIZ	164.496	3.094	NUXLEZ	163.934	2.82	NUXFIZ	158.163	3.469	NUXFIZ	171.981	3.149	NUXFIZ	164.872	3.005
NUXYEM	155.955	3.088	NUXYEM	165.078	3.431	NUXLEZ	153.506	3.146	NUXLOJ	176.855	3.384	NUXMAW	162.822	3.321
NUYXAG	151.474	3.356	NUYXAG	150.958	2.737	NUXZIT	151.619	2.678	NUYNIE	156.286	2.95	NUYNIE	155.033	2.95
NUYXAG	157.405	2.737	NUYXAG	162.393	2.988	NUYXAG	165.067	3.146	NUYXAG	162.713	3.313	NUYXAG	150.656	2.847
NUYXIO	155.788	3.413	NUYXIO	153.401	3.336	NUYXAG	170.055	3.238	NUYXIO	154.911	3.331	NUYXIO	152.291	3.092
NUZKOL	163.844	3.435	NUZKOL	151.943	2.783	NUYXIO	163.44	3.234	NUYXIO	153.967	2.819	NUYXIO	173.232	3.264
NUZKOL	155.231	3.307	NUZKOL	163.033	3.008	NUZKOL	169.571	3.435	NUZKOL	161.041	2.925	NUZKOL	151.763	2.783
NUZMIF	164.078	3.081	NUZMIF	161.505	3.044	NUZKOL	153.809	2.78	NUZKOL	152.857	2.901	NUZKOL	150.55	3.293
NUZQEF	152.202	3.202	NUZQEF	164.481	3.021	NUZMIF	165.469	3.317	NUZMIF	167.706	3.319	NUZPAZ	172.902	3.292
NUZQEF	156.122	3.235	NUZQIJ	150.081	2.918	NUZQEF	150.99	3.235	NUZQEF	162.551	2.986	NUZQEF	158.39	3.202
NUZQIJ	157.822	3.446	NUZQIJ	153.261	2.987	NUZQIJ	152.998	3.307	NUZQIJ	153.042	3.109	NUZQIJ	166.482	3.426
NUZTUA	161.536	2.978	NUZTUA	157.727	3.437	NUZREJ	162.61	3.049	NUZREJ	152.047	3.35	NUZTUA	150.408	3.14
NUZTUA	151.369	3.204	OBABAY	156.608	2.873	NUZTUA	167.391	2.673	NUZTUA	158.859	2.92	NUZTUA	161.013	3.22
OBAHEI	169.666	2.967	OBAHEI	160.797	2.942	OBABAY	169.116	2.873	OBAHEI	161.326	2.893	OBAHEI	156.026	2.715
OBANUC	174.082	2.71	OBANUC	156.49	3.343	OBAHEI	161.733	2.869	OBANEM	160.443	3.015	OBANUC	161.537	2.855
OBANUC	169.329	2.917	OBANUC	167.422	2.913	OBANUC	150.102	3.41	OBANUC	166.664	3.387	OBANUC	171.818	3.434
OBANUC	177.896	3.486	OBANUC	169.957	3.335	OBANUC	167.503	2.823	OBANUC	172.889	3.194	OBANUC	172.73	3.119
OBANUC	162.336	3.227	OBAPOA	171.405	3.441	OBANUC	152.001	2.963	OBANUC	157.624	3.138	OBANUC	158.113	3.15

OBARUG	165.519	2.972	OBARUG	164.035	3.199	OBAQAN	157.852	3.236	OBAQAN	170.903	3.462	OBARUG	155.493	3.18
OBEPES	157.482	2.87	OBEWAW	152.494	3.123	OBAVEV	158.473	3.469	OBAWAT	157.633	2.907	OBEJOV	157.357	3.223
OBIDEM	174.481	3.402	OBIDEM	157.506	3.084	OBEWAW	150.076	3.393	OBIDEM	162.779	2.914	OBIDEM	152.306	3.121
OBIKET	173.283	3.15	OBIRUP	162.722	2.97	OBIDEM	157.787	3.328	OBIKAP	168.14	2.803	OBIKET	154.654	3.015
OBIYUW0	155.063	3.477	OBOBUE	163.697	2.867	OBIVEC	154.858	3.389	OBIXUV	162.941	3.212	OBIYUW	155.123	3.475
OBOTAE	155.037	2.956	OBOTAE	171.404	3.379	OBOBUE	152.926	2.954	OBOPAZ	154.159	3.358	OBOPAZ	164.979	3.115
OBOTEI	163.548	3.475	OBOTUY	158.613	2.873	OBOTEE	152.609	3.23	OBOTEI	165.954	2.931	OBOTEI	164.886	3.232
OBOYOV	168.248	3.374	OBUDEV	166.306	3.123	OBOVEK	151.453	3.312	OBOYOV	158.132	3.189	OBOYOV	161.166	3.112
OBULIH	163.625	3.199	OBULIH	164.293	3.174	OBUDEV	152.311	3.25	OBUGEX	168.203	3.264	OBUKUS	164.397	3.263
OBUWUE	163.1	3.374	OBUWUE	160.385	3.068	OBUMUX	150.995	3.212	OBUNAE	160.356	3.469	OBUWUE	156.067	3.232
OCACEA	159.724	3.012	OCADIH	158.696	3.139	OBUZOA	151.691	2.921	OBUZOA	170.193	3.46	OCACEA	162.523	3.423
OCAPEP	153.614	3.225	OCAQOZ	163.838	2.942	OCADIH	150.722	3.196	OCANEP	150.456	3.39	OCAPAN	154.272	3.239
OCAXAU	155.87	3.013	OCEGEM	150.25	3.401	OCAQOZ	159.507	2.97	OCAVAT	164.064	3.269	OCAXAU	151.735	3.013
OCEGEM	150.589	2.958	OCEGEM	162.626	3.299	OCEGEM	160.524	2.937	OCEGEM	159.73	3.187	OCEGEM	177.248	2.839
OCEGIQ	150.207	3.287	OCEGIQ	159.131	3.354	OCEGEM	158.526	3.201	OCEGIQ	151.819	2.806	OCEGIQ	162.989	3.287
OCEHEJ	164.997	2.966	OCEHEJ	154.773	3.247	OCEGIQ	167.821	2.806	OCEGIQ	163.827	3.287	OCEHEJ	155.115	3.177
OCEHUD	161.553	2.751	OCELIR	156.87	3.4	OCEHEJ	163.14	2.911	OCEHEJ	173.104	2.868	OCEHUD	161.865	2.863
OCESIY	158.477	3.207	OCESIY	156.085	2.988	OCELIR	165.334	2.896	OCEQOD	155.777	3.269	OCEQOD	169.886	3.045
OCICEL	164.002	3.119	OCICEL	157.784	2.991	OCFCHX1	154.086	3.485	OCFCHX1	150.9	3.281	OCFCHX1	158.904	3.341
OCIFIQ	168.452	2.737	OCIFIQ	154.999	3.269	OCICOV	169.525	3	OCICOV	158.503	2.969	OCIFIQ	153.079	3.288
OCIFOW	151.208	2.962	OCIFOW	157.833	3.137	OCIFIQ	151.003	3.116	OCIFIQ	154.847	2.906	OCIFOW	158.486	3.24
OCIHOY	168.876	3.124	OCIHOY	158.252	3.283	OCIFOW	154.236	3.137	OCIFOW	168.902	3.039	OCIHOA	156.959	2.943
OCIHUE	152.155	2.945	OCILET	174.485	3.378	OCIHOY	161.636	2.779	OCIHUE	169.716	3.062	OCIHUE	157.027	2.84
OCIYOQ	156.134	2.781	OCIYOS	158.04	3.288	OCIPEW	153.96	2.996	OCIYOQ	150.816	2.998	OCIYOQ	170.278	3.073
OCOGOF	152.915	3.261	OCOGOF	155.173	3.105	OCIZEI	161.601	3.481	OCOGOF	155.238	3.238	OCOGOF	153.891	3.202
OCOHIA	162.701	3.137	OCOWUY	167.87	2.955	OCOGOF	159.081	2.899	OCOGOF	157.199	3.017	OCOGOF	162.67	3.017
OCUCUL	164.493	2.967	OCUCUL	150.312	2.932	OCUCIZ	150.557	3.027	OCUCIZ	152.239	3.323	OCUCIZ	151.745	2.871
OCUDAS	156.861	2.806	OCUDAS	158.484	2.974	OCUDAS	160.042	2.624	OCUDAS	157.754	2.82	OCUDAS	167.556	2.664
OCUDEW	157.601	2.907	OCUDEW	156.998	3.017	OCUDAS	155.649	3.115	OCUDAS	151.903	3.115	OCUDAS	161.483	2.664
OCUDEW	156.842	2.921	OCUFUR	159.835	3.074	OCUDEW	158.136	2.938	OCUDEW	167.629	3.384	OCUDEW	171.591	3.384
OCUKOP	160.941	3.317	OCUKUV	159.893	3.381	OCUFUR	150.222	2.85	OCUFUR	155.909	3.164	OCUKEC	150.737	2.854
ODADIJ	175.588	3.283	ODADUV	175.213	3.223	OCULIK	155.825	2.955	OCUXUF	156.719	3.189	OCUYES	166.114	3.379
ODAPOA	170.133	2.996	ODAPOA	156.802	2.942	ODAPOA	150.695	3.101	ODAPOA	157.953	3.098	ODAPOA	172.93	3.494
ODAPOA	151.585	2.921	ODAPOA	155.114	2.93	ODAPOA	159.914	2.994	ODAPOA	152.012	2.996	ODAPOA	159.047	3.498
ODAPOA	155.411	3.435	ODAPOA	152.194	3.17	ODAPOA	156.042	2.968	ODAPOA	172.756	3.134	ODAPOA	159.154	3.368
ODAPUI	169.375	3.065	ODAPUI	159.418	3.336	ODAPOA	154.098	2.942	ODAPOA	159.593	3.134	ODAPUI	156.641	3.147
ODAWIC	170.494	2.761	ODAWIC	166.55	3.259	ODAPUI	159.04	3.188	ODAWIC	160.898	3.483	ODAWIC	162.437	2.761
ODECIJ	158.391	3.333	ODECIJ	163.825	3.333	ODAWIC	153.134	3.479	ODAWIC	158.896	3.007	ODAWIC	156.764	3.035
ODEREY	154.301	3.262	ODERUO	162.305	3.409	ODEGAJ	152.396	3.378	ODEHUD	153.698	3.139	ODEHUD	153.658	3.225
ODEYUV	157.249	3.114	ODEYUV	163.25	2.87	ODEYOP	157.519	3.182	ODEYOP	161.234	3.151	ODEYUV	154.37	3.106
ODIMUJ	159.726	2.893	ODIMUJ	174.762	3.071	ODIKUL	161.145	3.175	ODIMUJ	158.138	3.314	ODIMUJ	168.524	3.376
ODIMUJ	164.579	3.276	ODIMUJ	157.431	2.692	ODIMUJ	163.737	3.085	ODIMUJ	163.508	3.099	ODIMUJ	169.65	3.115
ODIMUN	156.657	2.761	ODIMUN	166.25	2.741	ODIMUJ	150.531	2.839	ODIMUJ	163.643	3.03	ODIMUJ	153.052	3.369
ODIMUN	152.655	3.227	ODINAU	162.434	3.419	ODIMUN	168.352	3.191	ODIMUN	160.009	2.726	ODIMUN	170.027	2.967
ODIZUZ	154.488	3.055	ODOJUP	168.594	3.094	ODIYUZ	162.65	3.478	ODIZOS	161.441	2.828	ODIZOS	160.325	3.471
ODOJUP	176.598	3.45	ODOJUP	154.384	2.756	ODOJUP	162.611	2.756	ODOJUP	163.19	3.138	ODOJUP	169.121	3.226
ODOTUX	153.872	2.997	ODOWIP	165.767	3.104	ODOLAX	156.218	3.252	ODOLAX	152.519	2.707	ODOLAX	172.455	3.005
ODOYEL	150.333	3.365	ODOYEL	157.846	3.038	ODOXAK	172.002	3.126	ODOXAK	154.256	3.427	ODOXAK	161.835	2.96
ODUDIB	170.854	2.855	ODUDIB	153.133	3.413	ODOYEL	160.575	3.437	ODUCAV	150.025	3.113	ODUCAV	160.626	3.238
ODUGUQ	156.99	3.13	ODUJOQ	157.474	3.105	ODUGUQ	165.787	3.323	ODUGUQ	169.012	3.218	ODUGUQ	166.605	2.943
ODUKAB	167.455	3.293	ODUKAB	162.01	3.08	ODUJUU	155.468	3.027	ODUJUU	179.222	2.884	ODUJUU	173.131	2.984
ODUKAB	161.462	2.963	ODUKUV	152.202	2.892	ODUKAB	162.939	3.293	ODUKAB	156.153	3.441	ODUKAB	154.877	3.166
ODUPIM0	157.718	3.453	ODUPIN	152.819	3.002	ODUKUV	152.703	2.892	ODUNUY	167.35	2.902	ODUPIM	158.286	3.494
ODUPIN	150.195	3.08	ODUPIN	155.682	2.956	ODUPIN	163.003	3.495	ODUPIN	154.605	3.08	ODUPIN	171.478	3.399
ODUPIN	157.552	2.885	ODUPIN	179.068	3.059	ODUPIN	155.223	3.004	ODUPIN	164.403	3.322	ODUPIN	155.683	3.322
ODUTOA	173.027	3.087	ODUTOA	154.348	2.72	ODUSEM	156.129	3.257	ODUSEM	154.065	3.15	ODUTIT	159.52	3.471
ODUTOA	159.502	3.5	ODUTOA	154.519	2.988	ODUTOA	156.584	2.947	ODUTOA	156.732	3.379	ODUTOA	159.5	2.947
ODUTOA	157.919	2.988	ODUTOA	165.33	2.778	ODUTOA	155.776	3.11	ODUTOA	152.196	3.315	ODUTOA	158.48	3.326
OFAFSB0	167.81	3.053	OFAFSB1	152.906	3.204	ODUTOA	153.111	3.168	OFADUU	173.902	3.446	OFAFSB0	150.749	3.207
OFAFUZ	166.286	2.944	OFAFUZ	160.121	3.327	OFAFSB1	156.687	3.066	OFAFSB1	161.631	3.443	OFAFSB1	178.374	3.045
OFAJAJ	153.517	3.072	OFALEO	178.704	3.279	OFAFUZ	161.081	3.036	OFAJAJ	157.142	3.423	OFAJAJ	161.101	3.16
OFANTQ0	161.268	2.834	OFAZII	168.528	3.436	OFANTQ0	159.544	2.856	OFANTQ0	166.758	3.333	OFANTQ0	174.861	3.413
OFBUTI	176.218	2.859	OFBUTI	158.118	3.212	OFAZII	156.701	3.119	OFAZII	153.549	3.44	OFAZII	154.174	2.779
OFBZDO	169.283	3.328	OFBZDO	169.833	3.103	OFBUTI	164.39	3.212	OFBUTI	166.973	2.949	OFBUTI	153.39	3.204
OFELAM	163.053	3.048	OFELIU	154.018	3.218	OFEJIS	174.955	3.389	OFEJIS	162.098	3.327	OFELAM	172.276	2.628
OFELIU	158.699	2.892	OFELIU	150.628	3.172	OFELIU	158.678	3.015	OFELIU	160.815	3.497	OFELIU	163.402	3.417
OFERAU	151.159	2.98	OFERAU	150.635	3.435	OFELIU	163.017	3.145	OFELIU	150.939	3.096	OFERAU	156.343	3.045
OFESOK	162.464	3.467	OFIBAG	155.361	2.757	OFERAU	167.312	2.806	OFESOK	156.988	2.871	OFESOK	158.518	2.835
OFICAJ	158.442	3.485	OFICAJ	150.695	3.485	OFIBAG	150.309	3.087	OFIBAG	151.092	2.757	OFIBEK	165.581	3.47
OFIFUE	157.39	2.893	OFIFUE	161.334	2.982	OFIFUE	151.642	3.289	OFIFUE	172.833	3.163	OFIFUE	151.761	2.967
OFIFUE	165.589	2.95	OFIGES	156.676	2.712	OFIFUE	157.447	3.163	OFIFUE	169.537	3.043	OFIFUE	164.67	2.954
OFIKIX	157.707	2.982	OFINAT	160.684	2.714	OFIJAO	173.025	3.019	OFIJIW	162.046	3.262	OFIJIW	171.541	3.288
OFINAT	156.587	2.804	OFINAT	159.697	2.895	OFINAT	153.485	2.895	OFINAT	157.59	3.475	OFINAT	164.048	2.848
OFINIB	176.26	2.954	OFINIB	156.344	3.389	OFINAT	170.927	2.855	OFINIB	168.881	3.343	OFINIB	163.74	2.954
OFIPOI	153.06	3.221	OFIPOI	159.956	2.947	OFINIB	153.799	3.326	OFINIB	155.932	3.389	OFINIB	154.12	3.335
OFIPOI	152.267	2.876	OFIPUP	159.635	3.336	OFIPOI	151.108	2.876	OFIPOI	170.588	3.184	OFIPOI	156.869	3.341
OFIPUP	157.544	3.116	OFIQAW	158.752	3.024	OFIPUP	162.205	2.959	OFIPUP	168.431	3.09	OFIPUP	169.824	3.14
OFIVOR	170.97	2.801	OFIVOR	152.962	3.462	OFIQAW	162.813	3.03	OFIQAW	167.92	3.229	OFIQAW	163.875	3.184
OFNAPH0	150.347	3.205	OFNAPH0	163.632	3.481	OFNAPH0	169.372	3.057	OFNAPH0	156.27	3.105	OFNAPH0	156.858	3.026

OFNAPH0	154.422	2.881	OFNAPH0	172.199	3.076	OFNAPH0	163.949	3.137	OFNAPH0	159.565	3.268	OFNAPH0	153.299	3.368
OFNAPH0	153.287	3.287	OFNAPH0	152.882	2.844	OFNAPH0	169.667	2.979	OFNAPH0	172.801	3.015	OFNAPH0	163.364	3.428
OFNAPH0	156.767	2.975	OFNAPH0	156.624	3.017	OFNAPH0	163.106	3.096	OFNAPH0	159.091	3.256	OFNAPH0	150.878	3.152
OFNAPH0	152.917	2.837	OFNAPH0	153.3	3.282	OFNAPH0	172.819	3.009	OFNAPH0	156.666	3.015	OFNAPH0	169.807	2.977
OFNAPH0	163.219	3.42	OFNAPH0	150.917	3.147	OFNAPH0	156.798	2.966	OFNAPH0	159.161	3.25	OFNAPH0	163.174	3.084
OFUCEY	152.843	2.838	OFUCEY	168.113	3.336	OFOJUR	175.442	3.026	OFOLIG	161.999	3.038	OFUCEY	164.398	2.838
OFUCEY	156.077	2.648	OFUCEY	169.27	2.976	OFUCEY	173.915	3.326	OFUCEY	153.854	3.023	OFUCEY	175.511	2.862
OFUCIC	170.922	2.698	OFUCUO	156.748	3.475	OFUCIC	151.765	3.023	OFUCIC	157.965	3.148	OFUCIC	152.644	2.795
OFUCUO	159.152	3.211	OFUCUO	152.629	3.242	OFUCUO	150.992	3.177	OFUCUO	150.032	3.211	OFUCUO	169.259	3.018
OFUJIK	157.71	3.076	OFUJIK	154.105	3.387	OFUCUO	150.32	3.253	OFUHII	167.793	2.944	OFUHII	161.755	3.216
OFUTIS	153.885	3.063	OFUTIS	155.11	3.034	OFUKIL	167.131	2.939	OFUQOZ	174.307	3.103	OFUTIS	159.371	3.443
OFUWAN	158.417	2.744	OFUWAN	175.287	3.255	OFUTUE	154.751	3.226	OFUTUE0	154.47	3.211	OFUWAN	176.067	3.13
OFUWAN	162.339	2.75	OFUWAN	175.446	3.155	OFUWAN	155.015	2.744	OFUWAN	163.009	2.75	OFUWAN	166.502	3.271
OFUWER	154.361	3.275	OFUWER	157.784	3.43	OFUWER	162.334	3.245	OFUWER	150.13	3.432	OFUWER	157.318	2.778
OGAKEQ	162.649	2.914	OGAKEQ	165.09	2.772	OFUWER	161.968	3.372	OFUWER	150.388	2.911	OGAKEQ	153.314	2.772
OGAMIS	159.043	2.905	OGAMIS	159.247	3.386	OGAMIS	150.73	3.164	OGAMIS	161.172	2.869	OGAMIS	155.008	2.837
OGAWIE	150.73	3.228	OGAWIE	166.783	3.001	OGANIV	168.321	3.14	OGANIV	165.826	3.205	OGAWEZ	156.16	3.326
OGEDUD	160.736	2.88	OGEGIT	151.885	3.174	OGAWIE	156.934	2.968	OGAWUP	158.196	2.813	OGECEK	167.95	3.351
OGELUI03	154.372	3.49	OGENAP	164.331	3.206	OGEGUF	162.471	3.235	OGELUI03	152.897	2.856	OGELUI03	160.427	3.071
OGEPOI	150.749	2.681	OGESIF	153.354	3.231	OGENAP	159.327	3.445	OGENET	153.162	2.981	OGEPOI	150.395	2.807
OGETIE	150.59	3.287	OGETIE	150.204	3.414	OGETIE	161.319	3.251	OGETIE	152.415	3.212	OGETIE	151.671	2.916
OGIJAS	169.49	2.914	OGIJAS	163.878	3.335	OGETIE	158.668	3.251	OGEVIF	153.035	3.165	OGEZEI	150.58	3.101
OGIYOV	156.565	3.472	OGIYOW	162.873	3.037	OGIJAS	158.803	2.929	OGITEE	172.701	3.353	OGIYOV	156.32	2.734
OGOJIH	161.833	2.979	OGOJON	171.162	2.72	OGIYOW	153.452	3.291	OGIZAI	175.663	3.449	OGIZAI	167.548	2.992
OGOQOT	170.876	2.917	OGUBAT	166.196	3.171	OGOLIF	154.43	2.7	OGOLOO	152.877	3.241	OGOQOT	170.644	3.458
OGUPOY	151.111	3.109	OHAGEN	155.74	3.257	OGUBAT	167.458	3.393	OGUBIE	161.223	2.746	OGUBIE	161.27	3.331
OHARAT	155.19	3.046	OHARAT	173.81	3.181	OHAMIW	164.56	2.923	OHAMOC	164.689	2.918	OHARAT	160.997	3.161
OHETIH	155.892	3.12	OHETIH	155.836	3.198	OHEFAK	150.1	2.835	OHEJOA	176.438	3.028	OHENIZ	151.023	3.315
OHETIH	161.124	3.423	OHETIH	164.726	2.888	OHETIH	157.785	3.034	OHETIH	153.59	3.013	OHETIH	177.098	3.244
OHEWUV	156.961	3.31	OHEWUV	153.313	3.418	OHEVUV	174.996	3.32	OHEVUV	154.657	3.465	OHEWUV	151.807	2.846
OHEWUV	154.973	3.103	OHEWUV	150.962	2.856	OHEWUV	150.845	2.846	OHEWUV	152.028	2.856	OHEWUV	154.974	3.163
OHEWUX	157.943	3.228	OHIFAQ	150.792	3.359	OHEWUV	150.531	3.027	OHEWUV	154.885	3.264	OHEWUV	151.016	3.23
OHIRED01	151.591	3.034	OHIRED01	154.037	3.064	OHIFEU	155.436	3.059	OHINID	154.282	3.481	OHINID	165.585	3.434
OHIRED02	154.255	3.07	OHIRED02	165.404	3.038	OHIRED01	165.453	3.028	OHIRED01	155.609	2.926	OHIRED02	151.557	3.032
OHONEE	154.506	2.704	OHONEE	158.533	3.367	OHIRED02	155.253	2.927	OHOMEE	153.11	3.407	OHOMEE	166.753	3.331
OHOQIN	157.831	3.462	OHOQIN	172.541	3.184	OHOQIN	163.455	3.356	OHOQIN	170.974	3.096	OHOQIN	167.069	2.933
OHOQIN	153.554	2.901	OHOQIN	152.211	3.199	OHOQIN	175.62	3.358	OHOQIN	164.512	2.892	OHOQIN	163.872	3.261
OHOVIR	166.263	3.445	OHOVIR	164.606	3.396	OHOSEL0	155.404	2.968	OHOVIR	159.872	3.142	OHOVIR	163.917	3.009
OHUCAV	153.418	2.996	OHUCEC	169.401	3.416	OHUBIC	162.87	2.807	OHUBIC	166.768	2.825	OHUBOI	155.056	2.739
OHUCUS	150.478	3.217	OHUCUS	154.338	3.053	OHUCUS	177.084	3.219	OHUCUS	161.049	3.219	OHUCUS	168.66	3.134
OHUDEC	160.416	2.923	OHUKUZ	157.723	3.154	OHUCUS	153.019	3.381	OHUCUS	158.53	2.948	OHUDEC	164.253	2.795
OHUMUB0	150.315	3.093	OHUMUB0	153.467	2.932	OHUKUZ	160.475	3.476	OHUKUZ	167.624	3.09	OHUKUZ	163.314	3.272
OJAHAJ	156.538	3.132	OJAHEN	162.851	3.143	OHUMUB0	162.313	3.247	OJAHAJ	155.985	3.142	OJAHAJ	152.518	3.259
OJAHOX	150.997	2.927	OJAJEQ	154.123	3.116	OJAHEN	171.535	3.413	OJAHOX	162.33	2.784	OJAHOX	156.348	3.423
OJAVIG	159.554	3.046	OJAVIG	153.213	2.705	OJAVAY	150.986	2.947	OJAVAY	156.427	2.76	OJAVEC	153.915	3.178
OJECUC	163.644	3.133	OJECUC	174.058	3.26	OJAZOR	164.004	3.031	OJEBEM	158.858	3.155	OJEBEM	151.783	3.054
OJEMUN	174.645	2.716	OJEMUN	166.917	3.303	OJEDAJ	161.088	3.159	OJEDAJ	157.856	3.035	OJEDEO0	150.215	3.066
OJETIG	162.329	3.452	OJETIG	170.288	3.059	OJEMUN	164.917	3.125	OJEMUN	151.854	2.874	OJETIG	176.353	3.059
OJIHOG	151.631	3.27	OJIHUM	165.671	3.466	OJIHAS	158.263	3.324	OJIHAS	161.654	2.803	OJIHAS	164.801	3.218
OJIKOJ	161.226	3.032	OJILIE	151.145	2.819	OJIJEY	152.535	2.735	OJIJEY	152.904	2.735	OJIKEZ	174.699	3.097
OJIMUT	153.363	3.461	OJIMUT	160.076	2.671	OJILOK	160.194	3.35	OJIMAX	153.972	3.416	OJIMAX	158.597	2.926
OJIQII	161.29	3.244	OJISOP	163.227	3.013	OJIMUT	154.732	3.461	OJIMUT	161.565	3.008	OJIMUT	150.871	2.982
OJIZUD	157.123	3.197	OJOHIF	150.442	3.179	OJIZOX	152.007	3.391	OJIZOX	160.599	3.155	OJIZUD	167.322	3.452
OJOHIF	172.819	3.286	OJOHIF	173.712	2.732	OJOHIF	153.716	3.29	OJOHIF	159.946	3.293	OJOHIF	176.048	2.976
OJOJED	164.454	2.775	OJOJED	158.364	2.826	OJOHIF	171.754	2.976	OJOHUR	153.742	3.218	OJOHUR	163.478	3.053
OJOLAA	158.992	3.456	OJOLEF	152.055	3.316	OJOKEE	167.173	3.449	OJOKEE	164.631	3.14	OJOKEE	155.624	2.859
OJOVIT	154.112	3.335	OJUBAZ	159.536	3.234	OJOVAL	162.492	3.393	OJOVAL	156.092	2.741	OJOVIT	160.218	2.88
OJUCIJ	162.044	2.829	OJUCIJ	160.768	2.714	OJUBAZ	155.313	2.92	OJUCEF	158.883	2.741	OJUCEF	158.985	2.826
OJUHAE	165.895	3.327	OJUHAE0	163.155	3.271	OJUCOP	158.96	2.867	OJUDEC	165.752	3.294	OJUDEC	170.039	2.783
OJUSUI	167.731	3.171	OJUWUL	157.156	3.451	OJUPAK	169.324	3.016	OJUPAK	162.75	3.42	OJUSUI	151.342	3.193
OJUXUO	152.341	3.014	OJUXUO	168.03	2.842	OJUWUL	153.375	3.47	OJUWUL	150.51	3.451	OJUXUO	153.57	3.331
OKASOI	166.509	3.31	OKASOI	153.398	3.369	OJUXUO	174.04	2.842	OKAJAM	165.134	3.31	OKAJIV	153.021	3.095
OKEHIX	158.234	2.943	OKEHUJ	167.855	3.3	OKEBIP	150.635	3.237	OKECOX	167.9	3.17	OKECUD	159.693	2.613
OKEQIF	156.557	3.36	OKEQIF	165.123	2.709	OKEKOI	152.198	3.367	OKEKOI	156.084	3.427	OKEQIF	151.693	3.233
OKEWOQ	152.906	3.032	OKEWOQ	168.781	3.12	OKEQIF	158.671	3.467	OKEQUQ	177.624	3.316	OKEQUQ	155.051	2.989
OKEWOQ	159.756	3.12	OKEWOQ	151.842	3.346	OKEWOQ	166.251	3.185	OKEWOQ	154.588	3.169	OKEWOQ	164.725	2.986
OKIXAK	150.365	3.25	OKIXAK	157.375	3.252	OKEWOQ	169.939	3.098	OKEWOQ	166.233	3.121	OKIXAK	166.764	3.411
OKIXEO	162.579	2.832	OKOBID	153.871	3.024	OKIXEO	156.533	3.084	OKIXEO	168.074	3.087	OKIXEO	159.641	2.898
OKOBID	151.112	2.9	OKOBID	166.123	2.9	OKOBID	163.975	2.931	OKOBID	155.884	3.035	OKOBID	151.371	2.856
OKOJON	154.939	3.217	OKOJON	170.319	3.284	OKOBID	156.609	3.18	OKOBID	156.763	3.212	OKOJON	157.479	3.396
OKOLUV	161.936	3.32	OKOLUV0	160.077	3.13	OKOJON	166.606	2.95	OKOLUV	150.715	2.862	OKOLUV	168.228	2.862
OKOMOQ	153.931	2.885	OKOMOQ	167.942	3.457	OKOLUV0	154.628	3.409	OKOLUV0	152.675	2.77	OKOMEG	150.781	2.797
OKONEH	168.244	2.794	OKONEH	165.841	2.906	OKOMOQ	173.919	2.702	OKOMOQ	172.784	3.483	OKOMOQ	168.453	3.102
OKONOR	158.659	2.903	OKONOR	165.17	2.959	OKONEH	174.579	2.832	OKONEH	170.889	2.832	OKONOR	161.176	2.955
OKONOR	163.832	2.979	OKONOR	168.435	3.463	OKONOR	164.125	2.909	OKONOR	163.678	2.931	OKONOR	168.066	2.802
OKOWEQ	170.276	3.308	OKOWIU	160.042	3.107	OKONOR	169.087	3.44	OKONOR	168.141	2.777	OKOROZ	159.652	3.06
OKUTET	163.674	3.066	OKUVIA	165.809	3.37	OKUFOR	175.612	3.213	OKUTET	170.069	3.079	OKUTET	158.09	2.942
OLAJAN	169.783	3.398	OLAJER	176.671	2.907	OKUVIA	150.964	3.372	OLAJAN	159.226	3.285	OLAJAN	161.861	3.37
OLAJER	153.705	2.979	OLAJOB	163.712	3.477	OLAJER	155.449	3.024	OLAJER	161.835	2.838	OLAJER	156.755	3.497

OLAWUT	157.221	3.015	OLAWUT	153.746	2.941	OLAJOB	163.379	3.164	OLAJOB	166.673	2.841	OLARAV	161.457	3.21
OLAWUT	167.539	3.374	OLAWUT	157.404	3.038	OLAWUT	156.413	3.107	OLAWUT	151.251	2.941	OLAWUT	168.823	3
OLAWUT	155.811	3.174	OLAWUT	177.712	3.178	OLAWUT	156.361	2.97	OLAWUT	174.353	3.153	OLAWUT	158.769	3.068
OLAWUT	150.775	2.97	OLEBAJ01	156.829	3.499	OLAWUT	171.606	3.328	OLAWUT	161.386	2.923	OLAWUT	157.677	3.149
OLEBAJ01	164.348	2.826	OLEBAJ01	165.607	3.141	OLEBAJ01	171.512	2.809	OLEBAJ01	166.171	2.973	OLEBAJ01	153.154	3.48
OLESOQ	154.988	3.14	OLESUW	160.281	2.773	OLEBAJ01	164.648	3.358	OLEHUK	163.208	2.726	OLENIF	162.129	3.197
OLIKOK	150.911	3.356	OLIKOK	152.469	2.978	OLEZEL	150.977	3.164	OLIDOC	178.473	3.033	OLIHIE	162.861	2.826
OLIYEN	166.436	2.801	OLIYEN	169.385	2.847	OLIKOK	151.765	3.003	OLIMEB	156.623	2.865	OLIYEN	153.92	3.467
OLOLAF	169.309	2.674	OLOMUB	161.763	2.897	OLIYEN	157.908	3.105	OLIYEN	154.615	3.255	OLOLAF	160.686	3.241
OLONAI	152.737	3.206	OLONAI	164.453	2.972	OLOMUB	170.099	2.88	OLONAI	157.071	3.339	OLONAI	156.266	3.005
OLOTUI	154.637	3.195	OLOTUI	169.785	2.939	OLOQEO	153.976	3.177	OLOSEQ	168.145	2.89	OLOSEQ	166.696	2.898
OLOVAQ	166.692	2.802	OLOVAQ	151.786	2.906	OLOTUI	157.06	3.195	OLOTUI	152.137	2.804	OLOVAQ	159.551	2.684
OLOVAQ	154.387	3.429	OLOVAQ	150.494	3.264	OLOVAQ	166.379	3.049	OLOVAQ	168.377	3.038	OLOVAQ	150.579	2.732
OLUFEG	156.104	3.085	OLUFEG	157.303	3.209	OLOVAQ	176.119	2.732	OLUFEG	171.415	3.112	OLUFEG	161.073	3.15
OLUNAL	152.908	3.08	OLUNAL	156.887	3.026	OLUMUE	153.057	3.267	OLUMUE	150.793	3.064	OLUNAL	150.488	3.067
OMAFAL	157.14	2.993	OMAFAL	151.089	3.135	OLUNAL	158.92	3.016	OLUQOC0	173.078	3.344	OMADUD	150.106	3.459
OMAVEG	160.5	3.447	OMAVEG	164.5	3.447	OMAFEP	158.343	3.291	OMAFEP	163.722	3.453	OMAMOF	166.895	3.127
OMEMAY	161.074	2.854	OMICUI	156.687	3.482	OMEBUF	150.936	3.468	OMEBUF	168.239	3.445	OMEHEU	159.48	3.15
OMISOW	165.371	3.394	OMISOW	162.012	2.961	OMIJOM	171.449	2.803	OMISOW	164.682	2.96	OMISOW	174.272	3.281
OMIVEL	161.326	3.154	OMIWEP	152.77	3.275	OMISOW	151.437	2.981	OMITUD	152.454	3.049	OMIVAL	176.676	3.465
OMOBAX	168.666	2.813	OMOBAX	168.651	2.983	OMIYAN	156.551	3.208	OMOBAX	163.195	2.813	OMOBAX	155.125	3.18
OMOBAX	169.911	3.188	OMOBAX	152.893	3.451	OMOBAX	155.199	3.171	OMOBAX	151.173	3.313	OMOBAX	164.881	2.75
OMOJOS	152.947	2.606	OMOSEO	163.169	3.286	OMOJOS	167.839	3.356	OMOJOS	171.493	3.497	OMOJOS	160.678	2.601
OMOXIZ	152.219	2.899	OMOZAT	161.095	2.845	OMOSEO	154.424	3.191	OMOSEO	155	3.309	OMOXAR	154.531	3.468
OMOZAT	158.716	2.845	OMOZAT	161.075	3.133	OMOZAT	169.878	3.03	OMOZAT	162.569	3.117	OMOZAT	163.485	2.941
OMOZAT	165.269	3.028	OMOZAT	177.406	2.832	OMOZAT	159.99	3.29	OMOZAT	175.622	2.872	OMOZAT	159.375	3.429
OMOZEX	152.753	3.053	OMOZEX	169.532	2.986	OMOZEX	152	3.081	OMOZEX	164.792	2.902	OMOZEX	160.94	3.032
OMOZIB	151.749	3.063	OMOZIB	155.071	2.898	OMOZEX	158.612	3.029	OMOZEX	164.975	2.904	OMOZEX	174.722	2.709
OMOZOH	165.396	2.89	OMOZOH	157.599	2.876	OMOZIB	153.611	3.125	OMOZIB	154.451	3.246	OMOZIB	174.035	2.898
OMOZOH	174.363	3.35	OMUFEK	155.663	3.288	OMOZOH	163.203	3.102	OMOZOH	152.541	2.927	OMOZOH	151.045	2.89
ONAHER	161.063	2.951	ONAHER	156.844	3.129	OMUGUZ	174.254	2.979	OMUHAI	156.686	2.944	OMUMAN	161.438	2.843
ONASUU	154.86	2.991	ONASUU	159.103	2.889	ONAHER	177.841	3.019	ONAHER	153.485	3.39	ONAHER	171.626	2.924
ONEVAG	154.917	3.373	ONEVAG	163.331	3.32	ONASUU	153.458	2.629	ONAZOT	161.258	3.05	ONEVAG	151.763	3.17
ONEVOU	166.683	3.238	ONEVOU	163.558	3.098	ONEVAG	153.173	3.421	ONEVAG	162.951	3.056	ONEVAG	165.341	3.177
ONIDEX	153.003	2.91	ONIWUG	169.061	2.809	ONEVOU	160.188	3.24	ONEVOU	150.856	3.462	ONIBIZ	150.331	3.227
ONOJEI	151.889	3.022	ONOJEI01	150.691	3.039	ONIWUG	164.151	3.271	ONOBUQ	154.62	3.404	ONOCAX	168.891	3.28
ONORIV	154.794	3.26	ONOWEU	171.348	2.813	ONOLIO	175.118	3.445	ONOLIO	168.324	2.88	ONOLIP	165.951	3.217
ONUBEF	174.789	3.155	ONUFIP	156.801	3.41	ONOWEU	165.203	3.112	ONUBAB	164.225	3.182	ONUBEF	151.71	2.668
ONUFIP	157.267	3.267	ONUFIP	152.234	3.328	ONUFIP	156.527	3.054	ONUFIP	152.406	3.328	ONUFIP	158.8	3.241
ONUJAK	155.782	2.927	ONUJAK	164.125	3.313	ONUFIP	160.636	3.176	ONUJAK	151.377	3.029	ONUJAK	150.836	2.805
ONUKOY	158.788	2.912	ONUKUF	155.321	3.396	ONUJAK	152.036	3.07	ONUJAK	156.411	3.446	ONUJAK	157.951	3.489
OPADIT	174.256	3.222	OPAGES	172.322	3.294	ONUVEZ	164.534	3.472	ONUYII	163.565	3.315	ONUYII	158.145	3.444
OPALAU01	168.476	2.896	OPALOJ	152.746	2.998	OPAGES	172.224	2.933	OPAJIA	157.577	2.934	OPALAU01	168.854	3.102
OPALOJ	167.72	3.191	OPALOJ	151.668	2.664	OPALOJ	150.617	2.858	OPALOJ	174.479	2.824	OPALOJ	152.802	3.098
OPAMAW	173.788	2.813	OPAMAW	168.003	3.187	OPAMAW	152.048	3.073	OPAMAW	153.256	2.96	OPAMAW	150.325	2.849
OPEVIR01	166.825	3.291	OPEVIR02	166.48	3.293	OPAMAW	151.316	2.673	OPAMEZ	158.26	3.411	OPEVIR	166.939	3.294
OPEVIR13	165.582	3.3	OPEVIR14	166.355	3.305	OPEVIR03	166.363	3.292	OPEVIR04	166.443	3.29	OPEVIR05	166.687	3.29
OPEVIR18	166.285	3.295	OPEVIR19	166.378	3.292	OPEVIR15	166.113	3.3	OPEVIR16	166.379	3.298	OPEVIR17	165.936	3.298
OPEVIR23	166.556	3.287	OPEVIR24	166.307	3.286	OPEVIR20	166.554	3.294	OPEVIR21	166.431	3.297	OPEVIR22	166.46	3.287
OPEYAM0	163.565	3.297	OPEYAM0	163.649	3.298	OPEVIR25	166.814	3.283	OPEVIR27	166.522	3.278	OPEYAM	163.526	3.294
OPEYAM0	163.878	3.309	OPEYAM0	163.431	3.315	OPEYAM0	163.329	3.302	OPEYAM0	163.579	3.301	OPEYAM0	163.594	3.305
OPEYAM1	163.949	3.288	OPEYAM1	164.257	3.287	OPEYAM0	163.766	3.307	OPEYAM0	164.043	3.305	OPEYAM1	163.834	3.304
OPEYAM1	164.487	3.267	OPEYAM1	164.491	3.265	OPEYAM1	164.222	3.281	OPEYAM1	164.414	3.276	OPEYAM1	164.534	3.272
OPEYAM2	163.877	3.265	OPEYAM2	164.029	3.272	OPEYAM1	164.181	3.28	OPEYAM1	164.701	3.265	OPEYAM2	164.086	3.264
OPEYAM2	163.737	3.277	OPEYAM2	163.858	3.283	OPEYAM2	163.815	3.27	OPEYAM2	163.813	3.275	OPEYAM2	163.846	3.282
OPEYAM3	163.864	3.3	OPEYAM3	163.67	3.293	OPEYAM2	163.465	3.288	OPEYAM3	163.534	3.293	OPEYAM3	163.787	3.303
OPIJUU	161.463	3.164	OPIJUU	150.071	3.468	OPEYAM3	163.946	3.291	OPEYAM3	163.868	3.29	OPICIB	153.933	3.163
OPORER	151.534	3.144	OPOTUK	168.235	3.374	OPIPAF	170.04	3.178	OPOQUH	172.297	3.289	OPORAN	168.622	3.09
OPOVAS	175.782	3.053	OPUJIU	154.068	3.22	OPOTUK	165.052	3.493	OPOTUK	154.66	3.461	OPOTUK	151.906	3.216
OPULES	160.139	3.276	OPULUI	167.533	2.818	OPUJIU	168.591	3.423	OPUKUH0	153.445	3.473	OPUKUH0	151.285	3.437
OPUNEU	160.842	3.497	OPUNEU0	161.049	3.471	OPULUI	151.476	2.818	OPUMES	152.407	3.018	OPUNEU	153.677	3.164
OPUNEU0	153.769	3.157	OPUNEU0	160.862	3.493	OPUNEU0	153.68	3.157	OPUNEU0	153.552	3.174	OPUNEU0	160.949	3.47
OQACEP	151.586	3.294	OQACEQ	153.753	2.804	OPUNEU0	153.677	3.165	OPUWON	157.681	3.365	OPUWON	158.681	3.056
OQAWOT	155.551	3.013	OQEBIW	154.658	3.126	OQAWOT	154.995	2.993	OQAWOT	164.88	2.89	OQAWOT	164.878	2.901
OQEBUI01	156.414	3.323	OQEBUI01	177.003	2.915	OQEBIW	167.579	3.004	OQEBUI	156.242	3.308	OQEBUI	177.559	2.877
OQEHUO	165.119	3.322	OQEJAW	168.512	2.917	OQECET	157.709	3.054	OQECET	153.06	3.216	OQEHUO	161.911	3.498
OQEVAK	162.825	3.217	OQEVAK	160.429	3.064	OQEJAW	167.409	3.32	OQEJEA	159.124	3.003	OQEKAZ0	163.66	3.186
OQIJEF	152.479	3.251	OQIJEF	173.877	3.009	OQEVAK	160.475	3.098	OQEVAK	166.955	3.16	OQIHON	152.208	3.225
OQIJIJ	156.367	3.261	OQITIT	155.181	3.126	OQIJEF	153.883	3.006	OQIJEF	171.481	3.42	OQIJIJ	170.71	3.165
OQIWOC	156.058	2.78	OQIWOC	154.637	3.039	OQITOZ	171.737	3.142	OQITUF	168.922	3.003	OQITUF	154.614	3.418
OQIXAO	161.725	2.994	OQIXAO	167.831	2.774	OQIXAO	157.217	2.994	OQIXAO	160.981	2.966	OQIXAO	166.933	2.786
OQOLAK0	151.172	3.263	OQOLAK0	166.235	3.047	OQIXES	174.967	2.809	OQIXES	172.503	2.816	OQIXES	151.313	2.776
OQOLAK0	152.308	3.152	OQOLEO0	158.012	3.255	OQOLAK0	152.137	3.039	OQOLAK0	173.71	3.216	OQOLAK0	162.897	3.201
OQOPUI	154.753	3.228	OQOQAP	151.712	2.928	OQOLEO0	165.082	3.339	OQOLEO0	160.378	3.499	OQOPOC	155.691	2.846
OQUQEZ	155.732	2.928	OQUROK	162.308	3.269	OQUHUF	154.746	3.074	OQUNIY	152.96	3.036	OQUQEZ	156.1	2.928
ORALAW	163.241	2.997	ORANUT	161.496	2.935	OQUZIK	158.726	3.322	ORACUH	155.843	2.982	ORADAO	161.685	3.002
OREDEW	165.193	3.36	OREKUS	161.843	3.303	ORANUT	171.17	2.757	ORAYIS	175.82	3.493	OREDEW	169.21	3.36
ORELAA	166.883	2.9	ORELAA	161.562	3.288	ORELAA	168.523	3.015	ORELAA	171.726	2.846	ORELAA	156.406	3.415
ORIJEG	156.923	3.386	ORILIN	158.352	3.332	ORELAA	158.417	3.448	ORELAA	168.47	2.789	ORIHOO	162.277	2.901

OROQIY	161.829	3.204	OROQIY	156.89	3.028	ORILOT	172.334	3.334	ORISOZ	171.213	2.977	ORISOZ	151.149	3.353
OROYAX	154.151	3.431	ORUZAD	151.998	3.167	OROXUQ	173.11	3.328	OROXUQ	155.51	3.374	OROXUQ	157.767	3.338
OSASUX	165.985	2.647	OSAYEP	166.248	2.753	OSABIV	152.709	3.461	OSABIV	151.582	3.461	OSAJAU	159.22	3.488
OSELUW	155.288	3.05	OSELUW	150.51	2.904	OSAYEP	166.729	2.832	OSAYUF	157.944	3.244	OSELIK	167.572	3.419
OSEWUF	158.43	2.639	OSEXAM	156.984	2.922	OSELUW	156.366	2.795	OSEWUF	170.622	3.292	OSEWUF	160.541	3.391
OSIGON	172.199	3.141	OSIHUW	154.195	3.47	OSEXAM	163.352	3.061	OSEZAP	155.337	3.19	OSIGON	161.827	3.061
OSINOV	178.563	3.171	OSITIV	169.267	3.401	OSIHUW	166.357	3.206	OSIHUW	162.941	3.351	OSINOV	169.146	2.929
OSITIV	171.003	2.862	OSITOB	160.114	2.722	OSITIV	154.124	3.202	OSITIV	156.724	2.862	OSITIV	152.161	3.208
OSIZIA	151.115	2.95	OSOJEN	159.552	3.247	OSITOB	175.714	3.03	OSIXIZ	153.54	3	OSIZIA	159.899	3.484
OSOZUS	158.154	3.033	OSUDEN	165.245	3.067	OSOJEN	166.462	3.119	OSOJEN	154.519	3.396	OSOZUS	164.393	3.405
OSUYIM	157.831	3.334	OSUYIM	172.691	2.776	OSUJOD	150.075	2.951	OSULOF	153.013	3.009	OSUYIM	174.734	2.92
OTABER	163.611	3.399	OTABER	152.271	3.359	OTABER	165.214	2.991	OTABER	158.721	3.271	OTABER	166.359	3.308
OTACAO	156.825	3.117	OTACAO	163.013	3.281	OTABER	155.79	3.29	OTABER	159.275	3.367	OTABUH	160.996	3.222
OTACAO	155.331	2.754	OTACAO	171.385	2.926	OTACAO	164.402	3.495	OTACAO	150.345	2.754	OTACAO	154.594	2.964
OTAJUR	161.253	2.998	OTAKEC	175.537	2.922	OTACAO	155.905	3.127	OTACAO	164.648	2.923	OTAJUR	157.798	2.816
OTAKEC	163.384	2.958	OTAKEC	176.134	3.128	OTAKEC	176.025	3.135	OTAKEC	150.16	3.385	OTAKEC	156.442	3.476
OTAKOM	155.688	3.384	OTAKOM	165.307	2.997	OTAKEC	159.859	3.303	OTAKOM	151.88	2.914	OTAKOM	161.892	3.358
OTAROR	155.522	3.255	OTENUZ	170.973	2.967	OTAKOM	151.749	3.384	OTAPUV	167.163	2.888	OTAROR	154.457	2.642
OTIDOL	152.472	3.335	OTODAE	171.906	3.166	OTERAI	167.934	3.279	OTIDEB	167.728	3.423	OTIDEB	161.405	3.326
OTOWOM	163.688	3.45	OTOZIJ	165.716	3.335	OTOGUC	163.351	3.07	OTOGUC	153.77	3.178	OTOHEN	162.602	3.218
OTOZOP	159.377	3.206	OTOZUV	158.855	3.218	OTOZIJ	162.604	2.792	OTOZIJ01	165.491	3.332	OTOZIJ01	162.864	2.798
OTUFOB	167.91	3.08	OTUFOB	159.07	2.874	OTUFOB	167.92	2.882	OTUFOB	164.666	3.217	OTUFOB	164.375	3.269
OTUFOB	170.042	3.15	OTUFOB	157.141	2.916	OTUFOB	157.852	3.142	OTUFOB	151.477	3.336	OTUFOB	150.715	3.233
OTUFOB	156.183	3.08	OTUFOB	161.706	3.392	OTUFOB	156.78	3.271	OTUFOB	150.283	3.029	OTUFOB	155.187	3.235
OTUJUL	163.578	3.039	OTUREB	164.127	3.053	OTUFUH	160.956	2.825	OTUFUH	155.992	3.495	OTUFUH	151.709	2.639
OVAJEC	176.39	3.143	OVAJEC	153.905	3.122	OVAJEC	151.361	2.785	OVAJEC	158.144	2.765	OVAJEC	170.411	2.796
OVAJEC	159.317	3.038	OVAJEC	155.849	2.724	OVAJEC	160.366	3	OVAJEC	159.095	3.09	OVAJEC	160.718	3.111
OVANEG	175.595	3.061	OVANEG	154.454	3.082	OVAJEC	153.546	2.874	OVAJEC	150.835	3.388	OVAJEC	152.165	2.623
OVANEG	158.523	3.461	OVANEG	157.932	2.833	OVANEG	161.289	3.102	OVANEG	159.425	3.329	OVANEG	163.298	2.883
OVAREJ	151.277	3.018	OVAREJ	161.499	3.178	OVANEG	162.209	2.867	OVAPAF	161.284	2.631	OVAPAF	161.709	2.631
OVAXAL	157.08	3.342	OVAXAL	178.754	2.76	OVAREJ	154.264	2.836	OVAROU	160.175	3.19	OVAWUE	156.534	3.317
OVEBUN	173.796	2.916	OVEBUN	156.601	2.818	OVAXAL	160.693	3.143	OVEBOH	161.222	2.923	OVEBOH	171.963	3.364
OVECAU	167.307	3.206	OVECAU	161.865	3.306	OVEBUN	167.042	3.093	OVECAU	154.782	3.243	OVECAU	162.538	2.923
OVEJAC	158.282	2.791	OVEJIK	157.236	2.904	OVECAU	157.236	3.066	OVEHIJ	169.194	2.657	OVEHIJ	160.92	2.902
OVERUD	153.174	3.225	OVERUD0	157.265	3.068	OVEJIK	156.703	2.987	OVERIS	164.19	3.393	OVERUD	166.844	3.07
OVESAL	154.712	3.223	OVEZAT	156.757	3.152	OVERUD0	156.407	3.38	OVERUD0	157.245	3.347	OVESAL	169.743	3.306
OVIHAF	167.98	2.993	OVIHEJ	166.587	3.04	OVEZAT	171.124	2.758	OVEZAT	153.585	3.152	OVEZAT	155.039	3.007
OVIMIS	156.637	2.694	OVIMIS	155.638	3.386	OVIHIN	160.918	3.126	OVIHOR01	157.831	3.26	OVIHOR04	157.506	3.261
OVOMUK	159.03	2.909	OVONAR	158.657	3.302	OVIMIS	158.611	3.049	OVIMUE	151.598	3.405	OVIMUE	164.934	3.255
OVONIZ	160.598	2.913	OVUBUF	164.919	2.612	OVONAR	157.301	3.426	OVONAR	159.224	3.432	OVONAR	155.173	3.302
OVUCAM	164.361	3.22	OVUCAM	169.18	2.884	OVUBUF	161.795	2.794	OVUBUF	154.482	3.106	OVUCAM	154.644	3.179
OVUCEQ	165.319	3.211	OVUCEQ	164.561	3.182	OVUCAM	170.076	2.892	OVUCEQ	154.946	3.222	OVUCEQ	150.964	3.278
OVUMEY	167.873	2.942	OVURIH	159.929	3.104	OVUFOC	172.001	2.959	OVUFOC	151.973	2.98	OVUFOC	174.921	3.467
OVURON	171.096	3.135	OVURON	154.387	3.341	OVURIH	154.09	3.13	OVURIH	154.94	3.015	OVURON	165.441	2.881
OWAQAF	166.093	3.371	OWAQAF	174.045	3.349	OVURON	150.382	3.201	OVUZUD	152.336	3.227	OWALEF	151.545	3.394
OWAQEJ	150.295	2.748	OWAQEJ	165.501	2.762	OWAQAF	176.567	3.029	OWAQEJ	156.91	3	OWAQEJ	166.358	2.987
OWEPIR	165.151	3.089	OWEQUF	169.614	2.958	OWAQUZ	153.11	3.452	OWEBOK	168.06	3.214	OWEMOV	158.756	3.332
OWESOA	162.262	2.676	OWESOA	160.124	2.665	OWESAM	165.474	3.228	OWESOA	155.909	2.95	OWESOA	163.297	3.015
OWETER	157.991	2.995	OWETIV	176.474	2.861	OWESOA	164.412	2.995	OWESOA	160.796	2.884	OWETAN	161.082	3.176
OWICII	152.196	3.375	OWIGOT	162.075	3.138	OWEZIC	157.07	3.424	OWIBUT	161.019	3.422	OWICAA	157.203	3.228
OWITAS	154.4	2.957	OWITAS	159.634	2.776	OWIGOT	155.049	3.199	OWIRIY	153.802	3.395	OWISIY	160.952	2.971
OWITAS	161.618	2.967	OWITAS	155.531	3.085	OWITAS	177.634	2.694	OWITAS	177.805	3.264	OWITAS	154.196	3.004
OWIZOM	153.233	3.089	OWONIZ	156.987	3.283	OWITAS	163.46	2.694	OWITAS	169.302	2.902	OWIVUN	164.83	3.312
OWOWON	170.983	3.252	OWOWON	161.623	2.904	OWONIZ	163.14	3.313	OWONIZ	159.166	3.023	OWOWON	167.851	3.205
OWOXEE	166.242	2.932	OWOXOO	165.408	3.179	OWOWON	168.361	3.061	OWOXEE	154.403	3.441	OWOXEE	152.479	3.374
OWULID	162.358	3.157	OWULID	174.551	3.458	OWUKUO	170.265	3.055	OWULAV	155.564	3.277	OWULID	162.127	2.962
OXAROX	165.144	3.166	OXASEM	152.031	2.83	OXADUP	179.157	2.979	OXADUP	162.033	3.389	OXADUP	155.026	2.96
OXECUR	173.734	2.904	OXECUR	163.98	3.341	OXATIT	161.87	2.815	OXATIT	158.389	3.14	OXECUR	173.629	3.007
OXECUR	153.182	3.216	OXECUR	166.298	3.349	OXECUR	150.79	3.341	OXECUR	163.426	3.127	OXECUR	156.735	3.127
OXECUR	169.244	2.834	OXESOC	156.935	3.057	OXECUR	166.537	3.181	OXECUR	151.5	3.379	OXECUR	162.456	2.852
OXIFOT	168.002	3.247	OXIGUA	175.332	3.043	OXICIK	150.195	2.989	OXIDIL	155.157	3.132	OXIDOR	154.728	3.131
OXIMAK	159.528	3.389	OXIMAK	167.288	3.175	OXIHEL	161.368	2.949	OXIHEL	162.619	2.963	OXIKUE	167.561	2.78
OXINOB	159.959	3.451	OXIPIX01	169.042	2.981	OXINIU	150.632	2.844	OXINIU	152.819	2.844	OXINIV	151.269	2.924
OXIQAQ	157.719	2.975	OXIVUP	153.872	3.445	OXIQAQ	155.508	3.045	OXIQAQ	168.108	2.975	OXIQAQ	161.589	3.232
OXOTUS	173.974	2.872	OXOVIH	156.194	3.312	OXONOH	162.116	3.244	OXONOH	152.776	3.106	OXOQOI	150.12	2.941
OXOVON	166.663	2.909	OXOWEE	165.092	3.27	OXOVIH	161.418	3.426	OXOVIH	156.749	3.255	OXOVON	156.309	3.163
OXOXUV	156.369	3.012	OXOXUV	164.992	2.956	OXOWEE	176.274	3.35	OXOWEE	175.373	3.225	OXOXUV	158.63	3.152
OXULIF	157.507	3.207	OXUMOM	170.874	3.114	OXOXUV	174.014	3.175	OXOXUV	157.919	3.341	OXOXUV	174.931	3.175
OXUQEF	158.374	2.964	OXUVIO	154.01	2.95	OXUQEF	153.707	3.498	OXUQEF	167.76	2.699	OXUQEF	150.948	2.753
OXUVIO	155.562	3.275	OYAGEB	150.487	3.242	OXUVIO	153.024	3.184	OXUVIO	163.055	3.104	OXUVIO	157.966	3.244
OYEKUB	165.357	3.251	OYIBIK	166.36	3.022	OYAHAY	174.58	3.017	OYAPUA	154.793	3.172	OYAPUA	152.61	3.008
OYIBUW	157.629	2.969	OYICEH	157.832	2.969	OYIBIK	158.215	3.019	OYIBIK	164.576	3.205	OYIBIK	155.834	3.121
OYIWUQ	151.643	3.247	OYIWUQ	166.278	3.16	OYICEH	176.356	3.432	OYICEH	160.864	3.483	OYIKEN	159.05	3.078
OYOCUB	155.099	3.5	OYOHUI	161.823	3.204	OYOCAI	153.111	3.338	OYOCEM	154.859	3.288	OYOCIP	154.451	3.312
OYOHUI	176.612	3.125	OYOHUI	154.792	3.167	OYOHUI	155.354	2.86	OYOHUI	163.231	3.21	OYOHUI	165.426	3.344
OYOPAW	152.069	3.392	OZACUQ	167.536	3.369	OYOMAS	162.976	3.049	OYOMAS	165.258	3.031	OYONUO	158.976	3.405
OZANEK	152.134	3.027	OZANEK	152.42	3.229	OZALAF	159.817	3.204	OZANEK	150.684	2.894	OZANEK	168.739	3.021
OZAZIB	176.093	2.671	OZAZIB	161.06	3.236	OZANEK	165.875	3.021	OZANEK	165.707	3.247	OZANEK	153.567	2.742
OZEBIH	175.626	2.836	OZEBUT	174.92	2.915	OZAZOH	160.049	3.191	OZAZOH	156.372	3.222	OZEBIH	150.182	2.813

OZECII	162.093	3.27	OZECII	157.052	2.781	OZECEE	160.237	3.494	OZECEE	151.741	3.371	OZECEE	158.004	3.329
OZECII	164.551	3.277	OZECUU	157.152	3.276	OZECII	151.673	3.202	OZECII	154.151	2.811	OZECII	158.845	3.254
OZEDOP	173.34	2.982	OZEDOP	150.501	2.947	OZECUU	152.543	3.452	OZEDEF	154.896	3.402	OZEDOP	150.259	2.926
OZEFAD	168.166	2.882	OZEFIL	160.219	2.852	OZEDUV	171.469	3.236	OZEDUV	168.108	2.855	OZEFAD	172.511	3.31
OZEFOR	153.87	3.393	OZEFOR	165.957	2.86	OZEFIL	159.172	2.922	OZEFIL	173.304	3.183	OZEFIL	178.962	3.112
OZEFUV	175.759	3.076	OZEFUV	175.431	2.792	OZEFUV	155.255	3.438	OZEFUV	167.283	2.877	OZEFUV	150.743	3.382
OZEGEI	174.932	3.018	OZELUD	165.582	2.834	OZEFUV	150.38	2.976	OZEFUV	153.611	3.215	OZEFUV	172.714	2.726
OZERIX	157.055	2.853	OZETIX	154.771	3.025	OZEMAK	154.642	2.812	OZEMEO	170.275	2.677	OZEMEO	150.56	2.973
OZOGAM	150.044	2.622	OZOGAM	157.747	3.033	OZETIX	160.471	3.069	OZINUJ	151.199	3.135	OZIYIH	152.934	3.318
OZOGEQ	158.641	2.941	OZOLIB	154.754	2.902	OZOGEQ	152.595	3.196	OZOGEQ	163.003	3.351	OZOGEQ	153.633	2.962
OZUBUH	159.824	2.803	OZUJIE	154.729	3.342	OZOLIB	169.993	2.902	OZOMAS	176.712	2.932	OZOTOO	163.772	3.397
OZUYIT	166.26	2.834	OZUYIT	158.641	3.123	OZUXIS	153.364	3.179	OZUXIS	150.433	3.131	OZUYIT	151.902	2.84
OZUYOZ	169.089	3.045	OZUYOZ	173.71	2.871	OZUYOZ	163.028	3.29	OZUYOZ	151.159	3.232	OZUYOZ	153.792	2.906
OZUYOZ	168.103	3.087	OZUYOZ	167.687	2.96	OZUYOZ	162.123	2.803	OZUYOZ	164.056	2.966	OZUYOZ	150.034	3.29
PABDII	165.164	3.288	PABHEH	160.072	3.476	OZUYOZ	165.391	2.803	PABCAB	158.341	3.251	PABCAB	151.578	3.464
PABTET	177.837	3.106	PABTET	172.756	2.963	PABKEM	164.335	3.157	PABKEM	156.774	3.165	PABTET	167.1	2.926
PACBOO	174.927	2.8	PACBOO	152.181	3.284	PABTET	176.09	3.071	PABZUR	161.854	3.453	PABZUR	156.801	3.453
PACBOO	157.251	2.698	PACCAB	157.824	2.804	PACBOO	156.398	3.297	PACBOO	165.917	3.031	PACBOO	153.322	2.698
PACHOU	159.972	3.337	PACHOU	166.61	2.65	PACHIO	152.492	2.671	PACHIO	160.954	3.212	PACHIO	175.316	3.234
PACKUD	150.114	3.327	PACMOX	152.389	3.208	PACKAK	158.909	2.91	PACKEO	154.845	2.85	PACKIS	159.487	3.369
PACRIV	166.424	3.243	PACROB	165.951	3.298	PACMOY	153.13	3.448	PACNAL	152.521	2.866	PACNOZ	152.378	2.846
PACXAV	151.819	3.196	PACXEB	162.917	3.205	PACROB	154.725	3.173	PACROB	154.995	3.451	PACROB	156.423	3.298
PACXEB	162.946	3.238	PACYOL	169.802	3.173	PACXEB	168.52	3.182	PACXEB	156.01	3.081	PACXEB	165.314	2.72
PADDEE	150.916	3.017	PADDEE	157.838	3.403	PADDEE	151.862	3.157	PADDEE	150.353	3.206	PADDEE	153.867	3.025
PADDEE	155.72	3.328	PADDEE	163.381	3.332	PADDEE	155.529	3.079	PADDEE	166.771	3.101	PADDEE	166.573	3.415
PADNUH	159.739	2.978	PADNUH	153.631	3.056	PADDEE	172.735	2.979	PADFUA	174.553	2.969	PADHUY	154.291	2.971
PADQAR	156.473	2.779	PADQAR	154.479	2.932	PADQAR	171.911	2.927	PADQAR	166.216	3.343	PADQAR	165.693	3.121
PAFBIL	165.102	2.991	PAFBIL	174.516	3.072	PADQAR	170.75	3.408	PAFBIL	151.534	3.179	PAFBIL	170.255	3.086
PAFBIL	154.376	3.485	PAFBIL	163.689	3.152	PAFBIL	160.924	3.055	PAFBIL	166.268	3.287	PAFBIL	152.314	3.419
PAFBOR	155.412	2.637	PAFBOR	161.639	2.717	PAFBOR	160.375	3.022	PAFBOR	161.026	2.782	PAFBOR	156.933	3.216
PAFBOR	161.563	3.37	PAFBOR	168.038	2.872	PAFBOR	153.504	3.256	PAFBOR	155.185	3.359	PAFBOR	151.753	3.481
PAFBOR	173.453	2.847	PAFBOR	161.064	3.418	PAFBOR	160.406	2.782	PAFBOR	164.135	3.216	PAFBOR	155.064	2.957
PAFBOR	158.201	2.77	PAFBOR	155.622	2.905	PAFBOR	152.056	3.433	PAFBOR	162.949	3.335	PAFBOR	154.287	3.193
PAFCEI	150.252	3.195	PAFCEI	164.87	2.934	PAFCAE	163.467	2.85	PAFCAE	161.726	2.975	PAFCAE	151.055	2.707
PAFCEI	152.003	3.397	PAFCEI	176.516	3.011	PAFCEI	164.516	3.097	PAFCEI	160.986	2.809	PAFCEI	171.832	2.967
PAFZAC	156.489	2.758	PAFZAC	158.02	3.089	PAFCEI	154.704	2.99	PAFDAF	176.176	3.45	PAFZAC	171.573	3.195
PAGGAH	166.44	3.254	PAGGAH	169.224	2.865	PAFZAC	173.457	3.188	PAGFAF	169.771	3.4	PAGFAF	161.373	3.4
PAGGAH	158.527	3.309	PAGGAH	161.879	2.831	PAGGAH	150.628	3.436	PAGGAH	154.692	3.277	PAGGAH	169.029	3.346
PAGHIS	157.416	2.85	PAGHOY	168.56	2.786	PAGGAH	152.369	2.896	PAGGAH	173.847	3	PAGHIS	162.732	3.338
PAGLEP	166.07	3.207	PAGLEP	172.557	2.942	PAGLEP	172.36	3.073	PAGLEP	173.219	2.97	PAGLEP	166.595	3.239
PAGLEP0	166.482	3.178	PAGLEP0	166.134	3.178	PAGLEP	160.389	2.87	PAGLEP	174.14	3.012	PAGLEP	164.854	3.357
PAGLEP0	156.424	2.842	PAGLEP0	173.039	2.951	PAGLEP0	171.577	2.879	PAGLEP0	171.614	2.989	PAGLEP0	172.74	2.875
PAGLIT	166.919	3.192	PAGLIT	172.078	3.273	PAGLEP0	164.526	3.256	PAGLIT	175.902	3.156	PAGLIT	156.185	3.245
PAGLIV	154.958	3.482	PAGMIT	150.427	3.028	PAGLIT	159.291	3.221	PAGLIT	150.594	2.699	PAGLIT	177.335	3.156
PAHGEP	165.531	3.059	PAHGEP	168.48	2.892	PAGSUM	154.182	3.245	PAGSUM	152.965	3.068	PAHGEP	167.139	3.032
PAHHUC	157.563	3.215	PAHJAL	159.557	3.279	PAHGEP	175.967	3.317	PAHHAJ	155.45	2.963	PAHHAJ	175.202	3.221
PAHJEP	151.657	3.227	PAHJEP	157.906	3.016	PAHJAL	158.003	3.279	PAHJEO	157.457	3.302	PAHJEP	159.912	3.205
PAHKOY	162.457	2.967	PAHKOY	154.522	2.887	PAHJEP	174.336	2.992	PAHJOX	151.545	3.221	PAHKOY	155.395	3.406
PAHKOY	156.908	2.733	PAHKOY	154.097	3.013	PAHKOY	151.68	3.022	PAHKOY	154.948	2.915	PAHKOY	164.275	2.733
PAHKOY0	156.098	2.886	PAHKOY0	151.324	3.025	PAHKOY	162.663	2.6	PAHKOY0	154.531	3.444	PAHKOY0	162.998	2.974
PAHKOY0	156.456	3.004	PAHKOY0	161.908	2.638	PAHKOY0	154.564	2.951	PAHKOY0	163.746	2.725	PAHKOY0	158.461	2.725
PAHNUM	157.156	2.935	PAHNUM	155.111	3.282	PAHLUK	153.606	3.298	PAHNUM	155.088	2.952	PAHNUM	165.654	3.307
PAHNUM	169.714	3.209	PAHNUM	171.6	2.66	PAHNUM	153.295	2.72	PAHNUM	168.211	2.935	PAHNUM	172.982	2.66
PAHSID	154.956	3.383	PAHTUP	161.091	2.973	PAHNUM	155.341	3.257	PAHPAU	168.743	3.325	PAHPAU	169.547	3.325
PAHTUP	156.498	3.447	PAHTUP	150.559	2.983	PAHTUP	152.248	2.755	PAHTUP	163.045	2.767	PAHTUP	156.907	3.408
PAHVIE	167.143	2.996	PAHVIE	156.396	3.07	PAHTUP	169.476	2.898	PAHTUP	152.901	2.887	PAHTUP	151.76	3.184
PAHXOQ	151.21	2.851	PAHXUW	150.99	2.943	PAHVOK	162.356	3.458	PAHXOO	162.426	3.095	PAHXOQ	151.95	2.85
PAHYIL	162.707	3.075	PAHYUV	153.661	3.032	PAHYAD	165.088	3.079	PAHYAD	152.515	2.823	PAHYAD	174.693	3.206
PAJBIM	153.773	3.465	PAJBIM	155.489	3.432	PAHZIJ	161.52	3.196	PAJBIM	178.161	3.19	PAJBIM	160.272	3.332
PAJBUZ	170.817	3.063	PAJGUF	158.668	3.453	PAJBUZ	162.194	3.184	PAJBUZ	161.791	3.184	PAJBUZ	162.896	3.027
PAJHEQ	150.562	2.953	PAJHEQ	151.954	2.816	PAJGUF	169.5	3.453	PAJHAM	156.993	3.093	PAJHAM	150.633	2.829
PAJHEQ	164.27	3.378	PAJJIT	152.496	3.41	PAJHEQ	175.254	2.816	PAJHEQ	155.776	3.131	PAJHEQ	151.305	3.328
PAJJIT	151.824	3.117	PAJJIT	175.608	3.336	PAJJIT	164.099	3.079	PAJJIT	155.043	3.155	PAJJIT	158.975	2.874
PAJJIT10	164.099	3.078	PAJJIT10	155.039	3.155	PAJJIT	151.896	3.153	PAJJIT	159.66	3.396	PAJJIT10	152.551	3.41
PAJJIT10	151.938	3.153	PAJJIT10	159.548	3.4	PAJJIT10	159.042	2.872	PAJJIT10	151.822	3.118	PAJJIT10	175.666	3.337
PAJKUJ	157.883	3.403	PAJKUJ	171.472	2.669	PAJJUJ	169.966	3.304	PAJKEQ	171.384	3.197	PAJKOD	161.555	3.383
PAJYEF	161.036	3.184	PAJYIJ	165.864	2.807	PAJKUJ	150.101	3.403	PAJKUJ	165.55	2.669	PAJRUR	175.255	2.836
PAKTAZ	154.556	3.454	PAKTAZ	168.529	3.408	PAKBAI	150.667	3.057	PAKFAM	150.746	3.002	PAKFEN	172.237	3.148
PAKXEH	151.629	2.915	PAKYOQ	159.239	3.498	PAKTAZ	166.843	3.386	PAKXEH	156.095	3.475	PAKXEH	152.713	3.013
PALBEK	162.852	3.191	PALBEK	160.683	3.452	PAKYOQ	157.798	3.498	PALBEK	162.818	3.425	PALBEK	150.051	3.149
PALDEP	153.832	2.971	PALGAO	157.667	3.249	PALBEK	162.718	2.989	PALCUC	150.27	2.792	PALCUC	159.866	3.444
PALPIE	154.011	3.479	PALPOI	151.439	3.347	PALGES	153.353	3.314	PALNUM	164.891	3.403	PALNUM	169.019	3.261
PALRAW	175.962	3.101	PALRAW	154.675	3.47	PALPOI	150.185	2.753	PALPOI	178.631	3.125	PALQAV	166.366	2.741
PALXOS	164.819	3.26	PALXOS	163.457	3.055	PALRAW	172.427	2.698	PALSED	155.383	3.375	PALSED	157.483	3.375
PALXOS	169.563	3.053	PALXOS	166.7	2.938	PALXOS	154.686	3.081	PALXOS	153.239	3.042	PALXOS	169.504	3.053
PALXOS	159.118	3.236	PALXOS	165.755	3.074	PALXOS	159.888	3.381	PALXOS	163.703	3.381	PALXOS	151.348	3.191
PAMDOY	162.295	2.708	PAMGOB	153.147	2.923	PAMDIS	157.194	2.967	PAMDIS	160.762	2.967	PAMDOY	155.085	2.936
PAMVAD	154.064	2.508	PAMVAD	157.342	2.508	PAMGUH	155.709	2.961	PAMHAO	151.779	3.289	PAMLUM	152.601	3.263
PAMVAD	151.948	3.466	PAMVAD	155.924	3.28	PAMVAD	150.639	3.087	PAMVAD	164.963	3.359	PAMVAD	164.695	3.122

PAMVEH	162.249	2.913	PAMVEH	169.283	3.499	PAMVEH	150.018	3.054	PAMVEH	156.154	3.11	PAMVEH	153.686	2.99
PAMVEH	152.11	3.493	PAMVEH	151.239	3.211	PAMVEH	151.933	3.197	PAMVEH	151.142	3.054	PAMVEH	167.256	3.441
PAMVUX	163.037	3.13	PAMVUX	159.952	3.13	PAMVEH	154.591	2.873	PAMVEH	159.69	2.913	PAMVUX	151.736	3.071
PAMWAE	155.126	2.514	PAMWAE	154.287	2.514	PAMVUX	150.144	2.816	PAMVUX	150.5	2.874	PAMVUX	157.247	3.42
PAMWAE	153.474	3.464	PAMWAE	156.23	3.262	PAMWAE	150.411	3.04	PAMWAE	165.776	3.319	PAMWAE	164.73	3.163
PAMWEI	159.413	3.06	PAMWEI	151.105	3.058	PAMWEI	165.708	3.022	PAMWEI	175.048	2.807	PAMWEI	152.317	2.829
PAMZIP	152.151	3.486	PAMZIP	160.593	3.078	PAMWEI	167.015	3.233	PAMWEI	167.708	3.043	PAMZEL	154.694	3.27
PANHUH	171.879	3.237	PANHUH	150.177	3.22	PAMZIP	164.771	2.875	PANGOC	152.25	3.196	PANGOC	154.707	3.196
PANHUH	152.657	2.647	PANHUH	158.408	3.22	PANHUH	173.661	3.36	PANHUH	157.448	3.289	PANHUH	171.126	3.349
PANHUH1	157.448	3.289	PANHUH1	171.126	3.349	PANHUH1	171.879	3.237	PANHUH1	150.177	3.22	PANHUH1	173.661	3.36
PANJIB	159.951	2.97	PANJIB	171.74	2.915	PANHUH1	152.657	2.647	PANHUH1	158.408	3.22	PANJIB	166.308	2.593
PANJOH	153.334	2.863	PANJOH	151.883	3.402	PANJIB	164.991	2.893	PANJIB	152.887	2.72	PANJOH	170.482	3.002
PANJUJ	171.042	3.003	PANJUJ	153.691	3.109	PANJOH	153.579	2.795	PANJOH	163.473	2.848	PANJOH	161.218	2.895
PANPIG	165.151	3.221	PANPIG	158.32	2.891	PANJUJ	150.252	3.003	PANLAS	156.667	2.9	PANPIG	170.293	3.217
PANREF	178.408	2.88	PANREF	165.209	3.412	PANREF	151.524	2.632	PANREF	165.523	2.866	PANREF	167.827	3.346
PANWOU	162.775	2.95	PANWUA	154.23	3.111	PANREF	170.073	3.233	PANROL	171.934	3.017	PANVEI	150.505	2.98
PANXOT	166.274	2.751	PANXUZ	157.086	3.3	PANWUA	151.571	3.111	PANXEL	152.418	3.499	PANXEL	163.359	3.107
PAPTUW	164.847	3.061	PAPTUW	153.255	2.893	PAPRAA	161.666	3.099	PAPREF	173.246	2.801	PAPRIJ	155.896	3.007
PAPWAI	164.39	3.203	PAQJOJ	155.919	2.839	PAPTUW	159.866	3.263	PAPTUW	159.024	3.455	PAPTUW	168.288	3.135
PAQSIK	151.284	2.729	PAQTAE	158.279	2.686	PAQJOJ	162.448	2.947	PAQJOJ	164.063	3.157	PAQLOK	161.467	3.107
PAQTAE	167.295	2.684	PAQTAE	154.276	3.447	PAQTAE	167.291	2.838	PAQTAE	163.967	3.471	PAQTAE	165.483	2.662
PAQTAE	155.078	2.853	PAQTAE	169.288	3.093	PAQTAE	168.972	3.457	PAQTAE	171.578	2.951	PAQTAE	153.105	3.239
PAQTAE	154.216	3.397	PAQTAE	151.984	2.882	PAQTAE	159.537	3.217	PAQTAE	162.687	3.285	PAQTAE	159.408	3.13
PAQTAE	164.311	2.69	PARLUT	153.758	3.204	PAQTAE	159.661	2.817	PAQTAE	159.554	3.475	PAQTAE	159.802	3.274
PARTAH	159.109	3.368	PARVEN	151.635	3.102	PARLUT	151.359	3.064	PARLUT	157.578	3.05	PARTAH	162.715	3.131
PARVEN	175.473	2.754	PARVEN	167.456	2.754	PARVEN	161.31	3.114	PARVEN	165.313	3.176	PARVEN	170.772	3.102
PARVIR	173.047	2.753	PARVIR	173.06	3.047	PARVIR	156.245	3.047	PARVIR	161.7	3.112	PARVIR	170	2.753
PARYAM	151.002	3	PARYEQ	163.766	3.187	PARVIR	161.751	3.301	PARYAJ	155.804	3.5	PARYAJ	170.415	2.88
PARYEQ	162.629	3.153	PARYIT	152.714	3.394	PARYEQ	166.388	3.238	PARYEQ	153.191	3.481	PARYEQ	169.178	3.125
PARZUE	163.097	3.16	PARZUE	166.755	3.287	PARZAN	154.19	2.976	PARZOA	177.463	2.852	PARZUE	176.328	3.136
PASKIE	174.16	3.267	PASKUR	150.741	3.271	PARZUE	152.038	3.012	PARZUE	166.997	3.136	PASKIE	159.496	3.231
PASLIH	164.096	3.317	PASQIJ	154.676	3.422	PASKUR	163.985	2.85	PASLAY	161.991	3.437	PASLEC	152.131	3.234
PASQIJ	150.9	2.797	PASTIQ	175.167	3.344	PASQIJ	163.117	3.374	PASQIJ	165.384	3.358	PASQIJ	168.944	3.234
PATBIZ	159.563	3.498	PATBIZ	153.102	3.309	PASTIQ	158.643	2.664	PASVUB	169.727	3.404	PASWEM	152.663	3.186
PATBIZ	167.892	3.343	PATBIZ	175.828	3.053	PATBIZ	163.564	3.073	PATBIZ	167.918	3.027	PATBIZ	173.025	3.066
PATBIZ	157.121	3.349	PATBIZ	151.477	3.258	PATBIZ	151.754	3.048	PATBIZ	162.296	3.192	PATBIZ	157.745	3.273
PATCAS	151.532	2.666	PATCAS	155.483	2.796	PATBIZ	158.67	3.122	PATBIZ	156.863	3.053	PATCAS	155.705	3.213
PATCAS	170.334	2.774	PATCAS	159.46	2.74	PATCAS	172.492	3.117	PATCAS	161.104	2.827	PATCAS	160.203	2.786
PATCEW	153.022	2.901	PATCEW	159.893	3.139	PATCAS	155.138	3.087	PATCEW	161.472	2.967	PATCEW	176.364	3.139
PATCEW	151.361	2.901	PATCEW	155.541	3.166	PATCEW	168.581	3.01	PATCEW	161.426	3.216	PATCEW	156.491	3.317
PATCUM	153.211	3.046	PATDAT	155.887	3.499	PATCUM	172.109	3.024	PATCUM	156.786	2.834	PATCUM	171.363	2.862
PATGIE	155.92	3.234	PATGIE	162.836	2.983	PATGIE	158.12	2.817	PATGIE	155.648	2.866	PATGIE	157.986	2.872
PAVKOQ	164.941	3.08	PAVMEG	153.602	2.873	PATREK	168.611	3.154	PATREK	160.874	2.842	PAVBOG	153.558	3.086
PAWLUY	154.972	3.271	PAWLUY	176.517	3.256	PAWKUV	159.118	3.235	PAWLOS	156.339	3.484	PAWLUY	154.083	3.41
PAWMAF	162.602	3.47	PAWPOU	168.709	3.224	PAWLUY	155.953	3.025	PAWMAF	178.662	3.331	PAWMAF	159.061	3.104
PAXJUX	162.948	2.901	PAXJUX	167.836	3.158	PAWQIO	159.293	3.375	PAWXOD	173.942	3.326	PAXJUX	158.845	3.331
PAXREN	152.07	3.267	PAXREN	153.755	3.381	PAXLUW	164.256	3.153	PAXMUW	169.452	3.287	PAXMUW	155.49	3.473
PAXWEQ	150.17	3.064	PAXWEQ	156.743	3.003	PAXWEQ	155.599	2.978	PAXWEQ	151.309	2.811	PAXWEQ	159.741	2.859
PAXWEQ(	167.383	2.754	PAXWEQ(	158.821	2.997	PAXWEQ	151.985	2.909	PAXWEQ(	158.976	2.953	PAXWEQ(	157.049	2.949
PAXXER	155.343	3.128	PAXXER	169.996	3.13	PAXWEQ(	150.31	2.821	PAXWEQ(	161.935	2.833	PAXXER	168.481	3.033
PAZCUR	155.021	2.907	PAZCUR	158.318	3.093	PAYVER	159.979	3.374	PAYVER	154.681	3.425	PAZCUR	164.251	3.133
PAZCUR	169.273	2.757	PAZDAY	156.494	3.031	PAZCUR	151.236	2.769	PAZCUR	158.146	2.757	PAZCUR	160.204	3.384
PAZDAY	154.208	3.485	PAZDIG	150.154	2.866	PAZDAY	153.396	3.169	PAZDAY	162.696	3.002	PAZDAY	163.12	3.271
PAZDIG	168.994	2.747	PAZDIG	173.564	3.244	PAZDIG	167.072	2.782	PAZDIG	166.849	2.945	PAZDIG	157.771	3.037
PAZDIG	151.346	2.992	PAZDIG	174.345	3.221	PAZDIG	164.405	3.349	PAZDIG	162.537	2.81	PAZDIG	167.514	2.881
PAZJOT	150.795	3.337	PAZJOT	171.061	3.44	PAZHIL	170.556	2.783	PAZJIN	156.44	3.492	PAZJIN	150.365	3.441
PAZLOS	161.283	3.064	PAZMEI	155.864	3.394	PAZJOT	155.32	3.441	PAZKAE	162.179	3.391	PAZLOS	167.393	2.896
PAZYEY	150.13	3.248	PAZYEY	165.009	3.007	PAZMEI	165.747	3.023	PAZMEI	156.38	3.44	PAZWAR	157.045	2.944
PAZYEY	152.101	2.738	PEBCUY	153.718	3.401	PAZYEY	150.843	3.101	PAZYEY	162.007	3.264	PAZYEY	173.699	2.738
PEBMOY	163.969	3.499	PEBREW	160.916	2.952	PEBLIU	152.289	3.271	PEBLIU	156.364	3.054	PEBMOY	168.541	3.292
PECSAU	163.475	3.389	PECSAU	157.943	3.102	PECGIN	175.648	3.145	PECLOB	153.09	3.481	PECSAU	155.772	2.911
PECSEY	174.641	3.221	PECSEY	166.036	3.054	PECSEW	153.292	3.07	PECSEW	156.27	3.303	PECSEW	153.073	3.447
PECTAW	151.139	2.943	PECTIE	150.737	2.91	PECSIC	166.962	2.87	PECSIC	162.035	3.355	PECSIC	154.215	2.912
PECZOQ	159.587	2.746	PECZOQ	164.123	3.361	PECWAY	167.923	2.77	PECZOQ	151.424	3.142	PECZOQ	152.792	3.126
PECZUW	170.588	3.346	PECZUW	160.559	3.001	PECZUW	170.206	2.581	PECZUW	168.679	2.865	PECZUW	160.017	3.326
PEDBAD0	168.889	2.918	PEDBIN	153.316	2.817	PEDBAD	160.444	3.19	PEDBAD	168.874	2.909	PEDBAD0	160.909	3.191
PEDBIN	164.094	2.906	PEDBIN	165.694	2.906	PEDBIN	161.979	3.037	PEDBIN	150.341	2.811	PEDBIN	162.228	2.879
PEDGOV	154.293	2.993	PEDGOV	157.936	2.993	PEDBIN	156.99	3.214	PEDBIN	165.99	2.851	PEDCUA	153.958	3.215
PEDMAN	153.867	3.413	PEDPUK	168.727	2.76	PEDGOV0	166.594	3.386	PEDGUB	157.053	3.1	PEDLUG	155.749	3.462
PEDPUK0	169.043	2.794	PEDPUK0	158.256	3.079	PEDPUK	157.523	3.089	PEDPUK	158.316	3.203	PEDPUK	175.393	2.929
PEDPUK0	158.61	3.08	PEDPUK0	161.556	3.229	PEDPUK0	160.595	3.215	PEDPUK0	171.593	3.02	PEDPUK0	169.782	2.816
PEDQIZ	150.054	3.335	PEDQIZ	159.522	3.277	PEDPUK0	150.124	2.816	PEDPUK0	169.184	3.081	PEDQIZ	162.282	2.797
PEDRIZ	162.812	3.131	PEDRIZ	152.832	3.272	PEDQIZ	159.262	2.842	PEDRIZ	158.869	3.115	PEDRIZ	153.464	3.072
PEDRIZ10	153.464	3.072	PEDRIZ10	162.812	3.131	PEDRIZ	172.138	3.432	PEDRIZ	164.684	2.803	PEDRIZ10	158.869	3.115
PEDWOM	161.103	3.056	PEDWOM	166.191	3.464	PEDRIZ10	152.832	3.272	PEDRIZ10	172.138	3.432	PEDRIZ10	164.684	2.803
PEDYED	157.88	3.296	PEDZOQ	154.937	2.771	PEDWOM	152.663	3.111	PEDYED	162.859	2.84	PEDYED	151.287	3.315
PEFHIS01	161.894	2.619	PEFJAN	165.568	3.164	PEFHIS	175.175	3.059	PEFHIS	160.295	2.625	PEFHIS01	175.381	3.011
PEFJUG	174.478	2.698	PEFKAN	167.143	2.761	PEFJOA	156.792	2.872	PEFJOA	156.751	2.97	PEFJUG	166.52	2.698
PEFNEW	161.385	2.806	PEFPID	165.109	3.052	PEFNEW	167.496	2.958	PEFNEW	151.723	3.269	PEFNEW	162.415	2.966

PEHCAH	157.461	3.176	PEHCAH	154.486	2.925	PEFROK	156.485	3.293	PEGDIR	154.143	3.327	PEGLOE	153.222	2.923
PEHFIS	151.13	3.077	PEHFIS	166.547	2.773	PEHCOY	158.758	3.236	PEHCOY	158.797	3.218	PEHDEP	168.744	3.27
PEHJOD	163.798	3.422	PEHJUJ	152.612	3.078	PEHFIS	168.008	2.783	PEHFOY	167.566	2.753	PEHJOD	156.763	2.978
PEHKEU	166.965	2.878	PEHKEU	174.3	2.878	PEHJUJ	165.407	3.078	PEHJUJ	164.631	3.209	PEHKEU	178.897	3.474
PEHLOD	151.199	2.826	PEHLOD	170.878	3.098	PEHKEU	165.299	3.474	PEHLOD	153.393	3.175	PEHLOD	151.217	3.037
PEJKOE	167.413	3.358	PEJZEK	168.234	3.002	PEHLOD	158.593	3.075	PEHLOD	175.272	2.834	PEHYOS	153.864	3.44
PEKNAV	163.062	3.313	PEKNAV	154.279	3.374	PEJZOV	159.368	2.902	PEJZOV	164.854	3.001	PEJZOV	160.714	3.083
PEKQAX0	155.121	3.425	PEKQAX0	157.487	3.048	PEKNAV	151.106	3.141	PEKQAX	156.503	3.308	PEKQAX0	157.283	2.968
PELNUQ	151.958	3.382	PELNUQ	155.122	3.234	PEKQAX0	152.24	2.886	PELNIG	154.78	3.38	PELNIG	170.128	3.472
PELWOT	156.431	2.902	PELWOT	166.881	3.433	PELTIN	155.077	2.993	PELWOT	155.772	2.776	PELWOT	154.795	3.421
PEMFAC	155.449	3.302	PEMKUR0	153.222	3.144	PEMBIW	170.539	2.759	PEMBIW	152.135	3.121	PEMBOC	160.991	3.245
PEMMUP	160.593	3.259	PEMMUP	156.861	3.042	PEMMUP	151.888	3.052	PEMMUP	165.964	2.658	PEMMUP	176.057	2.658
PENFET	155.972	3.148	PENFOG	169.125	2.896	PEMVUC	151.486	3.068	PENBUH	152.836	3.451	PENDUH	152.7	3.3
PENFOG	158.029	3.135	PENFOG	155.615	3.185	PENFOG	173.303	3	PENFOG	166.748	2.82	PENFOG	151.561	3.266
PENNOM	157.741	3.076	PENQUV	160.653	3.397	PENNOM	151.57	3.338	PENNOM	157.62	2.744	PENNOM	152.025	2.744
PENRIL	159.442	3.271	PENRIL	167.81	2.816	PENQUW	168.234	2.962	PENRIL	154.984	3.187	PENRIL	163.792	2.825
PENRIL	153.139	3.43	PENRIL	152.061	2.751	PENRIL	173.724	2.852	PENRIL	160.433	3.245	PENRIL	154.411	3.267
PENRIL	162.261	2.805	PEPGOK	155.548	3.241	PENRIL	156.027	3.122	PENRIL	152.591	3.109	PENRIL	151.611	3.025
PEPGOK0	155.332	3.198	PEPGUN	154.852	3.16	PEPGOK	153.808	3.248	PEPGOK0	155.347	3.277	PEPGOK0	155.173	3.278
PEPRUX	162.79	3.364	PEPWIS	153.386	3.401	PEPGUN	154.485	3.16	PEPJEZ	153.836	3.28	PEPRIN	150.467	3.29
PEQCEV	154.426	2.816	PEQCIB	155.742	2.897	PEPWIS	153.448	3.04	PEPXEP	155.613	3.438	PEQCAT	164.373	3.244
PEQCIZ	154.558	2.78	PEQCOF	160.517	3.086	PEQCIB	155.738	3.478	PEQCIB	165.006	2.767	PEQCIB	165.168	3.357
PEQDEW	162.532	3.393	PEQDIA	171.482	3.352	PEQDAS	158.586	3.466	PEQDEW	168.238	2.966	PEQDEW	164.315	3.294
PEQRIQ	155.273	3.077	PEQRIQ	154.217	2.966	PEQGAW	165.349	3.144	PEQHEA	163.337	2.916	PEQRIQ	163.351	2.947
PEQRIQ	157.276	2.933	PEQRIQ	163.91	3.397	PEQRIQ	170.891	2.714	PEQRIQ	155.295	2.925	PEQRIQ	152.65	3.419
PEQRIQ	171.67	2.887	PEQRIQ	151.681	3.037	PEQRIQ	154.651	2.93	PEQRIQ	151.721	3.222	PEQRIQ	159.098	3.499
PEQRIQ	163.32	3.013	PEQRIQ	166.676	2.962	PEQRIQ	157.714	3.381	PEQRIQ	151.996	3.391	PEQRIQ	156.887	3.169
PEQROW	155.71	3.067	PEQROW	154.214	2.963	PEQRIQ	150.693	3.21	PEQROW	151.875	3.224	PEQROW	171.977	2.883
PEQROW	157.505	3.38	PEQROW	163.995	3.394	PEQROW	163.286	2.943	PEQROW	152.105	3.045	PEQROW	151.524	3.401
PEQROW	155.328	2.926	PEQROW	154.99	2.927	PEQROW	157.246	2.927	PEQROW	152.542	3.398	PEQROW	170.377	2.706
PEQROW	150.772	3.194	PEQSEN	150.401	2.961	PEQROW	157.084	3.172	PEQROW	162.567	3.031	PEQROW	166.704	2.973
PEQSEN	157.673	3.358	PEQSEN	156.446	3.471	PEQSEN	152.739	2.814	PEQSEN	172.102	3.091	PEQSEN	171.026	2.815
PEQVEO	168.793	3.232	PEQVEO	154.048	3.041	PEQSIO	153.103	3.082	PEQSIO	155.161	3.327	PEQVEO	154.107	3.103
PEQVIS	151.554	2.799	PEQVIS	152.514	2.807	PEQVEO	159.278	3.232	PEQVIS	154.882	2.893	PEQVIS	150.144	2.772
PEQVIS	159.173	3.408	PEQVIS	150.404	2.807	PEQVIS	157.252	3.241	PEQVIS	164.518	3.298	PEQVIS	152.991	3.039
PEQWIT	152.621	3.479	PEQWIT	166.714	3.15	PEQVIS	150.981	3.343	PEQWEP	164.419	3.208	PEQWIT	168.297	2.886
PEQWIT	173.453	3.474	PEQWIT	169.118	2.92	PEQWIT	152.821	3.09	PEQWIT	152.531	3.118	PEQWIT	156.479	2.676
PEQWIT	160.739	2.975	PEQWIT	164.361	3.041	PEQWIT	167.805	2.95	PEQWIT	169.836	3.019	PEQWIT	169.269	3.178
PEQWIT	170.456	3.282	PEQWIT	151.02	2.835	PEQWIT	170.2	2.987	PEQWIT	158.028	3.211	PEQWIT	155.894	2.987
PEQXUH	166.506	2.843	PEQXUH	156.25	3.149	PEQWIT	153.269	2.848	PEQXUH	165.146	3.167	PEQXUH	150.541	2.803
PERLAC	153.777	2.697	PERLAC	177.695	2.768	PEQXUH	156.579	3.047	PEQXUH	152.143	3.13	PERDAT	155.688	3.163
PESCEV	176.812	3.271	PESCEV	156.765	3.116	PERSAK	166.892	3.441	PERSIS	164.649	3.295	PERXUJ	169.268	3.492
PESNUY	166.386	3.482	PESNUY	160.509	3.371	PESCEV	160.207	2.848	PESCEV	153.919	2.919	PESCEV	154.99	3.493
PESQIQ	170.542	2.977	PESQIQ	154.092	3.266	PESPEL	166.157	3.389	PESPEL	170.985	3.389	PESQIQ	164.242	2.952
PETCEA	155.338	3.202	PETGIG	159.724	3.466	PESQIQ	174.035	2.773	PESQIQ	151.569	2.692	PESWAO	169.538	2.998
PETJOP	168.189	3.019	PETJUV	161.599	3.109	PETGIG	171.347	2.952	PETGIG	162.999	2.844	PETJIJ	154.449	2.986
PETNOV	177.662	3.151	PETNOV	156.619	3.472	PETJUV	159.799	3.109	PETNOT	158.296	3.115	PETNOT	158.838	3.229
PETPIP	167.16	3.299	PETPIR	152.115	3.357	PETNUZ	158.648	3.152	PETNUZ	160.012	3.31	PETPIP	159.035	3.215
PETPUB	171.523	3.176	PETQAI	156.157	3.338	PETPOV	161.333	3.277	PETPOV	169.237	3.429	PETPUB	170.732	3.192
PEVROB	157.844	3.431	PEVROB	161.19	3.035	PETTUF	173.727	3.126	PETXUK	157.887	3.467	PETZEX	158.785	3.471
PEVSEO	153.394	3.191	PEVVIV	151.004	3.341	PEVSEO	152.091	3.371	PEVSEO	159.981	2.942	PEVSEO	158.755	2.893
PEVZEW	155.792	2.957	PEWBAU	158.786	3.082	PEVVIV	163.482	2.964	PEVVIV	158.337	2.726	PEVZEW	152.16	2.96
PEWFUU	157.187	3.351	PEWGOQ	150.43	3.345	PEWBAU	164.368	2.952	PEWBAU	168.711	2.934	PEWFIJ	158.715	3.161
PEXDOO	155.46	3.004	PEXSAM	153.579	2.639	PEWVAR	163.953	2.741	PEWVAR	159.47	2.99	PEWVAR	152.646	3.305
PEXXUL	155.696	3.015	PEYCOM	164.047	2.999	PEXSAM	153.454	2.728	PEXTOF	159.786	3.296	PEXXUL	156.534	3.288
PEYLOV	156.189	2.892	PEYOFB1	153.734	2.695	PEYDEE	151.407	2.929	PEYDEE	165.283	3.325	PEYLOV	152.711	2.892
PEZJOW	163.53	3.242	PEZTUM	166.132	3.469	PEYOFB1	152.158	2.695	PEZJOW	160.479	3.212	PEZJOW	160.666	3.026
PFBZAC	155.324	3.264	PFBZAC	151.875	3.082	PEZYUR	171.909	3.42	PEZZUQ	167.214	3.336	PFBZAC	165.767	2.682
PFBZAC01	165.208	2.637	PFBZAC01	151.503	3.089	PFBZAC	153.3	2.826	PFBZAC	156.128	2.826	PFBZAC	155.382	2.691
PFBZAC01	154.773	2.627	PFDPAY01	150.707	2.941	PFBZAC01	155.234	3.254	PFBZAC01	152.161	2.76	PFBZAC01	155.382	2.76
PFPHHG0	177.138	3.403	PFPHHG0	152.133	2.999	PFDPAY01	150.528	2.897	PFPHGE0	158.412	3.019	PFPHHG0	159.413	3.481
PFPHHG0	154.1	3.263	PFPHHG0	165.634	2.762	PFPHHG0	151.2	3.465	PFPHHG0	158.758	3.418	PFPHHG0	155.317	2.832
PFPHHG0	158.546	3.436	PFPHHG0	175.049	3.401	PFPHHG0	168.43	3.184	PFPHHG0	178.34	3.39	PFPHHG0	168.349	2.896
PFPHSE01	161.92	3.026	PFPHSE01	164.397	3.104	PFPHHG0	158.983	3.285	PFPHHG0	154.256	2.819	PFPHHG0	152.581	2.936
PFPHSE01	159.741	3.009	PFPHSE01	152.504	3.088	PFPHSE01	174.26	3.126	PFPHSE01	162.492	3.043	PFPHSE01	154.082	2.884
PFPOPS	156.8	3.416	PFPOPS	155.761	3.268	PFPOPS	158.734	3.292	PFPOPS	163.188	3.149	PFPOPS	163.482	3.159
PFPOPS	164.686	3.027	PFTEBZ11	158.914	3.107	PFPOPS	175.923	2.939	PFPOPS	151.949	3.354	PFPOPS	169.149	2.942
PIBCUC	169.473	3.159	PIBCUC	157.454	3.03	PFTENT02	150.088	2.983	PFTENT11	150.066	2.98	PFTENT22	150.167	3.082
PIBCUC	162.909	3.276	PIBCUC	173.976	3.098	PIBCUC	170.774	3.152	PIBCUC	172.537	3.128	PIBCUC	173.432	3.164
PIBCUY	158.921	3.105	PIBCUY	151.35	3.09	PIBCUC	171.812	3	PIBCUC	171.289	3.343	PIBCUC	164.328	3.075
PIBCUY01	163.057	2.981	PIBCUY01	166.572	3.441	PIBCUY	152.056	3.224	PIBCUY	163.402	3.009	PIBCUY	150.004	3.056
PIBGOW	164.643	3.236	PIBGOW	177.694	3.269	PIBCUY01	152.318	3.093	PIBCUY01	157.67	2.89	PIBGOW	155.069	3.408
PIBJOC	156.613	2.832	PIBJOC	154.143	3.095	PIBGOW	160.782	3.298	PIBJOC	164.231	3.095	PIBJOC	169.521	2.923
PIBSUS	163.276	3.012	PIBSUS	160.785	2.877	PIBSUS	160.187	2.997	PIBSUS	151.555	2.721	PIBSUS	163.619	3.113
PIBSUS	158.7	2.783	PIBSUS	164.495	2.999	PIBSUS	155.282	2.978	PIBSUS	152.673	3.058	PIBSUS	157.204	3.41
PIBTED	158.577	3.332	PIBTED	166.54	3.162	PIBTAZ	166.664	3.403	PIBTAZ	158.977	2.858	PIBTAZ	155.307	2.687
PIBTIH	161.929	3.144	PIBTIH	157.242	3.145	PIBTED	171.63	3.04	PIBTED	159.401	3.074	PIBTED	154.636	2.915
PIBTON	161.083	3.109	PIBTON	159.134	3.13	PIBTIH	170.397	2.835	PIBTIH	160.997	2.94	PIBTON	157.201	2.809
PIBWEE	152.359	3.252	PICFIR	151.754	3.404	PIBTON	152.283	3.13	PIBTON	153.455	3.491	PIBTON	160.5	3.213

PICHAO	158.401	3.302	PICHAO	152.639	3.268	PICHAO	165.742	2.89	PICHAO	150.457	2.784	PICHAO	167.349	2.858
PICHUH	152.139	3.22	PICHUH	152.403	3.241	PICHOC	160.406	3.28	PICHOC	167.493	3.106	PICHOC	153.777	2.711
PICHUH	158.048	3.22	PICHUH	176.857	3.212	PICHUH	159.63	3.29	PICHUH	152.922	2.658	PICHUH	159.046	3.231
PICMIY	156.678	2.678	PICMIY	152.839	3.36	PICHUH	166.549	3.246	PICMIY	157.82	3.22	PICMIY	150.996	3.168
PICMOE	155.631	3.478	PICMOE	161.277	3.158	PICMOE	152.797	3.473	PICMOE	150.956	2.9	PICMOE	158.384	3.278
PICMOE01	164.518	3.277	PICMUK	160.587	3.377	PICMOE01	157.698	2.969	PICMOE01	174.167	3.076	PICMOE01	154.407	3.49
PICMUK	155.75	3.272	PICMUK	154.536	2.827	PICMUK	173.005	3.136	PICMUK	166.745	3.408	PICMUK	160.711	2.906
PICMUK	157.824	2.627	PICPEY	155.512	2.989	PICMUK	158.245	3.253	PICMUK	151.651	2.794	PICMUK	157.184	3.314
PICPIB	172.886	2.885	PICPIB	151.557	3.086	PICPIB	158.264	3.013	PICPIB	155.491	3.361	PICPIB	152.264	2.992
PICPUN	174.549	3.031	PICPUN	163.995	3.225	PICPIB	152.456	3.246	PICPOH	175.653	3.041	PICPOH	163.52	3.217
PICQAU	156.774	3.139	PICQAU	156.339	3.494	PICQAU	172.354	3.065	PICQAU	156.884	3.442	PICQAU	165.936	3.016
PICQAU	153.687	2.916	PICQEY	172.065	3.012	PICQAU	162.167	3.023	PICQAU	167.823	2.86	PICQAU	150.573	3.42
PICQEY	158.214	3.126	PICQIC	178.623	3.071	PICQEY	150.236	3.467	PICQEY	152.601	3.378	PICQEY	154.869	3.308
PICVIK01	156.377	3.295	PICYOT	157.112	2.841	PICQIC	161.071	2.673	PICQIC	163.743	3.162	PICVIK	158.924	3.238
PIDDEM	166.76	3.088	PIDDEM	159.027	3.49	PICYOT	164.013	3.45	PICZOU	166.188	3.27	PIDDEM	150.633	3.492
PIFJOF	160.558	3.324	PIFJOF	153.434	3.453	PIDMOF	154.812	3.119	PIFJOF	156.77	3.333	PIFJOF	159.382	3.349
PIFNOI	170.541	3.018	PIFNOI	152.106	3.266	PIFJOF	176.534	3.079	PIFJOF	170.889	3.079	PIFNOI	173.087	3.232
PIFPUS	165.841	3.329	PIFPUS	151.93	2.88	PIFNOI	157.157	3.159	PIFNOI	152.363	3.439	PIFNOI	155.776	2.893
PIFWAD	162.354	3.097	PIFWAD	174.01	3.192	PIFPUS	154.605	3.207	PIFVIK	176.869	2.977	PIFVIL	170.818	3.133
PIFWAD	161.053	3.25	PIFWAD	164.183	3.029	PIFWAD	161.276	3.029	PIFWAD	161.655	3.456	PIFWAD	163.747	3.073
PIGPUT	154.674	3.044	PIGXOS	166.034	3.17	PIFZUC	167.2	2.864	PIGBIT	164.018	2.968	PIGPON	154.961	3.002
PIGXOS	160.164	2.969	PIGXOS	158.289	3.236	PIGXOS	161.719	3.415	PIGXOS	169.288	2.971	PIGXOS	163.168	3.434
PIHJEZ	160.859	3.248	PIHJEZ	161.387	2.863	PIGXOS	154.901	3.348	PIGZOW	174.49	3.096	PIHJEZ	150.667	3.409
PIHMUS	153.322	3.252	PIHMUS	166.06	3.34	PIHJEZ	154.137	3.082	PIHMIG	171.509	2.939	PIHMIG	172.636	2.962
PIHMUS01	163.485	3.139	PIJHOJ	151.155	3.205	PIHMUS	150.253	2.771	PIHMUS01	174.358	3.399	PIHMUS01	163.609	3.279
PIJKUP	155.526	3.183	PIJLAY	154.053	3.224	PIJKAW	156.729	3.331	PIJKAW	157.27	2.797	PIJKEA	151.862	2.814
PIJXOZ	157.363	3.025	PIJXOZ	172.748	3.025	PIJLEC	154.143	3.231	PIJXOZ	154.309	2.887	PIJXOZ	151.374	2.769
PIJYAK	165.981	3	PIKBAP	165.113	3.15	PIJYAK	171.644	3.242	PIJYAK	161.487	3.444	PIJYAK	158.287	2.814
PIKBET	170.38	3.422	PIKBET	154.114	2.751	PIKBAP	171.065	2.747	PIKBAP	152.229	2.697	PIKBAP	155.931	3.195
PIKCUJ	166.535	3.231	PIKCUJ	177.048	2.759	PIKBET	150.873	3.433	PIKCUJ	153.423	3.432	PIKCUJ	151.671	2.834
PIKDAQ	157.494	2.99	PIKDIY	168.326	3.022	PIKCUJ	157.619	2.92	PIKDAQ	158.821	3.132	PIKDAQ	159.087	2.853
PIKGUP	151.739	2.657	PIKGUP	168.662	3.178	PIKDIY	170.882	2.943	PIKDIY	165.354	2.834	PIKDIY	151.237	3.043
PIKHOI	163.539	2.998	PIKKIE	170.599	3.107	PIKGUP	151.171	2.526	PIKHAW	158.887	3.144	PIKHAW	177.181	3.144
PIKQUY	159.947	3.397	PIKQUY	152.632	3.02	PIKKIE	150.475	3.192	PIKKIE	157.033	3.245	PIKQUY	161.499	3.155
PIKRUW	172.081	3.274	PIKXAK	155.793	3.434	PIKRUW	177.056	3.171	PIKRUW	153.572	3.149	PIKRUW	163.738	2.817
PIKXIT	161.82	2.978	PIKXIT	173.208	2.674	PIKXAK	159.377	3.149	PIKXAK	155.496	3.312	PIKXIT	157.473	3.28
PILBEV	174.319	2.873	PILHEX	151.365	3.109	PIKXIT	166.782	2.978	PIKXIT	169.841	2.878	PIKZER	162.053	3.43
PILKIE	161.601	3.436	PILMIG	155.638	3.261	PILJUS	170.208	2.919	PILKED	155.64	3.252	PILKED	157.724	3.252
PILMIG	151.716	3.408	PILNUU	160.716	3.369	PILMIG	159.903	2.961	PILMIG	162.949	3.323	PILMIG	160.557	2.965
PILRUY	165.158	3.371	PILSEK	153.61	2.744	PILNUU	155.944	3.421	PILNUU	159.193	3.186	PILRUY	160.111	3.403
PILXEP	160.591	3.201	PILXIT	151.386	3.454	PILSIM	163.101	3.358	PILXEP	169.714	3.126	PILXEP	154.735	3.298
PILXOZ	152.124	2.725	PILXUF	165.092	2.924	PILXOZ	161.807	3.063	PILXOZ	154.369	3.027	PILXOZ	171.135	2.725
PILXUF	168.677	2.882	PIMLUU	167.335	3.051	PILXUF	162.909	2.945	PILXUF	159.911	3.444	PILXUF	160.662	3.268
PIMLUU	170.521	3.114	PIMLUU	158.215	3.32	PIMLUU	166.807	2.839	PIMLUU	152.614	3.432	PIMLUU	151.827	3.007
PIMROU	157.031	3.094	PIMROU	168.312	2.797	PIMMAB	156.479	3.178	PIMMAB	165.514	3.43	PIMROU	167.324	3.057
PIMSAH	154.646	2.955	PIMSAH	152.237	3.177	PIMROU	155.078	2.666	PIMSAH	154.889	3.235	PIMSAH	153.237	3.288
PIMSAH	156.027	3.289	PIMSAH	154.236	2.662	PIMSAH	150.633	2.791	PIMSAH	156.38	3.171	PIMSAH	170.352	3.037
PIMSEL	162.777	2.757	PIMSOV	166.642	2.842	PIMSAH	157.377	3.409	PIMSAH	164.694	2.666	PIMSEL	175.176	2.757
PIMSOV	162.751	3.157	PIMSOV	160.387	2.943	PIMSOV	163.662	2.842	PIMSOV	151.259	3.071	PIMSOV	166.42	3.434
PINKUV	173.419	2.854	PINLUV	160.603	2.919	PINFIF	170.467	3.065	PINGUS	174.914	3.116	PINKOP	157.459	3.216
PINLUV01	165.863	3.446	PINLUV01	168.418	3.407	PINLUV	166.866	3.337	PINLUV	167.621	3.346	PINLUV01	162.978	2.964
PINWUG0	150.997	3.25	PINWUG0	152.38	3.392	PINQIO	156.718	3.043	PINQUB	160.042	3.449	PINRUZ	171.518	3.468
PINWUG0	158.825	3.25	PINWUG0	175.899	3.249	PINWUG0	161.321	3.309	PINWUG0	153.907	2.655	PINWUG0	161.607	3.219
PIPBEX	156.502	3.39	PIPBEX	165.957	2.767	PINWUG0	166.513	3.259	PINZIX	178.349	3.396	PINZIX	152.357	3.364
PIPBIB	156.283	2.851	PIPYUL	151.134	3.419	PIPBEX	157.657	3.274	PIPBIB	159.041	3.404	PIPBIB	173.329	3.34
PIQJEE	169.903	2.78	PIQJEE	167.599	2.78	PIPZAS	150.675	3.431	PIPZAS	163.864	3.382	PIPZAS	160.604	2.905
PIRBAV	159.267	2.766	PIRBAV	160.18	2.766	PIQSIU	152.15	2.938	PIQVIY	152.651	2.789	PIRBAV	154.586	3.189
PIRJEH	151.473	3.284	PIRJEH	165.876	2.987	PIRBAV	158.143	2.786	PIRHUV	175.045	3.019	PIRJAD	168.063	3.225
PIRNOV01	163.023	3.33	PIRNUB	168.554	2.896	PIRLEL	156.709	3.266	PIRMEM	165.071	3.489	PIRNOV	158.898	3.433
PIRQIS	172.363	3.205	PIRRAL	157.173	3.247	PIRNUB	168.427	2.939	PIRNUB	161.345	2.788	PIRQAM	154.398	3.377
PIRSEO	166.276	2.812	PIRSER	151.612	2.905	PIRSEO	165.584	3.485	PIRSEO	170.559	3.121	PIRSEO	150.743	2.812
PIRTIW	159.594	2.833	PIRVIZ	176.782	3.18	PIRSUH	176.009	3.412	PIRSUH	167.34	3.312	PIRTIW	150.303	2.873
PITJAE	168.374	2.628	PITJAE	153.984	2.968	PITHUW	158.565	3.164	PITHUW	155.038	3.225	PITHUW	162.053	2.838
PITJAE	163.462	2.827	PITLAF	152.456	2.756	PITJAE	177.475	3.194	PITJAE	157.823	2.628	PITJAE	171.389	3.416
PITLIO	158.31	3.466	PITLIO	152.29	3.345	PITLAF	168.975	3.237	PITLAF	152.01	3.411	PITLAF	173.436	3.168
PITXOH	157.588	3.445	PITXOH	153.197	3.054	PITXOH	172.48	3.419	PITXOH	159.716	3.233	PITXOH	154.066	3.146
PIVJEN	165.883	3.486	PIVJEN	173.253	2.894	PITYUN04	158.456	3.123	PITYUN04	162.978	3.354	PIVHUB	159.33	3.274
PIVJUB	151.513	2.949	PIVJUB	151.648	3.432	PIVJUB	166.239	3.019	PIVJUB	151.202	3.32	PIVJUB	159.348	3.466
PIVNUF	171.362	3.072	PIVSET	154.747	3.404	PIVNIR	152.703	3.303	PIVNUF	167.154	3.36	PIVNUF	150.173	2.912
PIVSIW	173.595	3.121	PIVSIW	158.862	3.268	PIVSET	150.954	3.157	PIVSIW	163.898	3.064	PIVSIW	150.082	3.31
PIVSIW	163.958	3.165	PIVSIW	159.261	2.907	PIVSIW	169.021	2.972	PIVSIW	151.204	3.072	PIVSIW	162.06	3.332
PIWCIJ	166.658	3.282	PIWCIJ	169.668	3.061	PIVSIW	160.978	3.095	PIVZAZ	150.503	3.146	PIVZAZ	151.323	2.929
PIWFOS	162.914	3.271	PIWFOS	169.13	3.006	PIWCOP	167.112	3.318	PIWCOP	170.326	3.064	PIWFOS	154.485	3.455
PIWFUY	155.996	3.404	PIWFUY	154.638	2.917	PIWFUY	161.301	3.403	PIWFUY	158.328	3.369	PIWFUY	162.364	3.235
PIWGAF	157.738	3.408	PIWGAF	156.078	2.956	PIWFUY	161.908	3.216	PIWFUY	160.214	3.102	PIWFUY	167.402	2.968
PIWRIA	177.632	3.414	PIWVIC	162.339	3.239	PIWQOF	167.544	2.966	PIWQOF	176.919	3.442	PIWRIA	177.723	3.429
PIXFIN	162.682	2.992	PIXLIT	172.032	3.221	PIWXAV	152.842	2.77	PIXDEF	163.012	2.726	PIXDIK	153.304	3.253
PIXXIH	161.767	2.827	PIXXIH	168.893	2.827	PIXQUM	157.842	3.134	PIXQUM	154.238	3.088	PIXVEY	167.704	3.44
PIYHOU	153.1	3.399	PIYHOU	169.995	2.951	PIXXON	157.863	2.834	PIYHOU	154.64	2.952	PIYHOU	154.277	2.787

PIYHOU	156.543	3.082	PIYHOU	172.781	3.11	PIYHOU	174.25	2.964	PIYHOU	154.35	2.938	PIYHOU	174.531	3.063
PIYKEO	155.186	3.495	PIYRAT	150.394	3.191	PIYHOV	161.609	2.969	PIYHUD	162.183	3.401	PIYJUG	173.616	3.17
PIYVOM	158.82	3.452	PIYVOM	152.002	3.239	PIYRAT	167.825	2.574	PIYREX	150.968	3.162	PIYRIB	155.749	3.44
PIZGEN	156.088	3.107	PIZGEN	155.596	2.697	PIYYAA	153.214	2.868	PIYYAA	169.881	2.868	PIZGEN	161.529	2.753
PIZGUC	150.144	3.376	PIZNUL	152.535	3.118	PIZGEN	154.77	3.404	PIZGEN	161.348	3.195	PIZGEN	158.501	3.099
POBMUP	172.897	2.629	POBMUP	154.393	2.823	PIZSUM	154.667	3.364	POBLEX	152.208	3.199	POBLEX	165.725	3.215
POBNUP	159.247	3.178	POBNUP	163.756	2.903	POBNUP	160.156	3.2	POBNUP	167.806	3.258	POBNUP	151.355	3.259
POBNUP	157.667	3.047	POBNUP	157.868	2.844	POBNUP	153.957	3.155	POBNUP	156.031	3.032	POBNUP	151.339	3.237
POBNUP	159.792	3.415	POBNUP	174.942	3.483	POBNUP	161.834	3.117	POBNUP	162.552	3.202	POBNUP	166.455	2.932
POBQAA	157.947	3.043	POBQAA	164.263	3.062	POBQAA	152.756	2.903	POBQAA	152.38	2.962	POBQAA	155.974	3.01
POBQAA	164.018	3.453	POBQAA	172.554	3.146	POBQAA	167.357	3.388	POBQAA	169.522	3.265	POBQAA	172.151	3.389
POBQAA	167.758	3.418	POBQAA	170.112	3.493	POBQAA	169.858	3.164	POBQAA	173.348	3.203	POBQAA	176.73	3.312
POBRIJ	159.801	3.095	POBSUV	154.36	2.983	POBQII	156.143	3.395	POBRIJ	153.823	3.196	POBRIJ	169.504	3.341
POCPAC	166.182	2.882	POCTUW	157.333	3.154	POCFOF	165.75	3.421	POCNUU	150.981	3.001	POCNUU	159.6	3.001
PODKAU	160.039	3.284	PODKAU	150.622	3.064	POCVAI	155.815	3.274	POCXAK	169.849	2.738	PODGAR	161.701	3.057
PODNAX	172.153	3.084	PODNAX	160.398	2.646	PODLOJ	157.903	3.163	PODLOJ	150.795	2.69	PODNAX	162.844	3.315
POFCIZ	177.995	3.021	POFGAV	163.948	3.172	PODPUV	152.263	3.024	PODPUV	164.597	3.105	PODPUV	151.921	3.148
POFSIM	152.693	3.22	POFSIM	150.818	3.125	POFSIM	155.653	3.37	POFSIM	157.251	3.051	POFSIM	170.949	3.311
POFSIM	150.755	3.038	POGMUV	171.771	3.163	POFSIM	162.991	3.311	POFSIM	163.757	2.935	POFSIM	166.04	2.884
POHDUP	154.135	3.185	POHRAI	166.838	2.824	POHBIZ	150.033	3.193	POHCOF	165.877	2.848	POHDOJ	151.163	3.494
POHYOB	161.401	3.33	POJBIB	152.549	2.942	POHRAI	162.229	3.057	POHWES	150.679	3.249	POHYOB	159.666	3.259
POJCIE	162.326	3.321	POJJIL	153.201	3.018	POJBIB	158.8	3.204	POJCIE	164.347	2.886	POJCIE	152.715	2.994
POJJIL	156.425	2.781	POJJIL	156.64	3.205	POJJIL	150.858	3.051	POJJIL	154.557	2.944	POJJIL	167.191	2.893
POJJIL	160.335	3.001	POJJIL	156.608	2.993	POJJIL	153.451	3.344	POJJIL	155.196	3.154	POJJIL	157.523	3.362
POJJIL	169.797	2.864	POJJIL	156.436	3.389	POJJIL	168.79	3.447	POJJIL	171.009	3.388	POJJIL	159.162	3.344
POJLIN	154.159	3.27	POJLIN	154.014	3.412	POJJIL	152.745	3.181	POJLAE	150.617	3.105	POJLAE	150.684	2.8
POJXOE	164.758	3.277	POJXOE	151.399	3.004	POJWIX	169.501	3.122	POJWIX	150.287	2.983	POJWIX	171.154	2.983
POJYEV	151.246	2.956	POKCAX	166.363	3.382	POJYAQ	167.14	3.048	POJYAQ	175.94	3.029	POJYEV	156.493	3.29
POKDAV	159.704	3.226	POKDIG01	167.205	3.381	POKCIF	163.125	3.198	POKDAV	153.044	2.816	POKDAV	166.814	3.205
POKGOO(	156.528	2.912	POKGOO(	154.362	2.959	POKGII01	168.111	2.727	POKGOO(	154.515	3.079	POKGOO(	154.556	2.896
POKGOO(	153.534	2.827	POKGUU	160.336	3.017	POKGOO(	150.561	2.998	POKGOO(	154.68	2.854	POKGOO(	155.199	2.998
POKGUU	161.387	2.827	POKGUU	170.253	3.3	POKGUU	162.339	3.342	POKGUU	171.908	2.654	POKGUU	176.974	3.331
POKPOY	167.363	3.245	POKPOY	168.776	3.115	POKKUZ	165.694	3.481	POKMEK	158.946	3.016	POKPEO	151.453	3.27
POKPUE	167.474	2.814	POKPUE	165.332	2.814	POKPUE	152.998	2.785	POKPUE	160.418	3.388	POKPUE	155.596	2.888
POKRAJ	150.575	3.113	POKRIR	161.994	2.745	POKPUE	155.609	3.037	POKQIQ	155.212	3.311	POKQIQ	158.834	3.3
POKYEW	153.589	2.834	POKYEW	154.28	3.066	POKRIR	155.349	3.128	POKWUI	155.51	3.1	POKYEW	160.98	2.912
POKYEW	158.204	2.931	POKYEW	163.71	3.392	POKYEW	169.562	3.392	POKYEW	153.863	3.011	POKYEW	155.063	2.885
POLPEN	155.06	3.418	POMBEC	170.194	3.112	POLCEB	163.95	3.262	POLCEB	164.69	3.262	POLHUU	155.086	3.194
POMLEI	153.276	3.22	POMLEI	159.393	2.938	POMBUS	167.646	3.063	POMBUS	162.94	3.486	POMLEI	158.958	3.03
POMQOZ	165.349	3.087	POMWIB	166.4	3.102	POMLEI	158.315	3.486	POMLUY	166.222	3.001	POMLUY	160.386	3.327
POMWIB	152.078	3.087	POMWIB	153.216	3.152	POMWIB	169.349	3.172	POMWIB	151.6	3.415	POMWIB	157.774	3.272
POMXEV	171.529	3.415	POMXEV	171.176	2.799	POMWIB	153.614	3.194	POMWIB	164.251	3.264	POMXEV	160.7	3.114
PONLUA	174.551	3.107	PONLUA	155.041	3.135	PONJEJ	170.967	2.783	PONLIO	155.068	3.33	PONLOU	166.496	2.809
PONSOD	158.429	3.341	PONTUJ	157.452	3.092	PONMAH	159.902	3.201	PONMAH	179.029	3.491	PONMUE	158.755	3.468
POPNIV	159.843	3.36	POPSIZ	174.67	2.79	PONTUJ	171.719	3.092	PONWIC	153.196	3.351	POPNIV	170.98	3.093
POPWIC	163.57	3.265	POQBIK	164.791	3.08	POPSIZ	166.87	3.415	POPSIZ	166.75	2.875	POPSIZ	152.016	2.823
POQBIK	165.717	2.959	POQBOQ	164.927	2.824	POQBIK	158.012	3.006	POQBIK	175.483	3.381	POQBIK	176.935	3.231
POQBOQ	163.132	3.181	POQBOQ	161.556	3.16	POQBOQ	153.816	3.388	POQBOQ	151.443	3.161	POQBOQ	164.057	3.388
POQGAG	155.405	3.132	POQGAG	162.771	3.366	POQBOQ	168.831	2.978	POQBUT	151.669	3.477	POQGAG	155.948	3.327
POQGAG	168.72	2.713	POQGAG	165.842	3.464	POQGAG	174.587	2.955	POQGAG	159.415	3.311	POQGAG	172.808	3.458
POQTUK	163.1	2.908	POQTUK	161.855	3.108	POQGEI	150.901	3.164	POQNAK	161.847	3.412	POQRAQ	150.271	3.167
PORBOO	159.623	2.975	PORBOO	160.906	2.69	POQTUK	173.015	3.108	POQYAY	163.757	3.23	POQZOL	153.811	3.374
PORBOO	154.815	2.69	PORBOO	156.291	3.104	PORBOO	150.562	3.104	PORBOO	154.047	3.262	PORBOO	159.389	3.386
PORBOO	162.298	2.727	PORBOO	154.458	3.305	PORBOO	152.104	2.714	PORBOO	150.679	2.828	PORBOO	170.727	3.118
PORDIL	162.62	2.75	PORDIL	169.225	3.144	PORBOO	164.969	3.386	PORCIK	177.717	3.203	PORDIL	154.886	2.951
PORJUD0	151.132	3.377	PORKAK	154.875	3.468	PORDIL	167.777	2.951	PORJUD	154.714	3.461	PORJUD	164.067	2.857
PORKUF	168.577	2.852	PORNER	161.462	3.075	PORKAK	168.04	2.835	PORKEO	155.911	3.459	PORKEO	170.188	2.847
PORPIX	168.385	2.752	PORPIX	156.442	2.935	PORNIV	162.776	2.961	PORNUH	154.314	3.138	PORNUH	151.346	3.298
PORXON	173.589	3.359	POSFIN	159.544	3.178	PORPIX	152.738	3.156	PORSIC	168.773	3.283	PORTUO	165.655	3.162
POSHAG	164.996	3.408	POSHAG	155.471	2.973	POSGUZ	172.565	3.36	POSHAG	159.199	3.337	POSHAG	153.354	3.266
POSWAZ	171.415	3.494	POSXII	158.08	3.193	POSHAG	154.573	3.322	POSHIR	155.952	2.859	POSRAQ0	154.638	3.384
POTRIB	152.599	3.219	POTROH	156.572	3.217	POTHAJ	159.077	2.847	POTHAJ	170.192	3.308	POTJEQ	160.133	3.292
POVZAE	155.049	3.057	POWFAK	150.221	3.104	POVCEK	154.14	2.864	POVCEK	160.439	2.796	POVCEK	154.922	2.796
POWJAP	169.271	2.955	POWJET	172.124	3.283	POWFAK	160.741	2.906	POWHER	158.957	3.234	POWJAP	163.995	3.266
POWJIX	161.676	3.215	POWPEW	154.305	3.339	POWJET	158.35	3.273	POWJET0	171.58	3.288	POWJET0	158.342	3.275
POWTUQ	165.245	3.268	POWVAY	158.363	3.347	POWPEW	157.751	3.339	POWTUQ	158.416	3.348	POWTUQ	155.59	2.867
POXLUN	156.844	3.305	POXLUN	157.245	2.952	POWVAY	155.141	2.849	POWVAY	164.638	3.271	POXHES	151.711	3.224
POYKIB	150.429	3.469	POYKIB	151.017	3.458	POXRUS	150.369	2.782	POXRUS	152.192	3.067	POYKIB	168.874	3.093
POYLAR	170.446	3.328	POYLAR	177.882	3.197	POYLAR	174.982	3.197	POYLAR	152.937	3.315	POYLAR	152.249	3.335
POYPOM	172.252	3.256	POYYUB	154.554	3.448	POYLIC	159.19	2.84	POYLIZ	155.097	3.348	POYPOM	159.581	3.061
POZFAO	156.437	2.752	POZFAO	153.789	3.416	POYZEM	175.304	3.494	POYZEM	151.206	2.995	POZFAO	156.842	3.323
POZLAR	159.082	2.882	POZLAR	166.213	2.882	POZFAO	165.907	2.78	POZFAO	161.286	2.642	POZLAR	160.243	2.896
POZMOG	170.308	3.406	POZMUM	170.951	3.248	POZLAR	159.517	3.354	POZLAR	156.171	3.155	POZMOG	152.857	3.04
POZXAG	162.41	3.49	POZYIO	162.128	3.246	POZNAT	159.379	3.342	POZPAY	156.866	3.081	POZPAZ	166.34	2.909
PUCBEU	172.708	3.199	PUCBEV	156.054	2.975	PRSFBC	167.083	3.281	PUBHIE	169.183	3.011	PUBWIV	157.31	2.869
PUCBEV	154.489	2.879	PUCBEV	152.325	3.272	PUCBEV	152.376	2.877	PUCBEV	163.798	2.92	PUCBEV	152.781	3.216
PUCBIA	175.205	3.06	PUCBUL	154.793	3.188	PUCBIA	174.042	3.177	PUCBIA	170.398	3.06	PUCBIA	172.12	2.807
PUCJOQ	151.702	3.303	PUCJOQ	162.783	3.078	PUCBUL	176.98	3.036	PUCBUL	151.251	2.93	PUCJOQ	164.553	2.808
PUCJOQ	165.662	3.259	PUCJOQ	161.553	3.448	PUCJOQ	168.224	3.159	PUCJOQ	156.562	3.076	PUCJOQ	167.161	2.747

PUCNEK	171.132	3.212	PUCNEK	153.693	3.357	PUCJOQ	151.178	2.852	PUCKEG	156.236	3.376	PUCNEK	158.979	3.237
PUCPAI	177.362	3.419	PUCPAI	153.755	3.391	PUCNEK	152.113	2.874	PUCNEK	165.684	2.798	PUCPAI	159.546	3.404
PUCROX	173.568	3.043	PUCROX	155.214	2.858	PUCPAI	169.704	3.242	PUCPAI	159.862	3.148	PUCROX	153.575	3.032
PUCYIW	153.541	3.37	PUDCEZ	170.789	3.299	PUCRUD	158.053	3.31	PUCRUD	158.813	3.08	PUCTOW	163.933	3.01
PUDSEQ	175.901	3.261	PUDYIX	177.468	3.121	PUDCEZ	172.662	3.078	PUDDUP	152.495	2.842	PUDROX	151.403	3.161
PUFSOA	155.547	3.178	PUFSOA	151.215	3.078	PUFFUS	167.626	3.222	PUFFUS	173.827	2.988	PUFFUS	156.2	3.12
PUFTAP	158.639	3.308	PUFWAR	150.26	3.211	PUFSOA	159.131	3.078	PUFSOA	167.931	3.236	PUFTAP	155.814	3.338
PUGPIR	161.405	2.884	PUGXEV	150.117	3.356	PUFWAR	150.741	3.202	PUGDII	164.879	3.283	PUGDII02	163.566	3.285
PUHLAF	151.229	3.435	PUHLAF	176.091	3.45	PUGXEV	155.065	3.433	PUGYUP	160.822	3.343	PUGYUP	152.95	3.386
PUHLAF	156.94	3.049	PUHLAF	174.628	2.908	PUHLAF	173.799	3.062	PUHLAF	177.226	3.054	PUHLAF	153.086	3.075
PUHLAF	162.887	3.29	PUHNUC	159.512	3.418	PUHLAF	154.323	3.191	PUHLAF	153.202	3.149	PUHLAF	167.315	2.826
PUHNUC	154.74	3.159	PUHNUC	159.084	3.159	PUHNUC	155.004	3.41	PUHNUC	170.464	3.265	PUHNUC	154.806	3.418
PUHQOB	159.238	3.073	PUHZIC	154.001	3.291	PUHQOB	155.394	3.124	PUHQOB	172.824	2.834	PUHQOB	151.361	3.436
PUJLOW	150.133	3.464	PUJNOY	156.295	2.959	PUHZIC	165.733	2.899	PUJFEG	155.654	3.224	PUJHIP	150.724	3.225
PUKJOV	157.899	3.084	PUKJOV	158.993	3.357	PUKBEF	152.819	3.056	PUKFEJ	160.393	3.168	PUKFEJ	153.71	3.088
PUKKUC	171.382	3.012	PUKMUE	166.135	3.237	PUKJOV	171.223	2.8	PUKJOV	152.783	3.02	PUKKUC	169.315	3.012
PUKSAR	168.645	3.157	PUKSAR	176.038	2.71	PUKPIW	168.544	3.415	PUKPIW	165.896	3.185	PUKPIW	164.329	3.2
PUKSAR	154.653	3.369	PUKSAR	164.779	3.242	PUKSAR	164.49	2.783	PUKSAR	159.131	2.783	PUKSAR	157.129	3.252
PUKSAR	160.322	3.011	PUKSAR	161.599	3.348	PUKSAR	160.092	3.196	PUKSAR	161.954	3.379	PUKSAR	155.916	3.213
PUKSAR	171.308	3.204	PULTOH	162.385	3.351	PUKSAR	151.871	3.071	PUKSAR	155.081	2.872	PUKSAR	156.756	3.149
PULTUN	173.785	2.951	PULTUN	159.086	3.334	PULTOH	157.323	2.932	PULTOH	154.815	3.161	PULTOH	156.246	2.931
PULTUN	161.489	3.068	PULTUN	152.57	3.275	PULTUN	155.9	2.784	PULTUN	151.666	3.237	PULTUN	158.993	2.871
PULTUN	162.671	2.912	PULVAV	154.183	2.805	PULTUN	157.242	2.966	PULTUN	154.535	3.326	PULTUN	158.319	3.097
PULVAV	153.858	2.877	PULVAV	165.736	2.944	PULVAV	173.433	3.498	PULVAV	162.636	2.99	PULVAV	165.545	3.233
PULVEZ	155.667	3.138	PULVEZ	154.581	3.382	PULVAV	159.04	3.305	PULVEZ	163.201	3.177	PULVEZ	155.906	2.929
PULVID	160.159	3.005	PULVID	151.646	3.095	PULVEZ	161.442	2.887	PULVID	153.594	2.804	PULVID	175.754	3.478
PULVID	159.038	3.284	PULVOJ	154.242	2.953	PULVID	166.927	3.187	PULVID	151.932	2.871	PULVID	166.131	2.924
PULVUP	153.703	2.806	PULVUP	175.124	3.48	PULVOJ	163.654	3.472	PULVOJ	154.706	3.167	PULVOJ	156.471	2.947
PULVUP	152.336	2.879	PULVUP	166.236	2.932	PULVUP	160.873	2.998	PULVUP	151.051	3.098	PULVUP	166.624	3.202
PULWAW	159.407	2.993	PULWAW	152.507	3.076	PULVUP	159.056	3.288	PULWAW	152.996	2.798	PULWAW	176.42	3.459
PULWAW	159.02	3.268	PUMGEJ	158.175	3.115	PULWAW	167.061	3.167	PULWAW	151.099	2.863	PULWAW	166.828	2.908
PUMJIU	155.916	2.778	PUMJOA	158.095	3.085	PUMGEJ	176.751	3.32	PUMJEQ	166.176	2.997	PUMJIU	157.691	3.423
PUMKUD	156.613	3.211	PUMLER	157.923	2.847	PUMJOA	173.39	3.15	PUMJOA	152.522	3.326	PUMKUD	151.039	3.121
PUMPAR	166.79	3.349	PUMPAR	172.005	3.349	PUMMIU	156.746	3.307	PUMMOA	165.838	3.168	PUMMOA	150.968	3.107
PUMXUR	168.41	2.607	PUMYUT	151.715	3.261	PUMPEW	170.482	2.939	PUMPIA	168.544	3.156	PUMVEC	162.022	3.428
PUNBEI	164.068	3.201	PUNCIK	154.569	3.166	PUMYUT	165.466	3.13	PUMYUT	153.829	3.173	PUNBAE	153.742	2.846
PUNMIU	169.86	3.224	PUNMIU	154.002	3.216	PUNCIK	152.577	2.793	PUNLUI	170.248	3.461	PUNMIU	164.352	2.811
PUNQUM	168.08	2.998	PUNQUM	164.583	3.051	PUNQIA	174.187	3.063	PUNQUM	171.083	3.153	PUNQUM	160.678	3.306
PUNREX	170.332	2.686	PUNREX	158.154	2.697	PUNQUM	167.892	2.827	PUNQUM	164.266	3.205	PUNREX	159.726	2.673
PUNRIB	157.604	2.765	PUNRIB	154.691	2.936	PUNREX	150.991	3.302	PUNRIB	175.805	2.921	PUNRIB	161.956	2.986
PUNRIB	164.401	2.765	PUNROH	156.701	2.748	PUNRIB	156.081	2.757	PUNRIB	159.816	2.911	PUNRIB	157.181	3.289
PUNSEY	170.192	2.748	PUNSEY	151.983	2.656	PUNROH	157.41	3.163	PUNROH	151.222	2.843	PUNROH	153.167	3.173
PUNWON	157.679	3.383	PUNXUR	175.142	3.236	PUNSEY	160.95	3.176	PUNSEY	171.531	2.769	PUNWON	158.173	3.371
PUNYAB	153.592	2.847	PUNYAB	156.463	2.922	PUNXUU	167.324	2.87	PUNXUU	158.473	3.29	PUNYAB	150.034	2.778
PUNYAB0	154.523	2.961	PUNYAB0	152.137	2.823	PUNYAB	151.594	2.847	PUNYAB	154.51	3.048	PUNYAB0	150.944	2.823
PUPGOY	156.707	3.254	PUPMIA	162.041	3.307	PUNYAB0	155.921	2.863	PUNYEF	157.897	3.216	PUNYEF	162.191	2.916
PUPPIB	156.841	2.775	PUPPIB	169.584	3.332	PUPMIA	164.171	2.826	PUPMIA	171.422	3.102	PUPPIB	155.562	2.91
PUPPIB	154.492	3.111	PUPPIB	154.297	2.884	PUPPIB	166.411	3.499	PUPPIB	164.716	3.013	PUPPIB	156.831	2.893
PUPSIE	165.043	3.052	PUPSIE	167.217	3.127	PUPPUN	151.496	3.476	PUPSIE	168.863	3.412	PUPSIE	170.034	3.245
PUQBEK	166.093	3.335	PUQBEK	151.76	3.335	PUPXOP	160.899	3.259	PUPXOP	166.534	2.779	PUQBAG	151.633	3.327
PUQCIR	154	3.006	PUQCIR	160.534	2.969	PUQBEK	151.441	3.271	PUQCAJ	170.637	3.493	PUQCEN	171.505	3.298
PUQFIS	172.979	3.31	PUQKAP	161.738	3.022	PUQDEO	155.924	3.264	PUQFIS	167.098	2.969	PUQFIS	171.636	3.169
PUQMOH	169.289	3.045	PUQNEU	168.669	3.309	PUQKAP	153.79	3.265	PUQMEX	166.091	3.129	PUQMIB	169.043	3.32
PUQNIA	157.288	3.367	PUQNIA	163.078	3.346	PUQNIA	159.87	3.421	PUQNIA	172.11	3.173	PUQNIA	169.423	3.479
PUQNUM	153.069	3.428	PUQSIF	157.945	3.257	PUQNIA	163.715	3.191	PUQNUM	160.305	3.221	PUQNUM	152.191	3.037
PUQVOO	152.472	3.49	PUQVOO	153.612	3.26	PUQSIF	158.738	3.257	PUQVOO	152.912	2.724	PUQVOO	152.58	3.428
PUQVOO	162.886	2.91	PUQVUU	152.998	3.356	PUQVOO	161.79	3.184	PUQVOO	156.915	3.245	PUQVOO	166.693	2.859
PURKOD	172.603	3.171	PURKOD	154.761	2.902	PURBEL	152.438	3.029	PURKOD	151.18	3.156	PURKOD	173.463	2.961
PURMEW	155.158	3.216	PURMEW	154.808	2.737	PURKOD	160.72	3.423	PURMEW	159.188	3.484	PURMEW	151.581	3.225
PURNAV	163.966	3.261	PURNAV	166.17	2.946	PURNAV	165.023	2.806	PURNAV	153.199	2.806	PURNAV	157.44	3.273
PURNEZ	173.487	3.433	PURNEZ	151.251	2.824	PURNAV	168.403	2.894	PURNAV	163.568	2.968	PURNEZ	161.695	2.617
PURNUO	151.701	3.456	PURNUO	159.694	3.06	PURNEZ	157.234	3.496	PURNEZ	159.498	3.079	PURNEZ	156.321	3.015
PURSAW	150.094	3.166	PURSAW	162.494	2.884	PURPEB	169.357	2.747	PURPEB	157.486	3.044	PURSAW	174.36	2.849
PURSAW	160.48	3.188	PURTEE	164.301	3.073	PURSAW	167.051	3.204	PURSAW	169.785	2.995	PURSAW	150.663	3.287
PURWEH	151.438	3.347	PURYAD	152.951	3.227	PURTEE	168.618	2.918	PURWAD	150.914	3.332	PURWAD	154.809	3.267
PURYIL	157.67	3.072	PURYIL	161.942	3.291	PURYAD	150.046	2.928	PURYEH	158.454	3.05	PURYEH	151.429	3.068
PURZIM	158.994	3.305	PUSCIT	153.014	2.976	PURYOR	179.064	3.451	PURZEI	153.428	2.886	PURZEI	176.28	2.886
PUSJUJ	174.7	3.234	PUSPAV	160.905	3.381	PUSGUF	152.41	2.858	PUSJAO	160.991	3.192	PUSJAO	150.926	3.392
PUSPAV	172.328	3.358	PUSPAV	153.774	3.375	PUSPAV	171.546	3.261	PUSPAV	169.854	3.288	PUSPAV	167.472	3.249
PUSROL	153.53	3.383	PUSTIH	172.089	2.867	PUSPAV	164.421	3.356	PUSPAV	175.759	3.361	PUSQOK	161.924	2.73
PUSYOS	160.951	3.116	PUSYOS	163.612	3.369	PUSTIH	157.933	3.076	PUSTIH	165.359	2.962	PUSVOR	154.358	3.321
PUTHES0	162.476	2.947	PUTHIV	150.693	3.368	PUTDAK	159.683	3.013	PUTHAN	160.463	3.142	PUTHES	163.068	2.977
PUTHIW	179.574	3.454	PUTHIW	154.876	3.095	PUTHIW	157.784	3.118	PUTHIW	170.293	3.184	PUTHIW	161.699	2.888
PUTHIW	160.807	3.097	PUTHUI	154.084	2.986	PUTHIW	173.258	3.279	PUTHIW	151.935	3.016	PUTHIW	167.806	3.353
PUTJAQ	154.467	2.907	PUTJAQ	164.397	3.082	PUTHUI	159.985	2.871	PUTHUI	171.397	3.42	PUTHUI	152.975	3.233
PUTJUM	166.213	2.865	PUTJUN	153.285	3.497	PUTJAQ	157.193	3.298	PUTJAQ	164.823	3.053	PUTJUM	153.759	3.298
PUTQAW	167.348	3.372	PUTQAW	168.341	3.151	PUTJUN	160.259	2.801	PUTJUN	161.063	3.268	PUTLEZ	165.213	2.784
PUTQEA	163.345	2.761	PUTQEA	153.386	3.294	PUTQEA	167.897	2.761	PUTQEA	154.241	3.306	PUTQEA	167.358	2.763
PUTQIF	159.474	3.174	PUTQIF	152.475	2.978	PUTQEB	152.043	3.039	PUTQEB	157.418	3.033	PUTQEB	155.033	3.295

PUTQOL	152.273	2.985	PUTREF	159.64	2.956	PUTQIH	161.471	3.004	PUTQIH	150.452	2.789	PUTQOL	159.839	3.165
PUVJIA	153.265	2.584	PUVVAE	162.123	3.119	PUTREF	176.92	3.383	PUTTEH	154.575	2.853	PUVFAR	163.756	3.212
PUVVAE	157.294	2.974	PUVVOS	158.779	2.857	PUVVAE	156.295	2.814	PUVVAE	160.404	2.809	PUVVAE	160.302	3.38
PUVYUE	171.358	2.763	PUVYUE	159.875	2.9	PUVVOS	155.196	3.248	PUVVOS	157.26	3.471	PUVVOS	168.924	3.042
PUVYUE	158.784	3.201	PUWZOA	151.143	3.301	PUVYUE	154.41	3.088	PUVYUE	153.149	2.97	PUVYUE	162.103	3.201
PUWZOA	168.466	3.119	PUWZOA	156.236	3.09	PUWZOA	151.088	2.958	PUWZOA	158.796	3.291	PUWZOA	156.818	2.935
PUXNOP	153.942	3.19	PUXNOP	164.135	2.771	PUWZOA	177.331	3.301	PUXBUF	161.98	3.076	PUXDUH	156.034	3.355
PUXNOP	162.81	3.322	PUXNOP	158.57	3.317	PUXNOP	170.887	3.477	PUXNOP	153.982	2.904	PUXNOP	161.13	2.762
PUXYIT	150.597	2.826	PUXYIT	169.66	2.68	PUXXIR	170.972	3.283	PUXXIR	157.416	2.794	PUXYIT	162.403	3.294
PUXYOZ	150.668	2.699	PUXYUE	151.214	2.855	PUXYIT	159.277	2.864	PUXYOZ	156.531	3.105	PUXYOZ	150.011	2.679
PUXZAM	154.235	3.177	PUXZEQ	169.398	2.721	PUXZAL	162.855	2.815	PUXZAL	163.149	3.092	PUXZAM	166.392	3.273
PUXZIT	154.925	2.708	PUXZOZ	163.662	3.171	PUXZEQ	160.754	2.95	PUXZEQ	169.35	2.708	PUXZEQ	165.633	2.897
PUXZOZ	155.37	2.996	PUYCOE	162.449	3.163	PUXZOZ	158.881	2.948	PUXZOZ	163.164	3.264	PUXZOZ	151.398	2.747
PUYDEW	171.821	2.906	PUYDEW	152.891	3.046	PUYCOE	164.468	3.436	PUYDAR	157.433	3.428	PUYDEW	167.529	3.433
PUYDUM	158.305	2.832	PUYDUM	154.779	2.665	PUYDUM	163.896	3.176	PUYDUM	173.081	3.077	PUYDUM	165.838	2.861
PUYFOG	164.699	2.62	PUYHID	174.489	3.065	PUYDUM	151.849	2.838	PUYFOG	152.168	3.337	PUYFOG	162.469	3.395
PUZFUL	159.097	3.338	PUZFUL	153.955	3.262	PUYHID	158.772	3.185	PUYHID	167.002	3.185	PUYHID	162.637	2.91
PUZHEA	164.244	2.537	PUZJIE	159.634	3.191	PUZGAS	157.962	3.267	PUZGAS	157.979	3.18	PUZGEW	167.414	3.107
PUZVUB	160.512	3.009	PUZVUB	154.837	3.345	PUZJIE	166.592	3.218	PUZMUW	173.91	3.448	PUZMUW	167.194	3.196
PUZVUE	155.724	3.391	QABCEE	155.102	3.478	PUZVUE	151.994	3.332	PUZVUE	179.165	2.754	PUZVUE	160.239	2.827
QABGUY	160.349	3.301	QABNOY	164.288	3.06	QABCII	168.464	3.011	QABCII	151.314	3.367	QABGIL	167.469	3.389
QABXUO	152.069	3.266	QABZAW	164.641	3.147	QABTOF	155.799	3.042	QABXUO	155.106	3.307	QABXUO	154.571	3.236
QABZAW	165.473	2.918	QABZAW	157.687	3.034	QABZAW	164.086	3.226	QABZAW	166.994	3.424	QABZAW	152.62	3.302
QABZAW	154.641	3.108	QABZAW	150.388	3.302	QABZAW	173.357	3.147	QABZAW	168.72	2.836	QABZAW	152.751	2.983
QABZEA	159.315	2.924	QABZEA	154.793	2.711	QABZEA	162.634	2.99	QABZEA	151.819	2.734	QABZEA	158.392	3.059
QABZEA	157.947	2.955	QABZEA	161.969	2.852	QABZEA	153.338	3.173	QABZEA	152.052	2.907	QABZEA	157.96	3.454
QABZEA	171.203	2.571	QABZEA	157.694	3.338	QABZEA	177.908	3.394	QABZEA	165.1	3.423	QABZEA	159.965	2.948
QABZEA	165.711	3.171	QABZEA	153.198	3.217	QABZEA	157.319	2.859	QABZEA	162.329	2.779	QABZEA	150.229	3.387
QABZEA	155.286	3.338	QABZEA	155.037	3.033	QABZEA	152.223	3.051	QABZEA	153.548	2.9	QABZEA	167.322	3.419
QACBAZ	170.094	3.364	QACBAZ	169.075	3.405	QABZEA	150.109	3.388	QACBAZ	170.061	3.315	QACBAZ	170.016	3.325
QACYIC	152.124	2.928	QACYIC	152.341	2.787	QACBED	153.488	3.298	QACBUT	151.09	3.479	QACNAK0	162.927	3.148
QACYOI	170.846	3.042	QADBON	176.083	2.813	QACYIC	151.166	3.39	QACYOI	152.779	3.17	QACYOI	155.189	3.383
QADBON	155.347	3.079	QADBON	166.456	3.177	QADBON	171.551	3.068	QADBON	153.98	2.994	QADBON	178.088	3.16
QADBUT	153.625	3.3	QADBUT	162.188	3.035	QADBON	161.709	3.44	QADBUT	163.104	3.128	QADBUT	160.269	3.236
QADHEK	163.559	3.232	QAFKAL	154.618	3.494	QADBUT	152.832	3.485	QADFOT	174.711	3.273	QADFOT	168.567	3.414
QAFMEQ	151.495	2.997	QAFTUO	162.634	3.007	QAFKAL	156.276	2.969	QAFKAL	154.702	3.45	QAFKAL	168.132	2.946
QAFTUO	161.043	2.896	QAFVAW	170.174	2.915	QAFTUO	160.795	3.047	QAFTUO	153.088	3.062	QAFTUO	163.077	3.304
QAFVAW	156.121	3.404	QAFVAW	150.119	3.115	QAFVAW	174.101	2.928	QAFVAW	155.599	3.428	QAFVAW	150.595	2.969
QAFVAW	172.098	3.334	QAFVAW	154.945	2.794	QAFVAW	156.922	3.403	QAFVAW	165.364	3.262	QAFVAW	156.971	2.802
QAFVAW	150.09	3.202	QAFVAW	156.263	3.102	QAFVAW	173.785	3.404	QAFVAW	167.687	3.414	QAFVAW	152.985	3.081
QAFVAW	165.181	3.129	QAFVAW	161.243	2.745	QAFVAW	151.683	3.489	QAFVAW	170.182	3.165	QAFVAW	160.908	2.976
QAFXEC	155.454	3.037	QAFXIG	157.641	2.994	QAFVAW	163.95	3.202	QAFXAY	160.401	2.83	QAFXAY	154.017	3.195
QAFXIG	164.835	3.289	QAFYAX	161.728	3.291	QAFXIG	160.098	3.243	QAFXIG	160.759	3.314	QAFXIG	168.806	2.784
QAGBEI	158.248	3.235	QAGBEI	156.723	2.813	QAFYAX	172.857	3.282	QAFYAX	153.132	2.982	QAGBEI	165.451	3.36
QAGBEI	151.916	3.36	QAGCAE	169.265	3.152	QAGBEI	159.495	3.415	QAGBEI	159.203	3.151	QAGBEI	173.791	3.082
QAGREX	159.56	3.41	QAGTIE	155.565	3.257	QAGREX	166.418	3.363	QAGREX	154.219	3.493	QAGREX	168.677	3.486
QAHDAH	152.578	3.494	QAHDOT	157.377	3.022	QAGWUT	152.818	2.987	QAGWUT	153.574	3.214	QAHDAH	152.625	3.183
QAHLAP	165.921	3.379	QAHLAP	154.387	2.707	QAHFIQ	151.878	3.167	QAHFIQ	154.502	3.407	QAHKUI	172.203	2.979
QAHLAP	158.177	3.035	QAHLAP	157.523	3.05	QAHLAP	167.644	3.082	QAHLAP	156.856	3.468	QAHLAP	150.655	3.164
QAHSAT	155.734	3.275	QAHSAT	157.487	3.063	QAHLAP	159.298	3.366	QAHPAR	152.927	3.093	QAHPAT	153.996	2.937
QAHWIF	156.606	3.077	QAHZIJ	171.925	2.939	QAHSAT	164.207	2.808	QAHVAZ	152.692	3.367	QAHWIF	158.074	3.113
QAJYEG	174.646	2.933	QAJYIM	153.812	3.118	QAJLIZ	152.37	3.249	QAJSUR	167.677	3.325	QAJVEF	151.949	2.981
QAKCEL	150.009	3.127	QAKCEL	172.911	3.245	QAJYIM	159.092	3.159	QAKCEL	173.616	3.337	QAKCEL	164.894	3.264
QAKCIP	166.868	3.369	QAKCIP	166.354	3.22	QAKCEL	168.044	3.255	QAKCEL	154.422	3	QAKCIP	172.902	3.317
QAKWEE	151.462	3.055	QALBEO	160.647	3.047	QAKCIP	171.511	3.25	QAKCIP	154.24	2.964	QAKCIP	169.176	3.242
QALBEO	179.696	3.106	QALBEO	165.693	3.147	QALBEO	159.749	3.207	QALBEO	163.292	3.5	QALBEO	159.234	2.718
QALGOA	167.48	2.74	QALGOA	164.938	2.74	QALCEP	151.844	2.908	QALGEQ	155.383	3.334	QALGEQ	165.737	3.334
QALSOM	168.074	3.161	QALSUS	162.587	3.161	QALQAZ	152.799	3.233	QALQAZ	155.794	3.024	QALSOM	178.615	3.161
QAMJUM	162.567	2.957	QAMJUM	157.588	3.391	QALWOS	167.852	3.143	QAMBEO	165.731	3.406	QAMBEO	161.273	2.839
QAMZIQ	162.061	3.166	QANCEQ	162.033	3.127	QAMVIM	157.26	3.434	QAMZEM	165.367	3.258	QAMZEM	157.044	3.316
QANREF	174.964	3.082	QANREF	156.811	2.895	QANNOM	167.234	2.968	QANPIG	155.748	3.233	QANQUT	156.632	3.375
QANZEK	160.152	2.883	QAPHAU	167.919	2.854	QANTIL	173.119	3.137	QANTIL	176.932	3.067	QANZEK	160.585	2.889
QAPSAF	162.604	3.019	QAPSAF	154.829	3.228	QAPHAU	165.613	3.034	QAPMEB	170.261	2.883	QAPNOO	170.757	2.942
QAPYOX	156.736	3.332	QAPYOX	156.494	3.218	QAPTOQ	154.346	3.356	QAPYIP	158.529	3.263	QAPYOX	167.5	3.344
QARDOE	150.853	3.005	QARFAS	161.181	3.029	QAQXEN	154.398	3.261	QAQXEN	155.679	3.498	QAQXEN	160.029	3.241
QARHAS	166.441	3.385	QARHIA	155.113	3.214	QARFIZ	156.156	3.097	QARHAS	153.214	3.489	QARHAS	154.881	3.17
QASFEV	157.019	3.05	QASLAB	153.44	3.018	QARNAC	168.356	2.773	QARNAC	175.135	3.491	QARNAC	177.311	2.847
QASTEK	173.938	3.487	QASTEK	154.038	3.321	QASLAB	164.891	3.481	QASNUV	155.892	3.094	QASTEK	154.579	3.222
QASWUF	172.051	3.218	QASWUF	151.033	2.913	QASVOZ	161.129	3.387	QASWUF	163.535	3.074	QASWUF	150.003	3.169
QASZOA	150.545	3.202	QASZOA	152.207	3.314	QASWUF	153.446	3.029	QASZOA	158.607	3.081	QASZOA	166.784	3.281
QATMUT	169.633	3.033	QATMUT	172.157	3.126	QATCAQ	166.094	2.904	QATFUP	163.308	2.706	QATMUT	168.479	2.953
QATPOQ	160.182	2.85	QATQEL	153.067	3.121	QATMUT	162.706	2.781	QATMUT	155.04	3.072	QATMUT	166.711	2.786
QATTAH	162.148	2.894	QATTAH	167.719	3.349	QATQEL	162.887	3.417	QATQEL	152.428	3.041	QATTAH	152.067	3.462
QAVBUO	173.258	3.389	QAVKIJ	156.026	2.784	QATWAL	167.813	2.929	QATYET	150.618	3.22	QATYIX	160.006	3.194
QAVKIJ	156.46	3.241	QAVKIJ	178.099	2.798	QAVKIJ	154.794	2.784	QAVKIJ	156.591	3.447	QAVKIJ	153.544	2.755
QAVKUV	156.868	3.005	QAVKUV	169.057	2.696	QAVKOP	167.792	3.175	QAVKOP	152.414	3.439	QAVKOP	152.719	2.963
QAVLAC	152.586	2.756	QAVLAC	156.963	2.766	QAVLAC	155.792	3.242	QAVLAC	179.15	2.79	QAVLAC	156.409	3.446
QAVLIK	155.516	3.35	QAVLIK	162.609	2.693	QAVLAC	155.276	2.766	QAVLEG	166.999	2.71	QAVLEG	156.126	3.023
QAVLOQ	168	3.185	QAVLUW	164.984	3.027	QAVLIK	156.019	2.978	QAVLOQ	153.745	2.969	QAVLOQ	153.093	3.429

QAVPAF	166.052	3.381	QAVPAF	153.164	2.713	QAVLUW	168.253	3.112	QAVLUW	166.115	3.293	QAVNUX	167.608	3.077
QAVPAF	150.763	3.331	QAWCUP	168.346	2.933	QAVPAF	151.36	3.365	QAVPAF	170.208	3.485	QAVPAF	165.782	2.923
QAWNOR	155.543	3.451	QAWPIN	151.408	3.483	QAWJUX	158.435	3.066	QAWJUX	164.315	2.916	QAWNOR	156.118	3.444
QAWPOT	154.036	2.895	QAWPOT	157.961	2.968	QAWPIN	174.516	2.775	QAWPOT	165.127	3.334	QAWPOT	155.65	2.793
QAWTUD	163.224	3.046	QAWTUD	161.018	3.429	QAWPOT	157.862	3.469	QAWPOT	168.709	3.151	QAWTUD	161.807	3.131
QAWYUM	156.635	3.226	QAXBID	152.008	3.324	QAWTUD	155.241	2.999	QAWWAP	171.675	3.208	QAWWAP	154.351	3.468
QAXJIM	171.502	3.063	QAXJIM	157.986	3.251	QAXBID	155.34	2.983	QAXCIE	164.305	3.12	QAXHUV	168.498	2.984
QAXRUG	156.15	3.227	QAXRUG	151.712	2.818	QAXJIM	153.524	3.285	QAXJIM	154.713	2.838	QAXNIQ	150.273	3.476
QAXWUH	151.311	3.212	QAXWUH	166.744	3.102	QAXRUG	160.125	3.271	QAXWUH	173.544	2.948	QAXWUH	155.318	3.197
QAXXIY	153.556	3.009	QAXXIY	150.87	2.957	QAXWUJ	157.883	3.037	QAXXAR	163.87	2.87	QAXXIY	153.61	3.344
QAYTEQ	154.927	3.308	QAYVIV	161.891	2.967	QAXXIY	150.327	2.839	QAXXIY	152.639	2.868	QAXXUJ	161.225	2.963
QAYVIV	154.68	3.443	QAYVIV	167.457	2.788	QAYVIV	153.34	3.482	QAYVIV	163.249	2.601	QAYVIV	151.74	3.005
QAYVIV	150.527	2.911	QAYXAR	157.853	2.943	QAYVIV	160.265	2.835	QAYVIV	169.156	3.371	QAYVIV	164.68	2.783
QAZKAG	154.531	2.89	QAZLIM	177.848	3.18	QAYZOI	152.549	3.135	QAYZOI	155.457	3.135	QAZHAE	162.298	3.057
QAZLOS	152.375	3.089	QAZLOS	162.4	3.244	QAZLIM	153.836	3.099	QAZLIM	170.634	2.899	QAZLOS	152.059	3.176
QEBBOR	169.957	3.196	QEBBOR	162.877	3.196	QAZLUY	152.694	3.389	QAZLUY	162.981	2.874	QAZLUY	154	3.255
QEBNES	172.672	3.301	QEBSOF	163.051	3.437	QEBJIU	150.387	3.108	QEBJIU	164.939	2.986	QEBJIU	154.595	3.273
QECYIJ	157.768	3.157	QECYIJ01	154.756	3.101	QEBSOF	155.171	3.415	QECCUW	164.669	3.066	QECGIQ	154.406	3.496
QEDCUX	167.235	3.26	QEDGIS	160.012	2.956	QECYIJ01	155.506	3.189	QEDCUA	158.624	3.165	QEDCUX	151.165	3.466
QEDSAT	150.051	3.017	QEDTEA	155.528	3.075	QEDHOY	156.892	2.966	QEDHOY	167.629	3.03	QEDPUM	160.047	2.955
QEFLIW	165.966	3.239	QEFLIW	151.953	2.799	QEFDIO	151.031	2.965	QEFLIW	168.55	2.889	QEFLIW	153.007	2.732
QEFNIY	169.938	3.392	QEFNIY	152.035	3.392	QEFLIW	153.302	2.889	QEFMEV	164.961	2.656	QEFNEY	157.081	3.458
QEFZUA	161.917	3.12	QEGBEL	153.586	3.421	QEFPUO	156.04	3.202	QEFQID	155.842	3.227	QEFVOP	167.541	3.344
QEGPAU	150.294	3.425	QEGPAU	170.514	2.821	QEGBEL	160.109	3.406	QEGKAO	153.317	3.125	QEGKAO	154.267	3.433
QEGRAW	152.151	2.746	QEGRAW	162.171	3.404	QEGPAU	157.99	3.432	QEGPEY	151.414	3.173	QEGPIC	154.786	3.014
QEGRAW	154.353	2.737	QEGRAW	154.113	3.404	QEGRAW	150.437	3.496	QEGRAW	151.827	3.238	QEGRAW	168.067	3.047
QEGRAW	155.916	2.835	QEGSAX	163.688	2.959	QEGRAW	153.612	2.863	QEGRAW	161.244	2.737	QEGRAW	168.561	3.346
QEHDIQ	160.172	2.933	QEHDIQ	175.646	3.333	QEGSAX	151.173	3.07	QEGXUY	163.018	3.006	QEHDIQ	155.524	3.295
QEHLEX	161.837	3.133	QEHLEX	158.633	3.25	QEHFAM	150.438	2.853	QEHFAM	150.014	2.849	QEHLEX	166.337	3.285
QEHLEX	160.756	3.021	QEHLEX	160.952	3.039	QEHLEX	151.538	3.243	QEHLEX	155.441	3.467	QEHLEX	166.833	2.975
QEHNUP	157.519	3.415	QEHSOM	159.443	3.389	QEHLEX	152.984	2.824	QEHLEY	165.669	3.203	QEHNUP	173.095	2.893
QEHWIK	172.324	3.223	QEHWUV	163.613	3.26	QEHSOM	169.365	3.116	QEHSOM	156.083	2.908	QEHWIK	150.947	2.769
QEHWUV	160.451	3.061	QEHWUV	158.779	3.38	QEHWUV	158.502	3.292	QEHWUV	166.152	3.296	QEHWUV	161.81	3.099
QEHXAC	155.59	3.135	QEHXAC	172.951	3.119	QEHXAC	161.667	3.135	QEHXAC	154.308	3.236	QEHXAC	156.027	3.057
QEHXEG	152.903	2.918	QEHXEG	159.722	2.837	QEHXEG	157.49	3.082	QEHXEG	152.016	3.076	QEHXEG	158.992	2.979
QEHXUX	155.885	3.448	QEHZOW	152.742	2.587	QEHXIL	152.934	2.81	QEHXIL	165.571	3.445	QEHXIL	152.811	2.81
QEJKAS	154.355	3.052	QEJKAS	150.14	3.436	QEHZUC	158.123	3.22	QEJDIV	174.882	3.462	QEJKAS	150.965	3.01
QEJKIA	154.597	2.876	QEJKIA	153.534	2.929	QEJKAS	158.792	2.969	QEJKAS	158.316	3.358	QEJKAS	158.889	3.123
QEJKIA	152.317	3.452	QEJKIA	157.294	3.177	QEJKIA	150.918	3.314	QEJKIA	167.032	2.964	QEJKIA	151.324	3.137
QEJKIA	173.66	2.669	QEJKIA	161.038	2.964	QEJKIA	155.208	3.075	QEJKIA	163.394	3.181	QEJKIA	152.864	3.189
QEJKOG	152.305	3.439	QEJKOG	169.859	3.062	QEJKIA	161.206	3.29	QEJKOG	164.64	3.49	QEJKOG	159.764	3.151
QEJPEB	150.661	2.94	QEKCAN	162.28	3.091	QEJNEA	163.027	2.903	QEJNEA	151.794	3.178	QEJPEB	169.677	3.471
QEKGAP	158.308	3.007	QEKGET	153.832	3.243	QEKCAN	162.449	3.091	QEKCER	157.951	2.6	QEKCER	158.872	3.304
QEKQEF	154.166	2.832	QEKQUV	156.898	3.308	QEKQAB	168.24	3.315	QEKQAB	163.608	2.966	QEKQAB	156.796	3.035
QELHOI	163.131	3.314	QELKAX	160.088	3.485	QEKRAZ	166.757	3.447	QELCIW	167.77	3.255	QELCIW	157.315	3.302
QELSAE	162.346	3.015	QELSIM	152.42	3.399	QELKAX	155.949	2.921	QELKAX	167.942	2.651	QELRIL	151.258	2.85
QELWUZ	154.343	3.149	QELWUZ	166.96	2.937	QELSIM	159.251	2.93	QELTAF	163.055	3.042	QELWUZ	150.833	2.797
QELXUE	160.089	3.391	QELXUE	173.916	3.487	QELWUZ	177.83	2.797	QELWUZ	155.732	3.397	QELXUE	162.169	2.886
QEMFOE	162.12	3.394	QEMFOE	161.137	3.265	QELXUE	164.512	3.189	QEMFOE	150.186	2.963	QEMFOE	151.013	3.214
QEMFOE	150.886	3.441	QEMFOE	171.178	3.052	QEMFOE	164.49	3.104	QEMFOE	165.449	3.424	QEMFOE	158.121	2.869
QEMPUU	177.57	3.068	QEMPUU	169.942	2.966	QEMKOJ	163.997	2.845	QEMKOJ	156.065	2.822	QEMKOJ	154.542	3.497
QEMSOR	171.658	3.339	QEMSOR	168.218	3.01	QEMPUU	174.965	3.09	QEMPUU	169.68	3.266	QEMSEK	168.081	2.922
QEMSOR	158.146	2.906	QEMWIO	162.269	3.234	QEMSOR	150.639	3.138	QEMSOR	173.969	3.138	QEMSOR	150.9	3.09
QEMWIO	163.647	3.49	QEMWIO	151.697	2.891	QEMWIO	159.563	3.19	QEMWIO	154.538	2.927	QEMWIO	155.312	2.986
QEMWUA	159.797	3.176	QEMWUA	157.808	3.207	QEMWUA	162.589	3.01	QEMWUA	168.003	3.11	QEMWUA	171.395	3.207
QEMXEL	164.026	3.448	QENDOF	160.272	3.144	QEMXEL	154.131	3.048	QEMXEL	165.178	2.993	QEMXEL	164.978	2.82
QENVIS	161.874	2.867	QEPDUO	162.669	2.917	QENDUL	173.281	2.841	QENNIK	151.364	3.291	QENVIS	151.975	2.867
QEPHIC	156.486	3.245	QEPKOM	156.776	2.629	QEPDUO	169.859	3.27	QEPGEX	157.894	2.855	QEPGIE	175.085	3.257
QEPNUX	169.207	2.856	QEPNUX	152.768	2.79	QEPKOM	161.551	3.22	QEPKOM	155.664	2.923	QEPKOM	150.343	3.361
QEPNUX	178.671	2.909	QEPNUX	153.929	3.363	QEPNUX	150.891	2.827	QEPNUX	159.944	3.189	QEPNUX	150.057	3.063
QEPPUY	154.315	3.324	QEPPUY	159.212	2.854	QEPPUY	152.02	2.963	QEPPUY	152.511	3.334	QEPPUY	156.542	3.037
QEPPUY	166.868	3.44	QEPPUY	159.187	3.115	QEPPUY	150.476	3.002	QEPPUY	171.512	3.329	QEPPUY	161.264	3.03
QEPQAF	156.305	2.957	QEPQAF	150.4	2.928	QEPQAF	151.688	3.401	QEPQAF	159.767	2.966	QEPQAF	164.457	3.377
QEPQAF	153.84	3.197	QEPQAF	161.275	2.887	QEPQAF	157.424	2.795	QEPQAF	153.529	2.997	QEPQAF	168.216	2.926
QEPQAF	151.416	3.401	QEPQAF	166.582	3.199	QEPQAF	150.952	3.23	QEPQAF	150.127	2.81	QEPQAF	152.276	3.27
QEPYUH	163.319	3.305	QEPZEU	155.757	3.282	QEPQAF	158.523	2.795	QEPQAF	159.095	2.764	QEPYUH	150.685	3.305
QEQDIZ	155.649	3.42	QEQGOI	159.553	3.387	QEQDIZ	154.22	3.405	QEQDIZ	155.33	3.115	QEQDIZ	165.348	3.312
QEQXIW	161.492	2.873	QEQXIW	176.652	3.156	QEQPIP	151.241	3.483	QEQPIP	160.074	3.209	QEQXIW	164.635	2.784
QEQXIW	151.798	3.235	QEQXIW	161.415	3.114	QEQXIW	165.215	3.023	QEQXIW	165.835	3.127	QEQXIW	154.095	2.848
QEQXIW	150.246	3.406	QEQXIW	157.201	3.072	QEQXIW	166.233	3.004	QEQXIW	157.493	3.099	QEQXIW	150.977	3.014
QERGUP	162.617	3.259	QERGUP	167.233	2.912	QEQXIW	162.455	2.728	QERGUP	163.859	3.374	QERGUP	154.73	3.204
QERPEJ	165.387	3.02	QERPEJ	161.338	3.052	QERGUP	164.646	2.981	QERGUP	150.539	3.27	QERMIM	150.967	3.113
QERSOY	164.841	2.993	QESBOI	162.96	3.064	QERPEJ	160.5	2.961	QERSAI	171.617	3.173	QERSOY	162.1	3.434
QESJEH	159.405	2.941	QESZAQ	163.315	2.609	QESCUM	160.087	3.332	QESCUM	160.609	3.145	QESCUM	158.115	3.218
QETGEE	155.38	3.461	QETPAJ	167.277	3.429	QESZEU	154.888	3.489	QESZEU	161.291	2.902	QETGEE	163.791	2.947
QETXOD	171.25	3.232	QETXOF	174.152	3.366	QETVER	162.11	3.109	QETXET	165.734	3.28	QETXIZ	173.965	3.393
QETYEU	165.405	2.793	QETYEU	169.758	2.817	QETXUJ	157.263	3.438	QETXUJ	156.408	2.86	QETXUL	174.415	3.324
QETYEU	158.526	2.793	QETZOH	161.275	3.04	QETYEU	165.863	3.214	QETYEU	157.618	3.122	QETYEU	154.923	3.18
QETZOH	160.596	3.022	QEVCUP	160.371	3.276	QETZOH	170.512	3.281	QETZOH	154.264	3.487	QETZOH	156.665	3.281

QEVCUT	162.773	3.385	QEVMIN	158.417	3.42	QEVCUP	155.873	3.227	QEVCUP	152.822	3.117	QEVCUT	158.723	2.533
QEWMUE	159.18	2.844	QEWVAP	153.129	3.064	QEVMIN	156.537	3.077	QEWMEL	164.995	3.059	QEWMUE	150.307	2.983
QEWVIX	169.102	2.733	QEWVIX	171.826	2.821	QEWVAP	153.373	3.099	QEWVAP	158.754	3.281	QEWVET	174.715	3.19
QEWWOH	154.774	2.802	QEXBAA	164.301	3.333	QEWVOH	159.401	3.487	QEWVOH	155.782	3.021	QEWWIB	174.972	2.977
QEXCOO	163.665	3.48	QEXHEH	162.78	2.63	QEXBAA	157.398	2.873	QEXBEE	150.372	2.881	QEXBEE	157.654	2.844
QEXLIS	158.22	3.36	QEXLIS	157.14	3.344	QEXHEH	154.861	3.08	QEXHEH	166.586	3.071	QEXHEH	172.068	2.97
QEXQIW	160.396	3.044	QEXRAQ	157.424	3.171	QEXLIS	151.631	3.383	QEXLOY	160.52	3.209	QEXQIW	156.31	3.365
QEYJAJ	173.138	2.872	QEYJOW	150.939	2.932	QEXVEV	166.718	3.148	QEYFOP	159.456	3.476	QEYFOP	151.858	2.987
QEYSAO	153.249	3.04	QEYSAO	165.781	2.986	QEYJUC	151.377	2.913	QEYSAO	156.669	3.158	QEYSAO	151.162	2.888
QEYSUJ	160.263	3.091	QEYSUJ	154.782	3.095	QEYSAO	167.684	3.252	QEYSAO	155.815	3.063	QEYSET	164.228	3.307
QEYSUJ	154.855	3.016	QEYSUJ	157.669	3.451	QEYSUJ	152.128	3.5	QEYSUJ	166.454	3.335	QEYSUJ	155.978	3.485
QEYSUJ	157.379	3.09	QEYSUJ	160.206	2.957	QEYSUJ	163.584	3.076	QEYSUJ	160.55	2.712	QEYSUJ	154.305	3.016
QEYSUJ	162.59	3.462	QEYSUJ	163.615	3.18	QEYSUJ	162.125	3.343	QEYSUJ	150.55	3.405	QEYSUJ	166.164	3.431
QEYSUM	152.003	2.918	QEYSUM	170.617	3.009	QEYSUJ	158.665	2.921	QEYSUJ	159.44	3.133	QEYSUJ	156.311	3.381
QEYZAW	167.044	2.832	QEYZIE	163.012	2.994	QEYTEX	150.196	3.402	QEYTUK	168.726	3.139	QEYZAW	165.297	2.886
QEZLUE	162.997	3.141	QEZLUE	158.231	3.206	QEZJOU0	165.524	3.028	QEZLUE	170.03	3.459	QEZLUE	167.748	3.026
QEZNES	163.282	2.838	QEZNES	155.859	3.43	QEZLUE	159	3.353	QEZNES	157.972	3.248	QEZNES	150.155	2.97
QEZNOC	165.328	3.02	QEZNOC	151.102	3.375	QEZNES	157.75	3.356	QEZNES	152.494	3.316	QEZNES	161.597	2.846
QEZPIY	152.202	2.989	QEZPIY	166.728	2.989	QEZNOC	169.872	3.15	QEZNUI	152.435	3.349	QEZPAQ	156.861	2.821
QEZWOH	172.277	3.254	QEZWOH	155.581	2.978	QEZWOH	161.812	2.852	QEZWOH	152.726	3.132	QEZWOH	169.797	3.053
QEZWOK	151.062	3.07	QEZWOK	157.443	3.04	QEZWOH	166.451	3.017	QEZWOH	154.019	3.268	QEZWOH	157.479	2.893
QEZXIE	164.526	3.275	QEZXIE	152.933	2.875	QEZXIE	162.945	3.246	QEZXIE	158.151	3.193	QEZXIE	151.59	2.826
QEZXOK	150.102	2.828	QEZXOK	166.534	3.163	QEZXIE	168.916	3.246	QEZXOK	160.704	3.28	QEZXOK	159.331	3.131
QIBHAL	172.989	2.617	QIBHAL	169.959	3.306	QEZXOK	153.339	2.879	QEZXOK	167.504	3.28	QIBFAL	169.914	3.276
QIBRUR	156.344	3.081	QIBTON	167.46	3.3	QIBHAL	150.536	3.25	QIBMUM	158.39	3.27	QIBQUO	150.971	3.312
QIBXEI	157.028	3.312	QIBXEI	163.57	3.418	QIBTON	150.479	3.277	QIBTUT	150.726	3.288	QIBTUT	167.446	3.29
QIBXEI	164.315	2.876	QIBXEI	150.756	3.306	QIBXEI	153.781	2.912	QIBXEI	170.419	3.003	QIBXEI	174.81	2.841
QIBXEI	156.624	2.912	QIBXEI	163.665	3.115	QIBXEI	159.745	3.115	QIBXEI	150.829	3.391	QIBXEI	165.847	3.095
QIBXEI	160.953	2.639	QIBXIM	178.135	2.976	QIBXEI	150.07	3.491	QIBXEI	158.385	3.357	QIBXEI	161.678	3.251
QIBXUY	150.574	2.863	QIBXUY	156.026	2.742	QIBXIM	166.811	2.876	QIBXIM	150.658	2.844	QIBXIM	162.687	2.844
QIBZAF	167.927	3.416	QIBZEJ	150.062	3.373	QIBXUY	154.304	2.742	QIBZAF	150.859	2.854	QIBZAF	151.736	2.854
QIBZEJ	157.934	3.157	QIBZOT	150.681	3.331	QIBZEJ	156.977	3.137	QIBZEJ	150.461	2.843	QIBZEJ	167.464	3.157
QIBZOT	151.909	3.36	QIBZUA	157.853	3.198	QIBZOT	158.239	3.39	QIBZOT	174.597	3.173	QIBZOT	159.134	3.291
QICCOW	154.073	3.041	QICCUC	150.617	3.428	QICBAI	172.396	2.814	QICCOW	175.477	3.082	QICCOW	153.246	2.827
QICDUF	152.972	2.747	QICDUF01	152.634	3.274	QICDUF	151.289	2.813	QICDUF	154.268	2.747	QICDUF	150.155	3.159
QICDUF01	161.347	3.173	QICDUF01	162.505	2.961	QICDUF01	165.07	3.301	QICDUF01	159.281	3.288	QICDUF01	152.673	3.351
QICJUI	154.036	3.45	QICJUI	153.926	2.94	QICHES	157.033	3.361	QICHES	150.119	3.393	QICJUI	157.069	2.976
QICLEU	166.284	3.381	QICPAV	157.813	2.926	QICKIZ	173.039	2.946	QICLAQ	154.324	3.128	QICLEU	165.004	3.448
QIDCEM	161.712	3.361	QIDCUF	152.026	3.382	QICQAY	159.336	3.11	QICQAY01	161.525	3.086	QICQIG	164.372	2.953
QIDCUF	154.213	3.009	QIDDIQ01	153.45	3.202	QIDCUF	165.966	3.382	QIDCUF	157.075	2.849	QIDCUF	162.085	2.865
QIDKOF	158.526	2.928	QIDLEY	164.468	2.993	QIDKEV	150.221	2.777	QIDKOF	159.662	3.154	QIDKOF	153.666	3.421
QIDLEY	150.511	2.826	QIDLEY	151.54	2.835	QIDLEY	166.307	3.006	QIDLEY	162.926	3.035	QIDLEY	165.49	3.048
QIDLEY	153.973	3.454	QIDREB	177.895	3.197	QIDLEY	153.622	2.844	QIDLEY	152.832	2.869	QIDLEY	151.124	2.932
QIDREB	152.434	3.381	QIDREB	162.355	3.085	QIDREB	164.929	2.878	QIDREB	152.95	3.261	QIDREB	174.23	3.193
QIDREB	157.99	3.134	QIDREB	157.861	2.839	QIDREB	166.061	3.172	QIDREB	150.236	3	QIDREB	173.079	3.318
QIDRIG	154.442	3.172	QIDRIG	169.258	2.768	QIDREB	164.725	3.284	QIDREB	155.601	2.918	QIDRIG	162.774	2.995
QIDROM	168.207	2.688	QIDROM	151.674	3.266	QIDRIG	164.63	2.768	QIDROM	167.928	3.173	QIDROM	162.945	2.892
QIDROM	161.017	3.445	QIDWAE	164.374	2.831	QIDROM	176.888	3.299	QIDROM	153.233	2.929	QIDROM	159.482	2.977
QIDWAE	156.801	3.472	QIDWAE	165.228	2.757	QIDWAE	153.026	3.289	QIDWAE	152.156	2.943	QIDWAE	170.913	3.201
QIDWAE	164.173	3.056	QIDWEI	155.344	3.214	QIDWAE	161.145	3.463	QIDWAE	154.395	2.873	QIDWAE	156.984	2.831
QIDWEI	154.53	3.345	QIDWEI	150.065	3.273	QIDWEI	166.863	3.09	QIDWEI	155.34	2.894	QIDWEI	163.275	2.837
QIDWUY	156.61	3.075	QIDWUY	156.491	3.378	QIDWUX	170.317	2.885	QIDWUY	169.506	3.294	QIDWUY	154.853	3.386
QIDWUY	152.946	2.957	QIDXEJ	173.148	3.466	QIDWUY	153.709	2.878	QIDWUY	150.707	3.075	QIDWUY	173.876	2.901
QIDXEJ	152.618	2.791	QIDXEJ	172.437	3.393	QIDXEJ	155.177	3.386	QIDXEJ	165.862	2.88	QIDXEJ	154.152	2.74
QIDXEJ	160.051	2.977	QIDXEJ	163.157	2.883	QIDXEJ	169.738	2.94	QIDXEJ	152.139	2.94	QIDXEJ	152.715	3.071
QIDYOT	173.592	3.017	QIDYOT	153.763	3.264	QIDXEJ	150.189	2.88	QIDXIK	154.094	3.345	QIDXOQ	155.911	3.18
QIDZIN	156.081	3.167	QIDZIN	151.923	3.106	QIDZIN	160.167	2.939	QIDZIN	174.944	3.182	QIDZIN	153.515	3.08
QIDZIN	170.411	3.39	QIDZIN	156.633	3.105	QIDZIN	153.621	3.39	QIDZIN	154.459	3.08	QIDZIN	156.692	3.075
QIDZIN	154.928	3.316	QIDZIN	161.985	3.204	QIDZIN	154.468	3.254	QIDZIN	152.748	3.275	QIDZIN	170.452	3.075
QIFBUD	150.862	3.061	QIFBUD	172.812	3.283	QIDZOT	153.797	3.371	QIDZOT	154.255	3.229	QIDZOT	157.622	3.229
QIFDAN	158.094	3.323	QIFDOB	157.756	3.085	QIFBUD	163.895	3.144	QIFBUD	159.972	3.364	QIFDAN	170.882	3.058
QIFGET	156.076	3.261	QIFGIX	156.533	3.013	QIFGET	151.668	3.033	QIFGET	169.9	3.069	QIFGET	172.496	3.398
QIFHEU	150.571	3.128	QIFHEU	171.847	3.019	QIFGIX	162.996	3.085	QIFGIX	163.651	3.392	QIFGIX	158.509	3.259
QIFJAQ	153.909	3.236	QIFNIF	163.256	3.159	QIFHEU	152.155	3.211	QIFHEU	154.457	3.27	QIFJAQ	172.345	2.655
QIFYIP	174.912	3.117	QIFYIP	167.878	3.467	QIFNIF	152.351	3.048	QIFXAH	176.726	3.12	QIFYIP	161.065	3.261
QIFYIP	158.89	3.082	QIFYOV	150.577	3.423	QIFYIP	160.548	3.16	QIFYIP	173.505	2.846	QIFYIP	160.42	3.318
QIGLAW	156.457	3.278	QIGLAW	167.796	3.037	QIGBAN	159.104	2.931	QIGBAN	164.986	3.009	QIGLAW	159.464	3.216
QIGRUV	170.888	2.765	QIGSAD	153.046	3.325	QIGROQ	161.307	3.439	QIGROQ	154.338	3.013	QIGROQ	165.713	3.406
QIGTOS	153.997	3.329	QIGXIQ	157.212	3.186	QIGSAD	152.945	2.987	QIGSAD	172.327	2.941	QIGSOR	171.135	2.879
QIGYIR	155.832	2.955	QIGYIR	164.424	3.182	QIGXUD	156.81	2.708	QIGYIR	163.195	3.118	QIGYIR	167.929	3.118
QIHCOB	155.572	3.242	QIHCUH	171.377	2.966	QIHCOB	173.118	3.321	QIHCOB	151.36	3.114	QIHCOB	166.013	2.805
QIHDAO	169.383	3.277	QIHDAO	159.826	2.799	QIHCUH	169.995	3.044	QIHCUH	154.489	3.394	QIHDAO	150.135	2.964
QIHSEF01	151.55	2.673	QIHSUY	157.962	3.381	QIHDAO	150.603	2.723	QIHDAO	169.754	3.322	QIHGOG	165.896	3.211
QIJBUI	154.701	3.107	QIJHAU	156.624	3.399	QIHWUB	173.29	3.073	QIHWUB	158.48	3.195	QIHZEM	164.926	3.263
QIJSUB	159.21	3.188	QIJTEM	169.227	2.769	QIJQEH	162.247	3.337	QIJQEH	155.651	3.277	QIJQEH	162.232	3.115
QIJTEM	159.08	3.452	QIJTEM	176.8	2.91	QIJTEM	162.643	2.694	QIJTEM	165.8	2.736	QIJTEM	161.649	2.972
QIJYER02	150.056	2.957	QIJYER02	163.101	3.393	QIJTEM	174.06	2.812	QIJVAG	152.026	2.982	QIJYER02	158.921	2.771
QIKWEM	151.979	2.922	QIKWEP	159.539	3.028	QIJYER02	154.188	3.02	QIJYER02	177.958	3.035	QIKWEM	166.794	3.068
QIKWOZ	167.067	2.803	QILKON	165.741	3.136	QIKWEP	168.93	3.161	QIKWEP	153.131	2.794	QIKWOZ	151.673	3.176

QILKON	174.01	3.326	QILKON	157.296	3.493	QILKON	158.615	2.702	QILKON	152.925	3.274	QILKON	177.818	3.473
QILZIY	162.931	3.403	QILZIY	162.291	3.43	QILRAE	160.945	2.85	QILSAI	152.169	3.495	QILSAI	150.176	3.127
QIMBOH	174.22	2.861	QIMBOH	155.191	3.497	QILZOA	167.117	3.141	QIMBOH	151.883	2.853	QIMBOH	164.532	2.786
QIMFEB	167.37	3.352	QIMFEB	159.03	2.831	QIMBOH	155.701	3.007	QIMFEB	151.626	3.362	QIMFEB	153.023	2.787
QIMKIK	172.61	2.748	QIMKIK	154.601	3.226	QIMFEB	159.382	3.062	QIMFEB	155.701	3.092	QIMKIK	160.002	3.275
QIMKIK	173.232	3.309	QIMKIK	157.764	3.314	QIMKIK	155.894	3.262	QIMKIK	157.232	3.007	QIMKIK	153.974	3.248
QIMKIK	168.507	2.867	QIMKIK	150.928	2.929	QIMKIK	155.631	3.097	QIMKIK	151.297	3.452	QIMKIK	172.484	3.343
QIMKOQ	151.419	3.125	QIMKOQ	152.931	3.176	QIMKIK	155.038	3.412	QIMKIK	156.887	3.309	QIMKOQ	152.236	2.815
QIMKOQ	160.617	3.474	QIMKOQ	170.401	2.919	QIMKOQ	160.094	3.099	QIMKOQ	170.282	3.463	QIMKOQ	156.237	2.858
QIMKUS	150.079	3.126	QIMKUW	167.091	2.964	QIMKOQ	151.562	2.705	QIMKOQ	164.909	3.331	QIMKOQ	166.373	2.975
QIMLAD	159.497	2.93	QIMLEH	164.462	3.289	QIMKUW	162.448	2.957	QIMLAD	170.112	2.911	QIMLAD	152.987	3.058
QIMLEH	156.282	3.437	QIMLIL	160.964	3.041	QIMLEH	152.779	2.996	QIMLEH	173.744	3.251	QIMLEH	156.917	3.305
QIMQAI	153.006	2.839	QIMQAI	156.527	3.085	QIMQAG	150.378	2.899	QIMQAG	155.701	3.384	QIMQAI	160.861	3.082
QIMQEM	168.3	2.762	QIMQEM	168.689	2.758	QIMQAI	151.324	3.064	QIMQAI	168.91	2.839	QIMQEM	150.562	2.779
QIMTIR	150.307	3.222	QIMTIR	152.948	3.448	QIMQEM	157.472	3.387	QIMQUA	150.592	3.361	QIMQUA	169.383	3.049
QIMVAL	156.183	3.459	QIMVIT	152.148	3.496	QIMTIR	176.419	3.31	QIMTOX	164.786	3.281	QIMVAL	154.314	3.434
QIMVUH	167.349	2.69	QIMVUH	164.254	2.938	QIMVUF	150.71	3.402	QIMVUH	153.702	3.365	QIMVUH	160.583	2.643
QINDAV	160.614	3.089	QINDAV	153.024	3.275	QIMVUH	157.345	2.643	QIMVUH	167.408	3.013	QIMVUH	160.216	3.013
QINKIH	172.457	2.763	QINKIH	159.008	3.025	QINDID	179.538	2.951	QINDID	150.185	2.962	QINKEH	152.624	2.813
QINKIH	153.849	2.904	QINKIL	172.606	3.124	QINKIH	158.39	3.084	QINKIH	150.527	3.164	QINKIH	173.726	2.73
QINKON	172.5	2.753	QINKON	158.467	3.038	QINKIL	169.44	2.876	QINKIL	171.888	3.366	QINKIL	153.643	3.433
QINKON	152.669	2.915	QINKOR	152.822	3.177	QINKON	159.216	3.096	QINKON	150.129	3.173	QINKON	173.092	2.73
QIPGUR	163.816	3.417	QIPGUR	156.644	2.878	QINLEI	169.971	3.402	QINXES	168.195	2.806	QINXES	175.308	3.154
QIPLUZ	164.971	3.07	QIPNIO	173.594	2.698	QIPGUR	150.041	2.769	QIPGUR	169.627	3.383	QIPLUZ	158.539	2.773
QIPRUE	166.303	3.402	QIPSUD	167.265	3.229	QIPNIO	165.14	2.935	QIPNIO	172.747	2.698	QIPNIO	160.882	3.409
QIQFUV	152.541	3.096	QIQFUV	156.616	2.898	QIPVES01	152.711	3.393	QIQCOL	158.767	3.321	QIQFUV	164.302	3.046
QIQGIK	157.5	3.362	QIQGIK	159.022	3.06	QIQFUV	163.557	3.221	QIQFUV	171.032	3.418	QIQGIK	155.138	3.394
QIQNEN	152.573	3.306	QIQPEP	152.816	2.863	QIQLEJ	172.345	3.476	QIQMUC	175.304	2.939	QIQNEN	172.878	3.12
QIQPUF	160.087	3.453	QIQQEQ	152.193	2.862	QIQPEP	165.949	3.179	QIQPEP	156.385	2.863	QIQPUF	150.104	3.481
QIQZAV	156.136	3.327	QIRCIF	159.893	2.896	QIQQEQ	154.247	3.435	QIQZAV	162.05	2.915	QIQZAV	152.771	3.44
QIRFUW	177.173	2.796	QIRFUW	162.875	2.788	QIRFUW	154.993	3.354	QIRFUW	167.748	2.988	QIRFUW	154.451	2.796
QIRGUX	161.378	3.098	QIRGUX	156.674	2.938	QIRFUW	157.151	3.199	QIRGAD	151.646	2.797	QIRGUX	152.029	3.018
QIRJIO	157.592	3.312	QIRJIO	152.652	3.377	QIRJAG	174.127	3.368	QIRJIO	150.647	3.252	QIRJIO	159.028	2.871
QIRMUD	164.095	2.902	QIRNOY	165.055	2.818	QIRLAI	152.365	2.973	QIRLAI	150.006	3.175	QIRLAI	170.44	2.877
QIRXAS	168.844	3.399	QIRXAS	153.884	3.29	QIRQAL	162.993	3.228	QIRSET	163.068	3.381	QIRXAS	161.384	2.893
QIRYOG	161.746	3.407	QIRYOG	150.837	3.398	QIRYEW	151.713	3.489	QIRYOG	154.77	3.037	QIRYOG	155.186	3.128
QISFAD	157.334	2.729	QISFAD	154.175	3.303	QIRYOG	153.293	3.391	QIRYOG	167.179	3.217	QISBUS	161.199	3.194
QISFOR03	168.645	3.024	QISHEF	172.471	3.024	QISFOR02	167.255	2.873	QISFOR02	168.82	3.02	QISFOR03	167.667	2.874
QITRAO	151.141	2.938	QITRAO	172.264	3.222	QISHOT	158.498	3.361	QISMAK	156.595	3.013	QISNOY	151.379	3.241
QITRAO02	151.431	2.935	QIVHOV	151.199	3.06	QITRAO01	171.824	3.22	QITRAO01	151.396	2.939	QITRAO02	171.793	3.218
QIVHOV	164.428	2.952	QIVHOV	157.009	3.38	QIVHOV	160.019	3.251	QIVHOV	164.339	2.86	QIVHOV	170.069	3.076
QIVKEO	168.316	3.106	QIVKEO	161.977	2.907	QIVHOV	155.993	2.944	QIVHUB	157.049	2.955	QIVHUB	153.714	2.955
QIVKIR	173.259	2.828	QIVLAL	156.818	3.2	QIVKEO	164.962	3.049	QIVKEO	164.332	2.764	QIVKEO	159.009	3.373
QIVLAL	157.809	2.971	QIVLAL	176.576	3.2	QIVLAL	164.328	2.639	QIVLAL	155.133	2.84	QIVLAL	150.459	3.449
QIVLIT	156.054	2.934	QIVLIT	156.538	3.153	QIVLAL	167.953	3.339	QIVLAL	162.176	3.139	QIVLAL	150.791	3.339
QIVLIT	159.538	3.225	QIVLOZ	162.958	3.17	QIVLIT	158.872	2.768	QIVLIT	161.836	3.003	QIVLIT	154.102	3.153
QIVMOZ	173.083	3.45	QIVMOZ	162.868	2.688	QIVLOZ	163.824	3.263	QIVLOZ	153.089	2.777	QIVMIT	150.602	3.408
QIVMUF	170.917	3.272	QIVROF	172.895	3.136	QIVMOZ	164.508	2.688	QIVMOZ	151.283	2.925	QIVMOZ	174.147	3.122
QIVSOH	151.595	3.291	QIVSOH	158.601	3.152	QIVSOH	151.606	2.881	QIVSOH	169.15	3.058	QIVSOH	153.309	3.414
QIVWEX	153.179	3.48	QIVWIB	177.206	3.007	QIVSOH	160.162	3.22	QIVSOH	166.564	3.425	QIVWAT	152.027	3.48
QIVXAW	167.598	3.397	QIVXAW	177.838	2.795	QIVWIB	153.593	3.425	QIVWIB	151.647	3.075	QIVWIB	162.546	3.072
QIVXIE	157.892	2.899	QIWDUZ	165.441	3.448	QIVXAW	168.338	2.951	QIVXAW	158.347	3.397	QIVXIE	159.025	2.879
QIXCEF	154.2	2.837	QIXCEF	158.88	2.934	QIWDUZ	174.68	3.448	QIXCEF	152.182	2.817	QIXCEF	151.642	3.343
QIXCEF01	161.687	2.844	QIXCEF01	158.999	2.919	QIXCEF	161.609	2.859	QIXCEF01	151.749	2.799	QIXCEF01	151.769	3.305
QIXCEF02	158.94	2.933	QIXCEF02	151.663	3.324	QIXCEF01	153.966	2.822	QIXCEF02	151.691	2.811	QIXCEF02	154.119	2.831
QIXREW	151.804	3.148	QIXSUM	150.396	2.958	QIXCEF02	161.909	2.857	QIXHEM	158.558	3.103	QIXJEP	151.691	3.468
QIXSUM	172.113	3	QIXSUM	175.402	3.235	QIXSUM	157.22	3.266	QIXSUM	168.035	2.781	QIXSUM	157.936	3.047
QIXTEX	154.326	3.044	QIXTEX	150.826	3.065	QIXSUM	156.86	3.137	QIXSUM	155.612	3.044	QIXSUM	174.045	3.015
QIXTEX	165.516	3.154	QIXTEX	158.064	3.154	QIXTEX	158.912	3.124	QIXTEX	161.383	3.025	QIXTEX	167.414	3.154
QIXVAX	154.275	3.014	QIXVAX	169.19	2.968	QIXTEX	156.678	2.83	QIXTEX	165.399	3.335	QIXVAX	157.946	2.968
QIXVIG	158.839	3.462	QIXVIG	168.003	3.046	QIXVAX	150.996	3.012	QIXVAX	151.162	3.056	QIXVIG	167.683	2.973
QIXWOM	151.223	3.458	QIXWOM	167.714	2.934	QIXWIG	174.384	2.867	QIXWIG	165.573	3.014	QIXWOM	159.517	3.365
QIYLIX	150.081	2.924	QIYLIX	157.038	2.692	QIXWOM	172.813	2.762	QIXWOM	168.848	2.882	QIYLIX	172.748	3.08
QIYMAQ	162.664	3.493	QIYMAQ	152.518	3.496	QIYLIX	176.348	3.086	QIYLUJ	174.491	3.254	QIYMAQ	170.59	2.871
QIYPIX	151.589	3.362	QIYPIX	157.002	3.195	QIYMAQ	166.503	3.2	QIYMAQ	151.684	3.213	QIYPET	153.854	3.323
QIYQUK	163.784	3.112	QIYRAR	153.916	3.258	QIYQUK	166.583	3.232	QIYQUK	150.534	3.032	QIYQUK	154.947	3.486
QIYREV	163.68	3.332	QIYREV	172.452	3.478	QIYRAR	162.771	3.355	QIYRAR	173.64	3.469	QIYREV	156.906	3.211
QIYTOJ	152.754	2.793	QIYZOR	152.217	3.022	QIYTOJ	163.155	3.27	QIYTOJ	150.409	3.281	QIYTOJ	155.447	3.439
QIZBEH	159.369	2.833	QIZBEH	150.072	3.452	QIYZOR	164.811	3.022	QIZBEH	168.213	3.141	QIZBEH	173.56	3.236
QIZTIE	164.972	3.241	QIZTOK	164.023	3.481	QIZHUG	166.908	2.878	QIZHUG	153.102	2.878	QIZKER	154.352	3.468
QIZZIJ	162.448	2.849	QIZZOP	152.33	3.262	QIZTOK	162.972	2.805	QIZXIK	165.558	3.118	QIZZIJ	156.014	2.849
QOBDOC	159.568	3.335	QOBKEB	150.578	2.936	QIZZOP	152.833	2.915	QOBDES	171.688	3.219	QOBDOC	158.777	3.347
QOCDOD	170.379	3.312	QOCJEB	152.59	2.89	QOBKEB	162.884	2.77	QOBPIK	167.973	2.905	QOCDOD	163.549	3.3
QOCKIG	152.677	3.305	QOCKIG	163.385	2.695	QOCJEB	158.748	2.913	QOCJEB	162.3	2.99	QOCKIG	159.814	2.778
QOCRUZ	153.128	3.085	QOCRUZ	159.231	3.063	QOCKIG	159.513	2.695	QOCMIG	168.301	3.235	QOCRUZ	152.161	3.287
QOCWOY	159.728	3.01	QOCWOY	160.258	3.301	QOCRUZ	163.648	2.748	QOCWOY	167.576	3.299	QOCWOY	150.277	3.431
QOCZUG	159.188	3.462	QODHOH	160.205	3.122	QOCXOW	152.368	3.37	QOCXOW	152.531	3.132	QOCXOW	151.271	3.39
QODKUR	175.528	3.176	QODKUR	177.79	3.215	QODHOH	153.085	3.438	QODHOH	153.334	2.87	QODHOH	158.171	3.107
QODKUR	169.914	3.476	QODKUR	178.499	3.064	QODKUR	171.508	3.428	QODKUR	170.896	3.449	QODKUR	171.381	3.433

QODLOM	159.844	2.856	QODLOM	162.512	3.4	QODKUR	175.251	3.229	QODLIG	151.098	3.024	QODLOM	153.712	3.014
QODMUU	151.458	3.403	QODNOP	152.707	2.883	QODLOM	152.222	2.856	QODLOM	156.608	2.931	QODMOO	153.461	3.336
QODPOP	163.821	2.7	QODPOP	176.891	2.937	QODPAD	152.967	3.246	QODPEH	158.258	2.935	QODPEH	159.466	3.1
QODPUV	151.66	2.99	QODQAE	155.764	3.311	QODPOP	150.241	3.464	QODPOP	152.452	2.999	QODPOP	164.901	2.865
QODRAF	150.754	3.303	QODRUZ	157.733	3.263	QODQIM	153.791	3.34	QODQIM	163.281	2.695	QODRAF	162.526	2.837
QOFFOG	166.354	3.303	QOFFOG	166.907	3.337	QODVAK	154.744	2.86	QODYAJ	157.765	3.278	QODYAJ	160.268	3.19
QOFJEB	160.606	3.302	QOFKEC	167.006	3.115	QOFFUM	168.781	3.016	QOFFUM	174.463	2.914	QOFJEB	165.952	2.981
QOFQEK	174.282	3.084	QOFQEK0	162.305	2.938	QOFKEC	150.654	2.865	QOFKEC	166.873	3.114	QOFQEK	162.519	2.925
QOGCEU	155.217	3.183	QOHCUM	155.412	3.416	QOFQEK0	173.627	3.087	QOFREL	155.044	3.326	QOFXUE	173.534	3.012
QOHGEA	159.007	3.287	QOHXIU	167.043	2.976	QOHCUP	151.811	3.352	QOHCUP	157.876	3.424	QOHGEA	155.835	3.225
QOJDAV	158.177	3.22	QOJDAV	154.093	3.459	QOHXIU	162.34	3.432	QOHXIU	153.448	3.397	QOJDAV	150.898	3.034
QOJHED	170.86	3.377	QOJHED	174.029	2.879	QOJDAV	167.525	2.886	QOJHED	172.293	3.117	QOJHED	150.804	2.915
QOJROW	162.104	2.697	QOKDIE	175.842	2.754	QOJHED	161.488	2.879	QOJHED	166.082	2.915	QOJHED	152.098	3.492
QOKHEH	166.028	3.037	QOKKAD	162.605	3.063	QOKDIE	166.543	2.754	QOKDOK	160.535	2.94	QOKDOK	154.702	2.94
QOLCUQ	162.382	2.798	QOLCUQ	153.482	2.926	QOKKAD	150.359	3.176	QOKQAL	161.178	3.493	QOKQAL	169.299	3.334
QOLJEK	153.481	2.991	QOLJOU	150.962	3.027	QOLGUX	154.454	2.976	QOLHAE	155.885	3.003	QOLJAG	155.443	2.997
QOLZEW	154.294	3.035	QOLZEW	160.099	2.946	QOLLAH	156.14	3.196	QOLNIS	154.839	2.937	QOLZEW	166.047	3.031
QOLZEW	150.072	3.337	QOLZEW	160.959	3.268	QOLZEW	161.19	2.873	QOLZEW	165.706	2.775	QOLZEW	158.066	2.833
QOMDUU	165.213	3.035	QOMHEJ	170.815	3.243	QOLZUM	154.604	3.01	QOLZUM	161.164	3.01	QOLZUM	171.681	3.32
QONBOM	169.252	3.446	QONBOM	170.237	3.465	QOMKIM	160.385	3.263	QOMLIO	154.489	2.998	QOMYEA	154.406	3.197
QONBOM	169.306	3.24	QONCOP	159.479	3.358	QONBOM	151.517	2.972	QONBOM	153.439	2.985	QONBOM	169.164	3.24
QONKEL	169.378	3.338	QONKEL	157.352	3.452	QONCOP	156.9	3.12	QONKEL	162.411	3.091	QONKEL	155.94	3.125
QONKIP	161.905	2.78	QONKIP	152.277	2.78	QONKEL	154.995	2.84	QONKEL	155.9	3.1	QONKIP	171.09	3.001
QONTES	170.165	2.996	QONTES	173.324	3.003	QONNOW	150.092	3.436	QONNOW	168.128	2.924	QONNUC	157.008	3.355
QONZIG	152.497	3.26	QOPDUW	175.516	2.966	QONTIW	168.597	3.197	QONZIG	156.314	2.832	QONZIG	155.664	3.26
QOPJAH	153.533	3.146	QOPQIZ	158.745	2.809	QOPDUW	161.932	2.966	QOPJAH	152.456	2.785	QOPJAH	166.119	3.146
QOPRIY	154.837	3.215	QOPROE	169.817	3.301	QOPQIZ	167.423	3.284	QOPQIZ	175.573	3.229	QOPQIZ	155.842	3.495
QOQHIR	165.079	3.124	QOQHIR	154.961	3.455	QOPRUK	168.039	3.349	QOPRUK	165.049	3.075	QOQHIR	161.957	3.128
QOQJEP	152.672	2.97	QOQJEP	153.232	3.437	QOQHIR	164.405	3.129	QOQHIR	151.623	3.05	QOQHIR	153.497	3.05
QOQJEP	165.249	3.219	QOQPEV	170.363	2.983	QOQJEP	170.009	3.19	QOQJEP	161.268	2.993	QOQJEP	151.861	2.97
QOSBIK	159.723	3.473	QOSCAD	166.944	2.906	QOQPEV	170.385	2.983	QOQXED	161.867	3.346	QOSBAC	159.32	3.401
QOSFAG	156.357	3.268	QOSFAG	164.678	3.455	QOSCAD	154.877	2.93	QOSFAG	155.511	2.82	QOSFAG	166.822	3.451
QOSFAG	173.275	2.82	QOSFAG	173.099	2.81	QOSFAG	162.813	2.928	QOSFAG	166.886	2.81	QOSFAG	156.557	3.451
QOSFAG	152.604	2.913	QOSFAG	150.605	3.019	QOSFAG	159.73	3.313	QOSFAG	161.272	2.85	QOSFAG	151.195	3.285
QOSFAG	151.013	3.038	QOSFAG	168.495	2.78	QOSFAG	153.967	2.86	QOSFAG	150.903	3.277	QOSFAG	173.377	2.976
QOSMUH	165.433	2.958	QOSMUH	157.774	3.238	QOSFAG	164.732	2.78	QOSFAG	157.058	2.78	QOSMUH	159.064	3.101
QOSNAO	154.628	3.281	QOSNAO	158.952	3.475	QOSMUH	151.287	2.971	QOSMUH	166.713	2.958	QOSMUH	172.254	2.855
QOSNAO	153.49	3.072	QOSNAO	150.229	2.921	QOSNAO	156.558	3.165	QOSNAO	173.547	2.743	QOSNAO	165.455	3.307
QOSPIY	156.591	2.886	QOSPIY	154.857	3.094	QOSNAO	157.531	2.763	QOSNAO	155.421	3.414	QOSNAO	163.173	3.281
QOSQOG	164.808	2.963	QOSQOG	155.387	3.311	QOSPIY	153.434	2.886	QOSPIY	165.762	3.431	QOSPIY	151.964	3.337
QOSTIF	171.103	3.19	QOSTOI	164.631	3.369	QOSTIF	162.949	2.878	QOSTIF	168.865	3.196	QOSTIF	159.908	2.901
QOSYOO	156.69	2.858	QOSYOO	164.547	3.102	QOSYOO	155.678	3.184	QOSYOO	156.334	3.184	QOSYOO	161.389	2.854
QOVTOO	168.653	2.741	QOVTOO	154.035	3.145	QOSYOO	172.66	3.049	QOVPEA	152.002	3.354	QOVREA	157.632	2.756
QOWGAK	167.614	3.225	QOWGAK	151.058	2.982	QOVXEF	170.505	3.018	QOWGAK	154.957	3.112	QOWGAK	150.085	2.989
QOWJEV	157.322	3.132	QOWMOH	176.115	3.193	QOWGAK	152.916	3.2	QOWGAK	170.31	3.19	QOWJEV	150.642	3.196
QOWQEA	165.026	3.35	QOWQEA	169.908	3.363	QOWNEZ	153.66	3.281	QOWQEA	165.604	3.354	QOWQEA	152.545	3.446
QOWYOR	167.078	3.359	QOXDIU	156.917	2.991	QOWQEA	160.622	2.931	QOWQOL	158.15	3.167	QOWQOL	153.834	2.989
QOXJUM	154.46	3.463	QOXJUM	158.64	3.22	QOXDIU	150.977	3.422	QOXDIU	167.75	3.064	QOXHEU	170.446	3.27
QOXPEB	160.05	3.028	QOXPIF	167.914	3.298	QOXMOH	154.076	3.002	QOXMOH	167.888	3.08	QOXNIC	161.07	3.042
QOXSAX	173.103	2.955	QOXSAX	152.353	3.341	QOXPIF	159.642	3.434	QOXPIF	170.832	3.149	QOXPOL	153.833	2.894
QOXSAX	160.383	2.723	QOXSAX	160.74	3.485	QOXSAX	159.166	2.807	QOXSAX	161.831	3.488	QOXSAX	170.166	3.069
QOXVIK	162.611	2.734	QOXVIK	163.716	2.745	QOXSAX	156.567	2.862	QOXVIK	174.122	2.745	QOXVIK	155.227	2.787
QOYFIX	150.027	3.049	QOYFIX	164.352	3.036	QOXXUZ	152.234	3.001	QOYFET	150.376	3.476	QOYFET	163.704	3.492
QOYJAT	160.818	3.266	QOYJIB	154.955	3.15	QOYFIX	155.037	3.049	QOYHOF	150.879	2.857	QOYHUL	172.59	3.088
QOYLOJ	165.453	2.927	QOYLOJ	152.84	3.017	QOYKEY	163.382	3.132	QOYKUN	162.512	3.047	QOYLOJ	155.532	3.017
QOYNEA	161.729	3.41	QOYNEA	164.577	3.436	QOYLOJ	153.18	3.499	QOYNEA	155.144	3.41	QOYNEA	170.066	3.191
QOYRAZ	151.087	2.789	QOYRAZ	166.004	2.578	QOYNEA	150.179	3.02	QOYRAZ	171.209	2.916	QOYRAZ	156.144	3.033
QOYRIH	155.639	3.056	QOYRIH	156.461	2.838	QOYRAZ	180	2.913	QOYRAZ	180	2.913	QOYRED	159.505	3.076
QOYXAF	168.138	3.264	QOYXAF	155.2	2.859	QOYRIH	160.761	2.92	QOYSAB	166.874	3.186	QOYXAF	161.764	3.121
QOZGAR	154.973	3.342	QOZGAR	159.061	3.342	QOZDAM	164.96	3.307	QOZFOE	154.286	3.327	QOZFOE	158.548	3.327
QOZHAS	174.531	3.444	QOZHEW	157.568	3.142	QOZGEV	161.042	3.089	QOZGIZ	161.061	3.11	QOZHAS	155.409	3.133
QOZMIE	150.868	3.073	QOZNIE	156.317	3.482	QOZHEW	172.048	3.43	QOZKEZ	157.215	3.17	QOZMIE	151.575	3.061
QOZNUQ	159.451	3.399	QOZNUS	154.547	3.421	QOZNOK	170.516	3.116	QOZNOK	150.554	3.476	QOZNOK	161.512	3.051
QOZROO	170.267	2.972	QOZROO	172.858	2.848	QOZNUS	151.852	3.06	QOZROO	170.314	3.407	QOZROO	162.472	3.139
QOZSAB	170.661	2.971	QOZSAB	162.9	3.12	QOZSAB	150.474	3.005	QOZSAB	169.932	3.434	QOZSAB	172.411	2.845
QOZSEF	163.199	3.144	QOZSEF	152.268	2.99	QOZSEF	170.336	3.432	QOZSEF	171.035	2.968	QOZSEF	172.594	2.849
QOZTUU	156.482	2.989	QOZWAD	154.115	2.951	QOZTUU	164.544	3.4	QOZTUU	161.646	3.206	QOZTUU	156.103	3.288
QQQAUD0	155.521	3.431	QQQBWP	152.193	3.364	QOZWAD	158.231	2.905	QOZWAD	170.25	2.905	QOZZEO	167.096	2.891
QUBPIN01	152.101	3.159	QUBPIN01	159.617	3.269	QUBPIN	160.788	3.164	QUBPIN	162.03	3.42	QUBPIN01	159.102	3.036
QUBQEN	159.291	3.35	QUBQEN	165.128	3.167	QUBPIN03	160.6	3.161	QUBPIN03	161.86	3.414	QUBQEN	155.599	3.116
QUBYIZ	161.44	3.407	QUBYIZ	162.943	3.078	QUBVEP	160.489	3.02	QUBVIT	160.714	2.848	QUBWOC	160.87	2.983
QUCDOL	171.99	2.752	QUCDUR	154.79	3.417	QUBYIZ	153.379	2.95	QUCDOL	166.684	3.01	QUCDOL	152.805	3.271
QUCGUQ	157.114	3.354	QUCNUZ	154.364	3.111	QUCDUR	164.249	3.488	QUCDUR	151.441	2.897	QUCDUR	168.489	2.942
QUCNUZ	172.698	3.347	QUCNUZ	156.512	3.379	QUCNUZ	156.302	3.222	QUCNUZ	151.413	3.168	QUCNUZ	153.116	3.222
QUCXES	150.451	3.286	QUCZUM	171.471	3.237	QUCSIU	161.143	2.904	QUCSIU	158.308	2.863	QUCSIU	154.517	2.904
QUDDUP	155.798	3.228	QUDHIK	161.101	2.802	QUCZUM	173.862	3.039	QUCZUM	167.245	3.155	QUDCIE01	151.988	3.296
QUDJOO	160.887	3.181	QUDMAH	162.01	3.141	QUDHIK	166.156	2.904	QUDHIK	157.428	3.095	QUDHUW	167.186	3.249
QUDWUK	153.708	3.447	QUDXOD	167.111	2.8	QUDWOC	154.495	2.952	QUDWOC	156.01	2.952	QUDWUK	157.99	3.252
QUDXOD	165.577	2.974	QUFCUT	154.633	2.771	QUDXOD	154.141	2.903	QUDXOD	156.051	3.489	QUDXOD	162.553	2.828

QUFDEA	150.469	2.951	QUFDEA	154.299	3.493	QUFCUT	162.464	3.462	QUFDEA	153.904	3.086	QUFDEA	153.566	2.975
QUFDEA	162.996	3.5	QUFDEA	152.963	3.455	QUFDEA	155.428	3.43	QUFDEA	151.254	2.802	QUFDEA	153.895	2.876
QUFGUU	171.518	3.337	QUFGUW	158.468	3.356	QUFDEA	167.195	3.293	QUFDEA	160.943	2.725	QUFGUU	154.801	3.142
QUFHOS	161.1	2.879	QUFHOS	165.474	3.255	QUFHAB	153.519	3.141	QUFHAB	172.115	3.225	QUFHOS	157.051	3.346
QUFHOS	167.005	2.748	QUFHOS	151.659	2.898	QUFHOS	159.014	2.714	QUFHOS	160.909	3.164	QUFHOS	157.894	3.336
QUFTUK	157.399	3.117	QUFWEX	174.891	3.456	QUFLAE	154.735	3.246	QUFLOS	164.677	3.307	QUFPAJ	169.568	3.482
QUFWEX	173.098	3.474	QUFWEX	168.067	2.974	QUFWEX	158.406	3.4	QUFWEX	155.078	2.821	QUFWEX	167.579	2.766
QUFZAS	151.746	3.045	QUFZAS	150.506	3.348	QUFWEX	168.235	2.937	QUFWEX	159.254	3.192	QUFWEX	172.764	2.945
QUGCON	154.836	3.076	QUGNAL	166.07	3.464	QUFZAS	163.24	3.362	QUFZIA	168.479	3.046	QUFZIA	169.533	2.805
QUGQOB	161.101	3.019	QUGSIU	174.533	2.846	QUGNUE	167.131	3.408	QUGQOB	155.388	3.209	QUGQOB	163.941	2.935
QUHBOL	150.941	2.91	QUHBOL	154.312	3.418	QUGSIU	169.206	3.133	QUGWAT	168.044	3.293	QUGYIE	167.842	3.209
QUHCEC	166.044	3.181	QUHCEC	152.007	2.84	QUHBOL	159.783	3.057	QUHBUR	152.445	3.072	QUHBUR0	151.086	3.039
QUHGEH	172.933	3.251	QUHGEH	160.319	3.447	QUHCEC	162.693	3.076	QUHGEH	173.454	3.105	QUHGEH	160.298	3.266
QUHJOW	172.257	3.288	QUHXAW	166.339	2.881	QUHGEH	162.706	3.219	QUHGOT	165.62	3.472	QUHJOW	154.809	3.288
QUJFAE	162.111	2.949	QUJFAE	158.291	3.279	QUHXAW	164.58	2.983	QUHXAW	164.192	2.871	QUJFAE	152.05	3.279
QUJNOA	152.773	2.872	QUJQOD	160.432	3.366	QUJKAK	150.909	3.281	QUJNOA	155.149	2.679	QUJNOA	150.524	2.884
QUJREV	175.166	2.717	QUJREV	151.3	3.243	QUJRAR	151.79	3.167	QUJRAR	158.608	3.382	QUJREV	167.752	3.01
QUJRUL	151.376	2.805	QUJRUL	167.777	2.924	QUJREV	156.893	3.089	QUJREV	166.978	3.439	QUJRUL	166.622	2.65
QUJSAS	175.424	3.274	QUJSAS	154.858	3.059	QUJRUL	153.007	3.01	QUJRUL	174.593	3.089	QUJSAS	153.316	2.687
QUJSAS	162.949	2.687	QUJSAS	152.921	3.144	QUJSAS	157.224	3.059	QUJSAS	154.524	3.274	QUJSAS	162.715	3.331
QUJSEW	165.109	3.309	QUJSEW	164.882	3.373	QUJSEW	167.27	3.437	QUJSEW	172.381	3.407	QUJSEW	163.802	3.49
QUJSIB	162.984	2.92	QUJSOG	157.838	3.407	QUJSIA	163.085	2.806	QUJSIA	157.73	3.01	QUJSIA	157.183	3.032
QUKGAH	165.535	3.359	QUKGAH	154.025	2.86	QUJSOG	153.587	3.102	QUKDAD	160.566	3.339	QUKGAH	171.968	3.18
QUKQOE	150.6	3.233	QUKTEW	168.464	2.935	QUKGAH	151.398	3.443	QUKGAH	157.591	3.23	QUKQOE	154.37	3.186
QULTAV	150.024	2.917	QULTEZ	155.273	2.696	QULCEF	156.347	3.409	QULTAV	164.217	3.397	QULTAV	152.996	3.324
QULTEZ	164.143	3.482	QULTEZ	151.129	3.071	QULTEZ	166.327	3.35	QULTEZ	159.942	3.24	QULTEZ	150.64	2.919
QUMLOC	152.289	3.235	QUMLOC	151.579	3.219	QUMCOU	154.752	3.224	QUMCOU	151.287	3.227	QUMHOY	155.529	2.922
QUMLOC	157.17	2.799	QUMLOC	155.678	2.774	QUMLOC	157.067	3.374	QUMLOC	151.371	3.179	QUMLOC	162.091	2.925
QUMLOC	168.004	3.226	QUMLOC	153.383	3.289	QUMLOC	171.738	3.179	QUMLOC	155.262	2.953	QUMLOC	156.013	2.98
QUMLOC	152.109	2.925	QUMLOC	158.57	2.827	QUMLOC	161.606	2.844	QUMLOC	171.751	3.034	QUMLOC	152.007	2.887
QUMLOC	156.208	2.918	QUMLOC	162.121	3.471	QUMLOC	156.097	3.007	QUMLOC	166.499	2.707	QUMLOC	150.441	2.99
QUMLOC	156.704	2.799	QUMLOC	151.963	3.051	QUMLOC	165.001	3.176	QUMLOC	164.642	3.028	QUMLOC	158.306	3.339
QUMLUI	174.096	3.186	QUMLUI	158.442	3.481	QUMLUI	152.941	3.373	QUMLUI	158.956	3.373	QUMLUI	150.566	2.679
QUMPIB	167.448	2.789	QUMPIB	175.06	3.024	QUMLUI	164.244	2.801	QUMLUI	152.718	2.746	QUMPIB	152.831	3.498
QUNBAC	157.175	3.133	QUNBAC	150.966	3.127	QUMRAU	169.055	3.29	QUMRAU	169.877	3.465	QUMRAU	159.416	3.412
QUNKIW	165.516	2.921	QUNKIW	163.471	3.425	QUNBOQ	152.557	3.427	QUNJIT	167.902	2.899	QUNKIW	152.247	2.844
QUNKIW	173.757	2.978	QUNKOC	154.76	3.326	QUNKIW	154.765	3.162	QUNKIW	150.646	3.051	QUNKIW	169.941	2.908
QUNQOG	172.599	3.107	QUNQOG(	162.992	3.347	QUNKOC	161.184	3.357	QUNQOG	157.329	3.04	QUNQOG	162.88	3.359
QUNQUM	159.077	3.118	QUNQUM(	158.582	3.085	QUNQOG(	172.174	3.056	QUNQOG(	157.276	3.009	QUNQOJ	153.936	2.985
QUNTUO	165.193	2.73	QUNTUO	154.682	3.37	QUNTUO	164.591	2.953	QUNTUO	166.763	2.73	QUNTUO	167.704	3.214
QUNVAW	156.306	3.476	QUNWUR	152.955	2.993	QUNTUO	151.001	3.475	QUNTUO	155.607	3.299	QUNVAW	150.555	3.169
QUNWUU	162.279	3.345	QUPFAI	165.394	3.383	QUNWUR	152.943	3.392	QUNWUR	161.186	3.042	QUNWUU	154.283	3.27
QUPFAL	171.61	3.172	QUPFAL	166.281	2.91	QUPFAI	157.772	2.976	QUPFAL	162.398	3.179	QUPFAL	177.257	3.155
QUPGAJ	154.207	3.009	QUPGAJ	178.672	3.455	QUPFAL	165.128	2.827	QUPFAL	157.414	2.827	QUPGAJ	171.409	3.336
QUPKOE	150.255	3.065	QUPPUM	161.9	3.441	QUPKIW0	155.712	3.164	QUPKOE	156.414	3.315	QUPKOE	151.954	3.121
QUPWOR	152.135	2.665	QUQHAL	161.736	3.268	QUPPUM	156.84	3.068	QUPQUR	159.153	3.023	QUPWOR	151.645	2.665
QUQNEW	169.047	3.474	QUQNEW	154.73	3.178	QUQHAL	175.202	3.039	QUQHAL	176.529	3.054	QUQNEW	156.921	2.906
QUQNEW	162.509	2.766	QUQNEW	171.304	2.988	QUQNEW	155.832	3.369	QUQNEW	166.558	3.269	QUQNEW	166.341	3.442
QUQNEW	175.826	2.993	QUQNEW	150.681	2.973	QUQNEW	153.383	3.186	QUQNEW	156.699	3.241	QUQNEW	175.319	2.958
QUSBOZ	164.262	3.003	QUSBOZ	171.787	3.003	QUQNEW	158.571	3.191	QUQNEW	159.981	3.192	QUQXIK	161.48	3.142
QUSMOH	162.968	2.685	QUSSON	153.758	3.414	QUSGOE	158.457	3.315	QUSMAT	153.396	3.057	QUSMOH	167.961	3.048
QUSTIH	158.138	2.948	QUSTIH	162.373	2.879	QUSTIH	163.802	3.037	QUSTIH	164.542	3.26	QUSTIH	166.196	3.037
QUTQAZ	168.213	2.848	QUTQAZ	174.18	2.848	QUSTIH	151.456	3.252	QUTQAZ	153.812	3.347	QUTQAZ	152.072	3.008
QUTTAD	161.011	2.823	QUTTAD	160.379	3.158	QUTQED	152.708	3.345	QUTQED	168.012	2.835	QUTQED	173.258	2.835
QUVFAQ	171.429	3.439	QUVGAS	168.001	3.26	QUTTAD	163.328	2.927	QUTTIL	160.719	3.352	QUVFAQ	164.091	3.361
QUVKUR	152.039	3.444	QUVXOV	151.195	3.382	QUVGAS	164.635	3.386	QUVGAS	155.9	3.312	QUVKUR	151.593	3.392
QUWHOG	156.527	3.084	QUWHOG	164.783	3.42	QUWDOC	162.51	3.18	QUWDOC	164.26	3.189	QUWHOG	162.413	3.455
QUWJOI	154.959	3.376	QUWJUQ	157.256	3.364	QUWHOG	170.559	3.144	QUWJOI	155.057	3.008	QUWJOI	153.679	2.98
QUWNUU	169.616	3.189	QUWNUU	169.554	3.435	QUWJUQ	162.18	3.05	QUWNUU	171.836	3.435	QUWNUU	155.44	2.958
QUXKUT	150.406	2.859	QUYBUL	166.011	3.307	QUWNUU	151.582	3.212	QUWPUV	171.819	3.025	QUWZEO	153.919	2.98
QUYCAS	174.048	3.199	QUYCAS	179.721	3.177	QUYBUL	158.595	3.406	QUYBUL	167.21	3.329	QUYBUL	169.458	2.698
QUYCEW	162.316	3.189	QUYDOH	160.304	2.618	QUYCEW	178.6	3.262	QUYCEW	152.184	3.31	QUYCEW	150.289	3.31
QUYGAW	160.559	3.277	QUYGAW	163.951	2.824	QUYGAW	158.678	2.752	QUYGAW	156.157	3.486	QUYGAW	157.092	2.928
QUYGAW	156.756	2.994	QUYQEJ	152.385	2.709	QUYGAW	163.67	3.176	QUYGAW	157.648	2.98	QUYGAW	163.061	2.789
QUYQOT	150.026	2.992	QUYQOT	168.731	3.322	QUYQIN	158.224	3.149	QUYQIN	158.161	2.9	QUYQOT	169.172	3.281
QUYREK	154.055	3.044	QUYREK	151.173	3.165	QUYQUZ	160.002	3.381	QUYQUZ	164.919	3.038	QUYQUZ	156.337	2.698
QUYRIO	161.805	3.315	QUYRIO	158.991	2.736	QUYREK	169.514	3.081	QUYRIO	152.489	2.736	QUYRIO	170.73	3.315
QUYXIS	160.557	3.181	QUZBOD	175.439	2.955	QUYRIO	159.755	2.827	QUYROU	150.989	3.412	QUYROU	150.614	3.104
QUZMAB	173.728	3.365	QUZMEF	173.046	2.803	QUZKIG	151.392	2.964	QUZKIG	162.354	3.14	QUZKIJ	152.911	3.296
QUZTAH	168.323	3.006	QUZYAP	156.479	2.984	QUZNAE	175.382	3.33	QUZTAH	157.856	3.473	QUZTAH	157.182	3.489
QUZZAN	172.376	3.386	QUZZAN	150.764	3.423	QUZZAN	156.501	3.078	QUZZAN	165.009	3.468	QUZZAN	159.84	3.12
QUZZER	159.528	3.153	QUZZER	172.73	3.421	QUZZAO	154.243	2.932	QUZZER	158.241	3.095	QUZZER	165.934	3.499
RABCAU	170.536	3.16	RABDAA	160.034	3.466	QUZZER	150.427	3.475	RABCAB	157.648	3.281	RABCAU	155.047	3.285
RABLEH	155.717	3.147	RABLEH	156.365	3.147	RABDAA	157.562	2.962	RABDAA	159.004	3.124	RABDAA	158.033	3.167
RABLIL	154.467	2.857	RABLIL	154.574	2.872	RABLEH	166.722	2.935	RABLIL	153.66	3.261	RABLIL	157.163	3.392
RABXAP	150.838	3.35	RABXAP	155.362	3.088	RABNOC	175.767	2.856	RABXAP	152.956	3.465	RABXAP	156.739	3.332
RABXET	152.255	3.293	RABXET	157.818	3.301	RABXAW	153.343	2.948	RABXAW	157.887	3.342	RABXAW	152.531	3.201
RABXET	176.68	2.909	RABXET	160.242	2.753	RABXET	171.591	2.725	RABXET	161.553	3.212	RABXET	160.435	3.325
RABXIF	152.45	3.233	RABXIF01	169.644	3.26	RABXET	152.894	3.059	RABXET	156.148	2.725	RABXIF	170.407	3.191

RACHOS0	156.734	2.917	RACVAV	156.519	2.667	RACCOQ	158.477	2.947	RACCOQ	158.294	3.451	RACHOS	156.734	2.917
RADFEK	152.398	3.087	RADFEK	155.298	3.296	RACXEB	153.376	2.604	RADFEK	162.3	3.153	RADFEK	154.854	3.426
RADFUA	156.747	3.237	RADFUA	176.653	3.352	RADFEK	162.677	3.068	RADFEK	156.98	2.87	RADFUA	151.68	3.308
RADGAH	169.694	3.292	RADGAH	152.731	3.451	RADFUA	160.625	3.435	RADGAH	170.042	3.032	RADGAH	150.91	2.877
RADHUC	158.715	2.888	RADHUD	154.9	3.449	RADGAH	165.443	3.409	RADHOW	172.022	3.104	RADHOW(	171.183	3.147
RADLAK	165.358	3.105	RADLAK	168.721	3.456	RADJEO	173.586	3.043	RADLAK	177.322	2.988	RADLAK	154.05	3.191
RADPUJ	168.855	3.039	RADPUJ	160.374	3.06	RADLAK	159.688	3.041	RADLAK	163.775	3.248	RADMOC	166.487	3.251
RADSIC	151.813	2.937	RADSIC	153.433	3.265	RADPUJ	161.79	3.311	RADPUJ	165.225	3.399	RADPUJ	150.212	3.108
RADWOM	155.61	2.898	RADYOK	158.617	3.058	RADSIC	158.109	3.345	RADSUM	172.302	2.955	RADWOM	150.998	2.746
RADZAX	166.043	3.006	RADZEB	173.382	2.946	RADYOK	152.419	2.874	RADZAX	152.768	3.376	RADZAX	156.178	2.971
RADZEG	164.445	2.83	RADZEG	155.479	2.983	RADZEB	158.34	3.261	RADZEB	160.186	2.896	RADZEG	166.311	2.72
RAFCEK	160.107	3.04	RAFFAF	164.433	2.92	RADZEG	154.43	3.156	RADZOL	155.276	3.32	RADZOL	170.288	3.293
RAFPIA01	158.463	3.018	RAFWIF	161.337	3.462	RAFPIA	158.596	3.016	RAFPIA	170.365	2.8	RAFPIA01	170.433	2.801
RAFYIJ	156.115	3.334	RAFZIJ	174.354	3.014	RAFXOM	158.765	2.945	RAFXOM	155.249	2.945	RAFYEF	160.701	3.304
RAGDAH	151.869	2.837	RAGDEM	150.839	3.21	RAFZIJ	159.597	3.032	RAGCUA	155.381	2.999	RAGCUA	157.643	3.086
RAGDIP	159.501	2.889	RAGDIP	158.914	3.131	RAGDEM	151.269	3.258	RAGDIP	164.586	2.617	RAGDIP	166.232	3.123
RAGMIW	160.682	3.336	RAGPEX	171.467	3.438	RAGDIP	159.113	2.645	RAGDIP	161.225	3.277	RAGMIW	153.586	3.161
RAGQIB	152.903	3.164	RAGQIB	169.201	3.479	RAGPOH	163.469	2.992	RAGPOI	159.601	3.164	RAGQAU	164.076	3.217
RAGQUO	169.85	3.211	RAGTOJ	164.884	2.937	RAGQIC	166.907	3	RAGQIC	174.036	3.375	RAGQOI	156.006	3.086
RAGTOJ	165.704	3.401	RAGYAC	168.716	3.244	RAGTOJ	162.065	3.048	RAGTOJ	169.46	2.887	RAGTOJ	161.975	3.068
RAHRAT	163.926	3.049	RAHRAT	160.899	2.877	RAHRAT	177.375	3.205	RAHRAT	163.952	2.774	RAHRAT	168.48	3.292
RAHRAT	158.007	3.36	RAHRUR	151.077	3.477	RAHRAT	160.514	3.241	RAHRAT	154.074	3.154	RAHRAT	162.239	3.084
RAHZIJ	170.846	2.871	RAHZIJ	150.342	3.226	RAHZIJ	165.333	3.402	RAHZIJ	158.6	2.871	RAHZIJ	153.164	3.075
RAHZIJ	161.439	3.404	RAJBAF	153.801	3.077	RAHZIJ	161.962	2.815	RAHZIJ	160.21	3.315	RAHZIJ	154.506	3.063
RAJBAF	151.608	2.741	RAJBAF	156.106	2.753	RAJBAF	163.361	3.14	RAJBAF	166.771	2.864	RAJBAF	156.238	3.496
RAJBAF	171.06	2.786	RAJBAF	161.156	2.805	RAJBAF	150.332	3.374	RAJBAF	167.31	3.383	RAJBAF	178.973	3.304
RAJLIA	151.621	2.91	RAJPOJ	150.692	3.116	RAJBAF	159.808	2.982	RAJBEL	157.491	3.106	RAJHIW	152.566	3.184
RAJRED	166.208	3.025	RAKCEM	164.535	3.069	RAJPOJ	156.191	3.427	RAJPOJ	153.702	3.421	RAJPOJ	159.606	3.081
RAKCOY	172.919	3.451	RAKGUI	155.83	3.16	RAKCIS	151.13	3.116	RAKCOY	156.238	2.832	RAKCOY	152.645	3.155
RAKJUJ	155.127	3.474	RAKJUJ	173.593	3.317	RAKJIX	167.278	3.3	RAKJUJ	176.08	3.362	RAKJUJ	162.617	3.112
RAKWIM	152.555	3.406	RAKWIM	164.586	3.311	RAKJUJ	158.249	3.207	RAKNUN	153.624	3.39	RAKTIJ	175.551	2.724
RALBAL	160.016	2.956	RALJEU	160.842	3.006	RALBAL	164.547	3.141	RALBAL	154.653	3.422	RALBAL	162.725	3.075
RALJIY	152.502	3.069	RALJIY	165.542	2.712	RALJEU	167.54	3.045	RALJIY	155.301	3.24	RALJIY	163.109	3.023
RALLAU05	162.164	3.208	RALLAU05	157.469	3.216	RALJOE	172.206	2.884	RALKUO	166.348	3.389	RALLAU05	162.882	3.425
RALWUA	166.055	2.853	RALZOV	152.812	3.419	RALLAU05	150.455	3.135	RALMOH	154.735	3.398	RALTIK	155.535	2.741
RAMNEY	155.184	3.016	RAMNOI01	159.008	3.091	RAMLUN	157.786	3.324	RAMMAU	154.356	3.046	RAMMAU	153.542	2.702
RAMTEG	164.816	3.162	RAMTEG	155.18	3.124	RAMPEC	151.608	3.024	RAMTEG	154.469	3.298	RAMTEG	170.134	2.811
RANKIE	175.214	2.774	RANNID	155.449	2.765	RANJEV	165.797	3.027	RANKEY	177.279	3.393	RANKIE	165.291	3.226
RANNID	167.503	3.324	RANVIO	171.623	3.011	RANNID	150.723	3.13	RANNID	162.471	2.96	RANNID	171.827	3.298
RANVUX	150.328	3.172	RANVUX	165.715	2.982	RANVIO	158.059	3.184	RANVUX	155.317	2.948	RANVUX	164.524	2.813
RANVUX	156.126	3.315	RANVUX	150.478	2.679	RANVUX	159.905	2.961	RANVUX	158.66	2.807	RANVUX	169.417	3.171
RANYIP	168.804	3.312	RANZEN	158.087	3.047	RANVUX	160.253	3.123	RANVUX	175.995	3.397	RANVUX	168.29	3.443
RANZEN	174.21	3.121	RANZEN	152.811	2.943	RANZEN	168.17	2.922	RANZEN	156.002	2.919	RANZEN	155.267	2.871
RAPBAN	159.06	2.872	RAPBER	158.655	2.791	RAPBAN	177.313	3.062	RAPBAN	151.184	3.034	RAPBAN	155.004	3.365
RAPBOB	157.975	3.25	RAPFUN	167.299	2.936	RAPBOB	162.708	2.828	RAPBOB	161.786	2.855	RAPBOB	161.547	3.455
RAPMOM	168.057	2.846	RAPMOM	160.707	2.789	RAPMOM	157.193	3.301	RAPMOM	158.661	3.453	RAPMOM	164.451	2.841
RAPMOM	156.649	3.458	RAPMOM	160.603	3.13	RAPMOM	156.192	2.921	RAPMOM	152.605	3.458	RAPMOM	157.915	3.09
RAPNED	157.432	3.374	RAPNED	175.344	3.002	RAPMOM	155.41	3.215	RAPNED	163.178	3.369	RAPNED	163.883	2.877
RAPPIJ	176.355	3.188	RAPROQ	155.723	3.478	RAPNED	158.624	3.175	RAPNED	163.938	3.422	RAPNED	150.943	3.409
RAPTUZ	162.172	3.255	RAPTUZ	156.186	3.111	RAPROS	167.429	2.987	RAPSAD	160.534	2.813	RAPTUZ	157.19	3.275
RAPTUZ	150.916	3.162	RAPTUZ	157.749	3.49	RAPTUZ	151.159	3.355	RAPTUZ	162.616	3.204	RAPTUZ	165.112	3.046
RAPTUZ	151.021	3.444	RAPTUZ	156.037	3.281	RAPTUZ	159.069	3.213	RAPTUZ	158.352	3.182	RAPTUZ	164.472	2.94
RAPTUZ	155.207	2.83	RAPVAH	157.732	3.12	RAPTUZ	166.645	2.974	RAPTUZ	153.311	3.4	RAPTUZ	170.708	2.81
RAPVEL	153.884	2.615	RAPVEL	150.19	3.061	RAPVAH	155.276	2.801	RAPVAH	155.255	3.222	RAPVAH	173.154	3.446
RAPVIP	171.936	3.069	RAPVIP	153.083	3.232	RAPVEL	154.466	3.061	RAPVIP	157.444	3.336	RAPVIP	163.606	2.699
RAPVIP	151.552	3.298	RAPVIP	166.638	3.106	RAPVIP	156.044	3.247	RAPVIP	163.416	3.165	RAPVIP	169.014	2.881
RAPVOT	151.536	3.208	RAPVOT	154.683	2.852	RAPVOT	156.156	3.32	RAPVOT	175.759	3.383	RAPVOT	168.974	3.489
RAPWEK	169.455	3.261	RAPWEK	162.94	3.074	RAPWEK	151.97	3.254	RAPWEK	162.485	3.044	RAPWEK	160.834	2.912
RAQFAT	175.429	3.498	RAQFAT	157.239	3.103	RAQDUL	163.034	2.99	RAQFAT	175.173	3.267	RAQFAT	150.544	3.041
RAQFUO	166.084	3.164	RAQFUO	151.524	3.225	RAQFAT	171.329	3.488	RAQFIZ	155.517	3.489	RAQFIZ	153.617	3.358
RAQQAF	154.731	3.39	RAQQEJ	151.063	3.372	RAQFUO	153.596	3.225	RAQGEZ	173.241	2.856	RAQKAW	151.453	3.122
RAQWAI	158.405	2.819	RAQWAI	175.905	3.362	RAQTIN02	163.607	3.472	RAQVEL	157.233	3.001	RAQVEL	152.07	3.359
RAQWAI	153.759	2.924	RAQWAI	161.444	2.819	RAQWAI	160.524	3.083	RAQWAI	153.207	2.886	RAQWAI	152.937	3.358
RAQWAI	158.701	3.006	RAQWEL	156.533	3.476	RAQWAI	156.523	2.825	RAQWAI	163.322	3.489	RAQWAI	156.238	2.918
RAQWEM	163.688	2.945	RAQWEM	154.184	2.976	RAQWEL	160.785	3.428	RAQWEL	151.617	3.145	RAQWEL	175.468	2.858
RAQWEM	151.362	3.222	RAQWEM	160.277	3.025	RAQWEM	153.064	3.191	RAQWEM	156.959	2.714	RAQWEM	171.917	3.351
RAQXUE	167.913	2.734	RAQXUE	151.232	3.079	RAQWEM	163.892	3.488	RAQXOY	163.494	3.26	RAQXOY0	163.518	3.263
RAQYAL	172.337	3.193	RAQYEP	173.35	3.295	RAQXUE	170.826	3.205	RAQXUE	151.286	3.079	RAQYAL	176.14	3.096
RAQYOZ	156.131	2.904	RAQZUG	166.699	2.283	RAQYIT	151.161	3.153	RAQYOZ	158.841	3.162	RAQYOZ	178.516	3.494
RARCAP	155.415	3.177	RARCAP	151.269	2.972	RAQZUG	164.552	3.313	RARCAP	154.28	3.005	RARCAP	156.306	2.966
RARQUW	151.107	2.852	RARQUW	156.839	2.473	RARFUO	161.737	3.446	RARKOO	162.655	3.246	RARLAB	160.279	3.29
RARWIQ	153.135	3.033	RARWIQ	151.582	3.076	RARQUW	153.667	3.049	RARQUW	154.983	2.473	RARQUW	172.992	2.852
RASLAB	163.238	3.343	RASPOQ	168.378	3.033	RARWIQ	156.613	3.147	RASKUU	159.203	3.047	RASKUU	152.5	3.261
RASPUX	165.219	2.98	RASPUX	162.048	2.971	RASPUX	152.546	2.822	RASPUX	162.964	3.312	RASPUX	169.092	2.953
RASPUX	157.175	3.346	RASPUX	170.283	2.872	RASPUX	175.851	3.222	RASPUX	162.243	2.794	RASPUX	176.714	2.794
RASPUX	167.911	2.954	RASPUX	171.746	2.926	RASPUX	152.034	3.31	RASPUX	170.267	2.953	RASPUX	169.17	2.98
RASQAE	161.52	2.653	RASQAE	157.627	2.807	RASPUX	166.403	3.325	RASPUX	156.206	3.312	RASPUX	164.113	2.771
RASQAE	163.124	2.942	RASQAE	150.139	3.031	RASQAE	154.627	3.17	RASQAE	157.047	3.45	RASQAE	153.635	2.803
RASQAE	168.936	3.375	RASQEI	174.361	3.275	RASQAE	170.171	3.496	RASQAE	166.054	3.283	RASQAE	158.332	2.64

RASQEI	163.637	3.037	RASQEI	152.332	2.964	RASQEI	163.852	3.321	RASQEI	158.449	3.373	RASQEI	161.868	2.858
RASQEI	156.985	3.011	RASQEI	159.665	2.844	RASQEI	172.649	2.777	RASQEI	172.63	2.967	RASQEI	156.801	2.824
RASQIM	151.473	2.942	RASQIM	152.103	3.108	RASQEI	164.659	3.306	RASQEI	150.108	3.215	RASQIM	154.351	2.93
RASRAF	151.993	3.221	RASREJ	173.338	3.154	RASQIM	162.057	3.193	RASRAF	156.412	2.833	RASRAF	171.337	2.67
RASREJ	164.258	2.731	RASREJ	150.995	2.965	RASREJ	161.179	3.068	RASREJ	168.792	2.961	RASREJ	163.843	3.205
RASREL	150.357	3.146	RASREL	154.301	3.039	RASREJ	158.412	3.316	RASREL	157.483	3.493	RASREL	154.144	3.447
RASRIN	150.246	3.003	RASRIN	153.942	3.016	RASREL	155.69	2.978	RASREL	155.398	3.155	RASREL	152.874	3.144
RASRIN	163.53	3.47	RASRIN	165.371	2.791	RASRIN	162.394	3.403	RASRIN	157.854	2.898	RASRIN	160.44	2.946
RASRIN	150.117	3.47	RASRIN	151.896	3.117	RASRIN	156.663	3.142	RASRIN	158.078	2.98	RASRIN	165.322	3.183
RASRIN	156.068	2.915	RASRIN	162.293	3.455	RASRIN	154.832	2.919	RASRIN	161.866	2.682	RASRIN	171.518	2.977
RASRIP01	168.609	2.849	RASRIP01	160.158	2.705	RASRIP01	163.105	2.849	RASRIP01	152.704	3.488	RASRIP01	158.353	2.705
RASRUZ	162.448	3.354	RASRUZ	167.389	3.181	RASRUZ	151.687	2.854	RASRUZ	165.014	3.313	RASRUZ	168.801	2.921
RASRUZ	168.977	3.072	RASRUZ	164.984	3.205	RASRUZ	168.641	2.807	RASRUZ	175.704	3.124	RASRUZ	166.918	3.009
RASRUZ	153.312	2.753	RASRUZ	163.633	3.143	RASRUZ	154.232	3.251	RASRUZ	168.983	3.115	RASRUZ	161.937	2.783
RASRUZ	168.543	2.897	RASRUZ	150.465	3.485	RASRUZ	151.601	2.893	RASRUZ	154.709	3.412	RASRUZ	156.452	3.42
RASRUZ	153.645	2.857	RASRUZ	171.933	3.488	RASRUZ	167.067	2.794	RASRUZ	152.254	3.33	RASRUZ	172.268	3.084
RATSUE	158.169	3.22	RAVBAU	170.133	2.72	RATHEC	151.808	2.88	RATLOQ	161.192	3.35	RATLOQ	159.02	3.179
RAVBAU	167.461	3.241	RAVBAU	171.821	3.24	RAVBAU	167.078	3.241	RAVBAU	174.121	3.323	RAVBAU	163.398	3.367
RAVBUO	156.027	2.947	RAVBUO	167.106	2.931	RAVBEY	153.704	3.259	RAVBEY	158.069	3.068	RAVBUO	157.351	3.004
RAVBUO	178.065	2.911	RAVBUO	152.925	3.072	RAVBUO	164.886	3.212	RAVBUO	160.652	2.892	RAVBUO	159.856	2.931
RAVJUX	157.884	3.432	RAVJUX	157.524	3.085	RAVBUO	164.741	3.066	RAVBUO	153.551	3.137	RAVBUO	170.325	3.066
RAVMAF0	161.782	3.416	RAVRUC	162.347	2.733	RAVJUX	169.671	3.297	RAVJUX	165.224	2.923	RAVMAF	152.718	3.357
RAWTIS	153.285	3.135	RAWTIS	157.093	2.836	RAWREQ	152.66	3.082	RAWTIS	156.265	3.112	RAWTIS	166.401	2.87
RAWXUI	169.029	2.926	RAWXUI	154.602	3.412	RAWXUI	163.475	2.971	RAWXUI	155.198	3.324	RAWXUI	158.245	3.307
RAXFII	159.217	3.352	RAXFUS	174.997	3.415	RAXBOK	164.658	2.976	RAXBOK	171.98	3.308	RAXDUS	153.253	3.304
RAXQOZ	157.841	3.262	RAXQOZ	157.465	2.83	RAXFUT	162.39	2.948	RAXGEF	152.856	2.877	RAXLUB	152.417	3.146
RAXQOZ	152.816	3.347	RAXQUF	168.318	3.402	RAXQOZ	172.725	3.444	RAXQOZ	153.785	3.46	RAXQOZ	151.128	2.915
RAXQUF	162.928	2.791	RAXRUG	165.162	3.123	RAXQUF	169.153	3.35	RAXQUF	169.761	3.029	RAXQUF	173.956	3.065
RAXSIW	152.275	3.32	RAXSIW	157.715	3.39	RAXSES	150.436	3.197	RAXSIW	156.963	3.457	RAXSIW	168.782	2.816
RAXSOB	174.051	3.034	RAXWUL	157.122	3.418	RAXSIW	160.155	3.241	RAXSIW	165.253	2.957	RAXSIW	156.983	3.345
RAYBEB	153.888	2.941	RAYGUU	153.33	2.633	RAXWUL	157.159	3.342	RAXZOI	156.263	3.003	RAYBAX	177.132	3.046
RAYGUU	164.023	3.212	RAYGUU	153.299	3.333	RAYGUU	153.235	3.333	RAYGUU	157.445	2.865	RAYGUU	151.803	2.952
RAYHAB	152.586	3.454	RAYHAB	173.305	2.94	RAYGUU	154.327	3.403	RAYGUU	171.541	2.633	RAYHAB	152.46	3.126
RAYHAB	171.561	2.973	RAYHAB	151.221	2.938	RAYHAB	159.057	2.964	RAYHAB	155.445	2.963	RAYHAB	163.064	2.938
RAYJUZ	154.362	3.03	RAYJUZ	171.912	2.924	RAYHAB	178.213	2.856	RAYHIK	166.21	3.355	RAYJEK	150.052	2.986
RAYVAO	156.917	2.707	RAYVAO	157.625	2.707	RAYMEM	162.609	3.211	RAYMEM	154.404	3.211	RAYMEM	161.509	3.431
RAZZAW	151.123	3.239	REBFIP	157.878	3.417	RAZTAR	158.704	2.962	RAZTAR	157.65	3.457	RAZYAV	151.118	3.022
REBWOL	160.964	3.379	REBYIK	151.855	3.075	REBFIP	154.855	3.161	REBFIP	153.241	2.956	REBJAK	152.488	2.866
RECFOX	152.335	2.902	RECFOX	151.1	2.852	RECBAF	150.25	3.271	RECFOX	152.049	2.848	RECFOX	152.551	2.891
RECPAQ	155.685	2.895	RECPAQ0	158.099	2.803	RECJIT	151.692	3.346	RECNET	175.426	3.035	RECPAQ	158.099	2.803
REDGAL	150.808	3.315	REDGAL	162.942	3.236	RECPAQ0	155.685	2.895	RECRUM	154.888	2.771	RECRUM	161.19	2.771
REDGAL	163.992	3.181	REDGAL	157.723	3.463	REDGAL	158.055	2.964	REDGAL	170.217	3.385	REDGAL	177.553	3.416
REFCEK	168.886	2.777	REFCEK0	168.886	2.777	REDGAL	157.005	3.202	REDVOP	170.387	3.242	REDVUV	152.9	3.481
REFCEO	157.75	2.948	REFCEO	172.651	3.418	REFCEO	175.219	2.97	REFCEO	158.462	2.98	REFCEO	173.632	2.998
REFCEO	157.411	3.114	REFCEO	159.652	3.277	REFCEO	155.322	3.463	REFCEO	168.156	2.82	REFCEO	173.279	2.777
REFLOD	155.404	3.062	REFLOD0	163.347	2.957	REFCEO	169.337	2.699	REFCIS	167.104	2.954	REFCIS	162.015	3.127
REFRIH	169.784	2.696	REFRIH	153.272	2.911	REFLOD0	162.285	3.166	REFLOD0	168.138	2.975	REFLUJ	165.593	3.067
REFRIH	164.3	3.482	REFRIH	173.188	3.35	REFRIH	153.184	3.112	REFRIH	168.276	3.229	REFRIH	154.442	2.994
REFRIH	157.849	3.221	REFRIH	164.727	2.766	REFRIH	155.304	2.751	REFRIH	151.241	3.455	REFRIH	163.457	3.455
REFVEH	166.071	3.378	REGGIU	167.158	2.837	REFRUS	155.335	3.309	REFRUS	164.65	2.997	REFRUS	154.313	3.308
REGGIU	155.934	2.726	REGGIU	173.753	3.016	REGGIU	173.857	3.17	REGGIU	167.346	3.339	REGGIU	173.809	2.803
REGGIU	155.773	2.743	REGGIU	151.542	3.203	REGGIU	158.324	3.18	REGGIU	164.039	3.098	REGGIU	163.429	2.726
REGGIU	159.738	3.002	REGGIU	158.041	2.889	REGGIU	150.772	3.302	REGGIU	150.849	3.108	REGGIU	156.028	3.03
REHPOJ	152.817	3.474	REHPOJ	161.68	3.444	REGLAS	159.576	3.02	REGXEJ	155.889	3.311	REGXIN	155.377	3.321
REHSUV	159.971	3.32	REHTAC	163.891	3.275	REHPOJ	153.479	2.939	REHPOJ	156.123	2.939	REHSUV	158.717	2.633
REHVEI	153.718	2.77	REHVEI	154.175	3.272	REHTAC	163.098	3.436	REHTUW	169.546	3.106	REHVEI	166.786	2.921
REHYOS	153.051	2.855	REHYOS	160.361	3.354	REHVEI	159.336	3.494	REHYOS	151.23	3.148	REHYOS	154.943	3.124
REJQAC	166.711	3.239	REJTIL	156.353	3	REHYOS	153.523	2.856	REHYOS	173.34	2.966	REHZOT	154.964	3.153
REKJAU	156.678	2.872	REKJAU	150.829	3.275	REJWIQ	151.132	3.384	REKJAU	169.212	3.057	REKJAU	151.352	3.064
REKJEY	159.857	2.999	REKJEY	167.528	3.158	REKJAU	151.374	3.251	REKJAU	160.996	3.405	REKJEY	155.142	3.222
REKJEY	162.954	3.359	REKJEY	161.535	2.875	REKJEY	170.284	3.178	REKJEY	150.663	3.178	REKJEY	155.584	2.999
REKJOH	155.068	3.214	REKJOH	159.169	3.334	REKJEY	150.818	3.429	REKJOH	168.173	3.388	REKJOH	152.632	2.922
REKJOH	162.894	3.113	REKJOH	154.692	3.15	REKJOH	167.742	3.308	REKJOH	163.844	3.104	REKJOH	152.603	3.129
REKJOH	150.747	3.067	REKJOH	153.59	3.283	REKJOH	163.519	3.293	REKJOH	156.382	3.447	REKJOH	164.694	3.109
REKJOH	154.057	3.186	REKJOH	158.984	3.118	REKJOH	158.386	3.214	REKJOH	164.63	3.016	REKJOH	171.902	3.484
REKJOH	150.431	3.353	REKJOH	154.584	3.23	REKJOH	163.402	3.129	REKJOH	165.639	3.281	REKJOH	161.511	3.371
REKJOH0	156.041	3.107	REKJOH0	161.205	3.098	REKJOH	151.095	3.275	REKJOH	158.481	3.388	REKJOH	159.583	2.994
REKJOH0	156.114	3.353	REKJOH0	163.38	3.452	REKJOH0	168.021	3.188	REKJOH0	168.938	3.199	REKJOH0	151.128	3.187
REKJOH0	166.031	3.169	REKJOH0	164.248	3.043	REKJOH0	151.173	3.098	REKJOH0	151.741	3.174	REKJOH0	150.586	3.282
REKJOI	165.836	3.258	REKJOI	173.059	3.352	REKJOH0	161.903	3.451	REKJOI	159.572	3.186	REKJOI	168.824	3.313
REKJOJ	172.169	3.069	REKJOJ	171.939	3.071	REKJOI	163.525	3.349	REKJOI	158.349	2.802	REKJOI	150.066	2.665
REKJOJ	172.962	3.12	REKJOJ	170.81	3.056	REKJOJ	152.864	3.424	REKJOJ	157.243	3.042	REKJOJ	160.731	3.306
REKJOJ	169.21	3.2	REKJUN	169.567	3.059	REKJOJ	160.428	3.042	REKJOJ	156.174	3.306	REKJOJ	170.215	3.022
REKKEZ	168.962	2.798	REKKEZ	155.879	2.931	REKKEZ	161.161	3.46	REKKEZ	161.997	2.913	REKKEZ	168.529	2.883
REKKID	170.498	3.062	REKKID	160.991	2.871	REKKEZ	158.578	2.865	REKKID	161.853	2.645	REKKID	162.811	2.876
REKXIP	160.954	2.794	REKXIP	160.173	2.89	REKKID	170.356	2.929	REKKID	154.105	3.455	REKKID	153.488	2.871
RELKAX	151.464	3.198	RELKAX	178.187	2.962	REKXIP	156.645	2.995	REKXIP	156.977	2.957	RELKAX	173.196	3.103
RELTOU	153.117	2.95	REMCIY	158.29	3.302	RELKAX	154.231	3.121	RELLIE	162.62	3.48	RELQEH	150.347	3.158
REMKUQ	154.759	3.356	REMKUQ	161.932	3.213	REMGIB	151.13	2.97	REMKUQ	154.704	3.471	REMKUQ	158.725	3.209

REMVAH	160.384	3.083	RENBOD	162.19	3.333	REMKUQ	152.225	2.917	REMMUT	157.897	3.251	REMMUT0	158.041	3.198
RENSEJ01	164.409	3.328	RENSEJ01	151.575	3.394	RENBOD	155.617	3.278	RENQEG	159.35	3.023	RENRIN	159.486	3.26
RENZIV	166.615	3.305	RENZIV	153.105	3.305	RENSEJ01	164.305	2.943	RENSEJ01	156.404	3.155	RENZIV	171.9	2.667
RENZIV	157.352	3.129	RENZOB	152.944	3.326	RENZIV	167.7	3.087	RENZIV	160.353	3.306	RENZIV	162.218	3.139
RENZOB	171.862	3.003	RENZOB	155.964	2.92	RENZOB	160.473	2.986	RENZOB	161.855	2.948	RENZOB	168.753	3.261
RENZUH	167.144	3.083	RENZUH	161.012	2.983	RENZUH	158.501	2.9	RENZUH	164.103	3.248	RENZUH	169.969	3.314
RENZUH	161.96	2.792	RENZUH	162.402	2.85	RENZUH	166.862	3.199	RENZUH	160.169	2.741	RENZUH	155.531	3.218
RENZUH	152.363	3.29	RENZUH	151.761	2.817	RENZUH	173.747	2.704	RENZUH	155.222	2.764	RENZUH	171.803	2.85
RENZUH	168.048	2.705	REPBOF	151.321	3.322	RENZUH	165.199	3.108	RENZUH	153.77	3.056	RENZUH	163.566	3.037
REPBOF	156.593	3.044	REPBOF	177.021	2.753	REPBOF	164.746	3.446	REPBOF	154.985	3.205	REPBOF	159.357	2.996
REPBOF	171.961	2.876	REPBOF	168.764	3.231	REPBOF	176.089	2.921	REPBOF	159.791	2.83	REPBOF	159.976	2.979
REPBOF	155.3	3.198	REPBOF	154.819	3.476	REPBOF	166.232	3.027	REPBOF	154.876	2.829	REPBOF	164.906	3.273
REPBOF	150.189	3.116	REPBOF	167.296	3.254	REPBOF	165.9	3.418	REPBOF	166.264	3.246	REPBOF	155.966	2.83
REPHEB	154.256	3.145	REPKAZ	167.065	3.165	REPBOF	165.713	3.305	REPFEB	150.672	2.92	REPFEB	157.618	3.398
REPKAZ	171.15	3.134	REPKAZ	155.906	2.975	REPKAZ	161.343	3.284	REPKAZ	152.572	2.941	REPKAZ	176.532	3.23
REPKAZ	168.29	3.023	REPKAZ	154.445	2.966	REPKAZ	152.276	2.98	REPKAZ	153.578	2.99	REPKAZ	163.297	3.026
REPKAZ	160.491	2.851	REPKED	155.159	3.111	REPKAZ	172.55	3.274	REPKAZ	154.754	2.941	REPKAZ	160.807	3.48
REPKED	151.502	3.348	REPKED	150.159	3.35	REPKED	155.595	3.449	REPKED	152.334	2.978	REPKED	151.312	2.898
REPMEI	160.561	2.899	REPMEI	173.723	3.322	REPKED	154.445	3.014	REPKED	162.34	3.01	REPMEH	155.371	2.958
REPNIL	155.425	3.12	REPNIL	160.435	2.972	REPMEI	176.539	3.13	REPMEI	153.094	3.123	REPNIL	176.091	3.007
REPNIL	172.11	3.465	REPNIL	159.071	3.12	REPNIL	157.71	3.272	REPNIL	151.923	3.465	REPNIL	153.729	3.271
REQSIQ	152.214	2.902	REQSIQ	169.079	3.155	REQCIZ	172.347	3.158	REQCIZ	150.015	3.158	REQCUL	165.645	3.172
REQSIQ	152.688	3.211	REQSIQ	151.904	3.291	REQSIQ	169.154	3.038	REQSIQ	165.169	3.226	REQSIQ	161.924	3.288
REQSOW	162.341	3.098	REQSOW	151.117	3.309	REQSIQ	176.071	2.826	REQSIQ	167.584	2.902	REQSOW	158.888	2.957
RERCAV	150.506	2.953	RERCAV	167.768	3.494	REQTUG	160.92	3.112	RERCAV	164.936	3.471	RERCAV	156.659	3.242
RERCAV	154.539	3.385	RERNOU	150.539	3.15	RERCAV	152.209	2.981	RERCAV	160.273	3.21	RERCAV	154.012	2.952
RERSOY	151.905	2.96	RERSOY	153.585	3.29	RERSOY	157.993	3.326	RERSOY	164.006	3.216	RERSOY	172.761	3.075
RERSUE	157.609	3.347	RERSUE	155.44	2.974	RERSOY	159.148	3.227	RERSOY	162.381	3.148	RERSOY	166.495	3.146
RERSUE	163.838	2.921	RERVER	154.533	2.737	RERSUE	153.38	3.498	RERSUE	167.742	2.971	RERSUE	151.696	3.329
RERVER	164.654	3.448	RERYOG	153.038	3.387	RERVER	159.207	2.937	RERVER	163.628	3.294	RERVER	162.805	2.977
RESHUV	174.193	3.052	RESHUV	173.903	3.207	RESHUV	159.409	3.252	RESHUV	153.534	3.366	RESHUV	177.49	3.135
RESJOP	155.123	3.15	RESMIP	173.005	3.389	RESHUV	155.131	3.115	RESHUV	165.148	3.208	RESJIJ	158.153	3.083
RESVIV	153.624	3.46	RESVIV	153.445	3.04	RESMIP	162.541	3.305	RESPOY	170.238	3.141	RESVIV	165.513	2.893
RESVIV	179.318	2.856	RESVIV	154.044	3.478	RESVIV	166.434	3.463	RESVIV	152.65	2.986	RESVIV	156.136	3.42
RESVIV	156.867	2.965	RESVIV	162.986	3.187	RESVIV	161.311	2.926	RESVIV	157.312	3.059	RESVIV	152.348	2.993
RESWAO	160.477	3.425	RESWAO	176.48	3.482	RESVIV	150.736	2.988	RESVIV	159.401	3.264	RESVIV	155.774	3.104
RESWAO	162.507	3.035	RESWAO	151.98	3.142	RESWAO	163.974	3.151	RESWAO	150.498	2.713	RESWAO	168.636	2.976
RESWAO	153.19	3.308	RESWAO	160.339	3.089	RESWAO	151.246	3.311	RESWAO	163.849	3.067	RESWAO	158.798	2.95
RESZUO	163.913	2.877	RESZUO	153.497	2.966	RESWAO	156.642	3.251	RESWAO	163.673	2.79	RESWAO	161.369	3.066
RETCID	167.722	3.238	RETCID	155.262	3.3	RESZUO	153.036	3.457	RETCID	169.694	2.834	RETCID	162.474	3.424
RETPIT	163.891	2.875	RETPIT	154.134	2.972	RETHUW	158.658	3.378	RETLIP	173.584	2.983	RETNAJ	160.118	3.464
REVCIH	162.574	3.293	REVDEB	166.375	2.975	RETPIT	153.238	3.452	RETTEP	152.17	2.779	RETTEP	150.865	2.873
REVMEL	153.219	3.142	REVMEL0	159.042	3.195	REVDEC	162.736	2.894	REVHAB	172.982	3.367	REVMEL	157.77	3.166
REVMEL0	153.279	3.195	REVMEL0	157.765	3.177	REVMEL0	152.397	3.173	REVMEL0	157.98	3.172	REVMEL0	152.926	3.145
REVWAT	158.703	2.738	REVWAT	153.348	2.951	REVPOY	159.878	3.397	REVQUF	156.308	3.432	REVSOB0	157.011	2.991
REVXIC	163.619	3.419	REVXIC	159.471	2.885	REVWAT	172.561	2.915	REVWEX	158.937	3.258	REVXIC	163.517	3.133
REVXUO	163.515	3.088	REVYOJ	157.985	3.112	REVXIC	167.952	2.949	REVXUO	163.664	2.813	REVXUO	168.455	3.046
REWCII	169.152	2.932	REWCII	160.303	2.916	REVYOJ	153.003	3.195	REVYOJ	150.48	3.382	REWCII	164.169	3.301
REWCII	170.254	3.265	REWCII	175.652	2.917	REWCII	159.04	3.275	REWCII	173.714	3.072	REWCII	151.816	2.927
REWCII	161.372	2.927	REWCOO	152.818	2.904	REWCII	150.387	3.335	REWCII	161.742	3.002	REWCII	178.825	3.434
REWKUD	150.443	3.431	REWQEQ	168.314	3.251	REWKAG	154.218	2.764	REWKUD	158.15	3.021	REWKUD	156.34	3.094
REWQUH	164.104	2.938	REWQUH	150.753	3.051	REWQUH	159.741	3.025	REWQUH	159.236	3.079	REWQUH	160.378	3.049
REWYEY	159.231	3.479	REWYEY	164.285	3.161	REWQUH	172.969	3.284	REWWAS	156.482	3.284	REWYEY	163.435	2.682
REWZEZ	161.548	3.301	REWZEZ	172.522	3.048	REWZEZ	160.256	3.194	REWZEZ	171.003	3.048	REWZEZ	159.145	2.82
REWZID	172.769	3.098	REWZID	172.59	3.098	REWZID	153.899	2.664	REWZID	172.076	3.008	REWZID	170.629	3.014
REWZOM	157.435	3.272	REWZOM	161.749	3.201	REWZID	164.76	2.881	REWZID	161.949	3.092	REWZOJ	152.476	2.766
REXBAB	164.291	3.498	REXBAB	160.052	3.139	REWZOM	166.1	3.192	REWZUQ	150.82	3.008	REXBAB	159.052	3.262
REXGUY	163.626	2.832	REXGUY	164.997	2.832	REXBAB	167.508	3.145	REXBAB	153.052	2.881	REXGUY	155.315	3.196
REXTOE	156.494	3.206	REXTOE	160.402	2.78	REXMOY	163.168	3.054	REXNAL	159.167	2.839	REXNEP	166.984	2.878
REYFUX	167.917	3.204	REYJAK	158.393	3.389	REXWEA	155.7	2.733	REYBOQ	151.444	3.492	REYDAC	164.089	3.142
REZBIH	151.266	2.863	REZBIH	152.654	3.408	REYYAW	161.667	2.911	REYZAZ	160.844	2.912	REYZAZ	156.969	2.953
REZDEG	158.003	3.241	REZGEK	157.546	3.035	REZCEI	161.218	3.051	REZCIM	154.578	3.178	REZDEG	158.264	3.241
REZNIX	154.843	3.172	REZNIX	162.644	3.357	REZHIP	160.253	3.272	REZNIX	152.88	3.098	REZNIX	151.102	3.185
REZREW	156.86	3.058	REZREW	154.086	3.097	REZNIX	171.896	3.305	REZREW	159.578	3.037	REZREW	152.172	3.024
RIBGIU	172.614	3.199	RIBGIU	164.153	3.393	REZZAB	156.922	2.758	REZZAB	156.905	2.758	RIBGIU	177.969	3.313
RIBTAX	157.903	3.111	RIBTAX	173.853	3.231	RIBJIW	151.872	3.189	RIBSUR	151.72	3.362	RIBTAX	165.305	3.245
RIBTIF	153.932	3.155	RIBTIF	155.446	3.318	RIBTAX	172.139	3.142	RIBTAX	168.392	3.002	RIBTAX	164.759	2.849
RIBTIF	154.671	2.931	RIBTIF	173.641	3.073	RIBTIF	162.015	3.432	RIBTIF	170.093	3.036	RIBTIF	150.994	3.044
RIBTIF	166.787	2.846	RIBTUR	157.533	3.241	RIBTIF	165.148	3.29	RIBTIF	165.242	2.84	RIBTIF	164.359	3.4
RIBTUR	151.17	2.943	RIBVON	159.463	2.879	RIBTUR	154.443	3.279	RIBTUR	161.109	3.189	RIBTUR	150.281	3.226
RICCOY	159.537	3.234	RICDEP	173.793	3.232	RIBVON	162.802	3.457	RIBVON	159.235	2.868	RICCOY	171.014	3.311
RICDEP01	152.502	3.217	RICDEP01	154.76	3.197	RICDEP	152.71	3.193	RICDEP	154.39	3.189	RICDEP01	173.674	3.244
RICDEP03	173.458	3.272	RICDEP03	152.006	3.271	RICDEP02	173.6	3.258	RICDEP02	152.237	3.243	RICDEP02	155.203	3.207
RICDEP04	156.194	3.228	RICDEP05	173.156	3.303	RICDEP03	155.637	3.218	RICDEP04	173.299	3.288	RICDEP04	151.617	3.3
RICDEP06	150.719	3.365	RICDEP06	157.541	3.251	RICDEP05	151.251	3.331	RICDEP05	156.77	3.24	RICDEP06	172.963	3.319
RICDEP08	171.841	3.385	RICDEP08	161.248	3.303	RICDEP07	172.69	3.335	RICDEP07	150.041	3.401	RICDEP07	158.43	3.263
RIDCOY	150.627	3.232	RIDFIU	170.041	3.46	RICDEP09	171.989	3.411	RICDEP09	163.97	3.29	RICVAE	170.777	3.352
RIDPID	155.771	2.855	RIDPID	150.698	2.852	RIDMUN	174.696	3.354	RIDMUN	156.724	3.043	RIDMUN	162.508	3.057
RIDZAF	174.549	3.239	RIFBIV	155.883	3.122	RIDPID02	154.782	2.805	RIDQAW	163.71	3.469	RIDQAW	159.274	2.913

RIFFAQ	168.703	2.852	RIFGEV	167.129	2.767	RIFBIV	170.301	3.277	RIFBIV	162.23	3.277	RIFBIV	163.103	2.939
RIGMOL06	157.493	3.246	RIGMOL07	157.128	3.257	RIFKAV	156.963	2.983	RIGDUI	156.152	3.177	RIGMOL04	157.951	3.221
RIHCOD	167.51	3.08	RIHCOD	160.327	3.289	RIGSEF	167.042	3.303	RIGSEF	152.621	3.303	RIGVEJ	150.88	3.191
RIHCOD	173.915	3.031	RIHCOD	156.069	3.209	RIHCOD	167.08	3.259	RIHCOD	155.987	3.49	RIHCOD	168.288	2.998
RIHCOD	153.267	3.067	RIHCOD	166.075	3.08	RIHCOD	154.392	2.86	RIHCOD	154.953	2.709	RIHCOD	162.582	3.407
RIHCOD	173.287	3.209	RIHCOD	153.98	2.717	RIHCOD	171.992	2.717	RIHCOD	157.164	3.122	RIHCOD	171.602	3.383
RIHCOD	157.138	3.219	RIHCUJ	158.138	2.772	RIHCOD	153.731	2.795	RIHCOD	168.313	3.324	RIHCOD	163.302	3.067
RIHCUJ	151.337	2.772	RIHGAR	154.347	3.162	RIHCUJ	166.926	3.314	RIHCUJ	153.493	2.961	RIHCUJ	157.153	3.223
RIHGAR	162.137	3.448	RIHGAR	163.755	3.352	RIHGAR	151.18	3.147	RIHGAR	152.404	3.413	RIHGAR	162.898	2.921
RIHGAR	154.721	3.044	RIHLOL	154.725	3.148	RIHGAR	155.634	2.944	RIHGAR	154.714	2.921	RIHGAR	150.337	3.068
RIHNAB	157.777	3.368	RIHPOR	151.441	3.203	RIHLUR	162.578	2.998	RIHMAY	155.818	3.08	RIHMAY	173.436	3.01
RIHTIN	150.13	3.274	RIHYEO	160.463	2.848	RIHPOR	173.244	3.255	RIHPOR	165.156	3.45	RIHTIN	167.712	3.237
RIJDIB	163.035	2.966	RIJDIB	152.103	3.007	RIHYEO	161.836	3.11	RIHYEO	155.438	3.35	RIJDIB	154.964	3.093
RIJDIB	172.591	3.358	RIJDIB	159.418	2.809	RIJDIB	153.114	2.955	RIJDIB	175.05	3.374	RIJDIB	157.633	3.442
RIJDUM	158.354	3.262	RIJDUM	150.938	2.813	RIJDIB	160.08	3.39	RIJDIB	151.212	3.035	RIJDIB	162.563	2.982
RIJHOJ	168.347	3.074	RIJHOJ	166.891	3.074	RIJDUM	158.788	3.047	RIJDUM	154.675	3.309	RIJDUM	151.647	3.2
RIJMEE	154.643	3.162	RIJMEG	153.184	2.942	RIJMAA	159.752	3.168	RIJMAA01	159.411	3.187	RIJMEE	154.94	3.294
RIJWAM	165.122	3.209	RIJWUE	158.763	3.411	RIJMEG	157.489	2.942	RIJMOO	169.406	2.907	RIJQAG	161.398	3.008
RIJWUE	159.655	3.158	RIJXIT	155.404	3.456	RIJWUE	156.044	3.149	RIJWUE	153.132	3.354	RIJWUE	162.317	2.755
RIKBES	155.456	2.872	RIKCEX	165.321	3.358	RIJXIT	165.004	2.997	RIJXIT	170.744	3.097	RIJXIT	156.662	3.247
RIKKIJ	161.81	2.514	RIKKUV	153.287	2.871	RIKJIE	163.138	3.22	RIKJIE	173.478	3.447	RIKKIJ	165.122	2.514
RIKKUV01	154.97	2.946	RIKNAA	167.709	3.301	RIKKUV	157.882	3.251	RIKKUV	167.677	2.927	RIKKUV	165.521	2.89
RIKXEO	167.295	2.972	RIKXEO	158.034	3.05	RIKNAA	163.336	3.353	RIKXEO	171.274	2.928	RIKXEO	172.857	2.798
RIKXIS	159.49	3.364	RILCEY	152.933	3.359	RIKXEO	157.993	3.216	RIKXIS	155.889	3.405	RIKXIS	161.14	3.238
RILKIJ	150.722	3.234	RILKIJ	159.647	2.782	RILCEY	152.315	3.382	RILCIC	168.753	3.494	RILKIJ	151.975	3.308
RILLAD	152.685	3.077	RILLIJ	158.76	2.902	RILKIJ	158.258	3.075	RILLAD	153.096	2.934	RILLAD	160.597	3.431
RILMAD	155.303	3.237	RILMAD	158.46	2.711	RILLIJ	159.304	3.197	RILMAD	174.363	3.372	RILMAD	172.53	2.813
RILPAG	175.605	3.156	RILPAG	162.834	2.973	RILMAD	164.728	2.93	RILMAD	153.328	2.66	RILNAD	152.279	2.902
RILQIP	164.551	3.355	RILQIP	166.972	2.892	RILPEK	160.277	2.902	RILPEK	173.668	3.159	RILPEK	156.075	2.784
RILQOV	170.856	3.267	RILQOV	171.005	3.213	RILQIP	159.034	3.026	RILQIP	172.273	2.923	RILQIP	159.473	3.076
RILQOV	152.746	3.157	RILQOV	157.08	3.321	RILQOV	151.704	3.234	RILQOV	163.482	3.028	RILQOV	156.687	3.28
RILQUB	156.533	3.097	RILQUB	164.359	3.19	RILQUB	155.396	3.314	RILQUB	164.318	2.998	RILQUB	160.119	3.252
RILQUB	152.658	3.31	RILQUB	160.761	3.097	RILQUB	152.792	3.426	RILQUB	156.086	2.969	RILQUB	153.692	3.459
RILRAI	170.291	3.356	RILRAI	158.868	3.028	RILQUB	155.56	3.19	RILQUB	153.602	3.438	RILRAI	152.614	2.997
RILREM	166.489	3.282	RILREM	162.56	2.804	RILRAI	153.015	3.449	RILRAI	176.225	2.606	RILRAI	156.347	2.975
RILTOZ	154.908	2.984	RILTOZ	162.774	3.079	RILREM	171.956	3.283	RILREM	167.596	3.364	RILREM	155.735	3.077
RILTOZ	166.984	3.018	RILTOZ	154.221	2.95	RILTOZ	175.035	2.914	RILTOZ	157.526	2.914	RILTOZ	171.604	2.95
RILVER	152.459	3.492	RILVER	150.544	2.852	RILVAN	165.7	2.794	RILVAN	154.594	3.354	RILVAN	165.776	2.794
RILVIV	174.838	3.302	RILVIV	161.564	3.07	RILVER	160.235	3.264	RILVER	173.44	2.713	RILVER	174.025	3.128
RILVOB	163.513	2.799	RILVUG	152.17	3.282	RILVOB	152.215	3.303	RILVOB	152.304	3.429	RILVOB	159.118	2.848
RIMMUZ	152.744	3.207	RIMMUZ	164.194	3.185	RIMMUZ	163.418	3.207	RIMMUZ	152.449	2.974	RIMMUZ	173.316	2.974
RINHOP	150.885	2.885	RINHOP	166.515	3.281	RINHOP	168.021	3.095	RINHOP	170.582	3.356	RINHOP	172.879	3.463
RINJIL	158.465	2.753	RINKAE	155.802	2.892	RINJIL	171.472	3.159	RINJIL	172.467	3.16	RINJIL	154.416	3.233
RINNOV	151.982	3.023	RINNOV	151.907	2.756	RINKAE	163.244	3.084	RINKAE	158.776	3.087	RINNOV	163.179	3.089
RINNUB	175.883	3.49	RINNUB	159.865	3.166	RINNOV	161.137	3.448	RINNUB	152.601	3.173	RINNUB	150.814	3.309
RINQUB	158.382	3.383	RINQUB	153.728	3.019	RINNUB	152.725	3.066	RINNUB	163.01	2.996	RINQOV	159.136	2.796
RINSAK	166.428	2.819	RINVAL	172.279	3.341	RINQUB	163.497	3.019	RINSAK	153.665	3.393	RINSAK	162.684	3.294
RINVAO	168.132	3.026	RINVEQ	151.317	3.454	RINVAL	172.145	3.359	RINVAL	170.667	3.338	RINVAM	169.858	3.368
RINXAR	163.549	3.082	RIPDOJ	151.452	2.911	RINXAR	166.798	3.152	RINXAR	160.561	3.083	RINXAR	157.357	3.101
RIPQUE	160.323	3.259	RIPQUE	172.387	2.902	RIPDOJ	152.643	2.976	RIPQUE	152.007	3.213	RIPQUE	172.272	2.863
RIQCAX	170.374	3.13	RIQKAF	156.196	3.196	RIPWOG	153.202	2.917	RIQBID	156.37	3.448	RIQBID	164.206	3.287
RIQPUE	161.925	3.47	RIRFED	168.846	3.067	RIQKAF	161.07	3.411	RIQKAF	171.002	3.49	RIQKAF	153.58	3.376
RIRJAG	171.962	2.992	RIRJAG	162.905	3.403	RIRFED	152.872	2.96	RIRFED	165.053	3.131	RIRFED	157.09	3.067
RIRJIO	173.427	3.314	RIRJOU	165.242	2.953	RIRJAG	168.623	2.939	RIRJAG	174.107	3.123	RIRJAG	166.563	2.814
RIRKOV02	169.084	2.999	RIRKOV03	169.66	3.091	RIRJOU	152.111	2.991	RIRKOV	167.373	2.881	RIRKOV01	168.182	2.934
RIRMAJ	164.081	3.221	RIRMEN	165.414	3.217	RIRKUY	177.757	3.204	RIRKUY	154.097	3.324	RIRKUY	154.327	2.931
RIRSET	170.922	3.142	RIRSET	156.701	3.273	RIRMIR	153.711	3.239	RIRQEQ	164.241	3.198	RIRQEQ	153.658	3.193
RIRSOD	172.378	2.736	RIRSUJ	159.17	3.067	RIRSIX	153.354	2.962	RIRSIX	152.317	2.958	RIRSOD	171.902	2.733
RIRTAQ	159.533	2.802	RIRTEU	165.012	3.436	RIRSUJ	160.927	3.028	RIRTAQ	159.505	2.713	RIRTAQ	165.987	2.713
RIRTEU	169.905	3.281	RIRTOE	161.717	2.81	RIRTEU	152.95	2.869	RIRTEU	150.888	2.827	RIRTEU	155.112	3.315
RIRTOE	174.381	3.229	RIRTOE	168.244	3.283	RIRTOE	168.477	2.915	RIRTOE	164.866	3.066	RIRTOE	156.882	3.284
RIRTUK	157.246	3.111	RIRTUK	151.304	2.993	RIRTOE	163.755	3.119	RIRTUK	161.871	2.902	RIRTUK	164.941	3.159
RIRVAS	171.927	3.009	RIRVAS	174.58	3.153	RIRTUK	158.778	3.091	RIRTUK	166.67	3.15	RIRVAS	165.874	3.033
RIRVIA	156.408	3.254	RIRVIA	158.009	3.254	RIRVAS	168.199	3.038	RIRVEW	173.275	2.712	RIRVEW	171.481	2.724
RISWEY	168.211	3.061	RISWEY	175.633	3.116	RISCAB	167.162	3.113	RISMAH	152.392	2.973	RISMEN	153.89	3.111
RITFAD	154.735	3.433	RITFOT	154.06	3.114	RISWID	151.297	3.398	RISYOK	176.705	3.331	RISYUQ	173.995	3.304
RITJAH	163.049	3.102	RITKEK	151.309	3.228	RITFOT	155.151	3.114	RITFOT	160.604	3.255	RITJAH	152.974	2.697
RITNOY02	169.836	2.883	RITNOY02	176.307	3.057	RITNOY02	155.614	3.327	RITNOY02	156.247	3.197	RITNOY02	152.303	2.874
RITPOD	152.672	2.698	RITPOD	162.382	2.739	RITPOD	156.714	2.939	RITPOD	160.012	3.361	RITPOD	154.987	2.94
RITSIZ	160.594	3.296	RITSIZ	173.689	3.058	RITSEV	171.062	3.122	RITSEV	161.725	3.317	RITSIZ	165.618	3.399
RITSIZ	172.037	2.685	RITSIZ	161.273	3.057	RITSIZ	151.16	2.929	RITSIZ	151.794	3.069	RITSIZ	162.079	3.175
RITSIZ	167.059	3.438	RITSOF	172.775	3.433	RITSIZ	150.652	3.076	RITSIZ	164.596	3.058	RITSIZ	169.18	2.685
RITYEC	161.899	3.261	RIVBOO	156.245	3.049	RITSOF	155.847	3.082	RITSOF	152.933	2.978	RITYEC	156.793	2.88
RIVTIA	167.215	3.407	RIVWID	156.093	3.058	RIVGEJ	152.353	3.396	RIVGEJ	161.375	3.191	RIVGEJ	164.079	3.347
RIVYAA	165.281	3.258	RIVYEE	162.815	3.443	RIVXIE	155.515	3.473	RIVXIE	150.229	2.742	RIVXIE	156.439	3.426
RIWSAU	164.228	3.23	RIWSAU	153.034	2.83	RIWCUV	175.68	3.494	RIWRUN	166.016	3.49	RIWRUN	153.033	3.256
RIWZON	163.75	2.971	RIWZON	157.833	3.12	RIWTIE	169.637	2.902	RIWTIE	157.005	3.429	RIWTIE	158.474	3.341
RIXCOR	152.591	2.676	RIXCOR	156.111	2.698	RIXCOR	159.472	3.388	RIXCOR	152.491	3.356	RIXCOR	154.941	2.893
RIXDAE	170.916	3.279	RIXDAE	153.286	2.954	RIXCOR	155.896	2.852	RIXCOR	159.955	2.844	RIXDAE	153.099	2.935

RIXDAE	172.671	2.814	RIXDAE	164.533	3.41	RIXDAE	160.107	2.769	RIXDAE	167.594	2.743	RIXDAE	164.321	3.251
RIXDEI	152.432	3.36	RIXDEI	154.43	2.91	RIXDAE	172.656	2.924	RIXDAE	152.356	3.125	RIXDEI	158.551	3.4
RIXDEI	156.138	2.835	RIXMOC	156.659	3.354	RIXDEI	152.736	2.68	RIXDEI	155.107	2.696	RIXDEI	160.609	2.86
RIXMUK	150.575	3.213	RIXNUL	169.016	3.477	RIXMOC	159.695	3.087	RIXMOC	153.725	3.107	RIXMOC	165.149	3.25
RIXREY	162.055	2.704	RIXREY	167.578	3.225	RIXPEX	172.791	3.483	RIXPIB	159.417	2.996	RIXREY	162.76	3.106
RIXREY	155.747	3.492	RIXREY	156.142	3.02	RIXREY	160.932	2.771	RIXREY	165.993	2.616	RIXREY	156.447	2.704
RIXREY	159.962	3.057	RIXROI	178.247	3.36	RIXREY	162.036	2.856	RIXREY	155.071	3.006	RIXREY	153.367	2.947
RIXYUS	171.215	3.155	RIXYUS	163.392	2.659	RIXROI	160.174	2.866	RIXROI	158.95	2.865	RIXYUS	164.639	2.903
RIXYUS	164.819	2.696	RIYCOV	150.737	3	RIXYUS	157.924	3.339	RIXYUS	157.646	2.746	RIXYUS	161.833	2.963
RIYWON	158.213	3.284	RIYWON	154.314	3.335	RIYFEL	172.396	3.102	RIYRUQ	176.194	3.088	RIYTIF	152.021	3.36
RIZHAJ	175.013	3.395	RIZHAJ	168.036	3.395	RIYWON	163.342	3.473	RIYWON	157.087	3.349	RIZBOT	155.97	3.158
RIZNIB	168.06	3.154	RIZNIB	177.206	3.214	RIZKER	151.282	3.116	RIZNIB	162.563	3.341	RIZNIB	169.461	3.081
RIZTIF	166.554	3.311	RIZTIF	162.096	3.311	RIZNIB	169.306	3.435	RIZNOF	162.774	3.471	RIZSOI	156.156	3.172
RIZYEF	156.095	3.152	RIZYEF	150.225	2.856	RIZYEF	157.775	3.384	RIZYEF	152.74	2.856	RIZYEF	162.196	2.741
ROBDES	160.643	3.186	ROBDIW	166.558	3.071	RIZYIJ	154.225	3.048	RIZYIJ	152.368	3.035	RIZYIJ	152.893	2.85
ROBJEZ	169.999	2.954	ROBPEE	153.093	2.853	ROBDIW	158.505	2.804	ROBJAV	159.06	3.007	ROBJEZ	163.187	2.922
ROBPOO	160.165	3.498	ROBPUU	156.887	3.167	ROBPII	178.182	3.34	ROBPOO	157.747	3.34	ROBPOO	153.558	3.254
ROCBUH	157.868	3.372	ROCCOB	150.004	3.191	ROBPUU	150.381	3.095	ROBTUZ	165.105	3.457	ROBTUZ	170.185	3.258
RODBAR	174.503	2.587	RODBIX	151.424	3.482	ROCCUH	166.186	3.196	ROCRUA	153.132	3.153	RODBAR	153.194	3.039
RODFAT	151.431	2.983	RODFAT	167.761	2.827	RODBIX	165.776	3.025	RODFAT	172.314	3.212	RODFAT	153.447	2.785
RODFAT	162.979	3.272	RODFAT	159.476	3.437	RODFAT	150.319	3.262	RODFAT	151.945	2.833	RODFAT	154.444	3.059
RODGIC	171.024	3.221	RODGIC	178.17	2.889	RODFEX	158.058	2.948	RODFEX	167.792	2.882	RODGEY	160.984	2.809
RODMII	167.572	2.845	RODMII	166.556	2.845	RODGIC	150.052	3.229	RODGIC	151.026	3.211	RODMAA	152.827	3.251
RODPAE	158.458	3.369	RODPIL	166.274	3.285	RODPAE	150.281	3.346	RODPAE	153.558	3.049	RODPAE	158.46	3.069
RODZUI	151.452	2.808	ROFCAT	155.999	3.225	RODPIL	175.023	3.29	RODYAO	164.985	3.177	RODZAN	153.699	3.343
ROFKUW	165.191	2.925	ROFKUW	156.202	2.996	ROFGUO	167.99	3.225	ROFKUW	160.696	2.852	ROFKUW	158.794	3.035
ROFPIL	153.731	3.23	ROFPIL	150.11	3.23	ROFKUW	157.325	2.864	ROFKUW	159.271	2.922	ROFKUW	160.676	3.04
ROFREN	153.414	2.999	ROFREN	167.292	3.125	ROFPIL	159.084	3.275	ROFRAJ	157.612	3.325	ROFREN	151.538	3.107
ROFRUB	162.532	3.405	ROFSAI	165.997	3.018	ROFROX	159.585	2.906	ROFRUB	169.756	3.161	ROFRUB	173.803	2.934
ROFSEM	161.521	3.034	ROFSIQ	158.013	3.288	ROFSEM	152.89	2.995	ROFSEM	151.598	2.997	ROFSEM	161.627	2.819
ROFSIQ	165.976	3.078	ROFVUF	161.96	2.961	ROFSIQ	154.815	3.131	ROFSIQ	171.101	2.819	ROFSIQ	158.075	3.023
ROFWOA	155.412	2.717	ROFWOA	157.138	3.261	ROFVUF	165.069	2.841	ROFWOA	162.875	2.899	ROFWOA	161.53	2.899
ROGNEH	158.536	3.025	ROGQEM	166.764	2.981	ROFWOA	156.205	2.993	ROFZAQ	165.253	3.098	ROGNEH	154.574	2.953
ROGQIQ	153.148	2.838	ROGQIQ	152.735	2.838	ROGQEM	164.332	3.104	ROGQEM	162.99	2.937	ROGQEM	166.147	2.978
ROGTOX0	161.514	3.206	ROGTOX0	154.888	3.022	ROGRAK	163.345	3.473	ROGTOX0	155.392	3.247	ROGTOX0	177.792	3.015
ROHPEM	155.401	2.623	ROHPIQ	155.28	3.465	ROGXIV	151.837	3.1	ROHJUU	168.87	3.183	ROHJUU	167.723	3.183
ROHSOX	159.194	3.334	ROHTOZ0	168.523	3.009	ROHPIQ	171.151	3.129	ROHPIQ	165.701	2.948	ROHSOX	151.443	3.269
ROJGEF	152.746	2.914	ROJNUZ	167.877	3.071	ROHXUH	150.041	3.315	ROHYOC	157.562	2.895	ROHYOC	155.578	2.933
ROKFUU	152.497	3.496	ROKGAD	159.09	3.308	ROJPAH	161.452	3.368	ROJZIZ	158.319	2.933	ROKFUU	164.661	3.374
ROKQUF0	164.611	3.487	ROKTIW	151.806	3.496	ROKQUF	164.224	2.699	ROKQUF0	157.686	2.635	ROKQUF0	163.905	2.69
ROKTIW	162.51	3.293	ROLBAW	166.771	3.157	ROKTIW	162.185	3.006	ROKTIW	156.178	3.109	ROKTIW	159.158	3.477
ROLFAD	170.96	3.138	ROLHUZ	168.488	3.297	ROLBAW	155.489	3.36	ROLBAW	170.322	2.974	ROLFAD	158.388	3.23
ROLLUB	168.318	3.267	ROLMOU	163.362	3.471	ROLHUZ	170.501	3.297	ROLHUZ	159.234	3.055	ROLLUB	157.23	3.401
ROLWEW	154.678	3.265	ROMDUV	171.856	2.848	ROLMOU	151.408	3.158	ROLWAS	154.752	2.876	ROLWAS	164.218	3.055
RONPOD	163.96	2.937	ROPQEU	158.531	3.052	ROMJIM	168.634	3.001	ROMJIM	163.4	3.387	ROMMEP	158.086	2.815
ROPRIZ	158.698	3.056	ROPROF	166.018	3.482	ROPQEU	170.843	3.491	ROPREW	163.577	3.153	ROPRIZ	167.902	3.491
ROQHAJ	150.687	3.406	ROQHAJ	160.516	3.115	ROPROF	156.093	3.007	ROPTAV	150.61	3.342	ROPYIH	171.566	3.01
RORCUZ	158.556	2.891	RORCUZ	151.828	3	ROQKIV	174.994	3.038	ROQKIV	169.786	3.494	ROQKIV	166.407	3.096
RORXUU	157.129	3.347	RORXUU	155.625	3.38	RORWIG	174.351	3.417	RORXII	150.553	3.147	RORXOO	160.208	3
ROSZOQ	153.238	2.866	ROSZUW	160.592	2.914	RORYIJ	150.008	3.269	ROSLAP	160.795	2.9	ROSZOQ	151.32	2.796
ROTBAF	155.457	3.371	ROTBEJ	152.587	2.931	ROSZUW	167.99	3.489	ROSZUW	167.749	2.853	ROTBAF	167.102	2.895
ROTLAP	172.494	2.721	ROTLAP	172.344	2.721	ROTBIN	173.581	3.189	ROTBIN	169.296	3.48	ROTBIN	169.659	2.91
ROTMAQ	169.721	2.93	ROTMAQ	154.328	3.142	ROTLUJ	162.769	2.684	ROTLUJ	163.863	3.394	ROTMAQ	155.43	3.409
ROTMAQ	159.944	2.752	ROTMEU	172.568	2.823	ROTMAQ	156.969	2.725	ROTMAQ	156.771	2.796	ROTMAQ	165.437	3.409
ROVKUK	173.326	3.251	ROVKUK	158.65	3.065	ROTMEU	164.107	3.003	ROTMEU	153.757	3.106	ROVGEQ	155.044	3.128
ROVLAR0	158.2	3.022	ROVLAR0	158.841	2.974	ROVKUK	175.022	2.803	ROVLAR	153.857	2.998	ROVLAR0	163.234	3.279
ROWCIR	155.585	2.992	ROWCIR	166.242	3.224	ROWCIR	164.303	2.729	ROWCIR	160.168	3.038	ROWCIR	175.941	3.499
ROWCIR	163.294	3.162	ROWCIR	171.747	3.131	ROWCIR	156.528	2.729	ROWCIR	168.064	2.882	ROWCIR	168.174	3.373
ROWCUE	160.679	3.133	ROWDAL	162.114	3.064	ROWCIR	150.719	3.369	ROWCIR	162.75	2.958	ROWCUE	165.961	3.27
ROWZIO	172.761	3.088	ROWZIP	175.467	3.359	ROWDAL	163.546	3.185	ROWMOI	162.454	3.114	ROWMOI	164.546	3.238
ROXMAU	161.812	3.239	ROXVUX	165.488	3.336	ROXBUF	163.811	2.667	ROXGES	156.004	3.252	ROXGES	152.302	3.374
ROXWAE	161.787	3.475	ROYCEP	159.767	3.214	ROXVUX	166.113	2.801	ROXWAE	159.5	3.264	ROXWAE	173.263	3.475
ROYRAC	172.264	3.409	ROYRIJ	160.622	2.971	ROYCEP	166.637	3.214	ROYRAB	163.656	2.831	ROYRAB	166.719	3.464
ROYVUA	160.188	2.814	ROYVUA	158.601	2.669	ROYVUA	167.727	2.894	ROYVUA	155.767	3.304	ROYVUA	157.089	2.862
ROZWOW	168.323	2.902	ROZZIS	163.345	3.427	ROYVUA	161.058	3.313	ROZHIA	160.728	3.084	ROZHIA	165.21	2.93
RUBCAU	158.205	3.344	RUBGED	154.47	3.057	RTFBCP1(	155.869	3.477	RUBBIC	158.521	2.68	RUBCAU	156.107	3.011
RUBYOD	157.734	3.138	RUBYOD	158.254	2.972	RUBGED	159.125	2.908	RUBNUZ	150.098	3.391	RUBYOD	158.653	3.059
RUCPAJ	152.84	3.352	RUCPAJ	157.196	3.353	RUBYUJ	156.938	3.461	RUCBUQ	166.597	3.139	RUCBUQ	158.388	3.45
RUCRAM	160.446	3.051	RUCRAM	167.594	3.042	RUCQIT	165.527	3.192	RUCRAM	151.102	2.852	RUCRAM	161.016	2.918
RUDDAX	173.413	2.884	RUDMEL	156.463	2.945	RUCTOB	150.754	2.741	RUDDAX	158.582	2.613	RUDDAX	152.406	3.071
RUDSUG	166.356	3.263	RUDSUG	151.884	3.415	RUDMEL	150.708	3.095	RUDMEL	152.867	3.261	RUDQAJ0	162.392	2.965
RUDXOH	160.68	3.275	RUDXOH	170.518	3.473	RUDSUG	174.009	3.404	RUDXEW	151.907	3.111	RUDXOG	150.429	3.266
RUFBUT	153.899	2.896	RUFPEQ	176.34	3.196	RUDXOH	176.068	3.391	RUDXOH	167.071	3.055	RUFBUT	173.778	3.306
RUFWAQ	166.596	3.174	RUFWAQ	156.966	2.859	RUFWAQ	151.791	3.152	RUFWAQ	164.68	2.939	RUFWAQ	162.443	3.096
RUFWEU	161.266	2.906	RUFWEU	154.159	2.975	RUFWEU	163.371	2.735	RUFWEU	170.627	2.735	RUFWEU	151.238	3.285
RUGGIM	174.622	3.273	RUGGIM	152.804	3.152	RUFWEU	151.255	2.899	RUGGIM	151.426	3.423	RUGGIM	157.627	3.481
RUGNOY	166.842	2.977	RUGNOY	157.014	2.889	RUGKEK	157.953	3.002	RUGNOY	158.301	2.889	RUGNOY	166.92	2.977
RUGZIE	158.743	3.013	RUGZIE	168.125	3.499	RUGPUG	157.96	3.239	RUGQAM	162.635	3.261	RUGYOK	166.724	3.495
RUHJEN	165.645	3.36	RUHPAL	170.027	3.293	RUGZIE	163.88	3.276	RUGZIE	159.057	3.436	RUGZIE	160.709	3.309

RUHRIZ	168.219	3.067	RUHSAS	160.426	3.486	RUHPIT	158.315	2.884	RUHPIT	167.003	3.028	RUHRIZ	155.03	2.866
RUHXUP	154.548	3.017	RUHXUP	169.269	2.838	RUHXUP	153.85	3.415	RUHXUP	157.555	3.228	RUHXUP	173.74	3.344
RUHXUP	169.041	2.737	RUHXUP	151.787	2.638	RUHXUP	156.543	3.26	RUHXUP	159.997	3.415	RUHXUP	154.9	3.472
RUHYEA	156.776	3.413	RUHYEA	163.974	3.075	RUHYEA	155.565	3.413	RUHYEA	150.884	3.104	RUHYEA	170.516	2.929
RUHYOK	165.581	2.974	RUJFEJ	163.808	3.431	RUHYEZ	168.163	3.073	RUHYOK	165.625	3.488	RUHYOK	156.703	3.223
RUJHIP	150.968	2.838	RUJHIP	160.903	3.131	RUJHIP	150.707	2.915	RUJHIP	158.581	3.425	RUJHIP	150.4	2.988
RUJLOA	151.744	3.29	RUJLOA	152.187	3.26	RUJHIP	157.999	3.067	RUJHIP	159.503	2.915	RUJHIP	164.008	3.26
RUJTEY	172.621	2.899	RUJTEY	163.091	3.087	RUJPAO	150.258	3.428	RUJTEY	151.494	3.234	RUJTEY	158.107	3.366
RUJWAX	154.192	2.657	RUJWAX	177.669	2.657	RUJVEA	153.053	3.499	RUJVUQ	163.301	2.98	RUJVUQ	151.68	2.965
RUKCEH	150.658	3.433	RUKCIL	153.812	3.018	RUJWIF	167.263	2.661	RUJWIF	156.247	3.358	RUJWIF	152.529	2.863
RUKCUV	152.424	3.065	RUKCUV	169.245	2.86	RUKCUV	150.482	3.492	RUKCUV	156.834	2.978	RUKCUV	161.338	3.19
RUKCUV	173.312	3.264	RUKCUV	171.145	3.286	RUKCUV	150.506	3.264	RUKCUV	160.567	3.19	RUKCUV	158.007	2.877
RUKHAH	157.075	3.242	RUKHAH	157.834	2.898	RUKCUV	167.354	3.489	RUKCUV	159.388	3.489	RUKCUV	169.846	3.399
RUKTAW	170.799	3.319	RUKTAW	151.957	3.307	RUKJOZ	151.018	3.095	RUKJOZ	153.441	2.858	RUKTAW	158.185	3.088
RULRAV	162.16	3.254	RULXAZ	157.658	2.959	RULQUO	163.147	3.27	RULQUO	161.131	3.267	RULRAV	163.787	3.261
RULXAZ	157.027	3.154	RULXAZ	159.178	3.192	RULXAZ	157.513	3.275	RULXAZ	176.995	3.2	RULXAZ	156.363	2.959
RULXAZ	160.273	2.858	RULXIH	170.288	2.692	RULXAZ	162.142	3.11	RULXAZ	161.437	3.105	RULXAZ	165.567	2.67
RULXON	153.55	2.886	RULXON	155.36	3.438	RULXIH	152.284	3.329	RULXIH	154.777	3.043	RULXIH	173.71	2.969
RUMFEL	162.687	3.013	RUMFEL	153.129	2.782	RULXON	163.519	3.458	RULXON	169.395	3.18	RUMFEL	159.338	2.692
RUMMOD	167.116	3.208	RUMMUJ	155.862	2.911	RUMMOD	174.37	3.495	RUMMOD	158.157	2.967	RUMMOD	159.057	2.925
RUMPOH	154.621	3.046	RUMPOH	151.812	3.307	RUMMUJ	155.989	3.245	RUMMUJ	156.517	2.94	RUMPOH	162.906	2.855
RUMPOH	152.734	2.971	RUNHOY	162.044	3.255	RUMPOH	158.584	3.477	RUMPOH	163.693	3.254	RUMPOH	170.763	3.317
RUNMOF	159.37	3.335	RUNMOF	162.373	3.239	RUNKUI	160.889	3.44	RUNLAP	151.193	3.496	RUNLAP	155.704	2.664
RUNMOF	156.017	3.131	RUNMUJ	167.554	3.356	RUNMOF	152.766	3.205	RUNMOF	155.858	3.359	RUNMOF	157.733	2.956
RUNNUK	159.202	3.078	RUNROI	151.283	3.087	RUNNOE	165.347	2.967	RUNNOE	151.051	2.838	RUNNOE	164.236	3.109
RUNTIE	163.441	3.271	RUNVON	166.897	2.789	RUNTAZ	168.699	3.477	RUNTAZ	153.885	2.981	RUNTAZ	169.106	3.477
RUPKAR	155.107	2.787	RUPSAX	175.245	3.115	RUNVON	160.77	3.435	RUNXAB	160.791	3.174	RUPJOE	160.913	3.429
RUPSEB	161.052	3.479	RUPSEB	156.181	3.049	RUPSAX	154.988	3.452	RUPSAX	153.576	2.946	RUPSEB	150.695	3.001
RUPYEG	154.585	3.066	RUQKEV	157.132	3.025	RUPSEB	156.422	3.068	RUPSOL	156.026	3.342	RUPSUR	150.608	3.086
RUQKEV	160.657	2.799	RUQKEV	163.964	3.084	RUQKEV	152.898	2.799	RUQKEV	155.653	3.466	RUQKEV	170.867	3.03
RUQMEY	160.308	3.157	RUQNOI	159.85	2.888	RUQKEV	164.807	2.786	RUQKEV	153.669	3.03	RUQMEY	152.288	2.959
RUQNOI	164.266	2.951	RUQNOI	158.723	2.749	RUQNOI	161.457	2.888	RUQNOI	167.866	2.704	RUQNOI	151.256	3.056
RUQNOI	154.731	3.135	RUQNOI	152.243	2.916	RUQNOI	170.048	3.124	RUQNOI	150.69	3.099	RUQNOI	163.757	2.897
RUQNOI	157.023	3.11	RUQNOI	150.108	3.135	RUQNOI	161.27	3.392	RUQNOI	171.913	3.339	RUQNOI	164.529	2.718
RUQPEA	158.19	3.16	RUQPEA	151.687	3.111	RUQNOI	161.458	2.626	RUQNOI	164.192	2.847	RUQPAW	152.753	3.08
RUQPEA	167.636	3.478	RUQTEC	153.828	3.182	RUQPEA	152.503	2.914	RUQPEA	161.484	2.76	RUQPEA	152.375	2.695
RUQXIL	177.461	3.033	RURBIQ	156.14	2.936	RUQTEC	155.127	3.448	RUQTEC	152.434	3.448	RUQVUY	163.169	3.005
RURFAL	163.897	3.472	RURFAL	158.851	2.842	RURBIQ	151.17	2.92	RURFAL	171.786	2.829	RURFAL	153.702	3.474
RURFIT	156.96	3.231	RURFIT	155.191	3.277	RURFAL	159.25	3.445	RURFAL	179.27	3.35	RURFIT	154.456	3.123
RURKEU	171.028	2.878	RURKEU	160.86	3.024	RURFIT	174.383	3.411	RURKEU	153.535	2.924	RURKEU	157.422	2.805
RURKEU	164.538	3.117	RURKEU	157.078	3.149	RURKEU	157.953	3.301	RURKEU	161.237	3.289	RURKEU	158.439	2.993
RURKEU	155.034	2.767	RURSIJ	167.439	2.897	RURKEU	168.428	2.929	RURKEU	163.796	3.117	RURKEU	154.434	2.922
RURVEF	164.394	3.463	RURVEF	164.526	3.455	RURTAZ	153.817	2.649	RURVAB	173.841	3.146	RURVAB	156.579	3.133
RURWAC	155.828	3.08	RURWOS	161.241	3.332	RURVEF	162.261	3.178	RURWAC	178.302	3.276	RURWAC	150.985	3.204
RURZEL	158.553	2.957	RURZEL	159.174	3.406	RURWOS	152.525	3.173	RURXAF	165.171	3.492	RURZEL	150.12	3.045
RUSJIC	173.175	2.842	RUSJIC	176.721	3.386	RUSCEQ	154.542	2.898	RUSDUI	157.588	3.112	RUSJIC	171.983	2.842
RUSLAT	167.414	3.276	RUSPAW	157.779	3.286	RUSJIC	160.937	2.709	RUSJIC	162.963	2.814	RUSJIC	165.853	2.673
RUSPAW	173.936	3.443	RUSPAW	170.891	3.152	RUSPAW	157.072	3.242	RUSPAW	160.544	3.072	RUSPAW	164.741	3.183
RUTCIV	153.653	2.884	RUTCIV	166.241	3.36	RUSPOO	150.86	3.207	RUSWUA	157.305	3.408	RUTCIV	154.356	2.995
RUTJEV	151.322	3.285	RUTJID	153.092	2.901	RUTCIV	152.261	3.368	RUTCIV	152.37	3.408	RUTCIV	173.751	3.413
RUTYEN	156.748	3.317	RUTZAK	166.756	2.87	RUTPUU	153.043	3.13	RUTWIM	153.722	2.988	RUTWIM	150.137	3.166
RUVKUQ	164.01	2.959	RUVLIE	174.375	2.72	RUVGIB	173.252	3.266	RUVJEB	155.919	3.233	RUVJEB	178.124	3.111
RUVYIQ	154.567	3.159	RUVYIQ	156.761	3.188	RUVNIJ	168.547	3.09	RUVYIQ	155.833	3.091	RUVYIQ	160.923	3.091
RUVYIQ01	155.392	3.182	RUVYIQ01	154.588	3.107	RUVYIQ	158.846	3.286	RUVYIQ01	156.577	3.204	RUVYIQ01	159.4	3.286
RUWXIS	154.136	3.077	RUWXIS	164.759	2.737	RUVYIQ01	161.283	3.107	RUWVEN	163.154	3.113	RUWXIS	150.72	3.047
RUWYIR0	159.291	3.403	RUXJIE	168.589	3.08	RUWXIS	176.77	2.737	RUWXIS	170.693	3.435	RUWXOY	155.251	2.982
RUXPUZ	154.203	2.949	RUXPUZ	153.192	2.982	RUXJIE	171.643	3.38	RUXJIE	168.107	3.201	RUXKEA	172.38	3.07
RUXPUZ	171.757	3.088	RUXPUZ	152.131	3.084	RUXPUZ	172.604	3.122	RUXPUZ	153.819	3.035	RUXPUZ	151.617	2.982
RUXPUZ0	151.798	2.855	RUXPUZ0	172.105	3.005	RUXPUZ	152.835	2.949	RUXPUZ	171.839	3.107	RUXPUZ0	152.813	2.831
RUXPUZ0	151.321	2.957	RUXPUZ0	151.48	2.831	RUXPUZ0	152.606	2.918	RUXPUZ0	150.53	2.855	RUXPUZ0	174.453	3.036
RUXPUZ0	173.311	3.063	RUXPUZ0	152.881	2.973	RUXPUZ0	171.018	2.989	RUXPUZ0	153.317	2.885	RUXPUZ0	152.564	2.908
RUXPUZ0	151.855	2.885	RUXPUZ0	172.013	3.046	RUXPUZ0	150.983	2.908	RUXPUZ0	172.871	3.048	RUXPUZ0	151.561	3.015
RUXPUZ0	152.611	2.919	RUXPUZ0	150.445	2.852	RUXPUZ0	152.805	2.832	RUXPUZ0	151.765	2.852	RUXPUZ0	172.123	3.012
RUXPUZ0	170.982	2.995	RUXPUZ0	153.341	2.885	RUXPUZ0	174.592	3.043	RUXPUZ0	151.342	2.954	RUXPUZ0	151.436	2.832
RUXPUZ0	150.81	2.905	RUXPUZ0	172.741	3.057	RUXPUZ0	152.484	2.905	RUXPUZ0	173.219	3.07	RUXPUZ0	152.949	2.973
RUXQAE	159.143	3.075	RUXZAN	158.193	3.097	RUXPUZ0	151.517	3.01	RUXPUZ0	151.873	2.885	RUXPUZ0	171.963	3.051
RUXZOB	165.62	3.187	RUXZOB	165.582	3.477	RUXZAN0	158.575	3.192	RUXZER	173.349	2.693	RUXZER0	173.568	2.748
RUYBEU	163.222	3.233	RUYBEU	165.682	3.112	RUXZUH	172.781	2.976	RUYBAQ	168.133	2.895	RUYBAQ	158.883	2.781
RUYBUK	156.826	3.298	RUYCAR	169.368	2.826	RUYBEU	161.181	3.035	RUYBOE	165.193	2.75	RUYBOE	174.681	2.824
RUYFEY	158.321	3.085	RUYHID	176.598	2.937	RUYCAR0	169.273	2.832	RUYCOF	172.955	3.328	RUYCOF0	173.073	3.366
RUYRIQ	160.316	3.141	RUYRIQ	156.541	2.736	RUYHID01	176.724	2.925	RUYHID02	176.689	2.926	RUYQOU	166.378	3.426
RUYRIQ	167.082	2.745	RUYRIQ	152.912	2.745	RUYRIQ	150.572	2.957	RUYRIQ	155.235	2.83	RUYRIQ	154.88	3.015
RUZBEU	166.486	2.918	RUZBEU	152.605	3.39	RUYRIQ	153.224	2.821	RUYRIQ	153.547	3.351	RUYRIQ	159.251	3.375
RUZBIX	153.168	3.105	RUZQEM	161.712	3.328	RUZBEU	172.113	2.967	RUZBEU	162.917	3.441	RUZBIX	157.227	3.105
RUZQUZ	170.632	3.416	RUZQUZ	152.055	2.878	RUZQEM	156.93	3.008	RUZQUZ	166.552	3.154	RUZQUZ	167.835	3.121
RUZRAG	170.959	3.424	RUZRAG	152.714	2.884	RUZQUZ	150.379	2.983	RUZRAG	166.616	3.164	RUZRAG	167.953	3.132
RUZREK	151.91	2.874	RUZREK	150.031	2.983	RUZRAG	150.8	2.999	RUZREK	166.408	3.157	RUZREK	167.964	3.133
RUZROU	171.356	3.424	RUZROU	152.965	2.888	RUZREK	170.418	3.418	RUZROU	166.518	3.154	RUZROU	168.16	3.101
RUZSIS	162.843	3.202	RUZSIS	155.624	3.354	RUZROU	151.144	3	RUZSIS	158.791	3.182	RUZSIS	150.632	3.38

RUZTAI	169.787	3.427	RUZTAI	151.043	2.867	RUZSIS	157.856	3.44	RUZTAI	166.311	3.187	RUZTAI	167.611	3.156
RUZVER	167.49	2.856	RUZVER	163.26	3.368	RUZVAN	171.042	2.813	RUZVAN	166.672	2.813	RUZVAN	161.168	3.223
RUZVOB	170.347	3.241	RUZVOB	157.718	3.39	RUZVER	162.948	2.764	RUZVER	152.165	3.173	RUZVER	168.005	2.856
RUZWUI	161.879	3.434	RUZWUI	167.667	3.158	RUZWER	155.973	3.118	RUZWUI	163.532	3.007	RUZWUI	160.329	2.862
RUZXAP	155.765	3.28	RUZXET	163.15	3.071	RUZWUI	171.709	3.089	RUZWUI	155.213	2.908	RUZWUI	165.13	2.988
RUZXUJ	156.074	2.793	RUZXUJ	152.874	3.107	RUZXUG	166.14	3.225	RUZXUJ	165.223	2.813	RUZXUJ	158.193	2.793
SABCUU	168.064	3.378	SABCUU	157.473	2.908	SABBUU	154.394	3.327	SABBUU	158.426	3.05	SABCUU	156.668	3.378
SABFIO	162.49	3.162	SABFIO	171.336	2.645	SABFIO	156.375	3.143	SABFIO	176.651	2.645	SABFIO	153.056	3.355
SABFIO	152.482	2.998	SABFIO	160.316	3.173	SABFIO	156.238	2.868	SABFIO	150.894	3.121	SABFIO	151.221	3.134
SABJUC	175.778	3.348	SABMOA	152.311	3.437	SABFIO	160.257	2.868	SABJAH	159.524	2.833	SABJAH	161.784	3.462
SABNIV	150.516	3.491	SABNIV	167.783	3.124	SABMOA	155.453	3.45	SABNAM	151.511	2.955	SABNIV	167.802	3.491
SACCAE	154.593	2.89	SACQIA	150.492	3.2	SABTIA	158.486	3.015	SABTOF	167.405	3.486	SACBAE	161.151	3.462
SACTAV	165.714	3.188	SACTID	155.708	2.71	SACQOC	157.057	3.04	SACQOG	166.162	3.229	SACSID	155.951	2.685
SACTUP	163.314	3.216	SACTUP	159.748	2.852	SACTOJ	154.711	3.334	SACTUP	169.483	2.819	SACTUP	153.624	2.963
SADCEJ	150.844	3.098	SADCEJ	173.752	3.419	SACVEB	152.75	2.66	SACXED	159.485	3.014	SACXED	165.317	2.967
SADNUH	155.274	3.121	SADNUH	175.064	3.464	SADCEJ	173.711	3.419	SADCEJ	158.564	3.058	SADKUH	162.595	2.854
SADPAQ	155.518	3.333	SADPAQ	172.928	2.722	SADNUH	163.322	3.29	SADNUH	169.804	2.758	SADNUH	150.522	3.35
SAFFUB	152.421	3.141	SAFFUB	150.586	3.382	SADPAQ	159.101	3.179	SADPAQ	169.034	3.179	SADXAA	167.36	2.939
SAFROK	153.791	2.878	SAFWUS	158.416	3.238	SAFFUB	152.856	3.084	SAFLIX	159.958	2.927	SAFLIY	165.924	3.083
SAGMOG	151.449	2.904	SAGMOG(	151.576	2.908	SAFYOQ	151.667	3.303	SAGBIP	150.703	3.167	SAGBIP	157.412	3.371
SAGZAE	158.048	3.262	SAGZAE	170.452	3.299	SAGMUM	172.731	3.004	SAGRUP	154.492	3.305	SAGVEC	168.63	3.165
SAHJIX	152.865	3.181	SAHQID	151.961	3.049	SAHDAI	162.188	3.27	SAHDAI	172.613	3.392	SAHGAJ	153.463	2.976
SAHVUX	155.198	2.964	SAHWOS	162.565	3.45	SAHSUQ	156.879	3.466	SAHSUQ	171.642	2.654	SAHVUX	154.343	3.054
SAJFIU	151.939	3.145	SAJFOA	152.867	3.134	SAHWOS	151.658	3.235	SAJCUE	168.338	3.133	SAJFIU	153.03	3.135
SAJPEZ	162.803	3.042	SAJPEZ	163.688	2.918	SAJFOA	152.171	3.155	SAJMEX	155.968	3.172	SAJPEZ	152.197	2.651
SAJPOJ	154.028	2.677	SAJPOJ	163.53	3.07	SAJPID	153.033	2.667	SAJPID	164.818	3.054	SAJPID	165.507	2.893
SAJPUP	165.651	2.876	SAJTII	157.833	3.313	SAJPOJ	165.03	2.886	SAJPUP	156.143	2.658	SAJPUP	165.802	3.068
SAJYAH	177.896	3.459	SAJZEK	154.291	3.159	SAJTON	166.76	2.933	SAJTON	150.223	2.944	SAJTON	152.501	2.944
SAKFAN	169.01	2.901	SAKFAN	172.994	2.823	SAKBEN	173.696	3.271	SAKCUE	153.187	2.725	SAKCUE	153.581	2.725
SAKSUT	158.026	3.473	SAKSUT	152.904	3.465	SAKFUH	150.208	3.396	SAKSUT	153.067	3.411	SAKSUT	164.188	3.249
SAKSUT	154.159	3.344	SAKTAA	161.393	2.706	SAKSUT	167.876	3.434	SAKSUT	161.031	3.34	SAKSUT	171.873	2.664
SALHEV	155.394	3.15	SALHEV	159.017	2.728	SAKTAA	158.824	3.037	SAKTAA	170.273	2.706	SALHEV	170.859	3.087
SALHEV	170.858	2.795	SALHEV	151.461	3.246	SALHEV	178.114	3.293	SALHEV	155.12	2.878	SALHEV	170.294	3.115
SALWAE	165.905	3.323	SALWAE	167.243	3.11	SALHEV	162.548	3.165	SALNIC	151.95	3.444	SALQAZ	172.153	3.104
SAMGEU	164.489	2.942	SAMGEU	163.288	3.3	SALWAE	165.216	3.325	SAMFOE	156.087	3.48	SAMGEU	161.652	3.413
SAMGEW	161.486	3.283	SAMGIY	156.691	3.464	SAMGEU	174.628	3.081	SAMGEU	159.775	3.3	SAMGEV	157.369	3.38
SAMLOK	165.24	2.908	SAMLOK	162.854	2.912	SAMGIY	166.671	2.771	SAMGIZ	155.175	2.869	SAMLOK	152.108	2.908
SAMZAI	151.6	3.493	SANCOD	150.388	3.165	SAMVEL	151.464	3.215	SAMXOT	169.934	3.161	SAMXOT	168.855	3.168
SANNIF	152.851	3.273	SANRIL	162.247	3.212	SANFIZ	163.415	3.345	SANNIF	153.274	3.03	SANNIF	152.91	3.125
SANXAG	168.92	3.168	SANYOX	156.238	2.942	SANXAG	166.852	3.035	SANXAG	172.864	3.171	SANXAG	175.775	3.258
SAPHOK	152.86	3.138	SAPLUR	156.792	3.351	SANYOX	161.398	3.258	SANYOX	151.656	3.028	SANYOX	159.294	3.034
SAPLUR	163.987	3.128	SAPPAA	169.551	3.36	SAPLUR	151.634	3.247	SAPLUR	157.56	3.212	SAPLUR	167.902	3.281
SAPPEE	160	3.484	SAPPEE	164.044	3.146	SAPPAA	151.618	3.27	SAPPAA	150.527	3.12	SAPPEE	170.815	3.243
SAPPEE0	173.575	3.471	SAPPEE0	164.878	3.062	SAPPEE	160.053	2.862	SAPPEE	173.059	3.18	SAPPEE0	161.025	3.428
SAQCAQ	170.231	3.499	SARLOP	160.511	3.112	SAPPEE0	159.85	2.79	SAPPEE0	174.108	3.089	SAPPEE0	170.397	3.189
SARYAM	165.921	3.415	SARYIX	159.963	3.484	SARLOP	166.511	2.861	SARWAJ	161.91	3.369	SARYAM	151.374	3.287
SARYUJ	164.851	2.971	SARYUJ	165.599	2.857	SARYIX	155.76	2.856	SARYUJ	166.233	2.857	SARYUJ	155.221	2.845
SASHUQ	164.24	3.086	SASHUQ	158.263	2.788	SARZET	156.404	3.155	SASCOG	156.52	3.062	SASHUP	150.359	3.496
SASHUQ	150.171	3.296	SASHUQ	173.049	2.849	SASHUQ	176.905	2.961	SASHUQ	151.141	3.014	SASHUQ	161.482	2.887
SASHUR	168.27	3.011	SASHUR	161.876	3.367	SASHUQ	166.146	3.059	SASHUQ	161.366	2.929	SASHUQ	154.908	2.788
SASJAX	155.988	3.081	SASJAX	152.076	3.497	SASJAX	150.612	3.497	SASJAX	156.548	3.398	SASJAX	167.337	3.185
SASJIH	169.048	3.092	SASPAF	159.675	3.347	SASJAX	165.108	3.101	SASJAX	163.41	3.131	SASJIH	161.88	3.037
SASTOV	155.239	3.203	SATCUO	152.924	2.942	SASPUB	152.663	3.145	SASTOV	154.634	3.124	SASTOV	154.881	3.172
SATDAV	162.058	2.999	SATDAV	153.754	3.089	SATCUO	155.122	3.249	SATCUO	152.349	3.001	SATCUO	156.992	3.133
SATDIA	152.278	2.971	SATDIA	155.144	2.882	SATDAV	157.327	2.857	SATDIA	164.212	3.113	SATDIA	159.777	3.271
SATDIA	163.968	2.882	SATDIA	163.769	2.854	SATDIA	165.125	3.485	SATDIA	168.467	3.014	SATDIA	154.845	3.381
SATFOL	167.604	2.876	SATFOL	150.531	3.242	SATDIA	176.844	3.469	SATFOL	161.367	3.369	SATFOL	164.456	3.047
SATGOM	161.818	3.482	SATKAD	153.52	2.853	SATGIF	173.536	3.184	SATGOM	159.477	3.417	SATGOM	153.499	3.294
SATKAD	150.02	3.095	SATKAD	157.76	3.109	SATKAD	175.277	3.444	SATKAD	164.006	3.396	SATKAD	151.542	2.851
SATREO	155.398	3.192	SATREO	175.854	2.884	SATQUA	171.422	3.327	SATQUA0	173.751	3.279	SATREO	165.54	2.987
SATSUF	151.317	3.486	SATSUF	153.748	2.813	SATREO	154.215	3.427	SATREO	159.246	3.371	SATREO	162.272	3.275
SATSUF	155.254	3.3	SATTIU	150.584	3.444	SATSUF	172.765	3.417	SATSUF	171.026	2.698	SATSUF	152.161	2.813
SAVMAD	168.933	3.222	SAVMAD	163.136	3.342	SATTIU	154.593	3.183	SATXOE	164.27	2.636	SAVDAW	159.791	3.435
SAVSUG	173.946	3.466	SAVSUG	170.685	3.425	SAVNOU	156.56	3.252	SAVPIP	156.219	2.8	SAVPUB	172.205	3.363
SAVVAN	161.402	3.323	SAWBIB1(	150.324	3.014	SAVTUF	158.642	2.99	SAVVAN	168.031	3.323	SAVVAN	150.399	3.396
SAWQEM	154.215	3.27	SAWQEM	159.568	3.327	SAWBIB1(	161.072	3.461	SAWLEL	150.427	3.27	SAWQEM	159.05	3.009
SAWQEN	163.741	3.018	SAWWEU	179.107	2.811	SAWQEM	158.3	3.035	SAWQEM	175.408	3.009	SAWQEM	168.498	2.726
SAXJIL01	156.312	2.918	SAXJIL01	172.033	3.302	SAWWEU	163.357	3.137	SAWWEU	155.131	3.235	SAXJAF	161.266	3.375
SAXKIL	152.435	3.099	SAXKIL	168.281	2.905	SAXKIL	155.764	3.179	SAXKIL	165.868	3.453	SAXKIL	162.707	3.117
SAXVOD	170.106	2.809	SAXVOD	170.034	3.443	SAXKIL	166.368	2.914	SAXKIL	163.477	3.204	SAXMEM	150.823	3.338
SAXYUN	154.442	3.401	SAXYUN	150.308	3.097	SAXYIA	154.821	3.444	SAXYUN	171.327	3.257	SAXYUN	154.814	3.476
SAXYUN	157.428	2.675	SAXYUN	174.569	3.424	SAXYUN	150.318	2.752	SAXYUN	160.425	3.037	SAXYUN	168.886	2.6
SAXZAU	161.959	2.889	SAXZAU	159.578	2.896	SAXZAU	170.003	2.868	SAXZAU	155.834	2.92	SAXZAU	166.387	2.912
SAXZAU	160.594	3.106	SAXZAU	169.032	3.028	SAXZAU	160.955	2.891	SAXZAU	159.465	3.058	SAXZAU	160.034	3.046
SAXZEY	163.258	3.126	SAXZOI	152.734	2.979	SAXZAU	158.347	3.048	SAXZAU	165.732	2.727	SAXZEY	151.339	3.386
SAYJIP	173.195	3.084	SAYJIP	157.32	2.944	SAXZOI	157.451	3.334	SAXZOI	152.611	2.763	SAXZOI	155.992	2.905
SAYJOV	154.858	3.413	SAYJOV	177.064	2.733	SAYJOV	151.064	3.201	SAYJOV	162.6	2.766	SAYJOV	151.398	2.896
SAYJOV	150.054	2.772	SAYJOV	178.074	2.703	SAYJOV	155.323	3.395	SAYJOV	154.214	3.389	SAYJOV	150.946	2.81
SAYJOV	153.735	2.891	SAYJOV	159.885	3.282	SAYJOV	157.46	3.174	SAYJOV	165.79	2.68	SAYJOV	157.696	3.153

SAYJOV	152.463	2.766	SAYJOV	155.974	2.891	SAYJOV	158.344	3.158	SAYJOV	173.379	2.695	SAYJOV	153.467	3
SAYJOV	151.071	3.289	SAYJOV	154.623	3.061	SAYJOV	156.774	2.836	SAYJOV	155.792	3.065	SAYJOV	174.653	2.816
SAYJOV	175.917	2.764	SAYJOV	155.245	2.643	SAYJOV	168.154	3.402	SAYJOV	150.209	3.267	SAYJOV	158.106	3.396
SAYJOV	176.311	2.773	SAYJOV	157.683	3.261	SAYJOV	167.413	3.344	SAYJOV	170.589	3.272	SAYJOV	157.372	3.246
SAYKAI	176.581	2.755	SAYKAI	163.978	3.284	SAYJOV	160.945	3.257	SAYJUA	157.795	2.875	SAYKAH	152.185	2.565
SAYKAI	152.593	3.252	SAYKAI	154.577	3.423	SAYKAI	159.184	3.311	SAYKAI	178.797	2.711	SAYKAI	167.2	3.434
SAYKAI	150.509	3.375	SAYKAI	162.063	3.129	SAYKAI	156.784	3.423	SAYKAI	164.127	3.221	SAYKAI	164.164	3.289
SAYKAI	160.694	3.095	SAYKAI	151.704	2.74	SAYKAI	153.165	2.791	SAYKAI	156.551	2.997	SAYKAI	153.001	2.819
SAYKAI	177.179	2.771	SAYKAI	176.853	2.711	SAYKAI	153.425	3.174	SAYKAI	166.939	3.098	SAYKAI	153.031	3.461
SAYKAI	174.279	3.449	SAYKAI	157.712	2.736	SAYKAI	170.967	3.466	SAYKAI	174.955	2.752	SAYKAI	173.776	2.826
SAYKAI	162.564	3.28	SAYKAI	161.349	3.274	SAYKAI	152.79	2.982	SAYKAI	160.635	3.347	SAYKAI	164.225	3.456
SAYKAI	157.423	3.203	SAYKAI	156.744	3.131	SAYKAI	161.298	2.647	SAYKAI	155.302	3.034	SAYKAI	153.335	2.834
SAZBUU	156.997	3.429	SAZCAB	166.939	3.238	SAYPAN	151.298	3.335	SAYXEV	173.58	2.94	SAYXEV	169.393	3.08
SAZRIX	174.585	2.915	SAZRIX	177.394	3.242	SAZHUZ	158.922	3.196	SAZHUZ	162.619	3.409	SAZRIX	154.526	3.242
SAZTIW	152.406	3.413	SAZTIW	159.47	3.248	SAZRIX	151.581	3.077	SAZRIX	151.24	3.283	SAZSOD	159.337	3.413
SAZYAU	150.944	3.348	SAZYAU	156.269	2.877	SAZXUP	157.95	2.687	SAZYAU	156.014	3.387	SAZYAU	157.543	2.877
SAZYAW	174.055	3.331	SAZZAW	173.25	3.235	SAZYAV	161.264	3.077	SAZYAV	157.337	3.32	SAZYAW	172.19	3.347
SEBCUY	158.773	3.217	SEBYUV	172.638	3.325	SAZZAW	173.061	3.247	SEBCUY	153.818	3.204	SEBCUY	162.715	3.106
SECFUB	160.016	3.482	SECFUB	178.078	3.249	SEBYUV	152.096	3.384	SEBYUV	165.621	3.379	SEBYUV	153.523	3.11
SEDQUR	150.651	3.017	SEDREC	151.41	2.907	SECGOY	161.218	2.779	SECQUO	157.231	3.217	SECWOO	156.147	3.268
SEFCIT	173.292	2.84	SEFDIR	161.272	2.78	SEDRIG	167.327	2.852	SEDZUZ	164.658	3.129	SEFCIT	173.146	2.84
SEFDIR	155.103	3.11	SEFHES	160.56	2.921	SEFDIR	161.968	3.229	SEFDIR	168.485	2.8	SEFDIR	156.731	3.124
SEFZEK	152.568	3.22	SEFZOW	157.995	2.999	SEFMOH	161.368	3.091	SEFRAX	176.177	3.357	SEFXUZ	151.865	3.018
SEGBAK	160.4	3.445	SEGBAK	176.06	2.888	SEFZOW	165.943	3.462	SEGBAK	161.476	3.372	SEGBAK	150.126	2.847
SEGPED	170.374	3.101	SEGPED	156.754	3.041	SEGNOL	177.291	3.075	SEGPAW	167.65	3.395	SEGPAW	169.095	3.423
SEGPIG	162.504	3.186	SEGPIG	155.369	3.1	SEGPED	164.157	3.455	SEGPIG	160.012	2.827	SEGPIG	167.156	2.636
SEGPIH	154.808	2.988	SEGPON	153.654	2.863	SEGPIG	165.374	2.78	SEGPIG	154.146	3.068	SEGPIH	171.111	3.173
SEGPON	164.011	3.208	SEGPUT	175.507	3.2	SEGPON	165.937	2.863	SEGPON	160.771	3.045	SEGPON	155.505	2.999
SEGZOX	159.087	2.653	SEGZOX	161.132	3.261	SEGPUT	160.97	3.125	SEGPUT	165.996	3.201	SEGPUT	153.505	2.974
SEHMOK	158.402	3.249	SEHMOK	164.68	2.848	SEGZOX	161.906	2.653	SEHBUF	152.871	3.325	SEHBUF	168.21	3.325
SEJHEW	175.324	2.578	SEJKAT	150.369	3.095	SEHWIN	155.459	3.142	SEHXIQ	164.329	3.351	SEHZOW	157.307	3.086
SEKCOE	162.386	3.402	SEKCOE0	162.718	3.329	SEJKAT	173.714	2.841	SEJKAT	160.085	2.791	SEKCIY	167.651	2.998
SELNUW	151.157	3.453	SELPAE	151.864	3.46	SEKHAT	154.694	3.099	SELGIC	150.474	3.405	SELHIA	172.362	3.08
SELSOT	151.393	2.966	SELSOT	162.941	3.382	SELPEI	172.157	3.409	SELQAC	159.1	3.446	SELQAC	156.92	3.483
SELSOT	166.403	3.468	SELTIO	156.537	2.954	SELSOT	161.172	3.123	SELSOT	157.284	2.926	SELSOT	150.098	2.748
SELTIO	150.307	3.245	SELTIO	152.002	3.141	SELTIO	151.82	3.335	SELTIO	156.871	3.141	SELTIO	156.177	3.256
SELYIS	157.513	3.498	SELYIS	160.274	3.44	SELTIO	165.222	3.004	SELYIS	150.458	3.318	SELYIS	160.901	3.166
SELZAN	154.055	2.891	SELZAN	167.019	3.331	SELYIU	163.594	3.3	SELYOA	158.094	3.02	SELYOA	161.925	3.4
SEMCIA	177.435	3.231	SEMCUM	165.037	3.003	SELZER	155.289	3.191	SEMCIA	163.623	3.182	SEMCIA	168.802	3.209
SEMCUM	151.383	3.285	SEMCUM	159.126	2.99	SEMCUM	156.655	3.083	SEMCUM	152.091	2.963	SEMCUM	150.23	3.119
SEMZIU	158.955	2.97	SEMZIU	157.221	2.981	SEMCUM	150.481	3.493	SEMJON	160.986	3.408	SEMKIH	161.953	3.136
SENWAK	160.687	2.991	SENWAK	160.39	2.997	SENWAK	157.465	3.114	SENWAK	157.919	2.988	SENWAK	161.538	3.091
SENWAK	171.367	3.061	SENWAK	177.22	2.979	SENWAK	154.562	2.991	SENWAK	158.434	2.988	SENWAK	161.35	2.98
SENWIV	162.696	3.154	SENWIV	159.575	3.099	SENWAK	156.25	2.852	SENWAK	166.299	2.891	SENWAK	154.884	3.378
SEPBAS	151.166	2.946	SEPBAS	167.702	3.358	SENWIV	165.606	3.122	SENWIV	162.465	3.221	SENXIW	164.895	3.287
SEPBAS02	154.959	3.249	SEPBAS02	157.534	2.983	SEPBAS01	152.498	3.15	SEPBAS01	157.497	2.982	SEPBAS01	155.063	3.246
SEPBOG	178.591	2.998	SEPCOH	167.131	3.133	SEPBAS02	152.376	3.151	SEPBOG	162.67	2.918	SEPBOG	155.53	3.46
SEPCOH	155.697	3.141	SEPCOH	166.342	3.329	SEPCOH	165.35	3.163	SEPCOH	151.019	3.008	SEPCOH	153.597	3.194
SEPDAU	176.382	3.429	SEPDEY	154.944	2.737	SEPCUN	167.849	2.85	SEPDAU	151.413	3.395	SEPDAU	154.994	3.362
SEPQUD	153.027	3.42	SEPQUD	169.257	3.407	SEPDEY	162.561	3.181	SEPFEA	150.318	2.802	SEPLOS	154.11	3.193
SEPVOZ	154.851	2.836	SEPXIV	150.374	2.788	SEPTIT01	154.465	3.303	SEPVOZ	152.036	3.346	SEPVOZ	165.054	3.372
SEQPAG	156.681	2.744	SEQPUD	153.732	2.835	SEPXIV	175.324	2.788	SEPXOB	151.816	3.336	SEQKIL	156.44	3.212
SEQQEL	173.632	2.965	SERGOO	151.585	2.665	SEQQAG	177.169	3.287	SEQQAG	168.174	3.289	SEQQAG	162.683	3.277
SERLUA	172.746	2.97	SERLUA	174.176	3.224	SERGOO	155.221	2.896	SERGUU	160.722	3.328	SERLOR	162.382	3.005
SERSAK	154.305	2.89	SERTAK	165.912	2.86	SERLUA	161.184	3.058	SERPEM	159.548	3.069	SERQEL	157.322	2.974
SERVIW	168.295	3.099	SERYOD	156.806	3.104	SERVIW	170.114	3.481	SERVIW	150.95	3.147	SERVIW	170.216	3.475
SERYOD0	169.326	2.898	SETLIM	151.811	3.157	SERYOD	166.25	2.84	SERYOD	150.353	3.438	SERYOD0	162.037	3.018
SETNOU	155.458	2.815	SETNOU	152.887	3.075	SETLIM	154.406	3.246	SETLIM	157.222	3.157	SETLIM	153.282	3.166
SETNOU	150.63	3.241	SETNOU	151.934	3.485	SETNOU	159.958	2.868	SETNOU	165.59	3.135	SETNOU	155.866	2.815
SETQIR	158.93	3.041	SETQIR	162.967	2.738	SETNOU	157.892	2.899	SETQIR	173.42	2.897	SETQIR	150.815	2.967
SEVBOM	159.913	3.056	SEVTEW	155.251	3.06	SETRIT	157.785	3.141	SETZAW	156.667	3.215	SEVBOL	162.562	3.234
SEVWUP	150.4	2.89	SEVWUP	159.322	3.343	SEVWIA	153.023	2.935	SEVWIA	154.554	2.935	SEVWUP	157.332	2.698
SEVXAW	163.831	3.117	SEVXAW	163.434	3.344	SEVXAW	164.005	3.356	SEVXAW	162.792	2.865	SEVXAW	162.044	3.004
SEWJUB	175.249	3.219	SEWJUB	176.466	3.219	SEVXAW	152.591	3.134	SEWCOQ	165.739	3.237	SEWFUW	153.586	2.979
SEWMAK	160.84	3.334	SEWMAK	166.448	2.961	SEWLUD	157.935	3.49	SEWLUD	162.008	2.832	SEWLUD	167.593	3.313
SEWSAQ	154.945	2.991	SEWSAQ	158.986	2.97	SEWMAK	170.417	3.304	SEWSAQ	152.37	3.351	SEWSAQ	171.427	3.412
SEWSAQ	171.635	3.018	SEWSAQ	166.063	3.205	SEWSAQ	155.773	2.991	SEWSAQ	154.487	3.415	SEWSAQ	155.712	3.409
SEWSEU	153.233	3.27	SEWSEU	151.799	2.875	SEWSEU	163.18	3.182	SEWSEU	170.192	3.116	SEWSEU	156.751	3.37
SEYDAD0	150.858	3.17	SEYDAD0	153.021	2.997	SEYBEH	166.829	3.138	SEYCEI	162	3.235	SEYCEI	165.806	2.801
SEYQUK	151.382	2.972	SEYQUK	151.547	3.461	SEYDAD0	162.362	3.165	SEYJEM	156.585	2.819	SEYJEM01	159.676	2.758
SEYYAX	153.874	2.874	SEYYAX	151.502	2.82	SEYTIB	151.925	3.359	SEYTIB	176.071	3.024	SEYYAX	158.735	3.089
SEYYEB	158.909	3.067	SEYYEB	156.384	3.483	SEYYAX	178.754	3.373	SEYYAX	161.392	3.074	SEYYEB	154.239	2.847
SEYYIF	154.955	2.83	SEYYIF	159.374	3.071	SEYYEB	176.662	3.253	SEYYEB	150.709	2.827	SEYYEB	162.816	3.057
SEYYIF	163.676	3.08	SEZBAA	157.307	3.445	SEYYIF	150.916	2.846	SEYYIF	175.467	3.178	SEYYIF	154.862	3.497
SEZKAL	171.554	3.266	SEZQOG	152.392	3.067	SEZCAF	165.092	2.975	SEZJIS	172.441	3.182	SEZJIS	151.19	3.031
SEZTUO	161.603	3.128	SEZTUO	156.615	3.441	SEZQOG	168.467	3.147	SEZQOG	151.923	3.062	SEZRAT	159.001	3.438
SIBDEP	150.953	3.089	SIBDEP	154.51	3.089	SIBDAL	155.129	3.226	SIBDAL	163.076	3.171	SIBDAL	158.813	3.418
SIBDOZ	156.888	2.875	SIBDOZ	151.279	3.135	SIBDEP	164.311	2.873	SIBDEP	169.917	2.873	SIBDOX	156.764	3.21
SIBGEQ01	170.16	2.521	SIBGEQ01	163.302	2.879	SIBDOZ	154.822	3.391	SIBDOZ	155.395	3.391	SIBDUC	161.374	2.961

SIBNOJ	154.974	3.054	SIBPAX	158.504	3.227	SIBGEQ01	174.453	2.879	SIBNOJ	154.322	2.994	SIBNOJ	155.713	3.087
SIBPEB	158.953	3.184	SIBPUR	169.246	3.222	SIBPAX	159.125	2.922	SIBPEB	154.7	2.908	SIBPEB	164.534	2.908
SIBPUR	152.129	2.815	SIBPUR	174.074	2.923	SIBPUR	167.535	3.197	SIBPUR	174.019	3.018	SIBPUR	152.412	2.815
SIBQAY	166.209	3.384	SIBQAY	166.854	3.377	SIBPUR	156.818	2.966	SIBQAY	157.773	2.96	SIBQAY	156.465	2.636
SICJOH	161.108	3.156	SIDCAM	176.889	3.35	SIBQAY	163.194	3.269	SIBSOO	169.384	3.061	SIBTIH	159.792	2.999
SIDLEX	151.768	2.995	SIDLIB	175.611	2.874	SIDCEQ	153.457	3.364	SIDCEQ	153.903	3.258	SIDLEX	162.791	2.784
SIDMIB	158.502	3.414	SIDZOU	166.83	2.98	SIDLIB	167.213	3.316	SIDLOH	164.874	2.941	SIDMIB	154.343	3.238
SIFCOC	174.833	2.767	SIFCOC	163.254	3.299	SIDZOU	155.31	3.386	SIFBAK	153.028	3.162	SIFCOC	168.175	3.148
SIFCOC	159.448	3.314	SIFCOC	151.599	3.217	SIFCOC	159.371	3.221	SIFCOC	151.833	3.331	SIFCOC	152.067	3.337
SIFDET	154.829	3.183	SIFDET	154.061	3.441	SIFCOC	160.281	3.449	SIFDAO	150.979	3.297	SIFDAO	162.746	3.315
SIFDIW	163.631	3.372	SIFDOC	167.229	2.875	SIFDET	177.059	2.932	SIFDET	151.005	3.447	SIFDIW	155	3.473
SIGBAP	168.47	3.489	SIGDIV	163.887	2.866	SIFZIQ	152.683	2.994	SIFZIQ	167.101	3.016	SIFZIQ	169.93	3.063
SIGJIE	157.827	2.899	SIGJIE	160.828	3.132	SIGDOB	165.604	3.345	SIGDOB	163.352	2.96	SIGDOB	173.295	2.898
SIGTAE	151.914	2.887	SIGTAE	162.595	2.834	SIGQAD	156.829	3.103	SIGTAE	164.661	3.421	SIGTAE	170.554	3.245
SIGTEI	160.414	3.044	SIGTEI	156.696	2.609	SIGTAE	151.338	3.391	SIGTEI	170.667	3.432	SIGTEI	164.333	2.984
SIHDAS	162.734	2.796	SIHKUS	165.263	2.874	SIGTUY	153.783	2.846	SIGTUY	160.341	3.156	SIHDAS	154.685	3.435
SIHQOQ0	158.607	2.999	SIHQOQ0	164.229	2.535	SIHKUS	156.448	2.778	SIHPUX	177.302	2.902	SIHPUX	150.245	3.309
SIHQOQ0	169.121	3.314	SIHQOQ0	169.076	2.839	SIHQOQ0	151.486	2.939	SIHQOQ0	157.027	2.917	SIHQOQ0	160.414	3.454
SIHRAD	169.159	2.989	SIHRAD	161.659	3.421	SIHQOQ0	157.852	2.698	SIHQOQ0	160.656	2.94	SIHQOQ0	151.477	3.448
SIHRAD	159.669	3.04	SIHRAD	166.768	3.027	SIHRAD	176.775	3.474	SIHRAD	162.664	3.034	SIHRAD	156.344	3.003
SIJGOL	169.389	3.384	SIJLIK	150.89	2.954	SIHRAD	178.773	3.432	SIHSIN	152.361	3.267	SIHSIN	167.67	3.055
SIJPUZ	168.725	2.946	SIJQAG	155.104	3.344	SIJPAF	165.097	3.342	SIJPOT	151.864	3.484	SIJPOT	152.599	3.158
SIKLEG	159.193	3.135	SIKNIJ	172.42	3.247	SIJVIU	166.583	3.468	SIJYER	166.995	2.8	SIKKUT	161.229	2.97
SIKVOZ	155.608	2.806	SIKVOZ	168.637	3.482	SIKPUB	175.222	3.426	SIKPUB	160.751	3.107	SIKVIT	160.126	3.335
SIKWAM	159.863	3.229	SIKWAM	160.359	3.111	SIKVOZ	160.006	3.355	SIKVOZ	159.936	2.85	SIKWAM	156.85	3.481
SILNEH	158.02	2.891	SILPAH	159.984	3.212	SIKWAM	150.107	3.36	SILFAX	170.708	2.912	SILJOQ	160.726	3.001
SILPAH	165.031	2.825	SILROX	150.194	3.034	SILPAH	155.519	2.871	SILPAH	151.281	3.256	SILPAH	151.481	3.211
SIMFAX	175.444	3.359	SIMFAX	153.883	3.046	SIMDID	151.458	3.263	SIMDID	152.756	3.263	SIMFAX	154.357	3.246
SIMGUU	150.615	3.42	SIMGUU	170.591	3.266	SIMFAX	154.43	3.171	SIMFAX	159.717	3.254	SIMFAX	173.605	3.412
SIMHAB	152.088	3.445	SIMHEF	168.029	3.406	SIMGUU	177.825	2.939	SIMHAB	163.66	3.4	SIMHAB	161.879	3.237
SIMHEF	173.162	3.125	SIMHEF	161.829	2.794	SIMHEF	166.778	3.289	SIMHEF	163.75	2.874	SIMHEF	165.673	3.441
SIMHIJ	160.511	3.34	SIMTEO	159.726	3.391	SIMHIJ	152.233	3.261	SIMHIJ	152.669	3.423	SIMHIJ	158.822	2.942
SIMXIV	151.376	3.248	SIMZEU	153.621	3.076	SIMWIU	169.31	2.789	SIMWIU	173.2	2.863	SIMXER	170.091	3.255
SIMZEU	152.518	3.2	SINFEE	167.833	2.852	SIMZEU	160.911	3.34	SIMZEU	161.744	3.397	SIMZEU	161.035	3.435
SINKAF	167.832	3.416	SINLOT	156.503	2.88	SINFEE	169.103	2.96	SINFEE	150.377	2.785	SINGUR	153.569	3.194
SINLUZ	159.521	3.201	SINLUZ	151.952	2.901	SINLOT	177.117	3.248	SINLUZ	154.704	3.484	SINLUZ	168.577	3.098
SINMIO	166.106	3.109	SINMIO	165.907	3.344	SINLUZ	163.861	3.315	SINMEK	164.763	3.373	SINMEK	164.727	3.079
SIPGII	151.609	3.251	SIQDON	175.271	3.06	SINQOW	155.286	3.444	SINQOW	164.218	3.246	SINYUK	166.276	3.083
SIQKEJ	170.341	3.477	SIQKEJ	169.337	2.991	SIQDON	152.132	3.055	SIQKEJ	160.205	3.49	SIQKEJ	161.117	3.431
SIQNEM	162.971	3.276	SIQNEM	155.238	3.175	SIQKEJ	155.322	3.475	SIQKEJ	151.969	3.164	SIQNEM	165.255	3.175
SIQRAM	162.003	3.438	SIRFOS	155.662	2.971	SIQNEM	157.646	3.303	SIQNEM	156.818	3.132	SIQQOZ	164.779	3.177
SIRLEM	150.212	3.356	SIRNOA	171.513	2.981	SIRKEJ	153.071	3.2	SIRKEJ	153.979	3.375	SIRKIR	161.707	2.805
SIRVIY	165.977	3.181	SIRVIY	150.864	3.054	SIRNOA	153.203	3.374	SIRNOA	152.821	3.029	SIRVIY	157.632	3.174
SIRVUN	154.772	3.307	SIRVUN	152.359	3.43	SIRVIY	152.399	3.001	SIRVOE	160.611	3.143	SIRVOE	150.534	2.889
SIRYEZ	156.599	2.887	SIRYEZ	163.881	3.422	SIRVUN	151.764	3.158	SIRVUN	167.809	3.041	SIRYEZ	166.458	3.467
SISHIN	158.917	2.875	SISHOT	165.784	3.2	SIRYEZ	164.674	3.26	SISHIN	150.01	3.13	SISHIN	169.318	3.245
SISHOT	150.449	3.126	SISJUB	168.385	3.018	SISHOT	152.835	3.343	SISHOT	165.408	2.933	SISHOT	153.213	2.686
SISJUB	171.792	3.018	SISJUB	162.025	2.829	SISJUB	155.649	3.114	SISJUB	161.246	2.829	SISJUB	157.803	3.114
SISMEP01	169.108	3.231	SISMEP01	170.219	2.981	SISKAG	150.231	2.85	SISKAI	172.882	3.184	SISMEP01	155.573	2.968
SISMEP01	169.317	2.809	SISMEP01	160.78	2.937	SISMEP01	156.561	3.018	SISMEP01	158.987	3.327	SISMEP01	158.159	3.053
SISMOZ	165.156	3.047	SISMOZ	150.703	3.135	SISMIT	154.419	2.941	SISMIT	158.034	2.859	SISMOZ	161.668	3.208
SISRIX	150.864	3.039	SISRIX	162.538	3.318	SISMOZ	162.893	2.789	SISMOZ	162.095	3.124	SISMOZ	158.033	2.8
SISWAT	163.631	2.938	SISWAT	155.955	3.179	SISRIX	161.007	3.472	SISRIX	175.169	3.109	SISWAT	150.667	3.179
SITGIN	158.46	3.157	SITHAH	151.345	2.748	SISXUQ	166.45	3.468	SITDUW0	153.761	3.218	SITFOR	163.691	3.05
SITSUM	155.128	3.26	SITZOK	157.15	3.057	SITHAH	163.22	2.744	SITHAH	155.003	3.448	SITKAK	163.708	3.455
SIVTAT	155.236	3.074	SIVTAT	150.748	3.488	SIVFOU	160.267	3.17	SIVFOU	166.624	3.17	SIVFOU	157.428	3.146
SIVTAT	168.769	3.187	SIVTAT	162.077	3.283	SIVTAT	154.659	3.091	SIVTAT	170.383	3.279	SIVTAT	153.858	3.307
SIVTAT	171.992	2.783	SIVTAT	160.014	3.317	SIVTAT	151.887	3.091	SIVTAT	155.659	2.648	SIVTAT	151.018	3.032
SIVTAW	164.673	3.221	SIVTAW	153.227	3.395	SIVTAT	171.403	3.006	SIVTAW	155.76	2.95	SIVTAW	157.767	3.352
SIVTUQ	163.848	3.203	SIVTUQ	153.167	3.387	SIVTAW	174.244	3.495	SIVTUQ	155.332	2.943	SIVTUQ	157.933	3.344
SIVYUS	153.592	3.157	SIVZAZ	169.835	3.026	SIVTUQ	173.939	3.486	SIVYUS	160.559	3.157	SIVYUS	170.077	3.054
SIVZED	154.082	2.948	SIVZED	164.714	3.365	SIVZAZ	150.016	2.815	SIVZAZ	150.792	3.422	SIVZED	152.768	3.187
SIVZED	153.133	3.406	SIVZED	151.709	2.897	SIVZED	150.84	3.098	SIVZED	163.429	3.304	SIVZED	151.959	3.01
SIVZED	173.416	3.437	SIVZIH	166.077	2.679	SIVZED	165.64	2.918	SIVZED	162.851	3.018	SIVZED	159.748	2.848
SIVZON	153.747	3.065	SIVZON	152.217	2.752	SIVZIH	163.497	3.226	SIVZIH	160.347	3.42	SIVZON	169.503	2.933
SIVZUT	170.48	3.334	SIVZUT	167.763	2.964	SIVZON	165.869	3.269	SIVZON	160.503	3.203	SIVZON	150.972	3.078
SIVZUT	151.767	2.997	SIVZUT	152.889	2.949	SIVZUT	165.821	3.088	SIVZUT	160.666	3.052	SIVZUT	168.821	3.367
SIVZUT01	157.935	2.735	SIVZUT01	158.535	3.239	SIVZUT	170.347	2.972	SIVZUT01	155.724	2.907	SIVZUT01	174.19	2.747
SIVZUT01	157.897	3.008	SIWBEG	156.32	2.879	SIVZUT01	169.42	3.198	SIVZUT01	171.882	3.297	SIVZUT01	159.242	2.663
SIWBIK	163.367	3.101	SIWBIK	159.663	2.84	SIWBEG	150.184	2.777	SIWBIK	151.419	3.056	SIWBIK	158.978	3.325
SIWMIV	158.519	3.386	SIWSOH	162.365	3.093	SIWBIK	160.258	2.875	SIWBUX	158.272	3.476	SIWBUX	165.737	2.798
SIWSOH	151.517	3.097	SIWZAD	152.306	2.98	SIWSOH	152.198	3.129	SIWSOH	159.83	2.894	SIWSOH	150.667	3.427
SIYKUI	165.846	3.014	SIYKUI	150.841	3.031	SIWZAD	156.86	3.491	SIXPEW	180	3.123	SIXZIM	168.066	3.127
SIYRAV	174.975	3.366	SIYRAV	156.243	3.118	SIYLOE	155.794	3.267	SIYRAV	169.169	2.688	SIYRAV	174.049	3.194
SIYTIF	154.997	3.092	SIYTOK	169.997	2.811	SIYRAV	159.987	2.688	SIYRID	160.568	3.062	SIYROJ	151.34	3.488
SIYYAA	158.028	3.04	SIYYAA	154.035	3.124	SIYTOK	170.591	3.002	SIYTOK	151.706	3.183	SIYVIJ	172.085	3.133
SIZFIR	150.621	3.131	SIZFIR	150.68	3.027	SIZBUZ	158.825	3.381	SIZDUE	154.315	3.32	SIZFIR	168.169	3.093
SIZPIB	166.472	2.906	SIZPIB	159.421	3.187	SIZFIR	152.159	3.386	SIZPIB	171.433	2.86	SIZPIB	160.895	3.285
SIZZIO	153.968	2.935	SIZZOQ	166.882	3.027	SIZZEH	161.11	2.961	SIZZEH	164.259	3.303	SIZZEK	151.311	2.852

SOBFOI	150.796	2.921	SOBFOI	153.698	3.198	SOBBEQ	167.024	2.991	SOBCUH	166.935	2.975	SOBFOI	153.004	2.987
SOBHIC	170.807	2.955	SOBNAC	157.117	2.955	SOBHIC	160.724	2.955	SOBHIC	153.826	3.319	SOBHIC	157.677	2.887
SOBNAC	171.168	3.222	SOBNAC	154.928	3.414	SOBNAC	172.107	2.989	SOBNAC	172.421	2.946	SOBNAC	172.851	2.861
SOBNIJ	153.798	3.299	SOBPIK	151	2.952	SOBNAC	153.825	3.206	SOBNAC	152.456	2.734	SOBNAC	150.582	2.869
SOBRUW	165.079	3.07	SOBRUW	176.992	3.36	SOBPIL	163.198	2.828	SOBPIL	156.929	3.148	SOBQIK	153.542	3.124
SOBTIM	164.486	3.097	SOBTIM	163.165	3.073	SOBRUW	154.907	3.272	SOBRUW	165.847	3.272	SOBRUW	156.789	3.04
SOBTIM1	164.405	3.102	SOBXAM	171.828	2.968	SOBTIM	164.405	3.102	SOBTIM1	164.486	3.097	SOBTIM1	163.165	3.073
SODDAQ	172.936	3.099	SODDEU	156.327	3.379	SOCMUW	157.608	3.333	SOCSAI	153.943	3.456	SOCSAI	171.497	3.12
SODKII	154.306	3.332	SODSEK	167.763	3.221	SODHAW	157.475	3.43	SODKAY	159.885	3.343	SODKII	152.83	3.493
SODXIV	174.926	2.67	SOFHUS	164.18	3.136	SODSEK	153.901	2.821	SODSEK	152.678	2.669	SODXIV	160.505	2.947
SOGTOB	156.882	3.331	SOGWEU	152.714	3.109	SOFSEO	164.155	3.111	SOFSEO	151.658	2.922	SOGFEC	152.084	3.492
SOGYAQ	157.029	2.704	SOGYAQ	168.938	3.417	SOGWEU	151.997	2.976	SOGWEU	167.372	3.3	SOGXUJ	154.603	2.813
SOHPEO	163.106	3.013	SOHPEO	154.5	3.019	SOGYAQ	161.888	2.91	SOGYAQ	163.997	2.888	SOHBAU	154.93	3.072
SOHREQ	159.808	3.486	SOHSEN	166.074	2.746	SOHPEO	162.296	3.308	SOHPEO	170.042	3.293	SOHRAM	167.357	3.005
SOHWUI	162.221	3.305	SOHWUI	167.201	2.993	SOHSEN	174.304	2.746	SOHWUI	160.031	3.032	SOHWUI	176.153	2.996
SOHWUI	165.13	2.971	SOHWUI	164.343	3.203	SOHWUI	171.454	3.14	SOHWUI	169.086	3.041	SOHWUI	158.955	3.156
SOJCIG	172.137	2.901	SOJCIG	160.295	3.096	SOHWUI	154.618	3.305	SOHWUI	156.681	2.865	SOHYUM	153.706	3.23
SOJCUS	151.03	2.817	SOJCUS	171.136	3.164	SOJCUS	168.297	3.164	SOJCUS	154.77	2.952	SOJCUS	158.852	3.427
SOJQAJ	152.387	3.461	SOJQAJ	164.763	3.247	SOJCUS	170.893	3.314	SOJCUS	152.326	3.089	SOJQAJ	159.019	3.453
SOJYUN	153.087	2.886	SOJYUP	162.448	2.892	SOJYOF	153.812	3.499	SOJYOF	170.156	3.352	SOJYUN	177.96	2.647
SOKCUT	150.826	3.281	SOKDUS	160.617	2.832	SOKBOL	157.847	3.369	SOKCUT	152.247	3.062	SOKCUT	150.292	3.04
SOKDUS	157.752	2.815	SOKDUS	159.696	2.787	SOKDUS	152.552	2.905	SOKDUS	158.474	2.742	SOKDUS	157.422	2.873
SOKFAD	170.426	3.465	SOKFAD	155.719	3.128	SOKDUS	161.146	3.177	SOKDUS	157.8	3.387	SOKDUS	156.221	3.231
SOKLOU	169.873	3.228	SOKLOU	162.282	3.242	SOKLOU	163.568	3.414	SOKLOU	173.959	2.877	SOKLOU	154.181	3.222
SOLFAA	152.716	3.113	SOLFAA	157.414	2.879	SOKNIS	154.247	3.467	SOKSOE	150.505	3.103	SOLFAA	156.952	3.033
SOLQAO	154.896	3.405	SOLQIW	167.39	3.04	SOLJOT	174.541	2.775	SOLJOT	168.179	3.494	SOLPAN	158.005	3.18
SOLQIW	157.799	2.808	SOLQOC	156.031	3.26	SOLQIW	163.65	3.346	SOLQIW	162.581	3.066	SOLQIW	154.726	2.808
SOLQOC	165.734	3.329	SOLQOC	164.17	2.977	SOLQOC	164.073	3.266	SOLQOC	159.287	3.305	SOLQOC	165.935	3.471
SOLQOC	155.316	2.966	SOLQOC	153.395	3.055	SOLQOC	152.569	2.979	SOLQOC	165.233	2.821	SOLQOC	163.993	3.299
SOLSOE	154.245	3.212	SOLSOE	150.343	2.831	SOLSOE	172.912	2.779	SOLSOE	152.232	3.158	SOLSOE	154.281	3.072
SOMHIN	152.225	3.277	SOMHIN	155.244	3.059	SOLSOE	151.927	2.886	SOLSOE	172.348	3.065	SOLTUI	153.782	2.815
SOMLIR	167.377	3.465	SOMLIR	152.283	3.031	SOMHIN	156.993	3.277	SOMHIN	153.181	3.059	SOMLIR	162.888	3.264
SOMNER	170.926	2.859	SOMQAM	151.005	3.114	SOMLIR	158.754	2.887	SOMLIR	163.758	3.454	SOMLIR	151.999	2.983
SOMVAT	166.12	3.44	SOMVUP	173.427	2.813	SOMQAM	160.003	2.852	SOMVAT	158.15	3.345	SOMVAT	158.326	3.069
SONJEM	172.913	2.933	SONQEU	166.498	3.025	SOMWIE	156.418	2.978	SOMWIE	160.665	2.826	SONGUA	169.778	3.163
SOPBUV	172.172	3.044	SOPCOS	153.613	3.315	SONYUS	157.431	2.944	SONYUS	177.836	3.044	SONYUS	153.744	3.483
SOPDOT	153.936	3.199	SOPFIN	154.75	2.845	SOPCOS	154.497	3.049	SOPCOS	165.228	3.286	SOPCUW	156.534	2.747
SOPFIN	163.285	3.282	SOPFIN	175.073	3.011	SOPFIN	172.747	3.049	SOPFIN	162.649	3.037	SOPFIN	154.186	3.243
SOPFIN	153.836	3.14	SOPFIN	150.428	3.16	SOPFIN	157.953	3.309	SOPFIN	158.811	2.808	SOPFIN	158.23	3.085
SOPGAJ	155.053	2.893	SOPGAJ	155.273	2.89	SOPFIN	172.716	3.2	SOPFIN	150.128	3.097	SOPFIN	151.484	2.857
SOPRUM	153.067	2.982	SOPRUM	155.626	3.35	SOPGEN	172.824	3.274	SOPNUI	156.879	3.251	SOPNUI	171.752	3.298
SOQGOU	150.8	2.82	SOQGOU	165.761	2.983	SOPVUQ	168.397	3.104	SOQGOU	160.859	3.098	SOQGOU	155.473	3.043
SOQLUH	168.499	2.907	SOQLUH	156.321	2.981	SOQGUA	166.369	2.884	SOQJUD	152.173	3.478	SOQLUH	160.226	2.892
SOQLUH	151.156	2.784	SOQQUK	170.639	3.163	SOQLUH	152.837	3	SOQLUH	163.956	3.328	SOQLUH	163.608	3.333
SORGOX	175.083	3.424	SORXIJ	165.22	3.184	SOQRIC	153.823	3.424	SOQZEH	167.716	2.979	SORGOX	156.346	3.424
SORXIJ	151.512	3.082	SORYAB	164.599	3.189	SORXIJ	154.138	3.41	SORXIJ	153.142	3.13	SORXIJ	154.081	3.322
SOSHUF	165.238	3.125	SOSHUF	161.39	3.107	SOSBOS	153.436	3.178	SOSBOS	153.386	3.199	SOSGUE	154.682	3.064
SOSKAO0	171.504	2.958	SOSKAO0	174.438	2.899	SOSJEQ	164.446	3.306	SOSJEQ	162.008	3.312	SOSJEQ	150.934	2.948
SOSVAA	165.732	3.125	SOSVAA	158.948	3.21	SOSKAO0	154.288	3.394	SOSNAR	154.645	3.39	SOSTEC	174.365	2.995
SOTHAM	164.526	2.85	SOTHEO	167.13	3.217	SOTBAE	156.467	3.178	SOTHAM	176.117	3.19	SOTHAM	173.714	3.071
SOTJIX01	154.534	3.49	SOTJIX01	155.219	3.478	SOTHOA	151.784	2.883	SOTHUG	154.417	3.388	SOTJIX01	155.231	3.493
SOTYOT	151.029	3.237	SOVDEO	163.543	2.754	SOTVAA	168.053	2.936	SOTVAA	156.226	3.317	SOTYOT	160.488	3.375
SOVWOS	161.246	3.148	SOVWOS	151.041	3.083	SOVKAS	156.911	3.382	SOVRIG	161.425	3.428	SOVWEF	172.21	3.028
SOVXEJ	156.936	2.923	SOWBIP	154.095	3.405	SOVWUY	152.306	3.101	SOVWUY	162.243	3.295	SOVWUY	153.829	3.188
SOWMOJ	155.286	2.856	SOWMOJ	171.48	2.798	SOWMAS	160.483	3.163	SOWMAS	150.552	3.012	SOWMAS	159.608	2.869
SOWNAU	162.867	3.332	SOWNAU	151.968	3.332	SOWMOJ	156.193	3.415	SOWMOJ	160.859	3.254	SOWNAU	177.736	3.037
SOWNEY	150.734	3.123	SOWNEY	151.375	3.141	SOWNAU	159.884	3.426	SOWNAU	154.243	3.199	SOWNEY	169.016	3.195
SOWPIE	151.249	2.981	SOWPIE	158.684	3.094	SOWNIC	167.519	3.32	SOWNIC	150.314	2.956	SOWPIE	164.587	2.883
SOWQIE	157.93	2.955	SOWQIE	168.537	3.466	SOWPIE	158.098	2.981	SOWQIE	164.055	3.013	SOWQIE	156.433	3.342
SOXDIW	158.308	3.358	SOXDIW	169.577	3.496	SOWRUS	170.743	3.279	SOWRUS	165.864	3.429	SOWVAC	162.674	2.93
SOXDUI	173.862	3.282	SOXFAQ	155.342	2.829	SOXDIW	168.973	3.431	SOXDUI	153.082	2.957	SOXDUI	152.279	3.07
SOXGUK	155.103	2.943	SOXGUK	161.049	2.826	SOXFAQ	165.868	2.829	SOXGOE	167.291	3.21	SOXGOE	154.11	3.452
SOXMAW	151.586	3.031	SOXMAW	160.201	3.49	SOXMAW	155.685	3.345	SOXMAW	151.097	2.906	SOXMAW	153.828	2.608
SOXMEY	150.873	3.174	SOYHIX	165.305	2.85	SOXMAW	160.839	2.608	SOXMAW	152.055	3.494	SOXMEY	156.227	2.922
SOZYAJ	156.061	2.942	SOZYAJ	161.409	3.494	SOZBAO	150.13	2.825	SOZVIO	160.795	2.874	SOZYAJ	171.399	3.438
SUBBID	163.587	2.755	SUBBID	154.18	2.959	SOZYAJ01	161.444	3.494	SOZYAJ01	171.382	3.438	SOZYAJ01	156.149	2.941
SUBHIK	164.33	3.059	SUBHIK	162.894	3.116	SUBBID	168.507	3.429	SUBBID	150.066	2.94	SUBBID	172.025	2.78
SUBYIX	175.576	3.242	SUBYIX	164.605	2.799	SUBHIK	164.535	3.2	SUBSAJ	154.356	3.302	SUBYIX	164.478	3.014
SUBYIX10	175.576	3.242	SUBYIX10	164.605	2.799	SUBYIX	155.283	3.373	SUBYIX	170.62	3.05	SUBYIX10	164.478	3.014
SUBYOD1	165.007	3.353	SUCBUN	161.847	2.814	SUBYIX10	155.283	3.373	SUBYIX10	170.62	3.05	SUBYOD	165.007	3.353
SUCFEB	171.114	2.745	SUCGEC	162.004	3.195	SUCBUN	153.462	3.321	SUCBUN	154.31	3.354	SUCFEB	157.15	2.934
SUDKOR	169.41	3.047	SUDKOR	170.919	3.001	SUCGEC	165.386	3.055	SUCGEC	164.2	2.83	SUCHOQ	165.195	3.058
SUFCOL	170.052	3.459	SUFCOL	165.337	3.022	SUDQEQ	163.486	3.264	SUDXOH	151.738	2.929	SUFCOL	163.629	3.185
SUFHOQ	165.189	2.822	SUFHOQ0	165.135	3.031	SUFCOL	166.313	3.363	SUFCOL	152.469	3.449	SUFHOQ	167.192	2.95
SUFSOB1	163.322	2.778	SUFSOB1	156.835	3.08	SUFHOQ0	167.009	2.925	SUFLEM	165.471	3.392	SUFSOB1	159.735	3.388
SUGDAD	151.316	3.361	SUGDAD	153.36	3.311	SUFWUP	150.225	3.333	SUFWUP	151.699	3.377	SUFXAV	150.948	2.796
SUHCEE	152.92	2.716	SUHCEE	153.893	3.339	SUGTIX	155.56	3.262	SUGVAT	156.777	2.93	SUHCEE	161.109	2.952
SUHCEE	160.772	3.492	SUHCEE	170.937	3.087	SUHCEE	153.287	2.84	SUHCEE	150.348	3.023	SUHCEE	154.181	2.957
SUHFUX	160.984	3.185	SUHFUX	167.526	3.181	SUHFAD	156.664	2.851	SUHFAD	156.606	2.851	SUHFIL	164.089	3.421

SUHGAE	153.053	3.465	SUHGAE	155.185	3.44	SUHFUX	156.997	3.338	SUHFUX	150.786	3.191	SUHFUX	168.915	3.181
SUHJAH	161.758	3.05	SUHLEQ	169.62	3.227	SUHGUY	158.564	2.905	SUHHIP	150.691	3.401	SUHHIP	169.141	3.1
SUHREW	150.958	3.301	SUHROG	172.458	3.46	SUHPEQ	151.801	3.447	SUHPEQ	160.009	3.296	SUHRAS	153.045	3.256
SUHVUP	175.804	3.388	SUHVUQ	160.214	2.923	SUHSAR	166.362	3.462	SUHSIB	154.971	2.76	SUHVOK	153.276	3.043
SUJHOY	167.26	3.133	SUJHOY	166.256	3.149	SUHXUP	158.219	3.168	SUHZEA	161.554	3.257	SUJHOY	151.172	3.284
SUJJEM	155.414	3.195	SUJJEM	164.381	3.099	SUJHOY	177.651	3.09	SUJHOY	170.728	3.172	SUJJEM	153.361	3.049
SUKDIL	151.301	3.321	SUKDIL	162.309	3.192	SUJPOF	169.898	3.067	SUJPOF	155.212	2.957	SUKBIN	158.411	3.116
SUKDIL	162.525	3.246	SUKDIL	151.82	3.06	SUKDIL	164.587	3.464	SUKDIL	168.222	3.066	SUKDIL	158.56	3.192
SUKFAI	168.175	3.18	SUKFAI	151.984	2.778	SUKDIL	150.02	2.959	SUKFAI	153.989	3.13	SUKFAI	150.513	3.339
SUKWON	159.108	3.297	SUKWON	166.38	3.303	SUKFAI	157.028	3.024	SUKPEX	153.077	3.213	SUKTIC	151.758	3.306
SUKXIG	160.778	2.89	SUKXIG	161.948	3.152	SUKXEE	158.344	3.113	SUKXEE	160.177	3.064	SUKXIG	175.506	3.346
SUKXIG	154.828	2.977	SUKXIG	161.31	3.02	SUKXIG	169.195	3.022	SUKXIG	162.528	3.145	SUKXIG	153.721	2.977
SUKXIG	150.155	3.146	SULBAD	156.331	3.005	SUKXIG	152.146	3.307	SUKXIG	156.827	2.943	SUKXIG	167.003	3.153
SULFIO	173.614	2.87	SULFUD	160.509	3.005	SULBAD	175.148	2.629	SULBAD	150.826	3.192	SULFIO	168.96	2.87
SULFUD	170.962	3.184	SULFUD	165.104	3.246	SULFUD	155.833	3.281	SULFUD	174.615	3.457	SULFUD	150.296	3.404
SULKET	174.053	2.992	SULKET	161.901	3.306	SULHOY	164.948	2.979	SULHOY	151.872	3.39	SULHOY	157.431	2.979
SULSUO	163.72	2.701	SULTAV	170.221	3.128	SULLIY	159.897	2.851	SULNIA	174.818	3.449	SULPUL	160.037	3.424
SUMPOG	174.084	2.944	SUMPOG	169.068	3.439	SULTAV	172.757	3.376	SUMBEL	158.504	2.936	SUMPOG	167.279	2.944
SUMPUM	155.713	2.864	SUMPUM	152.938	3.403	SUMPOG	175.317	3.357	SUMPOG	153.207	3.465	SUMPUM	163.525	2.877
SUMRIF	157.269	3.227	SUMRUR	153.948	2.808	SUMPUM	151.354	3.41	SUMPUM	154.613	2.873	SUMRIF	158.229	2.96
SUNGEP	153.074	2.925	SUNJAP	160.495	2.93	SUMRUR	154.34	2.921	SUMRUR	165.293	2.868	SUMRUR	159.586	3.163
SUNZIN	157.042	3.201	SUNZIN	155.172	3.061	SUNLOG	159.576	2.976	SUNPID	153.468	2.977	SUNVIH	162.001	3.333
SUPBIP	159.37	3.274	SUPBIP	160.359	3.416	SUNZIN	150.696	3.411	SUNZOT	152.966	3.21	SUPBIP	166.762	3.302
SUPKAP	157.01	3.369	SUPSUV	168.914	3.494	SUPBIP	165.982	2.882	SUPBIP	159.148	3.084	SUPJAR	154.725	3.202
SUPZUC	154.658	3.441	SUPZUC	167.656	2.588	SUPSUV	162.926	2.989	SUPYIN	156.21	2.905	SUPZUC	169.781	2.588
SUQPUQ	150.924	3.034	SUQPUQ	173.126	2.711	SUPZUC	172.049	3.333	SUQPUQ	152.95	2.996	SUQPUQ	167.191	2.999
SUQPUQ	170.69	3.45	SUQSEE	165.01	3.3	SUQPUQ	154.373	2.99	SUQPUQ	159.128	3.307	SUQPUQ	151.37	3.088
SUQTOQ	163.144	3.402	SUQWEK	160.91	3.302	SUQSEE	159.206	2.788	SUQSEE	176.013	3.16	SUQSEE	168.024	3.297
SURCOY	169.524	3.361	SURHAQ	165.925	3.043	SUQWOU	160.708	3.399	SURBEQ	150.227	3.015	SURCOY	153.874	3.447
SURHAQ	170.965	3.233	SURHAQ	150.332	3.324	SURHAQ	151.326	2.941	SURHAQ	155.715	2.716	SURHAQ	154.138	2.81
SURPUQ	150.092	2.92	SURPUQ	154.536	2.74	SURHAQ	171.158	3.064	SURHAQ	170.433	3.064	SURHAQ	154.37	2.795
SURQII	172.052	3.497	SURQII	150.184	3.499	SURQAX	172.977	3.189	SURQAX	157.454	3.319	SURQAX	169.009	3.189
SURXAG	152.092	3.012	SURXEK	163.402	3.145	SURSUU	153.595	3.025	SURWUZ	153.896	3.102	SURXAG	154.245	3.175
SURXUA	164.188	3.014	SURXUA	155.202	3.04	SURXIO	166.223	3.258	SURXOU	166.073	2.736	SURXOU	160.916	3.065
SURXUA	176.376	3.175	SURYAH	166.348	2.894	SURXUA	160.249	3.235	SURXUA	156.969	3.145	SURXUA	155.957	2.727
SUSJUP	156.424	2.919	SUSJUP	153.944	2.878	SURYEL	160.618	3.274	SUSBOZ	172.436	3.349	SUSBOZ	173.998	3.461
SUSLIE	158.815	3.302	SUSLIE	150.332	3.341	SUSLIE	164.608	3.015	SUSLIE	152.768	2.799	SUSLIE	154.981	2.757
SUSLIE	158.282	3.037	SUSLOK	165.426	2.947	SUSLIE	160.233	3.376	SUSLIE	150.718	3.038	SUSLIE	160.532	3.116
SUSLOK	154.396	2.943	SUSLOK	173.023	3.009	SUSLOK	161.303	3.041	SUSLOK	161.299	3.22	SUSLOK	156.577	3.287
SUSMIF	152.822	2.825	SUSMIF	154.83	2.75	SUSLUQ	171.627	3.344	SUSLUQ	154.698	3.167	SUSLUQ	163.719	2.934
SUSMIF	158.349	3.256	SUSMIF	150.3	3.031	SUSMIF	160.471	3.108	SUSMIF	163.27	3.13	SUSMIF	156.771	3.032
SUSTAD	153.369	3.225	SUSTAD	154.585	2.957	SUSMIF	152.414	3.321	SUSMIF	161.267	3.467	SUSREG	156.535	3.497
SUSYEL	177.408	3.293	SUSYEL	155.048	3.033	SUSVAH	157.744	2.958	SUSYEL	158.337	3.163	SUSYEL	163.086	3.052
SUTKAX	172.105	3.174	SUTKEA	159.045	2.957	SUSYEL	173.064	3.293	SUSYEM	170.995	2.97	SUSYEM0	170.797	2.929
SUTKIE	160.753	3.25	SUTKIE	154.094	3.022	SUTKEA	168.946	3.007	SUTKEA	164.455	3.137	SUTKEA	155.728	3.265
SUTROR	150.166	2.776	SUTROR	162.03	3.247	SUTKIE	153.843	3.078	SUTKOI	155.138	2.825	SUTROR	156.365	2.962
SUTROR	161.398	2.854	SUTROR	163.183	3.428	SUTROR	153.067	3.119	SUTROR	164.097	3.196	SUTROR	161.424	2.829
SUTWUA	152.814	3.2	SUTWUA	159.948	2.917	SUTVEM	154.29	3.256	SUTWUA	154.95	3.405	SUTWUA	157.489	3.479
SUTWUA	174.199	2.655	SUTWUA	150.687	2.705	SUTWUA	156.752	3.092	SUTWUA	174.083	2.82	SUTWUA	161.166	3.065
SUVQOQ	166.236	3.341	SUVRAD	157.023	3.495	SUTWUA	164.63	2.994	SUVFIY	178.719	2.985	SUVQOQ	159.783	3.173
SUVRAD	151.874	3.147	SUVRAD	177.599	3.463	SUVRAD	170.988	3.077	SUVRAD	169.256	3.463	SUVRAD	170.755	2.807
SUVRAD	155.332	2.991	SUVREH	169.947	3.107	SUVRAD	168.855	2.991	SUVRAD	152.792	3.244	SUVRAD	171.119	2.807
SUVREH	177.833	3.47	SUVREH	166.666	3.028	SUVREH	169.52	3.47	SUVREH	169.575	2.791	SUVREH	150.425	3.091
SUVRIL	150.443	3.479	SUVROR	150.332	3.46	SUVREH	152.106	3.196	SUVREH	171.881	2.783	SUVREH	156.648	3.028
SUVZOB	162.219	3.346	SUVZOB	170.076	3.107	SUVXOX	159.171	3.153	SUVZOB	150.777	2.991	SUVZOB	152.811	2.974
SUWBAQ	151.415	3.369	SUWCOF(	160.156	2.847	SUWBAQ	166.546	3.498	SUWBAQ	170.387	2.875	SUWBAQ	171.717	3.259
SUWCOF(	166.773	2.998	SUWCOF(	160.344	2.843	SUWCOF(	159.796	3.225	SUWCOF(	171.358	3.42	SUWCOF(	158.379	3.405
SUWCOF(	166.726	3.002	SUWGOJ	152.391	3.071	SUWCOF(	159.879	3.216	SUWCOF(	171.336	3.419	SUWCOF(	158.252	3.408
SUWGOJ	156.565	3.315	SUWGOJ	170.719	2.75	SUWGOJ	157.692	3.297	SUWGOJ	153.591	2.909	SUWGOJ	177.183	2.755
SUWJUR	156.178	3.47	SUWJUR	164.96	3.413	SUWGOJ	160.932	3.346	SUWJID	156.083	3.015	SUWJID	162.78	3.015
SUWKIG	151.521	2.882	SUWKIG	150.886	2.86	SUWJUR	151.94	2.885	SUWJUR	161.923	3.049	SUWKIG	154.637	3.285
SUWLIH	152.183	3.228	SUWLIH	152.108	3.028	SUWKOM	152.196	3.159	SUWKUS	154.51	2.869	SUWLED	153.237	2.983
SUWMAA	153.937	3.349	SUWPOP	151.977	3.32	SUWLUT	164.883	3.477	SUWLUT	174.215	2.844	SUWMAA	154.181	2.935
SUXFAS	154.202	3.421	SUXFEW	159.877	3.022	SUWPOQ	161.075	3.294	SUWWOA	153.693	2.799	SUXDUL	154.558	3.103
SUXFEW	154.181	2.844	SUXFEW	159.647	2.949	SUXFEW	159.385	3.191	SUXFEW	154.086	3.193	SUXFEW	173.624	2.844
SUXJEE	167.167	2.668	SUXKEF	154.848	2.885	SUXFEW	173.606	3.023	SUXGUQ	154.47	2.732	SUXJEE	154.02	2.668
SUYDAS	157.05	2.685	SUYDAS	161.313	3.487	SUXROS	155.718	3.45	SUYDAS	150.283	3.412	SUYDAS	159.849	3.225
SUYMEI	162.314	2.912	SUYYEQ	159.423	3.304	SUYDEZ	157.099	3.321	SUYDEZ	155.449	2.839	SUYFIF	167.996	3.361
SUYYEQ	166.373	3.037	SUZCUN	158.913	2.929	SUYYEQ	172.352	3.105	SUYYEQ	155.674	3.304	SUYYEQ	153.906	3.242
TABBAC	159.472	2.938	TABCIJ	150.372	3.346	SUZCUN	168.963	2.929	SUZRAK	162.784	3.477	SUZSUB	158.607	3.425
TABCOP	150.725	3.219	TABCOP	163.567	3.149	TABCIJ	157.975	3.415	TABCOP	159.403	3.039	TABCOP	157.737	2.857
TABNAQ	169.242	3.466	TABNAQ	154.849	2.944	TABCOP	164.409	2.898	TABCOP	157.592	3.212	TABCOP	157.726	3.366
TABNIU	156.421	3.328	TABNIU	150.83	3.076	TABNIU	159.346	3.194	TABNIU	161.901	3.485	TABNIU	165.488	3.453
TABPAS0	156.016	3.421	TABSAT	160.937	3.42	TABPAS0	151.015	3.28	TABPAS0	150.544	3.094	TABPAS0	152.164	3.072
TACFUY	157.65	3.339	TACHAI	155.716	3.03	TABSAT	154.62	3.036	TABWED	172.33	3.075	TABXUR	151.072	3.025
TADJEP	156.286	3.085	TADJEP	165.34	3.435	TACXIE	154.221	3.318	TACYEF	166.635	3.464	TADJEP	153.619	3.462
TADPAP	176.907	3.382	TADPAP	157.406	3.357	TADJEP	156.13	3.419	TADMAQ	162.526	2.922	TADMAQ	156.028	2.922
TADZUX	166.271	3.085	TAFACU	153.134	3.052	TADROJ	172.456	3.085	TADROJ	155.618	3.085	TADVAX	173.091	2.98
TAFBAH	162.333	2.866	TAFBAH	159.263	3.049	TAFBAH	151.489	2.696	TAFBAH	161.993	2.696	TAFBAH	151.165	2.866

TAFCIO	163.786	3.378	TAFCIO	159.301	3.139	TAFBAH	151.014	3.487	TAFBAH	156.087	3.497	TAFCIO	162.595	2.944
TAFDOU	159.967	2.909	TAFQOJ	166.408	3.381	TAFCIQ	158.242	3.019	TAFDOU	160.834	2.909	TAFDOU	172.352	3.416
TAFSOM	170.323	3.364	TAFSOM	167.457	3.024	TAFREB	174.761	3.033	TAFREY	156.002	2.92	TAFRIC	153.576	2.939
TAFVIG	150.808	3.146	TAFVIG	158.453	2.84	TAFSOM	154.85	2.794	TAFSOM	157.201	2.763	TAFSOM	161.445	3.024
TAFVOM	150.634	3.35	TAFVOM	167.635	3.097	TAFVIG	176.747	2.807	TAFVIG	158.816	3.5	TAFVIG	150.555	2.829
TAFWED	175.646	2.848	TAFWED	151.576	3.156	TAFVOM	154.062	3.395	TAFVOM	164.164	3.266	TAFVOM	150.573	3.444
TAGBIP	169.993	3.091	TAGDOW	155.501	2.964	TAFWED	161.168	2.841	TAFWED	151.837	2.838	TAGBIP	151.096	3.492
TAGKIW	150.902	3.181	TAGKIW	154.565	3.474	TAGDOW	167.169	2.971	TAGKIW	152.589	3.483	TAGKIW	166.875	3.073
TAGKUI	151.906	3.459	TAGKUI	158.124	3.152	TAGKUI	161.926	3.077	TAGKUI	173.224	2.799	TAGKUI	151.078	3.421
TAGPEA	160.618	2.953	TAGPEA	157.779	2.681	TAGMAS0	176.007	2.702	TAGPEA	150.593	3.057	TAGPEA	157.848	3.043
TAGPEA	176.325	3.152	TAGPEA	166.062	3.199	TAGPEA	171.506	2.92	TAGPEA	158.044	2.893	TAGPEA	163.012	2.62
TAGPEA	153.805	3.192	TAGPEA	150.639	2.811	TAGPEA	153.139	2.811	TAGPEA	150.258	3.085	TAGPEA	161.967	2.867
TAGPEA	174.777	3.014	TAGPEA	162.354	2.813	TAGPEA	160.392	2.59	TAGPEA	151.128	3.062	TAGPEA	175.117	2.649
TAGPEA	157.557	2.899	TAGPEA	156.035	2.738	TAGPEA	156.064	3.105	TAGPEA	155.548	3.205	TAGPEA	162.471	2.785
TAGPEA	163.919	3.353	TAGTOP	152.452	2.997	TAGPEA	171.821	2.649	TAGPEA	165.415	3.138	TAGPEA	156.225	2.905
TAGVEH	160.805	3.121	TAGVEH	176.347	3.489	TAGTOP	151.503	2.814	TAGTOP	162.016	2.813	TAGVEH	169.605	3.322
TAGXUU0	180	3.412	TAGXUU0	174.362	3.209	TAGVEH	170.022	2.936	TAGXUU0	180	3.412	TAGXUU0	166.065	3.23
TAGYAC	165.316	2.964	TAGZIL	175.782	3.444	TAGYAC	173.618	2.866	TAGYAC	157.892	3.482	TAGYAC	165.58	3.191
TAHQIG	165.99	3.384	TAHTAA01	155.904	3.169	TAGZOR	161.856	3.148	TAHMOH	161.768	3.423	TAHMOH	154.322	3.463
TAHYUX	160.956	3.441	TAHYUX	158.387	3.277	TAHVEE	153.537	3.354	TAHVUU	162.839	3.079	TAHYUX	157.778	2.904
TAJGUI	165.62	3.182	TAJGUI	165.833	3.213	TAJGUH	164.147	3.042	TAJGUI	161.015	3.337	TAJGUI	155.717	2.734
TAJSOR	150.544	3.33	TAJYEI	180	3.082	TAJGUI	165.363	3.182	TAJSOR	152.845	3.045	TAJSOR	163.859	2.696
TAKDOB	169.266	3.243	TAKFEQ	150.885	3.04	TAKCUI	152.514	3.257	TAKDES	154.423	3.35	TAKDOB	166.59	3.017
TAKRAZ	155.279	2.941	TAKRAZ	166.273	3.148	TAKJAU	165.511	3.036	TAKPIJ	158.927	2.959	TAKPOP	174.501	3.388
TAKROQ	156.636	3.334	TAKTEE	153.705	3.32	TAKRAZ	169.439	2.941	TAKRAZ	158.301	3.46	TAKRIL	163.089	3.004
TAKZAK02	162.231	3.479	TAKZUE	160.625	3.096	TAKTEJ	163.113	3.224	TAKVUY	172.766	2.906	TAKZAK	162.366	3.461
TALPEC	155.511	3.132	TALPEC	155.588	3.47	TALCET	156.13	2.971	TALCIX	157.74	2.967	TALDAQ	170.695	3.075
TALPEF	153.34	2.99	TALPIJ	157.626	3.326	TALPEC	154.588	3.132	TALPEC	163.35	2.942	TALPEC	168.238	3.334
TALPIJ	166.788	3.021	TAMBIT	158.781	2.544	TALPIJ	158.629	2.859	TALPIJ	159.729	3.357	TALPIJ	152.324	3.032
TAMGUJ	151.142	3.372	TAMHIY	163.586	2.944	TAMBIT	154.275	2.798	TAMBIU	161.458	3.365	TAMGUJ	153.457	3.316
TAMRUY	156.104	3.242	TAMRUY	152.226	2.891	TAMLUO	156.055	2.856	TAMRUY	150.741	3.369	TAMRUY	159.581	3.369
TAMTEK	162.99	2.735	TAMXIO	160.123	3.007	TAMSOT	151.935	2.535	TAMSOT	151.224	3.27	TAMSUZ	164.622	3.412
TAMXIO	157.365	3.296	TAMXIO	161.665	2.917	TAMXIO	169.577	3.357	TAMXIO	151.686	3.438	TAMXIO	160.304	3.282
TAMXIO10	160.304	3.282	TAMXIO10	157.364	3.296	TAMXIO10	160.125	3.007	TAMXIO10	169.577	3.357	TAMXIO10	151.686	3.438
TANFIB	151.436	3.276	TANFOH	155.006	3.316	TAMXIO10	161.665	2.917	TAMZAK	166.199	3.471	TAMZAK	150.059	3.135
TANLED	158.433	2.751	TANLED	157.192	3.129	TANFOH	164.237	2.961	TANLED	151.647	2.92	TANLED	150.616	3.428
TANZUH	179.248	2.818	TANZUH	172.701	2.951	TANXAL	162.978	3.335	TANZUH	161.994	2.869	TANZUH	152.612	3.226
TAPLOM	160.229	3.323	TAPLOM	167.771	2.896	TAPGIA	175.868	3.098	TAPLOM	158.865	3.419	TAPLOM	166.868	3.191
TAPNIH	173.132	3.357	TAPPUV	170.025	3.3	TAPLOM	158.804	2.708	TAPLOM	160.502	2.868	TAPLOM	163.734	2.812
TAPQAF	168.863	3.414	TAPQAF	163.374	3.069	TAPPUV	167.772	3.018	TAPPUV	174.429	3.203	TAPPUV	159.781	3.274
TAPRIP	155.827	2.984	TAPRIP	178.66	3.047	TAPRAD	150.659	3.125	TAPRAD	175.858	3.244	TAPRIP	157.512	3.417
TAPROV	159.441	3.462	TAPRUB	169.512	2.736	TAPROV	165.666	3.311	TAPROV	156.592	2.726	TAPROV	159.622	3.171
TAPTUB	157.023	2.708	TAPTUB	151.64	2.915	TAPSOW	152.531	3.092	TAPTUB	159.485	3.194	TAPTUB	150.862	2.733
TAPYAN	177.787	2.929	TAPYAN	168.09	2.757	TAPTUB	160.721	3.228	TAPWUE	153.696	3.285	TAPWUE	156.17	3.07
TAQFUN0	171.54	3.442	TAQNAC	154.334	3.328	TAQFUN0	168.533	3.268	TAQFUN0	172.534	2.891	TAQFUN0	156.084	3.009
TARTAH	157.452	3.423	TARTIQ	170.55	3.077	TARHED	165.348	2.87	TARQIN	156.746	3.334	TARTAH	164.476	3.495
TARVAN	158.602	3.422	TASGAX	154.436	2.858	TARVAN	152.448	3.422	TARVAN	159.209	2.982	TARVAN	152.769	3.006
TASJEE	170.802	3.116	TASMOS	159.24	3.453	TASJEE	165.023	3.043	TASJEE	160.774	3.362	TASJEE	165.526	3.09
TASNIL	159.249	3.49	TASNIL	159.432	2.837	TASMOS	171.109	3.364	TASNIL	154.682	3.419	TASNIL	153.243	2.735
TASNUX	164.186	3.022	TASPAF	165.18	3.489	TASNIL	164.42	2.763	TASNIL	157.548	3.253	TASNOU	160.561	3.255
TASPAF	163.391	3.425	TASPAF	152.696	3.419	TASPAF	152.658	3.049	TASPAF	173.292	3.168	TASPAF	171.171	3.193
TASYOF	163.19	3.461	TATQUC	158.445	2.932	TASPAF	165.91	3.375	TASTAI	163.661	3.427	TASTAI01	164.02	3.453
TATRUC	159.566	2.95	TATRUC	154.875	3.239	TATRUC	157.61	2.914	TATRUC	154.271	3.494	TATRUC	151.841	3.248
TATWEU	150.304	2.844	TATWOE	162.572	3.066	TATRUC	158.657	3.22	TATRUC	157.754	3.274	TATRUC	156.806	2.84
TATZAT	158.309	3.168	TATZAT	161.622	3.086	TATWOE	171.492	2.667	TATXOF	171.988	2.685	TATXOF	161.725	3.07
TAVDAV	154.062	3.025	TAVFEC	165.49	2.806	TATZAT	152.579	2.991	TATZAT	164.714	3.47	TATZAT	154.077	2.991
TAVXOH	151.624	2.83	TAWCUS	152.666	3.287	TAVWOC	150.43	3.226	TAVWOC	169.929	3.481	TAVXOH	152.796	2.888
TAWLOU	162.534	3.18	TAWLOU	172.221	2.591	TAWFAB	161.558	2.559	TAWFAB	167.798	3.457	TAWHAD	152.011	3.145
TAWMIQ	150.07	2.779	TAWYID01	151.835	3.149	TAWMIQ	168.955	2.829	TAWMIQ	167.185	2.856	TAWMIQ	153.01	2.771
TAXHIM	154.897	3.211	TAXHIM	158.037	2.85	TAWYID01	152.485	3.149	TAWYID01	152.286	3.181	TAXDOP	151.775	3.453
TAXHOP	166.714	3.334	TAXHOP	157.313	2.869	TAXHIM	155.153	3.314	TAXHIM	162.944	3.169	TAXHIM	151.212	3.496
TAXKUB	162.457	2.892	TAXQUG	178.354	3.223	TAXKUB	163.492	2.844	TAXKUB	159.683	3.321	TAXKUB	155.447	2.892
TAXYUP	166.414	2.689	TAYCUS	172.449	3.047	TAXTEU02	163.408	3.073	TAXTEU02	162.281	2.956	TAXYAS	176.362	3.082
TAYDAB	159.182	3.233	TAYDUT	163.873	3.107	TAYDAB	157.712	2.81	TAYDAB	162.013	3.421	TAYDAB	164.486	3.124
TAYXOH	166.32	2.877	TAYXOH	159.983	2.954	TAYDUT	172.135	3.422	TAYDUT	158.208	3.445	TAYKUA	157.214	2.919
TEBKUK	153.423	3.308	TEBSOI	166.267	2.788	TAZNOA	169.487	3.479	TAZNOA	151.496	2.842	TEBKUK	173.999	3.471
TEBSUO	160.334	3.07	TEBSUO	165.245	3.167	TEBSUO	172.318	3.148	TEBSUO	177.392	3.15	TEBSUO	172.197	3.411
TEBTAV	159.364	2.986	TEBTAV	156.578	2.883	TEBTAV	174.347	3.387	TEBTAV	153.215	3.449	TEBTAV	166.195	2.829
TEBTAV	150.21	3.035	TEBTAV	156.222	3.002	TEBTAV	165.007	3.229	TEBTAV	162.417	3.489	TEBTAV	164.992	3.075
TEBTAV	172.601	2.829	TEBTEZ	158.646	2.521	TEBTAV	176.751	2.942	TEBTAV	167.993	3.37	TEBTAV	169.485	3.035
TEBWED	165.743	3.411	TEBWIH	168.146	3.085	TEBTEZ	159.073	3.49	TEBWED	152.028	3.329	TEBWED	158.563	3.202
TEBWUW	154.508	2.847	TEBWUW	155.236	2.855	TEBWIH	154.241	2.942	TEBWIH	153.735	3.319	TEBWIH	153.485	3.364
TEBYOR	172.071	3.208	TEBYOR	157.354	3	TEBYOR	154.097	3.191	TEBYOR	162.519	2.92	TEBYOR	158.645	3.098
TECFEO	162.958	3.326	TECGOX	157.728	3.459	TECCEL	166.982	2.727	TECCEL	169.519	2.727	TECFEO	171.464	3.046
TECQUQ	156.469	2.665	TECQUQ	151.518	3.212	TECGOX	162.586	3.353	TECQUQ	159.099	3.122	TECQUQ	164.816	2.631
TECZIN	163.061	3.197	TECZOT	153.572	3.403	TECQUQ	151.191	2.966	TECWEF	157.516	3.403	TECZIN	154.532	3.197
TEDVUT	152.842	2.874	TEDWEH	151.989	3.169	TEDFIR	171.958	2.978	TEDKAP	164.913	2.889	TEDSEC	173.788	3.349
TEFDIT	159.002	3.153	TEFDIT	155.677	3.142	TEDZIM	152.505	2.957	TEFDIT	155.469	2.956	TEFDIT	153.374	2.964
TEFKAT	151.264	2.879	TEFKEX	155.327	2.858	TEFKAT	160.341	3.318	TEFKAT	168.339	2.879	TEFKAT	151.008	3.096

TEFKOH	176.563	3.458	TEFNUN	150.893	3.37	TEFKEX	153.67	3.251	TEFKEX	173.138	3.129	TEFKEX	172.144	3.234
TEGDAK	164.288	2.909	TEGDOA	165.09	2.871	TEFPAV	160.016	2.996	TEFPAX	162.767	2.806	TEFPIF	159.557	3.416
TEGKIB	151.775	3.413	TEGKUN	164.652	2.66	TEGFOC	162.35	2.903	TEGGET	156.16	3.349	TEGKIB	156.842	3.413
TEGKUN	156.072	3.241	TEGKUN	159.581	3.271	TEGKUN	159.183	3.345	TEGKUN	160.204	3.071	TEGKUN	159.12	2.788
TEGLEY	156.769	3.204	TEGLEY	166.035	3.286	TEGKUN	155.97	3.064	TEGKUN	161.087	3.314	TEGKUN	156.862	3.443
TEGNID	157.895	3.137	TEGPAW	155.185	3.394	TEGMAV	161.07	3.229	TEGMAV	150.503	3.424	TEGMAV	155.243	3.062
TEGRAC	152.844	3.441	TEGRAC	160.017	2.964	TEGRAC	157.52	2.884	TEGRAC	162.804	2.812	TEGRAC	155.872	3.065
TEGTAB	153.426	2.994	TEGTAB	159.594	3.088	TEGSAB	170.27	2.968	TEGSAB	162.86	2.926	TEGSIL	162.188	3.293
TEGTIJ	155.562	3.131	TEGTIJ	160.858	2.91	TEGTAB	173.621	2.94	TEGTIJ	152.583	2.842	TEGTIJ	156.851	3.393
TEGXOT	152.527	2.654	TEGXOW	151.319	3.123	TEGXOT	159.559	2.654	TEGXOT	163.583	2.874	TEGXOT	176.899	3.029
TEGXOW	152.671	3.308	TEGYEK	158.567	3.175	TEGXOW	165.365	3.491	TEGXOW	166.886	3.09	TEGXOW	154.532	3.101
TEGYOX	170.018	3.289	TEGYOX	152.76	3.47	TEGYEK	156.262	2.884	TEGYEK	154.498	3.159	TEGYIO	158.112	3.286
TEGZUE	156.163	3.034	TEHGAQ	153.796	2.888	TEGYOX	152.106	3.389	TEGYOX	176.358	3.183	TEGYOX	154.693	3.055
TEHJOH	167.786	3.404	TEHJOH	151.44	3.22	TEHGAQ	162.05	2.86	TEHJID	154.273	3.102	TEHJID	154.83	2.876
TEHNIF	167.009	2.946	TEHNOM	150.213	3.165	TEHMAU	162.806	3.075	TEHMOI	172.833	2.854	TEHNIF	174.576	3.106
TEHTOP	150.486	3.229	TEHTOP	150.387	3.119	TEHPEC	163.623	3.157	TEHRAA	163.741	2.939	TEHRAA	166.801	3.062
TEHTOP	153.562	2.926	TEHZAJ	170.42	2.8	TEHTOP	153.102	3.263	TEHTOP	152.597	3.119	TEHTOP	153.399	3.141
TEJNOP	164.699	2.91	TEJNOP	164.843	2.91	TEJHIC	152.72	3.294	TEJHOG	159.589	3.185	TEJJUQ	161.865	3.196
TEJPUW	153.517	3.457	TEJQAD	158.139	3.239	TEJPOQ	159.71	3.364	TEJPUW	154.901	3.112	TEJPUW	161.033	3.482
TEJWUA	172.165	2.916	TEKFIC	153.153	3.224	TEJWEK	158.081	3.4	TEJWEK	151.794	3.178	TEJWUA	172.05	2.863
TEKJOI	161.249	3.055	TEKQOP	150.886	2.941	TEKFOI	153.72	3.086	TEKFOI	152.069	3.276	TEKJOI	165.146	3.055
TEKQOP	152.439	3.107	TEKQOP	151.531	3.437	TEKQOP	161.572	3.07	TEKQOP	159.289	3.107	TEKQOP	165.461	3.354
TEKRUA	174.56	3.018	TELFID	152.88	3.228	TEKQOP	157.385	3.072	TEKQOP	153.791	3.448	TEKQOP	155.579	3.241
TELTIR	168.144	3.126	TELTIR	161.972	3.382	TELRAD	166.181	3.025	TELRAD	162.877	3.139	TELSEI	174.701	3.209
TEMCIZ	163.254	2.813	TEMGUQ	162.003	3.275	TELVIS	159.015	3.199	TELVIS	164.486	3.199	TELVIS	166.987	3.264
TEMREM	167.327	3.3	TEMREM	168.269	3.271	TEMJON	167.318	3.386	TEMJUT	166.508	3.122	TEMREM	175.261	2.668
TEMTEK	166.979	2.972	TEMTEK	165.659	3.334	TEMREM	155.154	3.293	TEMREM	160.587	2.76	TEMREM	159.275	3.413
TEMTEK	163.466	3.149	TEMTEK	167.334	3.261	TEMTEK	166.607	2.998	TEMTEK	158.821	2.839	TEMTEK	160.967	3.2
TEMTEK	161.598	3.404	TEMTEO	154.765	3.39	TEMTEK	155.607	3.072	TEMTEK	158.649	3.314	TEMTEK	154.18	3.39
TEMTOU	156.082	3.131	TEMTOU	159.687	3.296	TEMTEO	154.64	3.357	TEMTOU	150.613	3.465	TEMTOU	160.093	3.043
TENHAY	175.756	3.046	TENHAY	162.317	2.787	TEMTOU	150.059	3.386	TEMVEP	157.898	3.029	TEMVEP	169.968	3.137
TENYEU	163.043	2.696	TEPGAZ	158.895	3.494	TENHAY	175.503	3.029	TENJUT	150.122	3.444	TENTAK	172.109	2.957
TEPGAZ	157.107	3.222	TEPGAZ	166.663	3.325	TEPGAZ	169.32	3.038	TEPGAZ	170.984	2.746	TEPGAZ	163.447	3.386
TEPGON	158.463	3.369	TEPLEJ	153.755	3.358	TEPGED	163.45	3.305	TEPGIH	150.334	3.36	TEPGIH	166.343	2.989
TEPMIO	171.616	3.022	TEPPUZ	158.274	3.356	TEPLEJ	152.872	3.192	TEPLEJ	151.507	3.162	TEPMAG	169.829	3.024
TEPSEO	150.257	2.832	TEPTAM	155.992	3.357	TEPPUZ	154.315	3.347	TEPPUZ	165.906	3.326	TEPREP	159.794	3.292
TEPWEQ	167.816	2.841	TEPWEQ	159.928	3.007	TEPTEQ	152.274	3.498	TEPVUH	159.383	3.421	TEPWEQ	150.279	3.246
TEPYEW	164.878	2.972	TEPZOH	152.084	3.28	TEPWEQ	158.143	3.003	TEPWES	157.882	3.33	TEPYEW	150.947	3.477
TEQBEZ	150.584	3.184	TEQBEZ	161.823	3.14	TEPZOH0	152.202	3.305	TEPZOH0	152.127	3.342	TEQBEZ	154.158	3.079
TEQHOQ	159.322	3.135	TEQLUX	167.317	3.143	TEQDUR	170.358	2.641	TEQDUR	170.968	3.268	TEQDUR	164.017	3.007
TEQSAM	150.708	3.135	TEQSUE	162.585	3.384	TEQLUX	167.547	3.209	TEQLUX	165.734	3.308	TEQSAM	163.937	2.73
TEQSUE	159.916	2.902	TEQSUE	169.312	2.786	TEQSUE	157.198	3.068	TEQSUE	150.549	3.169	TEQSUE	154.574	2.827
TERHUW	156.186	3.162	TERHUW	160.813	2.729	TEQSUE	155.451	2.902	TEQWAO	151.114	3.24	TERHUW	171.073	2.854
TERJAE	177.256	3.271	TERJAE	167.336	3.239	TERHUW	151.687	3.099	TERHUW	158.978	2.758	TERHUW	161.299	3.128
TERJAE	172.812	3.119	TERJIK	152.796	2.784	TERJAE	153.351	2.817	TERJAE	153.487	3.336	TERJAE	153.681	2.947
TERNEM	165.36	3.278	TERNEM	150.378	3	TERJIK	153.215	3.404	TERKAD	167.254	3.086	TERMEL	159.298	3.423
TERPAH	154.483	3.432	TERPEL	156.678	3.362	TERPAH	161.802	3.093	TERPAH	157.564	3.151	TERPAH	168.107	2.941
TERVAR	164.618	3.428	TERVAR	166.01	2.989	TERQUF	153.123	3.135	TERQUF0	172.818	3.099	TERQUF0	166.864	3.244
TERVEV	152.551	2.878	TERVEV	158.088	2.599	TERVAR	150.641	3.071	TERVAR	163.08	3.429	TERVEV	162.982	3.388
TERVOF	154.011	2.967	TERWOG	155.69	2.965	TERVEV	161.895	3.328	TERVOF	168.801	2.82	TERVOF	173.167	3.178
TERWOG	167.728	2.861	TERWOG	162.821	3.473	TERWOG	156.266	2.986	TERWOG	154.728	3.066	TERWOG	153.189	3.382
TERWOG	156.412	3.369	TERWOG	157.327	2.844	TERWOG	155.839	3.319	TERWOG	164.782	3.19	TERWOG	157.637	3.244
TERWOG	177.981	3.063	TERWOG	158.129	2.909	TERWOG	153.674	2.924	TERWOG	151.752	3.112	TERWOG	152.903	2.989
TERWUM	156.258	3.191	TERWUM	164.154	3.04	TERWOG	155.411	3.147	TERWOG	160.134	2.695	TERWUM	163.975	3.16
TERXIB	156.361	2.852	TERXIB	152.446	2.976	TERWUM	166.802	3.016	TERXIB	175.199	2.797	TERXIB	166.461	3.017
TESGAA	151.091	3.259	TESGAA	168.669	3.325	TERXIB	152.434	3.334	TERXIB	152.99	3.172	TERXIB	156.891	3.029
TETHIJ	170.965	3.097	TETHIJ	160.69	3.334	TESHAC	154.483	3.29	TETBOJ	151.161	3.346	TETHIJ	170.355	3.097
TEVSUM	160.092	2.716	TEVWEW	161.119	3.096	TETZIF	156.666	3.298	TEVSES	169.085	2.96	TEVSES	168.02	2.795
TEVYEA	161.556	3.203	TEVYEA	153.665	3.23	TEVYEA	150.828	3.038	TEVYEA	170.632	2.727	TEVYEA	166.076	3.184
TEVYIE	162.959	2.955	TEVYIE	173.53	3.119	TEVYEA	154.123	3.269	TEVYIE	165.241	3.021	TEVYIE	170.406	2.957
TEVYIE	165.095	3.476	TEVYIE	172.185	2.975	TEVYIE	165.953	3.147	TEVYIE	151.902	3.288	TEVYIE	155.742	2.859
TEVYIE	155.12	3.191	TEWBOO	170.666	3.475	TEVYIE	162.25	3.194	TEVYIE	172.558	3.328	TEVYIE	151.197	3.012
TEWBOO	164.946	3.172	TEWBOO	155.174	3.07	TEWBOO	167.873	3.035	TEWBOO	151.083	3.182	TEWBOO	178.655	3.035
TEWMIV	163.6	2.957	TEWNIW	157.78	2.884	TEWJAK	151.665	2.921	TEWKAL	155.166	3.167	TEWMIV	169.477	3.464
TEWNOC	163.694	3.485	TEWPIY	152.887	2.909	TEWNIW	155.348	3.079	TEWNIW	166.426	2.91	TEWNOC	166.786	3.326
TEWQEV	162.249	3.459	TEWYAY	153.256	3.483	TEWPIY	156.681	3.163	TEWPOE	163.478	2.77	TEWPOE	153.404	3.314
TEXLAK	172.622	3.143	TEXQIW	153.468	3.025	TEXHAH	178.839	3.243	TEXLAK	167.733	3.222	TEXLAK	155.164	3.212
TEYBAA	175.162	3.125	TEYBIM	154.043	3.159	TEXXOL	158.037	2.932	TEXXOL01	152.924	2.948	TEYBAA	171.825	3.206
TEYDAC0	165.109	3.27	TEYDAC0	159.602	3.372	TEYBIM	150.298	3.06	TEYBIM	159.326	2.902	TEYBIM	150.526	3.149
TEYKEO	153.395	2.929	TEYKEO	152.08	3.356	TEYDAC0	157.461	3.435	TEYDAC0	157.988	3.466	TEYDAC0	150.461	3.21
TEYKEO	163.407	2.882	TEYKIS	154.053	2.993	TEYKEO	153.141	3.402	TEYKEO	165.518	3.016	TEYKEO	169.001	3.109
TEYKIS	157.386	3.013	TEYKIS	161.424	2.646	TEYKIS	152.484	2.831	TEYKIS	151.66	2.792	TEYKIS	170.675	2.646
TEYMEP	150.197	2.828	TEYMEP	152.1	3.273	TEYKIS	161.67	3.432	TEYKIS	161.351	2.844	TEYMEP	162.402	2.803
TEZKOB	170.799	3.11	TEZMUK	156.035	3.059	TEYMEP	154.586	2.933	TEYWEB	162.507	3.046	TEZKOB	169.232	3.11
TEZSOJ01	163.25	2.78	TEZSOJ01	159.548	3.178	TEZNAR	159.734	3.2	TEZNAR	151.517	3.258	TEZNAR	174.828	3.073
TEZSOJ01	166.492	3.368	TEZSOJ01	168.275	2.78	TEZSOJ01	158.459	3.041	TEZSOJ01	171.206	2.791	TEZSOJ01	152.353	2.677
TEZSOJ01	160.555	3.256	TEZSOJ01	158.54	3.368	TEZSOJ01	168.526	3.178	TEZSOJ01	168.208	3.208	TEZSOJ01	170.491	3.372
TFACET	155.217	3.041	TFACET	159.574	3.071	TEZVON	156.762	3.053	TEZYOO	168.775	3.228	TEZYOO	170.313	2.878
TFACMO0	153.125	3.274	TFACMO0	172.461	2.759	TFACET	156.893	3.23	TFACMO0	160.394	2.785	TFACMO0	168.701	3.15

TFACNI	164.31	2.886	TFACNI01	169.217	2.836	TFACNI	174.788	2.886	TFACNI	150.75	3.393	TFACNI	159.697	3.48
TFAERH	153.706	3.036	TFAERH	152.411	3.392	TFACNI01	154.525	2.836	TFAERH	161.653	3.363	TFAERH	156.703	3.461
TFALAH01	155.273	3.292	TFALAH01	160.32	3.029	TFAERH	166.22	2.82	TFAERH	150.798	3.251	TFAERH	161.014	3.423
TFAPDA10	158.72	3.358	TFAPDA10	170.439	3.431	TFALAH01	154.139	3.248	TFAPCU	160.746	3.273	TFAPDA10	164.735	2.762
TFAPDA11	166.485	3.227	TFAPDB10	170.846	3.485	TFAPDA11	161.571	2.707	TFAPDA11	160.843	3.449	TFAPDA11	158.042	3.247
TFBENQ02	169.781	2.968	TFESFE10	174.501	3.195	TFAPDB10	168.704	3.067	TFAPDB10	168.114	3.41	TFBENQ01	170.132	2.974
TFMESM	157.327	3.412	TFMSAD	155.033	3.143	TFESFE10	154.77	2.987	TFESFE10	163.503	3.151	TFMESM	157.39	3.349
TFMSAD0	156.145	3.149	TFMSPH	153.304	3.133	TFMSAD	156.773	3.229	TFMSAD0	154.231	3.401	TFMSAD0	151.071	3.066
TFMSUL	169.927	3.209	TFMSUL01	159.645	3.215	TFMSPH	154.915	3.288	TFMSPH	155.209	2.887	TFMSUL	160.109	3.357
TFPTPM10	157.644	2.887	TFPTPM10	166.557	3.021	TFMSUL01	163.728	3.116	TFPIOC	167.88	3.145	TFPIOC	176.134	3.437
TFPTPM10	151.466	2.929	TFPTPM10	173.08	2.877	TFPTPM10	163.959	3.189	TFPTPM10	167.965	3.037	TFPTPM10	158.75	3.348
TFSLAC	165.232	2.934	TFSLAC	156.379	3.268	TFPTPM10	153.732	3.114	TFSLAC	151.454	2.995	TFSLAC	151.938	3.265
THFAZN	153.06	3.063	THFAZN	169.042	2.943	TFSLAC	160.719	3.134	THFACO0	157.771	3.108	THFACO0	162.736	3.256
TIBVOT	152.867	2.864	TIBVOT	162.755	2.711	THTOXB	151.546	3.213	THTOXB	166.305	2.888	TIBDOY	160.376	3.008
TIBWEK	167.251	3.279	TIBXEK	150.189	3.42	TIBVUZ	156.758	3.054	TIBVUZ	169.519	3.113	TIBWAG	150.337	2.994
TICCEN	150.189	2.879	TICCEN	151.877	3.079	TIBXUY	152.117	3.098	TIBXUY	170.657	3.098	TICCEN	157.368	3.263
TICGIW	160.553	3.375	TICGIW	170.664	3.195	TICCEN	166.886	3.136	TICCEN	154.478	3.234	TICCEN	160.279	2.879
TICKOJ	160.33	3.08	TICNAY	157.47	3.456	TICHEV	175.114	2.915	TICJEX	164.132	3.046	TICKOJ	169.472	2.991
TICSAA	162.037	3.021	TICSAA	157.231	3.021	TICQEB	158.675	3.232	TICQIJ	155.027	2.821	TICQOM	169.038	3.452
TICSAA	165.618	3.253	TICSAA	151.376	3.173	TICSAA	150.819	3.068	TICSAA	155.094	3.275	TICSAA	158.264	3.254
TIDGAQ	159.526	3.246	TIDGIW	172.065	3.366	TICSUU	162.053	2.985	TICSUU	150.794	3.017	TICZOY	179.218	2.7
TIDQAZ	155.319	2.983	TIDQAZ	158.958	3.483	TIDGOG	152.786	3.375	TIDGOG	160.253	3.3	TIDGOG	160.165	3.332
TIDREE	156.174	3.422	TIDREE	151.106	3.296	TIDQAZ	151.128	2.722	TIDQAZ	165.686	2.942	TIDQAZ	160.51	2.983
TIDRII	157.006	3.107	TIDROO	157.049	3.47	TIDREE	174.867	3.315	TIDREE	172.098	2.962	TIDREE	165.091	2.981
TIDSOP	150.943	3.354	TIDTUZ	169.109	2.882	TIDROO	169.744	2.984	TIDSOP	158.51	2.929	TIDSOP	169.041	2.828
TIFCIU	154.264	3.414	TIFCIU	158.564	3.166	TIDVIP	150.099	2.929	TIDVIP	150.996	2.814	TIDYOX	161.021	3.054
TIFCIU	162.485	3.447	TIFGAU	152.116	2.891	TIFCIU	175.127	3.333	TIFCIU	172.513	3.462	TIFCIU	165.738	3.491
TIFGAU	162.178	2.917	TIFGAU	151.799	2.976	TIFGAU	158.054	2.803	TIFGAU	158.896	2.804	TIFGAU	163.746	2.899
TIFGAU	151.347	3.035	TIFGIC	156.44	2.921	TIFGAU	152.114	3.026	TIFGAU	158.837	3.329	TIFGAU	172.742	3.456
TIFZIV	156.277	2.938	TIFZIV	168.517	3.207	TIFRUX	158.17	3.378	TIFSEI	164.832	3.296	TIFZER	154.718	3.184
TIGBEU	159.942	2.961	TIGBEU	156.696	2.895	TIFZIV	166.575	3.254	TIFZIV	162.094	3.269	TIFZOY	156.395	3.31
TIHFUP	167.959	3.11	TIHRIN	170.983	2.906	TIGJAV	176.109	3.02	TIGNUW	151.533	2.824	TIHFOF	154.78	3.096
TIHRIN	174.562	3.074	TIHTAI	162.884	3.2	TIHRIN	159.906	3.435	TIHRIN	155.798	3.371	TIHRIN	153.555	3.222
TIHVOZ	167.017	3.395	TIHVOZ	174.511	3.462	TIHTAI	157.44	3.07	TIHVOZ	159.897	3.308	TIHVOZ	155.211	3.097
TIJCUM	153.344	3.16	TIJCUO	158.22	2.67	TIHVUF	151.738	3.257	TIHYUF01	174.068	2.87	TIJCAR	171.123	3.228
TIJCUO	162.309	3.21	TIJCUO	164.022	2.813	TIJCUO	151.764	2.845	TIJCUO	174.085	3.195	TIJCUO	151.149	2.858
TIKFOK	155.066	3.213	TIKFOK	163.13	3.217	TIJQEK	166.89	3.107	TIKCEY	165.383	3.351	TIKFOK	178.576	3.225
TILCAV	172.727	2.941	TILCAV	166.785	2.862	TIKHOO	152.695	2.764	TIKMIL	157.428	3.152	TIKNAE	161.228	3.232
TILNUA	156.784	3.259	TILNUA	159.533	3.151	TILGAZ	154.835	2.978	TILGAZ	164.555	3.328	TILNUA	166.469	2.873
TILYUK	162.563	2.82	TILYUK	155.18	3.047	TILNUA	151.067	3.167	TILNUA	167.141	2.967	TILYUK	170.156	2.921
TIMCAV	152.517	2.931	TIMCAV	165.918	3.195	TILYUK01	170.156	2.921	TILYUK01	162.563	2.82	TILYUK01	155.18	3.047
TIMCAV	165.512	3.232	TIMCAV	165.202	3.387	TIMCAV	169.093	3.361	TIMCAV	150.628	3.019	TIMCAV	154.552	3.086
TIMCID02	174.181	3.15	TIMCID02	161.415	3.012	TIMCAV	161.801	3.392	TIMCID	162.054	3.369	TIMCID01	161.464	3.31
TIMDOM	161.496	3.115	TIMDOM	163.018	3.115	TIMCID02	162.13	3.173	TIMCID02	157.438	2.922	TIMCID02	164.409	3.069
TIMMEL	174.757	3.395	TIMMEL	166.738	3.037	TIMDUR	167.349	3.498	TIMDUR	150.931	3.259	TIMDUR	175.78	3.087
TIMRIT	170.477	3.178	TIMROZ	164.001	3	TIMMIP	161.643	2.76	TIMMIP	159.784	2.906	TIMMIP	165.08	2.76
TINRAM	155.827	3.236	TINREQ	162.351	3.427	TINMUA	152.858	2.954	TINQOZ	167.49	3.262	TINRAM	158.271	3.43
TIPJOU	163.844	2.59	TIPNEO	161.808	2.916	TINREQ	168.044	2.944	TINZIB	169.024	2.815	TIPJOU	153.231	2.59
TIQCOL	157.408	3.285	TIQCOL	154.969	3.023	TIPNIT	157.328	2.891	TIPNIT	173.177	2.891	TIPZOJ	162.428	3.263
TIQHOT	166.617	3.274	TIQHOT	150.718	3.125	TIQCOL	159.121	3.261	TIQCUU	153.417	3.487	TIQHOT	156.891	2.651
TIQHOT	157.223	3.027	TIQLOY	171.5	3.465	TIQHOT	170.858	3.027	TIQHOT	176.243	3.292	TIQHOT	153.87	3.227
TIRGIO	153.949	3.015	TIRJAJ	173.539	3.315	TIQLOY	155.806	3.452	TIQMEO	156.739	3.047	TIRBOO	164.861	3.096
TIRLEN	160.393	3.143	TIRMIS	157.058	3.46	TIRJAJ	163.998	3.232	TIRKUC	158.381	3.266	TIRKUC	153.215	2.793
TIRMIS	165.145	2.683	TIRMIS	162.884	3.282	TIRMIS	154.628	3.373	TIRMIS	170.963	3.32	TIRMIS	164.529	3.419
TIRMIS	169.738	3.431	TIRMIS	161.866	2.901	TIRMIS	155.992	3.071	TIRMIS	171.35	2.781	TIRMIS	166.972	2.724
TIRMUE	173.175	3.066	TIRMUE	151.321	2.834	TIRMIS	160.611	2.737	TIRMIS	157.742	3.318	TIRMIS	158.075	3.286
TIRNAL	155.372	3.095	TIRNAL	168.836	3.142	TIRNAL	171.68	3.034	TIRNAL	155.752	2.929	TIRNAL	170.168	3.095
TIRNAL	167.629	3.383	TIRNUF	158.456	3.405	TIRNAL	162.58	3.331	TIRNAL	156.139	2.732	TIRNAL	164.889	3.366
TIRROB	160.1	3.147	TIRROB	151.395	2.974	TIRNUF	158.521	3.256	TIRNUF	156.035	3.495	TIRNUF	168.931	2.978
TIRTUK	157.5	3.27	TIRTUK	163.614	2.873	TIRROB	171.215	3.457	TIRSOG	174.361	2.971	TIRTUK	165.741	3.233
TIRTUK	154.289	3.364	TIRTUK	165.88	3.422	TIRTUK	160.44	2.907	TIRTUK	164.205	3.205	TIRTUK	153.073	3.366
TISLEP	161.759	3.003	TISLIT	153.78	2.85	TIRTUK	161.698	3.139	TISLAL	161.839	3.308	TISLEP	168.182	2.929
TISLOZ	152.938	3.471	TISLUG	156.784	3.244	TISLOZ	169.019	2.826	TISLOZ	150.383	2.752	TISLOZ	152.504	3.347
TITCEH	152.959	2.75	TITCEH	156.778	3.382	TITBUW	158.071	2.938	TITBUW	153.925	2.938	TITCEH	173.204	3.454
TITFIM	153.43	3.128	TITFIM	152.997	3.265	TITCEH	169.356	2.938	TITCEH	169.786	2.75	TITCOR	151.509	3.03
TITFIM	155.666	3.102	TITFIN	158.863	2.76	TITFIM	152.876	3.176	TITFIM	152.784	2.836	TITFIM	163.812	2.999
TITFUY	168.833	2.982	TITFUZ	171.497	3.345	TITFUY	152.197	3.336	TITFUY	152.974	2.97	TITFUY	152.126	2.85
TITWID	162.542	3.395	TITZED	157.203	2.919	TITFUZ	158.732	2.85	TITNAO	155.16	3.479	TITNAO	151.829	3.172
TIWLUI	156.863	2.808	TIWLUI	154.449	2.808	TIVQAT	154.664	2.885	TIWBOR	150.274	3.019	TIWLUI	176.29	3.451
TIWWOL	152.287	3.249	TIWXEE	166.639	3.066	TIWNET	176.446	3.151	TIWNIX	175.653	3.246	TIWWOL	162.255	3.249
TIWYEG	168.933	3.442	TIWYEG	155.007	3.111	TIWXEE	158.92	3.066	TIWXII	153.101	3.33	TIWYEG	166.155	3.153
TIXCOW	154.492	3.21	TIXFOZ	155.156	2.998	TIWYEG	164.886	3.188	TIXBAG	161.671	3.33	TIXBUY	158.32	3.351
TIXHOY	151.724	2.752	TIXHUE	155.24	3.258	TIXFUF	153.439	3.163	TIXGEQ	152.817	3.018	TIXHOY	166.551	3.366
TIXHUE01	160.576	3.057	TIXHUE01	158.243	2.861	TIXHUE	160.528	3.092	TIXHUE	168.173	2.839	TIXHUE	154.495	3.288
TIXZOS	162.28	3.084	TIYBEK	156.584	3.467	TIXHUE01	152.942	3.168	TIXHUE01	164.793	3.072	TIXNIB	171.246	3.305
TIYGIS	168.103	2.73	TIYGOY	153.939	2.842	TIYGAL	174.231	3.416	TIYGAL	164.466	3.077	TIYGIS	151.284	2.721
TIYKES	165.627	3.101	TIYMUJ	153.448	3.332	TIYGOY	173.523	3.39	TIYHUI	163.858	2.867	TIYHUI	150.646	2.862
TIZCIQ	153.621	3.152	TIZCOW	168.065	2.802	TIYVUT	166.738	3.252	TIYWII	168.797	2.983	TIYWII	150.757	3.205
TIZGER	154.441	2.932	TIZGER	167.787	2.766	TIZGER	155.083	2.847	TIZGER	173.117	2.78	TIZGER	150.777	3.008

TIZSIG	152.982	3.314	TIZSOM	167.956	2.996	TIZNEX	165.847	3.49	TIZQAV	156.76	3.381	TIZQAV	176.25	3.225
TIZWUU	154.056	3.242	TIZXIK	166.216	2.574	TIZSOM	170.547	2.996	TIZTED	154.351	3.032	TIZTED	155.788	3.192
TOBBIV	154.498	3.167	TOBBIV	150.598	3.484	TIZXIK	165.481	2.574	TIZXIK	153.117	2.755	TOBBIV	154.086	3.098
TOBBOB	151.726	3.007	TOBCIY	150.483	3.352	TOBBIV	155.416	3.098	TOBBIV	150.037	3.165	TOBBOB	172.159	2.756
TOBWIS	169.21	3.415	TOBWIS	156.156	2.918	TOBWIS	153.825	3.107	TOBWIS	156.479	3.023	TOBWIS	152.771	2.87
TOBYIS	162.6	3.496	TOBYIS	163.669	3.196	TOBYEO	150.388	3.434	TOBYEO	156.61	2.822	TOBYEO	160.684	3.183
TOCRUA	154.569	2.795	TODHON	152.627	3.482	TOCFUM	153.198	3.264	TOCQOT	164.392	2.972	TOCQOT	153.2	2.749
TODHUT	165.702	3.254	TODHUT	167.952	3.036	TODHON	158.897	3.482	TODHUT	155.362	2.956	TODHUT	155.438	3.227
TODRAJ	160.02	3.12	TODRAJ	157.68	3.493	TODHUT	154.16	3.112	TODJEE	176.215	3.323	TODJEE	167.752	3.181
TODSOU	153.953	2.885	TODSOU	170.861	2.862	TODSOU	160.861	2.87	TODSOU	164.591	2.919	TODSOU	164.947	3.153
TOFSUG	159.145	3.196	TOFSUG	150.187	2.576	TODSOU	168.125	2.769	TOFSOW	156.916	3.29	TOFSUG	156.415	3.009
TOFWIX	154.127	3.244	TOGJIK	177.848	3.014	TOFSUG	150.124	3.47	TOFWIX	156.875	3.15	TOFWIX	157.875	3.412
TOGNIM	162.392	3.074	TOGNIM	158.484	3.314	TOGJIK	164.105	3.014	TOGNAE	152.402	2.772	TOGNAE	170.49	2.772
TOGNIM	165.077	3.245	TOGNIM	173.096	3.037	TOGNIM	175.665	3.298	TOGNIM	166.307	3.062	TOGNIM	162.585	2.572
TOGWUK	176.883	3.242	TOHBUR	163.6	3.458	TOGVEU	165.017	3.036	TOGVEU	152.392	3.181	TOGWUK	177.72	3.035
TOHNEM	151.9	3.403	TOHNEM	157.273	3.3	TOHGUV	151.103	3.187	TOHGUV	160.19	3.012	TOHNEM	160.773	3.174
TOHRIS	161.279	2.966	TOHSUE	157.821	3.021	TOHPAL	155.176	2.992	TOHPAL	158.954	2.722	TOHPAL	159.675	3.115
TOHTAL	151.874	3.197	TOHTEP	165.753	3.447	TOHSUE	150.523	3.491	TOHSUE	174.035	3.142	TOHSUE	175.987	3.362
TOHTIT	158.97	3.049	TOHTIT	165.864	3.289	TOHTEP	153.512	3.063	TOHTEP	157.25	3.206	TOHTIT	173.522	3.241
TOHTIT	160.187	3.437	TOHVIW	165.329	3.391	TOHTIT	156.89	3.369	TOHTIT	169.535	2.955	TOHTIT	168.139	2.941
TOJFAD	176.092	3.169	TOJFAD	159.469	2.831	TOHVUI	167.152	3.213	TOHWES	150.303	3.247	TOHWES	159.568	3.303
TOJFEH	177.291	2.858	TOJFEH	151.39	3.229	TOJFAD	153.742	2.872	TOJFEH	173.25	2.751	TOJFEH	154.01	3.47
TOJGEI	151.498	3.423	TOJGEI	158.7	3.278	TOJFOR	170.466	3.122	TOJFUX	158.86	3.014	TOJFUX	159.391	3.142
TOJKEJ	153.514	3.36	TOJKEJ	150.843	3.197	TOJGOS	158.211	3.207	TOJGOS	161.103	3.097	TOJGOS	157.614	3.043
TOJKEJ	164.918	3.338	TOJKEJ	152.786	3.007	TOJKEJ	159.29	3.29	TOJKEJ	151.629	2.855	TOJKEJ	156.795	2.787
TOJPAL	151.704	3.265	TOJQAL	152.881	3.096	TOJKEJ	154.757	3.197	TOJKEJ	150.418	2.988	TOJMAG	154.482	3.41
TOJQOZ	176.038	3.114	TOJQOZ	162.374	3.134	TOJQAL	167.507	2.868	TOJQAL	159.35	3.041	TOJQAL	166.744	3.137
TOJQUF	162.493	3.15	TOJQUF	154.771	2.717	TOJQOZ	152.057	2.637	TOJQOZ	167.802	2.637	TOJQUF	155.23	3.337
TOJQUF	171.347	2.807	TOJRAM	165.946	2.926	TOJQUF	160.25	3.124	TOJQUF	166.428	3.201	TOJQUF	159.857	2.859
TOJREQ	163.256	2.839	TOJREQ	171.149	3.41	TOJRAM	169.306	2.972	TOJRAM0	169.001	2.984	TOJRAM0	166.805	2.931
TOKVAU	167.037	3.273	TOKVIC	152.27	2.775	TOKBUQ	150.584	3.374	TOKFIM	151.521	3.173	TOKGUX	171.983	2.966
TOLJIR	161.508	2.736	TOLLIQ	163.449	3.487	TOLCIH	167.64	3.412	TOLDUW	172.679	3.05	TOLDUW	163.745	2.925
TOLQER	159.258	2.934	TOLQER	166.083	2.666	TOLQER	155.671	2.779	TOLQER	164.023	3.102	TOLQER	169.646	3.233
TOLQIV	162.042	3.254	TOLQIV	158.817	3.205	TOLQER	173.302	3.404	TOLQER	168.816	2.666	TOLQIV	178.37	3.402
TOLROC	167.631	2.825	TOLROC	152.843	3.297	TOLQIV	160.816	2.89	TOLQIV	161.5	3.402	TOLQIV	150.948	3.205
TOLROC	161.795	2.988	TOLROC	150.596	3.116	TOLROC	162.958	2.871	TOLROC	166.245	3.192	TOLROC	155.786	3.103
TOLSAP	171.434	2.622	TOLSAP	175.054	3.364	TOLROC	170.804	3.148	TOLROC	165.633	3.127	TOLSAP	173.751	3.431
TOLTIB	172.245	3.346	TOLTIB	167.2	3.327	TOLSAP	164.381	3.159	TOLSAP	164.855	3.127	TOLSAP	178.406	2.732
TOLTUN	161.841	2.884	TOLZUT	155.899	3.238	TOLTUN	169.131	3.259	TOLTUN	175.647	3.078	TOLTUN	159.177	3.315
TOMBOM	159.851	3.176	TOMBOM	150.753	3.281	TOMBIG	156.772	3.485	TOMBIG	162.61	3.297	TOMBIG	171.45	3.165
TOMDOO	169.321	3.3	TOMDOQ	172.459	3.272	TOMCAZ	158.8	3.238	TOMCAZ	154.47	3.447	TOMDII	173.485	3.494
TOMJEL	175.532	2.841	TOMJEL	166.511	3.368	TOMDOQ	153.744	3.099	TOMGEJ	172.537	3.273	TOMGEJ	153.619	3.097
TOMSIA	177.534	3.205	TONBOR	154.751	3.287	TOMJEL	164.786	2.841	TOMJEL	174.889	2.718	TOMPAO	167.978	3.17
TONVIF	151.797	3.193	TONVIF	150.032	3.192	TONMEO	170.85	2.803	TONMEO	166.862	3.421	TONVIF	151.986	3.182
TONYAZ	158.17	3.292	TOPCEI	152.722	3.057	TONWIG	171.442	3.271	TONXOM	156.17	3.068	TONXOM	153.912	2.826
TOPGOV	160.773	2.83	TOPGOV	157.554	3.147	TOPDAH	161.727	3.439	TOPDUZ	154.483	3.253	TOPDUZ	166.486	3.13
TOQBOR	150.561	2.948	TOQBOR	156.811	2.818	TOPGOV	152.884	2.909	TOPLIX	162.498	3.412	TOPXUV	152.823	3.284
TOQCAE	157.493	3.13	TOQCAE	159.876	3.021	TOQBOR	170.703	3.188	TOQCAE	158.222	2.999	TOQCAE	153.975	2.931
TOQCAE	170.278	3.21	TOQCAE	152.611	3.311	TOQCAE	160.333	2.919	TOQCAE	153.818	3.09	TOQCAE	166.679	3.35
TOQCIM	168.769	3.106	TOQCIM	161.363	3.058	TOQCAE	158.334	2.665	TOQCAE	159.351	2.919	TOQCIM	162.215	2.901
TOQDUY	161.687	3.211	TOQKAL0	150.251	2.816	TOQCIM	163.955	3.25	TOQCIM	156.967	2.94	TOQDOS	156.282	3.117
TOQKEP0	166.81	3.293	TOQKEP0	166.086	3.038	TOQKAL0	152.339	2.971	TOQKAL0	151.789	3.292	TOQKAL0	162.432	3.362
TORCUB	161.202	2.798	TORDAG	152.008	2.821	TOQKEP0	163.797	3.253	TOQKEP0	155.831	3.139	TOQYAD	151.681	3.098
TORHEO	159.171	3.064	TORPIB	160.042	2.941	TORDAG	150.962	3.258	TORFEO	161.069	3.245	TORHEO	177.091	2.873
TOSJOC	150.463	3.33	TOSJOC	166.937	3.051	TOSHEQ	154.336	2.91	TOSHEQ	158.094	3.456	TOSJOC	162.185	3.17
TOTBIP	152.354	3.414	TOTBIP	156.343	3.465	TOSYAE	160.05	3.139	TOSYOR	151.93	3.084	TOTBIP	173.424	3.418
TOTWEG	158.34	3.131	TOVCAK	168.561	3.322	TOTBIP	152.052	3.424	TOTWEG	158.104	3.131	TOTWEG	154.773	2.793
TOVJAS	168.88	3.135	TOVJUN	153.882	3.24	TOVCUG	167.673	3.1	TOVCUG	166.228	2.902	TOVCUG	155.388	3.156
TOVNUQ	162.112	2.839	TOVRAB	166.394	3.087	TOVLAT	158.408	3.4	TOVLAT	157.93	3.386	TOVNOK	163.952	2.867
TOVSEG	155.488	3.457	TOVSEG	169.605	3.115	TOVRAB	152.368	3.136	TOVRAB	153.775	3.413	TOVRAB	160.744	3.238
TOVSOQ	163.508	3.03	TOVSOQ	166.548	3.234	TOVSEG	161.954	3.234	TOVSIK	153.295	3.144	TOVSIK	156.817	2.844
TOWZEO	158.51	3.274	TOWZEO	162.657	3.222	TOWYUD	159.534	3.171	TOWYUD	160.632	3.442	TOWYUD	153.69	2.958
TOXHOD	153.14	2.957	TOXHOD	160.157	3.179	TOXGOF	170.377	2.888	TOXGOF	159.708	2.888	TOXHOD	152.299	2.935
TOXNUP	151.711	2.979	TOXNUP	152.547	2.979	TOXJAR	167.461	2.961	TOXJAR	164.411	2.961	TOXJAR	154.855	2.711
TOXPAX	157.85	3.273	TOXPAX	176.947	3.095	TOXPAX	165.664	2.827	TOXPAX	159.465	2.908	TOXPAX	169.531	3.426
TOXPEB	151.048	2.867	TOXPIF	161.604	2.774	TOXPAX	152.756	2.908	TOXPEB	166.959	2.867	TOXPEB	161.61	2.985
TOXPIF	161.411	3.485	TOXPIF	171.569	2.859	TOXPIF	171.382	2.992	TOXPIF	153.483	2.981	TOXPIF	164.881	3.183
TOYBUG	154.093	2.922	TOYHAT	154.324	3.41	TOXPIF	163.232	2.981	TOXPIF	167.801	2.894	TOXZIR	150.558	2.903
TOYPAB	160.747	2.794	TOYPEF	159.041	3.168	TOYHAT	155.905	3.484	TOYLAY	158	2.893	TOYPAB	154.043	3.141
TOYPIJ	156.899	2.903	TOYPIJ	164.991	3.462	TOYPEF	156.655	2.906	TOYPEF	164.833	3.466	TOYPIJ	158.786	3.167
TOZBAP	170.953	3.218	TOZCUJ	171.021	3.415	TOYWEM	151.533	3.333	TOYYUF	168.949	2.852	TOYZAM	162.876	3.341
TOZPEG	154.809	3.14	TOZPEG	172.721	2.836	TOZCUJ	153.999	3.493	TOZGOE0	161.885	3.103	TOZHAR	160.819	2.864
TOZPIL	165.857	2.749	TOZWIR0	161.816	3.36	TOZPEG	176.387	3.113	TOZPEG	163.707	3.21	TOZPEG	151.103	3.401
TPFPCP	155.717	2.91	TPFPCP	164.069	3.068	TOZZOZ	161.104	3.255	TPFPCP	155.253	3.209	TPFPCP	152.726	2.74
TUCFUU	157.962	3.051	TUCFUU	155.58	3.064	TUBVAN	155.278	3.051	TUCFEE	155.737	3.053	TUCFEE	161.257	2.752
TUCHEE	153.371	2.715	TUCYUN	157.871	2.828	TUCHEE	169.106	2.875	TUCHEE	172.414	3.359	TUCHEE	158.9	3.197
TUCZIC	151.945	3.123	TUDBUQ	162.339	3.236	TUCYUN	165.913	3.373	TUCYUN	175.668	2.962	TUCZAV	156.539	3.435
TUDCAX	172.429	3.286	TUDCAX	161.678	3.02	TUDBUQ	176.466	3.183	TUDBUQ	174.73	3.185	TUDBUQ	167.385	3.172
TUDKAE	175.799	3.161	TUDKEI	159.994	2.893	TUDCAX	163.015	3.112	TUDHEI	157.15	3.106	TUDHUZ	160.836	3.132

TUDKIN	163.277	3.32	TUDKIN	152.992	2.646	TUDKIN	169.987	3.256	TUDKIN	158.849	3.372	TUDKIN	154.096	3.26
TUDLUZ	161.474	3.318	TUDLUZ	161.988	2.898	TUDLUZ	164.99	3.031	TUDLUZ	171.897	3.413	TUDLUZ	166.869	3.251
TUDLUZ01	153.949	3.463	TUDLUZ01	151.268	3.131	TUDLUZ	151.784	3.169	TUDLUZ01	150.279	2.832	TUDLUZ01	157.216	3.031
TUDTIX	164.615	3.481	TUDTIX	153.512	2.875	TUDLUZ01	157.944	3.026	TUDLUZ01	158.303	2.732	TUDLUZ01	151.592	2.902
TUDTIX	175.983	3.404	TUDTIX	163.877	2.768	TUDTIX	162.787	2.851	TUDTIX	157.07	3.308	TUDTIX	160.812	3.481
TUFMIT	164.169	3.286	TUFPER	151.406	3.131	TUDWUM	164.444	2.955	TUFGOU	157.249	3.496	TUFLIT	160.749	2.691
TUFPER	150.109	2.958	TUFPER	160.342	2.98	TUFPER	150.648	3.032	TUFPER	158.962	2.756	TUFPER	153.998	3.326
TUFTAT	165.833	3.171	TUFTAT	160.527	3.441	TUFPER	175.256	3.058	TUFPER	157.351	2.812	TUFPER	151.655	3.096
TUGGUY	164.769	3.186	TUGGUY	166.835	3.193	TUGDAD	162.817	3.101	TUGDAD	157.789	3.466	TUGDAD	164.307	3.196
TUGTEX	167.373	3.209	TUGWUN	160.922	3.019	TUGHAH	158.231	3.219	TUGHAH	169.705	3.235	TUGNIW	159.171	3.249
TUHJOX	175.794	3.172	TUHJOX	172.536	2.858	TUGYIE	162.883	3.302	TUHCOS	155.264	3.279	TUHCOS	153.463	2.979
TUHJOX02	176.256	3.096	TUHQAP	161.182	3.482	TUHJOX01	172.368	2.846	TUHJOX01	175.765	3.165	TUHJOX02	171.009	2.793
TUHQAP	176.207	3.051	TUHQAP	175.973	2.987	TUHQAP	158.117	3.494	TUHQAP	152.34	2.566	TUHQAP	156.989	3.186
TUHQAP	162.753	2.566	TUHQAP	170.259	2.987	TUHQAP	154.811	3.039	TUHQAP	151.247	3.056	TUHQAP	160.327	3.051
TUHRIB	157.536	3.417	TUJGOY	161.214	3.101	TUHQAS	159.489	3.142	TUHQAS	164.89	3.236	TUHQAS	165.728	3.332
TUJHEO	156.031	2.837	TUJHEO	152.202	3.006	TUJHEO	155.825	3.193	TUJHEO	170.101	3.417	TUJHEO	164.11	3.433
TUJHOW	155.453	3.286	TUJHUE01	157.119	2.795	TUJHEO	150.725	2.92	TUJHEO	151.502	3.357	TUJHOW	166.203	3.305
TUJJAK	152.692	3.241	TUJJAK	172.941	3.44	TUJHUE01	154.626	3.221	TUJHUE01	150.44	3.335	TUJHUE01	170.448	2.941
TUJJAK	159.809	3.27	TUJJIS	152.783	3.292	TUJJAK	152.524	3.329	TUJJAK	167.023	2.906	TUJJAK	167.148	3.257
TUJJIS	166.469	3.232	TUJJIS	159.17	3.256	TUJJIS	168.718	3.193	TUJJIS	155.325	3.159	TUJJIS	167.138	2.892
TUJMAN	159.062	3.112	TUJMET	168.97	2.887	TUJKIV01	161.43	3.416	TUJKUE	175.005	3.328	TUJLAL	175.228	3.251
TUJVED	177.776	3.372	TUJVEZ	154.854	3.446	TUJMET	158.708	3.363	TUJMET	166.393	2.887	TUJMET	161.134	3.261
TUJZII	156.921	3.283	TUJZII	150.217	3.051	TUJVEZ	151.006	3.185	TUJZAA	160.056	2.984	TUJZII	162.944	3.278
TUJZII	173.526	2.885	TUJZOO	159.093	3.001	TUJZII	150.069	2.932	TUJZII	156.306	3.299	TUJZII	155.076	3.291
TUJZOO	159.446	3.173	TUJZOO	154.959	2.922	TUJZOO	163.33	3.122	TUJZOO	156.854	2.83	TUJZOO	165.742	3.29
TUJZOO	164.546	2.83	TUKBEH	175.497	3.383	TUJZOO	157.04	3.125	TUJZOO	165.532	3.006	TUJZOO	156.28	3.139
TUKFAH	165.024	3.182	TUKFAH	150.751	2.893	TUKFAH	156.743	2.849	TUKFAH	151.179	3.127	TUKFAH	156.986	2.957
TUKFAH	178.863	3.417	TUKFAH	159.88	3.416	TUKFAH	160.608	3.377	TUKFAH	164.541	3.359	TUKFAH	166.463	3.127
TUKFUD	162.859	3.373	TUKMOB	155.959	3.467	TUKFAH	154.026	3.214	TUKFIP	165.384	3.284	TUKFIP	157.124	3.379
TUKNAR	153.047	3.096	TUKPEU	164.916	3.052	TUKMOB	159.948	2.844	TUKMOB	163.306	2.853	TUKMOB	151.269	2.989
TULKER	169.651	3.476	TULKER	162.128	2.721	TULHUG	153.002	2.831	TULKER	157.211	2.64	TULKER	151.124	3.387
TULKER	155.677	3.138	TULKER	156.229	2.891	TULKER	170.801	2.997	TULKER	172.167	3.171	TULKER	175.637	3.138
TULVOP	153.549	3.327	TULVOP	165.078	3.085	TULKER	171.348	3.157	TULKER	162.664	2.951	TULPAR	164.864	3.448
TUMTAZ	152.989	3.046	TUMTAZ	157.172	3.02	TULWAC	172.584	2.929	TUMTAZ	159.083	3.302	TUMTAZ	163.084	3.099
TUMTED	170.036	3.267	TUMTED	170.319	3.144	TUMTED	151.052	3.061	TUMTED	168.096	3.267	TUMTED	177.303	2.975
TUMVOP	151.353	2.825	TUMVUV	169.384	2.857	TUMTED	161.476	3.22	TUMVOP	163.851	2.765	TUMVOP	151.754	2.825
TUMVUV	155.396	2.631	TUMVUV	153.22	3.347	TUMVUV	150.46	3.121	TUMVUV	155.063	2.683	TUMVUV	166.247	3.372
TUMWAC	165.136	2.912	TUMWAC	159.78	2.991	TUMWAC	163.115	2.998	TUMWAC	159.311	3.32	TUMWAC	154.416	3.033
TUMWAC	177.376	3.033	TUMWAC	157.473	3.349	TUMWAC	164.518	2.75	TUMWAC	165.336	3.259	TUMWAC	168.947	2.935
TUMYIK	162.591	2.633	TUMZAF	158.432	2.872	TUMWAC	171.885	2.956	TUMYIK	162.854	2.857	TUMYIK	152.663	2.876
TUNFIR	171.655	3.115	TUNFIR	151.266	3.186	TUMZAF	162.92	2.872	TUMZEJ	165.044	2.84	TUMZEJ	165.133	2.84
TUNFOX	155.564	2.881	TUNFOX	159.547	3.028	TUNFOX	171.338	2.986	TUNFOX	169.588	3.135	TUNFOX	152.872	2.891
TUNFOX	169.67	3.362	TUNFUD	152.491	3.092	TUNFOX	151.001	3.141	TUNFOX	173.052	3.006	TUNFOX	162.331	3.425
TUNGOZ	152.409	2.699	TUNHIU	154.297	2.958	TUNFUD	165.446	2.999	TUNGAK	162.706	3.139	TUNGAK0	162.833	3.047
TUNKIW	159.725	3.301	TUNKOD	154.412	3.331	TUNHUJ	150.38	3.485	TUNKIW	165.751	2.928	TUNKIW	161.185	3.15
TUNRIH	160.923	3.057	TUNYAD	160.483	3.439	TUNLOG	160.066	3.042	TUNLOG	151.727	2.959	TUNRIH	153.469	3.057
TUNYOQ	165.451	3.297	TUNYOQ	153.362	3.34	TUNYEH	156.676	3.419	TUNYOQ	165.63	3.207	TUNYOQ	163.785	3.193
TUNZAE	157.039	3.188	TUPBOW	151.079	3.366	TUNYOQ	154.746	3.091	TUNYOQ	166.239	3.088	TUNYOQ	151.494	2.947
TUPDUG	160.848	3.368	TUPFUF	167.076	3.491	TUPBUB	169.286	2.859	TUPBUB	153.432	2.745	TUPBUB	168.914	2.859
TUPJUL	162.613	3.073	TUPJUL	153.49	2.811	TUPJOF	173.28	2.938	TUPJOF	154.256	3.116	TUPJUL	157.704	3.278
TUPKEY	166.008	3.01	TUPKEY	167.662	2.982	TUPJUL	167.27	3.278	TUPJUL	156.59	3.073	TUPKEY	150.884	3.047
TUPNOI01	163.895	3.23	TUPNOI01	163.782	3.223	TUPKEY	152.449	2.997	TUPKEY	166.922	3.478	TUPNOI	162.432	3.304
TUPNOI01	152.011	2.71	TUPVIN	158.41	2.855	TUPNOI01	152.161	2.919	TUPNOI01	150.087	2.895	TUPNOI01	157.04	2.651
TUPVUZ	150.404	3.329	TUPVUZ	153.233	2.911	TUPVUZ	173.037	2.809	TUPVUZ	166.08	2.864	TUPVUZ	176.244	2.809
TUPVUZ	172.221	3.203	TUPVUZ	165.663	3.161	TUPVUZ	164.615	3.332	TUPVUZ	154.747	3.259	TUPVUZ	151.342	2.827
TUPVUZ	165.015	3.101	TUPVUZ	163.794	3.422	TUPVUZ	154.528	3.469	TUPVUZ	172.288	3.47	TUPVUZ	162.03	2.832
TUPVUZ	172.726	2.827	TUPVUZ	160.818	3.438	TUPVUZ	166.537	3.438	TUPVUZ	151.383	3.028	TUPVUZ	164.67	3.164
TUPXIN	151.366	2.987	TUPYAE	150.953	3.156	TUPVUZ	165.039	2.832	TUPVUZ	165.879	3.014	TUPVUZ	164.153	2.761
TUQBUF	176.735	3.398	TUQBUF	163.219	3.149	TUPYAE	171.259	2.989	TUQBOZ	162.847	3.156	TUQBOZ	174.856	3.392
TUQDOB	153.848	2.929	TUQDOB	165.93	2.824	TUQCOA	162.563	3.163	TUQCOA	174.603	3.402	TUQDOB	164.505	2.824
TUQSUV	162.78	2.812	TUQTOO	170.089	3.106	TUQDUG	160.836	3.499	TUQSUV	163.508	3.076	TUQSUV	172.132	3.272
TURHUN	151.641	3.344	TURQAA	175.008	2.991	TUQTOO0	170.089	3.106	TUQXUC	168.247	3.315	TURBEP	151.713	2.988
TURQOO	150.325	2.735	TURSII	156.884	3.034	TURQAA	160.115	3.437	TURQEE	161.711	2.908	TURQOO	150.366	2.735
TURXAI	159.671	3.241	TUSGOG	159.191	3.168	TURWIO	151.735	2.809	TURWIO	168.003	2.852	TURWIO	165.642	2.809
TUSGUM	162.661	2.93	TUSHAU	158.296	2.929	TUSGOG	163.079	2.926	TUSGUM	157.545	3.128	TUSGUM	152.114	3.47
TUSLUP	153.563	3.311	TUSMED	160.069	3.399	TUSLID	164.233	2.984	TUSLID	158.361	3.403	TUSLUP	157.089	2.928
TUSQEE	154.059	3.009	TUSQII	155.804	2.818	TUSMED	160.138	3.431	TUSPUT	174.767	2.857	TUSPUT	162.816	3.477
TUTBOZ	150.582	3.235	TUTFAR	161.024	3.49	TUSTOT	150.85	3.107	TUTBOZ	168.671	3.026	TUTBOZ	169.042	3.039
TUTFOG	171.858	2.695	TUTFOG	164.791	3.416	TUTFIA	153.045	2.749	TUTFIA	151.544	2.749	TUTFOG	151.444	3.265
TUTFOG	160.829	3.397	TUTFOG	164.565	3.382	TUTFOG	156.166	3.482	TUTFOG	151.741	2.732	TUTFOG	155.819	3.105
TUTJEY	155.661	3.335	TUTJEY	158.967	3.166	TUTFOG	152.484	3.352	TUTFOG	171.88	3.082	TUTJEY	154.483	3.22
TUTKAV	166.891	3.003	TUTKEZ	151.396	2.865	TUTJEY	166.08	3.201	TUTJEY	158.387	3.166	TUTJUO	162.015	2.923
TUTRIK	156.159	2.742	TUTRIK	154.322	2.742	TUTKEZ	158.281	3.08	TUTKID	162.846	2.963	TUTKUP	160.676	3.318
TUTVAI	160.406	2.711	TUTVAI	160.685	3.437	TUTSAD	150.229	3.462	TUTSEH	152.315	2.833	TUTVAI	163.857	3.368
TUTVEK	164.174	3.319	TUTVEK	154.694	2.79	TUTVEK	170.302	2.812	TUTVEK	163.523	3.396	TUTVEK	156.321	3.029
TUTWIP	158.091	2.926	TUTXEM	158.254	3.233	TUTVOU	154.029	2.953	TUTWAH	164.401	3.469	TUTWIP	150.876	3.06
TUVMOM	153.839	2.966	TUVMOM	161.854	2.946	TUVJEZ	152.393	2.984	TUVJEZ	168.641	3.361	TUVMOM	151.26	3.214
TUVNEG	150.935	3.101	TUVNEG	154.319	3.192	TUVMOM	173.158	3.214	TUVNEG	150.329	3.025	TUVNEG	162.504	3.09
TUVTUA	167.396	2.799	TUVTUA	162.542	3.334	TUVNEG	156.428	3.258	TUVNIH	157.018	3.469	TUVNIH	158.878	3.207

TUVYOC	165.741	2.874	TUVZUJ	177.025	3.186	TUVTUA	166.259	2.799	TUVVUF	154.105	3.045	TUVWOA	176.889	3.137
TUWMOQ	158.605	3.212	TUWNEG	153.522	3.35	TUWFIB	160.879	3.046	TUWHUR	160.111	2.909	TUWJAZ	160.619	2.957
TUWVOW	158.971	2.753	TUWVOW	156.647	2.925	TUWVOW	156.752	2.901	TUWVOW	162.32	2.684	TUWVOW	161.644	2.822
TUWVOW	158.355	3.06	TUWVOW	163.973	3.059	TUWVOW	171.519	3.36	TUWVOW	158.408	2.901	TUWVOW	163.276	2.822
TUWVOW	153.985	3.036	TUWVOW	163.28	2.8	TUWVOW	152.545	2.684	TUWVOW	169.039	2.982	TUWVOW	168.786	2.886
TUXLIL	152.517	2.902	TUXNAD	156.729	3.13	TUWVOW	160.849	2.704	TUXKEG	167.634	3.322	TUXLEH	167.503	2.731
TUXQAF	168.574	3.159	TUXQAF	165.905	2.88	TUXNAE	167.25	2.699	TUXPUY	171.317	3.361	TUXPUY	165.513	3.02
TUXYAO	153.769	3.321	TUXYAO	154.3	3.22	TUXQEL	151.61	3.053	TUXRAF	154.918	2.923	TUXXET	153.159	3.389
TUZKAD	166.961	2.99	TUZSIR	154.199	3.298	TUYTUG	155.609	3.268	TUZDEA	163.035	3.015	TUZHOO	166.596	3.061
TUZSOX	163.472	3.187	TUZSOX	152.56	2.728	TUZSIR	153.428	3.052	TUZSIR	169.865	2.784	TUZSOX	161.906	3.472
UBADEI	169.97	3.098	UBADEI	156.154	2.999	TUZSOX	155.238	3.257	TUZXIZ	151.404	3.144	TUZZOF	159.705	2.865
UBAMAN	155.281	3.388	UBAMAN	150.218	3.293	UBADIO	156.664	2.784	UBAJAM	157.533	3.363	UBAJAM	173.94	3.363
UBAMOB	157.371	3.384	UBAMOB	166.529	3.191	UBAMAN	161.21	3.232	UBAMAN	159.21	3.022	UBAMAN	153.832	2.943
UBAQUJ	167.097	3.31	UBAXED	159.65	3.11	UBAMOB	161.033	3.338	UBAMOB	166.343	3.187	UBAQUJ	155.379	3.048
UBEREY	157.276	3.369	UBEREY	157.219	3.357	UBAYUV	163.685	3.163	UBAYUV	155.209	3.238	UBEREY	160.355	3.283
UBEREY	150.617	3.264	UBEXOQ	157.853	2.904	UBEREY	157.532	3.376	UBEREY	156.142	3.474	UBEREY	158.056	3.464
UBIZIS	159.873	3.347	UBIZOW	160.477	3.088	UBISOR	168.333	2.986	UBIXOS	155.87	2.771	UBIZIS	164.846	3.362
UBIZOW	158.135	3.139	UBODIY	152.613	3.451	UBIZOW	167.069	2.939	UBIZOW	171.604	2.819	UBIZOW	154.288	3.093
UBOGIE	157.67	3.169	UBOGIE	151.007	3.39	UBODIY	164.232	3	UBOGIE	150.873	3.39	UBOGIE	166.463	3.471
UBOHOL	158.8	3.057	UBOJUS	166.39	3.291	UBOGIE	153.33	3.197	UBOHOL	166.801	3.462	UBOHOL	174.845	3.204
UBOLIG	151.556	3.1	UBOLIG	167.272	3.289	UBOLAY	162.717	3.031	UBOLIG	162.222	3.431	UBOLIG	158.592	3.277
UBOQUX	159.225	3.145	UBOYUJ	166.962	3.463	UBOLIG	158.409	3.384	UBOLIG	159.249	3.266	UBOMOR	160.097	3.281
UBOZUI	178.216	3.469	UBOZUI	156.715	3.148	UBOZUI	169.56	2.809	UBOZUI	158.836	2.823	UBOZUI	168.925	2.951
UBOZUI	170.195	2.904	UBOZUI	163.172	3.308	UBOZUI	176.419	3.257	UBOZUI	171.057	3.331	UBOZUI	153.257	3.469
UBOZUI	173.578	3.02	UBOZUJ	170.091	2.844	UBOZUI	162.748	2.922	UBOZUI	170.465	3.277	UBOZUI	159.418	2.823
UBUBAW	174.392	2.979	UBUBAW	167.403	3.288	UBUBAW	165.414	3.415	UBUBAW	160.517	2.675	UBUBAW	176.95	3.456
UBUBAW	174.586	2.989	UBUBAW	173.69	3.388	UBUBAW	163.878	2.743	UBUBAW	165.788	3.443	UBUBAW	165.062	2.867
UBUBAY	160.115	2.938	UBUBEC	154.467	3.172	UBUBAW	163.222	3.01	UBUBAW	161.464	2.867	UBUBAW	162.634	2.902
UBUBEC	152.132	2.806	UBUBEC	173.104	3.417	UBUBEC	166.746	3.29	UBUBEC	158.796	3.036	UBUBEC	171.932	3.02
UBUBUQ	173.6	3.13	UBUBUQ	167.101	3.364	UBUBEC	178.509	3.266	UBUBUQ	170.589	3.331	UBUBUQ	162.115	2.833
UBUBUQ	155.553	2.983	UBUBUQ	160.107	2.792	UBUBUQ	169.872	3.017	UBUBUQ	164.006	3.104	UBUBUQ	153.257	3.459
UBUCIF	154.517	2.844	UBUCIF	159.018	3.183	UBUBUQ	155.172	3.017	UBUBUQ	159.315	2.833	UBUCIF	163.924	2.836
UBUCIF	150.473	3.228	UBUCIF	173.191	2.875	UBUCIF	157.192	3.36	UBUCIF	159.99	2.955	UBUCIF	175.04	2.968
UBUCIF	157.216	3.292	UBUCIF	161.719	2.968	UBUCIF	167.357	2.836	UBUCIF	156.436	2.825	UBUCIF	154.343	2.844
UBUMEL	157.968	3.145	UBUVES	152.817	3.212	UBUCIF	163.324	2.875	UBUMEL	155.883	3.474	UBUMEL	150.08	3.409
UBUVES	151.767	2.92	UBUVES	168.773	3.166	UBUVES	175.235	3.176	UBUVES	165.884	3.364	UBUVES	167.152	3.188
UBUYAU	163.877	3.159	UBUYAU	153.11	3.036	UBUVES	170.551	3.465	UBUYAU	168.4	2.92	UBUYAU	152.008	2.804
UCAQAU0	161.035	3.468	UCAQOI	160.57	3.262	UCAFUB	160.228	3.29	UCAQAU0	159.304	3.447	UCAQAU0	156.231	3.439
UCARUL0	154.283	3.292	UCASID	168.589	2.823	UCAQOI0	161.925	3.332	UCARAT	168.227	2.872	UCARAT	161.949	3.156
UCAYED	160.957	3.108	UCAYED	169.939	3.272	UCASID	167.394	2.823	UCASOJ	168.663	3.094	UCAVOL	168.074	3.002
UCAYED	162.599	3.108	UCAYED	154.012	2.979	UCAYED	174.912	3.294	UCAYED	177.21	3.254	UCAYED	179.001	3.138
UCAYED0	153.396	2.867	UCAYED0	164.616	2.712	UCAYED0	174.941	3.409	UCAYED0	154.336	3.236	UCAYED0	169.62	2.949
UCAYED0	168.851	3.487	UCAYED0	167.177	3.121	UCAYED0	161.631	3.21	UCAYED0	151.698	3.091	UCAYED0	169.144	3.288
UCEKAO	161.611	2.787	UCEKAO	151.242	2.883	UCAYED0	168.489	3.058	UCAYED0	156.441	3.304	UCAYED0	166.401	2.977
UCENEV	155.192	2.888	UCENEV	164.584	2.955	UCEKAO	157.09	2.802	UCEKAO	156.175	2.825	UCENEV	164.28	3.151
UCEPEY0	157.695	2.766	UCEPEY0	164.948	3.455	UCENEV	171.772	3.337	UCENEV	164.366	2.817	UCEPEY	157.414	2.851
UCEZUZ	152.804	3.187	UCEZUZ	151.1	3.33	UCEROL0	151.663	3.453	UCETIH02	173.883	3.223	UCETIJ	163.762	3.436
UCIRIL	156.542	3.315	UCISOS	152.753	3.088	UCIBAL	154.871	3.479	UCIBAL	169.546	3.19	UCILAX	155.369	3.12
UCIWIN	175.71	3.421	UCIYAI	177.534	2.88	UCISOS	153.245	3.36	UCISOS	151.459	3.394	UCISOS	154.694	3.048
UCOBUJ	168.732	3.304	UCOGOM	151.111	2.9	UCIYUE	151.429	3.298	UCOBUJ	164.526	3.393	UCOBUJ	167.556	2.932
UCOJOL	160.939	3.191	UCOJOL	172.499	3.004	UCOGOM	162.003	2.879	UCOGOM	154.607	2.874	UCOJOL	157.801	3.206
UCOQUC	172.846	3.152	UCOQUC	152.114	2.895	UCOJOL	166.985	2.942	UCOMAB	171.439	3.199	UCOQEM	155.387	2.775
UCORUD	151.618	3.1	UCOWAK	168.297	3.415	UCOQUC	161.486	3.355	UCOQUC	158.492	3.393	UCOROV	165.913	3.485
UCULIP	175.962	2.704	UCUPOZ	167.245	3.299	UCOWAK	153.691	3.094	UCUGUV	152.251	2.866	UCUGUV	170.398	2.866
UCUPOZ	153.031	2.965	UCUSIW	155.288	3.173	UCUPOZ	165.368	3.342	UCUPOZ	159.582	3.056	UCUPOZ	163.005	2.922
UDANIA	166.364	3.191	UDANIA	150.257	2.986	UCUSIW	155.795	3.407	UDAJEP	167.636	3.078	UDAJEP	166.854	2.76
UDANIA	161.25	3.457	UDANIA	171.262	2.828	UDANIA	150.463	3.457	UDANIA	152.799	3.065	UDANIA	150.615	3.236
UDANUM	156.454	3.5	UDAPEV	163.525	3.299	UDANIA	174.232	3.388	UDANIA	172.39	2.828	UDANUM	168.743	2.913
UDAQEV	150.364	3.381	UDAXED	173.142	2.887	UDAPEV	154.707	2.955	UDAQEV	170.482	3.247	UDAQEV	156.073	2.944
UDAXED	166.853	2.816	UDAXED	172.371	3.014	UDAXED	150.782	2.859	UDAXED	156.071	2.881	UDAXED	156.795	2.92
UDECOY	161.344	2.999	UDECUB	153.968	2.821	UDAXED	161.373	2.968	UDAXUU	162.425	2.9	UDAXUU	169.528	3.139
UDEJOE	154.932	2.995	UDEMEZ	163.257	2.919	UDECUB	163.707	3.159	UDEHEQ	151.056	3.281	UDEHEQ	177.756	3.384
UDESUT	162.981	3.352	UDESUT	158.723	3.1	UDEQEC	153.028	2.912	UDEQOK	150.144	2.883	UDESUT	176.139	2.715
UDESUT	150.699	3.4	UDETII	157.515	3.02	UDESUT	173.024	2.934	UDESUT	156.184	3.4	UDESUT	156.065	2.835
UDEZIO	153.645	3.33	UDIGEU	166.988	3.466	UDETII	159.46	3.312	UDETII	160.276	3.274	UDEXUZ	172.245	3.304
UDIHEW	167.356	2.816	UDIHEW	159.924	3.391	UDIHEW	163.909	3.222	UDIHEW	163.582	3.398	UDIHEW	153.859	3.422
UDIHUM	159.363	3.031	UDIJAU	150.954	3.167	UDIHEW	151.664	2.806	UDIHOH	172.057	3.482	UDIHUM	160.359	3.032
UDIJAU	162.472	2.758	UDIJEY	156.184	3.347	UDIJAU	166.914	3.031	UDIJAU	151.732	3.017	UDIJAU	150.075	2.965
UDIJEY	170.078	3.132	UDIJEY	161.422	3.132	UDIJEY	163.518	3.204	UDIJEY	155.562	3.033	UDIJEY	164.25	2.746
UDIJIC	168.144	2.809	UDIJIC	167.736	2.926	UDIJIC	166.413	3.004	UDIJIC	171.375	3.259	UDIJIC	170.018	2.809
UDIJIC	150.024	2.85	UDIJIC	156.684	2.859	UDIJIC	174.289	2.78	UDIJIC	164.949	2.887	UDIJIC	166.474	2.78
UDIJOI	170.712	2.729	UDIJOI	158.302	3.005	UDIJIC	167.143	3.104	UDIJIC	174.521	3.046	UDIJOI	170.051	2.729
UDIJOI	154.1	2.775	UDIKIE	153.752	3.492	UDIJOI	176.728	2.745	UDIJOI	165.658	3.04	UDIJOI	159.122	2.775
UDILAW	153.715	3.392	UDILAW	155.067	2.805	UDIKOJ	151.745	3.379	UDIKOJ	162.444	3.237	UDIKOK	175.38	2.989
UDILAW	156.203	2.896	UDILAW	160.304	2.886	UDILAW	160.142	3.156	UDILAW	153.603	3.015	UDILAW	157.495	3.261
UDILAW	158.086	3.089	UDILAW	169.541	3.201	UDILAW	150.496	3.038	UDILAW	161.024	3.35	UDILAW	151.506	2.992
UDIXAJ	166.246	3.245	UDIYIP	161.624	2.906	UDISAB	170.004	3.281	UDISAB	151.354	2.594	UDIVAH	155.952	3.356
UDIZAL	154.295	3.327	UDIZEQ	155.838	2.83	UDIYIP	154.144	3.472	UDIYIP	156.927	2.76	UDIYIP	152.163	3.476
UDONUX	153.328	3.259	UDONUX	155.503	3.358	UDIZEQ	163.243	2.83	UDIZEQ	158.984	3.27	UDONUX	176.757	3.054

UDONUX	161.475	2.748	UDOREO	167.221	3.338	UDONUX	163.515	3.126	UDONUX	169.916	3.49	UDONUX	163.905	3.204
UDOREO	162.248	3.42	UDOREO	153.745	2.803	UDOREO	162.424	3.178	UDOREO	162.404	2.951	UDOREO	158.522	3.034
UDOREO	152.226	3.286	UDOREO	151.394	3.228	UDOREO	153.877	3.194	UDOREO	154.473	2.782	UDOREO	157.933	3.067
UDOREO	151.423	3.005	UDOREO	171.625	3.034	UDOREO	162.044	2.798	UDOREO	153.256	3.004	UDOREO	155.677	2.843
UDOYUK	174.2	3.276	UDOYUK	162.018	3.255	UDOVIT	155.83	3.07	UDOVUE	150.507	3.108	UDOYUK	163.189	3.422
UDOYUK	156.321	3.038	UDOYUK	151.765	3.371	UDOYUK	162.922	3.037	UDOYUK	173.277	3.402	UDOYUK	156.028	3.255
UDOYUK	153.877	3.037	UDOYUK	174.26	3.372	UDOYUK	159.854	3.151	UDOYUK	155.219	3.306	UDOYUK	161.418	3.212
UDUPIU	163.286	3.303	UDUSAP	172.125	2.705	UDUBUU	163.868	3.458	UDUDII	150.979	3.161	UDUFEE	161.532	3.242
UDUTUI	161.226	3.003	UDUTUI	150.799	2.88	UDUSAP	152.787	3.475	UDUTUI	168.706	3.125	UDUTUI	165.209	2.911
UDUTUI	164.059	2.901	UDUTUI	159.633	3.471	UDUTUI	155.545	3.411	UDUTUI	165.029	3.043	UDUTUI	163.66	2.969
UDUXEY	151.024	2.838	UDUXEY	172.94	3.064	UDUXAU	154.824	3.005	UDUXAU	175.529	3.005	UDUXEY	178.935	3.225
UFAVUW	167.176	2.846	UFAVUW	150.254	3.411	UFACCU1	162.967	2.804	UFASAZ	156.995	3.253	UFAVIK	164.878	3.427
UFECOA	160.768	3.43	UFEDUF	151.009	2.868	UFAZOQ	160.662	3.323	UFAZOU	159.473	3.446	UFECOA	151.704	3.288
UFEPIH	159.334	3.203	UFEPIH	158.137	2.817	UFEDUF	163.466	3.318	UFEDUF	178.395	3.16	UFEPIH	158.516	2.717
UFEPIH	162.383	3.055	UFEPIH	162.336	3.219	UFEPIH	155.063	3.102	UFEPIH	151.483	3.343	UFEPIH	154.363	2.857
UFEVOU	154.776	3.427	UFEVUA	156.874	2.73	UFEPIH	156.344	3.04	UFEROO	153.46	2.866	UFEROO	157.859	2.868
UFEWAH	152.174	3.072	UFEWAH	164.915	3.074	UFEVUA	164.004	2.73	UFEVUA	156.447	3.431	UFEWAH	154.4	3.217
UFIMED	164.55	3.215	UFIMON	175.382	3.215	UFEWAH	152.594	3.216	UFIBOD	171.309	3.34	UFIJOL	166.854	3.062
UFIRIK	176.81	2.893	UFIROQ	177.764	2.887	UFINAC	154.699	3.083	UFIQIM	169.517	3.147	UFIQIM	161.393	2.895
UFIXOA	169.424	2.899	UFIXOA	160.511	3.072	UFITOW	157.124	3.267	UFIWEO	155.681	3.221	UFIXOA	150.431	3.243
UFIXOA	160.643	3.105	UFIYEP	156.328	3.16	UFIXOA	159.528	2.814	UFIXOA	159.676	3.119	UFIXOA	167.451	3.332
UFOJAC	170.866	3.474	UFOJAC	158.912	2.988	UFIYIS	164.996	2.732	UFOJAC	160.032	3.48	UFOJAC	151.958	2.822
UFOJAC	150.807	2.822	UFOJAC	150.338	2.817	UFOJAC	151.692	2.759	UFOJAC	151.325	2.894	UFOJAC	161.52	3.098
UFOJEI	171.915	3.041	UFOPUB	153.679	2.974	UFOJEG	153.703	3.101	UFOJEG	158.233	3.341	UFOJEI	163.399	2.971
UFOQAI	151.16	3.286	UFOQAI	156.248	3.424	UFOQAI	154.323	3.46	UFOQAI	152.526	3.198	UFOQAI	169.842	3.41
UFOQIQ	177.618	3.415	UFOQIQ	162.686	2.859	UFOQAI	166.866	3.194	UFOQAI	153.361	3.286	UFOQAI	157.374	3.045
UFOQIQ	150.807	3.109	UFOQIQ	153.555	2.886	UFOQIQ	155.357	2.944	UFOQIQ	151.003	2.966	UFOQIQ	171.083	2.871
UFOQIQ	155.065	3.108	UFOQIQ	150.599	3.432	UFOQIQ	161.763	2.939	UFOQIQ	169.582	2.899	UFOQIQ	157.723	3.108
UFOQIQ	173.948	2.939	UFOQIQ	162.151	3.063	UFOQIQ	162.523	3.063	UFOQIQ	164.528	2.859	UFOQIQ	161.861	3.067
UFOTIS	163.958	3.363	UFOTUI	152.95	2.78	UFOQIQ	169.845	2.965	UFOQIQ	157.283	2.944	UFOQIQ	168.534	2.871
UFOVEU	155.639	3.369	UFOVEU	160.249	3.369	UFOTUI	156.643	3.049	UFOTUI	162.19	3.019	UFOTUI	169.676	3.247
UFOVEU	155.088	2.899	UFOVEU	160.288	3.01	UFOVEU	161.656	2.95	UFOVEU	170.563	3.077	UFOVEU	152.291	2.895
UFOVEU	153.329	2.899	UFOVEU	171.142	2.946	UFOVEU	168.45	3.234	UFOVEU	158.196	2.628	UFOVEU	161.387	3.168
UFUJEK	159.422	3.202	UFUJEK	151.368	3.19	UFOVEU	168.192	3.135	UFUJEK	164.415	3.002	UFUJEK	172.416	3.183
UFUPER	153.899	3.224	UFUPER	163.437	3.257	UFUJEK	153.019	3.262	UFUJEK	172.201	3.312	UFUJEK	153.382	3.185
UFUVAS	163.006	2.922	UFUVAS	163.872	2.886	UFUPER	150.541	3.114	UFUPER	167.054	2.956	UFUPER	151.473	2.807
UGAHOD	154.241	3.286	UGAJIW	150.742	3.386	UFUVAS	173.375	3.001	UGAGAM	168.065	3.274	UGAHEQ	163.395	3.253
UGAMUO	155.063	3.215	UGAQEA	154.925	3.057	UGAMUO	152.048	3.215	UGAMUO	173.001	2.685	UGAMUO	156.169	2.806
UGAVOP0	160.117	3.121	UGAVUX	161.238	3.434	UGAQEA	150.761	3.17	UGAQEA	165.567	2.96	UGAQEA	164.406	2.96
UGEGIA	155.37	2.958	UGEGIA	165.855	3.102	UGAWIM	153.932	3.442	UGEBIV	161.507	3.247	UGEBIV	162.41	3.29
UGEGUM	157.106	2.88	UGEGUM	174.45	2.762	UGEGIA	164.75	3.008	UGEGIA	165.208	2.61	UGEGUM	158.36	2.903
UGEHEX	170.345	3.258	UGEHIB	165.357	3.287	UGEGUM	160.399	2.878	UGEHEX	150.1	3.054	UGEHEX	164.129	3.021
UGEHUN	173.629	3.139	UGEHUN	157.829	3.311	UGEHIB	150.731	3.263	UGEHIB	152.114	3.191	UGEHUN	153.561	3.442
UGEQII	151.931	3.49	UGEQII	161.414	3.151	UGEHUN	161.705	3.376	UGEJAV	160.371	3.416	UGEJIA	155.516	3.464
UGIJEB	155.607	3.389	UGILIF	171.685	3.435	UGEQII	155.377	2.928	UGEQII	163.178	3.151	UGEQUU	153.017	3.068
UGIRIN	152.228	2.816	UGITEN	164.967	2.803	UGILIF	169.947	2.893	UGILIF	166.692	2.851	UGILIF	169.871	3.196
UGOBIC	180	3.489	UGOBIC	152.209	3.044	UGITEN	152.779	3.062	UGOBIC	180	3.489	UGOBIC	166.719	3.303
UGOBOI	172.355	3.036	UGOLUY	158.059	2.879	UGOBIC	172.266	2.948	UGOBOI	165.352	3.424	UGOBOI	151.94	3.123
UGOROA	158.979	3.401	UGOROA	156.534	3.153	UGOLUY	155.052	3.212	UGOROA	169.909	3.469	UGOROA	151.038	2.882
UGORUH	168.424	3.137	UGOSES	164.541	3.016	UGOROA	156.494	2.772	UGORUH	151.624	3.31	UGORUH	159.458	2.696
UGOXOF	172.814	3.476	UGOXOF	160.107	3.334	UGOSES	175.384	2.796	UGOSOB	155.189	3.487	UGOXOF	161.839	3.001
UGOXOF	171.686	2.836	UGOXOF	158.545	2.842	UGOXOF	158.997	3.171	UGOXOF	165.002	3.137	UGOXOF	157.652	2.826
UGUCOS	154.968	3.065	UGUDAB	158.694	3.126	UGOXOF	151.582	3.403	UGUBOR	169.977	3.314	UGUBOR	163.847	3.103
UGUDOQ	156.978	3.258	UGUDOQ	150.985	2.698	UGUDAC	150.714	3.156	UGUDOQ	155.029	3.314	UGUDOQ	152.594	2.956
UGUDUW	154.091	3.243	UGUDUW	151.757	3.313	UGUDUW	164.815	3.467	UGUDUW	156.085	3.388	UGUDUW	150.221	2.871
UGUKEQ	176.601	2.913	UGUKIU	163.022	3.482	UGUDUW	173.635	3.395	UGUDUW	150.704	3.233	UGUDUW	164.301	3.239
UGUMUE(	154.649	3.407	UGUSOF	159.393	3.329	UGUMUE	150.563	3.35	UGUMUE(	170.758	3.161	UGUMUE(	158.084	3.287
UHAZAH	161.192	3.289	UHEBIV	170.725	3.207	UHAPIE	173.437	3.412	UHAPIE	163.574	3.412	UHAWIN	159.587	3.398
UHEFUL	157.212	3.154	UHEFUL	153.193	2.919	UHEFUL	170.042	3.195	UHEFUL	168.079	3.205	UHEFUL	150.21	3.486
UHIBIY	164.115	3.35	UHIHUR	158.365	3.132	UHEFUL	153.772	3.348	UHEMED	170.649	3.254	UHEVUB	170.536	3.317
UHINUV	153.138	3.352	UHIPAD	171.168	3.296	UHIHUR	160.229	3.373	UHIHUR	170.074	2.99	UHINUV	154.339	3.02
UHIQOT	150.564	3.24	UHIQOT	152.983	3.328	UHIPAD	164.913	3.354	UHIQOT	161.416	3.026	UHIQOT	177.123	3.026
UHIRAG	163.726	3.268	UHIVAM	163.401	2.597	UHIQUZ	176.538	3.476	UHIRAG	151.193	2.766	UHIRAG	159.861	3.038
UHIVAM	159.263	2.736	UHIVAM	162.243	3.491	UHIVAM	166.36	2.597	UHIVAM	170.11	3.284	UHIVAM	157.694	3.488
UHIXOB	151.094	3.308	UHIXOB	162.925	3.228	UHIVAM	158.104	2.68	UHIWAJ	170.488	2.873	UHIWAJ	154.66	2.948
UHIXOB	164.109	3.213	UHIXOB	158.601	3.486	UHIXOB	158.296	2.954	UHIXOB	170.484	3.409	UHIXOB	177.619	3.43
UHOCAV	178.641	3.458	UHOGUU	150.975	2.96	UHIXOB	161.078	3.148	UHIZOE	155.982	3.07	UHIZOE	166.102	3.446
UHOGUU	164.423	3.17	UHOGUU	159.749	3.174	UHOGUU	159.838	2.741	UHOGUU	166.979	2.933	UHOGUU	164.106	3.384
UHOGUU	152.571	3.088	UHOGUU	163.129	3.063	UHOGUU	153.044	3.212	UHOGUU	154.163	2.873	UHOGUU	158.082	3.247
UHOJIO	163.464	3.364	UHOJIO	166.397	3.346	UHOGUU	174.934	2.885	UHOGUU	150.042	3.422	UHOGUU	154.94	3.306
UHOLIQ	158.706	2.938	UHOMUC	162.068	2.905	UHOJIO	167.11	2.972	UHOJIO	162.658	2.98	UHOLIQ	155.64	3.013
UHOXOE	163.887	3.092	UHOZUN	154.356	2.945	UHONAJ	162.857	3.083	UHONEN	171.052	3.109	UHOXOE	159.067	2.785
UHUBEF	153.584	2.782	UHUBOP	157.576	3.456	UHOZUN	163.528	2.632	UHOZUN	152.914	2.996	UHOZUN	158.618	3.375
UHUBUY	159.728	3.137	UHUHUC	170.806	3.24	UHUBOP	150.411	2.955	UHUBOP	154.999	3.085	UHUBOP	162.165	3.07
UHUXON	158.162	2.978	UHUXON	173.213	3.15	UHUHUC	165.609	3.278	UHUNOA	154.919	2.955	UHUWAV	171.325	3.113
UHUXON	164.569	3.429	UHUXON	162.708	3.443	UHUXON	151.945	2.848	UHUXON	163.459	3.181	UHUXON	156.645	2.997
UHUYAA	158.459	2.895	UHUYAA	151.853	2.562	UHUYAA	164.421	2.714	UHUYAA	152.09	3.092	UHUYAA	167.099	3.469
UHUYII	160.729	3.486	UHUYII	156.292	2.784	UHUYAA	162.195	2.838	UHUYII	178.014	2.818	UHUYII	174.213	2.994
UHUYII	163.233	3.087	UHUYII	168.745	3.251	UHUYII	166.154	2.93	UHUYII	173.168	3.456	UHUYII	156.348	3.17

UHUYII	165.036	2.957	UHUYII	160.027	2.832	UHUYII	155.779	3.003	UHUYII	150.891	3.463	UHUYII	162.188	3.456
UHXAPY1	168.475	3.178	UHXAPY1	156.968	3.259	UHUYII	156.769	3.227	UHXAPY1	158.593	2.779	UHXAPY1	150.991	3.328
UJABAI	151.191	2.927	UJABAI	163.368	3.123	UHXAPY1	159.786	3.191	UHXAPY1	155.444	3.129	UJABAI	169.684	2.831
UJABAI	170.868	2.999	UJABAI	175.137	3.104	UJABAI	169.141	2.982	UJABAI	166.742	2.929	UJABAI	163.206	3.043
UJABEM	151.442	2.927	UJABOW	165.068	3.411	UJABEM	171.094	3.15	UJABEM	155.055	2.927	UJABEM	150.091	3.248
UJAGOB	156.671	3.451	UJAJUO	162.868	3.034	UJABOW	166.725	3.403	UJABUC	165.878	3.062	UJABUC	163.424	3.205
UJAMEA	160.152	2.914	UJAZIP	164.843	2.741	UJAJUO	163.331	2.767	UJAJUO	153.553	3.065	UJAJUO	174.733	3.345
UJAZOV	157.319	3.157	UJAZOV	172.085	2.871	UJAZIP	151.809	2.913	UJAZIR	166.126	3.041	UJAZOV	155.288	3.129
UJAZUB	160.039	2.761	UJAZUB	159.531	3.451	UJAZOV	167.816	3.099	UJAZOV	176.943	3.254	UJAZOV	171.628	2.945
UJAZUB	150.766	3.451	UJEBER	154.797	3.31	UJAZUB	153.884	3.399	UJAZUB	155.11	2.88	UJAZUB	165.829	2.88
UJERAF	153.403	3.088	UJIBIB	175.647	3.289	UJEBER	160.247	3.252	UJEBER	173.081	2.918	UJEKAW	152.187	2.934
UJIBUN	169.882	3.457	UJIBUN	176.533	2.942	UJIBIB	165.212	3.044	UJIBOH	172.881	2.889	UJIBOH	163.456	3.325
UJICEY	164.65	3.222	UJICEY	174.401	3.074	UJIBUN	160.092	3.247	UJIBUN	161.399	3.452	UJICEY	163.561	2.926
UJIDOJ	164.004	2.84	UJIFOL	173.206	2.674	UJIDAV	161.877	2.685	UJIDEZ	160.497	2.615	UJIDEZ	174.085	3.425
UJIGAY	174.1	3.049	UJIREM	165.287	2.94	UJIFOL	160.901	2.674	UJIGAY	162.598	2.955	UJIGAY	163.902	3.236
UJIREM	174.124	3.319	UJIREM	157.395	3.223	UJIREM	170.367	3.302	UJIREM	151.764	3.358	UJIREM	161.862	3.417
UJISAJ	172.863	3.01	UJISAJ	150.866	2.554	UJIROW	163.874	3.393	UJIROW	176.253	3.132	UJIRUE	168.584	3.3
UJISEL	165.623	3.061	UJISEL	154.347	2.964	UJISAJ	154.866	3.165	UJISAJ	173.833	3.01	UJISEL	158.394	3.201
UJISOX	171.101	3.367	UJISOX	159.461	3.074	UJISEL	157.21	3.009	UJISOX	163.812	2.719	UJISOX	159.345	2.828
UJIXIX	151.074	3.385	UJIXIX	157.952	3.4	UJISOX	172.723	3.299	UJISOX	175.122	3.127	UJIXIX	178.875	3.07
UJOJEM	150.714	3.146	UJOJEM	156.808	3.179	UJIXIX	156.507	2.944	UJIXIX	152.958	3.171	UJIXIX	159.676	3.287
UJOJEM	152.325	3.19	UJOJIQ	155.526	2.893	UJOJEM	162.894	3.006	UJOJEM	156.724	3.098	UJOJEM	162.022	2.961
UJOJIQ	151.665	3.268	UJOJIQ	161.702	2.769	UJOJIQ	160.561	2.878	UJOJIQ	152.995	2.826	UJOJIQ	163.282	3.25
UJOJIQ	167.932	3.26	UJOJIQ	157.593	3.22	UJOJIQ	153.665	3.283	UJOJIQ	151.01	3.29	UJOJIQ	158.359	3.102
UJONOY	157.008	3.011	UJONOZ	151.904	3.42	UJOJIQ	173.298	2.912	UJONOY	154.67	3.023	UJONOY	153.143	2.844
UJONUE	156.169	3.469	UJONUF	172.442	3.419	UJONUE	153.69	3.054	UJONUE	177.882	2.852	UJONUE	154.515	3.054
UJOPAM	164.973	3.161	UJOPAM	162.24	2.696	UJONUF	151.72	3.331	UJOPAM	157.751	2.971	UJOPAM	150.029	3.33
UJOPAM	152.224	3.454	UJOPAM	169.972	3.078	UJOPAM	171.6	3.324	UJOPAM	150.823	3.297	UJOPAM	157.021	2.978
UJOPAN	152.302	3.377	UJOPAN	171.816	3.487	UJOPAM	157.127	2.672	UJOPAM	152.965	3.066	UJOPAM	167.879	3.297
UJOQEQ	163.852	3.42	UJOQEQ	168.673	3.068	UJOPER	151.932	3.4	UJOPUF	164.198	3.27	UJOQEQ	171.334	2.764
UJOQIV	174.899	3.119	UJOSAQ	150.506	3.227	UJOQEQ	175.689	3.164	UJOQEQ	170.569	2.986	UJOQIV	170.073	3.119
UJOSIZ01	158.819	2.719	UJUCIL	150.033	3.086	UJOSAQ	167.872	2.595	UJOSEU	150.232	3.261	UJOSEU	167.673	2.629
UJUCOR	151.673	2.904	UJUCOR	158.119	3.229	UJUCIL	150.668	2.914	UJUCIL	158.829	3.248	UJUCIL	172.968	2.994
UJUHEN	160.366	2.679	UJUVAY	155.898	2.975	UJUCOR	172.801	2.998	UJUHAJ	154.283	2.956	UJUHAJ	165.444	3.325
UJUVAY	155.54	3.3	UJUVAY	173.604	3.296	UJUVAY	155.582	3.411	UJUVAY	167.733	3.166	UJUVAY	150.834	2.929
UJUVEC	159.184	2.992	UJUVEC	167.949	3.428	UJUVAY	159.318	2.889	UJUVAY	150.388	3.414	UJUVEC	152.145	3.229
UKAFIX	152.016	3.234	UKAFOD	154.805	3.499	UJUVEC	150.752	3.185	UJUVEC	159.543	3.235	UJUVEC	171.269	3.428
UKAJAR	151.544	2.753	UKAJAR	157.772	2.716	UKAFOD	150.644	3.499	UKAHUJ	163.714	2.571	UKAJAR	150.289	2.813
UKAMAX	159.432	3.354	UKAMAX	167.727	3.266	UKAJAR	154.833	3.332	UKAMAX	157.813	3.346	UKAMAX	172.786	3.1
UKAMAX	154.981	3.279	UKAMAX	151.099	3.071	UKAMAX	165.168	2.833	UKAMAX	169.5	3.328	UKAMAX	159.564	3.471
UKAMAX	153.124	3.187	UKAMAX	158.338	2.951	UKAMAX	154.776	3.071	UKAMAX	151.637	3.245	UKAMAX	151.595	3.245
UKAMAX	163.848	3.313	UKAMIF	168.6	2.894	UKAMAX	158.812	2.98	UKAMAX	153.569	3.093	UKAMAX	151.639	3.213
UKAPAZ	160.309	2.952	UKATUX	150.417	3.044	UKAMIF	175.437	2.933	UKAMIF	152.712	3.48	UKAMIF	160.571	2.948
UKEFOI	154.328	3.259	UKEFOI	161.646	3.048	UKATUX	170.628	2.828	UKATUX	155.287	2.869	UKEFOI	154.455	2.814
UKEMED	151.773	3.486	UKICAT	153.107	3.277	UKEFOI	153.262	2.909	UKEKAW0	151.313	2.931	UKEMED	157.047	3.164
UKITUF	163.727	2.791	UKITUF	152.583	2.75	UKITUF	167.884	3.476	UKITUF	154.072	3.114	UKITUF	170.211	3.476
UKIWUK	151.561	3.406	UKIXOF	151.492	3.437	UKITUF	175.233	3.112	UKITUF	171.363	3.242	UKITUF	155.665	3.098
UKOKIO01	156.317	3.371	UKOKIO01	155.016	3.021	UKOJEN	157.336	3.389	UKOKIO01	170.053	3.031	UKOKIO01	150.871	3.003
UKONIS	154.577	3.21	UKORER	151.657	3.3	UKOKUA	156.371	3.462	UKOLEL	158.937	3.388	UKOMAJ	152.826	3.199
UKOYEA	162.919	3.257	UKOZAW	174.31	3.417	UKORER	162.507	3.204	UKOROB	155.334	3.157	UKOROB	154.955	3.089
UKOZUR	152.76	3.164	UKOZUR	151.06	2.913	UKOZUR	152.769	3.18	UKOZUR	150.096	2.917	UKOZUR	151.801	3.248
UKOZUR	152.299	2.834	UKOZUR	158.864	2.896	UKOZUR	154.324	3.128	UKOZUR	155.779	3.212	UKOZUR	156.91	3.157
UKUBAF	152.413	3.26	UKUBAF	152.363	2.845	UKOZUR	170.115	3.074	UKOZUR	155.636	2.855	UKOZUR	159.113	3.114
UKUBAF	150.095	2.881	UKUBAF	150.005	3.271	UKUBAF	160.127	3.427	UKUBAF	155.446	2.812	UKUBAF	156.996	3.479
UKUBAF	162.475	2.742	UKUBAF	150.706	2.971	UKUBAF	168.702	2.849	UKUBAF	166.577	3.414	UKUBAF	155.262	2.893
UKUBAF	157.163	3.354	UKUBAF	165.139	3.081	UKUBAF	159.834	2.881	UKUBAF	156.933	3.081	UKUBAF	159.097	2.794
UKUBAF	163.673	3.032	UKUBAF	157.468	3.375	UKUBAF	153.426	2.948	UKUBAF	159.811	3.255	UKUBAF	162.154	3.108
UKULOF	154.916	3.051	UKULOF	158.872	3.004	UKUBAF	159.7	3.108	UKUBAF	159.474	2.948	UKULOE	157.236	3.405
UKULOF	153.753	3.452	UKULOF	160.919	2.969	UKULOF	156.603	3.054	UKULOF	155.774	3.393	UKULOF	152.207	3.324
UKUNUK	153.143	2.831	UKUNUK	154.239	2.831	UKULUI	175.236	2.811	UKULUL	156.14	3.078	UKUNUK	168.29	2.847
UKUPEW0	176.681	3.151	UKUQAV	156.952	3.453	UKUNUK0	152.875	2.841	UKUNUK0	168.367	2.859	UKUNUK0	154.418	2.841
ULANEB	160.555	3.395	ULAQOQ	150.041	3.185	UKUQAV0	155.42	3.489	UKURUQ	154.324	2.911	ULADUI	172.561	3.245
ULAVAE	160.166	2.806	ULAVAE	164.645	2.722	ULAQOQ	170.192	3.083	ULATAG	154.889	3.199	ULAVAE	158.144	3.268
ULEBOG	153.687	3.447	ULECOE	155.17	3.173	ULAZAM	150.731	3.116	ULAZAM	150.841	3.339	ULAZAM	154.843	2.658
ULELUS	168.003	2.819	ULELUS	163.908	2.812	ULEFAT	169.892	3.193	ULELUS	153.174	3.061	ULELUS	172.122	3.129
ULEMAZ	165.296	3.241	ULEMAZ	166.713	3.177	ULELUS	159.56	2.932	ULEMAZ	154.994	2.913	ULEMAZ	154.03	3.352
ULEPUX	161.739	3.248	ULEPUX	157.547	3.476	ULEPUX	152.716	3.289	ULEPUX	150.712	3.493	ULEPUX	166.868	3.367
ULETAJ	156.43	2.906	ULETAJ	151.937	2.906	ULEPUX	163.068	2.802	ULEPUX	152.852	3.243	ULEPUX	162.813	3.36
ULEVOZ	158.697	3.396	ULEVOZ	154.822	3.085	ULEVOZ	155.416	3.092	ULEVOZ	160.275	3.364	ULEVOZ	151.115	2.947
ULICOI	162.09	2.867	ULIHAB	159.194	2.888	ULEVOZ	175.149	2.911	ULEWEQ	160.72	3.175	ULEZOC	166.844	3.127
ULIKUW	157.597	3.347	ULIKUW	169.366	3.358	ULIJAE	159.931	3.333	ULIJAE02	158.435	3.187	ULIJAE02	152.568	3.42
ULINAE	161.327	3.108	ULINAE	156.644	3.119	ULIMOV	154.085	2.788	ULIMOV	157.656	2.855	ULINAE	152.061	3.287
ULINAE	156.145	2.813	ULINAE	151.414	3.19	ULINAE	159.888	3.305	ULINAE	159.008	3.007	ULINAE	159.426	3.21
ULINAE	172.203	2.777	ULIQOV	169.482	2.723	ULINAE	165.266	3.146	ULINAE	168.784	3.362	ULINAE	151.589	2.995
ULIVIV	152.358	3.334	ULIVIV	158.215	3.177	ULITIT	150.407	2.778	ULITIV	151.996	2.937	ULITOZ	157.944	2.912
ULIWES	152.18	3.189	ULIWES	153.619	2.932	ULIVIV	152.813	3.014	ULIVIV	160.5	2.996	ULIVIV	169.704	3.465
ULIWES	161.903	3.058	ULIWIW	157.886	3.089	ULIWES	153.303	2.904	ULIWES	166.344	2.982	ULIWES	174.456	3.476
ULIWIW	167.063	3.243	ULIWIW	163.672	3.104	ULIWIW	169.687	2.977	ULIWIW	153.31	2.78	ULIWIW	155.399	3.315
ULIWOC	171.671	3.318	ULIWOC	160.91	2.926	ULIWIW	155.905	2.772	ULIWOC	166.818	3.403	ULIWOC	166.395	3.348

ULOCII	172.9	2.658	ULOCII	158.018	2.954	ULIWUI	164.579	2.868	ULIWUI	159.749	2.742	ULIWUI	155.971	2.971
ULOCII	156.739	3.355	ULOCII	158.411	3.495	ULOCII	157.183	2.912	ULOCII	157.63	2.998	ULOCII	165.918	3.376
ULOCOO	164.858	3.2	ULOCOO	177.32	2.934	ULOCII	170.714	3.042	ULOCII	159.518	3.219	ULOCII	163.658	2.658
ULODAB	161.107	3.103	ULODEF	170.738	3.389	ULOCOO	153.775	3.392	ULOCOO	161.782	2.731	ULODAB	155.001	3.469
ULONAM	158.679	3.17	ULONEP	157.891	3.457	ULOKIS	158.689	2.928	ULOKOY	159.209	2.922	ULONAM	159.478	3.097
ULOSAR	176.426	3.428	ULOSAR	151.46	3.374	ULONEP	155.523	2.895	ULONEP	162.341	3.238	ULONEP	169.025	3.159
ULUQID02	162.619	2.72	ULUQID02	150.775	2.808	ULOSAR	164.955	2.958	ULUQID02	155.457	3.307	ULUQID02	153.973	3.133
ULUQUO	155.726	2.882	ULUSID	158.688	3.038	ULUQID02	165.698	3.429	ULUQID02	152.809	2.791	ULUQID02	157.2	2.947
UMAROP	172.429	3.42	UMAROP	160.519	2.804	ULUZUZ	155.137	3.094	UMADIV	152.871	3.441	UMADIV	157.825	2.988
UMAYOY	150.086	2.891	UMEBOD	151.989	3.469	UMAROP	155.738	3.457	UMAROP	153.442	3.268	UMAYOY	160.391	2.891
UMEFUQ	168.451	3.412	UMEFUQ	151.956	2.895	UMEBOD	157.197	2.836	UMEBOD	152.15	3.354	UMEFUQ	162.991	3.249
UMEGAX	161.326	3.111	UMEGEB	163.519	2.979	UMEFUQ	168.253	3.348	UMEFUQ	164.836	3.249	UMEGAX	162.429	3.329
UMEGIF	175.235	3.25	UMEGOL	150.963	3.215	UMEGEB	168.831	2.861	UMEGEB	151.141	3.43	UMEGIF	163.87	3.31
UMEGOL	150.86	3.063	UMEGUR	150.461	3.11	UMEGOL	153.689	2.976	UMEGOL	165.225	3.46	UMEGOL	154.059	3.129
UMEGUR	168.825	3.414	UMEQIQ	151.437	3.358	UMEGUR	154.08	3.03	UMEGUR	152.697	3.094	UMEGUR	165.996	3.118
UMEVEN	155.651	3.22	UMEVEN	157.524	3.22	UMEROX	152.688	3.097	UMESAJ	153.378	3.282	UMEVEN	159.065	3.019
UMEVOX0	150.822	3.281	UMEVOX0	153.759	2.921	UMEVOX	158.468	2.999	UMEVOX	155.378	3.228	UMEVOX	153.714	3.228
UMEYOD	173.916	3.467	UMEYOD	161.264	3.287	UMEVOX0	159.572	3.028	UMEVOX0	161.554	3.028	UMEYOD	167.608	3.084
UMIPUF	165.645	2.797	UMIPUF	156.105	3.227	UMIMIQ	151.102	2.58	UMIMIQ	155.463	3.265	UMINEN	160.612	3.16
UMITIX	164.824	2.837	UMITIX	173.773	3.238	UMIQUD	156.769	3.492	UMIQUD	177.063	3.313	UMITIX	163.914	3.214
UMITIX	153.422	2.98	UMITIX	151.933	3.447	UMITIX	153.354	3.166	UMITIX	152.637	3.045	UMITIX	155.958	2.683
UMOHAI	166.708	2.919	UMOHAI	169.551	3.461	UMITIX	166.843	2.893	UMITIX	164.801	3.309	UMIWIY	161.997	3.421
UMOHAI	159.159	3.125	UMOHAI	165.881	2.906	UMOHAI	164.837	3.125	UMOHAI	158.764	2.817	UMOHAI	151.63	3.092
UMOJIR	162.719	2.991	UMOJIR	152.694	3.074	UMOJIR	167.699	2.964	UMOJIR	164.687	3.273	UMOJIR	161.446	3.035
UMOSIB	157.025	3.194	UMOSIB	160.971	3.171	UMOLAL	150.299	2.946	UMOSAT	156.151	3.198	UMOSAT	162.931	3.128
UMUBEK	161.816	3.091	UMUBEK	173.728	3.284	UMOSIB	156.829	3.486	UMOSIB	162.471	3.486	UMUBEK	164.066	3.484
UMUHEP0	168.032	3.47	UMUHEP0	151.885	3.101	UMUFIS	164.512	3.205	UMUHEP0	178.04	3.117	UMUHEP0	151.894	3.002
UMUHEP0	152.629	3.427	UMUHEP0	162.146	3.095	UMUHEP0	154.058	2.958	UMUHEP0	166.421	3.438	UMUHEP0	160.617	3.148
UMUHOZ	166.685	2.866	UMUHOZ	162.731	3.213	UMUHEP0	151.93	3.398	UMUHEP0	156.737	3.39	UMUHEP0	161.072	2.753
UMUWOR	160.595	3.422	UNADUK	164.377	3.031	UMUHOZ	163.391	3.197	UMUPIC	162.341	3.134	UMUSAY	166.894	2.829
UNAFAS	161.594	2.966	UNAFAS	160.957	2.975	UNADUK	165.361	3.008	UNAFAS	164.253	2.932	UNAFAS	163.614	2.897
UNALIH	169.26	3.475	UNAMOO	154.207	3.243	UNAFUN	151.982	2.917	UNAFUN	164.288	3.367	UNAHOI	155.647	3.463
UNANUV	158.13	3.108	UNANUV	167.228	3.345	UNAMUU	163.735	3.289	UNAMUU	166.172	3.057	UNANUV	154.714	3.294
UNEKAA	155.631	3.121	UNEKAA	156.318	2.895	UNAVOU	159.557	2.968	UNAVOU	155.329	3.105	UNEKAA	150.912	2.863
UNEKOO	151.703	2.993	UNEKOO	163.195	2.726	UNEKAA	168.574	3.31	UNEKEE	153.891	3.49	UNEKEE	161.011	2.866
UNETAL	153.097	3.428	UNETAL	155.763	2.768	UNETAL	151.612	3.19	UNETAL	152.398	3.03	UNETAL	165.731	3.369
UNETAL	152.839	3.118	UNETOY	171.172	3.402	UNETAL	153.126	3.336	UNETAL	151.524	3.03	UNETAL	167.981	3.357
UNICOM	163.662	3.02	UNITOD	163.14	3.02	UNETOY	162.739	3.399	UNICOM	177.379	3.11	UNICOM	154.075	3.02
UNUJOF	159.088	3.343	UNUKOG	161.425	2.85	UNITOD	166.65	3.043	UNONET	172.736	3.302	UNOXIF	176.395	2.598
UPAFOH	167.328	3.455	UPATAI	151.77	2.972	UNULIB	178.129	2.898	UNUZIN	162.998	3.146	UNUZIN	152.929	3.326
UPAXOB	166.989	3.076	UPAXUH	164.135	3.228	UPAWEO0	154.529	3.495	UPAWEO0	159.267	3.321	UPAXOB	164.13	3.381
UPIGAE	166.119	3.152	UPIMAJ	154.674	3.471	UPAXUH	170.521	3.251	UPAXUH	170.639	3.048	UPECUO	166.965	3.051
UPIMAJ	154.927	2.962	UPIMAJ	174.05	2.837	UPIMAJ	167.422	2.969	UPIMAJ	173.906	2.911	UPIMAJ	162.655	3.08
UPIMUE	151.448	3.262	UPIMUE	150.3	2.642	UPIMUE	163.201	2.73	UPIMUE	174.483	3.093	UPIMUE	168.781	3.38
UPIPAN	170.65	2.789	UPIPAN	166.413	2.855	UPIMUE	155.927	3.438	UPIMUE	166.44	3.086	UPIPAN	174.027	2.921
UPIPAN	160.33	2.836	UPIPAN	156.23	2.845	UPIPAN	170.345	2.777	UPIPAN	166.728	2.872	UPIPAN	174.356	2.915
UPIPER	161.138	2.723	UPIPER	154.247	3.034	UPIPAN	155.48	2.827	UPIPAN	161.006	2.854	UPIPER	163.579	3.005
UPIPER	158.294	2.836	UPIPER	161.308	2.83	UPIPER	159.02	3.074	UPIPER	160.084	3.016	UPIPER	156.128	3.286
UPIPER	159.969	3.026	UPIPER	159.311	2.907	UPIPER	159.279	2.921	UPIPER	161.172	3.266	UPIPER	159.753	3.384
UPIPER	164.448	2.73	UPIPER	164.545	2.781	UPIPER	165.984	2.899	UPIPER	168.749	2.72	UPIPER	164.107	2.879
UPIPER	161.971	3.181	UPIQAO	170.254	2.891	UPIPER	161.457	2.755	UPIPER	164.099	3.115	UPIPER	152.838	2.98
UPIQAO	165.648	3.118	UPIQAO	162.306	3.203	UPIQAO	172.027	3.017	UPIQAO	166.622	2.917	UPIQAO	166.601	3.179
UPIQES	158.477	3.488	UPIQIW	155.569	2.851	UPIQES	151.793	3.074	UPIQES	161.762	3.415	UPIQES	166.915	2.91
UPIQIW	178.396	3.385	UPISAP	166.764	3.104	UPIQIW	164.681	2.956	UPIQIW	162.901	3.065	UPIQIW	157.679	3.126
UPIVAS	154.765	2.884	UPIYUP	159.776	3.002	UPISET	150.24	3.17	UPISET	164.473	3.189	UPISET	154.965	3.208
UPOFOW	152.393	3.066	UPOFOW	156.152	3.312	UPOBUZ	165.059	2.777	UPOFEM	169.23	2.985	UPOFOW	161.04	3.172
UPOGUD	150.378	3.22	UPOGUD	155.57	3.126	UPOGAJ	162.569	3.138	UPOGIR	155.056	3.285	UPOGUD	178.416	3.435
UPOQOH	151.681	3.279	UPOQOH	155.203	2.875	UPOQOH	157.532	3.186	UPOQOH	165.98	3.252	UPOQOH	169.156	2.858
UPOQUN	150.657	3.114	UPOQUN	152.783	2.811	UPOQOH	165.9	3.099	UPOQOH	155.813	3.279	UPOQOH	166.391	2.862
UPOQUN	162.16	3.005	UPOQUN	167.037	3.316	UPOQUN	160.049	2.773	UPOQUN	151.202	3.303	UPOQUN	155.128	2.729
UPOQUN	171.969	3.396	UPOQUN	151.348	3	UPOQUN	173.43	3.276	UPOQUN	157.304	3.302	UPOQUN	150.412	3.216
UPUJIZ	150.836	3.396	UPULOH	154.715	3.148	UPOQUN	159.551	2.868	UPUFIX	167.899	3.329	UPUJIZ	166.198	3.267
UPULOH	165.495	3.019	UPUNOL	152.271	2.851	UPULOH	151.417	3.286	UPULOH	157.069	2.983	UPULOH	161.441	2.949
UPUNOL	152.861	2.859	UPUSOP	154.261	2.757	UPUNOL	153.017	2.988	UPUNOL	164.677	3.197	UPUNOL	155.028	3.46
UPUSOP	155.382	3.453	UPUSOP	171.745	2.904	UPUSOP	153.019	3.151	UPUSOP	151.289	3.43	UPUSOP	166.978	3.413
UQAGEB	151.713	2.79	UQAGEB	154.648	2.79	UPUWAG	168.441	3.255	UPUWAG	161.997	3.255	UQAGEB	150.345	2.818
UQAHAY	157.894	3.447	UQEJEH	154.397	3.136	UQAGUR	174.606	3.037	UQAGUR	153.302	3.308	UQAHAY	174.324	3.029
UQEJEH	172.533	2.871	UQEJEH	170.271	3.427	UQEJEH	155.815	3.469	UQEJEH	150.116	3.328	UQEJEH	155.831	2.973
UQEJEH	164.674	3.389	UQEJEH	159.333	2.97	UQEJEH	151.568	2.883	UQEJEH	165.284	3.314	UQEJEH	154.694	3.095
UQEJEH	151.112	2.97	UQEJEH	163.033	2.859	UQEJEH	174.213	3.116	UQEJEH	155.274	3.136	UQEJEH	160.95	2.958
UQEJIL	171.203	3.287	UQEJIL	176.206	3.075	UQEJIL	175.557	2.865	UQEJIL	165.611	3.419	UQEJIL	167.49	2.975
UQEJOR	175.971	2.833	UQEJOR	156.985	2.962	UQEJOR	165.904	2.824	UQEJOR	159.341	2.825	UQEJOR	150.713	2.848
UQEJUX	152.67	3.154	UQEJUX	151.276	3.481	UQEJOR	152.484	2.939	UQEJOR	155.209	3.29	UQEJUX	158.436	2.983
UQEJUX	168.579	3.349	UQEKEJ	152.198	3.276	UQEJUX	154.372	2.891	UQEJUX	163.912	2.871	UQEJUX	167.719	3.11
UQEKOT	150.085	3.216	UQEKOT	152.525	3.322	UQEKEJ	156.356	3.372	UQEKOT	161.06	2.89	UQEKOT	155.698	3.026
UQEVAO	164.34	2.839	UQIFIK	150.941	3.431	UQELEK	171.887	3.069	UQEVAO	160.705	2.991	UQEVAO	173.288	3.253
UQOCOU	168.343	3.171	UQOCOU	162.336	3.171	UQIMAJ	160.254	3.166	UQIMAK	154.738	3.311	UQIMAK	176.957	3.311
UQOVAA	158.338	2.797	UQOVAA	161.383	2.649	UQOTUS	157.746	2.801	UQOTUS	161.181	2.645	UQOTUS	161.885	3.105
UQUHIY	164.985	2.903	UQUHIY	153.926	3.23	UQOVAA	161.552	3.108	UQUHIY	165.781	3.176	UQUHIY	164.719	2.933

UQUHIY	162.627	3.015	UQUPUU	156.385	3.356	UQUHIY	156.343	3.041	UQUHIY	154.064	3.026	UQUHIY	154.838	2.919
UQUPUU	159.202	3.236	UQUPUU	163.732	3.181	UQUPUU	157.046	2.725	UQUPUU	156.194	3.131	UQUPUU	156.894	3.162
URAPIO	154.325	2.802	URAPIO	155.173	3.424	UQUSUX	158.514	3.474	UQUSUX	152.585	3.44	UQUWEK	173.701	2.721
URELIO	156.692	2.807	URELIO06	157.002	2.651	URAPOU	161.353	2.561	URAQAH	153.869	2.791	URAQAH	154.272	3.442
URIGAF	163.102	3.116	URIGEJ	159.986	2.888	URIFIM	154.776	3.104	URIGAF	150.037	3.034	URIGAF	158.17	3.162
UROCAI	159.401	2.89	UROCEM	153.679	2.839	URIGEJ	165.066	3.289	URIGIN	164.125	3.038	UROCAI	169.058	3.269
UROGUG	159.302	3.333	UROGUG	163.031	2.907	UROGUG	169.582	3.195	UROGUG	150.784	3.456	UROGUG	155.34	3.488
UROHIV	168.772	3.1	UROZOT	173.81	3.432	UROGUG	158.251	2.929	UROGUG	169.058	2.897	UROHAN	153.679	2.845
UROZOT	155.421	2.897	UROZOT	153.276	2.897	UROZOT	159.844	3.063	UROZOT	152.193	3.099	UROZOT	163.379	2.916
URUNEC	153.547	3.156	URUNEC	161.831	3.481	URUKOI	156.994	3.352	URUNAY	152.742	3.08	URUNAY	152.095	3.299
URUNOM	151.214	3.145	URUNOM	167.773	3.26	URUNEC	167.437	3.042	URUNEC	161.352	3.026	URUNEC	172.674	3.031
URUNOM	162.884	3.059	URUNOM	155.16	3.164	URUNOM	169.934	2.875	URUNOM	150.581	3.181	URUNOM	162.617	3.177
URUPOO	166.541	3.34	URUPOO	161.345	2.655	URUPOO	158.465	3.184	URUPOO	150.625	2.757	URUPOO	165.633	2.845
URUPUT	171.575	3.16	URUVUB	172.683	3.2	URUPOO	166.278	3.379	URUPOO	167.65	2.936	URUPOO	154.964	3.076
USALOQ	171.251	3.471	USALOQ	150.68	2.901	URUVUB	167.548	3.017	URUVUB	156.549	3.172	URUVUB	162.299	3.188
USALOQ	163.275	2.725	USALUW	167.845	2.9	USALOQ	169.036	3.469	USALOQ	155.332	3.469	USALOQ	170.452	3.246
USALUW	163.806	3.188	USALUW	163.571	3.086	USALUW	173.519	2.845	USALUW	162.543	3.356	USALUW	151.03	3.356
USECOM	151.893	2.972	USECOM	151.089	2.713	USALUW	150.585	3.203	USALUW	150.935	3.353	USALUW	167.798	2.757
USECOM	159.572	3.295	USECOM	167.911	2.821	USECOM	152.237	3.334	USECOM	150.702	3.44	USECOM	155.318	3.412
USECOM	150.079	3.416	USECOM	170.35	3.288	USECOM	170.466	2.738	USECOM	153.356	2.92	USECOM	157.462	2.955
USECUS	171.106	2.84	USECUS	152.019	2.972	USECOM	154.937	2.738	USECOM	155.623	2.977	USECOM	167.548	2.955
USECUS	152.584	3.264	USECUS	154.812	3.372	USECUS	170.327	2.801	USECUS	150.467	2.751	USECUS	152.004	2.979
USECUS	154.521	2.998	USECUS	158.376	2.801	USECUS	151.815	3.459	USECUS	168.601	2.958	USECUS	151.089	2.883
USESES	156.071	3.35	USETIX	156.262	3.357	USECUS	172.36	3.345	USECUS	160.652	2.958	USECUS	161.797	3.449
USIHUC	168.388	3.274	USIJUE	163.191	3.085	USIGAH	176.389	3.355	USIGAH	154.262	3.3	USIHIO	172.615	2.668
USINOC	165.37	3.435	USINOC	159.394	3.041	USIKOX	168.147	3.021	USILOA	156.866	3.22	USILUG	171.621	3.037
USIZUS	153.682	3.071	USODEO	171.559	2.845	USIPEU	174.729	2.861	USIYED	150.732	3.239	USIYED	151.644	3.243
USUHEW	163.064	3.343	USURUW	178.607	3.379	USOFOA	162.238	2.838	USOFOA	162.598	3.113	USOMEW	177.063	2.915
USURUW	151.118	3.383	USURUW	173.199	3.282	USURUW	164.865	2.745	USURUW	156.937	3.481	USURUW	170.623	3.068
USUSAD	158.598	3.266	USUSAD	160.475	3.266	USURUW	158.113	3.068	USURUW	173.678	2.745	USURUW	154.822	2.713
USUSEH	178.064	3.132	USUSEH	154.916	3.262	USUSAD	155.648	2.802	USUSAD	158.066	2.891	USUSEH	166.997	2.816
UTATEP	154.844	3.096	UTATEP	161.553	3.183	UTAJEG	156.468	3.085	UTAQUE	163.553	3.477	UTAQUE	165.651	3.1
UTATIT	165.839	3.279	UTATIT	155.189	3.128	UTATEP	166.478	3.38	UTATEP	172.319	3.194	UTATEP	170.577	3.409
UTEBUS	150.481	3.207	UTEWIB	162.232	3.417	UTATIT	169.112	3.455	UTATIT	172.058	3.224	UTATIT	160.086	3.123
UTIHOV	155.821	2.665	UTIHOV	151.022	3.009	UTEXID	175.643	3.287	UTEXID	155.449	3.246	UTIGOW	174.211	3.485
UTODEP	156.72	3.206	UTODEP	150.928	2.972	UTIZAZ	173.061	3.238	UTIZAZ	153.024	3.238	UTODEP	159.971	2.742
UTOGOB	159.174	3.171	UTOGOB	157.825	3.095	UTODOX	151.342	3.456	UTODOX	161.019	3.353	UTODOX	150.682	3.074
UTOMAT	159.917	2.667	UTOMAT	151.218	3.296	UTOGOB	158.431	3.113	UTOMAT	166.809	2.767	UTOMAT	151.224	2.667
UTOZEK	173.407	3.315	UTOZEK	172.339	3.372	UTONIB	169.511	3.232	UTOQEB	152.299	3.176	UTOQEB	152.797	3.122
UTURAF0	153.572	3.375	UTUREJ	162.48	3.109	UTUNIJ	170.375	3.462	UTUQIM	159.269	3.159	UTUQUY	163.915	3.188
UVAKIO	161.719	3.131	UVAKIO	154.211	3.071	UTUXAK	174.399	3.48	UTUXAK	170.074	2.997	UTUXAK	175.451	2.756
UVALUA	170.805	3.396	UVALUA	153.35	2.829	UVALOU	172.803	2.978	UVALOU	176.229	2.836	UVALOU	177.959	3.487
UVAWAS	170.993	3.199	UVEDUX	161.204	3.033	UVALUA	175.231	3.457	UVALUA	161.279	3.04	UVALUA	156.792	3.162
UVEFAF	168.048	3.106	UVEFAF	162.812	2.888	UVEDUX	158.075	3.317	UVEDUX	151.627	2.932	UVEFAF	156.42	3.48
UVEFEJ	169.984	3.216	UVEHUB	153.567	3.131	UVEFEJ	151.155	3.287	UVEFEJ	170.721	3.117	UVEFEJ	165.872	3.189
UVEJUD	165.107	2.835	UVEPOD	154.027	3.242	UVEHUB	150.28	3.165	UVEHUB	170.062	3.491	UVEJUD	159.279	2.705
UVIMES	164.462	3.345	UVIMES	152.793	2.973	UVEWUQ	150.56	3.248	UVIMAO	160.495	2.905	UVIMES	159.004	3.217
UVUCOG	163.153	2.895	UVUCOG	175.236	2.812	UVOLOJ	155.114	3.147	UVOLOJ	152.424	3.193	UVUCOG	151.84	3.199
UVUCUM	170.966	3.196	UVUCUM	167.829	2.767	UVUCOG	154.544	3.416	UVUCOG	150.528	3.266	UVUCOG	153.556	2.809
UVUDAT	159.081	3.212	UVUDAT	151.694	2.899	UVUCUM	162.279	2.921	UVUCUM	151.082	2.626	UVUCUM	160.534	2.626
UVUDEX	151.806	2.792	UVUDEX	157.802	3.066	UVUDAT	150.479	3.068	UVUDAT	154.214	3.078	UVUDEX	163.525	2.888
UVUKOM	167.022	3.225	UVUKOM	167.489	3.018	UVUDEX	169.697	2.923	UVUDEX	156.424	3.486	UVUKOM	170.888	2.852
UVUMOO	174.551	3.341	UVUMUU	153.665	2.772	UVUMEG	165.861	3.342	UVUMII	172.058	3.037	UVUMOO	159.763	3.09
UVURIP	158.213	3.152	UVURIP	163.111	3.123	UVUQAG	158.933	3.4	UVUQAG	159.619	3.057	UVURIP	170.123	3.244
UVUYAM	155.987	2.823	UWAFII	170.504	3.293	UVURIP	162.359	3.402	UVURIP	154.801	3.467	UVUYAM	164.527	2.788
UWANAI	164.405	2.892	UWANAI	159.876	2.83	UWAFII	157.12	3.285	UWAFII	155.243	3.057	UWAKEL	159.305	2.949
UWANEM	151.39	3.172	UWANEM	153.941	3.051	UWANEM	160.512	3.021	UWANEM	166.973	3.169	UWANEM	166.861	3.489
UWETIA	152.653	3.07	UWEYED(	174.154	3.12	UWAQIT0	168.252	2.832	UWETIA	173.011	2.974	UWETIA	163.92	3.143
UWIBIM	150.806	3.328	UWIBIM	157.586	2.868	UWEZAY	161.24	3.354	UWEZAY	173.68	2.913	UWEZII	150.463	3.361
UWIBIN	153.803	2.892	UWIBIN	158.878	3.198	UWIBIM	166.977	2.875	UWIBIM	160.863	3.259	UWIBIN	166.351	2.588
UWIBIN	158.103	2.792	UWIBIN	159.292	2.73	UWIBIN	167.822	3.379	UWIBIN	164.315	3.135	UWIBIN	151.5	3.177
UWIBUY	168.32	3.428	UWIBUY	156.16	3.341	UWIBIN	151.131	3.104	UWIBIN	164.126	2.944	UWIBUY	159.592	3.054
UWICOT	157.764	3.01	UWICOT	157.272	2.772	UWIBUY	168.984	3.47	UWIBUY	159.233	2.959	UWIBUY	152.747	2.869
UWIDAG	174.53	3.233	UWIDAG	176.928	3.292	UWICOT	154.066	2.69	UWICOT	154.987	3.449	UWICOT	154.777	3.113
UWISEA	150.508	3.295	UWISEA	151.956	3.319	UWIDAG	156.383	2.997	UWIHIT	156.275	3.358	UWILUK	151.641	2.916
UWISEA	150.19	3.218	UWISEA	153.412	2.898	UWISEA	152.927	3.315	UWISEA	154.463	3.036	UWISEA	153.337	2.892
UWISIE	163.391	3.172	UWISIE	150.492	3.188	UWISEA	154.483	3.271	UWISEA	158.186	3.033	UWISIE	164.487	2.725
UWISIE	152.751	2.725	UWISIE	152.954	3.385	UWISIE	152.864	3.444	UWISIE	159.221	2.905	UWISIE	157.419	3.413
UWISIE	155.422	3.433	UWISOK	163.514	3.246	UWISIE	150.469	2.978	UWISIE	159.401	3.341	UWISIE	161.503	3.324
UWISOK	163.652	3.085	UWITIF	170.126	2.927	UWISOK	156.812	2.923	UWISOK	158.003	3.155	UWISOK	162.833	3.115
UWITIF	151.31	2.863	UWITIF	159.787	2.852	UWITIF	155.591	3.43	UWITIF	161.86	3.17	UWITIF	161.038	3.051
UWITIF	169.313	2.892	UWITIF	169.014	3.044	UWITIF	165.502	2.717	UWITIF	164.145	3.386	UWITIF	173.23	3.114
UWITIF	161.419	2.778	UWITIF	176.202	3.311	UWITIF	157.285	2.708	UWITIF	156.408	3.088	UWITIF	151.449	3.05
UWOFEU	153.598	3.427	UWOHIY	152.98	2.971	UWITIF	170.793	3.076	UWITIF	153.145	3.165	UWITIF	153.105	3.103
UWOHIY0	152.69	2.914	UWOHIY0	150.741	3.003	UWOHIY	151.861	3.046	UWOHIY	150.569	2.943	UWOHIY0	150.448	2.9
UWOHIZ	157.976	3.201	UWOHIZ	153.995	3.029	UWOHIY0	150.415	2.9	UWOHIY0	152.752	2.915	UWOHIY0	150.728	3.004
UWOHOF	155.086	3.025	UWOHOF	159.139	3.199	UWOHIZ	158.281	3.385	UWOHIZ	160.5	3.243	UWOHOE	169.564	3.154
UWOJUM	153.663	3.278	UWOKAT	155.455	3.008	UWOHOF	158.105	2.926	UWOHOF	158.943	2.7	UWOJUM	154.475	2.992
UWONUR	159.121	3.377	UWOPOO	169.367	3.305	UWOKAT	170.234	3.402	UWOKAT	155.619	3.38	UWONIF	159.648	3.378

UWUFUP	159.099	3	UWURIQ	171.222	3.411	UWUCEX(	164.77	3.306	UWUFUP	156.643	3.082	UWUFUP	159.023	3.497
UWURIQ	166.059	3.395	UXABEB	152.349	3.33	UWURIQ	155.31	3.397	UWURIQ	174.873	3.183	UWURIQ	173.857	3.273
UXAQIV	169.419	2.956	UXAQIV	160.566	2.742	UXABIF	160.696	3.118	UXABIF	164.628	3.118	UXAQIV	162.182	2.956
UXATAR	151.989	3.049	UXATUK	161.909	3.097	UXAQIV	158.47	3.305	UXAQUI	154.777	3.284	UXASEU	172.046	3.05
UXEBOQ	163.572	2.965	UXEFIO	160.386	3.152	UXAXEX	154.404	3.346	UXAXEX	151.692	3.158	UXEBOQ	158.99	2.908
UXEFUB	154.726	3.002	UXEFUB	160.72	3.085	UXEFIP	157.347	3.385	UXEFIP	157.543	3.327	UXEFIP	161.7	3.17
UXENOD	151.169	2.932	UXENOD	163.734	2.874	UXEMAO	158.197	3.062	UXEMAO	158.016	3.485	UXENES0	150.702	3.108
UXENOD	170.959	2.842	UXENOD	153.248	3.038	UXENOD	156.139	3.038	UXENOD	153.947	3.232	UXENOD	156.423	2.864
UXIBOV	168.265	2.957	UXIBOV	161.376	3.436	UXEXOM	165.013	3.471	UXEXOM	166.537	2.742	UXIBOV	159.692	2.794
UXIHIV	163.378	3.233	UXIHOB	151.125	3.334	UXIHER	164.614	3.166	UXIHER	162.238	3.486	UXIHIV	150.581	3.172
UXIHUH	156.421	3.223	UXIHUH	155.57	2.951	UXIHOB	156.494	3.455	UXIHOB	163.285	3.453	UXIHOB	150.579	3.345
UXIJET	172.104	3.091	UXIJET	168.196	2.85	UXIJET	155.259	2.839	UXIJET	164.64	3.499	UXIJET	151.173	3.005
UXIJUI	164.304	2.871	UXIJUI	160.717	3.079	UXIJIW	165.147	2.842	UXIJOD	154.915	3.141	UXIJUI	166.427	2.817
UXIPOJ	155.749	3.406	UXIPOJ	167.543	3.406	UXIPEZ	168.952	3.322	UXIPEZ	164.057	3.194	UXIPOJ	150.418	3.228
UXIQIE	166.354	3.484	UXIQIE	168.194	3.201	UXIPOJ	152.114	3.231	UXIPOJ	150.938	3.486	UXIQIE	161.72	3.054
UXIQIE	151.556	3.284	UXIQIE	161.431	3.406	UXIQIE	165.478	3.012	UXIQIE	167.487	3.406	UXIQIE	153.909	3.389
UXIWEG	152.718	3.166	UXIWEG	160.809	2.646	UXIWEG	163.575	2.826	UXIWEG	157.676	3.138	UXIWEG	151.306	2.826
UXIWEG	170.687	3.138	UXIXIJ01	150.178	3.442	UXIWEG	162.585	3.382	UXIWEG	158.063	2.975	UXIWEG	169.048	2.642
UXOBUH	174.434	3.189	UXOCES	152.371	3.106	UXIXIJ01	167.942	3.285	UXIXIJ01	157.628	3.283	UXIXIJ01	161.419	3.134
UXOCOC	168.629	3.356	UXOCOC	151.289	2.845	UXOCES	165.902	2.863	UXOCES	165.406	3.357	UXOCIW	156.384	3.263
UXOHUM(	162.219	3.155	UXOKOK	156.988	3.467	UXOCOC	168.757	2.667	UXOHUM(	165.364	3.155	UXOHUM(	158.683	2.908
UXOKOK	162.72	3.294	UXOKOK	152.75	2.867	UXOKOK	156.769	3.27	UXOKOK	159.054	2.867	UXOKOK	158.842	3.318
UXOKOK	171.293	2.696	UXOKOK	152.777	3.351	UXOKOK	171.322	3.034	UXOKOK	151.994	3.253	UXOKOK	153.612	2.881
UXOKOK	164.431	2.853	UXOKOK	158.214	2.998	UXOKOK	157.981	2.943	UXOKOK	155.856	3.469	UXOKOK	161.358	3.416
UXOQOP	156.36	2.786	UXOQOP	163.115	3.243	UXOKOK	153.065	3.302	UXOQII	152.727	3.431	UXOQOO	152.648	3.446
UXUWAM	162.75	2.959	UXUWIU	171.425	3.333	UXOVIO	154.753	3.195	UXUJII	151.262	3.134	UXUJUU	154.589	3.071
UXUZUJ	158.177	3.216	UYABIG	156.404	2.795	UXUWIU	170.928	3.333	UXUZUJ	161.431	2.782	UXUZUJ	158.834	2.804
UYABOM	172.903	2.926	UYACAZ	155.546	3.263	UYABIG	164.38	2.876	UYABIG	166.029	2.795	UYABIG	158.85	2.895
UYANUG	178.786	3.454	UYAPIV02	165.823	3.471	UYAKEM	166.72	3.204	UYAKEM	158.525	3.465	UYAKEM	158.604	3.204
UYEHAI	159.133	2.715	UYEHAI	159.476	3.411	UYAPIV02	170.305	3.467	UYAZIG	154.597	3.277	UYEHAI	153.441	2.89
UYEHAI	165.32	3.439	UYEHEM	151.237	2.894	UYEHAI	162.95	3.36	UYEHAI	157.976	3.379	UYEHAI	175.918	3.077
UYEHEM	155.695	2.957	UYEHEM	168.923	2.923	UYEHEM	170.245	2.967	UYEHEM	168.503	2.863	UYEHEM	164.852	2.858
UYEMET	160.746	3.158	UYEMET	168.232	3.289	UYEHEM	162.106	3.412	UYEHEM	171.214	2.907	UYEHEM	165.613	2.817
UYEYII02	150.703	3.16	UYEYOO0	150.796	2.993	UYEYII02	165.948	3.306	UYEYII02	157.107	3.03	UYEYII02	158.023	3.043
UYEYOO0	159.841	3.116	UYEYOO0	158.077	3.116	UYEYOO0	158.43	2.924	UYEYOO0	159.121	3.337	UYEYOO0	150.427	2.834
UYEYUU0	151.55	3.205	UYEYUU0	159.655	3.335	UYEYUU0	175.414	3.279	UYEYUU0	170.546	2.866	UYEYUU0	152.599	3.493
UYIGEQ	165.466	3.498	UYIGEQ	167.711	3.38	UYEYUU0	172.797	3.034	UYEZEG	156.548	3.447	UYIGEQ	156.455	2.95
UYIPUO	163.883	3.479	UYIQID	151.804	2.992	UYIGEQ	168.956	3.338	UYIGEQ	161.904	2.912	UYIPOI	160.246	3.345
UYISAZ	178.133	2.859	UYOCIV	162.994	3.055	UYISAZ	177.1	2.778	UYISAZ	156.329	2.778	UYISAZ	153.091	3.304
UYOQOQ	161.947	2.797	UYOQOQ	153.012	2.992	UYOPOO	156.978	3.379	UYOQOQ	163.723	3.138	UYOQOQ	166.788	3.14
UYOWUB	164.678	3.054	UYOZEO	167.102	2.889	UYOWUB	170.535	2.897	UYOWUB	160.28	3.309	UYOWUB	150.828	3.427
UYUHOO	176.258	3.057	UYUREN	151.649	3.233	UYUGEB	172.067	3.259	UYUGEB	172.127	3.086	UYUHOO	166.466	2.916
UZADOQ	153.43	3	UZADOQ	177.731	3.378	UYUREN	162.433	3.016	UYUREN	166.423	3.233	UYUREN	164.927	2.958
UZEGUE	155.062	3.275	UZEGUE	175.037	3.173	UZADOQ	158.861	2.699	UZAFOT	169.03	3.271	UZAFUZ	168.778	3.302
UZEJAM	171.82	2.991	UZEJAM	162.699	3.033	UZEGUE	159.151	3.418	UZEJAM	175.75	3.253	UZEJAM	165.936	2.858
UZIBAI	162.754	3.42	UZIBAI	155.464	3.177	UZEPIB	155.093	3.236	UZETID	159.89	2.947	UZETID	151.286	2.739
UZIFUH	163.64	2.807	UZIXUY	152.825	3.218	UZIFUH	171.247	2.807	UZIFUH	170.486	2.987	UZIFUH	159.235	3.347
UZOMUS	151.602	3.188	UZOMUS	151.204	3.255	UZIYAG	150.986	2.84	UZIYUA	154.75	2.867	UZOMUS	150.756	3.2
UZUHOP	153.119	3.461	UZUJUX	167.671	3.483	UZOMUS	155.455	3.301	UZUGUU	165.543	2.917	UZUGUU	162.527	2.944
UZULAF	163.18	2.773	UZULAF	150.641	3.147	UZUJUX	155.458	3.301	UZUJUX	151.8	3.407	UZULAF	165.261	2.779
UZUQOW	153.372	3.172	UZUQOW	158.395	3.172	UZULAF	168.242	2.801	UZULAF	151.402	2.908	UZULAF	154.292	3.01
UZUVAP	165.618	2.968	UZUZUM	152.564	2.969	UZUQUC	155.067	3.209	UZUQUC	159.953	3.209	UZUTOB	160.782	3.442
VABNUJ	163.906	2.981	VABPIC	151.47	3.343	VABFIQ	163.242	3.103	VABMIV	175.852	3.346	VABMIV	173.128	3.478
VABWIG	170.079	2.78	VABWUS	155.837	3.014	VABPIC	177.496	3.379	VABPIC	154.55	3.122	VABWAB	162.296	3.208
VABYUT	161.662	2.906	VACFUA	156.294	2.799	VABWUS	155.634	2.972	VABYED	158.376	3.311	VABYUT	169.457	2.906
VACMIY	162.237	2.736	VACMIY01	163.664	2.781	VACFUA	165.421	2.799	VACMAQ	155.412	2.82	VACMAQ0	155.105	2.836
VACZEG	170.644	3.033	VACZIL	152.543	3.423	VACSUQ	153.48	3.278	VACSUQ	152.475	2.942	VACSUR	164.696	3.382
VADGEP	155.3	3.16	VADGEP	159.652	3.099	VACZIN	162.569	3.258	VACZOQ	160.9	3.429	VADGEP	172.356	2.935
VADQIC	159.11	3.259	VADVOL	160.753	3.323	VADNOH	152.487	3.191	VADPOJ	152.959	3.062	VADQIC	151.187	3.225
VADYUU	164.832	3.494	VAFBAH	164.123	3.02	VADVOL	171.152	3.231	VADVOL	155.051	3.314	VADYUU	169.726	3.496
VAFFUF	158.691	3.048	VAFGEQ	159.62	3.004	VAFFEP	166.909	3.032	VAFFEP	151.773	2.913	VAFFUF	153.127	2.927
VAFXOP	159.241	3.406	VAGDUE	167.451	3.03	VAFGUG	152.899	2.979	VAFHAM	174.332	2.893	VAFNIZ01	164.197	3.177
VAGQIG	152.405	2.889	VAGQIG	154.422	2.965	VAGFAM	153.742	3.096	VAGFAM	175.099	2.747	VAGFOA	170.177	3.07
VAGQIG	159.6	2.889	VAGQIG	167.342	3.498	VAGQIG	163.756	3.028	VAGQIG	169.738	3.275	VAGQIG	171.073	3.148
VAGQUS	155.553	2.792	VAGQUS	153.895	3.128	VAGQIG	151.737	3.348	VAGQUS	157.905	2.777	VAGQUS	173.655	3.281
VAGQUS	155.15	2.777	VAGQUS	167.422	3.128	VAGQUS	164.374	3.411	VAGQUS	160.647	2.721	VAGQUS	155.011	2.792
VAHMEY	160.292	2.93	VAHPAY	151.169	3.094	VAGQUS	160.496	3.418	VAGQUS	159.284	3.351	VAGQUS	155.094	3.004
VAHZIP	154.277	3.136	VAHZIP	150.758	3.237	VAHZEL	165.955	3.282	VAHZEL	167.824	3.134	VAHZIP	155.398	3.207
VAJCAK01	160.672	3.009	VAJCAK01	166.717	3.293	VAJBIR	164.243	3.063	VAJBIR	170.592	3.147	VAJBIR	153.203	3.455
VAJXEL	165.719	2.827	VAJXIP	159.033	2.699	VAJLIC	155.245	2.786	VAJLIC	163.724	3.103	VAJNUT	163.045	3.485
VAJYIQ	154.116	2.773	VAKHIZ01	171.826	3.115	VAJXOV	162.551	2.812	VAJXUB	167.252	2.837	VAJYIQ	172.04	2.746
VAKWIQ	160.626	2.679	VAKWIQ	164.568	2.686	VAKHIZ01	158.438	3.07	VAKQIH	172.422	2.964	VAKWIQ	153.805	2.751
VAKXEN	151.78	3.073	VALDIZ	166.171	2.97	VAKWIQ	153.752	3.209	VAKXEN	168.863	3.093	VAKXEN	157.432	3.073
VALDIZ	162.038	2.903	VALDIZ	170.158	3.031	VALDIZ	164.37	3.048	VALDIZ	160.892	3.03	VALDIZ	158.257	3.03
VALDIZ01	158.094	3.079	VALDIZ01	161.091	2.954	VALDIZ01	165.684	3.026	VALDIZ01	160.496	3.079	VALDIZ01	162.552	3.097
VALDIZ02	163.695	3.046	VALDIZ02	158.479	3.021	VALDIZ01	168.701	3.076	VALDIZ02	166.207	2.969	VALDIZ02	160.905	3.021
VALDIZ03	160.173	3.096	VALDIZ03	162.825	3.128	VALDIZ02	162.166	2.904	VALDIZ02	170.571	3.034	VALDIZ03	165.241	3.048
VALJOL	156.385	3.432	VALJOL	171.229	3.164	VALDIZ03	167.632	3.091	VALDIZ03	158.776	3.096	VALDIZ03	161.198	2.971
VALVAG	170.649	3.33	VALVAG	168.77	3.382	VALTOV	167.56	3.237	VALTUB	175.642	2.908	VALTUB	150.254	2.908

VAMHAV	154.171	2.738	VAMLEA	165.132	3.24	VALVAG	169.42	3.036	VALWOU	171.824	2.893	VAMHAV	154.18	2.738
VAMQAD	157.386	2.895	VAMTOS	172.759	2.881	VAMLEA10	165.132	3.24	VAMNUU	162.968	3.219	VAMNUV	165.472	3.268
VANBOF	163.41	2.833	VANBOF	152.29	3.069	VAMTOS	168.737	3.14	VAMXEQ	172.122	3.244	VAMXIU	154.311	3.061
VANDEW	164.002	2.91	VANDEW	156.122	2.944	VANDAS	167.571	3.029	VANDAS	150.563	2.848	VANDAS	158.302	3.495
VANWUC	165.335	3.377	VANWUC	164.33	3.108	VANHEZ	166.876	3.463	VANQAG	161.128	3.027	VANWUC	150.31	3.172
VAPMIM	174.714	3.25	VAPPOU	170.433	3.139	VAPMIM	168.888	3.267	VAPMIM	150.124	3.428	VAPMIM	163.573	3.136
VAPREM	155.828	3.422	VAPREM	153.421	3.017	VAPQUB	170.251	3.183	VAPRAI	160.538	3.072	VAPRAI	159.533	3.136
VAPYUK	159.507	3.168	VAQDEA	159.314	2.897	VAPREM	176.774	3.059	VAPREM	159.461	3.463	VAPREM	160.762	3.055
VAQDUM0	151.91	3.181	VAQDUM0	157.102	3.389	VAQDEA	150.308	3.072	VAQDUM	151.605	3.159	VAQDUM	155.407	3.376
VAQSOX	153.515	3.314	VAQSOX	160.256	3.267	VAQSOX	159.079	3.112	VAQSOX	159.87	3.267	VAQSOX	156.828	3.029
VAQSOX0	153.066	2.934	VAQSOX0	162.762	2.93	VAQSOX	154.022	3.112	VAQSOX	153.801	3.163	VAQSOX0	162.455	2.93
VAQWIV	166.072	3.429	VAQWIV	150.632	2.985	VAQSOX0	164.961	2.934	VAQVIV	161.792	3.387	VAQWER	168.948	3.03
VAQXAO	151.83	2.88	VAQXAO	159.32	2.88	VAQWUH	150.8	3.141	VAQXAO	168.125	3.122	VAQXAO	178.352	3.24
VAQXUI	172.54	3.155	VAQYAP	161.824	2.962	VAQXIW	165.395	2.987	VAQXIW	153.547	3.03	VAQXIW	158.343	3.03
VAQYIX	170.896	3.441	VAQYIX	152.047	3.186	VAQYET	151.649	2.819	VAQYET	160.846	3.12	VAQYET	165.421	2.819
VAQYUJ	158.245	3.346	VAQYUJ	156.116	3.362	VAQYIX	179.596	3.091	VAQYIX	154.66	3.096	VAQYUJ	158.143	3.208
VAQYUJ01	157.796	3.332	VAQYUJ01	157.73	3.199	VAQYUJ	164.486	3.242	VAQYUJ	157.759	3.015	VAQYUJ	157.435	3.006
VAQYUJ01	155.867	3.345	VAQZAQ	172.85	3.166	VAQYUJ01	157.95	2.989	VAQYUJ01	157.896	2.983	VAQYUJ01	164.102	3.241
VARJAC	152.525	2.985	VARJAC	174.135	3.249	VAQZIY	151.317	3.344	VARJAC	169.235	2.644	VARJAC	175.302	2.655
VARNIN	156.659	3.263	VASCOI	174.304	3.085	VARJAC	171.538	3.261	VARJAC	174.025	3.441	VARNIN	161.531	2.83
VASHEF	173.79	2.802	VASHEF	150.127	3.385	VASCOI	161.543	3.057	VASGOL	155.193	3.014	VASGUR	159.764	3.011
VASQIR	171.317	2.722	VASTIT	153.383	3.443	VASKUX	163.155	2.956	VASKUX	156.227	2.956	VASLAE	164.849	3.283
VATNOU	158.919	2.812	VATYOG	154.457	3.191	VASYUM	158.158	3.427	VATGOO	173.472	3.373	VATNOU	156.001	3.159
VAVBAZ	155.13	2.815	VAVBAZ	157.398	2.928	VATYOG	153.758	3.405	VATZOJ	165.257	2.759	VAVBAZ	179.262	2.959
VAVBED	151.447	3.217	VAVBIH	166.158	3.023	VAVBAZ	158.511	3.25	VAVBED	156.095	2.908	VAVBED	156.854	3.147
VAVFEG	166.534	3.413	VAVFEG	162.693	3.109	VAVFEG	153.932	3.44	VAVFEG	158.148	3.441	VAVFEG	168.623	3.415
VAVFOQ	151.113	2.858	VAVFOQ	154.378	3.154	VAVFEG	160.38	2.931	VAVFEG	170.801	3.016	VAVFOQ	164.017	2.776
VAVFOQ	153.904	3.076	VAVFOQ	163.915	3.038	VAVFOQ	153.891	3.233	VAVFOQ	156.498	3.188	VAVFOQ	156.115	3.172
VAVFUW	155.041	3.325	VAVFUW	162.858	3.427	VAVFOQ	161.864	2.775	VAVFUW	160.422	3.465	VAVFUW	164.489	3.386
VAVFUW	159.826	3.162	VAVFUW	151.755	3.116	VAVFUW	161.807	3.138	VAVFUW	168.632	3.427	VAVFUW	177.838	3.465
VAVGEH	176.548	3.008	VAVGEH	165.551	2.99	VAVGEH	164.528	3.288	VAVGEH	154.901	3.176	VAVGEH	163.857	2.865
VAVJIM	162.038	3.061	VAVJIM	177.919	2.773	VAVGEH	173.034	2.777	VAVGEH	153.054	3.395	VAVJIM	153.467	3.485
VAVJIM	165.001	3.194	VAVJOS	177.059	3.423	VAVJIM	150.53	2.791	VAVJIM	157.836	3.352	VAVJIM	176.594	3.422
VAVJOS	158.809	2.727	VAVJUY	150.016	2.948	VAVJOS	161.927	3.16	VAVJOS	154.932	3.378	VAVJOS	170.772	2.804
VAVJUY	155.568	3.001	VAVJUY	155.326	3.444	VAVJUY	156.702	3.218	VAVJUY	155.32	2.96	VAVJUY	175.797	2.972
VAVJUY	158.006	2.93	VAVKEI	165.117	3.247	VAVJUY	150.621	2.925	VAVJUY	166.328	2.745	VAVJUY	172.735	3.384
VAVKIN	156.308	3.145	VAVKIN	167.948	3.398	VAVKIN	158.097	3.068	VAVKIN	170.291	3.262	VAVKIN	153.041	3.312
VAWBEB	177.633	3.122	VAWBEB	179.084	3.122	VAVNEM	151.645	2.858	VAVNIS	169.497	3.275	VAVNIS01	171.716	3.331
VAWCEB	161.247	3.248	VAWCEB	153.265	3.456	VAWBOL	173.711	3.318	VAWBOL	167.774	3.318	VAWCEB	155.003	3.453
VAWMIT	166.556	3.434	VAWMIT	159.813	3.197	VAWCEE	170.581	3.341	VAWCEE	158.872	3.079	VAWMIT	151.868	3.405
VAWTEW	161.174	3.355	VAWTEW	152.896	3.301	VAWTEW	166.189	3.499	VAWTEW	156.456	3.361	VAWTEW	166.445	3.442
VAWTEW	150.269	3.216	VAWTEW	156.784	3.268	VAWTEW	158.794	3.104	VAWTEW	154.468	3.273	VAWTEW	150.963	3.371
VAWWUL	162.509	3.252	VAWWUL	166.999	3.478	VAWWOJ	162.844	3.323	VAWWOJ	169.166	2.985	VAWWOJ	162.744	2.861
VAWWUL	151.197	3.013	VAWWUL	153.105	3.217	VAWWUL	160.577	3.26	VAWWUL	154.721	3.167	VAWWUL	159.526	2.916
VAXHAG	166.092	3.027	VAXHAG	154.898	3.494	VAXGUZ	150.248	3.276	VAXGUZ	158.576	3.024	VAXGUZ	152.119	3.305
VAXROE	155.23	2.959	VAXROE	150.78	3.25	VAXKOY	162.989	3.229	VAXKUE	162.813	3.228	VAXLEP	160.535	3.202
VAXROE	154.432	2.729	VAXROE	151.754	2.962	VAXROE	173.872	3.03	VAXROE	150.101	3.308	VAXROE	151.226	2.717
VAXRUK	165.749	3.201	VAXRUK	166.809	2.701	VAXROE	152.307	2.82	VAXRUK	160.425	2.928	VAXRUK	170.91	2.77
VAXSAR	155.515	3.315	VAXSAR	152.756	3.488	VAXRUK	178.337	2.701	VAXRUK	158.834	3.376	VAXSAR	159.998	2.897
VAXSEV	163.208	3.328	VAXSEV	150.606	3.128	VAXSAR	152.155	3.305	VAXSEV	158.178	2.92	VAXSEV	151.115	3.325
VAYBOM	155.247	3.096	VAYBOM	158.32	2.91	VAXSEV	152.556	2.925	VAXTIA	156.458	2.891	VAXVOG	172.987	3.173
VAYHIN	163.163	3.32	VAYHIN	163.712	3.423	VAYBOM	159.159	3.23	VAYFEJ	173.157	2.798	VAYHIN	158.192	3.356
VAYHIN	162.273	3.054	VAYHUZ	154.11	2.917	VAYHIN	164.465	2.721	VAYHIN	158.951	2.932	VAYHIN	157.825	3.097
VAYHUZ01	157.24	3.065	VAYHUZ01	152.887	2.87	VAYHUZ	157.521	3.074	VAYHUZ	152.833	2.878	VAYHUZ01	154.278	2.907
VAYJOU	151.947	3.288	VAYJOU	158.876	2.956	VAYJAH	153.093	3.231	VAYJOU	160.587	3.203	VAYJOU	155.558	2.942
VAYJOU	164.943	3.313	VAYVIA	161.862	2.912	VAYJOU	151.022	3.173	VAYJOU	150.368	3.463	VAYJOU	155.122	3.052
VAYYID	160.942	2.974	VAZNUG	171.836	3.492	VAYVIA	167.386	3.257	VAYVIA	163.22	2.981	VAYYID	153.564	3.182
VEBGOA	160.751	2.797	VEBHAN	155.516	2.93	VAZPER	150.332	3.44	VAZPER10	150.332	3.44	VAZVOH	173.234	3.25
VEBHER	164.868	2.767	VEBKAO	153.885	3.394	VEBHER	166.587	2.802	VEBHER	171.527	2.752	VEBHER	176.094	2.77
VEBKAO	153.391	2.742	VEBKAO	173.506	2.975	VEBKAO	166.697	3.001	VEBKAO	163.762	2.742	VEBKAO	161.081	2.975
VEBTAB	164.401	3.176	VECDEM	155.996	3.388	VEBLOG	161.5	3.474	VEBTAB	162.785	3.057	VEBTAB	159.083	3.142
VECJEU	161.573	3.036	VECJOE	154.781	3.213	VECDEM	169.227	3.388	VECJEU	172.862	3.29	VECJEU	157.698	3.198
VECJOE	153.046	2.975	VECJOE	155.171	3.008	VECJOE	153.125	3.079	VECJOE	151.492	3.284	VECJOE	153.505	3.246
VECKEU	157.087	3.266	VECKIZ	155.133	3.257	VECJOE	160.319	2.997	VECJOE	150.611	3.043	VECKEU	151.264	3.179
VECKIZ	156.637	3.108	VECKOE	151.936	3.017	VECKIZ	153.501	2.968	VECKIZ	164.043	3.241	VECKIZ	161.759	2.968
VECMEV	159.068	3.366	VECMEV	164.276	2.906	VECKOE	165.994	2.72	VECKOE	165.652	2.729	VECKOE	151.837	3.179
VEDKEU	159.01	3.473	VEDKEU	170.831	3.17	VECMEV	161.68	3.363	VECTIH	164.909	3.368	VEDCEO	160.814	3.022
VEFKEZ	167.083	3.436	VEFKOI	157.706	3.072	VEDVAC	154.115	2.836	VEFJOI	161.276	3.248	VEFKEZ	158.19	3.231
VEFSOQ	165.48	3.091	VEFTIM	175.321	3.231	VEFKOI	166.784	3.131	VEFSOQ	167.941	2.925	VEFSOQ	152.304	3.372
VEGCIX	164.2	3.367	VEGCIX	174.527	3.208	VEFXOX	164.411	2.847	VEFYIS	174.32	3.021	VEGCIW	176.077	3.142
VEGGUL	163.673	2.929	VEGKEA	154.656	3.193	VEGCIX	166.071	3.367	VEGGUL	159.596	2.929	VEGGUL	173.127	3.011
VEGTOS	162.674	2.832	VEHCET	150.386	3.288	VEGKEX	151.836	3.171	VEGKEX	156.127	3.115	VEGTOS	172.698	2.832
VEHWUA	163.254	3.197	VEHWUA	166.583	3.197	VEHCET	158.64	3.211	VEHWUA	157.36	2.709	VEHWUA	165.442	3.215
VEJJUP	172.576	3.177	VEJJUP	166.776	3.023	VEJJUP	158.844	3.188	VEJJUP	154.98	3.023	VEJJUP	170.554	3.096
VEJWEN	159.84	2.763	VEJWEN	155.359	3.269	VEJKOO	170.133	3.279	VEJKUU	170.152	3.277	VEJRUY	155.518	3.154
VEJWUC	158.356	2.618	VEJZIV	173.328	3.211	VEJWUC	164.854	3.415	VEJWUC	159.107	3.054	VEJWUC	176.691	3.072
VEKMOO	162.84	2.845	VEKMOO0	162.853	2.844	VEJZIV	155.232	3.365	VEKBAS	163.362	3.376	VEKBEW	161.651	3.225
VEKZOB	167.86	2.917	VEKZOB	156.142	2.91	VEKNOR	152.789	3.251	VEKZOB	169.163	2.897	VEKZOB	154.217	2.889
VEKZUH	156.775	2.906	VELKAB	168.608	2.906	VEKZUH	166.052	3.052	VEKZUH	152.515	2.906	VEKZUH	165.48	3.069

VELMEH	157.347	2.916	VELMEH	160.278	3.1	VELKUV	159.928	2.766	VELKUV	165.578	3.157	VELKUW	155.236	3.048
VELREM	160.798	3.409	VELROT	168.936	3.236	VELMEH0	152.154	2.773	VELMEH0	174.249	2.965	VELMEH0	161.601	3.129
VELRUZ	168.187	3.249	VELRUZ	169.35	3.031	VELROT	164.492	2.829	VELROT	153.637	2.829	VELROW	157.736	3.053
VELSAG	168.648	2.914	VELSAG	165.286	2.91	VELRUZ	156.199	2.913	VELRUZ	155.348	2.913	VELSAG	166.102	3.333
VEMCAR	167.392	3.063	VEMCAR	152.671	2.764	VELSAG	153.998	2.91	VELYIU	162.831	3.077	VELYIU	168.658	3.415
VEMZUI	167.681	2.946	VENHOL	152.619	3.481	VEMHAA	162.989	3.014	VEMJEF	153.22	3.338	VEMSUF	151.5	3.385
VENPOU	156.746	2.844	VENPOU	159.02	2.827	VENKEF	166.568	2.955	VENNUB	164.657	3.204	VENPOU	159.357	2.821
VENQEL	157.241	2.684	VENQEL	160.854	2.873	VENPOU	158.35	2.823	VENQEL	164.229	2.989	VENQEL	161.778	3.051
VENQEL	154.918	3.075	VENQIP	153.813	3.129	VENQEL	163.199	3.285	VENQEL	160.756	2.999	VENQEL	150.29	3.446
VENQUB	165.825	3.175	VENQUB	152.597	3.466	VENQIP	163.944	3.315	VENQUB	150.158	3.225	VENQUB	162.37	2.786
VENQUB	154.365	3.129	VENQUB	155.662	2.942	VENQUB	164.338	3.299	VENQUB	174.418	2.994	VENQUB	159.973	2.747
VENQUB	154.298	3.42	VENQUB	163.196	2.786	VENQUB	152.768	2.838	VENQUB	166.592	2.951	VENQUB	162.021	3.204
VENWIV	159.486	3.297	VENWIV	150.846	3.436	VENQUB	156.201	3.462	VENQUB	159.978	3.459	VENTOZ	157.207	2.978
VENWUH	164.915	3.222	VENWUH	151.643	3.222	VENWIV	156.429	3.133	VENWOB	159.441	3.319	VENWOB	152.982	3.319
VEPHII	171.931	2.843	VEPHII	153.072	2.843	VEPDIH	174.273	3.095	VEPDIH	167.768	2.953	VEPHII	152.751	2.98
VEPQIR	166.191	2.75	VEPQUF	164.742	3.481	VEPLUB	164.404	3.381	VEPQIR	152.531	3.376	VEPQIR	164.257	2.941
VEPXAQ	161.776	2.69	VEPXAQ	165.495	3.411	VEPQUF	153.022	3.231	VEPTIT	167.156	3.353	VEPWIX	159.918	3.175
VEPXOG	155.755	3.136	VEPXOG	154.678	3.02	VEPXAQ	158.74	3.365	VEPXAQ	153.378	3.399	VEPXOG	163.719	3.127
VEPZUN	151.778	3.441	VEPZUN	160.385	3.353	VEPYUO	165.642	2.818	VEPYUO	163.46	2.936	VEPZUN	154.501	2.99
VEPZUN0	154.657	3.043	VEPZUN0	159.348	3.437	VEPZUN0	154.662	3.002	VEPZUN0	152.582	3.475	VEPZUN0	159.994	3.368
VEQFOO	160.771	3.01	VEQFOO	164.301	3.463	VEQFII	161.09	3.356	VEQFOO	169.982	3.01	VEQFOO	159.422	3.175
VEQFOO	170.101	3.203	VEQFOO0	164.439	2.897	VEQFOO	157.16	3.026	VEQFOO	170.753	3.262	VEQFOO	164.439	2.897
VEQFOO0	160.771	3.01	VEQFOO0	164.301	3.463	VEQFOO0	170.752	3.262	VEQFOO0	169.982	3.01	VEQFOO0	159.423	3.175
VEQJEH	160.332	3.212	VEQJIL	166.277	3.025	VEQFOO0	157.159	3.026	VEQFOO0	170.101	3.203	VEQJEH	158.124	3.333
VEQXAR	154.559	3.495	VEQXEV	176.322	3.245	VEQLIN	151.885	3.242	VEQNAJ	156.607	3.277	VEQQOX	153.533	3.285
VEQYIA01	169.577	3.005	VEQYIA01	168.284	3.464	VEQXEV	157.18	3.249	VEQYIA	150.396	3.032	VEQYIA01	169.419	3.362
VERQIW	169.697	3.359	VERQIW	154.312	3.054	VERLAG	170.396	3.15	VERLIO	168.28	3.122	VERLOU	156.536	3.467
VERQIW	155.1	3.277	VERVUM	167.16	2.666	VERQIW	162.385	3.125	VERQIW	158.847	2.766	VERQIW	167.992	2.996
VESDUS	156.817	3.192	VESDUS	156.133	3.419	VESBAA	152.185	3.173	VESCAA	158.066	3.357	VESCAA	154.742	2.99
VESKIO	161.687	2.868	VESKIO	176.727	3.481	VESDUS	150.437	3.369	VESHOR	158.687	3.214	VESKIO	165.802	3.149
VESKIO	153.384	3.101	VESKIO	157.459	2.965	VESKIO	151.745	2.946	VESKIO	159.827	3.476	VESKIO	163.996	3.484
VESLOU	151.207	2.911	VESLOU	156.375	2.652	VESKIO	150.604	3.149	VESKIO	166.038	2.667	VESKIO	164.862	2.946
VESLOU	177.139	2.769	VESLOU	160.219	2.845	VESLOU	162.96	3.074	VESLOU	153.403	3.088	VESLOU	163.405	2.757
VESNOZ	160.147	3.089	VESPAN	179.944	2.846	VESLOU	155.707	2.964	VESLOU	161.74	2.757	VESLOU	153.932	2.929
VESYAX	162.984	3.382	VESYAX	150.759	3.24	VESPAN	167.573	3.313	VESPAN	153.05	3.45	VESXAU	156.654	3.439
VETBAB	158.427	2.892	VETBAB	177.485	2.892	VESYEZ	165.8	3.249	VESZEA	168.747	3.327	VETBAB	167.045	3.23
VETBEF	160.719	2.779	VETBEF	156.768	2.846	VETBAB	152.421	2.822	VETBAB	155.114	2.935	VETBEF	154.456	2.913
VETHAD	164.326	3.025	VETHAD	151.118	3.027	VETHAD	152.32	2.886	VETHAD	166.667	3.486	VETHAD	174.184	3.025
VETHEH	150.676	2.939	VETHIL	173.902	3.275	VETHAD	156.103	2.816	VETHAD	164.848	3.486	VETHEH	172.986	2.592
VETHIN	176.95	2.939	VETHOR	156.425	3.23	VETHIL	158.011	3.184	VETHIL	162.765	3.036	VETHIN	156.674	3.412
VETHOT	150.127	3.136	VETHOU	152.447	3.376	VETHOR	157.069	3.06	VETHOR	166.063	3.057	VETHOR	160.39	2.944
VETJIQ	150.367	3.254	VETJIQ	171.167	2.932	VETHOU	150.865	3.355	VETHOU	151.539	3.444	VETJIQ	167.469	2.815
VETTUL	155.624	3.467	VETVEX	159.909	3.471	VETJIQ	156.371	3.425	VETJIQ	157.704	3.186	VETTUL	153.623	3.467
VETZID	166.004	2.862	VETZID	152.402	3.02	VETWEX	163.821	2.804	VETWEY	150.485	3.196	VETWEY	157.935	3.323
VEVNIV	160.202	3.183	VEVNIV	166.187	2.842	VETZID10	166.004	2.862	VETZID10	152.402	3.02	VETZUP	165.682	2.918
VEVNUH	167.545	3.223	VEVPIX	169.013	3.296	VEVNIV	172.814	3.302	VEVNUH	158.576	3.044	VEVNUH	150.365	3.061
VEVQAS	166.627	3.093	VEVSUL	165.227	3.035	VEVPIX	175.156	2.791	VEVPIX	157.416	2.661	VEVQAS	159.139	3.093
VEWHAH0	170.163	3.223	VEWHAH0	161.698	2.859	VEWHAH	161.283	3.065	VEWHAH	161.068	2.952	VEWHAH	168.731	3.334
VEWQUN	150.767	2.97	VEWXON	158.875	3.063	VEWHAH0	161.923	2.968	VEWLIU	168.204	3.283	VEWLIU	153.747	2.722
VEXCUZ	153.116	3.355	VEXCUZ	172.168	2.833	VEWXON	174.237	2.986	VEWYEE	177.117	3.445	VEXCUZ	161.666	3.161
VEXFAH	164.147	3.036	VEXFOV	169.771	3.395	VEXFAH	152.395	3.475	VEXFAH	166.863	3.106	VEXFAH	169.88	3.106
VEXFUB	152.883	3.096	VEXFUB	161.246	3.092	VEXFOV	152.912	3.449	VEXFOV	150.019	3.388	VEXFUB	169.016	3.2
VEXFUB	151.729	3.209	VEXGAI	154.594	2.968	VEXFUB	151.935	3.027	VEXFUB	164.493	3.092	VEXFUB	154.604	3.134
VEXGEN	158.011	3.441	VEXGEN	167.366	2.72	VEXGAI	156.733	3.027	VEXGAI	163.018	2.848	VEXGEN	169.972	2.709
VEYNIX	157.207	3.085	VEYSAU	161.708	2.941	VEXNUI01	160.033	3.196	VEYKOC	165.673	2.931	VEYKOC	160.132	2.9
VEYSAU	152.197	2.968	VEYSAU	151.962	2.909	VEYSAU	152.478	2.684	VEYSAU	154.298	3.3	VEYSAU	154.43	2.806
VEYZEI	153.727	3.41	VEZBIN	150.521	3.069	VEYSAU	159.187	3.484	VEYSAU	169.871	3.075	VEYYAC	151.244	3.068
VEZBUZ	151.746	2.764	VEZFUB	156.617	3.096	VEZBIN	165.115	3.069	VEZBIN	172.61	3.025	VEZBUZ	167.394	2.942
VEZGAI	176.847	2.875	VEZGAI	177.241	3.244	VEZFUB	159.48	2.812	VEZFUB	156.818	3.374	VEZFUB	158.793	3.425
VEZKIW	151.641	3.435	VEZNAS	153.345	2.816	VEZGAI	159.549	3.098	VEZKES	154.238	3.478	VEZKIW	171.769	3.048
VEZNAS	170.413	2.688	VEZNEW	156.51	2.906	VEZNAS	156.114	2.816	VEZNAS	153.376	3.286	VEZNAS	170.915	3.441
VEZPIZ11	161.395	3.459	VEZPIZ11	151.696	2.848	VEZNEW	154.694	3.425	VEZNIA	154.412	3.408	VEZNIA	156.993	2.943
VEZQUP	165.81	2.729	VEZQUP	153.571	2.883	VEZPIZ11	153.627	2.696	VEZPIZ11	160.094	2.981	VEZPIZ11	171.671	3.055
VEZTIF	165.596	2.876	VEZTIF	160.61	2.842	VEZTIF	165.099	2.842	VEZTIF	155.337	2.934	VEZTIF	161.523	3.027
VEZTIF	163.331	2.877	VEZTIF	178.633	2.768	VEZTIF	161.428	2.895	VEZTIF	155.493	3.034	VEZTIF	169.953	2.768
VEZTIF	161.675	3.109	VEZTIF	174.731	3.079	VEZTIF	152.272	2.868	VEZTIF	162.014	2.822	VEZTIF	176.694	2.876
VEZXIL	154.421	3.37	VEZXIL	165.881	2.894	VEZTIF	174.214	3.008	VEZTIF	163.813	3.167	VEZXIL	153.929	3.126
VEZXOR	158.335	3.495	VEZXOR	161.053	2.82	VEZXIL	152.941	3.04	VEZXIL	161.582	3.091	VEZXIL	155.27	3.382
VEZXUV	156.235	3.233	VEZXUV	153.494	3.466	VEZXUV	152.562	2.888	VEZXUV	165.125	3.233	VEZXUV	161.124	2.957
VEZZAE	160.018	2.83	VIBQUW	162.989	3.241	VEZYOQ	152.447	3.337	VEZYOQ	160.186	3.256	VEZZAE	167.869	2.83
VICBOA	164.225	3.306	VICKUN	167.906	3.21	VIBRUU	152.772	3.453	VIBRUU	150.47	2.707	VIBRUU	168.444	2.84
VICLAU	153.863	3.088	VICLAU	175.629	2.998	VICKUN	159.206	3.472	VICKUN	153.156	3.24	VICKUN	152.279	3.369
VICVUY	151.421	3.299	VICVUY	151.421	3.15	VICLAU	153.449	3.049	VICSAB	157.492	3.202	VICVUY	163.701	3.457
VICVUY	150.296	3.347	VICVUY	158.797	3.369	VICVUY	151.84	2.869	VICVUY	150.389	3.079	VICVUY	151.254	3.157
VIDBER	151.552	3.275	VIDFIA	165.858	3.21	VICVUY	168.913	3.299	VIDBEP	153.916	3.268	VIDBER	176.15	2.967
VIDFIA	161.946	3.07	VIDFIA	172.678	3.153	VIDFIA	154.121	3.404	VIDFIA	172.575	3.272	VIDFIA	164.748	2.94
VIDFOG	165.998	2.959	VIDFOG	160.628	3.042	VIDFOG	167.294	3.206	VIDFOG	153.884	3.356	VIDFOG	172.628	3.26
VIDHUO	150.927	3.165	VIDHUO	176.467	3.203	VIDFOG	171.432	3.125	VIDHIA	159.295	3.414	VIDHUO	156.669	2.843
VIDMOM	153.84	2.831	VIDMOM	154.38	3.13	VIDHUO01	157.371	2.838	VIDHUO01	150.352	3.155	VIDHUO01	175.891	3.197

VIDMOM	163.825	3.141	VIDMOM	160.714	2.767	VIDMOM	153.368	3.24	VIDMOM	152.918	2.837	VIDMOM	163.176	3.277
VIDMOM	161.315	3.016	VIDMOM	160.32	2.768	VIDMOM	166.32	3.294	VIDMOM	157.497	2.847	VIDMOM	175.668	3.31
VIDQEE	165.615	2.89	VIDQEE	154.216	3.494	VIDMOM	169.456	3.071	VIDPIK	152.081	2.981	VIDQEE	161.789	2.916
VIDQII	166.883	3.277	VIDQII	153.547	2.939	VIDQII	155.196	3.462	VIDQII	158.974	3.143	VIDQII	150.265	3.322
VIDQII	150.303	3.179	VIDQII	150.489	3.013	VIDQII	160.207	2.824	VIDQII	172.196	3.155	VIDQII	166.868	2.978
VIDQII	153.462	3.202	VIDTOR	150.039	3.382	VIDQII	153.473	2.9	VIDQII	168.932	3.306	VIDQII	151.108	3.092
VIDWOY	151.33	3.158	VIDWOY	158.824	2.989	VIDWOY	162.926	3.158	VIDWOY	153.319	2.977	VIDWOY	158.206	2.739
VIDXAL	155.776	2.753	VIDXAL	155.939	2.827	VIDWOY	153.93	3.491	VIDWOY	166.57	2.862	VIDWOY	169.009	3.491
VIDXEP	158.243	3.472	VIDXEP	155.882	2.838	VIDXAL	157.32	2.827	VIDXEN	169.451	3.496	VIDXEN	151.218	3.333
VIFXUH01	167.102	2.784	VIFXUH01	166.522	2.804	VIFXIV	160.229	2.884	VIFXOB	164.266	3.235	VIFXUH	164.269	3.077
VIFYUG	162.197	2.9	VIFYUG	172.447	3.485	VIFXUH03	163.707	3.097	VIFYUG	159.994	3.473	VIFYUG	167.278	3.001
VIFYUG	166.348	2.765	VIFYUG	167.711	3.055	VIFYUG	161.333	3.024	VIFYUG	154.551	3.473	VIFYUG	150.936	3.315
VIFYUG	163.425	3.485	VIFYUG	162.674	2.912	VIFYUG	164.74	3.13	VIFYUG	157.252	3.001	VIFYUG	165.285	2.888
VIFZOC	172.041	3.114	VIFZOC	151.655	3.086	VIFZIW	168.09	3.218	VIFZIW01	168.095	3.222	VIFZOC	152.9	2.887
VIFZOC	169.997	2.964	VIFZOC	150.382	3.367	VIFZOC	152.079	3.143	VIFZOC	160.272	2.887	VIFZOC	151.221	3.476
VIGJAX	172.046	2.885	VIGJAZ	155.357	3.372	VIGDAR01	168.117	3.44	VIGDIZ	163.043	3.053	VIGHOI	150.424	3.191
VIHKUW	150.224	3.435	VIHNEF	150.96	2.959	VIGPOQ	173.488	3.119	VIHDEX	159.62	3.241	VIHHON01	153.327	2.931
VIHNUZ	159.162	3.046	VIHQUZ	155.721	2.972	VIHNOT	172.604	2.914	VIHNOT	158.17	3.15	VIHNUZ	161.817	3.046
VIHRAH	150.482	3.287	VIHRIN	153.711	3.21	VIHQUZ	168.872	3.393	VIHQUZ	167.042	3.449	VIHQUZ	156.964	3.186
VIHZOF	159.803	3.179	VIJQOY	159.498	2.964	VIHRIN	159.368	3.209	VIHZOF	151.184	3.179	VIHZOF	156.555	3.121
VIJYAR	156.71	3.4	VIJYAR	159.967	3.391	VIJTAM	166.039	2.863	VIJYAR	160.839	3.081	VIJYAR	153.482	3.289
VIKMAH	173.763	2.788	VIKMAH	164.917	3.049	VIJYAR	155.899	3.434	VIKMAH	153.264	3.045	VIKMAH	151.534	3.435
VIKMEL	159.041	2.809	VIKMEL	169.58	3.032	VIKMAH	174.742	3.291	VIKMAH	161.699	2.944	VIKMEL	164.131	3.189
VIKMUZ	150.6	3.173	VIKMUZ	150.588	3.036	VIKMEL	169.805	2.755	VIKMEL	155.027	3.039	VIKMUZ	165.087	3
VILGEC	165.237	3.077	VILGUU	156.955	3.115	VIKMUZ	150.916	3.495	VIKPUA	169.109	3.396	VIKSOA	152.879	3.284
VILGUU	155.06	3.344	VILGUU	169.214	2.991	VILGUU	163.139	2.96	VILGUU	155.496	3.144	VILGUU	158.988	2.786
VILWUJ	163.946	3.366	VILWUJ	161.439	3.384	VILGUU	159.048	2.96	VILGUU	162.311	2.991	VILPUF	166.823	3.18
VIMJEH	158.625	3.081	VIMJEH	155.365	2.789	VIMHUV	165.815	3.197	VIMJAD	150.649	2.967	VIMJEH	170.782	3.127
VIMJOR	154.629	3.429	VIMJOR	171.639	3.3	VIMJEH	164.59	2.913	VIMJEH	161.716	2.888	VIMJOR	151.562	3.452
VIMPIU	160.299	2.878	VIMPIU	156.152	2.878	VIMKOV	153.84	3.309	VIMPAM	160.379	3.044	VIMPEQ	165.006	3.267
VIMQIV	150.505	2.863	VIMQIV01	150.536	2.862	VIMQEP	151.458	3.157	VIMQEP	166.911	2.981	VIMQIV	153.389	2.794
VIMZIA	162.057	3.353	VINCOL	161.331	3.249	VIMQIV01	153.382	2.793	VIMTUI	170.078	2.941	VIMZIA	171.269	3.14
VINDAB	159.746	3.241	VINFAD	153.169	2.886	VINCUT	169.289	3.3	VINCUT	156.157	3.142	VINDAB	172.992	2.791
VINPUG	168.019	3.243	VINRUH02	158.689	3.387	VINFEG	173.15	3.48	VINFEG	167.822	2.965	VINJEL	171.849	2.832
VINWIY	166.029	3.078	VINWIY	170.62	2.932	VINSER	150.244	3.217	VINWIY	160.963	3.387	VINWIY	154.234	3.074
VIPDOQ	153.188	3.308	VIPLEO	167.578	3.331	VIPDOQ	165.968	3.477	VIPDOQ	153.683	3.308	VIPDOQ	158.776	3.416
VIPNUD	158.314	3.402	VIPNUD	171.102	3.129	VIPLIS	172.932	2.996	VIPLIS	156.17	3.219	VIPLIS	172.31	2.996
VIPSOF	157.774	3.107	VIPTEV	153.694	3.156	VIPNUF	173.639	3.038	VIPNUF	161.583	3.499	VIPROC	166.82	3.307
VIQGAE	169.253	3.372	VIQGAE	161.124	3.324	VIPZID	155.126	3.005	VIQGAE	153.159	3.209	VIQGAE	161.247	2.892
VIRYOM	159.745	3.081	VISCUZ	162.138	3.352	VIQGAE	168.174	3.094	VIQGAE	154.103	3.448	VIRMOZ	159.474	3.02
VITJUE	151.456	2.795	VITJUE	150.421	2.819	VISCUZ01	161.691	3.437	VISPIY	159.263	3.35	VISPIY	155.152	3.112
VIVJIU	166.312	2.849	VIVJUG	152.948	3.431	VITKAL	156.887	2.772	VITKAL	155.799	2.806	VIVCIQ	157.158	2.665
VIVTOK	158.512	3.286	VIVTOK02	158.527	3.286	VIVKAN	150.237	2.988	VIVKER	163.257	3.319	VIVKER	150.709	3.067
VIWKIY	153.402	3.084	VIWLAS	161.726	3.228	VIVVEC	157.375	3.099	VIVVEC01	157.375	3.099	VIVWUT	155.826	3.143
VIWREA	154.29	3.436	VIWREA	151.675	3.345	VIWLAS	159.14	3.382	VIWLAS	154.473	3.253	VIWLAS	150.019	3.337
VIWREA	155.563	3.389	VIWROK	157	2.852	VIWREA	168.738	3.492	VIWREA	170.954	3.461	VIWREA	164.889	2.792
VIWSAX	154.7	3.429	VIXDAK	162.954	3.083	VIWROK	151.105	3.13	VIWROK	168.491	3.467	VIWRUQ	152.448	3.4
VIXMAR	154.769	3.053	VIXSOL	174.945	3.026	VIXDAK	151.559	3.375	VIXDIP	160.173	3.306	VIXGUG	166.414	3.209
VIXXIJ	153.997	3.43	VIXXOP	152.072	3.374	VIXTAY	161.079	3.266	VIXTIJ	158.314	3.212	VIXWOR	167.665	3.479
VIYDOZ	172.107	2.931	VIYFIV	173.207	2.849	VIYDOZ	160.148	3.082	VIYDOZ	157.053	2.784	VIYDOZ	157.923	3.106
VIYFIV	161.846	3.153	VIYLEX	162.933	2.995	VIYFIV	152.125	3.177	VIYFIV	152.074	3.034	VIYFIV	163.945	2.942
VIYZUB	153.278	3.463	VIYZUB	152.637	2.878	VIYRUR	157.872	3.089	VIYVEH	174.372	3.124	VIYZIP	158.879	2.815
VIZHAR	159.856	2.973	VIZHAR	154.751	3.166	VIZBEP	159.811	2.941	VIZBEP	161.805	2.8	VIZGIY	152.133	2.948
VIZRUT	162.991	3.11	VIZVIM	166.498	2.751	VIZHAR	165.106	3.369	VIZMEW	159.026	2.756	VIZRON	150.473	3.453
VIZVIM	150.819	3.436	VIZVIM	162.546	2.857	VIZVIM	152.743	3.388	VIZVIM	154.165	2.797	VIZVIM	157.758	2.977
VIZVOS	158.117	2.958	VIZVOS	150.677	3.408	VIZVOS	167.196	2.747	VIZVOS	154.267	3.449	VIZVOS	154.241	2.785
VIZVUY	164.82	2.951	VIZWEJ	163.719	3.071	VIZVOS	162.3	2.856	VIZVUY	152.469	2.872	VIZVUY	166.813	2.794
VIZWEJ	150.42	3.028	VIZWIN	158.695	3.391	VIZWEJ	151.177	3.441	VIZWEJ	157.514	3.004	VIZWEJ	152.949	3.139
VIZWOT	159.54	3.459	VIZWOT	156.135	2.757	VIZWIN	161.37	2.87	VIZWIN	159.106	3.042	VIZWIN	165.239	2.7
VIZXEK	163.951	2.899	VIZXEK	170.299	3.223	VIZXEK	156.73	3.364	VIZXEK	159.642	3.026	VIZXEK	168.004	3.223
VOBFEZ	163.61	3.133	VOBFEZ	170.686	3.452	VIZXEK	166.659	2.899	VOBBET	171.569	3.019	VOBBET	155.055	3.019
VOBRUC	168.731	3.5	VOBTAK	171.226	3.477	VOBQEM	155.591	2.674	VOBQEM	156.969	3.481	VOBRUC	154.472	2.849
VOBVAM0	163.608	3.445	VOBVAM0	156.779	3.124	VOBTIT	157.614	2.707	VOBVAM	163.708	3.455	VOBVAM	156.934	3.13
VOBYET	151.203	2.801	VOBYIX	156.451	3.222	VOBVAM0	163.637	3.444	VOBVAM0	156.853	3.123	VOBXUI	152.017	3.084
VOBZEV	166.228	3.413	VOBZEV	153.476	3.233	VOBZAQ	164.705	3.227	VOBZAQ	167.553	3.356	VOBZEV	160.524	3.287
VOBZEV	164.682	3.18	VOBZEV	168.363	3.186	VOBZEV	159.7	3.028	VOBZEV	157.038	3.172	VOBZEV	151.051	3.471
VOCBOI	152.343	3.2	VOCBOI	170.814	2.963	VOCBOI	154.864	2.88	VOCBOI	166.216	2.994	VOCBOI	150.818	3.449
VOCDAW	172.682	3.043	VOCDIE	155.628	3.285	VOCBOI	168.468	3.23	VOCCID	164.111	3.149	VOCCUP	150.646	3.078
VOCDIE	154.196	2.585	VOCDIE	160.065	3.248	VOCDIE	159.122	2.86	VOCDIE	151.568	3.129	VOCDIE	157.666	3.25
VOCDIE	152.777	2.585	VOCDIE	157.914	3.107	VOCDIE	165.666	3.373	VOCDIE	164.461	3.42	VOCDIE	157.948	3.247
VOCDIE	150.38	2.86	VOCKAC	159.116	3.286	VOCDIE	165.137	3	VOCDIE	155.579	3.377	VOCDIE	168.637	2.991
VOCMEG	155.506	2.848	VOCMEG	166.209	2.932	VOCKIK	155.274	3.16	VOCMEG	150.517	2.804	VOCMEG	166.426	3.177
VOCMEG	153.569	3.075	VOCMEG	165.543	3.497	VOCMEG	165.375	2.825	VOCMEG	163.194	3.237	VOCMEG	168.457	3.129
VOCMIK	164.49	2.871	VOCMIK	166.571	3.209	VOCMEG	165.555	3.155	VOCMEG	170.193	2.901	VOCMIK	167.95	2.742
VOCYUI	163.594	3.39	VOCZUL	158.553	3.307	VOCMIK	166.603	2.871	VOCNIO	173.479	3.425	VOCPEL	170.659	3.158
VOCZUL	170.341	2.648	VOCZUL	153.974	2.84	VOCZUL	151.192	3.022	VOCZUL	165.066	2.84	VOCZUL	155.496	3.078
VOCZUL	166.14	3.267	VOCZUL	150.103	3.396	VOCZUL	167.854	2.648	VOCZUL	160.055	2.748	VOCZUL	170.498	3.175
VODDAT	161.698	3.332	VODDAT	167.255	3.221	VOCZUL	166.493	3.02	VODDAT	167.023	3.311	VODDAT	167.437	2.95
VODZIX	159.946	3.253	VOFLIM	159.748	2.881	VODKUV	160.676	3.029	VODZIX	153.976	3.056	VODZIX	153.324	3.072

VOFLIM	169.87	3.366	VOFROA	150.366	3.107	VOFLIM	157.695	3.131	VOFLIM	155.654	2.846	VOFLIM	157.692	2.846
VOFROA	155.546	3.05	VOFROA	160.807	2.822	VOFROA	164.872	3.107	VOFROA	163.276	2.848	VOFROA	163.154	2.913
VOFROA	154.165	3.098	VOFRUG	162.924	3.353	VOFROA	169.612	2.868	VOFROA	154.228	3.016	VOFROA	152.955	3.268
VOFSUH	151.792	3.47	VOFSUH	167.187	3.442	VOFRUG	156.587	3.182	VOFRUG	158.997	3.353	VOFRUG	166.996	2.872
VOFTES	157.549	2.771	VOFTIW	174.297	3.05	VOFTES	164.498	3.468	VOFTES	153.368	3.476	VOFTES	173.616	3.014
VOFVEU	169.749	2.977	VOFVEU	168.893	3.001	VOFTIW	163.246	3.494	VOFTIW	157.113	3.448	VOFTIW	158.712	2.779
VOFZOI	153.516	3.414	VOGBAX	161.44	3.401	VOFWAQ	159.975	3.146	VOFZOI	162.65	3.414	VOFZOI	160.713	3.131
VOGBAX	163.693	3.32	VOGBAX	158.576	3.094	VOGBAX	169.923	3.158	VOGBAX	157.57	3.452	VOGBAX	170.074	3.342
VOGBAX	155.525	2.909	VOGBOL	163.449	3.122	VOGBAX	157.063	3.065	VOGBAX	165.316	3.12	VOGBAX	160.61	3.32
VOGDEE	175.25	3.081	VOGDOO	154.535	3.389	VOGDEE	173.166	3.1	VOGDEE	156.069	3.477	VOGDEE	152.658	3.447
VOGROB	152.508	3.1	VOGROB	160.4	3.247	VOGQOX	175.713	3.405	VOGQOX	168.893	3.213	VOGQUD	163.816	3.128
VOGROB	157.886	2.887	VOGROB	161.329	2.755	VOGROB	156.139	3.145	VOGROB	156.941	3.175	VOGROB	162.037	2.754
VOGROB	156.267	3.136	VOGROB	155.856	3.21	VOGROB	161.288	2.832	VOGROB	158.721	2.804	VOGROB	162.093	2.816
VOGROB0	158.695	3.189	VOGROB0	160.235	2.8	VOGROB0	152.375	3.103	VOGROB0	161	3.278	VOGROB0	154.667	3.164
VOGROB0	163.369	2.841	VOGROB0	159.362	2.842	VOGROB0	159.476	2.892	VOGROB0	160.949	2.801	VOGROB0	160.75	2.871
VOGSES	150.287	3.268	VOGSET	176.346	2.925	VOGROB0	155.794	3.149	VOGROB0	156.237	3.206	VOGSES	157.608	3.097
VOGSET	151.517	3.234	VOGWIZ	158.185	3.484	VOGSET	170.018	3.16	VOGSET	160.223	3.138	VOGSET	160.185	2.96
VOHLUA	161.55	3.395	VOHLUA	153.271	2.849	VOGWUJ	153.263	3.102	VOHLUA	150.439	3.038	VOHLUA	162.302	3.303
VOHMAH	168.784	2.856	VOHMAH	154.819	3.226	VOHLUA	163.754	3.017	VOHMAH	157.476	2.832	VOHMAH	161.599	3.072
VOHNUC	170.125	2.774	VOJGAC	171.322	2.789	VOHMAH	155.855	3.052	VOHNUC	165.558	3.336	VOHNUC	150.265	2.834
VOJJAH	162.882	3.463	VOJJAH	152.783	3.463	VOJGEG	161.533	3.404	VOJGEG	158.134	3.2	VOJJAH	154.617	3.486
VOJNEQ	174.937	3.324	VOJNEQ	160.799	2.729	VOJJAH	165.783	3.433	VOJLUD	158.811	3.397	VOJNEQ	156.43	2.942
VOJNEQ	163.65	2.729	VOJNEQ	157.424	3.447	VOJNEQ	150.445	2.987	VOJNEQ	150.464	3.05	VOJNEQ	170.305	3.324
VOJQIW	157.281	3.431	VOJQIW	157.937	3.181	VOJNEQ	160.214	2.757	VOJQAM	153.902	3.007	VOJQIW	167.493	3.062
VOJVOI	167.318	2.936	VOKGOU	150.342	3.163	VOJQIW	163.005	3.229	VOJQIW	152.124	3.199	VOJQIW	162.434	3.044
VOKZUT	159.948	2.992	VOKZUT	151.601	3.381	VOKGOU	164.279	2.949	VOKLIT	150.891	2.876	VOKPOB	171.333	2.744
VOLJEN	150.209	3.387	VOLJEN	165.137	3.05	VOLJAJ	152.267	3.368	VOLJAJ	152.95	3.301	VOLJAJ	177.419	3.301
VOLJIS	152.027	2.986	VOLJIS	158.526	3.126	VOLJEN	159.592	3.343	VOLJEN	170.442	3.046	VOLJIR01	151.798	3.238
VOLJUD	152.934	2.926	VOLKAK	155.606	3.135	VOLJOY	166.313	2.827	VOLJOY	153.304	2.854	VOLJUD	153.667	3.307
VOLNES	167.799	3.152	VOLNES	166.356	2.864	VOLKAK	168.378	3.043	VOLNES	165.025	2.947	VOLNES	167.061	2.864
VOLSIC	165.959	2.749	VOLSIC	153.357	2.913	VOLNES	161.892	2.896	VOLNES	155.929	3.09	VOLRAR	166.956	2.751
VOMDAE	177.625	3.09	VOMDAE	173.219	3.279	VOLSIC	158.69	3.291	VOMDAD	160.452	3.073	VOMDAE	160.95	2.844
VOMDAG	162.02	2.949	VOMDAG	158.567	3.104	VOMDAE	151.255	2.802	VOMDAE	153.502	2.916	VOMDAE	173.969	2.844
VOMFUA	167.814	3.292	VOMGOV	164.199	3.264	VOMDAG	161.484	2.779	VOMDAG	150.902	3.037	VOMDEK	154.039	2.726
VOMHIS	172.118	2.825	VOMHIS	156.886	2.874	VOMHIQ	168.453	3.197	VOMHIQ	165.258	3.197	VOMHIS	157.868	2.874
VOMMOB	154.639	3.277	VOMMOB	164.04	3.244	VOMMET0	154.468	3.167	VOMMOB	152.598	3.423	VOMMOB	155.702	2.944
VOMNAO	155.164	2.879	VOMNAO	167.418	2.879	VOMMOB	156.349	3.003	VOMNAO	157.756	3.085	VOMNAO	158.455	3.23
VOMNUH	151.958	2.718	VOMNUH	162.188	2.931	VOMNUH	150.292	3.465	VOMNUH	178.19	2.949	VOMNUH	171.801	2.838
VONPAT	150.948	3.359	VONWOK	157.356	3.033	VOMPET	154.185	2.637	VOMVOM	154.243	3.319	VONMIU	174.597	2.951
VOPDAH	151.61	3.376	VOPDAH	166.024	3.376	VONWOK	163.514	2.953	VOPDAH	157.004	3.079	VOPDAH	162.394	3.287
VOPHER	165.427	3.301	VOPHER	150.864	3.003	VOPDIP	159.006	3.132	VOPDIP	158.665	2.939	VOPDIP	168.063	2.939
VOPJOB	161.859	2.596	VOPJUH	167.143	2.938	VOPJOB	150.382	3.253	VOPJOB	169.126	2.914	VOPJOB	167.541	3.066
VOPMAR	156.953	3.235	VOPMAR	153.195	3.27	VOPJUH	159.965	2.878	VOPJUH0	175.264	3.077	VOPKIY	163.609	3.077
VOPXEE	150.173	3.201	VOPXEE	155.989	3.401	VOPMAR	170.671	3.074	VOPWED	155.313	3.484	VOPWUT	167.266	3.087
VOQCIN	153.539	3.01	VOQCIN	163.738	3.142	VOPXEE	160.27	3.111	VOQBEM	155.533	3.262	VOQCIN	169.25	3.146
VOQJUK	153.892	3.242	VOQLIZ	178.012	3.274	VOQCOT	163.908	2.973	VOQCOT	178.035	3.09	VOQCOT	153.97	3.09
VOQVIK	153.288	2.732	VOQVIK	164.226	2.628	VOQLIZ	171.683	2.851	VOQVIK	151.79	3.443	VOQVIK	164.289	2.732
VORBUD	159.361	3.135	VORGER	158.313	2.945	VOQYEJ0	155.685	3.053	VOQZUZ	150.394	3.329	VOQZUZ	171.596	3.276
VORRAY	168.955	2.751	VORTUU	152.889	3.15	VORGER	160.823	3.187	VORGOC	151.315	3.089	VORGOC	159.951	3.098
VOSJOG	164.21	2.971	VOSJOG	164.883	3.363	VOSFUH	163.825	2.818	VOSJOG	158.71	2.885	VOSJOG	164.657	3.332
VOSZUA	162.088	2.821	VOTHUJ	160.891	3.477	VOSLIA	163.903	3.199	VOSMIC	170.293	3.172	VOSMIC	170.144	3.372
VOTXOV	163.998	3.264	VOVHOH	171.876	2.742	VOTHUJ	165.721	2.956	VOTHUJ	165.821	2.979	VOTQUT	152.78	3.337
VOVKOJ	177.9	3.088	VOVKOJ	151.452	3.482	VOVHOH	174.715	2.807	VOVHUN	166.703	2.84	VOVJID	167.159	3.428
VOVXEL	162.71	3.389	VOVXEL	164.266	3.475	VOVMAX	170.115	3.192	VOVMEB	153.901	3.15	VOVSOS	166.148	3.038
VOWJUQ	157.586	3.481	VOWKUP	152.155	3.292	VOVXEL	163.072	2.981	VOVXIP	159.84	3.081	VOVZUD	165.425	2.861
VOXJID	165.227	2.694	VOXJID	177.55	2.694	VOWRII	172.335	3.154	VOXHID	171.622	2.727	VOXJID	164.92	2.832
VOXJOL	151.546	3.231	VOXLID	167.29	2.855	VOXJOJ	162.131	3.45	VOXJOJ	158.447	3.23	VOXJOJ	161.995	3.424
VOXMEE	153.173	3.129	VOXMEE	164.405	3.045	VOXLID	171.024	2.855	VOXMAA	153.381	2.721	VOXMAA	169.11	3.406
VOXXOX	153.061	3.164	VOXXOX	161.566	3.187	VOXXIR	153.5	3.336	VOXXOX	155.884	3.164	VOXXOX	150.907	3.104
VOXXUD	156.914	3.458	VOXXUD	161.333	3.065	VOXXOX	156.119	3.137	VOXXUD	155.149	3.172	VOXXUD	152.462	3.172
VOXYAK	163.026	3.451	VOXYAK	170.462	2.965	VOXXUD	158.828	3.123	VOXYAK	155.11	3.21	VOXYAK	161.676	2.896
VOXYOW	156.491	3.296	VOYCAP	153.294	3.298	VOXYAK	173.432	2.969	VOXYAK	155.119	3.254	VOXYOW	169.988	3.472
VOYCAP	158.518	3.404	VOYCAP	158.27	3.231	VOYCAP	158.113	3.071	VOYCAP	151.179	2.832	VOYCAP	154.066	2.923
VOYZUG	150.045	2.833	VOZBAP	150.131	2.837	VOYZOA	151.453	2.836	VOYZOA	167.852	2.958	VOYZUG	168.709	3.022
VOZCIW	156.583	3.092	VOZCIW	153.858	3.002	VOZBAP	168.362	3.016	VOZCAO	151.524	3.483	VOZCIW	151.102	3.323
VOZCIW	167.041	3.367	VOZNAB	161.91	2.769	VOZCIW	160.862	3.04	VOZCIW	156.944	2.851	VOZCIW	164.959	3.376
VUBBIF	159.739	2.79	VUBBIF	155.969	3.245	VOZPUX	152.365	3.326	VOZPUX	160.888	2.941	VOZRUB	153.804	2.893
VUBJAD	169.426	2.931	VUBJAD	165.584	2.839	VUBBIF	158.404	3.5	VUBBIF	154.434	2.97	VUBBIF	158.903	2.744
VUBJAD	164.005	2.961	VUBLUD	157.974	3.193	VUBJAD	162.338	3.151	VUBJAD	154.417	2.897	VUBJAD	155.345	2.876
VUBPIT	157.058	2.696	VUBPIT	172.098	2.629	VUBLUD	165.893	3.203	VUBPIT	151.464	3.256	VUBPIT	150.317	3.047
VUBRIT	152.9	3.383	VUBRIT	169.49	3.131	VUBPIT	150.359	3.493	VUBPIT	159.513	3.274	VUBPOZ	162.856	3.246
VUBRIT	155.203	3.317	VUBRIT	159.991	3.043	VUBRIT	162.532	3.229	VUBRIT	158.086	3.218	VUBRIT	150.868	2.802
VUCBEB	166.004	3.106	VUCBEB	151.904	3.197	VUBRIT	171.65	3.183	VUBYOG	159.728	3.007	VUBYOG	151.557	3.394
VUCBIF	151.546	2.972	VUCBIF	160.881	3.046	VUCBEB	150.889	3.372	VUCBEB	151.589	2.851	VUCBIF	154.922	2.817
VUDJIQ	163.017	2.798	VUDJOW	163.901	2.864	VUCBIF	151.031	3.186	VUCTIW	172.216	3.494	VUDJIQ	172.137	2.845
VUDPAM	169.777	2.784	VUDPAM	152	3.104	VUDKEM	150.081	3.435	VUDKEM	166.917	3.126	VUDLIS	166.013	3.12
VUDRUI	158.987	3.346	VUDRUI	169.891	3.274	VUDPAM	168.63	3.34	VUDPAM	151.497	2.784	VUDRUI	155.768	3.127
VUDTAP	168.185	3.435	VUDTAP	175.907	3.384	VUDSAP	154.148	2.96	VUDTAP	153.035	3.089	VUDTAP	152.634	2.981
VUFHOW	172.233	3.186	VUFHOW	164.672	3.105	VUDTAP	167.347	3.052	VUDTAP	160.205	3.08	VUDTAP	163.943	2.986

VUFPOE	155.433	3.386	VUFPOE	156.895	3.001	VUFKEO	160.148	3.442	VUFKEO	158.577	3.143	VUFKEO	160.813	2.966
VUFSUM	153.963	3.148	VUFSUM	154.829	2.848	VUFQAS	166.247	3.266	VUFQIZ	165.727	2.911	VUFQOF	150.274	3.229
VUFSUM	157.435	3.006	VUFSUM	150.801	2.834	VUFSUM	168.26	3.38	VUFSUM	176.488	3.434	VUFSUM	151.275	2.663
VUFSUM	159.531	2.956	VUFSUM	171.668	3.468	VUFSUM	155.824	2.847	VUFSUM	152.261	2.559	VUFSUM	154.473	3.42
VUFTIC	151.356	2.761	VUFTIC	152.958	2.731	VUFTIC	166.337	3.179	VUFTIC	155.351	3.225	VUFTIC	173.154	2.956
VUFTIC	157.528	3.172	VUFTIC	160.693	3.307	VUFTIC	168.714	3.267	VUFTIC	174.284	3.138	VUFTIC	157.096	2.749
VUFTIC	159.352	3.056	VUFTIC	153.589	2.737	VUFTIC	168.921	2.776	VUFTIC	179.112	3.225	VUFTIC	156.105	2.776
VUFTIC	168.872	2.742	VUFTIC	171.664	3.322	VUFTIC	164.687	2.685	VUFTIC	174.741	3.138	VUFTIC	177.444	2.731
VUFWAV	163.149	3.463	VUFYOK	170.459	3.039	VUFTIC	174.799	3.293	VUFTIC	168.562	3.171	VUFTIC	159.779	2.929
VUGXIH	169.745	2.998	VUGXIH	161.618	2.965	VUGPAO	166.745	3.048	VUGTOJ	168.747	3.367	VUGXIH	150.93	2.974
VUHBUY	155.215	2.865	VUHFIR	167.024	2.97	VUGXIH	152.968	3.272	VUGXIH	158.734	2.844	VUHBUY	159.242	2.795
VUHKOZ	158.198	2.634	VUHLOB	156.775	2.807	VUHKIT	159.132	3.033	VUHKIT	156.325	2.915	VUHKOZ	177.533	3.336
VUHLUG	150.336	2.807	VUHMAN	173.834	2.755	VUHLOB	170.372	2.922	VUHLOB	153.12	2.915	VUHLOB	174.313	3.118
VUHQIZ01	170.921	2.867	VUHQUL	179.741	2.975	VUHMER	173.287	2.733	VUHQIZ	170.028	2.825	VUHQIZ	169.865	3.45
VUHREY	174.239	3.028	VUHREY	162.95	3.178	VUHQUL	168.775	3.385	VUHQUL0	179.253	2.854	VUHQUL0	167.757	3.37
VUHREY	175.425	3.006	VUHREY	155.5	2.73	VUHREY	174.488	2.982	VUHREY	152.013	2.822	VUHREY	173.395	3.025
VUHREY	165.031	3.104	VUHREY	157.888	3.178	VUHREY	161.626	2.822	VUHREY	154.436	2.905	VUHREY	164.707	2.862
VUHRIC	171.602	2.594	VUHRIC	172.256	2.594	VUHRIA	158.298	2.961	VUHRIC	175.842	3.327	VUHRIC	162.815	2.912
VUHRUO	154.726	3.162	VUHRUO	171.974	2.749	VUHRIC	155.933	3.027	VUHRUM	152.008	3.478	VUHRUM	155.326	3.224
VUHSAT	157.457	3.38	VUHSEZ	170.345	3.314	VUHRUO	150.678	2.749	VUHRUO	153.855	2.988	VUHSAT	151.844	3.274
VUHSEZ	154.332	2.938	VUHSEZ	150.069	3.211	VUHSEZ	175.139	2.744	VUHSEZ	155.39	3.003	VUHSEZ	156.504	2.919
VUJCIQ	169.451	2.862	VUJKIW	173.888	3.045	VUHYIJ	165.946	2.847	VUHYIJ	174.182	2.847	VUHZUU	169.688	3.166
VUKGUE	151.726	3.137	VUKGUE	168.85	3.462	VUJSUS	176.851	2.87	VUJSUS	172.748	3.421	VUKGOY	153.418	3.174
VUKHEP0	167.536	3.33	VUKHIT	178.525	2.812	VUKHAL	151.935	3.103	VUKHAL	168.214	3.424	VUKHEP	166.516	3.49
VUKHIT	151.26	3.069	VUKKUL01	167.808	3.07	VUKHIT	158.804	3.056	VUKHIT	172.797	2.926	VUKHIT	154.353	3.069
VUKLAS01	165.406	3.33	VUKLAS01	170.767	2.613	VUKKUL01	173.349	3.089	VUKKUL01	161.091	2.939	VUKKUL01	155.865	2.987
VUKLAS01	152.171	3.483	VUKLAS02	170.985	2.836	VUKLAS01	152	3.351	VUKLAS01	164.058	3.477	VUKLAS01	154.503	2.613
VUKLAS02	150.094	2.938	VUKLAS02	173.792	2.706	VUKLAS02	155.252	2.927	VUKLAS02	155.868	3.08	VUKLAS02	172.314	2.818
VUKLAS02	165.327	3.402	VUKLAS02	155.852	3.157	VUKLAS02	176.203	2.718	VUKLAS02	160.868	2.875	VUKLAS02	172.182	2.883
VUKLEW	153.073	3.132	VUKROJ	157.883	2.812	VUKLAS02	168.884	3.391	VUKLEW	160.241	3.408	VUKLEW	171.973	3.132
VUKYEG	155.934	3.304	VUKYEG	173.081	3.204	VUKROJ	155.819	3.159	VUKYAC	162.8	2.767	VUKYAC	169.114	2.988
VUMNID	151.362	3.287	VUMNUQ	151.583	3.208	VULNEV	150.626	3.435	VUMFUH	166.395	3.117	VUMNID	163.72	2.937
VUNWIN	159.529	3.491	VUNWIN	154.241	2.698	VUNJOG	166.593	2.943	VUNJOG	157.191	3.147	VUNWIN	164.766	2.814
VUNWOR	154.731	3.184	VUNWOR	154.73	3.049	VUNWIN	176.263	2.814	VUNWIN	152.102	2.571	VUNWIN	151.288	2.571
VUNXEI	158.257	2.939	VUNXEI	160.842	2.912	VUNWUX	170.849	3.29	VUNXEI	161.612	3.396	VUNXEI	175.599	3.044
VUNXIO	151.059	3.49	VUNXIO	158.226	2.89	VUNXIO	169.505	2.516	VUNXIO	153.829	3.443	VUNXIO	156.355	3.204
VUPGES1	157.43	3.175	VUPGES1	159.214	2.749	VUNXIO	166.845	2.516	VUNXIO	164.63	2.89	VUNYOW	170.563	2.919
VUPJEV	150.946	3.308	VUPJIZ	152.102	2.731	VUPGES1	153.075	3.431	VUPGES1	150.113	3.24	VUPGES1	160.828	3.093
VUPKAS	150.834	2.896	VUPKAS	165.713	3.477	VUPJIZ	159.087	3.198	VUPKAS	169.782	3.24	VUPKAS	170.584	3.483
VUPQON	150.088	3.447	VUQFOE	153.362	3.073	VUPKAS	163.606	3.378	VUPKAS	150.217	2.883	VUPMOL	152.111	3.215
VUQHAR	158.092	2.942	VUQHAR	166.849	3.322	VUQGIY	171.736	3.079	VUQGIY	163.893	3.114	VUQHAR	157.44	3.362
VUQHAR	157.432	2.772	VUQHEV	166.126	2.941	VUQHAR	174.281	3.244	VUQHAR	157.013	3.264	VUQHAR	154.583	2.76
VUQHEV	159.659	3.02	VUQHEV	163.357	3.207	VUQHEV	155.675	3.217	VUQHEV	165.04	3.052	VUQHEV	166.926	2.889
VURBIX	160.524	3.28	VURJUO	155.013	3.435	VUQQAB	157.739	3.451	VUQRUV	152.988	2.832	VURBIX	169.937	2.948
VURPOR	151.755	3.331	VURVUC	170.229	3.239	VURKUP	173.914	2.994	VURKUP	154.221	3.424	VURMIE	155.87	2.718
VUSLOO	167.287	2.843	VUSLOO	158.083	3.412	VURXIR	176.527	2.813	VUSLOO	155.177	3.222	VUSLOO	179.664	3.281
VUSLOO	156.527	3.222	VUSLUU	153.468	3.154	VUSLOO	177.644	2.949	VUSLOO	158.259	3.128	VUSLOO	169.874	3.313
VUSNUW	157.161	3.413	VUSNUW	172.674	3.437	VUSLUU	152.189	2.802	VUSNUW	155.036	2.894	VUSNUW	150.054	2.742
VUSXAL	166.563	3.039	VUSXAL	177.943	3.061	VUSTIP	159.056	2.996	VUSTOU	172.706	3.212	VUSTOU	155.451	3.04
VUTBUK	173.192	3.195	VUTBUK	153.734	3.051	VUSXAL	151.733	3.167	VUSXAL	177.362	3.384	VUTBUK	157.446	2.762
VUTBUK	174.364	3.026	VUTBUK	160.135	3.042	VUTBUK	157.909	3.068	VUTBUK	164.433	2.648	VUTBUK	155.305	3.005
VUTFIB	151.906	3.034	VUTFIB	151.597	3.071	VUTBUK	151.216	3.072	VUTBUK	157.455	2.709	VUTFIB	159.82	2.982
VUTFIB	154.921	3.132	VUTFIB	153.717	2.84	VUTFIB	157.667	3.071	VUTFIB	162.163	3.086	VUTFIB	159.937	2.954
VUTFIB	172.027	3.484	VUTFIB	167.572	2.963	VUTFIB	154.564	3.303	VUTFIB	165.493	2.82	VUTFIB	173.758	2.915
VUTHOL	172.659	3.022	VUTHUO	161.842	3.286	VUTFIB	157.295	2.954	VUTFIB	157.918	2.989	VUTHIF	166.215	2.851
VUTJAZ	165.967	2.92	VUTJAZ	175.896	3.151	VUTHUO	160.472	3.079	VUTHUO	152.931	3.028	VUTJAZ	163.086	2.917
VUTNOR	161.966	3.151	VUTTEN	156.17	3.047	VUTJED	157.823	3.118	VUTJED	163.301	3.406	VUTNOQ	165.192	3.21
VUTTEN	165.81	2.972	VUTTEN	152.286	3.448	VUTTEN	150.242	3.217	VUTTEN	175.833	3.029	VUTTEN	166.771	3.23
VUTTIR	153.041	2.791	VUTTIR	161.322	2.726	VUTTEN	159.55	3.336	VUTTEN	169.492	3.423	VUTTIR	168.779	3.392
VUTTIR	155.096	2.897	VUTTIR	152.002	3.318	VUTTIR	152.137	3.278	VUTTIR	155.12	3.007	VUTTIR	151.833	3.474
VUTTIR	157.251	3.199	VUTTIR	163.987	3.205	VUTTIR	157.227	3.485	VUTTIR	156.629	3.202	VUTTIR	162.413	3.481
VUVHAY	152.627	3.284	VUVJEC	161.304	2.933	VUTZAN	170.764	2.817	VUTZAN	159.527	3.058	VUVHAY	161.017	3.139
VUVLOQ	154.257	2.956	VUVLOQ	151.297	3.434	VUVJEC	165.395	3.262	VUVJEC	153.645	2.933	VUVLOQ	151.309	2.915
VUVLOQ	155.78	3.469	VUVLOQ	151.88	2.899	VUVLOQ	159.723	3.097	VUVLOQ	158.664	3.434	VUVLOQ	173.164	3.431
VUVWOC	176.394	3.071	VUVWOC	161.257	2.817	VUVLOQ	158.371	3.369	VUVWOC	159.092	2.995	VUVWOC	157.167	3.187
VUWHUU	152.295	2.719	VUWHUU	154.174	3.152	VUVZEV	163.114	3.301	VUVZIW	150.884	3.161	VUWHUU	154.63	3.252
VUWRAH	157.18	2.946	VUWRAH	152.996	3.061	VUWKOQ	153.538	3.426	VUWRAH	159.456	3.271	VUWRAH	158.836	2.89
VUWRAH	151.656	3.096	VUWRAH	156.795	3.324	VUWRAH	169.915	3.315	VUWRAH	152.487	2.972	VUWRAH	173.942	3.251
VUWRAH	150.091	2.953	VUWRAH	155.969	3.367	VUWRAH	176.173	3.13	VUWRAH	151.965	3.171	VUWRAH	153.122	2.889
VUWRAH	170.096	3.168	VUWRAH	164.444	3.232	VUWRAH	157.99	2.799	VUWRAH	162.904	3.251	VUWRAH	158.737	2.993
VUWWIX	157.888	3.079	VUWWIX	173.789	2.892	VUWRAH	152.333	2.777	VUWRAH	155.804	3.061	VUWWET	161.319	3.5
VUWWIX	160.908	2.847	VUWXAQ	152.531	3.48	VUWWIX	157.771	3.073	VUWWIX	176.122	2.884	VUWWIX	150.066	3.394
VUXFIH	157.622	2.805	VUXQUE	171.407	3.047	VUWXEU	165.742	3.143	VUXFEB	150.388	3.107	VUXFIF	157.08	2.897
VUXRAL	164.514	3.466	VUXRIT	150.919	2.857	VUXQUE	151.352	2.895	VUXQUE	159.443	3.015	VUXRAL	173.096	3.108
VUXWIX	162.277	3.141	VUXWIX	156.161	3.141	VUXRUF	156.711	3.062	VUXRUF	160.936	3.273	VUXRUF	155.873	3.319
VUXXOD	169.019	3.032	VUXXOD	166.543	3.149	VUXXOD	170.194	3.149	VUXXOD	159.506	3.308	VUXXOD	163.985	3.365
VUXYUJ	155.841	2.831	VUXYUJ	156.02	3.41	VUXYUJ	166.62	2.726	VUXYUJ	151.551	3.32	VUXYUJ	169.78	3.49
VUYLUA	158.072	3.357	VUYLUA	155.522	3.085	VUXZEU	173.112	3.377	VUXZEU0	162.641	3.105	VUXZEU0	156.316	3.241
VUYMOT	158.866	3.283	VUYMOT	170.789	2.94	VUYMAH	150.827	3.369	VUYMIP	168.751	3.069	VUYMIP	164.796	3.069

VUYRIU	150.834	3.141	VUYROA	157.12	2.873	VUYPAK	160.766	3.263	VUYPUD	164.418	3.384	VUYRIU	155.458	3.162
VUYVAQ	163.803	3.164	VUYVAQ	151.476	2.841	VUYROA	151.131	3.49	VUYROA	152.581	2.939	VUYROA	152.385	3.356
VUYWEV	155.74	3.417	VUYWIZ	152.949	3.132	VUYVAQ	172.291	3.285	VUYWEV	152.044	3.461	VUYWEV	161.507	3.307
VUYXET	162.697	3.437	VUYXUM	173.691	3.47	VUYWIZ	164.528	3.165	VUYWOF	172.02	3.13	VUYXAS	168.517	3.15
VUYZOI	152.479	3.353	VUYZOI	153.041	2.81	VUYZEY	162.534	3.47	VUYZEY	164.453	3.47	VUYZIC	156.352	3.408
VUZHAD	150.965	3.265	VUZHEH	165.665	2.915	VUYZOI	150.762	3.48	VUZGUW	161.855	2.844	VUZHAD	155.675	2.845
VUZKIN	158.709	3.429	VUZKIN	156.885	3.211	VUZHUX	168.581	3.049	VUZJIN	166.836	2.838	VUZJIN	162.807	3.218
VUZLAH	164.426	2.896	VUZLAH	163.776	2.657	VUZKIN	151.526	3.372	VUZLAH	164.131	2.657	VUZLAH	166.455	2.91
VUZLIP	173.097	3.254	VUZMOW	161.103	3.343	VUZLEL	168.173	2.553	VUZLEL	170.374	3.095	VUZLEL	165.687	2.553
VUZRAK	159.611	2.842	VUZRAK	151.727	3.459	VUZMOW	157.738	2.582	VUZMUC	163.319	2.953	VUZNEN	164.404	3.031
VUZSEP	152.176	3.315	VUZSEP	150.41	3.301	VUZRAK	152.987	3.314	VUZRUE	162.477	3.003	VUZSAL	161.455	3.362
WABDEL	154.002	3.052	WABLEU	163.896	3.222	VUZTAM0	165.044	3.289	WABCAE	171.196	3.273	WABDAH	151.993	2.988
WACCAE	170.224	3.006	WACCAE	176.262	3.424	WABPAU	167.891	3.245	WABSIB01	177.623	2.895	WABVON	154.512	3.258
WACCAE	155.128	3.24	WACGOY	154.264	3.214	WACCAE	167.979	3.289	WACCAE	177.342	3.305	WACCAE	167.4	2.807
WACHAJ	165.576	2.933	WACHAJ0	170.172	2.919	WACHAJ	169.39	2.97	WACHAJ	168.46	3.017	WACHAJ	168.153	3.026
WACHUD	163.115	3.279	WACHUD	166.012	3.158	WACHAJ0	169.471	2.976	WACHAJ0	165.88	2.883	WACHAJ0	168.901	2.978
WADHIS	166.036	3.276	WADHIS	171.006	3.015	WADBEL	159.766	2.942	WADBEL	169.165	3.243	WADHIS	177.171	3.027
WADJOA	158.088	3.377	WADJOA	153.499	2.919	WADHIS	154.23	3.07	WADHIS	173.786	3.384	WADHIS	158.84	2.961
WADJOA	157.994	3.255	WADJOA1	158.078	3.377	WADJOA	156.661	3.173	WADJOA	158.681	3.033	WADJOA	165.991	3.171
WADJOA1	165.998	3.171	WADJOA1	157.998	3.255	WADJOA1	153.507	2.921	WADJOA1	156.673	3.174	WADJOA1	158.692	3.034
WADZOT	163.567	2.886	WADZOT	174.881	2.758	WADWAC	155.408	3.012	WADWAC	150.466	3.306	WADWAC	158.283	3.497
WAFGOD	153.677	2.736	WAFGOD	153.321	2.924	WAFDUE	164.773	3.232	WAFGOD	167.749	2.905	WAFGOD	156.454	2.97
WAFPIG	170.024	3.457	WAFPIG	155.057	2.713	WAFGUJ	166.147	2.983	WAFGUJ	159.626	2.893	WAFGUJ	164.865	2.94
WAFSIJ	159.148	3.279	WAFZEL	178.314	2.901	WAFPOM	158.152	2.789	WAFSIJ	161.327	3.036	WAFSIJ	160.932	3.5
WAGFAL	166.772	3.317	WAGFAL	164.014	3.029	WAGBEO	163.489	3.271	WAGBEO	174.173	3.299	WAGDIU	157.131	2.896
WAGFUH	159.977	3.448	WAGGAP	166.233	2.907	WAGFAL	154.32	3.434	WAGFAL	162.393	3.219	WAGFUH	172.774	3.448
WAGGAP	157.745	3.204	WAGGAP	162.257	2.979	WAGGAP	158.202	3.073	WAGGAP	168.356	2.907	WAGGAP	159.075	3.071
WAGGET	152.737	3.328	WAGGIX	164.333	2.974	WAGGET	156.895	2.756	WAGGET	162.805	2.756	WAGGET	158.81	2.88
WAGGIX	159.462	3.008	WAGGIX	158.555	3.093	WAGGIX	160.908	3.037	WAGGIX	165.685	2.878	WAGGIX	164.735	2.878
WAGGIX	159.278	3.181	WAGGOD	160.281	2.827	WAGGIX	163.991	2.887	WAGGIX	162.72	2.887	WAGGIX	158.855	3.077
WAGGOD	158.011	3.01	WAGGOD	161.411	2.953	WAGGOD	162.47	3	WAGGOD	162.108	2.967	WAGGOD	158.198	2.827
WAGGOD	161.927	2.91	WAGHAQ	161.038	2.854	WAGGOD	160.409	2.816	WAGGOD	160.649	2.816	WAGGOD	164.229	2.955
WAGQUQ	160.151	3.372	WAGQUQ	176.902	3	WAGHEU	161.347	2.862	WAGQOK	160.457	3.156	WAGQOK	155.582	3.156
WAGSIK	169.644	3.106	WAGZAF	168.448	2.94	WAGQUQ	160.034	2.959	WAGQUQ	150.65	3.102	WAGQUQ	161.101	2.837
WAHHOE	159.035	3.394	WAHHOE	166.659	3.091	WAHDOZ	160.38	3.273	WAHDOZ	167.136	3.268	WAHHOE	156.199	2.969
WAHHOE	151.023	3.369	WAHHOE	151.164	2.696	WAHHOE	161.547	3.161	WAHHOE	151.018	2.972	WAHHOE	168.006	3.17
WAHHOE	153.614	2.919	WAHHUK	152.317	2.707	WAHHOE	157.114	2.901	WAHHOE	160.72	3.149	WAHHOE	166.97	3.481
WAHHUK	160.787	3.005	WAHJIZ01	163.216	3.48	WAHHUK	153.643	3.474	WAHHUK	154.214	3.5	WAHHUK	151.435	3.471
WAHJOH	165.393	3.038	WAHJOH	160.687	3.355	WAHJOH	151.833	3.363	WAHJOH	153.213	3.38	WAHJOH	159.488	2.956
WAHVEK	165.06	3.125	WAHVOU	151.742	3.061	WAHKAR	168.098	3.496	WAHVEK	161.653	2.81	WAHVEK	152.22	2.89
WAHVUA	174.168	2.9	WAHVUA	168.016	2.86	WAHVOU	150.132	3.175	WAHVUA	150.356	3.11	WAHVUA	169.778	2.86
WAJVUZ	163.4	3.232	WAJXEL	157.316	2.835	WAJQON0	151.463	3.297	WAJTEI	156.738	2.997	WAJTEI	154.488	3.017
WAJXEL01	163.751	3.467	WAJXEL01	173.071	3.199	WAJXEL	165.727	3.414	WAJXEL01	158.716	2.97	WAJXEL01	164.524	3.088
WAJXEL02	153.943	3.143	WAJXOX	154.43	2.915	WAJXEL02	178.459	2.887	WAJXEL02	161.62	3.348	WAJXEL02	155.509	2.632
WAKGAS	159.562	3.22	WAKGAS	166.309	3.167	WAJXOX	151.057	3.119	WAJXOX	154.844	2.915	WAJXOX	160.812	3.447
WAKLEC	159.093	3.279	WAKNED	167.401	3.241	WAKGAS	153.156	3.365	WAKGOG	169.545	3.319	WAKLEC	160.27	3.223
WAKTAG	153.47	3.387	WAKTEK	152.2	3.45	WAKPAB	153.44	3.195	WAKPAB	150.634	2.964	WAKTAG	160.833	3.136
WAKVAF	174.246	3.1	WAKVAF	160.412	3.1	WAKTEK0	151.775	3.464	WAKTUA	172.527	3.442	WAKTUA	154.127	3.162
WALPIL	178.165	3.006	WALPIL	152.969	2.938	WAKVEM	152.721	3.402	WALGOG	168.926	2.906	WALNOP	165.209	2.904
WALYAN	166.938	3.433	WALYER	157.927	3.201	WALPIL	150.997	2.87	WALYAN	154.674	2.898	WALYAN	162.41	2.755
WAMMAB	165.983	3.401	WAMMAB	151.551	3.184	WAMCIW	150.629	3.335	WAMCIW	159.385	2.997	WAMMAB	160.915	3.252
WAMXER	162.361	2.829	WAMXIV	170.193	3.246	WAMMAB	166.881	3.252	WAMMUT	165.12	3.445	WAMXER	157.013	2.791
WANKAZ	158.14	3.132	WANKAZ	157.041	3.121	WANJUS	161.255	3.031	WANJUS	161.233	3.434	WANKAZ	161.662	3.023
WAPDEA	165.494	3.001	WAPHAX	158.527	3.036	WANKAZ	159.557	3.082	WANKED	151.246	2.906	WANKED	164.976	3.08
WAPHEB	158.166	3.264	WAPHEB	154.647	3.485	WAPHAX	159.673	2.71	WAPHAX	176.435	2.71	WAPHEB	171.444	3.005
WAPHEB	158.99	3.253	WAPLAB	155.659	3.148	WAPHEB	151.777	2.81	WAPHEB	153.306	3.141	WAPHEB	164.188	2.924
WAPWOD	164.092	3.169	WAPWOD	167.971	3.283	WAPLAB	161.718	3.338	WAPPAF	151.918	3.209	WAPWOD	150.934	3.434
WAQLOT	154.404	2.63	WAQLOT	162.249	3.397	WAPWOD	150.84	3.428	WAPWOD	165.386	3.164	WAPWOD	167.178	3.277
WAQMEH	157.695	3.052	WAQMEH	153.951	3.264	WAQLUZ	157.973	3.437	WAQMEH	158.917	3.074	WAQMEH	154.535	2.819
WAQVER	159.687	3.23	WAQVOB	154.685	3.047	WAQQAG	153.107	3.396	WAQTUF0	152.742	3.405	WAQVER	169.486	2.788
WAQXUJ	160.74	3.417	WAQXUJ	150.399	3.077	WAQWOA	150.657	3.391	WAQXUJ	160.515	2.984	WAQXUJ	158.871	2.797
WARKEI	154.514	3.384	WARKEI	165.492	3.107	WAQXUJ	154.896	2.936	WAQXUJ	174.821	2.643	WAQXUJ	164.75	2.969
WARKEI	163.332	3.107	WARKEI	160.675	3.051	WARKEI	153.865	3.044	WARKEI	162.387	3.051	WARKEI	155.662	3.044
WARROZ	164.01	2.986	WARROZ	151.727	2.821	WARROZ	154.12	3.054	WARROZ	153.133	3.054	WARROZ	161.067	2.986
WARTIW	159.181	3.117	WARVER	156.823	2.853	WARROZ	164.966	2.993	WARROZ	170.362	2.993	WARTIW	156.869	3.181
WARWET	160.875	2.898	WARWET	155.426	3.295	WARVER	151.139	2.873	WARWES	157.844	3.047	WARWET	174.507	3.312
WASDON	150.631	3.195	WASDON	152.356	3.26	WARWET	156.944	3.251	WARWET	178.353	3.049	WASDON	176.631	3.389
WASDON	154.219	2.987	WASDON	168.522	2.987	WASDON	150.477	3.155	WASDON	152.589	2.926	WASDON	177.291	3.155
WASHUX	155.966	3.108	WASHUX	154.22	3.289	WASDON	169.374	2.926	WASHUX	158.584	3.408	WASHUX	158.922	2.953
WASJAF	153.445	2.777	WASJAF	158.658	3.31	WASHUX	165.731	3.271	WASHUX	153.982	3.141	WASJAF	150.438	2.965
WASJAF	164.351	2.947	WASJAF	155.697	3.137	WASJAF	161.922	3.205	WASJAF	158.9	2.761	WASJAF	152.965	3
WASJEJ	158.229	2.752	WASJEJ	153.207	3.001	WASJAF	172.692	3.324	WASJAF	152.491	3.325	WASJEJ	161.028	3.202
WASJEJ	154.396	2.757	WASJEJ	150.525	2.947	WASJEJ	164.284	2.96	WASJEJ	155.133	3.143	WASJEJ	153.109	3.318
WASJUZ	157.629	3.154	WASQOZ	157.804	3.271	WASJEJ	172.715	3.333	WASJEJ	157.96	3.321	WASJUZ	169.556	2.895
WASQOZ	154.658	3.197	WASQOZ	154.374	3.412	WASQOZ	156.376	3.03	WASQOZ	151.491	3.243	WASQOZ	157.632	3.31
WASTEP	164.974	2.983	WASWIX	152.746	3.435	WASQOZ	153.754	3.266	WASQOZ	166.294	2.904	WASQOZ	156.197	2.98
WATVUM0	152.861	3.26	WATVUM0	178.263	3.415	WATHUY	157.647	3.138	WATHUY	166.638	2.933	WATVUM0	161.444	3.197
WATXOG	164.678	3.108	WATXOG	155.439	2.803	WATWUL	153.45	3.314	WATWUL	173.287	3.103	WATXEW	155.41	3.242
WAVBUR	174.031	3.267	WAVFOS	170.366	2.689	WAVBED	156.644	3.309	WAVBOL	170.764	3.201	WAVBON	150.414	3.304

WAVFOS	167.101	2.948	WAVGAC	153.609	3.483	WAVFOS	155.159	3.414	WAVFOS	154.87	3.246	WAVFOS	160.796	3.459
WAVTAR	169.285	2.979	WAVTAR	163.869	3.008	WAVHAF	169.807	2.945	WAVHAF	167.349	3.261	WAVTAR	150.721	3.455
WAVTAR	155.178	3.047	WAVTAR	162.048	3.176	WAVTAR	155.738	3.394	WAVTAR	169.399	3.258	WAVTAR	159.308	3.035
WAVXOJ	177.179	2.859	WAWBON	175.024	3.028	WAVTAR	157.253	3.394	WAVTEV	164.879	3.458	WAVTEV	152.111	2.933
WAWCEE	156.957	3.058	WAWCEE	157.793	2.802	WAWBON	165.712	3.21	WAWBON	165.728	3.03	WAWCEE	167.377	3.272
WAWCII	153.135	2.835	WAWCII	156.698	3.438	WAWCEE	165.774	2.824	WAWCII	152.761	3.412	WAWCII	166.672	2.982
WAWCOC	161.071	3.169	WAWCOC	161.839	3.36	WAWCII	162.456	2.864	WAWCII	166.964	2.795	WAWCOC	152.236	3.18
WAWCOC	167.331	3.182	WAWCOC	154.646	3.423	WAWCOC	153.441	3.442	WAWCOC	150.943	3.226	WAWCOC	168.734	3.22
WAWCOC	150.43	3.261	WAWCUU	150.622	3.441	WAWCOC	159.277	2.822	WAWCOC	153.852	3.247	WAWCOC	165.799	3.358
WAWDAB	162.45	3.188	WAWDAB	156.068	3.303	WAWCUU	151.465	2.976	WAWDAB	150.548	3.015	WAWDAB	152.843	3.303
WAWDEG	166.047	3.352	WAWDEG	168.3	3.012	WAWDAB	160.055	2.794	WAWDAB	165.468	3.073	WAWDAB	153.969	3.167
WAWJOV	168.122	3.144	WAWKIR	162.276	3.265	WAWDEG	164.2	3.352	WAWDOC	151.04	2.867	WAWJOV	161.851	3.183
WAXFIO	162.685	2.851	WAXFIO	175.167	2.579	WAWYIE	150.304	3.263	WAXFIO	151.684	3.052	WAXFIO	151.578	3.27
WAXFIO	150.047	3.259	WAXFIO	169.919	2.743	WAXFIO	164.642	3.006	WAXFIO	156.284	2.881	WAXFIO	150.319	3.358
WAXFUA	153.675	2.818	WAXFUA	167.148	3.126	WAXFIO	164.554	2.916	WAXFIO	150.153	3.129	WAXFUA	158.075	3.041
WAXFUA	157.951	3.323	WAXFUA	166.809	2.996	WAXFUA	158.841	3.271	WAXFUA	150.7	3.126	WAXFUA	161.344	3.489
WAXTUM	155.9	2.863	WAXTUN	155.041	3.128	WAXNAN	152.612	3.343	WAXTOG	174.432	3.351	WAXTOG	156.021	2.923
WAXVAU	162.812	3.495	WAYCUU	161.509	2.808	WAXTUN	165.015	2.903	WAXTUN	172.352	3.437	WAXTUN	158.316	3.128
WAYJEO	158.592	3.274	WAYJEO	157.194	3.001	WAYJEO	173.991	2.935	WAYJEO	167.849	3.351	WAYJEO	176.11	3.137
WAYMOB	155.717	2.889	WAYMOB	169.818	3.285	WAYMIV	161.685	2.802	WAYMOB	174.631	3.291	WAYMOB	150.085	3.443
WAYTAV	169.419	3.396	WAYTAV	158.094	3.282	WAYNIW	154.203	3.162	WAYNIW	150.22	2.782	WAYTAV	156.466	2.633
WAYTAV	171.922	3.384	WAYTAV	158.232	2.618	WAYTAV	169.747	2.605	WAYTAV	171.603	2.633	WAYTAV	158.805	3.254
WAYTUL	170.131	2.655	WAYTUL	157.377	3.159	WAYTAV	172.167	2.618	WAYTAV	151.025	3.17	WAYTAV	156.893	2.605
WAYYAW	163.928	3.361	WAYYEA	151.204	2.71	WAYWUR	163.745	3.42	WAYYAW	158.152	3.361	WAYYAW	162.184	3.151
WAYYIE	161.202	3.13	WAZKEQ	165.674	3.435	WAYYEA	171.632	3.43	WAYYEA	158.525	3.442	WAYYIE	160.671	3.475
WAZXII02	168.413	3.192	WAZXII02	154.311	3.443	WAZREX	167.142	3.441	WAZVEC	156.39	3.471	WAZVEC	156.409	3.357
WAZYON	162.616	2.982	WAZYON	154.023	3.187	WAZYAZ	156.435	3.241	WAZYAZ	162.378	2.757	WAZYAZ	173.124	3.059
WEBCOX	165.674	2.774	WEBCUD	151.609	2.804	WEBCAH	157.508	3.247	WEBCAH	153.402	3.381	WEBCOX	165.625	3.247
WEBNAU	169.092	2.883	WEBNUO(	160.904	2.923	WEBCUD	159.927	3.271	WEBKEX	164.072	2.975	WEBNAU	166.52	3.248
WEBYAH(	163.529	2.725	WEBZIQ01	162.398	3.295	WEBNUO(	167.445	2.828	WEBVOR	162.619	3.117	WEBYAH	163.852	2.731
WECPOK	154.465	3.391	WECPOK	166.369	3.096	WECMOK	166.65	3.034	WECPAW	156.209	2.844	WECPIE	167.424	3.14
WEDTEE	159.097	3.33	WEDTEE	155.996	2.939	WECRAX(	150.007	3.218	WEDLUO	155.24	3.029	WEDSUW	168.137	3.183
WEDXIM	155.131	3.157	WEDXUB	159.393	3.432	WEDTEE	152.305	3.208	WEDTEE(	158.549	3.233	WEDTEE(	152.259	2.891
WEDYUD	157.762	2.892	WEDYUD	170.457	3.089	WEDYOX	178.415	2.893	WEDYOX	162.619	3.198	WEDYUD	155.107	3.173
WEFFEU	152.66	3.152	WEFFEU	159.662	3.395	WEFDUK	154.576	3.422	WEFDUK	152.373	3.125	WEFDUK	160.764	3.493
WEFHIA	176.305	3.14	WEFJUQ	171.465	3.036	WEFGIX	152.287	2.846	WEFGIX	169.948	3.35	WEFGIX	150.282	3.23
WEFJUQ	151.764	2.876	WEFJUQ	159.685	2.82	WEFJUQ	174.106	3.286	WEFJUQ	171.169	3.225	WEFJUQ	159.89	3.061
WEFJUQ	158.746	2.907	WEFKUO	150.028	2.908	WEFJUQ	155.627	3.473	WEFJUQ	160.592	2.868	WEFJUQ	155.578	3.224
WEFQIH	165.811	3.452	WEFQIK	166.772	3.058	WEFKUO	168.444	2.977	WEFKUO	160.662	2.994	WEFKUO	151.319	2.908
WEFYIT	160.576	2.985	WEGJUQ	158.914	3.147	WEFVIN	167.07	2.791	WEFVIN01	167.028	2.799	WEFYIT	152.039	3.252
WEGMED	153.611	3.385	WEGMED	165.367	3.037	WEGJUQ	165.955	3.006	WEGJUQ	151.317	3.459	WEGJUQ	163.723	3.461
WEGMED	157.277	2.942	WEGMED	163.67	3.156	WEGMED	162.832	3.384	WEGMED	170.98	2.835	WEGMED	173.359	2.721
WEGMUT	177.357	3.128	WEGRAD	157.211	2.994	WEGMIH	177.275	3.12	WEGMOM	150.165	3.245	WEGMOM	166.831	3.206
WEHBUK	156.764	3.334	WEHCIV01	174.94	2.992	WEHBOD	152.958	3.206	WEHBOD	150.197	3.206	WEHBOD	155.447	3.232
WEJBET	171.706	2.81	WEJBET	172.708	2.819	WEHCIV01	151.694	3.41	WEHDET	170.913	3.033	WEHMAZ	151.42	3.141
WEJBET	169.636	2.84	WEJBET	169.486	2.949	WEJBET	170.756	2.855	WEJBET	168.023	3	WEJBET	168.779	3.012
WEJSEK	154.477	3.399	WEJSEK	165.312	3.302	WEJBET	172.852	2.991	WEJBUL	163.805	2.982	WEJBUL	153.711	3.305
WEKKAZ	159.99	3.077	WEKKAZ	151.744	3.206	WEKFIF	153.926	3.083	WEKFIF	156.03	2.95	WEKFIF	155.065	3.413
WEKVUG	154.464	3.029	WEKWAN	153.107	2.906	WEKTIP	172.21	3.335	WEKTIP	172.912	3.335	WEKTIP	156.381	2.814
WELCEW	168.952	3.119	WELDEZ	153.038	3	WEKZUH	164.472	2.764	WEKZUH	159.403	2.764	WEKZUH	158.173	3.052
WELHII	165.06	2.82	WELHII01	164.801	2.826	WELDUN	154.305	2.995	WELFAV	151.363	2.973	WELFAV	151.327	2.763
WELQAH	158.768	2.949	WELQAH	171.906	3.045	WELJUS	154.961	3.13	WELMUX	157.952	3.421	WELQAH	160.025	3.34
WELYAP	150.322	3.153	WELYAP	164.202	3.483	WELQAH	154.287	3.434	WELQAH	150.351	3.176	WELYAP	160.732	2.743
WELYET	165.956	2.899	WELYET	155.083	3.201	WELYAP	153.602	2.966	WELYAP	153.418	2.835	WELYAP	155.639	3.221
WELYET	170.595	3.228	WELZUK0	170.718	3.092	WELYET	155.698	3.155	WELYET	158.751	3.01	WELYET	153.357	3.359
WEMKAA	161.038	3.078	WEMKAA	156.103	2.809	WEMGUU	165.941	2.76	WEMGUU	164.581	2.76	WEMKAA	177.994	2.725
WEMLAB	153.16	3.203	WEMLAB	164.784	2.831	WEMKAA	168.326	3.194	WEMLAB	151.752	3.385	WEMLAB	164.904	2.977
WENBIB	172.818	3.201	WENCEX	163.487	3.261	WEMSUF	152.552	3.367	WEMSUF	157.86	3.313	WEMYIY	168.331	2.974
WENDOJ	162.462	2.858	WENDOJ	161.396	2.849	WENCEZ	168.127	3.418	WENDAV	162.819	2.98	WENDAV	162.113	3.04
WENKEG	150.931	2.775	WENKEG	165.313	3.192	WENKAC	164.053	3.004	WENKAC	176.632	3.396	WENKAC	154.987	2.871
WENKIK	169.482	3.086	WENKIK	165.668	3.223	WENKEG	166.898	3.113	WENKEG	160.821	2.881	WENKIK	174.229	3.058
WENKIK	173.189	3.342	WENKIK	160.046	3.385	WENKIK	151.209	2.783	WENKIK	169.881	3.036	WENKIK	161.684	2.937
WENKIK	158.581	3.413	WENKIK	156.253	3.118	WENKIK	178.563	3.069	WENKIK	155.833	2.839	WENKIK	154.797	3.191
WENXIA	160.451	3.281	WENZAU	169.599	3.069	WENKIK	154.999	2.881	WENKIK	159.118	2.961	WENKIK	165.357	2.617
WEPHEH	150.581	3.277	WEPHEH	155.429	3.337	WEPCAZ	153.357	2.918	WEPCAZ	160.402	2.942	WEPGEH	158.634	3.118
WEPMUD	169.999	3.421	WEPMUD	155.863	3.24	WEPKAD	164.845	3.25	WEPKUA	171.04	2.991	WEPMUD	152.821	3.345
WEPNAK	159.527	3.401	WEPNAK	154.827	3.206	WEPMUD	157.585	2.857	WEPMUD	156.923	3.194	WEPMUD	151.466	3.473
WEPNAK	160.711	3.168	WEPNAK	159.264	3.245	WEPNAK	165.775	3.317	WEPNAK	154.926	3.474	WEPNAK	153.679	3.382
WEPQOZ	158.785	3.48	WEPQOZ	159.023	3.424	WEPNAK	161.189	3.453	WEPPEO	159.61	3.476	WEPQOZ	156.237	2.805
WEPQOZ	160.946	2.858	WEPQOZ	162.928	3.273	WEPQOZ	155.028	2.805	WEPQOZ	158.786	3.171	WEPQOZ	152.285	3.048
WEPRIS	166.231	3.21	WEPRIS	158.209	3.465	WEPQOZ	156.746	3.344	WEPQUF	166.292	3.341	WEPRIS	164.805	3.197
WEPSOD	173.694	3.004	WEPSUJ	151.469	3.157	WEPRIS	154.271	3.335	WEPRIS	151.455	3.179	WEPRIS	159.707	3.037
WEPSUJ	160.135	3.06	WEPTUJ	161.263	3.018	WEPSUJ	157.087	3.241	WEPSUJ	162.394	2.997	WEPSUJ	154.114	2.861
WEPWUJ	154.248	3.276	WEQGAB	150.11	3.152	WEPWUJ	152.688	3.375	WEPWUJ	151.007	3.426	WEPWUJ	157.992	2.923
WEQGAB	158.283	2.87	WEQJEK	168.69	3.161	WEQGAB	155.965	2.812	WEQGAB	159.963	3.484	WEQGAB	174.644	3.162
WEQVUM	160.232	3.365	WEQVUM	161.592	2.97	WEQPIU	166.477	3.465	WEQPIU	152.394	3.063	WEQPIU	157.047	3.244
WEQZAT(	168.592	2.782	WERFIL	160.841	3.197	WEQXOJ	165.919	2.91	WEQXOJ	162.7	2.91	WEQZAT(	159.09	3.062
WESBEB	164.543	3.076	WESJEK	161.04	3.227	WERHOT	172.073	2.664	WERXOG	167.887	3.04	WERXOG	159.295	3.011
WESJEK	153.399	2.98	WESJEK	150.99	3.216	WESJEK	159.645	3.304	WESJEK	158.574	3.296	WESJEK	170.485	3.048

WESJEK	151.654	3.116	WESJEK	166.966	3.46	WESJEK	172.668	2.708	WESJEK	152.601	3.072	WESJEK	157.92	3.226
WESJIO	167.226	3.169	WESJIO	151.076	3.386	WESJEK	158.22	2.98	WESJIO	158.696	3.215	WESJIO	163.562	3.288
WESJIO	150.793	3.39	WESLAI	150.975	3.068	WESJIO	174.575	2.789	WESJIO	150.872	3.24	WESJIO	160.678	3.335
WESSAS	153.243	3.297	WESXIC	161.113	3.417	WESMAJ	177.58	2.936	WESPUI	166.484	2.991	WESPUI	152.588	3.034
WESYUS	164.932	3.158	WESZAZ	154.492	2.67	WESYUS	150.597	3.019	WESYUS	150.39	3.421	WESYUS	154.087	2.976
WESZAZ	158.406	2.844	WESZAZ	157.481	2.986	WESZAZ	178.792	3.131	WESZAZ	155.963	2.649	WESZAZ	166.68	2.746
WESZAZ	154.632	2.67	WESZAZ	172.542	3.024	WESZAZ	172.582	2.746	WESZAZ	170.33	2.932	WESZAZ	156.544	2.649
WETFAG	170.312	2.933	WETFAG	166.926	3.186	WESZAZ	176.475	3.145	WESZIE	168.297	3.344	WESZIE	162.316	2.905
WETHUY	176.459	2.709	WETLOW	176.169	3.219	WETHUY	172.93	3.047	WETHUY	170.425	2.709	WETHUY	158.828	3.331
WEVJIU	161.039	3.234	WEVLEP	151.543	3.355	WETNIW	160.055	2.949	WETNIW	152.747	2.811	WETNIW	156.285	2.765
WEVNEQ	160.994	2.983	WEVNEQ	158.655	3.161	WEVMOA	163.923	3.063	WEVMOA	164.432	3.081	WEVNEQ	157.656	3.161
WEWBUV	156.138	2.926	WEWBUZ	153.201	3.241	WEVPAR	157.113	2.968	WEVVAV	159.761	3.053	WEVYOM	159.274	3.107
WEWDOR	174.459	2.848	WEWDOR	162.085	2.9	WEWCAD	160.187	3.353	WEWCUV	153.776	3.065	WEWDAE	170.515	3.271
WEWQUM	161.42	2.883	WEWWAV	161.649	3.213	WEWFOV	173.094	3.423	WEWFOV	162.36	3.318	WEWFOV	157.666	3.222
WEWWAV	161.531	3.327	WEWWAV	155.669	2.883	WEWWAV	162.51	3.102	WEWWAV	165.877	3.102	WEWWAV	157.22	3.327
WEWZAD	172.096	3.412	WEWZAD	170.204	2.837	WEWWAV	166.06	2.883	WEWZAA	169.932	3.097	WEWZAA	170.15	3.115
WEXFOU	151.164	3.37	WEXFOU	152.449	3.34	WEWZAD	162.265	3.497	WEWZAD	164.773	3.094	WEXFOU	152.322	3.457
WEXJOZ	150.718	3.209	WEXJOZ	151.987	3.397	WEXJOZ	171.818	2.956	WEXJOZ	170.92	3.334	WEXJOZ	155.449	3.367
WEXJOZ	169.421	3.358	WEXJOZ	176.07	3.388	WEXJOZ	155.074	3.016	WEXJOZ	164.277	2.872	WEXJOZ	174.772	3.072
WEXJUF	159.772	2.93	WEXJUF	174.614	3.238	WEXJOZ	157.19	3.335	WEXJOZ	151.269	3.155	WEXJUF	165.401	3.137
WEXJUF	164.951	3.202	WEXJUF	156.935	3.343	WEXJUF	176.445	3.151	WEXJUF	161.19	2.93	WEXJUF	172.996	2.833
WEXPOI	157.465	3.435	WEXPOI	152.631	3.296	WEXJUF	158.054	2.978	WEXJUF	153.776	3.124	WEXJUF	166.633	3.137
WEXSEB	153.46	2.704	WEXSEB	152.552	2.909	WEXQUP	175.049	2.946	WEXSEB	152.514	2.704	WEXSEB	151.863	3.095
WEXSEB	152.055	2.538	WEXSEB	175.903	2.75	WEXSEB	166.793	3.176	WEXSEB	157.032	2.909	WEXSEB	162.148	2.932
WEXSIF	158.723	3.122	WEXSIF	155.416	2.794	WEXSIF	158.415	3.302	WEXSIF	154.899	3.085	WEXSIF	151.649	2.619
WEXSIF	150.84	2.977	WEXSIF	166.729	3.349	WEXSIF	167.294	2.989	WEXSIF	157.579	2.798	WEXSIF	151.416	3.184
WEXSIF	162.606	3.309	WEXSIF	152.157	2.888	WEXSIF	167.982	3.337	WEXSIF	170.433	3.369	WEXSIF	160.278	2.831
WEXSIF	153.084	3.154	WEXSIF	170.98	3.131	WEXSIF	160.685	2.714	WEXSIF	170.72	3.474	WEXSIF	159.942	3.365
WEXVUR	162.301	3.051	WEXVUR	180	2.962	WEXTOK	159.407	3.327	WEXVIG	152.766	3.026	WEXVUR	180	2.962
WEYPUO	165.758	2.877	WEYPUO	164.226	3.243	WEXWEC	152.49	3.149	WEYPUO	154.21	3.076	WEYPUO	163.707	3.135
WEYQUQ	162.625	2.933	WEYSID	165.641	3.122	WEYPUO	166.482	2.927	WEYPUO	155.516	3.099	WEYQEZ	152.815	2.853
WEYSID	152.953	3.303	WEYSID	153.91	3.423	WEYSID	158.198	2.917	WEYSID	161.94	3.348	WEYSID	160.165	3.292
WEYVOM	163.695	2.946	WEYVOM	166.038	2.958	WEYSID	164.601	2.93	WEYSID	152.472	3.048	WEYVOM	157.978	3.179
WEYVOM	168.938	2.955	WEYWEE	155.153	3.385	WEYVOM	164.61	2.926	WEYVOM	168.378	3.013	WEYVOM	162.267	2.943
WEYXOQ	158.153	3.186	WEYXOQ	152.63	2.72	WEYWUV	164.643	3.056	WEYXOQ	156.133	3.279	WEYXOQ	157.708	2.955
WEZGUG	163.423	3.452	WEZGUG	162.054	3.107	WEYZAE	161.971	3.111	WEZCOT	169.24	3.168	WEZGUG	155.683	3.038
WEZKIY	161.813	3.31	WEZKIY	151.723	3.253	WEZKEU	150.94	3.1	WEZKEU	166.579	3.275	WEZKIY	155.825	3.016
WEZPAV	163.601	3.121	WEZPIA	150.136	3.392	WEZKIY	167.649	3.09	WEZKIY	160.889	3.063	WEZPAV	153.553	2.739
WEZPIB	158.967	3.5	WEZPIB	152.912	2.909	WEZPIA	167.334	3.132	WEZPIB	154.163	3.118	WEZPIB	150.354	2.795
WEZPIB	151.696	2.996	WEZPIB	157.278	3.118	WEZPIB	167.186	2.839	WEZPIB	166.642	3.499	WEZPIB	155.37	3.118
WEZPIB	159.88	3.173	WEZPIB	158.276	3.024	WEZPIB	172.449	3.03	WEZPIB	158.816	2.839	WEZPIB	166.948	3.07
WEZPIB	154.183	2.89	WEZPIB	151.999	3.054	WEZPIB	152.955	3.07	WEZPIB	165.13	3.004	WEZPIB	151.766	2.682
WEZPIB	155.431	2.996	WEZVEG	162.104	2.84	WEZPIB	169.619	3.134	WEZPIB	152.201	3.237	WEZPIB	168.143	3.218
WEZWEH	153.248	3.13	WEZWOR	153.404	3.089	WEZWEH	154.493	3.127	WEZWEH	150.867	3.13	WEZWEH	161.931	2.919
WIBGEW	154.275	2.866	WIBGEW	160.29	3.014	WEZWOR	172.606	3.212	WIBGAS	169.336	3.035	WIBGEW	156.463	2.866
WIBPII	156.485	3.142	WIBPII	153.98	3.425	WIBGIA	163.066	2.672	WIBGIA	165.469	2.929	WIBGIA	163.582	2.937
WIBPOQ	165.558	2.924	WIBPOQ	159.747	2.924	WIBPII	156.043	2.778	WIBPII	162.258	3.437	WIBPII	171.514	2.928
WIBQEF	166.754	3.445	WIBQEF	159.284	2.885	WIBQEF	160.64	3.213	WIBQEF	155.943	2.703	WIBQEF	173.834	3
WIBQEF	159.732	3.302	WIBQEF	152.348	3.466	WIBQEF	158.264	3.131	WIBQEF	152.271	3.23	WIBQEF	161.242	3.293
WIBQEF	158.729	3.126	WIBQEF	153.221	3.339	WIBQEF	164.023	2.924	WIBQEF	165.734	3.196	WIBQEF	162.959	2.868
WIBXUB	160.016	3.373	WIBYAI	159.76	3.384	WIBQEF	162.08	2.815	WIBQEF	155.788	2.864	WIBSUW	155.748	3.033
WIBZIT	163.25	3.309	WIBZIT	156.223	3.274	WIBYAI	164.195	2.896	WIBYOW	160.859	3.048	WIBZIT	172.248	3.216
WICKEC	163.948	3.338	WICKEC	170.796	3.381	WICKAY	154.045	3.177	WICKEC	162.205	3.338	WICKEC	173.051	2.853
WICSEJ	150.926	3.103	WIDBOC	155.027	3.043	WICLUT	164.437	3.121	WICLUT	161.393	3.291	WICSEJ	164.422	3.076
WIDDUJ	150.979	3.262	WIDDUJ	151.464	3.117	WIDDUJ	168.164	3.276	WIDDUJ	152.485	3.125	WIDDUJ	151.012	3.271
WIDFOF	156.708	3.134	WIDFOG	151.715	3.252	WIDDUJ	169.219	3.286	WIDFOF	168.633	2.914	WIDFOF	168.571	2.914
WIDMEF	156.306	3.259	WIDNII	156.38	3.48	WIDFUM	156.307	3.181	WIDFUM	158.757	3.147	WIDGAS	157.89	2.93
WIDREJ	153.318	3.224	WIDREJ	153.182	2.961	WIDNII	158.29	3.214	WIDNOQ	172.435	2.652	WIDNOQ	163.863	2.652
WIDSEJ	160.208	2.728	WIDSEJ	168.844	2.759	WIDREK	151.667	3.187	WIDSEJ	164.959	3.011	WIDSEJ	159.161	2.849
WIDSEJ	158.811	2.728	WIDSEJ	166.705	2.759	WIDSEJ	169.221	3.283	WIDSEJ	175.62	2.943	WIDSEJ	169.962	3.286
WIDSEJ	168.761	3.481	WIDSEJ	155.463	2.811	WIDSEJ	158.538	3.231	WIDSEJ	156.256	3.034	WIDSEJ	168.984	3.256
WIDSIN	171.299	2.929	WIDSIN	152.904	2.612	WIDSIN	178.089	2.929	WIDSIN	152.189	3.232	WIDSIN	153.386	2.612
WIDSOT	170.293	3.042	WIDSOT	160.72	3.082	WIDSOT	177.705	2.764	WIDSOT	177.272	3.063	WIDSOT	156.134	3.184
WIDSUZ	158.055	3.399	WIDSUZ	165.176	3.11	WIDSOT	170.354	2.793	WIDSUZ	171.091	3.12	WIDSUZ	154.837	2.788
WIDXEQ	154.563	2.835	WIDXEQ	162.124	3.103	WIDVUE	168.307	2.83	WIDVUE	157.884	2.753	WIDWAL	167.378	2.727
WIFBUJ	153.461	3.111	WIFBUJ	155.399	2.801	WIDXOA	152.428	3.296	WIDXOA	153.664	2.848	WIDZEO	170.07	3.19
WIFFIE	169.79	2.983	WIFGUO	162.29	2.986	WIFBUJ	156.251	2.861	WIFBUJ	151.388	2.984	WIFFIE	166.107	2.918
WIFPEI	155.731	2.943	WIFQIN	169.492	2.715	WIFHIC	157.034	3.156	WIFHIC	159.786	3.122	WIFHIC	177.308	3.128
WIFVEP	151.637	3.407	WIGLEG	151.359	3.273	WIFTAG	174.123	3.026	WIFTAG	159.114	3.006	WIFTAG	152.527	3.239
WIGPUZ	158.791	2.679	WIGPUZ	157.242	2.679	WIGLEG	152.186	3.401	WIGLEG	156.599	2.781	WIGPUZ	150.166	2.79
WIGPUZ	169.327	3.323	WIGPUZ	162.348	2.61	WIGPUZ	151.081	2.771	WIGPUZ	165.107	2.61	WIGPUZ	150.6	2.771
WIGRUC	158.728	3.08	WIGWIW	160.005	2.821	WIGQUB	158.88	3.247	WIGQUB	165.804	3.002	WIGQUB	171.374	3.325
WIHCUP	150.324	3.167	WIHHAW	151.875	3.252	WIGXER	167.931	3.032	WIGYOE	154.372	3.061	WIHBAS0	171.997	3.409
WIJFII	162.362	2.776	WIJGUV	169.539	3.169	WIJBOK	177.016	3.008	WIJDOK	161.343	3.485	WIJFII	176.137	3.031
WIJJAA	168.453	2.868	WIJJAA	155.838	3.303	WIJJAA	163.12	3.452	WIJJAA	167.443	3.303	WIJJAA	162.645	3.452
WIJMIP	172.925	3.023	WIJMIP	166.401	2.868	WIJKUY	165.421	3.465	WIJLUZ	167.135	3.22	WIJMIP	150.081	3.007
WIJPUC	164.652	3.034	WIJPUC	161.168	2.996	WIJPAI	153.245	3.074	WIJPAI	152.618	3.221	WIJPAI	153.26	2.928
WIJQEN	152.443	3.128	WIJQEN	163.698	2.96	WIJPUC	165.982	2.941	WIJPUC	150.507	3.361	WIJPUC	176.355	2.749
WIJQIR	161.615	2.932	WIJQIR	151.066	2.902	WIJQEN	159.957	2.965	WIJQEN	169.443	2.96	WIJQIR	156.067	2.887

WIJQIR	169.677	3.348	WIJQIR	158.254	2.774	WIJQIR	163.268	2.828	WIJQIR	166.379	3.234	WIJQIR	158.677	3.146
WIJXEV	173.801	3.066	WIJXEV	154.09	3.377	WIJQIR	171.755	3.312	WIJXEV	166.999	3.11	WIJXEV	158.726	3.375
WIKPAL	171.704	3.419	WIKVUI01	150.561	3.423	WIKBAV	157.374	2.795	WIKBAV	164.345	3.121	WIKBAV	158.919	2.775
WIKVUI01	150.648	3.421	WIKVUI01	155.205	3.471	WIKVUI01	155.212	3.479	WIKVUI01	165.433	2.77	WIKVUI01	154.981	3.164
WILCOL	164.218	3.492	WILCOL	168.71	3.285	WIKVUI01	165.619	2.77	WIKVUI01	154.942	3.167	WIKXIA	153.886	3.05
WILCOL	158.561	3.162	WILCOL	172.396	3.125	WILCOL	150.437	3.231	WILCOL	165.778	2.835	WILCOL	174.446	2.953
WILFED	162.234	3.38	WILGII	153.116	3.193	WILCOL	156.734	3.162	WILCOL	152.61	3.087	WILCOL	164.013	3.346
WILJOS	164.595	3.254	WILJOS	159.191	3.397	WILGII	167.574	3.268	WILGII	156.969	3.242	WILGII	156.852	3.268
WILLAH	164.744	2.945	WILLOV	167.876	3.349	WILJUY	161.283	3.325	WILJUY	162.482	3.251	WILJUY	150.149	3.367
WILVUI	152.861	3.466	WILWET	152.899	3.205	WILQAJ	164.632	3.465	WILTUI	170.113	2.891	WILTUI	156.988	3.108
WIMGAE0	173.626	3.032	WIMGAE0	174.84	3.118	WILXOH	173.347	3.299	WILXOI	154.207	3.372	WIMGAE0	175.031	3.156
WIMKOW	170.724	2.81	WIMKUC	166.886	3.189	WIMGAE0	156.155	3.274	WIMGAE0	152.536	3.344	WIMGAE0	158.279	3.389
WIMYOH0	159.633	2.701	WINMUB	154.192	3.255	WIMQEQ	174.753	2.683	WIMYOH	159.687	2.704	WIMYOH0	159.605	2.702
WINMUB	155.148	3.076	WINMUB	162.527	3.209	WINMUB	163.246	2.909	WINMUB	151.247	2.861	WINMUB	153.492	3.252
WINTUK	161.123	2.921	WINTUK	157.038	3.089	WINMUB	152.01	3.488	WINSES	150.039	2.937	WINTUK	151.204	3.156
WINTUK	172.365	2.963	WINZIF	160.474	2.88	WINTUK	153.676	3.012	WINTUK	151.59	2.895	WINTUK	151.835	2.963
WINZOL	151.68	3.462	WINZOL	164.403	3.034	WINZIF	161.654	2.911	WINZIF	159.334	3.191	WINZIF	157.24	3.195
WIPRIY	172.315	2.962	WIQKIQ	151.566	3.299	WINZUR	151.009	3.312	WINZUR	154.87	3.234	WIPCUX	152.709	3.029
WIQLUH	159.917	3.141	WIQMUE	170.369	3.226	WIQKIU	152.049	2.621	WIQKIU	156.968	2.621	WIQLUG	163.19	3.1
WIQNOD	151.327	2.848	WIQNOD	151.988	2.721	WIQNIX	160.534	3.263	WIQNIX	169.242	2.873	WIQNOD	159.627	2.721
WIQNUF	153.407	2.882	WIQNUF	156.102	2.956	WIQNUF	150.902	2.923	WIQNUF	151.982	2.814	WIQNUF	168.73	3.118
WIQWAY	155.233	3.398	WIQZIF01	162.77	3.416	WIQPOF	163.643	2.74	WIQPOF	156.992	2.74	WIQPOF	151.166	2.825
WIQZIF01	167.714	3.031	WIQZIF01	155.934	3.247	WIQZIF01	166.732	3.238	WIQZIF01	159.33	3.398	WIQZIF01	156.994	2.814
WIRDIN	155.78	3.073	WIRDIN	151.441	3.285	WIQZIF01	162.749	3.48	WIQZIF01	150.661	3.031	WIQZIF01	152.201	3.398
WIRDIN	155.625	2.802	WIRDIN	157.382	2.874	WIRDIN	166.073	2.874	WIRDIN	168.303	3.481	WIRDIN	150.435	3.135
WIRNIY	154.631	2.839	WIRNOC	158.272	3.177	WIRJEQ	156.528	3.391	WIRMIX	162.072	3.32	WIRNEU	167.13	3.147
WIRPOG	157.302	2.842	WIRTEW	156.745	3.124	WIRNOC	162.579	3.215	WIRPOG	158.936	3.06	WIRPOG	156.064	2.824
WIRXAY	156.626	3.308	WIRXAY	171.043	2.77	WIRVAX	166.98	3.082	WIRVAX	163.165	3.472	WIRVIF	171.527	3.406
WIRXEC	169.535	2.784	WIRXEC	163.62	3.384	WIRXAY	157.058	2.855	WIRXAY	168.522	2.951	WIRXAY	151.398	3.417
WIRXEC	153.724	3.351	WIRXEC	168.352	2.537	WIRXEC	169.66	3.374	WIRXEC	167.633	3.129	WIRXEC	172.007	3.366
WIRXEC	151.324	2.954	WIRXIG	157.325	3.023	WIRXEC	152.382	3.067	WIRXEC	155.493	2.979	WIRXEC	173.415	3.012
WIRXIG	156.828	3.198	WIRXIG	171.051	3.241	WIRXIG	154.427	3.198	WIRXIG	174.14	2.805	WIRXIG	163.328	3.341
WIRXOM	178.297	2.807	WIRXOM	160.79	2.931	WIRXIG	157.171	2.733	WIRXOM	165.214	3.305	WIRXOM	154.746	3.317
WIRXOM	160.302	3.191	WIRXOM	178.573	3.179	WIRXOM	165.378	3.064	WIRXOM	162.707	3.242	WIRXOM	153.043	2.974
WIRXUS	178.734	2.77	WIRXUS	150.957	3.187	WIRXOM	161.505	3.287	WIRXOM	171.554	2.729	WIRXUS	168.326	2.78
WIRXUS	161.691	2.846	WIRXUS	161.808	2.736	WIRXUS	150.077	3.116	WIRXUS	164.019	3.187	WIRXUS	150.716	2.835
WIRYED	161.27	2.876	WIRYIH	154.054	2.829	WIRXUS	152.322	2.859	WIRXUS	158.486	3.208	WIRYED	166.269	3.035
WIRYIH	152.334	2.895	WIRYIH	171.39	2.894	WIRYIH	172.133	3.495	WIRYIH	150.423	3.097	WIRYIH	166.153	2.952
WIRYIH	157.932	3.059	WISBOR0	151.695	3.499	WIRYIH	159.345	3.138	WIRYIH	150.449	3.434	WIRYIH	162.434	2.952
WISMEU	150.58	2.955	WISREZ	159.67	2.937	WISDUA	156.643	3.051	WISDUA	151.698	3.284	WISMEU	156.444	3.149
WISREZ	156.731	2.986	WISRUL	158.725	3.349	WISREZ	166.043	2.801	WISREZ	171.004	2.892	WISREZ	153.998	2.989
WISRUL03	160.776	2.963	WISRUL03	162.454	2.799	WISRUL	168.031	3.06	WISRUL	172.255	3.157	WISRUL03	164.052	2.785
WITJES	159.671	2.901	WITJES	156.175	3.35	WISSAS	163.733	3.125	WISWAY	150.086	3.292	WISXOO	160.594	2.89
WITKOD	166.668	2.973	WITKOD	152.366	3.392	WITKIX	171.28	2.889	WITKOD	159.94	3.179	WITKOD	161.479	3.44
WITWAB	171.348	3.053	WITWAB	159.125	2.926	WITSUP	162.844	3.301	WITVUU	161.811	3.467	WITWAB	165.969	3.185
WITWAB	171.385	3.132	WITWEF	153.297	3.272	WITWAB	153.205	3.498	WITWAB	157.458	3.188	WITWAB	151.495	3.338
WIVNEV01	152.715	3.473	WIVPIB	160.758	2.881	WITWEF	153.923	2.784	WITWEF	172.668	3.27	WITWEF	151.881	2.928
WIWCOX0	156.685	3.15	WIWCOX0	156.578	3.173	WIVQEX	156.183	3.293	WIVQEX	155.348	2.96	WIVTAA	163.701	3.026
WIWJER	163.752	2.726	WIWXUX	162.235	3.096	WIWCOX0	169.818	3.471	WIWJAQ	157.846	3.115	WIWJAQ	154.862	3.169
WIWXUX	172.236	3.234	WIWXUX	167.604	3.275	WIWXUX	168.863	2.968	WIWXUX	152.061	3.37	WIWXUX	168.167	3.09
WIXLEW	152.809	3.385	WIXSED	163.57	3.107	WIWXUX	168.793	3.284	WIWYUZ	172.079	2.742	WIXJAR	151.29	2.737
WIYCEQ	169.133	3.134	WIYCEQ	158.468	3.27	WIYBIT	162.114	3.389	WIYBIT01	162.604	3.431	WIYCEQ	150.232	3.053
WIYLAS	175.642	2.985	WIYLUM	173.186	2.957	WIYCEQ	151.211	3.424	WIYCEQ	152.086	2.894	WIYHIY	153.212	2.744
WIYRIF	158.344	3.419	WIYSUW	150.956	3.123	WIYLUM	159.724	3.01	WIYNUR	165.923	3.174	WIYNUR	165.303	3.359
WIZMAU	164.062	2.818	WIZNOM	163.366	3.194	WIYYAE	153.035	2.951	WIZJEX	157.552	3.352	WIZMAU	163.723	2.944
WIZPAW0	154.854	3.039	WIZPAW0	171.236	3.11	WIZPAW	171.17	3.122	WIZPAW	155.094	3.056	WIZPAW0	171.285	3.1
WIZPAW0	171.112	3.132	WIZPAW0	155.7	3.068	WIZPAW0	155.111	3.047	WIZPAW0	171.183	3.12	WIZPAW0	155.33	3.058
WIZPAW0	156.526	3.088	WIZPAW0	170.977	3.164	WIZPAW0	171.033	3.144	WIZPAW0	156.091	3.078	WIZPAW0	170.981	3.159
WIZPAW1	170.905	3.249	WIZPAW1	158.575	3.165	WIZPAW0	156.534	3.093	WIZPAW0	170.9	3.205	WIZPAW0	157.617	3.127
WIZPAW1	161.114	3.314	WIZRUT	157.804	3.18	WIZPAW1	171.423	3.354	WIZPAW1	160.713	3.281	WIZPAW1	171.215	3.377
WOBNAG	161.198	3.15	WOBNAG0	160.122	3.239	WOBBIY	156.688	2.824	WOBGIF	153.789	3.198	WOBMEI	173.719	3.093
WOBNAG0	163.01	3.066	WOBNAG0	163.32	3.023	WOBNAG0	159.926	3.239	WOBNAG0	160.712	3.199	WOBNAG0	161.36	3.14
WOBNAG	165.528	2.881	WOBNAG	166.901	2.874	WOBNAG0	164.006	2.972	WOBNAG0	164.529	2.943	WOBNAG0	165.179	2.901
WOBPEM	153.708	3.171	WOBPEM	175.091	2.779	WOBNAG	165.378	2.879	WOBNAG	162.725	3.01	WOBNAG	159.893	3.235
WOBRAG	164.778	3.026	WOBRAI	173.749	2.881	WOBPEM	172.7	2.779	WOBPOW	173.094	3.481	WOBQAJ	161.873	2.625
WOCCAS0	156.877	3.228	WOCNIN	151.871	2.994	WOBTOA	157.618	2.997	WOBTOZ	151.959	3.46	WOCCAS	157.155	3.232
WOCQAJ	175.487	3.058	WOCQAJ	159.78	3.125	WOCQAJ	156.995	3.229	WOCQAJ	164.688	3.177	WOCQAJ	172.976	3.106
WOCQAJ	168.509	2.866	WOCQAJ	167.098	3.351	WOCQAJ	156.31	3.235	WOCQAJ	164.05	3.106	WOCQAJ	159.62	3.01
WOCQAJ	152.449	2.936	WOCQAJ	167.099	2.866	WOCQAJ	150.55	3.228	WOCQAJ	165.77	3.052	WOCQAJ	167.881	3.068
WOCYAP	169.754	3.42	WODGOO	160.448	3.462	WOCYAP	172.249	3.402	WOCYAP	159.933	3.072	WOCYAP	163.159	3.072
WODGOO	156.936	2.931	WODGOO	151.779	3.054	WODGOO	155.803	3.258	WODGOO	162.527	2.962	WODGOO	167.118	3.251
WODGUU	160.828	3.489	WODGUU	172.943	3.074	WODGOO	165.723	2.903	WODGOO	157.574	3.21	WODGUU	171.132	2.995
WODGUU	175.615	2.886	WODGUU	159.532	3.261	WODGUU	152.845	3.034	WODGUU	150.274	3.097	WODGUU	152.059	3.19
WODGUU	167.636	3.122	WODGUU	150.988	2.96	WODGUU	170.93	3.274	WODGUU	154.806	3.169	WODGUU	156.39	3.448
WODYEV	167.002	3.019	WODYEV	167.045	3.269	WODNEJ	151.262	2.94	WODNEJ	159.932	3.199	WODNEJ	167.219	2.94
WODYEV0	164.166	3.019	WODZAR	157.004	3.078	WODYEV	169.695	3.269	WODYEV0	166.152	3.278	WODYEV0	169.827	3.278
WODZIZ	164.428	3.04	WODZIZ	155.907	2.992	WODZAR	159.45	2.866	WODZAR	169.709	2.866	WODZAR	151.31	3.261
WOFJUW	168.059	2.8	WOFJUW	166.466	3.088	WODZIZ	165.579	2.879	WODZIZ	163.486	3.209	WODZIZ	155.351	2.963
WOFLOW	158.197	2.557	WOFTOB	156.979	3.416	WOFJUW	150.977	3.077	WOFJUW	150.939	3.04	WOFJUW	152.899	2.956

WOGNEN	151.084	3.144	WOGNEN	158.834	3.274	WOGCEA	162.472	2.752	WOGNEN	172.508	3.297	WOGNEN	154.838	3.232
WOGNEN	160.769	3.038	WOGNEN	157.634	3.15	WOGNEN	163.059	2.715	WOGNEN	171.771	3.361	WOGNEN	172.233	3.339
WOGNEN	156.371	3.4	WOGNEN	179.586	2.979	WOGNEN	167.204	3.305	WOGNEN	160.467	3.038	WOGNEN	174.228	2.692
WOHFAD	154.911	3.219	WOHFAD	168.23	3.286	WOGNEN	152.038	2.779	WOGNEN	155.308	3.035	WOHFAD	170.391	3.259
WOHKAI	171.825	3.276	WOHLOX	164.501	3.218	WOHFAD	164.995	2.657	WOHFAD	152.812	3.439	WOHJUB	156.827	3.391
WOJDUW	162.678	2.778	WOJFAG	157.165	3.262	WOHNEQ	156.996	3.245	WOHNIQ	150.642	3.284	WOJBOO	161.231	3.414
WOJLIQ	168.174	3.084	WOJLOW	164.975	2.861	WOJFAG0	157.364	3.258	WOJFAG0	157.223	3.243	WOJHAF	160.165	3.171
WOJTOF	160.587	2.839	WOJTOF	152.317	2.839	WOJNEO	160.796	2.778	WOJNOA	175.652	3.158	WOJNOA	153.693	3.232
WOKBAB	155.89	2.977	WOKCII	169.078	3.231	WOJVAW	172.759	3.345	WOJYAV	151.993	2.839	WOJYAV	151.343	2.839
WOKLIT	157.032	2.986	WOKLIT	155.33	3.249	WOKCII01	168.594	3.229	WOKDAC	152.571	3.07	WOKDAC	155.76	3.47
WOKLIT	150.174	3.163	WOKLIT	166.512	3.281	WOKLIT	152.89	3.332	WOKLIT	159.28	3.191	WOKLIT	158.493	2.871
WOKLIT	162.747	3.251	WOKLIT	171.744	2.906	WOKLIT	156.276	2.976	WOKLIT	167.756	3.045	WOKLIT	150.776	3.12
WOKLIT	167.301	2.803	WOKLIT	153.459	2.847	WOKLIT	159.536	3.263	WOKLIT	156.564	2.952	WOKLIT	170.026	2.792
WOKLIT	158.573	2.804	WOKLIT	165.653	3.372	WOKLIT	157.809	2.891	WOKLIT	162.533	3.01	WOKLIT	157.166	2.793
WOKLIT	161.044	2.967	WOKLIT	151.639	3.402	WOKLIT	158.288	3.191	WOKLIT	150.414	3.161	WOKLIT	153.743	2.955
WOKLIT	150.812	2.903	WOKLIT	158.855	2.813	WOKLIT	156.367	2.792	WOKLIT	155.688	2.803	WOKLIT	161.025	3.076
WOKLIT	151.775	3.331	WOKLIT	150.285	3.135	WOKLIT	154.173	3.463	WOKLIT	172.044	3.051	WOKLIT	153.416	3.259
WOKLIT	155.999	3.331	WOKMIT	178.923	3.144	WOKLIT	163.736	3.392	WOKLIT	161.068	3.421	WOKLIT	166.593	2.983
WOLHEN	157.264	3.066	WOLLEP	154.888	2.993	WOKMIT	156.492	2.978	WOLCEJ	157.27	3.445	WOLCEJ	170.929	3.007
WOLPIZ	159.452	3.213	WOLPIZ01	159.324	3.198	WOLLEP	154.301	3.456	WOLLEP	162.686	2.901	WOLLEP	153.671	2.901
WOLPUL	166.086	3.095	WOLXUR	163.849	3.18	WOLPOF	163.806	3.221	WOLPOF0	163.597	3.212	WOLPUL	154.974	2.96
WOMGOV	169.451	3.03	WOMGOV	152.668	3.465	WOMCAF	158.823	3.243	WOMGEM	171.359	2.95	WOMGOV	153.467	3.137
WOMLOD	150.662	3.375	WOMZUW	150.337	3.232	WOMGOV	161.53	2.934	WOMLOD	164.394	3.167	WOMLOD	163.817	3.276
WONDIO	155.405	2.99	WONDIO	159.412	3.356	WOMZUW	157.144	3.31	WOMZUW	154.331	3.142	WONDIO	158.885	3.283
WONDIO	164.403	3.324	WONFEK	164.515	3.18	WONDIO	154.197	2.87	WONDIO	153.229	3.186	WONDIO	171.641	3.162
WONJUI	153.945	3.118	WONJUI	170.804	3.261	WONGAH	155.638	2.981	WONJUI	164.193	3.327	WONJUI	169.598	3.001
WOPFOZ	167.991	3.149	WOPFOZ	165.854	3.355	WONNUM	177.162	3.264	WONYEE	153.651	3.237	WOPFOZ	176.748	3.349
WOPKEU	167.554	2.91	WOPKEU	165.618	2.91	WOPGAJ	173.386	2.916	WOPGEP	161.639	3.326	WOPGEP	151.71	3.416
WOPLAR	152.46	2.999	WOPMIX	160.889	3.474	WOPKEU	173.854	2.888	WOPKEU	176.018	2.888	WOPLAR	153.511	2.849
WOPQIE	173.693	3.162	WOPQIE	153.291	3.243	WOPPEZ	153.399	3.492	WOPPEZ	153.079	3.301	WOPPEZ	156.304	3.324
WOQFAL	153.188	2.973	WOQFAL	165.552	2.999	WOPRIF	173.792	3.162	WOPRIF	153.787	3.253	WOPTOL	156.836	3.257
WOQGES	152.359	2.888	WOQGES	163.887	3.357	WOQFAL0	154.031	3.497	WOQFAL0	152.796	2.915	WOQFAL0	165.496	2.934
WORJOC	154.664	3.12	WORKAP	168.575	3.169	WOQRED	169.236	3.346	WOQRED	165.789	3.353	WORJOC	163.114	3.467
WORKUN	164.226	3.129	WORPAU	156.623	3.362	WORKAP	159.562	3.229	WORKAP	154.732	3.381	WORKEX	173.008	3.48
WORWAF	164.346	3.024	WORZUA	166.567	2.724	WORPAU	151.408	3.128	WORPAU	166.803	3.47	WORPAU	153.017	3.428
WOSSUS	153.045	3.373	WOSSUS	167.81	3.374	WOSFAL	158.211	3.202	WOSFAL	168.44	3.311	WOSFAL	166.885	3.311
WOTKOF	157.049	3.125	WOTKOF	158.314	2.925	WOSTUT	156.167	3.355	WOTDOB	156.222	3.172	WOTDOB	150.526	3.243
WOVGAP	158.314	2.967	WOVHIY	156.531	3.175	WOTSOR	159.615	3.44	WOVCUJ	153.377	3.082	WOVGAP	151.97	3.398
WOVYIP	152.548	2.915	WOVYIP	154.1	3.362	WOVLAW	162.458	2.991	WOVLAW	160.299	2.991	WOVNON	159.099	3.02
WOVYIP0	151.818	2.816	WOVYIP0	172.208	3.421	WOVYIP	151.076	3.491	WOVYIP	151.491	2.936	WOVYIP0	154.439	3.383
WOVYUF	155.87	3.046	WOVYUF	165.826	2.748	WOVYIP0	154.814	3.282	WOVYUF	151.549	2.972	WOVYUF	167.026	2.882
WOWPAB	164.339	3.432	WOWWO	176.63	3.145	WOVYUF	155.084	3.119	WOWKEB	173.894	2.885	WOWKEB	164.768	2.921
WOWZOA	153.988	3.046	WOWZOA	166.019	3.461	WOWWO	150.482	3.102	WOWWUI	161.637	3.43	WOWYUC	154.474	3.426
WOWZOA	152.174	2.962	WOWZOA	166.693	3.223	WOWZOA	162.043	2.91	WOWZOA	162.478	3.267	WOWZOA	166.708	3.028
WOXKUS	167.211	3.372	WOXKUS	153.294	3.304	WOWZOA	155.242	2.97	WOXBES	155.496	2.699	WOXBES	162.976	2.699
WOXLUS0	151.763	3.327	WOXLUS0	173.027	3.491	WOXKUS	170.871	3.27	WOXKUS	171.998	3.461	WOXLUS0	161.35	3.305
WOXPAC	163.277	2.811	WOXPAC	156.56	3.328	WOXLUT	154.366	2.92	WOXMON	152.68	3.219	WOXPAC	173.479	3.198
WOXPAC	157.236	3.278	WOXPAC	157.178	2.895	WOXPAC	170.066	3.182	WOXPAC	154.664	2.859	WOXPAC	164.251	2.824
WOXXUC0	170.834	2.832	WOXXUC0	158.129	2.819	WOXPAC	152.421	2.895	WOXPOQ	173.534	2.711	WOXXUC0	160.904	3.498
WOYCUI0	155.77	3.261	WOYCUI0	167.973	3.181	WOXXUC0	160.467	3.374	WOYBAR	173.772	3.018	WOYBAR	153.816	3.484
WOYHOL	153.986	2.954	WOYQOT	165.634	2.987	WOYDUM	165.661	2.668	WOYHOL	167.983	3.409	WOYHOL	160.108	3.076
WOYXAN	159.003	3.266	WOZBIA	162.186	3.024	WOYSEL	164.443	3.229	WOYXAN	166.895	3.476	WOYXAN	153.293	3.155
WOZCAT	152.208	2.984	WOZCAT	158.458	3.27	WOZBIA	152.974	2.768	WOZBIA	158.183	2.665	WOZBIA	155.229	3.373
WOZCAT	177.946	3.483	WOZCAT	171.077	3.117	WOZCAT	165.707	2.975	WOZCAT	150.601	3.4	WOZCAT	165.705	3.221
WOZCAT	153.28	2.8	WOZCAT	160.146	2.734	WOZCAT	164.428	3.497	WOZCAT	168.446	2.633	WOZCAT	155.588	3.029
WOZLOQ	154.42	2.802	WOZVOW	151.603	3.433	WOZCAT	167.472	2.633	WOZCAT	165.7	2.8	WOZGOJ	174.723	3.252
WUBFUX	162.119	3.344	WUBGEI	170.932	3.059	WOZVOW	156.293	2.896	WOZXOB	156.749	2.922	WUBFUX	160.312	3.268
WUBMOY	152.889	3.02	WUBMOY	163.727	3.25	WUBMOY	160.788	3.071	WUBMOY	165.816	3.467	WUBMOY	150.314	3.031
WUBNAL	167.823	3.339	WUBNAL	152.133	3.474	WUBMOY	176.471	3.467	WUBNAL	157.32	3.426	WUBNAL	154.07	3.474
WUCCAB	162.401	3.44	WUCTEX	158.208	3.228	WUBROA	171.957	3.258	WUBROA	156.564	2.864	WUBROA	172.864	3.183
WUCVEX	152.553	2.811	WUCVEX	155.441	2.939	WUCTEX	152.159	3.311	WUCVEX	152.027	3.483	WUCVEX	175.358	2.995
WUCWAT	160.528	3.041	WUDDEH	160.614	3.302	WUCVEX	167.893	3.251	WUCVUM	152.427	3.499	WUCVUM	154.613	3.39
WUDPUH0	160.472	3.28	WUDPUH0	162.169	3.269	WUDDEH	152.653	3.188	WUDDEH	176.418	2.92	WUDPUH0	158.428	3.331
WUDYUT0	150.718	3.258	WUFDIN	155.052	3.261	WUDPUH0	167.897	3.309	WUDPUH0	167.941	3.294	WUDYUT	150.881	3.168
WUFYIH	155.973	2.979	WUFYIH	153.646	3.019	WUFDIN	163.041	3.196	WUFYED	158.844	3.331	WUFYED	150.859	3.215
WUGLER	154.533	3.102	WUGLER	152.41	3.137	WUFZEC	156.361	3.08	WUGKUH	157.883	3.29	WUGKUH	158.074	3.195
WUGXOL	162.657	3.407	WUGYAY	165.293	2.85	WUGXOL	155.761	2.771	WUGXOL	160.498	3.366	WUGXOL	159.531	3.209
WUGYAY	164.037	2.777	WUGYIG	168.714	3.149	WUGYAY	167.156	3.08	WUGYAY	157.246	2.777	WUGYAY	159.253	2.937
WUGYIG	174.14	3.331	WUGZED	152.43	3.454	WUGYIG	163.483	2.84	WUGYIG	155.84	3.022	WUGYIG	157.131	3.331
WUGZON	161.442	3.001	WUHCUY	153.133	3.407	WUGZED	164.98	3.132	WUGZON	157.319	2.72	WUGZON	150.15	2.72
WUHRAW	163.739	3.272	WUHRAW	172.08	3.035	WUHKIU	151.182	3.266	WUHKIU	150.947	2.939	WUHQIB	158.658	2.755
WUHROK	161.086	2.932	WUHROK	172.795	2.725	WUHRAW	171.883	3.076	WUHRAW	163.174	3.327	WUHROK	158.91	2.957
WUHSOJ	153.622	3.063	WUHSOJ	162.826	3.063	WUHSOJ	170.722	2.94	WUHSOJ	165.366	2.962	WUHSOJ	161.413	2.744
WUHSOJ	151.851	3.424	WUHSOJ	153.397	2.89	WUHSOJ	151.17	3.46	WUHSOJ	151.78	2.768	WUHSOJ	173.211	2.744
WUHSOK	166.768	2.846	WUHWAB	156.501	3.206	WUHSOJ	158.84	3.075	WUHSOJ	163.515	3.282	WUHSOJ	151.346	3.425
WUHWUU	163.577	2.99	WUHWUU	162.517	3.051	WUHWAB	157.208	3.279	WUHWUU	166.822	3.143	WUHWUU	158.077	3.246
WUHWUU	163.959	2.991	WUHWUU	162.661	3.06	WUHWUU	174.584	3.062	WUHWUU	166.757	3.155	WUHWUU	158.419	3.256
WUJHAM	151.146	2.862	WUJHIU	154.803	3.157	WUHWUU	174.848	3.063	WUJCOV0	150.669	3.048	WUJCOV0	157.349	3.44
WUJPEY	159.236	3.232	WUKBOT	159.838	2.647	WUJKIX	157.273	3.121	WUJKIX	168.096	3.06	WUJNOG	159.382	3.236

WUKMUN	168.653	3.1	WUKTAZ	158.526	3.152	WUKBOT	156.172	2.647	WUKDIS	168.204	3.285	WUKMIA	162.172	2.81
WUKTED	157.011	2.846	WUKTIH	157.595	3.293	WUKTAZ	158.719	3.132	WUKTED	169.128	3.132	WUKTED	160.108	2.932
WUKTON	168.933	2.966	WULBAJ	158.471	3.126	WUKTIH	150.295	3.236	WUKTON	168.092	3.368	WUKTON	167.443	2.685
WULHAN	154.303	3.337	WULHIV	161.391	3.079	WULGUI	153.175	3.197	WULHAN	157.943	3.103	WULHAN	153.528	3.198
WULPUR	163.091	3.498	WULPUR	150.845	2.779	WULKEW	153.003	2.9	WULPOJ	157.883	2.822	WULPOJ	155.465	2.862
WULSEB	164.272	2.836	WULSIF	163.284	2.881	WULROK	157.237	3.136	WULRON	157.163	2.833	WULSEB	156.835	2.836
WULVIJ	150.38	3.116	WULWAB	150.348	3.302	WULSIF	155.591	2.881	WULSOO	176.072	2.699	WULSUU	151.718	2.732
WUMBUE	160.079	2.923	WUMDAL	155.094	3.374	WULWAB	155.944	3.12	WULWAB	164.692	3.424	WULWAB	150.555	3.12
WUMDEQ	159.067	2.898	WUMDOA	163.396	2.906	WUMDAL	163.115	3.264	WUMDEP	156.328	3.273	WUMDEP	163.268	3.3
WUMFAO	160.414	2.673	WUMFAO	158.973	3.495	WUMDUG	152.705	2.833	WUMFAO	157.374	3.268	WUMFAO	150.941	2.837
WUMFUI	159.588	3.13	WUMFUI	152.119	3.247	WUMFAO	150.267	3.357	WUMFAO	165.336	2.673	WUMFES	153.227	2.82
WUMFUI	169.617	2.792	WUMHAQ	156.813	3.422	WUMFUI	151.861	3.247	WUMFUI	154.628	2.925	WUMFUI	152.867	3.19
WUMHAQ	168.882	3.467	WUMHAQ	164.495	3.055	WUMHAQ	150.268	3.157	WUMHAQ	150.909	3.43	WUMHAQ	150.362	2.872
WUMHIZ	161.538	3.411	WUMJEU	159.332	2.826	WUMHIY	169.713	3.229	WUMHIY	153.407	3.385	WUMHIY	153.658	3.379
WUMLAV	165.894	2.913	WUMLAV	151.279	3.415	WUMLAV	156.623	3.4	WUMLAV	151.786	2.889	WUMLAV	153.323	2.878
WUMLUP	153.106	3.036	WUMLUP	173.867	2.791	WUMLAV	161.7	3.428	WUMLUP	178.984	3.036	WUMLUP	166.075	3.176
WUNFAP	154.052	2.981	WUNFET	154.335	2.957	WUMMID	153.193	3.365	WUNDUH	156.146	2.714	WUNFAP	153.284	3.36
WUNFIX	150.782	2.93	WUNFIX	164.945	3.044	WUNFET	154.842	3.418	WUNFIX	154.017	3.121	WUNFIX	152.651	2.583
WUNFOD	159.494	3.323	WUNFOD	160.734	3.437	WUNFIX	154.94	3.17	WUNFIX	165.871	2.834	WUNFOD	152.475	2.602
WUNFUJ	152.687	2.872	WUNFUJ	158.069	2.745	WUNFUJ	152.495	2.877	WUNFUJ	151.077	2.974	WUNFUJ	151.302	3.033
WUNGUK	152.844	3.138	WUNHAR	166.099	3.018	WUNGAQ	151.583	3.254	WUNGAQ	150.232	2.903	WUNGOE	155.604	3.283
WUNKAR	175.582	3.175	WUNKAR	177.481	3.228	WUNHAR	160.726	2.893	WUNHAR	160.794	2.786	WUNHIY	152.365	3.284
WUNKAR	156.783	3.401	WUNKOF	159.612	3.24	WUNKAR	162.961	3.098	WUNKAR	164.04	3.019	WUNKAR	158.293	3.3
WUNXIM	162.2	3.434	WUNXIM	165.077	2.081	WUNKOF	159.781	3.446	WUNRAY	152.324	3.414	WUNXIM	150.138	2.171
WUNXIM	153.022	2.119	WUNXIM	168.058	3.127	WUNXIM	158.539	2.793	WUNXIM	153.998	3.214	WUNXIM	150.997	2.171
WUNYUC	160.181	3.059	WUNYUC	163.721	3.059	WUNXIM	151.535	3.26	WUNXIM	163.521	2.665	WUNXUY	155.917	3.498
WUPCUF	163.36	3.137	WUPCUF	158.518	3.142	WUNYUC	160.852	2.861	WUNYUC	173.069	3.175	WUPCUF	151.274	3.21
WUPNOM	164.194	3.232	WUPNOM	177.977	3.164	WUPCUJ	152.986	3.289	WUPCUJ	178.348	3.057	WUPKEB	156.403	3.398
WUPPIG	159.325	3.064	WUPPIG	164.908	3.428	WUPNOM	168.981	3.232	WUPNOM	162.674	3.068	WUPPAY	164.173	3.292
WUQCOC	166.196	2.911	WUQCOC	167.224	3.079	WUPPIG	164.377	3.36	WUPPIG	160.809	3.018	WUPZAM	170.422	2.975
WUQCOC	151.353	3.446	WUQCOC	156.091	2.801	WUQCOC	175.371	2.661	WUQCOC	165.753	2.818	WUQCOC	160.89	3.098
WUQCOC	152.224	2.792	WUQCOC	160.66	3.171	WUQCOC	157.808	3.01	WUQCOC	154.236	3.014	WUQCOC	157.679	2.785
WUQJAT	162.376	3.233	WUQNUS	152.079	3.179	WUQCOC	154.69	3.367	WUQJAT	164.336	2.877	WUQJAT	153.957	3.009
WUQPAC	150.94	3.288	WUQPAC	170.25	3.003	WUQPAC	160.412	3.053	WUQPAC	159.795	3.173	WUQPAC	159.642	2.892
WUQPAC	151.651	3.333	WUQPAC	156.93	2.955	WUQPAC	167.872	2.772	WUQPAC	150.41	2.834	WUQPAC	159.701	2.96
WUQSOU	155.986	3.196	WURCUK	162.448	3.401	WUQPAC	173.869	3.043	WUQPAC	162.379	3.478	WUQPAC	158.672	3.091
WURDOG	153.301	3.124	WURDOG	151.999	2.829	WURCUK	157.181	2.974	WURDOG	167.962	3.041	WURDOG	177.929	3.114
WURDOG	150.052	3.248	WURHOG	151.678	3.313	WURDOG	156.164	2.85	WURDOG	151.371	3.222	WURDOG	172.931	3.176
WURZAL	152.831	2.803	WURZAL	153.204	3.223	WURMAA	169.242	3.413	WURZAL	154.582	3.468	WURZAL	151.132	3.403
WUSHOJ	152.431	2.875	WUSHOJ	157.514	3.087	WURZAL	152.536	2.866	WURZAL	153.849	3.234	WUSHOJ	164.141	3
WUSJUP	165.039	3.355	WUSJUP	153.484	3.063	WUSHOJ	161.532	2.827	WUSHOJ	159.763	2.769	WUSHOJ	175.735	2.885
WUSLUS	174.976	3.004	WUSMUT	168.225	3.285	WUSLEF	152.878	3.31	WUSLEF	154.888	2.737	WUSLUS	158.31	3.004
WUSNIH	153.236	3.237	WUSNIH	157.713	3.128	WUSNIH	155.904	2.98	WUSNIH	169.23	2.904	WUSNIH	158.715	3.107
WUSQUW	157.141	3.414	WUSQUW	163.936	3.08	WUSNIH	151.369	3.186	WUSNIH	154.463	2.917	WUSNIH	152.121	3.424
WUSWEO	164.2	2.605	WUSWEO	170.926	3.46	WUSREH	158.631	3.399	WUSREH	159.241	2.754	WUSREH	158.908	3.423
WUSWOY	174.574	2.723	WUSXUF	156.415	2.831	WUSWIS	173.802	2.735	WUSWIS	167.097	2.934	WUSWOY	172.829	2.835
WUSXUH	157.518	3.135	WUSYES	168.617	3.471	WUSXUF	154.324	2.865	WUSXUH	150.502	2.822	WUSXUH	150.7	3.219
WUSYES	171.196	2.692	WUSYES	172.936	3.098	WUSYES	175.271	2.857	WUSYES	158.282	2.791	WUSYES	172.835	2.809
WUTBOE	159.227	2.618	WUTBOE	155.202	3.338	WUSYES	161.031	2.839	WUSYIU	156.716	3.391	WUTBOE	152.286	3.065
WUTKUR	158.624	3.042	WUTKUR	159.255	3.179	WUTBOE	165.817	2.698	WUTBOE	153.604	2.618	WUTBOE	152.097	3.04
WUTPUX	153.313	2.721	WUTSUC	150.518	3.367	WUTNOO	168.794	2.659	WUTPOR	156.27	3.19	WUTPUX	169.816	3.452
WUTTEK	156.565	2.836	WUVCUM	175.241	3.491	WUTTEK	166.798	3.479	WUTTEK	169.565	3.226	WUTTEK	163.593	3.069
WUVDOH	172.442	2.911	WUVNAD	158.663	3.442	WUVDAV	164.752	3.299	WUVDAV	154.002	3.438	WUVDOH	155.515	2.855
WUVPOT	151.097	3.113	WUVQEK	175.222	3.061	WUVNAD	159.172	2.875	WUVNAD	156.295	3.211	WUVNAD	172.478	3.236
WUVQEK	155.237	3.058	WUVQEK	169.761	2.968	WUVQEK	159.197	3.245	WUVQEK	158.762	2.688	WUVQEK	153.043	2.919
WUVQEK	158.38	3.15	WUVVIS	172.517	3.179	WUVQEK	167.008	3.169	WUVQEK	166.396	2.706	WUVQEK	161.006	2.706
WUWFEA	158.167	3.311	WUWHAX	154.162	3.036	WUVVIS	169.181	3.136	WUVVIS	174.754	3.011	WUWBAT	156.013	3.455
WUWPUD	160.18	2.811	WUXBAV	153.501	3.447	WUWKOS	167.869	3.41	WUWKOS	165.934	2.985	WUWPIQ	156.078	3.426
WUXNIM	168.612	3.171	WUXNIM	171.192	3.261	WUXBUP	167.11	3.223	WUXHOM	152.664	3.063	WUXNIM	152.122	3.444
WUXPEO	164.357	2.843	WUXPEO	152.144	3.091	WUXNIM0	151.755	3.433	WUXNIM0	168.642	3.16	WUXNIM0	171.443	3.284
WUXZIA	158.322	3.06	WUYBIF	150.972	3.486	WUXPIR	164.151	3.288	WUXQIR	151.255	3.337	WUXSAJ	153.241	3.123
WUYFAX	150.95	3.454	WUYFAX0	150.377	3.421	WUYBIF	150.053	2.861	WUYBIF	173.039	2.827	WUYBIF	168.051	3.26
WUYJAE	158.212	3.317	WUYKIN	160.063	2.997	WUYHOQ	161.327	2.806	WUYJAE	152.67	3.236	WUYJAE	164.472	3.027
WUYTAM	164.761	3.407	WUYTIX	176.893	3.271	WUYLOU	169.866	3.22	WUYREO	167.362	2.986	WUYREO	160.882	3.271
WUZBAY	155.086	3.172	WUZBAY	154.36	3.031	WUYWOG	159.94	3.174	WUZBAY	163.455	3.172	WUZBAY	172.455	3.309
WUZDOK	161.388	3.434	WUZMUZ	154.72	3.159	WUZBAY	162.465	2.923	WUZBAY	160.759	3.081	WUZDOK	163.952	3.078
WUZWOD	150.275	3.219	WUZZID	164.792	3.234	WUZWIX	177.215	3.467	WUZWIX	166.37	3.142	WUZWIX	165.134	3.122
XABBAH	153.863	3.24	XABBOV	159.936	3.014	XABBAH	172.485	2.777	XABBAH	160.509	3.196	XABBAH	152.812	2.865
XABDAH	156.924	3.133	XABDAH	152.196	3.5	XABBOV	159.133	3.278	XABCOW	168.014	3.435	XABDAH	159.141	2.821
XABDEL	151.738	3.016	XABDOV	164.446	2.843	XABDEL	151.071	3.336	XABDEL	150.056	2.884	XABDEL	150.249	3.495
XABHIV	163.245	3.421	XABHIV	153.002	3.21	XABDOV	168.106	3.493	XABDOV	175.124	2.886	XABDUE	168.488	3.269
XABQEB	160.631	3.302	XABQEB	167.679	2.758	XABPUQ	154.311	2.758	XABPUQ	164.103	2.977	XABPUQ	159.892	3.157
XABQEB	150.771	3.33	XABQEB	153.133	3.368	XABQEB	153.393	3.364	XABQEB	153.001	2.935	XABQEB	169.701	3.467
XACLIZ	161.142	3.204	XACLOE	164.452	3.077	XABQEB	164.545	2.993	XABZEG	161.293	2.912	XACFAM	161.342	2.835
XACVEF	165.11	3.129	XACVEF	152.564	2.896	XACSII	162.226	2.823	XACSOM	164.114	3.044	XACVAB	153.019	3.263
XACYAG	167.044	3.343	XACYEK	151.295	3.171	XACVEF	163.012	3.166	XACXEJ	170.633	3.197	XACYAG	163.051	3.343
XACYEK	150.826	3.06	XACYEK	163.503	2.888	XACYEK	158.258	3.194	XACYEK	163.879	3.485	XACYEK	153.095	3.249
XADDAM	162.114	3.174	XADDAM	161.871	3.171	XACYEK	152.951	2.971	XACZUA	153.576	3.319	XADBAJ	158.229	3.214
XADDAM	164.14	3.216	XADDAM	164.622	2.86	XADDAM	175.927	2.976	XADDAM	171.653	3.392	XADDAM	154.612	3.207

XADDOA	165.494	2.698	XADDOA	150.701	3.433	XADDAM	154.102	3.418	XADDAM	164.631	3.176	XADDAM	154.629	2.756
XADDOA	166.441	3.408	XADDOA	154.11	2.83	XADDOA	155.837	2.698	XADDOA	152.209	2.984	XADDOA	152.156	2.94
XAFFEP	156.353	3.258	XAFFEP	169.076	3.059	XADFIW	161.64	3.497	XADSEB	150.061	2.96	XAFFEP	161.634	3.327
XAFJUN	158.681	2.936	XAFKUO	161.525	3.091	XAFFEP	164.988	3.052	XAFFEP	152.576	3.313	XAFFEP	152.409	3.143
XAFLIZ	154.895	3.419	XAFLIZ01	151.83	2.908	XAFLAW	152.734	3.265	XAFLIZ	151.877	2.908	XAFLIZ	153.595	3.446
XAFSIK	160.032	3.201	XAFSUW	162.449	3.324	XAFLIZ01	153.774	3.45	XAFLIZ01	155.036	3.422	XAFSIK	158.021	3.395
XAFWAG	162.275	3.047	XAFYEI	158.792	3.147	XAFVEJ	158.045	3.073	XAFWAG	163.774	3.236	XAFWAG	163.537	3.071
XAFYEI	156.724	3.168	XAFYEI	154.668	3.168	XAFYEI	162.953	3.261	XAFYEI	178.832	3.007	XAFYEI	161.072	2.829
XAGBAI	173.61	2.858	XAGBAI	170.199	2.737	XAFYEI	157.806	3.268	XAFYEI	162.548	3.007	XAGBAI	170.835	2.737
XAGJID	152.773	3.259	XAGQOO	165.078	3.183	XAGBAI	150.516	2.858	XAGJID	167.801	3.064	XAGJID	150.425	2.99
XAGWIP	161.528	3.43	XAGWIP	165.738	3.201	XAGREF	169.12	3.053	XAGREF	169.011	3.291	XAGWIP	161.024	3.034
XAGZIO	160.078	3.186	XAGZIO	176.425	3.356	XAGWIP	162.361	3.15	XAGZIO	171.475	3.387	XAGZIO	151.832	3.468
XAJCAO	156.331	2.892	XAJCAO	157.264	3.29	XAGZIO	152.839	2.749	XAGZIO	161.78	2.866	XAHNIH	157.994	3.368
XAJCAO	156.9	2.877	XAJCAO	161.154	3.192	XAJCAO	173.494	3.27	XAJCAO	164.356	3.47	XAJCAO	162.328	3.45
XAJNUT	162.78	3.17	XAJNUT01	162.402	3.05	XAJCAO	167.459	3.095	XAJCAO	159.46	3.236	XAJNUT	151.554	3.152
XAJXOY	152.581	3.214	XAJYAN	158.476	3.455	XAJNUT01	151.5	3.068	XAJNUT02	166.579	3.051	XAJWEK	150.704	3.367
XAKHIB	165.073	3.11	XAKHIB	150.105	2.962	XAKDAP	169.741	2.752	XAKFUM	170.89	2.989	XAKGAV	150.32	3.148
XAKHOH	177.515	3.222	XAKHOH	163.491	3.106	XAKHIB	157.034	3.08	XAKHIB	160.171	2.924	XAKHIB	158.904	2.793
XAKHOH	151.642	3.426	XAKHOH	151.843	3.369	XAKHOH	160.862	3.388	XAKHOH	171.103	3.493	XAKHOH	166.296	2.719
XAKHOH	159.102	3.304	XAKHOH	151.323	2.866	XAKHOH	159.084	3.159	XAKHOH	152.165	3.303	XAKHOH	159.433	3.47
XAKSAH	169.753	2.841	XAKSAH	154.811	3.28	XAKHOH	157.373	2.824	XAKRIK	159.197	3.027	XAKRIK	165.614	3.231
XALCOE0	160.769	3.327	XALCOE0	158.03	2.832	XAKSAH	175.367	2.908	XALBIY	150.085	3.195	XALCES	152.937	2.834
XALCUK	171.708	2.751	XALCUK	174.746	2.751	XALCOE0	166.442	2.946	XALCUK	156.565	3.001	XALCUK	156.715	3.255
XALVOY	169.346	3.287	XALVUC	160.421	2.907	XALPOP	161.899	2.712	XALPOP	155.784	2.662	XALPOP	176.451	2.662
XALVUC	159.24	3.356	XALVUC	161.177	2.856	XALVUC	164.619	3.026	XALVUC	166.606	3.468	XALVUC	164.178	3.016
XAMCIB	162.947	3.169	XAMDAR	167.468	3.283	XALZIU	168.036	2.754	XALZIU	163.097	3.461	XALZIU	162.107	3.463
XANCOG	163.674	3.381	XANCOG	151.171	3.416	XAMPUW	152.836	3.265	XAMYEP	165.658	3.178	XAMYEP	162.54	3.307
XANXUG	169.711	3.148	XAPBEY	166.158	3.379	XANCOG	166.954	3.498	XANDIA	151.217	2.972	XANXIU	156.133	3.136
XAPCAS	150.786	2.987	XAPCAS	156.91	2.85	XAPCAS	178.535	2.863	XAPCAS	175.205	2.863	XAPCAS	156.734	2.915
XAPMAG	161.838	2.995	XAPMAG	155.322	2.942	XAPKIM	154.207	2.932	XAPMAG	161.182	3.297	XAPMAG	177.142	3.481
XAPMAG	151.604	3.166	XAPMEK	165.861	2.84	XAPMAG	152.909	2.859	XAPMAG	154.986	2.962	XAPMAG	172.419	2.963
XAPNEL	151.587	3.437	XAPNOV	159.94	3.433	XAPMEK	167.704	3.193	XAPMEK	174.325	2.84	XAPNEL	162.509	3.383
XARBAT	152.886	3.304	XARBAT	151.28	3.152	XAQDUQ	153.625	3.305	XAQWUJ	153.924	3.457	XAQXUJ	153.439	3.101
XARBIB	157.659	3.452	XARBIB	150.478	3.141	XARBAT	162.151	3.099	XARBAT	151.31	3.236	XARBIB	150.09	3.017
XARBIB	177.732	3.325	XARDOL	154.716	3.183	XARBIB	175.447	3.402	XARBIB	154.653	3.04	XARBIB	164.087	2.93
XARDOL	156.16	3.487	XARDOL	168.3	2.782	XARDOL	173.234	3.011	XARDOL	151.059	3.175	XARDOL	154.226	3.011
XARDOL	156.846	2.802	XARDOL	153.716	2.966	XARDOL	162.3	2.845	XARDOL	157.631	2.842	XARDOL	158.688	3.026
XARFED	150.889	3.036	XARFED	159.69	3.421	XARDOL	160.02	3.026	XARDOL	154.97	3.323	XARDOL	165.469	3.221
XARFED	167.954	2.973	XARFED	155.115	3.149	XARFED	156.879	2.809	XARFED	153.558	3.289	XARFED	154.756	3.29
XARFED	170.109	3.364	XARFIH	155.252	2.986	XARFED	164.077	3.098	XARFED	153.001	2.905	XARFED	152.7	2.987
XARFIH	164.129	3.379	XARFIH	150.602	3.001	XARFIH	155.503	3.102	XARFIH	154.717	2.99	XARFIH	151.265	2.959
XARFIH	157.103	3.047	XARFIH	153.603	3.146	XARFIH	151.57	2.931	XARFIH	155.336	2.897	XARFIH	158.339	3.274
XARFIH	166.165	3.368	XARFIH	166.891	3.123	XARFIH	155.499	3.436	XARFIH	152.44	3.332	XARFIH	157.465	2.832
XARFIH	156.15	2.819	XARFIH	151.774	2.721	XARFIH	160.278	3.159	XARFIH	156.225	3.094	XARFIH	155.603	3.368
XARFIH	155.631	3.401	XARFON	156.544	2.833	XARFIH	157.281	3.049	XARFIH	161.227	3.436	XARFIH	157.564	3.365
XARFON	156.051	3.259	XARFON	152.512	3.281	XARFON	154.908	3.195	XARFON	151.248	2.951	XARFON	159.044	3.346
XARFON	177.192	3.229	XARFON	154.832	2.951	XARFON	168.88	2.945	XARFON	160.714	3.011	XARFON	153.42	3.237
XARNIP	151.436	2.985	XARNIP	151.708	3.326	XARGAZ	155.57	3.074	XARLAE	161.765	3.476	XARNIP	155.171	2.678
XARNIP	155.444	3.013	XARNIP	159.012	2.791	XARNIP	163.092	2.841	XARNIP	150.53	2.955	XARNIP	151.618	3.495
XARNIP	164.616	2.855	XASHEG	159.55	3.165	XARNIP	162.903	3.349	XARNIP	161.658	3.077	XARNIP	157.845	3.078
XASJUW	159.066	2.956	XATFAD	157.195	3.101	XASJIK	162.433	2.8	XASJIK	155.108	3.14	XASJIK	177.842	2.967
XATMAK	171.952	3.239	XATMAK	153.534	3.226	XATFAD	154.652	2.902	XATFAD	161.464	3.498	XATFAD	166.665	2.738
XATNOZ	159.81	2.724	XATNOZ	153.985	2.724	XATMAK	162.113	2.867	XATMAK	162.158	3.126	XATNOZ	174.342	3.243
XATNOZ	156.445	3.222	XATNOZ	167.349	2.905	XATNOZ	166.706	2.773	XATNOZ	154.608	3.403	XATNOZ	164.074	2.852
XATQIW	153.323	2.961	XATQOA	152.02	3.25	XATPIV	159.465	3.198	XATPIV	165.061	2.645	XATQIW	159.114	2.937
XATRET	157.453	3.158	XATRET	160.92	2.925	XATRAP	150.724	3.26	XATRAP	157.882	2.982	XATRAP	156.922	2.9
XAVCII	153.104	3.16	XAVSAO	165.075	3.285	XATRET	153.777	2.914	XATWUL	167.308	3.287	XAVBUT0	159.863	3.007
XAVTUJ	154.276	3.126	XAWMER	158.783	2.925	XAVSAO	159.75	3.332	XAVTIB	157.964	3.052	XAVTIB	160.009	3.073
XAWMER	150.418	3.219	XAWMER	155.24	2.921	XAWMER	153.948	2.942	XAWMER	160.293	3.213	XAWMER	151.147	2.854
XAWQUL	164.176	3.313	XAWRIX	169.523	3.428	XAWPET	153.817	3.265	XAWPET	156.707	2.886	XAWPIX	161.831	3.389
XAWROD	153.521	3.245	XAWXOJ	173.548	2.775	XAWRIX	152.016	2.93	XAWROD	158.593	3.245	XAWROD	168.692	3.329
XAXDIN	172.002	2.755	XAXDIN	168.968	2.939	XAWXOJ	172.011	2.796	XAWXOJ	156.039	2.921	XAXBOQ	170.322	3.134
XAXDOT	163.805	2.698	XAXDOT	170.654	3.029	XAXDIN	173.292	2.755	XAXDIN	155.32	3.148	XAXDOT	150.654	2.958
XAXDOT	162.522	3.477	XAXDOT	161.1	2.86	XAXDOT	157.536	3.022	XAXDOT	152.484	3.368	XAXDOT	158.792	2.642
XAXDOT	158.912	3.004	XAXDOT	152.653	3.051	XAXDOT	150.484	2.642	XAXDOT	157.269	2.698	XAXDOT	162.365	3.029
XAXDOT	157.761	2.965	XAXDOT	176.002	2.86	XAXDOT	160.044	3.24	XAXDOT	151.109	3.328	XAXDOT	170.141	2.977
XAXFAH	165.04	3.076	XAXFAH	168.871	3.013	XAXDUZ	162.099	3.267	XAXDUZ	165.141	2.786	XAXDUZ	171.709	2.79
XAXFUA	154.915	3.365	XAXMAN	154.048	3.431	XAXFAH	172.505	2.76	XAXFIO	157.47	3.304	XAXFUA	166.085	2.818
XAXMEO0	155.643	3.471	XAXMEO0	154.196	3	XAXMAN	165.496	3.419	XAXMEO	155.778	3.465	XAXMEO	154.356	3.01
XAXWOI0	154.122	3.207	XAXXON	176.097	3.121	XAXQAS0	150.836	3.326	XAXWOI0	161.827	3.369	XAXWOI0	164.345	3.19
XAYJEQ	159.032	3.333	XAYJOA	170.019	2.945	XAYCEH	159.12	2.951	XAYCUX	156.495	2.81	XAYJEQ	159.283	3.207
XAYKAN	153.274	3.154	XAYKAN	160.355	3.34	XAYJOA	150.967	2.911	XAYJOA	152.945	2.891	XAYJUG	157.457	2.961
XAYLOZ	159.367	3.439	XAYLOZ	160.644	2.89	XAYKAN	168.802	2.806	XAYKAN	164.391	2.969	XAYKAN	158.053	3.299
XAYMOB	166.425	3.005	XAYMOB	159.03	3.022	XAYMOB	151.825	3.04	XAYMOB	153.645	3.209	XAYMOB	151.583	3.156
XAYNES	157.573	2.976	XAYNES	155.481	3.365	XAYMOB	164.729	3.092	XAYMUH	150.558	3.166	XAYNAO	152.904	3.327
XAYPUK	156.285	2.984	XAYSIC	169.517	3.203	XAYNES	164.195	2.807	XAYNES	156.703	3.022	XAYNES	153.372	2.944
XAZSIE	159.8	2.856	XAZSIE	162.517	3.079	XAYSIC	167.729	2.933	XAYYIH	160.056	3.22	XAZCUY	157.045	2.936
XEBJOG	158.276	3.398	XEBJOG	165.893	3.119	XAZXOM	150.492	2.921	XAZXOM	150.576	2.908	XEBJIA	161.499	3.489
XEBNAX	155.023	2.774	XEBNAX	162.405	2.994	XEBJOG	160.234	3.404	XEBJOG	165.765	3.093	XEBNAX	165.509	2.696

XEBNOL	165.066	2.74	XEBNOL	171.304	2.737	XEBNAX	152.636	2.994	XEBNAX	163.081	2.774	XEBNAX	165.227	2.965
XECCEQ	151.995	3.397	XECCEQ	158.578	2.708	XEBNOL	165.308	2.74	XEBNUR	152.928	2.929	XEBNUR	169.479	3.19
XECDER	153.676	3.218	XECHET	157.776	3.41	XECCEQ	160.953	2.854	XECDAN	153.329	2.929	XECDER	169.549	2.798
XECPUT	155.072	3.384	XECSII	150.404	2.867	XECHET	151.215	3.341	XECJIB	155.69	3.301	XECJIB	155.152	2.955
XECSII	150.662	3.102	XECSII	156.14	2.858	XECSII	152.945	2.78	XECSII	166.146	3.102	XECSII	150.751	2.867
XECTOO	168.406	3.463	XECTOO	169.641	3.356	XECSII	164.702	2.87	XECSII	166.056	3.067	XECSII	153.713	3.184
XECXUB	171.21	3.109	XECXUB	169.561	3.145	XECTOO	168.261	3.025	XECTOO	159.581	3.315	XECTUU	167.112	3.377
XEDCAN	164.119	2.979	XEDCAN	153.618	2.913	XEDCAN	166.089	2.959	XEDCAN	152.058	2.791	XEDCAN	152.115	3.462
XEDHOF	158.792	3.256	XEDHOF	170.336	3.405	XEDCAN	171.429	2.965	XEDDOA	155.569	3.392	XEDHIB	153.282	2.971
XEDHUL	169.159	3.129	XEDHUM	167.532	3.383	XEDHOF	158.687	2.876	XEDHOF	160.589	3.042	XEDHOF	167.017	2.56
XEDNON	164.44	2.96	XEDQUV	159.605	3.382	XEDHUM	159.986	3.442	XEDHUM	172.348	2.825	XEDHUM	163.927	3.416
XEDYOU	160.894	2.933	XEDYOU	174.983	2.948	XEDWEI	174.613	2.769	XEDXAJ	153.01	3.077	XEDXUD	176.657	2.96
XEDYOU	154.674	3.383	XEDYOU	153.651	2.948	XEDYOU	161.501	2.773	XEDYOU	161.927	2.821	XEDYOU	157.871	2.849
XEFBIW	162.268	2.818	XEFJEY	162.215	3.047	XEDYOU	150.867	3.378	XEDYOU	156.189	3.149	XEFBAO	155.585	3.25
XEFSIN	173.596	3.396	XEFSIN	165.713	2.738	XEFSIN	154.353	2.745	XEFSIN	171.055	3.035	XEFSIN	160.749	3.222
XEFZUE	154.99	3.002	XEGBAN	161.787	3.425	XEFYAJ	170.693	2.86	XEFYAJ	172.05	2.86	XEFYAJ	155.389	3.156
XEGHIE	157.161	2.936	XEGHOK	158.968	3.38	XEGBAN	168.723	3.055	XEGBER	156.286	3.193	XEGHEA	156.749	2.915
XEHGAV	153.026	3.348	XEHKEA	173.723	2.865	XEGPEF	168.343	2.959	XEGPIJ	159.341	2.669	XEGPOP	165.312	3.112
XEHXOZ	153.777	3.223	XEHZUJ	155.688	3.163	XEHKEA	169.365	3.41	XEHSIO	154.959	3.051	XEHTOV	165.44	3.198
XEJLOR	154.891	3.336	XEJLOR	150.478	3.429	XEJCEX	150.096	3.36	XEJLON	160.882	2.979	XEJLOR	154.999	3.359
XEJWAK	164.393	2.847	XEJWAK	151.41	2.641	XEJLOR	157.482	3.432	XEJWAK	160.204	3.18	XEJWAK	160.447	2.824
XEKBOI	162.133	3.45	XEKLOP	160.723	3.106	XEJWEO	169.512	2.95	XEKBOI	154.583	3.259	XEKBOI	170.271	2.94
XEKWER	164.469	2.997	XEKZOE	160.953	3.317	XEKPIM	156.257	3.379	XEKPOS	159.896	2.737	XEKQAJ	157.665	3.327
XEKZUK	156.741	2.86	XEKZUK	159.152	3.122	XEKZUK	156.793	3.273	XEKZUK	159.39	3.226	XEKZUK	159.876	2.906
XELBEX	157.582	3.287	XELBEX	161.269	3.329	XELBAT	150.92	2.958	XELBAT	161.109	2.81	XELBAT	162.155	2.925
XELGOK	155.492	3.468	XELGOK	153.198	3.394	XELBEX	164.462	2.989	XELGOK	151.836	2.916	XELGOK	167.553	3.183
XELHEB	151.395	2.826	XELHEB	153.43	2.957	XELGOK	151.242	2.818	XELGOK	150.097	2.972	XELGOK	150.546	2.818
XELHEB	170.923	3.069	XELHEB	153.727	2.856	XELHEB	150.989	3.233	XELHEB	164.125	2.985	XELHEB	162.978	3.033
XELHIF	153.8	2.876	XELHIF	153.241	2.973	XELHEB	164.547	3.139	XELHEB	156.187	3.43	XELHIF	166.707	2.885
XELHIF	154.935	2.972	XELHIF	157.199	3.406	XELHIF	162.061	3.49	XELHIF	161.336	2.975	XELHIF	175.383	3.126
XELHOL	154.069	3.236	XELJII	171.333	3.192	XELHIF	174.173	3.048	XELHOL	172.452	2.866	XELHOL	157.343	2.889
XELSOX	166.816	3.409	XELSOX	172.264	2.76	XELJII	171.763	3.135	XELJII01	171.161	3.192	XELJII01	171.442	3.122
XELXAN01	152.199	3.011	XELXAN01	162.744	3.316	XELSOY	154.957	3.184	XELSUE	158.965	2.918	XELSUE	156.937	3.345
XELXAN01	171.709	3.187	XELXAN01	150.741	3.104	XELXAN01	172.884	3.485	XELXAN01	168.813	2.971	XELXAN01	157.189	3.435
XELXAN01	153.346	3.25	XELXAN01	154.109	3.05	XELXAN01	159.132	2.945	XELXAN01	162.238	3.237	XELXAN01	158.319	3.099
XELXAN01	173.648	3.344	XELYET	154.856	3.122	XELXAN01	154.815	2.913	XELXAN01	164.37	3.05	XELXAN01	153.299	2.891
XELYOD	164.955	2.912	XELYOD	162.946	3.496	XELYET	165.276	3.026	XELYET	155.962	3.41	XELYET	166.145	3.493
XEMCAX	169.722	3.354	XEMHIG	156.283	2.992	XELYOD	169.717	3.075	XELYOD	163.301	2.833	XELYOD	154.332	2.914
XEMTAL	162.66	2.834	XEMTAL	153.441	2.808	XEMHIG	161.225	3.144	XEMQOV	158.279	2.982	XEMQOV	173.293	3.407
XEMTEP	158.789	3.373	XEMTEP	151.857	3.156	XEMTEP	160.719	3.197	XEMTEP	153.344	3.227	XEMTEP	152.264	3.084
XEMZEW	151.559	2.985	XENBEA	174.4	2.918	XEMTOZ	173.746	3.09	XEMTOZ	159.774	2.942	XEMZEW	161.494	3.445
XENBOK	162.853	3.194	XENBUN	151.578	2.835	XENBEA	161.106	2.871	XENBOK	158.94	3.417	XENBOK	155.959	3.241
XENDUS	160.244	3.496	XENFIH	161.406	3.162	XENDIG	152.17	3.26	XENDUS	150.425	3.041	XENDUS	165.529	3.012
XENMOV	158.134	3.425	XENNEK	161.735	2.886	XENFIH	151.633	3.413	XENHUW	162.508	3.406	XENHUW	173.157	3.252
XENPOZ	156.15	2.96	XENPUF	166.85	3.076	XENNEK	154.566	2.953	XENNEK	151.925	3.115	XENNEK	150.594	2.768
XEPHUX	157.913	2.777	XEPMAJ	159.601	2.833	XENSES	164.651	3.444	XEPHAE	165.803	2.881	XEPHUX	165.242	2.954
XEPMUD	159.682	3.227	XEPNIS	168.675	3.338	XEPMIR	156.673	2.969	XEPMUD	172.951	3.018	XEPMUD	151.975	3.272
XEPQES	160.993	2.967	XEPQOB	158.332	3.302	XEPNIS	152.117	2.701	XEPNIS	158.174	2.776	XEPPAM	161.894	3.458
XEPWOI	152.43	3.05	XEPWOI	161.802	2.79	XEPRAL	150.707	3.064	XEPRAL	150.181	3.106	XEPRAL	163.143	2.925
XEPXOH	156.515	3.17	XEPYEY	172.458	3.176	XEPWOI	169.244	2.79	XEPXOH	152.706	3.11	XEPXOH	154.048	3.11
XEQMAI	159.939	2.695	XEQMAI	155.866	2.915	XEQDUV	153.566	2.7	XEQDUV	151.537	2.7	XEQDUV	177.463	2.815
XEQMAI	156.397	2.927	XEQMAI	163.486	3.137	XEQMAI	159.782	2.812	XEQMAI	155.124	2.822	XEQMAI	170.969	2.695
XEQWUN	153.413	3.055	XEQXIC	169.339	2.954	XEQMAI	152.236	3.069	XEQWUN	164.431	2.753	XEQWUN	150.66	3.213
XEQXUO	163.236	3.214	XEQXUO	171.694	3.359	XEQXIC	150.315	3.282	XEQXIC	160.443	3.316	XEQXUO	158.013	2.788
XERDIJ	171.222	3.216	XERDUV	170.352	3.248	XEQXUO	159.511	3.341	XERDEF	156.978	2.786	XERDEF	151.525	2.921
XERVOI	165.618	2.738	XERVOI	155.875	3.423	XERTAS	151.25	2.81	XERTAS	162.699	3.247	XERVOI	159.193	3.107
XERWAV	171.047	2.958	XERWAV	155.981	3.325	XERVUO	160.279	3.466	XERVUO	150.454	3.075	XERWAV	158.344	3.325
XERWIC	156.674	3.03	XERWIC	163.97	3.329	XERWIC	162.309	2.928	XERWIC	175.663	3.453	XERWIC	150.577	3.276
XERWIC	154.408	3.277	XERWIC	157.426	3.377	XERWIC	158.867	2.9	XERWIC	162.874	3.074	XERWIC	167.725	3.335
XERWID	152.095	2.859	XERWID	150.274	3.025	XERWIC	150.508	2.727	XERWIC	165.051	3.101	XERWID	164.694	2.837
XERWID	160.622	3.255	XERWOI	164.141	3.226	XERWID	165.829	3.119	XERWID	156.873	3.389	XERWID	161.299	3.208
XERWOI	166.541	3.142	XERWOI	163.048	2.894	XERWOI	167.58	3.362	XERWOI	158.435	3.496	XERWOI	170.737	2.81
XERWUO	157.555	3.055	XERWUO	150	3.272	XERWOJ	156.66	3.051	XERWOJ	171.255	3.085	XERWOJ	168.788	3.292
XERWUO	151.381	2.934	XERWUP	160.144	3.161	XERWUO	154.471	3.44	XERWUO	157.585	3.296	XERWUO	165.899	2.918
XERXID	156.793	2.753	XERXID	158.531	2.925	XERXIC	168.102	3.138	XERXIC	167.148	2.82	XERXIC	153.161	3.384
XERXOI	168.4	3.193	XERXOI	166.461	3.029	XERXID	165.552	2.808	XERXID	167.085	3.429	XERXID	172.452	3.082
XERXOI	159.848	2.835	XERXOJ	151.209	3.395	XERXOI	154.912	3.46	XERXOI	168.96	3.424	XERXOI	158.606	3.271
XERYIE	170.968	2.826	XERYIE	164.424	3.184	XERXOJ	168.439	3.5	XERXOJ	151.435	3.358	XERXOJ	154.285	3.295
XERYOK	170.283	3.048	XERYOK	158.104	3.478	XERYOK	157.257	3.414	XERYOK	152.015	3.414	XERYOK	174.346	3.186
XESFAE	173.206	3.289	XESFAE	160.326	3.107	XERYOK	158.509	3.485	XERYOK	167.659	3.048	XESFAE	166.397	2.922
XESFIM	157.125	3.242	XESKUB	160.486	3.272	XESFAE	160.637	3.086	XESFAE	169.365	3.049	XESFIM	174.849	3.025
XESNUG	151.2	3.402	XETBOP	168.016	3.226	XESKUC	167.424	3.33	XESLOW	150.842	2.876	XESNOA	151.333	3.382
XETRAQ	173.737	3.135	XETRAQ	152.368	3.334	XETQUJ	167.962	3.155	XETQUJ	155.351	3.464	XETRAQ	155.525	2.813
XETSIY	153.342	3.202	XETYEA	151.761	2.923	XETRAQ	152.746	3.065	XETROE	153.744	3.145	XETROH	175.043	3.063
XETZEE	174.937	2.572	XETZII	157.419	2.874	XETYIE	165.768	3.313	XETZAA	169.22	2.92	XETZEE	171.902	2.572
XETZOO	165.316	2.734	XETZUU	156.078	2.905	XETZOO	151.062	2.992	XETZOO	151.477	2.763	XETZOO	150.507	3.226
XETZUU	165.011	2.98	XETZUU	164.365	2.86	XETZUU	154.185	3.075	XETZUU	166.068	3.331	XETZUU	161.232	2.841
XEVBAE	162.642	2.861	XEVBAE	173.038	3.097	XETZUU	151.408	3.096	XETZUU	163.119	2.86	XETZUU	152.947	3.457
XEVMOD	171.211	2.695	XEVMOD	173.071	2.695	XEVDEH	161.159	2.867	XEVKIU	159.884	3.458	XEVKOC	155.507	2.769

XEVVED	169.554	3.464	XEVVUT	167.517	2.83	XEVNEU	164.9	2.935	XEVNIY	158.012	3.067	XEVVED	166.361	3.279
XEWSUQ	152.856	2.911	XEWSUQ	156.424	3.471	XEVWAA	159.562	3.13	XEWCAF	158.029	3.178	XEWCAF	151.77	2.973
XEWYAZ	152.74	3.206	XEWYIH	162.239	2.94	XEWSUQ	152.539	2.949	XEWYAZ	154.222	3.306	XEWYAZ	161.357	3.008
XEXGOX	163.099	3.018	XEXJOA	177.772	2.892	XEWYIH	162.492	2.859	XEXGOX	156.913	3.071	XEXGOX	158.863	3.328
XEXNEU	152.69	3.125	XEXPID	153.158	2.975	XEXJOA	160.498	3.415	XEXKAN	168.447	3.452	XEXKOA	151.657	3.267
XEXTUT	166.616	3.188	XEXWAC	157.848	2.701	XEXPID	165.273	3.206	XEXPOJ	156.931	2.841	XEXTUT	177.377	3.188
XEYBUA	161.113	3.197	XEYHUE	151.899	3.308	XEXWAC	152.465	3.336	XEXWAC	156.121	3.293	XEYBUA	156.927	2.984
XEYVAZ	151.002	3.439	XEYVAZ	165.169	3.276	XEYKOB	152.762	3.066	XEYPAS	165.26	3.039	XEYTIE	151.583	2.999
XEYZAD	153.683	3.386	XEYZAD	157.631	2.856	XEYXEH	158.006	3.264	XEYZAD	169.394	2.792	XEYZAD	163.17	2.969
XEZDIR	159.326	3.108	XEZDIR	152.712	2.868	XEYZAD	155.667	3.161	XEZDIR	152.492	3.072	XEZDIR	164.866	3.088
XEZDIR	152.027	3.028	XEZDIR	158.602	3.088	XEZDIR	160.715	3.482	XEZDIR	154.814	2.669	XEZDIR	150.429	2.979
XEZDUD	154.59	3.042	XEZDUD	160.408	3.27	XEZDOZ	164.297	2.843	XEZDOZ	153.991	3.155	XEZDUD	155.896	2.891
XEZDUD	165.676	2.888	XEZDUD	158.622	2.868	XEZDUD	150.906	2.974	XEZDUD	170.685	2.935	XEZDUD	170.445	3.446
XEZDUF	172.015	2.79	XEZDUF	172.746	3.489	XEZDUD	152.425	3.358	XEZDUD	154.024	3.126	XEZDUD	159.671	3.038
XEZFAL	155.127	3.42	XEZFAL	166.026	2.821	XEZDUF	153.324	3.431	XEZDUF	161.87	2.891	XEZFAL	153.871	2.727
XEZFAL	162.289	2.951	XEZFAL	166.715	3.415	XEZFAL	156.203	2.846	XEZFAL	163.94	3.196	XEZFAL	172.297	2.899
XEZFAN	172.105	3.491	XEZFER	156.923	3.013	XEZFAN	168.811	2.818	XEZFAN	163.456	3.44	XEZFAN	151.434	2.871
XEZHUF	157.922	3.411	XEZJAN	157.074	2.971	XEZFER	150.413	3.393	XEZFOB	170.637	3.493	XEZHUF	157.044	2.972
XEZLET	150.49	3.427	XEZRED	165.609	3.101	XEZJAN	157.908	3.411	XEZJET	169.03	3.358	XEZLET	156.688	3.495
XEZRON	151.097	3.339	XEZRUP	152.507	2.869	XEZRED	153.479	3.07	XEZRED	158.138	2.93	XEZRED	171.425	3.127
XFMANP	152.368	3.452	XIBBOD	150.899	2.876	XEZTAB	151.876	2.755	XEZYUY	153.023	3.16	XEZZEJ	158.913	3.427
XIBNOO	164.887	3.375	XIBRIM	168.413	2.665	XIBDAR	163.254	3.292	XIBHIB	157.103	2.991	XIBHIB	158.144	3.476
XIBTAF01	152.296	3.142	XIBTAF01	153.751	3.336	XIBRIM	154.722	3.104	XIBTAF01	152.692	3.063	XIBTAF01	155.011	2.704
XIBXOY	152.214	2.848	XIBXOY	168.381	2.848	XIBXIS	150.433	3.244	XIBXIS	179.216	2.815	XIBXOY	168.195	3.416
XIBXUE	160.036	3.373	XIBXUE	172.086	2.891	XIBXOY	170.062	3.089	XIBXUE	152.875	3.109	XIBXUE	155.351	3.076
XIBXUE	153.756	3.199	XIBXUE	154.939	2.973	XIBXUE	154.218	2.692	XIBXUE	156.281	3.288	XIBXUE	173.099	3.286
XIBXUE	162.807	3.305	XIBXUE01	171.451	2.929	XIBXUE	156.35	3.411	XIBXUE	171.066	2.976	XIBXUE	171.378	2.929
XIBXUE01	160.084	3.373	XIBXUE01	172.158	2.89	XIBXUE01	162.807	3.305	XIBXUE01	154.173	2.691	XIBXUE01	155.4	3.075
XIBXUE01	152.903	3.109	XIBXUE01	153.86	3.2	XIBXUE01	156.416	3.41	XIBXUE01	154.894	2.972	XIBXUE01	171.096	2.976
XIBZIU	173.018	3.492	XIBZIU	155.207	3.303	XIBXUE01	156.224	3.287	XIBXUE01	173.115	3.287	XIBZIU	157.392	3.417
XICRUA	172.802	3.144	XICXIQ	169.312	3.496	XIBZIU	154.275	3.417	XICFOG	153.519	3.067	XICHEX	172.17	3.497
XICXOW	154.595	3.271	XICXUC	162.944	3.007	XICXIQ	166.368	2.776	XICXOW	162.044	3.105	XICXOW	152.694	3.018
XIDDET	168.338	2.913	XIDDET	170.671	2.913	XICXUC	172.917	2.997	XICXUD	157.104	3.35	XIDDET	164.21	2.679
XIDJOK	176.662	2.822	XIDJOK	173.164	3.047	XIDDET	154.111	3.268	XIDHOI	159.648	3.219	XIDJOK	164.43	2.974
XIDKUU	150.233	3.249	XIDNAZ	171.308	3.313	XIDJOK	165.156	2.85	XIDJOK	159.702	3.247	XIDJOK	162.973	3.238
XIDNIH	153.848	2.853	XIDNIH	154.485	2.886	XIDNIH	153.393	3.135	XIDNIH	154.623	2.929	XIDNIH	157.968	2.961
XIDPOS	154.195	2.813	XIDPOS	159.35	3.125	XIDNIH	150.649	2.737	XIDNIH01	155.497	2.904	XIDNIH01	153.805	2.832
XIDWIQ	168.951	3.233	XIDWIQ	154.764	2.798	XIDRIP	150.573	3.226	XIDRIP	160.096	3.033	XIDVUF	180	3.027
XIDWIQ	162.275	3.061	XIDWIQ	150.949	3.421	XIDWIQ	167.837	2.909	XIDWIQ	155.167	2.769	XIDWIQ	167.769	2.978
XIDWIQ	168.612	3.485	XIDWIQ	154.134	2.79	XIDWIQ	169.596	2.824	XIDWIQ	169.794	2.966	XIDWIQ	171.967	2.865
XIDWIQ	158.063	2.79	XIDWOW	164.924	2.89	XIDWIQ	164.647	3.447	XIDWIQ	159.178	2.851	XIDWIQ	167.285	2.788
XIDWOW	178.225	2.759	XIDWOW	167.967	2.759	XIDWOW	153.19	3.265	XIDWOW	164.674	3.311	XIDWOW	155.496	3.347
XIFCEU	153.124	2.873	XIFCEU	157.7	3.311	XIDYIV	153.737	3.251	XIFCEU	160.605	3.105	XIFCEU	153.245	3.332
XIFCEU	152.325	3.225	XIFGIG	152.82	3.027	XIFCEU	153.017	3.243	XIFCEU	161.158	3.286	XIFCEU	156.098	3.116
XIFHIH01	159.77	3.389	XIFPUZ	157.534	2.794	XIFHIH	154.047	3.394	XIFHIH	159.597	3.389	XIFHIH01	153.281	3.483
XIFRIR	165.717	3.134	XIFRIR	175.538	2.969	XIFPUZ	151.096	2.788	XIFPUZ	150.066	2.788	XIFQOS	161.159	3.277
XIFROX	151.479	3.306	XIFWAK	163.275	3.414	XIFRIR	154.967	3.198	XIFROX	159.678	3.253	XIFROX	157.538	3.463
XIGQOV	169.522	3.479	XIGQOV	163.052	3.065	XIGDOK	159.721	3.1	XIGDOK	156.004	3.135	XIGDOK	154.617	3.43
XIGQOV	163.381	3.01	XIGQOV	155.356	3.124	XIGQOV	156.452	3.069	XIGQOV	157.061	3.303	XIGQOV	164.588	2.965
XIGVES	163.533	3.173	XIGVES	151.64	3.269	XIGQOV	164.771	3.127	XIGTUG	167.096	2.841	XIGVES	157.376	2.959
XIHBOG	160.968	3.456	XIHGII	153.001	3.163	XIGVIW	153.19	3	XIGXIX	158.214	2.864	XIGYEV	152.114	2.906
XIHWIY	163.016	2.704	XIHYIZ	162.164	2.929	XIHWIY	167.032	3.462	XIHWIY	152.589	3.361	XIHWIY	168.03	3.004
XIJFAZ	172.472	2.89	XIJFAZ	161.218	3.037	XIHYIZ	179.273	2.929	XIHYIZ	173.41	3.398	XIJFAZ	156.086	3.137
XIJGUS	152.865	3.072	XIJJIM	170.728	3.058	XIJFAZ	152.726	3.238	XIJFEF	168.834	2.867	XIJGUS	150.97	3.173
XIJMOT	165.67	3.355	XIJMOT	169.972	3.14	XIJJIM	167.837	3.024	XIJJIM	160.006	3.442	XIJJIM	174.318	3.195
XIJPUC	160.086	2.937	XIJPUC	159.837	2.925	XIJMOT	171.376	3.202	XIJMOT	154.803	3.232	XIJMOT	158.725	3.088
XIJPUC	153.187	3.056	XIJPUC	161.532	3.44	XIJPUC	158.457	3.467	XIJPUC	151.291	2.948	XIJPUC	153.336	3.083
XIJQUC	173.513	3.171	XIJWIW	177.673	3.217	XIJPUC	150.562	2.969	XIJPUC	170.199	3.374	XIJPUC	169.721	3.372
XIJYEX	157.476	3.299	XIKBEB	169.275	2.959	XIJWIW	156.434	3.279	XIJWIW	160.009	2.818	XIJWIW	166.073	2.739
XIKFEE	173.501	3.46	XIKFEE	158.254	3.107	XIKDAY	161.345	3.471	XIKDAY	159.594	2.902	XIKDAY	162.777	3.083
XIKMAH	154.334	3.123	XIKNOV	152.755	3.154	XIKFEE	166.904	2.988	XIKLUA	161.454	2.963	XIKLUA	168.315	3.172
XILGII	151.72	3.09	XILGOO	151.062	3.092	XIKNUB	152.742	3.111	XIKNUB	167.439	3.311	XIKNUB	150.035	3.038
XIMCEF	151.774	3.446	XIMCEF	164.65	2.938	XILSOC	150.778	3.314	XILTIW	158.208	3.276	XILXID	173.119	3.356
XIMMUD	153.646	3.218	XIMMUD	178.094	3.087	XIMKIR	160.381	3.228	XIMKIR	162.707	3.228	XIMMAL	151.147	3.409
XIMQAO	164.121	3.076	XIMQOC	170.203	3.201	XIMMUD	165.21	3.155	XIMMUD	174.329	3.483	XIMQAO	162.628	2.95
XIMRAP	171.771	3.273	XIMRAP	159.161	3.152	XIMQOC	151.591	3.129	XIMQUI	172.091	3.129	XIMQUI	154.428	3.034
XINCEC01	163.743	2.962	XINCEC01	159.912	2.825	XIMSUL	155.762	3.073	XIMSUL	155.928	3.269	XIMXOK	156.897	3.404
XINCEC01	152.786	3.004	XINCEC01	150.255	3.313	XINCEC01	162.267	2.825	XINCEC01	169.166	2.712	XINCEC01	154.315	2.72
XINCEC01	156.946	2.934	XINDIH	157.524	3.161	XINCEC01	172.792	2.72	XINCEC01	162.111	3.062	XINCEC01	151.795	3.145
XINDII	173.649	2.959	XINDII	166.558	3.489	XINDII	151.217	3.108	XINDII	174.412	3.185	XINDII	157.333	3.414
XINFOP	171.956	3.438	XINGAE	154.528	3.358	XINFAF	159.464	3.368	XINFAF	168.179	3.475	XINFAF	151.578	3.193
XINMAI	157.897	3.463	XINMAI	165.537	2.981	XINHUX	172.557	2.972	XINJOT	159.974	3.175	XINLEM	165.465	2.885
XINQUJ	153.683	3.285	XINQUJ	159.176	3.105	XINMIU	175.758	3.465	XINMIU	154.373	3.213	XINQUJ	152.788	3.31
XINRAQ	173.275	2.975	XINYID	178.038	3.341	XINRAQ	159.05	3.491	XINRAQ	164.019	2.935	XINRAQ	162.876	3.266
XINYID	162.131	3.327	XINYOJ	178.596	3.317	XINYID	158.705	3.439	XINYID	169.745	3.298	XINYID	157.759	3.022
XINYOJ	160.202	3.364	XIPCEE	177.82	2.865	XINYOJ	159.5	3.423	XINYOJ	167.075	3.323	XINYOJ	160.064	3.02
XIPCEE	159.423	3.028	XIPCIJ	158.336	3.389	XIPCEE	162.031	3.34	XIPCEE	154.054	3.064	XIPCEE	157.087	3.116
XIPCOO	160.525	2.919	XIPCOO	155.059	3.101	XIPCOO	153.042	3.385	XIPCOO	150.983	3.493	XIPCOO	160.912	3.42
XIPCOO	154.077	3.038	XIPCOO	173.512	2.893	XIPCOO	160.37	3.373	XIPCOO	155.827	3.068	XIPCOO	150.374	2.895

XIPCOO	168.494	3.047	XIPCOO	150.516	3.257	XIPCOO	161.857	2.896	XIPCOO	151.354	2.863	XIPCOO	154.593	2.927
XIPCUV	158.137	3.492	XIPCUV	163.99	3.335	XIPCOO	160.683	2.781	XIPCOP	169.829	3.177	XIPCUV	167.51	3.351
XIPNOZ	173.615	2.774	XIPRIZ	170.956	3.3	XIPDAC	161.356	3.016	XIPDAC	170.758	3.208	XIPNEQ	170.955	3.208
XIPSAS	162.893	3.007	XIPWUQ	151.539	3.358	XIPRIZ	167.079	2.677	XIPRIZ	173.577	2.858	XIPRUL01	152.984	2.965
XIPXAY	167.603	3.451	XIPXAY	151.595	3.229	XIPWUQ	166.595	3.058	XIPWUQ	163.926	3.29	XIPWUR	151.666	3.247
XIPZEC	176.049	2.704	XIPZEC	167.854	3.006	XIPXAY	150.874	3.433	XIPZEC	157.909	3.349	XIPZEC	164.293	2.87
XIPZIG	153.45	3.031	XIPZIG	159.703	2.981	XIPZEC	173.466	2.686	XIPZEC	169.018	3.322	XIPZIG	167.324	3.048
XIQCUX	163.121	3.224	XIQCUX	165.113	3.224	XIPZIG	164.876	2.945	XIPZIG	162.267	3.318	XIPZUU	154.985	2.652
XIQWEB	169.437	3.035	XIQWEB	151.296	3.483	XIQHEM	176.815	2.875	XIQJAK	152.869	3.102	XIQTUP	162.158	2.899
XIQWUR	163.567	2.788	XIQWUR	152.708	3.02	XIQWEB	152.17	3.418	XIQWEB	152.735	3.279	XIQWEB	155.28	2.937
XIQYUT	158.8	3.444	XIQYUT	163.494	2.941	XIQWUR	152.765	3.082	XIQWUR	156.161	3.35	XIQYON	159.105	3.151
XIRSIC	165.266	3.272	XIRSIC	171.458	3.113	XIQYUT	154.667	3.325	XIRCAE	171.602	3.078	XIRCAE	164.281	3.421
XIRWUS0	150.319	3.101	XIRWUS0	155.888	3.101	XIRWAW	158.159	2.761	XIRWUS	157.182	2.685	XIRWUS	153.156	3.109
XIRXOM	153.781	3.424	XISGEL	166.866	3.027	XIRWUS0	155.478	2.769	XIRWUS0	152.905	2.729	XIRXAZ	155.335	3.451
XISJUG	158.612	3.328	XISJUG	156.507	3.245	XISHOY	152.235	3.468	XISHOY	163.708	3.161	XISHUE	155.556	3.194
XISQOG	169.226	2.912	XISQOG	163.272	3.409	XISJUG	154.513	3.35	XISLES	163.83	3.29	XISQOG	170.829	2.912
XITCOT	168.687	3.068	XITCOT	151.681	3.057	XISSIE	168.172	3.436	XISTOK	171.399	3.377	XISTOK	158.849	2.87
XITFEL	157.256	3.493	XITHAN	174.622	2.592	XITCUZ	157.516	2.853	XITCUZ	157.849	3.154	XITCUZ	163.053	3.457
XITJOA	155.698	3.265	XITJOA	171.365	2.988	XITHAN	175.881	2.592	XITJOA	169.647	3.015	XITJOA	165.202	3.342
XITRAU	172.682	2.77	XITSOM	161.408	3.463	XITJOA	155.468	3.496	XITJOA	153.48	3.104	XITRAU	158.792	2.77
XIVFIR	160.185	3.03	XIVFIR	163.295	3.364	XITSUS	174.961	3.018	XITZIL	160.242	3.248	XIVFER	159.065	3.276
XIVFOX	160.93	3.017	XIVFOX	166.139	3.367	XIVFIR	166.535	3.491	XIVFIR	156.818	3.081	XIVFIR	162.149	3.422
XIVFUD	158.69	3.003	XIVFUD	164.974	3.371	XIVFOX	153.87	3.07	XIVFOX	175.34	3.487	XIVFOX	160.997	3.404
XIVGAK	165.267	3.377	XIVGAK	165.96	3.007	XIVFUD	166.124	3.016	XIVFUD	163.26	3.462	XIVGAK	158.926	3.004
XIVHUH	163.838	2.789	XIVHUH	154.786	3.407	XIVGAK	164.275	3.484	XIVHUH	152.425	2.847	XIVHUH	160.643	2.789
XIVJOD	158.925	3.059	XIVJOD	158.1	2.819	XIVJAP	177.275	3.076	XIVJAP	157.39	3	XIVJIX	162.962	3.232
XIWKAP	171.631	3.098	XIWKAP	159.698	3.098	XIVJOD	157.304	2.819	XIVJOD	157.716	3.133	XIVJOD	158.157	2.779
XIXJUK	163.214	3.303	XIXJUK	163.038	3.085	XIWKET	158.754	2.763	XIXJUK	154.104	3.343	XIXJUK	162.199	2.833
XIXPIF	153.562	3.436	XIXPIF	155.274	3.1	XIXMOI	168.449	3.406	XIXMOI	151.222	3.406	XIXPEB	166	3.031
XIXPUR	154.415	2.716	XIXWEJ	162.339	3.425	XIXPIF	151.492	2.934	XIXPIF	163.53	3.132	XIXPUR	151.778	2.866
XIXYIM	161.696	2.885	XIXZUC	160.147	2.661	XIXXAG	152.12	3.22	XIXXAG	154.344	3.388	XIXYIM	150.634	2.773
XIYBAL	165.558	2.843	XIYBAL	160.605	3.025	XIXZUC	151.176	3.126	XIXZUC	162.186	3.187	XIXZUC	154.194	3.469
XIYBOY	150.831	3.323	XIYBOY	164.328	3.173	XIYBOY	171.999	3	XIYBOY	171.605	3.193	XIYBOY	169.61	3.154
XIYBOY	167.587	3.087	XIYBOZ	151.285	3.195	XIYBOY	162.056	3.009	XIYBOY	169.795	2.924	XIYBOY	174.333	2.993
XIYPAY	154.736	3.155	XIYPAY	154.981	3.023	XIYBOZ	175.346	2.996	XIYHAP	168.102	3.314	XIYPAY	155.374	3.023
XIYQAZ	162.341	2.873	XIYSOQ	158.039	3.104	XIYPAY	154.241	3.159	XIYQAZ	165.544	3.474	XIYQAZ	177.46	3.448
XIYYAI	162.244	2.711	XIYYAI	162.918	3.343	XIYYAI	150.576	2.833	XIYYAI	160.375	2.815	XIYYAI	155.667	2.957
XIYYAI	161.467	3.339	XIYYAI	154.075	3.188	XIYYAI	162.924	3.147	XIYYAI	162.741	3.427	XIYYAI	152.298	3.147
XIYYIQ	165.858	3.373	XIYYIQ	157.379	2.899	XIYYAI	168.686	2.986	XIYYAI	161.414	3.188	XIYYAI	156.559	2.849
XIYYIQ	154.202	3.202	XIYYIQ	161.351	2.92	XIYYIQ	159.635	3.409	XIYYIQ	173.225	3.345	XIYYIQ	155.667	2.899
XIYYIQ	160.891	3.079	XIYYOW	154.277	2.97	XIYYIQ	164.169	2.675	XIYYIQ	156.802	3.14	XIYYIQ	161.093	3.103
XIYYOW	168.076	3.077	XIZGEV	155.606	3.224	XIYYOW	157.675	3.12	XIYYOW	168.858	2.92	XIYYOW	161.364	3.12
XIZGEV	173.683	3.115	XIZGEV	150.607	3.18	XIZGEV	152.062	3.225	XIZGEV	154.434	2.779	XIZGEV	170.018	3.459
XIZGEV	161.687	3.093	XIZGIZ	155.073	2.956	XIZGEV	159.734	3.292	XIZGEV	163.922	2.711	XIZGEV	172.943	3.044
XIZGIZ	154.832	3.036	XIZGIZ	162.253	3.036	XIZGIZ	158.039	3.076	XIZGIZ	161.104	2.859	XIZGIZ	161.522	3.159
XIZGIZ	171.007	2.979	XIZGIZ	162.069	3.055	XIZGIZ	160.702	2.721	XIZGIZ	155.629	2.827	XIZGIZ	157.731	3.055
XIZGOF	159.562	2.751	XIZGOF	156.126	3.438	XIZGOF	154.687	2.961	XIZGOF	150.89	2.847	XIZGOF	163.917	2.903
XIZGOF	169.518	3.22	XIZGOF	156.004	2.743	XIZGOF	151.485	3.278	XIZGOF	164.482	2.929	XIZGOF	153.168	3.278
XIZZIT	152.653	2.855	XIZZOZ	152.145	3.45	XIZGOF	154.375	3.471	XIZGOF	162.264	3.022	XIZGOF	167.833	3.022
XOBMEI	160.189	2.937	XOBMOS	171.137	3.482	XOBMAE	174.646	2.868	XOBMAE	154.416	2.8	XOBMAE	166.692	3.352
XOBMUA	171.49	3.245	XOBMUY	158.923	3.33	XOBMOS	170.529	2.937	XOBMOS	171.476	3.014	XOBMOS	169.97	3.075
XOBPAF	167.548	3.403	XOBPAF	170.578	2.987	XOBMUY	151.931	2.846	XOBNIP	166.707	3.332	XOBPAF	175.519	2.915
XOBPIN	161.753	3.066	XOBVAN	154.228	3.323	XOBPEJ	167.791	3.383	XOBPIN	154.24	2.986	XOBPIN	153.841	3.217
XOBVOB	169.84	3.31	XOBVOB	156.202	2.761	XOBVAN	160.165	3.444	XOBVAN	157.126	2.944	XOBVAN	150.274	3.35
XOBXOF	176.418	3.128	XOCBIA	172.945	3.085	XOBVOB	150.305	2.708	XOBVOB	159.073	3.187	XOBVOB	176.084	2.664
XOCBOG	157.196	3.059	XOCDIC	158.773	3.003	XOCBIA	152.841	3.27	XOCBIA	165.228	3.126	XOCBIA	160.457	3.186
XOCGOL	155.856	3.383	XOCGOL	154.156	3.312	XOCDIC	167.529	2.878	XOCGIF	161.733	3.112	XOCGIJ	151.478	3.472
XOCJIJ	168.307	3.464	XOCJIJ	167.518	3.219	XOCGOL	166.872	3.312	XOCGOL	160.88	3.045	XOCJEF	154.874	3.409
XOCPAG	154.13	2.877	XOCPAG	157.189	3.153	XOCMUX	174.588	3.44	XOCPAG	160.219	3.248	XOCPAG	155.061	3.474
XOCVAO	157.751	3.298	XOCVIW	161.097	2.93	XOCPAG	152.945	2.851	XOCPIO	167.734	3.083	XOCPIO	159.101	3.083
XODKOR	163.009	3.267	XODKOR	160.573	3.058	XOCVIW	157.293	3.282	XODKEI	165.423	3.411	XODKOR	175.252	2.939
XODKUX	152.523	3.41	XODSIU	162.056	3.049	XODKOR	160.341	2.653	XODKOR	157.049	2.867	XODKUX	154.228	2.994
XOFLAH	153.481	3.448	XOFLAH	155.586	3.285	XODTAN	151.429	3.269	XODXEX	171.947	2.972	XOFLAH	156.841	3.383
XOFTIX	165.389	3.152	XOFTIX	167.523	2.961	XOFTIX	151.107	3.201	XOFTIX	150.157	3.361	XOFTIX	155.244	3.03
XOFXUM	167.025	3.395	XOFXUM	156.519	2.859	XOFXUM	151.174	2.701	XOFXUM	150.756	3.268	XOFXUM	175.58	3.479
XOGHEH	165.948	3.04	XOGYOH	155.919	3.104	XOFXUM	163.964	3.193	XOFXUM	155.979	3.087	XOFXUM	163.441	2.961
XOHMIT	158.752	3.07	XOHMUF	165.522	3.152	XOHLIT	163.261	2.987	XOHLIT	167.508	2.748	XOHMIT	164.334	2.727
XOHMUF	168.723	2.753	XOHMUF	172.397	2.647	XOHMUF	170.128	2.725	XOHMUF	170.185	3.254	XOHMUF	177.741	3.015
XOHSAR	166.002	3.174	XOHSAR	162.308	3.384	XOHMUF	151.852	2.773	XOHNOA	171.669	3.468	XOHNOA	159.599	3.024
XOHSEV	151.547	2.846	XOHSEV	170.873	3.186	XOHSAR	154.118	2.911	XOHSAR	154.943	3.275	XOHSEV	161.359	2.82
XOHSIZ	159.035	3.044	XOHSIZ	153.551	3.227	XOHSIZ	175.385	3.101	XOHSIZ	157.057	3.155	XOHSIZ	165.365	3.274
XOHVAU	157.096	3.182	XOHVAU	162.123	3.124	XOHTAR	155.161	2.872	XOHTAS	161.758	3.066	XOHVAU	167.381	3.254
XOHVIC	171.532	2.929	XOHVOH	163.293	3.436	XOHVEV	172.079	2.854	XOHVEV	164.197	3.5	XOHVIC	151.143	3.003
XOHVOH	157.647	3.263	XOHVOH	166.326	2.767	XOHVOH	155.08	2.899	XOHVOH	170.418	2.735	XOHVOH	156.419	2.531
XOHVOH	155.443	3.214	XOHVOH	153.844	3.096	XOHVOH	159.497	3.101	XOHVOH	165.82	2.909	XOHVOH	165.823	3.048
XOHVOI	157.288	2.857	XOHVOI	160.275	3.189	XOHVOH	172.95	2.921	XOHVOH	172.993	2.926	XOHVOI	150.617	3.187
XOHXEA	158.516	3.361	XOHXEY	177.786	2.85	XOHVUN	150.962	2.881	XOHVUN	168.784	2.701	XOHWOK	165.65	2.668
XOHXEY	161.846	2.799	XOHXEY	156.777	3.105	XOHXEY	157.214	3.105	XOHXEY	171.188	2.883	XOHXEY	154.813	2.817
XOHXOI	174.81	3.259	XOJFEH	162.659	3.072	XOHXOI	172.241	3.275	XOHXOI	154.254	3.312	XOHXOI	150.185	2.872

XOJFEH01	162.695	2.999	XOJFEH01	163.412	3.1	XOJFEH	164.217	2.991	XOJFEH	165.189	3.206	XOJFEH	164.83	3.206
XOJROD	165.148	3.105	XOJZOP	156.828	2.899	XOJFEH01	163.231	3.1	XOJFEH01	166.091	2.926	XOJHIQ	154.143	2.82
XOKKEQ	161.637	2.931	XOKKEQ	158.099	3.253	XOJZOP	173.723	2.899	XOKGEN	162.904	3.022	XOKKEQ	168.951	3.148
XOKLEP	152.417	3.108	XOKLEP	153.275	3.121	XOKKER	157.645	3.378	XOKKER	171.121	3	XOKLEP	169.712	3.168
XOKLIT	158.78	3.238	XOKLOZ	169.408	3.168	XOKLEP	158.531	3.242	XOKLIT	168.817	3.182	XOKLIT	153.016	3.129
XOKPEW	163.69	3.165	XOKQAS0	157.933	3.23	XOKLOZ	150.883	3.134	XOKLOZ	160.087	3.233	XOKNOD	167.055	3.481
XOKQAS0	158.132	2.844	XOKQIA03	150.433	2.853	XOKQAS0	159.205	3.462	XOKQAS0	155.469	2.844	XOKQAS0	155.527	2.922
XOKQIA03	153.733	2.895	XOKQIA05	150.242	2.857	XOKQIA03	163.2	3.374	XOKQIA03	155.294	3.193	XOKQIA03	154.88	2.892
XOKQIA05	154.008	2.892	XOKVUQ	167.062	2.924	XOKQIA05	162.724	3.388	XOKQIA05	155.716	3.197	XOKQIA05	154.467	2.898
XOKYAZ	167.522	2.936	XOKYAZ	169.993	2.903	XOKVUQ	154.439	3.01	XOKYAZ	165.437	3.049	XOKYAZ	164.417	3.034
XOKYON	164.979	3.307	XOKYON	175.296	3.213	XOKYAZ01	166.474	2.89	XOKYAZ01	159.692	3.018	XOKYAZ01	151.107	2.89
XOKYON	153.727	3.106	XOKYON	152.743	3.276	XOKYON	178.539	3.245	XOKYON	155.082	3.47	XOKYON	174.788	3.166
XOKYON	154.082	3.276	XOKZAA0	165.931	3.387	XOKYON	155.659	3.054	XOKYON	157.441	3.106	XOKYON	161.618	2.945
XOLBOS	169.805	2.974	XOLBOS	164.822	3.167	XOKZAA01	153.494	3.079	XOKZAA01	161.98	3.023	XOKZAA01	159.346	3.214
XOLDAF	166.069	2.752	XOLDEJ	163.995	3.03	XOLCEI	155.005	3.394	XOLDAF	159.615	2.752	XOLDAF	175.296	3.425
XOLPET	151.742	3	XOLPET	154.838	3.038	XOLGAI	155.361	3.278	XOLJOZ01	150.912	3.222	XOLPET	157.102	3.154
XOLPOD	161.336	2.887	XOLPOD	154.804	2.769	XOLPOD	174.889	3.195	XOLPOD	172.809	3.134	XOLPOD	173.68	3.019
XOLPUJ	151.168	3.186	XOLPUJ	159.935	2.976	XOLPOD	161.956	2.839	XOLPOD	169.633	3.413	XOLPUJ	155.374	2.633
XOLVEA	161.233	3.068	XOLVID	163.441	3.105	XOLPUJ	158.659	2.793	XOLPUJ	163.215	3.361	XOLPUJ	172.167	3.356
XOMCIM	171.014	3.088	XOMCIM	162.86	3.071	XOLYIK	162.793	2.816	XOLYIK	169.932	2.902	XOLZUV	162.938	3.447
XOMDAE	174.298	2.956	XOMDAE	152.952	2.874	XOMCIM	166.79	3.208	XOMCIM	152.409	3.454	XOMDAE	152.535	2.874
XOMFIP	161.411	3.167	XOMMUL	171.276	3.252	XOMDIM	169.926	3.086	XOMDIM	150.787	3.01	XOMDIM	150.775	3.288
XOMNIA	170.739	3.264	XOMNIW	156.253	2.997	XOMMUL	160.595	2.999	XOMNEV	170.484	3.11	XOMNIA	157.838	2.994
XOMNUM	156.177	3.472	XOMNUM	167.667	3.335	XOMNIW	150.933	2.997	XOMNUM	151.286	3.441	XOMNUM	155.997	3.322
XOMPAU	156.146	3.1	XOMPAU	153.711	3.385	XOMNUM	154.238	3.375	XOMNUM	156.253	3.087	XOMPAU	167.746	3.315
XOMPAU	150.209	2.872	XOMPAU	151.187	3.444	XOMPAU	150.05	3.364	XOMPAU	156.859	3.424	XOMPAU	155.431	3.327
XOMPEY	168.294	3.379	XOMPEY	150.572	3.298	XOMPEY	157.176	3.362	XOMPEY	156.955	3.458	XOMPEY	150.246	2.804
XOMVOL	155.889	2.827	XOMVOL	167.607	3.359	XOMPEY	154.448	3.092	XOMPEY	153.088	3.345	XOMQIZ	161.942	3.047
XOMYEG	155.393	3.387	XOMYEG	152.55	3.435	XOMVOL	150.667	2.766	XOMVOL	150.2	2.917	XOMYEG	172.923	3.272
XONDOV	166.112	2.867	XONDOV	171.867	2.983	XOMYEG	167.709	3.392	XOMYUW	156.06	3.05	XONDOV	176.469	3.281
XONJES	170.887	3.04	XONJES	155.638	2.829	XONDOV	162.834	3.003	XONFEP	154.734	3.026	XONFEP	160.229	2.981
XONLIW	163.712	3.046	XONNIA	152.024	3.187	XONJES	165.933	3.119	XONJES	150.652	2.92	XONJES	163.244	3.112
XONVOO	166.609	2.99	XONVOO	166.414	2.917	XONNUK	172.283	2.967	XONPEY	161.855	3.231	XONVOO	165.237	3.134
XONVUU	155.802	2.915	XONVUU	176.061	3.4	XONVOO	158.456	2.897	XONVOO	166.945	3.056	XONVUU	153.609	3.043
XONWOP	153.917	2.901	XONWOP	164.477	3.387	XONWOP	165.879	3.211	XONWOP	161.116	2.799	XONWOP	163.881	3.387
XONZIN	168.449	2.728	XONZIN	160.402	3.166	XONWOP	158.699	3.106	XONWOP	151.131	3.029	XONZIN	160.731	3.185
XOPLIA01	152.555	2.94	XOPLIA01	161.187	3.256	XONZIN	152.765	3.044	XOPCUA	159.71	3.099	XOPLIA01	151.532	2.94
XOPMOE	153.95	3.47	XOPMOE	164.592	3.24	XOPLOG0	151.43	2.932	XOPLOG0	152.874	2.932	XOPLOG0	160.588	3.248
XOPNIZ	167.241	2.903	XOPNIZ	167.902	3.341	XOPMUK	155.717	2.926	XOPMUK	161.435	2.943	XOPNEV	155.138	2.829
XOPZEK	153.317	3.059	XOPZEK	160.875	3.286	XOPQIG	158.693	3.094	XOPVUV	156.301	3.333	XOPVUV	157.276	2.611
XOPZOV	153.062	2.762	XOPZOV	158.086	3.361	XOPZEK	162.356	3.452	XOPZEK	171.998	3.385	XOPZEK	159.233	3.059
XOQLUO	152.812	3.491	XOQNUN	154.389	3.373	XOPZOV	157.962	2.912	XOQBEO	167.062	3.26	XOQFAO	156.671	2.818
XOQPEZ	152.201	3.224	XOQPEZ	150.97	3.294	XOQNUN	156.037	3.428	XOQNUN	157.05	3.089	XOQNUN	170.756	3.116
XOQPID	150.73	3.225	XOQPID	155.246	3.019	XOQPEZ	166.06	3.224	XOQPEZ	159.26	2.972	XOQPID	150.517	3.368
XOQPID	153.847	2.846	XOQPID	154.942	3.303	XOQPID	150.017	2.974	XOQPID	154.703	3.049	XOQPID	162.354	3.456
XOQPOL	160.471	2.798	XOQPOL	175.4	2.755	XOQPID	159.174	3.026	XOQPID	154.932	3.188	XOQPOL	177.376	3.074
XOQRUS	159.177	2.801	XOQRUS	166.744	3.43	XOQPUR	155.98	3.273	XOQPUR	165.248	3.254	XOQROM	150.944	3.371
XOQYUX	165.718	3.134	XOQYUX	167.923	3.251	XOQYUX	168.157	2.968	XOQYUX	159.882	3.174	XOQYUX	168.972	3.079
XORHEV	154.078	2.863	XORHEV	175.297	3.268	XORCUD	157.119	3.428	XORCUD0	154.204	3.375	XORHEV	153.924	3.17
XORHEV	153.051	3.229	XORHEV	151.806	3.014	XORHEV	155.258	2.959	XORHEV	157.248	3.448	XORHEV	158.105	3.282
XORNEB	155.709	2.838	XORNEB	153.066	2.79	XORHEV	167.999	3.455	XORMUO	160.276	3.452	XORNEB	155.867	3.02
XORNEB	171.804	2.69	XORNEB	153.843	2.752	XORNEB	156.532	2.925	XORNEB	165.008	3.214	XORNEB	153.876	3.144
XORNEB	169.442	2.552	XORNEB	163.042	2.881	XORNEB	153.819	3.061	XORNEB	158.832	2.791	XORNEB	153.302	2.826
XORNOL	168.055	2.68	XORNOL	152.064	2.743	XORNOL	153.962	3.112	XORNOL	163.542	3.338	XORNOL	152.684	3.188
XORNOL	159.91	3.085	XORNOL	162.906	2.852	XORNOL	159.937	3.031	XORNOL	158.473	2.903	XORNOL	171.798	2.514
XORPAZ	150.923	2.76	XORPAZ	158.551	2.924	XORNOL	156.634	2.738	XORNOL	152.586	2.811	XORNOL	157.107	2.779
XORPED	154.794	2.928	XORPED	154.011	2.956	XORPAZ	157.023	2.834	XORPED	159.959	2.888	XORPED	154.231	2.633
XORWAD	164.38	2.763	XORWAD	160.158	3.445	XORPED	160.547	2.902	XORWAD	163.158	2.911	XORWAD	159.478	2.808
XORWEH	161.919	2.739	XORWEH	160.172	3.38	XORWAD	161.502	2.915	XORWEH	160.574	2.883	XORWEH	158.69	2.788
XORXUA	158.414	3.193	XOSJAT	151.583	3.061	XORWEH	160.313	2.886	XORXUA	160.377	3.253	XORXUA	160.122	3.115
XOSLOI	162.648	3.101	XOSQUV	172.289	3.294	XOSLAU	150.486	2.913	XOSLAU	170.628	3.379	XOSLEX	150.995	3.091
XOTJUL	151.507	3.499	XOTKAT	162.074	2.909	XOTBEQ	164.54	3.375	XOTCOA	151.613	3.215	XOTGOE	159.145	2.872
XOVBOA	151.026	2.923	XOVBOA	152.859	2.888	XOTZUE	161.254	3.32	XOVBOA	152.673	2.899	XOVBOA	152.167	2.923
XOVGUM	153.845	2.928	XOVHAT	162.513	3.368	XOVDEV	164.806	3.425	XOVGUM	152.138	2.796	XOVGUM	158.495	2.929
XOVHEX	160.606	2.972	XOVLED	166.138	3.248	XOVHEX	175.912	2.909	XOVHEX	157.362	2.951	XOVHEX	168.115	3.301
XOWJIF	166.845	3.477	XOWPIJ	158.913	3.179	XOVVUB	161.933	3.005	XOVVUB	167.943	3.147	XOWDAQ	165.335	3.14
XOWXEP	168.013	3.037	XOWXEP	157.718	3.414	XOWPIJ	159.238	3.376	XOWPIJ	153.375	3.215	XOWXEP	161.702	3.365
XOXBEU	164.73	3.177	XOXBEU	150.915	3.382	XOWZOB	164.759	3.433	XOXBAQ	159.678	3.482	XOXBAQ	163.935	3.403
XOXQUA	173.278	2.779	XOXXOX	159.412	3.241	XOXDEW	164.628	3.43	XOXGEW	165.101	2.908	XOXGOG	165.599	3.278
XOXYUE	161.171	3.22	XOXYUE	159.641	2.824	XOXYUE	157.777	2.824	XOXYUE	159.378	3.453	XOXYUE	154.202	2.74
XOXYUE	154.084	3.412	XOXZAL	176.093	3.118	XOXYUE	160.306	3.398	XOXYUE	157.522	2.994	XOXYUE	168.544	3.48
XOXZAL	171.15	3.012	XOXZAL	154.167	3.336	XOXZAL	159.906	2.636	XOXZAL	167.577	3.204	XOXZAL	158.61	3.121
XOYJIF	165.582	3.383	XOYJIF	155.458	2.924	XOXZAL	165.81	3.499	XOXZAL	169.035	3.38	XOXZAL	155.012	2.996
XOYKEC	152.071	2.598	XOYKEC	161.568	2.8	XOYJIF	172.578	2.78	XOYJIF	151.045	3.322	XOYJIF	153.221	3.036
XOYKEC	178.018	2.762	XOYLIH	151.976	3.479	XOYKEC	154.998	2.898	XOYKEC	152.166	2.936	XOYKEC	154.298	2.906
XOYLIH	152.31	2.825	XOYMEE	164.234	3.228	XOYLIH	152.229	3.088	XOYLIH	161.121	2.961	XOYLIH	173.341	3.088
XOYMII	161.389	2.678	XOYMII	167.86	2.678	XOYMEE	154.121	2.713	XOYMII	161.82	3.264	XOYMII	169.579	2.862
XOYYIU	171.658	3.222	XOYYIU	164.84	2.846	XOYMUU	173.741	3.469	XOYXIS	151.077	3.152	XOYYIU	150.738	2.875
XOZHUS	151.237	3.452	XOZYAP	168.841	3.396	XOZFAU	158.632	3.31	XOZHIG	155.224	3.032	XOZHOM	150.912	2.646

XUBQAQ	164.088	2.837	XUBTEV	152.543	3.332	XUBJEK	156.14	2.591	XUBLEL	155.068	3.011	XUBLIP	156.979	3.434
XUBXUP	150.385	2.882	XUCCII	152.447	3.075	XUBTEV	154.447	3.494	XUBTEV	150.076	2.976	XUBXUP	162.466	3.112
XUCJOW	160.381	3.199	XUCJOW	151.85	3.454	XUCFAG	154.1	3.076	XUCFAG	154.524	3.229	XUCGAG	161.467	3.128
XUCJOW	162.449	2.866	XUCJOW	152.701	3.338	XUCJOW	162	3.462	XUCJOW	150.469	3.203	XUCJOW	151.25	3.089
XUCJOW	163.073	2.992	XUCKEN	155.326	3.002	XUCJOW	158.896	3.087	XUCJOW	164.203	3.124	XUCJOW	158.307	3.14
XUCKIR	159.657	2.909	XUCKOX	159.127	3.128	XUCKEN	158.803	2.966	XUCKEN	157.378	2.867	XUCKEN	169.588	3.279
XUCKOX0	156.298	3.368	XUCKOX0	164.934	3.41	XUCKOX	156.155	3.131	XUCKOX	150.186	3.128	XUCKOX	156.734	3.312
XUCKOX0	163.977	2.995	XUCNAL	170.499	3.188	XUCKOX0	150.811	3.289	XUCKOX0	152.877	3.074	XUCKOX0	168.234	3.417
XUCNOA	153.367	3.399	XUCNOA	172.349	3.17	XUCNAL	150.096	2.966	XUCNOA	153.126	3.327	XUCNOA	167.334	3.19
XUCNOA	158.963	3.017	XUCNOA	150.948	2.812	XUCNOA	157.269	3.481	XUCNOA	174.216	3.475	XUCNOA	157.647	3.146
XUCNOA	163.251	3.016	XUCNOA	152.343	3.072	XUCNOA	170.912	3.316	XUCNOA	167.027	3.204	XUCNOA	152.212	3.118
XUCREV	169.61	3.047	XUCYEC0	165.96	2.809	XUCNOA	171.729	2.812	XUCNOA	156.499	3.321	XUCRAR	170.159	3.195
XUDZIJ	153.02	3.5	XUDZIJ	167.613	2.952	XUDJEN	170.621	3.424	XUDJEN	169.338	3.214	XUDLAN	169.58	3.195
XUFHUF	178.189	2.924	XUFHUF	165.314	3.127	XUFDAH	167.2	3.239	XUFHEN	161.846	3.406	XUFHUF	174.007	3.439
XUFHUF	151.035	2.958	XUFHUF	150.663	3.439	XUFHUF	151.018	3.401	XUFHUF	166.327	3.387	XUFHUF	157.962	3.344
XUFHUF	163.689	2.772	XUFHUF	155.788	2.693	XUFHUF	163.664	3.369	XUFHUF	158.64	3.021	XUFHUF	156.591	2.934
XUFJER	155.832	3.301	XUFJER	166.699	3.094	XUFHUF	163.821	3.462	XUFHUF	172.838	2.909	XUFJER	150.133	2.811
XUFJER	160.06	2.902	XUFJER	152.126	3.471	XUFJER	165.877	2.745	XUFJER	165.248	3.181	XUFJER	157.696	3.026
XUFJER	165.05	2.859	XUFJER	155.952	3.359	XUFJER	157.929	2.939	XUFJER	151.334	3.118	XUFJER	157.759	2.763
XUFJER	169.12	2.918	XUFJER	160.745	2.836	XUFJER	156.74	3.294	XUFJER	163.88	3.215	XUFJER	175.074	3.099
XUFJIV	155.677	3.095	XUFJIV	162.363	2.99	XUFJIV	162.807	2.807	XUFJIV	163.899	2.974	XUFJIV	165.005	2.689
XUFJIV	178.471	3.091	XUFJIV	154.1	3.307	XUFJIV	153.858	3.221	XUFJIV	152.73	3.485	XUFJIV	150.931	3.361
XUFJIV	154.11	2.829	XUFJIV	155.715	2.628	XUFJIV	155.171	2.993	XUFJIV	162.81	2.988	XUFJIV	166.465	2.721
XUFJIV	158.83	3.332	XUFJIV	170.379	3.086	XUFJIV	175.323	2.99	XUFJIV	158.16	3.175	XUFJIV	152.682	2.843
XUGBEJ	160.677	2.992	XUGCAG	167.554	3.012	XUFLEQ	160.382	2.996	XUFQOF	167.808	2.905	XUFQUL	172.299	3.167
XUGFOY0	157.119	3.156	XUGGOV	160.775	3.259	XUGDEM	166.945	2.985	XUGDIQ	164.744	3.395	XUGDIQ	163.819	3.037
XUGZOR	155.551	3.173	XUGZOR	165.308	3.203	XUGZOR	154.043	3.259	XUGZOR	152.656	2.854	XUGZOR	152.802	3.282
XUHCOT	159.392	2.973	XUHHIU	166.09	3.123	XUHBUA	165.127	2.879	XUHCEJ	162.863	2.917	XUHCIN	161.408	2.923
XUHHIU	159.129	3.439	XUHHIU	165.298	3.371	XUHHIU	170.865	3.476	XUHHIU	155.042	3.393	XUHHIU	157.411	2.772
XUHHIU	166.227	2.785	XUHHIU	155.189	3.021	XUHHIU	158.147	3.499	XUHHIU	162.25	2.802	XUHHIU	164.205	2.823
XUHHIU	152.327	3.459	XUHHIU	154.058	2.725	XUHHIU	161.793	2.845	XUHHIU	165.565	3.16	XUHHIU	158.617	3.191
XUHHIU	153.365	2.89	XUHHIU	174.561	3.259	XUHHIU	156.527	3.366	XUHHIU	157.956	2.886	XUHHIU	165.456	3.421
XUHHIU	160.461	3.267	XUHHIU	158.219	3.197	XUHHIU	151.096	3.371	XUHHIU	171.534	3.264	XUHHIU	171.502	3.461
XUHHIU	174.754	3.412	XUHHIU	154.432	3.17	XUHHIU	169.58	2.958	XUHHIU	165.247	2.97	XUHHIU	173.159	3.098
XUHHIU	161.522	3.045	XUHHIU	153.974	2.96	XUHHIU	166.331	3.148	XUHHIU	164.978	2.69	XUHHIU	160.973	2.801
XUHHIU	165.198	3.275	XUHHIU	153.496	3.259	XUHHIU	154.021	2.725	XUHHIU	153.227	2.643	XUHHIU	173.699	3.246
XUHHIU	165.379	3.324	XUHHIU	153.244	2.823	XUHHIU	176.442	3.17	XUHHIU	157.163	3.045	XUHHIU	159.578	2.643
XUHVUS	169.09	3.11	XUHVUS	158.571	2.824	XUHHIU	153.584	2.944	XUHNUJ	154.645	3.16	XUHNUL	168.013	3.204
XUJCEK	166.608	2.923	XUJCEK	150.561	3.256	XUHVUS	154.594	3.29	XUJCEK	163.116	2.92	XUJCEK	150.291	3.032
XUJCEK	169.239	3.035	XUJCEK	151.302	3.192	XUJCEK	150.981	3.032	XUJCEK	156.16	2.897	XUJCEK	163.443	2.971
XUJLEX	150.656	3.221	XUJLEX	155.167	3.225	XUJCEK	154.328	3.251	XUJCEK	168.654	3.102	XUJCEK	153.113	3.101
XUJWIL	164.629	3.18	XUJWIL	156.473	3.408	XUJLEX	157.927	3.289	XUJLEX	163.354	2.914	XUJTEF	172.698	2.942
XUJXAD	156.003	2.836	XUJXAD	151.757	3.057	XUJWIL	170.747	3.399	XUJWIL	156.561	3.022	XUJWIL	151.227	3.408
XUKHAN0	157.776	3.491	XUKHAN0	159.255	2.729	XUKFAK0	169.916	3.416	XUKFAK0	167.601	3.201	XUKFAK0	158.421	3.009
XUKRII	174.022	3.432	XUKZEI	161.414	3.332	XUKNOK	171.141	3.417	XUKNOK	160.067	3.164	XUKRII	153.855	2.638
XULKEW	159.67	3.499	XULPAV	166.188	3.102	XULCEP	151.817	3.496	XULCIT	159.166	3.41	XULDUG	162.993	3.498
XUMJAQ	166.848	3.079	XUMJAQ	156.727	3.179	XULPAV	166.377	3.106	XULPEZ	163.721	2.941	XUMFOD	156.472	3.414
XUMJEU	157.793	3.006	XUMJEU	167.19	3.371	XUMJEU	154.07	3.397	XUMJEU	173.446	3.244	XUMJEU	161.315	3.314
XUMJUM	154.889	2.999	XUMRAC	164.605	2.787	XUMJEU	157.091	3.355	XUMJEU	171.682	3.018	XUMJUM	164.857	3.31
XUMRAC	157.398	2.98	XUMRAC	168.41	2.855	XUMRAC	171.299	2.925	XUMRAC	173.402	2.804	XUMRAC	162.751	2.973
XUMRAC	164.451	2.903	XUMRAC	174.353	3.245	XUMRAC	153.158	2.728	XUMRAC	164.797	3.28	XUMRAC	168.054	2.77
XUMRAC	151.577	3.398	XUMRAC	160.842	2.787	XUMRAC	158.508	2.927	XUMRAC	152.116	2.792	XUMRAC	159.338	2.74
XUMRAC	171.928	2.873	XUMRAC	169.05	3.225	XUMRAC	158.509	3.394	XUMRAC	150.917	2.962	XUMRAC	154.833	2.925
XUMRAC	161.304	3.344	XUMRAC	166.462	3.393	XUMRAC	171.375	3.415	XUMRAC	166.295	3.155	XUMRAC	154.651	3.443
XUNFOF	163.116	2.862	XUNFOF	168.469	3.369	XUMRAC	179.451	3.388	XUMSIL	170.025	2.931	XUNFOF	159.862	3.149
XUNMAU	150.352	3.082	XUNMEB	160.375	3.221	XUNGIA	153.829	3.332	XUNLEZ	168.282	3.376	XUNLEZ	165.392	2.941
XUNWIO	159.911	3.45	XUPCOA	151.258	2.958	XUNSIL	166.768	3.176	XUNTOQ	157.078	3.191	XUNTOQ	163.97	2.893
XUPYOA	161.032	3.338	XUQCOD	159.039	3.141	XUPKUO	163.519	3.453	XUPLAV	157.172	2.822	XUPYOA	152.8	3.346
XUQHAS	167.726	3.136	XUQHAS	157.891	3.081	XUQCUJ	155.343	3.385	XUQFOI	159.49	3.342	XUQFOI	154.358	3.342
XUQHAS	155.759	3.423	XUQHAS	154.044	2.907	XUQHAS	158.028	3.456	XUQHAS	165.922	3.059	XUQHAS	152.823	3.22
XUQNUW	156.751	3.39	XUQNUW	161.451	2.895	XUQLOO	153.317	3.015	XUQLUU	174.405	3.295	XUQNAC	163.49	3.434
XUQRUY	159.46	2.958	XUQSAF	177.005	2.947	XUQRUY	158.017	2.952	XUQRUY	165.302	2.794	XUQRUY	165.619	2.958
XUQSAF	160.708	2.904	XUQSAF	154.119	2.723	XUQSAF	164.616	2.835	XUQSAF	170.878	2.87	XUQSAF	160.499	2.769
XUQSAF	163.993	2.943	XUQSAF	164.835	3.366	XUQSAF	168.023	2.73	XUQSAF	153.918	3.5	XUQSAF	172.553	3.1
XUQSAF	152.916	2.756	XUQSAF	156.89	2.952	XUQSAF	166.882	3.425	XUQSAF	156.377	2.769	XUQSAF	155.051	2.9
XUQSAF	163.454	3.425	XUQSAF	161.485	2.696	XUQSAF	153.296	3.489	XUQSAF	162.705	3.143	XUQSAF	153.583	2.908
XUQSEJ	154.88	3.061	XUQSEJ	150.21	3.119	XUQSAF	152.779	3.376	XUQSAF	150.33	3.452	XUQSAF	174.772	3.119
XUQSEJ	158.727	3.481	XUQSEJ	161.889	2.809	XUQSEJ	166.092	3.2	XUQSEJ	150.583	3.119	XUQSEJ	163.788	3.064
XUQVUE	173.831	3.364	XUQWOV	150.001	2.884	XUQSEJ	153.937	3.251	XUQVOY	154.698	3.278	XUQVOY	167.519	3.48
XUQWOV	156.539	2.845	XUQWOV	156.958	3.415	XUQWOV	155.545	2.884	XUQWOV	160.816	3.234	XUQWOV	160.153	3.323
XURPEI	174.076	3.247	XURPEI	161.851	2.763	XUQWOV	167.42	3.295	XUQWOV	153.662	3.201	XUQWUB	163.533	3.286
XURSIM	165.498	3.487	XURSIM	167.409	2.877	XURSIM	159.487	3.154	XURSIM	150.691	3.158	XURSIM	150.106	2.886
XURSIM	165.28	2.841	XURSIM	150.064	3.27	XURSIM	176.05	3.073	XURSIM	153.172	2.877	XURSIM	164.344	2.835
XURSIM	156.068	3.359	XURSIM	154.513	3.118	XURSIM	178.227	3.205	XURSIM	166.378	2.676	XURSIM	174.01	3.487
XURSOW	163.98	3.399	XUSNUU	171.531	3.257	XURSIM	164.364	2.968	XURSIM	163.366	2.911	XURSOW	152.205	3.399
XUSQIN	153.689	3.185	XUSQUZ	161.665	2.946	XUSNUU	164.358	2.812	XUSQEJ	167.503	3.056	XUSQIN	163.486	3.444
XUSREK	163.713	2.746	XUSRIO	167.995	3.478	XUSREK	159.26	3.246	XUSREK	151.953	2.993	XUSREK	155.974	2.758
XUSTEK0	170.697	3.152	XUSTEK0	154.847	3.462	XUSSEK	167.71	3.234	XUSTAH	156.398	3.5	XUSTAH	151.658	3.096
XUSTEK0	164.608	3.178	XUSTEK0	153.521	2.874	XUSTEK0	174.677	3.416	XUSTEK0	172.864	2.574	XUSTEK0	157.49	2.929

XUTBOG	151.927	3.003	XUTBOG	173.973	2.758	XUSXUH	161.02	3.118	XUTBOG	153.159	2.781	XUTBOG	160.073	3.211
XUTBOG	168.542	2.901	XUTBOG	170.124	2.915	XUTBOG	171.361	3.168	XUTBOG	160.479	3.228	XUTBOG	151.652	3.077
XUTCAT	153.056	3.014	XUTCAT	161.085	3.214	XUTCAT	168.72	2.932	XUTCAT	160.539	3.208	XUTCAT	152.007	3.048
XUTCAT	168.186	2.893	XUTLIK	161.057	3.12	XUTCAT	173.371	2.746	XUTCAT	170.753	3.148	XUTCAT	155.111	2.774
XUTLUW	160.992	3.008	XUTLUW	165.753	3.008	XUTLIK	163.794	2.927	XUTLIK	165.436	3.329	XUTLOQ	152.344	3.013
XUTMAD	155.925	2.696	XUTMAD	162.109	2.956	XUTMAD	169.038	2.989	XUTMAD	161.861	2.696	XUTMAD	169.039	3.068
XUTMAD	161.793	3.346	XUTMAD	164.602	3.361	XUTMAD	175.299	3.057	XUTMAD	163.758	3.158	XUTMAD	166.998	3.169
XUTMIL	160.196	2.655	XUTMIL	161.768	2.882	XUTMIL	152.441	2.882	XUTMIL	153.875	2.823	XUTMIL	170.272	3.323
XUTMOR	154.285	2.829	XUTMOR	161.135	2.92	XUTMIL	172.119	3.265	XUTMOR	161.156	3.383	XUTMOR	157.073	2.97
XUTMUX	155.767	2.898	XUTMUX	168.659	2.679	XUTMUX	153.117	3.239	XUTMUX	172.178	3.018	XUTMUX	165.725	3.13
XUTMUX	163.973	3.237	XUTMUX	152.78	3.099	XUTMUX	150.985	3.399	XUTMUX	156.242	2.97	XUTMUX	151.39	3.028
XUTNAE	167.19	2.739	XUTNAE	162.29	2.739	XUTNAE	160.135	3.096	XUTNAE	157.112	2.707	XUTNAE	154.341	2.984
XUTNIM	154.926	3.138	XUTNIM	164.278	2.738	XUTNIM	157.199	2.918	XUTNIM	159.443	2.918	XUTNIM	152.188	3.138
XUTPEK	176.142	2.918	XUTPOU	151.084	2.944	XUTNIM	168.046	2.738	XUTNOS	158.815	2.853	XUTPEK	169.434	3.178
XUTPUA	167.709	3.093	XUTPUA	155.226	3.094	XUTPOU	155.262	3.291	XUTPOU	161.888	3.026	XUTPOU	165.393	3.476
XUTQAH	155.727	3.16	XUTQEL	154.959	3.079	XUTPUA	175.239	3.467	XUTPUA	150.786	2.931	XUTPUA	165.532	3.194
XUTQUZ	172.199	2.857	XUTQUZ	154.418	3.494	XUTQIP	168.137	2.824	XUTQUZ	155.083	2.664	XUTQUZ	169.853	3.494
XUTRUD	157.606	2.648	XUTRUD	156.094	3.079	XUTQUZ	163.931	3.461	XUTRIQ	166.226	2.907	XUTRUD	158.566	3.079
XUTSIS	166.133	3.333	XUTSIS	163.826	3.164	XUTSIS	168.379	3.002	XUTSIS	152.041	2.951	XUTSIS	150.642	3.129
XUTXAO	162.42	3.037	XUVKIK	175.03	3.435	XUTWIU	173.16	2.948	XUTXAO	174.828	3.409	XUTXAO	156.742	3.243
XUVYEV	159.096	3.497	XUVYEV	179.637	3.167	XUVKIK	175.152	3.435	XUVKIL	162.618	2.798	XUVRAK	153.204	2.826
XUWCUR	164.523	3.248	XUWGEE	150.93	2.989	XUVYEV	159.969	3.141	XUVYEV	165.581	2.842	XUWCUR	158.749	3.248
XUWQOY	152.788	3.487	XUWQOY	159.611	2.728	XUWGEE	160.756	2.974	XUWGEE	160.776	3.382	XUWGEF	155.987	3.184
XUWRIU	165.194	3.372	XUWRIU	153.658	3.445	XUWRAL	154.295	2.868	XUWRIU	151.476	3.365	XUWRIU	161.576	2.948
XUWZUN	157.303	3.402	XUWZUN	166.362	3.438	XUWXOG	160.615	3.293	XUWZOH	157.092	3.401	XUWZOH	166.58	3.439
XUXBOI	157.044	2.967	XUXDAY	155.236	2.978	XUXBOI	159.059	3.262	XUXBOI	156.205	2.947	XUXBOI	152.159	3.152
XUXHEG	157.228	2.755	XUXHUW	175.148	3.35	XUXHAC	157.104	2.768	XUXHAC	173.171	2.809	XUXHEG	173.027	2.808
XUXHUW	163.924	3.106	XUXHUW	171.653	3.106	XUXHUW	151.717	3.424	XUXHUW	168.023	3.138	XUXHUW	171.561	3.35
XUXJEI	170.016	3.294	XUXJIM	172.859	3.085	XUXHUW	178.503	2.86	XUXJEI	159.149	3.247	XUXJEI	178.807	3.028
XUXJIM	168.449	3.124	XUXJIM	150.92	3.474	XUXJIM	178.336	2.86	XUXJIM	161.335	3.085	XUXJIM	176.093	3.341
XUXNUC	158.402	2.995	XUXNUC	163.495	3.218	XUXJIM	169.141	3.341	XUXNIO	158.638	3.43	XUXNUC	169.346	2.938
XUXNUC	171.187	2.879	XUXNUC	154.924	3.017	XUXNUC	163.394	3.388	XUXNUC	154.862	3.017	XUXNUC	172.344	3.322
XUXNUC	160.445	2.651	XUXNUC	174.951	2.938	XUXNUC	173.971	3.046	XUXNUC	151.568	2.968	XUXNUC	154.956	3.279
XUXNUC	150.387	3.004	XUXNUC	152.471	2.995	XUXNUC	154.283	2.651	XUXNUC	154.59	3.152	XUXNUC	173.559	3.322
XUXROB	155.847	2.835	XUXSOB	163.192	3.042	XUXPIQ	152.023	2.897	XUXPIQ	152.253	2.835	XUXQAJ	151.271	3.383
XUXSUG	154.081	3.016	XUXTOD	163.105	3.332	XUXSUG	151.511	3.158	XUXSUG	169.353	3.316	XUXSUG	155.627	3.158
XUXZOI	153.74	2.77	XUXZOI	157.22	2.883	XUXTOD	154.498	3.42	XUXZOI	172.832	3.168	XUXZOI	177.173	2.756
XUXZOI	173.43	2.873	XUXZOI	151.733	3.485	XUXZOI	162.161	3.041	XUXZOI	169.093	2.82	XUXZOI	171.522	3.289
XUXZOI	152.479	3.319	XUXZOI	163.647	3.188	XUXZOI	169.937	2.853	XUXZOI	166.478	2.81	XUXZOI	164.235	3.188
XUYLEI	174.69	2.831	XUYPOA	154.296	3.433	XUYKUX	171.341	2.849	XUYLAE	164.071	3.317	XUYLAE	165.864	3.349
XUZRES	171.782	3.249	XUZRES	156.087	2.966	XUYSIW	168.894	2.788	XUYSIW	151.69	3.114	XUZRES	152.305	3.406
XUZWOE	163.249	2.924	XUZWOE	153.328	3.246	XUZROC	174.459	3.078	XUZROC	156.789	3.327	XUZWIC	155.119	2.713
XUZXEZ	162.43	3.038	XUZXEZ	161.085	2.88	XUZWOE	160.618	3.345	XUZWUK	162.026	3.046	XUZWUK0	158.486	3.026
XUZXIC	171.2	2.927	XUZXIC	159.519	2.68	XUZXEZ	171.265	3.042	XUZXIC	175.503	3.319	XUZXIC	152.142	3.307
XUZYAV	154.896	2.983	XUZZOK	177.777	2.926	XUZXIC	166.617	2.68	XUZYAV	150.186	3.209	XUZYAV	150.239	2.983
YABHOA0	153.741	3.416	YABJES	161.546	2.882	XUZZOK	169.699	3.023	XUZZOK	174.592	2.926	YABHOA	152.871	3.39
YABJES	161.25	2.984	YABLIZ	156.524	3.031	YABJES	162.383	2.959	YABJES	163.014	3.022	YABJES	153.119	2.885
YACPIE	160.141	3.023	YACPIE	162.996	3.086	YABLUL	166.239	2.604	YABLUL	160.268	3.046	YABXAB	164.874	3.217
YACPOK	157.591	2.925	YACPOK	156.378	3.315	YACPIE	154.292	3.086	YACPIE	176.364	3.009	YACPIE	166.071	3.281
YACROL	176.329	2.814	YACROL	154.75	2.783	YACPOK	171.791	3.498	YACPOK	150.327	3.096	YACROL	178.704	3.458
YACROL	150.669	3.107	YACROL	165.419	3.07	YACROL	167.195	3.095	YACROL	178.501	2.714	YACROL	151.742	2.812
YADHAQ	157.075	2.775	YADHAQ	154.363	3.111	YACSUT	164.366	2.882	YACVUT	157.962	2.975	YACWEE	169.353	3.137
YADHAQ	153.384	3.22	YADKIX	172.564	2.741	YADHAQ	174.231	2.786	YADHAQ	152.544	3.081	YADHAQ	156.202	3.423
YADKIX	164.772	3.278	YADKIX	171.094	3.12	YADKIX	154.099	3.334	YADKIX	167.422	2.875	YADKIX	152.277	2.746
YADLUN	151.896	3.323	YAFBUG	162.171	3.493	YADKIX	157.047	3.035	YADKIX	158.36	3.052	YADKIX	158.253	2.696
YAGDIW	158.647	2.93	YAGLAT01	151.783	3.159	YAFMID	161.773	3.165	YAFWOU	160.544	2.874	YAFWOU	163.097	3.309
YAHLOK	158.584	3.029	YAHLOK	150.117	3.164	YAGSAD	158.788	3.428	YAHGAT	174.196	3.077	YAHKEB	154.984	3.437
YAHRUX	155.615	2.826	YAHYEL	157.188	3.248	YAHQUT	154.079	3.06	YAHQUT	158.889	2.952	YAHQUT	162.454	2.96
YAHYOZ	171.451	3.344	YAHYOZ	161.364	3.158	YAHYEL	155.946	2.993	YAHYEL10	157.188	3.248	YAHYEL10	155.946	2.993
YAJZAM	161.162	3.278	YAJZAM	165.17	2.964	YAJHIE	176.969	3.233	YAJZAM	167.876	3.294	YAJZAM	163.653	2.964
YAJZIU	168.534	3.361	YAJZIU	164.436	3.052	YAJZAM	152.077	3.112	YAJZAM	152.639	2.917	YAJZIU	167.408	3.023
YAJZOA	161.171	2.976	YAJZOA	164.279	3.056	YAJZOA	169.119	3.056	YAJZOA	155.43	2.953	YAJZOA	162.421	3.456
YAJZUG	159.08	3.134	YAJZUG	161.891	2.992	YAJZOA	161.996	3.291	YAJZUG	154.671	3.077	YAJZUG	170.119	3.137
YAKBET	153.072	3.244	YAKBET	169.552	2.829	YAJZUG	164.86	3.137	YAKBET	164.247	3.244	YAKBET	169.138	3.368
YAKBUL	177.734	2.609	YAKBUL	154.841	2.63	YAKBET	169.394	2.857	YAKBET	166.286	3.062	YAKBET	160.593	3.083
YAKBUL	154.268	3.246	YAKCEW	157.959	3.362	YAKBUL	167.04	3.183	YAKBUL	159.402	3.193	YAKBUL	151.792	3.32
YAKZEQ	160.451	2.783	YAKZET	150.531	3.362	YAKCEW	158.424	2.71	YAKSUB	165.02	2.762	YAKYIW	156.479	3.18
YAKZIX	153.29	2.766	YAKZIX	150.93	2.989	YAKZET	156.729	3.118	YAKZIU	167.507	3.33	YAKZIX	152.942	2.766
YALHUR	151.631	3.471	YALRUA	159.673	2.762	YALHOL	173.312	3.062	YALHUR	153.806	2.761	YALHUR	165.531	2.969
YALTAJ	177.397	2.818	YALTAJ	167.881	3.045	YALSAH	157.955	2.743	YALSEJ	152.079	3.13	YALTAJ	160.711	2.921
YALTAJ01	156.17	2.958	YALTAJ01	160.954	2.913	YALTAJ	156.275	2.97	YALTAJ01	177.314	2.81	YALTAJ01	168.134	3.032
YALZER	159.609	3.193	YALZER	152.713	3.183	YALTIR	166.939	2.963	YALZER	151.665	2.815	YALZER	158.993	3.298
YALZIV	160.487	3.155	YALZIV	170.085	3.093	YALZER	157.701	3.381	YALZER	152.643	3.462	YALZIV	162.042	3.155
YAMDIA	151.413	3.4	YAMDIA	161.869	2.998	YALZOB	176.614	2.716	YAMDIA	156.394	3.326	YAMDIA	163.732	3.014
YAMDUO	163.426	3.048	YAMDUO	166.101	3.489	YAMDIA	165.225	3.342	YAMDUO	163.208	3.313	YAMDUO	158.288	3.304
YAMFAT	177.355	2.767	YAMFAT	170.343	3.014	YAMDUO	164.65	3.02	YAMDUO	151.255	3.048	YAMDUO	164.929	2.862
YAMFAT	163.767	2.976	YAMFAT	154.54	2.706	YAMFAT	158.763	3.014	YAMFAT	163.057	3.167	YAMFAT	151.566	3.409
YAMFEX	157.204	3.254	YAMFEX	164.514	2.953	YAMFEX	162.85	2.953	YAMFEX	154.315	3.09	YAMFEX	155.331	3.469
YAMGAU0	161.369	3.01	YAMGAU0	159.398	3.02	YAMFEX	156.083	3.09	YAMGAU0	161.157	2.867	YAMGAU0	154.416	3.024

YAMGEY	160.266	2.995	YAMGEY	169.435	2.833	YAMGEY	160.064	3.009	YAMGEY	158.704	2.802	YAMGEY	162.702	2.723
YAMGEY	151.535	2.898	YAMGEY	155.403	2.723	YAMGEY	164.749	2.898	YAMGEY	152.684	2.897	YAMGEY	176.465	3.424
YAMGOI	160.766	3.114	YAMPUB	176.265	3.193	YAMGEY	154.104	3.428	YAMGEY	157.827	3.009	YAMGEY	163.011	2.908
YAMRIN	173.686	3.257	YAMSEM	153.673	3.407	YAMQAI	161.368	3.143	YAMQIQ	164.84	3.346	YAMQIQ	164.841	3.067
YAMSEN	162.841	3.153	YAMSEN	167.993	3.048	YAMSEM	158.335	3.084	YAMSEM	158.33	3.093	YAMSEN	167.044	3.041
YAMSIQ	158.814	2.74	YAMYAO	173.123	3.3	YAMSEN	161.295	3.353	YAMSIQ	151.899	3.453	YAMSIQ	155.586	2.74
YAMYAO	169.498	3.142	YAMYAO	156.799	3.414	YAMYAO	170.232	3.142	YAMYAO	171.293	2.922	YAMYAO	157.926	3.297
YAMYAO	166.077	3.297	YAMZAN	155.259	3.247	YAMYAO	168.628	3.051	YAMYAO	168.624	3.248	YAMYAO	159.802	2.939
YANLEG	172.425	3.283	YANLEG0	175.745	3.442	YAMZAN	152.426	3.322	YAMZAN	167.086	3.368	YANCUP	160.931	3.186
YANSUF0	175.604	3.318	YANSUF0	152.505	2.738	YANPEL	163.924	2.866	YANQIR	152.88	3.212	YANQIR	166.17	3.002
YANYOF	162.432	3.44	YANYOF	173.062	2.988	YANTAK	153.241	3.186	YANTIU	162.332	3.242	YANYOF	153.378	2.988
YAPCEZ	153.476	2.906	YAPCEZ	158.266	3.484	YANYOF	160.851	3.32	YANYOF	160.584	2.98	YAPCEZ	154.84	2.937
YAPCID	150.597	3.238	YAPCID	153.897	3.264	YAPCID	151.06	3.413	YAPCID	157.447	3.264	YAPCID	167.145	2.973
YAPDUS	156.978	3.368	YAPDUS	162.21	3.079	YAPDUS	158.621	3.02	YAPDUS	151.16	3.295	YAPDUS	169.805	3.457
YAPJAB	153.715	3.021	YAPJAB	154.946	3.325	YAPDUS	159.027	3.34	YAPHED	174.451	3.281	YAPJAB	168.472	3.265
YAPJAB	171.798	2.991	YAPJAB	165.318	3.177	YAPJAB	150.001	3.332	YAPJAB	155.264	3.325	YAPJAB	164.353	3.352
YAPJEF	170.825	2.85	YAPJEF	162.004	2.85	YAPJAB	161.672	2.813	YAPJAB	153.623	3.026	YAPJAB	162.753	3.265
YAPJIL	156.099	3.414	YAPJOP	156.905	3.277	YAPJEF	160.374	2.928	YAPJEF	173.532	3.456	YAPJEF	156.014	3.24
YAPJOR	158.957	3.078	YAPJOR	171.224	2.957	YAPJOP	153.976	3.245	YAPJOP	151.572	3.15	YAPJOP	156.623	2.798
YAPLIO	158.411	3.178	YAPRAL	169.592	3.453	YAPJOR	161.565	3.001	YAPJOR	162.864	3.12	YAPJOR	157.231	3.085
YAQLAH	150.821	3.025	YAQLAH	158.201	3.484	YAPWAP	167.15	3.049	YAQLAH	163.379	2.828	YAQLAH	156.401	3.134
YAQSAM	162.478	2.884	YAQZUP	153.221	3.256	YAQLEL	158.96	3.235	YAQLEL	156.379	3.364	YAQROZ	154.796	3.17
YARHOS	150.79	2.829	YARHOS	155.007	3.473	YARGOO	154.743	3.457	YARGOO	159.018	3.267	YARHOS	163.056	3.124
YARNEM	166.081	3.383	YARPAL	164.081	2.927	YARHOS	150.014	3.127	YARHOS	154.241	2.829	YARJIN	159.37	3.477
YASVAP	168.168	3.13	YASVAP	160.486	3.289	YARPAL	168.679	2.732	YARPEP	156.583	2.893	YARSIT02	168.54	2.724
YASVOD	152.952	3.405	YASVOD	153.179	3.007	YASVAP	152.473	2.947	YASVAP	151.086	2.947	YASVET	160.781	3.19
YASVOD	152.194	3.364	YASVOD	159.146	3.325	YASVOD	157.204	3.135	YASVOD	155.988	3.102	YASVOD	174.836	3.163
YASVOD	163.781	3.325	YATQOZ	171.354	2.851	YASVOD	169.15	3.302	YASVOD	161.279	3.299	YASVOD	158.866	2.972
YAVBUS	161.629	3.243	YAVBUS	157.309	3.251	YATQOZ	155.519	3.366	YATSEV	162.786	2.919	YATZAV	159.995	2.916
YAVJAG	166.651	3.151	YAVJIO	165.687	3.074	YAVBUS	150.582	3.364	YAVBUS0	168.598	3.084	YAVBUS0	150.584	3.206
YAVNAL	162.035	2.822	YAVNAL	159.452	2.822	YAVKEM	154.738	2.852	YAVKEM	172.808	3.344	YAVKIR	155.693	3.344
YAVQEV	166.877	2.89	YAVQEV	157.501	3.034	YAVQEV	165.671	3.475	YAVQEV	165.738	3.383	YAVQEV	165.218	3.178
YAVVIA10	153.317	3.402	YAVVIA10	150.114	3.195	YAVQEV	158.164	3.012	YAVTUK	154.608	3.276	YAVTUK	157.586	3.195
YAWQOD	166.248	3.406	YAWWAX	150.551	3.169	YAWQEU	155.258	3.245	YAWQEU	153.665	3.208	YAWQOD	173.651	3.428
YAXHUB	160.829	2.672	YAXHUB	170.039	3.148	YAXDOT	153.975	3.209	YAXDOT	153.541	3.353	YAXHUB	159.12	3.148
YAXMIW	167.807	3.467	YAXMIW	152.285	3.409	YAXHUB	151.743	3.282	YAXHUB	175.171	3.178	YAXMIW	158.441	3.443
YAXNOD	171.405	3.11	YAXNOD	154.397	3.484	YAXNOD	153.437	3.2	YAXNOD	160.465	3.108	YAXNOD	156.973	2.816
YAXNUJ0	151.197	2.702	YAXNUJ0	153.826	3.025	YAXNOD	161.619	2.816	YAXNOD	162.773	2.79	YAXNUJ0	150.725	3.337
YAXPEV	168.201	2.716	YAXPEV	151.25	2.716	YAXNUJ0	171.008	2.702	YAXNUJ0	151.524	3.374	YAXNUJ0	150.211	3.073
YAXXOL	159.79	2.885	YAXXOL	151.492	3.086	YAXPEV	152.751	3.004	YAXPEV	156.225	3.492	YAXXOL	165.072	3.178
YAXYEE	155.24	3.12	YAXYUU	159.352	2.894	YAXXOL	164.997	3.339	YAXXOL	176.195	3.086	YAXYEE	160.147	2.949
YAYHEP	166.062	3.357	YAYHEP	164.548	3.081	YAYBIK	165.365	2.885	YAYBIK	169.477	2.885	YAYFIR	163.384	2.961
YAYNAN	150.998	3.395	YAYNAN	152.291	3.26	YAYHOW	171.231	3.135	YAYHOW	160.765	2.964	YAYHOW	167.693	3.058
YAYZUX	173.983	3.149	YAZBAF	174.726	2.933	YAYQEY	174.244	3.104	YAYQIC	162.138	3.432	YAYQIC	168.516	2.968
YAZBUZ	154.863	2.72	YAZBUZ	170.043	2.853	YAZBAF	164.526	3.443	YAZBAF	168.57	3.367	YAZBEJ	153.928	3.157
YAZDEL	158.58	3.17	YAZDEL	165.686	3.266	YAZCAG	150.643	3.255	YAZCIO	157.376	3.1	YAZCUA	155.764	3.383
YAZFAJ	171.332	3.19	YAZFAJ	155.004	3.066	YAZDOV	156.82	3.011	YAZFAJ	173.341	3.377	YAZFAJ	165.93	3.325
YAZGIS	157.803	3.387	YAZGOY	152.276	3.163	YAZFEN	173.516	3.075	YAZFEN	167.241	3.162	YAZFIR	165.7	3.096
YAZHEP0	167.504	2.916	YAZHEP0	168.446	3.01	YAZGOY	168.485	3.281	YAZGUE	152.185	3.319	YAZHEP0	167.48	2.894
YAZHOZ0	155.66	3.328	YAZKES	164.734	2.861	YAZHEP0	166.298	2.822	YAZHEP0	168.03	2.95	YAZHEP0	167.336	2.866
YAZNUK	160.014	3.361	YAZNUK	162.551	3.226	YAZKES0	166.867	2.917	YAZKES0	167.931	3.04	YAZKES0	167.907	2.964
YAZPAS	157.253	3.347	YAZPEW	170.399	3.1	YAZNUK	150.351	3.247	YAZNUK	168.104	3.369	YAZPAS	170.264	3.089
YAZPIA	151.817	2.833	YAZPIA	152.207	2.853	YAZPEW	154.634	3.487	YAZPEW	156.37	3.343	YAZPIA	157.694	3.144
YAZQOJ	172.171	2.982	YAZQOJ	156.072	2.868	YAZPIA	150.66	3.281	YAZPIA	170.155	3.371	YAZPIA	163.652	3.076
YAZQOJ0	163.743	3.376	YAZQOJ0	153.378	2.895	YAZQOJ	151.286	3.197	YAZQOJ0	163.544	3.269	YAZQOJ0	152.092	3.434
YAZSAX	150.205	3.32	YAZXIJ	157.018	3.376	YAZRAW0	173.207	3.289	YAZSAX	154.871	2.951	YAZSAX	155.503	2.997
YEBKOH	170.835	3.158	YEBTIJ	170.385	3.091	YEBHOF	169.743	2.746	YEBHOF	157.398	2.917	YEBKOH	164.984	3.291
YECBEP	173	2.931	YECBIV	151.243	2.995	YEBZAJ	172.119	2.823	YEBZAJ	153.546	2.877	YEBZAJ	153.932	2.816
YECGAP	164.116	3.43	YECGAP	150.915	3.42	YECBIV	159.84	2.969	YECFIW	152.774	2.924	YECFIW	172.199	3.364
YECSUY	150.153	3.288	YECTIN	150.452	3.227	YECGAP	163.189	2.883	YECGUM	173.553	3.263	YECGUM	173.939	3.266
YECTUV	150.185	3.201	YECTUZ	155.678	3.046	YECTUV	172.661	3.444	YECTUV	163.188	2.957	YECTUV	155.143	2.91
YEDBIW	174.329	3.074	YEDBOC	168.518	3.252	YECVAH	152.706	3.186	YEDBIW	171.533	3.453	YEDBIW	161.805	2.835
YEDBUI	155.843	3.121	YEDCAP	159.269	3.196	YEDBOC	154.779	3.492	YEDBOC	155.581	3.168	YEDBUI	150.069	3.235
YEDCEP	157.73	2.949	YEDCEP	162.859	3.175	YEDCEP	156.128	2.918	YEDCEP	162.529	3.408	YEDCEP	154.193	2.918
YEDFAS	153.716	3.065	YEDFEW	151.877	3.168	YEDCEP	156.216	3.464	YEDCEP	165.226	3.079	YEDCET	155.241	2.826
YEDXEL	156.074	3.47	YEDXEL	157.336	3.401	YEDXEL	168.75	2.715	YEDXEL	151.27	3.328	YEDXEL	153.736	3.458
YEFKUT	163.999	3.427	YEFKUT	161.306	3.164	YEDXEL	152.831	2.969	YEDXEL	165.798	3.033	YEFKUT	167.144	3.427
YEFROS	154.966	3.039	YEFROS	155.64	3.021	YEFROS	168.153	3.116	YEFROS	173.763	2.583	YEFROS	161.518	3.245
YEFRUY	161.285	3.331	YEFRUY	171.581	2.936	YEFRUY	173.143	3.419	YEFRUY	154.396	3.49	YEFRUY	161.874	3.268
YEFVAJ	163.388	3.231	YEFZOZ	150.159	3.198	YEFRUY	154.067	3.146	YEFSAF	151.209	3.072	YEFVAJ	155.757	3.259
YEHRIN	159.22	3.001	YEHROT	160.746	3.053	YEFZOZ	151.133	3.286	YEGVOZ	164.542	3.417	YEHJAA	153.438	3.379
YEHWIU	163.047	3.49	YEHWIU	150.159	3.467	YEHWIU	158.838	3.374	YEHWIU	154.782	2.91	YEHWIU	156.561	3.467
YEHWOA	160.748	3.364	YEHWOA	154.379	2.909	YEHWIU	173.209	3.291	YEHWIU	157.353	2.853	YEHWIU	170.788	2.853
YEHWOA	172.528	2.894	YEHWOA	172.914	3.262	YEHWOA	156.303	3.479	YEHWOA	151.583	3.48	YEHWOA	156.817	2.894
YEHWUG	150.101	3.461	YEHWUG	156.974	2.862	YEHWUG	159.156	3.383	YEHWUG	154.676	2.909	YEHWUG	156.844	3.461
YEJBAT	150.79	3.004	YEJBAT	164.442	3.058	YEHWUG	171.21	2.862	YEHWUG	172.249	3.28	YEJBAT	150.872	3.004
YEJCOG	156.25	2.713	YEJCUM	159.765	3.155	YEJBAT	162.486	3.024	YEJCOG	152.892	3.226	YEJCOG	173.194	2.814
YEJCUM	164.542	3.142	YEJHIG	165.373	2.897	YEJCUM	177.03	3.221	YEJCUM	155.152	3.083	YEJCUM	168.286	3.052
YEJLAD	157.233	3.372	YEJNEK	173.852	3.186	YEJHUU	171.817	3.161	YEJJIJ	158.572	2.848	YEJJIJ	176.42	3.26

YEJTEP	152.386	3.413	YEJTEP	162.091	3.022	YEJQAJ	155.371	2.951	YEJSEO	154.214	3.152	YEJSOY	151.057	3.172
YEJZAR	164.189	3.008	YEKKII	150.047	3.058	YEJTUF	173.074	3.126	YEJXAQ	158.654	3.378	YEJYAO	160.263	2.61
YELHIH	161.734	2.998	YELHUT	158.42	2.974	YEKKII	159.989	2.968	YEKKII	168.395	2.984	YEKQEM	154.993	3.011
YELJEF	156.774	3.004	YELJEF	157.817	3.127	YELHUT	153.463	2.889	YELJEF	152.263	3.112	YELJEF	160.32	3.262
YELMIO	163.203	2.956	YELRIQ	152.558	3.091	YELJEF	160.602	3.383	YELLAF	170.993	2.716	YELLAF	171.852	2.811
YEMKOQ	164.858	2.808	YEMNUA	154.609	2.725	YELVEQ	169.698	3.215	YEMDOJ	158.116	3.031	YEMKOQ	158.971	3.232
YEMQAI	165.597	2.779	YEMQAI	160.986	2.982	YEMNUA	154.87	2.971	YEMNUA	152.854	2.725	YEMNUA	154.443	2.971
YEMWAR	157.165	3.079	YEMWUJ	158.589	2.842	YEMQAI	172.742	2.982	YEMQAI	167.458	3.21	YEMQOA	159.884	2.963
YENFED	163.687	2.909	YENMIP	157.775	3.058	YEMWUJ	159.703	3.08	YEMWUJ	155.383	3.081	YENDEA0	157.256	3.197
YENMIP	155.996	2.894	YENMIP	167.678	2.783	YENMIP	162.969	3.367	YENMIP	160.212	2.894	YENMIP	154.79	2.822
YEPQAP	155.613	2.639	YEPQAP	167.204	3.109	YEPNEM	168.726	3.376	YEPNEM	161.606	3.25	YEPPUI	154.442	2.963
YEQCED	176.88	3.301	YEQHOU	154.159	3.296	YEPQAP	152.288	3.051	YEPQAP	156.138	3.125	YEPQET	165.353	3.135
YEQRUH	155.125	3.424	YEQYIC	177.847	3.386	YEQHOU	168.791	2.945	YEQJEM	154.357	3.49	YEQRUH	155.719	3.247
YEQYOI	157.56	2.812	YEQYOI	160.448	2.941	YEQYIC	172.508	3.48	YEQYIC	175.67	3.399	YEQYIC	170.1	2.937
YEQYOI	156.551	2.887	YEQYOI	174.108	2.818	YEQYOI	160.146	2.899	YEQYOI	152.366	3.079	YEQYOI	168.477	2.987
YEQYOI	162.359	3.332	YEQZAV	151.232	2.906	YEQYOI	153.55	3.366	YEQYOI	157.605	2.679	YEQYOI	161.864	3.308
YEQZAV	152.526	2.914	YEQZAV	153.105	3.161	YEQZAV	161.589	2.893	YEQZAV	168.781	2.911	YEQZAV	156.922	3.086
YERCOR	158.586	2.78	YERCOR	157.349	2.78	YEQZAV	154.431	2.877	YEQZEZ	170.02	3.396	YEQZEZ	162.564	3.449
YERDAA	165.771	3.422	YERDOO	158.792	3.059	YERCOR	151.087	3.076	YERCOR	166.406	2.94	YERCOR	174.585	3.402
YERNAO	159.097	2.969	YERNAO	150.087	3.491	YERDUU	159.029	3.363	YERDUY	154.905	2.779	YERDUY	154.815	3.195
YERSEW	166.77	2.884	YERSEW	162.171	3.208	YERNAO	165.742	3.086	YERNAO	164.547	3.06	YERNOZ	158.05	3.149
YESNOD	150.218	2.732	YESNOD	154.187	3.157	YERSEW	169.466	2.787	YESMOB	171.736	2.935	YESNOD	161.641	3.148
YESSIZ	174.626	3.272	YESSIZ	169.8	2.955	YESNOD	158.673	3.396	YESNOD	159.266	2.936	YESSIZ	151.858	3.127
YESSIZ	157.769	2.989	YESSIZ	163.173	2.831	YESSIZ	156.883	3.364	YESSIZ	163.075	2.8	YESSIZ	150.684	3.489
YESSOF	175.448	2.985	YESSOF	154.631	3.266	YESSIZ	164.535	3.465	YESSIZ	161.515	3.442	YESSOF	160.343	3.242
YESSUL	162.31	3.495	YESTAR	166.045	3.173	YESSOF	158.927	2.856	YESSUL	158.887	3.33	YESSUL	162.449	2.969
YESTIA	167.108	2.662	YESTIA	155.578	2.662	YESTAR	172.479	3.427	YESTIA	157.66	2.972	YESTIA	167.302	2.924
YETJOA	164.577	3.444	YETZUV	158.795	2.983	YETGEM	150.116	2.94	YETJOA	163.511	3.396	YETJOA	176.017	3.238
YEVBAG	158.918	2.925	YEVBAG	158.325	3.242	YETZUV01	159.048	2.993	YEVBAG	167.42	2.925	YEVBAG	152.162	3.408
YEVGEL	166.536	3.449	YEVGEL	152.871	3.023	YEVBIO	160.901	3.023	YEVBIO	152.735	3.419	YEVBIO	155.617	2.936
YEVROH	155.431	3.046	YEVROH	156.437	3.233	YEVGEL	152.27	2.732	YEVJER	159.997	3.463	YEVLUJ	160.691	3.111
YEVRUN	157.626	3.24	YEWFOY	157.618	3.349	YEVROH0	158.567	3.133	YEVROH0	152.988	2.941	YEVRUN	155.003	3.029
YEWRUR	156.531	3.451	YEWRUR	170.024	3.278	YEWNOE0	150.576	2.902	YEWNOE0	150.472	3.18	YEWQOK	163.905	2.664
YEWSEC	176.423	3.367	YEWSEC	162.401	3.147	YEWRUR	165.041	3.235	YEWRUR	168.492	2.956	YEWRUR	171.782	3.234
YEWSEC	169.842	3.101	YEWSEC	163.873	3.03	YEWSEC	156.414	2.92	YEWSEC	154.457	2.861	YEWSEC	153.442	3.286
YEXGAL	162.184	2.959	YEXGAL	154.679	3.119	YEWSEC	153.946	3.201	YEWSUQ	156.931	3.486	YEWTAW	173.271	3.435
YEXGIT	170.136	3.198	YEXGIT	168.172	3.405	YEXGAL	150.448	3.04	YEXGAL	152.268	2.797	YEXGAL	174.093	2.852
YEXPIB	167.924	2.745	YEXPIB	151.474	3.402	YEXHOA	152.486	3.428	YEXHOA	158.274	3.248	YEXPIB	164.752	3.023
YEXQIB	167.788	3.108	YEXQOH	162.284	3.356	YEXPOH	164.943	2.723	YEXPOI	154.339	3.075	YEXPOI	156.95	3.145
YEYJUK	160.085	3.046	YEYJUK	157.606	3.059	YEYJAQ	155.113	3.491	YEYJOE	161.151	3.342	YEYJOE	159.488	3.343
YEYLUM	171.519	3.202	YEYLUM	155.443	2.735	YEYKAO	174.635	2.798	YEYKAO	156.397	2.876	YEYKAO	150.056	2.931
YEYNOF	160.337	3.059	YEYNOF	157.329	3.155	YEYMAT	164.831	2.859	YEYNAU	154.705	2.711	YEYNAU	157.127	2.949
YEYTAX	153.242	3.29	YEYTIJ	158.081	3.46	YEYTAX	165.562	3.406	YEYTAX	165.592	2.816	YEYTAX	156.641	3.008
YEZDIT	160.203	3.281	YEZDIT	158.509	2.713	YEYZIL	159.322	2.937	YEZDIT	156.367	3.36	YEZDIT	163.626	2.879
YEZDIT	154.379	3.217	YEZDOA0	155.249	3.343	YEZDIT	169.911	2.803	YEZDIT	170.638	2.721	YEZDIT	163.435	2.912
YEZDOZ	155.718	3.497	YEZDOZ	160.928	2.9	YEZDOA0	155.365	3.344	YEZDOA0	151.694	3.432	YEZDOZ	156.342	3.001
YEZDUF	151.92	2.726	YEZGAN	165.077	2.678	YEZDOZ	160.922	2.937	YEZDUF	157.119	2.726	YEZDUF	166.3	3.024
YEZHES	151.255	3.247	YEZTEE	156.964	3.148	YEZHES	164.638	2.891	YEZHES	164.485	3.426	YEZHES	163.598	3.213
YEZVAE	174.915	2.765	YEZVIM	150.962	2.92	YEZTIK	167.784	2.902	YEZTIK	171.996	2.677	YEZVAE	165.119	3.33
YEZXEI	153.012	3.332	YIBCUJ	156.485	2.851	YEZVIM	157.228	3.056	YEZVIM	167.351	3.014	YEZWUZ	151.603	3.333
YIBLED	164.507	3.163	YIBLED	175.141	3.233	YIBCUJ	167.999	3.047	YIBGOG	170.379	2.904	YIBKON	166.278	3.02
YIBLED01	150.483	3.279	YIBLED01	152.914	2.996	YIBLED	153.657	3.127	YIBLED	154.713	2.834	YIBLED	166.093	3.47
YIBLED01	163.843	2.751	YIBPIM	151.26	3.106	YIBLED01	152.631	3.023	YIBLED01	161.385	2.751	YIBLED01	151.334	3.021
YIBYUF	175.323	3.085	YIBYUF	166.471	3.478	YIBYAJ	166.79	3.15	YIBYUF	165.963	3.413	YIBYUF	153.297	3.199
YIBYUF	154.436	3.222	YIBYUF	173.037	2.988	YIBYUF	153.681	3.361	YIBYUF	162.401	3.125	YIBYUF	166.943	2.836
YIBYUF	157.659	3.138	YIBZEQ	152.99	3.473	YIBYUF	161.571	2.847	YIBYUF	163.379	3.471	YIBYUF	161.413	2.856
YIBZEQ	170.199	3.021	YIBZEQ	159.151	2.778	YIBZEQ	171.461	3.227	YIBZEQ	150.733	3.427	YIBZEQ	156.285	3.063
YIBZEQ	150.284	3.48	YIBZEQ	154.348	3.188	YIBZEQ	154.526	3.166	YIBZEQ	160.15	2.778	YIBZEQ	160.257	2.903
YICROU	155.231	2.988	YICSEL	162.684	3.314	YIBZUH	154.786	3.023	YICDUL	168.832	3.366	YICROU	151.159	3.37
YIDCET	153.129	3.095	YIDCET	165.917	3.155	YICXIV	174.501	3.067	YIDCET	152.122	3.379	YIDCET	164.943	3.028
YIDDUN	169.066	2.841	YIDFUP	158.532	3.17	YIDCET	155.895	3.105	YIDCET	151.789	3.088	YIDDOH	166.864	2.96
YIDHIF	159.627	3.085	YIDHUR	165.613	3.13	YIDHAX	152.223	3.268	YIDHAX	167.068	2.935	YIDHIF	158.769	3.148
YIDHUR	159.952	2.924	YIDHUR	164.699	3.258	YIDHUR	156.266	2.924	YIDHUR	151.965	3.444	YIDHUR	157.623	2.946
YIDJAZ	157.904	2.925	YIDJAZ	165.993	2.961	YIDHUR	176.967	3.077	YIDHUR	154.492	3.392	YIDHUR	151.848	3.392
YIDWOX	164.054	3.2	YIDWOX1	164.054	3.2	YIDJAZ	158.104	2.925	YIDJAZ	153.364	2.798	YIDJAZ	158.061	3.253
YIFSAL	152.407	3.071	YIFXER	167.852	3.084	YIFJUV	152.216	3.22	YIFLEF	162.898	3.166	YIFLEF	158.489	3.159
YIFXER	173.323	3.113	YIFXER01	167.801	3.079	YIFXER	168.725	3.111	YIFXER	167.215	3.086	YIFXER	164.978	3.094
YIFXER01	168.475	3.113	YIFXOE	165.136	3.094	YIFXER01	167.175	3.088	YIFXER01	173.365	3.116	YIFXER01	164.897	3.096
YIGTIU	170.718	3.41	YIGTIU	156.634	2.994	YIFZOG	158.652	2.927	YIGGUS	151.258	3.03	YIGRAL	151.5	2.828
YIHBAS	170.012	2.97	YIHBAS	172.621	3.165	YIGYOE	160.194	3.398	YIGYOE	158.011	3.16	YIHBAS	150.098	3.388
YIHSEO	170.682	3.118	YIHSIS	150.1	2.908	YIHBOI	151.362	3.115	YIHQEN	157.556	3.154	YIHSEO	167.832	3.324
YIHTIU	156.968	3.337	YIHTIU	159.692	2.767	YIHSOA	153.819	3.102	YIHSOA	163.098	2.912	YIHTIU	157.737	3.472
YIHWOD	162.843	3.098	YIHYEX	167.317	3.487	YIHTIU01	156.548	3.407	YIHTIU01	155.589	3.275	YIHTIU01	158.596	2.726
YIJQOY	153.026	3.038	YIJQOY	156.447	3.161	YIJHEH	155.87	3.345	YIJHEH	163.675	3.344	YIJHEH	162.671	2.789
YIJVUM	169.124	3.112	YIJWAT	174.846	3.218	YIJQOY	160.32	3.451	YIJVOF	163.39	3.304	YIJVUM	167.57	2.842
YIJWIB	162.868	3.312	YIJWOH	164.402	2.917	YIJWEX	163.094	3.16	YIJWEX	150.935	3.16	YIJWIB	153.596	3.477
YIJXEY	167.02	3.048	YIJXEY	156.078	3.393	YIJWOH	156.926	2.917	YIJXAT	155.086	2.957	YIJXAU	161.342	3.497
YIJXIC	150.45	2.975	YIJXIC	151.2	3.06	YIJXEY	163.019	3.279	YIJXEY	168.982	3.309	YIJXEY	173.308	3.275
YIKGUW	152.492	3.33	YIKGUW	175.853	3.33	YIJXIC	162.192	2.904	YIJXIC	159.574	2.919	YIKCEB	152.263	3.274

YIKMOY	152.992	3.06	YIKMOY	175.799	2.71	YIKLOU	150.471	3.496	YIKLOU	154.008	2.998	YIKLOU	161.57	2.998
YIKXUL	173.206	3.349	YIKZIE	150.625	3.004	YIKMOY	156.694	3.062	YIKMOY	155.01	3.086	YIKMOY	153.289	3.087
YILJUY	159.978	3.217	YILJUY	165.22	3.449	YIKZUQ	152.782	3.196	YIKZUQ	163.161	2.911	YILJUY	161.62	2.754
YILMOZ	159.481	2.953	YILPOA	150.692	2.755	YILJUY	157.041	3.397	YILMAI	165.987	2.821	YILMEM	169.136	3.085
YILZAW	166.921	3.045	YILZAW	150.139	3.042	YILWEY	172.708	3.048	YILZAW	168.704	2.875	YILZAW	167.652	2.875
YIMJIO01	157.442	2.958	YIMJIO01	157.925	2.958	YILZIE	168.525	3.172	YIMJIO	158.852	3	YIMJIO	158.984	3
YIMNIT	162.232	3.068	YINGUZ	170.541	2.846	YIMNIT	161.49	3.121	YIMNIT	164.732	3.188	YIMNIT	150.967	3.06
YINKUC	160.609	3.226	YINKUC	158.359	2.722	YINGUZ	167.056	3.197	YINGUZ	155.588	2.846	YINKUC	158.108	3.355
YINKUC	151.16	3.383	YINKUC	150.613	3.312	YINKUC	159.979	2.763	YINKUC	156.534	3.014	YINKUC	166.211	2.887
YINKUC	153.445	3.13	YINKUC	158.746	2.722	YINKUC	150.998	3.186	YINKUC	154.456	2.98	YINKUC	155.54	2.83
YINVOK	156.149	3.141	YIPCIK	153.644	3.136	YINKUC	167.444	2.778	YINVIB	150.256	3.143	YINVOK	175.033	3.272
YIPWUP	153.777	2.993	YIPXAA	167.371	3.041	YIPDOS	167.48	3.186	YIPPEU	167.333	2.835	YIPPEU	158.717	2.853
YIQREW	164.112	2.819	YIQSIE	154.65	3.431	YIQDUB	155.402	3.373	YIQDUB	173.635	3.36	YIQREW	162.938	2.819
YIQXAA	151.025	3.273	YIQXAA	163.769	3.415	YIQXAA	158.427	3.044	YIQXAA	154.718	2.888	YIQXAA	153.031	3.129
YIQXAA	168.106	3.494	YIQYEF	152.566	3.072	YIQXAA	156.452	3.126	YIQXAA	150.799	2.794	YIQXAA	151.736	3.372
YIRBEJ	156.552	3.13	YIRBUZ	155.391	3.403	YIQYUS	164.077	3.118	YIRBEJ	173.857	3.374	YIRBEJ	160.923	3.078
YIRGOY	174.217	3.369	YIRGOY	160.448	3.104	YIRCOU	152.529	3.486	YIRCOU	155.97	2.967	YIRCOU	171.689	3.12
YIRKES	159.534	2.881	YIRKOC	173.184	3.076	YIRGOY	157.326	3.14	YIRJUH	156.887	3.311	YIRJUH	156.029	3.314
YIRLIX	159.577	3.245	YIRLIX	169.498	3.424	YIRKOC	156.13	2.994	YIRKOC	154.604	3.481	YIRLAP	150.447	2.94
YIRNAO	154.514	3.395	YIRQEX	153.641	3.193	YIRLOE	158.889	3.252	YIRLOE	156.97	3.375	YIRNAO	174.215	3.252
YISKEQ	159.207	2.924	YISKEQ	160.251	2.93	YIRTIE	155.198	3.015	YISCIN	158.773	3.331	YISHOB	156.52	3.01
YISMAR	165.048	3.04	YISMAR	153.322	3.315	YISKEQ	177.757	3.279	YISKEQ	172.404	2.797	YISKEQ	163.631	3.153
YISTEC	171.361	3.126	YISVII	165.706	3.288	YISMEV	151.097	3.422	YISMEV	150.375	3.022	YISMEV	162.414	3.319
YITCAI	166.428	2.951	YITCAI	155.217	2.835	YISVUU	169.47	2.979	YITCAI	150.068	3.45	YITCAI	157.677	2.865
YITDUE	175.322	3.034	YITDUE	154.19	2.897	YITDUE	162.031	2.646	YITDUE	170.965	2.967	YITDUE	152.465	3.461
YITDUE	151.711	2.897	YITDUE	167.051	3.241	YITDUE	155.98	2.845	YITDUE	164.743	2.897	YITDUE	154.597	2.646
YITDUE	178.236	3.113	YITDUE	177.804	3.115	YITDUE	163.016	3.286	YITDUE	165.727	2.983	YITDUE	169.833	3.021
YITHOZ	163.987	3.196	YITJIW	163.649	3.363	YITDUE	151.854	2.899	YITHAL	171.722	3.456	YITHOZ	164.154	3.437
YITLUM	151.854	3.472	YITQUP	161.827	2.76	YITLUM	154.874	3.427	YITLUM	150.519	2.531	YITLUM	154.222	2.66
YITREA	153.106	3.187	YITRIE	163.449	3.41	YITQUP	177.541	3.055	YITREA	159.26	2.715	YITREA	164.244	2.828
YITVUV	160.816	2.701	YIVFOY	150.645	3.07	YITRIE	158.559	2.813	YITVAB	151.893	3.342	YITVAB	169.064	3.462
YIVWEJ	160.357	2.923	YIVYUY	154.022	3.406	YIVFOY	154.52	2.875	YIVFUE	167.609	3.377	YIVWAF	151.691	3.049
YIWDIR	172.534	3.21	YIWDUG	151.402	2.881	YIVYUY	165.818	3.092	YIVYUY	152.267	2.901	YIWDIR	153.17	3.014
YIWNAV	165.773	3.062	YIWNAV	162.544	2.637	YIWGIX	158.547	3.264	YIWGIX	159.504	3.103	YIWGUI	175.467	3.26
YIWNAV	165.241	3.063	YIWYOW	169.278	3.351	YIWNAV	155.704	3.174	YIWNAV	158.452	2.949	YIWNAV	156.56	3.182
YIXNUO	156.026	3.498	YIXVUW	154.167	3.435	YIXJEX	150.178	3.47	YIXLOI	164.033	3.031	YIXNUO	153.792	3.398
YIYHUK	166.573	3.29	YIYHUK	167.53	3.435	YIXVUX	166.297	3.317	YIXXIP	154.038	3.36	YIXXUZ	162.456	3.034
YIYHUK	163.262	2.765	YIYHUK	150.356	3.239	YIYHUK	161.367	3.29	YIYHUK	167.717	2.69	YIYHUK	153.97	3.432
YIYHUK01	161.201	3.303	YIYHUK01	167.574	2.695	YIYHUK01	165.981	3.303	YIYHUK01	150.646	3.236	YIYHUK01	163.067	2.783
YIYKIC	162.785	3.293	YIYLIE	157.007	3.149	YIYHUK01	167.992	3.433	YIYHUK01	154.013	3.432	YIYKIC	158.833	3.412
YIYLUQ	155.885	3.227	YIYLUQ	172.539	2.951	YIYLIE	177.279	2.854	YIYLIE	168.695	2.783	YIYLUQ	160.826	3.288
YIYMAX	165.358	3.398	YIYMAX	155.789	3.034	YIYLUQ	179.014	3.31	YIYLUQ	153.597	3.162	YIYMAX	166.839	3.034
YIYNEC	165.17	3.061	YIYNUQ	173.888	3.237	YIYMAX	154.785	3.433	YIYMIE	168.148	3.434	YIYMIE	163.766	2.969
YIYTOS	153.409	2.704	YIYTOS	168.444	2.984	YIYNUQ	157.588	3.256	YIYSOP	156.199	2.644	YIYSOP	165.497	3.087
YIYZIR	174.166	3.341	YIYZIR	150.536	3.043	YIYTUY	155.342	3.38	YIYTUY	170.85	3.143	YIYZIR	162.872	3.031
YIYZIR	170.322	3.285	YIYZIR	164.754	2.99	YIYZIR	163.374	3.292	YIYZIR	159.349	3.104	YIYZIR	150.65	3.145
YIYZIR	164.532	2.906	YIYZIR	152.053	3.206	YIYZIR	167.344	3.069	YIYZIR	166.342	2.916	YIYZIR	175.963	3.385
YIYZOX	159.648	3.327	YIYZOX	165.919	3.318	YIYZIR	157.658	3.04	YIYZIR	168.017	3.031	YIYZOX	150.391	3.181
YIYZOX	170.848	3.221	YIYZOX	151.237	3.387	YIYZOX	163.537	3.025	YIYZOX	168.957	3.072	YIYZOX	166.639	3.238
YIYZOX	153.415	3.237	YIYZOX	157.303	3.189	YIYZOX	171.814	3.134	YIYZOX	169.182	3.398	YIYZOX	169.232	3.323
YIYZOX	153.764	3.318	YIYZOX	174.614	2.972	YIYZOX	163.845	2.921	YIYZOX	151.182	2.876	YIYZOX	164.05	2.905
YIYZOX	165.793	3.071	YIYZOX	157.113	2.849	YIYZOX	176.959	2.894	YIYZOX	173.946	2.977	YIYZOX	167.864	2.888
YIYZOX	158.982	3.474	YIYZOX	167.368	3.061	YIYZOX	157.17	3.07	YIYZOX	153.78	3.139	YIYZOX	166.243	3.404
YIZCOZ01	154.892	2.814	YIZDEQ	159.476	3.053	YIZCOZ	156.86	2.814	YIZCOZ	154.86	2.814	YIZCOZ01	156.876	2.814
YIZJUN01	165.389	3.279	YIZJUN01	169.2	2.977	YIZHAQ	169.938	3.322	YIZJAT	169.098	3.199	YIZJUN01	152.556	3.005
YIZJUN01	161.803	3.383	YIZJUN01	166.858	3.372	YIZJUN01	157.983	2.685	YIZJUN01	156.213	3.277	YIZJUN01	158.477	3.065
YIZMON	174.228	3.305	YIZMON	169.061	2.853	YIZJUN01	150.886	2.685	YIZJUN01	152.145	2.947	YIZMON	163.712	3.028
YIZSOT	174.352	2.849	YIZSOT	153.841	2.889	YIZSEH	150.754	3.36	YIZSEH	157.355	3.189	YIZSEH	165.385	3.292
YIZZAM	156.416	2.848	YOBGEE	150.116	3.145	YIZSOT	155.271	2.889	YIZSOT	172.437	2.97	YIZTUA	152.037	3.357
YOBGIJ	169.626	3.412	YOBHAC	169.422	3.386	YOBGEF	169.904	3.454	YOBGEF	169.919	3.434	YOBGIJ	170.049	3.423
YOBKOQ	160.821	2.846	YOBLEH	157.976	2.966	YOBHAC	150.398	2.869	YOBHAC	151.536	2.917	YOBKOQ	157.981	2.933
YOBLOT	175.315	2.959	YOBNAH	150.376	3.443	YOBLEH	150.619	3.135	YOBLOT	160.859	3.009	YOBLOT	173.22	3.256
YOBRAM	156.371	3.199	YOBTER	151.702	2.872	YOBNAH	171.284	3.248	YOBNAH	164.807	3.363	YOBQUE	160.652	3.34
YOBTIV	155.377	2.737	YOBTIV	168.507	2.994	YOBTER	172.255	3.16	YOBTER	159.282	2.923	YOBTIV	150.283	3.437
YOCBAX	152.038	3.384	YOCBAX	157.547	2.942	YOBYIB	159.007	3.44	YOCBAX	150.526	3.299	YOCBAX	172.92	2.926
YOCSOC	154.24	3.156	YOCSOC	167.828	2.646	YOCBAX	170.697	3.161	YOCBAX	156.297	3.276	YOCDAZ	173.428	3.026
YOCSOC	155.101	3.419	YOCSOC	159.662	2.981	YOCSOC	155.413	3.419	YOCSOC	152.539	3.415	YOCSOC	160.359	3.156
YOCSOC	160.082	3.405	YOCSOC	153.515	3.222	YOCSOC	155.413	3.222	YOCSOC	159.578	3.279	YOCSOC	161.76	2.925
YOCTIX	170.02	2.798	YOCTIX	150.687	2.771	YOCSOC	159.228	3.279	YOCTIX	158.067	2.833	YOCTIX	167.809	2.782
YOCZOH	150.597	3.379	YODBAV	160.609	3.158	YOCTIX	154.661	3.423	YOCTIX	162.528	2.798	YOCTIX	174.624	3.416
YODJIM01	169.961	2.973	YODJIM01	161.5	2.973	YODBOK	161.364	2.777	YODCAX	162.539	3.433	YODJIM01	154.036	3.171
YODJIM01	169.263	2.786	YODJIM02	153.935	3.169	YODJIM01	155.582	2.748	YODJIM01	167.766	3.171	YODJIM01	150.044	2.906
YODJIM02	167.85	3.169	YODJIM02	169.001	2.781	YODJIM02	161.685	2.958	YODJIM02	155.532	2.742	YODJIM02	170.026	2.958
YODJOS	166.234	3.284	YODJOS	157.434	3.284	YODJIM02	150.341	2.896	YODJOS	152.927	3.239	YODJOS	158.135	2.905
YODJOS	156.517	3.361	YODJOS	159.19	2.849	YODJOS	156.341	2.803	YODJOS	154.412	3.005	YODJOS	157.118	3.398
YODJUY	167.935	2.76	YODJUY	165.811	2.831	YODJOS	161.522	2.803	YODJUY	154.819	3.153	YODJUY	174.974	2.831
YODTIX	169.678	3.099	YODWAS	166.345	3.357	YODJUY	150.186	3.128	YODJUY	167.067	2.802	YODPOX	159.158	3.246
YOFBEA	159.773	3.302	YOFBEA	155.359	3.052	YODXIZ	161.209	3.351	YODXIZ	170.037	2.937	YOFBEA	155.366	3.475
YOFDEF	154.306	3.29	YOFDIJ	169.074	3.388	YOFBEA	155.363	2.927	YOFDEF	152.999	2.991	YOFDEF	169.339	3.496

YOFPAK	164.399	2.562	YOFPAK	168.978	2.562	YOFDOP	169.11	2.868	YOFJIO	158.668	3.31	YOFPAK	153.323	3.117
YOHCUT	164.092	3.042	YOHCUT	162.751	3.098	YOFXEX	161.287	3.361	YOGBAZ	153.28	3.451	YOGXAU	164.005	3.38
YOHCUT	151.147	3.098	YOHDEE	158.194	3.367	YOHCUT	166.119	2.892	YOHCUT	157.584	2.892	YOHCUT	154.213	3.042
YOHHUZ	155.584	3.399	YOHLIU	150.706	3.062	YOHDEE	150.962	3.022	YOHDEE	158.03	3.248	YOHDEE	151.204	3.219
YOHSOF	164.336	3.015	YOHSOF	165.366	3.169	YOHLIU	153.803	3.357	YOHMIU	154.319	3.431	YOHMIU	152.518	2.985
YOJBUV	171.433	3.206	YOJBUV	176.304	2.923	YOHSOF	152.668	2.908	YOHSOF	166.628	2.911	YOJBUV	172.762	3.19
YOJBUV	169.783	3.177	YOJBUV	167.32	3.192	YOJBUV	166.875	2.981	YOJBUV	167.432	3.001	YOJBUV	168.063	3.028
YOJJIR	150.107	3.204	YOJJIR	152.342	3.445	YOJBUV	171.235	3.095	YOJBUV	169.837	3.102	YOJCOQ	160.74	3.241
YOJNIV	154.858	3.386	YOJNIV	166.043	3.353	YOJJIR	154.041	3.499	YOJJIR	153.946	3.14	YOJNIV	166.4	2.91
YOKFAG	175.247	3.268	YOKFAG	166.138	3.137	YOJPAP	157	3.313	YOKFAG	154.539	3.118	YOKFAG	175.047	3.268
YOKFEK	152.608	3.358	YOKPOG	176.295	3.032	YOKFEK	176.387	3.254	YOKFEK	157.375	3.123	YOKFEK	169.384	2.739
YOKSIB	153.034	3.055	YOKSIB	158.635	3.079	YOKRIA	165.228	3.246	YOKRIA	156.085	3.479	YOKRIA	162.604	2.832
YOKSOH	150.915	3.132	YOKSOH	151.299	2.834	YOKSIB	152.404	3.258	YOKSOH	165.324	3.084	YOKSOH	151.39	3.355
YOKSOH0	152.777	2.935	YOKTAW	157.512	3.427	YOKSOH0	163.387	3.162	YOKSOH0	152.336	3.419	YOKSOH0	153.784	3.233
YOKVUS	161.622	3.169	YOKWEE	158.139	2.697	YOKTEA	161.48	2.995	YOKTEA	163.613	3.449	YOKVOM	160.393	3.009
YOKWEE	166.253	3.029	YOKWEE	162.688	3.101	YOKWEE	158.898	2.947	YOKWEE	178.299	3.134	YOKWEE	156.498	3.262
YOKWUR	155.925	2.896	YOKXUV	177.306	3.394	YOKWEE	153.299	3.262	YOKWEE	161.196	3.256	YOKWIH	155.707	3.326
YOKXUV	161.298	3.031	YOKXUV	157.258	2.948	YOKXUV	152.645	3.142	YOKXUV	151.965	3.319	YOKXUV	169.54	2.929
YOLBUW	160.861	3.384	YOLBUW	157.034	2.979	YOKYOO	167.516	3.4	YOLBUW	151.469	3.068	YOLBUW	154.688	2.869
YOLBUZ	154.915	2.825	YOLBUZ	160.62	2.771	YOLBUW	163.933	3.316	YOLBUW	172.214	2.886	YOLBUW	161.465	3.105
YOLCAG	160.772	2.998	YOLCAG	176.942	3.482	YOLBUZ	150.652	2.979	YOLBUZ	161.094	2.891	YOLCAG	154.635	3.136
YOLREZ	154.694	3.209	YOLYII	155.462	2.899	YOLCIO	150.688	2.813	YOLLER	154.267	2.736	YOLQEZ	152.393	3.462
YOLYUU	171.235	3.361	YOLYUU	160.937	3.465	YOLYII	169.925	2.899	YOLYII	151.564	3.384	YOLYII	152.677	3.293
YOLYUU	167.903	3.03	YOMCUZ	178.301	2.89	YOLYUU	172.967	3.156	YOLYUU	165.346	3.181	YOLYUU	154.23	3.149
YOMCUZ	166.917	2.785	YOMCUZ	154.361	2.882	YOMCUZ	168.55	2.785	YOMCUZ	158.706	3.08	YOMCUZ	150.379	3.198
YOMDEK	159.097	2.93	YOMDEK	171.959	2.929	YOMCUZ	159.988	2.882	YOMDEK	155.634	3.037	YOMDEK	152.874	3.195
YOMDEK	157.046	2.99	YOMNEX	158.526	2.859	YOMDEK	152.961	3.225	YOMDEK	175.048	2.93	YOMDEK	159.769	3.328
YOMNUN	156.973	3.121	YOMNUN	151.734	2.879	YOMNEX	168.1	3.356	YOMNIB	170.107	2.867	YOMNIB	166.013	3.124
YOMPAV	152.263	2.709	YOMPEZ	173.595	2.813	YOMNUN	166.991	3.131	YOMNUN	157.598	3.467	YOMNUN	162.653	2.817
YOMPUM	169.864	3.257	YOMSAY	156.811	3.232	YOMPEZ	157.854	3.346	YOMPEZ	167.533	3.414	YOMPUM	171.328	3.257
YOMSID	151.77	2.892	YOMXUT	156.034	2.787	YOMSID	155.743	3.001	YOMSID	152.391	3.437	YOMSID	157.492	3.059
YOMXUT0	151.502	3.196	YOMXUT1	157.777	2.779	YOMXUT	165.413	3.252	YOMXUT	157.621	2.787	YOMXUT0	151.239	3.255
YOMXUT1	151.23	3.218	YONREZ	171.573	3.332	YOMXUT1	156.299	2.779	YOMXUT1	165.227	3.238	YOMXUT1	151.217	3.223
YONREZ	162.355	2.939	YONREZ	163.295	3.489	YONREZ	158.925	3.214	YONREZ	173.436	2.904	YONREZ	168.559	2.939
YOPKEV	158.151	3.005	YOPPOK	153.22	3.104	YONREZ	157.605	2.756	YONREZ	158.131	2.85	YONREZ	150.933	3.019
YOPPOK0	160.095	3.139	YOPPOK0	156.784	3.124	YOPPOK	160.081	3.133	YOPPOK	157.147	3.124	YOPPOK0	153.385	3.11
YOPPOK0	153.111	3.114	YOPPOK0	159.923	3.145	YOPPOK0	150.974	3.144	YOPPOK0	161.478	3.159	YOPPOK0	154.462	3.165
YOQFIU	165.285	2.91	YOQFIU	165.464	3.38	YOPPOK0	156.554	3.124	YOPROL	162.336	2.853	YOQFIU	172.346	3.056
YOQGAN	169.366	3.319	YOQGAN	162.829	2.882	YOQFIU	160.678	3.462	YOQFOA	170.198	2.808	YOQFOA	165.129	3.134
YOQGER	154.328	3.196	YOQKAS	150.875	3.458	YOQGAN	166.303	3.043	YOQGAN	155.065	3.411	YOQGER	170.406	3.295
YOQKIA	173.309	2.947	YOQWUA	159.482	3.463	YOQKAT0	157.567	3.132	YOQKIA	162.26	3.117	YOQKIA	151.246	3.08
YOQXOV	153.885	3.035	YOQXOV	153.939	3.085	YOQWUY	156.922	3.476	YOQXOV	152.642	3.214	YOQXOV	153.496	2.992
YOQXOV	150.481	3.424	YOQXOV	159.352	3.101	YOQXOV	162.838	2.928	YOQXOV	151.965	3.035	YOQXOV	157.21	3.416
YOQZAG	163.205	3.292	YOQZEK	160.618	2.933	YOQXOV	170.075	3.291	YOQXOV	157.834	3.148	YOQZAG	157.328	3.188
YOQZOU	163.647	3.222	YOQZOU	161.734	3.363	YOQZEK	155.665	3.079	YOQZEK	154.411	3.078	YOQZOU	156.834	3.496
YOQZOU	154.234	3.015	YOQZOU	173.701	3.292	YOQZOU	165.386	2.799	YOQZOU	156.466	3.307	YOQZOU	162.142	3.287
YORBEN	150.756	2.843	YORBIR	151.089	3.302	YORBEN	166.235	2.755	YORBEN	167.588	2.755	YORBEN	169.223	3.186
YORBIR	160.461	2.862	YORBIR	162.298	2.992	YORBIR	153.964	2.992	YORBIR	173.655	2.884	YORBIR	173.808	2.87
YORBUD	165.004	3.307	YORBUD	175.465	3.232	YORBIR	167.139	3.422	YORBUD	152.495	3.467	YORBUD	164.749	2.93
YORCAK	150.357	3.293	YORCAK	157.158	3.392	YORBUD	179.386	2.804	YORBUD	173.399	2.804	YORCAK	162.389	2.967
YORDEP	172.603	3.202	YORDEP	151.553	2.888	YORCAK	164.924	3.171	YORCAK	165.704	3.319	YORCIS	155.722	3.349
YORDOZ	157.344	2.803	YORDOZ	161.062	3.155	YORDIW	166.68	2.981	YORDOZ	176.88	3.058	YORDOZ	155.39	3.22
YORLIB	158.15	3.383	YORLIB	155.706	2.91	YORLIB	175.175	3.066	YORLIB	167.86	3.258	YORLIB	164.181	3.01
YORLIB	150.338	3.208	YORLIB	160.258	3.01	YORLIB	175.854	3.383	YORLIB	150.888	2.883	YORLIB	158.671	3.23
YORLOH	178.136	3.074	YORLOH	161.368	3.135	YORLOH	160.215	2.931	YORLOH	151.34	2.936	YORLOH	163.14	2.886
YORMEB	155.835	3.209	YORMEB	168.699	3.222	YORLUN	168.182	3.151	YORLUN	178.37	3.208	YORLUN	170.036	3.347
YOSBEQ	176.062	3.459	YOSFUL	170.521	3.43	YORZUD	160.716	3.189	YORZUD	167.076	3.498	YOSBEQ	161.719	3.409
YOSQIJ	166.53	3.175	YOSQIJ	152.294	2.931	YOSHAT	168.876	3.285	YOSQIJ	156.096	3.204	YOSQIJ	162.201	2.734
YOSVUX	153.333	2.879	YOSVUX	164.876	2.879	YOSQUV	161.68	3.18	YOSQUV	153.741	2.96	YOSVUX	159.836	2.802
YOTSEI	154.091	2.985	YOTSEI	157.742	3.499	YOTBUH	157.246	3.043	YOTBUH	166.542	2.898	YOTSEI	160.922	3.107
YOTSOS	160.676	2.979	YOTSOS	157.444	3.064	YOTSEI	154.097	3.011	YOTSOS	151.005	3.34	YOTSOS	166.548	2.812
YOTSOS	150.521	3.15	YOTSOS	153.084	3.244	YOTSOS	150.692	3.13	YOTSOS	150.202	3.465	YOTSOS	167.069	2.835
YOTWUZ	166.69	2.669	YOTWUZ	153.2	3.256	YOTSOS	161.216	3.425	YOTWUZ	156.899	3.114	YOTWUZ	166.407	3.267
YOTWUZ	169.658	3.161	YOTWUZ	153.138	3.446	YOTWUZ	156.865	3.312	YOTWUZ	167.813	2.702	YOTWUZ	155.693	3.36
YOTXAG	151.523	3.387	YOTXAG	163.539	3.072	YOTXAG	152.39	3.055	YOTXAG	170.931	3.246	YOTXAG	154.742	3.036
YOTXAG	162.496	3.087	YOTXAG	172.126	3.023	YOTXAG	161.531	2.797	YOTXAG	163.166	2.844	YOTXAG	161.719	2.902
YOTYUF	156.979	3.022	YOVDAO	150.075	3.364	YOTXAG	150.568	2.859	YOTXAG	152.127	2.797	YOTYEO	162.352	2.812
YOVDAO	151.839	2.946	YOVDAO	172.937	3.465	YOVDAO	156.706	3.149	YOVDAO	164.9	3.062	YOVDAO	169.086	3.062
YOVFIC	160.449	3.239	YOVFIC	164.617	3.078	YOVDAO	158.964	2.974	YOVDAO	172.289	3.131	YOVDAO	151.339	2.974
YOVVUD	167.658	3.039	YOVVUD	172.287	3.039	YOVGIC	168.111	3.198	YOVGOI	154.681	3.285	YOVKIF	167.47	3.449
YOWCEV	151.953	3.233	YOWJEB	150.497	3.421	YOVWIS	150.031	3.435	YOVWUB	151.974	3.071	YOVWUB	152.196	3.071
YOWPUX	166.443	3.155	YOWPUX	151.866	3.354	YOWPOR	156.386	3.454	YOWPOR	155.248	3.222	YOWPOR	152.415	3.156
YOXBIX	157.31	2.94	YOXDOH	164.918	2.806	YOWQOS	160.074	3.465	YOXBIX	156.504	3.257	YOXBIX	165.619	3.257
YOXDOH	150.862	3.123	YOXDOH	173.807	3.105	YOXDOH	165.367	3.133	YOXDOH	169.157	3.382	YOXDOH	167.435	3.074
YOXKII	169.165	2.903	YOXKII	157.136	3.219	YOXDOH	172.949	3.086	YOXKII	150.368	2.882	YOXKII	153.532	3.31
YOXQOS	162.622	3.487	YOXRAE	150.125	2.872	YOXKII	163.758	2.68	YOXKII	168.094	3.191	YOXKII	150.786	2.893
YOXRAE	165.892	3.157	YOXSEN	163.67	3.303	YOXRAE	169.336	3.42	YOXRAE	154.108	3.238	YOXRAE	150.237	2.92
YOXSIR	174.717	2.999	YOXSOX	162.02	3.366	YOXSEN	166.085	3.106	YOXSEN	165.937	3.026	YOXSIR	157.569	2.999
YOYCEX	161.255	3.36	YOYCEX	162.282	3.391	YOXSOX	167.941	3.098	YOXSOX	165.573	3.063	YOXVUG	153.62	3.016

YOYLII	174.314	3.334	YOYMAD	166.864	3.22	YOYLAA	158.461	2.878	YOYLEE	151.3	2.78	YOYLEE	158.338	2.78
YOYRIR	150.887	3.049	YOYRIR	176.52	3.2	YOYMAD	167.182	2.902	YOYRIR	152.566	3.2	YOYRIR	167.257	3.341
YOYTAL	153.456	3.37	YOYTAL0	153.445	3.369	YOYRIR	163.011	3.049	YOYSIS	151.517	3.281	YOYSIS	178.377	3.281
YOYVAN	158.235	3.257	YOYVOB	163.217	2.83	YOYTUF	167.579	2.773	YOYTUF	152.149	3.016	YOYVAN	173.526	3.171
YOYZAO	170.106	3.427	YOYZAO	169.748	3.428	YOYWAO	155.152	3.419	YOYZAO	166.514	2.941	YOYZAO	170.385	2.956
YOYZIW	163.883	3.435	YOYZIW	157.881	3.362	YOYZAO	166.134	3.139	YOYZAO	151.512	3.141	YOYZAO	158.261	3.397
YOYZIW	155.06	3.255	YOZFAX	150.582	2.786	YOYZIW	160.914	3.435	YOYZIW	167.638	3.231	YOYZIW	156.905	3.131
YOZFEB	165.05	2.939	YOZGON	150.624	3.457	YOZFEB	155.195	3	YOZFEB	158.381	3.38	YOZFEB	156.636	2.985
YOZHOO	152.559	3.255	YOZHOO	168.857	3.364	YOZGON0	150.86	3.403	YOZHOO	170.415	3.482	YOZHOO	164.818	2.663
YOZTAK	152.482	2.622	YOZTAK	164.972	2.767	YOZHOO	152.572	3.487	YOZKIH	158.561	3.299	YOZNAF	156.884	3.377
YOZTAK	169.804	2.999	YOZTAK	154.803	2.893	YOZTAK	151.336	2.949	YOZTAK	151.811	3.474	YOZTAK	176.813	3.439
YUBBAZ	173.64	2.959	YUBBAZ	168.964	3.412	YOZTAK	174.287	3.228	YUBBAZ	152.929	3.412	YUBBAZ	155.696	2.95
YUBBAZ	152.766	2.878	YUBBAZ	151.604	2.73	YUBBAZ	154.148	3.056	YUBBAZ	156.53	2.95	YUBBAZ	170.734	2.959
YUBBED	165.472	2.914	YUBBIH	159.811	2.953	YUBBAZ	153.725	2.74	YUBBED	165.305	2.914	YUBBED	155.535	3.139
YUBMIV	155.739	3.03	YUBMIV	158.714	3.023	YUBBOQ	162.152	3.009	YUBMIV	174.976	2.907	YUBMIV	155.503	3.283
YUBRIY	162.411	2.798	YUBRIY	162.418	2.984	YUBRIY	153.39	2.794	YUBRIY	154.522	2.863	YUBRIY	161.109	2.745
YUCRIY	152.05	3.208	YUCROE	162.649	3.099	YUBXAY	158.829	3.412	YUCBAB	151.539	3.161	YUCLUD	161.494	3.455
YUCZEF	160.406	3.416	YUCZEF	179.718	3.158	YUCTOF	150.197	3.072	YUCWIF	169.344	3.215	YUCWIF	164.689	2.752
YUDPOF	170.476	2.959	YUDPOF	154.127	3.118	YUCZEF	161.209	2.853	YUCZIJ	157.555	3.006	YUCZUV	170.567	3.156
YUFHOA	163.939	2.917	YUFHOA	153.282	3.132	YUDYOO	150.399	3.334	YUDYOO	169.107	3.076	YUDYOO	162.263	3.177
YUGCOT	177.305	3.107	YUGSOK	152.276	3.258	YUFNUJ	158.081	3.144	YUFRIC	172.262	3.113	YUFSAX	153.277	3.466
YUGZAC	156.218	3.317	YUHBAE	162.957	3.254	YUGTEC	167.554	3.262	YUGVEC0	154.615	3.411	YUGZAC	158.46	3.204
YUHQIF	165.375	2.885	YUJBEO	152.218	3.489	YUHLIX	154.274	3.313	YUHQEB	173.066	3.358	YUHQEB	166.511	3.341
YUJGOC	160.942	2.682	YUJGOC	152.789	3.007	YUJBEO	166.895	2.978	YUJDAI	150.489	2.995	YUJDAI	157.869	3.338
YUJGOZ	167.333	2.872	YUJGOZ	155.197	2.97	YUJGOC	159.891	2.978	YUJGOZ	165.293	2.872	YUJGOZ	160.457	2.97
YUJREB	150.231	3.245	YUJXEJ	166.033	3.107	YUJKOG	167.734	2.818	YUJKOG	163.725	3.418	YUJKOG	165.162	2.935
YUKNOH	151.965	2.83	YUKNUN	155.03	2.869	YUJXIN	161.868	3.29	YUJXIN	156.738	2.754	YUKNOH	153.362	3.224
YUKRUR	155.906	3.46	YUKRUR	165.57	3.127	YUKRUR	162.364	2.927	YUKRUR	164.54	3.211	YUKRUR	152.678	3.497
YULCAJ	154.71	3.087	YULCAJ	162.756	3.198	YUKRUR	161.046	2.931	YULBEM	160.866	2.826	YULBUC	150.646	2.87
YULVUX	167.08	2.944	YUMFAP	165.215	3.049	YULCAJ	159.508	3.481	YULHAO	161.727	3.241	YULVOR	167.946	2.928
YUMFAP	169.922	2.984	YUMFAP	170.015	3.227	YUMFAP	159.647	3.317	YUMFAP	155.237	3.013	YUMFAP	154.529	3.045
YUMFET	156.106	3.135	YUMFET	161.976	3.107	YUMFET	150.748	3.247	YUMFET	156.953	3.374	YUMFET	168.421	2.751
YUMFIX	167.375	3.178	YUMFIX	174.239	3.293	YUMFIX	153.001	3.346	YUMFIX	157.038	2.919	YUMFIX	166.662	3.113
YUMFIX	154.709	3.178	YUMHUN	165.814	3.291	YUMFIX	170.602	2.833	YUMFIX	157.599	3.171	YUMFIX	158.206	2.979
YUMLOL	156.836	2.971	YUNSAB	171.181	2.859	YUMLOL	161.128	2.989	YUMLOL	157.92	3.096	YUMLOL	157.235	3.423
YUNXUA	151.446	3.093	YUNXUA0	154.351	2.987	YUNSAB	150.477	3.033	YUNXUA	155.042	3.04	YUNXUA	160.422	2.986
YUNYEO	166.804	3.008	YUNYUE	150.561	3.029	YUNXUA0	159.611	2.962	YUNYEO	152.59	3.212	YUNYEO	170.874	3.168
YUPDOE	158.195	2.938	YUPDOE	166.24	2.809	YUNZIU01	155.032	3.039	YUNZIU01	159.35	3.067	YUPDOE	174.727	3.316
YUPHAT	162.033	2.881	YUPHAT	162.694	3.048	YUPGUO	158.823	2.659	YUPGUO0	158.637	2.662	YUPHAT	154.942	2.957
YUPQUZ	152.383	3.38	YUPQUZ	152.154	3.38	YUPHIB	161.116	3.377	YUPNEG	157.085	3.325	YUPQUZ	168.037	2.978
YUPRIO	156.45	3.201	YUPRIO	167.148	3.222	YUPQUZ	159.32	2.851	YUPRIO	165.287	2.943	YUPRIO	162.278	3.017
YUPYAN	162.932	3.169	YUPYAN	157.321	2.967	YUPVUE	163.37	2.902	YUPWAL	172.854	2.97	YUPYAN	171.442	3.421
YUQMON	152.977	3.346	YUQNEE	162.409	2.955	YUPYAN	150.51	3.001	YUPYAN	153.81	3.412	YUPYUG	157.342	3.27
YUQTUC	162.509	3.013	YURGUN	163.022	3.044	YUQNEE	163.938	3.003	YUQPAF	163.169	3.023	YUQTOW	156.976	2.807
YURGUN	174.095	3.27	YURGUN	160.545	3.044	YURGUN	160.464	3.345	YURGUN	165.567	3.422	YURGUN	151.125	3.037
YURNUX	159.851	2.737	YURNUX	167.752	2.751	YURGUN	162.128	3.027	YURJAA	179.295	3.356	YURJEE	179.093	3.388
YURVAM	170.586	3.413	YURVAM	151.217	3.49	YURNUX	151.501	2.811	YURNUX	164.772	2.737	YURNUX	157.749	2.751
YURVAM	154.699	2.842	YURVAM	165.441	3.078	YURVAM	151.606	3.444	YURVAM	160.89	3.455	YURVAM	168.621	3.054
YURVAM	152.726	2.985	YURVAM	166.324	2.852	YURVAM	158.404	3.238	YURVAM	159.027	3.175	YURVAM	164.412	3.039
YURVAM	152.824	3.211	YURVAM	153.342	3.139	YURVAM	169.252	3.226	YURVAM	150.956	3.252	YURVAM	170.823	2.769
YURVAM	152.827	2.914	YURVAM	154.806	3.215	YURVAM	152.319	2.794	YURVAM	173.22	2.903	YURVAM	160.055	3.362
YURVIU	150.276	2.927	YURVIU	176.2	3.377	YURVAM	152.136	2.87	YURVAM	153.793	2.794	YURVAM	151.982	2.852
YURVIU	153.833	3.139	YURVIU	154.707	2.959	YURVIU	157.843	2.962	YURVIU	150.451	2.885	YURVIU	174.406	3.003
YURVIU	159.278	2.916	YURVIU	150.308	3.21	YURVIU	177.673	2.851	YURVIU	157.625	2.772	YURVIU	150.222	3.339
YURVIU	161.338	3.063	YURVIU	158.648	2.772	YURVIU	153.998	2.882	YURVIU	167.204	2.831	YURVIU	151.969	3.433
YURVIU	167.697	3.229	YURVOA	163.367	2.912	YURVIU	166.347	2.982	YURVIU	165.309	2.885	YURVIU	157.974	3.216
YURVOA	161.247	2.912	YURVOA	159.118	3.005	YURVOA	155.771	2.897	YURVOA	161.482	2.95	YURVOA	152.172	3.005
YUSBIA	152.751	3.331	YUSBIA	159.982	3.138	YURVOA	164.22	2.95	YURVOA	170.134	2.897	YUSBIA	152.783	2.877
YUSBOG	151.344	2.807	YUSBOG	165.05	3.099	YUSBIA	155.892	3.195	YUSBIA	156.108	3.138	YUSBOG	169.122	2.701
YUSCAT	153.176	3.25	YUSSAG	162.816	3.02	YUSCAT	162.625	2.811	YUSCAT	158.793	3.038	YUSCAT	168.442	3.25
YUSSUA	163.931	3.399	YUTHOK	157.55	3.127	YUSSEK	174.4	3.099	YUSSEK	162.565	2.815	YUSSUA	152.319	2.967
YUTTAM	169.365	3.077	YUTTAM	173.148	2.614	YUTROY	165.479	3.238	YUTTAM	151.16	3.119	YUTTAM	172.848	3.022
YUTWIU	150.803	3.478	YUTWOA	155.161	3.301	YUTTEQ	174.33	2.762	YUTTEQ	161.398	3.395	YUTWIU	151.977	3.251
YUTZAQ	161.936	3.429	YUTZET	173.915	3.438	YUTZAQ	165.531	3.303	YUTZAQ	151.467	2.655	YUTZAQ	161.038	3.496
YUVQUD	161.994	2.839	YUVQUD	157.536	2.777	YUTZET	154.596	3.151	YUVDIC	153.295	3.494	YUVQUD	161.428	2.838
YUVSER	165.018	2.919	YUVSER	165.787	3.011	YUVREO	150.625	2.866	YUVREO	151.084	2.866	YUVRUG	162.346	3.438
YUVXIY	163.505	3.44	YUVXOE	170.979	2.886	YUVSER	151.503	2.916	YUVSER	151.428	3.049	YUVSER	151.916	2.927
YUWCAX	165.293	3.251	YUWCIF	161.944	3.251	YUVXOE	157.297	2.886	YUVYIB	155.574	2.839	YUWCAX	178.213	2.919
YUWJOR	155.084	2.814	YUWKAE	153.311	2.88	YUWCOL	169.411	3.33	YUWJOR	156.291	2.885	YUWJOR	154.462	3.462
YUWKEI	161.093	2.859	YUWKIM	169.395	3.355	YUWKEI	169.426	3.346	YUWKEI	150.93	3.044	YUWKEI	156.5	3.019
YUWKOS	152.112	3.244	YUWNUB	155.202	3.184	YUWKIM	159.337	2.926	YUWKIM	158.221	2.816	YUWKOS	169.183	3.303
YUWNUB	157.377	3.36	YUWNUB	153.451	2.848	YUWNUB	154.194	3.011	YUWNUB	151.839	3.384	YUWNUB	150.65	3.223
YUWNUC	160.329	3.018	YUWPAK	150.165	3.003	YUWNUB	156.206	3.141	YUWNUC	156.008	3.084	YUWNUC	150.318	3.456
YUWPAK	164.577	2.981	YUWPAK	166.019	3.364	YUWPAK	160.372	2.953	YUWPAK	159.612	2.824	YUWPAK	158.217	2.981
YUWPAK	176.526	3.182	YUWPAK	153.611	3.179	YUWPAK	153.214	3.302	YUWPAK	160.369	3.033	YUWPAK	164.042	3.29
YUWYAT0	173.088	2.691	YUWYEW	170.144	3.023	YUWPAK	163.679	2.962	YUWPAK	171.106	2.85	YUWYAT0	150.619	2.691
YUWZUO	150.463	3.375	YUWZUO	153.693	3.216	YUWYEW	170.85	3.159	YUWZOI	152.898	2.689	YUWZOI	174.134	3.268
YUYCIE	157.643	3.312	YUYKOU	152.815	2.992	YUWZUO	158.963	3.051	YUWZUO	154.267	3.217	YUWZUO	155.43	3.088
YUZSOD	157.81	3.376	YUZYAX	153.154	3.285	YUYNIS	152.487	3.364	YUYNIS	159.514	2.854	YUZCOM	152.101	3.274

ZABBAJ	173.637	2.96	ZABBAJ	165.029	3.105	YUZYAX	161.622	2.941	YUZYUR	154.392	3.397	YUZZOM	151.282	2.73
ZABLUM	161.499	3.479	ZABMAT	160.853	3.364	ZABFAJ	160.082	2.958	ZABLIA	157.756	3.373	ZABLUM	161.619	3.257
ZACFER	162.999	3.436	ZADDEN	175.556	3.086	ZABMAT	153.37	2.937	ZABQEY	166.316	3.451	ZACFER	157.521	3.482
ZADVAB01	166.392	2.742	ZADWOT	161.739	3.221	ZADKIY	160.398	3.139	ZADNIE	169.781	3.278	ZADVAB	166.432	2.77
ZAFNUS	163.911	3.279	ZAFQAC	166.637	3.289	ZADXIQ	152.94	3.428	ZAFNUS	154.099	3.463	ZAFNUS	154.523	3.394
ZAFQAY	166.999	2.956	ZAFQAY	154.583	3.209	ZAFQAC	167.758	3.324	ZAFQAC01	165.499	3.234	ZAFQAC01	167.59	3.282
ZAFQAY01	161.506	3.473	ZAFQAY01	154.583	3.209	ZAFQAY	161.506	3.473	ZAFQAY	154.979	3.217	ZAFQAY01	167.071	2.956
ZAGBOD	178.042	2.79	ZAGBOD	150.571	3.111	ZAFQAY01	154.979	3.217	ZAFXOW	158.391	3.486	ZAFXOW	159.665	2.884
ZAGDAM	153.712	3.101	ZAGGEW	153.485	2.983	ZAGBOD	163.791	3.101	ZAGBOD	165.986	2.79	ZAGBOD	163.807	3.123
ZAGREJ	169.111	3.497	ZAGSAB01	150.777	2.887	ZAGGEW	150.378	3.273	ZAGMEE	163.399	3.044	ZAGMEE	157.704	3.238
ZAGSAB01	157.012	3.237	ZAGSAB01	154.492	3.161	ZAGSAB01	152.652	3.161	ZAGSAB01	155.853	2.906	ZAGSAB01	170.427	3.006
ZAGZEM	156.048	3.019	ZAGZUC	170.869	3.125	ZAGSAB01	171.854	3.107	ZAGZEM	169.141	3.207	ZAGZEM	152.861	3.019
ZAHGAQ0	156.615	3.119	ZAHGAQ0	159.041	3.248	ZAHGAQ	160.038	2.848	ZAHGAQ	163.298	2.902	ZAHGAQ	160.804	2.848
ZAHGAQ0	159.806	3.195	ZAHGAQ0	155.309	3.073	ZAHGAQ0	155.75	3.085	ZAHGAQ0	159.52	3.208	ZAHGAQ0	155.36	3.074
ZAHGEU	150.817	2.898	ZAHGEU	154.666	3.099	ZAHGAQ0	159.813	3.193	ZAHGAQ0	155.362	3.072	ZAHGAQ0	159.733	3.193
ZAHGEU	155.241	3.122	ZAHGEU	162.961	2.763	ZAHGEU	153.045	3.152	ZAHGEU	167.75	2.814	ZAHGEU	158.137	3
ZAHWEO	157.04	3.198	ZAHWOY	150.524	3.097	ZAHGEU	153.374	2.677	ZAHGEX	166.38	3.45	ZAHHIE	153.989	2.646
ZAKGIE	178.169	3.089	ZAKGIF	162.066	3.157	ZAHYEQ	160.041	3.125	ZAJPEF	160.484	3.282	ZAJQAC	155.486	3.142
ZAKYET	165.131	2.913	ZAKYET	154.697	2.748	ZAKGUQ	166.715	3.428	ZAKGUQ	153.182	2.919	ZAKGUQ	173.064	3.208
ZALCOI	151.771	3.289	ZALDAV	152.454	3.203	ZALCIC	151.164	3.434	ZALCIC	156.979	3.449	ZALCOI	151.255	3.34
ZALDEZ	166.46	3.016	ZALDID	161.321	2.864	ZALDEZ	162.647	3.231	ZALDEZ	160.65	2.911	ZALDEZ	151.756	2.89
ZALFEA03	154.768	3.42	ZALFEA04	155.086	3.415	ZALDUP	159.559	3.17	ZALFEA	154.788	3.423	ZALFEA01	154.665	3.456
ZALJIF	155.478	3.494	ZALJIF	161.814	2.986	ZALFEA06	154.412	3.472	ZALJIF	164.271	3.144	ZALJIF	171.569	2.984
ZALJOB	170.529	3.015	ZALJOB	162.12	3.038	ZALJIG	166.487	3.305	ZALJIG	154.586	3.275	ZALJIG	175.07	3.427
ZALJOB01	169.934	2.999	ZALJOB01	154.803	3.427	ZALJOB	164.283	3.149	ZALJOB	153.523	3.47	ZALJOB01	162.482	2.98
ZALNOS	150.476	3.032	ZALPUX	154.113	3.189	ZALJOB01	164.043	2.965	ZALNOS	151.707	3.27	ZALNOS	159.645	3.155
ZALPUX	171.655	3.058	ZALPUX	151.797	2.774	ZALPUX	167.243	3.487	ZALPUX	159.673	3.487	ZALPUX	171.87	3.494
ZALTUB	167.585	2.82	ZAMBOI	150.162	2.756	ZALPUX	173.403	3.132	ZALTUB	150.662	2.554	ZALTUB	163.53	2.809
ZAMLAD	154.543	3.298	ZAMLAD	160.283	3.307	ZAMBUO	151.646	2.864	ZAMFEC	171.503	2.799	ZAMLAD	151.145	3.076
ZAMNAF	160.147	3.046	ZAMPIP	171.05	2.932	ZAMLIL	155.164	3.087	ZAMLIL	155.688	2.89	ZAMNAF	150.018	2.736
ZAMPIP	178.926	2.882	ZAMROX	165.306	3.398	ZAMPIP	150.906	3.162	ZAMPIP	165.392	3.074	ZAMPIP	166.339	2.959
ZAMROX	165.156	2.921	ZAMYAN	157.207	3.339	ZAMROX	177.044	3.141	ZAMROX	171.366	3.338	ZAMROX	161.244	3.132
ZAMYAN	153.655	2.972	ZAMYAN	160.333	2.984	ZAMYAN	153.761	3.223	ZAMYAN	166.704	3.224	ZAMYAN	153.008	3.07
ZANCIA	150.876	3.136	ZANFEC	161.811	3.466	ZAMYAN	168.117	2.91	ZAMYAN	168.054	2.859	ZAMYAN	175.152	2.91
ZANROZ	163.18	3.453	ZANZEX	151.891	3.119	ZANFEC	162.343	3.379	ZANFEC	156.417	3.252	ZANQUD	170.906	3.349
ZAPWOC	150.662	3.093	ZAPWOC	167.559	3.369	ZAPBEX	155.403	3.141	ZAPMOU	175.796	3.043	ZAPWOC	154.229	2.897
ZAQWOF	158.246	3.108	ZAQWOF	160.776	2.881	ZAQLEI	169.029	3.428	ZAQWOF	172.066	2.873	ZAQWOF	150.339	2.888
ZAQWUN	171.742	2.979	ZAQZII	151.23	2.945	ZAQWUN	162.927	2.946	ZAQWUN	173.408	3.222	ZAQWUN	159.786	3.262
ZAQZII	154.797	2.898	ZAQZII	155.596	2.857	ZAQZII	158.763	2.992	ZAQZII	151.405	2.848	ZAQZII	161.379	3.152
ZARBAZ	152.833	3.219	ZARBED	159.153	3.339	ZAQZII	152.366	2.857	ZAQZOK	170.754	3.273	ZARBAV	167.775	3.396
ZASLEK	157.094	3.058	ZASLEK	154.255	3.003	ZARTEU	165.447	3.43	ZASLAG	156.379	3.08	ZASLAG	166.35	3.405
ZASZAY	155.771	2.614	ZASZEY	173.232	2.887	ZASMUE	165.352	3.175	ZASYIE	154.788	3.254	ZASYUR	156.165	2.615
ZATQOD	158.64	3.38	ZATQOD	162.844	3.132	ZASZOM	164.634	3.007	ZATKAI	160.059	3.353	ZATKAI	153.911	3.103
ZATQOD	171.525	3.465	ZATQOD	163.4	3.406	ZATQOD	163.361	3.149	ZATQOD	167.023	3.149	ZATQOD	150.969	3.365
ZATSEU	156.861	3.401	ZATSEW	151.571	3.174	ZATQOD	166.034	3.166	ZATQOD	153.742	3.205	ZATQOD	151.42	3.251
ZAVDAD	164.191	2.856	ZAVFOR	159.247	2.877	ZATSEW	154.631	2.918	ZATWID	152.094	2.769	ZATWID	151.87	2.977
ZAVTEY	151.231	2.81	ZAVTIC	172.3	2.651	ZAVFOR	159.085	3.414	ZAVJOZ	151.998	3.207	ZAVNIV	161.687	3.434
ZAVTOI	153.957	3.176	ZAVTOI	163.625	3.486	ZAVTIC	166.709	2.651	ZAVTIC	165.409	3.31	ZAVTOI	150.199	3.372
ZAVTUO	170.073	3.014	ZAVVEB	153.998	3.008	ZAVTOI	151.667	3.007	ZAVTUO	164.08	3.016	ZAVTUO	156.734	3.009
ZAWFEI	157.185	3.076	ZAWFEI	162.474	2.975	ZAVVIE	158.569	3.093	ZAWFEI	164.724	2.936	ZAWFEI	160.992	2.944
ZAWFEI	159.926	3.183	ZAWFEI	158.789	3.301	ZAWFEI	155.886	3.129	ZAWFEI	154.026	3.109	ZAWFEI	172.98	3.28
ZAWFEI	166.744	3.396	ZAWFEI	164.252	2.968	ZAWFEI	151.601	2.807	ZAWFEI	171.544	3.159	ZAWFEI	152.03	3.103
ZAWKEQ	151.181	2.801	ZAWKEQ	151.08	3.079	ZAWFEI	158.53	3.185	ZAWFEI	166.154	3.427	ZAWKAL	161.607	2.839
ZAWKOZ	166.068	2.957	ZAWMEP	170.473	2.908	ZAWKOZ	168.672	3.32	ZAWKOZ	153.06	3.163	ZAWKOZ	151.489	2.929
ZAWVUO	155.092	2.808	ZAWVUO	171.955	2.943	ZAWMEP	179.798	3.079	ZAWMEP	151.382	3.057	ZAWVEB	150.738	3.357
ZAWWAV	166.554	2.823	ZAWWIG	177.43	2.89	ZAWVUO	165.215	2.842	ZAWVUO	169.496	2.979	ZAWVUO	158.028	2.88
ZAWWOM	158.261	3.367	ZAWWOM	161.236	2.95	ZAWWIG	156.087	3.168	ZAWWIG	159.227	3.1	ZAWWIG	153.289	2.758
ZAXREY	150.348	3.149	ZAXVIH	163.101	3.113	ZAXBIM	150.595	3.314	ZAXMIX	153.255	3.27	ZAXMUG	150.003	3.434
ZAXXED	157.683	3.427	ZAXXED	156.341	2.96	ZAXVIH	161.201	3.09	ZAXWEA	158.256	3.202	ZAXXED	154.289	3.395
ZAXXED	152.814	3.32	ZAXXIH	158.377	3.014	ZAXXED	153.719	2.716	ZAXXED	165.049	3.258	ZAXXED	159.084	2.716
ZAXXOL	174.395	3.494	ZAYFAI	158.545	3.331	ZAXXIH	152.235	2.722	ZAXXIH	167.387	3.333	ZAXXIH	157.008	2.722
ZAZHUF	174.539	3.092	ZAZHUF	168.129	2.769	ZAZHUF	169.563	2.742	ZAZHUF	166.471	3.115	ZAZHUF	167.262	3.157
ZAZTEC	155.706	2.843	ZAZTEC	161.266	3.395	ZAZHUF	153.5	3.348	ZAZHUF	152.949	3.278	ZAZTEC	153.016	3.478
ZAZXAB	152.445	3.226	ZAZXAB	163.087	3.226	ZAZTEC	174.147	3.059	ZAZTEC	155.076	2.849	ZAZTEC	150.169	3.343
ZEBDOD	169.614	2.976	ZEBDOD	172.639	3.479	ZAZYUU	163.461	2.66	ZAZZAB	163.671	2.639	ZEBDOD	164.621	2.943
ZEBNAZ	180	3.082	ZEBNED	153.692	3.441	ZEBHUM	162.304	2.793	ZEBKAU01	168.147	3.352	ZEBKIE	162.077	3.028
ZEBVEJ01	151.917	3.325	ZEBYAG	172.857	2.996	ZEBVEJ	150.655	3.2	ZEBVEJ	154.255	3.2	ZEBVEJ	152.305	3.289
ZECCAN	162.045	3.352	ZECCER	154.058	2.856	ZECCAN	154.983	2.847	ZECCAN	168.063	3.345	ZECCAN	164.858	2.759
ZECDEU	171.986	2.947	ZECDEU	172.1	3.32	ZECCER	166.92	3.357	ZECCER	165.344	2.772	ZECCER	161.258	3.407
ZECJIA	156.476	3.065	ZECPII	159.935	2.971	ZECDEU	150.05	2.999	ZECDEU	150.568	3.042	ZECFIZ	159.043	3.32
ZEDNII	164.124	2.689	ZEDWIQ	177.189	3.065	ZECQAB	166.476	2.87	ZECQOQ	158.515	3.258	ZECQOQ	161.002	3.342
ZEDWIQ	157.717	3.474	ZEDWIR	161.384	3.213	ZEDWIQ	159.052	2.874	ZEDWIQ	165.351	3.279	ZEDWIQ	163.335	3.473
ZEDXIP	151.497	3.104	ZEDXIP	157.711	3.123	ZEDWOX	168.816	3.387	ZEDXIP	169.442	2.982	ZEDXIP	176.125	2.675
ZEFKOM	157.144	2.763	ZEFKOM	160.07	2.763	ZEDYAL	152.248	3.364	ZEDYAL	151.552	3.369	ZEFKOM	157.239	3.08
ZEFWEM	152.278	3.104	ZEFWOW	151.68	3.266	ZEFLII	174.175	3.196	ZEFPOS	154.183	3.109	ZEFPOS	157.207	3.27
ZEGZAQ	153.783	3.142	ZEGZAQ	158.983	3.15	ZEFWOW	159.97	3.255	ZEGSOV	164.586	3.42	ZEGZAQ	156.6	2.847
ZEHGEB	150.201	2.995	ZEHGEB	156.761	3.301	ZEHCIB	151.768	3.292	ZEHFUR	164.297	3.11	ZEHGEB	152.252	2.8
ZEHGEB	157.671	3.222	ZEHGEB	157.896	2.85	ZEHGEB	171.283	3.05	ZEHGEB	174.822	2.875	ZEHGEB	163.145	3.075
ZEHGUS	171.761	3.453	ZEHSEK	155.558	3.403	ZEHGEB	165.114	3.05	ZEHGEB	150.257	3.094	ZEHGEB	159.518	3.156

ZEJRUF	180	3.308	ZEJSOA	172.38	2.893	ZEJMAE	154.679	3.369	ZEJMAE	178.91	3.369	ZEJRUF	174.023	2.983
ZEKGUT	152.006	3.406	ZEKGUT	153.881	3.099	ZEKGEF	173.262	3.254	ZEKGIH	164.463	3.011	ZEKGUT	163.613	3.077
ZEKHAA	151.642	2.931	ZEKHAA	157.338	2.882	ZEKGUT	151.367	2.932	ZEKHAA	154.42	2.882	ZEKHAA	152.457	3.197
ZEKHOO	154.526	3.404	ZEKHOO	170.201	2.893	ZEKHAA	166.923	2.959	ZEKHAA	166.949	3.112	ZEKHOO	150.941	3.307
ZEKHUU	158.979	3.228	ZEKHUU	159.92	3.15	ZEKHOO	162.567	3.289	ZEKHOO	170.492	3.171	ZEKHOO	150.802	3.057
ZEKJAC	151.831	2.897	ZEKJAC	150.024	2.922	ZEKHUU	162.889	2.854	ZEKJAC	150.787	3.391	ZEKJAC	161.594	2.976
ZEKVEU	160.454	3.21	ZEKVEU	161.391	2.838	ZEKJAC	166.549	3.107	ZEKVAQ	161.791	3.038	ZEKVAQ	151.228	2.809
ZELDOJ	164.429	3.018	ZELDOJ	167.797	2.828	ZEKVEU	159.161	2.981	ZEKVEU	155.064	2.775	ZEKYOH	151.782	3.102
ZEMJIO	174.331	3.391	ZEMJOT	151.207	3.299	ZELDOJ01	164.191	2.858	ZELKEG	158.052	3.304	ZEMJIO	174.705	3.392
ZEMTIU	174.239	3.41	ZEMTIU	152.866	3.382	ZEMJOT	169.404	3.428	ZEMJOT	163.682	3.297	ZEMJOT	155.75	3.245
ZENKEL	164.739	3.097	ZENKEL	155.341	3.063	ZEMTIU	154.101	2.982	ZEMXUN	158.231	3.08	ZENGOR0	150.496	3.239
ZENKUC	171.315	3.036	ZENLAJ	151.678	3.127	ZENKIP	156.423	3.331	ZENKOV	150.929	3.065	ZENKUC	165.162	3.036
ZENNEL	161.659	3.243	ZENNUE	151.783	3.288	ZENLAJ	158.086	3.429	ZENNEL	156.759	3.426	ZENNEL	159.298	3.101
ZENXAV	165.774	3.478	ZENXAV	168.613	3.322	ZENVEW	163.505	3.122	ZENXAV	158.902	3.496	ZENXAV	172.661	3.404
ZEPSUK	161.833	3.045	ZEPSUK	161.891	3.443	ZENZAX	154.367	2.886	ZENZAX	172.075	2.95	ZENZAX	158.148	2.886
ZEPTOG	159.934	3.341	ZEPTUJ	168.693	3.406	ZEPTEV	171.489	3.398	ZEPTOD	150.029	3.133	ZEPTOD	157.223	3.192
ZEPTUJ	166.443	2.965	ZEPVAR	152.628	3.306	ZEPTUJ	165.739	3.128	ZEPTUJ	150.507	3.062	ZEPTUJ	154.964	2.743
ZEPYAW	152.09	2.956	ZEPYAW	151.069	3.412	ZEPVAR	166.343	3.195	ZEPXEZ	159.855	3.164	ZEPXEZ	172.307	2.866
ZEPYIF	169.301	3.171	ZEPYIF	151.028	3.069	ZEPYAW	155.334	3.053	ZEPYIF	167.642	3.201	ZEPYIF	176.624	2.943
ZEPYIF	174.02	2.698	ZEPYIF	159.575	3.008	ZEPYIF	155.208	2.983	ZEPYIF	176.901	2.62	ZEPYIF	159.259	2.821
ZEQCOR	158.166	2.876	ZEQCOR	161.645	3.165	ZEQCOR	157.634	3.005	ZEQCOR	171.229	3.234	ZEQCOR	157.612	3.395
ZEQCOR	157.401	2.71	ZEQCOR	158.906	3.254	ZEQCOR	175.927	2.96	ZEQCOR	166.44	3.418	ZEQCOR	166.24	2.84
ZEQDAE	157.921	3.288	ZEQFAG	159.063	3.382	ZEQCOR	157.224	2.99	ZEQCOR	153.992	2.825	ZEQCOR	168.777	2.825
ZEQFAG	157.458	3.483	ZEQFAG	178.278	3.097	ZEQFAG	162.701	2.858	ZEQFAG	154.17	3	ZEQFAG	153.736	3.367
ZEQFAG	159.72	3.09	ZEQHIP	158.397	2.953	ZEQFAG	161.711	2.799	ZEQFAG	161.101	3.438	ZEQFAG	155.675	2.65
ZEQHIP	157.509	2.905	ZEQLOZ	153.659	3.35	ZEQHIP	154.879	2.953	ZEQHIP	154.733	2.914	ZEQHIP	157.162	3.141
ZEQRAQ	166.807	2.947	ZEQTOH	154.18	3.224	ZEQNOC	156.239	3.348	ZEQPIY	160.73	3.23	ZEQPOE	165.049	3.154
ZEQVAV	174.254	2.862	ZEQVAV	153.991	3.237	ZEQTOH	150.568	2.725	ZEQTOH	160.758	2.819	ZEQTOH	159.086	3.156
ZEQVAV	166.789	2.791	ZEQVAV	173.734	2.974	ZEQVAV	161.538	3.394	ZEQVAV	164.355	3.301	ZEQVAV	165.246	2.791
ZEQVAV	160.212	3.368	ZEQWUQ	151.669	3.007	ZEQVAV	153.556	2.734	ZEQVAV	154.499	3.349	ZEQVAV	159.041	3.036
ZEQXEB	172.757	2.87	ZEQXEB	150.068	2.984	ZEQWUQ	152.604	3.064	ZEQXAX	152.686	2.777	ZEQXEB	155.181	3.448
ZEQXEB	166.167	3.272	ZEQXEB	154.131	2.897	ZEQXEB	156.907	3.191	ZEQXEB	164.196	3.39	ZEQXEB	152.051	3.271
ZEQXEB	157.039	3.031	ZEQXEB	150.477	3.442	ZEQXEB	171.649	3.492	ZEQXEB	156.578	3.031	ZEQXEB	151.136	3.496
ZERJIS	168.747	3.178	ZERJOY	168.395	2.881	ZEQXEB	150.035	3.492	ZEQXEB	161.806	3.132	ZERFAH	169.577	3.493
ZERJUE	168.667	2.879	ZERJUE	156.579	3.072	ZERJOY	151.335	2.981	ZERJOY	156.177	3.074	ZERJUE	151.362	2.988
ZERKAM	163.917	3.065	ZERKAM	156.152	3.112	ZERKAM	150.321	3.301	ZERKAM	159.917	2.904	ZERKAM	166.883	2.691
ZERQUI	153.044	2.859	ZERVOK	151.823	3.307	ZERKAM	154.198	3.448	ZERKAM	150.323	3.204	ZERKAM	162.212	3.149
ZERYAZ	163.527	3.449	ZERYAZ	162.499	3.356	ZERXEB	169.394	3.461	ZERYAA	155.263	3.192	ZERYAA	160.101	3.278
ZERYAZ	150.098	2.96	ZESMUJ	159.125	3.343	ZERYAZ	164.171	3.283	ZERYAZ	164.713	3.407	ZERYAZ	167.376	3.356
ZESZOM	153.139	3.388	ZESZOM	161.616	3.008	ZESMUJ	157.574	3.238	ZESTEZ	164.28	3.203	ZESZOM	153.127	3.494
ZESZOM	154.979	3.159	ZESZOM	173.507	3.125	ZESZOM	164.661	3.058	ZESZOM	166.023	3.012	ZESZOM	150.849	2.66
ZESZUS01	161.532	2.835	ZESZUS01	164.151	2.821	ZESZUS01	155.915	2.881	ZESZUS01	150.896	2.821	ZESZUS01	176.098	2.817
ZETQUN	170.915	2.852	ZETSIC	152.934	2.783	ZESZUS01	155.762	2.821	ZESZUS01	157.102	2.9	ZETQUN	151.682	2.867
ZETSIC	159.408	3.161	ZETSIC	153.838	2.875	ZETSIC	150.34	3.007	ZETSIC	169.621	3.143	ZETSIC	150.95	3.067
ZETSIC	157.151	2.928	ZETSIC	161.675	2.903	ZETSIC	158.128	3.444	ZETSIC	156.82	3.165	ZETSIC	161.879	3.219
ZETSIC	155.868	3.25	ZETSIC	162.075	3.141	ZETSIC	152.894	3.381	ZETSIC	150.194	3.213	ZETSIC	163.704	2.799
ZETSIC	156.304	3.018	ZETTAX	151.284	3.271	ZETSIC	151.167	2.993	ZETSIC	158.061	3.288	ZETSIC	151.812	2.862
ZEWGUH	154.014	2.822	ZEWGUH	153.279	3.471	ZETTAX	154.846	3.199	ZEWGUH	154.338	3.414	ZEWGUH	154.577	3.478
ZEWHIV	164.525	3.424	ZEWKOE	171.819	2.798	ZEWGUH	153.469	3.404	ZEWGUH	157.604	3.123	ZEWGUH	169.218	3.356
ZEWQEX	157.05	3.287	ZEWQEX	151.232	3.166	ZEWQAX	153.316	3.3	ZEWQEX	170.551	3.21	ZEWQEX	159.802	2.907
ZEWQEX0	150.777	3.168	ZEXKAS	170.249	2.972	ZEWQEX0	170.617	3.21	ZEWQEX0	156.71	3.29	ZEWQEX0	159.744	2.907
ZEXZAF	150.507	3.151	ZEXZAF	163.361	3.152	ZEXSIG	168.807	3.393	ZEXWOS	150.97	3.462	ZEXZAF	156.879	3.135
ZEXZAF	160.76	3.19	ZEXZAF	171.507	3.112	ZEXZAF	158.735	3.112	ZEXZAF	178.107	3.119	ZEXZAF	170.756	2.884
ZEYDOW	155.357	3.056	ZEYDOW	162.721	3.154	ZEYDOW	165.673	3.051	ZEYDOW	165.566	3.44	ZEYDOW	164.333	2.981
ZEYJIY	172.378	3.001	ZEYLUN01	157.022	3.441	ZEYDOW	160.726	3.168	ZEYDOW	155.056	3.165	ZEYDOW	157.077	3.113
ZEZTUT	153.15	2.94	ZEZTUT01	155.918	3.205	ZEZPUS	171.654	2.87	ZEZPUS	154.529	3.146	ZEZTUT	156.646	3.124
ZIBBIZ01	168.501	3.108	ZIBBIZ01	160.099	3.322	ZEZTUT01	151.146	3.007	ZEZTUT01	155.069	3.002	ZIBBIZ	156.721	2.947
ZIBKEC	159.288	3.281	ZIBKEC	165.593	2.874	ZIBBIZ01	161.989	3.3	ZIBBUL	158.876	3.006	ZIBDEX	164.832	3.344
ZIBKEC	153.738	3.334	ZIBLIH	155.663	3.163	ZIBKEC	161.626	3.133	ZIBKEC	160.769	3.477	ZIBKEC	153.617	2.956
ZIBLIH	177.319	3.074	ZIBLIH	173.473	2.956	ZIBLIH	153.869	2.875	ZIBLIH	157.514	2.836	ZIBLIH	159.491	3.115
ZIBLIH	161.742	3.389	ZIBLIH	157.074	3.453	ZIBLIH	161.605	3.474	ZIBLIH	150.174	3.183	ZIBLIH	150.771	3.163
ZIBLON	156.275	2.836	ZIBLON	153.058	3.234	ZIBLIH	159.093	2.705	ZIBLIH	152.62	3.363	ZIBLON	154.951	2.739
ZIBLON	163.991	3.419	ZIBLON	165.88	3.322	ZIBLON	160.068	2.874	ZIBLON	152.577	3.399	ZIBLON	154.571	3.106
ZIBLUT	151.18	3.394	ZIBLUT	162.784	3.339	ZIBLON	160.099	3.438	ZIBLUT	163.305	3.34	ZIBLUT	151.613	3.404
ZIBMAA	151.19	3.253	ZIBMAA	157.046	2.809	ZIBMAA	166.777	3.407	ZIBMAA	162.144	2.915	ZIBMAA	155.725	3.411
ZIBMAA	172.923	3.138	ZIBMII	165.141	2.915	ZIBMAA	151.039	2.915	ZIBMAA	151.769	3.253	ZIBMAA	157.224	2.911
ZIBPEF	161.014	2.904	ZICBAS	174.364	2.905	ZIBMII	177.47	3.125	ZIBMII	161.493	2.915	ZIBMII	158.304	3.29
ZIDYIU	164.117	2.95	ZIDYIU	162.685	3.257	ZICBAS	171.103	3.24	ZICLOP	153.1	3.109	ZICZIW	170.413	2.983
ZIFCIA	166.245	3.24	ZIFCIA	169.931	3.403	ZIDYIU	152.499	3.237	ZIFCIA	168.489	3.309	ZIFCIA	161.245	2.868
ZIFLAD	172.816	2.963	ZIFLAD	156.808	3.067	ZIFDOK	171.79	3.27	ZIFDOK	162.137	3.377	ZIFLAD	171.351	2.934
ZIFPUC	152.943	3.492	ZIFPUC	165.802	2.856	ZIFLAD	153.375	3.316	ZIFPUC	150.211	2.715	ZIFPUC	164.253	3.193
ZIGGON	176.496	3.342	ZIGGON	155.578	3.031	ZIGGON	177.334	3.467	ZIGGON	163.702	2.911	ZIGGON	169.779	3.414
ZIGGUT	151.613	3.363	ZIGGUT	166.365	3.435	ZIGGON	158.844	3.332	ZIGGON	172.347	3.494	ZIGGUT	168.795	3.493
ZIGHOQ	151.093	3.278	ZIGHOQ	163.668	3.453	ZIGGUT	159.38	3.363	ZIGGUT	162.343	3.493	ZIGGUT	163.422	2.836
ZIGHOQ	151.422	3.405	ZIGHOQ	155.183	3.203	ZIGHOQ	154.983	2.84	ZIGHOQ	169.137	2.931	ZIGHOQ	169.302	3.405
ZIGRAM	154.632	3.066	ZIGRAM	159.581	2.865	ZIGNIM	152.839	2.978	ZIGNIM01	152.839	2.978	ZIGRAM	153.636	3.467
ZIHFOO	168.554	3.153	ZIHFUT	161.326	3.106	ZIGTIW	150.25	3.303	ZIGTIW	151.898	3.206	ZIGTIW	156.322	3.408
ZIHJOS	152.1	3.09	ZIHPAH	170.785	2.852	ZIHJEI	164.478	3.295	ZIHJOS	158.723	2.916	ZIHJOS	175.41	2.903
ZIJBOJ	150.355	3.433	ZIJBOJ	165.461	2.906	ZIHPAH	152.196	3.454	ZIHPAH	173.88	3.057	ZIHPAH	168.992	2.852

ZIJBOJ	178.57	3.228	ZIJBOJ	156.779	3.137	ZIJBOJ	153.394	3.487	ZIJBOJ	160.333	3.245	ZIJBOJ	163.392	3.334
ZIJGUX	150.341	2.891	ZIJGUX	161.33	2.891	ZIJBOJ	150.516	3.418	ZIJBOJ	152.98	3.107	ZIJBOJ	154.2	3.107
ZIJGUX	159.834	3.175	ZIJSOD	155.545	3.494	ZIJGUX	159.854	2.767	ZIJGUX	171.502	3.175	ZIJGUX	158.092	3.161
ZIJYUQ	158.762	3.111	ZIJZAW	175.014	3.317	ZIJSOD	175.614	2.817	ZIJYEX	172.369	2.908	ZIJYEX	176.159	3.055
ZIJZOH	169.563	3.473	ZIJZOH	155.646	2.971	ZIJZAW	151.244	3.483	ZIJZAW	155.905	3.121	ZIJZAW	155.049	3.184
ZIKTIY	161.103	3.28	ZIKTOF	165.892	3.084	ZIKDOO	155.361	3.069	ZIKGOR	155.533	3.221	ZIKLOY	159.181	2.932
ZILFEJ	165.768	2.88	ZILFEJ	156.989	3.255	ZIKTUL	165.582	3.133	ZIKVUO	151.678	3.281	ZIKXEW01	153.445	2.891
ZILRUH	158.772	3.017	ZILRUH	160.63	3.437	ZILFEJ	170.794	3.077	ZILPAP01	152.436	3.112	ZILPAP01	151.815	3.334
ZILWUM	156.422	3.323	ZILXEA	163.782	2.985	ZILWUM	168.398	3.256	ZILWUM	160.155	2.964	ZILWUM	159.217	3.143
ZILYUR	172.426	2.847	ZIMHUC	153.932	2.906	ZILXEA	178.814	3.439	ZILXEA	156.613	2.84	ZILYOL	163.964	3.292
ZIMJIS	152.981	2.987	ZIMJIS	166.816	3.454	ZIMHUC	162.276	3.499	ZIMJEO	167.836	2.962	ZIMJIS	159.128	3.451
ZIMMEQ	154.082	3.221	ZIMMEQ	155.793	3.282	ZIMJIS	163.836	3.286	ZIMMEQ	165.178	3.165	ZIMMEQ	177.399	3.437
ZIMPAM	165.863	3.386	ZIMPAM	156.23	3.013	ZIMMEQ	164.943	3.07	ZIMMEQ	156.046	3.371	ZIMMEQ	154.03	3.417
ZIMPAM	167.367	2.922	ZIMPAM	161.975	3.254	ZIMPAM	166.748	3.233	ZIMPAM	154.45	3.316	ZIMPAM	160.962	3.488
ZIMPAM	158.164	3.196	ZIMPAM	162.668	3.416	ZIMPAM	162.975	3.149	ZIMPAM	162.754	2.874	ZIMPAM	159.125	2.958
ZIMSOG	161.522	3.386	ZIMYAX	164.063	2.842	ZIMPAM	166.42	2.922	ZIMPAM	153.215	2.739	ZIMSAT	160.23	3.457
ZINJAK	163.14	3.499	ZINJAK	155.571	3.21	ZINGIQ	155.104	3.256	ZINHUC	163.243	3.139	ZINJAK	164.132	3.203
ZIPHUD	169.186	3.41	ZIPHUD	159.817	2.824	ZINJAK	158.61	2.892	ZINNAO	161.119	3.372	ZIPHUD	161.021	2.871
ZIPZEF	151.072	3.112	ZIPZEF	168.217	3.206	ZIPRIZ	167.074	3.262	ZIPRIZ	152.856	3.435	ZIPZEF	170.703	2.713
ZIPZEF	150.374	3.16	ZIPZEF	161.54	2.88	ZIPZEF	169.25	3.205	ZIPZEF	152.71	3.46	ZIPZEF	163.1	2.76
ZIQQEX	170.037	3.408	ZIQXIG	175.848	2.99	ZIPZEF	152.183	2.674	ZIQJOC	159.733	2.877	ZIQJOC	174.59	2.877
ZIRKAQ	158.552	3.444	ZIRKAQ	150.349	2.699	ZIQXIG	159.567	3.045	ZIQXIG	165.144	3.263	ZIQXIG	152.78	3.261
ZIRLIZ	158.428	3.365	ZIRLIZ	167.109	2.942	ZIRKIX	170.902	2.832	ZIRKOD	153.07	3.102	ZIRKUK	155.034	3.184
ZIRLOF	172.893	3.022	ZIRLOF	167.187	2.878	ZIRLIZ	152.902	3.133	ZIRLOF	153.828	3.243	ZIRLOF	175.65	3.244
ZIRMAO	150.395	2.981	ZIRMAS	166.808	3.489	ZIRLOF	152.902	2.923	ZIRLOF	154.779	3.106	ZIRLOF	163.894	3.239
ZIRSIE	167.439	3.375	ZIRVEE	150.338	3.373	ZIRMAS	153.998	2.779	ZIRNUM	161.725	3.104	ZIRNUM	163.925	2.98
ZISQAX	150.62	3.459	ZISQIB	172.919	3.054	ZIRVEF	153.504	3.303	ZISKOD	156.705	2.916	ZISQAX	156.031	2.825
ZITQAW	153.482	3.074	ZITQAW	157.441	3.342	ZISQIB	176.782	3.111	ZITKIY	173.009	3.441	ZITKIY	150.408	2.857
ZIVKEW	170.763	2.882	ZIVKEW	158.068	2.921	ZITQAW	151.564	3.342	ZITYIK	157.944	3.246	ZIVCIS	164.734	2.659
ZIWHOF	175.186	3.4	ZIWJOI	157.769	3.411	ZIVKEW	150.427	3.035	ZIVKEW	175.031	2.853	ZIVXOV	156.374	2.967
ZIWJOI	163.823	3.418	ZIWJOI	156.834	3.292	ZIWJOI	152.597	3.373	ZIWJOI	175.49	3.05	ZIWJOI	164.594	3.178
ZIXRON	150.705	3.323	ZIXWIM	157.418	3.317	ZIWZAK	172.121	3.368	ZIXCIW	155.5	3.027	ZIXKOG	156.805	2.981
ZIYFAR	150.323	3.39	ZIYFOF	153.811	3.131	ZIXYIQ	164.426	3.43	ZIYDUJ	163.363	3.199	ZIYFAR	157.96	2.746
ZIYMUS	154.787	3.179	ZIYSUX	161.78	3.43	ZIYFOF	157.606	3.331	ZIYGEX	163.564	2.861	ZIYMUS	157.105	3.349
ZIYYEN	150.765	2.896	ZIYYEN	155.902	2.755	ZIYYAJ	177.839	3.472	ZIYYAJ	176.306	3.405	ZIYYAJ	175.306	3.383
ZIZJAX	150.717	3.26	ZIZJAX	156.387	2.938	ZIYYEN	161.623	3.237	ZIZDET	151.248	3.081	ZIZGIC	160.729	3.455
ZIZJAX	151.569	3.018	ZIZJAX	152.389	3.228	ZIZJAX	163.38	2.938	ZIZJAX	157.03	3.009	ZIZJAX	167.254	3.098
ZIZNUR	150.94	3.001	ZIZNUR	171.501	3.104	ZIZNOL	157.18	3.042	ZIZNUR	155.422	3.427	ZIZNUR	155.481	3.194
ZIZXOY	158.339	2.611	ZIZXOY	153.452	3.342	ZIZVAH	150.366	2.921	ZIZXEP	150.649	3.308	ZIZXOY	150.684	2.611
ZIZYUC	156.041	2.783	ZIZYUC	155.8	3.397	ZIZYEO	152.134	3.335	ZIZYUC	161.005	3.134	ZIZYUC	169.587	2.783
ZOBSEQ	170.399	3.204	ZOBSOA	173.977	3.365	ZOBBEZ	174.761	3.398	ZOBRUF	153.475	3.417	ZOBSEQ	166.026	3.122
ZOBVUJ	155.542	3.257	ZOBVUJ	153.306	3.163	ZOBSOA	153.866	2.846	ZOBVET	177.393	3.218	ZOBVET	164.536	3.424
ZOBVUJ	155.185	3.14	ZOBVUJ	171.231	3	ZOBVUJ	154.222	3	ZOBVUJ	152.724	3.078	ZOBVUJ	154.31	3.079
ZOBWAQ	159.872	2.717	ZOBWUK	166.815	3.152	ZOBWAQ	164.454	3.119	ZOBWAQ	165.061	3.161	ZOBWAQ	166.338	2.654
ZOCLUA	157.322	3.411	ZOCLUA	156.212	3.369	ZOBXUJ	159.655	3.369	ZOBZIB	167.36	3.029	ZOCLUA	156.947	3.252
ZOCPAL	162.702	2.811	ZOCPAL	174.353	3.012	ZOCPAL	166.229	2.822	ZOCPAL	153.499	2.879	ZOCPAL	172.543	3.336
ZOCPUF	154.17	2.907	ZOCROA	169.039	3.313	ZOCPAL	167.678	2.777	ZOCPUF	154.637	2.833	ZOCPUF	171.154	3.19
ZODCON	167.828	3.04	ZODCON	157.152	2.869	ZOCROA	154.397	2.807	ZOCRUG	156.651	2.887	ZOCRUG	160.594	2.887
ZODCON	151.086	2.87	ZODCON	152.003	2.943	ZODCON	154.365	2.886	ZODCON	171.792	2.682	ZODCON	167.341	3.069
ZODCUT	175.905	2.822	ZODHUY	165.774	3.44	ZODCON	157.967	2.983	ZODCON	159.388	3.237	ZODCON	164.772	3.446
ZODRAN	174.554	2.752	ZODWOI	167.136	3.246	ZODQAM	175.162	2.804	ZODQIU	151.358	2.86	ZODRAN	153.241	3.232
ZOFJAF	162.851	3.388	ZOFJAF	155.257	2.994	ZOFBAX	154.218	2.759	ZOFBAX	150.643	3.063	ZOFJAF	153.867	2.736
ZOFJAF	161.657	3.213	ZOFJAF	167.907	2.913	ZOFJAF	153.121	3.23	ZOFJAF	167.223	3.194	ZOFJAF	154.231	3.309
ZOFJAF	158.267	3.178	ZOFJAF	162.582	3.397	ZOFJAF	161.549	2.863	ZOFJAF	163.654	3.125	ZOFJAF	157.305	2.955
ZOFTIA	157.97	3.25	ZOFTIA	177.453	2.733	ZOFJAF	167.11	2.913	ZOFPAL	156.017	2.95	ZOFPAL	161.194	3.047
ZOGGOR	162.584	3.472	ZOGGOR	150.505	2.986	ZOFTIA	160.015	3.25	ZOFXAX	150.226	3.278	ZOGBUS	159.628	2.97
ZOGGOT	161.07	2.911	ZOGGOT	159.391	3.115	ZOGGOR	166	3.089	ZOGGOR	159.892	2.881	ZOGGOT	152.204	2.86
ZOGPEQ	158.048	2.736	ZOGPEQ	175.762	2.736	ZOGGOT	158.104	3.468	ZOGGOT	168.16	2.691	ZOGGOT	175.983	2.691
ZOGPIU	171.628	3.245	ZOGPIU	168.835	3.206	ZOGPEQ	154.339	3.423	ZOGPEQ	161.48	3.403	ZOGPEQ	157.899	3.316
ZOGREU	155.139	2.996	ZOGREU	166.268	3.42	ZOGPIU	154.595	2.955	ZOGPIU	151.341	2.886	ZOGREU	152.461	2.996
ZOGVAV	164.964	3.17	ZOGVIC	151.014	3.214	ZOGTEW	167.35	3.048	ZOGTUM	159.598	3.415	ZOGTUM	151.088	2.618
ZOHFIP	156.275	3.07	ZOHFIP	168.391	3.013	ZOGVUO	157.601	2.782	ZOGYEC	177.247	3.448	ZOHFIP	151.547	3.219
ZOHGEM	160.644	2.781	ZOHGEM	163.38	3.203	ZOHFIP	154.882	3.244	ZOHFIP	152.802	2.909	ZOHFIP	156.044	3.238
ZOHGEM	174.143	3.259	ZOHGEM	161.713	2.807	ZOHGEM	156.254	2.791	ZOHGEM	155.51	2.96	ZOHGEM	161.697	2.678
ZOHGEM	164.103	3.183	ZOHGEM	151.623	3.163	ZOHGEM	157.816	3.188	ZOHGEM	163.588	2.71	ZOHGEM	173.447	3.121
ZOHGEM	167.57	2.844	ZOHGEM	165.832	3.08	ZOHGEM	154.471	2.799	ZOHGEM	152.337	2.673	ZOHGEM	152.69	2.912
ZOHGEM	151.58	2.757	ZOHGEM	158.883	2.81	ZOHGEM	150.858	3.366	ZOHGEM	158.106	2.919	ZOHGEM	177.944	3.405
ZOHXAY	157.567	3.121	ZOHXAY	161.701	3.054	ZOHGEM	166.133	3.27	ZOHGEM	176.147	3.232	ZOHPUL	169.731	3.136
ZOJBOR	162.498	2.953	ZOJBOR	165.753	2.953	ZOHXAY	156.972	3.128	ZOHZON	164.541	3.072	ZOHZON	162.145	3.223
ZOJDIL	159.716	2.935	ZOJDIL	162.49	3.026	ZOJBOR	166.18	3.171	ZOJDIL	158.142	3.2	ZOJDIL	158.118	3.026
ZOJDOR	162.969	3.083	ZOJDOR	151.411	3.228	ZOJDIL	169.944	3.136	ZOJDOR	166.954	3.17	ZOJDOR	152.891	3.17
ZOJLET	159.578	3.188	ZOJLET	163.383	3.158	ZOJDOR	168.547	3.047	ZOJHUD	152.738	3.075	ZOJLET	154.61	3.199
ZOJLIX	157.331	2.787	ZOJLIX	150.106	3.061	ZOJLIX	165.473	3.061	ZOJLIX	168.617	3.433	ZOJLIX	172.041	2.894
ZOJLOB	162.411	3.148	ZOJLOB	151.366	2.752	ZOJLIX	174.327	2.999	ZOJLIX	169.419	3.155	ZOJLIX	161.293	2.96
ZOKFIO	150.462	3.253	ZOKFIO	150.49	2.944	ZOJRUP	165.705	3.209	ZOJRUP	154.194	3.356	ZOJVEC	150.783	2.436
ZOKFIO	151.471	3.214	ZOKFIO	169.102	3.251	ZOKFIO	163.939	3.081	ZOKFIO	158.6	3.246	ZOKFIO	155.119	3.068
ZOKLET	151.759	3.304	ZOKLET	154.74	3.234	ZOKHUG	151.177	3.422	ZOKHUG	163.323	3.158	ZOKLET	167.566	2.888
ZOKRUP	157.576	3.399	ZOKRUP	154.635	3.469	ZOKROK	162.321	3.087	ZOKRUP	161.033	3.319	ZOKRUP	158.062	3.388
ZOKTAW	150.609	3.395	ZOKXOP	160.214	2.968	ZOKTAW	162.098	3.408	ZOKTAW	153.144	2.994	ZOKTAW	156.031	2.993

ZOKXOP	157.766	3.288	ZOLHIV	160.061	3.196	ZOKXOP	156.518	3.069	ZOKXOP	155.586	3.13	ZOKXOP	163.606	3.418
ZOLNEX	155.722	2.66	ZOLNEX	157.437	3.412	ZOLHIV	175.981	3.25	ZOLHIV	150.793	3.157	ZOLHIV	157.114	3.419
ZOLZUX	164.366	3.107	ZOMCAF	159.245	3.495	ZOLNEX	152.095	3.011	ZOLPEZ	154.452	3.002	ZOLSIG	166.822	3.093
ZOMQOJ	155.519	2.81	ZOMQOJ	170.991	3.436	ZOMFIS	155.806	3.41	ZOMFIS	156.717	3.194	ZOMHAO	162.212	3.39
ZOMTAA	152.882	3.415	ZOMTAA	173.99	2.89	ZOMQUP	167.242	2.947	ZOMTAA	160.584	2.889	ZOMTAA	157.784	2.92
ZOMXEH	166.055	3.076	ZOMXEH	170.336	3.076	ZOMTAA	155.983	3.081	ZOMXEH	152.338	3.124	ZOMXEH	166.899	2.849
ZOMZOT	168.067	3.027	ZOMZOT	153.982	3.29	ZOMXEH	150.424	2.899	ZOMZOT	159.741	3.173	ZOMZOT	166.321	3.41
ZONBEM	153.538	2.889	ZONBEM	152.183	3.446	ZOMZOT	159.637	2.757	ZOMZOT	152.547	3.281	ZONBEM	172.09	3.159
ZONQIG	159.953	2.92	ZONQIG	164.039	3.357	ZONQAX	166.09	3.486	ZONQAX	175.648	3.432	ZONQEB	168.326	3.226
ZONQIG	169.891	2.575	ZONRIH	163.637	3.484	ZONQIG	152.514	2.975	ZONQIG	164.104	2.92	ZONQIG	159.499	3.409
ZONXOP	172.76	3.171	ZOPDOX	167.258	3.453	ZONSIE	166.681	3.473	ZONSIE	166.593	3.317	ZONWOO	176.164	3.139
ZOPHUK	159.29	2.993	ZOPQAY	175.355	3.288	ZOPHOB	155.276	3.018	ZOPHOB	169.393	3.412	ZOPHOB	154.397	3.435
ZOPTON	150.995	3.083	ZOPTON	158.275	3.252	ZOPRII	159.557	2.888	ZOPRII	162.526	2.922	ZOPRII	165.384	3.4
ZOQCAN	161.455	2.751	ZOQCAN	161.634	2.66	ZOQBOA	154.351	2.829	ZOQBOA	156.316	3.182	ZOQCAN	152.975	3.388
ZOQFOA	162.458	3.26	ZOQFOA	165.792	3.013	ZOQCER	154.601	2.972	ZOQFOA	162.183	3.24	ZOQFOA	164.116	3.012
ZOQFUG	156.54	2.892	ZOQKEV	150.351	2.882	ZOQFUG	164.594	3.153	ZOQFUG	173.522	3.153	ZOQFUG	166.46	2.82
ZORHIB	156.189	2.846	ZORHIB	156.182	3.419	ZOQXOW	165.559	2.79	ZOQYOX	167.604	2.733	ZORHIB	151.042	3.13
ZORRIJ	154.532	2.91	ZORROP	166.189	3.237	ZORHIB	156.805	2.931	ZORHIB	153.41	2.802	ZORNUS	159.011	3.225
ZORSAE	154.6	3.085	ZORSAE	157.335	3.413	ZORROP	162.92	3.445	ZORRUW	169.445	3.111	ZORSAE	170.698	2.933
ZOSFUI	154.01	3.286	ZOSFUI	165.316	3.438	ZORSAE	172.536	3.345	ZORSAE	167.361	2.933	ZORZIR	154.141	2.814
ZOSGAP	169.71	2.96	ZOSGAP	157.338	3.051	ZOSFUI	168.617	3.464	ZOSFUI	174.328	3.365	ZOSFUI	151.288	3.094
ZOSTUY	162.451	2.897	ZOSTUY	151.869	3.138	ZOSGAP	161.648	3.051	ZOSHUM	156.824	3.098	ZOSTAE	166.089	2.998
ZOTHEV	161.674	3.243	ZOTHEV	163.518	3.493	ZOSTUY	156.54	3.149	ZOSTUY	165.905	2.958	ZOSVAG	153.017	3.168
ZOTHEV	162.757	3.154	ZOTRUX	150.563	3.042	ZOTHEV	151.739	3.078	ZOTHEV	155.16	3.319	ZOTHEV	150.943	3.201
ZOVBIY	166.492	2.833	ZOVBOE	151.679	3.326	ZOTSEK	151.592	3.295	ZOTTIP	160.607	2.931	ZOVBIY	152.524	3.184
ZOVBUK	163.363	3.349	ZOVBUK	172.189	3.11	ZOVBOE	153.97	3.326	ZOVBOE	171.786	3.102	ZOVBOE	151.607	3.468
ZOVJAY	158.7	2.787	ZOVNUW	162.708	3.111	ZOVBUK	162.935	3.04	ZOVBUK	168.748	3.349	ZOVJAY	172.453	3.491
ZOWFOK	159.763	3.015	ZOWFOK	165.065	3.069	ZOVZER	155.085	3.369	ZOWFIE	153.645	3.219	ZOWFOK	154.437	3.361
ZOWFUQ	169.549	3.061	ZOWGID	160.098	2.961	ZOWFOK	156.914	3.069	ZOWFUQ	152.455	2.91	ZOWFUQ	165.254	3.068
ZOWQEL	151.695	2.91	ZOWQEL	151.531	2.771	ZOWHEA	152.564	3.199	ZOWJAY	157.772	2.985	ZOWJAY	158.717	2.853
ZOWSIR	179.128	2.704	ZOWSOX	163.075	3.003	ZOWQEL	150.793	3.389	ZOWQEL	155.632	2.966	ZOWSIR	157.481	3.405
ZOWXAM	159.486	3.144	ZOWXAM	168.467	2.803	ZOWSUD	150.185	2.976	ZOWSUD	174.853	2.988	ZOWSUD	158.501	2.988
ZOXLIL	168.375	2.812	ZOXVEQ	154.838	3.392	ZOXLIL	171.439	2.987	ZOXLIL	166.201	2.874	ZOXLIL	155.993	2.64
ZOXYES	162.411	3.47	ZOXYES	154.443	2.945	ZOXVEQ	159.706	3.486	ZOXVEQ	150.722	3.418	ZOXVEQ	155.466	3.477
ZOYTOY	158.646	3.246	ZOYTOY	153.411	3.042	ZOXYES	150.626	2.986	ZOYJEG	160.194	2.989	ZOYJEG	166.895	3.094
ZOYWUH	151.952	3.172	ZOYWUH	155.033	3.116	ZOYVAM	150.429	3.367	ZOYVAM	174.349	3.286	ZOYWUH	151.876	3.315
ZOZQAJ	151.734	2.747	ZOZYOG	162.506	3.484	ZOYZEV	152.704	3.237	ZOZPEJ	179.498	2.696	ZOZPEJ	150.375	2.696
ZUBFUZ	161.54	3.001	ZUBFUZ	154.908	3.004	ZUBBUT	168.194	3.399	ZUBCOR	160.222	2.691	ZUBCOR	164.216	2.828
ZUBNOZ	161.286	3.272	ZUBNOZ	160.852	3.371	ZUBFUZ	171.531	3.443	ZUBNOZ	162.159	3.218	ZUBNOZ	165.204	3.218
ZUBNOZ0	154.517	3.306	ZUBNOZ0	157.317	3.357	ZUBNOZ	154.472	3.286	ZUBNOZ0	157.518	3.303	ZUBNOZ0	164.245	3.303
ZUBTUP	158.709	3.367	ZUBVAX	165.032	2.899	ZUBNOZ0	153.576	3.277	ZUBTID	166.967	3.461	ZUBTUP	162.333	3.373
ZUCVIG	169.759	2.718	ZUCVIG	164.891	3.116	ZUBVAX	158.661	2.755	ZUBVAX	163.043	2.755	ZUBVUR	155.083	2.934
ZUCVIG	152.946	3.018	ZUCVIG	152.736	3.492	ZUCVIG	153.555	2.925	ZUCVIG	173.018	2.718	ZUCVIG	166.308	3.069
ZUCVOM	160.392	3.359	ZUCVOM	155.098	3.492	ZUCVIG	162.649	3.244	ZUCVOM	156.49	3.002	ZUCVOM	152.053	3.33
ZUCWEB	164.539	2.835	ZUCXUS	150.423	3.308	ZUCVUQ	162.124	3.49	ZUCWAX	155.634	3.186	ZUCWAX	151.369	3.118
ZUCZII	159.586	3.338	ZUCZII	157.825	3.038	ZUCZII	159.647	3.256	ZUCZII	155.876	3.197	ZUCZII	157.489	2.722
ZUDDOT	150.395	2.892	ZUDDOT	174.532	3.114	ZUDCOQ	150.291	2.917	ZUDDOT	163.552	2.738	ZUDDOT	155.537	3.135
ZUDDOT0	163.757	2.736	ZUDDOT0	174.321	3.113	ZUDDOT	158.675	3.094	ZUDDOT0	150.796	2.887	ZUDDOT0	155.374	3.149
ZUDJEP	152.945	3.343	ZUDJEP	151.705	3.397	ZUDDOT0	158.59	3.105	ZUDDUZ	167.538	2.888	ZUDDUZ	165.554	3.148
ZUDKOA	160.274	3.066	ZUDKOA	172.337	3.083	ZUDJIT	162.013	2.831	ZUDJIT	163.231	3.366	ZUDKOA	150.027	3.071
ZUDKUG	152.293	2.933	ZUDLAN	158.25	3.371	ZUDKUG	158.173	3.45	ZUDKUG	158.719	3.481	ZUDKUG	153.419	2.968
ZUDLIV	161.523	3.157	ZUDLIV	155.586	3.054	ZUDLAN	158.702	3.475	ZUDLAN	156.618	3.431	ZUDLER	152.959	3.421
ZUDLUH	165.6	3.034	ZUDMAO	161.02	3.107	ZUDLIV	156.859	3.193	ZUDLUH	175.014	3.354	ZUDLUH	157.008	3.144
ZUDMAO	162.687	3.02	ZUDNEU	165.068	3.413	ZUDMAO	158.075	3.174	ZUDMAO	164.279	3.226	ZUDMAO	156.929	3.198
ZUFGIS	167.59	2.95	ZUFGIS	153.927	3.053	ZUDWEA	153.274	3.239	ZUDWEA	159.772	2.94	ZUFBAF	167.506	2.893
ZUFJIV	152.713	3.348	ZUFKAO	157.483	2.875	ZUFJER	176.834	3.036	ZUFJER	170.705	2.967	ZUFJIV	162.213	3.082
ZUFKIW	154.962	3.491	ZUFKIW	165.995	2.746	ZUFKAO	175.697	3.458	ZUFKAO	159.581	3.018	ZUFKAO	160.249	2.875
ZUFKUI	153.832	3.325	ZUFKUI	150.578	3.007	ZUFKIW	173.797	2.746	ZUFKIW	160.463	3.036	ZUFKUI	153.129	3.034
ZUFRUP	170.962	3.49	ZUFRUP	161.33	3.013	ZUFRAV	152.253	3	ZUFRAV	156.552	3.436	ZUFRAV	157.266	3.016
ZUFSIE	150.579	3.155	ZUFSIE	151.926	3.025	ZUFSAW	165.915	3.288	ZUFSIE	164.139	3.095	ZUFSIE	157.118	2.912
ZUFSUQ	156.432	3.116	ZUFTAX	155.462	3.155	ZUFSIE	151.434	2.965	ZUFSUQ	155.887	2.99	ZUFSUQ	166.906	3.221
ZUFTUR	150.12	3.247	ZUFWUU	153.295	2.711	ZUFTAX	172.631	2.761	ZUFTAX	152.881	3.067	ZUFTAX	167.284	3.072
ZUGJAO	169.708	3.044	ZUGJIW	156.8	3.462	ZUFWUV	151.038	3.283	ZUFXIJ	158.775	3.162	ZUGJAO	154.23	3.399
ZUGLAQ	158.782	3.122	ZUGNOH	151.704	3.447	ZUGLAQ	156.939	3.473	ZUGLAQ	153.954	2.97	ZUGLAQ	150.144	2.854
ZUGVAC	155.326	2.894	ZUGWUV	155.425	3.007	ZUGPIE	175.998	3.395	ZUGTUU	158.393	3.349	ZUGVAC	153.328	2.894
ZUHYIM	159.989	3.075	ZUHYIM	161.601	3.051	ZUGWUV	151.007	3.341	ZUGWUV	172.574	3.129	ZUHBAF	169.149	2.952
ZUJTON	167.431	3.454	ZUJTON	160.92	3.454	ZUHYIM	157.772	2.914	ZUJHAQ	166.998	2.818	ZUJPOJ	155.639	3.008
ZUKCAJ	167.355	2.97	ZUKCAJ	162.55	3.221	ZUJWEI	154.61	3.369	ZUKCAJ	163.925	3.262	ZUKCAJ	169.528	3.019
ZUKLEW	163.341	2.896	ZUKLEW	159.856	2.838	ZUKCAJ	160.055	3.204	ZUKCAJ	151.901	3.002	ZUKCAJ	156.408	3.221
ZULGUM	166.016	2.749	ZULTUY	158.467	3.104	ZUKLEW	160.05	2.938	ZUKQOO	152.693	3.178	ZULCIV	167.486	3.242
ZULVIO	174.845	2.827	ZULVIO	171.284	3.207	ZULTUY	171.432	3.201	ZULTUY	174.752	2.815	ZULTUY	153.023	3.298
ZULXEN	152.883	3.448	ZUMHOI	170.601	3.081	ZULVIO	158.997	3.115	ZULVIO	153.543	3.323	ZULWEI	175.66	3.455
ZUMMOM	170.443	2.637	ZUMMOM	170.118	3.053	ZUMKOK	175.671	3.145	ZUMMOM	162.807	2.667	ZUMMOM	156.832	2.804
ZUMMOM	161.928	2.919	ZUMMOM	163.202	3.441	ZUMMOM	176.366	2.941	ZUMMOM	163.109	2.838	ZUMMOM	172.987	2.905
ZUMMOM	169.64	2.637	ZUMMOM	169.798	2.941	ZUMMOM	170.585	3.101	ZUMMOM	165.2	2.804	ZUMMOM	172.411	3.05
ZUMZUE	150.825	3.316	ZUMZUE	166.627	3.432	ZUMMOM	158.299	2.633	ZUMPUV	151.466	3.469	ZUMQEG	158.696	3.453
ZUNBUH	167.341	2.892	ZUNBUH	156.833	3.006	ZUMZUE	157.045	2.977	ZUMZUE	162.183	3.119	ZUNBUH	164.351	3.289
ZUNCOA	166.539	3.123	ZUNCOA	173.783	2.729	ZUNCOA	164.376	3.208	ZUNCOA	165.51	3.434	ZUNCOA	176.463	3.042
ZUNCOC	168.236	2.924	ZUNDER	154.179	3.265	ZUNCOC	171.751	3.309	ZUNCOC	166.55	3.295	ZUNCOC	160.394	2.739

ZUNLEC	160.177	3.268	ZUNLUT	161.9	3.307	ZUNDER	151.01	3.115	ZUNDER	168.089	3.375	ZUNDER	157.3	3.028
ZUNRUZ	155.27	2.875	ZUNSEK	176.925	2.997	ZUNRUZ	161.306	3.5	ZUNRUZ	168.215	2.651	ZUNRUZ	166.132	3.226
ZUPFIC	160.283	3.046	ZUPFIC	152.61	3.076	ZUNVOV	165.327	2.912	ZUPCOF	150.401	2.781	ZUPDIA	166.303	3.271
ZUPHEB	153.224	3.464	ZUPHEB	156.809	3.176	ZUPHEB	157.349	3.334	ZUPHEB	171.45	3.019	ZUPHEB	159.107	2.833
ZUPHUR	159.284	3.428	ZUPHUR	157.392	2.835	ZUPHEB	155.443	3.399	ZUPHEB	160.48	2.773	ZUPHUR	155.812	3.187
ZUPJAZ	165.969	2.949	ZUPJAZ	175.693	2.98	ZUPHUR	171.217	3.004	ZUPHUR	154.629	3.425	ZUPHUR	157.149	2.787
ZUQFEA	173.824	3.444	ZUQFEA	151.772	3.31	ZUPJAZ	156.503	2.731	ZUPJAZ	155.409	3.374	ZUPYAN	171.21	3.469
ZUQGEA	155.456	3.297	ZUQTIS	157.257	2.822	ZUQFEA	177.162	2.716	ZUQFEA	169.673	3.144	ZUQFEA	158.219	3.281
ZURFIB	162.054	2.922	ZURFOH	156.02	3.002	ZUQVUG	153.978	3.32	ZUQZAQ	163.209	2.931	ZUQZAQ	164.334	3.232
ZURPIO	150.804	3.343	ZURWAK	162.641	3.215	ZURFOH	158.517	2.965	ZURFUN	163.817	3.325	ZURPIO	165.322	3.45
ZURWEO0	161.771	3.08	ZURWIS02	155.073	3.472	ZURWEO	161.038	3.156	ZURWEO	163.862	3.061	ZURWEO0	161.771	2.986
ZURWIS02	153.15	2.946	ZURWIS02	160.818	3.468	ZURWIS02	150.575	3.371	ZURWIS02	159.826	2.96	ZURWIS02	153.383	3.143
ZUSCID	151.038	3.001	ZUSFOI	164.846	2.99	ZURWIS02	155.603	2.936	ZURWIS02	154.573	3.211	ZUSBEX	165.932	3.206
ZUSTOZ	156.378	2.733	ZUSWIV	164.72	3.312	ZUSFOI	169.655	3.428	ZUSGUS	154.751	2.889	ZUSHIH	154.19	2.834
ZUSXET	156.692	3.027	ZUSYAP	162.541	3.183	ZUSXES	154.767	3.413	ZUSXES	164.113	2.98	ZUSXET	154.661	3.027
ZUSYER	170.503	3.136	ZUTKIL01	163.53	3.284	ZUSYAP	163.018	3.062	ZUSYAP01	163.731	3.062	ZUSYAP01	162.164	3.187
ZUTVIX	165.648	3.204	ZUTWEU	156.963	3.166	ZUTKIL01	152.919	3.235	ZUTKOR	154.958	2.857	ZUTVIX	165.94	2.907
ZUVQIQ	151.167	3.35	ZUVQIQ	164.318	3.231	ZUTWEU	165.375	3.412	ZUTWEU	159.184	2.663	ZUVQIQ	174.242	3.05
ZUVQUC	158.928	3.175	ZUVRAJ	175.765	3.347	ZUVQIQ	151.471	3.484	ZUVQIQ	159.095	3.484	ZUVQUC	168.127	3.356
ZUVRAL	152.325	2.937	ZUVRAL	153.682	3.008	ZUVRAJ	165.772	3.125	ZUVRAJ	151.286	2.922	ZUVRAJ	177.96	3.33
ZUVREN	150.703	3.193	ZUVREN	156.144	3.369	ZUVRAL	156.433	3.224	ZUVREN	154.717	2.976	ZUVREN	152.735	2.799
ZUWCEB	165.834	3.043	ZUWLUB	152.314	3.065	ZUVRIR	156.073	3.304	ZUVRIR	159.604	3.333	ZUVRIR	150.002	2.98
ZUWNAJ	163.756	3.155	ZUWNAJ	164.397	2.782	ZUWMUD	158.681	3.456	ZUWNAJ	161.383	3.406	ZUWNAJ	151.679	3.1
ZUWNAJ	157.676	3.199	ZUWNEN	164.279	2.975	ZUWNAJ	155.41	3.06	ZUWNAJ	164.861	2.991	ZUWNAJ	164.534	3.036
ZUWNEN	174.065	3.18	ZUWNEN	155.142	3.222	ZUWNEN	153.011	3.125	ZUWNEN	169.316	3.187	ZUWNEN	168.616	3.351
ZUWROC	153.45	3.146	ZUWSIX	160.699	3.079	ZUWNEN	156.28	3.256	ZUWNEN	172.634	2.923	ZUWQEP	162.509	3.058
ZUWVUM	160.603	3.374	ZUWVUM	167.12	3.264	ZUWTOC	176.691	3.189	ZUWTOC	165.964	3.062	ZUWTOC	161.937	3.437
ZUWXAU	166.119	3.049	ZUWXAU	160.372	3.08	ZUWVUM	162.568	2.907	ZUWVUM	157.688	3.058	ZUWXAU	155.413	2.851
ZUWXUN	164.753	3.186	ZUWXUN	153.399	3.429	ZUWXAU	150.084	3.407	ZUWXAU	159.433	3.121	ZUWXUN	166.087	3.038
ZUXNOV0	160.35	3.297	ZUXNUE	174.948	2.991	ZUWXUN	159.303	2.832	ZUXNAJ	154.972	2.754	ZUXNOV	160.35	3.297
ZUXQEQ	152.83	3.342	ZUXQEQ	156.998	3.194	ZUXQEQ	156.453	3.266	ZUXQEQ	158.876	3.243	ZUXQEQ	157.201	3.266
ZUXSIY	163.957	2.913	ZUXSIY	159.682	3.172	ZUXQEQ	154.69	3.328	ZUXQEQ	162.19	3.055	ZUXQEQ	162.234	3.358
ZUXTOF	162.708	3.186	ZUXTOF	154.24	2.793	ZUXSIY	151.889	3.326	ZUXTOF	159.547	3.077	ZUXTOF	158.257	3.1
ZUXTUL	155.4	2.983	ZUXYEW	150.28	3.415	ZUXTUL	155.682	2.971	ZUXTUL	152.502	2.89	ZUXTUL	154.104	2.897
ZUYFUY	173.247	3.175	ZUYFUY	152.845	2.996	ZUXYEW	165.459	3.446	ZUXYEW	151.131	3.17	ZUXYEW	150.354	2.893
ZUYGOP	164.151	3.265	ZUYKAI	157.598	2.846	ZUYGAF	161.116	3.375	ZUYGAF	155.372	3.144	ZUYGOP	155.69	3.387
ZUYLEN	164.699	3.301	ZUYPUI	155.865	3.434	ZUYKAI	160.166	2.846	ZUYLEN	163.033	2.945	ZUYLEN	175.447	2.945
ZUZBAX	152.784	2.645	ZUZCUS	156.52	3.359	ZUYQAP	153.098	3.45	ZUYWEV	150.687	3.027	ZUYXAV	158.51	2.799
ZUZKAI	159.827	2.974	ZUZKAI	156.637	3	ZUZHUB	152.193	3.47	ZUZHUB	171.079	3.101	ZUZKAI	155.333	3.23
ZUZKAI	156.11	2.906	ZUZKAI	164.71	2.8	ZUZKAI	151.668	3.294	ZUZKAI	155.928	2.958	ZUZKAI	153.589	2.657
ZUZKAI	153.353	3.434	ZUZKAI	152.612	3.118	ZUZKAI	156.832	3.461	ZUZKAI	153.758	2.799	ZUZKAI	168.763	3.294
ZUZROE	169.409	3.027	ZUZSEW	156.018	2.98	ZUZKAI	158.336	2.964	ZUZKAI	152.498	3.473	ZUZREV	164.202	3.151
ZUZTEX	153.118	3.211	ZUZTIB	154.713	2.75	ZUZSEW	152.254	2.98	ZUZSEW	165.612	3.084	ZUZSEW	163.953	3.243
ZUZVID	153.133	2.883	ZUZVID	159.263	3.197	ZUZTIB	151.947	2.825	ZUZTIB	153.115	3.271	ZUZTIB	156.385	2.75
ZZZAVM02	173.332	2.947	ZZZAVM03	172.741	2.919	ZUZVID	154.376	3.087	ZUZYOL	151.756	3.296	ZZZAVJ01	169.218	2.822
ZZZSXM01	171.864	3.466	ZZZSXW0	150.398	3.294	ZZZAVM04	172.984	2.919	ZZZSXM01	175.993	3.403	ZZZSXM01	152.666	3.324
ALIKUF	160.637	2.805	ALILUG	160.777	2.875	ZZZSXW0	176.028	3.389	AJUDIW	155.815	3.277	AJUDIW	158.004	3.308
ALURAE	153.155	3.259	AMUZAN	171.393	3.011	ALILUG	164.539	3.033	ALILUG	150.269	3.117	ALILUG	169.287	3.405
ANAGAB	156.25	3.056	ANAZAU	154.099	3.295	ANADEC	172.195	2.939	ANADEC	153.203	2.678	ANAFUU	153.687	3.418
ANEZUS	168.234	2.641	ANEZUS	168.466	2.641	ANEFAE	170.007	2.842	ANEFEI	158.246	3.309	ANEZAY	161.482	2.917
ANINEU	150.202	3.046	ANINEU	154.386	2.907	ANILIW	155.927	2.886	ANINEU	157.126	2.798	ANINEU	150.104	3.188
ANIXEE	170.811	3.241	ANIXEE	167.871	3.257	ANIXAA	153.142	3.122	ANIXAA	156.604	3.124	ANIXAA	157.935	3.128
ANOXAG	153.773	3.076	ANOXAG	161.572	2.927	ANIXII	150.371	3.444	ANOMID	175.769	3.466	ANOTOQ	150.857	3.166
ANUDEW	165.288	2.776	ANUDEW	173.976	3.07	ANOXAG	169.547	2.734	ANUDEW	158.542	3.419	ANUDEW	155.745	2.958
ANUDIA	166.545	2.971	ANUHEA	157.948	3.264	ANUDEW	166.733	3.202	ANUDEW	164.848	2.776	ANUDIA	174.426	3.171
ANUHEA	151.569	2.885	ANULOO	151.425	3.446	ANUHEA	151.493	3.23	ANUHEA	161.438	3.469	ANUHEA	166.969	2.907
APETOI	150.447	3.456	APETOI	171.001	3.299	ANUMAB	154.101	3.379	ANUSUB	152.336	3.071	APETOI	159.105	2.964
APIGIT	158.851	3.296	APOBOA	153.035	3.232	APETOI	157.182	3.039	APEXEC	155.214	2.91	APIGEP	162.836	3.238
APOBOA	156.418	2.824	APOBOA	155.206	3.023	APOBOA	151.935	3.146	APOBOA	152.25	2.95	APOBOA	157.811	2.762
APOKAV	169.431	3.361	APOKAV	159.572	3.2	APOKAV	160.813	2.909	APOKAV	151.766	2.944	APOKAV	174.167	3.256
AQAKIQ	151.105	3.225	AQAKIQ	158.305	3.179	APOKAV	151.698	3.2	APUMOR	154.022	3.459	APUTIS	151.841	3.028
AQAXID	151.935	3.276	AQAXID	161.438	3.489	AQAVUN	151.02	3.358	AQAVUN	155.745	3.075	AQAVUN	162.279	3.101
AQAXOJ	160.518	3.411	AQAXOJ	169.32	3.411	AQAXID	170.613	3.175	AQAXID	162.536	3.454	AQAXOJ	155.19	3.047
AQIGUG	162.928	3.01	AQIHIV	158.106	2.871	AQEXIH	157.392	3.142	AQIGIU	173.468	3.266	AQIGUG	155.672	2.909
AQUVIV	157.616	3.196	AQUVUH	158.098	2.965	AQIHIV	171.458	3.425	AQOPEF	167.932	3.35	AQOPEF	164.55	3.35
ARESEZ	159.639	3.175	ARESEZ	154.668	3.097	AQUVUH	166.531	2.965	ARAMOZ	150.195	2.976	ARAPIW01	156.026	3.135
AREYOP	153.814	3.282	ARIXIM	156.187	3.004	ARESEZ	154.813	3.091	AREYOP	155.568	3.3	AREYOP	166.28	3.102
ARUMIN	155.538	3.276	ARUMIN	153.072	3.405	ARUMIN	178.95	3.107	ARUMIN	153.925	2.924	ARUMIN	161.923	3.109
ASAGEK	154.714	3.477	ASEYAC	173.616	2.9	ASAGEK	153.344	3.492	ASAGEK	164.029	3.252	ASAGEK	173.621	3.252
ASISAA	153.087	3.428	ASISII	162.118	3.424	ASEYAC	159.281	3.19	ASEYAC	151.97	3.267	ASEYAC	170.748	3.187
ASODUL	161.608	3.217	ASODUL	156.845	3.374	ASISII	171.939	3.424	ASIZIP	161.658	3.259	ASOBET	170.641	3.04
ASOTEL	156.595	2.679	ASOTEL	155.902	2.679	ASOLIH	160.46	3.344	ASORIN	153.629	2.618	ASORIN	154.967	2.618
ATOHEA	161.954	2.886	ATOQEJ	155.092	3.241	ATEQUP	175.32	3.161	ATEZEI	171.424	3.465	ATIBIS	150.239	2.852
ATUDAY	158.79	3.332	ATUDAY	158.652	3.4	ATUCAX	153.952	3.3	ATUCAX	154.466	3.199	ATUCEB	151.651	2.835
ATUMOV	154.564	3.344	ATUMOV	154.226	2.894	ATUDAY	151.442	3.422	ATUMOV	168.609	3.193	ATUMOV	164.56	3.202
ATUTIW	171.028	3.394	ATUTIW	170.285	3.231	ATUROA	174.178	3.456	ATUTES	165.529	3.11	ATUTES	169.423	3.47
AVAHAK	165.861	3.335	AVAHAK	152.936	2.97	ATUVIY	161.608	3.47	ATUVIY	172.858	3.478	ATUYOH	152.657	3.273
AVAHAK	161.574	3.367	AVAHAK	161.221	3.167	AVAHAK	155.201	3.006	AVAHAK	163.319	3.448	AVAHAK	157.412	3.153
AVASUP	153.748	2.978	AVASUP	157.229	3.165	AVAHAK	167.047	3.387	AVAHAK	172.048	3.441	AVAHAK	158.193	3.162

AVIZOY	157.821	3.099	AVOHUS	158.033	3.086	AVAYAB	164.186	2.956	AVESAZ	163.591	3.071	AVIZOY	158.95	3.171
AWERON	165.119	3.213	AWERON	161.323	2.962	AVUDEE	168.117	3.399	AVUNEO	159.243	3.223	AWALOD	153.633	3.36
AWETIJ	156.33	2.816	AWETIJ	164.424	3.105	AWERON	154.923	3.193	AWERON	175.87	3.333	AWETIJ	151.251	3.175
AWETIJ	156.88	2.942	AWETIJ	166.672	2.952	AWETIJ	153.787	3.312	AWETIJ	156.736	2.816	AWETIJ	150.082	3.022
AWICES	163.304	3.181	AWICES	163.876	3.065	AWETIJ	150.109	3.393	AWETIJ	151.789	3.15	AWETIJ	154.58	2.942
AWUGEI	156.929	3.119	AXAVOO	167.236	2.673	AWICES	161.82	3.035	AWICES	156.466	3.281	AWICES	157.867	3.088
BAKPAE0	166.613	2.954	BAKPAE0	179.567	3.315	AXAVOO	163.966	3.193	AXAVOO	155.02	3.326	AXEFIW	159.264	3.293
BAKPAE0	179.617	3.316	BAKPAE1	166.396	2.952	BAKPAE0	179.698	2.992	BAKPAE0	166.613	2.955	BAKPAE0	179.612	2.993
BUNDUN0	163.811	3.154	BUNDUN0	166.461	3.353	BAKPAE1	179.692	2.989	BAKPAE1	179.735	3.31	BUNDUN0	160	3.087
BUNDUN0	164.466	3.127	BUNDUN0	166.909	3.345	BUNDUN0	176.629	3.102	BUNDUN0	164.102	3.378	BUNDUN0	160.093	3.045
BUNDUN0	166.731	2.957	BUNDUN0	155.385	3.285	BUNDUN0	175.862	3.062	BUNDUN0	163.655	3.35	BUNDUN0	164.524	2.847
CIYNUX01	166.574	3.312	CIYNUX01	163.31	2.679	BUNDUN0	154.458	2.847	CIYNUX01	171.528	3.312	CIYNUX01	169.251	3.103
CIYNUX01	162.147	2.957	COLZEM0	162.279	2.772	CIYNUX01	157.391	2.672	CIYNUX01	150.641	2.765	CIYNUX01	165.983	2.712
CUYCEF0	166.228	3.282	CUYCEF0	165.743	3.25	CUYCEF0	166.845	3.473	CUYCEF0	166.289	3.408	CUYCEF0	166.251	3.306
CUYCEF1	165.633	3.037	EMAFAD	158.144	2.94	CUYCEF1	165.885	3.187	CUYCEF1	165.198	3.141	CUYCEF1	164.92	3.084
ENAJUC	153.365	3.068	ENAJUC	159.495	2.655	EMIGEQ	153.613	3.306	ENAJUC	154.544	2.801	ENAJUC	170.323	3.301
ENAJUC	172.114	2.689	ENAJUC	151.843	2.918	ENAJUC	163.484	2.815	ENAJUC	151.063	3.073	ENAJUC	154.868	2.905
ENAJUC	151.629	3.413	ENAJUC	153.349	3.246	ENAJUC	164.736	3.034	ENAJUC	160.064	3.386	ENAJUC	171.119	3.275
ENAXUQ	173.069	2.933	ENAXUQ	167.512	2.7	ENAJUC	153.344	3.4	ENAXUQ	159.542	3.051	ENAXUQ	170.504	3.108
ENAYAX	150.342	3.006	ENISUT	156.217	3.188	ENAXUQ	156.057	3.076	ENAXUQ	170.605	3.342	ENAXUQ	151.394	2.963
ENIXAE	161.51	3.468	ENIXAE	165.169	3.304	ENIWIL	156.304	3.139	ENIWOR	178.248	3.072	ENIXAE	161.31	3.468
ENIXOS	157.918	2.783	ENIZEK	165.515	3.447	ENIXEI	165.957	3.157	ENIXEI	170.435	3.157	ENIXOS	162.663	3.441
ENIZIO	159.589	3.034	ENOYAL	168.722	2.745	ENIZEK	159.143	3.4	ENIZEK	155.353	3.235	ENIZEK	161.961	2.927
ENUTIU	162.408	3.266	ENUTIU	159.247	3.266	ENUKUX	163.317	3.13	ENUTIU	161.826	3.08	ENUTIU	169.299	2.992
EPASOH	154.617	3.202	EPASOH	171.039	2.798	EPADAE	168.197	3.49	EPADAE	164.818	3.013	EPAKEP	161.267	3.469
EPASOH	176.449	2.832	EPASUN	170.774	3.117	EPASOH	158.878	2.851	EPASOH	162.344	3.339	EPASOH	150.256	2.77
EPATUO	171.701	3.189	EPAVIE	164.976	3.389	EPASUN	162.937	3.305	EPASUN	159.89	3.327	EPASUN	171.749	2.848
EPEJIW	158.071	3.055	EPEJIW	165.074	3.044	EPAVIE	178.508	3.198	EPEGUF	168.2	3.143	EPEJIW	156.863	2.797
EPINIE	150.805	3.187	EPINIE	150.532	3.195	EPEKUJ	154.46	3.167	EPEKUJ	174.989	2.59	EPEKUJ	163.41	3.061
EPUKOT	175.19	2.975	EPUQEP	150.548	3.341	EPINIE	165.849	3.368	EPUKIN	163.605	3.457	EPUKOT	167.937	2.975
EPUQEP	153.187	2.957	EPUQUF	156.823	2.988	EPUQEP	156.716	2.789	EPUQEP	156.399	2.654	EPUQEP	160.883	2.911
EQACOS	160.811	2.801	EQACOS	159.574	2.801	EPUQUF	153.227	3.461	EQABOR	164.433	3.07	EQACOS	155.664	3.373
EQEJIX01	150.547	2.831	EQEMIA	170.337	2.793	EQAQUM	156.856	3.363	EQEJIX01	159.664	2.927	EQEJIX01	160.728	2.755
ERAKOB	157.434	2.939	ERAKOB	172.468	2.939	EQERIF	172.914	2.814	EQERIF	163.429	2.737	ERAKOB	166.767	3.094
ERIHUM	157.666	2.847	ERIWAH	159.027	2.751	ERAKOB	162.195	3.186	ERIBIU	156.32	3.263	ERICER	172.752	2.983
ERIWAH	171.201	2.919	ERIWAH	151.335	2.983	ERIWAH	168.092	3.092	ERIWAH	172.794	3.1	ERIWAH	158.712	3.175
ERIWAH	158.084	2.884	ERIWAH	170.039	2.722	ERIWAH	150.098	3.281	ERIWAH	168.184	2.735	ERIWAH	176.699	2.587
ERUFAC	161.233	2.85	ERUFAC	162.811	3.308	ERUFAC	164.313	2.849	ERUFAC	153.405	3.322	ERUFAC	171.669	3.275
ERUFAC	162.386	3.058	ESESEE	161.513	3.43	ERUFAC	154.148	3.278	ERUFAC	151.232	2.911	ERUFAC	157.496	3.199
ESEYEK	158.57	3.282	ETISIN	158.781	3.375	ESEYEK	154.984	3.093	ESEYEK	150.772	3.011	ESEYEK	163.599	2.962
ETISOT	165.792	2.953	ETITEK	156.073	3.091	ETISIN	161.239	3.053	ETISIN	152.989	3.473	ETISIN	153.038	2.96
ETOSEP	163.896	3.26	ETUBEE	169.963	3.164	ETITEK	152.071	3.091	ETOSEP	166.111	3.298	ETOSEP	171.871	3.26
ETUBEE	166.571	2.977	ETUBEE	166.086	3.366	ETUBEE	161.834	2.974	ETUBEE	163.298	2.929	ETUBEE	173.631	3.125
ETUBEE	168.342	2.847	ETUBII	156.722	2.747	ETUBEE	154.497	3.393	ETUBEE	156.022	3.176	ETUBEE	163.797	3.409
ETUBUU	172.279	3.202	ETUBUU	171.813	3.402	ETUBUU	157.671	3.004	ETUBUU	175.14	3.187	ETUBUU	156.217	3.185
ETUBUU	152.856	2.938	ETUBUU	170.963	2.938	ETUBUU	170.996	2.859	ETUBUU	150.021	2.804	ETUBUU	170.981	2.872
ETUCAB	176.588	2.858	ETUCAB	170.557	3.3	ETUBUU	173.585	2.804	ETUBUU	168.714	3.421	ETUCAB	156.617	3.099
ETUCAB	157.907	2.951	ETUCAB	172.017	3.29	ETUCAB	156.565	2.918	ETUCAB	177.143	2.843	ETUCAB	154.545	2.786
ETUGAF	173.953	3.051	ETUGAF	150.824	3.051	ETUCAB	173.679	3.494	ETUCAB	172.645	2.786	ETUCAB	170.201	2.918
EVEPUU	169.262	3.431	EVEQUV	152.16	2.909	ETUGIN	166.062	3.173	ETUGIN	174.661	3.228	EVEBOA	164.953	3.336
EVICOF	151.683	2.843	EVICOF	157.224	3.427	EVEQUV	166.211	3.151	EVEQUV	158.291	3.118	EVEZAK	158.86	2.929
EVIFEY	153.341	3.322	EVIFEY	151.78	3.323	EVIDIA01	164.516	3.132	EVIDIA01	166.869	2.894	EVIDIA01	168.86	2.894
EVITAI	152.109	2.822	EVITAI	166.902	2.9	EVIRAG	160.946	2.981	EVIREK	163.202	3.125	EVIREK	154.915	3.044
EVIVEO	176.02	3.2	EVIVEO	175.333	3.221	EVIVEO	171.538	3.305	EVIVEO	162.397	3.314	EVIVEO	171.928	2.968
EVIVOY	152.053	3.498	EVIVOY	170.838	3.102	EVIVOY	150.219	2.861	EVIVOY	151.267	3.431	EVIVOY	152.896	3.487
EVIVOY	164.253	3.012	EVIVOY	163.42	3.029	EVIVOY	163.599	3.146	EVIVOY	166.908	3.221	EVIVOY	161.08	3.272
EVIVOY	161.438	2.855	EVIVOY	164.631	2.915	EVIVOY	164.776	3.106	EVIVOY	164.243	3.138	EVIVOY	161.273	2.843
EVIZOC	170.18	3.029	EVOBEA	167.819	3.115	EVIVOY	163.809	2.916	EVIVOY	150.315	2.946	EVIZOC	167.624	3.058
EVOGOP	159.685	2.773	EVOGOP	171.167	2.856	EVOBEA	153.384	3.106	EVOGOP	176.876	3.113	EVOGOP	150.013	3.498
EWAXAF	152.549	2.974	EWAXAF	162.053	3.078	EVOZIC	172.279	2.769	EVUGEL	152.487	3.331	EVUJEO	171.06	3.037
EWIBOF	156.567	3.136	EWIBUL	176.467	3.287	EWAXAF	153.907	3.32	EWECUI	163.777	3.349	EWIBOF	161.317	3.111
EWICAS	176.991	3.286	EWICAS	171.358	3.286	EWIBUL	170.339	3.339	EWIBUL	171.139	3.287	EWICAS	168.973	3.333
FIYNIN04	168.1	3.041	FIYZUL04	160.876	3.069	EWILUV	167.334	3.408	FAGFIF01	150.843	3.498	FAGFIF01	150.969	3.47
IMIWUA	161.053	3.463	IMIWUA	161.285	3.197	HOZMIS0	173.931	2.832	HOZMIS0	162.68	2.832	IKOMEE	174.525	3.126
INULUC	161.666	2.744	INULUC	152.283	2.915	INIREG	167.672	2.646	INUHIM	155.577	2.992	INUHIM	158.458	2.965
INUWIB	172.799	3.364	INUWIB	162.007	3.084	INULUC	157.718	2.987	INUWIB	163.468	3.385	INUWIB	167.937	3.143
INUWIB	150.329	3.368	INUWIB	156.36	2.953	INUWIB	159.167	2.953	INUWIB	150.893	3.368	INUWIB	162.197	3.439
IPIBES	152.364	3.133	IPIBES	160.491	3.4	INUWIB	174.324	3.333	IPIBES	174.552	2.698	IPIBES	153.83	3.4
IPIKUR	162.654	2.946	IPOKOR	150.331	3.143	IPIBES	158.363	3.281	IPIBES	172.797	3.275	IPIKUR	154.933	3.457
IPOKOR	153.182	3.104	IPUGAF	157.513	3.333	IPOKOR	167.861	2.795	IPOKOR	151.244	3.306	IPOKOR	165.11	2.866
IQEMAW	153.658	3.218	IQEQUU0	155.78	2.756	IQAYUY	154.795	2.895	IQAYUY	156.553	3.135	IQAYUY	164.96	2.84
IQEQUU0	160.173	2.988	IQEQUU0	159.15	3.141	IQEQUU0	157.75	2.86	IQEQUU0	156.982	3.308	IQEQUU0	158.175	2.835
IQEQUU0	156.524	3.268	IQEQUU0	158.149	2.808	IQEQUU0	161.332	2.687	IQEQUU0	160.827	2.721	IQEQUU0	154.219	3.476
IQIDEV	170.892	2.962	IQIDEV	152.369	3.019	IQEQUU0	161.19	2.979	IQEQUU0	157.769	3.134	IQETOR01	172.313	3.474
IQIDEV	167.445	3.369	IQIDEV	165.412	3.477	IQIDEV	164.164	2.752	IQIDEV	171.9	3.241	IQIDEV	156.755	2.696
IQIDOF	169.73	3.346	IQIDOF	155.235	2.8	IQIDIZ	165.923	3.331	IQIDIZ	163.673	3.274	IQIDIZ	173.818	3.114
IQIDUL	150.1	3.005	IQIGOI	164.193	3.199	IQIDOF	155.187	2.865	IQIDOF	160.07	3.292	IQIDUL	175.225	2.938
IREBAM	155.482	3.273	IRELEA	165.762	2.935	IRAQIF	154.978	3.424	IRAQIF	155.11	3.424	IRAQOL	171.701	3.296
IROXEW	166.562	3.366	IROXUM	152.337	3.248	IRELEA	153.659	3.298	IROMOV	150.038	2.967	IRONAI	150.154	3.047

ISETEJ	157.986	2.845	ISETEJ	150.151	3.065	IRUQUL	151.634	3.272	IRUQUL	164.459	3.307	IRUQUL	150.875	3.268
ISIFOJ	160.769	2.916	ISIFOJ	150.108	3.198	ISETEJ	153.314	3.41	ISETEJ	166.496	3.266	ISIFID	151.219	3.237
ISIFOJ	167.466	2.952	ISIFOJ	172.481	3.198	ISIFOJ	152.037	2.98	ISIFOJ	167.402	2.994	ISIFOJ	167.425	3.418
ISIFUP	158.881	3.486	ISIFUP	169.32	2.925	ISIFOJ	153.383	3.026	ISIFUP	174.529	3.486	ISIFUP	166.822	3.487
ISIGAW	159.842	3.312	ISIGAW	166.748	3.312	ISIFUP	162.852	3.307	ISIFUP	170.852	3.089	ISIFUP	173.875	3.306
ISIRIP	160.779	2.878	ISIRIP	170.26	3.18	ISIGAW	150.015	3.28	ISIRIP	150.911	3.151	ISIRIP	156.904	2.731
ISIRIP	159.863	2.731	ISIVUF	163.422	3.342	ISIRIP	167.394	3.004	ISIRIP	178.076	2.866	ISIRIP	157.856	3.151
ISIXER	165.351	3.321	ISIYES	159.285	3.384	ISIVUF	155.89	3.093	ISIVUF	156.224	3.398	ISIXER	157.175	2.888
ITITOY	153.89	3.459	ITODEE	156.076	3.187	ISIYES	161.421	3.384	ISIZET	154.771	2.918	ISOKEK	175.35	2.945
IVATIM	155.355	3.275	IVATIM	162.056	3.124	IVATIM	150.166	3.239	IVATIM	166.303	2.915	IVATIM	156.102	3.293
IVIKOR	159.372	3.448	IVIKOR	150.566	3.005	IVATIM	173.79	2.797	IVATIM	158.121	2.809	IVATIM	165.585	2.989
IVUYEH	160.472	2.774	IVUYEH	154.226	2.774	IVIKOR	160.365	3.188	IVIKOR	168.16	2.887	IVUYEH	166.716	2.921
IWAHOH	160.984	3.242	IWICEA	163.278	3.201	IWAHIB	163.396	2.914	IWAHOH	153.963	3.258	IWAHOH	161.755	3.066
IWIREP	174.274	3.149	IWIREP	177.623	3.149	IWICEA	169.205	3.251	IWIREP	157.716	3.19	IWIREP	167.936	2.925
IWIWEU	151.124	3.458	IWIWEU	156.85	3.244	IWIREP	154.778	2.896	IWIREP	162.141	3.178	IWIREP	166.27	3.124
LAJRAU	169.158	3.16	LAJRAU	172.997	2.9	KAFPER	158.683	3.243	KIPCOE05	155.782	3.102	LAJRAU	155.275	2.845
LANLET05	151.939	3.423	NACVEX	173.158	3.446	LAJRAU	158.65	3.13	LAJRAU	167.022	3.13	LAJRAU	153.273	3.009
NINBIX01	162.871	3.247	NINBIX01	167.127	3.247	NAGQEW	163.56	3.084	NAKBOV	152.598	3.452	NAKMAS	168.885	2.665
OMEYAK	173.209	3.233	ONADIU	150.976	2.763	OMEYAK	156.687	3.256	OMEYAK	166.739	3.272	OMEYAK	150.103	3.003
ONADIU	158.071	3.225	ONADOA	157.499	3.137	ONADIU	163.319	2.903	ONADIU	151.943	3.097	ONADIU	173.339	3.331
ONADOA	154.561	3.1	ONADOA	158.701	2.978	ONADOA	170.916	3.133	ONADOA	159.319	2.768	ONADOA	161.898	3.067
ONAFIW	171.881	3.11	ONAFIW	156.435	2.849	ONADOA	171.018	3.013	ONADOA	151.33	2.718	ONAFIW	168.496	3.089
ONAFIW	157.251	3.089	ONAFIW	152.033	3.11	ONAFIW	161.749	2.979	ONAFIW	164.436	3.459	ONAFIW	164.632	3.03
ONAFIW	162.973	3.347	ONEFAS	163.019	2.936	ONAFIW	163.789	3.016	ONAFIW	161.911	2.981	ONAFIW	164.478	3.213
ONEQUX	170.759	3.462	ONEQUX	153.247	3.271	ONEQUX	150.384	2.739	ONEQUX	168.174	2.965	ONEQUX	150.108	3.145
ONEQUX	171.523	3.123	ONEQUX	151.622	3.308	ONEQUX	175.368	3.462	ONEQUX	167.518	2.874	ONEQUX	166.934	2.811
ONIKOP	169.907	3.422	ONIKOP	157.319	3.037	ONEQUX	170.644	3.328	ONIKOP	178.27	3.339	ONIKOP	152.496	3.121
ONIKOP	158.858	3.056	ONIKOP	152.743	3.037	ONIKOP	152.375	3.403	ONIKOP	163.99	3.463	ONIKOP	166.086	3.077
ONIKOP	164.597	3.087	ONIKOP	171.385	2.76	ONIKOP	170.405	3.107	ONIKOP	157.782	3.025	ONIKOP	151.253	3.025
ONIKOP	161.193	3.115	ONIKOP	166.736	2.982	ONIKOP	162.429	3.155	ONIKOP	167.009	2.998	ONIKOP	163.975	3.463
ONISUD	161.412	2.968	ONITUE	158.559	3.295	ONIKOP	175.63	3.138	ONIKOP	152.188	2.984	ONILAC	157.164	2.994
ONOYAV0	169.782	3.376	ONOYAV0	171.104	3.429	ONIVUG	167.248	2.99	ONONOY	169.016	3.471	ONOYAV	168.488	3.326
ONUPOG	159.958	2.741	ONUPOG	154.54	3.273	ONOYAV0	172.455	3.477	ONUPIA	171.043	3.072	ONUPOG	162.804	2.85
OQALOL	166.876	2.995	OQAPAB	155.251	3.232	OPOBUU	162.476	3.167	OPOLOY	157.595	3.485	OPOMAL	157.216	3.456
OQAZOZ	153.328	3.228	OQEBAR	155.903	3.095	OQAZAL	171.276	2.997	OQAZIT	172.758	3.012	OQAZOZ	154.299	2.882
OQEBIZ	160.749	3.047	OQEBIZ	175.18	2.957	OQEBAR	158.193	2.978	OQEBAR	162.187	3.375	OQEBEV	174.063	3.155
OQEGIE	160.414	2.941	OQEGIE	171.552	3.397	OQEBUL	157.591	2.921	OQEBUL	156.667	2.921	OQEBUL	166.106	3.096
OQEVOZ	156.914	3.381	OQICAW	159.05	2.933	OQEGIE	154.309	2.932	OQEGIE	159.996	2.734	OQEHEB	150.515	2.972
OQODIL	157.348	3.092	OQODIL	150.014	3.375	OQIQEO	166.36	2.87	OQIXIZ	152.781	2.986	OQIXUL	153.738	3.116
OQORIZ	167.042	3.022	OQOROF	155.745	3.327	OQODIL	159.813	3.061	OQORIZ	173.599	3.148	OQORIZ	152.559	3.101
OQOROF	166.597	3.027	OQORUL	164.246	2.876	OQOROF	167.693	3.027	OQOROF	156.661	2.968	OQOROF	156.913	3.021
OQORUL	164.692	3.163	OQOSIA	151.644	3.475	OQORUL	154.016	2.963	OQORUL	160.122	2.775	OQORUL	161.777	3.062
OQOSOG	153.343	2.765	OQOSOG	170.569	2.873	OQOSOG	150.034	3.078	OQOSOG	158.392	3.046	OQOSOG	171.344	3.482
OQOSUM	158.697	3.059	OQOSUM	150.966	3.498	OQOSOG	170.582	3.113	OQOSUM	151.914	3.053	OQOSUM	151.914	3.006
OQOTEX	160.73	3.071	OQOTIB	154.351	3.122	OQOTEX	158.081	3.489	OQOTEX	160.302	2.854	OQOTEX	153.929	3.118
OQOTIB	171.319	3.437	OQOZIH	153.367	3.12	OQOTIB	169.846	2.934	OQOTIB	150.137	3.071	OQOTIB	171.969	2.862
OQOZUT	150.496	2.981	OQOZUT	159.153	2.792	OQOZIH	166.877	2.91	OQOZIH	159.984	2.91	OQOZIH	163.209	3.217
OQUBAH	153.806	2.987	OQUBAH	152.103	3.058	OQOZUT	152.542	3.353	OQOZUT	156.899	3.353	OQUBAH	163.798	2.84
OQUBIP	160.418	3.474	OQUBIP	170.586	2.919	OQUBAH	158.872	2.865	OQUBAH	150.306	3.291	OQUBIP	161.066	2.721
OREJAA	156.073	2.66	OROCAD	168.265	3.073	OQUJAP	167.282	3.372	OQUMAS	167.245	3.251	OREJAA	157.875	3.235
OSUTUV	155.098	3.33	OSUTUV	171.749	3.351	OROQUL	162.942	3.381	OSASAG	163.633	3.085	OSIGEG	176.469	3.141
OSUTUV	151.167	3.1	OTIRES	163.892	2.79	OSUTUV	150.106	3.203	OSUTUV	155.005	2.763	OSUTUV	154.339	3.362
OTOKER	161.787	3.35	OVOJES	174.288	2.948	OTOHEO	152.241	3.072	OTOHEO	152.142	3.072	OTOKER	161.33	3.151
OVOTEC	158.448	3.492	OVOTEC	151.241	2.761	OVOTEC	150.23	3.276	OVOTEC	174.464	3.005	OVOTEC	171.291	2.627
OWAKIK	173.416	2.764	OWAKIK	167.421	3.103	OVUCUH	162.497	3.368	OVUDAO	161.522	3.38	OWAKIK	169.1	3.432
OWIFOT	158.972	3.272	OWIGAG	154.768	3.349	OWAKIK	151.984	3.077	OWAQUC	163.771	3.438	OWASEO	154.583	3.421
PFPDSE02	153.502	3.065	PFPDSE02	151.509	3.292	OWOSUS	166.958	3.349	PAFXII	165.467	3.174	PFPDSE02	159.604	2.903
QUHXAW0	164.237	2.872	TAFNOI	156.955	3.262	PFPDSE02	162.319	2.903	QUHXAW0	164.597	2.983	QUHXAW0	166.251	2.884
TFBENQ0	170.045	2.994	TFCYPH0	151.02	3.352	TALGOH	172.925	3.139	TFBENQ0	170.057	2.998	TFBENQ0	169.966	3
UCARUL0	150.297	3.283	UJEYAN	171.055	2.894	UCARUL0	173.753	2.815	UCARUL0	175.959	2.815	UCARUL0	156.485	3.017
ULIBIE	163.845	3.115	ULIBIE	150.516	3.437	ULIBIE	174.269	2.906	ULIBIE	156.108	3.468	ULIBIE	153.615	2.866
ULIBIE	150.207	3.022	ULIBIE	150.429	2.947	ULIBIE	154.962	3.138	ULIBIE	150.146	2.961	ULIBIE	153.839	2.989
UNABAQ	170.91	3.43	UNABIY	172.985	2.899	ULITES	155.408	3.01	ULORAS	157.083	3.331	UNABAQ	160.9	3.261
UNABUK	153.454	3.483	UNAFIC	165.58	2.807	UNABIY	164.894	3.459	UNABUK	167.025	2.721	UNABUK	167.307	2.885
UNICIH	163.708	2.737	UNICON	155.852	3.006	UNAFIC	168.288	2.807	UNAGEZ	156.427	3.372	UNENUA	177.09	2.703
UNIDEE	158.954	3.294	UNUDUG	179.146	3.174	UNICUT	165.075	2.645	UNICUT	157.399	2.92	UNICUT	155.751	3.09
UPAFIE	163.593	2.869	UPAFIE	155.316	3.047	UNUFAO	179.18	3.161	UNUJAS	168.176	3.426	UNULUO	155.406	3.436
UPIHOU	156.428	3.486	UPOWAB	159.454	3.146	UPAFIE	164.844	3.051	UPAJEE	165.922	3.019	UPIHOU	176.07	3.356
UPUKEZ	153.318	3.49	UPUKEZ	153.552	2.849	UPUKEZ	160.24	3.191	UPUKEZ	157.246	2.814	UPUKEZ	165.131	2.976
UPUKEZ	172.363	2.948	UPUKEZ	173.294	2.847	UPUKEZ	160.209	2.847	UPUKEZ	164.943	3.055	UPUKEZ	150.384	2.863
UQAKIK	153.685	3.149	UQAKIK	153.414	3.353	UPUPII	174.659	3.13	UQAKIK	155.309	3.418	UQAKIK	153.295	3.399
UQAMAE	176.631	2.961	UQAMAE	170.497	2.98	UQAKIK	152.468	2.878	UQAKIK	156.817	3.169	UQAKIK	154.813	2.991
UREYID	157.134	3.349	UREYID	169.075	3.216	UQAVER	158.606	3.204	URACOJ	152.229	3.442	UREFAC	156.654	3.418
USOGAO	151.355	3.035	USOGAO	151.769	3.352	USIPEV	151.401	3.235	USOGAO	156.035	3.028	USOGAO	155.756	3.442
USOGAO0	152.627	3.199	USOGAO0	152.279	2.908	USOGAO	157.507	2.781	USOGAO0	150.236	3.212	USOGAO0	156.422	2.718
USOGAO0	156.457	2.721	USOGAO0	152.508	3.2	USOGAO0	155.848	3.41	USOGAO0	157.322	2.944	USOGAO0	150.036	3.217
USOGAO0	150.023	3.253	USOGAO0	156.752	2.734	USOGAO0	152.142	2.909	USOGAO0	155.693	3.409	USOGAO0	157.227	2.945
USOGAO0	156.979	2.968	USOGAO0	156.649	2.986	USOGAO0	152.317	3.237	USOGAO0	152.031	2.944	USOGAO0	155.914	3.418
USOGAO0	157.032	2.749	USOGAO0	157.426	2.767	USOGAO0	155.771	3.424	USOGAO0	151.733	2.971	USOGAO0	152.08	3.276

USOGAO0	156.467	3.01	USOVEH0	152.906	2.725	USOGAO0	151.876	3.319	USOGAO0	151.501	3.005	USOGAO0	155.817	3.435
USOVEH0	174.374	3.405	USOVEH0	153.868	3.107	USOVEH0	153.877	3.121	USOVEH0	174.423	3.397	USOVEH0	152.785	2.718
UTIGOX	174.787	3.128	UTOKIB	177.679	2.886	UTADOM	175.153	3.207	UTADOM	172.354	3.008	UTADOM	176.52	3.17
UTOKIB	151.699	3.179	UTOKIB	156.212	2.789	UTOKIB	157.899	3.497	UTOKIB	161.986	2.861	UTOKIB	153.3	3.139
UTONAW	160.64	3.208	UTONIE	177.008	3.301	UTOKUN	150.273	3.359	UTOKUN	160.277	2.967	UTOKUN	163.2	3.234
UTULUU	173.347	2.888	UTULUU	174.388	2.902	UTOROO	151.82	2.976	UTULUU	166.565	2.414	UTULUU	167.419	2.447
UWAKUC	160.366	3.044	UWAVOH	160.29	3.481	UVUHUS	164.635	2.921	UWAJEL	165.056	3.404	UWAKUC	155.166	3.255
UWEBUX	153.062	3.105	UWEBUX	165.305	2.761	UWEBAD	155.234	3.215	UWEBOR	154.433	2.916	UWEBUX	163.804	2.942
UWEKIU	162.236	3.103	UWIGAM	172.524	2.707	UWEJEP	161.433	2.925	UWEJEP	165.298	3.386	UWEJEP	177.544	3.137
UXACOP	152.328	3.468	UXACOP	160.467	3.323	UXACIJ	153.1	3.458	UXACIJ	154.915	3.403	UXACOP	150.664	3.186
WUMHAQ	164.072	3.053	WUMHAQ	150.03	2.883	UXACOP	169.867	2.884	WEBMIB0	162.11	3.353	WEZCOT0	171.911	3.036
WUMHAQ	169.044	3.475	XAJNUW	176.132	3.477	WUMHAQ	150.055	3.159	WUMHAQ	151.02	3.425	WUMHAQ	156.865	3.424
XAJPIM	159.417	3.347	YESSOF0:	159.416	3.166	XAJNUW	174.744	2.836	XAJNUW	171.757	3.371	XAJPIM	179.459	3.216
YORCAK0	166.364	3.276	YORCAK0	157.187	3.259	YESSOF0:	166.967	2.92	YESSOF0:	165.591	2.883	YESSOF0:	156.706	3.163
ZACJAT	151.294	2.91	ZACJAT	157.287	3.361	YORCAK0	163.503	2.914	YORCAK0	165.304	3.19	ZABYIP	159.033	3.178
ZALJOB02	154.509	3.442				ZALJOB02	170.58	3.007	ZALJOB02	164.338	3.039	ZALJOB02	162.402	3
