# Supplementary material for: Negative σ-Holes on Fluorine in Molecules Revisited: Halogen Bonding or Counterintuitive, Anti-Electrostatic Interactions?
Source: Int J Mol Sci. 2026 Jul 22;27(14):6519. doi: 10.3390/ijms27146519 (PMC13410908; doi:10.3390/ijms27146519)

Table S2. Statistical distributions of (C6)centroid···F contacts extracted from the Cambridge Structural Database (CSD) including CSD ref, (C6)centroid···F distance, (C6)centroid···F–Y angle

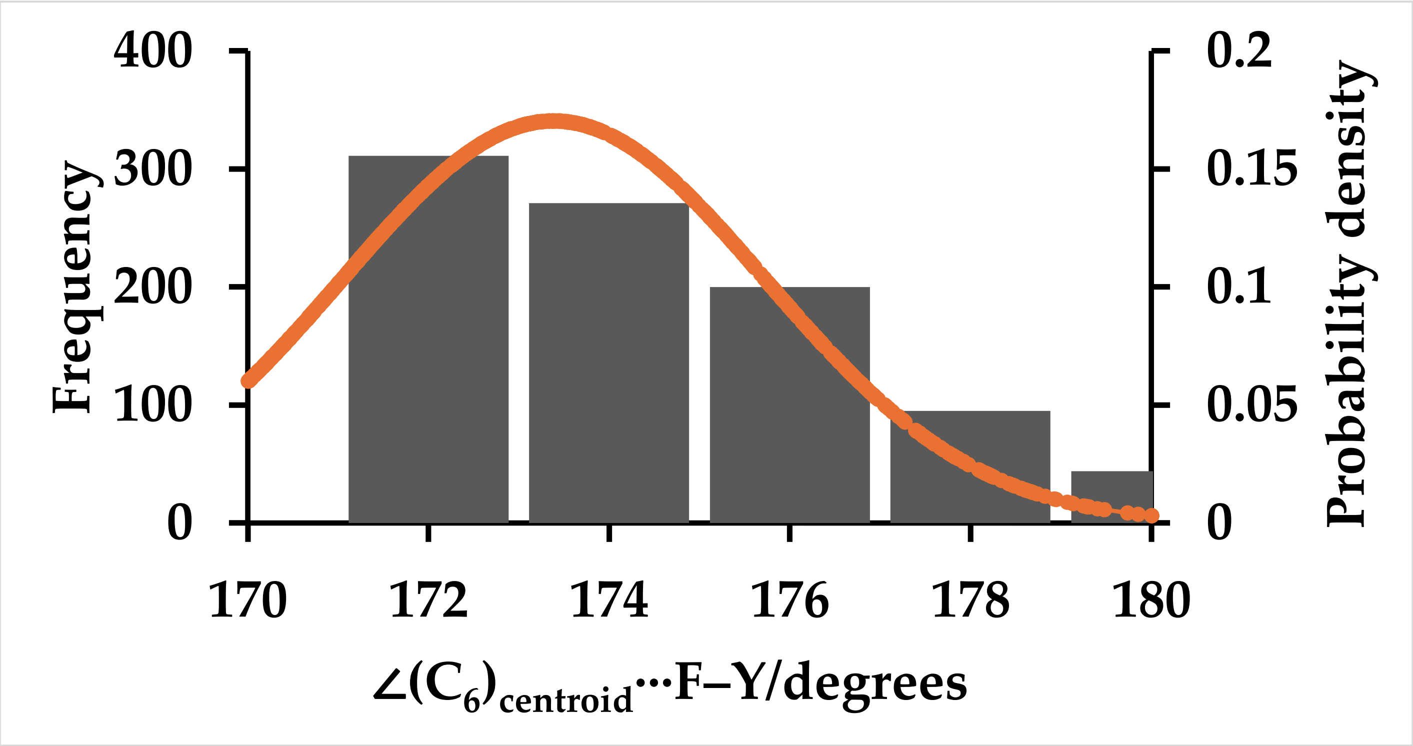

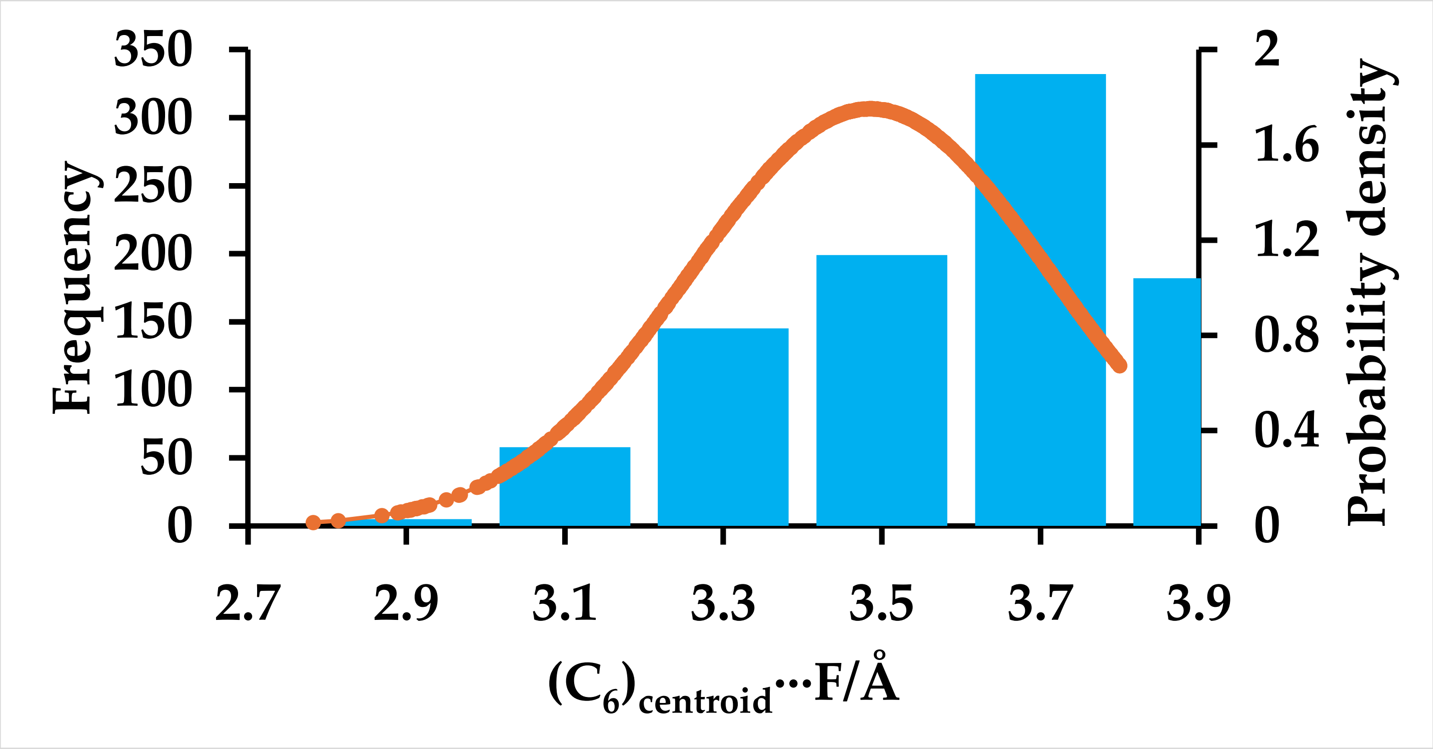

Refcode	ANG1 (A)	DIST1 (D)	Refcode	ANG1 (A)	DIST1 (D)	Refcode	ANG1 (A)	DIST1 (D)	Refcode	ANG1 (A)	DIST1 (D)
ABEVAE	172.72	3.037	ACUZEH	172.44	3.786	ADAFAP	171.483	3.796	AGILOU	170.719	3.163
AHAPAD	173.938	3.486	AHAVEL	174.799	3.783	AHIPAJ	175.907	2.889	AHOQAS	172.897	3.723
AHUCUD	170.176	3.531	AJICIF	170.939	3.31	AJIJUB	175.584	3.602	ALILEO	172.349	3.443
ANATEQ	173.481	3.766	ANIKIS	172.337	3.457	ANIPAS	172.685	3.445	ANOVEG	172.865	3.449
ANOWAE	171.625	2.922	APIWEE	170.345	3.46	APOVEK	172.788	3.711	AQAVUN	171.25	3.67
ARAWAV	172.274	3.744	AREVOL	172.383	3.682	ARUPOW	175.192	3.383	AVEYOQ	170.115	3.2
AWOWES	170.731	3.25	AWOXER	170.03	3.439	AWUCEC	172.663	3.564	AXUYID	171.179	3.251
AYEPIF01	170.845	3.218	AYUGUW	172.727	3.399	AYUGUW	173.91	3.649	AZEZAH	174.557	3.468
AZISUX	178.157	3.665	BABYIR	171.998	3.718	BACXUY	170.349	3.67	BADFAP	175.364	3.529
BADNEB	175.114	3.751	BAFYIS	171.648	3.505	BAHDEY	173.785	3.484	BAMNOW	172.14	3.426
BAPZAV	173.864	3.504	BAQYAT	172.79	3.169	BARKOY	171.45	3.689	BARMUF	170.842	3.09
BEBHEZ	171.306	3.571	BEBWAJ	170.459	3.219	BEDNUV	173.221	3.567	BEPMAO	174.044	3.631
BEPMAO	171.982	3.684	BESGAI	171.215	3.402	BEVREC	170.266	3.563	BEXCEP	170.652	3.598
BIKKAK	171.566	3.724	BIMKUG	172.019	3.628	BIVCEP	172.536	3.706	BIYSUB	175.675	3.451
BOGJAK	173.152	3.192	BOKJUK	171.789	3.632	BOQZAK	173.045	3.016	BORZIT	178.419	3.545
BTFPBU	172.536	3.769	BUFJIY	173.915	3.677	BUHVEH	172.799	3.137	BUJTEF	172.981	3.15
BULXUF	176.651	3.077	BUNWEN	170.835	3.093	BURVEQ	174.45	3.349	BUVDIJ	171.418	3.338
BUZDOS	171.737	3.737	BUZMES	171.452	3.362	CADQUU	170.787	3.698	CAFLIH	175.929	3.364
CALMIO	173.934	3.252	CARWOI	175.795	3.52	CEGROW	170.32	3.33	CEJFAB	174.802	3.566
CEKDUU	173.053	3.617	CELVOI	171.367	2.893	CEQLIW	173.208	3.715	CERTOK	173.258	3.238
CETFIT	172.683	3.65	CEXFAQ	178.686	3.604	CEZFAR	180	3	CEZFAR	180	3.095
CEZFEV	176.939	3.046	CIFROA	174.547	3.647	CIFWIA	171.481	3.206	CIHTUM	172.303	3.226
CIHZUS	172.337	3.525	CIPGIS	173.542	3.355	CIVSEH	174.423	3.662	COPGEX	178.236	3.77

COYLOT	171.931	3.758	COZGOR	172.635	3.656	CUCFOW	174.246	3.598	CUJBOC	175.218	3.111
CUKFEW	170.012	3.15	CUKQIJ	172.653	3.501	CUKQIJ01	172.735	3.51	CUKXAK	176.468	3.655
CUQYOD	170.517	3.528	CUVNUE	171.92	3.766	DADNUW	173.791	3.645	DAFMIK	175.162	3.526
DAFMOQ	172.253	3.503	DAFMUW	170.269	3.568	DAFMUW	171.11	3.515	DAGMOO	171.667	3.494
DAJYAS	173.642	3.782	DAVLAP01	175.753	3.619	DAVMUL	176.235	3.779	DEGVER	178.193	3.688
DEHQUD	172.329	3.596	DERGAL	174.085	2.782	DIBFED	175.985	3.247	DIBFED	179.737	3.284
DIBPUA	170.869	3.221	DIGPAL	174.391	3.772	DILRIB	174.907	2.914	DITXUB	170.498	3.688
DIVKOL	174.725	3.558	DIVLOL	173.672	3.189	DIVMOM	176.34	3.099	DOBFEI	172.918	3.769
DODZAX	174.534	3.251	DORWEN	172.885	3.551	DOTPEK	174.411	3.526	DOZGAC	173.537	3.77
DOZGAC	173.106	3.768	DUCWAD	171.296	3.752	DUDYAD	171.938	3.615	DUDYAD	174.864	3.703
DUGNOK	170.318	3.59	DULSUB	174.534	3.248	DURCAV	173.807	3.494	DUTWAS	175.841	3.611
DUVNAN	171.567	3.654	DUYKIU	171.279	3.572	EDEJIH	173.391	3.57	EDICID	171.895	3.642
EDOZIG	171.88	3.401	EFOTOL	172.265	3.711	EGOROL01	171.191	3.37	EHILEM	172.591	3.751
EHUQUV	171.707	3.098	EKIBAE	172.02	3.799	EKOLEW	174.524	3.331	EKUHUQ	172.667	3.467
ELAREO	174.17	3.367	ELELOA	173.446	3.185	ELOQOP	175.96	3.268	ELOQUV	172.579	3.568
ELORAC	178.554	3.048	ELUKAB	178.202	3.067	ENEXOM	171.877	3.745	ENIDAI	175.605	3.431
ENIWIL	171.972	3.201	ENUWET	172.314	3.382	EPASOH	173.89	3.125	EQOBAO	171.394	3.639
EREXUW	175.855	3.76	ETAMOB	170.205	3.313	ETAMOB	173.11	3.566	ETIMAX	171.128	3.252
ETIMAX	171.46	3.374	EVABIQ	171.319	3.568	EVUPAO	172.372	3.778	EVUVOJ	170.664	3.716
EVUWIE	173.071	3.693	EWEKAW	171.136	3.374	EWUNAL	173.006	3.694	EXOVIZ01	171.972	3.322
EYUJIU	171.546	3.305	EZECUI	178.463	3.579	EZIDEW	171.924	3.358	EZONUF	176.339	3.752
EZOWOG	176.013	3.49	FABBIY	174.665	3.497	FAGDUQ	174.546	3.69	FAHWOB	171.912	3.255
FAMBOO	170.271	3.708	FATMET	176.176	2.92	FAXXEL	172.314	3.219	FAYBOA	179.066	3.376
FAYBOA	171.769	3.626	FEFSIT	177.248	3.171	FENTOI	173.605	3.684	FESJAS	171.692	3.606
FESJEW	171.742	3.598	FESRII	177.697	3.175	FEVQUU	173.723	3.684	FEXZOZ	171.474	3.607
FEYDIY	173.52	2.912	FEZHEX	170.96	3.116	FIHTEZ	172.461	3.638	FIHZUV	171.274	3.495
FILJES	175.932	3.563	FINQEB	173.894	3.362	FIQFEU	171.339	3.538	FIRCIT	175.462	3.75
FIWDUL	171.114	3.675	FIZLAF	171.635	3.755	FOBPAR	172.485	3.503	FOBPOF	173.263	3.235
FOBZAA	171.986	3.409	FOBZUR	170.129	3.576	FOGQEA	172.454	3.502	FOGQIE	174.877	3.554
FOHFUH	170.193	3.463	FOLLIF	171.506	3.727	FONDOB	176.68	3.519	FONFEV	171.57	3.711
FONYEP	174.691	3.647	FOPMUW	172.196	3.392	FOPXAM	173.323	3.19	FOPZIT	177.197	3.765
FOYJOT	172.029	3.393	FPFAZP	175.936	3.119	FURJIP	176.619	3.582	FUZDAJ	174.856	3.745
GABWAJ	175.235	3.391	GAFDIE	170.116	3.525	GAHGUW	172.786	3.71	GAJVAT	170.608	3.543
GAJVIX	171.729	3.274	GAJWEX	175.089	3.272	GALJEI	173.335	3.784	GALLIS	172.885	3.77
GAVQAY	172.423	3.755	GAZQIL	170.111	3.587	GECHOP	170.461	3.212	GEDTOC	172.262	3.724
GEGXOG	174.117	2.927	GEHPAO	172.751	3.243	GERNID	173.337	3.657	GETJUL	177.6	3.73
GETJUL	170.049	3.611	GIGVUR	170.788	3.719	GIKZOR	170.712	3.285	GILGUH	178.672	3.711
GINFAO	175.592	3.165	GINFAO	172.782	3.507	GINFOC	176.755	3.792	GIVBEW	170.266	3.171
GIVBIA	170.17	3.183	GIWWOY	177.272	3.134	GIXHUS	170.91	3.124	GIYKIJ	170.403	3.257
GIZZAR	173.135	3.363	GIZZAR	172.598	3.24	GOKSUX	174.271	3.652	GOLJUQ	172.736	3.584
GOXDIJ	170.716	3.228	GOXDOP	170.728	3.227	GUBRIF	172.598	3.554	GUCYIM	173.443	3.179
GUFWEN	174.298	3.764	GUKTEN	174.684	3.377	GUTVUQ	173.208	3.47	GUYNIZ	172.858	3.103
GUZZOT	171.278	3.422	HADLAA	179.296	3.532	HAFBUN	171.208	3.742	HAFFUR	171.322	3.655
HAHDIF	173.944	3.299	HAQHIT	170.015	3.536	HASBOU	174.159	3.075	HASZIM	170.684	3.357
HATQEC	173.024	3.025	HAWTOR	175.117	3.734	HAYWIQ	170.385	3.671	HEKYIJ	171.442	3.67
HELGEO	170.87	3.357	HELHOZ	172.27	3.564	HEXQAG	170.409	3.551	HEXVEN	171.474	3.603
HEYJAY	175.129	3.746	HEYJAY01	174.934	3.741	HILYEJ	171.391	3.452	HIRTIO	170.358	3.305
HIYJEH	170.867	3.595	HOHZUB	175.196	3.778	HOMRIN	170.132	3.762	HOYPUH	172.071	3.539
HOYQAO	172.079	3.522	HUCKIB	174.696	3.297	HUGLEE	172.044	3.134	HUPSIU	175.779	3.194
HUXQAT	170.91	3.216	HUZLOF	173.522	3.461	HUZXEI	172.42	3.698	HUZYOU	174.268	3.614
IBABUK	177.473	3.525	IBOTOM	171.495	3.475	IBUPAZ	172.874	3.215	IBUREG	174.865	3.8
IDAKUW	174.815	3.608	IDEVAS	172.674	3.58	IDILIT	170.934	3.52	IFATUG	170.677	3.427
IFEYUP	174.695	3.36	IFEZAW	170.01	3.678	IFEZAW	174.568	3.446	IFIREU	172.629	3.501
IFUCIX	174.683	3.771	IFUFIC	175.079	3.203	IGIPAR	173.546	3.507	IGUDUJ	174.438	3.021
IGUDUJ	172.505	3.379	IHALIN	174.838	3.501	IHAXEY	175.258	3.589	IJUKOR	175.916	3.408
IJURAK	170.46	3.033	IKOMAX	172.158	3.589	ILUJEH	176.634	3.751	ILUKAE	172.134	3.062
IMIZUA	171.297	3.455	IMUCAX	173.74	3.527	IMUCIF	173.527	3.57	INEKIX	176.001	3.164
INELOE	175.562	3.376	INELOE	173.131	3.385	INELUK02	178.487	3.291	INIZOY	170.584	3.644
INOFOJ	170.262	3.757	INOVUD	177.917	3.787	IPAXUW	170.011	3.645	IPUQUG	170.177	3.697
IQAJOZ	176.168	3.271	IQAJOZ01	176.203	3.279	IQIDEV	175.126	3.783	IQIQUW	175.03	3.122
IQIQUW	173.369	3.196	IQOFON	172.589	3.744	ITAJIX	175.773	3.586	ITONUB01	172.691	3.502
ITONUB02	173.08	3.484	ITUPOD01	172.266	3.648	IVETIM	170.663	3.794	IVIYIY	174.542	3.658
IWENOP	173.601	3.38	IXEVEO	171.403	3.311	IXIZUL	175.31	3.463	IYUGIS	176.541	3.638
JAGCON	178.624	3.601	JAGHAF	170.405	3.551	JANXOM	174.924	3.251	JAVYAH	173.11	3.544
JEMVOQ	173.512	3.784	JETFOH	171.347	3.21	JEWKEF	174.993	3.189	JIDDIN	171.609	3.651
JIGVEE	171.009	3.705	JIRGID	170.026	3.627	JIYZUP	170.488	3.033	JOBQAS01	171.432	3.416
JOBXUW	175.897	3.714	JOCXEH	176.085	3.522	JOFXAE	172.138	3.02	JOTWOH	170.99	3.69
JOWHUB	171.054	3.559	JOYXAZ	176.655	3.776	JOZYEC	177.573	3.608	JUBPUV	170.359	3.478
JUDYAK	175.209	3.698	JUGHEA	172.978	3.135	JUGMAD	174.178	3.78	JUKJIL	170.777	3.647

JUNNAL	173.815	3.421	JUYBEO	172.534	3.227	JUYFAO01	172.546	3.736	KABWOD	170.917	3.432
KAJKOW	175.839	3.459	KAMVOL	175.534	3.543	KAPKAQ	171.07	3.774	KAQBOW	173.78	3.111
KASPUR	174.167	3.638	KEGBON	176.538	3.519	KEGBON	173.103	3.685	KEGMUE	172.637	3.584
KEWYOA	171.471	3.457	KEYKEE	170.823	3.676	KEYMUZ	174.439	2.911	KIJQUT	171.806	3.773
KITVOA	170.258	3.7	KOCVOP	176.341	3.555	KOKNOQ	170.809	3.06	KOQPAL	176.138	3.799
KOQPIT	175.109	3.791	KOXQOH	172.558	3.575	KUYFAP	179.121	3.777	LABKAD	173.782	3.681
LADVEV	171.53	3.317	LAJRAU	174.149	3.417	LAKNAQ	170.466	3.137	LAQFIV	170.909	3.502
LAYDOI	171.771	3.771	LETXAK01	172.588	3.35	LICMET	172.979	3.038	LICXEE	172.04	3.69
LIDHOW	177.401	3.522	LIKPUR	179.403	3.541	LIKPUR	172.752	3.231	LIRYIY	176.245	3.681
LIRZOF	172.912	3.541	LIVCOM	176.603	3.558	LOHZIT	172.527	3.457	LOHZIT01	174.311	3.38
LOHZUF	172.242	3.488	LOLMUX	173.505	3.487	LONYEW	172.755	3.391	LOPFIJ	171.037	3.654
LOXZAD	171.573	3.73	LOYXUS	178.926	3.647	LUJROX	174.803	3.577	LUJROX	171.735	3.754
LULFUU	173.681	2.906	LUVGUE	172.963	3.468	LUVJUL	172.068	2.814	LUWBEO	174.09	3.75
LUWROK	172.269	3.325	MABSOA	178.823	3.682	MAGHEK	177.538	3.636	MAMGAL	172.91	3.157
MAMLEV	173.462	3.286	MAPHUJ	171.58	3.652	MAPYOT	174.255	3.652	MASROQ	177.798	3.488
MASSEG	174.302	3.746	MAXFEA	176.547	3.581	MECGIN	171.543	3.099	MECGOT	170.248	3.101
MECPOA	176.286	3.199	MELHOE	171.691	3.722	MEMNEY	172.892	3.426	MENRIJ	178.153	3.615
MEPLPT	173.104	3.628	MEXMAF	172.517	2.869	MEYZUN	176.973	3.726	MICRAV	171.111	3.665
MIGJOE	175.482	3.334	MIHBAK	172.58	3.578	MIJBUF	175.55	3.548	MITVAQ	174.655	3.581
MIYXIE	175.409	3.737	MOCFOB	176.389	3.358	MOFJEX	178.229	3.517	MOGJIC	173.284	3.313
MOGJOL	178.611	3.59	MOSZAX	176.854	3.459	MOWRIZ	173.618	3.058	MUBFOI	172.878	3.531
MUBFOI	171.796	3.529	MUBGIB	176.666	3.694	MUCXAK	173.534	3.568	MUNVEW	174.947	3.773
MUPQOH	173.864	3.26	MUPXAX	176.481	3.335	MURGUB	170.412	3.454	MURTEB	170.676	3.68
MUSCAH	170.191	3.79	MUSCUB	171.792	3.537	MUSPAV	171.17	3.507	MUWJEV	170.846	3.696
MUYNIG	170.701	3.448	NACGOQ	173.364	3.713	NADBEZ	172.209	3.477	NAHKUD	172.33	2.901
NAJXIK	170.007	3.738	NATLIG	172.076	3.598	NAWQEJ	173.338	3.792	NAZNAH	171.783	3.494
NAZZAR	176.69	3.551	NEDSEW	174.228	3.542	NEGLAQ	174.284	3.79	NEHKES01	172.111	3.227
NEQVIQ	174.699	3.693	NERDEW	172.176	3.743	NICKUG01	170.11	3.459	NICKUG14	170.507	3.487
NIFSOO	174.628	3.562	NINHUP	171.091	3.493	NIVKEK	175.231	3.58	NIVMEO	171.489	3.35
NOCXIP	177.779	3.662	NOCZAK	173.203	3.329	NOCZAK	173.624	3.435	NOCZAK	170.779	3.561
NOCZAK	170.439	3.515	NOFTAH	172.514	3.486	NOJZES	172.002	3.054	NOPQER	172.191	3.076
NOVTUP	172.612	3.174	NOWJUG	175.433	3.57	NUBBUH	172.88	3.495	NUCNAE	174.354	3.114
NUFJIH	174.015	3.709	NUFKOQ	170.68	3.599	NUSKOE	170.234	3.112	NUTYIO	174.348	3.108
NUXVUZ	171.158	3.416	NUZREI	173.059	3.789	OBANUC	178.474	3.549	OCACII	175.56	3.674
ODUKAB	170.379	3.665	ODUSEM	177.062	2.911	OHAQUK	170.657	3.681	OJOHUR	172.859	3.157
OJUSOD	172.149	3.409	OKUWAW	171.827	3.782	OLIDEW	175.282	3.476	OLIQIM	175.714	3.504
OLOLIN	173.009	3.692	OMIQAC	170.336	3.498	ONABUE	174.034	3.696	ONIKUV	171.419	3.318
OPOLEN	173.75	3.52	OQESUZ	174.906	3.485	OQEWIT	170.036	3.8	OQUZEJ	170.517	3.133
OSITIV	175.058	3.546	OTOGOW	178.663	3.555	OTOGOW	177.485	3.612	OTOGUC	172.196	3.745
OTOZIJ	171.995	3.764	OTOZIJ01	172.122	3.767	OTUXOS	175.61	3.493	OVAREJ	170.465	3.734
OVEKUZ	173.369	3.431	OWAFEZ	171.09	3.748	OWIVOH	175.054	3.332	OXASEM	177.139	3.745
OXOKOE	174.362	2.991	OYAKOQ02	170.04	3.098	OYIBIK	171.625	3.303	OYIBIK	171.824	3.733
OYIFOT	174.197	3.208	OYIGEL	172.443	3.595	OYIMAL	170.634	3.794	OZUXIS	171.149	3.67
PABJEH	178.256	3.441	PABJEH01	175.294	3.353	PAFZAC	170.206	3.455	PAGGAH	171.605	3.355
PAGGOY	172.178	3.312	PAKTAZ	173.332	3.599	PAKYOQ	172.62	3.304	PAQGIZ	170.456	3.696
PAZJIN	175.337	3.716	PAZJOT	175.242	3.714	PEFNUL	173.819	3.768	PEJSAB	171.332	3.636
PENFOG	174.718	3.301	PEQXUH	170.695	3.2	PEYSOE	171.259	3.656	PFPDSE02	174.208	3.526
PIBTON	170.754	3.687	PIBTON	170.585	3.558	PIGRON	175.865	3.745	PIJPAB	175.83	3.71
PIKWOZ	170.312	3.673	PIKXAK	171.14	3.786	PIXLUF	170.073	3.726	PODQOS	170.39	3.364
POMCIH	172.761	3.583	PORJUD	171.898	3.759	PORKAK	172.56	3.737	PORKEO	173.536	3.705
POSGOW	172.158	2.968	POTHUC	173.181	3.661	POYGUG	175.897	3.544	POYHER	174.843	3.574
POYHER	171.336	3.552	POZWOR	172.884	3.509	PUDCUM	175.718	3.162	PUDREN	174.161	3.777
PUGPIR	177.659	3.538	PUKSAR	174.876	3.76	PUNSOI	174.602	3.481	PUSREB	174.898	3.663
PUTHAQ	176.306	3.257	PUVYUE	173.077	3.713	QACHAF	173.39	3.723	QADGUZ	172.675	3.174
QAGBAE	177.388	3.579	QAPXAK	175.591	3.517	QARHAS	175.607	3.589	QATFEZ	171.146	3.097
QAWZIX	171.873	3.652	QEKLIE	171.105	3.006	QEKWUB	175.756	3.795	QENXOA	176.317	3.738
QEXQIW	175.473	3.62	QEZMEN	172.636	3.797	QICDUF01	175.553	3.041	QICGOY	174.09	3.693
QICPAV	174.247	3.444	QIDXEJ	170.729	3.196	QIMKUS	173.37	3.409	QIMKUS	179.854	3.577
QIQQEQ	176.918	3.795	QIRNOY	171.731	3.534	QIXWIG	171.441	3.535	QOFCET	172.295	3.522
QOKBEY	172.456	3.344	QOLZOJ	179.276	3.336	QOMLIO	174.594	3.644	QOXMIA	175.414	3.578
QOXMIA	170.82	3.78	QUCXIV	171.222	3.313	QUHWIA	170.47	3.053	QUJSAS	173.447	3.306
QUMLUI	174.367	3.125	QUVWEM	174.596	3.531	QUYQEJ	172.025	3.071	QUZQUB	170.032	3.756
RADHUD	174.241	3.543	RAJCOU	170.039	3.536	RAJPOJ	176.929	3.536	RAPNED	171.281	3.699
RAQHIB	171.991	3.471	RASFUO01	179.248	3.586	RAVSAL	171.702	3.668	RAXYAQ	175.977	3.398
REKLOJ	176.506	3.027	REKLOJ	170.994	3.53	REPTEO	172.09	3.455	REQSIQ	171.729	3.466
REQSIQ	174.339	3.47	REQSOZ	171.816	3.613	RERTIT	176.95	3.775	REVSAO	170.462	3.13
REVTET	173.241	3.659	REVTOD	175.392	3.704	REVXIC	170.161	3.526	REVXUO	173.631	3.521
REVYUP	174.463	3.761	REWKUD	172.656	3.736	RICROL	172.28	3.637	RIFBIV	177.596	3.6
RIJFAU	171.426	3.512	RISSAN	170.102	3.32	RIWLEQ	172.141	3.71	RIWLEQ	175.106	3.781

RIWMOD	175.154	3.594	RIWTEA	178.19	3.653	ROQKIV	170.908	3.393	RORQEX	174.36	3.691
RUBCAU	175.237	3.074	RUDXOH	170.038	3.227	RUHXUP	177.974	3.548	RUHXUP	170.207	3.25
RUHXUP	178.733	3.038	RUPCIQ	178.225	3.686	RUSJIC	171.147	3.171	SACYEB	170.976	3.295
SAFGAM	174.701	3.272	SAKNUR	170.194	3.52	SASJIJ	173.723	3.539	SATXOD	175.529	3.302
SAWQEN	176.237	3.596	SAYJIP	176.064	3.638	SEBCOV	170.904	3.299	SEPCIB	170.116	3.117
SEPJEE	170.406	3.163	SEZKAL	173.437	3.305	SINFEE	172.414	3.314	SISHIN	173.105	3.669
SOBMUV	170.076	3.796	SOCRUY	178.087	3.758	SOKBIE	174.636	3.682	SOLQAO	174.954	3.557
SOPCUW	175.817	3.447	SOPKAL	176.887	3.182	SUBSIT	175.092	3.733	SUFKEL	172.044	3.566
SUGTIX	175.258	3.767	SUHJAK	174.159	3.446	SUSMOL	171.112	3.6	SUTREH	176.829	3.492
SUTROR	170.89	3.447	SUWVUF	177.06	3.157	TACRIZ	178.167	3.641	TAFVUS	172.028	3.667
TAGXUU01	173.374	3.627	TAPKII	170.946	3.75	TAXBIH	172.356	3.445	TEGTIJ	174.101	3.378
TEKLEA	172.559	3.369	TERDOL	172.354	3.684	TERVUL	170.294	3.021	TERWOG	171.03	3.611
TERXOH	173.079	3.698	TEWPAQ	171.342	3.422	TIBQEE	173.663	3.027	TITGEI	170.602	3.518
TITMAN	170.299	3.33	TODJEE	170.877	3.68	TOHPAL	172.74	3.593	TOHQEM	177.663	3.611
TOLQER	177.925	3.503	TOWZEO	177.83	3.529	TOZXAL	171.611	3.645	TUDDAY	170.782	3.191
TUDDEC	174.083	3.606	TUDKIN	177.433	3.483	TUSQII	173.71	3.53	TUWZOE	170.522	3.743
TUXVAJ	173.208	3.751	UBIPOM	171.955	3.774	UBISAC	173.115	3.49	UBOHEC	172.378	3.689
UBUBEC	176.481	3.281	UBUYAU	176.076	3.605	UDESUT	176.653	3.198	UHECAM	174.366	3.778
UHEFUL	172.733	3.333	UHIVAM	171.729	3.764	UHUBIJ	171.305	3.729	UHUWIG	171.589	3.634
UJAZOV	176.079	3.527	UJAZUB	170.532	3.259	UJEMUT	170.304	3.112	UJIDAV	171.213	3.785
UJONAK	172.843	2.966	ULAZAM	173.096	3.506	ULIGIJ	171.694	3.663	UMUHEP	174.524	3.634
UMUHEP01	173.454	3.489	UNAFIC	172.9	3.547	UNEFUP	179.48	3.785	UPOQUN	171.673	3.365
UPOQUN	177.821	3.572	UPUQIH	177.044	3.727	UQEVAO	171.245	3.452	UQOBEJ	174.548	3.626
UQOBIN	173.696	3.573	UQORAV	172.537	3.445	URANUY	177.86	3.568	UROCEM	174.089	3.709
URUVUB	178.596	3.638	UTEKIQ	175.593	3.258	UWIBIN	178.339	3.777	UWOHOF	171.529	3.442
UWOKOJ	171.63	3.524	UWUREM	170.368	3.445	UXICUC	177.816	3.522	UXOWOV	170.031	3.702
UYIPAW	171.93	3.583	UZESEY	174.529	3.613	VABFOW	177.756	3.444	VADPID	170.405	3.784
VAKPIK	170.462	3.705	VAMZIV	176.769	3.418	VEBPUR	171.934	3.762	VEGFAS	175.397	3.766
VEHGEY	174.863	3.642	VEHWAK	172.815	3.351	VELMIJ	171.718	3.776	VEMYIY	173.389	3.092
VEWXON	174.225	3.74	VEWYEE	177.508	3.545	VIBRUU	173.409	3.469	VIJMAG	178.229	3.449
VIKMAH	170.261	3.062	VIKMEL	179.309	3.7	VIZRUU	175.362	3.153	VIZTIH	176.006	3.533
VIZXAG	173.268	3.276	VOBTIS	171.885	3.507	VOGQOX	172.616	3.259	VOGSET	170.246	3.748
VOJLIR	176.792	3.579	VOKZUT	172.936	3.018	VOLJUE	173.561	2.95	VOLNIV	171.247	3.69
VOQDOW	170.569	3.263	VORGUI	172.301	3.657	VUBPIT	173.582	3.48	VUHSOJ	176.353	3.743
VUKHEP	172.839	3.147	VUKHEP02	171.938	3.082	VUPGIZ	171.674	3.45	VUPQON	172.775	3.254
VUVSEO	177.6	3.73	WABZIJ	177.597	3.525	WAHHOE	177.127	3.115	WAHHOE	172.105	3.458
WAJSEH	171.872	3.492	WAQWUK	173.142	3.337	WAVTEV	175.584	3.478	WAWCAA	171.617	3.43
WAWCUU	173.69	3.761	WAYNES	171.016	3.447	WEDTOO	173.132	3.768	WEGJUQ	174.79	3.261
WEGPUW	172.065	3.467	WEJYET	174.066	3.495	WEJYUJ	173.88	3.506	WEMYIY	170.713	3.152
WESYUS	175.25	3.78	WESZAZ	173.701	3.421	WEXSEB	170.799	3.354	WEZKIY	172.494	3.728
WILGIH	176.586	3.676	WITNAR	170.721	3.412	WIYBOZ	173.859	3.265	WOFDOO	177.055	3.43
WOGNOX	179.133	3.163	WOGNUD	170.234	3.142	WOGNUD	175.789	3.158	WOMZOQ	171.452	3.458
WOTFAM	174.738	3.637	WOVQIK	176.034	3.576	WOWPOR	172.119	3.447	WOXDUM	171.41	3.045
WUCXUO	174.703	3.22	WUCXUO	174.162	3.23	WUMREF	171.385	3.696	WUQPAC	173.861	3.771
WURZAL	175.331	3.74	WUSLUS	176.347	3.699	WUVPOT	178.944	3.244	WUZKOR	175.796	3.19
XABQEB	170.17	3.709	XACXEJ	170.1	3.785	XADTOO	171.225	3.374	XAFXEK	173.628	3.761
XAKNUW	172.765	3.793	XANNAB	171.857	3.358	XAPKUY	172.249	3.168	XARQOA	174.467	3.71
XARZOG	171.831	3.379	XATWUL	171.358	3.485	XAWHUC	171.004	3.633	XAWMER	173.247	3.77
XAWMIS	170.786	3.778	XECLUQ	174.942	3.457	XENFAA	178.096	3.713	XENFIH	173.313	3.787
XERDEF	175.749	3.371	XERYOK	170.345	2.905	XESFIM	172.46	3.582	XEXQAW	171.577	2.929
XICWEM	173.029	3.226	XIGYAR	174.696	3.179	XIMHEK	172.405	3.118	XINLUE	171.708	3.556
XIQSAT	170.001	3.458	XOCYAS	176.449	3.614	XODKEI	177.089	3.528	XOHSIZ	172.812	3.387
XOHVAU	171.011	3.156	XOLPET	175.4	3.609	XOLPUJ	172.144	3.229	XONVII	170.141	3.463
XOTSOO	176.087	3.797	XOVVUB	172.911	3.577	XOWSUB	170.922	3.675	XOWZOB	176.348	3.55
XOXBAQ	175.545	3.533	XOXBEU	172.459	3.334	XOXDEW	176.359	3.528	XOZHIG	173.017	3.696
XUHJES	172.411	3.554	XUNYIR	176.716	3.436	XUPKOJ	174.039	3.639	XUXZOI	178.124	3.145
YACMOI	170.045	3.326	YACRUR	176.926	3.173	YAPJEF	170.174	3.239	YAQZOJ	172.033	3.263
YAWWAX	174.243	3.333	YEBTEF	170.94	3.597	YEHQEJ	171.725	3.625	YEJCOG	170.68	2.989
YEYNAU	176.013	3.207	YEZDOZ	170.539	3.755	YEZFAN	171.478	3.314	YIRDAH	178.556	3.792
YIRSOI	173.078	3.501	YITRUO	174.379	3.724	YITSOJ	172.839	3.752	YIWZIN	171.336	3.25
YOBCEB	172.654	3.654	YOFDEF	171.088	3.357	YOGGUX	174.433	3.149	YOGGUX	173.706	3.165
YOPBIQ	170.21	3.679	YORYUB	173.419	3.66	YORZIQ	174.577	3.75	YOSFOF	173.813	3.116
ZABHOD	174.81	3.755	ZAHVEM	173.008	3.772	ZAMBUO	170.103	3.393	ZAMQUC	170.8	3.508
ZAMROX	173.16	3.153	ZAMROX	171.785	3.182	ZAXYAC	175.566	3.113	ZEPYAU	175.464	3.553
ZEVQUP	174.143	3.481	ZEYYAF	175.868	3.147	ZIBNUV	173.184	3.742	ZISNIC	174.123	3.43
ZIVCIS	170.45	3.602	ZIVKEW	174.185	3.389	ZODCON	175.51	3.77	ZOHDOT	170.824	3.066
ZOHGOW	174.508	3.246	ZOHZUT	172.836	3.58	ZOLHIV	173.556	3.788	ZOMZUZ	170.836	3.392
ZONRIH02	172.826	3.543	ZUDLIV	175.047	3.448	ZUFYUY	170.211	3.793	ZUJLEZ	174.626	3.343
ZUMMOM	172.586	3.574	ZUPHEB	176.829	3.688	ZUPHUR	175.791	3.707	ZUWNEN	171.375	3.671

ZUYGAF	171.293	3.68										

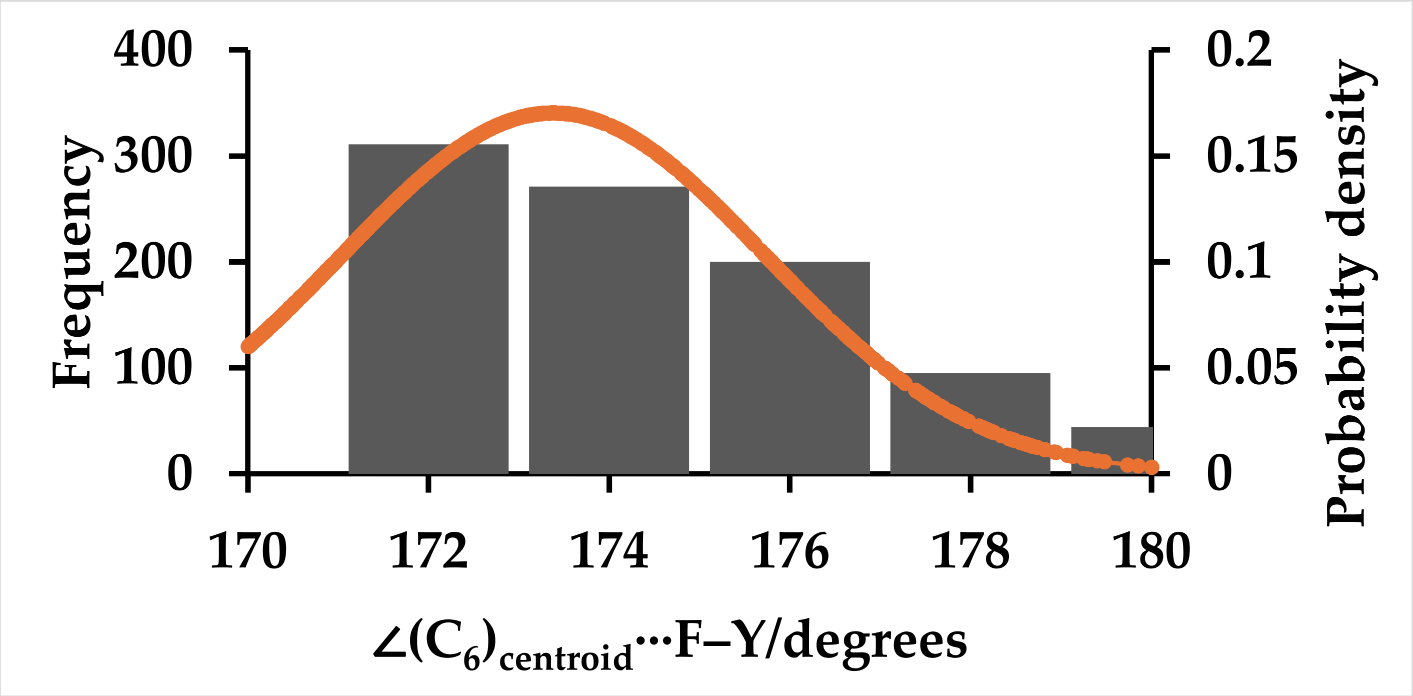

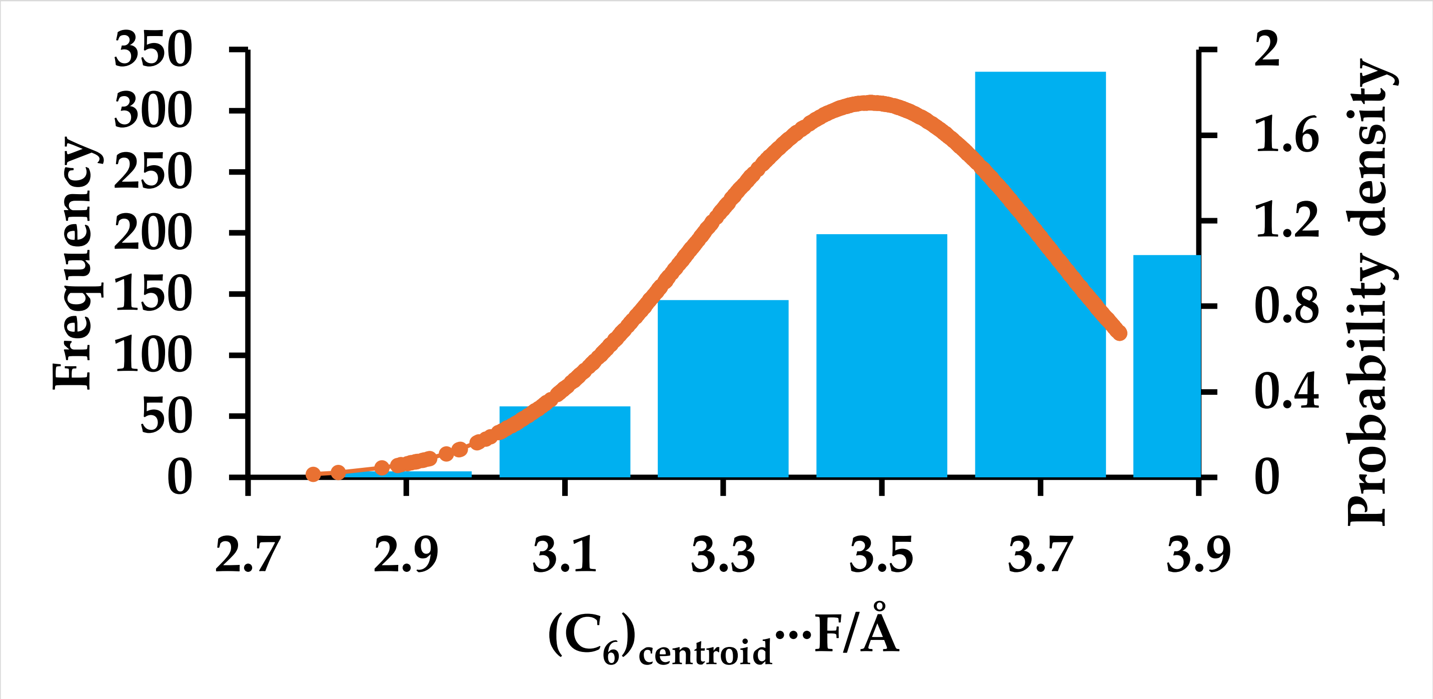

Supplement: Supplementary file 1 [file ijms-27-06519-s001.zip › Table S2 Formatted.pdf]
